# Proton-Secreting Cells as Drivers of Inflammation and Sperm Dysfunction in LPS-Induced Epididymitis

**DOI:** 10.1093/function/zqaf023

**Published:** 2025-06-02

**Authors:** A A S Da Silva, F Barrachina, M C Avenatti, M L Elizagaray, I Bastepe, E Sasso-Cerri, M A Battistone

**Affiliations:** Program in Membrane Biology, Nephrology Division, Department of Medicine, Massachusetts General Hospital and Harvard Medical School, Boston, MA 02129, USA; Department of Morphology and Genetics, Federal University of São Paulo, São Paulo, 04039-001, Brazil; Program in Membrane Biology, Nephrology Division, Department of Medicine, Massachusetts General Hospital and Harvard Medical School, Boston, MA 02129, USA; Program in Membrane Biology, Nephrology Division, Department of Medicine, Massachusetts General Hospital and Harvard Medical School, Boston, MA 02129, USA; Program in Membrane Biology, Nephrology Division, Department of Medicine, Massachusetts General Hospital and Harvard Medical School, Boston, MA 02129, USA; Program in Membrane Biology, Nephrology Division, Department of Medicine, Massachusetts General Hospital and Harvard Medical School, Boston, MA 02129, USA; Department of Morphology, Genetics, Orthodontics and Pediatric Dentistry, São Paulo State University (Unesp) School of Dentistry, Araraquara, 14801-385, Brazil; Program in Membrane Biology, Nephrology Division, Department of Medicine, Massachusetts General Hospital and Harvard Medical School, Boston, MA 02129, USA

**Keywords:** infertility, sperm maturation, clear cells, male reproduction

## Abstract

Proton-secreting cells in various organs, such as the kidney and epididymis, regulate pH balance, maintain cellular homeostasis, and support key physiological processes. More recently, these specialized cells have emerged as key contributors to mucosal immunity, orchestrating immune activation. Epididymitis is an inflammatory condition that significantly impacts male fertility, often due to a lack of diagnosis and treatment. This study investigates the role of region-specific epididymal proton-secreting clear cells (CCs) in shaping immune responses during LPS-induced epididymitis in mice. We found that in response to lipopolysaccharide (LPS), CCs rapidly shifted to a proinflammatory phenotype, marked by the upregulation of cytokines and chemokines, alongside the downregulation of genes involved in sperm maturation. Morphological changes in CCs, including increased apical blebs and altered shape across different epididymal segments, suggest their active role in immune responses. Moreover, mononuclear phagocytes reduced their luminal-reaching projections in the proximal epididymis after the LPS challenge. This bacterial antigen triggered dendritic cell migration and neutrophil infiltration in the distal epididymis. These immune landscape alterations contributed to epithelial damage and impaired sperm maturation, as evidenced by decreased sperm motility following LPS intravasal-epididymal injection. Our findings indicate that proton-secreting cells are immune gatekeepers in the epididymis, initiating immune responses and disrupting sperm maturation. This research enhances the understanding of epithelial immunoregulation and will help to develop novel diagnostic and therapeutic strategies for epididymitis and male infertility. Furthermore, insights into CC-mediated immune responses could inform the development of new approaches for male contraception.

## Graphical Abstract

**Graphical Abstract:**
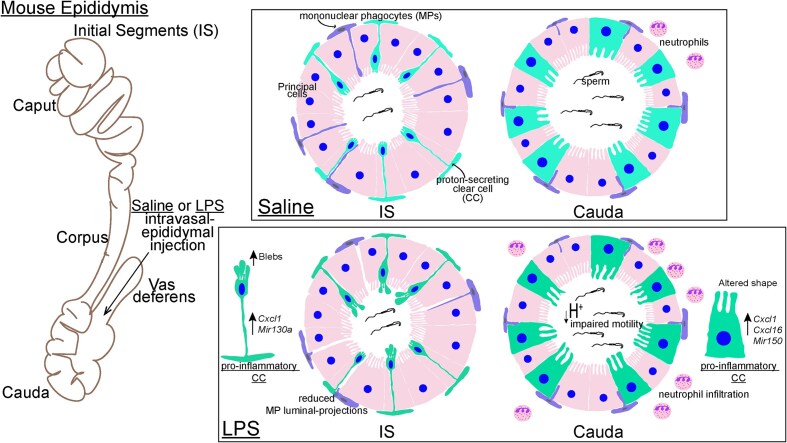

## Introduction

Epithelia provide a barrier to the environment and constitute the first line of defense against stressors. Some epithelia have an even more complex function, as they also need to provide a balance between tolerance and immune activation. This relates in particular—but not exclusively—to the epididymal epithelium, where control of autoimmune responses against antigenic spermatozoa, while protecting against pathogens, is a key determinant of male fertility.^1–3^ This balance is maintained through intricate interactions between different epithelial cell types and immune cells.^1,2,4,5^ The epididymis exhibits differential immune responses along its length, shaped by a region-specific immune cell distribution that protects developing sperm and combats infections; however, severe inflammation can compromise the epididymis, potentially leading to fertility issues.^1,5–13^

Epididymitis, the most common intrascrotal inflammation, affects approximately 400 per 100 000 men annually worldwide.^14–17^ It is frequently caused by bacteria ascending the urogenital tract, from sexually transmitted diseases or urinary tract infections, initiated by *Escherichia coli*, among others.^14–17^ Persistent oligozoospermia and azoospermia occur in up to 40% of males with epididymitis,^15,18^ underscoring the need for studies on the molecular mechanisms of this inflammatory process. Lipopolysaccharide (LPS)-induced epididymitis is a widely used mouse model that mimics this inflammatory male disease.^4,7,19–21^ The model is characterized by high levels of proinflammatory cytokines and immune infiltration in the distal epididymis .^4,19,20^ Additionally, LPS alters the transcriptional profile of *Wfdc* genes, which are involved in innate immunity and fertility.^19^ RNA sequencing (RNA-seq) of whole rat epididymis following proximal LPS injection revealed upregulated genes enriched in inflammatory-related processes.^22^

In the epididymis, proton-secreting clear cells (CCs), strategically located within the epithelium, work with resident mononuclear phagocytes (MPs) to balance inflammation and immune tolerance.^4^ These proton-secreting cells not only contribute to sperm maturation by establishing an acidic environment^23^ but also function as immune sensors and mediators, expressing chemokines in response to bacterial antigens and viral infection.^1,4,9^ A previous study by our group demonstrated that a 6-h intravasal-epididymal injection of LPS upregulates the expression of chemokines and cytokines in CCs, including *Cxcl10, Cxcl1, Cxcl2, Il6*, and *Ccl5*. This discovery highlighted the involvement of these cells in the epididymal immune responses.^4^ However, the specific mechanisms by which these cells contribute to the epididymal mucosal immunity remain poorly understood. Similar proton-secreting cells are found in the kidney and the respiratory tract^23–26^, making studying their role crucial for advancing our understanding of mucosal functions across other systems. Importantly, renal proton-secreting intercalated cells play a significant role in inflammation-induced acute kidney injury.^27^ Furthermore, CCs are also present in the human epididymis.^24,28^

Given the impact of inflammation on male fertility and the role of CCs in modulating epididymal immune responses, we aimed to characterize the molecular mechanisms by which epithelial CCs respond to LPS-induced epididymitis. RNA-seq analysis revealed an early activation of CCs following LPS-triggered inflammation, leading to the upregulation of several pro-inflammatory genes, activation of MPs, and neutrophil infiltration. LPS challenge also altered the distal epididymis/cauda luminal pH and sperm motility. Our findings demonstrate that CCs, in collaboration with MPs, are likely modulators of the early immune response during epididymitis.

## Materials and Methods

### Animals

Adult male transgenic mice (12-15 weeks) that express EGFP under the control of the ATP6V1B1 promoter^29^ were used in this study; they are referred to as B1-EGFP mice. Adult C57BL/6 wild-type male mice (12-15 weeks) were purchased from the Jackson Laboratories (Bar Harbor, ME, USA). All procedures performed were approved by the Massachusetts General Hospital Subcommittee on Research Animal Care and were executed following the National Institutes of Health (NIH) Guide for the Care and Use of Laboratory Animals.

### Animal Model of Epididymitis

Intravasal-epididymal injection was performed as previously reported.^4,30^ Males were anesthetized with 2% isoflurane (Baxter, Deerfield, IL) (mixed with oxygen). A single incision in the scrotum was made to expose the proximal portion of the vas deferens, adjacent to the cauda epididymis. LPS (25 μg, 1 mg/mL endotoxin units; purity ≥99.9%, S-form; Innaxon, Oakfield Close, UK) or sterile saline (Hospira, Lake Forest, IL) was injected into the vas deferens using a 31-G needle (25 μL). The injection was made bilaterally in a retrograde direction into the cauda epididymis. Mice were euthanized at 1, 4, 24, or 48 h post-injections.

### Isolation of EGFP^+^ CCs from B1-EGFP Mice, RNA Extraction, and RNA-seq

Double fluorescence-activated cell sorting (FACS) isolation was performed at the HSCI-CRM Flow Cytometry Core (Boston, MA). EGFP^+^ live CCs (complete gating strategy in Figure S1), 1 h after intravasal-epididymal injections of saline or LPS from the epididymis of B1-EGFP mice, were isolated as we reported.^4^ DAPI was used to distinguish live from dead cells. The CC RNA, from the 3 anatomical regions (IS/caput, corpus, and cauda), was isolated using a PicoRNA kit (Thermo Fisher Scientific). Each sample was obtained from 6 epididymides. DNA contamination was removed by digestion with an RNase-free DNase set (Qiagen, Hilden, Germany). RNA was analyzed by a Bioanalyzer (Agilent RNA 6000 Pico Kit, Agilent Technologies, Santa Clara, CA). RNA-seq libraries were set using the Clontech SMARTER Kit v4 and sequencing on an Illumina HiSeq2500 instrument. Transcriptome mapping was made with STAR (Ensembl annotation of mm9 reference genome).^31^ HTSeq was used for gene reading counts.^32^ Differential expression analysis was made using the EdgeR package,^33^ including only those genes with counts per million values of >1. Differentially expressed genes were defined based on the criteria of > 2-fold change in expression value and *P* < 0.05. Multiplot studio software was used to obtain volcano plots, and Morpheus was used to plot heatmaps. The RNA seq dataset from CCs is deposited in Gene Expression Omnibus under accession number GSE294713.

### Sperm Collection

Sperm were collected from the distal cauda 4 and 24 h after saline or LPS intravasal-epididymal injections. Sperm were recovered by incising the cauda region 3 times and placing it in modified Human Tubal Fluid (HTF) medium (90 126, FUJIFILM Irvine Scientific, Inc., Santa Ana, CA) supplemented with 0.3% Bovine Serum Albumin (BSA; A0281, Sigma-Aldrich). After 15 min of incubation at 37°C, tissues were removed from the tubes, and sperm were collected and analyzed.

### Sperm Capacitation and Computer-Assisted Sperm Analysis

The computer-assisted sperm analysis (CASA) was performed as previously described.^7^ Sperm was obtained from the distal cauda, as indicated above. After 15 min of incubation at 37°C, sperm were diluted (1:5 ratio) in HTF medium with 0.3% BSA and incubated for 45 min at 37°C to achieve sperm capacitation. Sperm analysis was performed using Hamilton Thorne’s CASA version 14 (Hamilton Thorne Inc., Beverly, MA) and 100 µm thickness slides (Leja, IMV Technologies). Sperm were considered hyperactivated when presenting curvilinear velocity (VCL) ≥ 238.5 µm/s, linearity (LIN) < 33%, and amplitude of lateral head displacement (ALH) ≥ 4.22 µm, as previously reported.^6^

### Distal Cauda Luminal pH Measurement

For the pH assessment in the distal portion of the cauda, the luminal content from the cauda distal portion was recovered by an incision in the respective region 4 and 24 h post-injections of saline or LPS using a pH strip (Hydrion MicroFine pH Paper (cat. MF-1606)). The measurement range is from 5.5 to 8.0, with color changes at intervals of 0.2 pH units. Two independent researchers, blinded to the experimental conditions, conducted the pH strip test with at least four mice per group.

### Confocal Microscopy

Epididymides from B1-EGFP mice, after 1, 24, and 48 h post-injections, were removed and fixed for 4 h by immersion in 4% paraformaldehyde (PFA) at room temperature. After several washes in phosphate-buffered saline (PBS), the epididymides were soaked in 30% sucrose in PBS (with 0.02% NaAzide) for 48 h at 4°C. Then, the tissues were embedded in Tissue-Tek OCT compound (Sakura Finetek, Torrance, CA, USA) and frozen on a cutting block in a Reichert Frigocut microtome. Epididymides were cut at 25-µm thickness, and sections were placed onto Fisherbrand Superfrost Plus microscope slides (Fisher Scientific, Pittsburgh, PA, USA).

For immunofluorescence (IF) antigen retrieval, the slides were exposed to 1% SDS in PBS for 4 min.^34^ For the blocking, the sections were incubated with 1% bovine serum albumin in PBS for 30 min at room temperature. Slides were incubated with the primary antibodies for 18 h at 4°C. The antibodies used were rat antibody against Ly6G (0.2 mg/mL; Clone 1A8, 551 461, BD Biosciences), rabbit antibody against AQP9 (0.5 μg/mL,^35^ rabbit monoclonal antibody against cleaved caspase-3 (ASP175) (5A1E) (Cell Signaling Technology, Danvers, MA), rat antibody against F4/80 (20 μg/mL; clone BM8, 14-4801, eBioscience, San Diego, CA), and rabbit antibody against neutrophil elastase (1 mg/mL; Ab68672, Abcam, Waltham, MA). The secondary antibodies (Jackson ImmunoResearch Laboratories, West Grove, PA) were Cy3 donkey anti-rat IgG (3 μg/mL; 712-166-153, Jackson ImmunoResearch, West Grove, PA), Alexa Fluor 647-conjugated donkey anti-rabbit IgG (1.5 μg/mL; 711–606–152), Alexa Fluor 647 donkey anti-rat IgG (3 μg/mL; 712-606-150, Jackson ImmunoResearch, West Grove, PA), and Cy3 donkey anti-rabbit IgG (7.5 μg/mL; 711-165-152, Jackson ImmunoResearch). All antibodies were diluted in DAKO antibody diluent (Dako North America, Cat: S0809). Slides were mounted with DAPI in the SlowFade Diamond Mounting Medium (Thermo Fisher Scientific, Waltham, MA). For negative controls, incubations were achieved with secondary antibodies alone. To evaluate the EGFP^+^ apical cellular protrusions in the CCs (CC blebs), the epididymal sections were only mounted with SlowFade Diamond Antifade Mounting medium containing DAPI.

Images were taken using a Nikon CSA-W1 SoRa spinning disk confocal microscope (Nikon, Yokogawa Electric Corporation, Tokyo, Japan) at the Molecular Imaging Core (MGH, Charlestown, MA), and the stitching images were taken using a Nikon E800. The number of F4/80^+^ luminal projections and CC blebs per tissue area (110 000 µm^2^) was evaluated in epididymal sections using Fiji software.

### Flow Cytometry Analysis

Epididymides from mice injected with LPS or saline were removed, and single-cell suspensions were made as previously described.^4,6,7^ Briefly, the epididymides were divided into proximal and distal regions. They were incubated for 30 min at 37°C, with gentle mixing for 10 s every minute at 1000 g in a medium containing RPMI 1640 with collagenase type I (0.5 mg/mL, Gibco) and collagenase type II (0.5 mg/mL, Sigma). After digestion, the cell suspensions were passed through a 70 μm nylon mesh strainer, washed with 2% fetal bovine serum (FBS) with 2 mm EDTA in PBS, and centrifuged for 5 min at 400 g. Cells were incubated with different antibodies (0.8 μg/mL for each one) against CD64 Alexa Fluor 647 (Clone X54-5/7.1; 558 539, BD Biosciences), CD45 Brilliant Violet 711 (Clone 30-F11; 563 709, BD Biosciences), F4/80 PE/Cyanine 7 (Clone BM8; 123 113, Biolegend, San Diego, CA), Ly6C R718 (Clone AL-21; 566 987, BD Biosciences, San Jose, CA), Ly6G PE (0.2 mg/mL; Clone 1A8, 551 461, BD Biosciences), CD11c Brilliant Violet 421 (clone N418, 117 329, Biolegend), CD11b APC/Cyanine 7 (Clone M1-70; 101 225, Biolegend), which were diluted in 2% FBS in PBS with BD Horizon Brilliant Stain buffer (BD Biosciences). After 30 min, the cell suspension was washed in 2% FBS in PBS and passed through a 40 μm strainer. Flow cytometry analyses were performed using a BD FACSAria II flow cytometer (BD Biosciences) equipped with multiple lasers, including 355 and 405 nm, at the HSCI-CRM Flow Cytometry Core (Boston, MA). DAPI was used as the viability dye for flow cytometry analyses. Data were analyzed using FlowJo software version 10.10 (BD Biosciences). Quantitative beads were added and analyzed in each sample to calculate absolute numbers of cells (Figure  S2).

### Statistical Analysis

Data analysis was performed using GraphPad Prism version 10.2.3 (GraphPad Software; https://www.graphpad.com). To examine whether the samples were normally distributed, we performed a test of normality (Shapiro–Wilk test). Student’s *t*-test (2-tailed) or one-way ANOVA was used as parametric tests. Mann–Whitney *U* test (2-tailed) was used as a nonparametric test. *P*-values < 0.05 were determined statistically significant. Data were expressed as the means ± SEM.

## Results

### CCs Change Their Transcriptomic Profile After Saline and LPS Intravasal-Epididymal Injection

**Figure 1.**
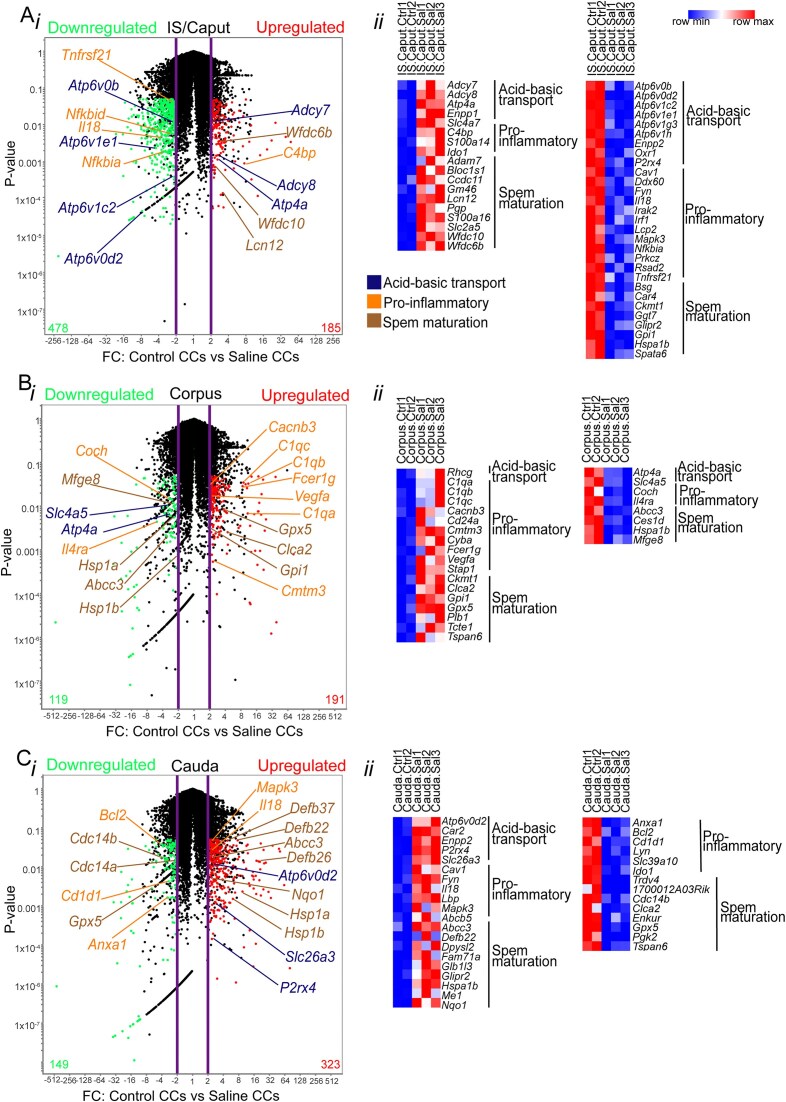
Saline injection changed the transcriptomic profile of clear cells (CCs). Volcano plots show the differential gene expression profiles of EGFP^+^ CCs from control (non-injected) mice compared to those subjected to saline (Sal, 25 µL) intravasal-epididymal injection in the (Ai) IS (initial segments)/caput, (Bi) corpus, and (Ci) cauda regions of the mouse epididymis. Upregulated genes are represented in red, while downregulated genes are shown in green. The most significant genes associated with acid-base transport, pro-inflammatory responses, and sperm maturation are highlighted. FC: Fold Change. The violet lines show ± 2FC. The black dots represent transcripts that are not significantly differentially expressed. Data was analyzed using the Student’s *t*-test, 2-tailed; the value of *P* < 0.05 was considered significant. Heat-map of the genes related to acid-base transport, pro-inflammatory, and sperm maturation genes upregulated (right) and downregulated (left) from Aii) IS/caput, Bii) corpus, and Cii) cauda. The complete list is provided in Table S1. Heatmaps represent row-normalized gene expression of the indicated genes using a color gradient scale ranging from higher (red) to lower (blue) relative levels. The double cell sorting strategy is in Figure S1.

We characterized the transcriptomic profiles of CCs that were isolated by fluorescence-activated double-cell sorting (FACS) from the IS (initial segments)/caput (proximal), corpus (middle), and cauda (distal) epididymal regions of B1-EGFP mice after LPS and saline intravasal-epididymal injections. The complete transcriptome dataset is listed in Table S1. Initially, we analyzed the transcriptomic profiles of saline-treated CCs (saline CCs) from the 3 epididymal regions and compared them to non-injected controls (previously reported in^4^). We observed region-specific variations in the gene expression profile of the CCs (Figure 1). CCs from the proximal epididymis of saline-injected mice showed increased expression of acid-base transport-related genes (*Adcy7, Adcy8, Atp4a*) and sperm function-related genes (*Wfdc6b, Wfdc10, Lcn12*). EnrichR analysis identified pathways related to epithelial secretion and gap junctions. Notably, there was downregulation of V-ATPase subunits (*Atp6v0b, Atp6v1e1, Atp6v1c2, Atp6v0d2*) and pro-inflammatory genes (*Tnfrsf21, Nfkbid, Il18, Nfkbia*) in the CCs of this region (Figure 1A). In contrast, CCs from the corpus of saline-injected mice displayed upregulation of pro-inflammatory genes (*Cacnb3, C1qa, C1qb, C1qc, Fcer1g, Vegfa, Cmtm3*) and downregulation of sperm maturation genes (*Mfge8, Hsp1b, Abcc3*) (Figure 1B). These upregulated genes were associated with the complement cascade, VEGF signaling, high-affinity IgE receptor signaling, and natural killer cell cytotoxicity. CCs from the distal regions of saline-injected mice showed elevated expression of sperm maturation-related genes (*Defb37, Defb22, Abcc3, Defb26, Nqo1, Hsp1a, Hsp1b*) and acid-base transport genes (*Slc26a3, P2rx4, Atp6v0d2*). The pathway-enriched analysis identified FoxO signaling, mitogen-activated protein kinase (MAPK) signaling, apoptosis, infection, Toll-like receptor signaling, and TNF signaling pathways. Interestingly, under the same condition, pro-inflammatory (*Bcl2, Cd1d1, Anxa1*) and other sperm maturation-related genes (*Cdc14a, Cdc14b, Gpx5*) were downregulated in cauda CCs after saline injections compared to CCs from non-injected epididymis (Figure 1C). The disruption in genes related to acidification following intravasal-epididymal saline injections may reflect a compensatory response to pH disturbances caused by the saline solution. These findings confirm that the saline-injected epididymis serves as an appropriate control for assessing LPS-induced effects on CC functionality, consistent with our previous report using flow cytometry.^4^

**Figure 2.**
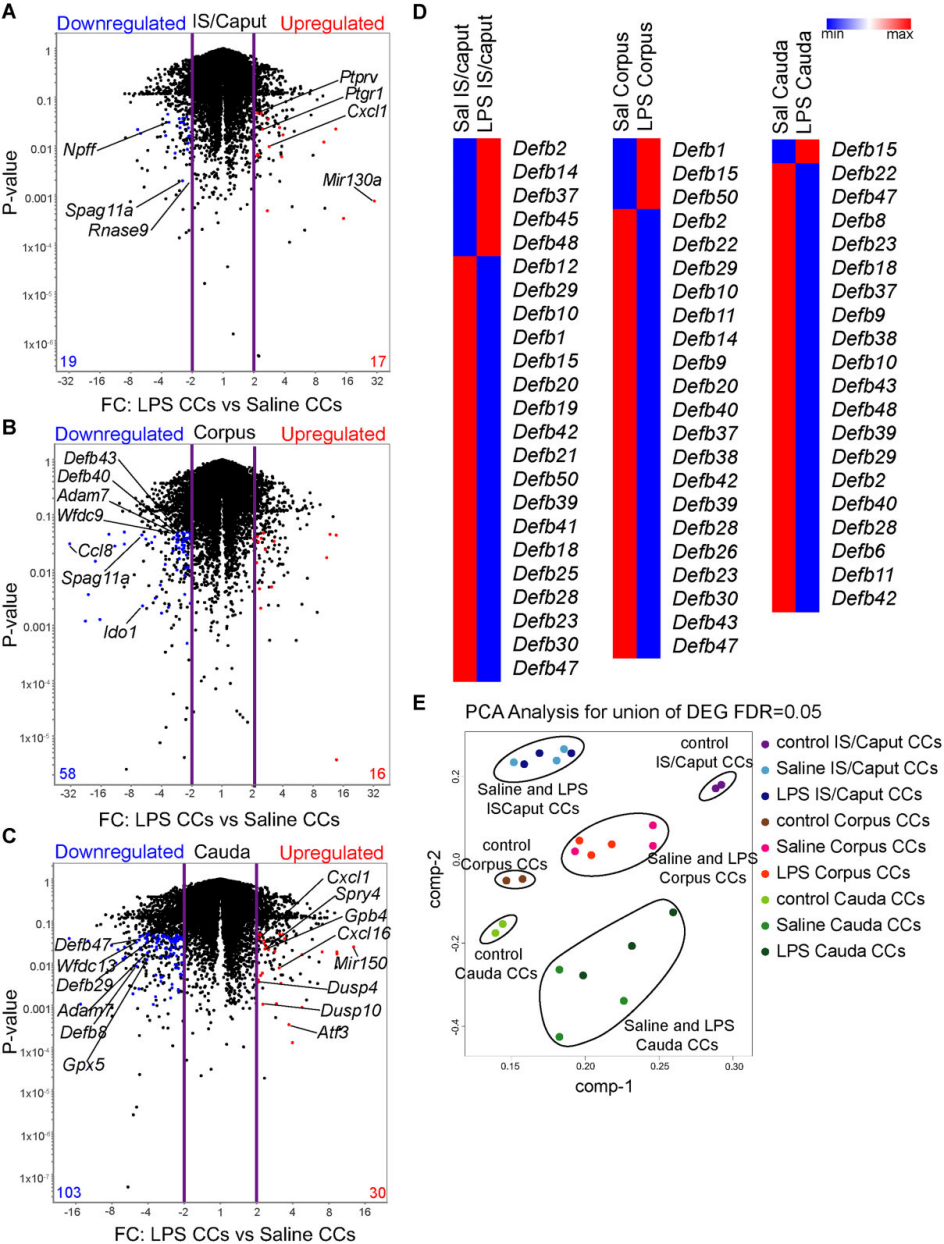
Lipopolysaccharide (LPS) injections changed the CC transcriptomic profiles. Volcano plots show the differential gene expression profiles of EGFP^+^ CCs from saline versus LPS (25 µg) intravasal-epididymal injected epididymis in the (A) IS/caput, (B) corpus, and (C) cauda regions of the mouse epididymis. Upregulated genes are in red, and downregulated genes are in blue; highlighted are some significant pro-inflammatory and sperm maturation-related genes. FC: Fold Change. The violet lines show ± 2FC. The black dots represent transcripts that are not significantly differentially expressed. Data was analyzed using the Student’s *t*-test, 2-tailed; the value of *P* < .05 was considered significant. Each sample of RNA was obtained from double cell sorting live EGFP^+^ CCs from a pool of 4-12 epididymides. (D) Heat-map of the upregulated and downregulated beta-defensins from IS/caput, corpus, and cauda. Heatmaps represent row-normalized gene expression of the indicated genes using a color gradient scale ranging from higher (red) to lower (blue) relative levels. (E) Two-dimensional PCA (principal component analysis) plot of the transcriptomes (population-level RNA-seq) from cauda, corpus, and caput CCs, showing differential gene expression patterns in cells from the 3 regions. FDR: false discovery rate. The double cell sorting strategy is in Figure S1.

Following the LPS challenge, CCs from all 3 evaluated epididymal regions changed their transcriptomic profile compared to saline injection (Figure 2). CCs isolated from the cauda region after LPS injection showed the highest number of distinct genes compared to saline CCs, as illustrated in the volcano plots comparing the gene expression profiles of proximal, middle, and distal CCs. Corpus CCs showed fewer genes differentially expressed between LPS and saline. Specifically, IS/Caput CCs showed an upregulation of immune-related genes, including *Cxcl1, Ptgr1*, and *Mir130a* (Figure 2A). Distal CCs exhibited an increased expression of pro-inflammatory-associated genes such as *Cxcl1, Cxcl16, Mir150, Dusp4, Dusp10*, and *Atf3* (Figure 2C). EnrichR analysis of upregulated genes in CCs from LPS-treated proximal revealed significant enrichment in pathways related to epithelial cell signaling during bacterial infections, even in regions far from the injection site. As expected, CCs from LPS-treated distal upregulated genes were associated with epithelial cell signaling during bacterial infections, chemokine signaling pathways, and NOD-like receptor signaling pathways. Following LPS exposure, CCs downregulated genes associated with sperm maturation^4,6,7,36–38^ in all epididymal regions. For example, in proximal CCs, genes such as *Spag11a* and *Rnase9* were downregulated (Figure 2A). In corpus CCs, the sperm maturation genes *Spag11a, Defb43, Defb40, Wfdc9*, and *Adam7* were downregulated (Figure 2B). Similarly, in distal CCs, genes such as *Defb8, Defb29, Defb47, Wfdc13, Adam7*, and *Gpx5* showed decreased expression (Figure 2C). Interestingly, enriched pathways downregulated in CCs were primarily linked to pathways involved in tyrosine, arginine, proline, and glutathione metabolism. We also observed a downregulation of several β-defensin genes in CCs across all regions of the epididymis following LPS challenge, compared to controls (Figure 2D). Given the critical role of β-defensins in supporting sperm maturation,^39–41^ this downregulation aligns with the rapid pro-inflammatory shift observed in CCs and suggests a disruption of their function related to sperm maturation.

Principal Component Analysis (PCA) of all the RNA-seq data revealed a significant separation of CCs from different epididymal regions based on global transcriptome expression profiles, consistent with our previous findings.^4^ Interestingly, while LPS- and saline-treated CCs clustered closely together, they were distinctly separated from control (non-injected) CCs, indicating transcriptomic differences induced by intravasal injections of saline and LPS (Figure 2E). Additionally, this analysis highlights that CCs from regions distant from the injection site also exhibit altered responses. These findings suggest that region-specific effects may be induced following cauda injections, offering new insights into the molecular mechanisms governing distant tissue responses.

Using previous transcriptomic data from whole epididymis^42^, we made Venn diagrams comparing segment 7 with other segments grouped into proximal (1, 2, and 3), middle (4, 5, and 6), and distal (8, 9, and 10) regions and analyzed the distinct immune response- and sperm maturation-related gene expression. Notably, we revealed that segment 7 shows enrichment in immune response genes compared to the other segments (Figure S3A, Table S2). However, concerning sperm maturation, segment 7 displays similar expression profiles to the other regions (Figure S3B, Table S3), suggesting that immune response may play a more prominent role in this segment than sperm maturation.

### CC Morphological Alterations After the LPS Challenge

**Figure 3.**
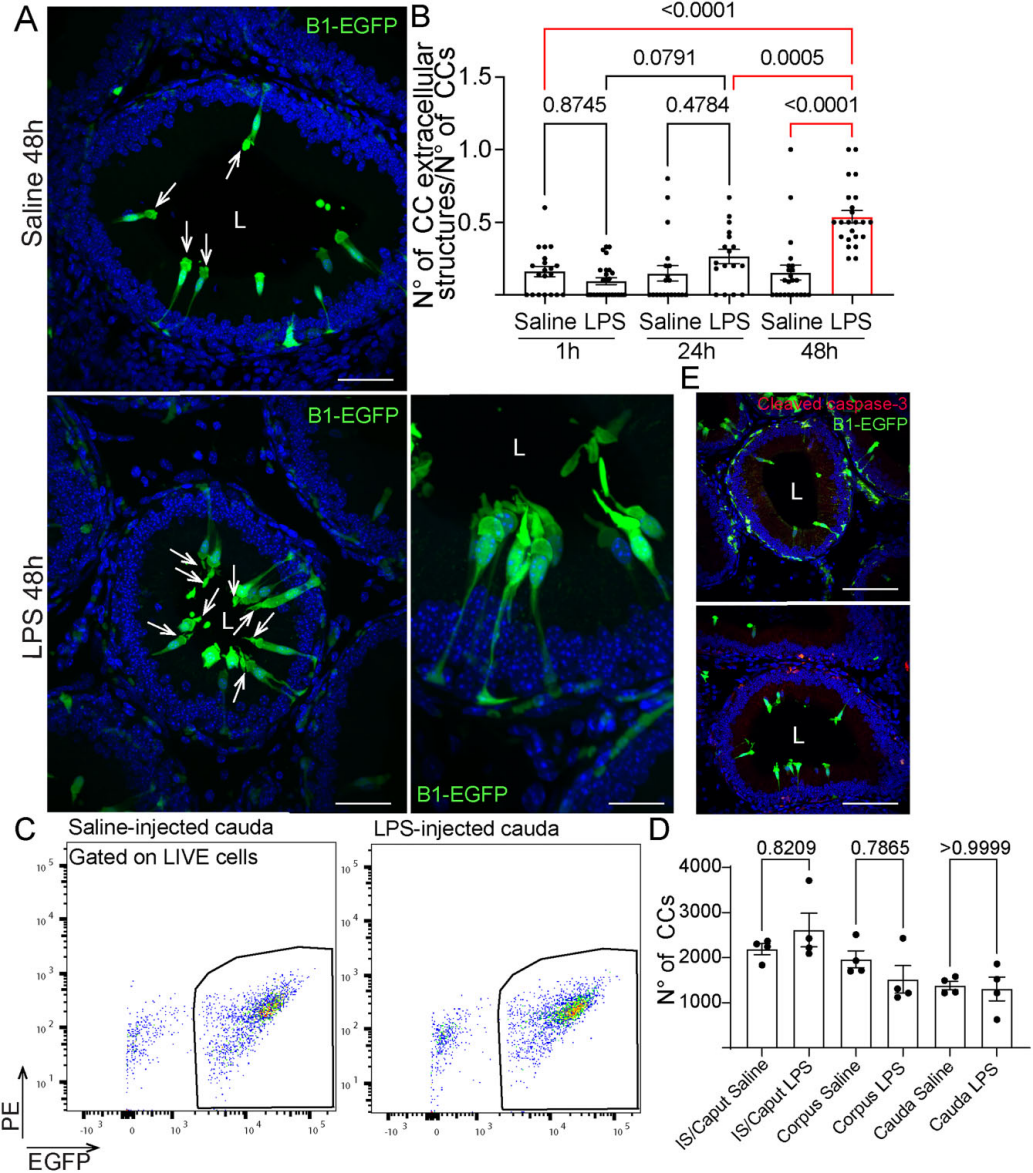
Altered morphology of proximal CCs after LPS injections. Confocal microscopy images of EGFP^+^ CCs from IS. (A) Apical blebs (arrows) in the CCs from both saline- and LPS-injected epididymal IS. Nuclei are labeled with DAPI (blue). Bars: 50 μm. Inset Bar: 16 μm. Low magnification images are in Figure S5. (B) Quantification of EGFP^+^ blebs in the CCs revealed no significant difference at 1 and 24 h following intravasal-epididymal injection, but a marked increase was observed 48 h after LPS injection. The quantifications were performed in a standardized area of tissue (110 000 µm^2^). Each image quantification is represented as a dot. (C) Cell sorting gating strategy for identifying CCs (DAPI^−^EGFP^+^) in the epididymis. The complete double-cell sorting strategy is in Figure S1. (D) The cell sorting analysis showed no difference in the number of EGFP^+^ live CCs between saline and LPS in the epididymal regions. Data was analyzed using one-way ANOVA. Data are shown as means ± SEM. (E) Immunolabeling for cleaved caspase-3 (red) in IS shows that the EGFP^+^ CCs are negative for this marker. Lumen (L). Nuclei are labeled with DAPI (blue). Bars: 100 μm.

**Figure 4.**
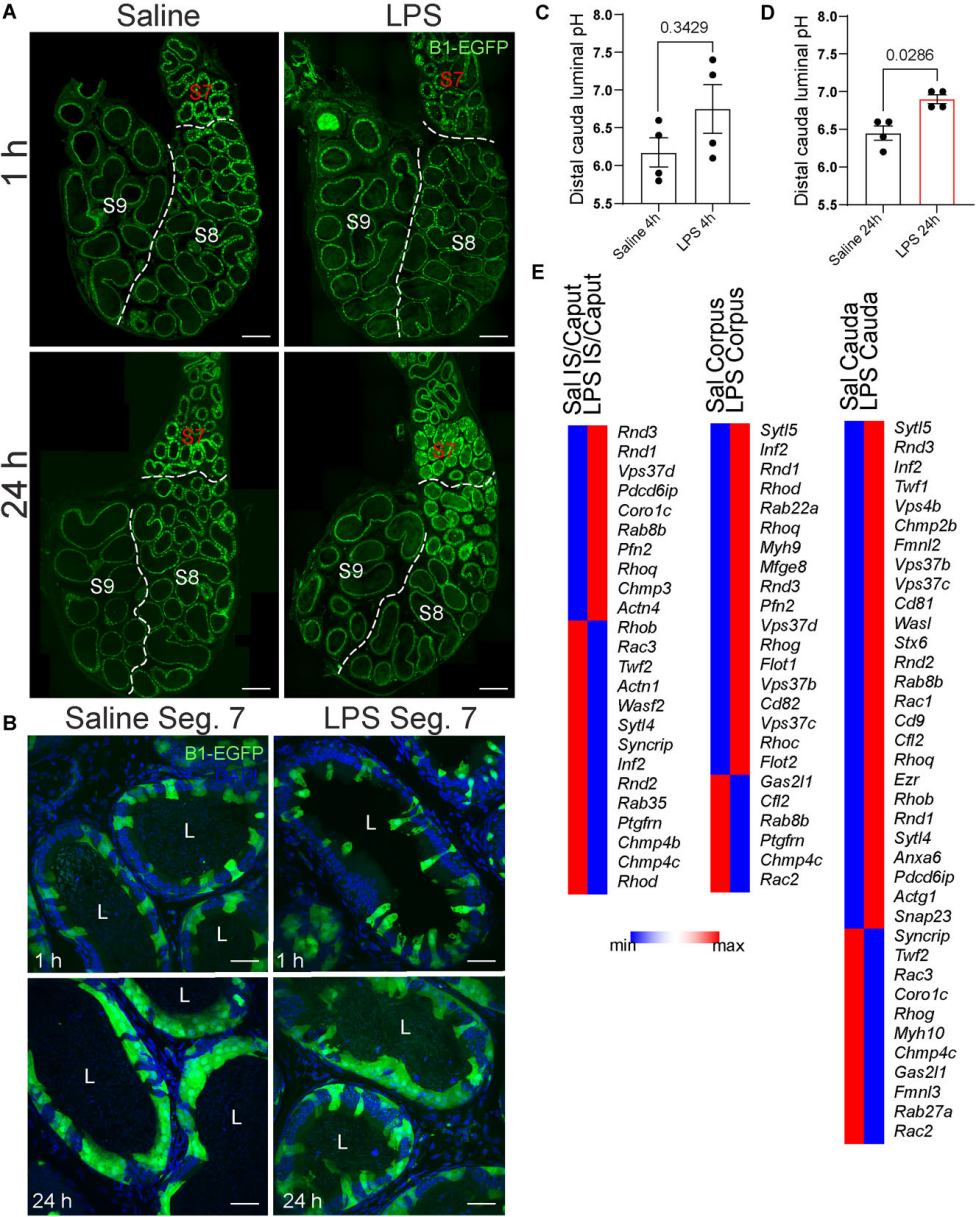
Altered morphology of cauda CCs post-LPS injections. (A) Confocal microscopy images of EGFP^+^ CCs from distal epididymis 1 and 24 h post-intravasal-epididymal injections of saline or LPS. Segmental division of distal epididymis: segments 7 (S7), 8 (S8), and 9 (S9). Bars: 250 μm. (B) CCs from segment 7 showed irregular shapes 1 and 24 h post-intravasal-epididymal injections. Nuclei are labeled with DAPI (blue). Bars: 50 μm. (C) In segment 10 (injection site), the luminal pH was similar at 4 h but showed a (D) marked increase at 24 h following LPS injection compared to saline. (E) Heatmap of genes related to GTPases and vesicle formation expressed in IS/Caput, Corpus, and Cauda CCs. Data was analyzed using the Student’s *t*-test, 2-tailed; the value of *P* < .05 was considered significant. Data are shown as means ± SEM. Lumen (L).

In previous work, we demonstrated that, under physiological conditions, CCs in the proximal epididymis exhibit apical membrane protrusions.^4^ To examine the effects of the LPS challenge on these CC membrane blebs, we performed intravasal-epididymal injections on B1-EGFP mice. Following 1, 24, and 48 h of LPS-induced epididymitis, we observed a marked increase in the number of EGFP^+^ apical blebs in CCs within the proximal epididymis (segments 1-2) compared to CCs in saline-injected controls only 48 h (Figure 3A, arrows and Figure S4), while no difference was observed at 1 and 24 h (Figure 3B). The number of CCs did not change in the epididymal regions following intravasal-epididymal injections (Figure 3C). These cells were negative for the cleaved caspase-3 (Figure 3D), indicating that their morphological changes might not be related to apoptosis. In the distal portion, CCs showed morphological changes at 1 and 24 h post-injection with either LPS or saline. Specifically, CCs at segments 7-8 displayed irregular shapes with vacuoles (Figure 4A, B). Importantly, we did not observe changes in CC morphology in the epididymal segments 8-9 (injection site) and no differences in the luminal pH of the distal portion (segments 9-10) 4 h post-LPS injections (Figure 4C). However, the luminal pH increased 24 h after LPS injections (Figure 4D). Interestingly, CCs from all epididymal portions showed upregulated genes related to vesicle formation, endocytosis, tight junctions, and regulation of actin cytoskeleton after the LPS challenge: CCs from IS/Caput showed upregulation of *Rnd1* and *Pdcd6ip;* CCs from corpus upregulated *Sytl5, Inf2, Rab22a, Rhoq, Rhog* and *Rhoc*, and CCs, from cauda, enhanced the expression of *Sytl5, Sytl4, Rnd3, Cd81*, and *Anxa6* (Figure 4E).

Analysis using aquaporin 9 (AQP9) immunolabeling revealed epithelial damage 24 h after both saline and LPS injections, with localized damage observed in segments 7 and 8. No damage was detected in the proximal regions (segments 1, 2, and 3) (Figure S5, arrows). These observations are consistent with our previous study using CX3CR1 heterozygous mice in which epithelial damage occurred in the cauda epididymis, 48 h after LPS intravasal-epididymal injection.^7^

### Immune Response Induced by LPS-mediated Epididymitis

**Figure 5.**
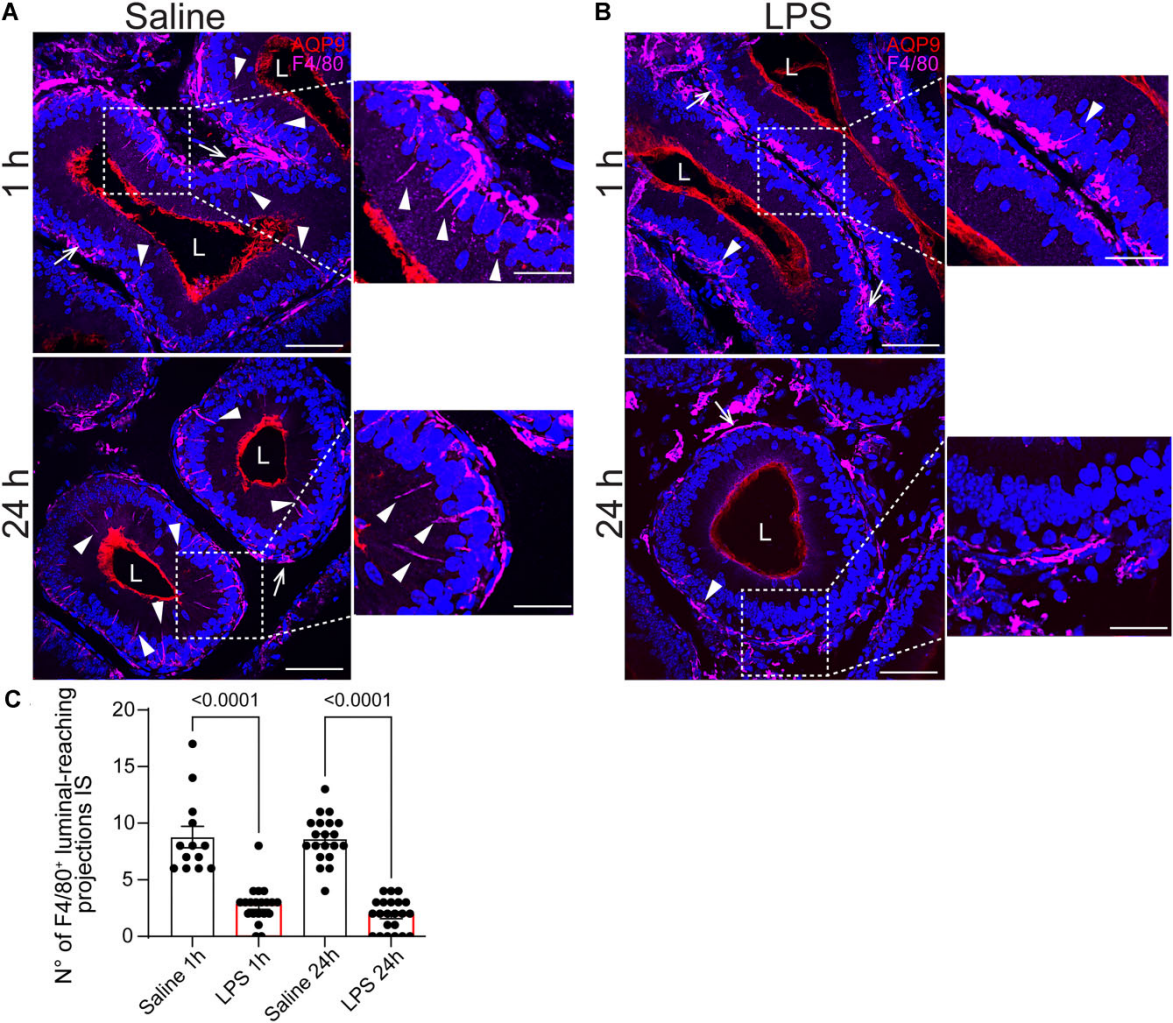
Response of proximal F4/80^+^ mononuclear phagocytes (MPs) post-LPS intravasal-epididymal injection. (A-B) Confocal microscopy images of B1-EGFP epididymis immunolabeled for aquaporin 9 (AQP9, red) and F4/80 (magenta), 1- and 24-h post-intravasal-epididymal injection with saline or LPS. Notably, F4/80^+^ MPs were observed in the basal portion of the epithelium in both saline- and LPS-treated groups (arrows). However, luminal-reaching projections were more numerous in the saline-treated group (arrowheads). Bars: 50 μm. Inset Bars: 25 μm. (C) Quantification of the number of F4/80^+^ luminal-reaching projections in the IS of saline and LPS-injected epididymis. Luminal-reaching projections decreased in the IS/S1 after the LPS challenge compared to saline at 1- and 24-h post-intravasal-epididymal injections. The quantifications were performed in a standardized tissue area (110 000 µm^2^). Each image quantification is represented as a dot. Data was analyzed using one-way ANOVA, the value of *P* < 0.05 was considered significant. Data are shown as means ± SEM. Nuclei are labeled with DAPI (blue). Lumen (L).

The epididymis contains a complex network of MPs that include DCs and macrophages and are strategically positioned in the interstitium and the epithelium, with some extending luminal projections between epithelial cells in the IS.^8,43,44^ LPS injections in the distal epididymal segments caused morphological changes in F4/80^+^ MPs in the proximal region at 1- and 24-post-injection (Figure 5A, B). The luminal-reaching projections in segments 1-2 decreased in the LPS-treated epididymis compared to the saline group (Figure 5C). These results are consistent with our previous findings, which showed, within the IS, reduced MP projections in CX3CR1⁺ cells 48 h after LPS treatment.^7^

**Figure 6.**
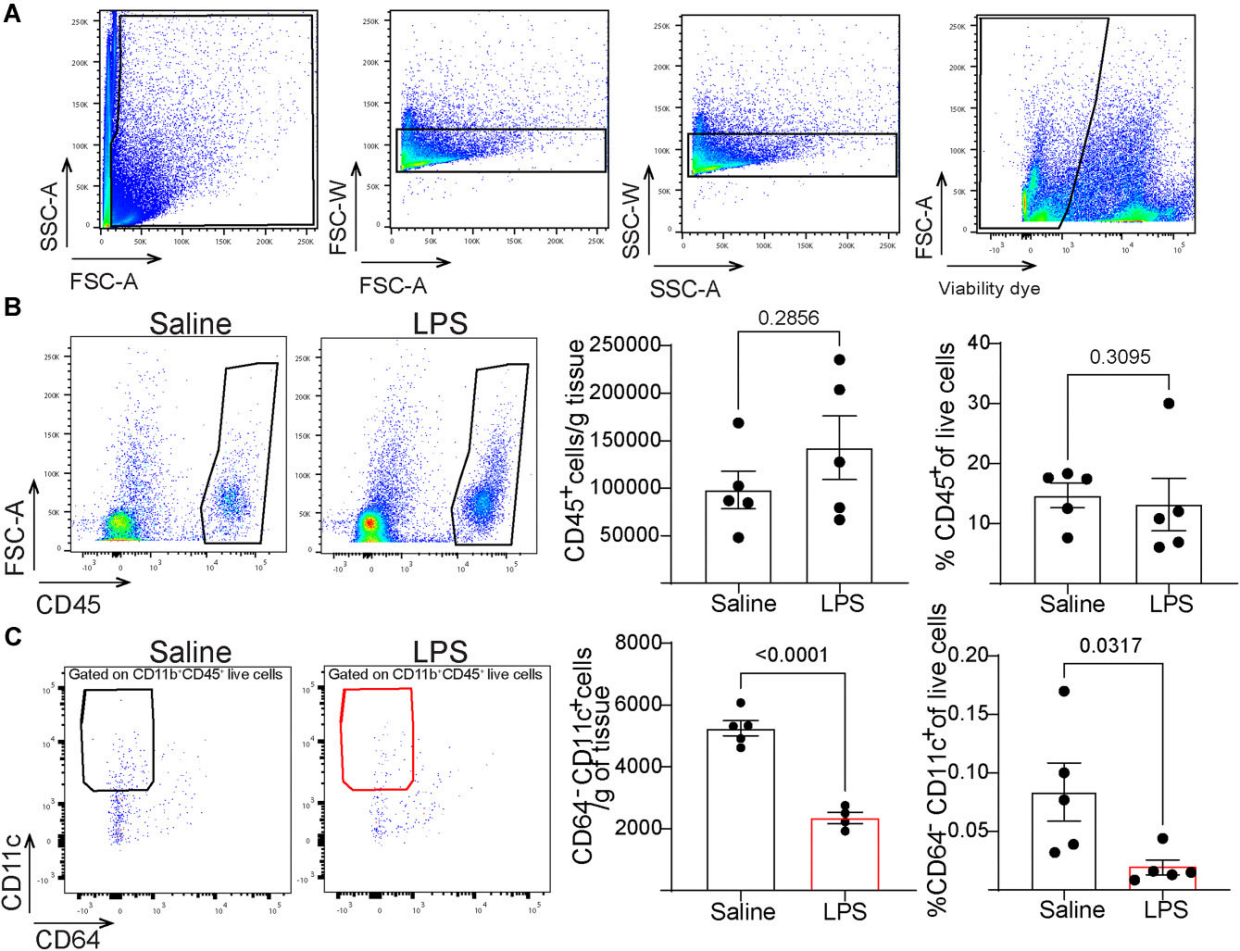
Reduced number of dendritic cells (DCs) 48 h post-LPS injections. (A) Flow cytometry gating strategy used to analyze myeloid cells in the distal epididymis. Key parameters include forward scatter area (FSC-A), side scatter area (SSC-A), and forward scatter width (FSC-W). DAPI was used as a viability dye. (B) The absolute number of CD45^+^ live cells per gram of tissue and the relative abundance of CD45^+^ live cells (%) 48 h after intravasal-epididymal injection of saline or LPS. (C) The number of CD64^−^CD11c^+^CD45^+^ live cells (DCs) per gram of tissue and the relative abundance of DC live cells (%) 48 h after intravasal-epididymal injection of saline or LPS, each dot represents a distal epididymis. The previous gating plots are shown in panel A and Figure S2. Data was analyzed using the Student’s *t*-test, 2-tailed; the value of *P* < 0.05 was considered significant. Data are shown as means ± SEM.

**Figure 7.**
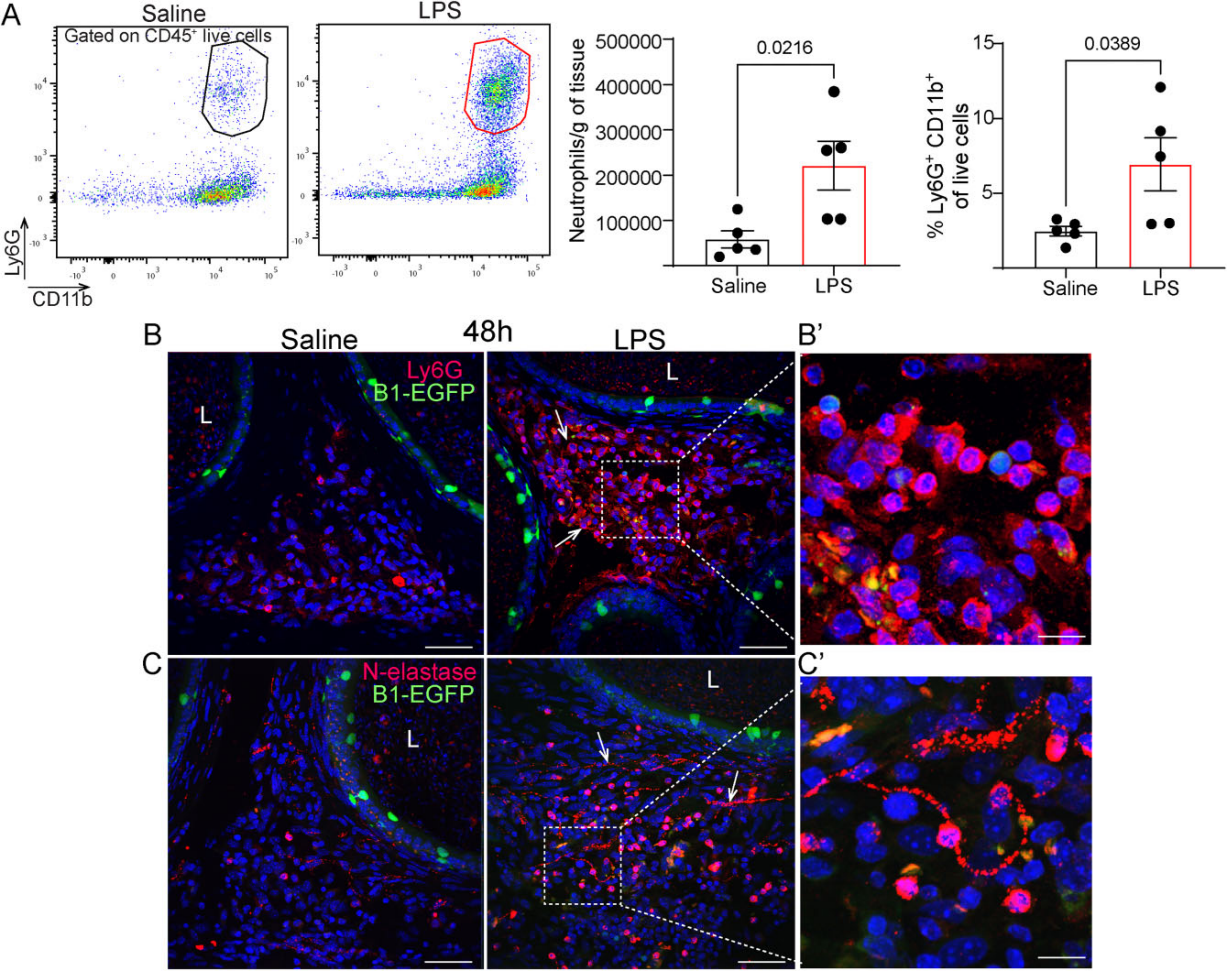
LPS caused infiltration of neutrophils in the distal epididymis. (A) Flow cytometry analysis of Ly6G^+^CD11b^+^CD45^+^ (neutrophils) 48 h after intravasal-epididymal injections of saline or LPS. The number of neutrophil live cells per gram of tissue and the relative abundance of neutrophils (%) increased after the LPS challenge. Each dot represents a distal epididymis. The previous gating plots are shown in Figure 6. Data were analyzed using the Student’s *t*-test, 2-tailed; the value of *P* < 0.05 was considered significant. Data are shown as means ± SEM. (B) Confocal microscopy images of B1-EGFP^+^ epididymis subjected to immunolabeling for Ly6G (red) and (C) N-elastase (red). Note the abundance of Ly6G-positive cells in the interstitial compartment and the presence of N-elastase-positive structures (arrows). Lumen (L). Bars: 50 μm. Inset Bars: 15 μm. Nuclei are labeled with DAPI (blue). Lumen (L).

Flow cytometry analysis revealed no significant differences in the absolute counts and the relative abundance of CD45^+^ live cells in the distal epididymis (Figure 6A, B) after saline or LPS challenges. Interestingly, 48 h after LPS challenge, we found a decrease in CD64^−^ CD11c^+^ live cells (DCs) in the cauda portion, indicating DC migration in response to LPS (Figure 6C). In contrast, we observed neutrophil infiltration in the distal epididymis, as evidenced by an increased number of Ly6G^+^ cells following LPS injection compared to saline (Figure 7A). Ly6G^+^ neutrophils predominantly localized to the epididymal interstitial compartment by IF (Figure 7B). Furthermore, N-elastase-positive structures were detected in the LPS-injected cauda (Figure 7C), suggesting the formation of neutrophil extracellular traps (NETs), which are formed during neutrophil activation and play a critical role in trapping and neutralizing pathogens.

### LPS Exposure Impaired Sperm Motility

**Figure 8.**
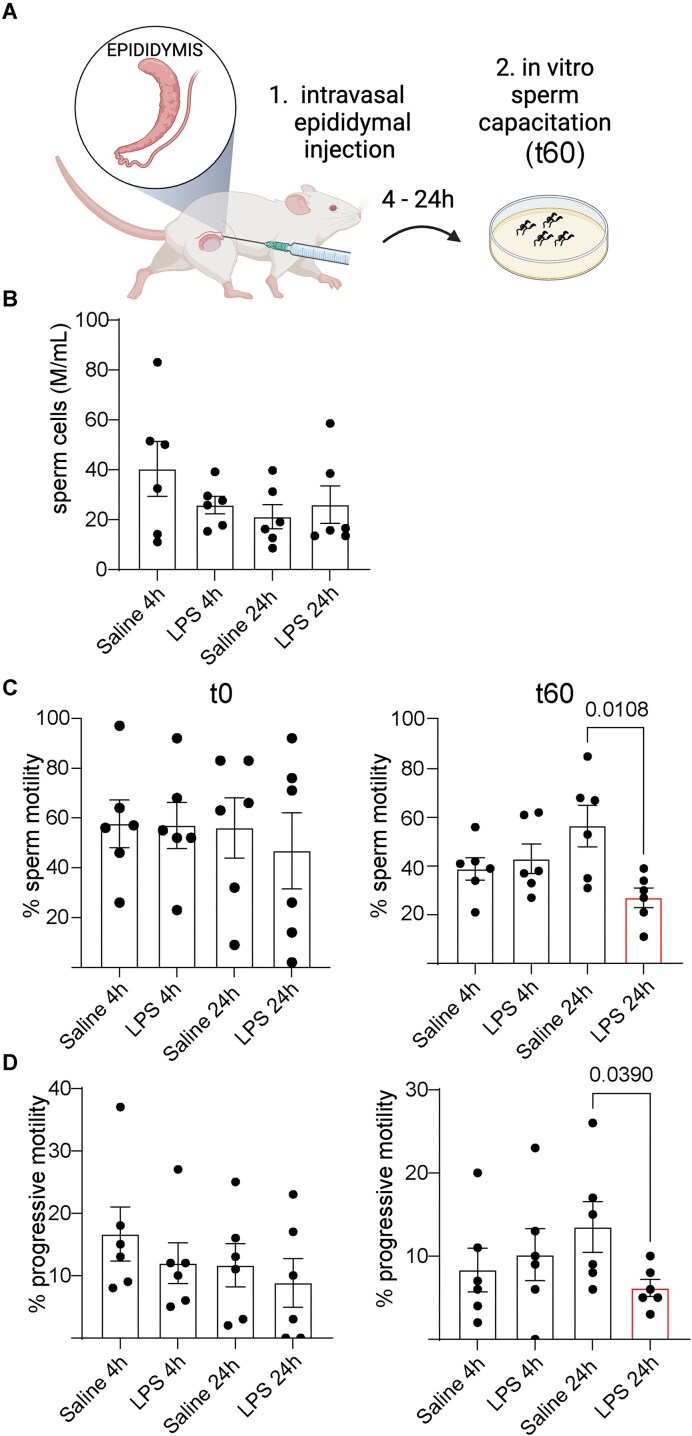
LPS-induced epididymitis disrupted sperm motility. (A) Computer-Assisted Sperm Analysis (CASA) was performed on sperm isolated from the cauda at 4 and 24 h following intravasal-epididymal injections of saline or LPS, assessed at time 0 (t0) and after 60 min of *in vitro* incubation in capacitating medium (t60). CASA analysis for: (B) Sperm concentration (Million (M) per mL), (C) Percentage of total sperm cell motility (relative to total sperm cells), and (D) Percentage of progressive motility (relative to total sperm cells). Under capacitating conditions (t60), the percentages of total and progressive sperm motility were reduced 24 h after LPS injections. No difference was observed in the sperm concentration at t0 and t60. Data was analyzed using the Student’s *t*-test, 2-tailed; the value of *P* < 0.05 was considered significant. Data are shown as means ± SEM.

We then evaluated the impact of the LPS injection on sperm functionality. Sperm count and motility under capacitated conditions (60 min) were analyzed after 4 and 24 h post-intravasal-epididymal injections. The total and progressive motility decreased 24 h after the LPS treatment, and no significant changes were observed between groups at 4 h (Figure 8, Table S4). No change was observed in the sperm concentration under the different conditions. The motility decline may be linked to the downregulation of sperm maturation-related genes in CCs and the inflammatory environment induced by LPS.

We provide insights into the underlying mechanisms of epididymal cell response to bacterial infections and their impact on sperm function following LPS intravasal-epididymal injections. Our results demonstrate that all regions rapidly respond to inflammatory stimuli, highlighting the cellular dynamics of the epididymal mucosa.

## Discussion

Our findings demonstrated that CCs exhibit significant functional plasticity within the epididymis, particularly in response to inflammatory stimuli. The shift after the LPS challenge toward a more pro-inflammatory profile, along with the downregulation of genes associated with sperm maturation, suggests that these cells play an active role in initiating early immune responses. Consequently, the CC immune response triggers immune cell infiltration, which disrupts the epididymal environment and impairs sperm maturation. These insights underscore the importance of CCs in modulating immune responses that could impact male fertility under inflammatory conditions. Our findings demonstrate that CCs are key immune regulators beyond acid secretion. This cellular heterogeneity extends to other tissues containing proton-secreting cells, including the kidney, inner ear, olfactory mucosa, and lung, highlighting broad implications for epithelial physiology, reproduction, and immunology.

pH homeostasis is essential for sperm maturation and successful fertilization, and the epididymal acidic luminal environment provides optimal sperm storage and maturation.^23,45^ CCs are pivotal in maintaining this acidity through their V-ATPase-mediated proton secretion.^23,45^ Our results demonstrate that CCs dynamically respond to external stimuli and stressors, exhibiting region-specific profiles within the epididymis. Extracellular acidosis also impacts immune cell function.^46^ For example, acidosis exacerbates neutrophil recruitment and IL-1β production during Pseudomonas aeruginosa infections.^47^ In our study, luminal pH increased, consistent with altered expression of V-ATPase subunits in the different epididymal regions. This pH imbalance likely affects sperm maturation, as evidenced by reduced motility after LPS exposure. Recent studies have demonstrated that sperm incubated *in vitro* with LPS exhibit a reduction in motility in both mouse and human sperm.^48^ Kidney proton-secreting intercalated cells, similar to CCs, are critical for urinary acidification.^23,26,49^ Emerging evidence indicates that these cells can act as sensors of tissue injury and bacterial infection.^27,50,51^ In response to such stimuli, intercalated cells secrete soluble mediators and contribute to the recruitment and infiltration of immune cells, thereby playing an active role in the innate immune response. Further research is needed to clarify how luminal pH changes are linked to immune responses in the epididymis.

Acute epididymitis is most commonly caused by Gram-negative bacteria such as *Escherichia coli*,^14–17^ which carry LPS as a major component of their outer membrane. In experimental acute epididymitis, LPS elicits stronger inflammatory responses than lipoteichoic acid from Gram-positive bacteria in the epididymis.^30^ LPS intravasal-epididymal injection led to the upregulation of multiple pro-inflammatory mediators and the downregulation of sperm maturation-related genes in both distal and proximal CCs. Although certain cytokines/chemokines are essential for normal sperm maturation,^52^ elevated levels of some of these mediators induce detrimental effects.^53^ We observed an upregulation of *Cxcl1, Cxcl16, Mir130a*, and *Mir150* in CCs following LPS treatment. CXCL1 plays a key role in recruiting several immune cells, particularly neutrophils and macrophages, while CXCL16 facilitates the migration of lymphocytes.^54^ MicroRNAs, such as *Mir130a* and *Mir150*, are also involved in immune responses. *Mir130a* contributes to the production of pro-inflammatory cytokines.^55^  *Mir150* is crucial for the maturation and differentiation of both myeloid and lymphoid cells.^56,57^ This pro-inflammatory response aligns with the immune cell infiltration observed at 24^4^ and 48 h (current study^7^) following the LPS challenge. Additionally, in response to the LPS challenge, CCs exhibited upregulation of several dual-specificity phosphatases (DUSPs) in the proximal and distal epididymal regions. DUSPs regulate MAPK signaling pathways, which control the production of pro-inflammatory soluble mediators.^58^ In the epididymis, DUSP6 modulates cell proliferation in the caput and corpus regions^59^; however, its role in the epididymal immune response remains largely unexplored.

LPS exposure downregulated the expression of important mediators involved in sperm maturation, like Spag*11a* (Sperm Associated Antigen 11A) and β-defensins genes, contributing to decreased sperm motility. It was reported that decreased SPAG11A levels result in lower sperm counts and subfertility.^60–62^ In addition, deletion of multiple β-defensin genes in experimental models leads to male sterility or subfertility, marked by reduced sperm motility and premature capacitation.^37,38^ LPS also led to the downregulation of *Adam7* in CCs. ADAM7 is crucial for sperm maturation and male fertility.^63,64^ This protein is secreted into the epididymal lumen and is transferred to sperm membranes during epididymal transit.^64^ Studies on *Adam7*-null mice revealed reduced fertility and impaired sperm motility,^64^ consistent with the motility defects observed following LPS injections.

We demonstrated that CCs changed their cellular morphology after LPS injections, increasing apical blebs in the proximal epididymis and showing irregular shapes in the distal regions. Interestingly, LPS induces the upregulation of genes related to GTPases and vesicle formation in CCs. These results provide novel evidence that proximal CCs actively respond during the LPS-induced inflammatory process, further supporting their previously reported role in immune activation.^4,7,9^ During the sperm transit through the epididymis, CCs and principal cells transfer proteins and RNAs via extracellular vesicles to sperm, contributing to their maturation.^36,65,66^ Studies from our research group using regulatory T cell-depleted mice revealed an increase in bleb formation in CCs, accompanied by the loss of extracellular vesicle-associated proteins crucial for sperm maturation, sperm-egg interaction, and embryogenesis.^6,10^ Extracellular vesicles are also crucial in the pathogenesis of inflammatory diseases, as they facilitate antigen presentation and enhance immune responses.^67^ Therefore, the increased formation of CC blebs, coupled with their altered morphology, may indicate CC involvement in responding to stressors and influencing the initiation of immune responses by producing extracellular vesicles. The cellular morphological changes may also be associated with disruptions in the sperm maturation process.

Several studies have highlighted the complexity of the epididymal segments, each expressing distinct and overlapping genes, proteins, and signal transduction pathways.^1,2,4,8–11,13,42,68,69^ Through microdissection and transcriptomic analysis, 10 anatomically distinct segments could be categorized.^42,68^ In our study, intravasal-epididymal injection of LPS resulted in epithelial damage and altered the morphology of CCs, particularly in segments 7 and 8 of the epididymis, not at the injection site. These changes were not limited to LPS, as similar alterations were observed in the saline group, suggesting that this region is susceptible to external stimuli. These results are consistent with a previous study using CX3CR1-EGFP transgenic mice.^7^ Furthermore, retrograde flow and shear stress have the potential to damage these epididymal segments, since these processes can impair the epithelial function in other organs.^70–72^ Epididymal segment 7 presents an enrichment of immune-related genes compared to the other segments. In contrast, sperm maturation-related genes displayed similar expression patterns across regions,^42,68^ suggesting that the noted alterations may be associated with an immune response in this specific segment. Our results revealed important inter-regional communication within the epididymis. Specifically, we observed signaling pathways facilitating coordinated immune responses by CCs and MPs across different epididymal regions, underscoring the epididymis’ capacity to maintain an integrated defense system. This regional communication likely supports a rapid and efficient immune response to localized inflammatory challenges, enhancing the overall protection of the reproductive tract. High-resolution 3-dimensional imaging by Damon-Soubeyrand et al. (2023)^73^ revealed notable connections between epididymal segments, particularly through lymphatic and vascular structures. The epididymal segments are drained by initial and collecting lymphatic vessels that connect to the proximal lymph nodes, supporting immune surveillance and fluid reabsorption across the epididymis. These lymphatic connections suggest a mechanism by which immune responses in the proximal epididymis may be activated following LPS challenge in distal regions. Moreover, the reaction of CCs and MPs in the proximal region following intravasal-epididymal injections may be associated with the release of soluble mediators, which could play a pivotal role in facilitating dynamic signaling across the different epididymal regions.

The luminal-reaching projections of epididymal MPs play a crucial role in sensing and capturing luminal antigens, contributing to the immune surveillance within the epididymis.^7,8,10^ These highly dynamic projections that extend into the lumen are in close interaction with CCs, particularly under physiological conditions where they help maintain tissue homeostasis and coordinate local immune responses.^4^ Furthermore, during autoimmune-associated epididymitis, the activity of epididymal MPs becomes more pronounced.^6,10^ However, using the LPS-induced epididymitis model, the number of luminal-reaching projections of F4/80^+^MPs decreased, as we previously reported for CX3CR1^+^ MPs^7^, suggesting that these cells perceive the inflammatory challenge initiated in distal epididymal portions. LPS exposure also induces significant morphological and functional changes in MPs across various organs.^74,75^ For instance, LPS activates microglia, leading to notable morphological remodeling and alterations in phagocytic activity.^76,77^ These findings highlight the pronounced sensitivity of MPs to LPS, further emphasizing their critical role in pathogen recognition and immune defense, particularly within the male reproductive tract.

DCs efficiently capture and present antigens, initiating antigen-specific immune responses and activating T and B lymphocytes.^11,78^ Their migration is influenced by environmental signals, including bacterial antigens and soluble mediators.^79^ The decreased number of DCs after LPS injections in the distal epididymis suggests their migration to nearby lymph nodes. We observed that 48 h after the LPS challenge, neutrophils accumulated in the interstitial compartment of the cauda, with N-elastase-positive structures indicative of NET formation. Late neutrophil infiltration was also observed after intravasal-epididymal injection of uropathogenic *Escherichia coli* intravasal injection, another mouse model of epididymitis.^13^ Neutrophil infiltration releases mediators like reactive oxygen species and elastases, exacerbating inflammation and epithelial damage.^80^ Neutrophils transmigrate across the epithelium, disrupting barrier function.^81,82^ The persistent presence of neutrophils may contribute to epididymal damage observed during epididymitis.^81,82^ Notably, our group showed that renal intercalated cells sense DAMPs, such as UDP-glucose, triggering chemokine production and neutrophil recruitment, highlighting the role of proton-secreting cells in immune activation.^27^

In conclusion, our findings indicate that CCs, together with MPs, serve as key gatekeepers of the epididymal mucosal barrier, monitoring potential threats and initiating immune responses during epididymitis. The distinct transcriptomic and morphological profiles exhibited by CCs in response to various stressors, such as saline and LPS, demonstrate their remarkable adaptability and underscore their dual role in the epididymal immune response and sperm maturation. CCs not only contribute to maintaining the delicate balance required for sperm storage and function but also actively participate in the immune defense, highlighting their functional plasticity in adapting to both physiological and pathological stimuli. The shift of CCs to a pro-inflammatory profile disrupts sperm maturation, impairs motility, and highlights their role in early immune defense. Sustained neutrophil infiltration likely worsens epididymal damage, contributing to the pathogenesis of epididymitis. Additionally, our findings reveal inter-regional communication within the epididymis, coordinating immune responses across regions to ensure rapid and effective defense against localized inflammation, thereby protecting the reproductive tract. By shedding light on the immunoregulatory mechanisms in the epididymis, our study may help identify new diagnostic and therapeutic targets for epididymitis and male infertility and support the development of novel male contraceptive methods.

## Supplementary Material

zqaf023_Supplemental_Files

## Data Availability

The authors declare that the data supporting this study's findings are available within the Supplementary Information files. The RNA sequencing dataset from CCs is deposited in Gene Expression Omnibus (GEO) under accession number GSE294713.
