# Supplementary material for: Proton-Secreting Cells as Drivers of Inflammation and Sperm Dysfunction in LPS-Induced Epididymitis
Source: Function (Oxf). 2025 Jun 2;6(3):zqaf023. doi: 10.1093/function/zqaf023 (PMC12203219; doi:10.1093/function/zqaf023)
Supplement: zqaf023_Supplemental_Files [file zqaf023_supplemental_files.zip › Suppl Table 1 23-05.docx]

| ID | Name | Length | IS.Caput.Sal1 | IS.Caput.Sal2 | IS.Caput.Sal3 | IS.Caput.LPS1 | IS.Caput.LPS2 | IS.Caput.LPS3 | Corpus.Sal1 | Corpus.Sal2 | Corpus.Sal3 | Corpus.LPS1 | Corpus.LPS2 | Corpus.LPS3 | Cauda.Sal1 | Cauda.Sal2 | Cauda.Sal3 | Cauda.LPS1 | Cauda.LPS2 | Cauda.LPS3 |
| --- | --- | --- | --- | --- | --- | --- | --- | --- | --- | --- | --- | --- | --- | --- | --- | --- | --- | --- | --- | --- |
| ENSMUSG00000000001 | Gnai3 | 3253 | 71.42123952 | 76.68212478 | 81.65356753 | 81.45804914 | 76.96964861 | 86.21840518 | 67.89289432 | 62.51907249 | 63.38140114 | 68.66058449 | 73.99023696 | 67.7406197 | 50.68160699 | 46.92951591 | 66.52031096 | 58.43054877 | 74.84997363 | 59.16903302 |
| ENSMUSG00000000003 | Pbsn | 1107 | 0 | 0 | 0 | 0 | 0 | 0 | 0 | 0 | 0 | 0 | 0 | 0 | 0 | 0 | 0 | 0 | 0 | 0 |
| ENSMUSG00000000028 | Cdc45 | 2338 | 1.454411498 | 0.769416718 | 1.386238894 | 1.520000624 | 1.605769909 | 1.208086319 | 2.12482091 | 1.045472093 | 1.973006743 | 1.189859492 | 0.980837471 | 0.883310315 | 1.730204113 | 3.357164572 | 3.048703665 | 1.588618015 | 1.950739663 | 3.317486761 |
| ENSMUSG00000000031 | H19 | 2893 | 0 | 0 | 0 | 0 | 0 | 0 | 0 | 0 | 0 | 0 | 0 | 0 | 0 | 0 | 0 | 0 | 0 | 0 |
| ENSMUSG00000000037 | Scml2 | 6101 | 2.605627903 | 1.774576401 | 2.505496737 | 2.882570581 | 1.964407309 | 1.730000369 | 2.132599034 | 1.201924482 | 1.694446067 | 1.769793648 | 2.490155187 | 2.433529954 | 1.560573682 | 2.512137164 | 2.167792074 | 1.951873283 | 2.186125442 | 2.497545085 |
| ENSMUSG00000000049 | Apoh | 1584 | 0.026834076 | 0.105154338 | 0.152694225 | 0.115053142 | 0.060772631 | 0.250266331 | 0 | 0.144668191 | 0 | 0.267901723 | 0.301609586 | 0 | 0.249151097 | 0.381169909 | 0.130055416 | 0.024173363 | 0.145175367 | 0.034727971 |
| ENSMUSG00000000056 | Narf | 4795 | 12.63188234 | 13.64473915 | 15.28381616 | 17.39776271 | 13.91258524 | 16.65881262 | 18.7573311 | 16.92572578 | 16.31999582 | 17.67045125 | 20.86355851 | 15.88913023 | 13.38494773 | 13.40534243 | 17.1594384 | 15.06869159 | 16.70530981 | 15.83160944 |
| ENSMUSG00000000058 | Cav2 | 3515 | 241.4027963 | 293.8453159 | 272.7083578 | 231.2790083 | 268.9088811 | 261.0717136 | 308.0102509 | 345.6113615 | 349.4585053 | 323.6832061 | 331.3665683 | 297.5617927 | 342.5024493 | 280.6068517 | 315.0541733 | 253.8509315 | 371.2798679 | 302.808309 |
| ENSMUSG00000000078 | Klf6 | 4213 | 94.13085505 | 63.98479469 | 72.29046929 | 76.24148174 | 89.20343223 | 78.19287273 | 66.26919496 | 83.83653868 | 60.59198307 | 55.08563053 | 91.52425585 | 60.75732937 | 78.11374796 | 90.08808026 | 70.0612263 | 137.0754666 | 141.0058395 | 166.2024494 |
| ENSMUSG00000000085 | Scmh1 | 5784 | 10.14862519 | 13.42537283 | 9.601108722 | 11.75260687 | 12.54891786 | 12.1911376 | 10.49484216 | 10.26783986 | 12.27620214 | 12.47243706 | 9.845738331 | 9.457010604 | 12.76795992 | 12.11689624 | 12.62260668 | 9.698433583 | 12.33809904 | 13.29576957 |
| ENSMUSG00000000088 | Cox5a | 685 | 266.0761968 | 231.0990852 | 236.9243713 | 228.9359337 | 241.8541332 | 252.5380919 | 292.7169829 | 260.2101343 | 254.6348821 | 266.0400994 | 268.6556789 | 234.4257829 | 279.2834674 | 242.729683 | 227.1799658 | 241.0910799 | 228.7828147 | 243.1643433 |
| ENSMUSG00000000093 | Tbx2 | 3619 | 60.78040507 | 58.34128863 | 60.25638984 | 62.38048485 | 51.61646746 | 64.90189477 | 51.70534544 | 47.06772996 | 56.90317182 | 57.86025785 | 47.14130332 | 44.66486843 | 40.77141878 | 45.99491827 | 47.24687951 | 39.28516863 | 44.24627479 | 48.06266799 |
| ENSMUSG00000000094 | Tbx4 | 3391 | 0 | 0 | 0 | 0.013435873 | 0 | 0 | 0 | 0 | 0 | 0 | 0 | 0 | 0 | 0 | 0 | 0 | 0 | 0 |
| ENSMUSG00000000103 | Zfy2 | 2807 | 0 | 0 | 0 | 0 | 0 | 0 | 0 | 0 | 0 | 0 | 0 | 0 | 0 | 0 | 0 | 0 | 0 | 0 |
| ENSMUSG00000000120 | Ngfr | 3440 | 0 | 0.009683981 | 0 | 0.026488979 | 0 | 0 | 0 | 0 | 0.011972824 | 0.0137066 | 0 | 0 | 0 | 0.283524952 | 0 | 0 | 0.022282731 | 0 |
| ENSMUSG00000000125 | Wnt3 | 3071 | 0 | 0 | 0 | 0 | 0 | 0.145221297 | 0.015406278 | 0 | 0 | 0 | 0 | 0 | 0 | 0 | 0.107330657 | 0.124684492 | 0 | 0 |
| ENSMUSG00000000126 | Wnt9a | 3462 | 2.08719813 | 1.347141881 | 1.732616339 | 1.473956374 | 1.459807044 | 1.302512639 | 2.815254725 | 1.092157088 | 0.951739243 | 1.811393279 | 2.621964792 | 1.725094135 | 1.695695597 | 1.985478028 | 2.237403959 | 1.880243575 | 2.358030399 | 1.509493108 |
| ENSMUSG00000000127 | Fer | 4416 | 7.940844706 | 9.142941076 | 10.72411373 | 10.21409282 | 11.29181905 | 10.23371672 | 8.721132393 | 10.81080231 | 8.375337671 | 7.8477733 | 7.73530238 | 8.658000197 | 6.63567909 | 6.752061618 | 6.549703858 | 8.0986022 | 8.036718961 | 9.429776673 |
| ENSMUSG00000000131 | Xpo6 | 9659 | 7.001318466 | 8.467041651 | 7.947902751 | 9.19334045 | 8.491230762 | 8.228862683 | 8.082194868 | 7.943735007 | 7.402401011 | 8.235141981 | 5.49023749 | 7.408187125 | 7.272869883 | 6.818274761 | 8.88100625 | 7.706485225 | 6.451864454 | 8.981194875 |
| ENSMUSG00000000134 | Tfe3 | 8492 | 6.461867905 | 7.775100841 | 5.861559437 | 6.78157796 | 7.067888461 | 6.477100126 | 5.404297993 | 6.980052818 | 5.368991162 | 5.15259687 | 6.784809223 | 6.80935309 | 5.321247789 | 5.660578244 | 5.91435429 | 7.615736682 | 6.192143117 | 8.051851114 |
| ENSMUSG00000000142 | Axin2 | 5492 | 5.00743789 | 4.876833021 | 5.082215407 | 4.836505617 | 4.881551617 | 5.576035934 | 5.263664753 | 4.819252525 | 5.504534335 | 5.46027809 | 5.924025259 | 6.148666399 | 6.018278328 | 4.997897969 | 5.911658056 | 5.821678292 | 6.67151126 | 6.700854406 |
| ENSMUSG00000000148 | Baat1 | 6223 | 2.773116096 | 3.292211143 | 3.11711163 | 3.675340552 | 3.828587854 | 3.105506363 | 3.565747456 | 3.878772026 | 3.190085264 | 4.477915093 | 3.239760964 | 2.807376248 | 3.353270289 | 3.365946234 | 3.44284253 | 3.310356224 | 4.187993234 | 1.255229496 |
| ENSMUSG00000000149 | Gna12 | 4017 | 27.16225712 | 32.9728821 | 31.16522195 | 36.58947698 | 32.71101608 | 35.04588402 | 27.2427748 | 25.43307852 | 21.13154319 | 27.26688665 | 26.81915145 | 28.63731157 | 27.01772915 | 27.08949384 | 27.76515106 | 29.0539635 | 27.42090548 | 33.63265275 |
| ENSMUSG00000000154 | Slc22a18 | 1807 | 9.785364265 | 11.46686232 | 12.20715542 | 11.49741905 | 10.57463957 | 8.96720746 | 10.91831052 | 10.77926133 | 15.06601379 | 12.08122613 | 13.32516828 | 14.239635 | 9.391355586 | 14.72742782 | 10.237686 | 10.38317522 | 14.76209333 | 12.93794707 |
| ENSMUSG00000000157 | Itgb2l | 3359 | 0.037962348 | 0.357030126 | 0.259221062 | 0.298405173 | 0.229267871 | 0.442566838 | 0.084512079 | 0.216033238 | 0.196184654 | 0.196519752 | 0.184898619 | 0.216017747 | 0.264356806 | 0.165921122 | 0.208522313 | 0.170991102 | 0.159740446 | 0.016376632 |
| ENSMUSG00000000159 | Igsf5 | 2802 | 25.93998965 | 27.22574153 | 25.01543379 | 26.03255959 | 26.29906322 | 29.67504887 | 20.85337562 | 25.6251665 | 26.73742761 | 26.385547 | 23.30776882 | 23.32624586 | 25.22934956 | 20.88492152 | 23.95336708 | 27.54955907 | 24.74385121 | 24.14746655 |
| ENSMUSG00000000167 | Pih1d2 | 2882 | 0.973400978 | 0.647301206 | 0.436402425 | 0.553309005 | 0.6179324 | 0.670560933 | 0.673081137 | 0.424065537 | 0.414437528 | 0.409010264 | 0.480733448 | 0.658477629 | 0.35946227 | 0.564032984 | 0.757696899 | 0.31886696 | 0.691522359 | 0.85892082 |
| ENSMUSG00000000168 | Dlat | 4667 | 36.24825382 | 35.29724918 | 39.20049107 | 38.10258325 | 35.48788287 | 35.51615466 | 38.71590333 | 36.46568355 | 35.8208844 | 39.16933288 | 40.33283403 | 34.56337366 | 29.66045851 | 29.14821769 | 31.41982803 | 31.13624493 | 36.55246358 | 29.83245106 |
| ENSMUSG00000000171 | Sdhd | 1283 | 362.3707057 | 348.759778 | 333.9017808 | 347.6559813 | 333.0969215 | 334.4326735 | 437.762129 | 387.6692568 | 405.701626 | 422.9598159 | 419.6600016 | 362.8235588 | 410.4584699 | 385.5619445 | 371.8737469 | 389.9195524 | 360.6794301 | 376.4457966 |
| ENSMUSG00000000182 | Fgf23 | 1811 | 0 | 0 | 0 | 0 | 0 | 0 | 0 | 0 | 0 | 0 | 0 | 0 | 0 | 0 | 0 | 0 | 0 | 0.212624929 |
| ENSMUSG00000000183 | Fgf6 | 1229 | 0 | 0 | 0 | 0 | 0 | 0 | 0 | 0 | 0 | 0 | 0 | 0 | 0 | 0 | 0 | 0 | 0 | 0 |
| ENSMUSG00000000184 | Ccnd2 | 5696 | 7.902559939 | 7.608861548 | 6.420363228 | 5.903098792 | 9.36274077 | 7.385932375 | 12.73355632 | 8.609382846 | 8.872165527 | 8.178527882 | 9.133941324 | 9.456129634 | 18.75929298 | 25.01588134 | 11.55900392 | 5.458562712 | 8.922168134 | 13.73296169 |
| ENSMUSG00000000194 | Gpr107 | 10361 | 11.17089029 | 11.24362427 | 12.53575224 | 10.82190234 | 11.17240386 | 11.89916042 | 10.890912 | 11.5377106 | 11.12246608 | 10.3302863 | 12.22386014 | 9.815282042 | 8.927453421 | 10.74027926 | 10.28348842 | 9.054337239 | 11.84449403 | 10.41674234 |
| ENSMUSG00000000197 | Nalcn | 7257 | 0 | 0 | 0 | 0.00627822 | 0 | 0 | 0 | 0.121045233 | 0 | 0 | 0.072416225 | 0 | 0.040787034 | 0.403193814 | 0.136259796 | 0.036934581 | 0 | 0.015160288 |
| ENSMUSG00000000202 | Btbd17 | 3162 | 0.430159909 | 0.716406327 | 0.887307008 | 0.778082351 | 0.669767439 | 0.705209681 | 0 | 0.193256938 | 0.273534735 | 0.328056759 | 0.075545475 | 0.264780183 | 0.234022378 | 0 | 0.182423081 | 0.096876932 | 0.181813553 | 0.556701902 |
| ENSMUSG00000000204 | Slfn4 | 3572 | 0 | 0 | 0 | 0 | 0 | 0 | 0 | 0 | 0 | 0 | 0 | 0 | 0 | 0 | 0 | 0 | 0 | 0 |
| ENSMUSG00000000214 | Th | 2574 | 0 | 0 | 0 | 0.017700483 | 0 | 0 | 0 | 0.163215395 | 0.30401857 | 0 | 0 | 0.021684407 | 1.629064867 | 2.093051335 | 0.640272819 | 0 | 0.11911825 | 0.577018602 |
| ENSMUSG00000000215 | Ins2 | 822 | 0 | 0 | 0 | 0 | 0 | 0 | 0 | 0 | 0 | 0 | 0 | 0 | 0 | 0 | 0 | 0 | 0 | 0 |
| ENSMUSG00000000216 | Scnn1g | 2978 | 0 | 0 | 0.09746178 | 0 | 0.0161625 | 0 | 0.301860611 | 0.371920641 | 0 | 0.316660196 | 0.882344767 | 0 | 0 | 0.109170118 | 0 | 0.385734796 | 0.012869811 | 0 |
| ENSMUSG00000000223 | Drp2 | 8620 | 0 | 0 | 0 | 0 | 0 | 0 | 0 | 0 | 0 | 0 | 0 | 0 | 0 | 0 | 0 | 0 | 0 | 0 |

| ENSMUSG00000000244 | Tspan32 | 2962 | 0.143501607 | 0.022493514 | 0 | 0.184582219 | 0.032499611 | 0.050188454 | 0.255571527 | 0.219200149 | 0.139049682 | 0.111429751 | 0.596783749 | 0.188439104 | 0.616232593 | 0.548799142 | 0.250380826 | 0.400745722 | 0 | 0 |
| --- | --- | --- | --- | --- | --- | --- | --- | --- | --- | --- | --- | --- | --- | --- | --- | --- | --- | --- | --- | --- |
| ENSMUSG00000000247 | Lhx2 | 4702 | 0.488149618 | 0.12752703 | 0.380650919 | 0.862384718 | 0.307094366 | 0.36885276 | 0.020124491 | 0.14620656 | 0 | 0.090250176 | 0.152408417 | 0.083094351 | 0.115408569 | 0.03951007 | 0.122675836 | 0.236160701 | 0.163021254 | 0.175486304 |
| ENSMUSG00000000248 | Clec2g | 3079 | 0 | 0 | 0 | 0.044392054 | 0 | 0 | 0 | 0.024808316 | 0 | 0 | 0.031032776 | 0 | 0.048066175 | 0 | 0 | 0.012436053 | 0 | 0 |
| ENSMUSG00000000253 | Gmpr | 1861 | 21.62944745 | 18.65235668 | 16.84365807 | 20.76075529 | 23.17367201 | 18.4790957 | 24.66055807 | 22.10274981 | 25.78306871 | 30.35279017 | 30.31822996 | 25.85335903 | 29.84832824 | 22.88513065 | 26.94373644 | 37.03551495 | 34.22799863 | 27.37153833 |
| ENSMUSG00000000263 | Glra1 | 3270 | 0 | 0.010187429 | 0 | 0.027866082 | 0.117753942 | 0.015153741 | 0 | 0 | 0 | 0.014419175 | 0 | 0 | 0 | 0 | 0.025199728 | 0.035128998 | 0.01172058 | 0 |
| ENSMUSG00000000266 | Mid2 | 8103 | 5.088241484 | 6.059879807 | 6.280260883 | 5.976970274 | 6.355813689 | 7.191659223 | 5.938170365 | 4.722792448 | 6.577236998 | 4.672592215 | 4.516304849 | 5.669047305 | 4.176440238 | 4.591103789 | 4.566086288 | 6.114777981 | 4.833947357 | 4.853944379 |
| ENSMUSG00000000275 | Trim25 | 6232 | 5.470017839 | 4.308439143 | 3.547288731 | 5.212616273 | 4.278736475 | 4.405040822 | 3.902233147 | 3.462565366 | 4.236289571 | 4.055323643 | 4.867955493 | 4.066160272 | 3.443418488 | 3.197129892 | 3.940328516 | 4.135041525 | 3.290206811 | 3.839692141 |
| ENSMUSG00000000276 | Dgke | 11704 | 0.461223287 | 1.203977636 | 0.991938421 | 0.860303383 | 1.213167932 | 0.927208532 | 1.099542778 | 0.789692531 | 0.953652236 | 0.862119831 | 0.751075902 | 1.068242346 | 0.830347548 | 0.634915754 | 0.883594543 | 1.079622389 | 0.975840437 | 1.085706054 |
| ENSMUSG00000000278 | Scpep1 | 2786 | 23.60212036 | 21.0447573 | 26.86067892 | 19.75511183 | 21.69910127 | 30.13005409 | 19.29188918 | 18.25997129 | 20.87414223 | 19.15814648 | 24.0589615 | 17.8105255 | 17.22898618 | 16.12039932 | 17.56907696 | 17.41356769 | 19.39701315 | 20.02126138 |
| ENSMUSG00000000282 | Mnt | 2671 | 7.781741324 | 10.96294797 | 9.417534925 | 10.30283444 | 9.94714409 | 7.940310734 | 7.61679219 | 9.065512214 | 8.804754961 | 8.967636561 | 8.335129409 | 8.066209569 | 8.071152321 | 7.963830784 | 7.064886783 | 9.963299193 | 9.728651928 | 8.794042894 |
| ENSMUSG00000000290 | Itgb2 | 3148 | 0 | 0 | 0.061465731 | 0 | 0 | 0 | 0.79656035 | 1.64998943 | 0.287834608 | 0.014977987 | 0.424936098 | 0.106383093 | 0.501468028 | 0.604894404 | 0.615143941 | 0.754135216 | 0 | 0 |
| ENSMUSG00000000295 | Hddc2 | 2549 | 15.40791787 | 12.99059115 | 14.0812709 | 13.28044564 | 15.71038073 | 13.89964869 | 14.75621018 | 16.4216842 | 18.19380806 | 14.16925802 | 14.84416128 | 17.97750451 | 18.40512096 | 19.7692565 | 16.03450115 | 10.56033621 | 16.50932679 | 20.0700154 |
| ENSMUSG00000000296 | Tpd52l1 | 1274 | 26.02357716 | 28.97228163 | 25.66758754 | 25.74878487 | 28.18399926 | 23.68729572 | 50.24651067 | 33.96545677 | 38.66489235 | 41.08106799 | 25.98747741 | 32.77089138 | 79.96107471 | 70.212961 | 73.02442167 | 67.8951979 | 70.06432142 | 60.83817219 |
| ENSMUSG00000000301 | Pemt | 2147 | 11.30435748 | 10.48882929 | 9.823409052 | 9.358370075 | 9.774345002 | 8.124155668 | 8.109485722 | 10.56650537 | 6.771700076 | 6.566399755 | 7.387650775 | 8.838996404 | 13.39567199 | 8.934051277 | 11.47579433 | 11.12870937 | 12.04948785 | 9.146833302 |
| ENSMUSG00000000303 | Cdh1 | 3837 | 129.2879618 | 170.1588256 | 150.7811892 | 171.3318501 | 156.3001743 | 169.5537762 | 83.29349652 | 105.0714099 | 92.62404079 | 101.0969079 | 104.1162895 | 114.817051 | 56.53178011 | 72.15355875 | 73.60872182 | 76.90055268 | 85.9519898 | 73.07308235 |
| ENSMUSG00000000305 | Cdh4 | 7257 | 0.122999683 | 0.146894394 | 0.106652403 | 0.163233726 | 0.172444538 | 0.122908806 | 0.091274288 | 0.173673595 | 0.181613408 | 0.019491816 | 0.059249638 | 0.146134434 | 0.101967584 | 0.102398429 | 0.07380739 | 0.184672903 | 0.089781872 | 0.14402274 |
| ENSMUSG00000000308 | Ckmt1 | 2658 | 75.11166734 | 87.69387541 | 91.68767717 | 79.36329393 | 87.82536862 | 93.92275085 | 141.0124313 | 129.9949829 | 139.8606062 | 148.7074287 | 143.9000442 | 130.5095346 | 122.123407 | 94.5656136 | 114.6451119 | 113.8778222 | 127.8985152 | 100.022202 |
| ENSMUSG00000000317 | Bcl6b | 3710 | 0.14893997 | 0.260397286 | 0.117348187 | 0.282453914 | 0.29839198 | 0.227061043 | 0.165785667 | 0.133827825 | 0.64388623 | 0.177927182 | 0.296178988 | 0.376116325 | 0.119673115 | 0.137704841 | 0.266532976 | 0.061925511 | 0.599171218 | 0.281717797 |
| ENSMUSG00000000318 | Clec10a | 1990 | 0.106796925 | 0 | 0.07292492 | 0 | 0 | 0 | 0.047550431 | 1.266682693 | 0.724385956 | 0 | 0 | 0 | 0 | 0 | 0.310564492 | 0 | 0.019259446 | 0 |
| ENSMUSG00000000320 | Alox12 | 3971 | 105.540427 | 133.7633086 | 102.6064584 | 106.3473483 | 110.117987 | 106.8422324 | 56.6298572 | 72.74926512 | 72.08418145 | 71.81250398 | 80.76386207 | 67.69283078 | 143.9085696 | 126.0107272 | 133.6378846 | 170.6540342 | 175.0887313 | 148.9166198 |
| ENSMUSG00000000325 | Arvcf | 5943 | 10.30623568 | 12.31506455 | 10.45936169 | 11.60683512 | 10.60149555 | 11.9400159 | 10.4290105 | 14.00966469 | 13.28530216 | 14.54269545 | 11.65635029 | 12.28451736 | 13.8873124 | 17.48979767 | 16.88133747 | 11.80997533 | 13.15592221 | 14.79127588 |
| ENSMUSG00000000326 | Comt | 3219 | 52.67261484 | 62.23788314 | 56.29302414 | 55.08654382 | 65.16275966 | 55.38699454 | 63.92135466 | 69.8829606 | 62.95049939 | 51.10556187 | 63.7444693 | 61.55501776 | 56.96388211 | 73.85749716 | 62.48710651 | 46.48639134 | 60.09096628 | 62.45985867 |
| ENSMUSG00000000339 | Rtcd1 | 1696 | 19.52330904 | 23.00082496 | 22.36134894 | 20.55082483 | 19.9509094 | 23.37393093 | 18.49546352 | 20.55994304 | 19.86472281 | 18.51554739 | 19.07054652 | 20.10811901 | 23.88060382 | 30.23247198 | 23.92896967 | 23.77359056 | 25.2194265 | 23.19074959 |
| ENSMUSG00000000340 | Dbt | 3285 | 7.789380812 | 10.93190258 | 12.17805467 | 11.63644329 | 11.69232118 | 10.10664581 | 10.41311016 | 13.2656107 | 9.578842631 | 11.65490747 | 11.89647368 | 12.48843592 | 8.544858731 | 8.751564717 | 8.879970037 | 9.896111319 | 10.61702593 | 12.14051823 |
| ENSMUSG00000000346 | Dazap2 | 1834 | 610.8091135 | 648.4207731 | 631.1506041 | 675.7393495 | 680.0405704 | 634.0534345 | 644.7835862 | 675.1767016 | 653.8190948 | 703.4041664 | 706.6994812 | 652.10589 | 669.5314901 | 682.6571776 | 676.0739493 | 681.1510722 | 751.4587297 | 689.4134777 |
| ENSMUSG00000000355 | Mcts1 | 1781 | 28.04243786 | 22.07142907 | 22.43488831 | 26.24684531 | 23.51194475 | 22.45314767 | 27.65440694 | 27.5131119 | 27.12621167 | 25.28294307 | 25.48355449 | 24.91490834 | 28.529969 | 28.05942618 | 28.87116325 | 30.61528632 | 29.97671622 | 26.3154177 |
| ENSMUSG00000000359 | Rem1 | 2112 | 0.563515591 | 1.719273423 | 0.893261214 | 0.906043495 | 0.045579473 | 1.220048365 | 0 | 0 | 0 | 0 | 0 | 0 | 0 | 0 | 0 | 0 | 0 | 0.026045979 |
| ENSMUSG00000000365 | Rnf17 | 5120 | 0.033207169 | 0.045544973 | 0 | 0.071189132 | 0.018801533 | 0.154852292 | 0.064685303 | 0.007459454 | 0.305681172 | 0.046045609 | 0.046655233 | 0.087211973 | 0.077081121 | 0.036284443 | 0.024141537 | 0.254273565 | 0.55393476 | 0.010743966 |
| ENSMUSG00000000374 | Trappc10 | 5025 | 11.13170381 | 9.665711397 | 10.9935466 | 10.06423069 | 10.63212642 | 9.772489348 | 8.850530984 | 9.736212434 | 11.17158627 | 9.186176814 | 7.815127536 | 10.43003128 | 10.24925804 | 10.07443886 | 9.322620706 | 12.42063484 | 9.701701425 | 8.30883821 |
| ENSMUSG00000000378 | Ccm2 | 3312 | 22.22794833 | 18.93966782 | 19.70286608 | 19.4239718 | 18.77610066 | 19.76423945 | 17.47083524 | 16.09800536 | 15.00969943 | 17.70998637 | 17.3963164 | 16.44809381 | 22.14872011 | 21.16066663 | 16.98071371 | 16.98336421 | 16.50160006 | 17.22356391 |
| ENSMUSG00000000381 | Wap | 2761 | 0 | 0 | 0 | 0 | 0.087163933 | 0 | 0.051408199 | 0.013832815 | 0.029834492 | 0.017077401 | 0 | 0.222373158 | 0.107204456 | 0 | 0 | 0 | 0 | 0 |
| ENSMUSG00000000384 | Tbrg4 | 3249 | 1.478327144 | 2.286480582 | 2.531086538 | 2.916804316 | 2.53325914 | 2.226746415 | 2.519265452 | 1.892575188 | 2.776191737 | 2.786375816 | 2.926197826 | 1.838188949 | 2.976009783 | 1.929809725 | 1.864151652 | 1.756017391 | 1.899210157 | 2.861353966 |
| ENSMUSG00000000385 | Tmprss2 | 3145 | 12.1906737 | 15.93087215 | 10.36685515 | 14.84898901 | 12.76375973 | 13.07750997 | 6.513955477 | 7.517042 | 5.146677486 | 8.650542378 | 6.82065385 | 5.572692544 | 12.21925709 | 13.0250191 | 9.196658898 | 12.36987508 | 13.28319988 | 11.71895119 |
| ENSMUSG00000000386 | Mx1 | 4963 | 0 | 0.080546994 | 0 | 0 | 0.019396302 | 0.019968863 | 0 | 0.02308628 | 0 | 0 | 0.009626226 | 0 | 0 | 0.037432269 | 0 | 0 | 0 | 0 |
| ENSMUSG00000000392 | Fap | 5810 | 0.04389519 | 0 | 0 | 0 | 0.024852973 | 0 | 0 | 0 | 0 | 0 | 0 | 0 | 0.050945181 | 0.007993819 | 0 | 0.059314194 | 0 | 0 |
| ENSMUSG00000000394 | Gcg | 1487 | 0 | 0 | 0 | 0 | 0 | 0 | 0 | 0 | 0 | 0 | 0 | 0 | 0 | 0 | 0 | 0 | 0 | 0 |
| ENSMUSG00000000399 | Ndufa9 | 1311 | 141.5218103 | 143.5935662 | 139.0323893 | 135.1192528 | 146.6353189 | 155.2727914 | 153.8834958 | 152.4200224 | 157.8031035 | 150.6952301 | 163.768622 | 135.5584055 | 157.1772833 | 132.0344117 | 128.3502321 | 132.6005779 | 139.9158329 | 133.8932567 |
| ENSMUSG00000000402 | Egfl6 | 2692 | 1.231576423 | 1.695344171 | 1.725059032 | 1.269345589 | 1.180054592 | 0.938777642 | 0.228478762 | 0.737743286 | 0.902676237 | 1.488785207 | 0.372687269 | 0.829356056 | 1.447704853 | 2.018558033 | 3.413056079 | 0.48361094 | 0.697618331 | 1.348663092 |
| ENSMUSG00000000409 | Lck | 3548 | 0.886522837 | 0.760525488 | 0.736237497 | 0.937417203 | 1.153104146 | 0.879882248 | 0.50673106 | 0.828865554 | 1.009928656 | 0.478417508 | 0.754057969 | 0.692189728 | 0.458836886 | 0.929405356 | 0.592243334 | 0.334557167 | 0.464495707 | 0.899246953 |
| ENSMUSG00000000411 | Tssk3 | 1278 | 0.798219268 | 1.40758708 | 0.264956739 | 0.427803233 | 0.71557633 | 0.116320971 | 0.296167004 | 1.016073303 | 1.675820673 | 0.516517876 | 0.934564915 | 0.786136093 | 0.694815736 | 1.417307831 | 0.257912713 | 1.198454066 | 0.239914222 | 1.979983497 |
| ENSMUSG00000000416 | Cttnbp2 | 9995 | 1.046150405 | 1.539825626 | 0.895357991 | 1.14415429 | 1.073878837 | 1.160114023 | 0.165677215 | 0.28276516 | 0.568658247 | 0.273610884 | 0.348931663 | 0.558435844 | 0.404724084 | 0.306684319 | 0.218477485 | 0.340956884 | 0.115036409 | 0.599899213 |

| ENSMUSG00000000420 | Galnt1 | 5503 | 13.57879329 | 14.34094974 | 14.20527441 | 12.73357917 | 16.04980555 | 15.4880432 | 12.526726 | 13.94997798 | 13.33715629 | 12.44099966 | 14.63721243 | 15.82272099 | 10.69470785 | 13.88343154 | 13.91104686 | 10.95210177 | 15.95594152 | 12.7451592 |
| --- | --- | --- | --- | --- | --- | --- | --- | --- | --- | --- | --- | --- | --- | --- | --- | --- | --- | --- | --- | --- |
| ENSMUSG00000000435 | Myf5 | 2080 | 0 | 0 | 0 | 0 | 0 | 0 | 0 | 0 | 0 | 0 | 0 | 0 | 0 | 0 | 0 | 0 | 0 | 0 |
| ENSMUSG00000000439 | Mkrn2 | 2314 | 24.88959098 | 25.98520919 | 23.28786206 | 21.63854266 | 25.79238779 | 24.94768134 | 21.42769556 | 22.61175078 | 20.13048804 | 23.8606195 | 20.79057182 | 21.73289196 | 17.56676743 | 24.40622743 | 21.68690366 | 20.53528253 | 20.38879494 | 22.91649908 |
| ENSMUSG00000000440 | Pparg | 1975 | 0 | 0.016867288 | 0 | 0.115344416 | 0.024370594 | 0 | 0 | 0.154703402 | 0.354516845 | 0 | 0 | 0 | 0 | 0 | 0 | 0 | 0 | 0 |
| ENSMUSG00000000441 | Raf1 | 6697 | 19.14224442 | 21.44421188 | 19.53865904 | 21.15115527 | 23.33647247 | 19.71898387 | 19.37156423 | 21.69387767 | 19.27408398 | 18.58006658 | 20.93765912 | 20.76939393 | 19.99205965 | 20.32665673 | 20.75150784 | 17.98177696 | 22.81150059 | 20.97853644 |
| ENSMUSG00000000486 | 1-Sep | 2354 | 0.668093251 | 0.679277367 | 0.410990062 | 0.774189368 | 0.674746593 | 0.652563611 | 1.065238735 | 1.151937371 | 1.154762124 | 0.761141344 | 1.095942123 | 0.42679776 | 1.257398061 | 1.617848414 | 0.402564055 | 0.374122332 | 1.090850422 | 0.443998737 |
| ENSMUSG00000000489 | Pdgfb | 2911 | 3.76720557 | 3.272926055 | 4.835691286 | 3.803275085 | 4.150158997 | 3.540696869 | 1.332751519 | 2.112324558 | 1.99494975 | 1.635939893 | 2.035072089 | 1.821534849 | 0.508401758 | 1.818834065 | 1.755064559 | 2.880674346 | 1.158610143 | 1.303891571 |
| ENSMUSG00000000530 | Acvrl1 | 4297 | 0.138485563 | 0.790764536 | 0.765510836 | 0.508943479 | 0.324837279 | 0.784171721 | 0.110106304 | 0.257756497 | 0.20128388 | 0.021945871 | 0 | 0.233810083 | 0.183688777 | 0.410722672 | 0.047942234 | 0.347529367 | 0 | 0.307241927 |
| ENSMUSG00000000531 | Grasp | 2005 | 1.929162603 | 2.807919762 | 2.605656498 | 3.204043517 | 3.048755265 | 3.163466283 | 1.274256689 | 2.571558267 | 1.273597994 | 1.363960493 | 0.381246551 | 0.890823543 | 0.910364558 | 1.389848002 | 1.787798186 | 0.802097512 | 1.204267683 | 0.384103488 |
| ENSMUSG00000000532 | Acvr1b | 3268 | 2.172082129 | 2.813451287 | 2.323942554 | 2.077293637 | 2.223965873 | 1.940865942 | 1.722830108 | 1.70627012 | 2.64662433 | 2.52489996 | 1.929710685 | 1.776263436 | 2.309603226 | 1.449601259 | 1.197719648 | 3.128394179 | 2.427644877 | 2.01991824 |
| ENSMUSG00000000538 | Tom1l2 | 12272 | 5.330464953 | 5.233642443 | 5.337170804 | 6.378249199 | 5.145785842 | 6.66652242 | 5.389759219 | 5.150621413 | 4.403252011 | 6.389473559 | 5.512495209 | 5.830808298 | 4.494221277 | 4.185720392 | 5.358350978 | 5.575726485 | 6.780181761 | 4.504901585 |
| ENSMUSG00000000544 | Gpa33 | 2997 | 0 | 0 | 0 | 0.030404434 | 0.016060035 | 0.016534112 | 0 | 0 | 0 | 0.015732634 | 0 | 0 | 0.11522303 | 0 | 0 | 0.012776312 | 0 | 0 |
| ENSMUSG00000000552 | Zfp385a | 3711 | 0.022907667 | 0.269303914 | 0.208562782 | 0.147327548 | 0.363161914 | 0.267058656 | 0.1784903 | 0.072041718 | 0.155378933 | 0.241407535 | 0.308973054 | 0.150405989 | 0.252575163 | 0.500609942 | 0.86599875 | 0.11349951 | 0.454421197 | 0.548460509 |
| ENSMUSG00000000555 | Itga5 | 4349 | 0 | 0.114898462 | 0.155720723 | 0.136190751 | 0.265616502 | 0.296245361 | 0.217579577 | 0.035127526 | 0 | 0.184309486 | 0 | 0 | 0 | 0 | 0 | 0 | 0.044063344 | 0 |
| ENSMUSG00000000560 | Gabra2 | 2382 | 0 | 0 | 0 | 0 | 0 | 0.020802995 | 0 | 0 | 0 | 0 | 0 | 0 | 0 | 0 | 0 | 0 | 0.016089965 | 0.230936636 |
| ENSMUSG00000000561 | Wdr77 | 6811 | 9.236185659 | 8.647364114 | 8.096582676 | 8.863365694 | 8.607353256 | 9.014217722 | 10.67678572 | 7.956984145 | 8.689623132 | 8.757251449 | 9.64477579 | 6.769011496 | 8.11937737 | 8.278244324 | 9.104146471 | 8.162966076 | 9.222845489 | 7.914979385 |
| ENSMUSG00000000562 | Adora3 | 2521 | 0 | 0 | 0 | 0 | 0 | 0 | 0 | 0.090898221 | 0 | 0 | 0 | 0 | 0 | 0 | 0 | 0 | 0 | 0 |
| ENSMUSG00000000563 | Atp5f1 | 2743 | 144.0961108 | 127.0334938 | 129.4957468 | 129.5076422 | 140.5002963 | 125.4444703 | 157.3754585 | 154.0227326 | 165.6770014 | 154.7911348 | 153.0784215 | 137.4534454 | 154.4702288 | 137.8761224 | 137.2884509 | 145.2753741 | 140.57633 | 137.4124678 |
| ENSMUSG00000000567 | Sox9 | 1698 | 8.686275666 | 12.30105105 | 9.515170282 | 14.5698746 | 13.54950501 | 12.54868989 | 13.59751903 | 36.39299593 | 16.13017255 | 27.29631408 | 23.85934322 | 20.97196274 | 34.51491032 | 25.27346098 | 24.21622661 | 47.44607654 | 27.71772238 | 20.44213566 |
| ENSMUSG00000000568 | Hnrnpd | 5344 | 26.8043494 | 30.02150048 | 28.44117372 | 30.49623046 | 28.10998012 | 28.49467633 | 30.26984455 | 31.22429007 | 33.04786295 | 30.19268461 | 29.27825393 | 30.11163085 | 29.74315821 | 29.84449777 | 29.96056254 | 29.71391259 | 28.75189451 | 29.73826896 |
| ENSMUSG00000000579 | Dynlt1c | 6502 | 0 | 0 | 0 | 0 | 0 | 0 | 0 | 0 | 0 | 0 | 0 | 0 | 0 | 0 | 0 | 0 | 0 | 0 |
| ENSMUSG00000000581 | C1d | 4056 | 27.17355065 | 22.95600082 | 21.62258642 | 23.53313311 | 25.3475811 | 24.31211534 | 28.20563551 | 25.7064247 | 26.73671006 | 25.73758802 | 23.26320094 | 22.85744959 | 26.66064168 | 28.76418824 | 25.13132379 | 25.62147645 | 27.54466358 | 24.22245183 |
| ENSMUSG00000000594 | Gm2a | 4123 | 26.1752709 | 26.27541402 | 25.14297248 | 27.53774496 | 26.64007999 | 27.57069387 | 27.03581653 | 25.64069121 | 26.4121144 | 26.38289411 | 24.80867963 | 25.23414869 | 26.51455947 | 28.31929073 | 28.77013811 | 29.32858069 | 32.1725233 | 28.4985331 |
| ENSMUSG00000000600 | Krit1 | 4763 | 15.89370533 | 16.44312709 | 17.69193573 | 18.68165433 | 17.62378225 | 16.16732059 | 17.50261186 | 18.15402037 | 15.72057226 | 17.62088008 | 18.3757556 | 18.93724769 | 18.99532569 | 16.09892679 | 16.85085679 | 16.08639627 | 18.78897822 | 17.50867222 |
| ENSMUSG00000000605 | Clcn4-2 | 4759 | 44.73805983 | 43.67286132 | 46.5134009 | 44.57495743 | 43.29749217 | 46.85591534 | 34.1697158 | 39.59682989 | 40.95284568 | 37.07458113 | 44.40189984 | 41.07300911 | 29.48097767 | 32.08828732 | 32.22361652 | 29.58490511 | 35.4914878 | 27.02485638 |
| ENSMUSG00000000606 | Vmn2r88 | 3174 | 0 | 0 | 0 | 0 | 0 | 0 | 0 | 0 | 0 | 0 | 0 | 0 | 0 | 0 | 0 | 0 | 0 | 0 |
| ENSMUSG00000000617 | Grm6 | 5410 | 0 | 0 | 0 | 0 | 0 | 0 | 0 | 0 | 0 | 0 | 0 | 0 | 0 | 0 | 0 | 0 | 0 | 0 |
| ENSMUSG00000000627 | Sema4f | 5143 | 0.15702865 | 0.058295946 | 0.018811406 | 0.062011921 | 0.121663428 | 0.154159778 | 0.009199432 | 0.170800166 | 0.096099201 | 0 | 0 | 0 | 0.095920508 | 0 | 0 | 0 | 0.067069157 | 0.128351017 |
| ENSMUSG00000000628 | Hk2 | 6685 | 9.906217539 | 8.625971562 | 7.843965132 | 6.04527245 | 6.271190055 | 7.627488834 | 14.44505276 | 12.23756559 | 14.04099767 | 10.87604853 | 10.09813258 | 12.15670976 | 14.83278291 | 9.712615433 | 11.21717752 | 8.872423465 | 8.949491319 | 10.95244892 |
| ENSMUSG00000000631 | Myo18a | 10638 | 6.924372068 | 8.605361282 | 7.002748331 | 8.625676188 | 7.87267787 | 9.958990824 | 5.408179881 | 6.049464002 | 5.067977922 | 6.550924931 | 5.056834297 | 5.461938781 | 4.159685072 | 4.234890465 | 5.495864618 | 5.996630187 | 5.227623313 | 5.057238802 |
| ENSMUSG00000000632 | Sez6 | 5750 | 0 | 0 | 0 | 0 | 0 | 0 | 0 | 0 | 0 | 0.008200122 | 0 | 0 | 0 | 0 | 0 | 0 | 0 | 0 |
| ENSMUSG00000000673 | Haao | 1212 | 0.140281109 | 0.082457659 | 0.239472923 | 0.112774862 | 0.079425617 | 0.367965843 | 0.156147455 | 0.252095065 | 0.067964547 | 0.116709662 | 1.182548477 | 0.322367688 | 0.529137727 | 0.229921225 | 0.543914929 | 0 | 0 | 0.090774104 |
| ENSMUSG00000000682 | Cd52 | 1183 | 15.198385 | 14.95278684 | 15.00683486 | 9.320179822 | 6.916675421 | 8.670681192 | 650.2993739 | 528.9793013 | 430.9091341 | 252.4136972 | 140.0133399 | 400.0515664 | 2926.804688 | 4307.168916 | 2011.527525 | 639.0614983 | 853.2865662 | 1970.516412 |
| ENSMUSG00000000686 | Abhd15 | 1892 | 0.022465738 | 0.035214476 | 0 | 0.02408089 | 0.178077942 | 0 | 0.050013403 | 0.100931296 | 0.108843858 | 0 | 0.176757246 | 0.029500879 | 0.13036976 | 0 | 0.021776721 | 0.161905317 | 0.060771084 | 0 |
| ENSMUSG00000000690 | Hoxb6 | 2073 | 65.98246814 | 62.49582519 | 65.05808135 | 67.03385684 | 59.41610838 | 59.83139997 | 56.14529193 | 55.60283187 | 56.60414057 | 59.91073439 | 58.51452946 | 62.33152865 | 66.56120242 | 64.27790001 | 54.55777661 | 57.90692436 | 50.36219622 | 60.02440878 |
| ENSMUSG00000000693 | Loxl3 | 4183 | 0.01016141 | 0.270771791 | 0.150336097 | 0 | 0.034519668 | 0.10661597 | 0 | 0.082173469 | 0.147692502 | 0.247983617 | 0.239845596 | 0.040030358 | 0.01179343 | 0.022206114 | 0.088647861 | 0.036615451 | 0.045811973 | 0 |
| ENSMUSG00000000701 | Tcl1b5 | 1035 | 0 | 0 | 0 | 0 | 0 | 0 | 0 | 0 | 0 | 0 | 0 | 0 | 0 | 0 | 0 | 0 | 0 | 0 |
| ENSMUSG00000000706 | Btn1a1 | 3925 | 0.270733605 | 0.101848339 | 0.357409524 | 0.510748013 | 0.306572762 | 0.151498803 | 0.277246271 | 0.204341516 | 0 | 0.300322951 | 0.170407495 | 0 | 0.012568641 | 0 | 0.419888468 | 0 | 0.234351879 | 0.196210827 |
| ENSMUSG00000000708 | Kat2b | 4198 | 8.798713189 | 6.872073938 | 8.527015832 | 6.099406126 | 7.624518637 | 7.318412296 | 4.643359628 | 5.031062061 | 5.866969134 | 4.560072778 | 4.904956433 | 6.408563451 | 5.264578118 | 5.210854016 | 3.268251099 | 5.20817933 | 5.331719245 | 5.123525662 |
| ENSMUSG00000000711 | Rab5b | 3160 | 22.39592345 | 25.11117533 | 24.73785603 | 24.40976204 | 25.87852478 | 25.82701019 | 26.66578518 | 26.84345751 | 22.33977469 | 27.93231535 | 21.74062984 | 23.86296207 | 26.0241475 | 19.76813213 | 24.48624113 | 23.33788287 | 23.35963539 | 25.72894295 |
| ENSMUSG00000000724 | Cryba1 | 1908 | 0 | 0 | 0 | 0 | 0 | 0 | 0 | 0 | 0 | 0 | 0 | 0 | 0.025855303 | 0 | 0 | 0 | 0 | 0 |

| ENSMUSG00000000730 | Dnmt3l | 2970 | 0 | 0 | 0 | 0 | 0 | 0.050053266 | 0 | 0 | 0 | 0.158756577 | 0.032171689 | 0 | 0 | 0 | 0 | 0.025784921 | 0 | 0 |
| --- | --- | --- | --- | --- | --- | --- | --- | --- | --- | --- | --- | --- | --- | --- | --- | --- | --- | --- | --- | --- |
| ENSMUSG00000000731 | Aire | 4111 | 0.661720084 | 1.337053648 | 1.106084129 | 0.587383933 | 0.854689961 | 0.819651151 | 0.414316819 | 0.483095336 | 0.45083756 | 0.481714799 | 0.650789996 | 0.488777391 | 0.287999517 | 0.610065852 | 1.202672514 | 0.707877928 | 0.84838069 | 0.548619162 |
| ENSMUSG00000000732 | Icosl | 2834 | 4.094535305 | 5.078041744 | 6.776390808 | 4.019146466 | 4.993220028 | 5.228040699 | 2.053443722 | 4.08337965 | 3.269924505 | 3.443964564 | 5.023619487 | 3.308761932 | 3.655505514 | 4.965616965 | 4.012572133 | 5.080193501 | 4.287027565 | 4.503215512 |
| ENSMUSG00000000738 | Spg7 | 3939 | 23.03847444 | 23.52791869 | 20.72975863 | 24.92613621 | 21.44491649 | 21.61248954 | 25.04364955 | 23.86176753 | 22.28191548 | 26.07114285 | 26.99849139 | 20.60319203 | 28.81765465 | 30.24348417 | 23.44064147 | 20.48192683 | 22.51511831 | 30.52802927 |
| ENSMUSG00000000739 | Sult5a1 | 2053 | 60.72463774 | 57.847281 | 61.23858032 | 68.64116421 | 69.06802101 | 65.19334311 | 65.61090936 | 72.88740021 | 77.23725557 | 75.42275183 | 92.24546294 | 66.74498376 | 49.69235505 | 43.72938184 | 42.44583084 | 43.90457371 | 55.61326764 | 46.3008945 |
| ENSMUSG00000000740 | Rpl13 | 2348 | 68.39205921 | 59.94334671 | 56.07868383 | 57.12594315 | 62.82978963 | 54.1112474 | 75.01920939 | 59.37064258 | 62.74453445 | 60.80593248 | 55.30338039 | 61.47329789 | 97.88645766 | 99.53434742 | 70.96213459 | 61.17038687 | 53.91471834 | 79.3742988 |
| ENSMUSG00000000743 | Chmp1a | 3395 | 55.35062834 | 59.34503218 | 52.94728683 | 64.01360279 | 58.52388244 | 58.32480806 | 59.72964415 | 52.1306606 | 46.03912439 | 54.59482018 | 52.0247486 | 53.95788059 | 59.12564695 | 64.21459387 | 57.80353776 | 61.10707243 | 64.32496016 | 68.29554782 |
| ENSMUSG00000000751 | Rpa1 | 6612 | 9.032028481 | 12.22782732 | 11.59589317 | 12.07243643 | 14.5880785 | 11.39143301 | 14.49720017 | 11.5928844 | 11.53621101 | 13.47774186 | 11.92206314 | 11.70845796 | 11.07207581 | 11.56887658 | 13.78370261 | 12.91410383 | 14.76941992 | 13.51932826 |
| ENSMUSG00000000753 | Serpinf1 | 2712 | 0.015673 | 0 | 0 | 0.03359959 | 0 | 0 | 0.069782712 | 0.042248233 | 0.030373537 | 0 | 0 | 0 | 0.054570705 | 0 | 0 | 0.2541412 | 0.042396346 | 0 |
| ENSMUSG00000000759 | Tubgcp3 | 9566 | 5.754150424 | 6.501690728 | 4.900057591 | 6.601255222 | 6.807703609 | 5.397652978 | 6.236806202 | 5.94884796 | 6.014798507 | 5.683123652 | 5.483682251 | 6.634163533 | 5.456112114 | 5.321212605 | 5.388160822 | 5.972149931 | 7.400028265 | 6.676309106 |
| ENSMUSG00000000766 | Oprm1 | 8203 | 0 | 0 | 0 | 0 | 0 | 0 | 0 | 0 | 0 | 0 | 0 | 0 | 0 | 0 | 0 | 0 | 0 | 0 |
| ENSMUSG00000000776 | Polr3d | 2040 | 20.33580971 | 15.33372925 | 14.60690917 | 14.76267172 | 16.42147984 | 12.19385895 | 13.45164401 | 17.59845993 | 15.86892225 | 15.41643092 | 18.66502054 | 16.58053506 | 16.37142549 | 19.98917094 | 20.33821901 | 13.40171259 | 20.59099089 | 23.27100936 |
| ENSMUSG00000000782 | Tcf7 | 4136 | 10.96543105 | 10.44652413 | 13.04073654 | 11.45635544 | 11.31146755 | 9.608629674 | 12.0111975 | 10.99786056 | 13.3637099 | 10.46526731 | 10.26884383 | 10.7690761 | 13.78812775 | 14.00284743 | 14.03602327 | 14.50710392 | 14.40016088 | 13.19367357 |
| ENSMUSG00000000787 | Ddx3x | 3287 | 87.35091697 | 91.25320948 | 80.83839435 | 82.14017303 | 86.96546841 | 87.99492117 | 72.30045216 | 85.72666411 | 65.78314808 | 77.31739901 | 73.51558868 | 79.28303274 | 64.7303192 | 71.35462164 | 75.38368029 | 73.79707876 | 85.09440669 | 78.17083587 |
| ENSMUSG00000000791 | Il12rb1 | 2991 | 0.454752803 | 0.111377112 | 0.129384234 | 0.10662899 | 0.434490785 | 0.497018391 | 0.442913745 | 0.114921973 | 0.302943279 | 0.29951968 | 0.047918715 | 0.335901681 | 0.214414886 | 0.465838389 | 0.44080568 | 0.102415533 | 0.038441622 | 0.294264697 |
| ENSMUSG00000000792 | Slc5a5 | 2913 | 0.029183094 | 0.068615642 | 0.265697386 | 0.093843552 | 0.363509207 | 0.017010894 | 0.064967633 | 0.131110204 | 0.678665553 | 0.226608255 | 0.246009055 | 0.459861278 | 0.524987791 | 0.015943731 | 0 | 0.184026263 | 0.197354773 | 0.03776801 |
| ENSMUSG00000000794 | Kcnn3 | 3072 | 0.083017922 | 0 | 0 | 0 | 0.031335888 | 0 | 0 | 0 | 0 | 0 | 0 | 0 | 0 | 0 | 0 | 0 | 0.174664113 | 0 |
| ENSMUSG00000000804 | Usp32 | 8579 | 4.003285025 | 4.34904319 | 3.670727155 | 3.844993134 | 3.360650691 | 3.685119947 | 3.16557614 | 3.579285643 | 3.71586003 | 3.511982676 | 2.689742971 | 3.662923189 | 3.685949292 | 2.214201159 | 3.357021514 | 4.789115485 | 2.92618306 | 2.718715614 |
| ENSMUSG00000000805 | Car4 | 2211 | 0.807425325 | 0.467073596 | 0.787628717 | 0.267884928 | 0.217693006 | 0.941300231 | 43.52509676 | 30.78193627 | 28.74300942 | 14.67195108 | 6.352708183 | 20.32184911 | 3.681486364 | 14.47307834 | 5.105936822 | 1.524004275 | 1.438752891 | 7.936637288 |
| ENSMUSG00000000811 | Txnrd3 | 2820 | 21.04156941 | 24.66571742 | 26.27952075 | 24.41231844 | 24.08302989 | 21.38499176 | 28.06883399 | 23.56552489 | 23.85020576 | 25.14704174 | 28.68191656 | 23.09818377 | 28.02472747 | 27.24061012 | 33.18040192 | 32.28902993 | 32.10167138 | 33.22003856 |
| ENSMUSG00000000817 | Fasl | 2382 | 0 | 0 | 0 | 0 | 0 | 0 | 0 | 0 | 0 | 0 | 0 | 0 | 0 | 0 | 0 | 0 | 0 | 0 |
| ENSMUSG00000000823 | Znf512b | 9357 | 6.050752856 | 6.746599823 | 7.366921102 | 8.228937152 | 6.177881834 | 7.371743629 | 7.023331306 | 6.751120399 | 7.51806871 | 6.938817825 | 7.086848602 | 6.722694425 | 5.878496074 | 6.139931185 | 6.838304617 | 6.273324906 | 6.533124245 | 7.283988803 |
| ENSMUSG00000000826 | Dnajc5 | 2094 | 76.95182537 | 101.4341039 | 93.60532235 | 101.0872073 | 94.5629102 | 98.37187845 | 94.03790339 | 88.0395064 | 95.13809778 | 111.4819159 | 99.10908292 | 90.76042555 | 87.21430647 | 85.01441593 | 84.41006436 | 86.41901174 | 101.251707 | 86.95326803 |
| ENSMUSG00000000827 | Tpd52l2 | 5140 | 47.70668492 | 50.29339672 | 48.88173476 | 46.85519073 | 47.59816498 | 49.01285944 | 45.49933306 | 48.39438071 | 48.21386485 | 50.35218097 | 52.18995951 | 50.73361393 | 47.46997332 | 50.49213236 | 46.45194871 | 53.62174943 | 51.21109088 | 42.31634397 |
| ENSMUSG00000000838 | Fmr1 | 8279 | 8.368575544 | 10.01921809 | 10.53478382 | 9.437998676 | 9.64498869 | 7.691177998 | 10.59520555 | 9.872175522 | 6.01952942 | 8.576997114 | 9.186826174 | 9.843080973 | 7.400681394 | 6.945015121 | 8.699156866 | 6.567539861 | 11.41132043 | 8.033096996 |
| ENSMUSG00000000861 | Bcl11a | 13496 | 0.062989295 | 0.051835416 | 0.007168573 | 0.016879462 | 0.067761303 | 0.044059929 | 0.098159085 | 0.053768201 | 0.070190417 | 0.059392557 | 0.056638955 | 0 | 0.204696752 | 0.203038023 | 0.088533278 | 0.079441094 | 0.122112535 | 0.04483552 |
| ENSMUSG00000000869 | Il4 | 1220 | 0 | 0 | 0.079300869 | 0 | 0 | 0 | 0 | 0 | 0 | 0.231888705 | 0 | 0 | 0.202179989 | 0 | 0 | 0 | 0.094244992 | 0 |
| ENSMUSG00000000876 | Pxmp4 | 3109 | 12.19511644 | 11.65790573 | 11.34265154 | 10.6392146 | 9.660443991 | 10.29625793 | 10.94172279 | 10.93317406 | 10.8762076 | 12.17819708 | 13.29216439 | 11.34625241 | 12.44008464 | 10.29269097 | 11.59580618 | 10.11147915 | 12.52477248 | 11.87234179 |
| ENSMUSG00000000881 | Dlg3 | 6526 | 7.809332831 | 7.856044162 | 7.59033023 | 10.33955052 | 8.186704761 | 8.428368721 | 8.852096379 | 7.157417735 | 7.743771811 | 7.882534057 | 8.023499003 | 6.748170055 | 7.211561306 | 5.814406895 | 8.662049446 | 7.252097877 | 7.54662757 | 6.262912396 |
| ENSMUSG00000000884 | Gnb1l | 4178 | 1.800721914 | 1.626575017 | 1.57462904 | 1.722988273 | 1.670447327 | 1.613013826 | 1.653339187 | 1.855686378 | 1.537840225 | 2.144239736 | 1.486535328 | 1.830240731 | 1.971859786 | 2.28996697 | 2.830264849 | 2.016259844 | 2.495153843 | 2.514777258 |
| ENSMUSG00000000889 | Dbh | 2398 | 0 | 0 | 0 | 0 | 0 | 0 | 0.808932375 | 0.079633867 | 0 | 0.117975071 | 0.179305516 | 0.069827768 | 0 | 0 | 0.137453064 | 0 | 0 | 0 |
| ENSMUSG00000000901 | Mmp11 | 2264 | 6.233091184 | 4.517251999 | 4.700608073 | 6.258606355 | 3.72044463 | 3.786494218 | 3.17646961 | 1.923115669 | 2.055687403 | 3.498815436 | 3.882770477 | 3.747341304 | 1.786756721 | 2.892498375 | 2.420409423 | 4.617197801 | 3.876643989 | 1.603622369 |
| ENSMUSG00000000902 | Smarcb1 | 7032 | 20.59373361 | 19.50358156 | 20.70596223 | 18.97728937 | 19.2883619 | 19.40674464 | 18.19304377 | 17.55916339 | 21.75880348 | 19.10971335 | 20.30707493 | 18.08135372 | 16.55621797 | 18.72425878 | 19.72190517 | 18.85670839 | 18.12214693 | 19.68187038 |
| ENSMUSG00000000903 | Vpreb3 | 856 | 0 | 0.15566773 | 0 | 0 | 0 | 0.057888707 | 0.331630927 | 0.17846917 | 0.096230177 | 0.055082597 | 0.279059337 | 0 | 0 | 0.434056891 | 0 | 0.134196054 | 0.626831959 | 0 |
| ENSMUSG00000000915 | Hip1r | 4878 | 21.87125704 | 29.84365472 | 25.1288491 | 26.45118441 | 25.75324329 | 30.24159243 | 29.27217405 | 28.17844532 | 31.97485346 | 35.86082597 | 25.36636787 | 24.77263417 | 32.79692655 | 40.09349159 | 36.7925333 | 36.42238998 | 34.42138309 | 34.96991389 |
| ENSMUSG00000000916 | Nsun5 | 3239 | 5.524754278 | 6.705775558 | 6.347252362 | 7.28639116 | 4.874118852 | 6.547875879 | 5.828267642 | 5.36509512 | 6.103589863 | 5.997557809 | 7.197956078 | 5.342036246 | 6.442544306 | 11.90138701 | 5.177225455 | 6.773855524 | 6.792001983 | 5.82529287 |
| ENSMUSG00000000934 | Top1mt | 1889 | 5.08532016 | 6.154685062 | 4.097281555 | 4.727350286 | 3.363374232 | 5.377612696 | 6.512068037 | 4.549121515 | 6.213952881 | 4.592763049 | 7.309138698 | 7.623314424 | 4.62241893 | 4.155135399 | 5.387392433 | 4.520278163 | 5.762132515 | 6.72689447 |
| ENSMUSG00000000938 | Hoxa10 | 4974 | 2.931096779 | 5.116817689 | 2.713352426 | 2.033484488 | 5.36089379 | 2.998667633 | 0 | 0 | 0.008280361 | 0.009479434 | 0.009604937 | 0.179543748 | 0.009917957 | 0.028012115 | 0 | 0.038490759 | 0.05393729 | 0 |
| ENSMUSG00000000942 | Hoxa4 | 1982 | 0.064336795 | 0.033615433 | 0.195251384 | 0.114937044 | 0.121422613 | 0.225012413 | 0.1193559 | 0 | 0.02078028 | 0.023789457 | 0.433879542 | 0 | 0.746699051 | 0.304628222 | 0.041575738 | 0.115915058 | 0 | 0.277543424 |
| ENSMUSG00000000948 | Snrpn | 2185 | 15.69870803 | 14.33140529 | 14.87735121 | 15.20091594 | 16.2128585 | 16.6914471 | 19.35813957 | 17.65415397 | 16.43690698 | 14.78179942 | 16.85789153 | 16.47647706 | 21.44865969 | 20.97954886 | 17.97028959 | 17.83974294 | 18.45275256 | 21.44977525 |
| ENSMUSG00000000957 | Mmp14 | 2587 | 107.09267 | 112.5581787 | 103.8151684 | 110.8117862 | 105.8083687 | 116.612695 | 75.84293758 | 107.4318177 | 88.07259575 | 97.72789751 | 78.96626303 | 97.49941215 | 64.26306966 | 59.78307345 | 69.64607805 | 76.47760667 | 80.60818765 | 62.13243519 |

| ENSMUSG00000000958 | Slc7a7 |  | 2259 | 0.978428133 | 1.253473222 | 0.663824454 | 0.766409776 | 1.470164999 | 0.789684997 | 1.424197506 | 0.862245473 | 1.239788876 | 1.064491309 | 1.83993864 | 0.815368245 | 2.05276681 | 1.007419337 | 0.893703514 | 1.101766039 | 0.373253002 | 0.462670663 |
| --- | --- | --- | --- | --- | --- | --- | --- | --- | --- | --- | --- | --- | --- | --- | --- | --- | --- | --- | --- | --- | --- |
| ENSMUSG00000000959 | Oxa1l |  | 2524 | 21.08418399 | 19.81087727 | 20.12369527 | 20.9934606 | 21.31992498 | 20.79094487 | 20.35719862 | 24.01400262 | 24.68906432 | 21.33363832 | 25.76137817 | 23.37446726 | 22.96553201 | 24.56531564 | 21.28638228 | 24.28813885 | 24.35633131 | 22.0777437 |
| ENSMUSG00000000976 | Heatr6 |  | 6626 | 6.671503632 | 5.987874587 | 5.760144197 | 6.786711554 | 6.167220524 | 6.244571768 | 6.719171577 | 5.666032533 | 6.483177775 | 4.959861179 | 5.948436575 | 5.517545879 | 6.797470642 | 6.287405129 | 6.354963797 | 7.333350557 | 7.062542789 | 6.326130293 |
| ENSMUSG00000000982 | Ccl3 |  | 763 | 0 | 0 | 0 | 0 | 0 | 0 | 0 | 0.700778026 | 0.377857943 | 0.185389397 | 0 | 0 | 0.387931197 | 1.400018362 | 0.215997672 | 0 | 0 | 0 |
| ENSMUSG00000000983 | Expi |  | 823 | 1.187872477 | 1.740527888 | 3.291516039 | 10.18618731 | 2.105406139 | 3.793222619 | 0 | 0 | 0.050044369 | 0 | 0 | 0 | 0 | 0 | 0.050062644 | 0 | 0 | 0 |
| ENSMUSG00000001014 | Icam4 |  | 904 | 0 | 0 | 0 | 0 | 0 | 0 | 0 | 0 | 0 | 0 | 0 | 0 | 0 | 0 | 0 | 0 | 0 | 0 |
| ENSMUSG00000001016 | Ilf2 |  | 1959 | 76.50497737 | 80.58693502 | 81.41272567 | 76.02810308 | 77.83661775 | 79.5524998 | 77.91255856 | 81.62919289 | 88.19675019 | 89.05441657 | 82.18561004 | 86.64391522 | 77.9892536 | 78.28401049 | 92.8139184 | 91.78800018 | 84.92825669 | 82.49961999 |
| ENSMUSG00000001017 | 2500003M10R |  | 4779 | 25.65964278 | 26.39797665 | 26.56039834 | 25.27355692 | 27.92840015 | 25.39331336 | 27.07683132 | 27.68329817 | 25.7340314 | 25.26740972 | 27.9112124 | 25.93985909 | 21.83239277 | 24.94705426 | 24.98474764 | 26.94524225 | 25.59898299 | 26.39378148 |
| ENSMUSG00000001018 | Snapin |  | 3836 | 33.99527634 | 34.05105793 | 31.66473794 | 35.67919113 | 34.31720835 | 34.69724776 | 33.74543523 | 31.06368496 | 34.74441215 | 29.65970984 | 30.21429856 | 30.48326724 | 27.99676327 | 30.83761485 | 30.57894411 | 32.57097288 | 31.4023856 | 28.10684285 |
| ENSMUSG00000001020 | S100a4 |  | 619 | 0.206002469 | 0.16145183 | 0.390739341 | 0.220812816 | 0.07775755 | 0 | 1.987285383 | 5.367914393 | 1.53035519 | 0 | 2.701330447 | 0.090170699 | 1.19544226 | 1.275524209 | 0.865299235 | 0.433011715 | 0 | 0.266603102 |
| ENSMUSG00000001021 | S100a3 |  | 709 | 0.119901766 | 0.046985746 | 0.409366971 | 0 | 0 | 0.139782041 | 0 | 0.161603959 | 0.755182941 | 0.332515538 | 0.404301482 | 0 | 0.139159146 | 0.327532351 | 0.348673252 | 0 | 0.054056836 | 0 |
| ENSMUSG00000001023 | S100a5 |  | 429 | 0 | 0 | 0 | 0 | 0 | 0 | 0 | 0.089026579 | 0 | 0 | 0 | 0 | 0 | 0 | 0 | 0 | 0 | 0 |
| ENSMUSG00000001025 | S100a6 |  | 716 | 332.2052585 | 320.5668173 | 344.3573802 | 274.8934519 | 362.8044581 | 361.7487967 | 41.96026693 | 42.83309935 | 36.87228576 | 27.78993963 | 39.63453259 | 46.77290161 | 76.54715097 | 50.85497837 | 52.42265002 | 93.74781393 | 52.83247911 | 54.62496492 |
| ENSMUSG00000001027 | Scn4a |  | 7015 | 0 | 0 | 0 | 0 | 0 | 0 | 0.04046701 | 0 | 0.035227241 | 0.047049882 | 0 | 0 | 0 | 0 | 0 | 0 | 0 | 0 |
| ENSMUSG00000001029 | Icam2 |  | 1897 | 0 | 0 | 0 | 0 | 0 | 0 | 0 | 0 | 0 | 0.074566215 | 0.503689599 | 0.088269366 | 0 | 0.220346224 | 0 | 0.141293754 | 0 | 0 |
| ENSMUSG00000001034 | Mapk7 |  | 4299 | 10.16406628 | 9.577747675 | 8.101638022 | 7.291462783 | 9.270349569 | 7.377006162 | 4.743373952 | 8.271022712 | 9.475102137 | 9.520076861 | 7.301267206 | 8.114628778 | 4.991715286 | 5.585390365 | 5.970823289 | 7.748971491 | 6.427834396 | 6.845748336 |
| ENSMUSG00000001036 | Epn2 |  | 5209 | 20.42435316 | 18.72531278 | 18.13659143 | 19.05884346 | 16.65998433 | 17.79864936 | 14.3691031 | 10.76338006 | 11.53601968 | 13.65007881 | 11.67546979 | 15.11919753 | 12.47266943 | 11.87627652 | 13.85777038 | 14.89283367 | 13.14086509 | 12.51407016 |
| ENSMUSG00000001039 | B9d1 |  | 1654 | 11.38439721 | 8.922377351 | 8.686178063 | 12.1753214 | 9.981408596 | 8.987799319 | 13.21551249 | 11.22219321 | 13.47152662 | 12.22953549 | 12.6802943 | 13.09339606 | 14.22691931 | 12.86057559 | 11.90710019 | 12.2928129 | 10.31148859 | 11.74015397 |
| ENSMUSG00000001052 | Sec24b |  | 6548 | 8.295909432 | 9.401684257 | 9.108821461 | 7.361573749 | 8.203302812 | 9.44438172 | 10.00733969 | 8.229914446 | 8.076280733 | 7.964061974 | 7.128303976 | 8.413264962 | 8.807118402 | 6.823337213 | 7.588435615 | 7.239427606 | 10.14932786 | 8.274888534 |
| ENSMUSG00000001053 | N4bp3 |  | 2721 | 9.278968967 | 11.43485601 | 9.493371999 | 12.30700757 | 10.77263562 | 14.40507617 | 9.580774013 | 10.51308688 | 11.18590046 | 12.70175137 | 12.83480141 | 15.40520493 | 11.63950419 | 11.23124201 | 11.37168999 | 15.18396376 | 11.11335842 | 11.72557218 |
| ENSMUSG00000001054 | Rmnd5b |  | 2624 | 31.16614279 | 34.16349022 | 34.03107357 | 34.41386809 | 34.46641943 | 33.67093139 | 28.59676402 | 30.60923104 | 27.10713137 | 25.96523102 | 29.05824456 | 28.86503587 | 28.70801741 | 28.53196222 | 27.00711743 | 29.17032172 | 32.39623724 | 34.75804076 |
| ENSMUSG00000001056 | Nhp2 |  | 1349 | 44.80530787 | 41.5114716 | 37.90275877 | 39.41418735 | 41.35277949 | 39.59810734 | 42.78833824 | 41.16512461 | 43.75113201 | 39.77576005 | 39.55865724 | 37.40352778 | 51.48950298 | 57.28907444 | 47.218388 | 44.56356829 | 38.29788601 | 48.64778675 |
| ENSMUSG00000001062 | 1300018I17Ri | k | 5563 | 4.508008962 | 5.940390269 | 5.669520365 | 6.183460089 | 5.745029178 | 5.602852673 | 6.438198445 | 4.634167108 | 6.122820157 | 6.661954478 | 5.410430315 | 5.789257112 | 4.903927782 | 6.645603732 | 5.03632177 | 5.00400352 | 5.174015634 | 5.933033262 |
| ENSMUSG00000001065 | Zfp276 |  | 5861 | 3.444797579 | 3.455765174 | 3.936900525 | 3.389287721 | 3.005678905 | 3.060585148 | 3.559954173 | 2.906297811 | 4.244438751 | 2.944404947 | 3.700704854 | 2.84744636 | 3.728721951 | 2.646702813 | 2.66428761 | 2.992168263 | 3.328456769 | 3.040940211 |
| ENSMUSG00000001076 | C1ql4 |  | 872 | 0 | 0 | 0 | 0 | 0 | 0 | 0 | 0 | 0 | 0 | 0 | 0 | 0 | 0 | 0 | 0 | 0 | 0 |
| ENSMUSG00000001082 | Mfsd10 |  | 2961 | 20.90089034 | 17.60711903 | 18.75474936 | 22.24966907 | 17.97835447 | 16.90248596 | 18.51921475 | 20.03134108 | 18.5833114 | 18.24880308 | 21.89483913 | 17.54960549 | 13.77828287 | 17.86552365 | 18.13091095 | 16.08696913 | 14.65226886 | 13.37607458 |
| ENSMUSG00000001089 | Luzp1 |  | 4366 | 14.59350867 | 13.39840638 | 12.30943477 | 10.09105128 | 9.877737885 | 11.47453359 | 9.091923413 | 10.36600936 | 10.01834167 | 9.946357696 | 7.988025582 | 10.34238915 | 8.926297446 | 8.510139696 | 8.880133782 | 12.32210339 | 10.23556166 | 10.23073629 |
| ENSMUSG00000001095 | Slc13a2 |  | 2400 | 0.15939441 | 0.555214904 | 0.161245101 | 0.379675369 | 0.320879491 | 0.53682128 | 0.532267638 | 0.47740503 | 0.223093627 | 0.078584505 | 0.039812465 | 0.11628263 | 0.883863518 | 1.122398779 | 0.480684819 | 0 | 0.143723613 | 0.068761383 |
| ENSMUSG00000001098 | Kctd10 |  | 4695 | 5.640196947 | 7.819128739 | 5.800702362 | 8.005934308 | 8.057868371 | 8.51737082 | 6.832374082 | 5.279418352 | 6.631950142 | 5.854922258 | 5.433829143 | 5.611287059 | 5.684465867 | 6.331036407 | 6.397430084 | 5.472416947 | 5.526496912 | 5.881697886 |
| ENSMUSG00000001100 | Poldip2 |  | 3524 | 25.30529492 | 27.15889475 | 25.46336516 | 27.75810505 | 26.27860986 | 25.43725739 | 26.81141312 | 30.04237783 | 25.22148156 | 26.90694218 | 33.37739721 | 25.92799082 | 26.72378833 | 30.33890158 | 28.43421791 | 29.22864186 | 23.75273338 | 27.44211397 |
| ENSMUSG00000001103 | Sebox |  | 1432 | 0 | 0 | 0 | 0 | 0 | 0 | 0 | 0 | 0 | 0 | 0 | 0 | 0 | 0 | 0 | 0 | 0 | 0 |
| ENSMUSG00000001105 | Ift20 |  | 1668 | 51.65347231 | 47.41295616 | 46.66247622 | 44.46845334 | 45.30402887 | 47.56230603 | 45.9795279 | 46.8704123 | 53.97705243 | 49.5251991 | 42.24704062 | 43.30063992 | 56.72578977 | 67.68919451 | 55.38002896 | 50.31955121 | 49.03376354 | 53.29419452 |
| ENSMUSG00000001119 | Col6a1 |  | 4358 | 0 | 0 | 0 | 0 | 0 | 0 | 0 | 0.026291236 | 0 | 0.075735411 | 0 | 0 | 0.033959558 | 0 | 0 | 0 | 0 | 0 |
| ENSMUSG00000001120 | Pcbp3 |  | 3578 | 5.880397466 | 7.690455736 | 6.300185899 | 6.32863025 | 6.726093302 | 5.622808785 | 6.99508305 | 7.557356317 | 5.018815224 | 6.457195248 | 6.75632448 | 7.846640101 | 5.349576273 | 8.359416512 | 6.137626968 | 6.881179586 | 6.41628056 | 4.689149679 |
| ENSMUSG00000001123 | Lgals9 |  | 2688 | 6.98931838 | 6.419672323 | 5.254862673 | 7.508758423 | 7.108769976 | 8.129745353 | 2.886636661 | 1.662392515 | 2.72738088 | 2.578554084 | 2.043943536 | 3.17700758 | 4.881804314 | 2.660859173 | 4.19986098 | 5.84045723 | 3.279407845 | 4.931992083 |
| ENSMUSG00000001127 | Araf |  | 4311 | 41.4699073 | 44.96583889 | 41.49508589 | 47.43167872 | 46.12224001 | 42.20775637 | 48.94793505 | 47.0959896 | 41.84572931 | 41.16799978 | 46.67783 | 44.435016 | 44.54863233 | 49.65455782 | 47.35646241 | 48.36287574 | 48.48796644 | 48.94802445 |
| ENSMUSG00000001128 | Cfp |  | 1835 | 0.416944506 | 0.199695824 | 0.34270076 | 0.347604698 | 0.23606938 | 0.324050574 | 0.747720811 | 2.351902709 | 1.436477934 | 0.590989741 | 1.536088583 | 0.121669019 | 0.914051873 | 1.341436856 | 0.359250624 | 0.375602688 | 0.167090123 | 0.569576582 |
| ENSMUSG00000001131 | Timp1 |  | 985 | 2.243928075 | 0.777864535 | 1.718856409 | 1.063861949 | 1.465946914 | 0.653995469 | 0.288199059 | 0.426514139 | 0 | 0.33508114 | 0 | 0.679987768 | 1.201995954 | 0 | 0.125486972 | 0.466484557 | 0.389099461 | 0 |
| ENSMUSG00000001134 | Uxt |  | 1631 | 13.00434264 | 12.5408443 | 12.87192653 | 11.92799873 | 13.25029658 | 11.4235609 | 13.57592504 | 10.35011764 | 12.67665905 | 12.8645389 | 13.97219816 | 11.66961431 | 14.88124053 | 15.74713693 | 12.75707279 | 13.31132704 | 12.2662949 | 13.05243673 |
| ENSMUSG00000001138 | Cnnm3 |  | 5605 | 4.716899106 | 5.854273114 | 6.472818513 | 6.137125508 | 5.56458279 | 6.215088618 | 6.761365891 | 6.221170989 | 6.275340671 | 6.847577601 | 5.011895733 | 5.008970255 | 4.559131695 | 4.085091005 | 5.998299662 | 4.303850609 | 5.962628171 | 4.789374501 |
| ENSMUSG00000001143 | Lman2l |  | 7302 | 6.280893582 | 6.14066771 | 7.863514179 | 6.763649967 | 6.967329953 | 6.677607483 | 6.6154814 | 6.511851684 | 7.501789368 | 5.805050976 | 6.712833114 | 6.619606108 | 6.1614227 | 5.527240748 | 6.105188096 | 5.348729054 | 6.487442204 | 6.765020246 |

| ENSMUSG00000001150 | Mcm3ap | 6640 | 3.648787701 | 5.097274176 | 3.897566075 | 4.761952525 | 3.820109002 | 4.440342843 | 3.512823902 | 2.605596128 | 5.31578976 | 3.784838078 | 3.280930883 | 4.043273148 | 4.457703368 | 3.420355231 | 4.318717304 | 3.021728656 | 3.157301868 | 4.63103775 |
| --- | --- | --- | --- | --- | --- | --- | --- | --- | --- | --- | --- | --- | --- | --- | --- | --- | --- | --- | --- | --- |
| ENSMUSG00000001151 | Pcnt | 10004 | 5.328017867 | 5.194733605 | 4.463091615 | 5.064362385 | 4.373442484 | 4.918618997 | 4.92800994 | 4.753052878 | 4.401078104 | 4.967697045 | 4.656195973 | 5.384057816 | 4.482478289 | 2.883024617 | 3.908464268 | 5.817845187 | 4.072456378 | 3.1947512 |
| ENSMUSG00000001155 | Ftcd | 3285 | 0.129391708 | 0.050704557 | 0.04417674 | 0.01386942 | 0 | 0.105591822 | 1.353848347 | 0.964983074 | 0.514047838 | 0.473660033 | 0.189063763 | 0.696633841 | 1.561801947 | 3.845598712 | 1.605418312 | 0.314717321 | 0.350011844 | 2.696032323 |
| ENSMUSG00000001156 | Mxd1 | 4656 | 4.162878065 | 4.493234014 | 4.623329254 | 5.685345094 | 5.551297897 | 5.012744312 | 3.078982327 | 3.231917214 | 3.432209647 | 4.121635787 | 3.847854259 | 2.577398509 | 2.70181248 | 4.209494175 | 4.30067787 | 3.125092528 | 3.300868784 | 2.646486234 |
| ENSMUSG00000001157 | Gmcl1 | 3808 | 16.01757553 | 16.70893591 | 19.21984019 | 18.96382754 | 17.01301714 | 18.34804473 | 19.02198305 | 16.74929412 | 17.47831131 | 16.04708808 | 18.73109585 | 15.74212753 | 12.98071983 | 12.56234507 | 12.55089413 | 15.94771621 | 14.52333152 | 11.28206732 |
| ENSMUSG00000001158 | Snrnp27 | 1936 | 51.63851964 | 44.49749197 | 48.74832527 | 38.100894 | 46.19272426 | 46.63485567 | 49.1944332 | 44.62356107 | 43.01608206 | 43.91152788 | 45.35659798 | 40.16075309 | 43.47120396 | 40.83049418 | 41.94641873 | 42.28580507 | 42.78054112 | 39.60882994 |
| ENSMUSG00000001166 | Oas1c | 3433 | 4.283947249 | 3.570971475 | 3.987681063 | 3.782376241 | 3.294786502 | 3.348742846 | 1.529772025 | 3.415399807 | 4.223027541 | 4.326382618 | 2.658030023 | 2.422526573 | 2.859613032 | 3.652753742 | 2.436328531 | 2.710345936 | 3.36038898 | 2.48366197 |
| ENSMUSG00000001168 | Oas1h | 1981 | 0 | 0.016816201 | 0 | 0 | 0 | 0 | 0 | 0 | 0 | 0 | 0 | 0 | 0 | 0 | 0 | 0 | 0 | 0 |
| ENSMUSG00000001173 | Ocrl | 9714 | 3.706185309 | 3.775735694 | 3.859325306 | 2.945474145 | 3.166182749 | 3.376972373 | 4.865695517 | 4.521439445 | 3.599686214 | 3.926798326 | 3.108274013 | 3.17748213 | 8.541927616 | 8.414823328 | 6.514884687 | 3.551558294 | 4.434708433 | 6.155538296 |
| ENSMUSG00000001175 | Calm1 | 4878 | 257.2704638 | 233.7639133 | 229.4718107 | 208.0415542 | 233.6046111 | 237.828741 | 184.8569798 | 176.3286377 | 190.1266835 | 162.8622795 | 175.2042296 | 185.5258617 | 177.6980563 | 138.5705715 | 145.8018159 | 175.2513532 | 148.1038738 | 152.1039015 |
| ENSMUSG00000001211 | Agpat3 | 9772 | 11.40053892 | 10.82021349 | 9.771730344 | 11.84251459 | 11.3483373 | 10.21277174 | 14.14732376 | 13.58542681 | 13.59256174 | 16.95533885 | 11.16150534 | 11.92051656 | 13.50418325 | 15.5320587 | 14.74857169 | 12.05299922 | 12.85648805 | 14.50659722 |
| ENSMUSG00000001225 | Slc26a3 | 5215 | 177.3725102 | 175.5777968 | 196.147013 | 167.9774112 | 180.2247504 | 187.8823013 | 124.6187838 | 127.3714789 | 139.1890995 | 121.0367142 | 127.9251237 | 125.1169886 | 213.4657805 | 209.0028462 | 221.7852497 | 258.4377372 | 205.5435996 | 234.2132705 |
| ENSMUSG00000001227 | Sema6b | 3959 | 1.095106834 | 1.001322155 | 0.708680162 | 0.932165848 | 0.692982988 | 0.863636933 | 0.728990506 | 0.71387668 | 0.561776169 | 0.488299781 | 0.555101816 | 1.071480262 | 0.361360344 | 1.161395466 | 0.624423681 | 0.406215082 | 0.542124937 | 1.19494397 |
| ENSMUSG00000001228 | Uhrf1 | 4188 | 1.461496024 | 1.909048379 | 1.975136984 | 2.154032182 | 2.057214503 | 2.236262332 | 1.638094184 | 0.939306936 | 1.90787585 | 1.891432222 | 1.311872069 | 1.545992564 | 2.014268829 | 1.563661968 | 0.895258258 | 1.865158527 | 1.610656221 | 1.563057235 |
| ENSMUSG00000001229 | Dpp9 | 3611 | 10.6880919 | 12.38970283 | 9.578253726 | 10.6994643 | 9.183853616 | 12.28183233 | 10.28536498 | 11.82473162 | 12.52362069 | 10.36767056 | 11.62951772 | 11.25278382 | 7.185984073 | 7.498449999 | 11.06771233 | 9.10873214 | 7.694977916 | 10.29802164 |
| ENSMUSG00000001240 | Ramp2 | 3294 | 0.038711454 | 0.232603694 | 0.220280193 | 0.428777284 | 0.014611999 | 0.255736634 | 0.488351877 | 0.857995682 | 0.750209759 | 0.58687882 | 0.841210562 | 0.593062596 | 0.134786659 | 0 | 0.025016124 | 0.290608739 | 0.442137001 | 0 |
| ENSMUSG00000001247 | Lsr | 2351 | 31.45853096 | 33.52543927 | 31.29567829 | 37.77051272 | 35.78673015 | 34.37707719 | 20.2452382 | 25.71610932 | 25.77004027 | 27.15527529 | 26.09232101 | 24.02613804 | 21.69681092 | 26.2149315 | 27.37423662 | 28.53472742 | 22.57844373 | 26.97809445 |
| ENSMUSG00000001248 | Gramd1a | 2663 | 36.45581001 | 34.78901946 | 33.47818868 | 41.12983499 | 36.6547282 | 37.2157218 | 32.21099771 | 31.79592795 | 27.4989204 | 34.45560216 | 33.53037829 | 29.5322075 | 37.95760364 | 42.03163748 | 37.34906343 | 43.71139561 | 38.06723819 | 40.77655902 |
| ENSMUSG00000001249 | Hpn | 3272 | 13.61412729 | 12.91994583 | 13.74920025 | 15.5397694 | 12.44486779 | 16.58320393 | 29.97530056 | 28.70266428 | 31.39338946 | 32.81239343 | 28.45763878 | 27.00372673 | 45.71343924 | 40.15596676 | 39.26236286 | 61.23922632 | 48.70438341 | 41.62669567 |
| ENSMUSG00000001260 | Gabrg1 | 5023 | 0 | 0.053056571 | 0 | 0 | 0.019164612 | 0 | 0 | 0 | 0 | 0 | 0 | 0 | 0.206245324 | 0 | 0 | 0 | 0 | 0 |
| ENSMUSG00000001270 | Ckb | 1470 | 24.3175871 | 19.01327772 | 15.79543848 | 23.21443688 | 19.97311118 | 17.09063668 | 28.67727682 | 25.12384566 | 20.06091516 | 27.52061455 | 33.34497102 | 17.80785425 | 41.24416757 | 42.65273332 | 32.45673587 | 54.38829133 | 45.15724234 | 40.30259044 |
| ENSMUSG00000001280 | Sp1 | 8316 | 4.855689902 | 4.454538044 | 4.141649064 | 4.350104522 | 4.896537689 | 4.218775297 | 3.783421295 | 4.790124544 | 5.9035987 | 4.099321604 | 5.067041051 | 4.604322335 | 3.434725842 | 3.579925447 | 3.111421008 | 2.979079243 | 4.166302597 | 3.300810997 |
| ENSMUSG00000001281 | Itgb7 | 2727 | 0.202628269 | 0.097727596 | 0.10643241 | 0.183781257 | 0.017650137 | 0.127198069 | 0.260245759 | 0.420158442 | 2.008729958 | 0.622451528 | 2.01471222 | 0.388888005 | 1.302492865 | 0.681248073 | 0.513697433 | 0.379114925 | 0.014054381 | 0.100860115 |
| ENSMUSG00000001285 | Myg1 | 3050 | 15.39941623 | 11.35915082 | 12.48195685 | 12.51808366 | 13.00350987 | 11.04782257 | 12.44090771 | 10.09281191 | 12.07237544 | 12.10459038 | 12.96972643 | 12.7369512 | 10.99859139 | 9.882692694 | 13.19800659 | 11.03522751 | 9.675819218 | 10.65914166 |
| ENSMUSG00000001288 | Rarg | 5378 | 0.885195187 | 0.706149115 | 0.206878244 | 0.279567583 | 0.080548031 | 0.414628674 | 0.184746422 | 0.411892774 | 0.275699994 | 0.604945802 | 0.328686029 | 0.705739133 | 0.266014429 | 0.310893854 | 0.628212641 | 0.455671044 | 0.220921384 | 0.398913195 |
| ENSMUSG00000001289 | Pfdn5 | 1501 | 81.38566016 | 74.26045573 | 78.92523374 | 67.65860615 | 76.38257307 | 70.84621336 | 85.57889626 | 69.13308283 | 77.48882113 | 63.98799636 | 69.25936686 | 64.25680543 | 87.91664138 | 78.59292603 | 70.87436197 | 82.11689888 | 69.01797504 | 91.95059878 |
| ENSMUSG00000001300 | Efnb2 | 7055 | 5.259676637 | 7.611677602 | 6.033835112 | 5.624900017 | 7.38864257 | 7.009727585 | 3.923163313 | 5.678785276 | 5.411750547 | 4.424346642 | 4.49646668 | 5.023805208 | 4.419244751 | 4.285627341 | 5.226845148 | 5.687959824 | 5.997481471 | 3.508731116 |
| ENSMUSG00000001305 | Rrp15 | 3689 | 5.738025932 | 4.66868157 | 4.301034957 | 4.791999242 | 5.519057661 | 3.976039344 | 4.001506052 | 4.513929913 | 4.744990295 | 4.562971283 | 4.947149401 | 5.295603661 | 4.212402803 | 6.01796524 | 4.836073109 | 5.252113674 | 4.477808069 | 4.652437327 |
| ENSMUSG00000001313 | Rnd2 | 1983 | 3.579608874 | 3.5950375 | 2.707746782 | 1.952944412 | 2.791311761 | 4.198113586 | 5.654616695 | 2.657867641 | 3.240088986 | 2.995959965 | 4.023408001 | 4.475385956 | 3.880877002 | 3.278957254 | 3.44904603 | 4.692192435 | 4.367999546 | 3.439802942 |
| ENSMUSG00000001323 | Srr | 6885 | 3.167052331 | 3.420801193 | 3.210850163 | 3.732233697 | 3.928851286 | 3.677770059 | 4.693472724 | 4.698469838 | 4.636100177 | 3.670701081 | 3.601336739 | 4.337164776 | 3.625555576 | 4.07439779 | 3.716218767 | 3.637190594 | 4.598042301 | 4.106707459 |
| ENSMUSG00000001333 | Sync | 2356 | 6.296394921 | 5.768956215 | 8.294951725 | 8.953634774 | 7.232046256 | 10.20079617 | 8.956474873 | 9.38599363 | 9.894553447 | 8.405473416 | 10.78789385 | 9.736942837 | 5.130016864 | 4.829711974 | 5.473721135 | 5.753342538 | 5.840042694 | 6.420842253 |
| ENSMUSG00000001334 | Fndc5 | 2757 | 0 | 0 | 0 | 0.016525587 | 0 | 0 | 0 | 0.027705769 | 0 | 0.06840871 | 0.017328603 | 0 | 0.089466662 | 0 | 0 | 0 | 0 | 0.019952523 |
| ENSMUSG00000001348 | Acp5 | 1605 | 0.317795709 | 0.456625341 | 0.602785425 | 0.766447475 | 0.2399099 | 0.710101478 | 1.267567099 | 0.571101344 | 1.539682832 | 1.057585867 | 2.798034951 | 0.417313365 | 1.936393015 | 2.37284434 | 0.487744275 | 0.978140127 | 0.382069003 | 0.239915107 |
| ENSMUSG00000001349 | Cnn1 | 2361 | 0 | 0.084657927 | 0 | 0.077189402 | 0.163089957 | 0.293832389 | 0 | 0 | 0 | 0.079882598 | 0 | 0 | 0.083578005 | 0.059014088 | 0.052352676 | 0.340577194 | 0 | 0 |
| ENSMUSG00000001366 | Fbxo9 | 3675 | 35.54242338 | 33.82958401 | 32.99930356 | 35.34545235 | 34.99551021 | 32.25309897 | 35.23668088 | 31.61395595 | 32.26557248 | 32.30625056 | 32.91597139 | 31.19601931 | 31.29053039 | 29.49673468 | 32.62490548 | 30.52829379 | 30.6923243 | 35.65488224 |
| ENSMUSG00000001376 | Ccdc132 | 10045 | 5.378205946 | 6.500076921 | 5.302066393 | 6.844361961 | 7.010154736 | 7.246686474 | 6.895546237 | 6.630915858 | 7.183551579 | 6.435402107 | 6.316092072 | 4.778643089 | 6.364774992 | 6.718094473 | 5.947462025 | 5.858901302 | 6.944137418 | 6.664617509 |
| ENSMUSG00000001379 | Apbb3 | 2048 | 8.115001869 | 12.42727109 | 13.15627754 | 11.34576788 | 12.94955564 | 11.78329163 | 14.62349891 | 12.21485526 | 12.9914498 | 11.74163021 | 12.97015474 | 14.03567687 | 11.08041111 | 13.31185513 | 16.19494751 | 14.37767436 | 12.85652635 | 13.51053744 |
| ENSMUSG00000001380 | Hars | 1974 | 43.90478922 | 40.46824738 | 41.38951002 | 38.17526207 | 41.15840281 | 39.68737173 | 40.38594934 | 36.74132329 | 44.54569967 | 38.26516039 | 37.9731053 | 36.02287444 | 36.18673567 | 33.73901789 | 40.74236935 | 37.4953111 | 31.62793194 | 37.75954388 |
| ENSMUSG00000001383 | Zmat2 | 2251 | 41.95757492 | 40.13530392 | 47.08414261 | 39.20557214 | 38.48843296 | 41.03344975 | 46.89230861 | 39.61761867 | 46.34626585 | 43.02423123 | 37.8209578 | 40.61575092 | 51.30431735 | 49.6421476 | 42.70245664 | 50.16392769 | 44.62604362 | 50.14601821 |
| ENSMUSG00000001403 | Ube2c | 2770 | 0.168793118 | 0.144315787 | 0.034926737 | 0 | 0 | 0.214668882 | 0.034160779 | 0.15166658 | 0.550144795 | 0.051065744 | 0 | 0.120900352 | 0.213712277 | 0.201201823 | 1.978269651 | 1.354685749 | 1.259094952 | 0.635484265 |
| ENSMUSG00000001415 | Smg5 | 4425 | 6.704771269 | 8.52207825 | 8.909475082 | 6.908804689 | 8.10356681 | 7.693271857 | 5.944824742 | 6.999782678 | 7.390303619 | 7.053958318 | 4.25386071 | 4.868891697 | 4.749242206 | 4.450235718 | 6.210494413 | 4.966962406 | 4.90230148 | 4.102374061 |

| ENSMUSG00000001416 | Cct3 | 4575 | 47.62437865 | 50.57006565 | 49.08195146 | 49.62419321 | 51.06718196 | 50.02930632 | 42.45214142 | 45.02946854 | 39.74611303 | 39.6168969 | 41.65558675 | 45.05513485 | 35.24940712 | 46.67757679 | 44.42563393 | 41.79744117 | 44.60091906 | 45.87098189 |
| --- | --- | --- | --- | --- | --- | --- | --- | --- | --- | --- | --- | --- | --- | --- | --- | --- | --- | --- | --- | --- |
| ENSMUSG00000001417 | Rhbg | 2327 | 20.16575605 | 28.30236006 | 33.57251892 | 22.96652557 | 24.11767211 | 25.68139093 | 39.28151942 | 45.67660331 | 60.47886737 | 60.88863914 | 63.1935205 | 53.15320511 | 73.33008245 | 49.09860549 | 66.89277102 | 69.22586392 | 72.48561782 | 69.66559412 |
| ENSMUSG00000001418 | 0610031J06R | 2546 | 72.40571423 | 74.33250277 | 71.53430549 | 74.53328738 | 71.59292809 | 75.94452727 | 78.43944138 | 77.26986048 | 63.947485 | 78.35609799 | 77.59208692 | 68.46516664 | 88.27816776 | 87.85338761 | 90.42980924 | 83.90545903 | 83.39657692 | 88.34730455 |
| ENSMUSG00000001419 | Mef2d | 3903 | 19.61358494 | 26.34816922 | 23.59805324 | 25.26110683 | 22.80192848 | 23.65276522 | 17.95287663 | 25.83344667 | 20.02869765 | 24.86193884 | 21.43324425 | 18.96273856 | 14.62388632 | 20.11030174 | 18.45255437 | 19.90562194 | 23.29233314 | 23.1565366 |
| ENSMUSG00000001420 | Tmem79 | 3547 | 3.990477479 | 4.40477795 | 4.255015921 | 4.187454314 | 3.419578451 | 3.981541886 | 3.268002914 | 4.156263696 | 4.156970015 | 4.586127044 | 4.3235866 | 3.918268955 | 5.591043345 | 6.271981353 | 5.378156295 | 4.641940001 | 4.613850797 | 4.900726734 |
| ENSMUSG00000001424 | Snd1 | 7518 | 30.28168699 | 38.89162976 | 36.37988037 | 37.1918235 | 34.21988986 | 41.6432789 | 31.3592816 | 36.50071978 | 32.61831802 | 35.27208767 | 37.93146145 | 36.11164975 | 31.1162479 | 33.24848076 | 40.7905268 | 35.15829514 | 35.00244141 | 37.36785156 |
| ENSMUSG00000001435 | Col18a1 | 7962 | 71.01813071 | 104.4657552 | 80.37345304 | 104.8842479 | 89.6927093 | 103.0263692 | 59.04874284 | 71.03153665 | 65.02317296 | 79.57347396 | 66.58612337 | 65.41270379 | 43.99722991 | 34.91764721 | 47.5924026 | 42.37359229 | 44.28078438 | 42.04790549 |
| ENSMUSG00000001436 | Slc19a1 | 4637 | 1.329146113 | 1.602065001 | 2.159439462 | 1.552435843 | 1.39091606 | 2.051784526 | 1.173378925 | 2.479170396 | 2.433708285 | 2.094682957 | 1.699993131 | 1.576849644 | 1.372399683 | 2.293658834 | 2.798898017 | 1.544175886 | 1.892758678 | 2.586151664 |
| ENSMUSG00000001440 | Kpnb1 | 6740 | 8.393826305 | 7.018443589 | 7.866081494 | 7.361420962 | 7.526861654 | 6.932971478 | 7.546161698 | 7.145492497 | 9.343350534 | 5.624505255 | 6.386533767 | 8.157036743 | 7.238763529 | 5.195673871 | 6.522560851 | 8.595502819 | 6.255033619 | 6.357877468 |
| ENSMUSG00000001441 | Npepps | 6811 | 13.04923258 | 12.59443586 | 12.99714587 | 13.17129589 | 10.56485478 | 11.909826 | 13.35813854 | 11.39435644 | 12.95279941 | 11.43634735 | 11.93146445 | 13.0053526 | 10.81376486 | 8.980599485 | 10.50757636 | 12.51429923 | 10.17382834 | 8.649941756 |
| ENSMUSG00000001444 | Tbx21 | 2482 | 0 | 0 | 0 | 0 | 0 | 0 | 0 | 0 | 0 | 0 | 0 | 0 | 0 | 0 | 0 | 0 | 0 | 0 |
| ENSMUSG00000001445 | Mrpl10 | 1065 | 71.56035739 | 65.24948107 | 65.22477896 | 65.79613727 | 72.03970547 | 70.81620705 | 77.21073801 | 66.73808533 | 83.22383279 | 66.49798713 | 71.90916286 | 66.4022953 | 59.24462178 | 69.68793582 | 62.94359759 | 71.08031064 | 67.47587483 | 70.24637103 |
| ENSMUSG00000001467 | Cyp51 | 3896 | 10.51719448 | 10.5257117 | 10.87659461 | 9.870000364 | 12.2924189 | 10.72201088 | 10.71092988 | 9.920613766 | 9.514338857 | 10.11755337 | 9.515751483 | 9.039959726 | 13.92841607 | 20.06298744 | 16.15913178 | 13.77911488 | 16.77267357 | 15.09361784 |
| ENSMUSG00000001472 | Tcf25 | 3984 | 124.346844 | 129.6644203 | 122.8158282 | 126.7795526 | 123.0601844 | 124.2052203 | 108.0804947 | 124.0582027 | 122.4533833 | 112.6101761 | 126.5005113 | 123.5818172 | 103.2329805 | 105.5717007 | 114.7934916 | 108.0767772 | 115.1039514 | 110.8328563 |
| ENSMUSG00000001473 | Tubb6 | 1753 | 0.484942111 | 0.931164756 | 0.52429953 | 0.909661467 | 0.384396424 | 0.254406504 | 2.806913068 | 3.725556652 | 1.76211562 | 0.322765795 | 1.062877 | 1.751204474 | 1.744768324 | 0.794821804 | 0.86962782 | 0.873716084 | 0.983846754 | 1.53761907 |
| ENSMUSG00000001482 | Def8 | 4028 | 5.487262049 | 6.260640746 | 6.076714588 | 7.46531511 | 6.380945193 | 7.516564105 | 6.237098439 | 7.888798113 | 4.693299587 | 6.871266835 | 9.986721708 | 9.58898672 | 7.360596396 | 4.566002633 | 6.669169424 | 6.274031997 | 6.850777997 | 6.350356163 |
| ENSMUSG00000001493 | Meox1 | 2225 | 0 | 0 | 0 | 0 | 0 | 0 | 0 | 0 | 0 | 0 | 0 | 0 | 0 | 0 | 0 | 0 | 0 | 0 |
| ENSMUSG00000001494 | Sost | 2064 | 0 | 0 | 0 | 0 | 0 | 0 | 0 | 0 | 0 | 0 | 0 | 0 | 0 | 0 | 0 | 0 | 0 | 0 |
| ENSMUSG00000001496 | Nkx2-1 | 2829 | 0 | 0 | 0 | 0 | 0 | 0 | 0 | 0 | 0 | 0 | 0 | 0 | 0 | 0 | 0 | 0 | 0 | 0 |
| ENSMUSG00000001497 | Pax9 | 5052 | 0 | 0 | 0.076600998 | 0 | 0 | 0 | 0 | 0.022679574 | 0 | 0 | 0 | 0.011048231 | 0.097648292 | 0 | 0.016310988 | 0 | 0 | 0 |
| ENSMUSG00000001504 | Irx2 | 3777 | 0.078775809 | 0.03527974 | 0 | 0.04825104 | 0.025486854 | 0 | 0.025053047 | 0.050559177 | 0.032713674 | 0.112352748 | 0 | 0 | 0.143672515 | 0.036889664 | 0.01090854 | 0 | 0 | 0.160206559 |
| ENSMUSG00000001506 | Col1a1 | 6702 | 0 | 0 | 0 | 0 | 0 | 0 | 0 | 0.005698657 | 0 | 0.035176592 | 0 | 0 | 0.022082326 | 0 | 0.012295302 | 0 | 0 | 0.065662915 |
| ENSMUSG00000001507 | Itga3 | 6963 | 20.81612096 | 21.73973737 | 22.5506936 | 23.98776225 | 19.66613857 | 23.43489181 | 12.32589395 | 14.21724932 | 12.65821395 | 13.40778292 | 10.97115591 | 12.85772265 | 9.918811459 | 9.791744979 | 9.78115351 | 13.23646302 | 10.28749805 | 12.50601981 |
| ENSMUSG00000001508 | Sgca | 2007 | 0 | 0 | 0 | 0.045402137 | 0.047964049 | 0 | 0 | 0 | 0 | 0 | 0 | 0 | 0 | 0 | 0 | 0 | 0 | 0 |
| ENSMUSG00000001510 | Dlx3 | 2634 | 0 | 0 | 0 | 0 | 0 | 0 | 0 | 0 | 0 | 0 | 0 | 0 | 0 | 0 | 0 | 0 | 0 | 0 |
| ENSMUSG00000001517 | Foxm1 | 5205 | 0.171490624 | 0.115203092 | 0.083642992 | 0.087533226 | 0.240428437 | 0.114242613 | 0.118168074 | 0 | 0.522249768 | 0.153998454 | 0.477290651 | 0.053617351 | 0.246422642 | 0 | 0.221641415 | 0.205982134 | 0.559615478 | 0.232507271 |
| ENSMUSG00000001518 | Itfg2 | 3622 | 17.52076969 | 13.9156292 | 15.65261667 | 16.42814023 | 15.69403696 | 14.39245603 | 16.889921 | 15.5743176 | 14.75982812 | 16.85813383 | 17.2264207 | 16.93578498 | 14.41004099 | 13.59214043 | 18.48496088 | 16.95696694 | 14.99402615 | 15.9620572 |
| ENSMUSG00000001520 | Nrip2 | 3639 | 0.303691832 | 0.26547786 | 0.252568584 | 0.388126511 | 0.621654414 | 0.19063981 | 0.260031211 | 0.230896634 | 0.282952156 | 0.440539684 | 0.078771572 | 0.030676374 | 0.433806362 | 0.40841187 | 0.350988797 | 0.747082475 | 0.337027068 | 0 |
| ENSMUSG00000001521 | Tulp3 | 6894 | 0.579559986 | 0.642676955 | 0.736759601 | 0.700532448 | 0.537591837 | 0.488770798 | 0.404909785 | 0.642633983 | 0.645219568 | 0.861762201 | 0.547464711 | 0.688182669 | 1.624361071 | 0.390739349 | 0.705219553 | 1.049742501 | 0.806108653 | 0.726113825 |
| ENSMUSG00000001524 | Gtf2h4 | 2952 | 5.71631263 | 5.980973556 | 6.112238083 | 5.185809924 | 4.549053761 | 5.472286974 | 6.13846749 | 6.701783349 | 7.464358378 | 5.798003144 | 7.331319848 | 5.596692454 | 4.929849457 | 5.223386522 | 6.21093915 | 6.187201806 | 6.335783499 | 6.074853927 |
| ENSMUSG00000001525 | Tubb5 | 3753 | 43.35460001 | 40.09441598 | 35.0846655 | 35.26640922 | 34.24253562 | 36.60010058 | 24.8854858 | 25.34966011 | 29.05992372 | 25.65460593 | 25.31958231 | 26.68086829 | 27.53806733 | 21.78033408 | 22.41768642 | 30.25090619 | 25.57128895 | 21.94208174 |
| ENSMUSG00000001542 | Ell2 | 3635 | 13.28359834 | 13.92084906 | 12.13663265 | 12.972677 | 11.58608892 | 13.86385971 | 10.23047198 | 10.73800145 | 10.76401375 | 10.63647226 | 8.595549614 | 8.752387256 | 6.50068456 | 12.17640035 | 9.917843578 | 10.35479149 | 11.69294725 | 11.1834195 |
| ENSMUSG00000001552 | Jup | 6380 | 35.44984979 | 41.38004494 | 38.59267547 | 43.00448417 | 38.27921327 | 43.89067358 | 31.16110923 | 32.95441618 | 30.27015242 | 36.51592865 | 32.97820018 | 33.30567829 | 26.97017672 | 26.6580326 | 29.97768377 | 26.61737364 | 34.22934792 | 29.87563555 |
| ENSMUSG00000001555 | Fkbp10 | 3185 | 0.974215965 | 1.077308667 | 0.410073884 | 0.729548905 | 0.831163517 | 0.933489487 | 0.653613147 | 0.791428119 | 1.435385636 | 1.051083181 | 1.439998748 | 0.946325206 | 1.130684446 | 1.312391794 | 1.526462667 | 0.420775906 | 1.359771287 | 1.070820916 |
| ENSMUSG00000001558 | Klhl10 | 2453 | 0.121294836 | 0.353092234 | 0.039440302 | 0 | 0.019621657 | 0 | 0.964383998 | 0.264684403 | 0.369385792 | 0.11532989 | 0.525855638 | 0.364064656 | 0.120665105 | 0.075734346 | 0.386317075 | 0.093658233 | 0.249988076 | 0 |
| ENSMUSG00000001566 | Mnx1 | 3262 | 0 | 0 | 0 | 0 | 0 | 0 | 0 | 0 | 0 | 0 | 0 | 0 | 0 | 0 | 0 | 0 | 0 | 0 |
| ENSMUSG00000001569 | Nom1 | 4884 | 7.841352089 | 8.164523829 | 8.50795712 | 8.98347373 | 8.968069316 | 8.755939615 | 4.475523682 | 8.359475465 | 6.999346454 | 7.221278877 | 7.199502342 | 6.605539102 | 4.706935596 | 5.781737333 | 11.88636205 | 7.698889531 | 7.894401041 | 7.794083098 |
| ENSMUSG00000001576 | Ergic1 | 6555 | 46.73299827 | 44.25964848 | 46.53600037 | 40.82770014 | 41.38390874 | 42.48457097 | 36.32718734 | 38.30310502 | 40.72155738 | 39.92884116 | 36.28856114 | 40.15662315 | 43.00268121 | 32.67736553 | 36.66969902 | 41.73705414 | 35.841373 | 33.94536029 |
| ENSMUSG00000001583 | Tnk1 | 4467 | 12.46514005 | 19.09133853 | 14.19693268 | 11.85178722 | 13.87819962 | 16.73943921 | 12.20152365 | 13.84228778 | 15.53599263 | 11.89586805 | 12.01058392 | 12.54509184 | 14.18002726 | 12.9132654 | 11.75079073 | 11.03201516 | 14.75738318 | 12.46232729 |
| ENSMUSG00000001588 | Acap1 | 4708 | 0.162509169 | 0.714656397 | 0.051373758 | 0.280643646 | 0.112457766 | 0.305231367 | 1.467215617 | 0.811223501 | 0.874819791 | 0.160240283 | 0.446494939 | 0.130410427 | 1.257398061 | 2.308393468 | 1.163935203 | 0.073197848 | 0.276782943 | 0.514103801 |
| ENSMUSG00000001604 | Tcea3 | 1388 | 22.50814437 | 21.55257854 | 20.42283054 | 19.0713017 | 20.70227553 | 23.77674392 | 11.48730029 | 12.79500513 | 15.01468082 | 13.58809892 | 13.28611958 | 13.79306359 | 22.42682983 | 15.7936666 | 18.90878322 | 28.05587012 | 17.81013077 | 19.06295413 |

| ENSMUSG00000001622 | Csn3 | 4218 | 0 | 0 | 0 | 0.02160315 | 0 | 0 | 0 | 0 | 0 | 0 | 0 | 0 | 0.011695571 | 0 | 0 | 0 | 0 | 0 |
| --- | --- | --- | --- | --- | --- | --- | --- | --- | --- | --- | --- | --- | --- | --- | --- | --- | --- | --- | --- | --- |
| ENSMUSG00000001627 | Ifrd1 | 5303 | 17.94627364 | 18.23006204 | 16.89379186 | 19.11622169 | 20.70317139 | 17.67003945 | 12.01775948 | 23.01050833 | 13.44406162 | 14.59066642 | 16.1802424 | 13.17767482 | 12.81904243 | 15.95721803 | 11.59983461 | 23.23574856 | 23.63322475 | 20.74641023 |
| ENSMUSG00000001630 | Stk38l | 4568 | 7.313718993 | 8.554296173 | 8.852949075 | 6.762344146 | 9.830797894 | 9.687082111 | 8.14092943 | 7.098369011 | 8.132713927 | 7.462775494 | 9.193123578 | 8.027778091 | 5.712912487 | 4.504100419 | 8.685879668 | 5.783826424 | 6.921890312 | 5.503319127 |
| ENSMUSG00000001632 | Brpf1 | 6772 | 5.724264698 | 8.5348304 | 6.214555731 | 6.768223656 | 6.268806361 | 7.397787012 | 7.643247305 | 6.8184605 | 5.084454692 | 5.799842855 | 5.460398385 | 7.590996049 | 7.051579433 | 7.242314868 | 6.722935508 | 6.474120714 | 7.300786031 | 8.837848215 |
| ENSMUSG00000001642 | Akr1b3 | 3257 | 18.72733423 | 19.68907626 | 17.31763475 | 21.23477596 | 18.9749432 | 15.85322333 | 17.08311649 | 14.84543489 | 15.6298844 | 14.91102989 | 15.06444039 | 14.65225407 | 17.72132122 | 16.14206537 | 13.86457024 | 14.31929991 | 15.9565424 | 13.44404328 |
| ENSMUSG00000001655 | Hoxc13 | 2459 | 0 | 0 | 0 | 0 | 0 | 0 | 0 | 0 | 0 | 0 | 0 | 0 | 0 | 0 | 0 | 0 | 0 | 0 |
| ENSMUSG00000001656 | Hoxc11 | 2019 | 0 | 0 | 0 | 0 | 0 | 0 | 0 | 0.018916495 | 0 | 0 | 0 | 0 | 0 | 0 | 0 | 0 | 0 | 0 |
| ENSMUSG00000001657 | Hoxc8 | 2297 | 0 | 0 | 0 | 0 | 0 | 0.021572805 | 0 | 0 | 0.071722274 | 0 | 0 | 0 | 0 | 0 | 0 | 0 | 0 | 0 |
| ENSMUSG00000001661 | Hoxc6 | 2498 | 0 | 0 | 0 | 0 | 0 | 0 | 0 | 0 | 0 | 0 | 0 | 0 | 0 | 0 | 0 | 0 | 0 | 0 |
| ENSMUSG00000001663 | Gstt1 | 1771 | 24.48067733 | 23.36228945 | 22.56156752 | 20.45230392 | 24.8677076 | 23.22347198 | 19.31511398 | 16.71321957 | 19.3025455 | 22.25747483 | 26.62556975 | 21.02148331 | 10.91931766 | 12.06339932 | 13.47018682 | 10.37803024 | 14.10996363 | 10.84030392 |
| ENSMUSG00000001665 | Gstt3 | 2457 | 48.71574102 | 43.27828993 | 47.01505514 | 50.32668878 | 45.62444046 | 46.26535239 | 47.7170608 | 47.81433854 | 44.32118342 | 49.53030733 | 49.31106825 | 50.06826225 | 33.87177226 | 31.26516913 | 35.24854313 | 32.80493634 | 30.94805578 | 31.43377527 |
| ENSMUSG00000001666 | Ddt | 1043 | 10.96250465 | 10.79554961 | 10.85273835 | 9.522826141 | 13.79812577 | 8.646785725 | 17.32832536 | 13.14580294 | 14.09739801 | 13.83328399 | 15.9402546 | 15.41218679 | 17.73678713 | 16.74302671 | 16.31471005 | 16.26341235 | 13.19188935 | 15.0312516 |
| ENSMUSG00000001670 | Tat | 2407 | 0 | 0.179920077 | 0.080388085 | 0.094642801 | 0 | 0.185282344 | 0.039312571 | 0 | 0 | 0.137122943 | 0 | 0 | 0 | 0.019295425 | 0 | 0 | 0 | 0 |
| ENSMUSG00000001672 | Marveld3 | 3280 | 31.77521086 | 29.90035384 | 29.70252748 | 31.78160654 | 28.10141275 | 26.45330381 | 20.51177727 | 23.62572698 | 21.00763441 | 24.43786449 | 23.91661031 | 23.02396082 | 27.23783603 | 27.58325678 | 26.06500868 | 26.42985828 | 22.32974188 | 24.3851345 |
| ENSMUSG00000001674 | Ddx18 | 2469 | 9.19310004 | 8.189925796 | 10.07047169 | 8.599208854 | 9.57180013 | 8.449453559 | 8.412420433 | 9.157514064 | 9.475067215 | 9.013824195 | 8.339816567 | 10.05993109 | 9.990262713 | 11.11723599 | 10.24615444 | 9.739368752 | 9.577693475 | 13.61302722 |
| ENSMUSG00000001687 | Ubl3 | 3657 | 63.77519849 | 63.15512596 | 59.3524831 | 71.46244198 | 64.75500805 | 70.3927949 | 60.62543436 | 57.63846565 | 55.8050822 | 57.86501401 | 67.71058512 | 63.6301606 | 55.483231 | 53.88631742 | 51.51039473 | 62.23663396 | 65.2186343 | 60.01813941 |
| ENSMUSG00000001700 | Gramd3 | 3197 | 35.81762409 | 37.63721424 | 36.97995251 | 38.76322821 | 37.33724451 | 41.01236567 | 29.12460185 | 31.29937263 | 25.12158453 | 27.12234698 | 30.61960898 | 31.42577187 | 17.62184846 | 13.94630087 | 16.74095124 | 17.86974859 | 16.95132431 | 14.93522197 |
| ENSMUSG00000001707 | Eef1e1 | 1028 | 23.56804507 | 20.96638381 | 21.78691105 | 17.50643241 | 17.74513528 | 20.72731074 | 31.89463667 | 20.99096047 | 23.19746364 | 19.21803956 | 20.03016255 | 18.94909168 | 24.85791162 | 26.38457883 | 20.24006393 | 18.28860723 | 16.81435788 | 18.72878147 |
| ENSMUSG00000001729 | Akt1 | 4183 | 60.53151652 | 69.47685612 | 61.56841396 | 71.2660562 | 65.44929042 | 70.74561916 | 65.4324283 | 69.13527875 | 62.0013124 | 70.44989131 | 76.08816003 | 63.14121811 | 72.1404107 | 79.26472385 | 80.16721573 | 73.9357485 | 74.99420037 | 81.99421 |
| ENSMUSG00000001739 | Cldn15 | 2117 | 0.020078024 | 0.047207691 | 0 | 0 | 0 | 0 | 0.446978544 | 0.036081627 | 0.233461591 | 0 | 0 | 0 | 0.023302748 | 0 | 0 | 0 | 0.108624365 | 0 |
| ENSMUSG00000001741 | Il16 | 8831 | 0.206966659 | 0.173524305 | 0.306750957 | 0.237323977 | 0.207112796 | 0.280561282 | 0.498253781 | 0.320035984 | 0.223865107 | 0.186872904 | 0.254266 | 0.151690171 | 0.111724419 | 0.210368418 | 0.032658916 | 0.099726415 | 0.078119505 | 0.199330927 |
| ENSMUSG00000001750 | Tcirg1 | 4391 | 5.082035394 | 6.607955105 | 5.442159871 | 6.837788253 | 5.22863302 | 5.461972911 | 4.320743396 | 4.670762945 | 5.580955791 | 6.475033948 | 6.495479438 | 4.131197982 | 8.549667285 | 6.20876322 | 9.561463323 | 10.22013024 | 5.743271091 | 9.546103239 |
| ENSMUSG00000001751 | Naglu | 2651 | 27.94663215 | 27.5701584 | 31.002123 | 30.11050535 | 29.59450606 | 32.07562837 | 26.34232897 | 31.89663483 | 29.95382965 | 32.38831785 | 28.58207625 | 28.73948677 | 27.01997129 | 23.79142613 | 32.46701254 | 30.44760483 | 33.32407181 | 27.47342788 |
| ENSMUSG00000001755 | Coasy | 2930 | 32.00219055 | 25.76348747 | 24.6820573 | 31.02193974 | 27.49926287 | 28.07424496 | 21.18574565 | 23.18917539 | 22.63149842 | 26.39151991 | 34.51863041 | 25.46946788 | 24.6996323 | 25.94845429 | 23.93346352 | 25.47044158 | 25.75579474 | 26.47195921 |
| ENSMUSG00000001761 | Smo | 3767 | 4.378021847 | 5.712803203 | 4.956777998 | 7.982556215 | 7.628021882 | 6.932384018 | 3.353460386 | 5.920988321 | 5.685422935 | 4.280738124 | 3.91889094 | 5.704547411 | 3.077515413 | 4.154939594 | 3.062499509 | 3.049424534 | 3.550803722 | 5.782746552 |
| ENSMUSG00000001763 | Tspan33 | 5497 | 0.146916199 | 0.284829185 | 0.087999873 | 0.024865041 | 0.070048279 | 0.027043515 | 0.232389 | 0.069478629 | 0.172328518 | 0 | 0.15643974 | 0.152307611 | 0.098717681 | 0 | 0.059962243 | 0.034828641 | 0.034861103 | 0 |
| ENSMUSG00000001767 | Crnkl1 | 3830 | 8.279076062 | 8.863143397 | 9.194759817 | 7.875042128 | 8.533048608 | 8.552051409 | 5.670109563 | 9.194097916 | 9.000812975 | 7.866657802 | 8.44481642 | 8.029877307 | 5.512809555 | 8.524854683 | 7.734704618 | 6.848320123 | 9.236337348 | 7.368060509 |
| ENSMUSG00000001768 | Rin2 | 5384 | 12.37102727 | 18.53122552 | 13.77351821 | 18.3124257 | 16.04695449 | 16.41940503 | 9.473080959 | 10.84624504 | 10.22778075 | 11.39358561 | 12.85771078 | 8.272826661 | 7.238524266 | 9.385432221 | 8.632123894 | 10.59676914 | 10.38597087 | 7.989807106 |
| ENSMUSG00000001773 | Folh1 | 2652 | 1.586731684 | 2.047511975 | 1.71459723 | 2.628521789 | 2.069019343 | 1.382693169 | 6.600939368 | 5.602128407 | 4.612517037 | 5.102659063 | 1.116910794 | 6.58759517 | 6.640835018 | 14.6757712 | 3.153814422 | 1.371646947 | 2.76030268 | 4.604834726 |
| ENSMUSG00000001774 | Chordc1 | 2194 | 38.57238171 | 26.90540044 | 39.62043484 | 37.37916126 | 32.29270357 | 33.49439558 | 38.88092986 | 27.08631638 | 38.20171357 | 37.32943097 | 29.33129857 | 28.44207419 | 32.96289459 | 31.24497401 | 29.05141612 | 33.92750264 | 36.9637394 | 31.71673656 |
| ENSMUSG00000001783 | D10Wsu52e | 2603 | 104.2462711 | 104.2515698 | 99.33020351 | 104.4245834 | 104.2149527 | 105.5398982 | 86.39153398 | 94.740047 | 94.31917805 | 86.71164675 | 87.2723502 | 95.4848811 | 93.75528036 | 91.90683601 | 96.99697829 | 94.58647929 | 92.02433947 | 89.22337633 |
| ENSMUSG00000001785 | Pwp1 | 2845 | 35.25912669 | 33.9451952 | 35.31522761 | 33.87051275 | 34.91890701 | 32.72744689 | 30.23355019 | 31.64129788 | 29.61954701 | 33.09664478 | 33.85388973 | 30.27190417 | 30.032647 | 32.4047499 | 30.31102161 | 34.49519394 | 31.40196769 | 30.72389124 |
| ENSMUSG00000001786 | Fbxo7 | 6326 | 8.929715293 | 8.12548147 | 8.801443794 | 9.355642838 | 8.650963833 | 9.227492911 | 7.651101888 | 9.635642466 | 7.995106126 | 8.504418656 | 10.2482799 | 8.487933514 | 11.08135543 | 10.0288687 | 7.737503699 | 10.5804587 | 10.16016439 | 9.269634487 |
| ENSMUSG00000001794 | Capns1 | 2928 | 68.6063736 | 73.19052203 | 70.82558901 | 67.05004783 | 69.79786472 | 64.0733092 | 50.5928954 | 51.32756266 | 53.41024602 | 52.88350734 | 52.71888349 | 52.21280732 | 51.18523384 | 40.68616262 | 43.84701098 | 43.44310034 | 43.58830898 | 46.78028543 |
| ENSMUSG00000001802 | Lrp3 | 4942 | 0.086008045 | 0.080889262 | 0.039152999 | 0 | 0.019478723 | 0.06016115 | 0.019147179 | 0.100465648 | 0 | 0.057244885 | 0.23201113 | 0.180706314 | 0 | 0.009397832 | 0.083370206 | 0 | 0.186125278 | 0.178095044 |
| ENSMUSG00000001804 | Dsg4 | 3717 | 0 | 0 | 0 | 0 | 0 | 0 | 0 | 0 | 0 | 0 | 0 | 0 | 0 | 0 | 0 | 0 | 0 | 0 |
| ENSMUSG00000001815 | Evx2 | 7406 | 0 | 0 | 0 | 0 | 0 | 0 | 0 | 0 | 0 | 0 | 0 | 0 | 0 | 0 | 0 | 0 | 0 | 0 |
| ENSMUSG00000001819 | Hoxd13 | 2481 | 0 | 0 | 0 | 0 | 0 | 0 | 0 | 0 | 0 | 0 | 0 | 0 | 0 | 0 | 0 | 0 | 0 | 0 |
| ENSMUSG00000001823 | Hoxd12 | 2617 | 0 | 0 | 0 | 0 | 0 | 0 | 0 | 0 | 0 | 0 | 0 | 0 | 0 | 0 | 0 | 0 | 0 | 0 |
| ENSMUSG00000001827 | Folr1 | 2131 | 0.398922347 | 0.84415593 | 0.953396656 | 0.470362729 | 0.293625062 | 0.953384363 | 1.198913497 | 1.613006202 | 1.372239615 | 0.708034962 | 0.851923239 | 0.65480599 | 0.162047593 | 0.370506563 | 0.290015644 | 0.682798255 | 0.55753881 | 0.077441258 |
| ENSMUSG00000001829 | Clpb | 10260 | 8.869744822 | 9.09773193 | 8.283849204 | 9.796068597 | 9.856254544 | 9.75599628 | 8.079124121 | 9.17957742 | 8.450060008 | 10.01837555 | 9.79246956 | 7.692334006 | 8.188328959 | 8.745611777 | 7.678106712 | 7.785010433 | 8.371270113 | 7.924313814 |

| ENSMUSG00000001833 | 7-Sep | 3625 | 84.18956245 | 80.76883511 | 79.42600074 | 87.1378539 | 85.33624094 | 83.75437204 | 72.17630744 | 73.33994845 | 77.29430806 | 72.74865747 | 71.87989615 | 75.18562218 | 63.1040277 | 60.62715077 | 69.86647284 | 77.37343427 | 86.55928076 | 64.14441217 |
| --- | --- | --- | --- | --- | --- | --- | --- | --- | --- | --- | --- | --- | --- | --- | --- | --- | --- | --- | --- | --- |
| ENSMUSG00000001844 | Zdhhc4 | 3175 | 28.22075939 | 28.4130134 | 27.28724185 | 27.39402634 | 29.66745657 | 29.13856806 | 35.12314464 | 33.05597537 | 30.78286674 | 32.25553621 | 35.06004827 | 32.13575786 | 34.50903568 | 33.07404144 | 30.40480172 | 34.12989573 | 30.12990144 | 31.84464194 |
| ENSMUSG00000001847 | Rac1 | 4266 | 88.43786742 | 95.80775883 | 94.30910735 | 97.51943175 | 97.32441949 | 98.89638389 | 94.95807712 | 92.84889952 | 88.62921113 | 101.0766951 | 92.82855856 | 92.96068511 | 88.85758377 | 80.20478007 | 92.06123553 | 104.5945729 | 95.47434533 | 98.94160241 |
| ENSMUSG00000001855 | Nup214 | 9661 | 3.141361731 | 3.841275661 | 3.079362506 | 4.234946467 | 3.592083321 | 3.810959637 | 3.036317248 | 4.174637919 | 3.419064863 | 4.011787401 | 3.6445652 | 3.772655798 | 3.232284819 | 2.600791976 | 4.657085088 | 3.963420702 | 3.673548383 | 3.342339887 |
| ENSMUSG00000001864 | Aif1l | 4009 | 29.75044249 | 39.42870618 | 30.61203455 | 30.94605231 | 34.33706702 | 33.29884367 | 3.351658971 | 5.001499442 | 5.280585957 | 5.210217397 | 5.005109145 | 7.420740374 | 2.313394973 | 1.007891146 | 1.747135071 | 3.237843829 | 3.871825953 | 0.754677194 |
| ENSMUSG00000001865 | Cpa3 | 1441 | 0 | 0 | 0 | 0 | 0 | 0 | 0 | 0 | 0 | 0 | 0 | 0 | 0 | 0 | 0 | 0 | 0 | 0 |
| ENSMUSG00000001870 | Ltbp1 | 11195 | 0.2050272 | 0.324350645 | 0.133950821 | 0.032558138 | 0.146180027 | 0.309842908 | 0.815662977 | 0.368448366 | 0.3347899 | 0.223223517 | 0.046942791 | 0.453704805 | 0.308462189 | 0.406567268 | 0.088328481 | 0.08892861 | 0.095858536 | 0.275168377 |
| ENSMUSG00000001891 | Ugp2 | 3514 | 41.36815652 | 36.34651008 | 35.51613782 | 36.67962277 | 37.5850878 | 38.04589505 | 34.84496672 | 36.58384362 | 43.98776143 | 36.81887585 | 37.53746738 | 33.34008986 | 40.23485342 | 39.30697663 | 41.29535568 | 41.28716888 | 43.29977197 | 39.0730593 |
| ENSMUSG00000001901 | Kcnh6 | 3759 | 0.011307575 | 0 | 0 | 0 | 0 | 0 | 0 | 0 | 0 | 0 | 0 | 0.01484854 | 0 | 0 | 0.010960776 | 0 | 0 | 0 |
| ENSMUSG00000001909 | Trmt1 | 6253 | 7.518107258 | 6.808552505 | 6.049592314 | 6.601360334 | 6.119443358 | 6.411028541 | 6.870288256 | 6.596480825 | 7.291455773 | 6.499905039 | 7.021459594 | 7.265944242 | 7.605304375 | 6.892709595 | 6.220097355 | 8.750564205 | 6.497021384 | 7.002598585 |
| ENSMUSG00000001910 | Nacc1 | 4294 | 6.651951162 | 7.036514918 | 7.559068204 | 6.981641167 | 6.994485414 | 8.193395631 | 6.842378578 | 7.124386196 | 7.136182517 | 6.445613137 | 7.587918417 | 5.680355042 | 6.364667483 | 5.343125098 | 8.443728268 | 5.332506573 | 6.756638741 | 7.238040358 |
| ENSMUSG00000001911 | Nfix | 6243 | 32.69419434 | 30.85298004 | 30.47462676 | 30.75352248 | 29.10427869 | 29.82847554 | 17.46848069 | 18.53031986 | 20.87364022 | 22.66526677 | 18.96305416 | 21.68075953 | 20.4502646 | 16.70886117 | 18.92776907 | 22.96945775 | 20.97724756 | 21.84327656 |
| ENSMUSG00000001918 | Slc1a5 | 5242 | 3.640752392 | 3.304598435 | 4.604805732 | 3.337550749 | 3.608516979 | 3.667771963 | 4.693359985 | 8.947018343 | 6.144192164 | 4.335490074 | 4.702762033 | 6.633566919 | 3.547907824 | 2.622558159 | 4.299361136 | 4.331615832 | 4.101688776 | 3.442004389 |
| ENSMUSG00000001924 | Uba1 | 4895 | 101.0659334 | 121.355573 | 107.1332876 | 109.5978135 | 109.0859165 | 116.0617141 | 99.09082418 | 108.0388552 | 100.1433934 | 110.7054203 | 120.467071 | 102.0189445 | 94.73340601 | 85.93342175 | 89.40611372 | 81.91608595 | 91.58379875 | 89.52043803 |
| ENSMUSG00000001930 | Vwf | 16008 | 3.170363579 | 3.475295686 | 4.019040191 | 3.796753695 | 3.55998236 | 3.773412183 | 4.601814161 | 4.032056476 | 4.114020409 | 4.335696851 | 3.276918067 | 3.65409885 | 3.035478418 | 2.675003034 | 3.538989215 | 2.049915701 | 2.784450481 | 2.463863663 |
| ENSMUSG00000001942 | Siae | 3423 | 4.482725254 | 6.082547148 | 6.048457783 | 6.162653674 | 4.96364857 | 6.442000129 | 9.744504424 | 5.868888012 | 5.943949397 | 8.209704686 | 6.071313742 | 7.549708378 | 5.908877033 | 5.929321119 | 4.200801346 | 5.100939812 | 4.377908878 | 4.467581557 |
| ENSMUSG00000001943 | Vsig2 | 1310 | 0.032446699 | 0 | 0 | 0 | 0 | 0 | 0.036116549 | 0.116618023 | 0 | 0.143971613 | 0 | 0 | 0 | 0 | 0 | 0 | 0 | 0 |
| ENSMUSG00000001946 | Esam | 2857 | 0.565347109 | 0.757906239 | 0.643400124 | 1.355508844 | 0.606492563 | 0.537674043 | 0.480247704 | 0.574824397 | 0.850544078 | 0.907696423 | 0.902991865 | 0.664239597 | 0.86335172 | 1.430549419 | 0.591271891 | 0.160828593 | 0.389031365 | 0.635386952 |
| ENSMUSG00000001948 | Spa17 | 572 | 11.36939149 | 7.978787601 | 11.50139883 | 7.64660884 | 7.909791652 | 8.663065311 | 23.40819604 | 16.69248357 | 20.52125348 | 14.1781835 | 16.28691766 | 20.39418441 | 21.1299296 | 20.86736094 | 16.71112057 | 10.9784259 | 8.844530055 | 11.25186274 |
| ENSMUSG00000001962 | Fam50a | 2532 | 34.79985383 | 33.26026721 | 32.36364946 | 32.44335029 | 32.33507194 | 32.80030864 | 36.04508596 | 31.90242144 | 30.74348925 | 31.35931449 | 31.86884079 | 32.86775392 | 34.13488115 | 32.46683834 | 33.42337907 | 31.34930061 | 27.9273372 | 30.61130781 |
| ENSMUSG00000001964 | Emd | 2791 | 19.20423754 | 12.24615889 | 14.94015878 | 9.125268285 | 15.19320127 | 12.76546594 | 11.86631145 | 13.06834264 | 17.61974196 | 12.67037888 | 12.59848421 | 13.09898208 | 15.13010433 | 11.13260282 | 12.53318558 | 21.36097303 | 11.9057325 | 9.854730692 |
| ENSMUSG00000001983 | Taco1 | 1378 | 6.199956736 | 4.061368816 | 3.124270103 | 4.364338064 | 4.226388072 | 4.063467993 | 3.77677408 | 4.018794157 | 5.290285406 | 5.132514868 | 4.160373743 | 4.415027012 | 4.510755861 | 4.280405732 | 4.156035028 | 2.945431339 | 2.169413032 | 2.754447289 |
| ENSMUSG00000001985 | Grik3 | 8962 | 0.01897129 | 0.026019891 | 0.059373894 | 0 | 0.010741335 | 0.005529205 | 0.021117018 | 0.021307968 | 0.027574101 | 0.010522362 | 0.01599251 | 0.037368219 | 0.022018263 | 0.02072934 | 0.036778894 | 0.02563531 | 0.017106136 | 0.012276078 |
| ENSMUSG00000001986 | Gria3 | 6707 | 3.586988166 | 5.488407352 | 3.923542644 | 5.033656456 | 4.53546679 | 3.457682916 | 1.3050314 | 1.264158839 | 0.730758219 | 1.188082429 | 1.346275108 | 1.955670301 | 1.684360975 | 0.574751641 | 0.860029495 | 0.490978416 | 1.428593145 | 1.410700217 |
| ENSMUSG00000001988 | Npas1 | 2079 | 0.306675152 | 0.352517399 | 0.093070765 | 0.306808379 | 0.231514784 | 0.190679109 | 0.204816792 | 0.09185282 | 0.435836146 | 0.498949241 | 0.068939334 | 0.187931524 | 0.11864338 | 0.089358513 | 0.158543746 | 0.036835601 | 0.055304902 | 0.052918814 |
| ENSMUSG00000001995 | Sipa1l2 | 6344 | 2.318220508 | 3.082387911 | 2.851781266 | 3.598058514 | 3.983174641 | 4.335083092 | 3.497737455 | 2.39605551 | 1.610065559 | 3.552653871 | 3.102661238 | 3.070565297 | 2.597235241 | 2.174321241 | 2.253615998 | 2.927324179 | 2.646109402 | 2.835431573 |
| ENSMUSG00000001998 | Ap4e1 | 7188 | 1.555211644 | 1.408892577 | 2.03238817 | 2.313547744 | 1.52002597 | 1.516639036 | 1.45466083 | 1.833107795 | 1.260577834 | 1.63991038 | 1.103317071 | 1.467607155 | 1.489291326 | 1.337496674 | 1.272502144 | 1.060076638 | 1.898186101 | 1.9667975 |
| ENSMUSG00000001999 | Blvra | 2047 | 20.90997179 | 19.61017955 | 18.59793277 | 16.82664852 | 18.88125779 | 18.54293792 | 14.35328462 | 15.46726995 | 13.0179168 | 15.20247394 | 16.52402081 | 14.3970053 | 17.95421513 | 15.74606578 | 12.55973174 | 18.59348498 | 15.54021809 | 14.9682816 |
| ENSMUSG00000002006 | Pdzd4 | 3962 | 0.05364106 | 0.269059216 | 0.134303088 | 0.022999013 | 0.072890344 | 0.062534999 | 0.011941615 | 0.086757098 | 0.103953851 | 0.059503664 | 0.108524641 | 0.18314074 | 0.012451266 | 0 | 0.103991812 | 0.115973571 | 0.058040833 | 0.194378469 |
| ENSMUSG00000002007 | Srpk3 | 2192 | 0.426603044 | 1.048626688 | 0.331023246 | 0.644339587 | 0.219579944 | 1.401582793 | 0.97129131 | 1.550695171 | 1.146157601 | 0.817393578 | 2.615417435 | 1.731507781 | 2.160521925 | 0.296631945 | 0.244352293 | 1.292657367 | 0.576992608 | 0.527003303 |
| ENSMUSG00000002010 | Idh3g | 2398 | 89.92024937 | 80.21211477 | 83.37272767 | 80.88130348 | 88.1950261 | 82.42946382 | 97.52567637 | 96.30919823 | 94.6362393 | 88.2650154 | 103.3198226 | 97.17697719 | 93.12993724 | 86.92287915 | 81.95638932 | 75.11218399 | 85.58687236 | 87.078636 |
| ENSMUSG00000002012 | Pnck | 2254 | 0.226291975 | 0.251250755 | 0.107305968 | 0.363841525 | 0.405726065 | 0.351749662 | 0.167924326 | 0.355829836 | 0.310634768 | 0.230105473 | 0.233151971 | 0.569547577 | 0.240750262 | 0.061815556 | 0.383865428 | 0.254817707 | 0.187040491 | 0.122025525 |
| ENSMUSG00000002014 | Ssr4 | 1639 | 100.7261157 | 91.97123023 | 96.09775158 | 88.87044463 | 98.61317857 | 84.04917593 | 114.8898488 | 109.4740125 | 113.2817651 | 102.2413663 | 107.4717949 | 102.5727735 | 168.7037195 | 169.2276326 | 140.9500451 | 135.8744189 | 125.2210616 | 155.8982921 |
| ENSMUSG00000002015 | Bcap31 | 1211 | 319.0871637 | 279.7897994 | 285.3279334 | 294.0587304 | 284.6579994 | 288.682523 | 265.5917352 | 266.9998999 | 282.6598947 | 277.88156 | 299.826329 | 272.3951824 | 284.3815974 | 252.2401014 | 266.7724193 | 297.5980156 | 274.930257 | 268.0955804 |
| ENSMUSG00000002017 | Fam98a | 3471 | 10.76405927 | 9.866221624 | 11.60908983 | 10.17280592 | 8.44492697 | 9.679272073 | 9.296239421 | 8.384503207 | 8.32985712 | 8.028252845 | 9.469654691 | 6.882484468 | 7.60373833 | 9.205857714 | 7.323929699 | 5.934988989 | 8.435981226 | 7.084145982 |
| ENSMUSG00000002020 | Ltbp2 | 8635 | 94.27436379 | 111.4429155 | 88.37202563 | 104.3181711 | 92.42332673 | 109.0447068 | 50.10705834 | 60.20997961 | 48.29816544 | 54.02536862 | 44.13443472 | 55.96433198 | 21.72661047 | 15.8184205 | 21.64811338 | 17.71966036 | 19.20087555 | 21.66600718 |
| ENSMUSG00000002028 | Mll1 | 16633 | 4.587073346 | 4.115793761 | 4.55728504 | 4.012921899 | 4.305916096 | 3.861019823 | 3.632434978 | 4.286972602 | 4.580956782 | 3.980014872 | 4.127494459 | 3.976526193 | 5.335665194 | 5.210405042 | 3.448119149 | 6.18800894 | 3.979409292 | 3.925678451 |
| ENSMUSG00000002031 | Ift46 | 5301 | 11.26575596 | 10.49472427 | 8.751219696 | 8.826861447 | 11.24984973 | 9.600199469 | 7.800656741 | 7.874796421 | 9.051554586 | 6.742168095 | 8.823181353 | 8.118067507 | 6.05830563 | 6.518468405 | 6.05470893 | 6.551514981 | 7.511983113 | 5.717792455 |
| ENSMUSG00000002032 | Tmem25 | 4167 | 1.856477571 | 3.293715483 | 2.414613466 | 1.825940582 | 2.263944574 | 2.628066744 | 2.214056249 | 2.493000589 | 2.678556701 | 2.749609045 | 2.843338062 | 1.285889995 | 2.142807062 | 1.560396505 | 1.483137361 | 1.856180152 | 1.922293269 | 1.702945708 |
| ENSMUSG00000002033 | Cd3g | 1226 | 0 | 0 | 0 | 0 | 0 | 0 | 0 | 0 | 0 | 0 | 0 | 0.227633208 | 0 | 0 | 0 | 0 | 0 | 0 |
| ENSMUSG00000002043 | Trappc6a | 1430 | 93.77890235 | 77.87762614 | 76.9578892 | 80.4168363 | 79.40084471 | 76.51219283 | 113.5835152 | 97.91143162 | 101.7854173 | 108.9082328 | 108.3789967 | 100.3901288 | 149.6516484 | 150.4398691 | 107.9019768 | 97.06535093 | 100.7472378 | 115.7114637 |

| ENSMUSG00000002052 | Supt6h | 7798 | 8.230676558 | 6.566032113 | 6.786437831 | 6.952762601 | 6.289616596 | 6.748525655 | 5.812457863 | 7.366167378 | 5.772872753 | 6.929303146 | 5.556794991 | 7.20063562 | 7.629429627 | 5.670014259 | 6.509402008 | 5.956207589 | 7.308438509 | 6.151313293 |
| --- | --- | --- | --- | --- | --- | --- | --- | --- | --- | --- | --- | --- | --- | --- | --- | --- | --- | --- | --- | --- |
| ENSMUSG00000002055 | Spag5 | 5247 | 0.032403412 | 0 | 0 | 0 | 0 | 0 | 0 | 0.029115611 | 0.125592577 | 0 | 0.109262341 | 0.042550534 | 0.159832779 | 0.008851551 | 0 | 0.145952382 | 0.07304421 | 0.272581813 |
| ENSMUSG00000002057 | Foxn1 | 3205 | 0 | 0.031182116 | 0 | 0 | 0 | 0 | 0 | 0 | 0 | 0 | 0 | 0 | 0 | 0 | 0 | 0 | 0 | 0 |
| ENSMUSG00000002058 | Unc119 | 2732 | 11.06192539 | 10.76694202 | 7.613696213 | 9.155568573 | 8.615121037 | 9.050810416 | 7.100365432 | 6.961865446 | 7.432266571 | 8.853700868 | 8.568715101 | 7.334488605 | 6.446374256 | 6.545012315 | 7.751683649 | 8.689669322 | 6.467211886 | 5.698234701 |
| ENSMUSG00000002059 | Rab34 | 2266 | 17.4259967 | 18.37648622 | 16.33086969 | 18.21637518 | 17.26886758 | 20.27157283 | 23.65633946 | 20.42771038 | 22.75618611 | 22.34768548 | 21.96889088 | 21.40503565 | 19.31041952 | 18.87687753 | 19.92802527 | 15.17430073 | 16.67684411 | 21.48413921 |
| ENSMUSG00000002064 | Sdf2 | 1511 | 60.78999695 | 50.09059938 | 52.85552523 | 50.86795618 | 53.99312417 | 57.25947904 | 56.36189416 | 56.26491579 | 53.39800422 | 59.00859024 | 70.76132169 | 54.11644321 | 72.12058588 | 58.64680261 | 58.78924851 | 67.30632247 | 64.9847602 | 65.78521234 |
| ENSMUSG00000002068 | Ccne1 | 2651 | 0.304639134 | 0.251323231 | 0.127730936 | 0.223422699 | 0 | 0.224305094 | 0.124929744 | 0.288135816 | 0.217507062 | 0.035572013 | 0.504601598 | 0.273709398 | 0.260523139 | 0.42046703 | 0.062167568 | 0.808854777 | 0.274688661 | 0.332005171 |
| ENSMUSG00000002076 | Hsf2bp | 3447 | 0.283614461 | 0.086978836 | 0.098234613 | 0.277569461 | 0.069817122 | 0.273136623 | 0.219612087 | 0.08863917 | 0.131433616 | 0.05471506 | 0 | 0.340043201 | 0.071557756 | 0.21558033 | 0 | 0.011108386 | 0.04447496 | 0 |
| ENSMUSG00000002083 | Bbc3 | 2003 | 14.74842603 | 14.41951038 | 12.48582885 | 12.28305738 | 11.94286873 | 14.00241997 | 7.062651522 | 9.247785904 | 13.92075445 | 9.062916003 | 9.516828973 | 10.44976209 | 5.443012341 | 5.63450486 | 7.734291071 | 10.24651301 | 7.577240925 | 7.250326598 |
| ENSMUSG00000002100 | Mybpc3 | 4131 | 0 | 0.008064124 | 0 | 0 | 0 | 0 | 0 | 0 | 0 | 0 | 0 | 0 | 0 | 0 | 0 | 0 | 0 | 0 |
| ENSMUSG00000002102 | Psmc3 | 2869 | 94.41808533 | 91.68302989 | 96.91566834 | 86.21502604 | 93.36150408 | 91.17772066 | 79.37118288 | 78.95403927 | 83.89473618 | 78.93510901 | 78.33161544 | 79.43372267 | 74.40195401 | 75.92288946 | 73.47060298 | 64.63625362 | 67.92932023 | 73.9909874 |
| ENSMUSG00000002103 | Acp2 | 4354 | 16.58619524 | 17.67404356 | 18.97611159 | 17.99839142 | 18.06328968 | 17.8908813 | 17.47330908 | 15.93835443 | 13.89595582 | 13.69904447 | 17.09540315 | 15.33200102 | 12.75786377 | 13.8030889 | 15.02711777 | 15.10869626 | 15.1755939 | 14.85776516 |
| ENSMUSG00000002104 | Rapsn | 2155 | 0.118343878 | 0.139125776 | 0.067341342 | 0.338272255 | 0.089339998 | 0.298926003 | 0.724509701 | 0.248117695 | 0.401353518 | 0.350074827 | 0.731588691 | 0.207204316 | 0.206026569 | 0.086207123 | 0.344142926 | 0.071073053 | 0.231202719 | 0 |
| ENSMUSG00000002105 | Slc39a13 | 4475 | 5.794001648 | 5.136513299 | 4.58332444 | 5.304425497 | 4.097935847 | 5.126908524 | 4.79999022 | 6.13638823 | 4.583437955 | 5.858277321 | 6.843295728 | 5.787367026 | 4.982799242 | 5.230797775 | 4.226036682 | 4.509307285 | 4.667671913 | 5.593104706 |
| ENSMUSG00000002107 | Celf2 | 18155 | 3.240273403 | 3.734053414 | 3.319934939 | 3.377866465 | 3.640051291 | 3.589195519 | 3.043856182 | 2.688509517 | 2.910619649 | 2.28027086 | 2.284145633 | 3.019057045 | 2.994424282 | 2.895880304 | 2.564459276 | 2.324221942 | 2.239834812 | 2.469425612 |
| ENSMUSG00000002108 | Nr1h3 | 2896 | 0.132094815 | 0.034509214 | 0.267257074 | 0.613563788 | 0.216061812 | 0.359325761 | 0.865874304 | 0.303323638 | 0.31288099 | 0.521002246 | 0.676371995 | 0.539654196 | 0.18737952 | 0.240559845 | 0.512174037 | 0.423100634 | 0.198513278 | 0.531856004 |
| ENSMUSG00000002109 | Ddb2 | 2775 | 6.463850192 | 5.294049135 | 5.66536842 | 5.861366785 | 4.891244135 | 3.946361845 | 8.627104698 | 6.041969245 | 4.259650459 | 6.099856746 | 6.559372678 | 6.919130785 | 5.973161876 | 5.255294933 | 6.473469682 | 7.395951556 | 7.844805997 | 7.314724462 |
| ENSMUSG00000002111 | Sfpi1 | 2803 | 0.045492518 | 0.011884729 | 0 | 0.016254386 | 0 | 0 | 0.354465308 | 0.204383174 | 0.514280432 | 0 | 0.221574906 | 0 | 0.211196221 | 0.29824958 | 0.117592739 | 0 | 0 | 0 |
| ENSMUSG00000002129 | Sf3a1 | 6327 | 11.00418497 | 10.28819271 | 10.68086327 | 10.39831642 | 11.34261075 | 11.0822061 | 9.205295995 | 11.95210317 | 10.08343811 | 9.538944234 | 11.17542888 | 9.271734057 | 12.17898764 | 8.595863179 | 12.60727236 | 7.074714723 | 11.1398862 | 8.042267065 |
| ENSMUSG00000002147 | Stat6 | 4430 | 19.14172148 | 23.69501799 | 20.74711234 | 26.71954699 | 21.72998811 | 26.68912468 | 16.29777608 | 16.70809839 | 18.50139196 | 20.70160673 | 15.85309006 | 17.08488453 | 13.58576503 | 12.03562355 | 14.93672658 | 17.07086899 | 19.05933004 | 16.21713169 |
| ENSMUSG00000002190 | Clgn | 4109 | 5.265304112 | 4.880594383 | 3.625954575 | 4.978561428 | 6.442579221 | 5.004717555 | 5.031793792 | 5.400288585 | 2.375566862 | 5.232592038 | 3.511325739 | 5.569340876 | 5.522677524 | 4.803781246 | 3.359094968 | 3.503837523 | 4.048092688 | 2.382969335 |
| ENSMUSG00000002204 | Napsa | 2149 | 41.63489788 | 42.25730556 | 33.76467917 | 41.25723233 | 43.18210741 | 47.0162977 | 189.8671676 | 135.6016893 | 151.6177414 | 206.9233515 | 158.7533637 | 118.9296971 | 343.8312969 | 212.8777388 | 179.9332722 | 234.269384 | 205.2748615 | 182.3056576 |
| ENSMUSG00000002205 | Vrk3 | 3169 | 30.28611154 | 30.67435321 | 27.644198 | 31.60087581 | 30.6804941 | 29.99120953 | 24.9627009 | 28.26165467 | 25.16159499 | 30.91800611 | 34.20680997 | 32.16137579 | 28.02065355 | 23.41989639 | 24.97576171 | 34.48450411 | 26.66755591 | 29.07549819 |
| ENSMUSG00000002210 | 1500002O20R | 3855 | 8.115125696 | 9.557473672 | 6.336869735 | 6.724833781 | 7.179210404 | 7.686779424 | 7.903856091 | 8.975957608 | 8.867654496 | 8.255959714 | 8.030654499 | 9.194019648 | 7.537353898 | 8.553904552 | 7.27840715 | 6.356936119 | 6.571642608 | 7.16331299 |
| ENSMUSG00000002221 | Paxip1 | 5031 | 8.592280664 | 11.38902366 | 10.94197526 | 10.55936746 | 11.41351321 | 10.90337429 | 10.73963016 | 11.48580895 | 7.71991341 | 10.74972304 | 8.499023621 | 9.130648044 | 6.687411565 | 7.966854982 | 8.46798028 | 8.958068955 | 9.78154745 | 10.52947123 |
| ENSMUSG00000002222 | Rmnd5a | 9948 | 4.853827902 | 6.004223898 | 5.368353188 | 5.743219702 | 5.597973028 | 5.519142421 | 5.402814964 | 5.985319195 | 4.955796076 | 5.597605604 | 5.282715554 | 6.457964177 | 6.526015595 | 5.719140234 | 6.266380585 | 6.616561532 | 6.93094171 | 6.724072555 |
| ENSMUSG00000002227 | Mov10 | 4201 | 8.418068663 | 8.79409657 | 9.234842024 | 9.88005508 | 8.283594576 | 8.881982477 | 10.654081 | 9.291272378 | 7.676515554 | 9.023843232 | 6.834741739 | 10.16400426 | 8.267000658 | 6.01418318 | 6.747600679 | 10.98314257 | 9.889480086 | 7.241141637 |
| ENSMUSG00000002228 | Ppm1j | 1711 | 2.210964738 | 2.745247273 | 1.781141094 | 1.757468689 | 2.531778568 | 1.940405114 | 0.580693312 | 1.026797493 | 1.56465431 | 0.964508833 | 1.396112171 | 1.304866454 | 0.374818775 | 0.4885994 | 0.698331456 | 0 | 0.134399638 | 0.900207474 |
| ENSMUSG00000002233 | Rhoc | 910 | 125.5070417 | 102.0985296 | 100.3617861 | 92.97457069 | 109.4869033 | 99.92227046 | 67.64153328 | 61.77935862 | 59.19995892 | 61.14047236 | 67.51494132 | 84.03017334 | 60.71620588 | 61.04080057 | 62.61730967 | 75.52927504 | 55.29937125 | 52.16797704 |
| ENSMUSG00000002240 | 4930432K09R | 1525 | 0.139361233 | 0.131067125 | 0 | 0.059752189 | 0 | 0.032493596 | 3.691940192 | 3.280789977 | 2.619732478 | 1.205821264 | 1.065146615 | 2.781633021 | 3.817158189 | 5.634200765 | 2.539636888 | 1.054560991 | 0.578035953 | 2.128221178 |
| ENSMUSG00000002249 | Tead3 | 5375 | 0.988492466 | 1.530843697 | 1.115966096 | 1.279947478 | 0.993980191 | 1.14317004 | 1.038678347 | 1.136890118 | 1.271992859 | 1.570228071 | 1.066603724 | 1.277270046 | 1.321636481 | 1.512133078 | 1.563742772 | 0.854860072 | 0.563307434 | 1.176939028 |
| ENSMUSG00000002250 | Ppard | 4719 | 1.116898035 | 1.122430638 | 1.086584916 | 0.975135723 | 1.019960239 | 0.777050707 | 1.203119617 | 1.618665073 | 1.274259653 | 1.338884136 | 1.194627061 | 1.797834438 | 1.578537722 | 1.348344982 | 1.676350653 | 1.582256504 | 0.860900079 | 1.457117682 |
| ENSMUSG00000002257 | Def6 | 2612 | 4.312355148 | 4.030197004 | 4.629932078 | 5.006133126 | 3.96185436 | 5.255018071 | 6.756366478 | 5.673297141 | 5.518866967 | 8.339825768 | 6.36511698 | 5.940564395 | 6.988058726 | 7.556943775 | 8.896507474 | 5.438673563 | 7.116483154 | 10.36159284 |
| ENSMUSG00000002265 | Peg3 | 13121 | 4.107656673 | 3.259946359 | 2.425865633 | 3.461958784 | 2.531135381 | 2.964628905 | 1.972413335 | 1.740649085 | 1.393705739 | 2.281891363 | 1.07412642 | 1.36125387 | 9.083599736 | 9.390760144 | 5.79666734 | 13.78006616 | 10.24975046 | 8.737061078 |
| ENSMUSG00000002266 | Zim1 | 5888 | 0 | 0 | 0 | 0 | 0 | 0 | 0 | 0 | 0 | 0 | 0 | 0 | 0 | 0 | 0 | 0 | 0 | 0 |
| ENSMUSG00000002274 | Metrn | 1771 | 0 | 0.018810217 | 0.081942739 | 0.025726168 | 0 | 0.055960173 | 0.026715234 | 0.043130889 | 0 | 0.053247547 | 0.026976261 | 0 | 0 | 0 | 0 | 0.021620896 | 0 | 0 |
| ENSMUSG00000002279 | Lmf1 | 14147 | 1.715590267 | 1.940328327 | 1.873803254 | 1.542640859 | 1.673917187 | 1.951005344 | 1.953106983 | 2.202940578 | 2.334882706 | 1.993081257 | 2.076878452 | 2.098956139 | 2.102717616 | 1.687443339 | 1.453281713 | 1.588788191 | 1.668834304 | 1.248174402 |
| ENSMUSG00000002280 | Narfl | 3347 | 14.97269272 | 12.2124055 | 15.18989555 | 14.11616462 | 13.96357869 | 13.84278634 | 12.8636205 | 14.9140932 | 14.23746601 | 14.96087447 | 15.24459386 | 14.15820064 | 13.16205621 | 13.27965092 | 13.01166554 | 12.46990202 | 15.7450428 | 14.20013989 |
| ENSMUSG00000002289 | Angptl4 | 3850 | 0.077282138 | 0.173053996 | 0.502582134 | 0.355021125 | 0.037505395 | 0.218804278 | 0 | 0.30752324 | 0.192560333 | 0 | 0.111681721 | 0 | 0 | 0.096507195 | 0 | 0 | 0.119458588 | 0 |
| ENSMUSG00000002290 | Rab11b-ps2 | 1544 | 1.2112874 | 1.165088269 | 0.845910181 | 1.091812591 | 0.716991091 | 0.898624702 | 0.858001949 | 0.51945625 | 0.693555317 | 0.793988526 | 0.742615935 | 1.048351176 | 0.70291592 | 0.691848452 | 0.640438693 | 0.768788102 | 0.968086515 | 0.534414897 |
| ENSMUSG00000002297 | Dbf4 | 7708 | 0.463211571 | 0.825475194 | 0.489508999 | 0.419672308 | 0.499552918 | 0.752162666 | 0.601536395 | 0.386482536 | 0.951115699 | 0.452666326 | 0.458659435 | 0.572059853 | 0.275204001 | 0.421780762 | 0.379516148 | 0.362638083 | 0.64639577 | 0.727935766 |

| ENSMUSG00000002307 | Daxx | 3002 | 8.226351523 | 8.089640202 | 7.380105564 | 8.408000853 | 9.28327242 | 8.500885341 | 7.86443264 | 6.742829205 | 6.681490065 | 8.984078036 | 7.352441978 | 7.585872863 | 5.866587098 | 8.47813454 | 6.505508825 | 8.992297874 | 9.447521555 | 9.107103944 |
| --- | --- | --- | --- | --- | --- | --- | --- | --- | --- | --- | --- | --- | --- | --- | --- | --- | --- | --- | --- | --- |
| ENSMUSG00000002308 | Cd320 | 2774 | 27.35102351 | 23.88584953 | 26.52348222 | 21.97573083 | 25.40199576 | 22.73995308 | 27.86911225 | 26.55845142 | 26.33917771 | 25.78500968 | 28.62364131 | 26.03657801 | 28.91625721 | 27.4244467 | 25.16057523 | 23.2587143 | 23.16986298 | 21.9322538 |
| ENSMUSG00000002319 | Ipo4 | 5645 | 7.492054942 | 5.623948307 | 7.403849464 | 7.425360636 | 5.371676158 | 6.302721472 | 5.514923425 | 6.082368426 | 7.566061443 | 7.584205236 | 8.624035906 | 6.23909355 | 6.903846705 | 6.606661147 | 8.795017688 | 7.000160645 | 7.251104534 | 7.181879833 |
| ENSMUSG00000002320 | Tm9sf1 | 4075 | 24.59563315 | 26.92008851 | 29.11908465 | 33.18409315 | 26.94206574 | 30.04780483 | 23.44154571 | 25.40849151 | 23.45862652 | 26.50853035 | 26.17950485 | 23.95621935 | 24.79307155 | 21.44976011 | 23.55819024 | 23.10591499 | 25.20600631 | 24.15001029 |
| ENSMUSG00000002324 | Rec8 | 2812 | 4.791657468 | 3.4355403 | 2.976038674 | 5.865255352 | 5.032285049 | 6.097171344 | 2.742520151 | 3.368319985 | 3.837434968 | 3.437373459 | 4.994963653 | 4.30725419 | 2.666590123 | 4.872334915 | 2.27106727 | 2.274015447 | 3.16205579 | 3.540771094 |
| ENSMUSG00000002325 | Irf9 | 3689 | 18.55064608 | 15.95659639 | 16.9418816 | 15.75925524 | 15.48728474 | 17.65039087 | 14.99282235 | 14.60815392 | 13.19665536 | 12.0017648 | 13.35212312 | 14.05604515 | 11.68774619 | 19.86683922 | 12.10693591 | 10.88827519 | 10.65946886 | 17.6852265 |
| ENSMUSG00000002326 | Gmpr2 | 1533 | 17.80060209 | 16.62385132 | 19.24843674 | 16.49470294 | 14.56830566 | 16.84081813 | 19.65960631 | 15.89481587 | 18.86036143 | 15.34781535 | 18.29347725 | 17.76780388 | 13.38687383 | 15.84491696 | 14.02949261 | 14.93658397 | 16.02554228 | 14.38920535 |
| ENSMUSG00000002329 | Mdp1 | 1727 | 38.34572336 | 38.98399491 | 41.17492161 | 35.29859718 | 36.78873147 | 28.8651129 | 34.60099217 | 36.86550944 | 43.33288891 | 28.58528449 | 35.82430297 | 28.57037969 | 34.22098257 | 35.74070188 | 31.01448909 | 28.75675611 | 32.33434545 | 27.17010309 |
| ENSMUSG00000002332 | Dhrs1 | 3150 | 24.31565943 | 28.47988068 | 27.51916394 | 27.03288631 | 28.75691439 | 26.12923507 | 22.93538436 | 22.06672139 | 25.07801182 | 24.65308199 | 25.35864463 | 25.30310037 | 22.64569917 | 22.18995286 | 23.97538157 | 23.2904139 | 22.24141928 | 20.43195393 |
| ENSMUSG00000002341 | Ncan | 7180 | 0 | 0.004639679 | 0 | 0 | 0 | 0 | 0 | 0 | 0 | 0 | 0 | 0 | 0 | 0 | 0 | 0 | 0 | 0 |
| ENSMUSG00000002342 | Tmem161a | 3697 | 12.56644777 | 10.6777873 | 9.682556793 | 12.59491136 | 8.931160302 | 10.57536024 | 9.662178142 | 10.69221977 | 10.69490266 | 13.88886012 | 11.68205639 | 9.70772817 | 12.20954945 | 11.08025022 | 11.33404986 | 9.404347179 | 11.10291155 | 10.93635203 |
| ENSMUSG00000002343 | Armc6 | 3933 | 1.826436499 | 1.134993091 | 1.562023482 | 1.598633135 | 1.431842123 | 1.814287728 | 1.407470998 | 1.903308129 | 1.685997721 | 1.330721602 | 2.538765909 | 2.057785679 | 1.128876826 | 0.82661737 | 2.996095852 | 1.625866116 | 1.120651956 | 1.552507207 |
| ENSMUSG00000002345 | 2310045N01R | 2858 | 10.91630483 | 10.09411 | 11.627927 | 10.66491906 | 9.212093158 | 10.99245384 | 11.25704047 | 11.21183542 | 13.34454639 | 9.684206722 | 10.11331346 | 9.139863573 | 13.18739846 | 12.05791212 | 11.61947307 | 11.89715163 | 8.743437922 | 11.20199444 |
| ENSMUSG00000002346 | Slc25a42 | 6877 | 0.939477498 | 1.118987722 | 1.29427506 | 0.781765775 | 1.23881787 | 1.505965002 | 1.809398656 | 1.810487594 | 2.850775266 | 2.242006684 | 1.639507082 | 2.142698113 | 2.56810039 | 1.55331396 | 2.198774322 | 1.22494309 | 1.549325366 | 1.735782486 |
| ENSMUSG00000002365 | Snx9 | 3288 | 24.79468602 | 25.0251973 | 24.5251445 | 24.60961519 | 22.99733944 | 27.23290437 | 19.82873223 | 20.37392756 | 20.58073157 | 20.59258208 | 24.5268035 | 20.54043544 | 18.45445811 | 17.16227682 | 17.83145196 | 19.72758179 | 22.35700653 | 19.94247421 |
| ENSMUSG00000002372 | Ranbp3 | 2463 | 27.37036508 | 32.75835558 | 29.75472939 | 34.73960262 | 31.59939934 | 29.33328687 | 22.57101004 | 28.56289291 | 26.95601438 | 24.69525262 | 32.31550176 | 29.2108766 | 24.97641122 | 30.9250115 | 25.00865857 | 23.67705849 | 22.88996457 | 25.95232725 |
| ENSMUSG00000002379 | Ndufa11 | 2657 | 75.41189304 | 64.35644939 | 67.43528658 | 61.86836581 | 68.9462181 | 65.01348485 | 77.51301896 | 66.68218094 | 63.46164679 | 68.49895166 | 71.07806205 | 64.93271097 | 75.04689862 | 71.8773381 | 70.40085199 | 66.17631509 | 63.75695608 | 68.92183526 |
| ENSMUSG00000002384 | Bmp8b | 3870 | 0.01098325 | 0 | 0 | 0 | 0 | 0.012804324 | 0.012225498 | 0.069081865 | 0 | 0.109652798 | 0 | 0 | 0.089230858 | 0 | 0 | 0.019788428 | 0.059420615 | 0 |
| ENSMUSG00000002393 | Nr2f6 | 3217 | 11.36290065 | 13.0890576 | 11.80392332 | 14.70076594 | 12.26862835 | 13.00046849 | 10.60380526 | 10.43553675 | 11.71453588 | 12.73669914 | 8.55404914 | 11.62464841 | 6.609280804 | 9.254168485 | 9.080479679 | 7.248672648 | 7.10055112 | 9.387628098 |
| ENSMUSG00000002395 | Use1 | 2334 | 42.25021782 | 34.69736326 | 35.42003572 | 34.57095524 | 36.06800964 | 29.3834547 | 37.88663107 | 36.98150361 | 37.58666606 | 37.4741022 | 37.43804587 | 39.41054496 | 38.46790465 | 41.58875006 | 32.07507586 | 33.3689355 | 35.32127877 | 36.01281708 |
| ENSMUSG00000002396 | Ocel1 | 7775 | 2.804521582 | 2.943531618 | 3.01129115 | 3.463225364 | 2.426715637 | 3.377871228 | 2.683587319 | 3.114338666 | 3.501516002 | 3.062521562 | 2.556190704 | 3.058195789 | 2.918672919 | 2.634320584 | 3.068257345 | 2.216176634 | 2.272465964 | 2.143763259 |
| ENSMUSG00000002409 | Dyrk1b | 2829 | 7.52742778 | 8.148647154 | 7.814317205 | 7.907554885 | 7.264875011 | 9.020734462 | 9.917433229 | 7.357673846 | 6.172881825 | 7.300108882 | 8.173587806 | 8.187875565 | 5.248818346 | 4.760970429 | 5.927547984 | 5.468153196 | 5.947417582 | 6.416756882 |
| ENSMUSG00000002413 | Braf | 10697 | 5.408015759 | 6.016688009 | 5.783840827 | 6.116262472 | 5.660461809 | 5.716375922 | 4.865284362 | 6.623063145 | 5.140132611 | 5.373161379 | 5.895386107 | 5.656181943 | 4.966857521 | 5.075548112 | 5.615767831 | 5.505371056 | 7.104893592 | 5.461313578 |
| ENSMUSG00000002416 | Ndufb2 | 4711 | 10.4751665 | 8.549201679 | 10.22713569 | 9.342383534 | 10.26800749 | 9.308887543 | 10.65564686 | 10.02844444 | 10.46492451 | 9.618303084 | 10.17157363 | 9.276939886 | 9.078915788 | 8.241829135 | 8.535919879 | 9.005729782 | 9.510388682 | 8.488987601 |
| ENSMUSG00000002428 | Hltf | 6079 | 7.551503555 | 7.617194105 | 7.774459476 | 7.262485929 | 7.862312914 | 7.523798831 | 7.860800414 | 7.074295955 | 6.571955961 | 5.351864657 | 4.511075204 | 5.160125411 | 4.54447667 | 6.073868971 | 4.439384621 | 6.909820089 | 7.87457556 | 5.24844249 |
| ENSMUSG00000002455 | Prpf6 | 3837 | 37.45373994 | 39.31216706 | 35.79162176 | 40.98950353 | 34.94801651 | 34.72694829 | 29.06332662 | 28.14910451 | 27.14638998 | 30.00834437 | 31.71300997 | 30.08256196 | 31.57654126 | 32.12473493 | 33.33063057 | 34.22902877 | 33.06229939 | 32.07072498 |
| ENSMUSG00000002458 | Rgs19 | 2429 | 31.568274 | 28.84191706 | 23.53952774 | 24.79691256 | 28.37583973 | 29.37667203 | 12.89460414 | 13.30208828 | 13.12406883 | 14.92749724 | 13.90567956 | 18.865648 | 10.88592329 | 8.699901096 | 9.838164848 | 18.4753363 | 10.50856885 | 9.806069663 |
| ENSMUSG00000002459 | Rgs20 | 4922 | 0.112264788 | 0.040608973 | 0 | 0.027769836 | 0 | 0 | 0.115349888 | 0.007759529 | 0.075310573 | 0.009579582 | 0 | 0 | 0.060136429 | 0.122668252 | 0 | 0 | 0.023360197 | 0 |
| ENSMUSG00000002475 | Abhd3 | 2775 | 0.091903083 | 0 | 0.400933765 | 0.049255183 | 0.26017256 | 0.089284205 | 1.108224912 | 0.192682391 | 0.074209938 | 0.271859911 | 0.223810616 | 0.422388798 | 3.591008032 | 5.221821717 | 1.870773349 | 2.249141984 | 3.715233826 | 4.202497523 |
| ENSMUSG00000002477 | Snrpd1 | 833 | 65.21202277 | 61.06697416 | 61.49768145 | 48.02232524 | 60.9016177 | 51.87272951 | 72.30376984 | 56.71548833 | 55.6240459 | 53.20727618 | 58.15583181 | 55.68165139 | 67.51306806 | 62.39007658 | 67.16892305 | 61.87173777 | 54.06170331 | 58.37701122 |
| ENSMUSG00000002486 | Tchp | 3978 | 3.216203616 | 3.34133856 | 2.614456769 | 4.203344211 | 2.976483967 | 3.213827366 | 2.045696525 | 3.331514237 | 3.199254241 | 3.413625575 | 2.498037045 | 3.591958175 | 3.534337965 | 3.560946871 | 3.925437326 | 2.87805219 | 3.391366644 | 2.931606492 |
| ENSMUSG00000002489 | Tiam1 | 10002 | 0.293227069 | 0.193176151 | 0.256328445 | 0.323418731 | 0.235799266 | 0.302211233 | 0.898761147 | 0.450580232 | 0.630027686 | 0.636407213 | 0.520642919 | 0.641751769 | 0.305796728 | 0.431843644 | 0.395455845 | 0.287122131 | 0.310380929 | 0.23099205 |
| ENSMUSG00000002496 | Tsc2 | 6299 | 11.073344 | 16.19369457 | 11.31198765 | 11.57289585 | 13.46379576 | 14.12083772 | 9.892173067 | 12.05373805 | 8.559001291 | 11.33293614 | 10.08742575 | 10.60665949 | 8.841996286 | 10.16033536 | 11.11964518 | 9.97537494 | 10.61137669 | 9.52769256 |
| ENSMUSG00000002500 | Rpl3l | 1584 | 2.280896441 | 2.208241094 | 1.526942246 | 2.070956561 | 1.640861034 | 2.846779517 | 9.438640493 | 7.522745928 | 6.968425647 | 4.554329292 | 3.860602705 | 4.721779537 | 7.287669598 | 9.177398581 | 4.057728989 | 2.731590048 | 3.629384176 | 5.174467741 |
| ENSMUSG00000002504 | Slc9a3r2 | 3350 | 4.808794542 | 5.021794502 | 3.609964952 | 5.222519709 | 2.686767082 | 6.360500131 | 2.019615847 | 2.929984304 | 1.930233724 | 2.449021602 | 1.711341796 | 2.765791042 | 2.253069655 | 2.606414456 | 2.152320084 | 2.011685643 | 1.567373933 | 1.757007289 |
| ENSMUSG00000002524 | Puf60 | 1900 | 203.6445355 | 196.5811421 | 191.1772839 | 202.7706268 | 182.090667 | 202.1229922 | 166.6900383 | 170.2778109 | 170.9890718 | 165.3500715 | 167.5140913 | 168.827691 | 154.1232952 | 158.0810069 | 161.9437997 | 155.480019 | 169.9468692 | 176.0580937 |
| ENSMUSG00000002546 | Golga2 | 5394 | 15.89413052 | 18.29924093 | 16.80608007 | 18.71770008 | 16.60613829 | 17.99662682 | 16.09543304 | 16.49764509 | 16.79835645 | 19.38825729 | 17.11183162 | 15.8734198 | 17.6146223 | 18.2624971 | 18.8133912 | 19.38666088 | 19.26972881 | 22.68080337 |
| ENSMUSG00000002550 | Uck1 | 3952 | 12.90642997 | 12.94752164 | 11.28552505 | 12.2433778 | 11.20480004 | 11.22208921 | 12.15140919 | 13.70365046 | 14.20476239 | 13.78012709 | 14.04719174 | 10.79027485 | 17.41346776 | 16.06504743 | 17.4105765 | 14.62057859 | 14.87665472 | 15.71489916 |
| ENSMUSG00000002565 | Scin | 3848 | 0.12150648 | 0.424202655 | 0.113139754 | 0.213123388 | 0.562873328 | 0.373448356 | 0.196726316 | 0.079402084 | 0.321100695 | 0.441118845 | 0.297972714 | 0.188566428 | 0.807669124 | 2.426003525 | 0.813752143 | 1.044832063 | 0.527882987 | 0.643297766 |
| ENSMUSG00000002578 | Ikzf4 | 8382 | 0.471603599 | 0.413331066 | 0.421291782 | 0.385926288 | 0.356022342 | 0.484767854 | 0.445920023 | 0.565003328 | 0.363612761 | 0.208133622 | 0.381880484 | 0.426175424 | 0.382554835 | 1.257791438 | 0.614435038 | 0.319773624 | 0.745309758 | 0.741592586 |
| ENSMUSG00000002580 | 1810046J19R | 743 | 160.6958808 | 118.4109739 | 135.0943142 | 123.5605711 | 133.8365468 | 118.2462943 | 209.245576 | 169.5783789 | 170.5110666 | 183.081802 | 166.7944042 | 151.445997 | 237.2978093 | 231.0327685 | 184.3256686 | 156.8729595 | 162.4869923 | 180.1307627 |

| ENSMUSG00000002588 | Pon1 | 2890 | 0.04412302 | 0.023053906 | 0 | 0 | 0 | 0 | 0 | 0 | 0 | 0.016315122 | 0 | 0 | 0.017069868 | 0.032141237 | 0 | 0 | 0 | 0 |
| --- | --- | --- | --- | --- | --- | --- | --- | --- | --- | --- | --- | --- | --- | --- | --- | --- | --- | --- | --- | --- |
| ENSMUSG00000002602 | Axl | 6694 | 1.866824283 | 1.687043791 | 2.232958004 | 2.143969072 | 2.05642817 | 2.494662566 | 1.300497897 | 1.55188728 | 1.735075806 | 1.80319391 | 0.656602357 | 1.776028702 | 0.567457071 | 0.867270828 | 0.535484817 | 0.680696487 | 0.904624277 | 0.62454319 |
| ENSMUSG00000002603 | Tgfb1 | 2551 | 5.79843248 | 6.620790815 | 5.631884953 | 6.108144713 | 5.075455691 | 6.332493589 | 4.914880406 | 4.9406087 | 4.230053755 | 5.378618051 | 3.651947042 | 6.367055199 | 3.287505267 | 2.057303753 | 4.02163364 | 3.527358973 | 2.554084858 | 2.11324675 |
| ENSMUSG00000002608 | Ccdc97 | 3902 | 13.13717127 | 13.94155722 | 14.43023817 | 17.98155005 | 15.06127084 | 14.95979502 | 13.83489663 | 14.05543051 | 12.19129311 | 15.72092899 | 12.7946775 | 14.9766783 | 14.18513869 | 12.95006845 | 13.89575796 | 13.35559115 | 10.68657382 | 14.73206472 |
| ENSMUSG00000002617 | Zfp40 | 7290 | 2.664590596 | 3.162211632 | 3.822106102 | 2.949905751 | 3.75679898 | 2.875282072 | 5.555507978 | 5.768152956 | 4.734471908 | 3.54438757 | 4.095932654 | 5.41312393 | 4.033171838 | 3.510383011 | 4.950968856 | 4.38582403 | 3.60130499 | 3.516357165 |
| ENSMUSG00000002625 | Akap8l | 2075 | 48.22032982 | 50.97274176 | 47.32446584 | 55.90285247 | 49.91802397 | 48.81242768 | 51.73612939 | 51.38948796 | 47.81605613 | 61.39816877 | 48.67289746 | 47.1272486 | 49.59343587 | 61.64193574 | 57.70203442 | 60.54529296 | 58.92090946 | 62.80316811 |
| ENSMUSG00000002633 | Shh | 2689 | 0 | 0.061942905 | 0.395767076 | 0.169434899 | 0.1431965 | 0.055283823 | 0.879744867 | 0.781175951 | 0.45949999 | 0.227950592 | 0.177668124 | 0.477411766 | 0.660449618 | 2.193528858 | 0.551601343 | 0.455671044 | 0.085517955 | 0.061371261 |
| ENSMUSG00000002635 | Pdcd2l | 1466 | 26.99339624 | 21.72382461 | 19.23722251 | 20.54287197 | 20.55292238 | 22.61308238 | 20.04172825 | 21.44089166 | 22.13845458 | 20.19825487 | 22.45357871 | 24.48122172 | 21.16628647 | 22.11321487 | 18.18376989 | 16.42891682 | 15.47692208 | 18.98677217 |
| ENSMUSG00000002658 | Gtf2f1 | 1718 | 50.99136659 | 56.44577129 | 50.28819861 | 46.11796048 | 48.4120862 | 46.98568277 | 47.69823044 | 42.63854937 | 52.04652254 | 47.20559348 | 48.4979788 | 47.27112285 | 48.03975413 | 52.5807625 | 45.56633073 | 57.90395694 | 50.79684403 | 56.28987754 |
| ENSMUSG00000002660 | Clpp | 1201 | 94.81379399 | 77.97047931 | 86.95957705 | 80.84145336 | 90.17221337 | 84.33454407 | 97.57993814 | 85.25714475 | 92.04380375 | 98.18810062 | 101.0394626 | 85.2339094 | 97.84232053 | 94.70572025 | 96.39997673 | 103.8086742 | 90.66195629 | 96.87282325 |
| ENSMUSG00000002661 | Alkbh7 | 1250 | 27.4073375 | 22.59946744 | 26.66348993 | 23.58239655 | 22.2562015 | 20.49501061 | 27.40350364 | 22.05993163 | 24.67896024 | 25.49910032 | 21.47962133 | 22.99605299 | 30.70418531 | 23.44497531 | 25.5779259 | 28.88643422 | 17.84472384 | 21.69559169 |
| ENSMUSG00000002664 | Pspn | 472 | 0.270160017 | 0.352890829 | 0.614917759 | 0.675693454 | 0.407897658 | 0.104984605 | 1.503580898 | 0.485496641 | 0.174519135 | 0 | 0.708526927 | 0 | 1.567751608 | 1.082383393 | 0.43645716 | 0 | 0.081199782 | 0.699268306 |
| ENSMUSG00000002668 | Dennd1c | 2496 | 5.279088369 | 5.125060649 | 5.019528991 | 5.366565318 | 3.991710016 | 3.950718743 | 4.151232646 | 3.044987211 | 3.679724766 | 3.06026199 | 3.923824715 | 2.526911007 | 2.628663861 | 2.939970275 | 2.244956571 | 2.132369563 | 1.965451123 | 2.975252167 |
| ENSMUSG00000002679 | Med6 | 1838 | 22.82514077 | 18.63202136 | 19.73892697 | 19.50845587 | 22.0233666 | 24.15628362 | 21.62276948 | 23.58453576 | 23.93209947 | 19.23994964 | 21.23620298 | 17.88652082 | 20.63995886 | 25.54677495 | 23.44767544 | 21.62440179 | 24.56386167 | 24.96060664 |
| ENSMUSG00000002688 | Prkd1 | 3759 | 1.413446928 | 2.37506136 | 1.801621242 | 1.902922043 | 2.343214161 | 1.700532756 | 0.85588246 | 0.477532033 | 0.208178718 | 0.576997167 | 0.266899209 | 0.089091241 | 0.813668229 | 0.197686991 | 0.350744823 | 0.295405058 | 0.428226781 | 0.453653181 |
| ENSMUSG00000002699 | Lcp2 | 4725 | 3.454388909 | 2.453521098 | 1.832563372 | 2.487777659 | 2.58740923 | 2.265267821 | 8.891779659 | 4.025357968 | 3.86150825 | 5.857664087 | 3.983774315 | 4.158119202 | 14.73171117 | 5.809196962 | 6.853846123 | 24.94359568 | 5.86453178 | 8.917878466 |
| ENSMUSG00000002718 | Cse1l | 5087 | 23.80523816 | 24.06622494 | 25.6844713 | 22.91934587 | 23.25698218 | 24.13832786 | 18.46189653 | 25.72187353 | 22.69428026 | 26.34210707 | 23.36624665 | 20.53998828 | 18.09580453 | 18.150353 | 20.10266596 | 18.69743833 | 20.62100936 | 15.90689915 |
| ENSMUSG00000002728 | Naa20 | 3592 | 22.94474842 | 20.56088154 | 19.99852243 | 17.94790583 | 21.01081746 | 21.36892659 | 20.09998554 | 21.99890885 | 21.40735661 | 19.47985625 | 20.16337334 | 21.0707234 | 20.83422007 | 20.37747264 | 22.1836885 | 22.11943495 | 19.68597377 | 21.68510443 |
| ENSMUSG00000002731 | Prkra | 2020 | 8.374782207 | 7.685053815 | 9.123918349 | 7.668690631 | 6.242853466 | 8.095248555 | 8.525651054 | 8.394765677 | 12.66179519 | 8.263044036 | 8.940066488 | 7.957876646 | 8.840670324 | 6.966613108 | 6.894121728 | 7.866634689 | 8.651876925 | 11.32860812 |
| ENSMUSG00000002732 | Fkbp7 | 1768 | 6.82775452 | 6.217904464 | 6.210854859 | 5.102424648 | 7.786046476 | 7.287166703 | 3.960563621 | 6.567019418 | 3.820468657 | 5.840500008 | 5.998891845 | 5.619450193 | 3.90637354 | 4.150546271 | 5.56966734 | 4.331516674 | 4.790787113 | 4.729289718 |
| ENSMUSG00000002733 | Plekha3 | 3118 | 45.58605152 | 53.37755597 | 49.3509301 | 49.65247872 | 53.1951921 | 54.16155101 | 50.33231431 | 51.98478377 | 53.20695494 | 50.8404953 | 53.49017962 | 51.25087941 | 44.07912814 | 56.09635443 | 51.00641621 | 46.5062637 | 54.19520303 | 54.37397913 |
| ENSMUSG00000002741 | Ykt6 | 2977 | 40.86322264 | 35.64043268 | 36.10547009 | 35.49078328 | 34.04965779 | 38.40045561 | 41.8455773 | 44.18361904 | 39.41564777 | 42.03492323 | 36.76601608 | 36.54172871 | 53.12668014 | 42.07581582 | 47.41569717 | 42.75377192 | 50.05463297 | 44.79075401 |
| ENSMUSG00000002748 | Baz1b | 10776 | 6.93430767 | 5.613976977 | 4.84812665 | 5.403397797 | 5.645763875 | 4.759380035 | 5.224766882 | 5.11075021 | 5.431146891 | 5.036234173 | 5.098478308 | 5.200345698 | 5.87807923 | 6.107207854 | 4.882552605 | 6.509687524 | 5.605256488 | 4.027671233 |
| ENSMUSG00000002763 | Pex6 | 4686 | 12.98013378 | 13.49293069 | 11.6753015 | 13.56330705 | 12.23327381 | 11.60037318 | 13.60012346 | 14.39346621 | 13.83430982 | 14.40883633 | 12.22410909 | 13.31666897 | 12.23296796 | 9.574261293 | 12.2215456 | 10.92499831 | 8.702343129 | 11.22251516 |
| ENSMUSG00000002767 | Mrpl2 | 1209 | 65.84962341 | 60.23323967 | 60.05679823 | 61.16259302 | 62.86212363 | 64.96367471 | 71.41905628 | 65.64417882 | 69.70025744 | 66.57258103 | 70.29913245 | 68.92703411 | 74.9159637 | 66.38162366 | 62.46687506 | 65.90798511 | 54.93752898 | 68.70450881 |
| ENSMUSG00000002768 | Mea1 | 836 | 137.3791694 | 129.6254126 | 129.3239717 | 115.8100708 | 136.4505492 | 112.027113 | 166.6130703 | 130.8409575 | 140.0637133 | 150.1377656 | 149.49676 | 132.8626418 | 181.1000647 | 160.9987623 | 142.5297843 | 159.9411496 | 148.3080987 | 152.3936497 |
| ENSMUSG00000002769 | Gnmt | 1201 | 85.47044139 | 77.99821694 | 81.52208613 | 79.93099119 | 84.84203364 | 78.76450325 | 128.1499955 | 111.4607581 | 102.0575112 | 105.5296339 | 111.7400986 | 115.8140143 | 130.2099732 | 143.160709 | 133.9650922 | 116.9760521 | 112.4259317 | 134.1104617 |
| ENSMUSG00000002771 | Grin2d | 6606 | 1.293300088 | 1.623789273 | 1.391306504 | 1.213857675 | 1.13663035 | 1.500234138 | 2.821858235 | 2.266336927 | 1.87664869 | 2.690859087 | 1.706765092 | 2.458728098 | 0.672096966 | 1.701403141 | 0.979207429 | 1.008562774 | 1.189356776 | 1.249071451 |
| ENSMUSG00000002778 | Kdelr1 | 2143 | 65.9693026 | 61.40267483 | 61.14974043 | 52.72580026 | 58.14911357 | 58.38574535 | 59.65415701 | 56.88761012 | 65.4602299 | 53.70735728 | 57.04905215 | 55.73752587 | 68.27739927 | 49.00143798 | 51.64133417 | 56.56932479 | 56.35378513 | 55.67650606 |
| ENSMUSG00000002781 | Tmem143 | 5232 | 1.706056377 | 2.005650168 | 2.16349505 | 1.854836094 | 1.793907706 | 1.96998635 | 3.626220231 | 2.569519428 | 2.841806611 | 3.11814666 | 2.218905755 | 2.635028413 | 1.857490004 | 2.361262853 | 2.582972158 | 1.954050492 | 2.681082696 | 2.4918116 |
| ENSMUSG00000002791 | Pdxk-ps | 2639 | 0.257704743 | 0.037869906 | 0.128311752 | 0.103587066 | 0.072954791 | 0.037554175 | 0.233067384 | 0 | 0.046820594 | 0.303737005 | 0 | 0.063450924 | 0.037386826 | 0 | 0.359088968 | 0.087057084 | 0.072615189 | 0.187602107 |
| ENSMUSG00000002797 | Ggct | 4786 | 1.021332061 | 1.106717547 | 0.990515248 | 0.704454091 | 0.995624831 | 1.045722125 | 0.939135917 | 0.909722916 | 1.144547972 | 1.034438747 | 1.167921049 | 0.909657685 | 3.07164884 | 2.435742987 | 2.840892905 | 2.272154723 | 1.761760409 | 2.666550932 |
| ENSMUSG00000002799 | Jag2 | 4028 | 0.590935913 | 1.703688235 | 1.77737897 | 2.533682704 | 1.445869604 | 1.845310337 | 1.891097643 | 2.597993609 | 1.186106214 | 2.903022445 | 2.395864353 | 2.521958935 | 1.947312524 | 2.236880078 | 2.403765053 | 1.891715708 | 1.341610691 | 2.144098748 |
| ENSMUSG00000002803 | Btbd6 | 2083 | 23.56864057 | 24.19702782 | 22.47987392 | 20.12297685 | 21.46642203 | 22.43313863 | 21.3736106 | 22.51573219 | 20.08905426 | 18.90102603 | 20.0916292 | 20.49879111 | 26.69086451 | 22.85414766 | 21.85680233 | 25.27584502 | 21.95068277 | 19.62158728 |
| ENSMUSG00000002804 | Nudt14 | 788 | 74.92346384 | 72.92480016 | 74.46331513 | 75.97361962 | 79.83302568 | 79.35983474 | 87.00009107 | 74.73690927 | 76.31004189 | 82.81291029 | 81.30230878 | 84.07765418 | 98.91425038 | 88.2319782 | 74.14188611 | 94.65749139 | 87.79056588 | 88.79642608 |
| ENSMUSG00000002808 | Epdr1 | 2383 | 2.104746442 | 3.019548951 | 2.760721833 | 3.537051281 | 2.928715456 | 1.892278118 | 2.183967554 | 1.378324216 | 1.935748957 | 1.503757217 | 1.343232152 | 1.31165636 | 0.828064075 | 0.604182421 | 0.553273096 | 1.333663623 | 2.139067347 | 0.715603151 |
| ENSMUSG00000002812 | Flii | 5313 | 29.94482851 | 31.81434694 | 29.98193779 | 33.31538815 | 29.97713823 | 33.83725154 | 26.23435952 | 26.33859635 | 21.24055208 | 24.50274641 | 19.76460733 | 19.92891246 | 22.57216091 | 26.71431038 | 27.23505821 | 21.47675702 | 24.53373532 | 24.87989525 |
| ENSMUSG00000002814 | Top3a | 4995 | 2.867716581 | 3.121208107 | 3.389536662 | 3.356649513 | 2.958258372 | 3.115026695 | 3.788803117 | 3.035512263 | 3.726988013 | 3.294413501 | 3.175432676 | 2.81592532 | 3.891246328 | 2.817329025 | 3.546880687 | 2.115757286 | 3.237977436 | 3.601196776 |
| ENSMUSG00000002820 | Atg4d | 2531 | 15.71902203 | 12.80657688 | 15.90153191 | 15.53503803 | 12.0187182 | 14.78163329 | 18.24463636 | 16.20649553 | 16.6959167 | 18.64790753 | 20.25386426 | 17.44377286 | 18.69194337 | 17.01053695 | 18.3461689 | 17.44333083 | 16.62673805 | 16.62661661 |
| ENSMUSG00000002825 | Qtrt1 | 1309 | 11.62479222 | 10.40868887 | 11.04941602 | 9.153976058 | 8.346788902 | 9.009587923 | 7.445692788 | 8.957270847 | 10.79218862 | 9.365303932 | 12.11710177 | 8.527985116 | 12.13512404 | 10.43129235 | 9.663008142 | 11.64221677 | 9.544975394 | 8.824990383 |
| ENSMUSG00000002831 | Plin4 | 5746 | 7.11625119 | 6.186017774 | 6.802264624 | 4.717859621 | 6.081409082 | 7.459644021 | 3.812351261 | 2.512483138 | 3.440572149 | 3.438243067 | 2.82692062 | 3.322129587 | 3.142269705 | 2.675425152 | 2.39493903 | 3.365255262 | 2.521291373 | 2.096587952 |

| ENSMUSG00000002833 | Hdgfrp2 | 2454 | 31.14274918 | 30.78795603 | 32.05189909 | 33.60451925 | 29.51855954 | 33.27746737 | 32.21657966 | 30.02165617 | 27.42411033 | 30.18490416 | 28.44303763 | 29.97760525 | 31.46065629 | 41.95865597 | 34.04920756 | 32.26771642 | 29.97072688 | 33.96038985 |
| --- | --- | --- | --- | --- | --- | --- | --- | --- | --- | --- | --- | --- | --- | --- | --- | --- | --- | --- | --- | --- |
| ENSMUSG00000002835 | Chaf1a | 2830 | 0.690896854 | 1.294847478 | 0.837562893 | 0.91766061 | 1.03747256 | 0.857980193 | 2.373993077 | 1.376545952 | 1.004194201 | 0.766407191 | 1.856977185 | 2.268127632 | 1.011042828 | 1.920126581 | 0.975443196 | 0.554740249 | 1.029257443 | 0.699762488 |
| ENSMUSG00000002844 | Adprh | 1579 | 26.08455704 | 31.60399971 | 32.10605435 | 25.94007527 | 31.76280207 | 31.79032243 | 36.04632853 | 35.9671326 | 36.38707377 | 38.1027849 | 39.3334047 | 43.12546444 | 36.30379219 | 37.14938719 | 39.11407997 | 27.88739614 | 38.61756705 | 39.4365477 |
| ENSMUSG00000002845 | Tmem39a | 3017 | 6.325762027 | 6.602953511 | 7.391513919 | 5.844257261 | 5.440167707 | 5.962095555 | 7.041230639 | 5.304148693 | 6.784785659 | 5.704344279 | 7.949297036 | 6.734140266 | 8.371873267 | 9.313448084 | 8.863045997 | 9.264880147 | 7.92695037 | 8.988892812 |
| ENSMUSG00000002846 | 4930455C21R | 1776 | 3.566031097 | 5.83350794 | 4.929960019 | 3.745446212 | 4.715627657 | 4.743223372 | 3.250082674 | 4.709536107 | 3.68730631 | 4.460201659 | 4.357850942 | 4.179889146 | 6.722029269 | 3.582680165 | 3.363865768 | 3.06152379 | 2.438553801 | 2.137178133 |
| ENSMUSG00000002847 | Pla1a | 1961 | 3.749819201 | 3.703320214 | 5.821597738 | 2.880963538 | 3.878043824 | 5.180168477 | 1.713003674 | 1.577554612 | 2.121284086 | 2.067802387 | 2.022091562 | 2.447805702 | 2.289242464 | 3.434162505 | 2.374184506 | 5.603979768 | 3.302980202 | 4.796816547 |
| ENSMUSG00000002870 | Mcm2 | 3354 | 19.18689222 | 20.54990404 | 19.52825286 | 19.12640144 | 21.38239901 | 22.64887912 | 23.75508387 | 23.45746838 | 21.84579951 | 22.56317195 | 24.18660689 | 21.63397775 | 16.88522392 | 19.20630567 | 21.87834557 | 19.0425561 | 19.75735461 | 20.66531737 |
| ENSMUSG00000002871 | Tpra1 | 2339 | 50.04671004 | 42.81255238 | 43.65819691 | 47.5284088 | 47.43227193 | 45.97239498 | 41.99278386 | 34.28988673 | 42.41911777 | 39.59127029 | 40.03374032 | 51.52031446 | 45.72535128 | 33.08073946 | 41.92377213 | 48.80047057 | 41.42320589 | 33.44290285 |
| ENSMUSG00000002881 | Nab1 | 4162 | 11.26458653 | 11.74195479 | 12.81979913 | 14.80022392 | 14.37481526 | 12.81084607 | 8.821393284 | 9.873864726 | 9.28230461 | 9.96939425 | 7.90892513 | 10.63474782 | 7.135467131 | 11.58312415 | 10.42413223 | 9.752052822 | 12.34876601 | 7.652636423 |
| ENSMUSG00000002885 | Cd97 | 4133 | 2.026014923 | 1.418598931 | 2.598336254 | 1.201585733 | 2.072945176 | 2.325969106 | 6.502202185 | 6.579479921 | 4.882988803 | 4.91699809 | 4.138261586 | 6.239253839 | 7.424256604 | 14.88952717 | 6.021229073 | 4.372892982 | 3.783481523 | 8.571464972 |
| ENSMUSG00000002897 | Il17ra | 3957 | 4.167805989 | 4.11675645 | 4.339803709 | 4.674699014 | 4.050525798 | 4.984075806 | 5.882698517 | 4.121343398 | 3.372360654 | 5.385928196 | 6.350676816 | 5.487059072 | 4.737459834 | 4.847462242 | 5.674715178 | 4.557714452 | 4.93970468 | 5.143636464 |
| ENSMUSG00000002900 | Lamb1 | 9879 | 6.423750158 | 8.305461936 | 7.056003365 | 5.801780927 | 6.129158819 | 6.03922373 | 2.686750975 | 4.492314161 | 2.63486972 | 2.601187699 | 2.287433481 | 3.101811799 | 3.720242775 | 1.99805012 | 2.894410344 | 2.693792099 | 2.160921892 | 2.171631908 |
| ENSMUSG00000002908 | Kcnn1 | 4039 | 0.210473761 | 0.329912297 | 0.37127493 | 0.428650578 | 0.774591493 | 0.662502504 | 0.983972525 | 0.160750394 | 0.112169268 | 0.198455547 | 0.260225077 | 0.373117824 | 0.610694693 | 0.333468317 | 0.520247425 | 0.227525273 | 0.389051293 | 0.190672814 |
| ENSMUSG00000002910 | Arrdc2 | 2135 | 65.40023571 | 64.98744938 | 54.67228513 | 58.85590645 | 56.60855285 | 55.70330707 | 73.43991474 | 64.95391716 | 53.82219156 | 58.89973095 | 71.2708397 | 60.78286441 | 56.31001518 | 59.69207297 | 45.54364024 | 42.45146029 | 45.38120776 | 53.54047952 |
| ENSMUSG00000002930 | Ppp1r17 | 1733 | 0 | 0 | 0 | 0 | 0 | 0 | 0 | 0 | 0 | 0 | 0 | 0 | 0 | 0 | 0 | 0 | 0 | 0 |
| ENSMUSG00000002944 | Cd36 | 3910 | 0 | 0 | 0 | 0 | 0 | 0 | 0.012100429 | 0.117214534 | 0.273874529 | 0.012059003 | 0 | 0.142751055 | 0 | 0 | 0 | 0 | 0 | 0 |
| ENSMUSG00000002948 | Map2k7 | 5614 | 8.653975098 | 9.832466284 | 10.51223852 | 10.15263029 | 10.40829272 | 9.982925129 | 9.304096462 | 9.932473728 | 8.14339731 | 10.36408404 | 11.53100619 | 9.643960226 | 9.411201176 | 11.60688539 | 9.093111462 | 9.003135335 | 10.23354454 | 9.347824691 |
| ENSMUSG00000002949 | Timm44 | 4159 | 17.91574262 | 15.53907545 | 15.71354641 | 16.89231314 | 12.96171063 | 12.99880556 | 17.63276084 | 15.53776025 | 16.20128391 | 15.08958549 | 19.20647525 | 15.4201001 | 16.26209632 | 15.7568183 | 16.75206324 | 14.31639685 | 16.91923085 | 13.00167153 |
| ENSMUSG00000002957 | Ap2a2 | 4617 | 40.52583601 | 43.00299969 | 43.34444338 | 46.93271104 | 43.37815343 | 45.77483403 | 34.53405414 | 34.03152339 | 33.62182757 | 33.7928692 | 38.97948204 | 33.06385904 | 32.56739958 | 32.35091727 | 32.13489339 | 38.45647531 | 36.28422 | 39.62752631 |
| ENSMUSG00000002963 | Pnkp | 3116 | 21.36171555 | 21.11455908 | 19.52949332 | 22.22489968 | 21.30100216 | 21.64353992 | 18.52421961 | 15.5907368 | 17.34169085 | 19.39897355 | 19.73246841 | 18.44997833 | 16.84504492 | 21.99983087 | 19.91320384 | 19.20674563 | 17.02297654 | 19.80751532 |
| ENSMUSG00000002968 | Med25 | 2521 | 32.27088731 | 43.15744249 | 34.30855703 | 41.94652277 | 38.43298784 | 43.04660315 | 32.07352967 | 35.34425816 | 32.21729833 | 41.1469842 | 37.78788862 | 26.19195908 | 32.95317283 | 37.60110367 | 42.06775282 | 29.64826087 | 39.19285736 | 38.88386679 |
| ENSMUSG00000002980 | Bcam | 3244 | 28.70802734 | 35.58236081 | 34.37145113 | 36.40389239 | 35.13452381 | 35.97308495 | 35.57201723 | 44.49108776 | 41.2246044 | 46.45303564 | 53.57746577 | 46.404082 | 38.91503585 | 31.68334322 | 46.06598126 | 36.99222059 | 44.18629791 | 40.40897081 |
| ENSMUSG00000002981 | Clptm1 | 4116 | 109.4952336 | 124.5915193 | 122.1913496 | 116.9687939 | 115.3365314 | 129.2151335 | 95.38401597 | 109.1861028 | 101.3852716 | 111.8740933 | 113.0070446 | 103.3593246 | 100.1739953 | 103.991426 | 108.2692003 | 103.550231 | 102.1382776 | 108.1872495 |
| ENSMUSG00000002983 | Relb | 3316 | 30.63551589 | 37.59253624 | 32.7206962 | 38.51254743 | 41.93399502 | 39.37619209 | 27.03785482 | 38.2845433 | 34.55394658 | 34.282372 | 40.00936662 | 40.21218876 | 23.92211188 | 28.9505213 | 28.10552456 | 37.20516799 | 32.06186599 | 31.71815804 |
| ENSMUSG00000002984 | Tomm40 | 4759 | 17.0235061 | 16.05094903 | 17.10709216 | 17.27088127 | 15.36297374 | 16.39957037 | 18.12376837 | 17.47910327 | 16.72039261 | 15.03987551 | 18.16030676 | 16.45500622 | 17.77773443 | 17.29332273 | 18.16366133 | 17.11370497 | 16.75919812 | 14.95729861 |
| ENSMUSG00000002985 | Apoe | 2810 | 31.88644522 | 37.98381248 | 26.13203526 | 44.40985781 | 31.70540595 | 29.66110245 | 57.81912436 | 43.79214254 | 53.01481406 | 91.71734662 | 39.08705678 | 51.92247046 | 209.5640913 | 153.8439735 | 176.8147909 | 146.8397101 | 116.0973093 | 142.769187 |
| ENSMUSG00000002992 | Apoc2 | 1189 | 0.035748676 | 0.028017573 | 0.081368428 | 0 | 0 | 0.041675974 | 0 | 0.128485794 | 0 | 0.356901875 | 0 | 0 | 0.414902584 | 0 | 0.207913655 | 0 | 0.161170298 | 0 |
| ENSMUSG00000002996 | Hbp1 | 6805 | 32.24272133 | 40.91048596 | 40.5825841 | 38.58461403 | 41.15084965 | 40.22473186 | 35.43062628 | 42.295069 | 41.98543128 | 39.95164658 | 48.51211801 | 40.25617516 | 33.47031037 | 37.87189433 | 37.69594224 | 41.21656124 | 48.45277476 | 34.85661546 |
| ENSMUSG00000002997 | Prkar2b | 3885 | 0 | 0.128621213 | 0.161867669 | 0.164183944 | 0.099113356 | 0.063774432 | 0.621093082 | 0.540690382 | 0.159021296 | 0.315551682 | 0.860810063 | 0.114935727 | 1.561859929 | 0.549917122 | 0.275737568 | 0.551936683 | 0.414338345 | 0.48141818 |
| ENSMUSG00000003031 | Cdkn1b | 4550 | 2.223347669 | 1.486267588 | 1.584100225 | 1.762361275 | 2.009904505 | 1.688060155 | 1.798921638 | 2.081695779 | 3.005257853 | 2.093283969 | 2.362497947 | 2.674244933 | 3.881500304 | 2.031290855 | 2.472093353 | 4.199343537 | 2.594395483 | 3.191737181 |
| ENSMUSG00000003032 | Klf4 | 4212 | 7.104378899 | 6.358871546 | 6.546275475 | 5.884427379 | 8.513362567 | 7.63526213 | 6.346548812 | 7.95221674 | 7.040430045 | 8.474141113 | 8.665733209 | 8.679786086 | 14.23036075 | 15.27185685 | 11.71877112 | 28.45432126 | 24.08587557 | 29.3590864 |
| ENSMUSG00000003033 | Ap1m1 | 3045 | 24.70744222 | 21.68346678 | 18.96814294 | 20.19947778 | 20.43828483 | 24.03592364 | 21.6752667 | 21.07167029 | 20.53239111 | 22.23593099 | 25.41721929 | 22.98615464 | 24.04222175 | 22.61956703 | 21.28408782 | 29.3624198 | 26.55779523 | 21.46168105 |
| ENSMUSG00000003037 | Rab8a | 2209 | 97.84464468 | 97.91789143 | 85.5131897 | 92.62772753 | 99.03105164 | 89.84096785 | 95.71768317 | 91.75512882 | 76.38802855 | 86.59592717 | 92.93299981 | 85.20163614 | 91.49518564 | 90.38620719 | 92.12063583 | 98.8899571 | 93.86385978 | 81.30590016 |
| ENSMUSG00000003038 | Hmgn2 | 3092 | 29.56941579 | 32.63413861 | 29.08356822 | 24.4161224 | 35.74091098 | 26.44308228 | 38.8967755 | 47.59227893 | 43.22452899 | 36.76595911 | 45.78176002 | 36.64482376 | 37.1744396 | 50.72499454 | 38.58981431 | 44.11097787 | 47.77152272 | 36.11529322 |
| ENSMUSG00000003039 | Fam32a | 1906 | 73.65928459 | 77.49704773 | 80.70714928 | 90.50058439 | 83.3343904 | 75.3950301 | 76.40526009 | 70.23314293 | 71.71985615 | 84.90095151 | 77.10166437 | 72.97619683 | 73.89434618 | 62.33157164 | 69.45466898 | 71.45838957 | 79.74926208 | 79.10805956 |
| ENSMUSG00000003051 | Elf3 | 1991 | 133.4504547 | 147.2225797 | 130.8101896 | 133.2277248 | 141.712374 | 131.4852293 | 149.8274438 | 170.8970934 | 135.5990009 | 149.4329169 | 165.4726839 | 173.3060905 | 127.5295722 | 167.9778369 | 144.6296706 | 232.5322622 | 204.3363343 | 207.2995117 |
| ENSMUSG00000003053 | Cyp2c29 | 3087 | 0 | 0 | 0 | 0 | 0 | 0 | 0 | 0 | 0 | 0 | 0 | 0 | 0 | 0 | 0 | 0 | 0 | 0 |
| ENSMUSG00000003062 | Stard3nl | 1274 | 73.96701354 | 74.41797249 | 71.38323161 | 74.1350431 | 79.67835715 | 68.96153582 | 62.2417974 | 69.2499604 | 71.96325282 | 63.84220025 | 62.54994564 | 71.7191834 | 63.19441836 | 62.77607416 | 61.18786793 | 67.6246991 | 67.92839749 | 56.95212855 |
| ENSMUSG00000003068 | Stk11 | 5550 | 7.145464727 | 5.510132774 | 6.746146395 | 5.311350574 | 5.324865069 | 4.589208119 | 4.305027542 | 5.099201835 | 8.25214514 | 5.624101902 | 4.209361207 | 5.923500047 | 7.155350163 | 7.330634334 | 6.503164497 | 9.369125198 | 4.861569913 | 5.649583934 |
| ENSMUSG00000003070 | Efna2 | 2127 | 1.458804819 | 1.080672168 | 0.977932207 | 0.835392914 | 0.701499593 | 1.397820411 | 0.355901675 | 0.574591856 | 0.193636651 | 0.908875803 | 0.516607449 | 0.498588429 | 0.46386382 | 0 | 0.368043988 | 0 | 0.216227345 | 0.155173785 |
| ENSMUSG00000003072 | Atp5d | 1920 | 191.0740491 | 194.8110293 | 188.6063793 | 195.2005993 | 191.550015 | 194.339627 | 220.9896378 | 198.7397356 | 186.2188247 | 213.8235277 | 225.711796 | 203.9597337 | 202.5691853 | 189.1145183 | 185.0636552 | 182.8376503 | 163.4656486 | 195.8266898 |

| ENSMUSG00000003099 | Ppp5c | 3267 | 18.91721027 | 17.66083036 | 20.4184568 | 17.3207276 | 18.19495737 | 18.17085241 | 18.58040008 | 18.90331028 | 17.44785363 | 16.4529543 | 18.86430867 | 20.36494331 | 17.69727795 | 17.03092032 | 20.1782949 | 16.47890848 | 17.11602913 | 14.29529587 |
| --- | --- | --- | --- | --- | --- | --- | --- | --- | --- | --- | --- | --- | --- | --- | --- | --- | --- | --- | --- | --- |
| ENSMUSG00000003119 | Cdk12 | 11101 | 3.254607659 | 2.865851183 | 2.74091979 | 2.561038795 | 3.078431295 | 2.133700266 | 2.78736078 | 3.53677954 | 3.465291931 | 2.811797636 | 3.180406658 | 3.087182851 | 3.261834725 | 2.736188916 | 3.39975004 | 3.056074062 | 2.737839244 | 2.958331386 |
| ENSMUSG00000003123 | Lipe | 5029 | 1.622786597 | 1.523556506 | 1.712167111 | 1.676037622 | 1.521768913 | 1.438595964 | 1.787514217 | 1.860636029 | 1.719858483 | 1.687637023 | 1.84297911 | 1.942282949 | 2.305229779 | 2.382695277 | 1.687715354 | 2.223276469 | 1.463242992 | 1.607941676 |
| ENSMUSG00000003131 | Pafah1b2 | 6876 | 26.09283712 | 25.56120272 | 25.1998234 | 27.06760706 | 27.30695669 | 26.63567529 | 19.0599361 | 25.02835446 | 21.92900439 | 24.48051347 | 22.55344899 | 23.75169121 | 19.06266786 | 22.31693786 | 21.42169318 | 20.67113651 | 24.57542802 | 23.12046516 |
| ENSMUSG00000003134 | Tbc1d8 | 4416 | 1.973179594 | 2.843967645 | 2.93571244 | 3.404697607 | 2.321580557 | 2.367669109 | 2.399918496 | 2.836754526 | 1.66947154 | 2.071385062 | 2.564009321 | 1.933830701 | 2.904505999 | 4.396202112 | 2.173904557 | 2.653289372 | 2.8900944 | 2.815230552 |
| ENSMUSG00000003135 | D1Bwg0212e | 3492 | 17.45487469 | 17.10481653 | 18.52102236 | 17.84865654 | 20.19280303 | 16.43243571 | 15.35087779 | 14.46980194 | 13.92934568 | 18.29587712 | 17.2657496 | 14.73712512 | 17.31985412 | 17.95518842 | 16.23520644 | 16.82067347 | 19.88752863 | 17.59598288 |
| ENSMUSG00000003153 | Slc2a3 | 7010 | 0.060635059 | 0.166326861 | 0.11041034 | 0.051995486 | 0.096126524 | 0.070688636 | 0.425206672 | 1.836068418 | 0.352523673 | 0.443929588 | 0.156750934 | 0.17517041 | 1.224501228 | 2.312266262 | 1.157875394 | 0.229415907 | 0.087477996 | 1.184932256 |
| ENSMUSG00000003154 | Foxj2 | 5272 | 8.731620948 | 12.65662502 | 11.30428479 | 13.4730023 | 12.10601874 | 13.7980677 | 10.16791829 | 16.05355913 | 11.41378974 | 12.85196521 | 11.94373962 | 13.30809709 | 10.26500631 | 9.038625505 | 10.15971598 | 11.12693675 | 10.86832585 | 11.4567525 |
| ENSMUSG00000003161 | Sri | 3725 | 47.04666866 | 44.50066084 | 45.59448727 | 44.15446176 | 48.22237292 | 43.49998357 | 52.87588789 | 46.90744725 | 46.75913427 | 53.36573555 | 45.6074503 | 45.35678138 | 51.22573316 | 55.79524592 | 48.51275816 | 58.52038334 | 50.96111371 | 45.17392147 |
| ENSMUSG00000003166 | Dgcr2 | 8258 | 10.92740236 | 10.25043161 | 9.536462511 | 10.41095794 | 8.684495794 | 10.26098019 | 8.800227032 | 7.79757695 | 8.88268159 | 9.044164926 | 8.938279347 | 9.171936804 | 8.895038124 | 9.448542845 | 8.846010971 | 9.482234456 | 8.980550316 | 9.978643966 |
| ENSMUSG00000003178 | Mical3 | 3295 | 5.443758508 | 5.510023475 | 7.296401999 | 7.6741668 | 6.39811307 | 5.88015746 | 5.930238664 | 5.760735659 | 5.074878725 | 5.967175496 | 4.639753175 | 5.522277998 | 4.596327346 | 4.891076881 | 5.264295912 | 5.77554837 | 5.54829852 | 5.47586859 |
| ENSMUSG00000003184 | Irf3 | 3282 | 24.9436225 | 21.39658166 | 19.69135788 | 19.96245635 | 21.3675237 | 21.83219158 | 23.02204395 | 21.01629456 | 24.93528544 | 19.35161404 | 21.20905378 | 20.62900631 | 25.02670392 | 25.79755372 | 21.55486638 | 25.13039864 | 19.27992571 | 21.2024741 |
| ENSMUSG00000003190 | Bcl2l12 | 1877 | 6.295385688 | 6.513496099 | 5.412062533 | 7.961652926 | 5.718390505 | 4.672793736 | 7.889647578 | 5.738016771 | 6.473107166 | 8.189200459 | 8.170357843 | 8.028891262 | 7.043662136 | 7.051979172 | 6.497421707 | 8.649556493 | 6.125673453 | 6.330296778 |
| ENSMUSG00000003198 | Zfp959 | 1616 | 5.207936172 | 5.442205491 | 6.346032448 | 5.977067698 | 6.433474946 | 5.028866526 | 9.603068495 | 6.688397203 | 7.008843957 | 4.755918707 | 5.587541555 | 6.562485081 | 5.922272247 | 4.598424494 | 4.614778228 | 6.492343087 | 6.118926114 | 8.03350816 |
| ENSMUSG00000003199 | Mpnd | 2282 | 74.44925004 | 71.3555771 | 67.74837994 | 71.3363766 | 69.47701865 | 70.55075872 | 68.89571958 | 72.65259371 | 68.11477234 | 68.90779814 | 67.51719592 | 72.39893131 | 79.92116484 | 68.4653418 | 65.12445754 | 71.74874551 | 61.13397052 | 67.85742131 |
| ENSMUSG00000003200 | Sh3gl1 | 2060 | 44.89867138 | 45.00474981 | 37.12551043 | 53.21353042 | 39.95416965 | 47.33969887 | 38.99850914 | 44.60724281 | 40.56671868 | 49.66845926 | 32.28288456 | 40.96761254 | 41.26159877 | 45.11389265 | 38.42145091 | 41.85944071 | 45.52643132 | 38.15922986 |
| ENSMUSG00000003206 | Ebi3 | 1165 | 0.547276945 | 0.200163313 | 1.204147966 | 0.117324578 | 0.784984163 | 0.127603606 | 0.649787865 | 0.426181314 | 1.095950205 | 0.283308947 | 0.656136769 | 0.81447748 | 0.465794927 | 0.358795525 | 0.601224421 | 0.78881938 | 0.230286763 | 0.472181174 |
| ENSMUSG00000003208 | Ccdc94 | 1303 | 37.48154049 | 33.67082248 | 36.19661711 | 34.47673808 | 37.75197697 | 34.15069436 | 54.28430928 | 52.55485611 | 52.75540661 | 60.82911142 | 58.33458092 | 50.58963739 | 59.66776794 | 60.16701421 | 61.09087182 | 52.77815111 | 55.6804912 | 64.33912406 |
| ENSMUSG00000003226 | Ranbp2 | 9448 | 8.142926398 | 8.73017846 | 10.1733917 | 9.114137785 | 9.745593774 | 9.403900435 | 7.786961868 | 9.471295389 | 9.747359149 | 8.858223781 | 8.131047125 | 7.998984668 | 6.297025004 | 7.879961059 | 8.817691153 | 8.462191814 | 9.861476267 | 7.528236134 |
| ENSMUSG00000003227 | Edar | 3625 | 0.469022632 | 0.909786628 | 0.587154575 | 1.219150704 | 0.969277359 | 0.738164859 | 1.070245427 | 1.717341129 | 1.238435922 | 0.416226898 | 0.263585978 | 0.585102118 | 0.979833943 | 0.525298644 | 1.420743307 | 0.073940483 | 0.729521238 | 0.424897928 |
| ENSMUSG00000003228 | Grk5 | 3149 | 0.499425695 | 0.370260812 | 0.184338636 | 0.245963085 | 0.489114499 | 0.267512376 | 0.150246678 | 0.315338985 | 0.680120298 | 0.269518151 | 0.561344384 | 0.141799079 | 0.642301876 | 0.353972086 | 0.353268342 | 0.59582082 | 0.425982976 | 0.314437574 |
| ENSMUSG00000003233 | Dvl3 | 4272 | 8.337859904 | 9.716260814 | 9.76076385 | 10.32375724 | 9.712012688 | 10.45107981 | 8.450274818 | 10.06663041 | 11.39570731 | 11.75456437 | 8.174975339 | 8.24430784 | 6.859353666 | 9.186623091 | 8.83441601 | 7.224304674 | 7.966702165 | 7.120794948 |
| ENSMUSG00000003234 | Abcf3 | 3538 | 24.19599336 | 25.58257026 | 25.70441975 | 26.10294993 | 23.91631481 | 22.07323576 | 29.86128096 | 26.89012843 | 24.97034378 | 25.66768074 | 29.01876364 | 25.84116883 | 30.53615004 | 30.92771902 | 32.95658654 | 28.61514018 | 29.07512179 | 35.3718818 |
| ENSMUSG00000003235 | Eif2b5 | 3193 | 23.69533772 | 27.91898056 | 25.70932697 | 26.59749034 | 26.99789392 | 28.02763446 | 24.5972587 | 23.74316279 | 21.01247547 | 27.70269944 | 22.68300565 | 21.0641633 | 21.7072796 | 24.46569214 | 20.2072147 | 19.39114067 | 18.49696947 | 21.77623266 |
| ENSMUSG00000003269 | Cyth2 | 3989 | 32.26514741 | 31.87648966 | 32.65728685 | 35.81835925 | 30.82904612 | 32.37263066 | 33.9455721 | 32.25625563 | 33.97964988 | 29.3495102 | 35.43898274 | 32.04258394 | 34.23182427 | 35.25512575 | 31.63709343 | 30.65936326 | 27.55573315 | 32.50350076 |
| ENSMUSG00000003271 | Sult2b1 | 2077 | 3.745039583 | 3.271945316 | 2.957842251 | 2.588446428 | 3.313849343 | 2.791367274 | 4.510308343 | 4.597063361 | 4.144430329 | 2.474447114 | 2.093173434 | 2.552955198 | 9.619367596 | 13.50612845 | 6.942968012 | 1.991037843 | 3.044698598 | 7.336313222 |
| ENSMUSG00000003273 | Car11 | 1918 | 0.84212549 | 0.712110877 | 0.857507837 | 0.736388099 | 0.526991865 | 0.465041295 | 1.52939838 | 0.83632998 | 0.472420932 | 0.884997559 | 0.797079599 | 0.989433018 | 0.617291978 | 0.920164401 | 1.224446656 | 1.137937759 | 0.359683704 | 0.487567682 |
| ENSMUSG00000003279 | Dlgap1 | 15584 | 1.309194334 | 1.496344068 | 2.185251885 | 1.932485261 | 1.914899427 | 2.133591134 | 2.914538743 | 3.656510805 | 3.377590294 | 3.936284968 | 3.387533953 | 4.083024599 | 3.197204598 | 2.273924453 | 3.006042675 | 2.803489672 | 2.980715596 | 2.721510606 |
| ENSMUSG00000003282 | Plag1 | 5429 | 0.180073503 | 0.128858128 | 0.240575671 | 0.134274583 | 0.124119899 | 0.018254829 | 0.339877414 | 0.429128117 | 0.257937288 | 0.260549106 | 0.184799066 | 0.411240837 | 0 | 0.230979989 | 0.424993024 | 0.246854164 | 0.2259056 | 0.101324566 |
| ENSMUSG00000003283 | Hck | 2079 | 0 | 0 | 0 | 0 | 0 | 0 | 2.116440183 | 0.238817331 | 0.832050823 | 0.340192664 | 2.412876691 | 0.241626245 | 2.538968325 | 0.156377399 | 0.614357014 | 0.368356012 | 0.018434967 | 0.07937822 |
| ENSMUSG00000003299 | Mrpl4 | 1276 | 86.97571678 | 80.38432704 | 83.21308709 | 87.19441244 | 85.85286698 | 80.07659611 | 85.76350028 | 95.87011356 | 84.53564638 | 95.70558106 | 101.9898016 | 87.70407797 | 100.4035965 | 98.23870836 | 93.44616203 | 96.1766432 | 87.94623615 | 88.37669967 |
| ENSMUSG00000003308 | Keap1 | 4465 | 12.64207699 | 12.97449564 | 12.46986191 | 13.65300724 | 11.57753326 | 14.5717221 | 13.15006373 | 14.47291039 | 10.71864083 | 14.17161115 | 13.20365034 | 15.03835209 | 11.75569093 | 12.84623694 | 13.69386538 | 12.21183089 | 13.30476152 | 11.65478499 |
| ENSMUSG00000003309 | Ap1m2 | 1727 | 107.3581806 | 101.5975761 | 109.5196895 | 109.7475069 | 106.8545428 | 105.5613821 | 110.0766323 | 102.3034473 | 115.7610582 | 105.3860536 | 115.661321 | 118.2568092 | 101.6631694 | 90.54849001 | 92.51860668 | 116.9116229 | 96.9364591 | 92.2764228 |
| ENSMUSG00000003316 | Glg1 | 7600 | 31.68872729 | 35.43878286 | 32.6712278 | 33.34948548 | 31.41874649 | 37.01458796 | 26.35814244 | 32.71983317 | 28.82514176 | 30.95195507 | 33.29160265 | 28.53942958 | 23.51055321 | 25.53202594 | 28.88988044 | 26.1131866 | 28.24547223 | 24.56590898 |
| ENSMUSG00000003341 | Atp8b3 | 5471 | 0 | 0.012177991 | 0.008841808 | 0.008327736 | 0.052785879 | 0.01811469 | 0.129718549 | 0.195464681 | 0.127978572 | 0.112037862 | 0 | 0.091818856 | 1.442717376 | 2.045882848 | 1.227536759 | 0.475920545 | 0.357273102 | 1.749512807 |
| ENSMUSG00000003344 | Btbd2 | 4393 | 8.050149432 | 10.69986199 | 9.888329219 | 11.97883137 | 10.58398321 | 11.08817143 | 10.25305494 | 9.41551828 | 8.991092332 | 11.96745598 | 10.17923085 | 9.986594763 | 8.905124152 | 8.880726931 | 7.878285215 | 8.777291521 | 8.384150085 | 8.27703609 |
| ENSMUSG00000003345 | Csnk1g2 | 2499 | 46.55328803 | 47.86978917 | 45.082814 | 53.87470429 | 46.41774151 | 48.2241889 | 35.63123719 | 46.79677318 | 42.52149286 | 42.24506786 | 39.15290715 | 37.7018161 | 48.50280942 | 49.67788939 | 41.46535137 | 46.93242516 | 49.35335071 | 42.57207379 |
| ENSMUSG00000003346 | Fam108a | 1879 | 64.44770969 | 67.68953173 | 63.1507557 | 63.14047869 | 59.55653141 | 64.63744013 | 58.21549424 | 63.84371259 | 55.39027426 | 66.6985467 | 50.49551226 | 61.22196945 | 54.55653225 | 54.03234435 | 57.60323966 | 56.28454372 | 54.41967011 | 54.7456251 |
| ENSMUSG00000003348 | Mob3a | 2640 | 2.09305791 | 3.470093148 | 3.041668954 | 3.986591379 | 3.755748589 | 2.94688605 | 3.172100064 | 3.124832924 | 3.010983917 | 3.37556171 | 3.836473938 | 2.959921501 | 3.214049156 | 3.465714097 | 2.856016942 | 3.800052704 | 3.977805057 | 4.271540484 |
| ENSMUSG00000003352 | Cacnb3 | 3106 | 14.02698179 | 14.22179579 | 14.07909576 | 16.61965977 | 13.91579763 | 16.0176898 | 12.96300379 | 11.81677357 | 10.75410956 | 10.82371263 | 9.136614725 | 14.41215756 | 5.146020991 | 3.738255585 | 6.446862899 | 5.63387237 | 7.058158992 | 6.393524638 |
| ENSMUSG00000003354 | Ccdc65 | 1904 | 0.558103677 | 0.367421627 | 0.431906521 | 0.789660958 | 0.075838115 | 0.130127977 | 0.422434633 | 0.180531314 | 0.670578776 | 0.941032943 | 0.351286459 | 0.322464437 | 0.64774051 | 0.073178709 | 0.735742069 | 0.221216744 | 0.644139444 | 0.433370063 |

| ENSMUSG00000003355 | Fkbp11 | 725 | 56.10683236 | 50.81939449 | 63.25256107 | 51.09121248 | 56.96164208 | 56.31924478 | 94.10328692 | 71.74903735 | 75.10148116 | 68.80750903 | 80.19603375 | 70.90513827 | 67.49967164 | 53.23453327 | 57.28437012 | 54.61032835 | 54.87268439 | 57.7405934 |
| --- | --- | --- | --- | --- | --- | --- | --- | --- | --- | --- | --- | --- | --- | --- | --- | --- | --- | --- | --- | --- |
| ENSMUSG00000003360 | Ddx23 | 5089 | 1.344730466 | 1.341942094 | 1.216705028 | 1.727899697 | 1.702445718 | 2.083765963 | 1.338774959 | 1.388405275 | 1.934285177 | 1.185948127 | 1.398795208 | 1.17356571 | 1.521931816 | 1.797894521 | 1.659720762 | 1.444644649 | 1.061899757 | 1.156607274 |
| ENSMUSG00000003363 | Pld3 | 3199 | 64.44204556 | 66.41751776 | 64.09984219 | 65.65689728 | 65.97639427 | 67.36631395 | 68.18113469 | 67.39484576 | 74.31340074 | 72.07469176 | 76.62810627 | 73.42054022 | 84.67694835 | 78.44245207 | 73.09122528 | 74.61820773 | 80.39057587 | 85.18759447 |
| ENSMUSG00000003378 | Grik5 | 3816 | 2.483924071 | 2.610208431 | 2.307123303 | 1.946134755 | 2.081175945 | 1.42840689 | 2.516895656 | 3.272776621 | 2.892554042 | 2.532991134 | 1.940544697 | 2.779081733 | 2.288194276 | 2.04470825 | 1.705934442 | 1.163970246 | 1.416144618 | 0.605446143 |
| ENSMUSG00000003379 | Cd79a | 1452 | 2.107694679 | 1.078309937 | 0.799562485 | 2.039578431 | 0.828717694 | 1.228580171 | 26.71928149 | 6.654736783 | 14.77835724 | 10.09908314 | 28.8558117 | 6.227367313 | 26.56856702 | 19.73553851 | 9.534244338 | 13.81837347 | 6.519693757 | 7.804322304 |
| ENSMUSG00000003380 | Rabac1 | 1112 | 372.9523404 | 340.0791144 | 340.0937593 | 327.5724366 | 331.9457937 | 350.0330236 | 397.3073704 | 371.9981209 | 364.9377157 | 404.5123283 | 379.8796609 | 376.2035891 | 447.0039555 | 430.7354975 | 363.1813407 | 444.1985931 | 350.6925099 | 417.2678194 |
| ENSMUSG00000003382 | Etv3 | 6118 | 4.071270538 | 3.582851323 | 3.731988612 | 4.75866416 | 4.618084209 | 3.588077963 | 4.106412637 | 5.481028001 | 5.257709841 | 5.040300741 | 5.083605422 | 6.705542988 | 4.144590631 | 5.139366977 | 4.343740364 | 6.771892319 | 6.17054633 | 6.060172921 |
| ENSMUSG00000003402 | Prkcsh | 1995 | 76.59454026 | 78.11414494 | 67.81993203 | 81.71299079 | 73.5368939 | 76.52730434 | 72.85441086 | 62.65851282 | 75.25055135 | 67.83083627 | 72.27309508 | 71.51119477 | 90.28111778 | 76.00999766 | 73.4606187 | 69.13421948 | 71.56163206 | 88.51089352 |
| ENSMUSG00000003410 | Elavl3 | 5020 | 0 | 0.066360347 | 0 | 0 | 0 | 0.03948425 | 0 | 0.030432193 | 0.057431396 | 0 | 0 | 0 | 0 | 0 | 0 | 0.068648499 | 0 | 0 |
| ENSMUSG00000003411 | Rab3b | 3536 | 1.202069458 | 1.931318811 | 1.846840101 | 1.636383663 | 1.361196936 | 1.219199044 | 1.806338138 | 1.080101878 | 0.79204838 | 0.68005822 | 0.959282255 | 1.168087961 | 0.362734686 | 0.958828727 | 0.034956071 | 0.173260667 | 0.010838885 | 0.155568741 |
| ENSMUSG00000003418 | St8sia6 | 9750 | 0.156942188 | 0.228919376 | 0.019845551 | 0.233646381 | 0.059239291 | 0.274445909 | 0 | 0 | 0 | 0.024179848 | 0 | 0 | 0 | 0 | 0 | 0 | 0 | 0 |
| ENSMUSG00000003420 | Fcgrt | 1731 | 104.9983436 | 97.22515401 | 95.46157117 | 98.30762599 | 102.2421048 | 94.98322935 | 110.4782485 | 124.307334 | 117.7539379 | 133.8251907 | 135.5417799 | 120.047205 | 129.6138416 | 126.5339781 | 120.3675726 | 150.5079681 | 132.2046902 | 128.4816976 |
| ENSMUSG00000003421 | Nosip | 2987 | 27.36439689 | 24.03390929 | 20.72919948 | 26.9675013 | 25.00861919 | 25.86297678 | 21.57344114 | 23.68005666 | 22.19962852 | 24.34093888 | 23.79951062 | 25.82432062 | 25.18619814 | 24.27158367 | 21.86289457 | 27.71486214 | 22.46713956 | 22.90971837 |
| ENSMUSG00000003423 | Pih1d1 | 1997 | 19.13477879 | 18.01598686 | 20.44429625 | 19.05031148 | 20.72781891 | 19.5282931 | 20.11440381 | 21.24775116 | 24.15093138 | 19.07750037 | 22.67934934 | 20.85051792 | 23.44315949 | 21.76848563 | 17.92896949 | 21.47495257 | 14.35556839 | 18.37309674 |
| ENSMUSG00000003429 | Rps11 | 748 | 214.4011085 | 199.0757181 | 209.0150403 | 175.5440238 | 203.9815482 | 199.6683677 | 216.3859286 | 190.7067152 | 212.9805387 | 179.7147794 | 201.702298 | 190.1314282 | 279.9652257 | 272.9521499 | 248.1457343 | 207.5269016 | 192.0413914 | 229.5232916 |
| ENSMUSG00000003435 | Supt5h | 3479 | 60.6239389 | 65.26607847 | 61.554935 | 64.2621571 | 65.13512407 | 65.22045618 | 45.62634027 | 47.76520347 | 43.94491133 | 50.28143406 | 47.82988801 | 43.22144151 | 42.01508218 | 46.95138124 | 45.38211043 | 50.13329023 | 46.11494074 | 37.93240788 |
| ENSMUSG00000003436 | Dll3 | 2750 | 0.030912855 | 0.060568899 | 0 | 0.082838262 | 0 | 0 | 0.103227663 | 0.027776293 | 0.014976915 | 0 | 0 | 0 | 0 | 0.016888759 | 0 | 0 | 0.097557847 | 0 |
| ENSMUSG00000003437 | Paf1 | 3665 | 26.4425106 | 25.7777266 | 26.27877188 | 28.76623645 | 25.42521261 | 28.1903 | 21.86839785 | 26.21884978 | 26.22901167 | 25.88464255 | 27.97409574 | 25.41782834 | 26.38214403 | 27.28352528 | 23.32693819 | 27.3832693 | 25.38006073 | 27.93231858 |
| ENSMUSG00000003438 | Timm50 | 3021 | 27.38003064 | 29.16670149 | 25.42772797 | 24.22080013 | 26.43193028 | 27.47462057 | 23.46057366 | 29.87376593 | 27.21227589 | 25.39364257 | 27.13731504 | 26.25423917 | 27.98904542 | 29.14862287 | 24.41270607 | 27.08607349 | 25.44937159 | 28.22380517 |
| ENSMUSG00000003444 | Med29 | 1277 | 14.34591297 | 9.860825384 | 13.56125596 | 12.5587217 | 11.83510104 | 12.06805023 | 10.81856088 | 12.74077011 | 12.80426528 | 12.22152136 | 13.16897837 | 11.97610928 | 13.90719673 | 13.49315303 | 10.71175924 | 13.04339406 | 10.47445389 | 10.51074553 |
| ENSMUSG00000003452 | Bicd1 | 14252 | 2.523111066 | 2.433253079 | 2.498099799 | 2.247362768 | 2.874001336 | 2.53118089 | 2.217574342 | 2.795023555 | 2.173186911 | 2.448184143 | 2.215776561 | 2.725771903 | 2.405675169 | 3.252259277 | 1.968724359 | 2.823703928 | 2.374552355 | 2.227073715 |
| ENSMUSG00000003458 | Ncstn | 4055 | 70.08375017 | 83.09739211 | 81.17925378 | 87.03226866 | 79.72925556 | 76.62037966 | 62.72576126 | 83.25096051 | 71.57617178 | 82.25501229 | 76.36924312 | 71.41101295 | 80.11114063 | 83.58790376 | 83.37853703 | 83.01177057 | 88.76956363 | 84.24329291 |
| ENSMUSG00000003464 | Pex19 | 3899 | 24.0052315 | 30.55319562 | 29.34172847 | 29.98451905 | 24.56592154 | 22.81281271 | 29.54767202 | 29.29814711 | 31.97527141 | 31.41767814 | 27.41025445 | 22.26041429 | 24.87472413 | 30.75625382 | 29.52478768 | 32.92854748 | 29.27307314 | 24.23843174 |
| ENSMUSG00000003469 | Phyhip | 2904 | 0.014636769 | 0.034414147 | 0 | 0.109823454 | 0.049723062 | 0.102381681 | 0.03258449 | 0 | 0 | 0.178601149 | 0.016451432 | 0 | 0.135900599 | 0.015993143 | 0.141878636 | 0.026370942 | 0.039593282 | 0.01894253 |
| ENSMUSG00000003476 | Crhr2 | 4057 | 0 | 0 | 0 | 0 | 0 | 0 | 0 | 0 | 0 | 0 | 0 | 0 | 0 | 0 | 0.081245365 | 0 | 0 | 0 |
| ENSMUSG00000003477 | Inmt | 1597 | 0.958163016 | 0.041719342 | 0.393773259 | 1.512044677 | 0.150694814 | 0.713658655 | 1.777558382 | 0.6217924 | 0.025789928 | 1.092408278 | 0.029915441 | 0.559205135 | 16.52634674 | 26.84276309 | 17.38875934 | 5.107012759 | 3.911826171 | 19.18601906 |
| ENSMUSG00000003484 | Cyp4f18 | 2096 | 0.243350244 | 0.111254895 | 0.230789744 | 0.586902766 | 0.022963704 | 0.283698856 | 4.085684583 | 1.731048773 | 1.611304529 | 0.74235363 | 4.034431131 | 0.95866596 | 1.482781865 | 2.570378882 | 0.353830156 | 0.767273622 | 0.127998129 | 0.262448028 |
| ENSMUSG00000003500 | Impdh1 | 4059 | 8.471714069 | 9.003263109 | 8.533000181 | 9.035880929 | 7.755180606 | 9.497912472 | 8.171024373 | 7.725046163 | 6.362144711 | 8.584471473 | 7.638814499 | 9.350739236 | 9.358358291 | 14.14261937 | 9.501023975 | 8.858063649 | 10.26378042 | 12.56305542 |
| ENSMUSG00000003505 | Psg18 | 2531 | 0 | 0 | 0 | 0 | 0 | 0 | 0 | 0 | 0 | 0 | 0 | 0 | 0 | 0 | 0 | 0 | 0 | 0 |
| ENSMUSG00000003518 | Dusp3 | 5071 | 3.746758762 | 3.599973582 | 2.451586926 | 2.695388148 | 2.572224672 | 2.628610793 | 2.052610799 | 2.259459815 | 3.208178609 | 1.91541015 | 2.562569258 | 1.893175698 | 1.605159998 | 1.666894874 | 2.104358702 | 1.970784566 | 2.312728624 | 1.57292851 |
| ENSMUSG00000003526 | Prodh | 3444 | 23.09151694 | 20.12895844 | 20.4505982 | 22.74083485 | 19.10462824 | 23.48143473 | 19.16410775 | 18.30884331 | 12.81996077 | 17.60621498 | 23.06903483 | 18.23246818 | 14.59617413 | 16.80294219 | 17.31087438 | 15.3762805 | 15.35722698 | 21.24335422 |
| ENSMUSG00000003527 | Dgcr14 | 2809 | 19.76210747 | 22.41415809 | 18.15083695 | 22.28582233 | 21.24727139 | 21.18648381 | 20.04346313 | 20.28588978 | 15.27815928 | 18.22914337 | 21.48087311 | 17.6448232 | 22.42679187 | 20.73367233 | 20.05073937 | 19.76553248 | 18.84251193 | 23.26479843 |
| ENSMUSG00000003528 | Slc25a1 | 2363 | 58.60425878 | 59.21039176 | 56.45966852 | 65.53617846 | 59.53855814 | 61.67354611 | 69.03686709 | 64.78000374 | 67.45315954 | 69.15968578 | 73.51237792 | 62.66565926 | 87.47386093 | 85.55696674 | 78.00920908 | 92.88267525 | 83.7242254 | 78.1255024 |
| ENSMUSG00000003531 | Dgcr6 | 3046 | 30.95091281 | 29.84599091 | 30.71385677 | 29.81062422 | 31.7771827 | 30.92572112 | 35.92719185 | 31.59712871 | 31.88371772 | 34.27171931 | 32.10615233 | 32.56219055 | 35.50084132 | 30.49513289 | 31.13787973 | 28.77452408 | 27.17820135 | 31.85688255 |
| ENSMUSG00000003534 | Ddr1 | 5540 | 41.40802076 | 51.89956498 | 46.39143805 | 53.93309183 | 50.4255749 | 51.77097869 | 33.72522908 | 46.38929166 | 38.48783251 | 44.23995587 | 38.73738511 | 40.76356874 | 34.6926263 | 34.04502507 | 36.08482837 | 39.42411997 | 39.25332286 | 33.35299449 |
| ENSMUSG00000003541 | Ier3 | 1129 | 130.3768154 | 91.32277025 | 91.81973034 | 102.2197744 | 128.2807425 | 115.3889607 | 127.312594 | 125.6053057 | 105.6840887 | 135.1040966 | 121.1935173 | 169.3256372 | 162.4151785 | 206.7564067 | 200.7161713 | 211.7684257 | 195.6032974 | 249.8553581 |
| ENSMUSG00000003545 | Fosb | 3747 | 5.342924449 | 2.613821964 | 3.266213819 | 3.051994163 | 3.866482259 | 3.068116944 | 6.97000234 | 9.754504698 | 2.561104396 | 6.027538527 | 8.784880275 | 3.113283555 | 15.24589277 | 26.11627759 | 13.92078189 | 33.72271268 | 31.73912444 | 37.67103492 |
| ENSMUSG00000003546 | Klc4 | 2438 | 13.65117015 | 13.82799383 | 10.97233892 | 13.67952603 | 11.19392975 | 12.03249314 | 12.0319364 | 10.90316328 | 10.93013769 | 16.26486523 | 11.18929503 | 10.7830915 | 9.206735997 | 13.79225565 | 15.26046143 | 15.59580523 | 13.62957318 | 15.34298301 |
| ENSMUSG00000003549 | Ercc1 | 2664 | 16.91272019 | 17.38172784 | 14.3631616 | 15.08439981 | 13.73132957 | 16.66638487 | 13.2489709 | 14.83826445 | 13.07950163 | 11.75227739 | 12.78661614 | 15.67196532 | 10.64784251 | 12.43041828 | 13.17707418 | 14.45959123 | 11.46623823 | 11.41893244 |
| ENSMUSG00000003555 | Cyp17a1 | 2158 | 1.496938544 | 1.096022006 | 1.27770678 | 0.717829243 | 1.003677741 | 1.125154748 | 4.472560937 | 4.760776759 | 4.943145312 | 1.616845246 | 1.261896494 | 3.388253846 | 2.148841624 | 1.506527394 | 0.992808576 | 0.443589057 | 0.959045428 | 1.096103609 |
| ENSMUSG00000003559 | As3mt | 1740 | 15.14552249 | 15.73747072 | 14.7900679 | 14.27055699 | 16.81851126 | 16.20431345 | 11.36591942 | 17.25243005 | 16.64029918 | 15.93368593 | 13.31658325 | 18.70145476 | 9.044184834 | 14.73398634 | 9.850486926 | 11.66323099 | 8.34808421 | 11.6024955 |

| ENSMUSG00000003573 | Homer3 | 3396 | 4.005199155 | 4.904725297 | 4.415722736 | 4.494390416 | 3.996820517 | 4.596322461 | 4.095973971 | 3.621305528 | 3.771792227 | 2.998984659 | 4.150062648 | 3.747341304 | 3.282983894 | 3.706227233 | 3.19081543 | 3.608066657 | 3.577572473 | 2.996668064 |
| --- | --- | --- | --- | --- | --- | --- | --- | --- | --- | --- | --- | --- | --- | --- | --- | --- | --- | --- | --- | --- |
| ENSMUSG00000003575 | Crtc1 | 11168 | 4.076203754 | 4.250615532 | 3.690387524 | 4.299905151 | 4.322736339 | 4.392658152 | 3.855169934 | 5.033955618 | 3.378120383 | 4.673695246 | 3.89283777 | 4.318117163 | 3.335028433 | 3.222973471 | 4.246327976 | 4.11774888 | 3.994610458 | 2.999690723 |
| ENSMUSG00000003581 | Rnf215 | 2572 | 5.321410063 | 5.426945054 | 6.488673395 | 6.625128531 | 5.09015678 | 6.704646689 | 6.861442162 | 7.291006836 | 5.732804293 | 6.416308764 | 8.154434981 | 7.356730022 | 6.732310639 | 4.893603298 | 5.574705074 | 5.344606554 | 7.361271645 | 7.14348431 |
| ENSMUSG00000003585 | Sec14l2 | 4153 | 1.146298992 | 1.427810058 | 1.45598153 | 1.37132929 | 1.066250175 | 1.384087911 | 0.535443272 | 0.64374384 | 0.426443578 | 0.726618109 | 0.253081889 | 0.349435884 | 0.427630393 | 1.476190594 | 0.783752207 | 0.756038961 | 0.599857765 | 1.496756335 |
| ENSMUSG00000003604 | Aven | 5112 | 2.153529067 | 2.385078107 | 1.920936358 | 1.960764819 | 2.297376638 | 1.938682847 | 2.970925261 | 2.517769877 | 2.304253425 | 2.121412705 | 2.084079761 | 1.998095902 | 1.534384751 | 1.517246204 | 2.377632822 | 2.54671489 | 2.436628813 | 2.464218591 |
| ENSMUSG00000003617 | Cp | 9266 | 10.54143045 | 15.62103663 | 10.75431389 | 10.36998084 | 14.88214562 | 13.31602705 | 4.886491877 | 5.366556004 | 5.698371812 | 4.208248607 | 4.660971559 | 6.445365742 | 6.873246838 | 8.069823084 | 4.695536696 | 3.925758367 | 5.017245645 | 7.165550595 |
| ENSMUSG00000003623 | Crot | 5437 | 27.23708539 | 29.97976668 | 32.59007572 | 32.12820379 | 32.4184485 | 33.81288239 | 36.91380982 | 32.46740554 | 31.1266127 | 35.20909605 | 37.23933677 | 30.64369189 | 35.9124018 | 33.69908493 | 35.24525224 | 39.57940291 | 40.78645464 | 39.55961142 |
| ENSMUSG00000003644 | Rps6ka1 | 4802 | 67.44886325 | 58.1900368 | 70.00159568 | 67.56355617 | 69.41140596 | 77.44549469 | 71.78679273 | 78.9696717 | 84.79173153 | 90.41309363 | 99.55935225 | 89.18650125 | 63.37538477 | 51.71523017 | 76.22545242 | 70.13033988 | 72.75770982 | 79.85599395 |
| ENSMUSG00000003657 | Calb2 | 1730 | 0 | 0 | 0 | 0 | 0 | 0 | 0 | 0 | 0 | 0 | 0 | 0 | 0 | 0 | 0 | 0 | 0 | 0 |
| ENSMUSG00000003660 | Snrnp200 | 7214 | 9.338883283 | 12.6158618 | 11.48653417 | 11.95551108 | 12.81027078 | 13.38074924 | 10.71651197 | 8.645438471 | 11.89236378 | 8.88895986 | 12.60268173 | 9.214922947 | 7.624769582 | 8.710680654 | 9.989121327 | 8.232427513 | 10.57771793 | 10.82033857 |
| ENSMUSG00000003662 | Ciao1 | 3485 | 13.84314915 | 14.95973585 | 14.46347737 | 14.30237662 | 15.17847464 | 13.62166966 | 14.43138528 | 12.65774025 | 16.49824276 | 15.08551912 | 16.50532281 | 15.471429 | 15.62767193 | 12.98035613 | 14.4235002 | 17.16210294 | 14.52770106 | 15.76875111 |
| ENSMUSG00000003665 | Has1 | 2085 | 0.305792633 | 0.319548146 | 0.046401468 | 0.349629117 | 0.207763699 | 0.285195589 | 0.136151594 | 0.18317699 | 0 | 0.067842738 | 0 | 0 | 0.047320784 | 0 | 0 | 0.165283197 | 0.073527668 | 0 |
| ENSMUSG00000003680 | Taf6l | 4101 | 4.5500542 | 4.971346321 | 4.057667506 | 4.266140216 | 3.755721915 | 4.506990886 | 4.26863965 | 3.818308946 | 3.364419112 | 3.288247045 | 2.469712557 | 3.606718016 | 3.05542721 | 3.850521754 | 3.677095698 | 3.585367774 | 3.411143226 | 3.058297082 |
| ENSMUSG00000003721 | Insig2 | 3532 | 54.88847901 | 54.94929834 | 58.13862864 | 56.99000393 | 60.68274521 | 55.80995871 | 41.43208265 | 41.43637769 | 39.05255982 | 41.25019056 | 41.79632551 | 40.07602501 | 38.84259964 | 30.04664204 | 40.24500788 | 46.62737895 | 42.75357016 | 33.23596652 |
| ENSMUSG00000003731 | Kpna6 | 7408 | 5.571345292 | 5.117450543 | 5.367581257 | 6.144098716 | 4.963929493 | 5.498426971 | 4.164128869 | 5.846407172 | 4.353272387 | 5.079138931 | 4.920665945 | 4.980312226 | 3.922313617 | 3.836903548 | 3.442732599 | 4.042016062 | 5.023600741 | 4.024694363 |
| ENSMUSG00000003746 | Man1a | 9592 | 22.0901066 | 26.29398688 | 25.1197409 | 25.15547235 | 24.52259289 | 26.65164226 | 18.04313798 | 17.08942777 | 15.43635573 | 16.06918776 | 17.30298225 | 16.30478384 | 8.593998516 | 9.979281079 | 11.61048849 | 10.32313498 | 11.85110473 | 7.960033375 |
| ENSMUSG00000003752 | Itpkc | 4111 | 6.141589531 | 6.563717907 | 6.471768839 | 8.533691106 | 7.856122788 | 8.268833674 | 5.973067469 | 7.943202154 | 6.291688616 | 8.246498575 | 7.00761371 | 6.354106079 | 4.727992072 | 8.394054228 | 5.692649902 | 7.293005496 | 11.35524924 | 7.26585866 |
| ENSMUSG00000003762 | Adck4 | 3157 | 10.51521776 | 9.338584537 | 9.637931769 | 10.26097641 | 9.284872193 | 11.85059039 | 9.666353471 | 11.71056241 | 11.35010095 | 9.618325276 | 11.80375914 | 9.211263924 | 11.26649109 | 9.076972417 | 11.6544154 | 13.64489494 | 11.42383446 | 13.71307158 |
| ENSMUSG00000003778 | Brd8 | 8817 | 11.79654823 | 13.44681757 | 13.37034065 | 12.50512956 | 12.741285 | 11.07728682 | 12.46539108 | 14.60641725 | 14.18662225 | 12.39597711 | 15.02009583 | 14.99052841 | 13.00299147 | 11.44641056 | 13.19646068 | 14.85677304 | 14.67935858 | 14.81134392 |
| ENSMUSG00000003779 | Kif20a | 3873 | 0.504838135 | 0.980549946 | 0.462127711 | 0.223511449 | 0.38525423 | 0.652514695 | 0.574153346 | 0.857923834 | 0.478542011 | 0.523490896 | 0.456409363 | 0.389109964 | 0.522233051 | 0.49166217 | 1.361683231 | 0.622852638 | 1.266657889 | 0.781177606 |
| ENSMUSG00000003808 | Farsa | 4608 | 9.528612595 | 9.788554421 | 9.783882442 | 10.371861 | 9.108298055 | 8.688934186 | 10.88355895 | 10.31891081 | 8.79503722 | 7.797056398 | 10.68923225 | 9.714444747 | 9.774314339 | 11.2380984 | 9.07542952 | 11.74976538 | 10.3051827 | 10.66040198 |
| ENSMUSG00000003809 | Gcdh | 2496 | 25.40774144 | 24.74443345 | 22.3456252 | 25.02571786 | 25.41581546 | 23.72416531 | 30.78356994 | 25.53810882 | 28.18372152 | 23.87759973 | 25.95466494 | 22.29495817 | 30.35810294 | 31.07436937 | 29.21744949 | 31.69406847 | 28.63723707 | 30.17126086 |
| ENSMUSG00000003810 | Mast2 | 8848 | 4.01128187 | 4.762749908 | 4.83844647 | 4.320266297 | 5.195070875 | 5.320422344 | 2.882180599 | 3.056082833 | 3.398073746 | 3.37856531 | 3.185717168 | 2.90180886 | 3.24493398 | 2.372595784 | 4.004672024 | 3.08990661 | 3.335358117 | 3.4194178 |
| ENSMUSG00000003812 | Dnase2a | 3756 | 9.539899731 | 7.840414933 | 7.096329932 | 7.532856372 | 6.458596786 | 6.33261771 | 5.895190026 | 6.324726916 | 6.34903957 | 6.465019217 | 5.647519053 | 6.285949221 | 9.903159112 | 8.680444448 | 7.48121968 | 6.3817679 | 6.326492034 | 6.282989027 |
| ENSMUSG00000003813 | Rad23a | 3937 | 24.38892371 | 27.51677216 | 27.27692085 | 28.93132102 | 26.57830888 | 26.36854886 | 26.55855231 | 30.11156136 | 22.89999568 | 27.15027795 | 25.33759545 | 24.48403589 | 22.42929437 | 22.63810102 | 24.02813623 | 21.63998005 | 22.10795536 | 22.97052869 |
| ENSMUSG00000003814 | Calr | 2936 | 171.888268 | 193.2170557 | 188.9632219 | 196.0087027 | 185.2161014 | 194.7341417 | 194.2301496 | 201.2515864 | 183.4314987 | 196.1668393 | 177.6753309 | 196.59052 | 221.2879259 | 240.6518738 | 200.3944885 | 181.9847192 | 184.6606935 | 194.9113546 |
| ENSMUSG00000003824 | Syce2 | 2023 | 4.496345858 | 4.528445828 | 4.232385009 | 4.842127796 | 4.758469962 | 4.947924954 | 8.676719665 | 7.041901185 | 5.598760175 | 3.845707384 | 5.974821797 | 5.076659375 | 6.291465476 | 7.851645026 | 8.248457803 | 7.571054355 | 6.384558604 | 6.825153627 |
| ENSMUSG00000003847 | Nfat5 | 18323 | 4.038722451 | 4.265243128 | 4.319112354 | 4.311676629 | 3.837839899 | 3.677984919 | 4.438710641 | 5.519482703 | 4.862000414 | 4.474980787 | 4.993125879 | 4.886117054 | 3.860828979 | 4.035310109 | 4.735604252 | 4.405206593 | 5.386138434 | 5.22080645 |
| ENSMUSG00000003848 | Nob1 | 2988 | 9.289116448 | 8.551201428 | 8.175579929 | 8.188179655 | 6.765531439 | 7.545680649 | 6.618708096 | 8.678929462 | 8.008489176 | 9.57847285 | 9.881166111 | 7.826895122 | 8.651246537 | 11.44004294 | 9.569571549 | 9.034430461 | 9.183945309 | 9.610024668 |
| ENSMUSG00000003849 | Nqo1 | 1547 | 6.649161344 | 6.309423404 | 7.442081593 | 5.890244904 | 6.782649877 | 6.726615418 | 21.56136952 | 26.53964614 | 19.00916112 | 18.89685586 | 13.77351744 | 14.90101399 | 24.07601651 | 18.34346309 | 22.50505154 | 14.6281506 | 11.49541161 | 16.89032042 |
| ENSMUSG00000003863 | Ppfia3 | 6091 | 0.418701455 | 0.508635554 | 0.428857435 | 0.718085742 | 0.561051811 | 0.561342738 | 0.372846591 | 0.194379326 | 0.784376265 | 0.232231341 | 0.651013225 | 0.256581604 | 0.299668517 | 0.228751046 | 0.142051005 | 0.364612581 | 0.201353062 | 0.25287391 |
| ENSMUSG00000003865 | Gys1 | 5066 | 17.83774264 | 20.50327757 | 19.36469 | 21.39552398 | 17.72881357 | 21.90062584 | 17.38969762 | 20.37028648 | 23.723303 | 19.39638089 | 22.17112661 | 19.9089819 | 18.1610789 | 16.89625998 | 17.19306932 | 15.88009596 | 18.98914434 | 20.07734669 |
| ENSMUSG00000003868 | Ruvbl2 | 2662 | 16.94139435 | 15.96816417 | 19.98906213 | 17.13245882 | 15.40510855 | 16.3624541 | 18.18214521 | 14.57681474 | 16.84902918 | 17.65937308 | 15.59595752 | 16.2498642 | 17.08641162 | 13.12019298 | 15.72531209 | 15.16089414 | 12.71304289 | 17.64754964 |
| ENSMUSG00000003872 | Lin7b | 1563 | 0.625476039 | 0.554149232 | 0.309491557 | 0.932791695 | 0.739069845 | 0.855997317 | 0.817301555 | 0.342094455 | 0.869580947 | 0.422335154 | 0.183397153 | 0.999896707 | 0.063124654 | 1.099444167 | 0.158163362 | 0.097992597 | 0.269730816 | 0.17597283 |
| ENSMUSG00000003873 | Bax | 1438 | 99.08021561 | 81.42894519 | 84.905974 | 79.14568063 | 76.51570063 | 85.52843167 | 92.32223718 | 79.67816913 | 82.28710694 | 81.48087454 | 81.23071869 | 84.53860439 | 85.21591274 | 92.88817478 | 79.39465325 | 84.11684894 | 73.21442114 | 81.09826582 |
| ENSMUSG00000003882 | Il7r | 3200 | 0 | 0 | 0 | 0 | 0 | 0 | 0 | 0.011935126 | 0 | 0 | 0 | 0 | 0 | 0 | 0 | 0 | 0 | 0 |
| ENSMUSG00000003923 | Tfam | 4682 | 9.087501325 | 8.730226657 | 9.2779646 | 7.522145936 | 8.337246923 | 7.72607764 | 7.629447371 | 10.11503182 | 10.84642758 | 8.167283285 | 9.061119848 | 9.739725829 | 8.671543767 | 7.90600975 | 8.632790758 | 8.104654407 | 10.11774946 | 9.035028422 |
| ENSMUSG00000003929 | Zfp81 | 7454 | 2.394979062 | 2.377577103 | 2.641270037 | 1.601421199 | 2.247103493 | 2.78543002 | 2.748375366 | 2.177592034 | 2.060983416 | 1.613017082 | 1.410047071 | 1.782147531 | 1.594981495 | 1.862997334 | 1.65823273 | 1.839017635 | 1.953849319 | 0.870817627 |
| ENSMUSG00000003934 | Efnb3 | 3178 | 0.080248916 | 0.146752838 | 0.197877877 | 0.200709446 | 0 | 0.202701553 | 0.029775128 | 0 | 0.194398281 | 0.16320256 | 0.075165133 | 0 | 0 | 0 | 0.051858472 | 0.265070284 | 0.096479036 | 0 |
| ENSMUSG00000003948 | Mmd | 5907 | 0.582854115 | 0.485002354 | 0.425837748 | 0.632470905 | 0.562231714 | 0.335552623 | 1.233477663 | 1.325451582 | 1.366608615 | 0.646556114 | 0.784521918 | 1.266175518 | 2.53048433 | 1.281595775 | 1.206681762 | 0.538026141 | 0.843476993 | 0.996440565 |
| ENSMUSG00000003949 | Hlf | 11048 | 0.015389274 | 0.027137586 | 0.179517989 | 0.156708878 | 0.03485295 | 0 | 0.393986827 | 0.335324315 | 0.357884279 | 0.187783395 | 0.159999408 | 0.308178441 | 0.955560309 | 1.135038341 | 0.499729226 | 0.145565307 | 0.270587541 | 0.468035484 |

| ENSMUSG00000003955 | Fam162a | 630 | 210.5692927 | 162.0169966 | 173.6072256 | 162.7180155 | 169.1493317 | 149.995338 | 243.8480452 | 234.7317176 | 245.2232073 | 246.6805047 | 237.9647932 | 225.3889613 | 255.1947911 | 210.693971 | 235.3066636 | 233.2688114 | 213.2280486 | 198.0327842 |
| --- | --- | --- | --- | --- | --- | --- | --- | --- | --- | --- | --- | --- | --- | --- | --- | --- | --- | --- | --- | --- |
| ENSMUSG00000003970 | Rpl8 | 856 | 169.325526 | 149.0518515 | 149.2459038 | 145.9975988 | 148.9503105 | 136.9646818 | 165.981279 | 147.7278556 | 157.0476489 | 137.320915 | 147.2317062 | 154.992792 | 189.7204108 | 202.1619972 | 156.1904776 | 140.1901444 | 128.4557778 | 174.1526626 |
| ENSMUSG00000003974 | Grm3 | 3571 | 0 | 0 | 0.081277284 | 0 | 0 | 0 | 0 | 0 | 0 | 0 | 0 | 0 | 0 | 0 | 0 | 0 | 0 | 0 |
| ENSMUSG00000003992 | Ssbp2 | 5785 | 1.388673858 | 1.359004846 | 1.747634891 | 2.008308782 | 1.256338889 | 1.54183095 | 3.696686409 | 1.980591309 | 3.566887536 | 2.298443962 | 2.964772704 | 3.280436522 | 2.114834137 | 2.296112186 | 1.588236294 | 1.621641973 | 2.895175756 | 1.530936246 |
| ENSMUSG00000004018 | Fancl | 3142 | 5.857651566 | 5.863154202 | 5.234564074 | 4.422698448 | 5.805855847 | 4.825950501 | 5.631744723 | 6.819203612 | 4.941857556 | 4.8621349 | 5.230612895 | 4.778616561 | 4.349121924 | 5.158811744 | 5.717338755 | 4.301904651 | 3.866784252 | 4.622025517 |
| ENSMUSG00000004031 | Fam5b | 4091 | 0.405207007 | 0.724724416 | 0.756760191 | 1.124826565 | 0.529439395 | 0.835770867 | 0.011565065 | 0.364092812 | 0.312095328 | 0.311187726 | 0.607259311 | 0.191009356 | 0.361759354 | 0.102174722 | 0.59420479 | 0 | 0.009368442 | 0.067231859 |
| ENSMUSG00000004032 | Gstm5 | 1432 | 1068.684607 | 943.2062124 | 1085.060174 | 893.8809641 | 1014.938245 | 1041.195671 | 1063.973603 | 923.0452897 | 946.4845396 | 968.2357402 | 817.3121039 | 958.8055056 | 1469.794868 | 1197.102839 | 1163.454829 | 1290.890819 | 1066.017043 | 1241.469491 |
| ENSMUSG00000004035 | Gstm7 | 1862 | 821.2036533 | 735.8893475 | 936.7370483 | 809.2835555 | 711.1220301 | 810.6742053 | 861.4617367 | 639.979134 | 745.4493919 | 694.4473073 | 525.7041484 | 668.5589273 | 594.8178704 | 604.5214296 | 525.0211153 | 278.1724202 | 342.4254969 | 497.2386011 |
| ENSMUSG00000004038 | Gstm3 | 1384 | 0.307118324 | 0.216630092 | 0.139807891 | 0.197518978 | 0.069554803 | 0.143215993 | 0.786265618 | 0.55191333 | 1.577229289 | 0.374752699 | 0.310675308 | 1.048560135 | 0.356444489 | 0.335578666 | 0.267929193 | 0.110666495 | 0.083077233 | 0.397464644 |
| ENSMUSG00000004040 | Stat3 | 9647 | 29.59985203 | 33.03318608 | 29.56968057 | 33.36672315 | 33.49327289 | 35.1805403 | 25.68430595 | 30.65843933 | 25.78266494 | 27.1457454 | 30.84300212 | 28.66866467 | 26.38160684 | 27.60551437 | 26.71886946 | 27.96282048 | 31.34596067 | 28.13464627 |
| ENSMUSG00000004043 | Stat5a | 5012 | 19.16634035 | 19.24863162 | 20.09451121 | 21.02607653 | 18.3039598 | 20.17899626 | 12.33792326 | 13.04576874 | 12.17845498 | 12.06990269 | 14.07893329 | 12.50618298 | 12.48062073 | 15.39178555 | 14.5750915 | 12.4375716 | 11.69976741 | 13.48886516 |
| ENSMUSG00000004044 | Ptrf | 4805 | 4.3080168 | 3.244627366 | 3.855788164 | 3.75487483 | 3.505967385 | 4.052908282 | 6.754734182 | 5.110866753 | 8.331590702 | 6.476475369 | 6.144625253 | 6.075253181 | 7.5666229 | 8.91185194 | 9.012036471 | 8.494856918 | 9.778988554 | 8.597677242 |
| ENSMUSG00000004054 | Map3k11 | 3789 | 15.35750488 | 18.55112346 | 18.52467473 | 19.25131485 | 16.83156476 | 19.87863685 | 13.81045735 | 19.6657105 | 14.53322026 | 21.09275324 | 18.79980551 | 18.07490578 | 17.02986218 | 18.2393249 | 20.17125526 | 18.08925501 | 18.26795783 | 16.49256934 |
| ENSMUSG00000004056 | Akt2 | 5865 | 19.21248451 | 16.86376521 | 18.25245058 | 18.12342991 | 19.16249646 | 20.41251566 | 22.37772742 | 20.83813942 | 20.01390792 | 25.10684506 | 25.22745889 | 19.78529625 | 20.10286143 | 20.47816027 | 21.50348893 | 16.7133768 | 23.55776647 | 22.6789463 |
| ENSMUSG00000004069 | Dnaja3 | 3897 | 39.40754452 | 38.17690161 | 38.5672463 | 42.14718112 | 35.94146725 | 37.56191301 | 39.70040033 | 34.36042156 | 35.69075282 | 35.60803687 | 44.86947602 | 42.66740027 | 35.22985008 | 32.63123204 | 34.74167633 | 35.8440174 | 40.36210482 | 35.93871843 |
| ENSMUSG00000004070 | Hmox2 | 2274 | 30.50503398 | 25.51937631 | 28.27107381 | 26.32683037 | 25.35709963 | 26.84651177 | 28.77459761 | 28.06486562 | 27.42145333 | 28.65535264 | 26.68170504 | 25.03604918 | 31.75986231 | 24.75384077 | 25.81891695 | 29.80403478 | 28.80371214 | 32.2942645 |
| ENSMUSG00000004071 | 5730403B10R | 5739 | 23.41154581 | 24.02545203 | 22.35347665 | 24.3484445 | 23.13083211 | 22.48393766 | 20.02482961 | 22.21401624 | 23.50336977 | 21.91164412 | 25.82295194 | 19.72367376 | 21.10295468 | 23.16134834 | 23.43298108 | 29.17665554 | 22.92632467 | 20.81891964 |
| ENSMUSG00000004085 | B230120H23R | 10280 | 2.427095168 | 2.210057768 | 2.098695967 | 2.433172504 | 2.266133371 | 2.771675273 | 2.163128317 | 2.359161044 | 2.391862832 | 3.077638316 | 2.732653267 | 2.318413222 | 1.972316926 | 2.692672771 | 2.841624818 | 2.886694624 | 2.736527425 | 2.215347294 |
| ENSMUSG00000004096 | Cwc15 | 1104 | 100.1797718 | 90.5543438 | 96.83469392 | 83.65238847 | 94.12755724 | 89.50013655 | 99.38233916 | 90.25722634 | 93.49040625 | 87.1262995 | 96.24230766 | 98.99190883 | 101.3896017 | 98.44127218 | 99.19722424 | 97.94626386 | 89.08086762 | 109.2708071 |
| ENSMUSG00000004098 | Col5a3 | 6742 | 0.290008617 | 0.014823299 | 0.107624289 | 0.195976014 | 0.014278233 | 0.051448997 | 0.007017603 | 0 | 0 | 0.006993578 | 0 | 0 | 0.05121973 | 0 | 0.006111177 | 0 | 0.034108244 | 0 |
| ENSMUSG00000004099 | Dnmt1 | 5225 | 5.312130133 | 5.999508796 | 6.230695872 | 5.432446052 | 4.47695979 | 4.599631729 | 5.342484319 | 5.138614142 | 4.138358043 | 4.268379453 | 6.025588065 | 4.796408134 | 3.550009741 | 6.151063823 | 4.699737284 | 3.810739875 | 2.890059518 | 3.779573074 |
| ENSMUSG00000004100 | Ppan | 2474 | 10.78951113 | 10.87987814 | 9.659063862 | 11.25214151 | 9.785916574 | 10.41528758 | 10.44168258 | 11.25394558 | 11.02080575 | 11.32074282 | 14.25138212 | 10.51337864 | 22.45260261 | 21.43862078 | 13.92259528 | 19.45484782 | 17.89283465 | 20.36715512 |
| ENSMUSG00000004105 | Angptl2 | 6092 | 13.88465205 | 19.24295383 | 14.4358303 | 15.54849166 | 14.71136603 | 15.17003416 | 10.08073822 | 13.70462765 | 11.26341682 | 11.21493254 | 15.9903382 | 11.15946767 | 8.956188527 | 7.440812425 | 8.339054237 | 10.30175731 | 9.732564233 | 8.641450285 |
| ENSMUSG00000004110 | Cacna1e | 12649 | 0.184819723 | 0.189621977 | 0.240930699 | 0.12967014 | 0.213090974 | 0.207628657 | 0.089770282 | 0.042271613 | 0.140012663 | 0.085735329 | 0.135971105 | 0.101491046 | 0.046800775 | 0.040389356 | 0.02605838 | 0.018162989 | 0 | 0.00434889 |
| ENSMUSG00000004113 | Cacna1b | 13098 | 0 | 0 | 0 | 0.01043542 | 0 | 0 | 0.126427223 | 0.19828091 | 0.031444889 | 0.053997599 | 0.0036475 | 0 | 0.128056588 | 0.223391167 | 0.044038921 | 0.055544476 | 0.002926118 | 0.096595622 |
| ENSMUSG00000004127 | Rg9mtd2 | 7073 | 1.195890008 | 1.257534675 | 1.962845074 | 1.288308902 | 1.429054711 | 1.618362994 | 1.959934247 | 1.565926297 | 1.793503019 | 1.666573705 | 2.72208515 | 1.815015184 | 1.618125945 | 1.451172531 | 1.998039541 | 1.504989235 | 1.300482293 | 2.278759829 |
| ENSMUSG00000004151 | Etv1 | 5276 | 0.636449755 | 0.423040924 | 0.348406777 | 0.561309303 | 0.492631516 | 0.572918262 | 1.147843613 | 0.723889356 | 2.740043031 | 1.054545676 | 0.40748164 | 1.322395342 | 0.776070723 | 0.202466643 | 0.531028393 | 0.928960907 | 0.290570864 | 0.865382081 |
| ENSMUSG00000004187 | Kifc2 | 3199 | 6.032306945 | 5.040150298 | 5.715909495 | 5.355096177 | 4.393410976 | 6.567789633 | 7.394916994 | 6.638004294 | 8.65187202 | 6.499987539 | 4.793923624 | 7.188512968 | 4.040313325 | 4.253866085 | 5.486671713 | 4.153435689 | 2.743582992 | 5.812153195 |
| ENSMUSG00000004207 | Psap | 2867 | 410.0778406 | 432.3704593 | 396.9261603 | 398.1784189 | 411.3961596 | 400.9329476 | 315.9404388 | 361.8487013 | 365.4926174 | 371.2521714 | 379.4501498 | 364.2327281 | 346.6827587 | 309.1358631 | 311.9792734 | 372.3684775 | 343.9073297 | 322.1879735 |
| ENSMUSG00000004221 | Ikbkg | 8205 | 7.47531615 | 7.251289345 | 7.575866728 | 8.201543264 | 8.13637759 | 7.639757218 | 6.856157861 | 7.135765129 | 6.088877955 | 7.194720351 | 7.371492679 | 6.224415755 | 5.284918377 | 5.507629132 | 5.609035701 | 5.478753576 | 8.473358023 | 5.162341521 |
| ENSMUSG00000004231 | Pax2 | 3611 | 3.284116342 | 2.638462406 | 4.487713284 | 3.709484086 | 2.825801113 | 3.773747365 | 4.80857191 | 4.410476822 | 4.756238458 | 5.079375122 | 4.326339358 | 4.405279379 | 5.027456538 | 6.032200771 | 4.826435376 | 4.44302534 | 3.714816925 | 4.29592027 |
| ENSMUSG00000004233 | Wars2 | 5972 | 1.886113806 | 2.632901218 | 2.235615268 | 1.975939465 | 1.628038945 | 1.999700066 | 1.830078505 | 2.730769562 | 1.931048964 | 2.952840425 | 3.159931112 | 2.710405584 | 3.130742908 | 2.441969766 | 1.579898911 | 2.038917139 | 2.849443373 | 2.054090891 |
| ENSMUSG00000004263 | Atn1 | 5070 | 7.821958429 | 8.180385267 | 7.71877437 | 7.58452099 | 7.224535288 | 6.958884873 | 6.02840051 | 7.977466301 | 8.472886773 | 7.412053352 | 6.011917852 | 8.432898923 | 8.367938631 | 7.749841801 | 7.005077155 | 8.428465061 | 7.415798276 | 8.278490815 |
| ENSMUSG00000004264 | Phb2 | 2313 | 75.41774424 | 71.58023531 | 65.16728084 | 68.15443727 | 68.33775933 | 66.11315859 | 70.91788061 | 70.9027998 | 69.42768048 | 68.12695646 | 73.24254335 | 70.70466553 | 67.65276334 | 63.77277196 | 60.90277544 | 69.39655263 | 58.30965793 | 65.6637024 |
| ENSMUSG00000004266 | Ptpn6 | 2742 | 14.16839201 | 12.48929805 | 11.00841829 | 12.1795206 | 11.93643621 | 9.523811304 | 15.70187375 | 12.0761535 | 11.98644769 | 12.7076476 | 16.72646249 | 17.03782261 | 15.22056966 | 10.33220033 | 10.7887371 | 13.92258774 | 11.15404264 | 9.368801181 |
| ENSMUSG00000004267 | Eno2 | 5770 | 1.605914796 | 0.617760777 | 1.006035293 | 1.002816747 | 1.092769844 | 1.090675418 | 1.106969091 | 0.966393544 | 1.020740339 | 2.917296545 | 1.697377208 | 2.360315714 | 1.795442397 | 1.577650109 | 1.363864328 | 2.236383829 | 1.122555317 | 1.220306007 |
| ENSMUSG00000004268 | Emg1 | 1377 | 37.13415161 | 37.25625062 | 36.35918948 | 38.67891128 | 38.62438319 | 32.60332362 | 35.8023322 | 36.47277354 | 33.9183071 | 29.58475499 | 31.43363281 | 35.50800321 | 35.39574021 | 40.03568027 | 37.76061259 | 37.1783167 | 32.39782832 | 41.14697162 |
| ENSMUSG00000004270 | Lpcat3 | 3370 | 53.6296761 | 52.69763771 | 52.00513367 | 55.67370343 | 58.24391238 | 58.19872982 | 46.41415921 | 52.50605351 | 43.9241358 | 50.39668639 | 50.62444413 | 44.1059672 | 44.17914728 | 42.90220891 | 43.81791582 | 47.5962476 | 48.01591262 | 44.43168798 |
| ENSMUSG00000004285 | Atp6v1f | 1059 | 233.1964804 | 196.1969039 | 201.4878587 | 187.9231744 | 207.4354104 | 188.1974452 | 275.6108747 | 223.5283382 | 247.8191487 | 258.41613 | 237.1604879 | 213.0376848 | 310.8050539 | 287.4358345 | 239.1168673 | 297.1770559 | 241.0684265 | 260.3972162 |
| ENSMUSG00000004296 | Il12b | 2014 | 0 | 0 | 0 | 0 | 0 | 0 | 0 | 0 | 0 | 0 | 0 | 0 | 0 | 0 | 0 | 0 | 0 | 0 |
| ENSMUSG00000004317 | Clcn5 | 11787 | 380.9310486 | 408.4482457 | 402.1524233 | 384.1398648 | 408.3556996 | 403.3416785 | 340.2603275 | 383.4631257 | 358.8157233 | 349.3600296 | 382.0780721 | 379.0085689 | 301.1226592 | 282.8175801 | 314.7879966 | 335.2629037 | 374.2561104 | 306.7713254 |

| ENSMUSG00000004319 | Clcn3 | 18308 | 10.96293649 | 10.3752525 | 10.63752639 | 11.13642525 | 10.22685072 | 11.20539202 | 9.688400333 | 10.51396702 | 9.093068424 | 10.26045454 | 8.679239234 | 10.65521852 | 8.940534276 | 9.117328494 | 8.695805766 | 9.394877018 | 11.68127249 | 9.230280525 |
| --- | --- | --- | --- | --- | --- | --- | --- | --- | --- | --- | --- | --- | --- | --- | --- | --- | --- | --- | --- | --- |
| ENSMUSG00000004328 | Hif3a | 7124 | 1.115731038 | 1.22515136 | 1.262981 | 0.91454651 | 0.939126529 | 0.674707349 | 2.523697079 | 2.181963473 | 1.959886137 | 2.303262877 | 2.957433561 | 2.178095761 | 2.638329658 | 2.092722074 | 1.833365134 | 1.521089542 | 2.620003733 | 1.366733842 |
| ENSMUSG00000004341 | Gpx6 | 1270 | 0.769778778 | 2.334525658 | 0.723698486 | 1.721992227 | 0.378991525 | 0.936429611 | 0 | 0 | 0 | 0 | 0 | 0 | 0.543816411 | 1.828507378 | 0.87593859 | 0 | 0 | 0 |
| ENSMUSG00000004344 | Gpx5 | 1669 | 345.7206498 | 766.8752 | 395.7992394 | 464.0729501 | 263.9613525 | 576.7890684 | 0.765393847 | 0.800919164 | 0.814352918 | 0.819275251 | 0.744247406 | 0.601966403 | 10.22698824 | 16.66866887 | 10.41764924 | 2.248340039 | 2.066726616 | 5.141654074 |
| ENSMUSG00000004347 | Pde1c | 13929 | 3.689335761 | 3.243038593 | 2.708834351 | 3.607856408 | 2.577817148 | 2.760637609 | 1.980277968 | 2.363547338 | 2.398037496 | 2.024274574 | 1.591469649 | 2.592629312 | 0.417917025 | 0.236738474 | 0.328334604 | 0.835691338 | 0.258645409 | 0.169817761 |
| ENSMUSG00000004356 | Utp20 | 8769 | 1.803161761 | 1.382813718 | 1.125350689 | 1.189814021 | 1.053863536 | 1.1640852 | 0.954994203 | 1.010450149 | 0.831339182 | 0.731268747 | 0.964325197 | 0.719257597 | 0.815729046 | 1.477694193 | 1.033680271 | 1.109113272 | 0.799830349 | 0.558308872 |
| ENSMUSG00000004359 | Spic | 3907 | 0 | 0 | 0 | 0 | 0 | 0 | 0 | 0 | 0 | 0 | 0 | 0 | 0 | 0 | 0 | 0 | 0 | 0 |
| ENSMUSG00000004360 | 9330159F19R | 5638 | 0 | 0.200893651 | 0.051479458 | 0.056567455 | 0.179278183 | 0.026367187 | 0.025175246 | 0.013548209 | 0 | 0 | 0 | 0.108898951 | 0.314996279 | 0.214179899 | 0.007307832 | 0.088289801 | 0.088372093 | 0.175623257 |
| ENSMUSG00000004364 | Cul3 | 5320 | 27.58042625 | 28.29717462 | 29.29690128 | 29.68319167 | 28.17346058 | 31.57589602 | 25.25714439 | 28.71609203 | 28.06412023 | 30.16935975 | 30.62267075 | 25.69408979 | 18.74983773 | 24.6712389 | 25.14688947 | 25.23437398 | 28.12516224 | 25.62266287 |
| ENSMUSG00000004366 | Sst | 597 | 0 | 0 | 0 | 0 | 0 | 0 | 0 | 0 | 0 | 0 | 0 | 0 | 0 | 0 | 0 | 0 | 0 | 0 |
| ENSMUSG00000004371 | Il11 | 1828 | 0.255775129 | 0.109342104 | 0.052925088 | 0.099695939 | 0.157982244 | 0.189753356 | 0.414115352 | 0.167143993 | 0.20277825 | 0.180555209 | 0 | 0.122134929 | 0.18890778 | 0 | 0.270469732 | 0.104733609 | 0.104831228 | 0.180555055 |
| ENSMUSG00000004383 | Large | 7868 | 0.394366783 | 0.410695315 | 0.387332538 | 0.335859249 | 0.428219961 | 0.16374823 | 0.162359218 | 0.364060775 | 0.581050235 | 0.125847073 | 0.206449999 | 0.212820269 | 0.175558424 | 0.230213448 | 0.083785574 | 0.043799627 | 0.068196258 | 0.062923483 |
| ENSMUSG00000004393 | Ddx56 | 2519 | 29.20859853 | 29.86126048 | 29.47732001 | 32.80973975 | 31.64210623 | 29.5663988 | 26.89629068 | 27.80661612 | 29.51237037 | 26.76677477 | 29.37809078 | 27.38712146 | 35.62316694 | 36.15595688 | 35.91846635 | 34.00400506 | 32.02733425 | 31.2060395 |
| ENSMUSG00000004394 | Tmed4 | 2308 | 145.0664088 | 150.2399982 | 145.5816906 | 141.4405904 | 145.855578 | 147.5632313 | 157.4971023 | 145.620945 | 152.2723306 | 148.3571954 | 151.2942684 | 151.4616529 | 150.8385356 | 148.2669133 | 163.9136422 | 171.3786718 | 146.380549 | 164.5506381 |
| ENSMUSG00000004415 | Emid2 | 6701 | 0 | 0 | 0 | 0.013598282 | 0 | 0 | 0 | 0 | 0 | 0 | 0 | 0 | 0 | 0 | 0 | 0 | 0 | 0 |
| ENSMUSG00000004446 | Bid | 3541 | 23.5633043 | 20.56537327 | 23.22366608 | 21.48741713 | 23.5698265 | 22.20846885 | 21.04418789 | 16.68558219 | 16.55137304 | 18.28238226 | 18.7268123 | 17.24437585 | 10.28154616 | 14.1129167 | 16.59232384 | 19.71301251 | 16.13795783 | 15.03779026 |
| ENSMUSG00000004451 | Ralb | 4728 | 15.04942146 | 18.05857612 | 13.94524574 | 16.79629871 | 18.44649866 | 17.3141108 | 14.11975253 | 15.16225451 | 12.75318508 | 14.38056559 | 18.76440311 | 12.00603402 | 14.38847587 | 17.16112958 | 15.29372474 | 14.5047542 | 16.46377094 | 18.20838664 |
| ENSMUSG00000004455 | Ppp1cc | 3676 | 31.64762426 | 32.72384685 | 29.26624905 | 31.85306963 | 32.74699159 | 30.37059542 | 27.46606551 | 29.64170948 | 29.53418268 | 30.12976114 | 31.04852443 | 25.22029803 | 25.83349857 | 27.11344166 | 27.9533951 | 29.4679348 | 30.694401 | 26.93590645 |
| ENSMUSG00000004460 | Dnajb11 | 3246 | 50.78098356 | 49.00464261 | 45.45263943 | 47.7787415 | 47.98364294 | 47.96509208 | 45.89883732 | 47.88757788 | 47.17543611 | 49.34409703 | 46.95074478 | 44.65596911 | 54.57514323 | 58.14811188 | 56.34433352 | 54.68738629 | 50.64124689 | 52.12816152 |
| ENSMUSG00000004462 | Tbccd1 | 2694 | 9.845296897 | 10.06558868 | 11.83302023 | 12.56564809 | 12.9530975 | 11.71681191 | 11.90719982 | 14.48872875 | 12.64337362 | 12.5490179 | 12.59102469 | 12.88691245 | 13.38590628 | 13.42982334 | 12.43387711 | 9.579758496 | 11.02556797 | 15.21224369 |
| ENSMUSG00000004473 | Clec11a | 1948 | 4.756739412 | 4.754098867 | 4.296006546 | 3.344573583 | 3.805090475 | 4.858609914 | 6.169107006 | 4.823065191 | 5.687460339 | 5.663893512 | 4.610724946 | 4.670407085 | 6.888234856 | 8.535412364 | 5.393427491 | 4.697872263 | 5.646636145 | 6.38196002 |
| ENSMUSG00000004500 | Zfp324 | 5226 | 1.586014031 | 1.52986885 | 1.508780223 | 1.124641163 | 0.976269405 | 2.067067723 | 2.308597996 | 2.572469508 | 1.08758882 | 1.163880735 | 2.395147172 | 1.762262596 | 1.633069592 | 1.635230019 | 1.206245322 | 1.428754008 | 1.188071201 | 1.894687947 |
| ENSMUSG00000004508 | Gab2 | 5987 | 2.044678586 | 2.442685913 | 2.779437856 | 1.735079023 | 2.572609917 | 2.383695886 | 0.418836142 | 1.116363859 | 0.935588132 | 1.63023143 | 1.165048261 | 0.978895035 | 0.41199196 | 0.806778869 | 0.867111415 | 0.447693756 | 0.896222076 | 1.06581867 |
| ENSMUSG00000004530 | Coro1c | 9649 | 7.114297781 | 8.976425015 | 7.179075082 | 8.938445116 | 8.205722296 | 8.879332196 | 9.046729466 | 7.55219233 | 8.075954796 | 9.29429346 | 7.902252434 | 6.640727604 | 7.83257304 | 6.873474639 | 6.844864141 | 5.162823114 | 7.80904754 | 6.379437291 |
| ENSMUSG00000004535 | Tax1bp1 | 6482 | 67.65942707 | 72.0220456 | 72.88865643 | 73.5288622 | 71.97512128 | 73.79397365 | 67.16619431 | 68.48353798 | 69.14403957 | 68.54382549 | 62.6926561 | 66.9925725 | 61.30339304 | 60.87457057 | 69.99560837 | 67.36594983 | 74.81964841 | 68.10368427 |
| ENSMUSG00000004540 | Psg17 | 2095 | 0 | 0 | 0 | 0 | 0 | 0 | 0 | 0 | 0 | 0 | 0 | 0 | 0 | 0 | 0 | 0 | 0 | 0 |
| ENSMUSG00000004542 | Psg19 | 3082 | 0 | 0 | 0 | 0 | 0 | 0.01607811 | 0 | 0 | 0 | 0 | 0 | 0 | 0 | 0 | 0 | 0 | 0 | 0 |
| ENSMUSG00000004552 | Ctse | 3221 | 0.593832015 | 1.044583147 | 2.312798409 | 0.69310499 | 1.255225578 | 0.415375289 | 1.131038894 | 0.260860867 | 0.690491106 | 0.453794412 | 1.587060092 | 0.086643376 | 0.964890031 | 1.095855524 | 1.048906421 | 1.48597514 | 0.725831764 | 0.085391349 |
| ENSMUSG00000004558 | Ndrg2 | 2141 | 12.52721442 | 10.44042598 | 10.39319195 | 11.64030418 | 10.72345987 | 10.64654715 | 11.82264513 | 14.98440823 | 10.58037537 | 15.19569605 | 14.9505942 | 13.24351079 | 15.94473926 | 13.83994757 | 11.43097814 | 15.57735593 | 13.92707099 | 15.8013081 |
| ENSMUSG00000004561 | Mettl17 | 3719 | 14.02362221 | 12.54048182 | 12.29174138 | 12.65516504 | 11.69972008 | 11.12571458 | 15.58430537 | 13.23743122 | 14.96181307 | 13.13474821 | 15.36403612 | 16.08883848 | 15.62597433 | 14.58636571 | 13.98127549 | 13.59064312 | 12.12961857 | 16.15217654 |
| ENSMUSG00000004562 | Arhgef40 | 5620 | 2.647119504 | 2.649619878 | 2.521965195 | 3.396810957 | 2.046891416 | 2.636346502 | 2.037307527 | 1.420322438 | 2.249868388 | 1.929655116 | 0.918095287 | 1.082545769 | 0.93045965 | 0.801615031 | 0.527849115 | 0.354290318 | 0.272785031 | 0.362159599 |
| ENSMUSG00000004565 | Pnpla6 | 4461 | 16.82675204 | 16.59297264 | 15.33292354 | 21.81537563 | 17.7271353 | 17.77278048 | 15.62241113 | 17.13136006 | 11.94695167 | 15.65348387 | 14.39353154 | 15.01430063 | 10.2069849 | 13.36790141 | 12.28380841 | 11.106937 | 11.28911772 | 12.84902246 |
| ENSMUSG00000004567 | Mcoln1 | 3350 | 47.16171322 | 41.83502865 | 45.87543461 | 44.0106088 | 42.70091855 | 46.0766463 | 49.14869333 | 46.34391516 | 41.48158334 | 45.65877056 | 44.86567743 | 46.35199203 | 40.6583354 | 40.67670221 | 41.55822608 | 44.25708414 | 35.90087155 | 43.74455532 |
| ENSMUSG00000004568 | Arhgef18 | 8150 | 10.21167296 | 10.76640041 | 11.14075049 | 11.59989779 | 11.33314865 | 12.08113885 | 11.06478111 | 9.091197628 | 10.1879774 | 11.27567125 | 10.41083758 | 11.33434621 | 7.233330202 | 6.969462439 | 10.49502209 | 9.387071606 | 9.677977795 | 9.132186674 |
| ENSMUSG00000004591 | Pkn2 | 7617 | 13.94517985 | 12.15830982 | 11.65359435 | 13.39854789 | 12.73913064 | 12.38655701 | 10.69613143 | 12.80099821 | 12.83665333 | 12.68370631 | 11.34004528 | 12.83093412 | 11.16558033 | 11.1278009 | 11.06172795 | 11.44646358 | 12.67479873 | 13.93824025 |
| ENSMUSG00000004609 | Cd33 | 2564 | 0 | 0 | 0 | 0 | 0 | 0 | 0 | 0 | 0 | 0 | 0 | 0 | 0 | 0 | 0 | 0 | 0 | 0 |
| ENSMUSG00000004610 | Etfb | 1115 | 129.3453473 | 122.4360901 | 118.3090738 | 117.6823387 | 121.0852429 | 127.7260595 | 112.9563689 | 118.0025348 | 110.1139045 | 128.300658 | 123.1865521 | 113.7337986 | 135.9171748 | 119.5466644 | 114.034082 | 127.4750984 | 98.54842441 | 117.6158838 |
| ENSMUSG00000004612 | Nkg7 | 1204 | 0 | 0 | 0 | 0 | 0 | 0 | 0 | 0 | 0 | 0 | 0 | 0 | 0 | 0 | 0 | 0 | 0 | 0 |
| ENSMUSG00000004613 | Lim2 | 1850 | 0 | 0 | 0 | 0 | 0 | 0 | 0 | 0 | 0 | 0 | 0 | 0 | 0 | 0 | 0 | 0 | 0 | 0 |
| ENSMUSG00000004626 | Stxbp2 | 4119 | 72.379535 | 71.51070908 | 71.72059963 | 79.1206968 | 72.77630993 | 72.36214918 | 56.37548633 | 63.50564799 | 55.97526468 | 62.78747448 | 61.86735337 | 64.33910317 | 59.61987963 | 60.53855431 | 60.07684989 | 62.37678457 | 58.50831632 | 73.26534583 |
| ENSMUSG00000004630 | Pcp2 | 966 | 0 | 0 | 0 | 0 | 0 | 0 | 0.146933785 | 0.158146594 | 0 | 0.146430755 | 0.049456479 | 0 | 0 | 0 | 0.042651714 | 0 | 0.039675256 | 0.056945245 |

| ENSMUSG00000004631 | Sgce | 3352 | 16.2691351 | 15.00670354 | 16.52377454 | 17.61548731 | 14.25865161 | 16.17264037 | 11.44707118 | 13.9347578 | 15.16233904 | 16.21860407 | 14.26692525 | 15.65236361 | 12.31826216 | 12.17910287 | 15.14329262 | 13.52508328 | 16.20177885 | 12.84968096 |
| --- | --- | --- | --- | --- | --- | --- | --- | --- | --- | --- | --- | --- | --- | --- | --- | --- | --- | --- | --- | --- |
| ENSMUSG00000004633 | Chn2 | 6308 | 0.033691484 | 0.042248439 | 0.076686002 | 0.02166822 | 0.030521194 | 0.062844304 | 0 | 0.151364943 | 0 | 0.007474747 | 0 | 0 | 0.039102661 | 0.265058203 | 0.006531635 | 0.006070166 | 0.127592301 | 0 |
| ENSMUSG00000004637 | Wwox | 10339 | 2.277576895 | 2.661422829 | 2.938250997 | 2.52504869 | 2.932886344 | 2.952363273 | 3.262785577 | 2.785285948 | 2.210902045 | 3.461397018 | 2.758646892 | 2.413154191 | 3.053721545 | 3.050153336 | 3.447078619 | 4.114601492 | 2.620823282 | 2.91033769 |
| ENSMUSG00000004642 | Slbp | 3912 | 18.38414055 | 15.60903249 | 18.04115051 | 15.26854016 | 16.17931483 | 17.29025596 | 19.44754287 | 17.97346954 | 16.05558194 | 19.10374864 | 16.01047305 | 16.7504059 | 15.24598364 | 17.30968288 | 18.82085388 | 18.20565689 | 18.50674204 | 16.73334279 |
| ENSMUSG00000004651 | Tyr | 5394 | 0 | 0 | 0 | 0 | 0 | 0 | 0 | 0 | 0 | 0 | 0 | 0 | 0 | 0 | 0 | 0 | 0 | 0 |
| ENSMUSG00000004654 | Ghrhr | 1585 | 0 | 0 | 0 | 0 | 0 | 0 | 0.208951894 | 0.072288459 | 0.545688852 | 0 | 0.150709648 | 0 | 0 | 1.7581358 | 0 | 0 | 0.072541887 | 0 |
| ENSMUSG00000004655 | Aqp1 | 2623 | 0.016204795 | 0.076201817 | 0.018442063 | 0 | 0.055049856 | 0.094458127 | 0 | 0 | 0.219828906 | 0.053927606 | 0 | 0.297910513 | 0.037614882 | 0.389542479 | 0.062831195 | 0.072990102 | 0.08766976 | 0 |
| ENSMUSG00000004661 | Arid3b | 6532 | 1.444603349 | 2.09608076 | 1.644048337 | 2.469168661 | 1.45162109 | 1.729642262 | 2.491665884 | 2.040592229 | 2.402336575 | 2.779090746 | 3.006048367 | 2.409678023 | 2.522483217 | 1.990867188 | 1.608450207 | 2.514799537 | 2.7518422 | 2.501179531 |
| ENSMUSG00000004665 | Cnn2 | 2570 | 23.66727895 | 23.65604356 | 25.10906206 | 24.48241332 | 26.89394645 | 26.04892726 | 13.25491394 | 15.32154353 | 12.86878294 | 15.0075001 | 20.72726798 | 19.00338707 | 15.66336362 | 12.37906609 | 13.73919587 | 20.47132084 | 14.00326568 | 11.4513121 |
| ENSMUSG00000004667 | Polr2e | 2954 | 36.44739705 | 32.39943977 | 33.52085871 | 32.08089784 | 32.37580648 | 29.74170502 | 38.79191211 | 31.50808553 | 37.61720363 | 31.93925431 | 33.76916496 | 34.44548168 | 39.56239405 | 38.31558597 | 33.68373645 | 33.49455976 | 32.0596749 | 39.66465718 |
| ENSMUSG00000004668 | Abca13 | 19682 | 0 | 0 | 0 | 0 | 0 | 0 | 0 | 0 | 0 | 0 | 0 | 0 | 0 | 0.002359724 | 0 | 0 | 0 | 0 |
| ENSMUSG00000004677 | Myo9b | 7346 | 7.452581912 | 7.759101825 | 6.025289991 | 7.765100396 | 6.899389548 | 7.028852217 | 5.719256587 | 5.734579343 | 5.982304971 | 5.256796347 | 4.292332235 | 5.067934515 | 4.767990916 | 6.10108145 | 5.468488564 | 5.00915787 | 5.968592929 | 5.002189394 |
| ENSMUSG00000004698 | Hdac9 | 9564 | 0.075552906 | 0.05573048 | 0.146678417 | 0.028582838 | 0.070456601 | 0.08289876 | 0.143461699 | 0 | 0 | 0.019720077 | 0.09491052 | 0.005836017 | 0 | 0.16996477 | 0.090467657 | 0.208188162 | 0.01202205 | 0 |
| ENSMUSG00000004707 | Ly9 | 3789 | 0 | 0 | 0 | 0 | 0 | 0 | 0 | 0.040319242 | 0.152180317 | 0.099552818 | 0.214350566 | 0 | 0.403612942 | 0.183864162 | 0.097865928 | 0.090951562 | 0 | 0 |
| ENSMUSG00000004709 | Cd244 | 2243 | 0 | 0 | 0.08626577 | 0 | 0 | 0 | 0.253121778 | 0.442711753 | 0 | 0 | 0.149097062 | 0 | 0.021993721 | 0 | 0 | 0.187782738 | 0 | 0 |
| ENSMUSG00000004730 | Emr1 | 3274 | 0 | 0.010174983 | 0 | 0 | 0 | 0 | 0 | 0 | 0.239017654 | 0 | 0 | 0.238674183 | 0 | 0.099300126 | 0 | 0.01169536 | 0 | 0 |
| ENSMUSG00000004748 | Mtfp1 | 1733 | 1.079185081 | 1.441700558 | 1.032787434 | 1.551126149 | 1.305366655 | 0.428904214 | 1.283148246 | 2.247908278 | 1.8537497 | 3.482567811 | 2.01244776 | 2.222320091 | 2.96048436 | 2.519183044 | 1.640454331 | 1.966453583 | 0.575005031 | 2.920275718 |
| ENSMUSG00000004768 | Rab23 | 8551 | 1.655271152 | 1.562211505 | 1.702775422 | 1.641071413 | 1.992597471 | 2.086186889 | 1.803757845 | 1.28186405 | 2.408286502 | 1.929920026 | 1.821381881 | 2.284584482 | 2.255733792 | 2.569062488 | 1.48886455 | 1.89415588 | 1.523908426 | 1.833422455 |
| ENSMUSG00000004771 | Rab11a | 3332 | 94.23341396 | 97.15927552 | 100.2313486 | 99.79126698 | 98.61844024 | 95.75931918 | 98.07583234 | 87.60640204 | 97.02069694 | 91.62689183 | 85.71389609 | 88.1961175 | 93.21541152 | 89.18045352 | 91.14545873 | 106.6207249 | 99.76109635 | 81.37451592 |
| ENSMUSG00000004788 | Eif2b2 | 2001 | 35.2404233 | 36.82564818 | 31.76552668 | 35.04170276 | 31.77524796 | 32.19318024 | 44.09702459 | 36.03561007 | 35.23803847 | 35.01546479 | 39.65727438 | 36.56888238 | 39.79096176 | 43.31067819 | 37.00109742 | 35.9943191 | 35.58733615 | 37.03011832 |
| ENSMUSG00000004789 | Dlst | 2765 | 55.03382647 | 48.14405978 | 47.09639917 | 49.2850212 | 41.44741781 | 44.19422821 | 50.73493419 | 46.86684317 | 43.00378698 | 50.4589262 | 52.76843515 | 47.21621511 | 44.81800224 | 42.44636847 | 36.80572986 | 43.10982309 | 50.62120661 | 47.13076811 |
| ENSMUSG00000004791 | Pgf | 1575 | 19.94369847 | 13.07134516 | 17.99802463 | 16.95160126 | 16.93021315 | 14.56693057 | 19.4657879 | 11.05760984 | 14.59179416 | 9.280455879 | 7.61366005 | 12.79330425 | 9.615808637 | 7.313100744 | 5.676658812 | 3.111871586 | 5.913200094 | 10.26836659 |
| ENSMUSG00000004798 | Ulk2 | 6287 | 6.551219273 | 6.67635545 | 6.124595222 | 6.993225351 | 6.231809427 | 7.432515948 | 5.237732549 | 6.141644478 | 4.54643581 | 7.709706211 | 5.00014676 | 7.484110635 | 6.206703764 | 5.082476877 | 6.560005956 | 5.859004982 | 7.303149943 | 6.75473682 |
| ENSMUSG00000004814 | Ccl24 | 1286 | 0 | 0 | 0 | 0 | 0 | 0 | 0.331115171 | 0.801862259 | 0.192161038 | 0 | 0.074300091 | 0 | 0 | 0 | 0 | 0 | 0 | 0 |
| ENSMUSG00000004815 | Dgkq | 8837 | 1.721947835 | 1.960247255 | 2.320966037 | 1.752942749 | 1.481485033 | 2.562589029 | 2.237942717 | 2.281949584 | 1.496081426 | 1.78742623 | 1.459685277 | 1.939052667 | 1.54075016 | 1.424278339 | 1.841644741 | 2.26181929 | 1.192682092 | 2.029305057 |
| ENSMUSG00000004821 | Tmed11 | 758 | 0 | 0 | 0 | 0 | 0 | 0 | 0 | 0 | 0 | 0 | 0 | 0 | 0 | 0 | 0 | 0 | 0 | 0 |
| ENSMUSG00000004837 | Grap | 1787 | 0 | 0 | 0 | 0.050991656 | 0.24241036 | 0.083188697 | 0.741329049 | 0.042744714 | 0.507052796 | 0.158312378 | 1.390205843 | 0 | 0.496907952 | 0.441829595 | 0.368900333 | 0.492828187 | 0 | 0 |
| ENSMUSG00000004842 | Pou1f1 | 3898 | 0 | 0 | 0 | 0 | 0 | 0 | 0 | 0.068585638 | 0 | 0.012096127 | 0.012256275 | 0.042957154 | 0.075934198 | 0.035744552 | 0.05284961 | 0.009823142 | 0.009832298 | 0.028224272 |
| ENSMUSG00000004843 | Chmp2b | 1832 | 82.75980507 | 79.24541103 | 82.62051118 | 80.08000151 | 79.08138114 | 81.19940295 | 80.21461831 | 67.37873612 | 73.24545216 | 72.03865634 | 64.75175868 | 71.96320689 | 53.31724684 | 58.02975766 | 58.94611244 | 72.19200762 | 74.12121721 | 59.00267179 |
| ENSMUSG00000004846 | Plod3 | 4812 | 5.034902397 | 6.085210727 | 5.10676505 | 5.98391106 | 5.761427272 | 5.313634773 | 5.751853112 | 5.865375182 | 5.751732875 | 5.898737191 | 6.512961921 | 4.778897129 | 4.623585763 | 3.252630393 | 5.085977598 | 5.07676798 | 3.472623743 | 5.018494982 |
| ENSMUSG00000004849 | Ap1s1 | 2640 | 22.36351875 | 23.91209642 | 20.72366016 | 22.4008468 | 22.46156438 | 21.36023137 | 18.01107664 | 21.12155588 | 20.09402739 | 22.48588462 | 21.73398679 | 20.40231606 | 21.26504616 | 19.65077485 | 22.11462299 | 23.59803721 | 20.45520922 | 21.19100816 |
| ENSMUSG00000004864 | Mapk13 | 1693 | 26.91408665 | 28.84625099 | 35.34439282 | 28.47228878 | 30.98865729 | 41.06466935 | 26.38108028 | 24.58931992 | 29.9715224 | 25.51095345 | 27.17500592 | 35.57300646 | 20.83421196 | 24.05875053 | 23.04658797 | 17.82220829 | 26.87142021 | 30.25013488 |
| ENSMUSG00000004865 | Srpk1 | 9869 | 6.770507317 | 6.545111145 | 7.631734397 | 7.326515084 | 6.106106843 | 7.225289656 | 6.155586153 | 5.615277727 | 6.381009484 | 5.279311693 | 5.412139384 | 6.639739373 | 5.108645198 | 5.242548723 | 5.907407193 | 4.989535021 | 5.81360487 | 5.969678113 |
| ENSMUSG00000004872 | Pax3 | 7743 | 0 | 0 | 0 | 0 | 0 | 0 | 0 | 0 | 0 | 0 | 0 | 0 | 0 | 0 | 0 | 0 | 0 | 0 |
| ENSMUSG00000004880 | Lbr | 3537 | 9.049023904 | 12.4040378 | 10.42143911 | 12.10839177 | 10.17887444 | 9.176432144 | 8.989007701 | 14.01575864 | 11.2718539 | 9.051548632 | 10.88680139 | 10.79386859 | 8.828697664 | 10.91180001 | 9.563606838 | 10.71747281 | 13.33889496 | 10.65344589 |
| ENSMUSG00000004885 | Crabp2 | 1318 | 0.096749263 | 0.126376685 | 0 | 0.069136638 | 0.03651891 | 0.075193829 | 2.799991621 | 1.912517875 | 2.687435778 | 0.608165368 | 0.507473004 | 0.846975153 | 20.21186292 | 27.9087384 | 14.817555 | 4.503068397 | 4.856215161 | 13.02188262 |
| ENSMUSG00000004891 | Nes | 7307 | 2.751464111 | 4.499770986 | 2.005909361 | 3.136335747 | 3.642665087 | 2.339632282 | 0.24604924 | 0.256114372 | 0.20855359 | 0.335546267 | 0.405371209 | 0.084025221 | 0.35782012 | 0.368654313 | 0.186075181 | 1.634962298 | 0.902165467 | 0.210791705 |
| ENSMUSG00000004892 | Bcan | 3253 | 0 | 0 | 0 | 0 | 0 | 0.015232934 | 0 | 0 | 0 | 0 | 0 | 0 | 0 | 0.014277309 | 0 | 0 | 0 | 0 |
| ENSMUSG00000004894 | Hapln2 | 3096 | 0 | 0 | 0 | 0 | 0.062185948 | 0.016005405 | 0 | 0 | 0 | 0 | 0.108018317 | 0 | 0 | 0 | 0 | 0 | 0.049517179 | 0.106606796 |
| ENSMUSG00000004895 | Prcc | 2095 | 6.025793451 | 4.182000563 | 5.379967815 | 8.568520987 | 4.778730369 | 4.470389807 | 5.239399289 | 4.047118536 | 4.973836987 | 6.14422052 | 6.271175934 | 3.06386692 | 5.463009455 | 2.615943824 | 3.89398953 | 7.054975874 | 4.628426309 | 5.225208705 |
| ENSMUSG00000004896 | Rrnad1 | 5313 | 4.000110675 | 4.4392112 | 5.025821336 | 4.66501188 | 4.121969748 | 3.404243884 | 4.675165902 | 4.291523477 | 4.248110416 | 4.392922664 | 5.566101882 | 4.275733987 | 6.323176297 | 4.991450009 | 5.032902272 | 4.360214093 | 4.479697041 | 7.030149344 |

| ENSMUSG00000004897 | Hdgf | 2863 | 58.79165109 | 53.5589424 | 52.93547698 | 64.67344889 | 58.55518342 | 52.21816179 | 66.87894224 | 60.91041194 | 57.1115849 | 73.17169912 | 60.34028986 | 63.55538248 | 69.25077734 | 68.19802423 | 62.48590838 | 64.27686314 | 65.52819537 | 61.29201902 |
| --- | --- | --- | --- | --- | --- | --- | --- | --- | --- | --- | --- | --- | --- | --- | --- | --- | --- | --- | --- | --- |
| ENSMUSG00000004902 | Slc25a18 | 1372 | 0.588628531 | 0.437049633 | 0.282061401 | 0.332077583 | 0.280652616 | 0.902928819 | 1.172471635 | 0.361881364 | 0.480309222 | 0.721694438 | 0.800892161 | 0.569547577 | 0.934861406 | 0.338513756 | 0.510514905 | 0.613989332 | 0.363150044 | 0.481129213 |
| ENSMUSG00000004929 | Thop1 | 3685 | 4.613858999 | 7.277307964 | 6.760479819 | 7.257620902 | 4.780538416 | 7.597637577 | 6.714933807 | 6.30148729 | 5.130152167 | 6.85828411 | 6.560143552 | 5.377085541 | 7.055066596 | 8.393965319 | 7.692447884 | 5.018823168 | 7.051622605 | 9.762810257 |
| ENSMUSG00000004931 | Apba3 | 2227 | 13.78030404 | 12.10154083 | 13.94513089 | 15.83486678 | 11.67096488 | 13.79555223 | 10.5800243 | 14.62870195 | 13.11236627 | 14.58771196 | 13.75112186 | 13.93511827 | 13.71192492 | 9.739285501 | 12.74713606 | 14.40840997 | 11.75431557 | 9.336974558 |
| ENSMUSG00000004933 | Matk | 3814 | 1.081017849 | 1.109265224 | 1.217582306 | 0.943713294 | 1.577475212 | 0.94843984 | 1.017210192 | 1.381896049 | 1.025883324 | 0.914827488 | 1.691038121 | 1.961011743 | 0.983016705 | 0.511445115 | 0.864217218 | 0.963790852 | 0.552686505 | 0.692301291 |
| ENSMUSG00000004934 | Pias4 | 4551 | 7.91076337 | 8.029937367 | 7.727434974 | 8.299298122 | 6.324519963 | 6.859640113 | 8.244112149 | 6.529046159 | 7.89159344 | 7.573536383 | 10.08827403 | 8.192674711 | 7.913051991 | 8.929592719 | 7.532343331 | 8.346359601 | 7.495144857 | 8.569865231 |
| ENSMUSG00000004936 | Map2k1 | 3649 | 23.78612481 | 26.27418733 | 29.56233836 | 29.87875558 | 26.47321754 | 23.41159569 | 26.65795228 | 31.62988218 | 28.88361026 | 32.54936188 | 32.01144793 | 32.93261758 | 25.42979896 | 20.0209782 | 27.12144074 | 24.81701466 | 26.09003056 | 23.89406253 |
| ENSMUSG00000004937 | Sgta | 1957 | 67.48266833 | 73.07728916 | 67.35711304 | 77.75875732 | 66.30744313 | 71.2779484 | 74.94599115 | 68.71509906 | 70.10336434 | 71.38862225 | 76.63034985 | 70.73216311 | 65.59103155 | 69.10842232 | 76.84500715 | 86.99031196 | 75.63421494 | 79.63249839 |
| ENSMUSG00000004939 | Itgb1bp3 | 1833 | 0 | 0 | 0 | 0 | 0.052517102 | 0 | 0 | 0.041672016 | 0 | 0 | 0 | 0 | 0 | 0 | 0 | 0 | 0 | 0.210072966 |
| ENSMUSG00000004945 | 5730437N04R | 999 | 70.7568646 | 61.92396853 | 62.36747457 | 56.32421397 | 65.57312123 | 59.12598441 | 66.16197444 | 65.56553566 | 73.05556149 | 66.97382174 | 65.56503311 | 71.01271987 | 73.13370319 | 62.39035563 | 68.29807463 | 73.39991309 | 56.20422919 | 63.15860402 |
| ENSMUSG00000004947 | Dtx2 | 7175 | 1.350687127 | 1.323229945 | 1.436036511 | 1.536693063 | 1.341656409 | 1.167165432 | 1.826566141 | 1.623509789 | 1.773746811 | 2.083173928 | 1.424925591 | 1.952575792 | 1.945774577 | 2.142577411 | 2.153391423 | 1.750427767 | 1.543735164 | 2.238698141 |
| ENSMUSG00000004948 | Zp3 | 1589 | 0 | 0 | 0 | 0 | 0 | 0 | 0 | 0 | 0 | 0.059346385 | 0 | 0 | 0 | 0.029228501 | 0.440797011 | 0 | 0 | 0 |
| ENSMUSG00000004951 | Hspb1 | 1408 | 51.44092326 | 52.19335557 | 49.50728497 | 50.70607704 | 53.73819887 | 56.20434341 | 43.64997864 | 47.87612944 | 49.9328 | 49.36089247 | 45.12833436 | 60.21590303 | 80.23443932 | 79.69383177 | 73.53658377 | 91.62006842 | 72.59675699 | 123.0281797 |
| ENSMUSG00000004952 | Rasa4 | 6211 | 0.793849689 | 1.458880571 | 1.355175379 | 0.982962476 | 1.635136998 | 1.372254094 | 0.891254779 | 1.918536395 | 1.13393885 | 1.070399156 | 2.099913648 | 1.015483798 | 4.090474545 | 3.664080312 | 4.610381797 | 3.791454444 | 4.683570979 | 3.737537118 |
| ENSMUSG00000004961 | Syt5 | 1739 | 4.155192596 | 5.382934604 | 3.838727538 | 3.772737426 | 2.6847594 | 4.388223675 | 3.727335833 | 3.052756719 | 1.16051712 | 2.982505669 | 3.406610035 | 2.664002297 | 1.616974862 | 2.003051498 | 1.705872354 | 3.787225114 | 2.46839865 | 2.245915226 |
| ENSMUSG00000004980 | Hnrnpa2b1 | 4614 | 225.7822625 | 212.844413 | 214.8816381 | 191.0127528 | 192.2043116 | 195.2253123 | 226.0323757 | 246.9510475 | 256.4472066 | 204.1868664 | 211.912072 | 241.9767444 | 219.5879751 | 224.3488989 | 219.3219363 | 225.1295183 | 224.6499133 | 217.3777726 |
| ENSMUSG00000004988 | Fxyd4 | 1191 | 1.284791215 | 0.531439958 | 0.487390734 | 0.956361132 | 1.212391024 | 0.374453906 | 0.31780137 | 0.7696202 | 1.037443722 | 1.543977689 | 1.52430598 | 0.702968043 | 4.349161473 | 3.236657643 | 2.352397817 | 2.73274696 | 2.316954977 | 1.570369126 |
| ENSMUSG00000004994 | Ccdc130 | 3310 | 5.406247465 | 5.062352203 | 5.100411509 | 5.905041698 | 6.5872391 | 6.70683524 | 7.10404877 | 6.715401269 | 6.196641949 | 7.435851088 | 6.884790089 | 7.487055646 | 6.811083443 | 7.63310681 | 8.900033976 | 7.102849832 | 7.039996531 | 9.472867321 |
| ENSMUSG00000004996 | Mri1 | 3388 | 18.00322538 | 18.83928729 | 18.54699408 | 16.41972702 | 15.96820608 | 15.48888573 | 12.8196692 | 16.01871423 | 17.06784774 | 13.95872354 | 14.84859247 | 15.14008085 | 15.66739758 | 15.99771251 | 14.64187523 | 13.33616196 | 12.55672655 | 14.77517329 |
| ENSMUSG00000005034 | Prkacb | 8580 | 16.84354295 | 20.23622432 | 20.93367345 | 18.85101485 | 21.09838752 | 20.58344318 | 17.8883835 | 19.10510386 | 18.53873239 | 17.67876601 | 15.98066793 | 21.18783369 | 16.40947458 | 16.29332203 | 17.53227046 | 16.34714393 | 20.94545527 | 18.297901 |
| ENSMUSG00000005043 | Sgsh | 7753 | 1.946257899 | 1.916361514 | 1.647183286 | 2.045046231 | 1.887282961 | 1.962168091 | 1.482907388 | 1.867008966 | 1.423705175 | 1.489993847 | 1.750043623 | 1.684620798 | 1.775261824 | 2.096663303 | 1.913138156 | 1.555725697 | 1.576949402 | 1.560944599 |
| ENSMUSG00000005045 | Chd5 | 9686 | 0.074601279 | 0.048149961 | 0.00499417 | 0.014111412 | 0.069569165 | 0.020463652 | 0 | 0 | 0 | 0.029207539 | 0.024661862 | 0 | 0.015279347 | 0 | 0 | 0 | 0.055396258 | 0.005679239 |
| ENSMUSG00000005054 | Cstb | 596 | 358.9405217 | 317.5344833 | 353.3053316 | 310.977061 | 369.0652536 | 331.3215492 | 449.7086009 | 360.9694677 | 368.8819147 | 381.7941173 | 376.588515 | 351.6574044 | 445.891004 | 417.139597 | 403.374293 | 418.49835 | 362.3635622 | 402.0464243 |
| ENSMUSG00000005057 | Sh2b2 | 2839 | 0 | 0 | 0.034077866 | 0 | 0 | 0 | 0.116656834 | 0.06726383 | 0 | 0 | 0 | 0 | 0 | 0 | 0 | 0 | 0 | 0 |
| ENSMUSG00000005069 | Pex5 | 5279 | 10.20961606 | 10.64573831 | 12.26977783 | 11.91888657 | 10.284677 | 13.01944336 | 12.08135844 | 11.73481279 | 10.44681656 | 12.94210438 | 12.34420219 | 10.99607674 | 11.45689157 | 12.27306344 | 10.63794671 | 11.79400031 | 13.77975214 | 11.62912732 |
| ENSMUSG00000005078 | Jkamp | 2683 | 29.89462064 | 22.77146776 | 24.97105462 | 22.24560868 | 24.20050877 | 27.77760391 | 23.10086075 | 20.58375471 | 24.6842778 | 24.69129261 | 25.21406679 | 21.88523185 | 23.3512989 | 20.54757053 | 22.37445655 | 24.58990554 | 22.25582206 | 21.95853645 |
| ENSMUSG00000005087 | Cd44 | 8637 | 4.940974497 | 6.136484277 | 5.00145451 | 5.259275351 | 5.678641915 | 6.425733658 | 1.610504528 | 2.794673882 | 2.045735239 | 2.216416061 | 2.290011903 | 2.281223676 | 2.341795309 | 1.516409939 | 1.836586664 | 1.901895401 | 1.593046265 | 1.09546907 |
| ENSMUSG00000005089 | Slc1a2 | 13209 | 0.074011587 | 0.052961676 | 0.117189263 | 0.106926518 | 0.116603949 | 0.101288804 | 0.046564072 | 0.017348354 | 0.031180646 | 0.057113427 | 0.054252735 | 0.076060408 | 0 | 0 | 0 | 0.005797654 | 0.020310703 | 0 |
| ENSMUSG00000005102 | Eif2ak4 | 7651 | 3.999964284 | 3.50066226 | 3.167577925 | 3.566993276 | 2.799464911 | 3.063448305 | 3.104295494 | 3.683961962 | 3.294490609 | 4.048884073 | 3.465573383 | 3.341206831 | 4.197500738 | 3.818236958 | 4.054995633 | 4.35404894 | 4.292986073 | 4.098182046 |
| ENSMUSG00000005103 | Wdr1 | 2933 | 159.8762707 | 174.6172641 | 160.6403633 | 188.7684319 | 177.413647 | 170.14851 | 177.2007426 | 185.5450194 | 183.6752902 | 215.3692368 | 173.3450761 | 161.1860423 | 193.7450921 | 187.6448809 | 204.5046883 | 191.3877615 | 205.3129141 | 202.38775 |
| ENSMUSG00000005107 | Slc2a9 | 7127 | 0.035783788 | 0.009348364 | 0 | 0.038356429 | 0.040520772 | 0.041716908 | 0.139408764 | 0.128611991 | 0.011557883 | 0.019847356 | 0 | 0.031326315 | 0.256107891 | 0.052133113 | 0.017343155 | 0.059098735 | 0.069909058 | 0.239270704 |
| ENSMUSG00000005124 | Wisp1 | 5759 | 0 | 0 | 0.025198922 | 0.015822554 | 0 | 0 | 0.073938897 | 0 | 0 | 0 | 0 | 0.087227116 | 0.145622954 | 0 | 0.07869719 | 0.059839463 | 0.059895237 | 0 |
| ENSMUSG00000005125 | Ndrg1 | 5894 | 27.1661008 | 34.46025043 | 25.25370765 | 30.89709776 | 32.15055709 | 28.77825026 | 13.77478063 | 20.91704699 | 13.01840497 | 16.89553534 | 15.71694002 | 19.88681565 | 10.09404347 | 10.29113983 | 11.66002634 | 15.42278622 | 14.48778241 | 16.23953948 |
| ENSMUSG00000005131 | 4930550C14R | 1351 | 1.038246343 | 0.394527245 | 0.572891551 | 0.370963351 | 0.463149525 | 0.440142711 | 0.665389267 | 0.113078912 | 0 | 0.488608324 | 0.070725327 | 0.619714981 | 0.949392931 | 0.068755126 | 1.372368627 | 0.935299811 | 0.283688356 | 0 |
| ENSMUSG00000005142 | Man2b1 | 4301 | 25.95177685 | 26.74480905 | 21.57182777 | 27.57391267 | 26.51116651 | 25.88816376 | 31.30711096 | 32.59807236 | 24.62955558 | 20.17142385 | 28.84714419 | 27.83645577 | 24.96991023 | 31.76900839 | 29.82107498 | 26.23632179 | 31.30441316 | 26.44939147 |
| ENSMUSG00000005148 | Klf5 | 1717 | 2.376527023 | 4.423610881 | 4.507725601 | 4.325247657 | 6.615686654 | 2.510825755 | 4.133314991 | 6.005794205 | 9.594994936 | 4.366314396 | 7.790907614 | 3.478320266 | 5.889949354 | 8.845204762 | 13.07795455 | 6.110440552 | 11.94209018 | 11.11715785 |
| ENSMUSG00000005150 | Wdr83 | 2322 | 14.16839201 | 15.52306268 | 14.54122489 | 13.75464775 | 13.39070745 | 12.31349151 | 15.44487968 | 12.74724886 | 19.01461882 | 16.04179827 | 14.97853995 | 14.06208554 | 15.04177323 | 14.02123396 | 16.83905105 | 14.06627395 | 14.12890187 | 15.51721141 |
| ENSMUSG00000005161 | Prdx2 | 3195 | 155.3866842 | 134.8676954 | 135.0219542 | 130.9077894 | 144.9983616 | 127.084538 | 161.1888921 | 147.1632757 | 148.9675669 | 148.0930539 | 147.4668671 | 143.8978444 | 150.6669323 | 134.200881 | 136.3326601 | 147.3259583 | 134.4839169 | 135.7924334 |
| ENSMUSG00000005198 | Polr2a | 8097 | 7.344046099 | 7.833487785 | 7.927834356 | 7.208064443 | 7.941737683 | 7.704939061 | 8.507751083 | 9.910119483 | 7.960589992 | 8.298104501 | 8.744286583 | 9.09925585 | 9.492296791 | 8.053291181 | 8.462169624 | 10.28554663 | 9.386321694 | 8.166104271 |
| ENSMUSG00000005202 | Shbg | 1498 | 0 | 0 | 0 | 0 | 0 | 0 | 0 | 0 | 0 | 0 | 0 | 0 | 0.197591124 | 0 | 0 | 0 | 0 | 0 |
| ENSMUSG00000005204 | Senp3 | 4111 | 9.946480014 | 9.667303041 | 8.954574702 | 9.398142933 | 8.944974381 | 9.184914373 | 9.678901235 | 8.324104246 | 9.427514311 | 9.359030372 | 8.134874954 | 9.476850519 | 7.487987444 | 8.06642627 | 7.406458235 | 10.60885474 | 7.654071943 | 8.202525522 |

| ENSMUSG00000005220 | Corin | 7030 | 0.042323788 | 0.094773526 | 0.233954485 | 0.149061738 | 0.089006402 | 0.021146259 | 0.228823767 | 0.266205934 | 0.492129065 | 0.617050455 | 0.903850566 | 0.293766645 | 1.059618707 | 0.905098147 | 1.113555565 | 1.88457328 | 2.273409077 | 0.876389751 |
| --- | --- | --- | --- | --- | --- | --- | --- | --- | --- | --- | --- | --- | --- | --- | --- | --- | --- | --- | --- | --- |
| ENSMUSG00000005225 | Plekha8 | 6990 | 0.826996272 | 1.41544057 | 0.989615857 | 1.323160515 | 1.18436207 | 1.474530556 | 1.286038483 | 2.677292872 | 2.280283491 | 1.774053642 | 1.736028534 | 2.379551853 | 1.764374724 | 2.053107726 | 1.438223124 | 1.654329533 | 1.535245083 | 1.455891952 |
| ENSMUSG00000005232 | G6pc2 | 1968 | 0 | 0 | 0 | 0 | 0 | 0 | 0 | 0 | 0 | 0 | 0 | 0 | 0 | 0 | 0 | 0 | 0 | 0 |
| ENSMUSG00000005233 | Spc25 | 3830 | 2.441550581 | 1.235099472 | 0.618878065 | 0.951666722 | 0.92996406 | 0.996229892 | 3.705953963 | 1.575561248 | 2.408819721 | 1.834322398 | 1.347178986 | 1.544767685 | 0.811464958 | 1.236892145 | 1.065001053 | 0.709825881 | 1.641126029 | 1.838424454 |
| ENSMUSG00000005237 | Dnahc2 | 14507 | 0.049809609 | 0.020666992 | 0 | 0.012562499 | 0.016589206 | 0.013663124 | 0.022829582 | 0 | 0 | 0.039002443 | 0.019759409 | 0.015389995 | 0.085013989 | 0.016007475 | 0 | 0.023755116 | 0.002641917 | 0 |
| ENSMUSG00000005251 | Ripk4 | 3944 | 16.47829973 | 19.1143711 | 17.41643334 | 20.10046074 | 17.29283363 | 21.35893689 | 14.31136561 | 18.04068095 | 16.82339678 | 19.57041106 | 20.82280771 | 18.28444119 | 20.45073143 | 24.01102794 | 21.66633543 | 25.36849826 | 24.39122851 | 34.61779992 |
| ENSMUSG00000005262 | Ufd1l | 4187 | 31.07435964 | 31.94441612 | 31.51731331 | 32.97109251 | 31.98065719 | 29.76477787 | 30.43062917 | 30.35689401 | 31.29073481 | 33.07418569 | 35.38336428 | 29.5941655 | 34.28609488 | 32.88911371 | 32.46332288 | 36.159795 | 33.34671593 | 34.51371468 |
| ENSMUSG00000005267 | Zfp287 | 6994 | 0.540886569 | 0.962139639 | 0.650144674 | 1.009717168 | 0.880881646 | 0.828949075 | 1.68442337 | 0.895561052 | 0.441662665 | 1.301127499 | 0.89484123 | 0.630460015 | 0.726506646 | 1.089052092 | 1.390272689 | 1.478190449 | 1.39736999 | 1.517980783 |
| ENSMUSG00000005268 | Prlr | 15933 | 0.181406638 | 0.146356781 | 0.321822269 | 0.080067109 | 0.111773123 | 0.189714225 | 1.167004508 | 0.872530878 | 0.638490516 | 0.322564906 | 0.197900412 | 0.50795651 | 2.167347147 | 2.066707962 | 0.982651807 | 0.28598395 | 0.654286874 | 1.332675277 |
| ENSMUSG00000005299 | Letm1 | 9714 | 10.12965642 | 9.938312482 | 9.44165262 | 10.84853773 | 10.35079149 | 9.712621447 | 9.497603861 | 10.24990664 | 10.26058968 | 9.707783253 | 11.63635493 | 10.80229006 | 8.6993591 | 11.25960735 | 8.432025233 | 8.537930372 | 9.303418581 | 9.173847323 |
| ENSMUSG00000005312 | Ubqln1 | 3651 | 85.37125604 | 97.01890009 | 100.8013747 | 106.8956192 | 100.0737969 | 107.0860224 | 87.23882622 | 94.38785179 | 73.56265935 | 90.96947514 | 93.52167303 | 87.55308125 | 72.46427619 | 72.33116431 | 77.7085494 | 64.6042568 | 85.28152179 | 82.40065863 |
| ENSMUSG00000005320 | Fgfr4 | 3534 | 0 | 0.037705596 | 0 | 0 | 0 | 0 | 0 | 0 | 0 | 0 | 0 | 0.031587811 | 0.265225362 | 0.052568294 | 0.174879269 | 0 | 0 | 0.373576277 |
| ENSMUSG00000005338 | Cadm3 | 7359 | 0 | 0 | 0 | 0 | 0 | 0 | 0 | 0 | 0 | 0 | 0 | 0.015169361 | 0 | 0 | 0 | 0 | 0 | 0 |
| ENSMUSG00000005339 | Fcer1a | 1021 | 0 | 0 | 0 | 0 | 0 | 0 | 0 | 0 | 0 | 0 | 0 | 0 | 0 | 0 | 0 | 0 | 0 | 0 |
| ENSMUSG00000005354 | Txn2 | 4387 | 69.79878917 | 64.36291119 | 67.7582275 | 63.89139383 | 66.94802786 | 62.93773228 | 79.5914225 | 71.57049012 | 69.83963774 | 78.4376185 | 78.03860325 | 72.07561626 | 76.84848932 | 72.90038426 | 74.07263992 | 74.21587297 | 67.34839818 | 69.64225638 |
| ENSMUSG00000005355 | Casp14 | 2322 | 0.18305416 | 0.157813022 | 0 | 0.117728797 | 0.373115687 | 0.128043239 | 0.163006646 | 0 | 0.248325246 | 0 | 0.308623763 | 0 | 0.276190751 | 0 | 0.283903917 | 0 | 0.759263418 | 0.994996762 |
| ENSMUSG00000005357 | Slc1a6 | 2079 | 0 | 0 | 0 | 0.175319074 | 0.185211827 | 0 | 0 | 0 | 0.059432202 | 0 | 0.091919112 | 0 | 0.213558083 | 0 | 0.277451555 | 0 | 0 | 0.582106949 |
| ENSMUSG00000005360 | Slc1a3 | 9684 | 0.065838253 | 0.003439993 | 0 | 0.037638203 | 0.004970252 | 0.00511697 | 0 | 0 | 0.004253048 | 0.058427142 | 0.009866782 | 0.005763699 | 0.005094167 | 0.004795961 | 0.008509202 | 0.003954007 | 0.039576928 | 0 |
| ENSMUSG00000005362 | Crbn | 5166 | 9.116479876 | 10.22730357 | 10.00993108 | 10.54800794 | 9.643155439 | 10.1772068 | 9.772092221 | 9.366971322 | 9.22431257 | 11.07119687 | 9.691861495 | 9.74559188 | 8.718552163 | 7.291164319 | 10.16081731 | 9.783895039 | 10.19363762 | 9.636709559 |
| ENSMUSG00000005364 | Il5ra | 3774 | 0.011262633 | 0 | 0 | 0 | 0 | 0 | 0.501459236 | 0.091078861 | 0.327396787 | 0.099948497 | 0.316474288 | 0.059158095 | 0 | 0.418415202 | 0.021834423 | 0 | 0.010155352 | 0.495577538 |
| ENSMUSG00000005370 | Msh6 | 4244 | 6.319690404 | 7.362746178 | 6.64509147 | 7.557722717 | 7.734677649 | 7.134005706 | 7.781397241 | 5.768456631 | 5.473420097 | 7.621437897 | 4.874306555 | 6.575832067 | 4.300850469 | 3.687949446 | 3.757069305 | 4.989327496 | 5.192653327 | 6.04011398 |
| ENSMUSG00000005371 | Fbxo11 | 4878 | 11.50201565 | 8.577489778 | 11.61242395 | 8.770360933 | 9.344184443 | 8.563541289 | 8.370406297 | 9.160539322 | 9.76892143 | 7.384816992 | 7.747025862 | 10.59559318 | 8.515267394 | 5.731722142 | 6.4192666 | 9.607975289 | 7.220554911 | 7.882608774 |
| ENSMUSG00000005373 | Mlxipl | 4673 | 1.082413 | 0.64872103 | 0.579695634 | 0.974984899 | 0.525300258 | 0.583222843 | 1.366833224 | 1.683636828 | 1.383754114 | 0.807202281 | 0.817889296 | 0.692768763 | 1.604633303 | 1.451067143 | 1.816289432 | 1.352011603 | 1.377876713 | 1.518547992 |
| ENSMUSG00000005374 | Tbl2 | 6138 | 2.202125444 | 1.845289025 | 2.32489271 | 2.969113349 | 2.619104351 | 2.809441395 | 3.345340934 | 3.185811344 | 2.543123081 | 2.327576261 | 2.202722915 | 2.755318632 | 3.158592944 | 3.246092129 | 2.819265799 | 2.981901326 | 2.734916592 | 2.858896228 |
| ENSMUSG00000005378 | Wbscr22 | 3098 | 18.12438268 | 16.18331369 | 18.23766412 | 18.4273688 | 18.02228259 | 17.33865823 | 17.56280851 | 17.41958186 | 19.42333748 | 19.42036713 | 16.40818458 | 15.92674168 | 20.28691498 | 19.08435224 | 17.86110057 | 22.3959266 | 18.24767202 | 20.24221487 |
| ENSMUSG00000005397 | Nid1 | 6026 | 0 | 0 | 0 | 0.325111999 | 0.087861129 | 0.041115776 | 0.17273132 | 0 | 0.116191631 | 0.062596353 | 0.015856276 | 0.101887204 | 0.008186511 | 0 | 0.205118931 | 0 | 0 | 0 |
| ENSMUSG00000005410 | Mcm5 | 3479 | 2.846710553 | 1.417162502 | 0.903788293 | 1.702482255 | 1.992238289 | 2.435618696 | 1.631940636 | 2.3712443 | 1.681082276 | 2.019389131 | 2.059857365 | 1.411836248 | 2.084447209 | 1.561931079 | 2.877832159 | 1.926086891 | 1.101646936 | 1.296564171 |
| ENSMUSG00000005413 | Hmox1 | 2153 | 11.74666964 | 14.54441271 | 13.45831151 | 13.16254973 | 12.58628566 | 12.06021012 | 14.30587737 | 14.59932521 | 12.41525719 | 12.89910089 | 17.88509825 | 8.658816212 | 11.22742195 | 15.68269927 | 12.9364848 | 11.56009977 | 15.8788002 | 15.45773784 |
| ENSMUSG00000005417 | Mprip | 13343 | 37.95298413 | 40.58815269 | 38.98740504 | 45.42446023 | 42.35666999 | 42.53000861 | 45.74894578 | 48.09328826 | 44.72393217 | 48.41578246 | 48.95295153 | 47.84677724 | 42.37745903 | 39.32936696 | 47.15189676 | 39.23761336 | 46.46952194 | 49.36101587 |
| ENSMUSG00000005442 | Cic | 10261 | 4.54835623 | 4.100398148 | 3.879876765 | 3.849666255 | 4.099727247 | 3.617093602 | 3.804011316 | 4.414403007 | 4.395208533 | 4.066696461 | 3.305742181 | 4.308157564 | 4.913480115 | 3.697965052 | 3.437143733 | 4.063782425 | 3.634293625 | 3.768775171 |
| ENSMUSG00000005447 | Pafah1b3 | 2083 | 50.07570906 | 44.42785412 | 46.07445234 | 47.0702676 | 43.41809149 | 45.72268552 | 47.94866309 | 46.88332835 | 47.65218578 | 46.13208509 | 43.62360587 | 49.97417702 | 54.40010273 | 39.79966202 | 40.66749828 | 44.55901697 | 39.55906785 | 38.1076049 |
| ENSMUSG00000005465 | Il27ra | 2769 | 0 | 0 | 0 | 0 | 0 | 0 | 0.478423622 | 0 | 0 | 0.153252557 | 0.448591159 | 0 | 0 | 0.318684601 | 0 | 0.124454845 | 0 | 0 |
| ENSMUSG00000005469 | Prkaca | 2304 | 55.75114668 | 66.11973319 | 65.31686325 | 61.81589609 | 57.09398758 | 64.65083209 | 60.1675995 | 60.28896161 | 58.02579434 | 61.70111559 | 58.01837406 | 52.52098806 | 58.5174175 | 50.17332184 | 60.63996355 | 62.37180971 | 61.59821069 | 63.34164936 |
| ENSMUSG00000005470 | Asf1b | 2557 | 2.692936455 | 1.667598928 | 2.970138547 | 3.26072394 | 2.352948947 | 2.887507745 | 2.830989392 | 2.718426765 | 4.622811898 | 2.304981583 | 3.587325783 | 1.789943032 | 2.971104912 | 3.033305668 | 4.447254371 | 4.417571053 | 4.106924267 | 2.430985162 |
| ENSMUSG00000005474 | Myl10 | 816 | 0 | 0 | 0 | 0 | 0 | 0 | 0 | 0 | 0 | 0 | 0 | 0 | 0 | 0 | 0 | 0 | 0 | 0 |
| ENSMUSG00000005481 | Ddx39 | 5211 | 19.9434188 | 17.77838396 | 20.44108882 | 17.57392038 | 17.75274559 | 16.63168893 | 13.67355488 | 15.98496059 | 15.5862232 | 16.92032529 | 15.69578371 | 15.3811728 | 13.94471582 | 15.1159417 | 16.07422052 | 15.38678409 | 14.50382988 | 13.70213405 |
| ENSMUSG00000005483 | Dnajb1 | 2467 | 54.46244889 | 55.70153573 | 56.25604321 | 56.62349491 | 57.75050428 | 58.77231392 | 60.60318825 | 66.75542731 | 59.81811349 | 69.39773147 | 58.25175318 | 57.28628199 | 95.82429979 | 153.1883012 | 100.0060465 | 94.30633586 | 98.61991599 | 168.6618903 |
| ENSMUSG00000005493 | Msh4 | 4441 | 0.114852986 | 0 | 0 | 0.020518372 | 0 | 0.033474038 | 0 | 0 | 0.148386456 | 0 | 0 | 0 | 0.055541452 | 0 | 0 | 0 | 0 | 0 |
| ENSMUSG00000005501 | Usp40 | 5548 | 9.637979712 | 10.12956967 | 11.29994509 | 10.68401562 | 12.48410926 | 10.44108608 | 9.235694174 | 9.616940548 | 11.56607634 | 9.86697305 | 11.27206573 | 12.3442354 | 7.913735828 | 10.12092676 | 10.20384603 | 7.446929594 | 9.878623751 | 11.77916705 |
| ENSMUSG00000005503 | Evx1 | 3538 | 0 | 0.112988901 | 0 | 0 | 0 | 0 | 0 | 0 | 0 | 0 | 0 | 0 | 0 | 0 | 0 | 0 | 0 | 0 |
| ENSMUSG00000005505 | Kbtbd4 | 2016 | 17.77374176 | 18.4906392 | 16.67639067 | 18.1701784 | 17.57197213 | 20.17995747 | 18.30550078 | 18.88781012 | 20.0825124 | 21.09619759 | 18.69764 | 16.41799996 | 22.85516406 | 16.51806084 | 16.77900664 | 18.63248307 | 16.93885443 | 21.69257929 |

| ENSMUSG00000005506 | Celf1 | 8674 | 17.10693677 | 20.05148959 | 19.90375027 | 22.37081944 | 19.10516638 | 20.06331569 | 17.50361848 | 16.24740199 | 14.41100707 | 16.17170189 | 17.79581402 | 18.13333378 | 16.16908471 | 13.54663836 | 15.48977101 | 15.57404461 | 15.73879059 | 14.78916727 |
| --- | --- | --- | --- | --- | --- | --- | --- | --- | --- | --- | --- | --- | --- | --- | --- | --- | --- | --- | --- | --- |
| ENSMUSG00000005510 | Ndufs3 | 2411 | 57.34937272 | 55.93139602 | 55.1550539 | 52.0049747 | 56.51658063 | 50.92976931 | 63.3452209 | 60.97161213 | 59.0379919 | 59.15838961 | 61.42777739 | 51.25502985 | 68.07436283 | 67.90352885 | 62.52861593 | 68.57687381 | 61.5191908 | 61.488819 |
| ENSMUSG00000005514 | Por | 4002 | 74.96290165 | 88.31829277 | 72.78995497 | 81.3313595 | 85.11484852 | 77.26363257 | 54.55972345 | 60.50470546 | 56.51053425 | 75.02640638 | 81.73791622 | 58.87004292 | 60.6602611 | 60.41677135 | 65.94102086 | 59.41645976 | 66.08939903 | 71.21493801 |
| ENSMUSG00000005533 | Igf1r | 4454 | 4.666598805 | 7.748576204 | 7.157197239 | 7.457117495 | 6.7540306 | 6.653016318 | 3.516052251 | 5.042014507 | 5.335567938 | 4.139183874 | 4.086721864 | 4.824658755 | 3.533201978 | 3.660052688 | 3.894444326 | 2.579070997 | 3.03753543 | 2.581253549 |
| ENSMUSG00000005534 | Insr | 9647 | 13.43845619 | 11.70975685 | 10.70565847 | 10.08322065 | 10.69709177 | 9.014724518 | 9.84311668 | 10.23795508 | 12.43237627 | 10.17598882 | 8.844830086 | 10.91781438 | 11.54674709 | 9.465022889 | 9.237997864 | 11.06605301 | 10.13479666 | 8.028695166 |
| ENSMUSG00000005540 | Fcer2a | 2970 | 0.028623014 | 0.022432925 | 0.048862152 | 0.046021257 | 0 | 0.250266331 | 0 | 0 | 0.249615247 | 0 | 0.144772601 | 0 | 0.066440293 | 0 | 0.291324133 | 0 | 0 | 0 |
| ENSMUSG00000005547 | Cyp2a5 | 2148 | 0 | 0 | 0 | 0 | 0 | 0 | 0 | 0 | 0 | 0 | 0 | 0 | 0 | 0 | 0 | 0 | 0 | 0 |
| ENSMUSG00000005553 | Atp4a | 3771 | 5.038401932 | 4.10779523 | 4.002265041 | 3.914552735 | 3.624891626 | 5.059083115 | 2.45910503 | 2.420573899 | 1.845802483 | 1.712979673 | 1.292242314 | 2.486616631 | 0.261638384 | 1.170031372 | 0.830368138 | 0.406158657 | 0.355720072 | 0.525146604 |
| ENSMUSG00000005566 | Trim28 | 3829 | 43.04911795 | 41.5867418 | 43.20644393 | 38.07661057 | 39.7474909 | 39.95019288 | 33.72063223 | 37.74354941 | 36.27080991 | 37.36099078 | 35.63470395 | 38.57096976 | 37.76230074 | 35.78220366 | 37.93039554 | 33.97053887 | 33.3515856 | 35.097218 |
| ENSMUSG00000005575 | Ube2m | 2676 | 86.82111068 | 83.91712255 | 82.88215123 | 87.41046398 | 81.11919871 | 85.60623592 | 93.44077285 | 90.07184289 | 91.33063697 | 93.05039757 | 82.05295559 | 80.36537666 | 105.7981589 | 95.12707137 | 88.02290547 | 80.30152793 | 79.15898467 | 87.50888166 |
| ENSMUSG00000005580 | Adcy9 | 6730 | 0.517893675 | 0.460341629 | 0.625333899 | 0.500968392 | 0.772399369 | 0.596399914 | 0.780342847 | 0.595869577 | 0.471227595 | 0.609526179 | 0.425928307 | 0.563962118 | 0.542431186 | 1.035157966 | 1.040752526 | 0.807914748 | 0.671991536 | 0.989019601 |
| ENSMUSG00000005583 | Mef2c | 7382 | 0 | 0 | 0 | 0 | 0 | 0.073839077 | 0.455052859 | 0.093126963 | 0.820159553 | 0.083034292 | 0.401252699 | 0.060488391 | 0.387598375 | 0.270535865 | 0.24557958 | 0.181545822 | 0 | 0.022355367 |
| ENSMUSG00000005609 | Ctr9 | 5287 | 23.12982626 | 24.51052743 | 26.23168366 | 25.39595189 | 23.47876511 | 25.20281835 | 25.0299911 | 22.92122051 | 20.39460909 | 25.28319344 | 29.91921459 | 29.06383944 | 22.21662475 | 26.88960687 | 25.24929848 | 27.10108812 | 28.32245924 | 27.46813727 |
| ENSMUSG00000005610 | Eif4g2 | 12073 | 84.04568559 | 99.26265689 | 99.07899102 | 96.1676545 | 97.51967074 | 102.0483405 | 87.82217633 | 94.69805069 | 88.66034607 | 90.73571513 | 88.63277727 | 88.94075349 | 70.70649354 | 79.51236994 | 84.2869235 | 75.67096844 | 90.61742277 | 80.34710411 |
| ENSMUSG00000005611 | Mrvi1 | 9724 | 0 | 0 | 0 | 0 | 0 | 0 | 0 | 0.129612225 | 0 | 0 | 0.004913097 | 0 | 0 | 0 | 0 | 0 | 0 | 0 |
| ENSMUSG00000005615 | Pcyt1a | 5849 | 7.848451036 | 7.250352939 | 6.748640925 | 6.987221195 | 6.056607252 | 7.040232793 | 8.671429614 | 7.078229476 | 6.99938394 | 6.715085624 | 6.575284933 | 8.340381108 | 7.489612334 | 5.780696806 | 6.544066579 | 6.337033333 | 8.158016694 | 6.451743411 |
| ENSMUSG00000005621 | Zfp592 | 5829 | 7.459734959 | 7.72671693 | 8.356861394 | 8.324328739 | 6.7875178 | 7.370427177 | 8.157358436 | 7.056650779 | 5.81514882 | 8.202232476 | 7.163375143 | 7.602956956 | 7.718426584 | 7.425954614 | 8.694100831 | 7.666004261 | 8.98159574 | 7.276037276 |
| ENSMUSG00000005625 | Psmd4 | 2517 | 92.0692172 | 91.85200075 | 80.87239401 | 82.5601602 | 89.89597662 | 84.55661133 | 82.57632043 | 77.59870715 | 77.03858411 | 81.37570717 | 80.91563289 | 84.31114388 | 81.18108914 | 87.4447875 | 82.74686731 | 77.7221745 | 72.06927423 | 90.30497774 |
| ENSMUSG00000005628 | Tmod4 | 1770 | 0.480284475 | 0.244670974 | 0.081989035 | 0.25740703 | 0.054386354 | 0.335950736 | 0 | 0.28050917 | 0.744614974 | 0.106555262 | 0.782753557 | 0.126137091 | 0.891876471 | 0.760948324 | 0.58194288 | 0.757158903 | 0.281492576 | 1.025593515 |
| ENSMUSG00000005640 | Insrr | 6185 | 10.29470553 | 16.79379211 | 12.54505136 | 13.78249215 | 14.7625318 | 14.78970836 | 10.64822135 | 9.651531925 | 8.117439404 | 13.04363028 | 8.782559061 | 5.802663063 | 4.163502153 | 4.50548948 | 4.443239738 | 2.148236179 | 3.296619232 | 2.632610443 |
| ENSMUSG00000005649 | Cabp5 | 2595 | 0 | 0 | 0 | 0 | 0 | 0 | 0.036464492 | 0.11774151 | 0 | 0 | 0 | 0.064526778 | 0 | 0 | 0 | 0 | 0 | 0 |
| ENSMUSG00000005656 | Snx6 | 1914 | 74.52840687 | 83.09078213 | 70.43731928 | 80.07698701 | 66.46430212 | 65.94086334 | 59.37568171 | 70.29876367 | 73.24916387 | 76.95861911 | 68.46745617 | 63.33940394 | 68.01824958 | 70.0058475 | 68.06652023 | 73.2002782 | 73.02821568 | 55.69887609 |
| ENSMUSG00000005667 | Mthfd2 | 2965 | 19.3674512 | 16.54971102 | 18.58261419 | 16.54949233 | 18.81446864 | 15.81007958 | 20.10589391 | 18.17520398 | 20.58631243 | 18.24008656 | 18.85217586 | 16.84823542 | 22.07873667 | 25.37585888 | 14.85479368 | 14.41224886 | 15.82171582 | 22.74575543 |
| ENSMUSG00000005672 | Kit | 5973 | 68.12356439 | 81.63973641 | 83.95111597 | 90.61865172 | 87.76239422 | 94.55089647 | 66.69558958 | 88.73834934 | 82.69711762 | 84.93915403 | 84.44001953 | 86.69042388 | 56.79819104 | 63.18510868 | 85.72103383 | 78.90185768 | 95.35695601 | 77.55427553 |
| ENSMUSG00000005674 | Tomm40l | 4632 | 2.128929369 | 1.438380579 | 1.83802706 | 1.396733224 | 1.433982182 | 1.273051661 | 1.532146338 | 1.236800596 | 1.627187475 | 1.028113348 | 1.217065004 | 1.626751824 | 1.086324603 | 1.534098741 | 1.005132948 | 1.157315422 | 1.075651683 | 0.950070928 |
| ENSMUSG00000005677 | Nr1i3 | 3574 | 0.059464432 | 0.027962698 | 0.148883278 | 0 | 0.040401727 | 0.013864783 | 0 | 0.096175608 | 0.092191417 | 0 | 0.080204183 | 0.015617141 | 0.013802999 | 0.311879714 | 0 | 0.010713656 | 0.107236421 | 0.046174404 |
| ENSMUSG00000005681 | Apoa2 | 572 | 0 | 0.174717977 | 0 | 0.159304351 | 0.252440159 | 0 | 0 | 0 | 0 | 0.412156497 | 0 | 0.09757983 | 0.517467664 | 0.324783828 | 0.504214845 | 0.535532971 | 0 | 0 |
| ENSMUSG00000005682 | Pan2 | 4418 | 8.543367205 | 10.4658889 | 8.211894017 | 10.87978764 | 10.13188977 | 8.535452751 | 8.834984182 | 10.13161965 | 10.89792597 | 8.644651344 | 11.73287233 | 9.551073091 | 10.02717558 | 11.97370202 | 8.812917682 | 8.450281172 | 8.310681854 | 12.88692292 |
| ENSMUSG00000005683 | Cs | 2817 | 95.70831791 | 105.5203959 | 105.1441698 | 112.9726286 | 109.8644903 | 104.1187895 | 92.81145323 | 100.219467 | 94.02573052 | 110.6376104 | 99.58628193 | 99.7033774 | 84.42640156 | 84.38084461 | 99.61796137 | 85.5931682 | 94.02663752 | 80.55114137 |
| ENSMUSG00000005686 | Ampd3 | 5623 | 7.377743518 | 5.2490173 | 5.376748439 | 6.765689493 | 7.301534926 | 5.41969254 | 5.570157114 | 4.693393217 | 6.606835714 | 6.062592647 | 6.100377056 | 4.744778004 | 3.851451482 | 2.395302391 | 3.634353854 | 3.615918999 | 2.446939461 | 3.19900016 |
| ENSMUSG00000005687 | Bcas2 | 4548 | 38.60793363 | 39.56089307 | 40.3112753 | 36.75538075 | 37.25250029 | 38.79887517 | 41.25815405 | 38.96498398 | 40.32619936 | 39.89355896 | 39.69691471 | 39.55450319 | 40.25302221 | 35.83383963 | 38.60154566 | 41.96980605 | 39.43024257 | 42.79292427 |
| ENSMUSG00000005696 | Sh2d1a | 2724 | 0 | 0 | 0 | 0 | 0 | 0 | 0 | 0 | 0 | 0 | 0 | 0 | 0 | 0 | 0 | 0 | 0 | 0 |
| ENSMUSG00000005698 | Ctcf | 4628 | 21.19748191 | 19.50690219 | 16.41020801 | 18.42918647 | 16.78585238 | 15.96437463 | 16.21389559 | 16.81026873 | 20.1126892 | 15.86293107 | 17.64204927 | 17.45143988 | 15.75466157 | 17.38140868 | 15.97138964 | 16.50599865 | 16.07418805 | 17.23491892 |
| ENSMUSG00000005699 | Pard6a | 1424 | 9.283082686 | 8.234648009 | 6.08066147 | 8.158754428 | 5.61088436 | 7.34243454 | 8.53887534 | 7.456101031 | 10.81724501 | 8.940091208 | 13.48703182 | 12.11168521 | 10.2197441 | 10.37164311 | 6.915148777 | 9.007972949 | 5.3290778 | 8.923527846 |
| ENSMUSG00000005705 | Agrp | 654 | 0.324963119 | 0.814994354 | 0.961553356 | 0.278660822 | 0.220788641 | 1.288067998 | 0.289374183 | 0.525583519 | 0.377857943 | 0.648862889 | 1.899310276 | 0.938795548 | 1.659483456 | 0 | 0.062999321 | 0.93677327 | 0.527426104 | 0 |
| ENSMUSG00000005716 | Pvalb | 1613 | 0 | 0 | 0 | 0 | 0 | 0 | 0 | 0 | 0 | 0 | 0 | 0 | 0 | 0 | 0 | 0 | 0 | 0 |
| ENSMUSG00000005718 | Tfap4 | 2106 | 0.181646051 | 0.775086333 | 0.528295916 | 0.41104456 | 0.617075944 | 0.423527637 | 0.673969785 | 0.380835921 | 0.488918753 | 0.649273692 | 0.839351122 | 0.477056945 | 0.304518008 | 0.441064458 | 0.489097292 | 0.327270149 | 0.254780701 | 0.522403673 |
| ENSMUSG00000005732 | Ranbp1 | 2049 | 97.62291771 | 88.10276905 | 93.79601567 | 84.91831543 | 87.00861165 | 86.674962 | 94.78699219 | 86.52471057 | 87.61933734 | 84.29137434 | 83.82185248 | 82.37509207 | 86.21649379 | 91.55083893 | 85.80138555 | 89.19525092 | 79.2338671 | 85.8292407 |
| ENSMUSG00000005763 | Cd247 | 5557 | 0.045893658 | 0.035968574 | 0.087049722 | 0.06559085 | 0.025984483 | 0.062420215 | 0.008514069 | 0 | 0.014823292 | 0.067879364 | 0.060180801 | 0 | 0.026632311 | 0.016715525 | 0.022243057 | 0 | 0.048278582 | 0 |
| ENSMUSG00000005774 | Rfx5 | 5528 | 3.544661025 | 4.549789279 | 4.777848693 | 5.225344086 | 5.467953702 | 5.145309167 | 3.911341221 | 5.637662873 | 3.971131133 | 3.829715225 | 5.859519148 | 5.351356941 | 3.757007448 | 5.116579092 | 3.503026641 | 3.830446073 | 5.754491033 | 4.816282137 |
| ENSMUSG00000005779 | Psmb4 | 1495 | 125.667477 | 114.199721 | 119.8822274 | 112.5159704 | 118.9937056 | 109.9772375 | 106.524734 | 113.8874448 | 108.4073174 | 108.5570038 | 112.2631633 | 112.5273626 | 122.9503169 | 108.825176 | 108.7776195 | 123.0421926 | 108.1596299 | 112.0419598 |

| ENSMUSG00000005800 | Mmp8 | 2443 | 0 | 0 | 0 | 0 | 0 | 0.040567117 | 0 | 0 | 0.13487193 | 0.038600658 | 0 | 0.182777446 | 0.020193171 | 0.133077614 | 0.033730295 | 0 | 0.12550568 | 0 |
| --- | --- | --- | --- | --- | --- | --- | --- | --- | --- | --- | --- | --- | --- | --- | --- | --- | --- | --- | --- | --- |
| ENSMUSG00000005802 | Slc30a4 | 7410 | 1.428311583 | 3.133480064 | 1.945386241 | 1.979845651 | 2.435826097 | 2.353922027 | 2.969014265 | 2.757481145 | 3.501687575 | 2.83795056 | 2.939997445 | 4.647538976 | 3.082412645 | 2.833026653 | 3.536327874 | 2.614716241 | 3.356783629 | 2.642812685 |
| ENSMUSG00000005803 | Sqrdl | 3886 | 1.968845004 | 2.280295899 | 1.767638011 | 1.899353881 | 1.981757022 | 2.244282324 | 3.981277923 | 2.162204974 | 3.285594412 | 3.045503479 | 3.774295485 | 1.68050245 | 2.665903919 | 2.617409969 | 2.671845621 | 2.680145449 | 2.130334568 | 3.029322913 |
| ENSMUSG00000005804 | Pldn | 4082 | 13.96360633 | 14.75495168 | 14.09018314 | 16.82030716 | 15.82387103 | 16.30311641 | 15.45022073 | 17.30914857 | 14.75127047 | 12.72907276 | 17.36845624 | 15.32811312 | 17.52358649 | 15.37137728 | 16.3514259 | 15.74961534 | 17.81111838 | 18.74514146 |
| ENSMUSG00000005813 | Metap1 | 2538 | 28.23629897 | 28.77142007 | 28.05588522 | 29.31488786 | 31.06386563 | 32.95705842 | 29.39798844 | 25.26597464 | 28.85328015 | 28.88863025 | 24.86632 | 30.10702998 | 29.1754168 | 30.48693837 | 27.61380874 | 21.15186429 | 32.2708024 | 23.25641116 |
| ENSMUSG00000005823 | Gpr108 | 2092 | 57.23569831 | 50.68591888 | 50.45460958 | 63.13645675 | 51.79013393 | 49.64748068 | 47.90069501 | 50.05906663 | 57.68474722 | 47.98463061 | 63.78368023 | 51.89362511 | 59.25961191 | 52.61591162 | 51.0686589 | 52.69534355 | 53.05590623 | 64.79084081 |
| ENSMUSG00000005824 | Tnfsf14 | 1855 | 0 | 0 | 0 | 0 | 0 | 0 | 0 | 0 | 0 | 0 | 0 | 0 | 0 | 0 | 0 | 0 | 0 | 0 |
| ENSMUSG00000005836 | Gata6 | 3187 | 0 | 0.08362195 | 0 | 0 | 0 | 0 | 0 | 0 | 0 | 0 | 0 | 0.140108347 | 0 | 0 | 0 | 0 | 0 | 0 |
| ENSMUSG00000005846 | Rsl1d1 | 3011 | 44.77795362 | 38.88901467 | 41.72237075 | 38.05580422 | 37.03808274 | 38.32889954 | 46.5584416 | 40.67852358 | 43.42981984 | 40.83993162 | 38.01687164 | 39.94777578 | 41.2546555 | 43.45167525 | 43.36357709 | 39.82935317 | 41.85216347 | 44.50421255 |
| ENSMUSG00000005864 | Cnga2 | 3423 | 0 | 0.048660377 | 0.056527643 | 0.013310267 | 0 | 0.347433715 | 0.082931953 | 0.066945491 | 0 | 0.110197378 | 0.209355646 | 0.048918197 | 0 | 0.25779657 | 0 | 0 | 0 | 0.016070437 |
| ENSMUSG00000005871 | Apc | 15806 | 6.782111473 | 5.734808628 | 5.915856901 | 5.940865012 | 5.791909327 | 6.477030721 | 5.217385763 | 5.054947857 | 5.232349972 | 5.972143991 | 5.422515216 | 5.519415451 | 6.872635825 | 4.827763861 | 5.043971318 | 5.770481262 | 5.436388565 | 6.114829844 |
| ENSMUSG00000005873 | Reep5 | 2889 | 102.3861267 | 99.22376419 | 102.6409626 | 101.8460451 | 104.6940147 | 108.2818993 | 99.9805832 | 91.58773412 | 91.86774232 | 109.0880237 | 89.7950933 | 97.3344784 | 97.94665195 | 75.30083258 | 94.08330364 | 99.61654733 | 93.12931958 | 92.46252068 |
| ENSMUSG00000005881 | Ergic3 | 2230 | 101.8789981 | 91.03532618 | 95.22860908 | 92.26622252 | 91.34273586 | 97.127802 | 86.88135435 | 98.49529427 | 93.65776746 | 93.01163391 | 100.2845653 | 100.543281 | 102.4245637 | 91.82600058 | 90.92056347 | 94.43871781 | 87.80674927 | 100.0771058 |
| ENSMUSG00000005882 | Uqcc | 6351 | 9.958699721 | 11.27214764 | 9.993083267 | 11.95878774 | 12.00456103 | 11.78155058 | 12.41110425 | 12.26775325 | 11.42016285 | 12.3017974 | 13.53285314 | 12.2247814 | 8.761833205 | 11.40077661 | 12.19633523 | 12.13048372 | 11.11589339 | 11.68434655 |
| ENSMUSG00000005883 | Spo11 | 3020 | 1.548201776 | 2.779751438 | 3.94036042 | 1.689681114 | 1.466270522 | 2.772984098 | 1.190650198 | 1.327881541 | 2.318446252 | 0.733802335 | 0.838434702 | 1.792754725 | 0.245026079 | 0.276819064 | 0.818573958 | 0.355012254 | 0.431488111 | 0.983606544 |
| ENSMUSG00000005886 | Ncoa2 | 17932 | 4.456264272 | 3.693176093 | 3.204759874 | 3.643460717 | 4.042308557 | 3.443157821 | 3.429984531 | 3.678244421 | 3.764482452 | 3.399836009 | 3.711271312 | 4.348342663 | 3.694666791 | 3.519825717 | 2.963975413 | 3.482711391 | 4.674303554 | 3.273182962 |
| ENSMUSG00000005891 | Prl4a1 | 860 | 0 | 0 | 0 | 0 | 0 | 0 | 0 | 0 | 0 | 0 | 0 | 0 | 0 | 0 | 0 | 0 | 0 | 0 |
| ENSMUSG00000005892 | Trh | 1381 | 0 | 0 | 0 | 0 | 0 | 0 | 0 | 0 | 0 | 0 | 0 | 0 | 0 | 0 | 0 | 0 | 0 | 0 |
| ENSMUSG00000005893 | Nr2c2 | 9134 | 5.919266905 | 5.029393598 | 6.291630618 | 5.147689704 | 5.654209995 | 6.097796425 | 3.170063445 | 5.598820541 | 3.963537043 | 3.685745799 | 5.099691758 | 7.375684776 | 4.110092955 | 3.508476056 | 4.001070733 | 4.414277381 | 4.980656276 | 3.607450726 |
| ENSMUSG00000005897 | Nr2c1 | 4591 | 7.036361094 | 7.321435475 | 8.313377358 | 8.107900941 | 8.324275189 | 6.778287233 | 6.502788152 | 6.96297992 | 5.239147289 | 5.145393668 | 5.962764367 | 6.431384341 | 4.018762157 | 6.272126809 | 6.712865129 | 4.929154645 | 5.710125698 | 5.823224977 |
| ENSMUSG00000005899 | Smpd4 | 5463 | 7.072525171 | 7.597998572 | 7.074949799 | 8.114753091 | 7.462518643 | 8.825701953 | 7.794510532 | 7.25676619 | 7.855820872 | 9.071094476 | 8.640245099 | 6.743243146 | 6.447554262 | 8.238023189 | 7.33075459 | 7.85717937 | 8.594126617 | 10.46210175 |
| ENSMUSG00000005907 | Pex1 | 7883 | 6.680694291 | 6.398036515 | 6.455531388 | 7.571352033 | 6.869011052 | 7.166068284 | 4.147286711 | 5.692765803 | 5.308321707 | 6.023183836 | 5.351425642 | 6.287493134 | 6.270529127 | 7.995005276 | 7.238888102 | 6.756594557 | 7.526209261 | 6.259440407 |
| ENSMUSG00000005917 | Otx1 | 6170 | 0 | 0 | 0.086241302 | 0 | 0.03120384 | 0 | 0 | 0 | 0 | 0.007641929 | 0 | 0 | 0 | 0 | 0 | 0 | 0 | 0 |
| ENSMUSG00000005936 | Kctd20 | 4368 | 16.28002736 | 17.09116208 | 17.67494378 | 18.02414941 | 17.77399104 | 18.01505058 | 12.69470231 | 14.89923391 | 12.49362028 | 17.3037036 | 14.53592487 | 13.54500969 | 10.89864931 | 14.15226198 | 15.22420128 | 15.28819123 | 16.61859119 | 16.14507207 |
| ENSMUSG00000005947 | Itgae | 4550 | 0.140126954 | 0.051250606 | 0.244525538 | 0.130174412 | 0.179833561 | 0.076234975 | 0.051991955 | 0.016787869 | 0 | 0 | 0 | 0 | 0 | 0.132697393 | 0 | 0.008415518 | 0 | 0 |
| ENSMUSG00000005949 | Ctns | 4819 | 8.202908011 | 8.924454542 | 10.31915111 | 7.781020853 | 8.389876713 | 9.809775438 | 6.656566988 | 5.349630966 | 7.657837336 | 6.526150461 | 6.553070669 | 4.92252679 | 8.220280052 | 7.440306176 | 7.746131902 | 9.082001299 | 6.267093164 | 8.915150934 |
| ENSMUSG00000005950 | P2rx5 | 2530 | 0 | 0 | 0 | 0 | 0.019024476 | 0 | 0.093503318 | 0.030191622 | 0 | 0.093183208 | 0 | 0.220615267 | 0 | 0 | 0 | 0 | 0 | 0.08697092 |
| ENSMUSG00000005951 | Shpk | 2977 | 3.926410281 | 4.520795856 | 3.607297191 | 2.938434838 | 4.575523815 | 3.911619883 | 1.986592162 | 3.309922681 | 4.496344516 | 4.466408572 | 3.065172009 | 5.868425391 | 4.407890492 | 6.505604448 | 6.020381865 | 6.636867121 | 4.814926114 | 3.751040868 |
| ENSMUSG00000005952 | Trpv1 | 3636 | 0 | 0 | 0 | 0 | 0.092663219 | 0 | 0 | 0 | 0 | 0 | 0 | 0 | 0 | 0 | 0 | 0 | 0 | 0 |
| ENSMUSG00000005958 | Ephb3 | 6757 | 1.063101191 | 1.838938811 | 1.145443963 | 0.903534104 | 1.595612091 | 1.217367733 | 0.406117416 | 0.480443126 | 0.451058483 | 1.095554301 | 0.721184811 | 0.148687572 | 0.445352516 | 0.19245737 | 0.469516028 | 0.612015036 | 0.436750756 | 0.203526368 |
| ENSMUSG00000005968 | Tuft1 | 4536 | 4.741538596 | 5.772472411 | 4.788296986 | 5.68508622 | 7.915876335 | 6.128545753 | 4.860605904 | 5.750751949 | 6.265144593 | 5.769100597 | 6.298374156 | 5.229642107 | 4.948417628 | 5.785032931 | 6.094850971 | 6.364884916 | 5.323096792 | 6.209140794 |
| ENSMUSG00000005973 | Rcn1 | 4287 | 158.1624838 | 165.1113544 | 172.4950218 | 170.7662376 | 160.4734278 | 173.8449062 | 137.9649637 | 145.6514082 | 145.992604 | 144.4987029 | 138.6554778 | 146.8627651 | 130.8268177 | 124.533423 | 133.0617078 | 129.689671 | 122.0952267 | 132.794319 |
| ENSMUSG00000005980 | Dnase1 | 2259 | 0.771452951 | 0.516136033 | 0.535342301 | 0.907590525 | 0.617895434 | 1.360013051 | 0.733042834 | 0.456482897 | 0.109393136 | 0.730533251 | 0.12689232 | 0.518870701 | 0 | 0.226155361 | 0.98489775 | 1.220417766 | 0.339320911 | 0.706181538 |
| ENSMUSG00000005981 | Trap1 | 2787 | 42.61193463 | 42.93502549 | 41.70850141 | 41.60490055 | 39.94586991 | 34.72425141 | 43.00072326 | 46.78466516 | 41.82197331 | 47.03227666 | 44.00370234 | 44.92089386 | 39.64962135 | 33.59572306 | 43.99563342 | 38.73025557 | 36.90986038 | 32.646237 |
| ENSMUSG00000005982 | Nat15 | 3756 | 24.27412209 | 22.99795919 | 25.37163333 | 25.76455223 | 22.68197681 | 24.38057818 | 29.13584301 | 24.08887149 | 24.95753724 | 28.84779449 | 29.17884844 | 23.79150048 | 29.73574566 | 29.19448624 | 27.68709453 | 26.49555076 | 27.16309967 | 25.73242825 |
| ENSMUSG00000005983 | 1700037C18R | 3362 | 2.895206815 | 2.14026923 | 2.762557355 | 1.788833389 | 2.276316438 | 2.667770606 | 5.009908893 | 3.82832826 | 3.72418227 | 3.099436472 | 2.927317503 | 2.573297948 | 3.448245259 | 3.080616147 | 3.088278431 | 3.040925692 | 2.667564984 | 2.977887395 |
| ENSMUSG00000005986 | Ankrd13d | 2653 | 12.91337048 | 12.97105908 | 12.28939294 | 13.91045832 | 12.24615472 | 11.5616819 | 17.49481268 | 15.79233526 | 13.16478152 | 16.75956019 | 21.1952982 | 15.40032605 | 12.7374155 | 14.3901394 | 12.78133453 | 11.21439953 | 12.68393844 | 13.00064452 |
| ENSMUSG00000005994 | Tyrp1 | 2251 | 0 | 0 | 0 | 0 | 0 | 0 | 0 | 0 | 0 | 0 | 0 | 0 | 0 | 0 | 0 | 0 | 0 | 0 |
| ENSMUSG00000006005 | Tpr | 9887 | 21.71044189 | 19.62654079 | 21.16066742 | 18.1009186 | 19.98397357 | 18.38367824 | 18.9786674 | 19.44965572 | 23.65298235 | 16.60551722 | 20.03388064 | 18.4772594 | 19.4892757 | 19.01544106 | 19.12765667 | 20.22001226 | 20.39391605 | 20.7918511 |
| ENSMUSG00000006007 | Pdc | 1313 | 0 | 0 | 0 | 0 | 0 | 0 | 0 | 0 | 0 | 0 | 0 | 0 | 0 | 0 | 0 | 0 | 0 | 0 |
| ENSMUSG00000006010 | BC003331 | 5645 | 8.907639192 | 9.377181384 | 7.21532552 | 9.265558706 | 8.449731861 | 9.638423644 | 12.50496315 | 10.04707131 | 11.56432728 | 9.739188662 | 9.48728582 | 12.03324382 | 10.64415859 | 11.7570595 | 9.386218047 | 9.83549703 | 11.75928188 | 11.90808298 |

| ENSMUSG00000006014 | Prg4 | 4527 | 0.122060369 | 0.198685254 | 0.07479892 | 0.120771489 | 0.127586279 | 0.032838127 | 0.177670762 | 0.202477945 | 0.109175655 | 0.062492648 | 0.295493448 | 0.283578582 | 0.305123411 | 0.123112226 | 0.373153035 | 0.169165484 | 0.152390843 | 0.194421407 |
| --- | --- | --- | --- | --- | --- | --- | --- | --- | --- | --- | --- | --- | --- | --- | --- | --- | --- | --- | --- | --- |
| ENSMUSG00000006019 | Dhx34 | 4778 | 3.558407369 | 6.386482023 | 4.191427703 | 4.443584483 | 4.281303382 | 3.329097421 | 5.41649966 | 5.819185632 | 6.327103928 | 5.279536677 | 4.999472423 | 5.595584424 | 4.460315668 | 6.823723179 | 5.889632205 | 3.798607766 | 5.18183085 | 5.641369254 |
| ENSMUSG00000006021 | Kptn | 2933 | 10.36181414 | 6.735269782 | 9.978174519 | 8.497064866 | 6.662652918 | 7.822337418 | 7.75908577 | 10.88607174 | 10.72843438 | 11.52644195 | 9.919860114 | 8.90614732 | 8.443444416 | 6.777384726 | 6.419744644 | 7.310855828 | 8.911876743 | 7.539604808 |
| ENSMUSG00000006024 | Napa | 3943 | 98.67927501 | 104.2981687 | 108.5858781 | 104.1560369 | 113.2803073 | 108.6188882 | 122.7513836 | 119.9530082 | 125.1785962 | 126.0022724 | 125.4410716 | 119.4027679 | 122.9607109 | 128.2719025 | 120.8042576 | 124.6119894 | 128.7230915 | 135.0045969 |
| ENSMUSG00000006050 | Sra1 | 2217 | 57.05701572 | 51.49449188 | 52.75922255 | 50.14386476 | 55.77397921 | 50.42443255 | 60.65071426 | 51.06101972 | 54.24656113 | 52.34004543 | 60.46753879 | 49.97478134 | 55.3398639 | 58.34315894 | 54.82390161 | 57.7036172 | 55.87306793 | 59.37609039 |
| ENSMUSG00000006056 | Calcoco2 | 2535 | 0 | 0 | 0 | 0 | 0 | 0 | 0 | 0 | 0 | 0 | 0 | 0 | 0 | 0 | 0 | 0 | 0 | 0 |
| ENSMUSG00000006057 | Atp5g1 | 5133 | 33.76896704 | 30.58711678 | 29.7893492 | 27.46266729 | 31.43156208 | 30.05214487 | 39.91114353 | 31.79351948 | 30.01729115 | 34.58453103 | 32.36188012 | 28.73968366 | 36.22287088 | 36.32827019 | 26.76134567 | 32.48696576 | 27.11886038 | 27.54206201 |
| ENSMUSG00000006058 | Snf8 | 2785 | 18.78774567 | 19.5212364 | 21.90270082 | 17.65183728 | 19.63298574 | 19.16276268 | 23.00228627 | 23.31313612 | 23.33656082 | 20.60409546 | 18.62966065 | 20.44236116 | 22.37206877 | 25.81524139 | 21.68814396 | 19.81212397 | 19.37645459 | 22.5171927 |
| ENSMUSG00000006095 | Tbcb | 1589 | 102.3976173 | 88.80643166 | 88.19264785 | 86.13302529 | 92.17460272 | 87.69181046 | 85.54394371 | 92.0319124 | 96.78442408 | 85.75552701 | 83.37316543 | 87.7454532 | 96.64585178 | 95.54796833 | 88.21126063 | 95.66627526 | 78.75101283 | 86.47750069 |
| ENSMUSG00000006127 | Inpp5k | 5181 | 21.83097344 | 22.38863109 | 21.84791759 | 24.86906647 | 24.44221022 | 23.46127301 | 23.05819616 | 25.79332484 | 21.12985117 | 23.70731171 | 19.63190245 | 21.18000244 | 20.96677897 | 25.81721129 | 25.67840648 | 23.64986367 | 25.48428736 | 24.74932016 |
| ENSMUSG00000006134 | Crkl | 3860 | 10.35100142 | 9.942086565 | 11.07829037 | 11.80337936 | 11.44691863 | 10.44972154 | 9.89153676 | 11.79413048 | 11.76910023 | 10.32185084 | 11.53530086 | 12.63805417 | 9.700239692 | 10.31154997 | 10.4818466 | 12.26093025 | 11.38866905 | 11.52911071 |
| ENSMUSG00000006143 | 2310043J07R | 1288 | 0 | 0 | 0 | 0 | 0 | 0 | 0.110200339 | 0.029652486 | 0 | 0 | 0 | 0 | 0 | 0.108177222 | 0.031988786 | 0.029728732 | 0.059512883 | 0 |
| ENSMUSG00000006154 | Eps8l1 | 4944 | 2.622184201 | 2.883883237 | 3.874578888 | 2.709333947 | 3.300307873 | 3.016863432 | 2.545544619 | 2.572425162 | 2.315908451 | 2.651273363 | 2.879639488 | 2.314363032 | 2.524469068 | 2.480024084 | 2.741770204 | 2.137582452 | 2.441906053 | 2.492321987 |
| ENSMUSG00000006169 | Clint1 | 4700 | 51.01525489 | 49.93390208 | 52.92270066 | 51.41612322 | 50.31322148 | 58.65145891 | 54.49011299 | 55.3058491 | 59.58023844 | 53.62138487 | 53.26399626 | 56.69446238 | 47.63154055 | 52.71898005 | 58.707834 | 57.76993555 | 59.16112426 | 57.79467425 |
| ENSMUSG00000006179 | Prss16 | 2683 | 0.079212031 | 0.099330285 | 0 | 0.084906903 | 0 | 0.055407455 | 0 | 0 | 0 | 0 | 0 | 0 | 0 | 0.08655253 | 0 | 0 | 0 | 0 |
| ENSMUSG00000006191 | Cdkal1 | 12791 | 2.920963938 | 3.810238781 | 3.312892861 | 2.988485357 | 2.837266081 | 3.385903303 | 3.088584701 | 3.063513788 | 3.435697937 | 3.653064415 | 3.354070261 | 3.416751138 | 2.780729603 | 3.111764748 | 2.415852312 | 3.319856431 | 3.454790113 | 2.791096104 |
| ENSMUSG00000006200 | Rhox6 | 1006 | 0 | 0 | 0 | 0 | 0 | 0 | 0 | 0 | 0 | 0 | 0 | 0 | 0 | 0 | 0 | 0 | 0 | 0 |
| ENSMUSG00000006204 | 5430419D17R | 3123 | 0 | 0.053334765 | 0.015489443 | 0 | 0 | 0 | 0.015149753 | 0.061146978 | 0 | 0.090587326 | 0 | 0.035744901 | 0 | 0.148716258 | 0.079157648 | 0.036782524 | 0 | 0 |
| ENSMUSG00000006205 | Htra1 | 3408 | 3.404904066 | 4.340060162 | 3.76143942 | 3.155048845 | 3.827391817 | 3.635030339 | 0.999563639 | 1.692210318 | 1.824872031 | 1.63256543 | 0.71494216 | 1.768806209 | 2.330527782 | 3.21619854 | 1.414490035 | 0.584246357 | 1.113351935 | 1.840093359 |
| ENSMUSG00000006215 | Zbtb17 | 3846 | 13.07426501 | 16.37927274 | 15.696871 | 18.82384281 | 17.99628347 | 15.65433471 | 15.22961428 | 16.9115084 | 16.44890489 | 17.58037142 | 15.62685849 | 15.35438664 | 13.14748185 | 17.17199487 | 17.06554304 | 16.89525761 | 16.60208285 | 19.98120699 |
| ENSMUSG00000006216 | Clcnkb | 2899 | 299.574252 | 306.5958367 | 295.8310865 | 328.2312559 | 299.5336443 | 324.0499218 | 311.1147287 | 361.464279 | 381.8738728 | 388.4931694 | 396.306382 | 382.9505101 | 221.4065456 | 155.8336109 | 215.1178855 | 215.716523 | 232.0598446 | 216.355238 |
| ENSMUSG00000006218 | Fam131c | 1426 | 6.22972075 | 5.186158848 | 7.598647125 | 4.153531391 | 6.311830102 | 6.637147345 | 3.085609495 | 4.472742778 | 4.072439497 | 1.88470553 | 3.986830336 | 3.718434745 | 4.531894224 | 2.800975817 | 2.195875349 | 2.067585393 | 3.735873261 | 2.468851915 |
| ENSMUSG00000006219 | Fblim1 | 4441 | 7.599439263 | 6.661078601 | 7.777235009 | 8.44330995 | 5.939269122 | 8.937568024 | 0.500719637 | 1.694191235 | 1.799185782 | 1.36961061 | 1.183347317 | 1.998354053 | 0.866446644 | 0.763435798 | 2.152389319 | 1.12086894 | 2.373278912 | 2.118116922 |
| ENSMUSG00000006221 | Hspb7 | 2766 | 36.15859696 | 35.80594162 | 32.84363341 | 31.16467675 | 35.5682039 | 31.4586407 | 17.49850707 | 19.81420732 | 22.70767771 | 21.06951165 | 19.96668547 | 21.41012943 | 14.08973776 | 8.345159592 | 12.67625599 | 12.1821166 | 8.701704431 | 7.756164722 |
| ENSMUSG00000006235 | Epor | 1821 | 1.167083362 | 0.89639309 | 0.371899739 | 1.326049066 | 0.502200192 | 1.278955782 | 0.753469351 | 0.734065944 | 0.339262898 | 1.061602874 | 0.708360175 | 0.429115473 | 0.352177334 | 0.535599031 | 0.339386787 | 0.441572079 | 0.31570261 | 0.181249116 |
| ENSMUSG00000006241 | Ccdc159 | 1789 | 2.25712226 | 1.824853904 | 3.244730935 | 3.896500717 | 2.986385426 | 3.40692355 | 2.406625926 | 2.817997271 | 3.361224875 | 3.399910967 | 5.234148609 | 2.839142144 | 4.798073564 | 4.491239306 | 2.579415461 | 2.73962982 | 3.770502099 | 3.074852248 |
| ENSMUSG00000006262 | Mob1b | 8972 | 4.893875038 | 4.470432974 | 4.647568342 | 5.540247366 | 5.037436059 | 4.84923095 | 4.31361696 | 5.018818818 | 4.406938824 | 4.677232044 | 4.329139684 | 4.056153813 | 7.972724036 | 6.103163066 | 7.834357373 | 8.475829949 | 7.313265749 | 7.394224555 |
| ENSMUSG00000006269 | Atp6v1b1 | 1912 | 224.3081727 | 187.38503 | 227.0165732 | 183.0305343 | 199.5511291 | 188.5699213 | 185.1677701 | 182.4925671 | 296.5558381 | 205.4212124 | 230.2043371 | 249.8860208 | 159.941713 | 142.7815615 | 171.831175 | 192.0538311 | 175.9962797 | 158.4101159 |
| ENSMUSG00000006270 | Vax1 | 2272 | 0 | 0 | 0 | 0 | 0 | 0 | 0 | 0 | 0 | 0 | 0 | 0 | 0 | 0 | 0 | 0 | 0 | 0 |
| ENSMUSG00000006273 | Atp6v1b2 | 5245 | 44.80669557 | 49.26751582 | 44.51840439 | 47.34179059 | 49.04041946 | 51.40446585 | 45.78820939 | 44.04687172 | 40.03219397 | 45.2448979 | 41.72680358 | 40.04467842 | 42.68221937 | 43.66364059 | 45.9933479 | 48.35786147 | 47.45299754 | 41.17554871 |
| ENSMUSG00000006276 | Eps15l1 | 3156 | 9.939423292 | 12.2126168 | 12.92106657 | 12.66065776 | 10.32487716 | 13.73847968 | 15.18623059 | 12.06521711 | 10.60983438 | 14.37229928 | 13.66945105 | 11.63710582 | 14.25561108 | 14.42180153 | 13.668577 | 13.68561633 | 12.76328835 | 13.82199671 |
| ENSMUSG00000006281 | Tep1 | 8113 | 9.257567613 | 10.83192592 | 9.152393578 | 9.322240059 | 9.059219455 | 8.612024448 | 10.91113303 | 12.41853292 | 11.5391286 | 11.82690511 | 10.36409798 | 11.76442537 | 12.13079933 | 11.20314052 | 10.55304242 | 12.42686667 | 12.93447565 | 12.16397602 |
| ENSMUSG00000006288 | Ttc5 | 1943 | 38.96125502 | 37.37627864 | 39.31127351 | 35.50150341 | 36.2661535 | 34.70986639 | 39.39882373 | 38.36931008 | 37.87972397 | 35.28415982 | 42.46389773 | 33.83986131 | 41.13108903 | 43.36056332 | 36.17594151 | 39.90657745 | 39.19421099 | 37.39939783 |
| ENSMUSG00000006289 | Osgep | 4876 | 13.68603904 | 11.9423583 | 12.49021221 | 11.14731868 | 12.33898781 | 12.51013434 | 13.44859988 | 13.4096376 | 13.56553411 | 13.51859786 | 12.60020439 | 15.71675651 | 17.51303298 | 13.46840434 | 15.91955113 | 16.97791083 | 12.8121132 | 15.50092548 |
| ENSMUSG00000006299 | Aamp | 1741 | 168.1163941 | 164.4405588 | 158.6518428 | 164.789142 | 158.6335313 | 158.2784328 | 168.3798729 | 170.0779413 | 167.0880878 | 188.3590702 | 156.2770756 | 152.9240152 | 176.4728207 | 161.2871639 | 158.0379036 | 176.5194802 | 170.8943382 | 172.4524437 |
| ENSMUSG00000006301 | Tmbim1 | 5505 | 15.78984287 | 16.2358756 | 13.66409441 | 15.2615015 | 15.46691607 | 14.9243292 | 20.34316277 | 19.86976212 | 22.28037129 | 23.91367184 | 23.49260901 | 22.44793405 | 33.5869257 | 29.79845898 | 29.83277053 | 37.00382041 | 35.63893076 | 35.56356237 |
| ENSMUSG00000006304 | Arpc2 | 1669 | 234.6061603 | 227.8410351 | 228.3903051 | 213.0094841 | 217.2425291 | 223.1802866 | 221.9642157 | 226.2253386 | 227.4512377 | 219.3680113 | 217.5206168 | 201.8594005 | 230.166351 | 227.8793479 | 238.9640869 | 246.8126749 | 222.058293 | 221.5195963 |
| ENSMUSG00000006307 | Wbp7 | 10101 | 3.627310339 | 3.232020229 | 2.839867686 | 2.607096686 | 2.758972756 | 2.492108569 | 3.737799998 | 2.695889804 | 3.983687348 | 3.374909262 | 2.918240707 | 3.000485574 | 3.316144127 | 4.037017001 | 3.463035437 | 3.654306062 | 2.849524698 | 3.523501836 |
| ENSMUSG00000006310 | Zbtb32 | 2873 | 0.340278123 | 0.162332238 | 0.067349155 | 0.333025385 | 0.301557475 | 0.413945564 | 1.03748652 | 0.957136433 | 0.430071519 | 0.295409906 | 0.848076186 | 0.291414876 | 1.304986325 | 0.646628436 | 0.415887616 | 0.733025899 | 0.346844316 | 0.019146922 |
| ENSMUSG00000006311 | Etv2 | 1290 | 0 | 0 | 0 | 0 | 0 | 0 | 0 | 0 | 0 | 0 | 0 | 0 | 0 | 0 | 0 | 0 | 0 | 0 |
| ENSMUSG00000006313 | Upk1a | 1300 | 0.752014653 | 0.743133794 | 1.265153871 | 0.911220887 | 0.66644202 | 0.838584722 | 6.405408839 | 4.906254771 | 3.389967067 | 2.611423565 | 1.543498659 | 5.538631134 | 12.10529355 | 17.22003856 | 11.85337069 | 3.210520159 | 3.714702623 | 4.739246118 |

| ENSMUSG00000006315 | Tmem147 | 861 | 196.0372288 | 170.162728 | 185.3475919 | 168.0096583 | 178.4403024 | 185.8946916 | 162.8193585 | 155.9190412 | 136.8578648 | 159.5238079 | 153.5346227 | 157.0747392 | 133.671734 | 148.8260594 | 117.5751717 | 142.4446173 | 112.4416097 | 134.7435611 |
| --- | --- | --- | --- | --- | --- | --- | --- | --- | --- | --- | --- | --- | --- | --- | --- | --- | --- | --- | --- | --- |
| ENSMUSG00000006333 | Rps9 | 2549 | 75.27201435 | 63.20170908 | 66.68677351 | 59.39558663 | 68.39302767 | 62.59701927 | 70.79268632 | 63.19951092 | 65.94043579 | 65.4079587 | 61.41959158 | 65.58176126 | 82.79401417 | 92.66952862 | 72.10676376 | 68.93511078 | 58.8802584 | 75.07912212 |
| ENSMUSG00000006335 | Tfpt | 1338 | 21.09374954 | 16.7311397 | 19.55910308 | 18.28571062 | 17.446923 | 15.96205394 | 19.94346107 | 15.38542967 | 18.62320033 | 16.98552987 | 19.03142965 | 18.52178938 | 20.68401016 | 22.87492794 | 18.01413318 | 17.34238272 | 18.24652551 | 16.36294804 |
| ENSMUSG00000006342 | Susd2 | 3240 | 0.039356644 | 0 | 0.223951529 | 0.449985623 | 0.044566596 | 0.091764321 | 0 | 0.011787779 | 0 | 0.087316117 | 0.132708218 | 0 | 0 | 0.258022708 | 0 | 0 | 0 | 0.322584268 |
| ENSMUSG00000006344 | Ggt5 | 4678 | 1.172117937 | 1.004086807 | 0.785888907 | 1.402477637 | 1.389014471 | 1.207569822 | 0.353985413 | 1.053189378 | 0.387389203 | 0.53419993 | 1.000843508 | 0.906795715 | 0.116000661 | 0.099281931 | 0.044037576 | 0.122778775 | 0.286750832 | 0 |
| ENSMUSG00000006345 | Ggt1 | 3089 | 6.439761211 | 8.95102046 | 9.067100704 | 8.038449065 | 7.245498383 | 7.635837223 | 5.268877161 | 4.920872825 | 5.173314379 | 5.159254678 | 5.304891796 | 5.818272371 | 5.174341598 | 3.728758068 | 7.30930807 | 5.144254474 | 5.049790495 | 5.80542855 |
| ENSMUSG00000006356 | Crip2 | 1430 | 152.8105664 | 118.3656053 | 117.8555103 | 125.882298 | 104.712178 | 121.9413073 | 112.6901989 | 76.67859252 | 99.68288885 | 83.55236507 | 88.66765021 | 104.4104178 | 130.7468297 | 111.8880287 | 124.8147833 | 203.797072 | 135.8305404 | 141.6003649 |
| ENSMUSG00000006360 | Crip1 | 440 | 2222.344488 | 1730.226299 | 1951.76201 | 1561.81189 | 1854.501141 | 1933.908048 | 754.6372289 | 667.5858339 | 638.2973886 | 468.8280153 | 583.5059701 | 856.6435669 | 1026.215997 | 1026.83655 | 886.488931 | 1111.907024 | 634.5615294 | 986.9133829 |
| ENSMUSG00000006362 | Cbfa2t3 | 6273 | 0.067758929 | 0 | 0 | 0 | 0 | 0 | 0.052795911 | 0.182651374 | 0.006565681 | 0 | 0.152319332 | 0 | 0 | 0 | 0.03940847 | 0 | 0 | 0 |
| ENSMUSG00000006369 | Fbln1 | 4556 | 0.009329494 | 0.036559366 | 0.084940352 | 0.320007335 | 0.274677352 | 0.130516419 | 0.020769394 | 0.234720647 | 0.063280424 | 0 | 0.010486163 | 0.147012281 | 0.30318123 | 0.091746441 | 0.027130085 | 0.016808871 | 0.016824538 | 0.241479836 |
| ENSMUSG00000006373 | Pgrmc1 | 1946 | 1243.505743 | 1208.491145 | 1281.625117 | 1263.230291 | 1307.625149 | 1261.812234 | 1572.721101 | 1545.320339 | 1613.978067 | 1642.521158 | 1582.956718 | 1514.365546 | 1722.637089 | 1637.070548 | 1758.370615 | 1841.805763 | 1898.884849 | 1817.222731 |
| ENSMUSG00000006378 | Gcat | 3840 | 6.397914517 | 5.422020543 | 5.467216712 | 4.959509514 | 5.452444478 | 5.574682528 | 7.466532143 | 7.290372647 | 7.786825636 | 7.232230265 | 7.39018889 | 7.296735057 | 7.348400178 | 7.256888655 | 7.135165278 | 4.457266018 | 6.447600989 | 4.884923279 |
| ENSMUSG00000006386 | Tek | 5081 | 0 | 0 | 0 | 0 | 0 | 0 | 0 | 0 | 0 | 0 | 0 | 0.010985173 | 0.038836384 | 0 | 0 | 0 | 0 | 0 |
| ENSMUSG00000006389 | Mpl | 4209 | 0.01009864 | 0.015829363 | 0.022985759 | 0 | 0.011435477 | 0.035319126 | 0.044963344 | 0 | 0 | 0 | 0 | 0.013261027 | 0 | 0 | 0.009788918 | 0 | 0 | 0.117624605 |
| ENSMUSG00000006390 | Elovl1 | 2317 | 31.70001907 | 36.74914011 | 33.57127182 | 37.32190857 | 34.10989152 | 34.5821192 | 24.89173742 | 33.60997346 | 32.31639433 | 32.58017951 | 36.80548161 | 32.11147097 | 38.49465103 | 39.54863376 | 38.23191418 | 43.47975316 | 43.3714072 | 37.44038036 |
| ENSMUSG00000006392 | Med8 | 2925 | 21.15813207 | 20.92163647 | 21.10243581 | 19.11227398 | 18.59455513 | 18.63521605 | 21.0925584 | 21.71417614 | 18.99508026 | 21.37498548 | 18.16265088 | 19.97914486 | 23.20708314 | 23.45227934 | 22.69254924 | 21.3511045 | 20.95170897 | 20.36747439 |
| ENSMUSG00000006395 | Hyi | 1452 | 10.09937034 | 9.337705198 | 7.762419127 | 8.62898567 | 9.281638173 | 9.72625969 | 7.983199957 | 7.601655851 | 9.814417668 | 8.63780101 | 9.114093319 | 7.495905099 | 13.59005986 | 8.796228672 | 9.61937152 | 6.98829956 | 7.8130743 | 7.463356766 |
| ENSMUSG00000006398 | Cdc20 | 1819 | 1.939488516 | 2.80201914 | 3.536932126 | 1.878547732 | 2.169773359 | 1.552779447 | 4.005581394 | 0.629891189 | 3.735995108 | 1.762643112 | 2.074888246 | 2.761632563 | 1.111934364 | 3.472455132 | 4.62073524 | 2.589194453 | 4.930375742 | 2.721726006 |
| ENSMUSG00000006403 | Adamts4 | 5847 | 0.00726957 | 0.017092301 | 0 | 0.046753252 | 0.008231901 | 0 | 0 | 0.150235207 | 0 | 0 | 0 | 0 | 0 | 0 | 0 | 0 | 0 | 0 |
| ENSMUSG00000006411 | Pvrl4 | 3736 | 16.55380919 | 21.16831126 | 18.45082461 | 20.90247055 | 21.06415824 | 22.33586813 | 13.9683846 | 14.27103687 | 13.26214627 | 14.50111297 | 14.78261563 | 17.65902387 | 9.163905404 | 11.57372734 | 14.40289936 | 12.90360672 | 13.05925481 | 14.7535131 |
| ENSMUSG00000006412 | Pfdn2 | 1989 | 45.47562322 | 42.37386746 | 43.75263002 | 39.1243156 | 41.57397932 | 41.75484237 | 41.6751199 | 38.74925493 | 40.93803 | 44.25860381 | 38.35928039 | 41.27945684 | 47.05009908 | 43.47857754 | 40.39368225 | 42.35260748 | 41.10105143 | 38.94058435 |
| ENSMUSG00000006418 | Rnf114 | 3348 | 39.26777463 | 37.30287946 | 39.47326312 | 40.89335073 | 40.31120489 | 39.71027007 | 48.33015589 | 41.64888327 | 40.31308607 | 48.30552932 | 42.78056318 | 36.72697272 | 45.75137489 | 45.72273358 | 47.93311236 | 50.23068928 | 48.65195993 | 44.59220897 |
| ENSMUSG00000006423 | C330007P06R | 7441 | 6.386344147 | 8.215180875 | 6.78048544 | 7.506765268 | 7.322313881 | 7.525142984 | 7.299416128 | 9.705931051 | 7.405934429 | 7.179377341 | 6.446184426 | 7.71112769 | 7.160122384 | 6.69104444 | 7.380953367 | 7.060168439 | 8.642860664 | 6.402081229 |
| ENSMUSG00000006435 | Neurl1a | 4404 | 26.91801452 | 30.3250211 | 28.97578862 | 36.23985883 | 26.75452978 | 27.53304702 | 23.46296339 | 23.72716008 | 22.62265705 | 26.33758629 | 17.37862931 | 18.51650387 | 26.96228994 | 29.05391934 | 31.74316057 | 32.38703737 | 25.73361942 | 26.18053762 |
| ENSMUSG00000006442 | Srm | 1591 | 21.26594602 | 20.85458368 | 19.70210413 | 17.61159162 | 17.60702676 | 19.71519821 | 37.17212361 | 28.27822127 | 28.29469625 | 25.78322554 | 26.57500833 | 27.92537236 | 33.08432164 | 40.86846156 | 35.47839822 | 19.90341441 | 19.65698194 | 30.39158065 |
| ENSMUSG00000006445 | Epha2 | 4654 | 0.803707669 | 0.737264311 | 0.893881309 | 0.910437714 | 0.806680285 | 0.926318827 | 0.609961482 | 1.075033243 | 0.3451384 | 0.466036173 | 0.287429918 | 0.251854086 | 0.413395955 | 0.319340524 | 0.531176054 | 1.217664352 | 0.897629214 | 0.791923109 |
| ENSMUSG00000006456 | Rbm14 | 5516 | 14.194078 | 15.32175719 | 14.93475747 | 15.21454744 | 15.34881322 | 16.62837379 | 14.23840591 | 17.14365271 | 13.85079528 | 17.87384346 | 18.08450205 | 15.13782292 | 17.10876681 | 14.1033079 | 17.16482513 | 16.7504053 | 16.91193014 | 21.73039223 |
| ENSMUSG00000006457 | Actn3 | 2873 | 4.053748079 | 7.745566773 | 5.70784086 | 4.820938906 | 3.350638612 | 5.829733362 | 0.411701 | 0.491861778 | 0.702450147 | 0.623643134 | 1.080881414 | 0.36912551 | 3.073586214 | 0.792119834 | 2.681758076 | 3.971667597 | 2.801434859 | 1.416872223 |
| ENSMUSG00000006462 | A530013C23R | 6038 | 0 | 0 | 0.008011515 | 0 | 0 | 0.041034062 | 0 | 0 | 0 | 0 | 0 | 0 | 0 | 0 | 0 | 0 | 0 | 0 |
| ENSMUSG00000006463 | Zdhhc24 | 2939 | 29.66250971 | 34.45770617 | 36.40770641 | 35.23656134 | 37.53602213 | 34.95162188 | 31.63300922 | 32.46159279 | 27.98552976 | 32.03809269 | 27.79693059 | 33.44381824 | 35.93726944 | 36.20394835 | 38.94451251 | 44.53123379 | 40.84313096 | 39.47404086 |
| ENSMUSG00000006464 | Bbs1 | 5564 | 5.790604498 | 6.286581404 | 5.094696044 | 6.870186233 | 5.276864385 | 6.902115119 | 2.482980274 | 3.459556221 | 2.672237992 | 3.804936334 | 2.26682046 | 2.166819396 | 1.604794577 | 1.944908764 | 2.917579623 | 2.55316595 | 3.023947581 | 2.521085948 |
| ENSMUSG00000006469 | Slc34a3 | 2344 | 0 | 0 | 0 | 0 | 0 | 0 | 0 | 0 | 0 | 0 | 0 | 0 | 0 | 0.01981403 | 0 | 0 | 0 | 0 |
| ENSMUSG00000006471 | Ndor1 | 5895 | 6.828227599 | 7.171172649 | 5.744100297 | 7.705574419 | 7.936256116 | 8.094874035 | 8.082081031 | 7.606086586 | 8.935971783 | 8.486326744 | 10.21144151 | 8.881270824 | 7.774275004 | 9.22578903 | 8.254289653 | 8.086820393 | 9.61570706 | 7.707806981 |
| ENSMUSG00000006476 | Nelf | 3467 | 31.94937662 | 37.25240579 | 31.71417205 | 38.72754475 | 35.01260787 | 41.83468451 | 38.66074976 | 33.02590782 | 34.41515321 | 43.57394094 | 36.19983153 | 34.22673743 | 43.75415218 | 40.00058983 | 45.99077626 | 46.46339352 | 43.48879493 | 40.57060453 |
| ENSMUSG00000006488 | Prl7a1 | 1600 | 0 | 0 | 0 | 0 | 0 | 0 | 0 | 0 | 0 | 0 | 0 | 0 | 0 | 0 | 0 | 0 | 0 | 0 |
| ENSMUSG00000006490 | Prl8a9 | 920 | 0 | 0 | 0 | 0 | 0 | 0 | 0 | 0 | 0 | 0 | 0 | 0 | 0 | 0 | 0 | 0 | 0 | 0 |
| ENSMUSG00000006494 | Pdk1 | 6591 | 7.990276604 | 10.73027984 | 10.05488341 | 10.11315549 | 10.61078518 | 10.08196264 | 14.77309865 | 17.49398617 | 18.34052856 | 16.06028354 | 17.08474847 | 18.00395974 | 13.30786662 | 10.98563681 | 14.84031159 | 9.010579894 | 12.67076331 | 12.51914127 |
| ENSMUSG00000006498 | Ptbp1 | 4717 | 20.73445199 | 24.06127424 | 21.53580957 | 26.5523237 | 23.74453813 | 23.92019808 | 19.99028389 | 22.0474691 | 18.20519088 | 21.24130685 | 23.75074786 | 22.97943963 | 17.94648506 | 20.33221125 | 21.30392089 | 22.76980152 | 22.01101088 | 22.09927014 |
| ENSMUSG00000006517 | Mvd | 1751 | 5.461830158 | 6.829999443 | 7.983980738 | 8.27436442 | 5.525138011 | 5.292039508 | 8.673540797 | 6.150918034 | 6.656644181 | 8.051433623 | 11.84142318 | 5.73776086 | 9.945269445 | 11.53807996 | 9.270938333 | 11.48062187 | 8.536411075 | 9.204836248 |
| ENSMUSG00000006519 | Cyba | 1060 | 192.0752766 | 176.3069213 | 207.0022016 | 170.6390057 | 199.3388159 | 191.3857465 | 218.4417459 | 205.8786673 | 232.5483932 | 214.491218 | 241.3536817 | 237.216566 | 119.9324064 | 102.8342199 | 135.6153099 | 125.1669383 | 120.9447765 | 123.6666994 |
| ENSMUSG00000006522 | Itih3 | 3852 | 0.231726038 | 0.406465739 | 0.238602564 | 0.248385756 | 0.399849833 | 0.192962358 | 0.307065673 | 0.436257971 | 1.464837139 | 0.64875059 | 0.384481753 | 0.608581991 | 0.858057751 | 1.265999267 | 0.641768783 | 1.172972916 | 0.318390836 | 0.37129719 |

| ENSMUSG00000006526 | Tmem110 | 4345 | 9.577115611 | 8.517980547 | 8.939918268 | 9.972049059 | 9.526686848 | 10.95976455 | 8.852752122 | 8.798755997 | 8.028763832 | 9.332475213 | 8.807305349 | 8.28563921 | 10.28647113 | 11.03113882 | 12.89622923 | 11.57971418 | 11.88159308 | 9.710468321 |
| --- | --- | --- | --- | --- | --- | --- | --- | --- | --- | --- | --- | --- | --- | --- | --- | --- | --- | --- | --- | --- |
| ENSMUSG00000006527 | Sfmbt1 | 8604 | 1.5759125 | 2.34630566 | 2.822351492 | 1.885138515 | 1.840469885 | 1.975428593 | 2.535000579 | 1.908732338 | 2.704600349 | 2.290678052 | 2.565322038 | 2.328896196 | 2.356510693 | 3.022860174 | 2.284186676 | 2.532235659 | 3.193858075 | 2.81311099 |
| ENSMUSG00000006529 | Itih1 | 3271 | 0 | 0 | 0 | 0 | 0 | 0 | 0 | 0 | 0 | 0 | 0 | 0 | 0 | 0 | 0 | 0 | 0 | 0 |
| ENSMUSG00000006538 | Ihh | 3891 | 0.032771917 | 0.308214904 | 0.186482384 | 0.163930769 | 0.123700652 | 0 | 0.158073715 | 1.207315728 | 0.137605938 | 0.169650436 | 0.282401451 | 0.129103306 | 0.278926286 | 1.336863991 | 0.381201751 | 1.190738498 | 0.748598963 | 0.791701356 |
| ENSMUSG00000006542 | Prkag3 | 3091 | 0 | 0 | 0 | 0 | 0 | 0 | 0 | 0 | 0 | 0 | 0 | 0 | 0 | 0 | 0 | 0 | 0 | 0 |
| ENSMUSG00000006546 | Cryba2 | 1187 | 0 | 0 | 0 | 0 | 0 | 0.08349239 | 0.119577116 | 0.225228995 | 0.277583931 | 0.039722581 | 0 | 0.517247084 | 0 | 0 | 0 | 0 | 0 | 0 |
| ENSMUSG00000006567 | Atp7b | 6757 | 0.125810792 | 0.024650654 | 0.329315139 | 0.14834142 | 0.099725756 | 0.110003108 | 0.098028342 | 0 | 0.097526158 | 0.083736634 | 0.007070439 | 0.016520841 | 0.153318079 | 0.089355207 | 0.23780682 | 0.028334029 | 0.232555598 | 0.29307797 |
| ENSMUSG00000006570 | Defb2 | 303 | 18.79766861 | 13.96283025 | 17.24205042 | 21.50240706 | 24.46308992 | 18.80714311 | 148.8085248 | 85.96441729 | 64.83817828 | 84.34218206 | 53.45119117 | 68.52616001 | 1177.290739 | 1780.356683 | 930.5024652 | 224.3096638 | 385.7927576 | 900.4791068 |
| ENSMUSG00000006574 | Slc4a1 | 4866 | 0 | 0 | 0 | 0 | 0 | 0.050917318 | 0 | 0 | 0.025392426 | 0.135657593 | 0 | 0 | 0 | 0 | 0 | 0 | 0 | 0 |
| ENSMUSG00000006575 | Rundc3a | 2853 | 2.905190791 | 3.432874483 | 3.492796093 | 2.363489156 | 2.463112813 | 3.803732441 | 5.207213874 | 4.056185744 | 5.514633376 | 5.883508714 | 4.554780479 | 4.930090071 | 7.48710837 | 3.499992565 | 5.05451965 | 8.428496827 | 7.872138095 | 6.92193106 |
| ENSMUSG00000006576 | Slc4a3 | 5432 | 3.184758219 | 2.974365555 | 3.161377628 | 3.120159885 | 3.136726984 | 3.229320266 | 3.031077369 | 2.263982617 | 4.109552935 | 3.246384944 | 2.849610925 | 2.846269981 | 4.513616143 | 4.728199618 | 3.693880287 | 4.158957726 | 4.056999396 | 2.825394105 |
| ENSMUSG00000006585 | Cdt1 | 2179 | 0.546188586 | 0.963153894 | 0.244198639 | 1.840005462 | 1.016093845 | 0.591267129 | 1.129077239 | 0.823792067 | 2.079172434 | 0.973741003 | 1.008558095 | 0.768457952 | 0.882948496 | 0.42628809 | 0.397077868 | 0.738047504 | 0.897035861 | 0.429166964 |
| ENSMUSG00000006586 | Runx1t1 | 7944 | 0 | 0 | 0 | 0 | 0 | 0 | 0 | 0 | 0 | 0 | 0 | 0 | 0 | 0 | 0 | 0 | 0 | 0 |
| ENSMUSG00000006587 | Snai3 | 1586 | 0 | 0.168034775 | 0.122001338 | 0.028727014 | 0 | 0 | 0.238651596 | 0.07224288 | 0 | 0.416210495 | 0.271106322 | 0.175963627 | 0 | 0.52710818 | 0 | 0 | 0 | 0 |
| ENSMUSG00000006589 | Aprt | 861 | 45.26974032 | 41.78620878 | 45.28346744 | 40.26940156 | 43.77154034 | 40.63209048 | 54.01668221 | 40.89825205 | 43.38695679 | 49.45073756 | 54.59995255 | 39.28489144 | 46.92548229 | 41.96689894 | 41.72794046 | 49.8089202 | 43.17829036 | 49.19513609 |
| ENSMUSG00000006599 | Gtf2h1 | 3226 | 13.4393303 | 13.20743698 | 12.98557882 | 13.23332255 | 13.74132105 | 12.6877117 | 16.67529942 | 14.77499014 | 11.19670624 | 13.30041536 | 11.29953296 | 16.004181 | 14.75675765 | 16.00924525 | 11.74997874 | 15.08597706 | 14.58917935 | 14.28940838 |
| ENSMUSG00000006611 | Hfe | 4152 | 1.422981567 | 1.123267146 | 1.479633515 | 1.689884592 | 0.834657636 | 1.229270607 | 0.182322462 | 1.40737899 | 1.626827694 | 1.374093231 | 1.185168768 | 1.559397124 | 0.344563006 | 0.324392711 | 1.667114978 | 1.300331321 | 1.818468326 | 0.755182824 |
| ENSMUSG00000006638 | Abhd1 | 1669 | 0.076402354 | 0.339316478 | 0.144917706 | 0.163790453 | 0.259549019 | 0.29690074 | 0.198435442 | 0.205950642 | 0.049354722 | 0.084752612 | 0.314873902 | 0.133770312 | 0.059115539 | 0.333929927 | 0.123431863 | 0.045884491 | 0.344454436 | 0.098877963 |
| ENSMUSG00000006641 | Slc5a6 | 4888 | 4.843572637 | 3.775643085 | 3.315289008 | 4.22241266 | 5.090876542 | 4.602483847 | 5.120377895 | 5.320995507 | 4.111910739 | 5.1607255 | 5.883904461 | 5.789390534 | 3.249770328 | 4.323252815 | 3.759389101 | 5.24850797 | 4.735900845 | 4.085168932 |
| ENSMUSG00000006642 | Tcf23 | 4098 | 0 | 0 | 0 | 0 | 0 | 0 | 0 | 0 | 0 | 0 | 0 | 0 | 0 | 0 | 0 | 0 | 0 | 0 |
| ENSMUSG00000006649 | Nphs1 | 5306 | 0 | 0 | 0 | 0 | 0 | 0 | 0 | 0 | 0 | 0 | 0 | 0 | 0 | 0 | 0 | 0 | 0 | 0 |
| ENSMUSG00000006651 | Aplp1 | 2350 | 1.501246643 | 1.063177475 | 0.82337924 | 1.279586777 | 1.781905259 | 1.159744828 | 1.389180786 | 0.991377254 | 0.9113612 | 1.725515098 | 0.853850322 | 1.116313252 | 1.196561398 | 0.217397856 | 0.999356888 | 1.645681424 | 1.141634376 | 1.825834181 |
| ENSMUSG00000006673 | Qrich1 | 3351 | 32.345031 | 47.19078152 | 40.23187977 | 33.88186287 | 48.26119472 | 39.55641967 | 30.60993373 | 44.70026924 | 35.70481298 | 32.92528965 | 43.74024847 | 36.41093358 | 23.81946946 | 40.88631984 | 36.8367238 | 39.07904426 | 42.88976824 | 43.0256248 |
| ENSMUSG00000006675 | P4htm | 1801 | 5.215793394 | 5.105140924 | 6.150770934 | 6.147325804 | 4.810519856 | 5.69540025 | 5.490477455 | 5.004667944 | 5.397011505 | 6.204728857 | 5.252327473 | 6.322262725 | 6.957416429 | 3.352432738 | 7.389285149 | 5.825444991 | 5.490385675 | 7.055582262 |
| ENSMUSG00000006676 | Usp19 | 5174 | 32.16230463 | 36.22310454 | 31.52600393 | 37.37167997 | 32.87557368 | 36.26907655 | 30.3042555 | 32.75954424 | 26.49182923 | 35.14887176 | 31.58835194 | 32.15819292 | 27.91725044 | 28.0154613 | 35.50787354 | 28.95847444 | 32.74846513 | 33.75607148 |
| ENSMUSG00000006678 | Pola1 | 6395 | 0.485203729 | 1.453369427 | 1.08169114 | 0.876310939 | 0.963391123 | 0.898845519 | 0.79162731 | 0.489722752 | 0.856576481 | 0.774171046 | 0.64247794 | 0.724425331 | 0.763699736 | 0.806144441 | 0.721590971 | 0.359255112 | 1.16866738 | 1.101042324 |
| ENSMUSG00000006699 | Cdc42 | 4447 | 196.7166692 | 203.0535891 | 199.9990396 | 191.0859091 | 205.8189027 | 201.8881104 | 197.5812369 | 192.2502649 | 208.1553726 | 193.162022 | 192.6038803 | 182.2073249 | 175.4627694 | 158.4028499 | 179.0836235 | 176.2211763 | 197.0180228 | 167.0559897 |
| ENSMUSG00000006705 | Pknox1 | 7004 | 4.272364923 | 3.824038685 | 5.283516665 | 4.065627172 | 4.947884786 | 4.959518309 | 4.478627374 | 4.77132383 | 4.745505171 | 3.830489742 | 4.733840832 | 4.741621822 | 3.70482417 | 3.116607806 | 4.241336636 | 3.662865071 | 4.011018794 | 4.09976495 |
| ENSMUSG00000006711 | D130043K22R | 5253 | 0 | 0.063416894 | 0 | 0 | 0 | 0.018866451 | 0 | 0.007270589 | 0 | 0 | 0.03637918 | 0.010625483 | 0.103303082 | 0 | 0 | 0.036446419 | 0.007296078 | 0 |
| ENSMUSG00000006715 | Gmnn | 1596 | 7.270622215 | 4.404148296 | 5.879990532 | 6.394532538 | 6.15220077 | 5.122932983 | 6.521797847 | 5.264616873 | 5.496696652 | 7.769821401 | 7.004599175 | 8.078582743 | 8.252895935 | 9.166596195 | 7.925361941 | 9.644626676 | 7.372288941 | 7.272507217 |
| ENSMUSG00000006717 | Acot13 | 718 | 177.4798297 | 171.9464986 | 192.1466693 | 155.2755926 | 177.7127154 | 169.8457902 | 203.2863712 | 172.504124 | 170.4249837 | 194.7757463 | 185.1113294 | 162.083087 | 188.326999 | 152.7868167 | 174.8483855 | 163.2950416 | 158.5363535 | 177.51546 |
| ENSMUSG00000006720 | Zfp184 | 3405 | 2.072205351 | 3.199211868 | 3.011802771 | 4.014188928 | 2.431333589 | 2.735951223 | 2.723431739 | 2.400344821 | 3.132836294 | 2.49254819 | 2.97453486 | 3.22927622 | 2.593366576 | 1.936875304 | 3.291284347 | 3.216186114 | 3.23043971 | 3.473409088 |
| ENSMUSG00000006724 | Cyp27b1 | 2564 | 0 | 0 | 0 | 0 | 0 | 0 | 0 | 0 | 0 | 0 | 0 | 0 | 0 | 0.036227837 | 0 | 0 | 0 | 0 |
| ENSMUSG00000006728 | Cdk4 | 2710 | 79.80307588 | 79.43465773 | 80.11085027 | 76.26010963 | 77.43733845 | 75.40792372 | 85.65166156 | 78.68198621 | 83.37609796 | 82.52243009 | 82.4338398 | 82.19937616 | 83.17252031 | 79.28057131 | 77.02106352 | 86.76849448 | 74.34735897 | 72.32378127 |
| ENSMUSG00000006731 | B4galnt1 | 2417 | 21.45482613 | 32.63753972 | 24.65709119 | 26.97470188 | 26.58507162 | 31.96223072 | 16.42339165 | 17.20791321 | 21.69236018 | 16.32815003 | 22.79045392 | 22.07686116 | 8.735647743 | 9.761521057 | 12.29057584 | 9.030056359 | 11.65487308 | 12.99553163 |
| ENSMUSG00000006732 | Mettl1 | 2900 | 3.869436714 | 3.675905569 | 3.903243484 | 3.016455349 | 4.099512119 | 4.032567284 | 4.502861857 | 5.360106131 | 4.360089772 | 3.918386029 | 3.574884824 | 4.311278765 | 6.22602818 | 6.566233045 | 5.782425258 | 5.86242403 | 4.281972482 | 5.614722617 |
| ENSMUSG00000006736 | Tspan31 | 1510 | 100.4360716 | 93.16579622 | 105.9091996 | 103.8550428 | 102.8620647 | 105.1764974 | 96.91265955 | 92.57231311 | 93.91071111 | 94.45753467 | 98.08104059 | 101.318365 | 91.01902088 | 83.63011497 | 79.64724612 | 92.96249453 | 86.75449193 | 87.54098242 |
| ENSMUSG00000006740 | Kif5b | 7579 | 41.88265663 | 44.3629821 | 42.66750896 | 40.6256152 | 43.08312049 | 40.51045484 | 33.62889582 | 33.4807127 | 31.47543202 | 33.43289079 | 32.13573536 | 31.30655517 | 33.34574643 | 33.82041408 | 31.74786732 | 34.3296843 | 34.07849516 | 31.04287498 |
| ENSMUSG00000006763 | Saal1 | 6895 | 2.527501403 | 2.753930342 | 2.778233215 | 1.777508474 | 2.959816626 | 3.126242075 | 2.895714359 | 2.73633746 | 2.341568409 | 2.420790276 | 3.208093659 | 3.853264016 | 2.668717207 | 1.818695228 | 2.581446286 | 2.376849887 | 2.645876334 | 2.249828585 |
| ENSMUSG00000006764 | Tph2 | 2866 | 0 | 0 | 0 | 0 | 0 | 0 | 0 | 0 | 0 | 0 | 0 | 0 | 0 | 0.048615583 | 0 | 0 | 0 | 0 |
| ENSMUSG00000006777 | Krt23 | 1557 | 0.682485164 | 0.68465807 | 0.372821043 | 1.375317331 | 0.278219212 | 1.241205273 | 0 | 0 | 0 | 0 | 0 | 0 | 0 | 0 | 0 | 0 | 0 | 0 |

| ENSMUSG00000006782 | Cnp | 3633 | 67.51923228 | 76.95984618 | 70.7960531 | 76.82547691 | 69.34282645 | 73.3129488 | 46.42711268 | 45.08869087 | 45.01834685 | 48.40961276 | 58.032175 | 47.68836132 | 42.78691752 | 46.41851949 | 48.27828886 | 56.34505565 | 46.62874548 | 52.20792733 |
| --- | --- | --- | --- | --- | --- | --- | --- | --- | --- | --- | --- | --- | --- | --- | --- | --- | --- | --- | --- | --- |
| ENSMUSG00000006784 | Ttc25 | 6350 | 0.856797249 | 0.414443881 | 0.975088486 | 0.724671729 | 0.742823389 | 0.515036286 | 0.946253579 | 0.46312047 | 1.413962273 | 0.727680145 | 0.631983703 | 0.56255156 | 0.761342975 | 0.797229217 | 0.824031118 | 0.844202368 | 0.362138238 | 0.407154018 |
| ENSMUSG00000006800 | Sulf2 | 6692 | 8.536604391 | 14.10272405 | 11.87652576 | 10.55965029 | 11.40004468 | 13.46187532 | 3.294636638 | 3.133238034 | 2.548000228 | 1.268249639 | 1.263623379 | 1.309482819 | 2.454801024 | 3.386837215 | 1.890149082 | 0.909773846 | 0.950712087 | 2.359177171 |
| ENSMUSG00000006818 | Sod2 | 3819 | 50.6634096 | 48.83099812 | 47.689537 | 46.28878031 | 47.85465152 | 47.54155953 | 54.73354687 | 51.4032334 | 61.17044184 | 56.36107891 | 55.71868685 | 55.26028286 | 68.77274878 | 67.9088201 | 60.52388807 | 53.68104531 | 57.93603352 | 62.85931965 |
| ENSMUSG00000006850 | Tmco6 | 1805 | 7.394252229 | 5.610592711 | 6.699934952 | 5.729837709 | 5.679833652 | 4.639563421 | 4.613313846 | 6.495882293 | 6.411862011 | 4.414664183 | 5.796518508 | 5.040417175 | 7.543273777 | 7.0245074 | 10.203377 | 8.867298556 | 10.29820166 | 8.624696511 |
| ENSMUSG00000006906 | Stambp | 2147 | 37.71418739 | 39.5348181 | 41.74948847 | 37.75179221 | 42.46011338 | 42.49025734 | 34.81790065 | 33.46060034 | 35.96866188 | 38.38818318 | 43.68059781 | 37.7737111 | 38.50968484 | 40.3438393 | 39.64714228 | 43.46260374 | 44.41351965 | 42.6596007 |
| ENSMUSG00000006920 | Ezh1 | 8686 | 9.586419507 | 10.35898311 | 10.82083462 | 12.42621621 | 11.29875574 | 10.54264928 | 12.89850607 | 13.88574796 | 12.26681053 | 13.04434031 | 14.00357406 | 14.20131411 | 11.33055203 | 9.897306672 | 13.77021837 | 13.89500282 | 15.72587177 | 14.03409861 |
| ENSMUSG00000006930 | Hap1 | 4919 | 17.0400909 | 24.3057486 | 23.35579073 | 27.2680859 | 22.03559506 | 26.87674186 | 14.71608025 | 19.41841806 | 16.86311095 | 21.4138384 | 13.3641681 | 21.49112928 | 12.38563079 | 9.857212286 | 17.77387713 | 15.68521527 | 15.20118017 | 16.11468241 |
| ENSMUSG00000006931 | Leprel4 | 2027 | 16.96432531 | 19.1627206 | 15.75063149 | 16.49817787 | 16.47930687 | 18.11473882 | 14.91504777 | 13.28349467 | 18.02291045 | 18.63232033 | 18.66688067 | 15.99847063 | 12.65544992 | 16.74919974 | 16.34240303 | 9.18067844 | 14.82378726 | 14.38323955 |
| ENSMUSG00000006932 | Ctnnb1 | 4606 | 107.4072392 | 111.3081724 | 103.6050536 | 110.1833419 | 113.9972113 | 112.4350019 | 89.91052511 | 93.83092176 | 87.0675432 | 91.14847195 | 90.18742162 | 98.39843252 | 63.79090302 | 74.91957649 | 76.52612042 | 74.81881603 | 93.25288958 | 71.38285516 |
| ENSMUSG00000006941 | Eif1b | 913 | 69.04181385 | 55.38770364 | 64.37441334 | 56.93882978 | 57.62124049 | 56.60843496 | 74.36330149 | 67.93478807 | 76.73413287 | 64.81285059 | 75.29919524 | 63.15178472 | 70.40469684 | 64.90980888 | 71.79811109 | 68.44498497 | 59.65133395 | 72.48187885 |
| ENSMUSG00000006948 | Klk4 | 1243 | 0 | 0 | 0 | 0 | 0 | 0 | 0 | 0 | 0 | 0 | 0 | 0 | 0 | 0 | 0 | 0 | 0 | 0 |
| ENSMUSG00000006958 | Chrd | 4921 | 0.181387664 | 0.264011964 | 0.24575051 | 0.203686847 | 0.08802831 | 0.161114354 | 0.596095528 | 0.124177695 | 0.56912885 | 0.201212105 | 0.038833537 | 0.090738732 | 0.160396398 | 0.45302097 | 0.058608188 | 0 | 0 | 0.167676611 |
| ENSMUSG00000006998 | Psmd2 | 3755 | 77.76579476 | 85.4424725 | 77.82276881 | 88.8410031 | 85.9452858 | 89.73597559 | 69.16093066 | 70.1804465 | 58.91152494 | 71.69920522 | 68.36081276 | 67.27608225 | 58.58083066 | 59.579539 | 63.7279991 | 57.43081249 | 64.74133189 | 60.5466413 |
| ENSMUSG00000007021 | Syngr3 | 2066 | 1.419582355 | 2.273532471 | 1.96678439 | 1.918591896 | 2.213229791 | 1.463076838 | 0.93892538 | 0.961280215 | 1.295800351 | 1.072644266 | 0.878726245 | 0.945570276 | 0.620827613 | 0.15736138 | 0 | 0.537477064 | 0.426672231 | 1.304669037 |
| ENSMUSG00000007029 | Vars | 5708 | 42.40092367 | 54.725825 | 54.5854781 | 61.59680784 | 55.96558819 | 60.84707597 | 40.30032322 | 45.78003105 | 38.6538481 | 46.21727133 | 43.80766165 | 43.17206558 | 37.82862683 | 38.21791844 | 40.13326047 | 34.73524266 | 40.75694152 | 40.05218072 |
| ENSMUSG00000007030 | D17H6S56E-3 | 4425 | 1.508093251 | 2.687616551 | 1.508598235 | 1.544442181 | 1.59895882 | 2.508432163 | 0.64152785 | 0.578280443 | 0.465384359 | 0.905719724 | 0.248321818 | 0.52977578 | 0.479383603 | 0.146941745 | 0.68902037 | 0.597073878 | 0.606291702 | 0.484826025 |
| ENSMUSG00000007033 | Hspa1l | 2469 | 0.206586518 | 0.175402035 | 0.019592357 | 0.147625903 | 0.116967008 | 0.060209883 | 0.038325378 | 0.278437928 | 0.033362913 | 0.057291255 | 0.038699845 | 0.090426347 | 0.099902627 | 0.20691979 | 0.08343774 | 0.29466243 | 0.015523004 | 0 |
| ENSMUSG00000007034 | Slc44a4 | 2780 | 24.84565146 | 26.50651871 | 25.89201913 | 27.48412639 | 27.56331744 | 27.28965292 | 30.07248333 | 31.18588254 | 27.0971717 | 32.9885316 | 28.99149459 | 32.40520842 | 34.97597444 | 32.81157828 | 28.05559184 | 29.98512673 | 29.61326825 | 34.7467595 |
| ENSMUSG00000007035 | Msh5 | 5510 | 0.655705982 | 0.489717683 | 0.272155978 | 0.405170812 | 0.506651828 | 0.242817388 | 0.377814496 | 0.304984702 | 0.074748667 | 0.444979414 | 0.372835429 | 0.263376992 | 0.196969543 | 0.219155403 | 0.747759635 | 0.368312558 | 0.153026957 | 0.32945563 |
| ENSMUSG00000007036 | Abhd16a | 3344 | 70.41826411 | 71.86579512 | 64.25694433 | 75.15392361 | 76.15610291 | 70.56522647 | 81.83508819 | 80.30226714 | 69.79784235 | 81.005021 | 87.7493406 | 74.15938662 | 88.9862814 | 83.88824397 | 83.06844791 | 82.07747424 | 82.3832004 | 96.38108741 |
| ENSMUSG00000007038 | Neu1 | 3961 | 32.63272918 | 32.25320609 | 32.03326688 | 32.32177091 | 32.23781709 | 31.86337097 | 44.62513721 | 43.91981645 | 42.71502316 | 41.98448129 | 42.98660743 | 47.60043126 | 52.59497265 | 62.37882982 | 50.54237821 | 49.9005593 | 50.2276716 | 65.39709254 |
| ENSMUSG00000007039 | Ddah2 | 1757 | 136.7568356 | 127.734188 | 121.5257615 | 123.7434852 | 121.1379433 | 129.1699031 | 80.5150313 | 78.71069386 | 92.64035873 | 88.88055162 | 97.09981601 | 102.0375118 | 64.32522623 | 61.64348992 | 67.34824617 | 70.32657375 | 54.16288857 | 58.9539829 |
| ENSMUSG00000007041 | Clic1 | 1793 | 232.5338938 | 223.1016362 | 209.3038753 | 202.2930697 | 212.9025581 | 221.840375 | 140.0381411 | 158.37173 | 172.6250228 | 159.2026534 | 157.2869938 | 164.8632175 | 166.5124168 | 148.9681799 | 149.9846934 | 179.921008 | 157.2596577 | 155.5472231 |
| ENSMUSG00000007044 | Ly6g6c | 594 | 0 | 0 | 0 | 0 | 0 | 0 | 0 | 0 | 0 | 0 | 0 | 0 | 0 | 0 | 0 | 0 | 0 | 0.092607924 |
| ENSMUSG00000007050 | Lsm2 | 1805 | 15.18883021 | 19.02802988 | 14.79345637 | 13.17610257 | 16.29285616 | 14.19322064 | 18.45325538 | 14.57870651 | 14.07871481 | 17.21457809 | 17.15134243 | 14.53371824 | 16.09778353 | 16.21040169 | 15.45343675 | 13.85250229 | 14.20514827 | 13.37894618 |
| ENSMUSG00000007080 | Pole | 9649 | 0.251092863 | 0.131193904 | 0.060159847 | 0.061383934 | 0.084800778 | 0.092439549 | 0.318719466 | 0.142494195 | 0.298793254 | 0.171030637 | 0.237661727 | 0.092553695 | 0.148266722 | 0.115520582 | 0.064050507 | 0.21825924 | 0.22640677 | 0.12542236 |
| ENSMUSG00000007097 | Atp1a2 | 6946 | 0 | 0 | 0 | 0.013118642 | 0 | 0 | 0 | 0 | 0 | 0 | 0 | 0 | 0.007102205 | 0 | 0 | 0 | 0 | 0 |
| ENSMUSG00000007107 | Atp1a4 | 3447 | 0 | 0 | 0 | 0 | 0 | 0 | 0.027451511 | 0 | 0 | 0 | 0 | 0 | 0 | 0.040421312 | 0 | 0 | 0 | 0 |
| ENSMUSG00000007122 | Casq1 | 2575 | 0 | 0 | 0 | 0 | 0 | 0 | 0 | 0 | 0 | 0.054932858 | 0 | 0 | 0 | 0.126255772 | 0.032001208 | 0.029740278 | 0 | 0 |
| ENSMUSG00000007207 | Stx1a | 2705 | 1.099949102 | 1.564043462 | 0.91203329 | 1.499050997 | 1.281145473 | 1.355601584 | 0.979486144 | 1.567229821 | 1.141955151 | 1.499061176 | 0.794777498 | 1.217421106 | 1.696069614 | 1.253389419 | 1.111908902 | 1.910991497 | 1.671905004 | 2.257305304 |
| ENSMUSG00000007209 | Ceacam9 | 1371 | 0 | 0 | 0 | 0 | 0 | 0 | 0 | 0 | 0.300412223 | 0 | 0.313621172 | 0 | 0.215894605 | 0 | 0 | 0 | 0 | 0 |
| ENSMUSG00000007216 | Zfp775 | 4081 | 2.843399426 | 3.183540491 | 3.164845039 | 2.612419597 | 1.94603465 | 2.173472019 | 2.260713646 | 1.684546051 | 2.068913436 | 2.495602035 | 2.505229384 | 2.065220547 | 3.058312931 | 1.627420852 | 3.119647334 | 2.664673487 | 2.657765749 | 1.374890685 |
| ENSMUSG00000007279 | Scube2 | 4843 | 0.210638907 | 0.110057053 | 0.01997668 | 0.131706509 | 0.32796892 | 0.266027477 | 0.625234659 | 0.449507937 | 0.034017358 | 0.924905391 | 1.006203957 | 0.126775199 | 1.059368036 | 0.546626674 | 1.284624187 | 0.490195676 | 1.005046398 | 0.624716265 |
| ENSMUSG00000007338 | Mrpl49 | 2025 | 29.15540222 | 30.76301837 | 28.85690107 | 30.9140123 | 30.9232754 | 27.28459158 | 38.94826458 | 31.89301356 | 32.96955163 | 30.89826332 | 31.28375059 | 29.13439771 | 35.32408891 | 35.36631247 | 36.56256589 | 32.22083704 | 27.67064004 | 32.16334931 |
| ENSMUSG00000007379 | Dennd2c | 6916 | 0.178231652 | 0.568380786 | 0.559555007 | 0.289862052 | 0.361894163 | 0.487216004 | 0.287323961 | 0.546710214 | 0.613390851 | 0.872656162 | 0.352302325 | 0.597217905 | 0.534975968 | 0.315626389 | 0.345530689 | 0.852624861 | 0.847877881 | 0.397694525 |
| ENSMUSG00000007411 | Mark3 | 3397 | 45.79598006 | 45.79664881 | 47.76120719 | 45.72199003 | 47.79186886 | 51.9012735 | 45.62741718 | 41.84637084 | 37.25821694 | 43.48635658 | 48.53440733 | 44.33048503 | 41.54801746 | 43.02606506 | 37.24756495 | 49.03271775 | 44.97162775 | 46.58851929 |
| ENSMUSG00000007415 | Gatad1 | 4078 | 7.973629147 | 9.312579551 | 8.528829898 | 8.289920279 | 7.872484818 | 7.849697374 | 9.722419064 | 10.27392483 | 10.0188876 | 8.266982057 | 8.493586292 | 8.896562207 | 9.169591294 | 8.177257172 | 8.638384082 | 7.79333108 | 8.505468047 | 9.267105522 |
| ENSMUSG00000007440 | Pcdha4 | 24675 | 0.006890403 | 0.033751666 | 0.015683414 | 0.007385782 | 0.040963339 | 0.018073945 | 0.001917434 | 0 | 0.110164541 | 0.009554347 | 0 | 0.104053515 | 0.011995603 | 0 | 0.081818693 | 0 | 0 | 0.031210841 |
| ENSMUSG00000007457 | 2310003L06R | 1600 | 0 | 0 | 0 | 0 | 0 | 0 | 0 | 0 | 0 | 0 | 0 | 0 | 0 | 0 | 0 | 0 | 0 | 0 |
| ENSMUSG00000007458 | M6pr | 4330 | 46.89312376 | 54.80086502 | 54.67437819 | 57.30379849 | 58.0695541 | 51.30367312 | 48.55832452 | 53.60166961 | 46.94121369 | 52.7151396 | 56.48037239 | 49.77004001 | 45.59499606 | 40.84505422 | 50.04133543 | 52.17426875 | 54.63931427 | 50.20692604 |

| ENSMUSG00000007476 | Lrrc8a | 5762 | 8.92593943 | 7.562177306 | 6.833747607 | 6.428519446 | 7.275755903 | 7.593728523 | 4.647514105 | 5.19660595 | 5.039308159 | 5.024389413 | 5.538644961 | 5.434326052 | 5.950309356 | 3.715849408 | 5.248514756 | 6.851373869 | 4.616183626 | 3.694641496 |
| --- | --- | --- | --- | --- | --- | --- | --- | --- | --- | --- | --- | --- | --- | --- | --- | --- | --- | --- | --- | --- |
| ENSMUSG00000007480 | Mc5r | 1301 | 0 | 0 | 0.037181807 | 0 | 0 | 0 | 0 | 0.088068568 | 0 | 0 | 0 | 0.085804247 | 0.037918461 | 0 | 0 | 0 | 0.206213742 | 0.591950418 |
| ENSMUSG00000007564 | Ppp2r1a | 3075 | 130.7500683 | 148.8084276 | 148.2982994 | 148.9216444 | 156.6518023 | 148.1586447 | 142.9843009 | 143.3550272 | 140.5297312 | 136.7140391 | 151.2951368 | 137.7149372 | 142.2523937 | 138.8791491 | 139.6833236 | 144.9688622 | 160.8084171 | 144.8841481 |
| ENSMUSG00000007570 | Fance | 3084 | 6.160769677 | 6.329881975 | 6.180016524 | 7.815107799 | 8.34973384 | 7.43933711 | 8.453075904 | 8.854593943 | 7.171582025 | 8.592313629 | 9.589072405 | 9.700776636 | 8.142005802 | 6.611204398 | 8.884252486 | 7.325375605 | 8.077851163 | 9.649781692 |
| ENSMUSG00000007589 | Tinf2 | 2786 | 10.02365422 | 10.40280616 | 10.73038111 | 11.05501291 | 11.29873585 | 9.515690045 | 8.932688125 | 10.63794123 | 10.65882192 | 10.98377833 | 11.88372083 | 13.94389848 | 11.35023653 | 11.53600448 | 12.57046752 | 13.53777756 | 11.41809994 | 15.8748463 |
| ENSMUSG00000007591 | Tssk4 | 1685 | 0.454061228 | 0.830351073 | 0.315791593 | 0.216313564 | 0.628428677 | 0.058816301 | 1.432015801 | 1.201304052 | 0.92883537 | 0.559652264 | 0.623768004 | 0.496875335 | 0.995421476 | 0.110253026 | 0.586847087 | 0.681731883 | 0.363929229 | 0.750866145 |
| ENSMUSG00000007594 | Hapln4 | 3571 | 0 | 0 | 0.013546214 | 0 | 0 | 0 | 0 | 0.010695156 | 0 | 0 | 0 | 0 | 0 | 0 | 0 | 0 | 0 | 0 |
| ENSMUSG00000007603 | Dus3l | 2290 | 22.86741348 | 20.88965768 | 18.14535484 | 24.49154829 | 20.36673975 | 20.01584217 | 15.06154713 | 21.71463228 | 19.17241301 | 24.31658539 | 23.49109312 | 21.838792 | 18.00938115 | 22.89754352 | 20.11499541 | 17.17225057 | 21.97485932 | 23.22873633 |
| ENSMUSG00000007610 | Gtpbp3 | 3919 | 1.789577455 | 1.921080402 | 2.406950349 | 2.429767355 | 1.977351291 | 2.946105365 | 1.400426322 | 1.98807096 | 3.215890233 | 2.538606376 | 1.6335403 | 2.634829696 | 1.145497441 | 2.156882854 | 1.524426028 | 0.654623806 | 1.466941703 | 3.15821613 |
| ENSMUSG00000007613 | Tgfbr1 | 6610 | 5.227943738 | 6.19388003 | 7.010868696 | 5.913975195 | 6.553514569 | 6.679498581 | 6.033977055 | 7.499809126 | 6.635951481 | 5.492593269 | 6.519366497 | 6.772187805 | 2.343452651 | 3.976906727 | 5.4727634 | 3.776925269 | 4.893705686 | 5.192992828 |
| ENSMUSG00000007617 | Homer1 | 12527 | 0.91952604 | 1.132856465 | 1.243416363 | 0.876523644 | 1.340946864 | 1.337017957 | 1.397436833 | 1.47257367 | 1.890496253 | 1.087774666 | 1.13650017 | 1.452535005 | 0.88606062 | 1.345829306 | 1.503082226 | 0.773331498 | 0.755695325 | 1.312981792 |
| ENSMUSG00000007646 | Rad51c | 5269 | 0.750233701 | 0.644888064 | 1.312851555 | 0.838759025 | 0.712524207 | 0.978076351 | 1.436710691 | 0.971300422 | 1.438284096 | 0.876972655 | 1.405412519 | 1.218220003 | 0.411957556 | 0.185106441 | 0.641208499 | 0.537769016 | 0.574639866 | 0.532447228 |
| ENSMUSG00000007653 | Gabrb2 | 10181 | 1.34850917 | 0.870369302 | 2.081092849 | 1.499160186 | 1.115718887 | 1.382278395 | 2.133043869 | 1.459271637 | 2.354439856 | 1.106867506 | 1.614240813 | 2.001052632 | 0.02907293 | 0.136855183 | 0.169970076 | 0.312161911 | 0.203282588 | 0.091852943 |
| ENSMUSG00000007655 | Cav1 | 2380 | 79.93830584 | 94.12992169 | 85.60895372 | 65.91036792 | 83.5837145 | 75.78653371 | 120.5678142 | 154.5187575 | 138.5978171 | 117.2427991 | 150.5111927 | 123.4980165 | 157.6963137 | 127.7407546 | 138.2848859 | 127.983942 | 165.5921475 | 129.3638531 |
| ENSMUSG00000007656 | Arpp19 | 1879 | 82.45415999 | 79.01839784 | 83.20556153 | 80.45319058 | 88.47667075 | 80.2232121 | 92.48508233 | 83.27529146 | 80.20301287 | 87.02428893 | 77.70105009 | 82.28280222 | 79.10434633 | 88.31544664 | 89.96805951 | 91.8240963 | 91.01220691 | 87.41734576 |
| ENSMUSG00000007659 | Bcl2l1 | 3376 | 84.20456673 | 85.36429144 | 88.19285525 | 89.09775317 | 85.72786049 | 96.49279341 | 72.95906591 | 68.0019345 | 67.95287109 | 73.11431622 | 71.39356206 | 66.84322389 | 59.58991666 | 58.26146626 | 64.02350776 | 69.1181758 | 68.26304007 | 65.9261984 |
| ENSMUSG00000007670 | Khsrp | 4574 | 16.55973408 | 19.35847679 | 15.96939896 | 18.20848033 | 18.04684064 | 18.17872474 | 13.45732297 | 15.63934624 | 13.46170552 | 18.132507 | 13.47391702 | 15.14368983 | 14.85134458 | 11.97148645 | 13.13333373 | 13.385807 | 12.51847127 | 11.7618947 |
| ENSMUSG00000007682 | Dio2 | 5811 | 0 | 0 | 0 | 0 | 0 | 0.017054804 | 0 | 0 | 0 | 0 | 0 | 0 | 0 | 0 | 0 | 0 | 0 | 0 |
| ENSMUSG00000007721 | Ccdc124 | 906 | 84.77577603 | 72.72947546 | 65.56588883 | 66.98378704 | 76.97920242 | 70.93807445 | 87.62767687 | 78.95625795 | 79.41814905 | 81.86319672 | 75.5118549 | 77.62443141 | 71.22091368 | 78.73964485 | 68.39640183 | 66.56479764 | 58.29319772 | 68.54887582 |
| ENSMUSG00000007739 | Cct4 | 4377 | 49.82729225 | 43.89965133 | 47.61095928 | 47.45551774 | 49.979345 | 45.6469321 | 41.30266077 | 41.71758645 | 45.72201967 | 43.26187441 | 45.17718829 | 38.30711684 | 41.76926786 | 38.00838954 | 39.15889251 | 37.32831204 | 45.69909608 | 40.02833103 |
| ENSMUSG00000007777 | 0610009B22R | 995 | 77.1500984 | 65.95618252 | 74.67511822 | 63.32756213 | 74.30214548 | 71.81411238 | 81.21613629 | 77.61310318 | 84.27713175 | 83.87612539 | 89.83612795 | 72.81279401 | 86.02098138 | 73.56370023 | 74.4112522 | 85.35534394 | 83.47043758 | 76.45989407 |
| ENSMUSG00000007783 | Cpt1c | 2737 | 7.96680866 | 8.057411755 | 11.68246385 | 8.323172148 | 8.142155885 | 10.31971434 | 8.159146677 | 7.130550833 | 7.794890451 | 6.839177637 | 4.8350981 | 7.076373737 | 7.732331937 | 7.26272174 | 11.36542736 | 6.841105962 | 6.511409594 | 9.325621308 |
| ENSMUSG00000007805 | Twist2 | 1327 | 0 | 0 | 0 | 0 | 0 | 0 | 0 | 0 | 0 | 0 | 0 | 0 | 0 | 0.034999312 | 0 | 0 | 0 | 0 |
| ENSMUSG00000007812 | Zfp655 | 4583 | 7.224859726 | 8.97696364 | 8.296224058 | 8.718532813 | 10.08218344 | 7.838911596 | 7.236785496 | 8.533497723 | 8.869974047 | 7.911606111 | 7.057308944 | 5.541376059 | 6.210891614 | 8.441615709 | 10.15879515 | 7.318911648 | 10.35303416 | 7.585807429 |
| ENSMUSG00000007815 | Rhoa | 2120 | 153.7003205 | 162.2589367 | 159.4044731 | 154.5207117 | 165.7599881 | 151.8136814 | 156.2211097 | 163.9210705 | 170.1857915 | 153.8625307 | 148.4854724 | 155.2044014 | 148.484417 | 135.2136355 | 139.191294 | 162.5544654 | 144.0671038 | 134.2014623 |
| ENSMUSG00000007817 | Zmiz1 | 10261 | 7.659299335 | 9.466952494 | 8.59890063 | 10.38122226 | 8.246362129 | 9.890263948 | 7.252981576 | 8.635257146 | 6.518555852 | 9.208655036 | 7.072425878 | 7.343450394 | 5.586559582 | 5.13731987 | 6.693596498 | 7.500645247 | 6.473001904 | 5.977502582 |
| ENSMUSG00000007827 | Ankrd26 | 5670 | 1.731692357 | 2.009349175 | 1.817206696 | 1.422275987 | 1.774174964 | 1.258482123 | 1.285035724 | 1.677232487 | 2.832934947 | 1.629900853 | 2.072775976 | 1.791613861 | 2.323037374 | 1.539945049 | 2.274442151 | 2.120502773 | 2.717314172 | 1.057494292 |
| ENSMUSG00000007833 | Aldh16a1 | 6665 | 4.457782152 | 4.193476106 | 4.020845584 | 5.09955575 | 4.159638114 | 4.512904618 | 3.563535607 | 4.25760765 | 4.097023248 | 5.029879972 | 3.935251644 | 4.304463701 | 3.241917444 | 2.710690172 | 3.752339749 | 3.418291283 | 3.639991889 | 4.481612146 |
| ENSMUSG00000007836 | Hnrnpa0 | 2677 | 19.48220059 | 16.76222208 | 16.19076698 | 17.07049961 | 16.59535508 | 15.14162722 | 15.46454765 | 17.39131062 | 18.66239956 | 14.61900773 | 13.97377381 | 17.20131552 | 15.81127569 | 14.17438155 | 16.05275413 | 16.73515527 | 15.51950162 | 14.30195677 |
| ENSMUSG00000007837 | Prrg2 | 2152 | 12.60144159 | 13.85457264 | 13.2173029 | 12.53352149 | 14.42615742 | 13.07897429 | 14.92811756 | 14.19791911 | 11.55978416 | 11.98486742 | 13.16475389 | 12.9164498 | 15.88616109 | 13.31598602 | 11.04707144 | 15.15966427 | 12.66263806 | 14.08458076 |
| ENSMUSG00000007850 | Hnrnph1 | 3739 | 46.91598328 | 43.54999383 | 45.4755173 | 41.5155063 | 45.78904854 | 39.81182446 | 43.60510607 | 52.29876981 | 50.26319107 | 35.561643 | 44.54225869 | 47.441074 | 48.8569376 | 49.89727174 | 50.66722492 | 50.6206131 | 58.06859373 | 56.74515235 |
| ENSMUSG00000007867 | 1700019E19R | 854 | 149.6640097 | 139.5708847 | 138.6065911 | 128.6806077 | 137.5761425 | 130.8447473 | 116.8412645 | 108.3608794 | 142.513061 | 127.3731527 | 116.6402675 | 126.9904361 | 135.4028268 | 110.2367273 | 122.63976 | 132.8962065 | 119.6013832 | 131.9832081 |
| ENSMUSG00000007872 | Id3 | 1786 | 0.499780905 | 0.503610383 | 1.137563424 | 1.198974851 | 0.565940872 | 1.05431348 | 1.801378593 | 2.480581567 | 2.144650597 | 4.620029714 | 2.407472712 | 3.000169993 | 245.3337566 | 251.3597697 | 189.7209382 | 279.0540571 | 158.9489816 | 234.4201071 |
| ENSMUSG00000007877 | Tcap | 946 | 17.34354963 | 17.64245244 | 15.85179113 | 22.00993368 | 16.2305324 | 19.48585295 | 6.501742347 | 9.124189158 | 8.925196333 | 13.50723106 | 13.9385714 | 14.27842531 | 11.10750357 | 10.60456964 | 7.970279839 | 14.16671521 | 18.109783 | 16.92140598 |
| ENSMUSG00000007880 | Arid1a | 13569 | 6.919738658 | 6.685165521 | 6.253015863 | 6.665094923 | 5.686157686 | 5.664108614 | 5.659111055 | 5.499886086 | 6.538120344 | 5.469467678 | 5.372878373 | 6.096161246 | 6.645938889 | 5.319044278 | 5.101231771 | 6.036082927 | 4.894942334 | 5.039156876 |
| ENSMUSG00000007888 | Crlf1 | 1674 | 7.084196005 | 6.029753255 | 5.923879165 | 6.259880644 | 7.303171214 | 5.209845346 | 4.748225842 | 7.118297222 | 5.732651238 | 6.619125009 | 7.106311028 | 6.035026839 | 3.948910941 | 1.692407008 | 2.1412995 | 1.692655285 | 1.831603197 | 2.365983085 |
| ENSMUSG00000007891 | Ctsd | 2509 | 795.0619435 | 823.4219852 | 749.7415205 | 841.2722447 | 812.1422832 | 831.7117244 | 771.3531812 | 803.6083331 | 804.9673868 | 882.8964487 | 902.106988 | 772.0758811 | 1094.779256 | 982.4340574 | 999.29784 | 1136.448135 | 1083.18761 | 1049.60182 |
| ENSMUSG00000007892 | Rplp1 | 492 | 318.0112865 | 232.6485865 | 275.9847555 | 236.9729928 | 272.1605928 | 250.9866099 | 507.4573307 | 327.5852401 | 353.3501688 | 406.5310635 | 391.0360931 | 327.4065086 | 627.979264 | 628.411158 | 461.4239288 | 492.4856984 | 393.3898361 | 509.7286942 |
| ENSMUSG00000007907 | 4931407G18R | 1490 | 0 | 0 | 0 | 0 | 0 | 0 | 0 | 0 | 0 | 0.189868604 | 0 | 0 | 0 | 0 | 0 | 0 | 0 | 0 |
| ENSMUSG00000007908 | Hmgcll1 | 6675 | 0 | 0.084841828 | 0.036234854 | 0.143338117 | 0 | 0.096507196 | 0.014176084 | 0 | 0.080213439 | 0 | 0 | 0 | 0.059124395 | 0.424432859 | 0.037035106 | 0.022945682 | 0.223928926 | 0.057687453 |
| ENSMUSG00000007944 | Ttc9b | 911 | 0 | 0 | 0 | 0 | 0 | 0 | 0 | 0 | 0 | 0 | 0.052442325 | 0.061268565 | 0 | 0 | 0 | 0.042031402 | 0 | 0 |

| ENSMUSG00000007946 | Phox2a | 2046 | 0.457044904 | 0.276793354 | 0.283715721 | 0.489903703 | 0.094099558 | 0.726579671 | 0.092497906 | 0.597339627 | 0 | 0.069135929 | 0.093401678 | 0.518327267 | 0.07233419 | 0.249699395 | 0.120825677 | 0.037429724 | 0.224787665 | 0.026886171 |
| --- | --- | --- | --- | --- | --- | --- | --- | --- | --- | --- | --- | --- | --- | --- | --- | --- | --- | --- | --- | --- |
| ENSMUSG00000007950 | Abhd8 | 1980 | 8.157559036 | 10.48178439 | 7.280460629 | 11.39026108 | 7.754587702 | 8.859428125 | 8.60230526 | 7.850660494 | 9.381373035 | 9.430140651 | 7.697076644 | 10.20468175 | 9.368081261 | 8.678945623 | 8.677297377 | 9.379264944 | 5.710231104 | 8.084671746 |
| ENSMUSG00000007987 | Rabl5 | 906 | 26.27251498 | 21.62028896 | 25.68175287 | 22.52908153 | 25.07535096 | 24.66697886 | 32.06400095 | 23.31169816 | 31.50359318 | 31.7981012 | 27.52596945 | 27.23015769 | 33.81359893 | 31.16777608 | 31.15128674 | 27.21760614 | 29.65423193 | 27.99028499 |
| ENSMUSG00000007989 | Fzd3 | 4821 | 4.346619328 | 5.051177282 | 4.645705155 | 5.00878521 | 4.832161596 | 4.604775906 | 4.641961654 | 4.444293248 | 2.85341138 | 4.000132261 | 4.181919203 | 3.334351965 | 4.574023444 | 3.525935695 | 3.640709979 | 3.875921264 | 4.428074544 | 3.46873438 |
| ENSMUSG00000008028 | 1700008O03R | 3999 | 0.223207976 | 0.191597041 | 0.193542507 | 0.205075969 | 0.276827768 | 0.260216906 | 0.248453678 | 0.210110741 | 0.339873723 | 0.106115611 | 0.442028873 | 0.181446265 | 0.086352443 | 0.116139253 | 0.247271153 | 0.315976505 | 0.086255732 | 0.096290009 |
| ENSMUSG00000008035 | Mid1ip1 | 1968 | 33.21796785 | 32.61887559 | 32.96184157 | 41.25497002 | 32.28360734 | 32.63228797 | 41.15818411 | 44.55780281 | 43.71881678 | 37.25576401 | 32.69962859 | 45.23677938 | 59.78486926 | 77.40681232 | 49.68053462 | 51.40436217 | 54.49033472 | 65.49102492 |
| ENSMUSG00000008036 | Ap2s1 | 1404 | 90.67165399 | 84.96686196 | 82.79315772 | 72.20322197 | 82.85958654 | 76.23497474 | 92.6034485 | 90.33972107 | 87.03731643 | 101.3874453 | 90.9901987 | 87.18215673 | 112.0860514 | 105.9547093 | 103.1506189 | 117.8990711 | 91.83024415 | 86.58840873 |
| ENSMUSG00000008090 | Fgfrl1 | 2649 | 10.09277302 | 11.80853442 | 8.911394041 | 9.855220236 | 7.885713428 | 11.18630981 | 12.14519504 | 9.904937883 | 8.333700434 | 10.32367229 | 10.15375675 | 7.690719835 | 15.64319008 | 9.081931773 | 17.0778816 | 12.72017158 | 11.31414276 | 15.09687451 |
| ENSMUSG00000008129 | 4930432K21R | 2396 | 1.809485791 | 0.8620198 | 1.372871479 | 1.540252334 | 1.245483834 | 1.757922518 | 1.97465271 | 2.040328676 | 1.443934609 | 2.02692923 | 1.236246839 | 1.840332781 | 1.791267447 | 1.880248947 | 1.668009567 | 1.438294936 | 0.879777266 | 1.216812461 |
| ENSMUSG00000008136 | Fhl2 | 1557 | 0.08189822 | 0.021395565 | 0.093205261 | 0.204834496 | 0.247305966 | 0.063651552 | 0 | 0.024529481 | 0.079357448 | 0.302830464 | 0 | 0.215089259 | 0.316839546 | 0.05965843 | 0.132310713 | 0.024592555 | 0.049230953 | 0.035330191 |
| ENSMUSG00000008140 | 2310044H10R | 3942 | 44.29509465 | 46.87636333 | 41.44023643 | 46.47411448 | 45.10383715 | 48.52195627 | 46.53253582 | 48.51074552 | 44.42543506 | 47.74875885 | 47.09626805 | 39.41928073 | 39.22025082 | 37.07750964 | 36.21597949 | 35.78452503 | 38.13184588 | 39.67298083 |
| ENSMUSG00000008153 | Clstn3 | 5134 | 0 | 0.025954729 | 0 | 0.044371878 | 0 | 0.067563135 | 0.018431118 | 0.044634674 | 0 | 0 | 0 | 0.021743538 | 0.048044329 | 0.045231873 | 0 | 0.037291203 | 0.007465192 | 0 |
| ENSMUSG00000008167 | Fbxw9 | 3632 | 18.49068781 | 17.06918947 | 14.69052973 | 16.55852933 | 14.56414761 | 16.61762927 | 15.00666468 | 16.71969158 | 16.81707128 | 14.53986444 | 18.20499519 | 19.11747915 | 18.36364322 | 17.48047012 | 19.45502983 | 20.41041187 | 18.61442394 | 21.79463232 |
| ENSMUSG00000008193 | Spib | 2834 | 2.20474978 | 1.269510436 | 1.467933525 | 1.736271273 | 1.358699327 | 2.238091001 | 1.368962481 | 2.371864087 | 1.860223718 | 0.715412929 | 1.955502888 | 0.905970529 | 1.671088235 | 0.147493573 | 0.639685413 | 0.608001882 | 0.081142478 | 0.271745764 |
| ENSMUSG00000008200 | Fnbp4 | 6549 | 11.25423351 | 15.47887267 | 14.65461662 | 13.39212251 | 16.04397456 | 13.84661818 | 13.66858887 | 16.73140976 | 15.44572953 | 13.97457857 | 16.09277117 | 18.40919701 | 14.82443323 | 19.31092533 | 15.16826252 | 14.79237085 | 17.21142758 | 18.18517576 |
| ENSMUSG00000008206 | Lass4 | 5747 | 6.619476691 | 5.135762011 | 4.738872036 | 6.025125056 | 4.773829214 | 5.061328451 | 10.86701517 | 9.084568312 | 10.37008671 | 9.443267696 | 7.357027711 | 8.779773617 | 7.485197819 | 8.129937692 | 7.061690021 | 6.809291937 | 4.935002559 | 9.083633597 |
| ENSMUSG00000008226 | Scrn3 | 3362 | 6.890339362 | 6.539711536 | 6.417190523 | 7.439920684 | 6.728734124 | 7.118967971 | 13.00324668 | 11.00786375 | 9.016441285 | 10.06966239 | 11.9934756 | 10.3762014 | 9.699106877 | 10.51277528 | 9.889844023 | 11.45757021 | 12.36883764 | 9.800849175 |
| ENSMUSG00000008301 | Phax | 2259 | 29.61626696 | 23.75700424 | 27.70931752 | 26.21928182 | 26.54819694 | 27.92413893 | 27.91845994 | 27.89617706 | 31.77870605 | 29.30481955 | 30.26381832 | 26.38828138 | 30.79150215 | 26.41905812 | 23.54635177 | 31.62916043 | 26.72152175 | 26.64008975 |
| ENSMUSG00000008305 | Tle1 | 9188 | 10.39498591 | 9.640725482 | 10.60342731 | 11.59357005 | 10.71286285 | 11.64392161 | 7.353341914 | 7.918646776 | 10.41317764 | 9.45785221 | 9.338683656 | 8.273936922 | 6.926226958 | 6.753297862 | 6.506688898 | 6.701273041 | 5.944163375 | 7.741268502 |
| ENSMUSG00000008307 | 1700109H08R | 1011 | 0.420427063 | 1.482769772 | 0.47847211 | 0.901306515 | 0.428474098 | 1.029285267 | 0.374383216 | 0.566652855 | 0.162953574 | 0.513014576 | 0.850592733 | 2.318751561 | 0.878313067 | 0.229693805 | 0.978078478 | 0.757479869 | 0.379092947 | 0.816158853 |
| ENSMUSG00000008318 | Relt | 4013 | 0.01059187 | 0.049807467 | 0 | 0.079473539 | 0.023988001 | 0 | 0.212217349 | 0.209377736 | 0 | 0.14099388 | 0 | 0 | 0.135223297 | 0.370349065 | 0 | 0.238541038 | 0 | 0 |
| ENSMUSG00000008333 | Snrpb2 | 2886 | 16.20086404 | 13.10122486 | 13.39239458 | 12.62953412 | 14.12603581 | 13.82188168 | 17.63979298 | 14.94082547 | 19.77980279 | 15.52084827 | 14.03782564 | 14.36972879 | 18.54647618 | 21.24261793 | 16.87464971 | 18.49518597 | 17.33049808 | 21.8625937 |
| ENSMUSG00000008348 | Ubc | 4948 | 118.6244898 | 112.9056661 | 116.7788642 | 131.0386463 | 133.4425483 | 131.212594 | 114.1891697 | 116.0823645 | 117.9326102 | 132.7328508 | 133.1191092 | 120.2495884 | 134.9648675 | 142.166966 | 143.3477738 | 165.335252 | 179.0522795 | 190.4862641 |
| ENSMUSG00000008373 | Prpf31 | 5489 | 13.02490546 | 11.34301317 | 9.861537706 | 10.15972277 | 11.88171918 | 11.56441095 | 11.00715813 | 11.76595964 | 12.01304641 | 11.26153616 | 12.76842122 | 12.24304204 | 13.03175078 | 12.64118538 | 11.31185001 | 12.39613943 | 11.91892673 | 11.80556162 |
| ENSMUSG00000008384 | Sertad1 | 1341 | 21.52200934 | 20.94166281 | 19.62356489 | 21.60837002 | 25.48371797 | 19.10422316 | 22.22743305 | 34.34752968 | 22.26713194 | 27.53094754 | 27.68168735 | 25.389675 | 40.76045067 | 53.40550541 | 30.23290902 | 46.48553978 | 52.18778385 | 68.66908624 |
| ENSMUSG00000008393 | Carhsp1 | 2845 | 23.11265635 | 23.91034446 | 21.23674496 | 28.61778075 | 24.44661852 | 25.15084263 | 18.17671637 | 21.29108971 | 20.68735713 | 27.66064103 | 23.15700096 | 20.65866176 | 26.42595498 | 22.26704225 | 22.89618976 | 23.1358714 | 23.91183726 | 29.62177557 |
| ENSMUSG00000008398 | Elk3 | 5202 | 2.410424246 | 2.241351976 | 1.99929047 | 1.812982733 | 1.572938682 | 2.324272778 | 0.391089042 | 0.337725973 | 0.395871932 | 0.426005969 | 0.743900738 | 0.354078598 | 0.293981053 | 0.803530924 | 0.491060451 | 0.581499036 | 0.663084722 | 1.734235583 |
| ENSMUSG00000008429 | Herpud2 | 2736 | 35.3122314 | 36.04026568 | 35.46685011 | 32.60545497 | 32.63330351 | 37.11021605 | 28.01408621 | 34.64676271 | 34.63822104 | 33.19161348 | 35.06290812 | 40.61731879 | 27.00994594 | 26.00597291 | 29.93738783 | 26.10087091 | 30.52375766 | 26.3585303 |
| ENSMUSG00000008435 | Rdh13 | 6586 | 1.819990835 | 2.453196735 | 1.961089068 | 1.770972874 | 2.265547576 | 1.948703006 | 2.076125753 | 3.24745602 | 2.914199263 | 2.255158142 | 2.306777528 | 2.16957328 | 2.606666749 | 2.454075677 | 1.883034972 | 2.331389852 | 2.083330442 | 1.545199627 |
| ENSMUSG00000008438 | Adam21 | 2909 | 0.029223222 | 0.080161657 | 0.399094097 | 0 | 0 | 0 | 0 | 0.013129049 | 0.084949843 | 0.016208561 | 0 | 0 | 0 | 0.143690886 | 0 | 0.078976846 | 0 | 0 |
| ENSMUSG00000008450 | Nutf2 | 1961 | 22.32551316 | 24.07158139 | 23.08904865 | 22.76890538 | 20.00383365 | 24.28361906 | 19.92874696 | 19.94340646 | 22.32598993 | 21.88023456 | 18.80788778 | 17.84621134 | 22.18804234 | 21.07865261 | 23.15355155 | 24.3489992 | 20.59965168 | 23.0583813 |
| ENSMUSG00000008461 | Fut1 | 2578 | 11.85462435 | 13.77484299 | 16.81252257 | 11.11632928 | 15.98174036 | 12.59000795 | 7.047350158 | 7.733294824 | 8.419431267 | 5.395445097 | 6.115491194 | 8.595352229 | 1.913573207 | 2.72034802 | 3.020595059 | 1.336755107 | 2.155668367 | 0.341406403 |
| ENSMUSG00000008475 | Arpc5 | 2272 | 167.1957562 | 163.6466604 | 162.3877271 | 171.9768997 | 173.9909723 | 169.7486468 | 170.300655 | 167.7473519 | 171.1814561 | 172.7683119 | 172.7636703 | 157.2763521 | 169.3830487 | 165.6410388 | 158.7674392 | 177.7850429 | 180.3798824 | 157.4974645 |
| ENSMUSG00000008482 | Rnf151 | 1185 | 0.035869347 | 0 | 0 | 0 | 0 | 0.167266611 | 0 | 0 | 0 | 0 | 0.080632841 | 0 | 0 | 0 | 0.347692455 | 0 | 0 | 0.185684748 |
| ENSMUSG00000008489 | Elavl2 | 11068 | 0 | 0 | 0.034964605 | 0 | 0 | 0 | 0 | 0 | 0 | 0 | 0 | 0 | 0 | 0 | 0 | 0 | 0 | 0 |
| ENSMUSG00000008496 | Pou2f2 | 3568 | 0 | 0 | 0.067788019 | 0 | 0 | 0.083328588 | 0.609972879 | 0.010704149 | 0.34629918 | 0.118933949 | 0.241017167 | 0.078217016 | 1.02313954 | 0.169218928 | 0.09238017 | 0.429266899 | 0.02148335 | 0.154173505 |
| ENSMUSG00000008540 | Mgst1 | 2084 | 63.22746914 | 69.80681816 | 59.00456534 | 58.8970506 | 71.94383024 | 64.10468797 | 46.06402377 | 45.04650917 | 51.28551267 | 47.51270482 | 43.7402211 | 46.5219798 | 76.6017679 | 51.97102293 | 71.11420179 | 69.13990194 | 67.18136401 | 67.28321163 |
| ENSMUSG00000008575 | Nfib | 20790 | 10.06712298 | 10.50021144 | 10.77992141 | 10.55859123 | 9.913463066 | 11.37400888 | 7.905928167 | 8.78480367 | 7.595435374 | 8.014939168 | 8.040624325 | 8.642165362 | 6.385386695 | 6.205948762 | 6.314004669 | 7.672855721 | 6.612622751 | 6.371425156 |
| ENSMUSG00000008590 | Htr3b | 2402 | 0 | 0.221901044 | 0 | 0 | 0 | 0 | 0 | 0.159002508 | 0.051440278 | 0 | 0 | 0 | 0.164302805 | 0 | 0.171530208 | 0 | 0.095735962 | 0 |
| ENSMUSG00000008601 | Rab25 | 1728 | 64.00374307 | 53.45871277 | 61.22274936 | 56.37124583 | 56.01464033 | 57.20931915 | 59.3051288 | 55.74145768 | 60.58803418 | 59.92068539 | 53.58094302 | 62.8895226 | 59.12407447 | 59.4796096 | 57.22438318 | 49.72460822 | 50.25890555 | 44.66306524 |
| ENSMUSG00000008604 | Ubqln4 | 3361 | 15.27707606 | 15.75944713 | 14.79559334 | 17.86654461 | 16.18240814 | 14.4928108 | 16.63897248 | 14.52243091 | 15.08497498 | 15.10898763 | 15.97710602 | 14.79671388 | 12.82835338 | 15.17274857 | 13.53362621 | 15.24333017 | 12.24708208 | 10.68757712 |

| ENSMUSG00000008658 | Rbfox1 | 4213 | 0.635610275 | 0.616759019 | 0.264085259 | 0.49746215 | 0.479834036 | 0.458712701 | 0.774881283 | 1.377936189 | 0.62566746 | 0.928912502 | 1.020590141 | 0.84789993 | 0.854789927 | 1.56540717 | 1.242012247 | 1.027020801 | 1.26450398 | 0.56145065 |
| --- | --- | --- | --- | --- | --- | --- | --- | --- | --- | --- | --- | --- | --- | --- | --- | --- | --- | --- | --- | --- |
| ENSMUSG00000008668 | Rps18 | 2514 | 30.16278203 | 24.18298805 | 24.74477328 | 26.60446026 | 25.92308061 | 22.52934546 | 34.10126262 | 28.56074643 | 25.72109377 | 27.27013625 | 25.12271086 | 24.28891602 | 48.23303606 | 51.21042571 | 32.38435737 | 30.73605638 | 30.53602733 | 33.76255039 |
| ENSMUSG00000008682 | Rpl10 | 1731 | 91.37016753 | 78.51912676 | 79.78418785 | 82.46259498 | 86.36496529 | 80.81303691 | 99.10789936 | 100.390197 | 88.13105395 | 89.04428016 | 90.27838774 | 90.31754529 | 138.3345618 | 142.0421714 | 110.7276938 | 104.983953 | 98.90442996 | 127.4329971 |
| ENSMUSG00000008683 | Rps15a | 9620 | 4.281446525 | 2.763377296 | 3.610415259 | 2.945838833 | 3.662429119 | 3.842654808 | 4.150925261 | 3.473841175 | 3.994492641 | 3.053522675 | 3.640233323 | 3.069281238 | 4.420385935 | 4.934080802 | 3.983102597 | 3.132506032 | 3.127457699 | 3.854068392 |
| ENSMUSG00000008686 | Zfp955a | 1634 | 1.768881255 | 5.504578604 | 3.937380378 | 2.202767749 | 2.916193662 | 3.851405853 | 3.474615344 | 3.926758632 | 3.982539468 | 3.751279941 | 4.151801777 | 2.322806032 | 2.686989374 | 2.558119868 | 5.471687655 | 2.038728791 | 3.283770848 | 2.625893711 |
| ENSMUSG00000008690 | Ncaph2 | 4139 | 29.93539215 | 33.264603 | 28.65713854 | 34.72942616 | 28.86287377 | 30.10996012 | 29.85762681 | 31.9638758 | 32.84771407 | 30.58701093 | 30.54180723 | 36.26197552 | 34.14736482 | 33.11343365 | 32.01357906 | 32.15707933 | 29.96470084 | 27.93649247 |
| ENSMUSG00000008730 | Hipk1 | 13193 | 6.40815547 | 6.166155361 | 7.542208136 | 6.623670358 | 6.490312454 | 5.923191151 | 5.712809636 | 6.823276925 | 7.888905126 | 5.789747539 | 5.68533956 | 6.087981389 | 5.470521866 | 6.72740476 | 5.305953419 | 6.628950755 | 5.429534519 | 4.895072485 |
| ENSMUSG00000008734 | Gprc5b | 4521 | 0.02820516 | 0.029473917 | 0.08559793 | 0.050388237 | 0.042585201 | 0.109605692 | 0.010465092 | 0.152059996 | 0 | 0.12515117 | 0 | 0.024691733 | 0.087293815 | 0.205459356 | 0.236947678 | 0.287962984 | 0.203457449 | 0 |
| ENSMUSG00000008763 | Man1a2 | 13145 | 15.0489988 | 15.17010154 | 14.43661922 | 14.67519678 | 13.77499405 | 14.38895276 | 11.08581598 | 11.05240766 | 9.362138387 | 11.74732242 | 11.2668217 | 11.23107094 | 9.637456338 | 8.15112283 | 9.221375232 | 10.27976824 | 9.919061397 | 9.733829002 |
| ENSMUSG00000008789 | Ceacam5 | 3244 | 0 | 0 | 0 | 0 | 0 | 0 | 0 | 0 | 0 | 0 | 0 | 0 | 0 | 0 | 0 | 0 | 0 | 0 |
| ENSMUSG00000008813 | Tppp2 | 737 | 0.519059137 | 0.045200671 | 0.196907179 | 0.061819599 | 0.195923706 | 0.537885846 | 0 | 0 | 0 | 0.063976531 | 0 | 0 | 0.066936116 | 0 | 0 | 0 | 0 | 0 |
| ENSMUSG00000008822 | Acyp1 | 1793 | 52.24841493 | 38.21786024 | 41.87156671 | 45.17988669 | 48.31983413 | 47.12069757 | 63.67289138 | 45.28558144 | 48.1236757 | 54.51389172 | 53.26388288 | 43.64392579 | 50.95522074 | 47.42840157 | 45.79737919 | 42.1986839 | 44.46106947 | 48.44360263 |
| ENSMUSG00000008843 | Cldn13 | 1065 | 0 | 0.031279713 | 0 | 0 | 0 | 0 | 0 | 0 | 0 | 0 | 0 | 0 | 0 | 0 | 0 | 0 | 0 | 0 |
| ENSMUSG00000008845 | Cd163 | 4475 | 0 | 0 | 0 | 0.020362478 | 0 | 0.011073237 | 0 | 0.03413846 | 0.092036907 | 0.01053647 | 0 | 0.012472774 | 0 | 0 | 0 | 0.059895922 | 0 | 0 |
| ENSMUSG00000008855 | Hdac5 | 7756 | 16.30936099 | 18.57206738 | 15.04968138 | 18.74488041 | 13.5285706 | 18.81547194 | 14.75013637 | 18.13105024 | 15.71311245 | 22.1406474 | 12.79994375 | 14.84627539 | 15.43689572 | 12.38948128 | 15.03357442 | 19.130493 | 16.09941662 | 16.81621867 |
| ENSMUSG00000008859 | Rala | 2679 | 68.77937218 | 55.77018685 | 61.62704707 | 56.24127421 | 61.04974865 | 56.02658828 | 68.84091917 | 69.0285974 | 70.91952117 | 60.01638601 | 59.61615759 | 68.33720541 | 56.71605271 | 54.74820007 | 56.36569703 | 61.93101974 | 55.5795683 | 51.45682025 |
| ENSMUSG00000008874 | Clec3a | 588 | 0 | 0 | 0 | 0 | 0 | 0 | 0 | 0 | 0 | 0 | 0 | 0 | 0 | 0 | 0 | 0 | 0 | 0 |
| ENSMUSG00000008892 | Vdac3 | 1330 | 98.49695678 | 96.6825351 | 96.89254501 | 93.79408977 | 101.800828 | 91.31860902 | 89.68064931 | 87.72766116 | 88.04004835 | 89.62178785 | 92.67623524 | 80.11436081 | 91.17131779 | 82.37714447 | 92.87388313 | 88.35629633 | 90.68635816 | 79.90796556 |
| ENSMUSG00000008932 | Slc1a7 | 3349 | 0 | 0 | 0.028888343 | 0 | 0 | 0 | 0 | 0 | 0 | 0 | 0 | 0 | 0 | 0 | 0.012302644 | 0 | 0 | 0 |
| ENSMUSG00000008958 | Vps72 | 3650 | 36.81064697 | 35.83189663 | 34.81568883 | 33.74013776 | 35.97366788 | 35.21638107 | 30.00790458 | 32.79314771 | 32.08034639 | 36.70004054 | 30.69377469 | 30.76742825 | 33.00508 | 32.82897136 | 34.41740929 | 39.38162745 | 32.15208798 | 33.65351652 |
| ENSMUSG00000008976 | Gabpa | 4457 | 5.50268938 | 6.203657663 | 8.834654186 | 8.453889088 | 7.851000338 | 9.138960512 | 4.755683231 | 7.112338792 | 4.121423834 | 7.468789881 | 7.213943697 | 5.29729084 | 5.810916888 | 4.897626447 | 5.879333531 | 4.90552767 | 7.550031115 | 5.46758678 |
| ENSMUSG00000008999 | Bmp7 | 4012 | 19.46211574 | 17.91024747 | 25.07899879 | 24.90413017 | 22.87825983 | 21.02162327 | 31.29806826 | 25.67420471 | 23.24184239 | 32.94202922 | 33.65205201 | 31.77541709 | 19.93196359 | 17.89694893 | 22.14121498 | 26.20787835 | 29.47074425 | 23.3637881 |
| ENSMUSG00000009013 | Dynll1 | 3324 | 47.90144085 | 41.81149058 | 40.87883477 | 32.8823542 | 38.69088449 | 37.43288629 | 47.7966233 | 39.52522993 | 44.19744336 | 41.64695089 | 35.70186428 | 39.1246973 | 63.31226206 | 57.95730279 | 55.05935959 | 63.33386266 | 47.66573748 | 49.11763801 |
| ENSMUSG00000009030 | Pdcl | 5540 | 10.81810437 | 12.88619717 | 15.00103342 | 14.93481165 | 13.24931112 | 13.01430097 | 11.58050408 | 13.72582549 | 11.03263346 | 11.5919238 | 11.9178687 | 13.15798833 | 9.786241351 | 9.749904988 | 9.348439667 | 11.31439067 | 11.42178993 | 9.800358905 |
| ENSMUSG00000009035 | Tmem184b | 3352 | 11.1715573 | 9.888523195 | 12.07895134 | 11.7708426 | 9.319098586 | 11.35337094 | 8.962873246 | 6.722409731 | 9.17849859 | 8.116335256 | 8.537350874 | 7.759576004 | 6.593287273 | 4.918750305 | 5.420610426 | 6.419845274 | 7.397707069 | 6.564332545 |
| ENSMUSG00000009047 | Gm5965 | 586 | 0 | 0 | 0 | 0 | 0 | 0 | 0 | 0 | 0 | 0 | 0 | 0 | 0 | 0 | 0 | 0 | 0 | 0 |
| ENSMUSG00000009070 | Rtdr1 | 3004 | 0 | 0.088716096 | 0 | 0 | 0 | 0.016495584 | 0 | 0 | 0 | 0 | 0 | 0 | 0.016422076 | 0 | 0 | 0.01274654 | 0.012758421 | 0.036623906 |
| ENSMUSG00000009073 | Nf2 | 9019 | 6.833629586 | 7.224750093 | 6.323582691 | 8.456501632 | 6.436090469 | 8.845759071 | 6.153428582 | 6.500203758 | 5.776798142 | 7.502079962 | 7.749724388 | 6.665203305 | 6.016754519 | 7.013731791 | 6.208328468 | 5.761210139 | 5.919562212 | 7.288599904 |
| ENSMUSG00000009075 | Cabp7 | 2623 | 0 | 0.076201817 | 0.018442063 | 0 | 0.091749759 | 0 | 0 | 0 | 0.094212388 | 0 | 0 | 0 | 0.131652086 | 0 | 0.047123396 | 0 | 0 | 0 |
| ENSMUSG00000009076 | Zmat5 | 1255 | 18.11973639 | 16.77589573 | 19.38795679 | 19.05940102 | 19.6746429 | 17.29410144 | 23.71288848 | 17.58980764 | 20.31430538 | 20.81393594 | 20.9372328 | 17.78985262 | 24.60699619 | 17.20836704 | 18.35192808 | 18.42830826 | 14.71974112 | 17.09446344 |
| ENSMUSG00000009079 | Ewsr1 | 6125 | 43.34487012 | 48.57971774 | 48.57097334 | 48.21660235 | 44.2735115 | 44.0190079 | 44.82538381 | 43.66716637 | 44.25280983 | 43.97909677 | 45.89516011 | 44.7618832 | 42.41336757 | 44.58632389 | 45.12999812 | 41.69143849 | 46.50465936 | 47.92303567 |
| ENSMUSG00000009090 | Ap1b1 | 5027 | 28.13103653 | 34.67801382 | 34.77659413 | 39.60647777 | 33.26244337 | 35.96935047 | 32.9504056 | 35.37374689 | 32.51825762 | 31.9465477 | 36.79821747 | 30.7891053 | 36.36842758 | 38.67414955 | 39.95575591 | 32.63880102 | 40.82699819 | 35.77178633 |
| ENSMUSG00000009092 | Derl3 | 1329 | 0.735605003 | 3.609523527 | 2.038312791 | 2.262625227 | 1.37623258 | 2.013429355 | 3.310819519 | 3.85085171 | 0.898727582 | 1.028871629 | 2.516363502 | 1.553934925 | 2.115815865 | 1.502705612 | 0.496030771 | 2.333739052 | 1.384245486 | 0.703652983 |
| ENSMUSG00000009093 | Gstt4 | 1266 | 0.033574389 | 0 | 0.152838958 | 0 | 0 | 0 | 0 | 0 | 0 | 0 | 0 | 0 | 0 | 0 | 0 | 0 | 0 | 0 |
| ENSMUSG00000009097 | Tbx1 | 1803 | 0 | 0 | 0 | 0 | 0.080086395 | 0 | 0 | 0.021182697 | 0.068529976 | 0 | 0 | 0 | 0 | 0.025759339 | 0.068555001 | 0.233608808 | 0 | 0 |
| ENSMUSG00000009108 | Gnat2 | 3178 | 0.200622291 | 0.031447037 | 0.03044275 | 0.344073337 | 0.01514535 | 0 | 0 | 0.060088739 | 0.349916905 | 0.029673193 | 0.060132106 | 0 | 0.263890055 | 0.131528253 | 0 | 0 | 0 | 0 |
| ENSMUSG00000009112 | Bcl2l13 | 6926 | 6.720064648 | 9.114631035 | 7.962146203 | 9.545058523 | 9.965518126 | 9.107801017 | 7.070260279 | 7.549147978 | 7.225182883 | 8.182954854 | 8.525822795 | 8.034683165 | 5.769398353 | 4.834852297 | 6.002363542 | 6.147726744 | 7.359800012 | 6.020344051 |
| ENSMUSG00000009114 | 2610028H24R | 1887 | 0.901010621 | 1.535888605 | 0.333256966 | 0.579472742 | 1.071298778 | 0.525201204 | 1.604669556 | 1.679898993 | 2.575521388 | 1.224369083 | 0.962081835 | 0.946529519 | 1.699297627 | 0.861443062 | 1.834091518 | 1.846552874 | 2.884119852 | 3.410739527 |
| ENSMUSG00000009115 | 1700027D21R | 1586 | 0 | 0.021004347 | 0 | 0 | 0 | 0 | 0 | 0 | 0 | 0 | 0 | 0 | 0 | 0.175702727 | 0 | 0 | 0 | 0.034684178 |
| ENSMUSG00000009145 | Dqx1 | 3137 | 0.392939147 | 0.371677175 | 0.10794221 | 0.043571289 | 0.383582433 | 0.552867604 | 0.226232127 | 0 | 0.131292687 | 0.345701682 | 0.350278624 | 0.462609891 | 0.110080784 | 0.10363679 | 0.210145009 | 0.012206123 | 0.268784996 | 0.052606733 |
| ENSMUSG00000009185 | Ccl8 | 508 | 0 | 0.131153127 | 0 | 0.17937419 | 0.473739406 | 0.975447511 | 4.191083763 | 4.886823142 | 50.75346234 | 0.18563269 | 0 | 0.988860164 | 0 | 0 | 0.567737975 | 0.376876057 | 0.075445466 | 0 |

| ENSMUSG00000009207 | Lnp | 6881 | 2.341151245 | 2.536834264 | 2.439415061 | 2.807423746 | 2.553139389 | 3.110998533 | 2.434048589 | 1.881590527 | 3.172337357 | 2.274964901 | 2.082907651 | 2.157675665 | 2.157957724 | 2.888834385 | 3.430968104 | 2.520802958 | 3.013301355 | 1.47895433 |
| --- | --- | --- | --- | --- | --- | --- | --- | --- | --- | --- | --- | --- | --- | --- | --- | --- | --- | --- | --- | --- |
| ENSMUSG00000009210 | 2310007L24R | 1269 | 0 | 0.840041462 | 0.266835865 | 0.466740407 | 0 | 0 | 0 | 0 | 0 | 0 | 0 | 0 | 0 | 0 | 0 | 0.090521531 | 0.060403935 | 0 |
| ENSMUSG00000009214 | Tmem8c | 2204 | 0.05785641 | 0 | 0 | 0 | 0 | 0 | 0 | 0 | 0 | 0 | 0 | 0 | 0 | 0 | 0 | 0 | 0 | 0 |
| ENSMUSG00000009216 | Fam163b | 2956 | 0 | 0 | 0 | 0 | 0 | 0 | 0 | 0 | 0 | 0 | 0 | 0 | 0 | 0 | 0 | 0 | 0 | 0 |
| ENSMUSG00000009246 | Trpm5 | 6371 | 0 | 0.010457666 | 0 | 0.007151318 | 0.098212998 | 0.046667148 | 0 | 0.029973632 | 0 | 0.051805827 | 0.022496449 | 0.008760895 | 0 | 0.014579842 | 0 | 0 | 0.018047228 | 0.069074377 |
| ENSMUSG00000009248 | Ascl2 | 2119 | 0.060177219 | 0 | 0 | 0.150508405 | 0 | 0 | 0.0669835 | 0.054071358 | 0 | 0.022251394 | 0 | 0 | 0.232807538 | 0 | 0 | 0 | 0 | 0 |
| ENSMUSG00000009281 | Rarres2 | 702 | 0.060548684 | 0.189817061 | 0 | 0 | 0 | 0.282351758 | 0 | 0 | 0 | 0 | 0 | 0 | 0.070273386 | 0 | 0 | 0 | 0 | 0 |
| ENSMUSG00000009291 | Pttg1ip | 3588 | 147.1569946 | 141.5612314 | 144.7296121 | 148.873379 | 145.4957759 | 151.2546093 | 152.2893336 | 156.1758439 | 141.4437701 | 164.4361064 | 163.4308362 | 167.7892243 | 189.6281502 | 175.8739731 | 183.052955 | 209.5845147 | 193.2978452 | 175.4066862 |
| ENSMUSG00000009292 | Trpm2 | 12123 | 0.364640626 | 0.376463459 | 0.327198671 | 0.315691473 | 0.297772356 | 0.277949838 | 0.476131884 | 0.52926863 | 0.251406596 | 0.217804123 | 0.299504813 | 0.345308479 | 0.032554264 | 0.045972866 | 0.214113505 | 0.129498878 | 0.180202831 | 0.18150328 |
| ENSMUSG00000009293 | Ube2g2 | 2521 | 31.09065632 | 31.7800518 | 30.37496968 | 30.7053607 | 30.14688912 | 28.83532731 | 27.06262714 | 27.22401711 | 21.92475373 | 26.93257148 | 35.83595656 | 30.31005239 | 31.93561642 | 35.27982045 | 34.94205732 | 29.35967636 | 34.51039031 | 35.82901755 |
| ENSMUSG00000009350 | Mpo | 3689 | 0.023044281 | 0 | 0 | 0 | 0 | 0 | 0 | 0.238120156 | 0 | 0 | 0 | 0 | 0 | 0 | 0 | 0 | 0 | 0 |
| ENSMUSG00000009356 | Lpo | 3121 | 0.054476355 | 0.234823349 | 0.061997476 | 0.248169738 | 0.10795369 | 0.190526371 | 0.545740609 | 0.4527776 | 0 | 0.16618319 | 0.35207435 | 0.482865392 | 1.817741265 | 0.863107039 | 1.267333984 | 1.030570657 | 0.552606011 | 0.475887818 |
| ENSMUSG00000009376 | Met | 7964 | 17.84747723 | 20.75572339 | 18.42859004 | 24.88580398 | 19.1826627 | 21.04933422 | 15.77287325 | 21.58033787 | 15.6026768 | 20.37237191 | 17.51668493 | 21.01847967 | 12.43828351 | 13.94372337 | 18.41239797 | 15.28451041 | 17.71460308 | 17.49599037 |
| ENSMUSG00000009378 | Slc16a12 | 4044 | 2.38592358 | 2.70193603 | 1.901926639 | 2.241999956 | 3.130241327 | 3.051095613 | 1.240144403 | 0.349435927 | 0.224061164 | 0.338123243 | 0.047255152 | 1.090365318 | 0.683132386 | 0.367510088 | 0.275084572 | 0.511298912 | 1.184665458 | 0.122423828 |
| ENSMUSG00000009394 | Syn2 | 5683 | 87.67300253 | 98.92458259 | 95.41068129 | 92.37275257 | 94.55301704 | 111.6615003 | 64.33791708 | 71.29069237 | 64.37793762 | 73.25242989 | 64.99176562 | 68.32827109 | 77.44841913 | 91.52337404 | 83.62835601 | 83.39307545 | 85.88516471 | 83.94139953 |
| ENSMUSG00000009406 | Elk1 | 5375 | 2.641251869 | 2.057652257 | 2.591921254 | 2.737900897 | 2.847618926 | 3.014650024 | 3.547350625 | 2.401680374 | 2.406058781 | 2.938694994 | 2.799834776 | 2.315700978 | 2.679985087 | 3.283488969 | 2.291956318 | 2.429227372 | 2.110620257 | 3.653628112 |
| ENSMUSG00000009418 | Nav1 | 12737 | 10.78897967 | 12.78428413 | 11.31766668 | 12.33728837 | 10.70186134 | 11.56243418 | 8.123799075 | 10.6178297 | 10.35725917 | 11.65718494 | 11.17762238 | 8.619722722 | 8.462765113 | 7.781399269 | 8.151677855 | 10.37156281 | 9.475505138 | 7.678903333 |
| ENSMUSG00000009470 | Tnpo1 | 11095 | 10.6885481 | 10.4787743 | 10.61645303 | 9.84726312 | 10.59379519 | 9.843553385 | 8.976402807 | 7.83813432 | 8.482306311 | 7.292528778 | 7.277123013 | 7.440411443 | 6.887349244 | 6.91114811 | 6.338986562 | 8.486399603 | 8.487400765 | 8.8153395 |
| ENSMUSG00000009471 | Myod1 | 1851 | 0 | 0 | 0 | 0 | 0 | 0 | 0 | 0 | 0 | 0 | 0 | 0 | 0 | 0 | 0 | 0 | 0 | 0 |
| ENSMUSG00000009487 | Otog | 9988 | 0 | 0 | 0 | 0 | 0 | 0 | 0 | 0 | 0 | 0 | 0 | 0 | 0 | 0 | 0 | 0 | 0 | 0 |
| ENSMUSG00000009535 | Rnmt | 7359 | 9.784450087 | 9.456534315 | 9.314484646 | 9.429198332 | 10.11169371 | 8.73350937 | 7.901519555 | 8.703445962 | 8.926823297 | 8.348602574 | 8.783736761 | 7.319216523 | 7.09913037 | 8.658960171 | 7.866311459 | 8.881922385 | 9.921401767 | 6.899497962 |
| ENSMUSG00000009545 | Kcnq1 | 3260 | 138.0375149 | 137.8193265 | 141.7819272 | 159.5335341 | 148.7217384 | 168.6920973 | 99.21149483 | 106.5404011 | 87.91931386 | 104.758449 | 117.224507 | 114.918628 | 76.93357897 | 75.05136576 | 79.71110828 | 83.19397921 | 87.32752558 | 86.32717485 |
| ENSMUSG00000009549 | Srp14 | 1608 | 269.3051824 | 237.8308617 | 259.9174765 | 231.7436453 | 255.1770207 | 229.9518022 | 321.6261775 | 274.1872471 | 300.3183441 | 291.0852184 | 267.9913716 | 246.3803315 | 455.4612213 | 462.3035216 | 419.1643363 | 510.5890572 | 417.1560002 | 438.4650005 |
| ENSMUSG00000009551 | 6330409D20R | 1846 | 0 | 0 | 0 | 0 | 0 | 0 | 0 | 0 | 0 | 0 | 0 | 0 | 0 | 0 | 0 | 0 | 0.020761808 | 0 |
| ENSMUSG00000009555 | Cdk9 | 5316 | 20.05323203 | 20.59809693 | 18.67240111 | 19.19803223 | 17.7371028 | 19.44450757 | 19.77591659 | 17.57310314 | 18.79580272 | 18.32455849 | 19.37599896 | 17.61826219 | 19.51561739 | 21.09903519 | 18.72516159 | 18.63389792 | 19.04058505 | 21.92706869 |
| ENSMUSG00000009563 | Tor2a | 1909 | 12.15705925 | 13.52409273 | 13.04995712 | 15.39385731 | 12.45530136 | 14.06892698 | 15.04389529 | 16.32530139 | 17.3030831 | 17.06712203 | 18.89475833 | 16.84118473 | 13.33434747 | 17.51688995 | 15.71227485 | 11.91441634 | 17.40644286 | 13.11112811 |
| ENSMUSG00000009566 | Fpgs | 3786 | 5.736963801 | 7.364736519 | 6.81011402 | 6.751121467 | 7.538888203 | 6.792886616 | 8.260348857 | 8.715857284 | 7.887011074 | 8.256977353 | 12.69456107 | 8.993014838 | 8.495618083 | 13.37138279 | 8.194604222 | 6.230061849 | 6.316854006 | 8.354526246 |
| ENSMUSG00000009569 | Mkl2 | 9018 | 5.203561137 | 4.91307932 | 5.519667635 | 5.355367819 | 5.598845412 | 4.983846679 | 4.039782965 | 3.798911616 | 3.808999129 | 4.862514308 | 3.607756346 | 5.236199883 | 3.88944258 | 3.625708308 | 4.573381842 | 4.844708699 | 4.037478605 | 4.300445802 |
| ENSMUSG00000009575 | Cbx5 | 12103 | 3.473322242 | 3.300186745 | 2.817759142 | 2.668989543 | 2.934921893 | 3.24264769 | 3.682437706 | 3.385974368 | 3.001446493 | 3.202336452 | 3.414470717 | 3.767776281 | 3.134449672 | 4.386151523 | 3.199988642 | 3.742690949 | 4.237014398 | 3.604248668 |
| ENSMUSG00000009580 | Odam | 1364 | 0 | 0.170960601 | 0.035464465 | 0.200415151 | 0.070574668 | 0.072657967 | 29.06746697 | 16.01616875 | 19.6873961 | 13.27409828 | 5.709177589 | 15.67257974 | 42.89417441 | 46.88673632 | 27.60866722 | 6.203976712 | 7.69897753 | 23.79426171 |
| ENSMUSG00000009585 | Apobec3 | 6192 | 17.56633486 | 9.888420474 | 42.9049465 | 8.461757265 | 10.77371547 | 24.43225058 | 11.43084103 | 10.62750474 | 48.85577491 | 7.447252549 | 9.004098279 | 27.98895871 | 19.20853562 | 7.673175291 | 37.82797888 | 10.26524682 | 8.931661245 | 21.03707441 |
| ENSMUSG00000009588 | St6galnac1 | 2642 | 0 | 0.012608968 | 0 | 0 | 0.018217988 | 0 | 0 | 0.072279338 | 0.031178286 | 0.124926163 | 0 | 0 | 0.05601656 | 0.01757914 | 0 | 0 | 0 | 0 |
| ENSMUSG00000009596 | Taf7l | 2338 | 0 | 0 | 0 | 0 | 0 | 0 | 0 | 0 | 0 | 0 | 0 | 0 | 0 | 0 | 0.070490258 | 0 | 0 | 0 |
| ENSMUSG00000009614 | Sardh | 5435 | 1.274764249 | 2.13913525 | 1.682170605 | 1.492155638 | 1.354955717 | 1.841702334 | 0.679004408 | 1.370288587 | 1.606539345 | 1.414087329 | 0.94055576 | 1.72530475 | 0.571832712 | 0.98271758 | 0.758078305 | 0.796106465 | 0.90262484 | 0.941278183 |
| ENSMUSG00000009621 | Vav2 | 6883 | 10.02882549 | 11.64475134 | 10.75982493 | 11.78245807 | 12.09067209 | 12.310791 | 9.898337594 | 9.410767758 | 6.701859313 | 10.66593709 | 9.294009793 | 8.741723706 | 6.708510035 | 7.321202211 | 7.811714432 | 7.243116495 | 8.157493094 | 7.224790422 |
| ENSMUSG00000009628 | Tex15 | 5814 | 0.116973309 | 0.853736023 | 0.38272831 | 0.705279324 | 1.076221203 | 0.698886163 | 0.504538372 | 1.497741271 | 1.459308952 | 0.778546184 | 1.536621472 | 0.345607818 | 0.212125547 | 0.734925359 | 0.517322597 | 0.381984044 | 1.357966488 | 0.766380744 |
| ENSMUSG00000009630 | Ppp2cb | 1819 | 120.4819613 | 118.3257886 | 126.7179066 | 126.4888806 | 108.8061958 | 133.7842081 | 97.72057984 | 104.8978793 | 108.0721494 | 111.9019164 | 97.88744929 | 103.9294388 | 112.8477778 | 106.8545936 | 108.0663129 | 115.1665273 | 105.6870287 | 112.4980083 |
| ENSMUSG00000009633 | G0s2 | 840 | 0 | 0 | 0 | 0 | 0 | 0 | 0.450596942 | 0.090934291 | 0 | 0 | 0 | 0 | 0 | 0 | 0 | 0 | 0 | 0 |
| ENSMUSG00000009640 | Dmap1 | 2736 | 17.77263208 | 16.83908359 | 15.64713975 | 14.92057592 | 15.05881822 | 15.50334062 | 14.92355333 | 16.1508076 | 14.93312267 | 15.6824342 | 17.09491387 | 16.91198256 | 15.56046952 | 18.84244773 | 18.53768834 | 16.31829248 | 17.04791788 | 14.1744957 |
| ENSMUSG00000009646 | Pla2g12b | 1178 | 0 | 0 | 0 | 0 | 0 | 0 | 0 | 0 | 0 | 0 | 0 | 0 | 0 | 0 | 0 | 0 | 0 | 0 |
| ENSMUSG00000009647 | Ccdc109a | 7183 | 2.71020056 | 2.671338865 | 2.78132647 | 2.448359058 | 2.961758355 | 3.925310512 | 2.601769202 | 3.264671457 | 3.033226624 | 3.203333313 | 3.012955059 | 3.092667926 | 3.70178246 | 3.659662183 | 3.303925406 | 3.273065981 | 3.174738502 | 3.714244294 |

| ENSMUSG00000009654 | Oit3 | 2779 | 0.275312403 | 0 | 0 | 0 | 0.103919231 | 0 | 0 | 0.082459307 | 0 | 0 | 0 | 0 | 0 | 0.050137554 | 0 | 0 | 0 | 0 |
| --- | --- | --- | --- | --- | --- | --- | --- | --- | --- | --- | --- | --- | --- | --- | --- | --- | --- | --- | --- | --- |
| ENSMUSG00000009670 | Tex11 | 3593 | 0 | 0 | 0.134632703 | 0 | 0 | 0.041374395 | 0 | 0 | 0.011462988 | 0 | 0 | 0 | 0 | 0 | 0 | 0.021314004 | 0 | 0 |
| ENSMUSG00000009681 | Bcr | 7332 | 7.061007147 | 7.583090624 | 6.643773195 | 7.885565366 | 6.682801186 | 6.454290857 | 6.201239014 | 5.761156173 | 8.055300546 | 6.623734909 | 5.929516123 | 6.630584031 | 7.952854094 | 5.504625197 | 6.52976104 | 7.562029394 | 6.664761123 | 5.544425787 |
| ENSMUSG00000009687 | Fxyd5 | 2277 | 0.485346762 | 0.131671519 | 0.488621519 | 0.100046211 | 0.253659677 | 0.544057242 | 1.724616755 | 1.039933662 | 0.850138885 | 0.144951657 | 1.091039895 | 0.343179304 | 1.494906584 | 0.734293872 | 0.705691998 | 1.008975162 | 0.353470459 | 0.048317178 |
| ENSMUSG00000009731 | Kcnd1 | 6030 | 0.324251757 | 0.359094216 | 0.344952207 | 0.075557287 | 0.343229306 | 0.304054916 | 0.721851818 | 1.121070518 | 0.853783494 | 0.500438642 | 0.205994846 | 1.008939838 | 1.570767515 | 2.010266469 | 1.366552434 | 0.228600641 | 0.184322158 | 0.748050871 |
| ENSMUSG00000009733 | Tfcp2 | 3332 | 11.92747286 | 10.98765629 | 13.44354415 | 14.15236521 | 14.06977601 | 13.97946265 | 13.39011291 | 11.01527572 | 11.52035795 | 10.117873 | 12.02976896 | 13.26710827 | 11.3114 | 11.36012342 | 13.18153019 | 10.29663392 | 11.68652991 | 9.856073443 |
| ENSMUSG00000009734 | Pou6f2 | 7404 | 0 | 0 | 0 | 0 | 0 | 0 | 0 | 0.015475041 | 0 | 0 | 0 | 0 | 0.066628738 | 0 | 0 | 0 | 0 | 0 |
| ENSMUSG00000009739 | Pou6f1 | 10168 | 2.257355926 | 1.657781715 | 2.055209 | 1.653424996 | 1.685185368 | 2.007840896 | 1.563440216 | 1.821726511 | 1.936187503 | 1.534901925 | 1.442457932 | 1.899313459 | 1.974635162 | 1.379436899 | 1.580311447 | 1.325559973 | 1.270255906 | 2.14236883 |
| ENSMUSG00000009741 | Ubp1 | 3451 | 24.41183972 | 28.18709102 | 24.57223956 | 24.76746426 | 25.38397597 | 25.01328945 | 27.6801793 | 26.29531965 | 29.00122669 | 21.39611744 | 25.72178292 | 27.75418053 | 22.27155234 | 24.00934567 | 22.08718586 | 26.28526483 | 28.06448921 | 29.64848986 |
| ENSMUSG00000009772 | Nuak2 | 4218 | 0.846475767 | 0.971428637 | 1.009215427 | 0.864126019 | 0.661842478 | 0.892842046 | 0.235553878 | 0.470840428 | 0.732334918 | 0.514208713 | 1.076012578 | 0.172025513 | 0.128651278 | 0.407404275 | 0.146520469 | 0.163402308 | 0.318022852 | 0.221705741 |
| ENSMUSG00000009828 | Ick | 11726 | 5.013189361 | 4.849489206 | 4.319212543 | 4.930663932 | 4.970813539 | 4.863994231 | 2.150576315 | 3.227756335 | 2.620257612 | 3.248999508 | 3.059781154 | 2.651315373 | 2.128769413 | 3.085446365 | 3.369630914 | 2.612355954 | 2.467708866 | 2.603620521 |
| ENSMUSG00000009863 | Sdhb | 2306 | 147.8096299 | 132.8181914 | 141.6803226 | 127.1797235 | 140.3465112 | 140.8793241 | 162.1064511 | 146.6581627 | 145.4566281 | 159.874826 | 166.7565658 | 148.3980171 | 149.7499657 | 137.2376459 | 133.3779769 | 137.5373379 | 144.4133538 | 139.5742773 |
| ENSMUSG00000009876 | Cox4i2 | 783 | 0.597135295 | 0.170180813 | 0.185339197 | 0.29093898 | 0.553240502 | 0.316428695 | 1.389772178 | 0.438993131 | 1.420224681 | 1.204360236 | 0.366091636 | 0.427706227 | 1.827108047 | 2.49125373 | 2.315285389 | 0.342317052 | 0.734220247 | 0.70254287 |
| ENSMUSG00000009894 | Snap47 | 2731 | 65.29081415 | 55.24500107 | 61.39240432 | 61.19293688 | 61.89649063 | 64.5582666 | 61.96904157 | 51.40800851 | 43.25262805 | 55.76593611 | 51.22119313 | 59.80103578 | 53.52269165 | 45.8488684 | 53.31611077 | 58.46643459 | 57.20175254 | 62.09925889 |
| ENSMUSG00000009900 | Wnt3a | 2757 | 0 | 0 | 0.193002841 | 0 | 0.069832316 | 0 | 0 | 0.013852884 | 0 | 0 | 0 | 0 | 0 | 0.336917573 | 0 | 0 | 0 | 0 |
| ENSMUSG00000009905 | Kdsr | 5572 | 16.30941607 | 14.94058196 | 15.6962209 | 13.56528581 | 14.74536858 | 16.73694268 | 14.25663818 | 13.70868715 | 16.03997473 | 12.65926993 | 12.18381479 | 14.39467106 | 13.94432335 | 15.79532405 | 12.86624323 | 16.13538158 | 12.24350475 | 14.63092178 |
| ENSMUSG00000009907 | Vps4b | 9167 | 16.54859531 | 18.10823082 | 17.13416091 | 17.20156806 | 18.56074508 | 19.1735078 | 15.23584904 | 16.56101228 | 16.97871093 | 16.21753762 | 19.3872418 | 17.06679418 | 16.74712846 | 16.84085813 | 17.847865 | 19.43978257 | 22.36362628 | 20.89469942 |
| ENSMUSG00000009927 | Rps25 | 678 | 97.17260006 | 74.92944496 | 88.3280687 | 81.57980505 | 86.254111 | 81.34541663 | 102.0223254 | 88.77761975 | 95.55514645 | 87.62520074 | 83.57094507 | 89.32152494 | 126.6767964 | 136.592198 | 111.2685086 | 114.5894431 | 92.14139226 | 114.5617385 |
| ENSMUSG00000009941 | Nxf2 | 2805 | 0.106073523 | 0.059381273 | 0.155209188 | 0.146185169 | 0.308867959 | 0.459312326 | 0 | 0.394859062 | 0 | 0.201714239 | 0.187352778 | 0 | 0.334155589 | 0.016557607 | 0.014688612 | 0.245715128 | 0 | 0.902110128 |
| ENSMUSG00000009995 | Taz | 3381 | 85.36236179 | 83.7699576 | 85.91631168 | 88.14397841 | 80.77507687 | 77.37026932 | 77.62301982 | 86.07693177 | 87.90354858 | 86.38019993 | 97.24556766 | 83.17045493 | 98.31365235 | 80.29153824 | 95.22299844 | 87.72524251 | 94.2570715 | 93.61798287 |
| ENSMUSG00000010021 | Kif19a | 4211 | 0 | 0.055376457 | 0.218261001 | 0.205571086 | 0.205740828 | 0.164744305 | 0.033706492 | 0.009069675 | 0.20539464 | 0.123167356 | 0.068071658 | 0.053018915 | 0 | 0.04411692 | 0 | 0.081836967 | 0.100116188 | 0 |
| ENSMUSG00000010025 | Aldh3a2 | 5606 | 171.6205243 | 185.1521866 | 173.9238098 | 171.2237035 | 187.9257127 | 178.8706951 | 166.6915308 | 165.9046349 | 170.4251884 | 163.3957746 | 171.9165604 | 157.8390473 | 154.7716306 | 142.944396 | 168.1723157 | 150.2527259 | 180.2621246 | 155.5582178 |
| ENSMUSG00000010044 | Zmynd10 | 1743 | 0 | 0.114674335 | 0 | 0 | 0 | 0 | 0.841476217 | 1.073681995 | 1.086964845 | 0.162308789 | 0.191867303 | 0.672477862 | 1.047206506 | 1.092488573 | 0.732787282 | 0.483300839 | 0.087954783 | 0.504960245 |
| ENSMUSG00000010045 | Tmem115 | 2175 | 35.72389047 | 33.00656876 | 31.67076194 | 34.77302694 | 31.95517139 | 32.14662394 | 32.06385689 | 31.44946791 | 38.25138475 | 30.26316402 | 36.37486494 | 33.95132028 | 32.366274 | 37.0698555 | 34.17361233 | 33.25561259 | 30.13239894 | 28.6047355 |
| ENSMUSG00000010047 | Hyal2 | 1889 | 35.37222695 | 33.96539665 | 34.7756772 | 29.8353689 | 29.53144496 | 40.58130167 | 26.02322573 | 27.21385582 | 28.71500331 | 29.27886444 | 27.99728906 | 28.95677572 | 23.89555548 | 26.92232699 | 26.56616997 | 30.99329736 | 22.80505967 | 22.62682685 |
| ENSMUSG00000010048 | Ifrd2 | 1968 | 8.617665262 | 7.600350358 | 6.538292213 | 5.834036165 | 5.698545841 | 4.93512997 | 5.866002875 | 7.219295577 | 8.036393319 | 7.115731132 | 5.097937643 | 6.721703269 | 8.522790586 | 5.097521787 | 7.41125548 | 6.323398072 | 6.56298885 | 8.050113179 |
| ENSMUSG00000010051 | Hyal1 | 3939 | 6.452931014 | 7.679133778 | 6.828048458 | 8.362689783 | 8.125851546 | 8.277658973 | 4.48023083 | 6.24420085 | 4.673869649 | 4.919761117 | 5.445787346 | 4.931162879 | 6.136745231 | 7.593295831 | 6.359620711 | 7.85448357 | 5.915813282 | 6.368152492 |
| ENSMUSG00000010054 | Tusc2 | 1658 | 39.09553284 | 33.59418524 | 33.0853941 | 35.06386766 | 38.69713767 | 37.08983255 | 33.58686556 | 32.45663148 | 27.92137739 | 38.73296613 | 38.84236672 | 39.25275186 | 43.61917414 | 43.08263354 | 33.12525573 | 38.66011869 | 39.08912428 | 39.64769755 |
| ENSMUSG00000010057 | Nprl2 | 1445 | 28.88587049 | 21.64761777 | 23.46702061 | 24.78268571 | 20.78499679 | 24.48488012 | 22.00285138 | 21.22387483 | 24.59789834 | 23.75481797 | 24.82975351 | 19.97003291 | 22.94190201 | 24.84517616 | 25.06309178 | 24.11380812 | 19.09683998 | 23.3360432 |
| ENSMUSG00000010064 | Slc38a3 | 4246 | 0.090095757 | 0.156914245 | 0.205069135 | 0.150224828 | 0.13602993 | 0.385124872 | 0.289714944 | 0.557684632 | 0.291002231 | 0.444189385 | 0.46132202 | 0.486382364 | 0.569303803 | 0.010938315 | 0.378441046 | 0.261523225 | 0.487428175 | 0.012955513 |
| ENSMUSG00000010066 | Cacna2d2 | 6399 | 0.026569887 | 0.08329525 | 0.007559545 | 0.056960205 | 0.052652518 | 0 | 0.044362568 | 0.071621945 | 0 | 0.007368449 | 0.029864015 | 0.052335361 | 0.20815155 | 0.043548136 | 0.019316247 | 0.029919212 | 0.023957679 | 0 |
| ENSMUSG00000010067 | Rassf1 | 5579 | 4.784594111 | 4.639562432 | 3.81508395 | 3.617770683 | 4.632880984 | 4.059078553 | 2.561109345 | 5.216456468 | 3.255646792 | 2.814336653 | 3.151314702 | 3.271504152 | 3.660766043 | 5.053156667 | 3.190369626 | 5.209279623 | 4.08749716 | 4.496173627 |
| ENSMUSG00000010080 | Epn3 | 5052 | 3.171902487 | 5.156508963 | 4.002402155 | 4.139453553 | 3.629901607 | 4.050926161 | 2.650334152 | 2.865186166 | 1.728333599 | 3.369240672 | 3.404391341 | 1.922392179 | 2.714622526 | 1.902994079 | 3.73521627 | 3.16814606 | 2.814540014 | 3.266574033 |
| ENSMUSG00000010086 | Rnf112 | 4258 | 0 | 0 | 0 | 0 | 0 | 0 | 0 | 0 | 0 | 0 | 0 | 0 | 0 | 0 | 0 | 0 | 0 | 0 |
| ENSMUSG00000010095 | Slc3a2 | 2948 | 97.35242488 | 104.1988459 | 91.15161503 | 102.8059928 | 98.89249038 | 107.7620675 | 66.00985361 | 73.50871481 | 76.70080446 | 80.32253452 | 79.50608762 | 85.90083486 | 73.06077029 | 85.08972727 | 81.32695174 | 92.83004447 | 92.33153345 | 104.7934679 |
| ENSMUSG00000010097 | Nxf1 | 2262 | 54.26832377 | 58.36383722 | 58.68124107 | 60.78834297 | 58.83499953 | 54.83222464 | 53.83856568 | 68.38162234 | 68.40748882 | 62.72169147 | 61.60899818 | 60.33125333 | 54.26074721 | 64.22506868 | 65.55455334 | 65.49352787 | 59.09908205 | 57.56257985 |
| ENSMUSG00000010110 | Stx5a | 4420 | 39.649059 | 33.66712635 | 32.73421477 | 35.55204568 | 33.14786779 | 31.52540426 | 32.69070621 | 36.35190881 | 33.99285283 | 31.03198999 | 33.92886757 | 32.98427843 | 31.94297449 | 34.80154241 | 37.06275706 | 33.56925423 | 37.6672927 | 36.30352134 |
| ENSMUSG00000010122 | Slc47a1 | 4491 | 0.132503332 | 0 | 0 | 0.010144966 | 0.064304507 | 0 | 0 | 0 | 0 | 0 | 0 | 0.012428337 | 0 | 0.268881379 | 0 | 0 | 0 | 0 |
| ENSMUSG00000010136 | 1700027A23R | 1079 | 0 | 0.987963499 | 0.224159084 | 0.464477746 | 0.0446079 | 0 | 0.087697273 | 0 | 0 | 0 | 0.39849363 | 0 | 0 | 0 | 0 | 0.141948498 | 0.071040402 | 0 |
| ENSMUSG00000010142 | Tnfrsf13b | 2885 | 0.132598469 | 0.242485539 | 0.368879608 | 0.157923897 | 0.050050527 | 0.515279725 | 0.360789926 | 0.238288819 | 0.599595723 | 0.359554756 | 0.480233551 | 0.193468501 | 0.23939232 | 0.048295411 | 1.042535036 | 0.756521533 | 0.531387132 | 0.648287566 |
| ENSMUSG00000010154 | Spire2 | 2315 | 25.64999175 | 29.44197908 | 22.42107908 | 29.52119503 | 23.53578298 | 27.2486522 | 19.00682134 | 17.85061745 | 19.28560824 | 17.82154011 | 23.0310383 | 18.58914724 | 13.8086749 | 13.34139011 | 14.54067869 | 14.38999069 | 14.48618133 | 19.41358108 |

| ENSMUSG00000010175 | Prox1 | 9269 | 0.298072763 | 0.097038315 | 0.005218851 | 0.004915422 | 0 | 0.13899785 | 0.056148394 | 0.123613343 | 0 | 0 | 0.01546282 | 0 | 0 | 0.075160353 | 0.040005826 | 0 | 0.037214011 | 0.136499024 |
| --- | --- | --- | --- | --- | --- | --- | --- | --- | --- | --- | --- | --- | --- | --- | --- | --- | --- | --- | --- | --- |
| ENSMUSG00000010205 | Raver1 | 3560 | 11.68892341 | 14.75686353 | 17.05302826 | 15.35765539 | 13.2092386 | 16.61965278 | 10.55232221 | 14.70836621 | 13.33934064 | 13.37702536 | 16.19785811 | 12.52716697 | 12.59626764 | 9.784568972 | 12.01326264 | 10.57294019 | 11.01342745 | 10.73913741 |
| ENSMUSG00000010277 | 2610507B11R | 8888 | 15.43251609 | 16.62646251 | 16.63248298 | 16.29084146 | 15.17390303 | 18.18643305 | 15.82589947 | 18.58055221 | 15.88516832 | 17.00778068 | 15.22799925 | 15.78764354 | 15.25248747 | 16.17286796 | 17.11477772 | 13.23455737 | 13.79884677 | 14.20408415 |
| ENSMUSG00000010290 | AI597479 | 3454 | 9.697185498 | 10.62849144 | 10.04163905 | 12.16192324 | 12.33258612 | 10.17165261 | 12.45142593 | 9.266135847 | 11.36385336 | 10.70241788 | 10.40149646 | 10.60077437 | 5.355955118 | 10.55547441 | 7.944480667 | 4.977557245 | 9.864527489 | 7.501241826 |
| ENSMUSG00000010307 | Tmem86a | 1788 | 5.990662393 | 9.092109832 | 7.656436852 | 9.963293252 | 7.187485246 | 6.873086089 | 6.615307456 | 8.501440804 | 4.630027779 | 8.069415658 | 9.138163199 | 9.084092736 | 5.104253186 | 5.065210873 | 8.065181522 | 7.966502211 | 4.930116492 | 6.522332564 |
| ENSMUSG00000010311 | Optc | 2971 | 0.11445352 | 0.269104497 | 0.472175153 | 0.1380173 | 0.113404061 | 0.050036419 | 0.748467152 | 0.269956395 | 0.346571152 | 0.507850052 | 0 | 0.394523364 | 0.763806191 | 0.56276915 | 0.748867054 | 0.463972355 | 0.103201069 | 0.555460519 |
| ENSMUSG00000010342 | Tex14 | 4982 | 0.051190497 | 0 | 0 | 0 | 0 | 0.149195304 | 0.028490172 | 0 | 0.082670646 | 0 | 0.04794757 | 0.02240693 | 0 | 0.037289512 | 0 | 0.076857903 | 0.03846477 | 0.022083142 |
| ENSMUSG00000010358 | Ifi35 | 1901 | 31.28076868 | 24.11285768 | 28.60170864 | 27.34621335 | 27.54736084 | 27.8652668 | 22.4741447 | 20.99477144 | 24.6339129 | 19.61925633 | 21.96492042 | 24.57533382 | 26.7549746 | 20.00931487 | 22.4105254 | 21.53217218 | 17.92323932 | 17.27534809 |
| ENSMUSG00000010362 | Rdm1 | 1567 | 16.22086488 | 15.05139062 | 15.18811547 | 14.7121177 | 16.61733931 | 15.74809274 | 12.22822908 | 16.2080074 | 16.76898344 | 19.64863384 | 15.48798909 | 19.30573652 | 15.67791628 | 12.56687495 | 11.59533258 | 16.10306974 | 15.72674468 | 15.69181283 |
| ENSMUSG00000010376 | Nedd8 | 733 | 350.7111932 | 286.9090057 | 289.9112126 | 282.7519655 | 332.1299726 | 287.1084031 | 378.5659781 | 321.6915313 | 314.9391826 | 310.6285758 | 313.3072652 | 300.0187048 | 434.2285543 | 417.2998084 | 361.7642752 | 371.6226208 | 331.0806439 | 356.5460655 |
| ENSMUSG00000010392 | Gosr1 | 4280 | 18.64070453 | 20.68824132 | 17.98184271 | 19.5337655 | 20.65849154 | 18.45491994 | 15.00077227 | 17.13304033 | 16.18591577 | 17.26288598 | 17.41330262 | 13.75830001 | 14.98399357 | 14.97496276 | 21.32276783 | 19.395803 | 19.05569154 | 19.75443856 |
| ENSMUSG00000010406 | Mrpl52 | 407 | 130.3352818 | 103.7856262 | 120.5178373 | 118.8841009 | 123.9367469 | 125.7689774 | 127.0583736 | 99.65683379 | 112.2256658 | 109.0142795 | 113.0400123 | 119.7225392 | 149.0866296 | 137.7346769 | 108.6222837 | 119.0113473 | 96.80450422 | 134.8871954 |
| ENSMUSG00000010435 | Spaca7 | 754 | 0 | 0 | 0 | 0 | 0 | 0 | 0 | 0 | 0 | 0 | 0 | 0 | 0 | 0.369581597 | 0 | 0 | 0 | 0 |
| ENSMUSG00000010453 | 4632411B12R | 4797 | 16.66713281 | 19.78495635 | 17.02199692 | 20.61976803 | 18.3417045 | 20.21569723 | 16.67828644 | 18.11292797 | 17.42935731 | 19.16695254 | 19.70953572 | 17.41839627 | 20.25930311 | 21.53255167 | 20.67378466 | 18.67834507 | 18.54395145 | 17.95794478 |
| ENSMUSG00000010461 | Eya4 | 4172 | 0 | 0 | 0 | 0 | 0 | 0 | 0 | 0 | 0 | 0 | 0 | 0 | 0 | 0 | 0 | 0 | 0 | 0 |
| ENSMUSG00000010476 | Ebf3 | 6951 | 0 | 0 | 0 | 0 | 0 | 0 | 0 | 0 | 0 | 0 | 0 | 0 | 0 | 0 | 0 | 0 | 0 | 0 |
| ENSMUSG00000010492 | Gm16119 | 1673 | 2.159557658 | 1.413757017 | 1.358969472 | 0.844227361 | 1.438491443 | 2.014097958 | 5.062145564 | 3.835220325 | 3.889700831 | 4.142948822 | 6.168195476 | 5.237931277 | 2.005122758 | 2.415203588 | 2.881399904 | 3.02113579 | 2.359598674 | 2.466038855 |
| ENSMUSG00000010505 | Myt1 | 6879 | 0 | 0 | 0 | 0 | 0 | 0 | 0 | 0 | 0 | 0.006854296 | 0 | 0 | 0.007171379 | 0 | 0.005989469 | 0 | 0 | 0 |
| ENSMUSG00000010517 | Faf1 | 5148 | 18.95724246 | 22.57097417 | 20.75701798 | 21.5149376 | 22.91595666 | 22.36995976 | 19.7779497 | 20.77286844 | 20.06522563 | 19.57285409 | 22.34695028 | 18.97385577 | 18.56175675 | 21.39062378 | 22.25748386 | 19.98579295 | 22.7739204 | 24.65151694 |
| ENSMUSG00000010529 | Gm266 | 1212 | 0.631264991 | 0.879548362 | 0.758330921 | 1.165340243 | 0.71483055 | 1.063012436 | 0.858811004 | 1.102915911 | 0.407787285 | 1.283806277 | 0 | 0.782892957 | 0.284920314 | 0.689763674 | 0.305952148 | 0.347522014 | 0.505957715 | 0.136161155 |
| ENSMUSG00000010538 | 1700021C14R | 1139 | 2.873484244 | 2.398294404 | 2.335859675 | 2.400055013 | 2.070644653 | 2.479811953 | 3.073870273 | 2.514864074 | 3.109780821 | 3.10474341 | 3.397516801 | 2.156180117 | 3.248370321 | 2.813557533 | 2.098059914 | 2.823890274 | 2.79287326 | 2.028430625 |
| ENSMUSG00000010554 | Mettl16 | 13643 | 2.872518676 | 2.055959608 | 2.368505334 | 2.120593942 | 2.085022861 | 2.567894352 | 2.465683114 | 2.331911691 | 3.716235498 | 2.823581658 | 2.339197557 | 1.824656272 | 2.29610551 | 3.148924785 | 1.821046559 | 2.09934577 | 2.53392361 | 2.149076734 |
| ENSMUSG00000010592 | Dazl | 4918 | 0 | 0 | 0 | 0.009264141 | 0 | 0 | 0 | 0 | 0 | 0 | 0 | 0 | 0.02006178 | 0.132211717 | 0.016755411 | 0 | 0.05455146 | 0 |
| ENSMUSG00000010601 | Apol7a | 4815 | 0 | 0 | 0.020092847 | 0 | 0 | 0.020582652 | 0.009826102 | 0 | 0 | 0 | 0 | 0 | 0 | 0 | 0.008556917 | 0 | 0 | 0 |
| ENSMUSG00000010607 | Pigyl | 821 | 72.32610342 | 62.48703666 | 58.21321314 | 62.70886736 | 60.97101171 | 63.37438521 | 62.35361583 | 59.77738988 | 58.74471371 | 62.65702467 | 68.89835669 | 56.49551231 | 72.58581738 | 62.62314828 | 54.80158226 | 68.41939228 | 50.23032335 | 59.09626325 |
| ENSMUSG00000010608 | Rbm25 | 4225 | 38.69228568 | 39.47873642 | 32.89400064 | 32.04909438 | 38.50553893 | 36.28784797 | 36.73031642 | 32.68727269 | 37.58939758 | 32.99991232 | 38.57050494 | 35.27285659 | 38.7999908 | 42.75054576 | 37.37883165 | 41.06384429 | 37.93623045 | 40.73928874 |
| ENSMUSG00000010609 | Psen2 | 4534 | 10.03099655 | 9.228274182 | 8.044473288 | 8.571585977 | 7.664589521 | 11.75975768 | 7.304560046 | 7.294799401 | 8.475301986 | 8.974648635 | 8.766820788 | 10.02072107 | 10.83691875 | 8.389437047 | 9.559778738 | 10.50584817 | 10.65088974 | 10.36122125 |
| ENSMUSG00000010651 | Acaa1b | 1950 | 2.070764986 | 1.998773653 | 1.83571346 | 1.705618583 | 1.382250116 | 1.372229545 | 1.60135221 | 1.899827197 | 1.351762569 | 2.055287065 | 1.567998637 | 1.202183502 | 1.897381434 | 1.33377892 | 0.993063142 | 1.217444953 | 1.533051876 | 0.818084151 |
| ENSMUSG00000010660 | Plcd1 | 2636 | 4.579465096 | 5.598487154 | 3.3032001 | 5.219816157 | 5.03960961 | 5.225971144 | 8.615358834 | 4.868227317 | 7.499820776 | 6.063766466 | 7.811459448 | 6.5005343 | 7.785310162 | 5.215269297 | 8.409118767 | 8.018366952 | 8.345711086 | 5.613600041 |
| ENSMUSG00000010663 | Fads1 | 3444 | 24.21462119 | 29.58889182 | 28.24598419 | 29.61997046 | 29.41861188 | 31.84094059 | 25.89558646 | 20.59329015 | 22.18379406 | 23.46582619 | 18.11675255 | 26.12510107 | 19.73849652 | 26.53947851 | 32.70762306 | 27.26148936 | 30.53639916 | 30.98654676 |
| ENSMUSG00000010721 | Lmbr1 | 4935 | 12.87644137 | 13.16990002 | 12.80158676 | 13.74678724 | 13.54716149 | 14.58969035 | 14.25612028 | 13.18740703 | 11.23344483 | 13.4716295 | 13.59190308 | 11.6155391 | 15.05448175 | 14.04145459 | 15.42866774 | 14.03600989 | 18.70105835 | 14.83629606 |
| ENSMUSG00000010751 | Tnfrsf22 | 5991 | 0.014189677 | 0.00556049 | 0 | 0 | 0.008034038 | 0 | 0 | 0.006374963 | 0 | 0 | 0 | 0 | 0 | 0 | 0 | 0 | 0 | 0 |
| ENSMUSG00000010755 | Cars | 4925 | 12.61778017 | 14.86059464 | 14.02586829 | 13.80245242 | 12.65600836 | 14.08605625 | 9.356862797 | 9.569426308 | 8.872871721 | 8.472765966 | 7.789500844 | 7.026540265 | 8.824653629 | 8.496674668 | 9.444986112 | 8.295650375 | 9.618538675 | 7.796214516 |
| ENSMUSG00000010760 | Phlda2 | 755 | 0.168894739 | 0.088246077 | 0.320354506 | 0.422420279 | 0.510007138 | 0.525062078 | 0 | 0 | 0.054551677 | 0.062451263 | 0 | 0 | 0.261361151 | 0 | 0.327429583 | 0 | 0.101526614 | 1.384335136 |
| ENSMUSG00000010796 | Asz1 | 3237 | 0 | 0.010291286 | 0 | 0 | 0 | 0 | 0 | 0 | 0 | 0 | 0 | 0 | 0 | 0 | 0 | 0 | 0 | 0 |
| ENSMUSG00000010797 | Wnt2 | 3078 | 0 | 0 | 0 | 0 | 0 | 0 | 0 | 0 | 0 | 0.030637234 | 0 | 0 | 0 | 0.331959039 | 0.120472386 | 0 | 0.012451688 | 0 |
| ENSMUSG00000010803 | Gabra1 | 5148 | 0 | 0 | 0 | 0 | 0 | 0 | 0 | 0 | 0 | 0 | 0 | 0 | 0 | 0 | 0 | 0 | 0 | 0 |
| ENSMUSG00000010825 | Grid2ip | 4762 | 0 | 0 | 0 | 0 | 0 | 0 | 0 | 0 | 0.008648995 | 0 | 0 | 0 | 0 | 0 | 0 | 0 | 0.024145084 | 0 |
| ENSMUSG00000010830 | Kdelr3 | 1409 | 4.675871032 | 5.083230843 | 3.536177166 | 3.362915977 | 4.850768744 | 4.60710298 | 2.014734376 | 4.174329291 | 5.90466727 | 3.279466941 | 2.882094727 | 3.089866346 | 6.407197205 | 6.691376678 | 5.351231177 | 2.445815945 | 2.176084991 | 5.348649838 |
| ENSMUSG00000010841 | 1700006E09R | 885 | 0.288170685 | 0 | 0.218637425 | 0.154444218 | 0.326318127 | 0.335950736 | 0.588067196 | 0.345242056 | 0.093076872 | 0.213110523 | 0.26991502 | 0.315342726 | 0.167226838 | 0.367354364 | 0.512109734 | 0.5624609 | 0.173226201 | 0.621571827 |
| ENSMUSG00000010911 | Apip | 1139 | 71.6878342 | 61.01026983 | 68.80168497 | 60.96139734 | 64.65482284 | 60.21157443 | 59.48354366 | 57.07064872 | 67.25805032 | 64.49586978 | 57.50611771 | 60.03001463 | 50.28477257 | 50.31782602 | 49.34058142 | 45.6865105 | 50.33901683 | 55.15399462 |

| ENSMUSG00000010914 | Pdhx | 3919 | 23.6658061 | 23.94550217 | 24.85947694 | 21.91440892 | 22.31582172 | 24.3022082 | 25.44912661 | 22.09292581 | 22.00677826 | 24.14683885 | 27.45322952 | 23.84164817 | 19.57415957 | 15.4181571 | 19.76497195 | 16.73687432 | 21.54448382 | 21.0547742 |
| --- | --- | --- | --- | --- | --- | --- | --- | --- | --- | --- | --- | --- | --- | --- | --- | --- | --- | --- | --- | --- |
| ENSMUSG00000010936 | Vac14 | 3681 | 23.33685432 | 27.01412367 | 26.72962802 | 25.99244583 | 27.47220093 | 28.01391974 | 19.25411384 | 24.79756635 | 25.49961136 | 26.42540093 | 24.67269657 | 20.24284421 | 22.39435854 | 21.20959275 | 24.80376198 | 25.61028944 | 24.53049593 | 26.91426276 |
| ENSMUSG00000011008 | Mcoln2 | 3300 | 0 | 0 | 0 | 0 | 0 | 0 | 0 | 0 | 0 | 0.042864276 | 0.332976983 | 0.033827674 | 0.224235988 | 0.126665693 | 0.17479448 | 0 | 0 | 0 |
| ENSMUSG00000011034 | Slc5a1 | 4102 | 0 | 0.016242269 | 0.070756017 | 0 | 0.058668849 | 0 | 0 | 0 | 0 | 0 | 0 | 0 | 0 | 0 | 0 | 0 | 0 | 0 |
| ENSMUSG00000011052 | 1700049J03R | 416 | 0.102175904 | 0.320316291 | 0 | 0.328565224 | 0.11570174 | 0 | 0.113732401 | 0.09180866 | 0 | 0.566715183 | 0 | 0 | 0 | 0 | 0 | 0 | 0 | 0.264466859 |
| ENSMUSG00000011096 | Akt1s1 | 3301 | 27.05343073 | 27.14682988 | 26.55341927 | 25.42364243 | 22.71721814 | 26.60025565 | 30.50026682 | 24.42416282 | 28.09816222 | 27.8104875 | 27.15111242 | 30.09750966 | 25.33099054 | 25.12848836 | 25.71196763 | 24.28977034 | 24.219526 | 23.96337336 |
| ENSMUSG00000011114 | Tbrg1 | 3809 | 44.7258455 | 43.27440289 | 43.61110613 | 40.50136417 | 42.35710374 | 42.03331116 | 45.8097033 | 42.64433905 | 43.11122036 | 42.66959153 | 46.11932852 | 45.44089516 | 43.08933809 | 47.4317406 | 53.39219739 | 49.16759275 | 42.01906429 | 43.81665261 |
| ENSMUSG00000011118 | Panx3 | 2654 | 0 | 0 | 0 | 0 | 0 | 0 | 0 | 0 | 0 | 0 | 0 | 0 | 0 | 0 | 0 | 0 | 0 | 0 |
| ENSMUSG00000011148 | Adssl1 | 1777 | 144.546302 | 139.6629499 | 135.2389976 | 137.1190012 | 150.8964247 | 125.5131423 | 112.9966288 | 91.27919697 | 84.01863795 | 100.1919277 | 106.8954614 | 97.55962183 | 103.0223664 | 81.33595946 | 97.72907039 | 100.6071165 | 95.65398242 | 102.650646 |
| ENSMUSG00000011154 | 1700026D08R | 3636 | 0.035070277 | 0.183239242 | 0.079824308 | 0.050122161 | 0 | 0.013628365 | 0 | 0 | 0 | 0 | 0 | 0 | 0.122108706 | 0 | 0 | 0.052654851 | 0.147571 | 0 |
| ENSMUSG00000011158 | Brf1 | 2737 | 18.16991449 | 20.31392782 | 17.21440211 | 17.31219807 | 20.06522649 | 20.82047629 | 17.77034488 | 15.76814568 | 16.206751 | 16.55528894 | 18.78182511 | 17.3340567 | 14.38321885 | 25.16499145 | 20.5029299 | 16.99783997 | 18.30196202 | 21.56549927 |
| ENSMUSG00000011171 | Vipr2 | 4611 | 0 | 0.057797257 | 0.073436286 | 0.039523786 | 0.156577501 | 0.032239905 | 0.071825798 | 0.265052457 | 0 | 0.398802305 | 0.279749269 | 0.108944039 | 0.171179934 | 0.423043086 | 0 | 0.282342366 | 0.033247709 | 0.155089652 |
| ENSMUSG00000011179 | Odc1 | 2288 | 24.42932975 | 26.10577768 | 25.24387904 | 28.95356576 | 26.63243678 | 25.10123174 | 36.12554637 | 30.9812495 | 26.06559214 | 24.95607589 | 24.05452455 | 27.1027977 | 29.236923 | 42.87146528 | 34.89887033 | 23.5467153 | 31.10661423 | 30.41368881 |
| ENSMUSG00000011254 | Thg1l | 3458 | 4.670898465 | 1.676241641 | 2.336142154 | 2.068561007 | 2.129607959 | 2.063503076 | 4.40563407 | 2.065349696 | 2.691773442 | 2.26345192 | 2.583550386 | 1.791653715 | 4.493798132 | 2.538442023 | 1.227229687 | 2.746116436 | 2.648925668 | 2.05210375 |
| ENSMUSG00000011256 | Adam19 | 5766 | 0 | 0 | 0 | 0 | 0 | 0 | 0.065643675 | 0.072860983 | 0.078572958 | 0.008177368 | 0.099427593 | 0.1355219 | 0.017111314 | 0.072493373 | 0.1572033 | 0 | 0 | 0 |
| ENSMUSG00000011257 | Pabpc4 | 3647 | 1.85311845 | 1.616776056 | 1.40597593 | 1.486636763 | 1.821279261 | 1.535360267 | 1.725414395 | 1.508008211 | 2.574863064 | 1.874650938 | 1.716073365 | 1.698804098 | 2.272487552 | 2.292277414 | 2.68877716 | 2.141838198 | 1.387187055 | 1.19158745 |
| ENSMUSG00000011263 | Exoc3l2 | 2197 | 0.077387667 | 0.151629013 | 0.022017993 | 0.124427067 | 0 | 0 | 0 | 0 | 0 | 0 | 0 | 0.152432397 | 0.157179527 | 0.232537534 | 0.18753553 | 0.400857519 | 0.087224162 | 0 |
| ENSMUSG00000011267 | Zfp296 | 1832 | 0.580037882 | 0.145471154 | 0.264047655 | 0 | 0.341547493 | 0.162290612 | 0.361559773 | 0.187626431 | 0 | 0 | 0.104312136 | 0.274203583 | 0.080783707 | 0.760547283 | 0.224899323 | 0.250811839 | 0.125522806 | 0.840750539 |
| ENSMUSG00000011305 | Plin5 | 3012 | 7.295875168 | 5.795470309 | 8.174676942 | 7.517874846 | 4.953816845 | 6.959099038 | 7.115751512 | 6.847243459 | 5.510679234 | 6.011245037 | 2.90266182 | 6.20791732 | 6.74792494 | 6.553365584 | 5.49901244 | 4.525715881 | 5.611519566 | 7.871489043 |
| ENSMUSG00000011306 | Sugp1 | 2273 | 24.53444388 | 23.69861415 | 25.68713205 | 23.61236702 | 25.49530845 | 26.90192399 | 21.73094448 | 25.06953999 | 27.4697571 | 23.48200444 | 24.57057917 | 23.54915109 | 26.26116142 | 22.68057061 | 25.97528799 | 24.79708495 | 24.76961291 | 26.37920208 |
| ENSMUSG00000011349 | Dmrtc2 | 1610 | 0 | 0.041382477 | 0 | 0 | 0 | 0 | 0 | 0 | 0 | 0 | 0 | 0 | 0 | 0 | 0 | 0 | 0.190441227 | 0 |
| ENSMUSG00000011350 | Gm5893 | 2077 | 0.020464697 | 0 | 0 | 0 | 0 | 0 | 0 | 0 | 0 | 0 | 0 | 0 | 0 | 0 | 0 | 0 | 0 | 0 |
| ENSMUSG00000011382 | Dhdh | 3670 | 0.613834967 | 0.635395803 | 0.369062357 | 1.191787536 | 0.642633313 | 0.810126435 | 2.114245053 | 1.207171302 | 1.470145386 | 1.516017162 | 1.158575287 | 1.657740387 | 0.846842177 | 1.18957608 | 2.08814425 | 0.855539457 | 1.378493513 | 1.289041738 |
| ENSMUSG00000011427 | Zfp790 | 2103 | 8.812295173 | 9.932089717 | 9.01684446 | 6.889401854 | 8.742936206 | 7.940690069 | 12.80119558 | 10.04298551 | 6.756703727 | 6.008743924 | 8.791682801 | 8.652356635 | 11.16594989 | 9.098889208 | 6.308559675 | 8.776068839 | 10.67960532 | 7.324084585 |
| ENSMUSG00000011463 | Cpb1 | 3484 | 0 | 0 | 0 | 0 | 0 | 0 | 0 | 0 | 0 | 0 | 0 | 0 | 0 | 0 | 0 | 0 | 0 | 0 |
| ENSMUSG00000011486 | Slc25a41 | 1581 | 0.053769989 | 0 | 0.09179038 | 0 | 0 | 0.062685305 | 0 | 0 | 0 | 0 | 0 | 0 | 0 | 0.088129198 | 0.104241761 | 0 | 0 | 0 |
| ENSMUSG00000011589 | Fsd1 | 1735 | 0.097994642 | 0.384010308 | 0.41821496 | 0.210079743 | 0.360642656 | 0.399849147 | 0.109078222 | 0.264154945 | 0.28486351 | 0.054352396 | 0.165215995 | 0.546896982 | 0.540234253 | 0.508609603 | 0.21372565 | 0.198625629 | 0.066270254 | 0.31705537 |
| ENSMUSG00000011632 | 2310033E01R | 750 | 0.340041408 | 0 | 0 | 0 | 0 | 0.264281246 | 0 | 0.050923203 | 0 | 0.188602813 | 0.573299502 | 0.446525301 | 0 | 0 | 0.109870816 | 0 | 0 | 0 |
| ENSMUSG00000011658 | Fuz | 3956 | 9.229511175 | 10.14712779 | 8.314964773 | 9.916091803 | 9.258946894 | 11.49883959 | 8.69472133 | 10.11770418 | 10.35909585 | 10.76266053 | 9.926950422 | 11.10387423 | 6.409657604 | 8.875563717 | 11.4356189 | 7.05608008 | 7.924901635 | 10.28987335 |
| ENSMUSG00000011751 | Spnb4 | 10752 | 0.031625875 | 0.040277867 | 0.040491236 | 0.012712345 | 0.013429666 | 0.018434797 | 0.013201082 | 0.010656362 | 0.003830591 | 0.039467665 | 0.004443356 | 0 | 0.032117134 | 0.008639153 | 0.01149597 | 0.042735053 | 0.014258295 | 0.025580872 |
| ENSMUSG00000011752 | Pgam1 | 1827 | 62.8620611 | 62.34087867 | 59.52035919 | 62.51863062 | 63.62265772 | 58.63875752 | 83.02378141 | 82.25895099 | 76.53433006 | 83.02343316 | 80.46171168 | 78.51464305 | 86.37810803 | 66.80627349 | 72.45790864 | 74.75785255 | 77.15605912 | 71.14752008 |
| ENSMUSG00000011831 | Evi5 | 11690 | 5.406774744 | 5.636689886 | 5.871859673 | 6.298258995 | 5.364918438 | 6.07858169 | 4.638180501 | 5.413585183 | 5.771044724 | 5.614523433 | 5.676596863 | 5.73435507 | 6.629635761 | 6.47992357 | 5.79429922 | 5.686269842 | 5.773533691 | 5.486793707 |
| ENSMUSG00000011832 | Evi5l | 7711 | 6.901372116 | 8.009610411 | 8.048662877 | 8.561529405 | 8.245658302 | 7.77574992 | 6.638868966 | 6.889590422 | 5.891418349 | 7.991955539 | 7.366674313 | 6.550794273 | 5.834614 | 8.064924525 | 6.748484644 | 8.40198518 | 8.265676533 | 6.962636255 |
| ENSMUSG00000011837 | Snapc2 | 1508 | 30.83598314 | 27.54720099 | 24.66793425 | 28.12820443 | 26.55554409 | 30.09967106 | 24.00145847 | 23.85758824 | 21.11218613 | 24.8885675 | 23.95084125 | 26.57536189 | 21.26375745 | 28.55017839 | 22.9504688 | 24.09667535 | 23.83956664 | 21.2667833 |
| ENSMUSG00000011877 | Git1 | 4721 | 14.35146168 | 18.77687175 | 16.15866498 | 19.22423222 | 14.92589202 | 15.82832498 | 14.73197162 | 15.92900664 | 15.06653521 | 15.83009207 | 16.42422318 | 16.37464365 | 15.69509421 | 11.3035917 | 15.15931002 | 16.75670301 | 15.16490629 | 15.71855221 |
| ENSMUSG00000011884 | Gltp | 2258 | 32.58479172 | 35.127547 | 31.0850277 | 36.60218531 | 36.70645374 | 37.10968666 | 40.20949108 | 33.5409805 | 41.55132104 | 37.79573645 | 37.87297417 | 38.14152674 | 38.2770235 | 38.50457555 | 35.78221927 | 36.59483205 | 36.9514386 | 36.37227473 |
| ENSMUSG00000011958 | Bnip2 | 3628 | 22.79908284 | 23.59816377 | 20.57341713 | 21.49958873 | 23.68122485 | 23.62905984 | 15.15361988 | 17.88558205 | 20.04834257 | 16.86924279 | 17.4217944 | 17.26162443 | 16.52102522 | 15.10584981 | 18.45439039 | 17.55162076 | 17.6102362 | 16.89088889 |
| ENSMUSG00000011960 | Ccnt1 | 11225 | 5.903391486 | 5.902837114 | 5.425592402 | 5.930038822 | 5.53141929 | 5.46514781 | 4.990486579 | 6.355760151 | 5.283615385 | 5.653883883 | 6.503352923 | 5.405044564 | 4.684884082 | 5.424338402 | 5.263521706 | 5.041738774 | 6.087820702 | 5.679782155 |
| ENSMUSG00000012017 | Scarf2 | 3291 | 0.232480452 | 0.172081192 | 0 | 0.318415077 | 0.292506373 | 0.346312024 | 0 | 0.162471478 | 0 | 0.085962996 | 0.145168516 | 0.203521103 | 0.164889423 | 0.437486086 | 0.350544991 | 0.197794083 | 0.279498975 | 0.468020355 |
| ENSMUSG00000012042 | 4930579F01R | 1707 | 0 | 0 | 0 | 0 | 0 | 0 | 0 | 0 | 0 | 0 | 0 | 0 | 0 | 0 | 0 | 0 | 0.134714576 | 0 |
| ENSMUSG00000012076 | Brms1l | 2556 | 17.09519599 | 13.71094081 | 17.75210152 | 16.45259934 | 16.1946222 | 13.4156853 | 14.34558927 | 14.67329388 | 10.151606 | 14.83144187 | 11.14001379 | 12.49082903 | 11.48376009 | 12.21065208 | 14.74938327 | 13.24291743 | 12.8504055 | 12.76228493 |

| ENSMUSG00000012114 | Med15 | 4570 | 19.03897053 | 24.80607856 | 19.00010656 | 25.32276862 | 21.54877808 | 24.158313 | 19.19424655 | 18.75355602 | 18.80880928 | 20.34599274 | 20.11357114 | 19.1141164 | 21.67581836 | 19.29919518 | 18.42800443 | 17.25172005 | 21.08365655 | 22.76197392 |
| --- | --- | --- | --- | --- | --- | --- | --- | --- | --- | --- | --- | --- | --- | --- | --- | --- | --- | --- | --- | --- |
| ENSMUSG00000012117 | Dhdds | 5588 | 13.57001325 | 12.21512531 | 11.03726757 | 13.38783729 | 13.48865291 | 13.97550253 | 12.68332016 | 11.96075594 | 14.99902641 | 13.24742379 | 14.08113039 | 12.97504397 | 12.61548135 | 13.84678769 | 13.86902767 | 13.84162973 | 12.88059871 | 15.0320161 |
| ENSMUSG00000012123 | Aim1l | 6922 | 0.749152192 | 1.010648337 | 0.426290863 | 1.02021986 | 0.827463004 | 0.77314291 | 1.168804984 | 0.943499106 | 0.815162187 | 0.933205193 | 1.35967449 | 0.862796287 | 0.94786839 | 1.315088288 | 0.702366887 | 1.322082515 | 1.129523919 | 1.430459291 |
| ENSMUSG00000012126 | Ubxn11 | 3280 | 1.218136142 | 0.893760577 | 0.988117846 | 1.472399604 | 1.159274991 | 0.891344903 | 1.687677876 | 2.247296849 | 2.461145454 | 1.193142796 | 1.587643437 | 2.093087347 | 4.767749322 | 5.451516355 | 2.235938094 | 2.136335718 | 2.103272391 | 2.817539613 |
| ENSMUSG00000012187 | Mogat1 | 1549 | 2.524516588 | 1.376388141 | 1.061781815 | 1.353007127 | 1.491499249 | 0.863733897 | 0.946864459 | 1.405401509 | 2.073949793 | 0.913183407 | 0.771061305 | 1.585467498 | 0.891732527 | 0.359799257 | 1.968311902 | 0.692147842 | 0.940219033 | 0.781278469 |
| ENSMUSG00000012211 | Tex22 | 1504 | 0 | 0.022149531 | 0.032163252 | 0.030293248 | 0 | 0 | 0 | 0 | 0 | 0.094050605 | 0 | 0 | 0 | 0 | 0 | 0.076377541 | 0 | 0 |
| ENSMUSG00000012282 | Wnt8a | 1740 | 0 | 0 | 0 | 0 | 0 | 0.11391433 | 0 | 0 | 0 | 0 | 0 | 0 | 0 | 0 | 0 | 0 | 0 | 0 |
| ENSMUSG00000012296 | Tjap1 | 4152 | 7.10467056 | 7.959150064 | 8.260316238 | 9.557723896 | 7.477141321 | 8.843587568 | 7.532196718 | 8.140721611 | 6.626346948 | 7.93794354 | 9.021090426 | 9.006862701 | 7.497215752 | 7.81898292 | 6.192141348 | 8.263098322 | 8.56618582 | 10.73154539 |
| ENSMUSG00000012350 | Ehf | 7022 | 36.17964072 | 36.9326234 | 36.92425558 | 41.58369882 | 39.433631 | 38.71351416 | 28.54796648 | 33.13960522 | 28.54081255 | 34.50690174 | 28.59557825 | 33.27319333 | 23.82994352 | 20.96664156 | 29.60738408 | 31.20177379 | 28.93848279 | 26.12580047 |
| ENSMUSG00000012396 | Nanog | 1159 | 0.036674009 | 0 | 0 | 0 | 0 | 0 | 0 | 0 | 0 | 0 | 0 | 0 | 0 | 0.040072552 | 0.071098457 | 0 | 0.066136837 | 0.04746256 |
| ENSMUSG00000012405 | Rpl15 | 2762 | 26.99278738 | 26.73956788 | 25.88561834 | 25.15589885 | 24.20537363 | 24.00490859 | 28.04158414 | 24.28162876 | 24.45542572 | 25.06054757 | 21.24100254 | 21.98676354 | 30.61365178 | 32.05011968 | 24.8372884 | 25.84130782 | 24.3806313 | 25.27391616 |
| ENSMUSG00000012422 | Tmem167 | 10849 | 6.127578146 | 6.018367838 | 6.425132016 | 6.278344666 | 6.286564277 | 5.709366483 | 8.857226556 | 6.442261628 | 7.349718363 | 7.218851333 | 7.191124269 | 6.374376066 | 8.725960849 | 7.941172652 | 8.108150228 | 8.749416144 | 8.443160623 | 7.524519714 |
| ENSMUSG00000012428 | Steap4 | 3135 | 0.244048858 | 0.765080824 | 0.447474443 | 0.101731199 | 0.353120971 | 0.474188838 | 0.316927036 | 0.316747197 | 0.262752892 | 0.195521258 | 0.563851185 | 0 | 0.031471718 | 0.592588037 | 0.289133726 | 0 | 0.440110586 | 0 |
| ENSMUSG00000012429 | 2810021B07R | 3070 | 17.37589443 | 15.88601861 | 16.57620649 | 16.96295559 | 18.15529889 | 17.01256716 | 16.42844161 | 16.9439909 | 17.01123843 | 15.1127987 | 18.05177584 | 16.92650875 | 17.43489585 | 15.21913743 | 14.70908966 | 14.04404688 | 14.5065658 | 12.43528341 |
| ENSMUSG00000012443 | Kif11 | 4797 | 0.097468613 | 0.138890532 | 0.231934792 | 0.180458587 | 0.120405062 | 0.020659885 | 0.019725945 | 0.015923453 | 0.30050616 | 0.098292064 | 0.019918682 | 0 | 0.226246025 | 0.10650093 | 0.060123179 | 0.359198944 | 0.239689161 | 0.160543568 |
| ENSMUSG00000012483 | Rpa3 | 1132 | 12.69147482 | 13.06618819 | 15.46927384 | 11.79274381 | 15.13689472 | 12.65083039 | 18.80804374 | 16.05970278 | 20.92071251 | 13.70369379 | 17.85230339 | 14.2990655 | 18.43409983 | 18.2986422 | 17.17944733 | 16.87898683 | 16.59000484 | 14.57838517 |
| ENSMUSG00000012519 | Mlkl | 2567 | 1.010056774 | 0.09084155 | 0.923374752 | 0.976181316 | 0.112501575 | 0.482593821 | 0.258035647 | 0.49098141 | 0.513427544 | 0.440832442 | 0.818892938 | 0.543588455 | 1.153063902 | 0.452318732 | 0.337059865 | 0.402744994 | 0.283677305 | 0.064288009 |
| ENSMUSG00000012520 | Phox2b | 3644 | 0 | 0 | 0 | 0 | 0 | 0.040795335 | 0 | 0 | 0 | 0 | 0 | 0 | 0 | 0.089217511 | 0 | 0 | 0 | 0 |
| ENSMUSG00000012535 | Tnpo3 | 10705 | 8.18338793 | 8.632411823 | 8.522417398 | 8.814285177 | 9.608399895 | 9.151401615 | 7.858191792 | 9.279629954 | 7.725597725 | 9.218722272 | 9.443420097 | 8.649993856 | 5.645175027 | 8.343015418 | 7.539826996 | 6.856898121 | 8.603278044 | 6.104700494 |
| ENSMUSG00000012609 | Ttll5 | 13635 | 6.487221952 | 7.019284568 | 7.844068619 | 7.160786066 | 7.691929716 | 6.955917277 | 4.719123091 | 5.198760459 | 3.570404227 | 5.173263812 | 4.018912899 | 4.028061018 | 3.918332703 | 4.084082199 | 4.816668873 | 4.400541386 | 4.652000101 | 4.962317658 |
| ENSMUSG00000012640 | Zfp715 | 5168 | 6.810039812 | 8.392683489 | 8.948354299 | 8.577959779 | 10.30067871 | 8.85966057 | 8.807042788 | 8.631719429 | 8.399881504 | 8.649161511 | 7.950167239 | 8.812999356 | 6.872867732 | 8.995845874 | 6.824406316 | 7.690721843 | 8.409833724 | 7.280617066 |
| ENSMUSG00000012705 | Retn | 1628 | 0.182761813 | 0.061387397 | 0 | 0.111943598 | 0 | 0 | 0 | 0.023459707 | 0 | 0 | 0 | 0 | 0 | 0 | 0 | 0 | 0 | 0 |
| ENSMUSG00000012777 | Acpt | 1889 | 0.022501417 | 0.123446405 | 0.02560801 | 0.072357402 | 0.22932097 | 0.052464514 | 0.150278493 | 0.28305645 | 0.283443465 | 0.124803344 | 0.025291137 | 0.177286382 | 0.026115361 | 0.122933 | 0.327169581 | 0.222973362 | 0.040578398 | 0.058241511 |
| ENSMUSG00000012819 | Cdh23 | 17146 | 0 | 0 | 0 | 0.005314481 | 0 | 0 | 0.005518801 | 0.002227482 | 0 | 0.013749768 | 0.025077256 | 0.003255317 | 0 | 0.035213644 | 0 | 0 | 0 | 0 |
| ENSMUSG00000012848 | Rps5 | 1750 | 68.37261172 | 63.12317556 | 65.78800128 | 64.61857831 | 74.64573762 | 65.52287171 | 72.75338228 | 64.22870873 | 67.89891313 | 65.87626827 | 66.28434239 | 58.74997171 | 96.63417853 | 99.41688649 | 77.41183758 | 65.92548577 | 71.79062928 | 82.82799784 |
| ENSMUSG00000012889 | Podnl1 | 3466 | 0.012263467 | 0.096113371 | 0.027913174 | 0.157741642 | 0.152755672 | 0.042890421 | 0.04095154 | 0 | 0.059415054 | 0.027207561 | 0.068919444 | 0.53142437 | 0 | 0 | 0 | 0.198854857 | 0.011057789 | 0.269808083 |
| ENSMUSG00000013033 | Lphn1 | 8863 | 1.875157828 | 2.262705892 | 1.964850607 | 1.830049846 | 2.297168421 | 1.593425372 | 2.097922015 | 1.422034615 | 2.016805578 | 1.436386086 | 1.444622461 | 1.983745201 | 2.337741764 | 1.734513475 | 1.366722039 | 2.242223315 | 1.738369779 | 1.768881351 |
| ENSMUSG00000013076 | Amotl1 | 10303 | 1.732716095 | 2.192190845 | 1.84517106 | 2.043016838 | 2.083552165 | 2.140247155 | 1.249058397 | 1.475354378 | 1.934802837 | 1.748186804 | 1.67395516 | 1.825670027 | 2.446725199 | 1.4515186 | 1.663578302 | 2.148109393 | 2.060833591 | 1.740557973 |
| ENSMUSG00000013083 | 2200002J24R | 331 | 1.027315433 | 0.40257274 | 0.438430789 | 1.651759915 | 0.145413667 | 0.299412287 | 0 | 0 | 0.871013929 | 0.142449255 | 0.433005666 | 0.505882138 | 0.149039025 | 0.561257854 | 0 | 0.925452747 | 0.115789417 | 0 |
| ENSMUSG00000013089 | Etv5 | 6467 | 1.38025158 | 2.53954799 | 1.271609736 | 1.810605906 | 0.915451772 | 2.329369261 | 1.638787704 | 2.315048978 | 1.955197207 | 2.500802724 | 1.713899856 | 2.244019216 | 3.196238339 | 2.032422566 | 2.866970798 | 3.860441631 | 3.733658118 | 1.718237136 |
| ENSMUSG00000013091 | Tmem190 | 581 | 0.073158651 | 0.458697339 | 0.083259088 | 0.235254962 | 0.331372968 | 0.597020886 | 1.465797282 | 0 | 0 | 0.243463184 | 0 | 0 | 0 | 0 | 0.070914898 | 0.527237279 | 0 | 0 |
| ENSMUSG00000013150 | Gfod2 | 4836 | 3.796988429 | 3.685359472 | 2.840794587 | 3.664856544 | 2.468303778 | 3.033004371 | 2.876329116 | 3.909271958 | 4.011341796 | 3.675726867 | 2.687094439 | 3.254759481 | 3.906973597 | 3.121242432 | 3.365304917 | 2.335758723 | 3.098755602 | 2.183984386 |
| ENSMUSG00000013155 | E130303B06R | 1265 | 3.763304123 | 4.055482774 | 4.129914054 | 4.646146023 | 4.489776279 | 3.564663048 | 3.403520777 | 3.56261145 | 4.818659552 | 5.292806215 | 4.720845699 | 4.63292061 | 3.275795297 | 5.103342409 | 3.322180791 | 4.056080152 | 3.787183488 | 3.00049673 |
| ENSMUSG00000013158 | 4933405L10R | 1120 | 0 | 0 | 0 | 0 | 0 | 0.132730536 | 0 | 0 | 0 | 0 | 0 | 0 | 0 | 0 | 0.367871035 | 0 | 0 | 0 |
| ENSMUSG00000013160 | Atp6v0d1 | 1785 | 61.41224032 | 57.81700072 | 68.34624289 | 62.53476674 | 58.37849565 | 61.15667903 | 60.59203587 | 59.28915802 | 68.34423512 | 63.2902437 | 60.14024185 | 60.34970801 | 72.82330646 | 68.92458683 | 66.15331047 | 75.46574613 | 61.4079603 | 73.62283247 |
| ENSMUSG00000013236 | Ptprs | 7912 | 11.92637649 | 16.93433526 | 17.02733595 | 16.6074383 | 17.25862834 | 17.56140861 | 6.70342683 | 9.05573141 | 9.593876207 | 8.301431956 | 9.419733977 | 11.25907451 | 3.323295236 | 2.647406902 | 4.540919709 | 3.17475208 | 3.366629973 | 2.85752564 |
| ENSMUSG00000013275 | Slc41a1 | 4900 | 7.416719491 | 6.594593345 | 6.367536139 | 6.462229758 | 7.2787256 | 6.82614187 | 4.59608881 | 5.596356108 | 5.463517397 | 5.513745503 | 5.469745246 | 5.706866726 | 4.308991958 | 4.843454828 | 6.070923133 | 3.250794424 | 6.312106449 | 3.98535365 |
| ENSMUSG00000013338 | Fer1l4 | 6808 | 0 | 0.004893198 | 0 | 0.086999644 | 0.035349533 | 0.072786036 | 0 | 0 | 0 | 0 | 0 | 0.00819854 | 0 | 0 | 0 | 0 | 0 | 0 |
| ENSMUSG00000013353 | 4931406B18R | 5695 | 0 | 0 | 0 | 0 | 0 | 0 | 0 | 0 | 0 | 0 | 0 | 0 | 0 | 0 | 0 | 0 | 0 | 0 |
| ENSMUSG00000013367 | Iglon5 | 2611 | 0 | 0 | 0 | 0 | 0 | 0 | 0 | 0 | 0 | 0 | 0 | 0 | 0 | 0.106727125 | 0 | 0.014665112 | 0 | 0 |
| ENSMUSG00000013415 | Igf2bp1 | 9821 | 0.004327989 | 0 | 0 | 0 | 0 | 0 | 0 | 0 | 0 | 0 | 0 | 0 | 0 | 0.004729059 | 0 | 0 | 0 | 0 |

| ENSMUSG00000013418 | B4galnt2 | 1711 | 0.0248423 | 0.720384036 | 0.169632485 | 0.452681329 | 0.056261746 | 0.434419055 | 0.276520625 | 0.223216846 | 0.04814321 | 0.05511479 | 0.027922243 | 0 | 0 | 0 | 0.024080395 | 0 | 0 | 0 |
| --- | --- | --- | --- | --- | --- | --- | --- | --- | --- | --- | --- | --- | --- | --- | --- | --- | --- | --- | --- | --- |
| ENSMUSG00000013419 | Zfp651 | 6347 | 5.987021801 | 6.833683358 | 5.708488142 | 6.316955887 | 5.763394042 | 6.542491057 | 4.539691424 | 5.505915897 | 4.587815762 | 6.188204792 | 3.914129259 | 6.226168128 | 3.171170986 | 4.771001257 | 4.881608639 | 3.704180391 | 4.450367231 | 3.804789326 |
| ENSMUSG00000013465 | Cobra1 | 4174 | 26.53773089 | 30.71905363 | 27.90691449 | 26.9938818 | 27.57149724 | 27.34066733 | 23.02157425 | 25.89470503 | 26.37555262 | 27.28053387 | 27.87063342 | 29.17819256 | 25.78875024 | 25.66998314 | 23.75949809 | 27.26393991 | 29.32778924 | 30.12717249 |
| ENSMUSG00000013483 | Card14 | 4583 | 0.148392498 | 0.145375929 | 0.031664977 | 0.28829812 | 0 | 0.108122919 | 0.061941103 | 0.733347461 | 0 | 0.185187139 | 0.271034021 | 0.341007757 | 0.075348772 | 0.202679849 | 0 | 0 | 0.016725419 | 0.156037178 |
| ENSMUSG00000013495 | Tmem175 | 3931 | 13.22407283 | 14.22005507 | 13.15474534 | 13.83868912 | 15.39095092 | 16.4377422 | 10.11006113 | 15.14677063 | 13.68343668 | 12.21048484 | 12.26276599 | 14.52541919 | 11.82158893 | 12.94905108 | 9.286333889 | 12.01999734 | 14.58563728 | 11.16694662 |
| ENSMUSG00000013523 | Bcas1 | 4762 | 0 | 0 | 0 | 0 | 0 | 0 | 0 | 0 | 0.060542967 | 0.059408698 | 0 | 0 | 0 | 0 | 0.051912922 | 0 | 0 | 0 |
| ENSMUSG00000013539 | D16H22S680E | 7285 | 5.041108042 | 4.367030059 | 4.608267682 | 6.379171616 | 5.212915274 | 5.121922908 | 6.981623864 | 5.567650152 | 6.026743418 | 6.116323209 | 6.977701554 | 4.918827094 | 8.207314171 | 6.815199646 | 6.88295038 | 8.751387965 | 6.507842041 | 5.670808394 |
| ENSMUSG00000013562 | 4833413E03R | 1855 | 0 | 0.017958434 | 0 | 0 | 0 | 0 | 0 | 0 | 0 | 0 | 0 | 0 | 0 | 0 | 0 | 0 | 0 | 0 |
| ENSMUSG00000013584 | Aldh1a2 | 2250 | 0.037782379 | 0.325726077 | 0 | 0.040498706 | 0 | 0 | 0 | 0.95056646 | 0 | 0.125735209 | 0 | 0 | 0 | 0 | 0 | 0.102108286 | 0 | 0 |
| ENSMUSG00000013593 | Ndufs2 | 2128 | 132.4891131 | 123.9371163 | 130.0493267 | 129.8960789 | 136.9769407 | 127.9801803 | 143.2942743 | 133.8708316 | 134.6303589 | 140.3442455 | 154.9992074 | 130.6999279 | 131.8376943 | 113.2292882 | 118.5125581 | 112.1367788 | 119.3914578 | 113.8181846 |
| ENSMUSG00000013611 | Snx31 | 3978 | 0.138905804 | 0.209357053 | 0.376968185 | 0.240518334 | 0 | 0.411070942 | 0.392488287 | 0.480045279 | 0.093182162 | 0.308174531 | 0.012009793 | 0.056124346 | 0.545652177 | 0.525385604 | 0.652513329 | 0.375398112 | 0.308306059 | 0.622274963 |
| ENSMUSG00000013622 | 0610007C21R | 1581 | 76.2727289 | 68.94357361 | 76.49198348 | 69.65277935 | 75.47061276 | 73.09106559 | 97.97704669 | 97.42565395 | 103.5784862 | 92.84006277 | 97.12126284 | 102.3816708 | 93.54654522 | 107.987644 | 89.12670534 | 87.94003509 | 92.91884632 | 91.6122568 |
| ENSMUSG00000013629 | Cad | 8254 | 8.563860884 | 10.79621905 | 11.70365158 | 9.499704059 | 10.04157652 | 8.837124282 | 19.15091596 | 17.42580415 | 13.19325753 | 9.911130379 | 8.43325836 | 13.07144121 | 28.84369188 | 39.18544034 | 21.81875464 | 8.303875363 | 10.96297395 | 20.55343895 |
| ENSMUSG00000013643 | 2210415F13R | 1277 | 3.594799539 | 4.382589059 | 3.409254293 | 6.850211834 | 7.500589514 | 7.333959786 | 0 | 0 | 0.032252557 | 0.036923025 | 0.074823741 | 0 | 0.077262204 | 0.909242118 | 0.258114681 | 0.089954442 | 0.330140381 | 0.129230478 |
| ENSMUSG00000013646 | Sh3bp5l | 3303 | 11.90350827 | 11.94140683 | 9.695209535 | 15.17322094 | 11.93461868 | 14.82231328 | 12.26147537 | 9.400673072 | 9.464294721 | 12.37652429 | 10.52987277 | 10.37566359 | 10.30548681 | 10.58807079 | 8.918895689 | 6.886049287 | 6.938881486 | 8.993314451 |
| ENSMUSG00000013653 | 1810065E05R | 920 | 0 | 0 | 0 | 0 | 0 | 0 | 0 | 0.124540443 | 0 | 0 | 0 | 0.121338397 | 0 | 0 | 0 | 0 | 0 | 0 |
| ENSMUSG00000013662 | Atad1 | 2830 | 53.70972066 | 54.3835941 | 60.44127326 | 55.52651658 | 55.19013862 | 58.4126923 | 54.85261469 | 54.96736926 | 44.14088421 | 51.28263768 | 57.38059501 | 49.05072539 | 38.47192277 | 42.65306824 | 36.8630175 | 45.09632313 | 57.25921672 | 46.63139471 |
| ENSMUSG00000013663 | Pten | 12426 | 9.04081605 | 9.289327094 | 9.4987457 | 10.01712322 | 9.478417609 | 10.36037356 | 9.827299558 | 11.39993727 | 10.75900774 | 9.877135086 | 11.2190028 | 10.82085395 | 8.341087896 | 9.359085371 | 9.111691259 | 9.540296195 | 11.14071981 | 9.566608694 |
| ENSMUSG00000013668 | 4933402N03R | 2157 | 0 | 0 | 0 | 0 | 0 | 0 | 0 | 0 | 0 | 0 | 0 | 0 | 0 | 0 | 0 | 0 | 0 | 0 |
| ENSMUSG00000013698 | Pea15a | 3622 | 27.07328578 | 28.67741695 | 28.20676314 | 33.42233429 | 30.81665682 | 28.96276559 | 20.82175876 | 18.76931979 | 18.11433451 | 19.33152798 | 21.46047969 | 24.31725996 | 16.42581232 | 19.7214264 | 18.56458841 | 19.81131924 | 21.70271534 | 18.84768124 |
| ENSMUSG00000013701 | Timm23 | 2230 | 15.78219092 | 14.96839462 | 13.6877568 | 13.83176099 | 14.74175061 | 14.22141233 | 15.488007 | 15.56811381 | 14.23982227 | 13.55318421 | 15.53221744 | 13.29063535 | 15.2420139 | 14.2039765 | 14.13416603 | 15.04151215 | 14.62586487 | 13.32059087 |
| ENSMUSG00000013707 | Tnfaip8l2 | 1120 | 0 | 0 | 0 | 0 | 0 | 0.132730536 | 0.295704243 | 0.068200719 | 0.147094699 | 0.126296527 | 0.383905916 | 0 | 0.220231774 | 1.161102185 | 0.183935517 | 0.44444455 | 0.17109954 | 0.098230548 |
| ENSMUSG00000013736 | Trnt1 | 3724 | 11.08284799 | 11.71855301 | 12.62595905 | 12.27114057 | 11.69693634 | 9.873289021 | 12.7302106 | 11.51720406 | 9.98695589 | 9.166785488 | 12.44406813 | 11.34598727 | 12.71714301 | 11.97269707 | 13.33186757 | 11.09440531 | 14.23342337 | 14.7271964 |
| ENSMUSG00000013766 | Ly6g6e | 1299 | 13.67757012 | 11.66848874 | 14.15084029 | 10.94306839 | 13.07972983 | 12.09254545 | 6.155383171 | 8.761613485 | 8.592413989 | 9.038125567 | 8.569334354 | 8.46473097 | 7.519414643 | 8.008834161 | 6.08983736 | 5.246903863 | 6.904044246 | 5.33575631 |
| ENSMUSG00000013787 | Ehmt2 | 5392 | 14.70969185 | 13.64766011 | 12.21001017 | 12.75912035 | 10.15840674 | 10.07229154 | 12.96886859 | 14.47798044 | 11.94653387 | 14.69087193 | 9.684352674 | 11.87325018 | 13.11980144 | 14.4620962 | 12.06551498 | 13.44290056 | 12.36790738 | 12.96672378 |
| ENSMUSG00000013822 | Elof1 | 1778 | 55.15154167 | 48.54539309 | 44.37414399 | 44.12605081 | 49.70203141 | 44.89845545 | 54.86993473 | 43.56253773 | 46.4448617 | 51.65892573 | 48.58106014 | 44.16908732 | 61.06836329 | 62.58719538 | 47.1338384 | 56.05762152 | 46.75463329 | 47.7075605 |
| ENSMUSG00000013833 | Med16 | 5366 | 13.08582758 | 16.82406696 | 15.42435901 | 17.49082209 | 15.06028696 | 17.38870617 | 12.89063298 | 13.18869207 | 11.92766409 | 14.9905143 | 13.03439046 | 13.42862848 | 9.855351346 | 11.39896815 | 14.01282883 | 11.96670679 | 14.14201787 | 13.10131166 |
| ENSMUSG00000013846 | St3gal1 | 4790 | 7.70239933 | 7.21895286 | 6.544060056 | 6.601119994 | 8.310041936 | 7.127731406 | 6.104224546 | 8.651097413 | 6.801364085 | 5.975047365 | 7.759690542 | 7.259531898 | 6.436836805 | 4.382615344 | 4.705062854 | 10.78372221 | 7.561242291 | 5.294195866 |
| ENSMUSG00000013858 | ORF61 | 6618 | 7.264030536 | 6.568952396 | 5.4601129 | 6.044514495 | 5.294657061 | 5.233810936 | 5.018661319 | 5.540149034 | 6.87686611 | 5.792312141 | 6.937403309 | 5.70132788 | 7.126218331 | 5.445846451 | 6.985213918 | 5.704826065 | 5.907044854 | 7.5307118 |
| ENSMUSG00000013878 | Rnf170 | 5241 | 4.103724303 | 4.5955395 | 3.894987562 | 4.642167082 | 5.363297734 | 4.046664753 | 3.68318508 | 4.860586225 | 4.974444663 | 4.786142746 | 3.427472694 | 4.238625016 | 3.416807092 | 3.872556037 | 4.142953626 | 3.243852258 | 4.087845825 | 4.807130486 |
| ENSMUSG00000013921 | Clip3 | 3783 | 0.292131794 | 0.061641623 | 0.191806227 | 0.28904707 | 0.101785723 | 0.09169155 | 0.15007987 | 0.363448714 | 0.195970733 | 0 | 0.113659695 | 0.014754339 | 0.508576467 | 0.626129648 | 0.294063444 | 0.708523002 | 0.293804549 | 0.348987196 |
| ENSMUSG00000013928 | Thap8 | 1181 | 0.07198167 | 0 | 0.040959806 | 0 | 0.081510455 | 0.083916568 | 0 | 0.032339037 | 0.104622817 | 0 | 0 | 0.283568142 | 0.417713101 | 0 | 0.104661023 | 0.032422191 | 0.032452411 | 0 |
| ENSMUSG00000013936 | Myl2 | 1901 | 0 | 0.017523879 | 0.02544636 | 0.047933766 | 0.050638531 | 0.286733335 | 0 | 0 | 0 | 0.049606211 | 0 | 0 | 0 | 0 | 0 | 0 | 0 | 0.057873863 |
| ENSMUSG00000013973 | Dedd | 5978 | 1.265627523 | 1.09222604 | 0.922479502 | 1.089867738 | 0.917872081 | 1.044437008 | 1.036795072 | 1.201099306 | 1.191914892 | 1.041132959 | 1.086884301 | 1.139095155 | 1.006773822 | 0.908992677 | 0.94423104 | 1.037651121 | 1.166842763 | 1.067423282 |
| ENSMUSG00000013974 | 1810033B17R | 1125 | 0 | 0 | 0 | 0 | 0 | 0 | 0 | 0.611078438 | 0 | 0 | 0 | 0 | 0 | 0 | 0.512730473 | 0.034036095 | 0 | 0 |
| ENSMUSG00000013989 | D430018E03R | 3830 | 0 | 0.09567672 | 0 | 0.261708349 | 0.100536655 | 0.051752202 | 0 | 0.039887627 | 0.107536595 | 0.012310889 | 0.012473879 | 0 | 0.038641188 | 0.048505574 | 0.021515173 | 0 | 0 | 0 |
| ENSMUSG00000013997 | Nit1 | 4599 | 16.10926763 | 17.29749759 | 16.3874669 | 18.05005931 | 16.35794666 | 16.78694475 | 18.06502809 | 17.44775765 | 17.51703084 | 19.69482517 | 19.92441191 | 17.94985105 | 16.89449222 | 15.92570685 | 16.99485791 | 18.49172408 | 18.07561165 | 18.03734137 |
| ENSMUSG00000014030 | Pax5 | 3496 | 0 | 0 | 0 | 0 | 0 | 0 | 0.1624005 | 0 | 0.08246728 | 0.067435216 | 0.095659242 | 0.063862314 | 0.155220564 | 0.079709532 | 0.035356026 | 0.109526909 | 0 | 0 |
| ENSMUSG00000014039 | Prdm15 | 13654 | 0.762689917 | 0.619706689 | 0.91758784 | 0.750786215 | 0.525242173 | 0.616959478 | 1.282092515 | 0.581808972 | 0.669650395 | 0.66302439 | 0.668303579 | 0.674497168 | 0.863507267 | 0.734724101 | 0.866035341 | 0.482348357 | 0.729810839 | 0.467339709 |
| ENSMUSG00000014074 | Rnf168 | 5410 | 5.774732788 | 7.844847918 | 6.107046439 | 5.82777129 | 7.446658061 | 6.924559443 | 5.457137089 | 7.687897638 | 5.938166783 | 6.048537534 | 6.82625562 | 6.819621621 | 4.632276151 | 6.833547793 | 5.696629169 | 6.738014463 | 6.935572027 | 6.619395282 |
| ENSMUSG00000014075 | Tctex1d2 | 2562 | 11.5636642 | 8.139684536 | 9.610510129 | 9.638597201 | 10.65214704 | 8.935738842 | 13.3147703 | 10.82267141 | 12.28200548 | 11.07912699 | 12.32601388 | 10.17404935 | 11.76494983 | 9.190925959 | 10.21193911 | 8.743171479 | 9.948082529 | 9.297011402 |

| ENSMUSG00000014077 | 1500003O03R | 3688 | 43.41567194 | 47.90083461 | 45.55348995 | 46.79643057 | 45.79580264 | 48.58532663 | 43.0406827 | 41.19560108 | 42.10226801 | 43.40472819 | 41.22015127 | 39.54618393 | 39.44680695 | 32.96925726 | 45.99425315 | 39.92065766 | 42.28571098 | 39.89950122 |
| --- | --- | --- | --- | --- | --- | --- | --- | --- | --- | --- | --- | --- | --- | --- | --- | --- | --- | --- | --- | --- |
| ENSMUSG00000014104 | Sval2 | 1203 | 0 | 0 | 0 | 0 | 0 | 0 | 0 | 0.095242899 | 0.171182526 | 0 | 0 | 0 | 0 | 0 | 0 | 0 | 0 | 0 |
| ENSMUSG00000014158 | Trpv4 | 4716 | 9.634867085 | 9.140560882 | 7.108324117 | 7.60316834 | 8.236315182 | 9.677283212 | 8.296773849 | 6.503074455 | 5.580615685 | 6.008815237 | 5.348850313 | 8.249261413 | 3.901782261 | 5.081668595 | 5.075933836 | 4.157080363 | 4.859865468 | 4.654078368 |
| ENSMUSG00000014164 | Klhl3 | 3237 | 1.142400468 | 0.679224905 | 0.821916642 | 0.900805325 | 1.903270383 | 1.316507596 | 1.154680765 | 2.430532869 | 2.086681676 | 2.432550956 | 2.110540334 | 2.12089172 | 1.493521129 | 1.320004955 | 1.83287737 | 1.656065813 | 1.491848443 | 1.478465334 |
| ENSMUSG00000014177 | Fam18b | 2332 | 51.63686264 | 48.64082539 | 53.10301789 | 55.05618479 | 52.42499405 | 52.61259363 | 67.8445962 | 69.12955856 | 68.685403 | 64.47838451 | 57.34224219 | 63.6183667 | 78.0595089 | 72.2751257 | 68.97550352 | 74.18394693 | 66.62727601 | 61.40167444 |
| ENSMUSG00000014195 | Dnajc7 | 4835 | 39.41948486 | 38.32877571 | 36.28765142 | 33.28264914 | 34.34439228 | 33.87213743 | 39.3668888 | 36.93643716 | 36.16917186 | 38.01312126 | 36.85637955 | 37.54137223 | 36.34338973 | 34.70578857 | 36.54880522 | 38.44107721 | 37.28788017 | 29.38749176 |
| ENSMUSG00000014198 | Zfp385c | 5945 | 0.271689939 | 0.184915981 | 0.048821057 | 0.329541616 | 0.113346835 | 0.400089354 | 0.047750391 | 0.45612457 | 0.041567552 | 0.214141125 | 0.385735577 | 0.300437545 | 1.352582425 | 0.429676166 | 0.693045515 | 1.301043347 | 0.857425986 | 0.897541354 |
| ENSMUSG00000014226 | Cacybp | 1436 | 106.5589371 | 97.78123199 | 106.5160884 | 89.3770626 | 102.4646871 | 97.0005112 | 96.53631564 | 97.79489112 | 95.99670491 | 88.7523335 | 87.39889688 | 91.14744343 | 84.57881639 | 81.56823704 | 93.93725208 | 102.9793355 | 108.8936569 | 94.46549943 |
| ENSMUSG00000014232 | Cluap1 | 2157 | 3.94113825 | 4.16990331 | 4.036734105 | 3.717544647 | 3.34714351 | 5.07703019 | 6.7996896 | 5.5066468 | 4.372606449 | 5.31183166 | 4.075378934 | 5.201181354 | 5.626171372 | 6.308816692 | 5.233762779 | 6.656920619 | 5.863550291 | 7.905805788 |
| ENSMUSG00000014243 | Zswim7 | 727 | 7.483717375 | 12.50951874 | 10.51309188 | 8.397771583 | 10.52678936 | 10.97385159 | 20.76030891 | 19.64781087 | 16.48593685 | 13.49015994 | 18.86026559 | 17.04412255 | 14.04636434 | 24.02060091 | 25.84306669 | 21.75243584 | 16.13183886 | 17.40315618 |
| ENSMUSG00000014245 | Pigl | 4932 | 4.050574358 | 3.809503718 | 3.540722721 | 4.868343545 | 2.81062328 | 3.596893475 | 4.489524278 | 4.228112675 | 4.868560156 | 3.929225271 | 3.419416128 | 3.791209847 | 2.750664487 | 1.949295639 | 3.809389596 | 3.579069346 | 2.984042581 | 3.312592193 |
| ENSMUSG00000014294 | Ndufa2 | 526 | 296.8897656 | 231.1636196 | 262.9276108 | 241.5774765 | 274.2421582 | 253.7928978 | 337.1253244 | 278.3834046 | 286.269775 | 304.7764089 | 288.9204238 | 260.6145766 | 406.8476372 | 376.1441298 | 308.5416895 | 332.8225419 | 278.9221759 | 328.9042598 |
| ENSMUSG00000014301 | Pam16 | 883 | 36.53615924 | 28.10657553 | 32.70554657 | 27.50174024 | 32.9782716 | 26.09515415 | 43.99061087 | 29.75806665 | 32.46411661 | 33.5875338 | 34.03221844 | 25.97988372 | 42.29248174 | 41.81545807 | 36.16218099 | 40.63227535 | 32.72709837 | 35.57214036 |
| ENSMUSG00000014303 | Glis2 | 4203 | 1.243905937 | 0.943191628 | 0.771122183 | 0.737128483 | 0.69855992 | 0.565912732 | 0.236394541 | 0.408912231 | 0.921135494 | 0.437515448 | 0.602444159 | 0.531198312 | 0.023474622 | 0.430958698 | 0.382312796 | 0.856368569 | 0.282682656 | 0.170144751 |
| ENSMUSG00000014313 | Cox6c | 1590 | 224.6358454 | 189.883497 | 226.3820373 | 184.3646285 | 222.5571716 | 200.5483274 | 338.4493145 | 259.3240103 | 297.8639904 | 286.4331087 | 286.259138 | 242.9912053 | 360.7435234 | 366.4409285 | 305.6170756 | 275.7164554 | 291.7137391 | 331.5077111 |
| ENSMUSG00000014329 | Bicc1 | 7694 | 1.325869801 | 0.718734136 | 0.81733285 | 1.048129042 | 0.70690244 | 1.127076732 | 0.873199949 | 1.131773817 | 0.797607337 | 0.998903643 | 0.738916046 | 0.797988418 | 0.878408197 | 0.645895159 | 0.696153141 | 0.408088096 | 0.537982852 | 0.536220822 |
| ENSMUSG00000014349 | Ube2z | 3990 | 46.48937047 | 52.98336509 | 53.65945999 | 54.37636419 | 52.05244377 | 51.56464908 | 48.05972123 | 50.234017 | 53.6044051 | 47.88337083 | 53.0673223 | 53.63339608 | 48.15734782 | 45.23351469 | 48.30598458 | 44.56681222 | 48.64269863 | 50.18374648 |
| ENSMUSG00000014351 | Gip | 680 | 0 | 0 | 0 | 0 | 0 | 0 | 0 | 0 | 0 | 0 | 0 | 0 | 0 | 0 | 0 | 0 | 0 | 0 |
| ENSMUSG00000014353 | Tmem87b | 7302 | 14.73885315 | 15.92650147 | 15.00493649 | 15.67369808 | 15.2002487 | 16.74152201 | 13.28279811 | 13.04985538 | 10.30508961 | 13.54081412 | 14.99591959 | 14.30935639 | 11.17433459 | 12.72092232 | 14.1908947 | 12.78975506 | 16.13462568 | 14.59225414 |
| ENSMUSG00000014355 | Anapc1 | 15679 | 4.809246908 | 6.764988532 | 5.750893589 | 6.253419696 | 6.382184406 | 6.276658517 | 3.841382759 | 4.637944651 | 4.780882626 | 5.307799684 | 5.701061759 | 6.04114927 | 4.499307463 | 4.90241499 | 4.430500876 | 4.684060527 | 4.673759786 | 4.017183953 |
| ENSMUSG00000014361 | Mertk | 4317 | 0.147689979 | 0.231500307 | 0.179285728 | 0.200523475 | 0.167240874 | 0.022957023 | 0.295909736 | 0 | 0.333918937 | 0.010922099 | 0.055333517 | 0 | 0.102846249 | 0.225926763 | 0.085896225 | 0.266091782 | 0.195315852 | 0.038227315 |
| ENSMUSG00000014372 | Cyp2d10 | 1633 | 0 | 0 | 0 | 0 | 0 | 0 | 0 | 0 | 0 | 0 | 0 | 0 | 0 | 0 | 0 | 0 | 0.02346987 | 0 |
| ENSMUSG00000014402 | Tsg101 | 3395 | 61.61059536 | 51.33816276 | 56.72312941 | 51.70742381 | 57.40387597 | 55.36186111 | 53.6814254 | 50.80320744 | 55.47744818 | 51.31718661 | 51.64480048 | 47.16793401 | 59.21283149 | 54.57009266 | 57.68217825 | 58.01675537 | 59.84321057 | 53.50222987 |
| ENSMUSG00000014418 | Hps5 | 6930 | 5.268679106 | 4.874354219 | 5.891379455 | 5.522550829 | 5.29242797 | 5.491558353 | 4.758576798 | 5.637926069 | 5.271636289 | 5.225359322 | 6.101131061 | 5.339940014 | 3.60913161 | 4.496967192 | 4.940619473 | 4.735216529 | 6.100130661 | 5.183397791 |
| ENSMUSG00000014426 | Map3k4 | 5364 | 6.743457271 | 6.235299365 | 5.50109126 | 4.807522575 | 5.374910939 | 5.976998252 | 3.669290536 | 3.916074072 | 3.923622214 | 3.674309091 | 4.052499274 | 4.911445328 | 3.402835457 | 3.065101964 | 4.30911127 | 3.583498306 | 3.251018846 | 2.768914768 |
| ENSMUSG00000014444 | Fam38a | 9248 | 0.712402929 | 0.997801871 | 0.800297377 | 0.955757202 | 0.978460386 | 1.034134686 | 0.634382806 | 1.160474165 | 1.429592513 | 1.136960081 | 0.645747168 | 1.182944406 | 0.272051014 | 0.863795743 | 0.552443007 | 0.803241548 | 0.646507603 | 0.166550064 |
| ENSMUSG00000014453 | Blk | 2462 | 0.017264491 | 0 | 0 | 0.018505704 | 0 | 0 | 1.268333391 | 0.387818871 | 0.987004236 | 0.344724882 | 1.494180261 | 0.204037759 | 1.082016057 | 1.452556754 | 0.267759908 | 0.155526431 | 0 | 0.089373041 |
| ENSMUSG00000014470 | Rnf166 | 2192 | 25.5380095 | 22.58346752 | 22.2006257 | 20.05766778 | 25.95434935 | 21.65671477 | 22.05910487 | 21.72715593 | 25.15909882 | 24.60784878 | 27.2875219 | 25.64159317 | 27.05153493 | 22.88303576 | 23.42022748 | 25.69593225 | 26.41926762 | 25.29615857 |
| ENSMUSG00000014496 | Ankrd28 | 5201 | 10.70597589 | 11.45230818 | 11.30978906 | 12.14143577 | 11.98439553 | 14.58666316 | 7.95973737 | 13.02697979 | 12.01306372 | 10.53434285 | 9.5347831 | 10.58147343 | 8.754731714 | 9.501347622 | 11.61343606 | 12.18437901 | 12.92526913 | 11.22181546 |
| ENSMUSG00000014498 | Ankrd52 | 8502 | 1.204863258 | 1.387057699 | 1.48500252 | 1.114643287 | 1.539859238 | 1.596971183 | 0.367282617 | 1.239838046 | 0.823536542 | 1.208992391 | 1.854356187 | 1.378650805 | 1.746519301 | 1.059768637 | 0.969220322 | 0.725098541 | 1.149518432 | 0.860528251 |
| ENSMUSG00000014503 | Pkd2l2 | 2699 | 2.063052264 | 2.283395862 | 1.595125677 | 2.110089123 | 2.086489466 | 2.148080707 | 1.47249538 | 2.009381675 | 2.319507372 | 1.99154508 | 1.982510318 | 1.840531297 | 2.211619856 | 2.305856877 | 2.564602219 | 2.539465997 | 2.087427064 | 1.487834306 |
| ENSMUSG00000014504 | Srp19 | 2394 | 35.79383244 | 29.09660059 | 31.72366026 | 30.86884458 | 29.07216442 | 30.36502095 | 35.6524949 | 32.449184 | 31.29418219 | 34.05328569 | 31.0517274 | 29.39997933 | 36.45286619 | 42.1760426 | 29.36084142 | 35.58755283 | 32.88313026 | 27.80326614 |
| ENSMUSG00000014529 | 4930511M11R | 2303 | 0 | 0 | 0 | 0 | 0 | 0 | 0 | 0 | 0 | 0 | 0 | 0 | 0 | 0 | 0 | 0 | 0 | 0 |
| ENSMUSG00000014542 | Clec4f | 2549 | 0 | 0 | 0 | 0 | 0 | 0 | 0 | 0 | 0 | 0 | 0 | 0 | 0 | 0 | 0 | 0 | 0 | 0 |
| ENSMUSG00000014543 | Klra17 | 1521 | 0 | 0 | 0 | 0 | 0 | 0 | 0 | 0 | 0 | 0 | 0 | 0 | 0 | 0 | 0 | 0 | 0 | 0 |
| ENSMUSG00000014547 | Wdfy2 | 1999 | 2.168848402 | 2.699694279 | 2.274693273 | 2.507110994 | 2.02255207 | 2.429298595 | 1.349085892 | 2.560171047 | 1.916131048 | 3.066328876 | 3.035227477 | 2.848022803 | 2.196368503 | 1.417253292 | 3.689383944 | 1.896333233 | 3.18267398 | 1.651098751 |
| ENSMUSG00000014550 | Zfyve20 | 6360 | 4.751757886 | 5.997368535 | 5.004682857 | 5.279636741 | 5.963515071 | 6.451204938 | 4.054309751 | 6.203262843 | 4.500727744 | 5.211783709 | 6.039475883 | 4.844378262 | 4.242855149 | 5.681367923 | 4.994716917 | 4.635812531 | 5.333140371 | 4.393809153 |
| ENSMUSG00000014551 | Mrps25 | 6942 | 5.816755579 | 4.021349086 | 4.515420292 | 4.561354914 | 4.111528484 | 4.382797237 | 4.886659578 | 4.962481557 | 4.918412787 | 5.08048488 | 4.810529527 | 4.687486501 | 6.317498482 | 6.094866553 | 4.534426491 | 5.565431125 | 4.781125485 | 4.849549599 |
| ENSMUSG00000014554 | Dguok | 1724 | 44.40360906 | 34.53024476 | 38.83350697 | 40.24911283 | 35.51264902 | 32.45071706 | 40.45179161 | 37.86010192 | 39.70533016 | 39.24667005 | 42.62058361 | 41.2789848 | 43.58034223 | 43.15744083 | 46.24420571 | 45.08696811 | 41.26079295 | 40.71439686 |
| ENSMUSG00000014592 | Camta1 | 17895 | 2.864556708 | 2.645298837 | 2.286896154 | 2.518014688 | 2.504097286 | 3.040452722 | 2.892432006 | 3.145884389 | 2.623784743 | 3.283027452 | 3.385227569 | 2.969349583 | 3.228146137 | 2.815972887 | 2.454364827 | 3.500610992 | 3.174047053 | 3.156990925 |
| ENSMUSG00000014599 | Csf1 | 5381 | 0.868903431 | 0.730518773 | 0.278680439 | 0.474153221 | 0.447239581 | 0.46044168 | 0.026377632 | 0.461346619 | 0.160735334 | 0.674708075 | 0.461679584 | 0.487518331 | 0.705920392 | 0.699121182 | 0.321588059 | 0.718704952 | 0.754987451 | 0.163565454 |

| ENSMUSG00000014601 | Fam40a | 3213 | 82.02056999 | 93.28232471 | 91.98950217 | 84.4999263 | 92.56369634 | 94.55580752 | 80.90129413 | 75.31498962 | 70.96437948 | 76.53000856 | 76.50238449 | 76.88768211 | 62.3059198 | 60.95696126 | 58.64136791 | 55.47549874 | 59.84532573 | 53.51959776 |
| --- | --- | --- | --- | --- | --- | --- | --- | --- | --- | --- | --- | --- | --- | --- | --- | --- | --- | --- | --- | --- |
| ENSMUSG00000014602 | Kif1a | 9009 | 10.80911958 | 10.4091239 | 10.73356501 | 8.921043646 | 8.943594208 | 11.4902498 | 4.794813615 | 4.133376884 | 5.705491361 | 6.584036486 | 5.801510109 | 5.935331863 | 5.4539449 | 5.294491925 | 5.556653392 | 6.847171553 | 6.304758798 | 4.76879924 |
| ENSMUSG00000014603 | Alx3 | 1863 | 0 | 0 | 0 | 0 | 0 | 0 | 0 | 0 | 0 | 0 | 0 | 0 | 0 | 0 | 0 | 0 | 0 | 0 |
| ENSMUSG00000014606 | Slc25a11 | 2172 | 126.3022127 | 122.1779537 | 116.724527 | 125.0411534 | 118.158111 | 115.6458593 | 159.0594758 | 136.8736926 | 130.6135914 | 133.3114497 | 147.9879929 | 133.782845 | 136.730268 | 120.0447084 | 125.7482109 | 140.0639391 | 128.6542499 | 136.7629725 |
| ENSMUSG00000014609 | Chrne | 1920 | 0 | 0 | 0 | 0 | 0 | 0 | 0 | 0 | 0 | 0 | 0 | 0 | 0 | 0 | 0 | 0 | 0 | 0 |
| ENSMUSG00000014633 | 2310061C15R | 2618 | 4.497300901 | 2.430390678 | 3.104183766 | 4.002689151 | 3.621844524 | 3.179854561 | 6.397512735 | 4.3765167 | 4.955596816 | 4.736682566 | 4.03295104 | 4.349272409 | 4.786213517 | 4.275410642 | 5.240686826 | 4.753417649 | 3.864836662 | 4.39148331 |
| ENSMUSG00000014668 | Chfr | 3386 | 8.285119958 | 7.319785381 | 9.557558124 | 9.163340577 | 11.15875962 | 10.37592679 | 9.292064822 | 9.316870758 | 7.88212115 | 6.628391834 | 10.1306616 | 9.560863644 | 13.30184302 | 15.55451716 | 9.661557997 | 11.64776303 | 11.6699386 | 10.52743684 |
| ENSMUSG00000014686 | Ceacam16 | 1629 | 0.052185606 | 0.061349713 | 0 | 0 | 0 | 0 | 0 | 0 | 0.126416562 | 0 | 0.234622264 | 0 | 0 | 0 | 0 | 0 | 0 | 0 |
| ENSMUSG00000014704 | Hoxa2 | 2421 | 0.684717829 | 0.316479375 | 0.059942417 | 0.28228652 | 0.457263215 | 0.347953933 | 0.039085237 | 0.141979191 | 0.051036575 | 0.428465705 | 0.019733564 | 0.13832878 | 0.244520036 | 0.460412267 | 0.289312867 | 0.189792354 | 0.237461567 | 0.227216467 |
| ENSMUSG00000014725 | Adam28 | 3656 | 6.522265796 | 15.33523002 | 3.426899443 | 6.978715763 | 3.699417547 | 13.49959591 | 0 | 0.0104465 | 0 | 0.025793601 | 0.013067549 | 0.045800598 | 0 | 0.012703525 | 0.011269572 | 0.010473361 | 0.031449368 | 0 |
| ENSMUSG00000014747 | Ankrd53 | 1897 | 0.11203262 | 0.298534107 | 0.663000416 | 0.456330966 | 0.202981228 | 0.287337939 | 0.773164495 | 0.161064428 | 0.73818742 | 0.74566215 | 0.453320639 | 0.794424296 | 1.066214343 | 0.856901981 | 0.933930893 | 0.98905628 | 0.747534521 | 0.898936378 |
| ENSMUSG00000014748 | Tex261 | 4137 | 41.14895576 | 43.20127568 | 40.28204304 | 39.73513366 | 38.26586825 | 43.32420531 | 46.11184951 | 47.00887432 | 43.80485119 | 50.72946101 | 41.67750185 | 39.08580481 | 50.96558241 | 59.76995921 | 54.36772869 | 49.98974391 | 50.13824482 | 47.85539645 |
| ENSMUSG00000014763 | Fam120b | 4507 | 23.53958717 | 24.46542708 | 26.60705164 | 29.73042631 | 24.56255257 | 23.12167711 | 16.11381455 | 16.49044044 | 18.04822912 | 19.52145824 | 16.96026926 | 16.80538144 | 13.97755899 | 20.19756186 | 19.98371448 | 20.52587253 | 22.43283142 | 19.45518441 |
| ENSMUSG00000014767 | Tbp | 13512 | 3.941606392 | 3.335727196 | 3.673123308 | 3.830472644 | 3.832885573 | 3.377595295 | 4.033763035 | 3.581244364 | 3.697398136 | 3.691936356 | 4.363106776 | 4.324970303 | 3.238411088 | 3.585049078 | 3.909147029 | 4.055199763 | 4.572379411 | 3.098129827 |
| ENSMUSG00000014769 | Psmb1 | 1030 | 354.4849146 | 312.0738993 | 338.7086417 | 305.8340394 | 346.9236906 | 337.9207773 | 320.7156546 | 297.1220587 | 332.8110395 | 327.6744989 | 320.7415902 | 307.7989937 | 381.0531397 | 340.8004005 | 357.9735181 | 362.6083346 | 348.8809318 | 379.776464 |
| ENSMUSG00000014771 | Pdcd2 | 2574 | 0 | 0 | 0 | 0 | 0 | 0 | 0 | 0 | 0 | 0 | 0 | 0 | 0 | 0 | 0 | 0 | 0 | 0 |
| ENSMUSG00000014773 | Dll1 | 4576 | 4.857999795 | 4.564507141 | 5.031861986 | 3.594304415 | 4.722734643 | 4.504793962 | 0.827144737 | 2.136637897 | 2.340142941 | 2.297772471 | 2.286432672 | 1.793029371 | 1.832697976 | 3.156492827 | 2.467051205 | 2.669297151 | 2.705287128 | 2.368180512 |
| ENSMUSG00000014776 | Nol3 | 2865 | 0.05934405 | 0.220923208 | 0.101305823 | 0.0954158 | 0.067199893 | 0.242142503 | 0.280738409 | 0.039992044 | 0.10063023 | 0.148117392 | 0.416884454 | 0.097409533 | 0.017218819 | 0 | 0 | 0.066824795 | 0 | 0.057601159 |
| ENSMUSG00000014778 | Fhod1 | 4546 | 1.589502843 | 0.732795737 | 0.744862984 | 0.330733494 | 0.603501903 | 0.839322588 | 0.353856376 | 0.781322794 | 0.380517744 | 0.477107864 | 1.050922976 | 0.282393365 | 0.629399736 | 0.480174243 | 0.806629669 | 1.11182582 | 0.598584927 | 0.847038599 |
| ENSMUSG00000014782 | Plekhg4 | 4816 | 0.070606605 | 0.269768039 | 0.050221688 | 0.104063847 | 0.069959191 | 0.010289189 | 0.186657164 | 0.174466955 | 0.102624209 | 0.048952142 | 0.40672203 | 0.301330404 | 0.491680239 | 0.51111641 | 0.419201877 | 0.286225471 | 0 | 0.571107836 |
| ENSMUSG00000014786 | Slc9a5 | 5206 | 0.530702351 | 0.307149236 | 0.399550865 | 0.621366529 | 0.406800738 | 0.285551673 | 0.172674011 | 0.044017367 | 0.071202198 | 0.172082859 | 0.211068775 | 0.139378335 | 0.018951947 | 0.401456768 | 0.007914244 | 0.183877293 | 0.161962837 | 0.063398894 |
| ENSMUSG00000014791 | Elmo3 | 3165 | 24.3615764 | 24.92414961 | 26.57869488 | 27.55255572 | 25.47266102 | 22.74885368 | 16.9966875 | 25.44953449 | 28.18641173 | 22.09304674 | 27.78947822 | 25.48298023 | 29.50563014 | 25.47454525 | 27.42865032 | 24.11158943 | 27.98485692 | 21.82983824 |
| ENSMUSG00000014813 | Stc1 | 4193 | 0.212880681 | 0.158897659 | 0.461469405 | 0.162989665 | 0.309936069 | 0.401810861 | 2.708095145 | 2.82366915 | 2.661947477 | 1.147000175 | 1.150791988 | 2.276288648 | 4.08256029 | 4.796157844 | 2.21091106 | 0.776222664 | 1.014600276 | 1.784220967 |
| ENSMUSG00000014837 | 4931428F04R | 3544 | 1.918969572 | 2.744741849 | 2.252153642 | 2.044076199 | 2.90638591 | 2.894304698 | 2.443064403 | 3.362310821 | 2.777533089 | 2.700788025 | 2.453454414 | 2.850630623 | 2.296773801 | 2.175428473 | 2.406524286 | 2.452586874 | 2.703604466 | 2.266176519 |
| ENSMUSG00000014846 | Tppp3 | 2198 | 6.88436882 | 10.24545791 | 6.184241142 | 5.783226283 | 5.080348631 | 5.207771363 | 8.545556658 | 5.560313362 | 4.928140876 | 6.263878686 | 9.933192004 | 8.633905951 | 4.264360456 | 3.46534592 | 1.743286942 | 4.546791871 | 3.068893656 | 4.429759732 |
| ENSMUSG00000014850 | Msh3 | 7430 | 1.853523154 | 2.134177342 | 2.916735074 | 2.115553203 | 1.768508097 | 2.814435205 | 1.84665907 | 1.840226119 | 2.17850615 | 1.93552685 | 1.99330244 | 2.328782692 | 1.500540149 | 2.737888328 | 1.591500207 | 1.360527638 | 1.098721567 | 1.710242753 |
| ENSMUSG00000014852 | Adamts13 | 5210 | 0.277385026 | 0.204608947 | 0.250688161 | 0.332307041 | 0.351058176 | 0.21875487 | 0.018162257 | 0.564455851 | 0.339927097 | 0.660652848 | 0.15588758 | 0.503519413 | 0.274592246 | 0.115887358 | 0.260969548 | 0.102892227 | 0.066206655 | 0.285075985 |
| ENSMUSG00000014856 | Tmem208 | 1607 | 49.75248047 | 45.58497473 | 45.75467712 | 42.32896029 | 44.29814257 | 44.557374 | 37.95024464 | 45.39358344 | 45.77418616 | 43.98189433 | 47.89387561 | 42.51298756 | 54.48920545 | 57.68662006 | 47.17539691 | 47.84538871 | 42.47612619 | 40.32403716 |
| ENSMUSG00000014859 | E2f4 | 1982 | 43.34155437 | 41.6159062 | 36.19472529 | 37.1476527 | 35.55254099 | 42.85236395 | 41.10617211 | 36.13055222 | 33.76795566 | 38.89576177 | 37.24132737 | 37.28553848 | 31.78448959 | 36.50852076 | 32.53301462 | 31.37434229 | 34.74891803 | 35.80310175 |
| ENSMUSG00000014867 | Surf4 | 3337 | 119.7711028 | 126.5530596 | 119.0130909 | 125.6922308 | 128.125825 | 124.082302 | 118.4305685 | 119.3727171 | 121.9056686 | 118.4066207 | 132.9309228 | 123.1390195 | 138.3422481 | 147.6412583 | 140.5692879 | 138.8995513 | 134.1362965 | 138.6189926 |
| ENSMUSG00000014873 | Surf2 | 2473 | 13.50953027 | 13.92864239 | 12.38190243 | 12.95164342 | 12.59254129 | 10.49964917 | 13.88960975 | 14.51712829 | 14.33950266 | 14.18524999 | 13.96736554 | 13.99341318 | 15.26037878 | 16.50802783 | 17.04374916 | 16.16473681 | 17.6211078 | 12.1451566 |
| ENSMUSG00000014905 | Dnajb9 | 1862 | 31.57070808 | 35.10198628 | 37.33231102 | 37.437378 | 33.60445798 | 36.64560373 | 39.3848831 | 41.76140242 | 36.01055191 | 41.02263119 | 38.40983504 | 40.01821136 | 40.37692907 | 46.81823417 | 43.21516577 | 49.148524 | 49.25608404 | 48.18466867 |
| ENSMUSG00000014907 | Naf1 | 3338 | 1.146035303 | 0.948090159 | 1.14484988 | 0.996391922 | 1.110293026 | 0.905547258 | 1.233134532 | 1.304354067 | 2.097575698 | 1.257163748 | 1.373995211 | 1.320981829 | 2.054264979 | 1.711390877 | 1.13557314 | 1.365063595 | 1.733753994 | 1.021738951 |
| ENSMUSG00000014932 | Yes1 | 4564 | 8.111745907 | 8.97783959 | 8.330761363 | 9.283911313 | 8.80590628 | 9.521855247 | 8.220629796 | 7.782413286 | 8.022526843 | 9.773129992 | 7.620545524 | 10.32173953 | 7.317639789 | 6.370288936 | 6.319257038 | 6.770490835 | 8.237970478 | 6.20720639 |
| ENSMUSG00000014956 | Ppp1cb | 7249 | 112.7275499 | 115.0671208 | 120.4633701 | 129.4929227 | 120.2733709 | 117.8834171 | 94.30554529 | 98.96621421 | 98.02606655 | 107.3623269 | 102.4502317 | 96.34727355 | 83.3041798 | 77.38332011 | 92.88396005 | 103.1981786 | 114.5823571 | 89.31675902 |
| ENSMUSG00000014959 | Gorasp2 | 4301 | 54.87822425 | 59.22894724 | 57.95601068 | 58.63296452 | 54.94716234 | 60.86656394 | 57.52104118 | 58.00343467 | 60.52052537 | 57.94899266 | 55.06172266 | 57.64547157 | 54.99803379 | 52.55600403 | 56.77787067 | 62.71967662 | 52.2453101 | 61.92841078 |
| ENSMUSG00000014980 | Tsen15 | 1891 | 10.11492819 | 8.825891065 | 9.41378063 | 8.601424023 | 10.43579519 | 7.258650027 | 14.41147703 | 14.01667227 | 14.35320671 | 10.02357626 | 8.867800331 | 11.1867457 | 11.13946519 | 13.5574491 | 8.606353557 | 10.65090402 | 11.77555711 | 11.57780247 |
| ENSMUSG00000015001 | Oc90 | 2428 | 0 | 0 | 0 | 0 | 0 | 0 | 0 | 0 | 0 | 0 | 0.019676672 | 0 | 0 | 0 | 0 | 0 | 0.063140522 | 0 |
| ENSMUSG00000015002 | Efr3a | 11134 | 14.00678021 | 16.49787127 | 15.07599698 | 15.49664765 | 14.73267432 | 17.74000324 | 12.86714494 | 12.55471464 | 11.19367673 | 14.01731882 | 13.01432091 | 11.3195407 | 10.31920562 | 11.3211113 | 12.93330679 | 12.82085364 | 13.3078376 | 9.940571468 |
| ENSMUSG00000015013 | Trappc2l | 602 | 98.63742676 | 87.93054637 | 88.87229995 | 77.57486785 | 91.70650573 | 75.06992197 | 106.0212689 | 89.45396577 | 86.82008057 | 92.49996769 | 99.51793676 | 92.71704749 | 107.759919 | 100.8345884 | 100.1293626 | 89.55676947 | 85.24736138 | 104.3528237 |
| ENSMUSG00000015016 | Acsf3 | 2196 | 7.103551731 | 7.053959841 | 6.520293709 | 7.448276374 | 7.057595365 | 7.649534009 | 6.312666178 | 7.148031598 | 4.726321481 | 7.600796427 | 7.96249308 | 9.277193463 | 3.414595367 | 6.767808727 | 7.504837139 | 7.91618204 | 6.858941112 | 7.214309078 |

| ENSMUSG00000015023 | Ddx19a | 2946 | 6.781205951 | 5.439070645 | 6.075426419 | 7.098614851 | 6.110434301 | 5.971222139 | 5.637050341 | 7.039536492 | 6.347141261 | 6.41800136 | 6.470539183 | 6.403833657 | 6.195794091 | 4.28811669 | 7.272508168 | 6.134815578 | 7.168292462 | 6.180588705 |
| --- | --- | --- | --- | --- | --- | --- | --- | --- | --- | --- | --- | --- | --- | --- | --- | --- | --- | --- | --- | --- |
| ENSMUSG00000015027 | Galns | 3296 | 25.10848839 | 26.60180145 | 25.88923166 | 23.40256313 | 27.0888102 | 25.84379521 | 13.96700139 | 18.91080119 | 14.88262751 | 16.26527597 | 18.59686642 | 16.17231728 | 11.12063548 | 13.75286083 | 18.11318401 | 14.98631176 | 16.02355496 | 11.56593172 |
| ENSMUSG00000015053 | Gata2 | 3344 | 0.012710878 | 0 | 0 | 0.013624714 | 0 | 0.044455204 | 0.113188227 | 0.342635189 | 0.012316542 | 0 | 0 | 0.15022158 | 9.028448975 | 10.49991928 | 5.137873447 | 9.607003472 | 7.266409162 | 6.251034855 |
| ENSMUSG00000015083 | C8g | 1580 | 2.636396994 | 4.153569722 | 4.837353036 | 4.037054561 | 3.686052382 | 4.610285972 | 5.419996763 | 8.170273426 | 6.77752791 | 8.14692531 | 6.833633301 | 4.274490616 | 6.306991956 | 7.583907941 | 7.431926221 | 3.97446811 | 6.525173333 | 6.893546286 |
| ENSMUSG00000015085 | Entpd2 | 2295 | 1.240891849 | 1.204780052 | 0.927422804 | 1.846264542 | 1.363213524 | 1.446637538 | 1.752321442 | 1.381250283 | 1.381856956 | 1.253243093 | 1.602907104 | 3.064389318 | 1.182246384 | 0.465452728 | 2.369762692 | 0.483846455 | 1.102194159 | 1.893559665 |
| ENSMUSG00000015087 | B230208H17R | 4171 | 13.52298456 | 13.81714385 | 13.82432227 | 13.27179786 | 12.00124685 | 12.12978686 | 11.86503528 | 10.92388781 | 14.16989933 | 13.10181373 | 10.81264944 | 12.13733061 | 12.57248335 | 13.13930068 | 12.76250544 | 12.52179058 | 12.74480312 | 13.21484654 |
| ENSMUSG00000015090 | Ptgds | 1306 | 110.2661075 | 106.1880388 | 108.7108052 | 105.1811246 | 106.9516405 | 102.482338 | 541.8497234 | 374.7592931 | 338.2592405 | 314.7832938 | 209.0977509 | 276.5995163 | 2498.400974 | 3042.372221 | 1530.483217 | 389.5028479 | 572.018514 | 1706.040474 |
| ENSMUSG00000015092 | Edf1 | 1470 | 141.308024 | 124.1415201 | 130.8388821 | 120.6282888 | 130.6437928 | 126.2751973 | 135.4043811 | 123.5667115 | 140.2863159 | 122.1427742 | 120.6073952 | 126.7812907 | 153.4658896 | 156.9620586 | 137.0582369 | 158.31994 | 132.5516282 | 147.2148475 |
| ENSMUSG00000015093 | Clic3 | 1071 | 28.97178198 | 25.6922975 | 31.25535295 | 26.97077695 | 26.91972201 | 34.00677795 | 42.14406694 | 34.55503075 | 29.95732566 | 31.83002657 | 31.80536451 | 29.13254471 | 82.26592367 | 89.33223158 | 55.43552011 | 51.41166521 | 45.19711764 | 63.74071096 |
| ENSMUSG00000015094 | Npdc1 | 3441 | 21.51816816 | 23.51555364 | 23.16756118 | 22.72093929 | 23.51344484 | 23.55950948 | 26.88064147 | 28.16981032 | 26.26074269 | 27.63817741 | 25.67157751 | 28.9702916 | 30.19268172 | 23.18830053 | 27.13243989 | 30.5011784 | 29.85018184 | 27.88023311 |
| ENSMUSG00000015095 | Fbxw5 | 3256 | 63.9796891 | 74.49360651 | 76.60132755 | 77.81479347 | 82.76739577 | 71.45272855 | 72.68366708 | 72.54914278 | 63.07001462 | 73.66573325 | 82.28561645 | 75.22086223 | 68.42227838 | 75.38605708 | 73.96286676 | 64.2214395 | 80.21919945 | 73.33984407 |
| ENSMUSG00000015112 | Slc25a13 | 3117 | 45.13703325 | 50.50905938 | 46.20083409 | 49.62455743 | 51.39013217 | 44.83115454 | 36.73297498 | 37.33469687 | 37.01104609 | 39.40570484 | 40.73976248 | 34.5960411 | 32.46062314 | 29.41309865 | 34.84353588 | 30.50227083 | 37.09670765 | 30.4076647 |
| ENSMUSG00000015120 | Ube2i | 4847 | 29.15818244 | 30.5087554 | 26.25763531 | 29.6095089 | 28.53953964 | 24.7099148 | 29.01037379 | 30.80922084 | 26.73257243 | 30.05893812 | 30.3287698 | 25.18441388 | 32.08050408 | 24.29043976 | 27.59237681 | 33.76398928 | 29.84975672 | 28.3273634 |
| ENSMUSG00000015126 | 0610007P22R | 1373 | 17.95557327 | 17.97877248 | 17.29890998 | 20.04287748 | 15.3895954 | 17.50406102 | 18.02223677 | 17.16293684 | 16.13863473 | 21.05126081 | 16.91087387 | 14.35027596 | 22.85149263 | 17.79285504 | 18.30513408 | 21.11142739 | 19.20501986 | 17.30803649 |
| ENSMUSG00000015127 | Unkl | 5654 | 0.939714716 | 1.579033543 | 1.15501001 | 1.450475412 | 1.140728294 | 0.929004202 | 1.238464181 | 1.202378427 | 0.655604248 | 1.626173883 | 1.157617494 | 1.135267279 | 1.012115742 | 1.289657184 | 1.289825857 | 0.799131884 | 0.982899372 | 1.605324126 |
| ENSMUSG00000015133 | Lrrk1 | 9063 | 1.575829101 | 1.966500983 | 1.414430712 | 2.307461034 | 2.097772244 | 1.645743441 | 1.012761747 | 0.644757538 | 0.80437088 | 0.364178443 | 0.790714308 | 0.689766546 | 0.244944972 | 0.281852014 | 0.177298983 | 0.139422933 | 0.359454401 | 0.418804851 |
| ENSMUSG00000015134 | Aldh1a3 | 4600 | 0.388090738 | 0.724193353 | 0.262899621 | 0 | 0.104634617 | 0.193902001 | 0 | 0 | 0 | 0 | 0 | 0.01213384 | 0.075070309 | 0.201930815 | 0 | 0 | 0.041659018 | 0.358755044 |
| ENSMUSG00000015143 | Actn1 | 3941 | 10.93637363 | 12.15527579 | 12.37262588 | 10.73996706 | 10.38115583 | 10.39840413 | 7.695363408 | 8.867304796 | 9.395249345 | 8.362938741 | 8.643376147 | 9.687366965 | 7.260216194 | 8.779712029 | 8.426402956 | 7.588166552 | 9.083167041 | 7.858443818 |
| ENSMUSG00000015149 | Sirt2 | 3662 | 27.84541706 | 27.87294044 | 25.85117392 | 28.29213949 | 25.84035006 | 24.97934085 | 28.97933884 | 27.69001321 | 26.62165014 | 30.25782432 | 26.44452235 | 26.41412978 | 27.00996563 | 28.09220469 | 26.23758283 | 27.67756357 | 24.16587099 | 25.65689631 |
| ENSMUSG00000015165 | Hnrnpl | 5874 | 38.59041603 | 38.32627496 | 40.09716025 | 38.69663776 | 39.65106888 | 36.19863462 | 33.6601439 | 35.96873853 | 35.78052263 | 36.04933748 | 36.40461596 | 37.69505167 | 32.74517287 | 31.54782238 | 34.69235537 | 33.62976568 | 33.56976465 | 37.5811279 |
| ENSMUSG00000015176 | Nolc1 | 2690 | 22.18485767 | 23.33140993 | 24.47448878 | 21.51023283 | 22.41981426 | 21.38688613 | 20.87737914 | 22.8870456 | 19.98081898 | 20.82343326 | 20.5308 | 20.29283197 | 19.45768187 | 22.68681443 | 21.81078646 | 20.93884145 | 19.83279007 | 21.7582489 |
| ENSMUSG00000015189 | Casd1 | 4198 | 13.58788619 | 13.99808133 | 15.42928946 | 15.67178371 | 12.93301808 | 15.84082145 | 11.64220994 | 12.69137717 | 13.87273304 | 15.26388893 | 12.26808129 | 12.07256351 | 8.731208799 | 8.275411474 | 9.78512416 | 9.714029748 | 10.69995712 | 14.04710872 |
| ENSMUSG00000015202 | Cnksr3 | 3824 | 2.94557313 | 2.961920511 | 3.478744992 | 4.503680638 | 2.391492023 | 3.680171636 | 3.897357182 | 2.526850891 | 1.981777955 | 2.404389941 | 2.5236772 | 3.736613395 | 2.193103017 | 2.076866879 | 2.057922901 | 1.722276274 | 1.984468302 | 4.373108903 |
| ENSMUSG00000015214 | Mtmr1 | 5972 | 4.512438312 | 4.880907978 | 3.928526829 | 5.187794733 | 4.626209676 | 5.318704324 | 6.528072243 | 6.420825856 | 5.275901634 | 4.373993571 | 3.551922566 | 6.009623416 | 4.055922871 | 4.051803337 | 4.077381033 | 6.38604236 | 7.515086014 | 4.439783898 |
| ENSMUSG00000015217 | Hmgb3 | 2308 | 20.7369273 | 22.38661132 | 20.03686959 | 19.01008912 | 20.93780388 | 20.48236908 | 21.44240128 | 23.23315987 | 18.98719791 | 19.65293611 | 19.33354039 | 21.35408582 | 20.32697285 | 21.37071959 | 18.47643429 | 16.72310757 | 17.40292858 | 18.54293112 |
| ENSMUSG00000015222 | Mtap2 | 9478 | 0.134538434 | 0.246033192 | 0.117386706 | 0.216316416 | 0.34024466 | 0.292778337 | 0.134779735 | 0.257893411 | 0.073873261 | 0.074621286 | 0.070568624 | 0.005888971 | 0 | 0 | 0.017388291 | 0.008079892 | 0.161748457 | 0.029019364 |
| ENSMUSG00000015224 | Cyp2j9 | 3625 | 1.102203185 | 2.021748063 | 1.868219103 | 1.74703039 | 1.978388035 | 1.913760745 | 2.297112136 | 2.475921259 | 1.329330302 | 2.055120307 | 1.818743247 | 2.709946652 | 2.204626372 | 1.268404042 | 1.829917379 | 1.478809665 | 1.205295958 | 1.471967821 |
| ENSMUSG00000015242 | Nipsnap3a | 1594 | 0 | 0 | 0 | 0 | 0 | 0 | 0 | 0.023960102 | 0 | 0 | 0 | 0 | 0 | 0 | 0 | 0 | 0 | 0 |
| ENSMUSG00000015243 | Abca1 | 10313 | 9.656707789 | 14.45830646 | 12.15318696 | 11.11525139 | 9.34355776 | 14.31373929 | 5.220772677 | 7.025209673 | 6.609491282 | 6.025853476 | 5.234723464 | 7.00334213 | 3.750239808 | 5.142940735 | 5.025846728 | 5.146008131 | 5.70453464 | 6.027372307 |
| ENSMUSG00000015247 | Nipsnap3b | 1778 | 88.452841 | 89.50264099 | 81.13040918 | 80.30838748 | 89.577354 | 87.87388581 | 79.53745631 | 80.46610765 | 80.03341505 | 81.04192866 | 77.78881035 | 76.25210597 | 91.53318059 | 74.6814656 | 73.29613121 | 80.67301199 | 84.84381587 | 71.31383071 |
| ENSMUSG00000015289 | Lage3 | 2513 | 18.08119108 | 15.78816435 | 14.14824704 | 15.301839 | 17.0080176 | 15.38047441 | 19.71204729 | 16.915298 | 18.58555864 | 16.54871479 | 15.64615632 | 15.68080294 | 18.49210747 | 19.18382917 | 15.98548229 | 18.31488663 | 14.99194184 | 15.41043022 |
| ENSMUSG00000015290 | Ubl4 | 4482 | 24.24938311 | 24.95122398 | 25.26604966 | 25.57599008 | 25.36537343 | 26.68904481 | 27.74157078 | 28.93828616 | 30.70150584 | 28.37247806 | 30.81602074 | 30.53547627 | 25.50246594 | 25.98879321 | 27.96410822 | 25.80899709 | 27.3466081 | 26.22812607 |
| ENSMUSG00000015291 | Gdi1 | 4038 | 98.32611423 | 104.4678503 | 97.36999869 | 108.7011147 | 98.81467241 | 99.21591158 | 99.87451094 | 96.18091631 | 87.76868948 | 99.40414483 | 103.4532533 | 92.19674825 | 87.04554497 | 91.17441326 | 92.51473682 | 92.66364921 | 95.36964618 | 100.8364061 |
| ENSMUSG00000015305 | Sash1 | 7163 | 1.744593292 | 1.753310222 | 1.681559271 | 1.634676587 | 1.5253311 | 1.093024139 | 1.657892281 | 1.492932106 | 1.374225501 | 1.974760712 | 1.420643048 | 2.127275707 | 2.190080929 | 1.945166301 | 1.938423054 | 2.646077155 | 2.19909368 | 2.296205907 |
| ENSMUSG00000015312 | Gadd45b | 1262 | 45.83957573 | 51.63234635 | 43.08387331 | 43.61152263 | 59.00165286 | 43.78074322 | 66.84509234 | 78.04929144 | 63.05257405 | 72.78095875 | 73.93383036 | 82.21974386 | 89.47762175 | 118.5023466 | 108.4560766 | 113.5671502 | 134.9316459 | 168.4708379 |
| ENSMUSG00000015314 | Slamf6 | 3047 | 0 | 0.120263156 | 0 | 0 | 0 | 0 | 0.310552536 | 0.225619706 | 0.608268201 | 0 | 0.141114088 | 0 | 0.097141944 | 0.19815331 | 0.081132043 | 0.163366556 | 0 | 0.252749424 |
| ENSMUSG00000015316 | Slamf1 | 2957 | 0 | 0 | 0 | 0 | 0 | 0 | 0 | 0 | 0 | 0 | 0.242348454 | 0 | 0 | 0 | 0 | 0 | 0 | 0 |
| ENSMUSG00000015335 | Zdhhc12 | 2396 | 12.47125991 | 11.0811255 | 12.3760326 | 9.355606767 | 8.838917535 | 13.04998952 | 11.65045099 | 12.03475118 | 12.51409995 | 12.81097989 | 10.12924829 | 10.62268036 | 13.32126481 | 12.52207031 | 14.1178954 | 14.12725248 | 9.581574226 | 11.13498195 |
| ENSMUSG00000015337 | Endog | 1060 | 14.47581938 | 13.23087591 | 13.41680936 | 10.27272603 | 13.25898275 | 13.65037566 | 16.24699541 | 16.61009199 | 14.2598597 | 15.21277407 | 16.8113769 | 14.16454079 | 16.75423606 | 16.47450647 | 13.52654854 | 16.00258404 | 12.90800754 | 16.39894125 |
| ENSMUSG00000015340 | Cybb | 5793 | 0.007337334 | 0 | 0 | 0 | 0 | 0 | 0.555370649 | 0.448314062 | 0.369704612 | 0.048835529 | 1.014382857 | 0.077080149 | 0.545009961 | 0.224483764 | 0.348502717 | 0.337100117 | 0.006615967 | 0.246890519 |
| ENSMUSG00000015341 | Golga7 | 2743 | 32.06095851 | 32.26845058 | 29.75069111 | 29.58228945 | 27.58417208 | 26.86289276 | 36.22188325 | 34.39126283 | 35.2255071 | 31.3879636 | 27.36218001 | 31.43827878 | 26.74318665 | 27.86983881 | 32.09906122 | 34.88451619 | 33.57568044 | 33.8918667 |

| ENSMUSG00000015342 | Xk | 5073 | 2.052782994 | 2.66608221 | 1.420787704 | 2.559609232 | 1.593960807 | 2.090338061 | 0.96061619 | 2.123054894 | 1.428903366 | 1.301221852 | 2.589811469 | 1.782404364 | 1.332243774 | 2.902183265 | 2.931945925 | 1.577515661 | 2.024728478 | 1.496403849 |
| --- | --- | --- | --- | --- | --- | --- | --- | --- | --- | --- | --- | --- | --- | --- | --- | --- | --- | --- | --- | --- |
| ENSMUSG00000015354 | Pcolce2 | 4615 | 0 | 0 | 0 | 0 | 0 | 0 | 0 | 0 | 0 | 0 | 0 | 0 | 0 | 0 | 0 | 0 | 0 | 0 |
| ENSMUSG00000015355 | Cd48 | 1341 | 0.126786506 | 0.02484183 | 0.072145459 | 0 | 0 | 0 | 1.975771827 | 1.025299393 | 1.013538419 | 0.14064341 | 1.888197464 | 0.499469016 | 0.367874103 | 0.900481933 | 0.430143015 | 0.171322628 | 0 | 0 |
| ENSMUSG00000015357 | Clpx | 3750 | 22.64675779 | 21.62228921 | 21.87773533 | 22.98706557 | 24.74622635 | 24.2478043 | 25.28389562 | 24.34129113 | 23.51475473 | 25.4236592 | 24.44803875 | 22.11788656 | 17.61478326 | 20.46016863 | 25.77569337 | 19.75795342 | 23.78274477 | 25.42154185 |
| ENSMUSG00000015363 | Trabd | 3582 | 30.61511841 | 27.82584576 | 26.29348509 | 30.09420181 | 25.85310473 | 31.59643872 | 29.75864479 | 30.70745922 | 35.75937019 | 34.29022389 | 32.75692295 | 35.0445073 | 34.59569408 | 35.39708503 | 40.46540302 | 36.93301189 | 33.0834478 | 33.6934618 |
| ENSMUSG00000015365 | Mov10l1 | 4223 | 2.174074834 | 1.490915701 | 2.16495317 | 1.413330999 | 2.290910883 | 1.677963747 | 3.293849776 | 2.016790371 | 2.730813264 | 2.344695166 | 2.183416289 | 2.180815611 | 1.740576764 | 2.947434388 | 2.70254108 | 1.323805039 | 2.631926617 | 3.425857227 |
| ENSMUSG00000015377 | Fam116b | 4586 | 5.106923679 | 3.92984644 | 4.198138919 | 5.523755048 | 4.345097339 | 4.322087534 | 4.302090517 | 8.711132341 | 5.101164621 | 6.487591312 | 5.802584359 | 8.057123557 | 4.055414918 | 7.180300471 | 5.678016425 | 6.061705402 | 5.883496079 | 9.883886598 |
| ENSMUSG00000015396 | Cd83 | 2299 | 0.795007642 | 1.391056044 | 1.725371679 | 3.151024815 | 1.737690154 | 1.120809981 | 7.923175897 | 7.243100239 | 7.810926869 | 3.548095547 | 29.79960438 | 7.526252893 | 4.205765892 | 8.302966471 | 9.1758141 | 2.764785049 | 0.7501885 | 1.866337679 |
| ENSMUSG00000015401 | Tmem27 | 1536 | 0 | 0.043376164 | 0.346425022 | 0 | 0.062671776 | 0.032260894 | 0 | 0.248648453 | 0 | 0.030697072 | 0.186620932 | 0.145353288 | 8.414689015 | 8.678029349 | 5.096546627 | 0.498575617 | 0.474088308 | 6.732885458 |
| ENSMUSG00000015405 | Ace2 | 3743 | 0 | 0 | 0 | 0 | 0 | 0 | 0 | 0 | 0 | 0 | 0 | 0 | 1.107101537 | 0.54596309 | 0.979678994 | 0 | 0 | 0.558468089 |
| ENSMUSG00000015437 | Gzmb | 1413 | 0 | 0 | 0 | 0 | 0 | 0 | 0 | 0 | 0 | 0 | 0 | 0 | 0 | 0 | 0 | 0 | 0 | 0 |
| ENSMUSG00000015441 | Gzmf | 901 | 0 | 0 | 0 | 0 | 0 | 0 | 0.315067784 | 0 | 0 | 0.104663048 | 0 | 0 | 0 | 0 | 0 | 0 | 0 | 0 |
| ENSMUSG00000015443 | Gzmn | 1233 | 0 | 0 | 0 | 0 | 0 | 0 | 0 | 0 | 0 | 0 | 0 | 0 | 0 | 0 | 0 | 0 | 0 | 0 |
| ENSMUSG00000015451 | C4a | 6491 | 0.065483248 | 0.071850334 | 0.029809601 | 0.021057331 | 0.059321428 | 0.09924288 | 0.014577932 | 0.058839012 | 0.076142072 | 0.007264012 | 0.007360185 | 0 | 0.060800391 | 0.035775757 | 0.114254815 | 0.153374795 | 0 | 0.059322716 |
| ENSMUSG00000015452 | Ager | 1814 | 2.718081819 | 1.799704318 | 2.293342674 | 2.411168829 | 2.096153236 | 2.185346575 | 2.686442078 | 1.389580242 | 3.519244732 | 1.949450245 | 1.948923334 | 1.907701808 | 2.148413156 | 3.123582504 | 2.498440545 | 4.876036554 | 3.655154005 | 4.427414323 |
| ENSMUSG00000015461 | Atf6b | 2854 | 37.45638322 | 40.86490633 | 39.74629596 | 43.11856368 | 43.62904223 | 44.83011846 | 36.73612351 | 38.35298714 | 35.32746692 | 41.33533972 | 38.78576692 | 38.78152029 | 28.01928448 | 29.56864288 | 33.27595877 | 27.47693201 | 36.27166361 | 38.24038814 |
| ENSMUSG00000015467 | Egfl8 | 1820 | 0.6072168 | 0.402683337 | 0.451840668 | 0.250335408 | 0.105784448 | 0.108907107 | 0.181971842 | 0.35674222 | 0.135779722 | 0.233162818 | 0.419999635 | 0.61335893 | 0.704741675 | 0.663486963 | 0.294296828 | 1.009862173 | 0.505401717 | 0.241798271 |
| ENSMUSG00000015468 | Notch4 | 8237 | 0.361219172 | 0.392297043 | 0.317126459 | 0.265500805 | 0.327229298 | 0.258682475 | 0.292939981 | 0.333841565 | 0.255009385 | 0.366352435 | 0.475603569 | 0.379467902 | 0.113792209 | 0.293200503 | 0.370148736 | 0.209187487 | 0.307094281 | 0.440767396 |
| ENSMUSG00000015474 | Ppt2 | 3328 | 2.822609346 | 2.382352411 | 2.340185813 | 2.313646783 | 2.400811096 | 2.278115456 | 4.023283695 | 4.246150508 | 3.725102537 | 3.541969896 | 4.091305038 | 3.471707378 | 3.913349207 | 4.59137763 | 4.110251369 | 3.693294764 | 3.558541389 | 3.272777383 |
| ENSMUSG00000015476 | Prrt1 | 2302 | 0.35082465 | 0.4341385 | 0.357232848 | 0.138543575 | 0.167269935 | 0.15068164 | 0.061658574 | 0 | 0 | 0.163859959 | 0.083014698 | 0.169726168 | 0.128580149 | 0.403510751 | 0.572741003 | 0.582176915 | 0.316333467 | 0.143377342 |
| ENSMUSG00000015478 | Rnf5 | 1735 | 44.31807691 | 38.76584059 | 36.24529652 | 39.23239207 | 40.41971918 | 38.12847223 | 53.96645049 | 42.06667493 | 47.61968335 | 45.46577899 | 49.15175843 | 41.17812571 | 55.90002845 | 50.88772918 | 45.54731078 | 52.54751367 | 46.41126789 | 45.24380132 |
| ENSMUSG00000015484 | Fam163a | 3877 | 0 | 0 | 0 | 0 | 0 | 0 | 0.122034251 | 0.108361214 | 0 | 0 | 0 | 0 | 0.16541525 | 0.083855716 | 0 | 0.019752699 | 0 | 0.070942877 |
| ENSMUSG00000015488 | 5930434B04R | 4214 | 38.04687327 | 39.8268536 | 37.58304186 | 38.45767316 | 40.17085324 | 37.34681581 | 44.99981422 | 47.91723577 | 41.88045089 | 47.03881265 | 44.41914744 | 48.06716648 | 48.73487699 | 52.08703299 | 46.041321 | 47.84962947 | 52.8055244 | 51.02766924 |
| ENSMUSG00000015501 | Hivep2 | 9757 | 3.968659973 | 3.632768212 | 3.10855832 | 2.713023138 | 3.32981946 | 3.204650496 | 2.346964907 | 2.77528065 | 2.980186546 | 2.034482533 | 2.095693577 | 3.163478673 | 1.966804942 | 2.99884955 | 2.934824367 | 2.751021399 | 2.60039034 | 2.463767514 |
| ENSMUSG00000015519 | 2310057J18R | 2166 | 0 | 0 | 0 | 0 | 0 | 0 | 0 | 0 | 0 | 0 | 0 | 0 | 0 | 0 | 0 | 0 | 0 | 0 |
| ENSMUSG00000015522 | Arnt | 6715 | 9.13402368 | 10.5321332 | 10.07811898 | 9.87212502 | 9.389846613 | 9.821993804 | 7.243102597 | 8.690691121 | 6.127375912 | 7.773019882 | 7.95419264 | 7.713616512 | 6.964505968 | 8.175266015 | 6.356636173 | 8.091492465 | 8.561347037 | 6.26686026 |
| ENSMUSG00000015533 | Itga2 | 4184 | 2.834355667 | 2.133808712 | 1.745794236 | 2.068976679 | 2.611363927 | 2.060749437 | 0.237468035 | 0.209948675 | 0.423283981 | 0.38315581 | 0.102766402 | 0.173423426 | 0 | 0 | 0 | 0.338611968 | 0 | 0.078884952 |
| ENSMUSG00000015536 | Mocs2 | 2266 | 25.19172613 | 21.47843709 | 23.56768646 | 23.34350065 | 22.81274758 | 23.28934742 | 27.74869828 | 23.37725602 | 28.37252917 | 21.24486674 | 21.73697361 | 23.81895222 | 28.01861321 | 28.63300533 | 25.85552183 | 24.80609517 | 22.44439364 | 21.43558748 |
| ENSMUSG00000015542 | Nat9 | 2025 | 10.20124225 | 11.54847 | 9.531377092 | 9.967181551 | 9.792766692 | 10.39995643 | 11.89242152 | 11.69347629 | 11.71527559 | 11.59558036 | 16.20809702 | 11.7695249 | 17.36970717 | 17.15564314 | 14.34424538 | 14.42752269 | 13.32430273 | 16.10883965 |
| ENSMUSG00000015568 | Lpl | 7082 | 0 | 0 | 0 | 0 | 0 | 0.027987988 | 0 | 0 | 0.197732496 | 0 | 0 | 0.039406709 | 0 | 0.059022421 | 0 | 0 | 0 | 0.124279258 |
| ENSMUSG00000015575 | Atp6v0e | 3423 | 314.8090046 | 284.8773122 | 272.5904257 | 268.2151926 | 308.2805418 | 275.0951201 | 356.455354 | 310.2477187 | 320.491976 | 340.427251 | 329.1908182 | 292.6123474 | 379.5949077 | 344.5926051 | 326.23014 | 363.9900889 | 348.2508947 | 334.1365314 |
| ENSMUSG00000015579 | Nkx2-5 | 1523 | 0 | 0 | 0 | 0 | 0.094810093 | 0 | 0 | 0 | 0 | 0 | 0 | 0 | 0 | 0 | 0 | 0 | 0 | 0 |
| ENSMUSG00000015597 | Zfp318 | 9165 | 1.539740146 | 1.781049445 | 2.269570982 | 1.461532682 | 1.423213455 | 1.605800532 | 1.992656199 | 1.654379024 | 1.649258187 | 1.810916263 | 1.0946799 | 1.680864471 | 1.490991935 | 1.930736202 | 2.099413704 | 1.946909225 | 1.467815626 | 2.166752594 |
| ENSMUSG00000015599 | Ttbk1 | 6944 | 0 | 0 | 0 | 0 | 0 | 0 | 0.020440385 | 0 | 0 | 0.027160543 | 0 | 0 | 0.007104251 | 0 | 0 | 0.011028401 | 0 | 0 |
| ENSMUSG00000015605 | Srf | 4086 | 5.544605684 | 5.788584164 | 4.652667017 | 4.616317268 | 6.361047181 | 6.403290095 | 3.832720687 | 4.832469908 | 3.165092009 | 3.473412061 | 3.016627334 | 3.04622926 | 4.672406262 | 5.46735341 | 3.821681273 | 5.285340816 | 5.421585808 | 7.175686217 |
| ENSMUSG00000015619 | Gata3 | 4748 | 38.02011007 | 35.79662728 | 39.1532176 | 39.5252615 | 36.13949197 | 39.35622543 | 35.31510828 | 34.62091406 | 37.83815959 | 37.08102906 | 36.52550532 | 40.09840461 | 40.43804171 | 37.02419351 | 37.60900237 | 44.976141 | 41.0465922 | 44.47766654 |
| ENSMUSG00000015627 | Gata5 | 3428 | 0 | 0 | 0 | 0 | 0 | 0 | 0 | 0 | 0 | 0 | 0 | 0 | 0 | 0 | 0 | 0 | 0.022360733 | 0 |
| ENSMUSG00000015647 | Lama5 | 11476 | 17.09688851 | 16.21521672 | 14.98921871 | 15.78914895 | 14.7381997 | 13.89946405 | 12.97429423 | 13.11907026 | 15.07707849 | 14.21998815 | 13.39247485 | 13.38972805 | 18.50591703 | 13.94617682 | 14.78459456 | 19.23872798 | 15.56964066 | 14.2268237 |
| ENSMUSG00000015652 | Steap1 | 1719 | 0.296720252 | 0.271309202 | 0.112562025 | 0.21203511 | 0.111999822 | 0.086479465 | 0.412850601 | 0.244395827 | 0.335434102 | 0.576012082 | 0.194546079 | 0.714336578 | 0.344376386 | 0 | 0 | 0 | 0.022295693 | 0.032000644 |
| ENSMUSG00000015653 | Steap2 | 19419 | 1.188542694 | 1.229998721 | 0.78716904 | 1.053448113 | 0.812980636 | 0.803806122 | 1.103694503 | 1.400329086 | 1.221660903 | 1.602526605 | 1.217807531 | 1.586603108 | 0.52332123 | 0.346793999 | 0.398882152 | 0.120280501 | 0.378940677 | 0.101978879 |
| ENSMUSG00000015656 | Hspa8 | 3064 | 183.0330589 | 143.1019301 | 158.4769901 | 168.4896192 | 148.3544018 | 157.1326238 | 155.341237 | 133.7357328 | 133.3991457 | 157.4408763 | 130.897773 | 128.0262652 | 177.459658 | 165.7526422 | 169.1630461 | 167.02153 | 148.3142632 | 182.7472903 |

| ENSMUSG00000015659 | Serac1 | 4905 | 1.083210398 | 0.550121189 | 1.114415684 | 0.761672912 | 0.765400621 | 0.727379575 | 0.974226416 | 1.105671996 | 0.806096945 | 1.076631756 | 0.798684321 | 0.785174458 | 1.53879375 | 0.359811482 | 0.982789407 | 0.733805728 | 0.859509207 | 0.796258222 |
| --- | --- | --- | --- | --- | --- | --- | --- | --- | --- | --- | --- | --- | --- | --- | --- | --- | --- | --- | --- | --- |
| ENSMUSG00000015665 | Awat1 | 1383 | 0 | 0 | 0 | 0 | 0 | 0 | 0 | 0 | 0 | 0 | 0 | 0 | 0 | 0 | 0 | 0 | 0 | 0 |
| ENSMUSG00000015668 | Pdzd11 | 2365 | 32.24282697 | 27.94620809 | 31.00814884 | 23.21397819 | 28.26860126 | 27.09162136 | 31.00830966 | 30.87690207 | 32.20036687 | 31.63981652 | 30.92746784 | 25.41795713 | 32.72802461 | 32.87416587 | 31.2539498 | 32.72106451 | 29.38079336 | 30.93535389 |
| ENSMUSG00000015671 | Psma2 | 2660 | 111.2005714 | 99.66316248 | 100.2204984 | 96.15778305 | 110.9748451 | 98.86329214 | 100.5483361 | 98.61106004 | 103.5237009 | 98.87467021 | 100.2196497 | 89.05326016 | 114.2237889 | 97.63734462 | 115.9994181 | 103.9892285 | 97.7320571 | 102.0150088 |
| ENSMUSG00000015672 | Mrpl32 | 765 | 78.954059 | 63.14208708 | 76.25944785 | 64.26191744 | 73.23602503 | 66.91238403 | 78.42153841 | 76.13518127 | 86.73395672 | 86.04232908 | 69.88258632 | 67.56248831 | 86.02454594 | 82.68868892 | 84.07271731 | 84.08917704 | 77.45418956 | 76.94084208 |
| ENSMUSG00000015697 | Setdb1 | 6832 | 5.53089893 | 6.446084039 | 7.42029564 | 5.815168422 | 6.861898954 | 7.253034774 | 6.322669183 | 6.708267401 | 5.473851919 | 6.708208953 | 5.811034909 | 5.506404652 | 5.632156832 | 5.03733442 | 5.228593231 | 4.735884551 | 5.104937087 | 6.143435075 |
| ENSMUSG00000015702 | Anxa9 | 2553 | 0.566069716 | 0.991688195 | 0.79580426 | 1.267071738 | 0.754123363 | 0.465830633 | 0.389175972 | 0.433834575 | 0.435580073 | 0.295499903 | 0.449118294 | 0.218627742 | 4.289731937 | 7.986272763 | 5.180454148 | 2.93966277 | 3.497856317 | 5.106603327 |
| ENSMUSG00000015709 | Arnt2 | 5969 | 11.5929681 | 9.5602258 | 10.93246649 | 10.16708176 | 10.03117994 | 10.50992808 | 8.734892307 | 7.428611022 | 9.646297376 | 7.251523805 | 4.69825777 | 8.275567329 | 9.884565767 | 7.415180982 | 9.905215732 | 6.825465953 | 10.14500739 | 9.105209824 |
| ENSMUSG00000015711 | Prune | 3609 | 49.65414855 | 48.87552643 | 56.06718689 | 54.94088804 | 55.78715342 | 58.86189217 | 57.17112574 | 53.1667026 | 53.51165764 | 57.87686767 | 57.47821272 | 56.15590769 | 42.97576833 | 42.60913642 | 50.69994727 | 49.09133844 | 47.61848582 | 47.61663879 |
| ENSMUSG00000015714 | Lass2 | 3036 | 129.4615819 | 139.3413846 | 138.0462013 | 138.5589995 | 136.6908583 | 131.9121188 | 118.1258585 | 126.7796546 | 116.3017127 | 118.0631246 | 127.9349185 | 125.4197794 | 126.677084 | 119.5369232 | 125.6946017 | 121.6571802 | 130.1907444 | 128.1190361 |
| ENSMUSG00000015721 | Nlrp5 | 3662 | 0 | 0 | 0 | 0 | 0 | 0 | 0 | 0 | 0 | 0 | 0 | 0 | 0 | 0 | 0 | 0 | 0 | 0 |
| ENSMUSG00000015733 | Capza2 | 3522 | 113.6005741 | 106.5785611 | 104.671969 | 114.1095889 | 109.7932637 | 110.0515281 | 106.8363815 | 109.0467912 | 114.1107384 | 111.4106055 | 107.9209454 | 113.0576198 | 95.35823192 | 96.97609843 | 111.8127403 | 106.8157915 | 122.1718726 | 102.9741739 |
| ENSMUSG00000015745 | Plekho1 | 2980 | 0.98418025 | 0.849590591 | 0.990196427 | 0.795024935 | 1.744378442 | 1.213875688 | 0.349288234 | 0.499833454 | 0.760153814 | 0.443026742 | 0.545083419 | 0.59936282 | 0.331086693 | 0.779263211 | 0.58069307 | 1.105030952 | 0.463002244 | 0.221513181 |
| ENSMUSG00000015747 | Vps45 | 3976 | 9.706916457 | 9.777703107 | 9.891267651 | 9.80891699 | 8.824993047 | 9.733572667 | 9.983736826 | 10.66236586 | 11.79865228 | 10.0918633 | 8.483179247 | 10.33207436 | 12.40742386 | 10.01070998 | 8.673466368 | 12.76995609 | 10.29489062 | 11.05439544 |
| ENSMUSG00000015748 | Prpf3 | 6021 | 4.7439758 | 4.575612609 | 4.491081792 | 4.668853073 | 4.660506809 | 3.431903322 | 3.13531953 | 4.192854674 | 3.132938751 | 4.236593666 | 4.022903828 | 2.994263248 | 3.408416806 | 4.967612071 | 3.291471248 | 3.090721674 | 4.423978799 | 3.901160699 |
| ENSMUSG00000015749 | Anp32e | 3929 | 22.35064741 | 25.4955136 | 23.82356356 | 24.73446362 | 23.37381277 | 25.9808173 | 24.85450988 | 26.54707329 | 26.36398247 | 24.46940289 | 23.97867603 | 26.72162416 | 25.21213792 | 25.23749722 | 29.26789068 | 26.68355385 | 31.13706788 | 26.86751738 |
| ENSMUSG00000015750 | Aph1a | 2242 | 10.88223508 | 8.157350101 | 8.371511944 | 9.063437009 | 9.789543797 | 7.470483474 | 7.006159413 | 7.529456674 | 6.778690596 | 8.685655863 | 6.563196794 | 7.767389263 | 8.427352505 | 7.789017332 | 7.608137439 | 8.129495595 | 8.000315321 | 6.992683058 |
| ENSMUSG00000015755 | Tab2 | 6375 | 6.760823293 | 5.392769699 | 5.857939676 | 5.067102813 | 5.337924711 | 4.795927313 | 4.794351465 | 5.643489108 | 4.961763781 | 3.823829582 | 4.414031458 | 5.463368385 | 7.513928106 | 7.197922877 | 5.965338994 | 10.33095604 | 6.877691554 | 6.117875556 |
| ENSMUSG00000015757 | Ppil4 | 6549 | 11.92273757 | 15.08719564 | 13.87904467 | 12.71729868 | 13.47164698 | 13.35479841 | 13.41573443 | 15.1276671 | 14.3766033 | 14.87453854 | 15.28302611 | 14.86372203 | 16.7377493 | 18.75776625 | 16.87948915 | 17.0024563 | 18.13608094 | 14.09456117 |
| ENSMUSG00000015759 | Cnih | 1564 | 51.50083668 | 43.685899 | 48.77561213 | 42.91011401 | 44.03886366 | 43.21606688 | 57.56779284 | 55.26176895 | 55.51226038 | 54.0544827 | 43.71225421 | 52.3539495 | 57.31208037 | 58.05510924 | 65.06895336 | 52.14769422 | 51.58366687 | 57.47115114 |
| ENSMUSG00000015766 | Eps8 | 7160 | 52.09258654 | 56.37602504 | 55.87953486 | 63.55638419 | 56.19872652 | 61.89247156 | 52.99548673 | 58.78749538 | 50.7065833 | 63.21223472 | 60.91307206 | 59.68222245 | 45.72848254 | 43.82349922 | 51.4501552 | 44.03419851 | 57.64467756 | 52.98083798 |
| ENSMUSG00000015776 | Med22 | 4224 | 14.68159371 | 21.14390848 | 17.72779948 | 24.47396061 | 18.56224045 | 17.78455116 | 21.74098243 | 23.08361822 | 21.59756923 | 27.95107977 | 19.31809401 | 16.5967027 | 22.35352502 | 24.13465242 | 22.28824697 | 19.7889195 | 21.44966048 | 18.60985168 |
| ENSMUSG00000015787 | Abo | 1913 | 4.77711074 | 5.380911821 | 6.877992816 | 6.620998602 | 7.070083926 | 5.750500185 | 9.719750559 | 11.95883379 | 8.56886214 | 12.24981679 | 10.96351635 | 7.469320242 | 21.81641506 | 20.75781219 | 25.58674773 | 18.9151197 | 15.36658114 | 22.11291326 |
| ENSMUSG00000015790 | Surf1 | 1745 | 37.21943207 | 30.08660246 | 33.1268589 | 29.52982301 | 34.20262771 | 30.52675564 | 36.81983839 | 37.16372452 | 36.8436396 | 35.01851657 | 34.46915342 | 29.01135012 | 44.58248341 | 45.45931304 | 37.40015159 | 41.62594971 | 39.797851 | 41.92671171 |
| ENSMUSG00000015804 | Med28 | 5182 | 42.24269713 | 38.58432866 | 37.65704775 | 36.76007842 | 39.74459695 | 38.02995396 | 43.53277752 | 38.30295548 | 42.07669132 | 40.49027972 | 36.29679883 | 36.67547879 | 43.91511663 | 42.41988916 | 41.24131038 | 43.54429746 | 36.47728605 | 39.76536352 |
| ENSMUSG00000015806 | Qdpr | 2256 | 30.95567563 | 29.99046461 | 27.4245325 | 28.19291573 | 29.95444186 | 28.97165585 | 35.71520045 | 32.25023341 | 30.08660371 | 33.52381561 | 31.02407543 | 33.30136606 | 39.25123737 | 34.95658705 | 37.12888436 | 34.69237656 | 34.24903128 | 31.11330682 |
| ENSMUSG00000015812 | Gnrh1 | 517 | 0.822150407 | 0 | 0.654960759 | 0.440629055 | 0.186196997 | 0 | 0.183027771 | 0.221619356 | 0.477986643 | 0 | 0.369632174 | 0.539803313 | 0 | 0.269501474 | 0.557854722 | 0.592504563 | 0.074132102 | 0 |
| ENSMUSG00000015829 | Tnr | 4927 | 0 | 0 | 0.029454149 | 0 | 0 | 0 | 0 | 0 | 0 | 0 | 0 | 0 | 0 | 0 | 0 | 0 | 0 | 0 |
| ENSMUSG00000015837 | Sqstm1 | 4024 | 155.6340118 | 174.9175303 | 165.7250222 | 213.3579323 | 177.875208 | 184.1972636 | 143.713438 | 158.1985495 | 134.2451145 | 166.117177 | 179.3341819 | 160.2342282 | 137.4649077 | 157.7990961 | 151.5467765 | 229.1346487 | 209.4993108 | 200.9800912 |
| ENSMUSG00000015839 | Nfe2l2 | 3790 | 119.5191717 | 107.7439201 | 109.6381598 | 109.8153404 | 116.6341918 | 109.1598345 | 79.2206492 | 76.54603923 | 77.4284762 | 88.44178086 | 74.43565431 | 80.54244292 | 70.3012362 | 80.91564882 | 74.1628006 | 81.71357062 | 81.00095995 | 80.67034701 |
| ENSMUSG00000015843 | Rxrg | 2583 | 0 | 0 | 0 | 0 | 0 | 0 | 0 | 0 | 0 | 0 | 0 | 0 | 0 | 0 | 0 | 0 | 0 | 0 |
| ENSMUSG00000015846 | Rxra | 5359 | 2.934673471 | 2.567312057 | 3.448159842 | 3.247680339 | 2.613620039 | 3.559955669 | 4.370176538 | 4.026629475 | 4.61897667 | 4.707151753 | 4.698153222 | 5.384716842 | 9.426363742 | 8.657892014 | 6.750320222 | 11.31069817 | 6.79417467 | 9.187003269 |
| ENSMUSG00000015850 | Adamtsl4 | 4944 | 7.075598679 | 8.591007308 | 8.98197833 | 9.897362787 | 8.314049922 | 8.238743325 | 7.627064139 | 8.999625567 | 9.955074097 | 8.583259088 | 7.701586146 | 8.376865218 | 4.939178611 | 3.954886891 | 8.458652757 | 7.148509432 | 6.604774466 | 7.243310776 |
| ENSMUSG00000015852 | Fcrls | 2325 | 0 | 0 | 0 | 0 | 0 | 0 | 0 | 0 | 0.336577978 | 0 | 0 | 0 | 0 | 0 | 0 | 0 | 0 | 0 |
| ENSMUSG00000015854 | Cd5l | 2031 | 0 | 0 | 0 | 0 | 0 | 0 | 0 | 0 | 0 | 0 | 0 | 0 | 0 | 0 | 0 | 0 | 0 | 0 |
| ENSMUSG00000015869 | Prpsap1 | 3653 | 32.76610339 | 27.29413972 | 29.79481065 | 32.45273402 | 32.34707709 | 30.53468472 | 25.6832856 | 29.27422029 | 34.1510967 | 30.50016748 | 31.93716086 | 34.31754125 | 28.67004117 | 24.16923354 | 27.79103031 | 27.95539281 | 25.28507406 | 29.19864712 |
| ENSMUSG00000015879 | Fam184b | 4106 | 0.051759834 | 0.105471901 | 0.294529532 | 0 | 0 | 0.181025573 | 2.846135337 | 1.43244763 | 1.95600842 | 0.378951098 | 0.698124089 | 1.046713595 | 0.78094852 | 0.803100391 | 0.270930835 | 0.307742339 | 0.158681697 | 0.388520237 |
| ENSMUSG00000015880 | Ncapg | 6244 | 0.047651543 | 0.053351849 | 0.077472022 | 0.021890316 | 0.131044635 | 0.03968028 | 0.197009874 | 0.110099815 | 0.224269945 | 0.113270427 | 0 | 0.232416276 | 0.023702074 | 0.438853484 | 0.362922097 | 0.245295371 | 0.135038202 | 0.32596684 |
| ENSMUSG00000015882 | Lcorl | 12290 | 2.621556015 | 2.775622757 | 2.723717088 | 3.314204527 | 2.772774773 | 3.25378812 | 3.283784795 | 2.989511075 | 2.121323391 | 2.547442389 | 2.989336295 | 3.219959705 | 2.681344243 | 2.414790223 | 2.410408355 | 2.0687521 | 3.199575315 | 2.810880311 |
| ENSMUSG00000015889 | Lta4h | 2047 | 30.81469527 | 26.77074303 | 26.37267214 | 30.98240045 | 24.66555346 | 28.39538666 | 31.17967947 | 23.58338869 | 30.50256859 | 28.70042807 | 23.4323685 | 27.67606132 | 26.05168665 | 24.48127518 | 26.46802442 | 42.29363133 | 25.0890268 | 34.10188394 |
| ENSMUSG00000015890 | Amdhd1 | 2412 | 0.105734269 | 0.110490528 | 0.441217939 | 0.056667966 | 0.159641538 | 0.225986762 | 1.176932312 | 1.203409031 | 0.785480815 | 0.254128998 | 0.356529541 | 0.046281644 | 0.143168914 | 0.231065111 | 0.153737149 | 0 | 0.190678094 | 0.159645003 |

| ENSMUSG00000015932 | Dstn | 1907 | 319.802971 | 344.1691882 | 340.7708478 | 331.3030948 | 368.0943759 | 320.2608502 | 296.603606 | 321.2808177 | 325.8853363 | 304.1183273 | 292.7374881 | 306.7958968 | 249.6603742 | 225.8395503 | 245.8049824 | 260.5849517 | 293.3258014 | 261.8629632 |
| --- | --- | --- | --- | --- | --- | --- | --- | --- | --- | --- | --- | --- | --- | --- | --- | --- | --- | --- | --- | --- |
| ENSMUSG00000015937 | H2afy | 7022 | 46.39734752 | 43.25173122 | 43.33088948 | 42.56992479 | 42.55240418 | 44.30954345 | 43.74839423 | 41.11312586 | 47.41552171 | 41.41633975 | 41.35234942 | 43.71776477 | 43.24797533 | 43.99026278 | 41.38344826 | 42.34760141 | 44.01902371 | 40.42252787 |
| ENSMUSG00000015942 | Gtf2ird2 | 4504 | 4.888472731 | 4.807589086 | 4.618254452 | 4.491364051 | 5.663836488 | 5.643994744 | 4.958167064 | 5.452423345 | 5.614680435 | 3.716363156 | 5.261185481 | 4.213438117 | 4.819281439 | 4.310308288 | 3.238310565 | 4.191223235 | 3.820716543 | 4.836502278 |
| ENSMUSG00000015943 | Bola1 | 1493 | 27.53014415 | 25.32493968 | 27.92899073 | 22.24648447 | 27.72501966 | 27.282215 | 33.55936168 | 28.67627803 | 31.03471549 | 29.52840425 | 30.55933379 | 33.49687453 | 37.9654206 | 36.08515832 | 32.094715 | 25.85193052 | 26.54346349 | 37.21312645 |
| ENSMUSG00000015944 | Gatsl2 | 7551 | 3.540690733 | 3.917606948 | 3.376089326 | 4.187440706 | 2.849287496 | 4.062129795 | 2.706804039 | 3.232014983 | 2.907219296 | 4.514628321 | 4.156819987 | 3.185876119 | 3.469109797 | 3.838049335 | 4.523386284 | 4.873165635 | 4.126510314 | 3.110699056 |
| ENSMUSG00000015947 | Fcgr1 | 2953 | 0.028787793 | 0 | 0 | 0.015428732 | 0 | 0.016780472 | 0.064087611 | 0 | 0.013947347 | 0 | 0 | 0 | 0 | 0 | 0.055809761 | 0.025933361 | 0 | 0 |
| ENSMUSG00000015950 | Ncf1 | 3200 | 0.58444617 | 0.218615868 | 0.136050554 | 0.327470006 | 0.58660782 | 0.278734126 | 1.552447277 | 0.202897138 | 1.094016825 | 0.235753516 | 0.477749585 | 0.331405497 | 0.662897638 | 0.246734214 | 0.025750972 | 0.562393296 | 0.071861807 | 0.017190346 |
| ENSMUSG00000015957 | Wnt11 | 3843 | 0.497718689 | 0.26872228 | 0.276923671 | 0.37937898 | 0.137770273 | 0.06447142 | 0 | 0.029814522 | 0 | 0.061346218 | 0 | 0 | 0.038510474 | 0 | 0.268029898 | 0.009963728 | 0 | 0 |
| ENSMUSG00000015961 | Adss | 5639 | 17.83423417 | 17.3564964 | 16.77932707 | 17.92062357 | 19.06840175 | 18.03195767 | 17.07418011 | 15.01552689 | 15.6960139 | 16.07087279 | 14.47058505 | 16.51011264 | 17.84662374 | 15.8794468 | 18.8508626 | 20.12650476 | 17.7188608 | 19.68582681 |
| ENSMUSG00000015962 | 1700016C15R | 762 | 0.446248567 | 0 | 0 | 0.17937419 | 0.126330508 | 0 | 0 | 0.200485052 | 0 | 0.433142943 | 0.188090388 | 0.073248901 | 0.453180342 | 1.036154181 | 0.108140567 | 0.703501973 | 0.251484888 | 0.721904288 |
| ENSMUSG00000015966 | Il17rb | 3277 | 3.437251037 | 2.490588674 | 2.760405913 | 4.407339351 | 4.465091485 | 3.674493213 | 5.255360125 | 5.139716039 | 4.700566645 | 5.23736832 | 4.548607582 | 4.019681529 | 4.230170508 | 5.584061773 | 5.016606897 | 5.807272468 | 4.467697716 | 4.683411894 |
| ENSMUSG00000015968 | Cacna1d | 9297 | 0.100582325 | 0.168409813 | 0.057234466 | 0.083310504 | 0.150137226 | 0.261168543 | 0.371498931 | 0.328642809 | 0.124042427 | 0.192720956 | 0.128468749 | 0.144086899 | 0.679196021 | 0.364678754 | 0.252607151 | 0.226522901 | 0.061836555 | 0.218924056 |
| ENSMUSG00000015970 | Chdh | 2784 | 9.099527627 | 8.316257716 | 7.818997363 | 8.068101601 | 6.915506274 | 8.970753493 | 9.890797103 | 12.01743696 | 8.136703904 | 12.88853634 | 11.13719398 | 11.90894525 | 15.22130637 | 9.24210647 | 13.42306437 | 10.37037284 | 11.16473663 | 12.27035031 |
| ENSMUSG00000015971 | Actr8 | 2108 | 37.88767825 | 38.84397248 | 36.16540979 | 39.53090611 | 34.40930217 | 34.27319049 | 39.21027044 | 32.63022615 | 38.06040451 | 39.05366598 | 38.8908106 | 40.51136801 | 37.70100509 | 40.12081743 | 35.39659285 | 40.74280474 | 41.63530356 | 37.4990922 |
| ENSMUSG00000015980 | Lrrc27 | 3781 | 4.991351007 | 4.405302065 | 6.652799731 | 2.952249633 | 5.562986153 | 4.311782425 | 6.594493995 | 8.798091112 | 8.300482679 | 7.956135593 | 8.680612662 | 6.84963434 | 7.423925134 | 8.426512549 | 10.14510673 | 9.641009851 | 6.923263894 | 5.630395213 |
| ENSMUSG00000015981 | Stk32c | 2289 | 4.939439416 | 4.162292594 | 4.142075994 | 2.448234361 | 4.478855282 | 3.983269104 | 3.472490197 | 2.819797294 | 3.688613251 | 3.52239854 | 4.362152172 | 2.682272995 | 3.254311712 | 2.962357693 | 4.967946451 | 6.038841971 | 4.805437838 | 2.37916189 |
| ENSMUSG00000015994 | Fnta | 1865 | 83.80243016 | 80.55825894 | 85.3345388 | 77.44156061 | 77.19173388 | 76.22884324 | 79.22653957 | 87.93467878 | 86.37022153 | 84.54260144 | 88.42850224 | 88.28750919 | 91.12517695 | 90.04923324 | 94.35487679 | 88.57141036 | 91.03779908 | 96.33230164 |
| ENSMUSG00000016018 | Skiv2l2 | 3282 | 20.04814518 | 21.35598094 | 21.43056464 | 22.89157895 | 23.99263471 | 24.76126845 | 19.53341863 | 23.087668 | 14.68257875 | 20.75952657 | 18.89454482 | 21.53035613 | 14.53502864 | 15.63702516 | 21.22846771 | 17.72194779 | 23.68242843 | 19.71075853 |
| ENSMUSG00000016024 | Lbp | 4963 | 0.32544765 | 0.489994213 | 0.438607468 | 0.550808515 | 0.446114948 | 0.289548514 | 0.076264645 | 0.253949079 | 0 | 0.731534183 | 0.009626226 | 0.134956267 | 1.162972863 | 1.207190666 | 1.419598238 | 1.002977828 | 0.872631785 | 0.709365874 |
| ENSMUSG00000016028 | Celsr1 | 11039 | 4.913180959 | 6.681288867 | 4.693183351 | 5.984556055 | 6.191442305 | 5.997142138 | 4.097374858 | 6.231045994 | 5.30175187 | 5.155439699 | 4.682716285 | 4.939931366 | 3.735979966 | 4.017945779 | 3.54201255 | 4.391331549 | 5.457825775 | 3.94167981 |
| ENSMUSG00000016087 | Fli1 | 3099 | 0 | 0 | 0 | 0 | 0 | 0 | 0.977093079 | 0.098592843 | 0.03987078 | 0.030429625 | 0.04624875 | 0 | 0.382047762 | 0 | 0 | 0.148269535 | 0 | 0 |
| ENSMUSG00000016128 | Stard13 | 8028 | 1.636036297 | 1.622488993 | 1.373837185 | 2.167952035 | 1.942544005 | 1.715951661 | 1.573553223 | 1.084687064 | 0.867030539 | 1.28037535 | 1.791263391 | 1.578245566 | 1.235141427 | 1.243831439 | 0.8262893 | 1.12086357 | 1.312871406 | 1.836377753 |
| ENSMUSG00000016150 | Odz1 | 18543 | 0 | 0 | 0 | 0 | 0 | 0 | 0 | 0.037074003 | 0 | 0 | 0 | 0 | 0 | 0 | 0 | 0 | 0 | 0 |
| ENSMUSG00000016179 | Camk1g | 2907 | 0 | 0 | 0 | 0.062691495 | 0 | 0 | 0 | 0 | 0 | 0 | 0 | 0.019200434 | 0 | 0 | 0 | 0 | 0.079104844 | 0 |
| ENSMUSG00000016181 | Diexf | 5536 | 0.875287223 | 0.758205324 | 0.917489286 | 0.658396594 | 1.025933344 | 0.769785962 | 0.692255598 | 0.55191333 | 0.996927947 | 0.570646156 | 1.458447974 | 0.846913955 | 1.113889028 | 1.166135865 | 0.625168117 | 1.00983177 | 0.720002687 | 1.122837619 |
| ENSMUSG00000016194 | Hsd11b1 | 3290 | 0 | 0 | 0.117625606 | 0 | 0.014629764 | 0.015061621 | 0.014380753 | 0.046434532 | 0.062593489 | 0 | 0 | 0 | 2.77398319 | 2.442196692 | 0.801489233 | 0.151300272 | 0.209687947 | 1.337607458 |
| ENSMUSG00000016200 | Syt14 | 2226 | 0.171853811 | 0.314272587 | 0.325967186 | 0.163741399 | 0.38920693 | 0.178087093 | 0.191291155 | 0.428935337 | 0.592079292 | 0.529545185 | 0.794103083 | 0.025074422 | 0.354587006 | 1.084947235 | 0.129564641 | 0.447239799 | 0.55096204 | 0.09884835 |
| ENSMUSG00000016206 | H2-M3 | 1430 | 10.49253646 | 7.361450751 | 7.983323891 | 11.24688717 | 10.43419324 | 9.771937671 | 6.650243681 | 7.825436297 | 8.381311949 | 8.144212381 | 8.051583911 | 6.752524215 | 4.070745621 | 7.015330683 | 6.252264076 | 11.11230914 | 7.022020831 | 9.424637166 |
| ENSMUSG00000016239 | Lonrf3 | 3983 | 1.333956064 | 0.635646488 | 0.485799953 | 0.789282465 | 0.954662809 | 0.734022416 | 0.451388852 | 0.393142982 | 0.341239021 | 0.639251312 | 0.239894343 | 0.882848793 | 0.445882255 | 0.373138538 | 1.034435247 | 0.644105121 | 0.789042517 | 0.566249906 |
| ENSMUSG00000016252 | Atp5e | 727 | 221.6466607 | 172.9337865 | 202.410288 | 172.7810168 | 212.1247365 | 183.1474486 | 293.6379743 | 212.8162615 | 243.7199323 | 268.5060681 | 239.9262358 | 227.178192 | 314.6521326 | 278.0256786 | 230.8307251 | 221.1058733 | 209.8720605 | 235.8505993 |
| ENSMUSG00000016253 | Th1l | 2975 | 32.68969504 | 33.6824154 | 31.98344007 | 35.16240598 | 35.35067335 | 34.64527255 | 29.07145448 | 30.66946196 | 30.69260687 | 32.90247394 | 39.71343607 | 36.0409707 | 32.46786354 | 31.90982004 | 30.99464944 | 32.7046835 | 33.39218876 | 35.16884739 |
| ENSMUSG00000016255 | Tubb1 | 1402 | 0 | 0 | 0 | 0 | 0 | 0 | 0 | 0 | 0 | 0 | 0 | 0 | 0.070373634 | 0 | 0 | 0.027311418 | 0 | 0 |
| ENSMUSG00000016256 | Ctsz | 1555 | 99.36097419 | 99.46737483 | 100.0134406 | 99.99989988 | 100.7829861 | 116.4409572 | 112.4550877 | 116.7140169 | 111.3757548 | 133.4471029 | 134.5379699 | 118.2358795 | 101.5190581 | 102.6847411 | 100.2880315 | 104.8744032 | 101.8666142 | 106.7282797 |
| ENSMUSG00000016257 | Slmo2 | 2204 | 70.75838968 | 67.89542687 | 64.32977199 | 68.27955056 | 64.48891588 | 67.17947728 | 93.29442043 | 78.11767243 | 78.48610082 | 84.03265081 | 70.81705506 | 68.68061567 | 63.05263656 | 63.66043013 | 61.85841581 | 64.88902842 | 61.3325087 | 68.36204325 |
| ENSMUSG00000016262 | Sertad4 | 6621 | 0.276049323 | 0.76980408 | 0.306855199 | 0.681248058 | 0.603375572 | 0.329303772 | 0.371584248 | 0.017305121 | 0.11819118 | 0.07121387 | 0 | 0.067440764 | 0.305483856 | 0.014029327 | 0.416931618 | 0 | 0.040520175 | 0.191090387 |
| ENSMUSG00000016283 | H2-M2 | 2176 | 0 | 0 | 0 | 0 | 0 | 0.091089584 | 0.065228877 | 0 | 0 | 0 | 0 | 0 | 0 | 0 | 0 | 0.035193573 | 0 | 0 |
| ENSMUSG00000016308 | Ube2a | 863 | 107.8144036 | 96.19435967 | 90.07678248 | 85.68432788 | 94.92530021 | 90.55001258 | 119.73452 | 107.983154 | 112.4396653 | 102.9338644 | 98.53931181 | 111.3079436 | 119.5284346 | 135.1345347 | 99.54257709 | 117.9776652 | 106.5408879 | 111.2293062 |
| ENSMUSG00000016319 | Slc25a5 | 1830 | 351.3993487 | 319.1484489 | 335.495545 | 325.5000512 | 351.3893444 | 332.030393 | 425.1677623 | 417.6954873 | 391.3394186 | 427.1132284 | 435.6136378 | 390.4351348 | 431.6138402 | 426.4988462 | 389.523562 | 391.9660921 | 398.9495244 | 432.5278889 |
| ENSMUSG00000016327 | Atp1b4 | 4193 | 0 | 0 | 0 | 0 | 0 | 0 | 0 | 0 | 0 | 0 | 0 | 0 | 0 | 0 | 0 | 0.082188282 | 0 | 0 |
| ENSMUSG00000016344 | Ppdpf | 1490 | 37.68411915 | 36.1970307 | 31.71875111 | 32.35139927 | 35.82436466 | 37.41397669 | 47.53495326 | 42.19107004 | 40.08083749 | 42.84701491 | 44.37620304 | 46.41315835 | 50.62315538 | 45.85184735 | 40.51025462 | 40.24368536 | 33.7734415 | 39.35550857 |
| ENSMUSG00000016346 | Kcnq2 | 14716 | 0.002888365 | 0 | 0 | 0 | 0 | 0 | 0 | 0 | 0 | 0 | 0 | 0 | 0.040227168 | 0.085212718 | 0.044796473 | 0.054641394 | 0.033857153 | 0.022428285 |

| ENSMUSG00000016349 | Eef1a2 | 2459 | 0 | 0 | 0 | 0 | 0 | 0 | 0 | 0 | 0 | 0 | 0 | 0 | 0 | 0 | 0 | 0 | 0 | 0 |
| --- | --- | --- | --- | --- | --- | --- | --- | --- | --- | --- | --- | --- | --- | --- | --- | --- | --- | --- | --- | --- |
| ENSMUSG00000016356 | Col20a1 | 4157 | 1.441719947 | 1.161984523 | 1.664040135 | 1.665931859 | 0.856810765 | 1.406596719 | 0.864990041 | 0.983061597 | 1.377177217 | 0.782631351 | 0.482691426 | 0.510222559 | 1.649539692 | 1.418907649 | 0.535213861 | 1.575100762 | 0.682257871 | 1.667343625 |
| ENSMUSG00000016382 | Pls3 | 5453 | 212.5102905 | 226.6229826 | 252.6015545 | 239.3521432 | 235.8402473 | 246.7458967 | 174.5701632 | 182.9145023 | 168.0240656 | 174.638319 | 172.7538293 | 184.8477995 | 157.2686686 | 152.1165232 | 186.8765967 | 178.1534936 | 209.1113122 | 183.5788968 |
| ENSMUSG00000016386 | Mpped2 | 3413 | 30.06372544 | 28.41307796 | 32.78288184 | 27.35264572 | 28.98069239 | 30.17010852 | 21.4175473 | 20.9146206 | 24.83499837 | 22.75335724 | 21.43084132 | 27.06560843 | 21.11747177 | 18.1666735 | 20.43780666 | 23.7282844 | 21.21253292 | 21.19454302 |
| ENSMUSG00000016409 | Nkap | 4317 | 7.217116986 | 7.585493402 | 7.182634459 | 7.166075771 | 9.454684102 | 6.611622548 | 6.082589021 | 6.900642547 | 7.670595013 | 7.842067394 | 6.949889721 | 7.473118595 | 6.330757973 | 5.346933387 | 5.917295495 | 5.871758652 | 5.806207592 | 7.772887445 |
| ENSMUSG00000016427 | Ndufa1 | 416 | 391.2315361 | 293.8901966 | 377.9182059 | 311.5893537 | 372.9067067 | 333.170663 | 517.7098906 | 401.0202253 | 442.7550444 | 425.6031027 | 407.5801145 | 414.5923014 | 666.0994696 | 761.3034421 | 515.9108285 | 456.5418575 | 493.8195947 | 624.9351884 |
| ENSMUSG00000016458 | Wt1 | 4845 | 0 | 0 | 0 | 0 | 0 | 0 | 0 | 0 | 0 | 0 | 0 | 0 | 0 | 0 | 0 | 0 | 0 | 0 |
| ENSMUSG00000016477 | E2f3 | 4786 | 1.802873116 | 2.143830217 | 1.465558274 | 1.332750983 | 1.397897491 | 1.936138984 | 1.04787797 | 1.17306376 | 1.411322312 | 1.06399414 | 1.407494598 | 2.040898653 | 1.968741371 | 1.814677046 | 0.912529236 | 2.176148185 | 2.090088486 | 1.195350418 |
| ENSMUSG00000016481 | Cr1l | 1776 | 41.18885582 | 43.98577531 | 42.5175005 | 44.94535455 | 45.09657713 | 45.64654962 | 46.59339834 | 47.93404559 | 46.98416719 | 46.03565284 | 47.04864999 | 52.76717201 | 49.22080935 | 53.34793822 | 53.47386618 | 58.3198722 | 55.11563192 | 55.53565786 |
| ENSMUSG00000016487 | Ppfibp1 | 7148 | 12.26751233 | 15.67775268 | 15.07104814 | 15.04891238 | 13.83759137 | 16.81798149 | 8.280380713 | 8.009290879 | 8.124368666 | 9.215099671 | 11.10827938 | 8.206807691 | 8.751101162 | 9.349893922 | 10.69810964 | 11.86537429 | 11.60834258 | 7.857344427 |
| ENSMUSG00000016493 | Cd46 | 3358 | 0.405052303 | 0.337295534 | 0.273703716 | 0.35276568 | 0.258003164 | 0.236105937 | 0.056358164 | 0.523183595 | 0.564198846 | 0.561652213 | 0.41258898 | 0.747976419 | 0.807997454 | 0.442588087 | 0.147236054 | 0.490320464 | 0.593498344 | 0.622497336 |
| ENSMUSG00000016494 | Cd34 | 2613 | 0.032533621 | 0.509956283 | 0.037025282 | 0.12205408 | 0.349983371 | 0.436170253 | 0.235386462 | 0 | 0 | 0 | 0 | 0 | 0 | 0.462130223 | 0 | 0 | 0.058670183 | 0 |
| ENSMUSG00000016495 | 5033414D02R | 3045 | 27.17818645 | 24.14501069 | 24.70306723 | 25.19697821 | 24.73775387 | 23.93828279 | 24.00594054 | 25.43651628 | 30.78506071 | 23.412763 | 22.07532564 | 25.277438 | 29.09692067 | 26.6614991 | 25.53278685 | 26.8725872 | 25.77742399 | 26.95355902 |
| ENSMUSG00000016496 | Cd274 | 3615 | 4.080026579 | 6.469059956 | 3.893968834 | 6.717575832 | 4.859793123 | 7.292407818 | 5.941896607 | 4.680286104 | 5.696613522 | 4.604204219 | 7.043998027 | 4.909925506 | 6.932396674 | 5.640098026 | 5.083234835 | 5.75153522 | 4.675490162 | 4.215082313 |
| ENSMUSG00000016498 | Pdcd1lg2 | 1639 | 1.244812965 | 1.422759363 | 1.032991801 | 0.833942239 | 1.673898505 | 1.330274727 | 0.548469127 | 1.001997134 | 0.929774913 | 0.949343019 | 0.408083843 | 1.123805287 | 0.481580645 | 0.28336844 | 0.377073422 | 0.163535236 | 0.631366697 | 1.040440701 |
| ENSMUSG00000016503 | Gtf3a | 2818 | 7.828313115 | 7.896740007 | 7.518667954 | 7.194700046 | 7.856878952 | 6.523798495 | 8.915199613 | 8.009833152 | 8.623152697 | 7.495924436 | 8.425888703 | 6.872971937 | 6.792329277 | 7.960430876 | 7.295804255 | 7.432562899 | 9.221160149 | 8.159619095 |
| ENSMUSG00000016510 | Mtif3 | 6417 | 4.067037257 | 4.433413069 | 4.455159177 | 4.231631981 | 4.26039156 | 4.447931205 | 4.93255138 | 4.880437895 | 4.788084893 | 6.230917308 | 6.320857059 | 5.323232897 | 5.758081041 | 6.137538742 | 6.144598564 | 5.34648344 | 5.787467929 | 5.443475576 |
| ENSMUSG00000016520 | Lnx2 | 5236 | 7.32232024 | 8.741771707 | 6.855072483 | 8.892931114 | 8.594986369 | 9.075834893 | 6.153539792 | 7.607844864 | 7.669376025 | 7.285125847 | 8.284885848 | 7.611226716 | 6.623441147 | 6.129271273 | 6.861680049 | 5.857673134 | 6.485313036 | 6.471659614 |
| ENSMUSG00000016524 | Il19 | 1459 | 0 | 0 | 0 | 0 | 0 | 0 | 0 | 0.026177109 | 0.169375665 | 0 | 0.032745002 | 0.038256109 | 0 | 0 | 0.112958344 | 0 | 0 | 0 |
| ENSMUSG00000016526 | Dyrk3 | 2069 | 0.842296867 | 0.434726024 | 0.1870412 | 0.308291262 | 0.279160505 | 0.407151508 | 1.326309994 | 1.292154745 | 0.218971326 | 0.227891268 | 0.161635916 | 0.458611051 | 0.667614154 | 0.987694464 | 1.035515179 | 0 | 0.629818315 | 0 |
| ENSMUSG00000016528 | Mapkapk2 | 2581 | 65.6269378 | 95.62775408 | 68.98991292 | 104.6262339 | 86.43605819 | 102.8684799 | 61.37266527 | 73.6620609 | 52.99512547 | 85.66046012 | 69.76513696 | 56.70231201 | 57.05376717 | 57.42079925 | 63.72592449 | 74.45972822 | 78.4345216 | 72.27271635 |
| ENSMUSG00000016529 | Il10 | 1305 | 0 | 0 | 0 | 0 | 0 | 0 | 0.761353454 | 0.029266209 | 0 | 0 | 0 | 0.171082491 | 0 | 0.213536034 | 0 | 0.088024385 | 0 | 0 |
| ENSMUSG00000016534 | Lamp2 | 9540 | 77.8905123 | 79.98237338 | 79.06080559 | 79.54079596 | 79.82634133 | 83.34104929 | 71.35585162 | 79.10311102 | 74.46815622 | 80.15619554 | 72.4036006 | 75.45060636 | 78.20711917 | 64.54949211 | 73.38541276 | 66.61522128 | 80.60574644 | 73.0629341 |
| ENSMUSG00000016541 | Atxn10 | 2666 | 139.2180034 | 129.2530299 | 142.6710687 | 125.8653757 | 135.6576423 | 137.4688738 | 110.6151267 | 113.4598001 | 111.4785813 | 108.1318078 | 108.2373403 | 101.0585909 | 124.6250798 | 112.8525124 | 124.8101145 | 125.7873742 | 123.0294257 | 114.3512639 |
| ENSMUSG00000016552 | Foxred2 | 8797 | 0.724767126 | 0.310521465 | 1.138265407 | 1.00993562 | 0.37752674 | 0.456265934 | 3.076372894 | 1.992760339 | 2.724855311 | 2.197543292 | 1.813895207 | 2.360284925 | 3.931075823 | 5.406245935 | 4.18713095 | 1.497325105 | 2.095594954 | 3.264152974 |
| ENSMUSG00000016554 | Eif3d | 1895 | 35.28266063 | 38.02416369 | 33.56791157 | 32.84242035 | 37.8451537 | 35.7459561 | 28.51244292 | 36.76144696 | 34.88356612 | 33.46580258 | 38.74939904 | 32.28177636 | 32.7751366 | 28.52818877 | 34.15706823 | 32.7743352 | 33.24983225 | 33.67312074 |
| ENSMUSG00000016559 | H3f3b | 4546 | 457.328018 | 412.8791023 | 470.7427649 | 524.0722766 | 490.4352917 | 504.8143857 | 415.3441247 | 483.277553 | 460.0097121 | 484.5445236 | 501.1851671 | 536.2158892 | 357.9005639 | 410.8656882 | 466.5763522 | 598.2717891 | 533.1536933 | 610.1461039 |
| ENSMUSG00000016619 | Nup50 | 5088 | 9.364839294 | 10.03708075 | 7.948166544 | 9.706794823 | 9.686928034 | 9.865746681 | 7.569284718 | 9.03766755 | 9.55999904 | 9.989869912 | 10.67612574 | 8.403065554 | 8.735860351 | 11.86661038 | 9.280067424 | 12.15395656 | 10.92992469 | 8.497869081 |
| ENSMUSG00000016624 | Phf21b | 6107 | 0.027840299 | 0.070913317 | 0.007920997 | 0 | 0.023644305 | 0.016228175 | 0.007747287 | 0.068792603 | 0.020232446 | 0 | 0.062583866 | 0.228490513 | 0 | 0.022815173 | 0 | 0.131669028 | 0.050206382 | 0.054045299 |
| ENSMUSG00000016626 | Nlrp14 | 3634 | 0 | 0 | 0 | 0 | 0 | 0 | 0 | 0 | 0 | 0 | 0 | 0 | 0 | 0 | 0 | 0 | 0 | 0 |
| ENSMUSG00000016637 | Ift27 | 1005 | 39.84067246 | 40.30694465 | 48.08473316 | 39.16889782 | 40.56491477 | 40.48039231 | 38.69753441 | 33.63211555 | 45.81743743 | 44.66414876 | 44.54242397 | 34.32246714 | 45.6995174 | 40.20532938 | 43.21038797 | 47.20603241 | 38.89833119 | 44.11675624 |
| ENSMUSG00000016664 | Pacsin2 | 5187 | 12.30822718 | 10.67399849 | 10.59424147 | 11.48907769 | 9.557717636 | 10.94803021 | 10.03353515 | 9.734018889 | 11.27527518 | 9.308332396 | 8.796044987 | 11.78294786 | 7.608547103 | 5.399225891 | 7.220399905 | 6.503571452 | 6.583522371 | 3.234582138 |
| ENSMUSG00000016756 | Cmah | 9953 | 0.128117681 | 0.063593388 | 0.165246663 | 0.141906197 | 0.096718424 | 0.069701424 | 0.35176713 | 0.076745509 | 0.041381006 | 0.023686679 | 0.225602637 | 0.022431694 | 0.272606797 | 0.214651665 | 0.020698059 | 0.019235712 | 0 | 0.011053774 |
| ENSMUSG00000016757 | Ttll12 | 5401 | 4.359908817 | 5.890356226 | 5.37384155 | 5.668769079 | 4.527127795 | 5.486490405 | 5.133351204 | 5.261090055 | 4.415292099 | 5.299106994 | 4.891603784 | 5.900896748 | 4.667396728 | 4.746738796 | 4.203306294 | 3.934694891 | 4.633784832 | 4.481393623 |
| ENSMUSG00000016758 | Bik | 938 | 155.9278366 | 144.5808021 | 139.6025018 | 164.0780467 | 168.8209263 | 159.3294717 | 161.6096197 | 157.8184986 | 153.681029 | 179.4039018 | 162.2718739 | 183.1563 | 143.8935242 | 124.3295346 | 126.1962368 | 174.4304535 | 147.1762489 | 143.7977928 |
| ENSMUSG00000016763 | Scube1 | 10802 | 0.428907997 | 0.157281763 | 0.205997259 | 0.299466224 | 0.316364246 | 0.26606726 | 0.065699887 | 0.141427152 | 0.224958751 | 0.074204958 | 0.216716623 | 0.139513321 | 0 | 0 | 0 | 0 | 0.117086447 | 0.198607217 |
| ENSMUSG00000016831 | Tox4 | 6057 | 14.96137284 | 18.21016886 | 16.18840119 | 18.94803875 | 17.80015008 | 18.16197268 | 16.05990885 | 17.85086531 | 14.57201639 | 16.83008427 | 17.82588842 | 16.02500202 | 15.3118713 | 14.63023258 | 17.7812229 | 18.57951051 | 21.28605956 | 19.27180526 |
| ENSMUSG00000016833 | Mrps18c | 1194 | 68.2076359 | 52.50826375 | 59.91997604 | 54.83351818 | 66.07053843 | 54.98942378 | 64.1534566 | 62.18260492 | 62.60764331 | 60.93260898 | 63.53989453 | 52.87061506 | 72.01468326 | 68.96596896 | 63.1481133 | 70.07117016 | 70.65006657 | 66.98763918 |
| ENSMUSG00000016918 | Sulf1 | 4586 | 0 | 0 | 0.073836614 | 0.069543679 | 0.052477021 | 0 | 0.196018513 | 0 | 0 | 0 | 0 | 0 | 0 | 0.597514426 | 0.035936813 | 0.108542934 | 0.242359924 | 0 |
| ENSMUSG00000016921 | Srsf6 | 4003 | 60.42891751 | 55.35782472 | 57.15883052 | 60.80117383 | 49.88742226 | 55.84121438 | 56.44900189 | 57.20750558 | 56.43468373 | 56.05550757 | 48.62232846 | 59.69194393 | 45.90591852 | 58.66133046 | 56.99051086 | 49.60656757 | 53.17618111 | 50.90025763 |
| ENSMUSG00000016933 | Plcg1 | 7278 | 10.36640388 | 11.15464732 | 10.12932952 | 10.36673391 | 8.994148968 | 10.68263795 | 8.080469897 | 7.173538622 | 8.296157201 | 9.361468289 | 8.448251108 | 8.389988303 | 9.374284363 | 8.327773295 | 9.204964259 | 8.354696971 | 10.14767438 | 9.478073623 |

| ENSMUSG00000016940 | Kctd2 | 2620 | 19.72759315 | 20.42004127 | 17.59541009 | 18.39831485 | 18.86697924 | 19.99131275 | 19.90021839 | 26.06412805 | 23.18706517 | 21.0198555 | 22.97574339 | 24.30750802 | 15.94814272 | 18.4535477 | 19.61005351 | 21.51289088 | 16.35450379 | 18.0354285 |
| --- | --- | --- | --- | --- | --- | --- | --- | --- | --- | --- | --- | --- | --- | --- | --- | --- | --- | --- | --- | --- |
| ENSMUSG00000016942 | Tmprss6 | 3173 | 0 | 0 | 0 | 0.028717961 | 0 | 0.031233995 | 0 | 0 | 0 | 0 | 0 | 0 | 0 | 0.043911838 | 0 | 0 | 0 | 0 |
| ENSMUSG00000016946 | Kctd5 | 2744 | 18.97552501 | 16.86283166 | 17.54069341 | 16.08915888 | 18.27750163 | 17.89604919 | 16.55254073 | 13.75149183 | 16.78080343 | 16.94263608 | 12.18748941 | 16.45585679 | 13.21390641 | 13.18511081 | 12.2073123 | 10.07500676 | 14.73551138 | 17.36074578 |
| ENSMUSG00000016982 | Pom121l2 | 4900 | 0 | 0 | 0 | 0 | 0 | 0 | 0 | 0 | 0 | 0 | 0 | 0 | 0 | 0 | 0 | 0 | 0 | 0 |
| ENSMUSG00000016984 | Etaa1 | 4179 | 2.817404585 | 4.846671377 | 2.893846037 | 2.845521075 | 3.754727714 | 3.28454348 | 5.264512013 | 4.670092756 | 5.174185397 | 2.865824031 | 4.618588951 | 3.913373807 | 5.229490154 | 4.489928525 | 4.900017535 | 3.573423519 | 4.062825922 | 2.790603165 |
| ENSMUSG00000016995 | Matn4 | 5221 | 0 | 0 | 0 | 0 | 0 | 0 | 0 | 0.014630302 | 0 | 0 | 0 | 0.192430938 | 0 | 0.169016981 | 0.007891507 | 0 | 0 | 0.052680623 |
| ENSMUSG00000016998 | Svs4 | 728 | 0 | 0 | 0 | 0 | 0 | 0 | 0 | 0 | 0 | 0 | 0 | 0 | 2.032908679 | 0.893155527 | 1.697866314 | 0.105193976 | 0.421168098 | 0 |
| ENSMUSG00000017000 | Svs6 | 531 | 0 | 0.062736147 | 0 | 0 | 0 | 0 | 0 | 0 | 0 | 0 | 0 | 0.105114242 | 0.278711397 | 0 | 0 | 0 | 0 | 0 |
| ENSMUSG00000017002 | Slpi | 1325 | 0 | 0 | 0 | 0 | 0 | 0.448779474 | 0 | 0 | 0 | 0 | 0 | 0 | 0 | 0 | 0.093286542 | 0 | 0.115702028 | 0 |
| ENSMUSG00000017003 | Svs3a | 1288 | 0 | 0 | 0 | 0 | 0 | 0 | 0 | 0 | 0 | 0 | 0 | 0 | 0 | 0 | 0 | 0.059457465 | 0 | 0 |
| ENSMUSG00000017004 | Svs5 | 643 | 0 | 0.310851268 | 0 | 0 | 0 | 0 | 0 | 0.118794409 | 0.192161038 | 0 | 0 | 0.43402537 | 0.306885955 | 1.227915219 | 0.320385349 | 0 | 0.178816315 | 0 |
| ENSMUSG00000017007 | Rbpjl | 4128 | 0.03089039 | 0 | 0 | 0 | 0 | 0 | 0 | 0.009252035 | 0 | 0 | 0.023146782 | 0 | 0.131456175 | 0.337529705 | 0 | 0.278274763 | 0.111413654 | 0 |
| ENSMUSG00000017009 | Sdc4 | 2878 | 118.6984363 | 190.0154522 | 155.8105026 | 142.3030672 | 174.5325766 | 164.3780221 | 98.01188039 | 165.9339123 | 153.8984679 | 124.1678874 | 158.5305259 | 167.6215329 | 79.58585543 | 98.48793097 | 102.4885124 | 83.48629654 | 149.7628683 | 122.3465226 |
| ENSMUSG00000017049 | Ccdc70 | 1112 | 0 | 0 | 0 | 0 | 0 | 0 | 0 | 0 | 0 | 0 | 0 | 0 | 0 | 0 | 0 | 0 | 0 | 0 |
| ENSMUSG00000017057 | Il13ra1 | 3815 | 50.22629975 | 50.16581318 | 53.48349962 | 55.27038353 | 51.22296464 | 58.35922201 | 37.1804486 | 40.59506992 | 39.43757328 | 35.48353055 | 41.50097311 | 39.70739404 | 29.18535709 | 27.82993022 | 36.62240525 | 40.31805241 | 41.91279441 | 44.95895013 |
| ENSMUSG00000017064 | Prl5a1 | 1056 | 0 | 0 | 0 | 0 | 0 | 0 | 0 | 0 | 0.312019059 | 0 | 0 | 0 | 0 | 0 | 0 | 0 | 0 | 0 |
| ENSMUSG00000017119 | Nbr1 | 7212 | 19.82631893 | 24.07009037 | 21.64467311 | 27.58174148 | 25.03367245 | 25.80699769 | 19.57585052 | 23.8411253 | 18.74865935 | 27.41304753 | 24.83476419 | 21.22883562 | 19.46736503 | 17.85122161 | 24.46842261 | 21.18938077 | 23.76528005 | 22.80604951 |
| ENSMUSG00000017132 | Cyth1 | 3950 | 45.44287554 | 43.16339054 | 39.04172526 | 41.15488764 | 43.51369605 | 44.71036518 | 32.65173741 | 42.5917804 | 36.54651589 | 38.95006196 | 35.8735511 | 39.05683326 | 35.86867504 | 33.65138686 | 32.51272145 | 30.92330066 | 33.46516406 | 30.15056103 |
| ENSMUSG00000017144 | Rnd3 | 5138 | 0.496362507 | 1.31617702 | 0.696699347 | 0.753734677 | 1.489485376 | 1.533453608 | 0.423585681 | 0.839965253 | 0.240481797 | 0.642380153 | 0.316144139 | 0.716978149 | 0.64329281 | 0.795461209 | 0.737746816 | 1.013531064 | 1.320310345 | 0.920744099 |
| ENSMUSG00000017146 | Brca1 | 8453 | 0.08045461 | 0.378331698 | 0.005722646 | 0.097018668 | 0.02847032 | 0.093794361 | 0.240677297 | 0.027109241 | 0.02436207 | 0.027889923 | 0.192162379 | 0.125458132 | 0.169244718 | 0.390101763 | 0.331445144 | 0.108715791 | 0.04080642 | 0.247290436 |
| ENSMUSG00000017165 | Gast | 457 | 0 | 0 | 0 | 0 | 0 | 0 | 0 | 0 | 0 | 0 | 0 | 0 | 0 | 0.3048846 | 0 | 0 | 0.503189894 | 0 |
| ENSMUSG00000017167 | Cntnap1 | 5850 | 0.087190105 | 0.005694512 | 0 | 0.023364638 | 0.057593755 | 0.127058291 | 0 | 0.006528616 | 0.098566021 | 0.064479594 | 0.089833255 | 0.190822778 | 0.160223321 | 0.063513282 | 0.147903021 | 0.098181045 | 0.058963534 | 0.009403266 |
| ENSMUSG00000017176 | Nt5c3l | 3406 | 18.78164707 | 20.41221675 | 22.14161298 | 21.08168111 | 20.77331996 | 20.26628241 | 21.22541791 | 20.60999284 | 19.10589985 | 23.13236205 | 22.23232801 | 17.60012379 | 21.74022544 | 23.93111373 | 23.03222619 | 21.61856666 | 20.47970064 | 23.07927584 |
| ENSMUSG00000017188 | Ccdc56 | 944 | 244.9450822 | 207.4645181 | 220.3967734 | 209.2236517 | 234.2862174 | 218.4729631 | 309.5873069 | 241.1299982 | 260.9933658 | 275.6617916 | 280.627272 | 250.1061999 | 271.3778034 | 231.3840498 | 232.8498948 | 218.7918817 | 211.6472307 | 220.7356952 |
| ENSMUSG00000017195 | Zpbp2 | 1747 | 0 | 0 | 0.027689485 | 0.078238771 | 0 | 0 | 0.189575703 | 0.174893657 | 0.377209074 | 0 | 0 | 0 | 0.084714225 | 0 | 0.023584176 | 0 | 0 | 0 |
| ENSMUSG00000017204 | Gsdma | 2714 | 0.328890456 | 0.896035843 | 0.855537751 | 0.889732996 | 0.691652551 | 0.931167801 | 0.104596932 | 0.098506565 | 0 | 0.225850826 | 0.017603153 | 0 | 0 | 0.154015028 | 0 | 0.126976959 | 0.324799126 | 0.324298345 |
| ENSMUSG00000017210 | Med24 | 6749 | 8.773108639 | 10.03977283 | 9.855327343 | 10.48396827 | 11.09694373 | 10.79308318 | 8.188058822 | 7.792404521 | 7.494005239 | 10.13017035 | 9.513934538 | 9.833282385 | 10.85462989 | 9.703091305 | 9.425870839 | 8.124485079 | 8.296743188 | 10.30249087 |
| ENSMUSG00000017211 | Gsdma2 | 3163 | 0 | 0 | 0 | 0 | 0 | 0 | 0 | 0 | 0 | 0 | 0 | 0 | 0 | 0 | 0 | 0 | 0 | 0 |
| ENSMUSG00000017221 | Psmd3 | 3525 | 70.16670051 | 85.41331573 | 76.38214751 | 75.49561985 | 77.12031344 | 76.20577836 | 54.85586348 | 52.7651063 | 51.78401074 | 55.06934619 | 65.55673026 | 60.69260559 | 44.93752805 | 50.91062516 | 52.46915869 | 48.6643748 | 52.03678212 | 52.46542321 |
| ENSMUSG00000017264 | Exosc10 | 3692 | 24.55675527 | 25.30047546 | 22.6800057 | 23.34818415 | 24.3658086 | 23.42078009 | 21.88788072 | 19.03413338 | 24.62043345 | 24.16281976 | 23.65455691 | 22.41999665 | 21.21860364 | 17.43540225 | 21.02484596 | 23.32491226 | 23.01446377 | 19.65249506 |
| ENSMUSG00000017286 | Glod4 | 5898 | 29.48265092 | 32.14372347 | 33.55308794 | 32.67602875 | 32.93666394 | 32.40503449 | 31.68617866 | 30.91395881 | 33.80534466 | 33.99998999 | 30.57824148 | 31.6932272 | 31.00600498 | 26.99395245 | 29.40972369 | 31.27910249 | 31.86060253 | 29.06211878 |
| ENSMUSG00000017288 | Vps53 | 5462 | 14.12429412 | 13.27148624 | 11.30959324 | 15.34828297 | 13.46495411 | 12.96427248 | 14.86425431 | 12.41148192 | 14.47786713 | 14.58892137 | 16.29526687 | 13.06998031 | 12.67167337 | 14.23423696 | 12.66521647 | 14.50444283 | 14.48287748 | 14.93564725 |
| ENSMUSG00000017291 | Taok1 | 12401 | 7.379537456 | 6.425646559 | 6.405075143 | 5.68364935 | 5.82194061 | 6.449327634 | 5.41762794 | 6.202685142 | 6.240582462 | 5.330641559 | 5.790320345 | 6.260752089 | 5.091916306 | 5.067240564 | 5.422219112 | 7.336382807 | 6.23370219 | 6.108179982 |
| ENSMUSG00000017299 | Dnttip1 | 2251 | 16.80568932 | 20.05285281 | 17.79355093 | 16.37444907 | 17.83297394 | 17.45682707 | 16.20527563 | 18.66354627 | 17.96764037 | 18.99852859 | 17.95540421 | 20.95256992 | 17.81734729 | 18.2598922 | 18.68804468 | 16.73831972 | 16.77094733 | 17.54621885 |
| ENSMUSG00000017300 | Tnnc2 | 793 | 0 | 0 | 0 | 0 | 0 | 0 | 0 | 0 | 0 | 0 | 0 | 0 | 0 | 0 | 0 | 0 | 0 | 0 |
| ENSMUSG00000017307 | Acot8 | 1308 | 12.08862804 | 12.55600677 | 9.985361771 | 12.40040656 | 10.81864339 | 12.08510857 | 15.7347212 | 13.08118981 | 15.64961646 | 14.09474386 | 13.44127272 | 12.97244757 | 17.57543842 | 17.11471722 | 13.85985061 | 19.35022285 | 15.32465847 | 14.34105917 |
| ENSMUSG00000017309 | Cd300lg | 2729 | 0 | 0 | 0 | 0 | 0 | 0.018157836 | 0.069348009 | 0.055980069 | 0 | 0 | 0 | 0 | 0 | 0 | 0.030195351 | 0 | 0 | 0 |
| ENSMUSG00000017310 | Spint4 | 747 | 5.576314927 | 24.83973505 | 146.7395178 | 74.47126524 | 34.53642716 | 115.2913667 | 699.3029291 | 754.5428041 | 532.7781818 | 210.6948427 | 510.3670263 | 807.1975943 | 0.594360449 | 1.492179515 | 9.707461629 | 1.025183598 | 1.38528784 | 1.39916068 |
| ENSMUSG00000017311 | Pyy | 813 | 0 | 0 | 0.059500037 | 0 | 0.177608574 | 0 | 0 | 0 | 0 | 0 | 0 | 0 | 0 | 0 | 0 | 0 | 0 | 0 |
| ENSMUSG00000017314 | Mpp2 | 6761 | 0.999604051 | 1.128332018 | 1.223468968 | 0.822133916 | 0.754619717 | 1.289939522 | 0.629809363 | 1.417880935 | 1.139162616 | 0.976349424 | 0.939812081 | 0.43754328 | 0.233489329 | 0.817459902 | 0.383922204 | 0.668287483 | 0.425154898 | 0.13831605 |
| ENSMUSG00000017316 | Ppy | 496 | 0.342783678 | 0.402978559 | 0.487636395 | 0.275570833 | 0.291120506 | 0.899142343 | 0 | 0 | 0 | 0 | 0 | 0 | 0 | 0 | 0 | 0 | 0 | 0 |

| ENSMUSG00000017344 | Vtn | 2436 | 0 | 0 | 0 | 0 | 0 | 0 | 0 | 0.015678326 | 0 | 0 | 0 | 0 | 0 | 0 | 0 | 0 | 0 | 0 |
| --- | --- | --- | --- | --- | --- | --- | --- | --- | --- | --- | --- | --- | --- | --- | --- | --- | --- | --- | --- | --- |
| ENSMUSG00000017376 | Nlk | 6137 | 2.6665297 | 3.137502503 | 3.01102959 | 2.457341645 | 2.494044603 | 2.511145534 | 2.451593922 | 3.018301314 | 2.516692751 | 2.358687616 | 2.67795107 | 2.182786218 | 2.041764215 | 2.641190565 | 1.604557904 | 2.788968797 | 2.816548787 | 1.568615558 |
| ENSMUSG00000017386 | Traf4 | 4590 | 27.2162772 | 21.88199914 | 24.3659264 | 25.91718666 | 24.52735718 | 26.90313989 | 21.94524911 | 25.20365727 | 22.34301182 | 23.86298119 | 32.41203065 | 29.08737798 | 20.22716085 | 22.22030848 | 24.77479178 | 27.49582614 | 22.22758221 | 27.98393556 |
| ENSMUSG00000017390 | Aldoc | 3308 | 2.569841356 | 3.363514713 | 3.070871032 | 2.506683818 | 2.589928178 | 2.861116117 | 6.822293787 | 8.301190245 | 4.855725861 | 7.21229016 | 10.29732328 | 5.871780707 | 11.11012043 | 8.929395278 | 11.90710019 | 12.57061658 | 10.18404322 | 14.23452097 |
| ENSMUSG00000017400 | Stac2 | 3127 | 0.380602791 | 0.063919848 | 0.015469629 | 0.18941272 | 0.261670196 | 0.190160794 | 0.378259345 | 0.073282512 | 0.197568832 | 0 | 0.534737303 | 0 | 0.094656701 | 0.089115614 | 0 | 0.159187047 | 0.024513142 | 0 |
| ENSMUSG00000017404 | Rpl19 | 1015 | 146.0670483 | 127.146948 | 119.2896024 | 118.5484907 | 125.2378428 | 116.3390779 | 143.0101467 | 123.9089469 | 128.4288891 | 116.7850916 | 115.8366235 | 118.3951936 | 171.1793228 | 171.3626673 | 132.2914982 | 135.1677304 | 120.4918359 | 149.6356095 |
| ENSMUSG00000017405 | Nek8 | 4506 | 21.07336068 | 23.89420198 | 21.93233966 | 22.719855 | 25.82844905 | 23.0058408 | 15.32989597 | 18.15537638 | 16.77258243 | 14.44029527 | 17.17608361 | 17.01968939 | 17.80153073 | 24.36613906 | 22.00890924 | 25.16167078 | 21.31506881 | 23.13410059 |
| ENSMUSG00000017412 | Cacnb4 | 8920 | 0.028590926 | 0.093365735 | 0.086768664 | 0.117478029 | 0.188858445 | 0.055552392 | 0.07956168 | 0.171266378 | 0.180075573 | 0.116290972 | 0.133898426 | 0.250294451 | 0.215688876 | 0.33323112 | 0.193998357 | 0.193170105 | 0.137493442 | 0.185008206 |
| ENSMUSG00000017417 | Plxdc1 | 5773 | 0.331323908 | 0.236589063 | 0.242998853 | 0.36303621 | 0.266797429 | 0.137336521 | 0.02458653 | 0.198470825 | 0.021403005 | 0.03266981 | 0 | 0.10635238 | 0.051271696 | 0.329847148 | 0.171286565 | 0 | 0.086305536 | 0 |
| ENSMUSG00000017418 | Arl5b | 7223 | 2.789347008 | 3.5605087 | 3.810679603 | 4.194669041 | 4.358061481 | 4.191709846 | 3.766411516 | 4.520906003 | 4.886729061 | 4.432414165 | 4.008254858 | 4.34284956 | 3.435408333 | 3.832296287 | 3.7647829 | 4.622651205 | 5.926965754 | 2.734081312 |
| ENSMUSG00000017421 | Zfp207 | 5191 | 53.77220015 | 54.41341362 | 55.2787097 | 56.29522989 | 57.47828835 | 51.76738089 | 46.11869686 | 48.96367521 | 54.38111713 | 47.0144558 | 51.5943782 | 48.68682724 | 50.65288366 | 44.90519642 | 48.16240438 | 48.91992069 | 52.69404373 | 47.32627059 |
| ENSMUSG00000017428 | Psmd11 | 6518 | 20.93957045 | 19.33456257 | 18.59835334 | 17.6288668 | 18.45377067 | 18.23826448 | 14.6844967 | 15.43985584 | 14.79251816 | 15.91462829 | 15.83214334 | 16.95535623 | 14.67545069 | 14.27240059 | 14.86745312 | 14.25176642 | 15.22350149 | 12.54963819 |
| ENSMUSG00000017446 | C1qtnf1 | 3193 | 2.276349859 | 3.067331945 | 3.272371612 | 3.096381654 | 2.728430374 | 3.165912199 | 1.881838467 | 4.736671266 | 3.250548692 | 4.119964362 | 3.935112458 | 4.003065059 | 0.633450864 | 0.27636632 | 1.406504727 | 0.647570561 | 1.908512749 | 0.826945544 |
| ENSMUSG00000017453 | Pipox | 2656 | 0 | 0.100340043 | 0 | 0 | 0.018121959 | 0 | 1.050997009 | 1.064095549 | 0.434195196 | 0.656843381 | 0.035975119 | 0.52537333 | 2.043114044 | 3.427349822 | 2.156256125 | 0.216249665 | 0.461762613 | 2.050414746 |
| ENSMUSG00000017466 | Timp2 | 4085 | 0.104051839 | 0.252802869 | 0.272360146 | 0.267678106 | 0.212086812 | 0.351781952 | 0.208476921 | 0.308531035 | 0.383130379 | 0.115423998 | 0.046780865 | 0.259607733 | 0.156992638 | 0.375190914 | 0.282409685 | 0.112481588 | 0.234555061 | 0 |
| ENSMUSG00000017478 | Zc3h18 | 6400 | 7.332142865 | 9.468149154 | 8.004307601 | 8.286414939 | 8.204988863 | 8.617530074 | 8.043155418 | 9.476489847 | 8.250173939 | 7.735662253 | 7.584274658 | 6.567061551 | 6.598143936 | 7.271402432 | 7.190959052 | 7.741882184 | 8.7731289 | 7.31449216 |
| ENSMUSG00000017485 | Top2b | 6319 | 15.7334399 | 13.76483096 | 16.85686245 | 15.00435722 | 13.71824569 | 16.10718702 | 10.8866332 | 11.76173684 | 11.3085306 | 12.58794689 | 9.851364282 | 12.17185995 | 10.09434547 | 10.73087001 | 10.04120843 | 9.901385107 | 11.73018804 | 9.297313812 |
| ENSMUSG00000017491 | Rarb | 3479 | 17.42235729 | 16.95807291 | 15.67029857 | 19.34281762 | 18.70490394 | 17.5620927 | 10.73000968 | 12.3063188 | 11.30587024 | 13.17346466 | 14.94083209 | 13.8295778 | 12.70520203 | 11.00026675 | 16.15375747 | 17.37880687 | 16.87723106 | 14.35707643 |
| ENSMUSG00000017493 | Igfbp4 | 4493 | 0.151364971 | 0.170531175 | 0.376824741 | 0.304213517 | 0.128551766 | 0.573501479 | 0.263257728 | 0.059502964 | 0.320838649 | 0.041977034 | 0.021266396 | 0.124228049 | 0.724662039 | 0.713252177 | 1.109590087 | 0.392024914 | 0.119423137 | 0 |
| ENSMUSG00000017499 | Cdc6 | 5347 | 0.047696102 | 0.137064461 | 0.018093709 | 0.170417222 | 0.090016689 | 0 | 0 | 0.142855442 | 0.084730068 | 0.052908962 | 0.062544363 | 0 | 0.295234964 | 0 | 0.069349916 | 0.078772523 | 0 | 0.246908278 |
| ENSMUSG00000017548 | Suz12 | 4928 | 7.857592403 | 8.970416118 | 10.90563966 | 7.812313812 | 11.06604495 | 11.02065666 | 8.016657245 | 10.25335803 | 9.736666165 | 9.127794421 | 8.172542613 | 10.20493344 | 8.178607227 | 7.501926453 | 7.608241855 | 6.542348099 | 10.1182046 | 7.668680259 |
| ENSMUSG00000017550 | Atad5 | 7603 | 0.743547075 | 1.038426401 | 1.107062249 | 1.414232075 | 1.33576692 | 1.21225943 | 0.678295673 | 1.311089968 | 1.316365031 | 1.0728754 | 1.319576651 | 0.763491899 | 0.986249037 | 0.781907561 | 1.240978074 | 1.163373709 | 1.214867494 | 1.787090538 |
| ENSMUSG00000017561 | Crlf3 | 2722 | 3.451008046 | 2.839306194 | 2.932267637 | 2.845473011 | 3.112130259 | 1.765839514 | 4.501830949 | 3.156976687 | 2.239384399 | 3.533704432 | 2.19392719 | 2.624689497 | 3.588434835 | 2.183998231 | 2.028291142 | 3.657442294 | 2.421793926 | 1.798607825 |
| ENSMUSG00000017588 | Krt27 | 1542 | 0 | 0 | 0 | 0 | 0 | 0 | 0 | 0 | 0 | 0 | 0 | 0 | 0 | 0 | 0 | 0 | 0 | 0 |
| ENSMUSG00000017607 | Tns4 | 5301 | 0 | 0.006284266 | 0.374139737 | 0.154706432 | 0.009079782 | 0.009347809 | 0 | 0.064842788 | 0.007769575 | 0.071157447 | 0 | 0 | 0.018612306 | 0.280364232 | 0.70728949 | 0.216698401 | 0.086760151 | 0.020754238 |
| ENSMUSG00000017615 | Tnfaip1 | 6409 | 34.6328646 | 35.21010227 | 30.22119138 | 32.93560905 | 34.93676219 | 32.94495206 | 25.21768002 | 30.71362802 | 21.90102133 | 24.97685092 | 27.71529031 | 25.9265451 | 25.15473641 | 20.97194397 | 24.6026454 | 24.0772582 | 22.86182447 | 20.67669498 |
| ENSMUSG00000017631 | Abr | 12142 | 35.35680101 | 32.06455236 | 40.41751486 | 41.68861823 | 40.06897417 | 34.7791464 | 36.60478553 | 28.34705407 | 33.39493063 | 34.30095222 | 36.84833521 | 33.06556259 | 28.01380083 | 24.05208545 | 31.20826137 | 31.2171572 | 33.58522476 | 29.2125449 |
| ENSMUSG00000017639 | Rab11fip4 | 7435 | 5.431058134 | 5.152633269 | 6.681858194 | 5.827646693 | 4.253217734 | 5.671738571 | 4.906264352 | 4.731029269 | 4.309765873 | 6.500265076 | 5.648175992 | 5.930648748 | 4.034136611 | 5.084799884 | 4.51637768 | 4.470241725 | 5.654868554 | 4.735148393 |
| ENSMUSG00000017652 | Cd40 | 3519 | 1.099167667 | 0.549061627 | 0.412391563 | 0.893353811 | 0.848019115 | 0.478770373 | 0.658801156 | 0.596925869 | 0.526681787 | 0.723540203 | 0.543051531 | 0.999257386 | 0.448599418 | 0.488329421 | 0.526874116 | 2.078290996 | 0.446541112 | 0.046896084 |
| ENSMUSG00000017664 | Slc35c2 | 3025 | 6.323084037 | 10.25266265 | 7.835712355 | 11.73291026 | 7.430614332 | 10.15626275 | 9.884830771 | 9.241930103 | 11.9543011 | 9.211922521 | 7.059638493 | 8.801345803 | 7.827874477 | 8.459732943 | 8.853226886 | 9.873280586 | 9.160301706 | 7.764921842 |
| ENSMUSG00000017667 | Zfp334 | 8200 | 2.690266629 | 2.80722072 | 2.589753637 | 3.428190775 | 2.195285299 | 3.118196406 | 2.948387675 | 2.026060371 | 1.185368015 | 2.725540652 | 2.48779357 | 2.647840579 | 3.152429836 | 1.438633926 | 1.999782835 | 2.124661752 | 1.874249397 | 1.811275465 |
| ENSMUSG00000017670 | Elmo2 | 8032 | 5.67829356 | 6.490871446 | 6.636906182 | 6.648100593 | 6.699637781 | 7.181197944 | 5.160097951 | 6.191049294 | 5.850823517 | 7.032687065 | 7.363719945 | 6.8379746 | 4.864402202 | 5.689863296 | 5.411807951 | 5.630130396 | 6.627891733 | 6.677524783 |
| ENSMUSG00000017677 | Wsb1 | 4976 | 19.39896599 | 20.21803467 | 16.19580016 | 20.49148256 | 20.60309434 | 18.94077558 | 18.91176013 | 25.04457577 | 21.67754919 | 21.08326261 | 28.39998537 | 23.66781512 | 21.6521116 | 20.68329937 | 23.92108018 | 34.32765266 | 30.1850397 | 25.10564336 |
| ENSMUSG00000017679 | Ttpal | 6037 | 6.949247762 | 8.183372888 | 7.027262899 | 5.833806074 | 7.781473828 | 8.33950262 | 5.93269802 | 8.477359487 | 6.706368228 | 8.435110074 | 7.185632317 | 9.541454992 | 4.739524933 | 6.246910545 | 5.999033896 | 7.719010967 | 8.475336486 | 8.665506128 |
| ENSMUSG00000017686 | Rhot1 | 7325 | 12.18578425 | 11.82437201 | 12.55400426 | 12.2781572 | 11.99848363 | 11.27705213 | 9.953425014 | 11.71581272 | 11.13863314 | 13.76864905 | 10.99639317 | 11.84892223 | 10.21658956 | 9.104943275 | 10.61959557 | 12.70777701 | 13.54631845 | 10.01053096 |
| ENSMUSG00000017688 | Hnf4g | 4342 | 0.009789308 | 0.352923338 | 0.044563363 | 0.22035512 | 0.199533539 | 0.057062107 | 0.021793035 | 0 | 0 | 0.032577639 | 0 | 0 | 0 | 0.042785893 | 0 | 0.008818657 | 0.008826876 | 0.354733991 |
| ENSMUSG00000017692 | Rhbdl3 | 4725 | 0.278869938 | 1.163307417 | 1.054491773 | 0.752118827 | 0.937171847 | 0.629241061 | 0.39051735 | 0.533481176 | 0.034866892 | 0.169642742 | 0.202222046 | 0.43707503 | 0.020881235 | 0.226077039 | 0.04359953 | 0.235011135 | 0.056779699 | 0.011642139 |
| ENSMUSG00000017697 | Ada | 1990 | 2.734001272 | 2.427321438 | 1.312648562 | 1.579754804 | 2.587997906 | 1.494052269 | 1.450288148 | 1.055568911 | 0.186270674 | 1.208384857 | 1.008315707 | 1.318259368 | 2.429411002 | 1.120259394 | 1.49070956 | 1.539320901 | 1.386680089 | 1.713851566 |
| ENSMUSG00000017707 | Serinc3 | 3987 | 1314.654071 | 1292.944696 | 1361.386687 | 1335.440262 | 1348.756218 | 1301.619935 | 881.8409322 | 966.6978884 | 947.8373632 | 922.0818493 | 940.9761574 | 996.5202683 | 735.2769461 | 689.3574626 | 809.6565594 | 795.3056156 | 834.4597079 | 759.9038302 |
| ENSMUSG00000017713 | Tha1 | 2684 | 7.569848786 | 6.801589431 | 6.668482202 | 5.856393553 | 5.057079908 | 5.187897964 | 14.87775746 | 10.72916223 | 12.85927728 | 12.45523421 | 12.78033539 | 10.70978623 | 16.06411911 | 18.41151601 | 17.23895204 | 16.40618424 | 10.92385139 | 18.11775348 |
| ENSMUSG00000017715 | Pgs1 | 5450 | 5.311197225 | 5.427862398 | 5.87583066 | 7.156009899 | 7.180046595 | 6.255464349 | 6.675862403 | 5.725356471 | 6.325341962 | 5.934932557 | 6.653429997 | 5.089978772 | 8.037934411 | 7.41401028 | 6.577129106 | 8.142901647 | 6.32911325 | 10.62836502 |

| ENSMUSG00000017716 | Birc5 | 3553 | 0.346932199 | 0.159391838 | 0.068074205 | 0.179525646 | 0.135468403 | 0.041840192 | 0.545966742 | 0.096744053 | 0.637562164 | 0.291954819 | 0.309266548 | 0.12567557 | 0.2221533 | 0.692805131 | 0.834931614 | 1.023813032 | 0.873748958 | 0.603815131 |
| --- | --- | --- | --- | --- | --- | --- | --- | --- | --- | --- | --- | --- | --- | --- | --- | --- | --- | --- | --- | --- |
| ENSMUSG00000017718 | Afmid | 3560 | 0.585043153 | 0.299441746 | 0.557110883 | 0.550315985 | 0.365045488 | 0.835158431 | 0.292381724 | 1.062092089 | 0.439631348 | 0.582761501 | 0.536797286 | 0.501713821 | 0.582005766 | 0.874088162 | 0.91430419 | 0.53778943 | 0.904328354 | 0.880763787 |
| ENSMUSG00000017720 | Trp53tg5 | 929 | 0 | 0 | 0 | 0 | 0 | 0.160019592 | 0 | 0 | 0 | 0.152262766 | 0 | 0 | 0.053102171 | 0 | 0 | 0 | 0 | 0 |
| ENSMUSG00000017721 | Pigt | 3953 | 18.68808397 | 9.977856503 | 12.22493216 | 11.98671543 | 9.692135401 | 11.26939223 | 13.90774928 | 9.53602357 | 10.70036724 | 13.00133229 | 8.846766913 | 11.15466062 | 19.03141256 | 9.927967074 | 11.23584044 | 19.49885978 | 10.7716964 | 9.796714175 |
| ENSMUSG00000017723 | Wfdc2 | 1133 | 30.27509007 | 21.16971213 | 19.89590922 | 25.93722294 | 21.87814712 | 26.28525409 | 36.28836539 | 34.01247487 | 38.49648737 | 33.0845623 | 29.76974465 | 40.54394555 | 13.6718641 | 5.615922306 | 8.109397144 | 13.07896299 | 9.505462868 | 15.34234574 |
| ENSMUSG00000017724 | Etv4 | 3747 | 2.83594716 | 2.533807006 | 2.155959319 | 3.818032538 | 3.339818562 | 2.036594868 | 4.5456537 | 8.256163851 | 8.210922678 | 7.915076688 | 5.380579791 | 8.177955367 | 10.07176854 | 7.11473343 | 9.841311055 | 15.40003879 | 8.060080587 | 9.483822509 |
| ENSMUSG00000017733 | Spinlw1 | 774 | 13.28973204 | 12.43853544 | 8.562239869 | 9.065117348 | 3.979900665 | 10.49954562 | 79.4657398 | 42.23862592 | 47.83808485 | 33.01767592 | 16.9125822 | 34.2537981 | 107.6506567 | 72.96642153 | 31.9924226 | 17.7106427 | 12.57736358 | 43.92199994 |
| ENSMUSG00000017734 | Dbndd2 | 1831 | 0.603568857 | 0.418457983 | 0.343449424 | 0.174182037 | 0.131436165 | 0.37888491 | 0.439276648 | 0.542327942 | 0.427386018 | 0.463524117 | 0.782768298 | 0.64015779 | 0.215540873 | 0.735597233 | 1.057604111 | 0.627372049 | 0.732616271 | 0.300432041 |
| ENSMUSG00000017737 | Mmp9 | 3524 | 0 | 0 | 0 | 0 | 0.054633285 | 1.335842704 | 0.214813525 | 0 | 0.023374867 | 0 | 0.027114051 | 0 | 0 | 0 | 0 | 0 | 0 | 0 |
| ENSMUSG00000017740 | Slc12a5 | 7038 | 0.084551359 | 0 | 0.027492771 | 0.103577253 | 0.020516592 | 0.014081482 | 0.147894137 | 0.032559593 | 0.128744437 | 0.140688373 | 0.08145773 | 0.158612284 | 0.042056195 | 0.092386647 | 0.046833255 | 0.065286628 | 0.010891247 | 0.046896084 |
| ENSMUSG00000017747 | Ghdc | 2897 | 5.634099964 | 5.232090739 | 5.142922799 | 4.324917912 | 4.336359019 | 4.618307928 | 5.389431842 | 6.222579888 | 6.980524418 | 5.110569839 | 5.837878944 | 4.546943863 | 6.607105248 | 6.957795625 | 5.034639553 | 4.996151052 | 5.516764173 | 6.493998792 |
| ENSMUSG00000017754 | Pltp | 2547 | 0.534026554 | 0.248506082 | 1.55737318 | 1.001734779 | 0.321257441 | 0.622570661 | 0.798761364 | 1.409535071 | 0.662994561 | 2.795349899 | 0.150058762 | 0.876571065 | 2.730977753 | 2.060534698 | 4.998539761 | 1.68376444 | 0.752381172 | 1.403844498 |
| ENSMUSG00000017756 | Slc12a7 | 5718 | 3.501213345 | 6.059008392 | 5.152060158 | 5.968034664 | 5.824989713 | 7.84281635 | 2.159602868 | 2.110667919 | 2.989227709 | 4.081776515 | 2.673659796 | 3.318874655 | 2.795302763 | 3.240851848 | 3.04075841 | 4.178618226 | 4.242837695 | 3.424841202 |
| ENSMUSG00000017760 | Ctsa | 4871 | 40.69885875 | 41.73173486 | 41.07430128 | 40.73462289 | 41.53119999 | 42.48249136 | 46.4093574 | 47.61701084 | 47.41818526 | 48.74788372 | 54.56212154 | 49.62792746 | 55.80350322 | 49.80034252 | 49.42325828 | 58.65042534 | 52.71734536 | 54.02659958 |
| ENSMUSG00000017764 | Zswim1 | 2965 | 9.905928039 | 8.628769902 | 9.00579722 | 10.12638388 | 7.98681499 | 8.389703965 | 8.712554029 | 9.738096532 | 10.15424723 | 10.84545687 | 9.957815966 | 8.433530131 | 7.720070697 | 9.304481588 | 10.01899555 | 8.407145166 | 8.89325203 | 9.814440963 |
| ENSMUSG00000017765 | Slc12a4 | 4805 | 1.096907768 | 1.497520323 | 1.087271858 | 1.469711108 | 0.871483321 | 1.000336141 | 0.374168949 | 0.461011309 | 0.128573931 | 0.343449451 | 0.049713797 | 0.32525256 | 0.164268611 | 0.396297104 | 0.145770333 | 0.382507629 | 0.271195441 | 0.228966105 |
| ENSMUSG00000017767 | 1700020C07R | 1134 | 0 | 0 | 0 | 0 | 0 | 0 | 0 | 0 | 0 | 0 | 0 | 0.049220161 | 0 | 0 | 0 | 0 | 0 | 0 |
| ENSMUSG00000017774 | Myo1c | 7137 | 14.6686631 | 19.86544456 | 17.24963357 | 20.77282307 | 18.9101743 | 20.78756961 | 10.79898472 | 13.39436503 | 10.59527013 | 14.0916982 | 14.80039697 | 12.34873634 | 10.36820456 | 11.14736187 | 12.60235344 | 12.5918531 | 13.66149633 | 9.156623061 |
| ENSMUSG00000017776 | Crk | 3453 | 40.08017757 | 44.80309318 | 43.0640696 | 44.14921933 | 42.91867737 | 48.56253994 | 42.8321559 | 43.03696431 | 27.55309916 | 39.39466519 | 42.9739418 | 43.91866645 | 34.77378704 | 36.70602794 | 35.03265801 | 38.90050703 | 44.54197205 | 37.23031637 |
| ENSMUSG00000017778 | Cox7c | 1215 | 138.6053559 | 109.2882275 | 120.6750375 | 108.0340483 | 120.9438378 | 118.763424 | 174.648037 | 133.4062188 | 133.9324476 | 135.3593852 | 133.8878466 | 132.2119152 | 176.0926134 | 160.0123044 | 131.3363177 | 136.4595309 | 133.8739128 | 150.8109749 |
| ENSMUSG00000017781 | Pitpna | 4289 | 53.04971025 | 52.02372709 | 49.87357222 | 51.33982916 | 52.38512933 | 49.02127502 | 47.42299061 | 46.03747271 | 48.465457 | 47.73335359 | 48.32076705 | 41.46157636 | 49.3549212 | 45.31790761 | 42.12376372 | 50.15542839 | 45.46612232 | 46.32615842 |
| ENSMUSG00000017801 | Mlx | 2068 | 17.98454015 | 16.93029585 | 18.68977309 | 17.77938239 | 14.52336575 | 14.71246539 | 17.77657231 | 16.12280817 | 18.58173076 | 20.01852875 | 24.09539734 | 17.78651917 | 16.48373058 | 17.67480502 | 18.36935906 | 19.27491407 | 15.66040662 | 15.7472878 |
| ENSMUSG00000017802 | Fam134c | 5685 | 5.749600768 | 6.123478357 | 6.160498853 | 5.105080957 | 7.331969375 | 5.569779553 | 5.367929272 | 5.838029497 | 5.679899447 | 5.158792863 | 6.109480178 | 5.164298772 | 5.666445379 | 4.746528544 | 5.935633118 | 5.725068829 | 5.265230938 | 6.918471646 |
| ENSMUSG00000017817 | Jph2 | 5924 | 0 | 0.011246757 | 0 | 0.007690926 | 0.016249805 | 0 | 0 | 0 | 0 | 0 | 0 | 0 | 0 | 0 | 0.020865069 | 0 | 0 | 0 |
| ENSMUSG00000017830 | Dhx58 | 3832 | 2.273893812 | 2.434136321 | 3.484106049 | 2.056905185 | 1.519823268 | 2.870748135 | 1.691502352 | 1.784039674 | 2.009884772 | 2.411674801 | 2.393734872 | 3.816206576 | 3.076807993 | 2.121011298 | 2.731000834 | 3.337438119 | 2.510412454 | 2.856683778 |
| ENSMUSG00000017831 | Rab5a | 2351 | 69.76923619 | 78.54248093 | 74.99851899 | 83.7191457 | 73.12940508 | 82.62301813 | 83.51663869 | 81.51827957 | 75.33050522 | 88.86634034 | 79.55719372 | 79.6043882 | 70.7559443 | 80.97588865 | 79.31869075 | 86.20679922 | 86.5153797 | 72.8385152 |
| ENSMUSG00000017832 | Hspb9 | 1376 | 0.308903896 | 0 | 0 | 0.099333672 | 0.034979596 | 0 | 0.068768429 | 0 | 0 | 0 | 0 | 0 | 0 | 0 | 0.089828974 | 0.055654953 | 0 | 0 |
| ENSMUSG00000017837 | Nkiras2 | 3570 | 6.107886639 | 5.048256519 | 5.33870335 | 4.977256944 | 4.718816046 | 5.774212092 | 5.950530207 | 6.001663235 | 5.087746065 | 5.56034904 | 5.165583746 | 6.441471425 | 5.195742548 | 5.190809767 | 5.435835525 | 5.234122244 | 6.623900613 | 5.531728099 |
| ENSMUSG00000017843 | Ppp2r5c | 4570 | 36.60839668 | 38.79457834 | 36.14783505 | 37.8744874 | 38.34755669 | 35.17485071 | 35.37580392 | 39.39583915 | 38.58194179 | 41.45547608 | 41.62798351 | 37.019097 | 35.59022566 | 38.85246085 | 36.27900676 | 39.65633366 | 41.14416031 | 37.21841531 |
| ENSMUSG00000017858 | Ift52 | 2708 | 36.0854504 | 36.99109044 | 39.62056511 | 35.44945362 | 41.59110095 | 36.139826 | 39.67765652 | 37.00770455 | 35.55911147 | 38.75829596 | 41.97068915 | 36.48217237 | 34.01133292 | 39.20649327 | 41.56671001 | 38.24818797 | 43.12416051 | 38.51450013 |
| ENSMUSG00000017861 | Mybl2 | 4617 | 0.248568281 | 0.194812247 | 0.11524991 | 0.088812952 | 0.187648825 | 0.214653383 | 0.256187345 | 0.363973512 | 0.428189897 | 0.102124114 | 0.227647625 | 0.084624136 | 0.309860429 | 0.120712378 | 0.526508945 | 0.157574516 | 0.423357406 | 0.154888106 |
| ENSMUSG00000017868 | Sgk2 | 3511 | 0 | 0 | 0.013777707 | 0 | 0 | 0.014113567 | 0 | 0 | 0 | 0.228300187 | 0 | 0.206665797 | 0 | 0.224878805 | 0 | 0 | 0.043664252 | 0.360355869 |
| ENSMUSG00000017897 | Eya2 | 2930 | 34.19273034 | 41.97652063 | 31.81426382 | 40.35184643 | 39.29404826 | 34.24719641 | 10.88353092 | 9.919596666 | 10.40205518 | 9.349678701 | 12.6367211 | 10.03919938 | 14.63120686 | 16.3267611 | 15.38378913 | 24.05904718 | 20.1703583 | 11.11446798 |
| ENSMUSG00000017914 | BC034902 | 2314 | 0 | 0 | 0 | 0 | 0 | 0 | 0 | 0 | 0 | 0 | 0 | 0 | 0 | 0 | 0 | 0 | 0 | 0 |
| ENSMUSG00000017922 | Prl3c1 | 924 | 0 | 0 | 0 | 0 | 0 | 0 | 0 | 0 | 0 | 0 | 0 | 0 | 0 | 0 | 0 | 0 | 0 | 0 |
| ENSMUSG00000017929 | B4galt5 | 4581 | 3.562974808 | 3.388737984 | 4.181601838 | 3.112989932 | 3.39371564 | 3.645333162 | 2.42708569 | 2.384419797 | 2.517403277 | 2.470239856 | 2.283937111 | 2.717068927 | 3.769083398 | 2.423081617 | 2.338442378 | 6.444457172 | 4.066051145 | 3.626446241 |
| ENSMUSG00000017943 | Gdap1l1 | 2821 | 0.015067414 | 0.059044478 | 0.034295307 | 0 | 0 | 0 | 0.117401188 | 0.013538604 | 0 | 0.083570903 | 0 | 0 | 0 | 0.36220132 | 0.116842413 | 0.665097399 | 0.013586068 | 0.058499582 |
| ENSMUSG00000017950 | Hnf4a | 5054 | 0 | 0 | 0 | 0 | 0 | 0 | 0 | 0 | 0 | 0 | 0 | 0 | 0 | 0.183791402 | 0 | 0 | 0 | 0 |
| ENSMUSG00000017969 | Ptgis | 2556 | 0.232813953 | 0.013033214 | 0.018925481 | 0.017825135 | 0.018830956 | 0.019386828 | 0 | 0.119538036 | 0.225591244 | 0.073788268 | 0 | 0.065511341 | 0 | 0.018170613 | 0.096717267 | 0 | 0.149946389 | 0.04304312 |
| ENSMUSG00000017978 | Cadps2 | 10481 | 3.260582151 | 3.864944125 | 3.743052487 | 3.603673863 | 3.953972548 | 3.947765723 | 4.315515796 | 4.044801705 | 3.281246127 | 4.70562309 | 4.453404678 | 4.148497391 | 2.602857576 | 5.224461314 | 5.153634174 | 3.182055056 | 5.218168656 | 3.721157968 |
| ENSMUSG00000017999 | Ddx27 | 4916 | 7.894065442 | 9.84614225 | 8.304975513 | 10.12055745 | 8.860738592 | 9.293657518 | 7.025682591 | 8.949887626 | 8.185359215 | 8.565007732 | 9.290655066 | 7.504913135 | 7.064619561 | 11.2047778 | 10.64401464 | 8.334204622 | 7.67149637 | 8.526635338 |

| ENSMUSG00000018001 | Cyth3 | 4804 | 8.626674985 | 9.347581475 | 8.750332614 | 9.958179965 | 9.738599043 | 8.674822843 | 9.366019496 | 7.846773766 | 9.14779607 | 8.921729655 | 7.389007941 | 10.79366165 | 6.11001057 | 6.342073549 | 7.984731169 | 6.551806679 | 8.640170597 | 11.59954728 |
| --- | --- | --- | --- | --- | --- | --- | --- | --- | --- | --- | --- | --- | --- | --- | --- | --- | --- | --- | --- | --- |
| ENSMUSG00000018008 | Cyth4 | 2778 | 0 | 0 | 0.034826156 | 0 | 0 | 0 | 0.80046649 | 0.563674766 | 0.311345152 | 0.067891581 | 1.100646992 | 0.020092031 | 0.142064557 | 0.08359267 | 0.326290219 | 0.027567032 | 0 | 0.27722372 |
| ENSMUSG00000018012 | Rac3 | 1334 | 64.45874895 | 50.61861813 | 60.3762579 | 52.08440226 | 51.19880036 | 55.12463016 | 75.61516602 | 80.16396307 | 91.9441109 | 78.07788868 | 82.94363106 | 88.53518893 | 54.39823862 | 43.27586254 | 54.66773158 | 46.95909573 | 49.47367561 | 40.45272391 |
| ENSMUSG00000018040 | Rrp7a | 5820 | 7.274081671 | 6.525206084 | 6.624347714 | 7.131634259 | 6.434130002 | 6.675144867 | 5.804338961 | 6.41133628 | 5.944445574 | 6.861966608 | 7.264748841 | 7.317414184 | 7.72188602 | 6.878832187 | 8.771259063 | 7.710754618 | 6.71697987 | 7.31564409 |
| ENSMUSG00000018042 | Cyb5r3 | 5513 | 87.98640828 | 78.39074491 | 86.87580701 | 94.20466976 | 90.44925253 | 83.19610049 | 107.6013728 | 76.28781703 | 90.5610274 | 108.464578 | 74.82114847 | 73.86741778 | 91.84523833 | 105.7523361 | 97.00638409 | 88.27051142 | 87.08057228 | 90.18180783 |
| ENSMUSG00000018068 | Ints2 | 9573 | 2.748428284 | 3.041415392 | 3.779734744 | 3.307732771 | 3.328458526 | 3.58717689 | 2.999978701 | 2.968259416 | 3.523634849 | 3.167022061 | 2.140964921 | 2.670382687 | 1.571736631 | 3.536795122 | 2.737301739 | 2.0319261 | 3.591213655 | 1.90776386 |
| ENSMUSG00000018076 | Med13l | 9289 | 3.381561533 | 4.17083604 | 4.025485947 | 4.080825588 | 3.155597106 | 4.422350752 | 4.064540616 | 3.412605662 | 3.759948904 | 3.106494821 | 4.04253605 | 3.202685774 | 3.97778082 | 2.599948912 | 3.158091053 | 4.723978478 | 3.601986995 | 2.641195134 |
| ENSMUSG00000018102 | Hist1h2bc | 734 | 53.85533202 | 43.75153955 | 57.27056932 | 52.82349963 | 44.98433193 | 58.26159275 | 107.9038481 | 94.54440759 | 102.5735025 | 85.95046452 | 80.25411962 | 108.9698154 | 145.5762028 | 162.6808565 | 142.4653254 | 139.2338299 | 130.0170018 | 185.0374448 |
| ENSMUSG00000018126 | Baiap2l2 | 3171 | 0 | 0 | 0.045764929 | 0 | 0.030357568 | 0 | 0 | 0.084309939 | 0 | 0 | 0 | 0 | 0.062228846 | 0 | 0 | 0.108677221 | 0 | 0.052042674 |
| ENSMUSG00000018143 | Mafk | 4075 | 12.08920221 | 14.05272765 | 11.78770936 | 16.83802031 | 14.79982831 | 13.29107676 | 10.05466993 | 15.08951359 | 8.833623258 | 10.84176907 | 13.75215369 | 10.57415743 | 8.17154458 | 10.90723721 | 9.018843645 | 17.18614011 | 18.94212564 | 20.0597626 |
| ENSMUSG00000018160 | Med1 | 8223 | 5.644612942 | 6.259080818 | 7.100431854 | 7.258296009 | 6.128435374 | 6.194844961 | 7.013760867 | 7.617115401 | 6.145671268 | 6.571166963 | 7.355356614 | 6.930292047 | 6.581188526 | 5.376963541 | 6.12286286 | 6.551731073 | 6.254881484 | 4.78310973 |
| ENSMUSG00000018166 | Erbb3 | 5988 | 24.19132263 | 30.89843261 | 30.56063215 | 28.89793694 | 30.83401122 | 32.77034485 | 22.40794212 | 30.07300891 | 25.68310786 | 28.72507774 | 27.66128608 | 28.36457269 | 29.05705949 | 26.44086405 | 26.79339657 | 27.82911213 | 31.11931455 | 27.89957534 |
| ENSMUSG00000018167 | Stard3 | 3609 | 34.80265868 | 32.6113758 | 35.45247653 | 33.79521077 | 31.87456292 | 36.92083696 | 39.48622581 | 38.20298495 | 40.14800844 | 37.84859721 | 40.37506882 | 40.22625059 | 47.8829887 | 41.3994539 | 45.14019174 | 46.35402154 | 41.36351522 | 48.79028834 |
| ENSMUSG00000018168 | Ikzf3 | 9172 | 0.074147712 | 0.036320207 | 0.073836614 | 0.12418514 | 0.115449446 | 0.18368872 | 0.505521428 | 0.129084657 | 0.300860942 | 0.113095887 | 0.468790478 | 0.206904986 | 0.155977497 | 0.045573134 | 0.076365727 | 0.07097038 | 0.062679291 | 0.101957568 |
| ENSMUSG00000018169 | Mfng | 2823 | 0.030113479 | 0.023601059 | 0.205626059 | 0.161392293 | 0.102299519 | 0.035106435 | 0.234636027 | 0.229993213 | 0.277202905 | 0.233832747 | 0.033846942 | 0.434978596 | 0.104849984 | 0.477108939 | 0.116759634 | 0 | 0.176493751 | 0.136402319 |
| ENSMUSG00000018171 | Vmp1 | 4225 | 87.75684035 | 82.04827864 | 90.53006024 | 79.91784605 | 88.80197515 | 80.49240486 | 69.37326532 | 83.46282874 | 81.40795104 | 65.20746962 | 76.28162601 | 76.30562535 | 68.06053155 | 64.01039547 | 71.42253144 | 79.76228065 | 78.11307518 | 83.71800147 |
| ENSMUSG00000018189 | Uchl5 | 1840 | 30.7469507 | 31.44809634 | 25.05433393 | 30.38228337 | 30.10861095 | 28.95064598 | 35.2788019 | 35.20343178 | 32.79252478 | 33.2617461 | 32.06634441 | 31.6086524 | 37.85688434 | 31.82934467 | 31.79685292 | 34.44073655 | 36.30583451 | 33.90235164 |
| ENSMUSG00000018196 | Glrx2 | 2340 | 22.32429972 | 20.14433561 | 19.18403255 | 18.59435784 | 20.07553739 | 20.05403363 | 24.88970417 | 19.01459351 | 22.68778582 | 22.82980632 | 22.25414733 | 21.15747552 | 21.60906633 | 22.30904027 | 19.01610272 | 23.72708578 | 23.12680822 | 24.26042655 |
| ENSMUSG00000018199 | Trove2 | 11579 | 4.772150344 | 4.695279676 | 5.539623564 | 5.319849032 | 5.179409007 | 4.801638058 | 4.637696743 | 4.624384504 | 4.649000441 | 3.884771648 | 5.079106476 | 4.266073183 | 4.124129538 | 3.914796553 | 5.212909524 | 4.199764349 | 5.484642367 | 4.142666989 |
| ENSMUSG00000018209 | Stk4 | 5846 | 9.183037517 | 9.715785947 | 10.87287357 | 10.20174599 | 10.25870285 | 9.951233189 | 9.323333241 | 11.49822584 | 7.939993716 | 9.541444056 | 9.463462525 | 9.66223923 | 5.468197468 | 6.768792757 | 7.646884732 | 9.602126317 | 9.198049017 | 6.32331846 |
| ENSMUSG00000018211 | Wfdc15b | 706 | 37.38769733 | 39.44699655 | 28.57190107 | 25.10374822 | 29.79270629 | 29.82990336 | 362.4848163 | 256.9602145 | 233.8180668 | 173.9099593 | 115.6478811 | 216.5426343 | 1069.789877 | 1765.13846 | 867.2168846 | 314.2433134 | 454.3783358 | 905.9360961 |
| ENSMUSG00000018217 | Pmp22 | 2346 | 291.1224077 | 262.6123895 | 274.8795964 | 261.7720872 | 278.1844642 | 270.7235236 | 206.5744971 | 215.2351885 | 239.7982177 | 211.133051 | 239.5468187 | 256.1905604 | 241.7179834 | 245.8606655 | 270.040547 | 262.8602865 | 278.3149165 | 277.2027535 |
| ENSMUSG00000018238 | Gdf9 | 2005 | 0 | 0.01661491 | 0 | 0 | 0.048011894 | 0.098858321 | 0 | 0 | 0 | 0.070549681 | 0 | 0.139191179 | 0.073813343 | 0 | 0.143845831 | 0 | 0.01911536 | 0 |
| ENSMUSG00000018239 | Zcchc10 | 1386 | 12.54301371 | 13.05115553 | 9.35361193 | 14.36520662 | 14.96743081 | 11.9055269 | 18.4676474 | 10.44366559 | 13.52082588 | 11.94076251 | 15.44241082 | 12.56456474 | 11.67450856 | 11.2591727 | 10.64224892 | 13.17793631 | 13.24552397 | 10.83512708 |
| ENSMUSG00000018259 | Prl8a2 | 998 | 0 | 0 | 0 | 0 | 0 | 0 | 0 | 0 | 0 | 0 | 0 | 0 | 0 | 0 | 0 | 0 | 0 | 0 |
| ENSMUSG00000018263 | Tbx5 | 3932 | 0 | 0.008472252 | 0 | 0 | 0 | 0 | 0 | 0.097132254 | 0 | 0 | 0 | 0 | 0 | 0.011811823 | 0 | 0.009738201 | 0 | 0.125910977 |
| ENSMUSG00000018286 | Psmb6 | 1977 | 164.5167461 | 161.9306593 | 158.3823278 | 147.7449951 | 167.2322629 | 153.9969626 | 170.8234204 | 155.8219109 | 163.5169258 | 156.3819733 | 164.0587724 | 148.4182793 | 176.1176895 | 154.4142572 | 154.2609594 | 161.5487892 | 152.8399215 | 157.1529766 |
| ENSMUSG00000018287 | Spag7 | 1590 | 101.2371708 | 103.1233115 | 100.7629764 | 95.73550259 | 97.86824479 | 103.4374357 | 110.1581996 | 107.8034604 | 110.6078128 | 108.5059266 | 111.1744317 | 105.5583002 | 104.0313954 | 107.2887629 | 108.9379503 | 99.50741496 | 93.33296957 | 95.34911832 |
| ENSMUSG00000018293 | Pfn1 | 1023 | 769.7467166 | 758.6417367 | 676.0945621 | 664.2648839 | 708.381469 | 694.8523589 | 689.1093998 | 653.1908822 | 641.4709244 | 635.4052738 | 640.7355135 | 598.6407135 | 766.9353005 | 761.9009527 | 647.1020513 | 747.1721456 | 628.8809578 | 649.1397227 |
| ENSMUSG00000018322 | Tomm34 | 3033 | 30.04652074 | 34.91648227 | 33.06242282 | 30.95987879 | 36.29334304 | 32.59403346 | 27.15838246 | 30.86369215 | 26.27626377 | 26.94103816 | 28.38459452 | 28.6347415 | 28.75662042 | 34.89814545 | 32.33092747 | 27.28189997 | 32.07126328 | 34.40562989 |
| ENSMUSG00000018326 | Ywhab | 3375 | 150.0338258 | 146.0535987 | 140.21874 | 148.6842496 | 149.0021744 | 152.4315496 | 151.2323497 | 141.8380953 | 146.3433176 | 147.5013704 | 148.4067155 | 140.7216216 | 157.8621353 | 138.0937532 | 154.4173275 | 164.7800835 | 156.9050205 | 138.2154741 |
| ENSMUSG00000018334 | Ksr1 | 9548 | 0.093486458 | 0.125603707 | 0.025331761 | 0.042946104 | 0.055451525 | 0.041518838 | 0.232896513 | 0.156001644 | 0.181172357 | 0.004938281 | 0.070051259 | 0.134453314 | 0.366837676 | 0.029185644 | 0.073358447 | 0.433115375 | 0.136478225 | 0.190123641 |
| ENSMUSG00000018339 | Gpx3 | 1806 | 150.039035 | 142.806438 | 174.208993 | 155.7299705 | 145.275258 | 170.827973 | 1295.099444 | 1207.120998 | 951.4176363 | 814.3025662 | 569.9663651 | 1045.848296 | 3434.249889 | 4141.907373 | 2307.127434 | 1334.128721 | 1233.890299 | 2641.685945 |
| ENSMUSG00000018340 | Anxa6 | 2904 | 87.6449704 | 78.87722474 | 73.84292702 | 76.31161145 | 76.34147397 | 93.09907521 | 78.47974325 | 69.25660849 | 64.72977202 | 66.34220851 | 74.26176397 | 72.22977273 | 62.88800198 | 64.18048302 | 60.07141448 | 79.81165517 | 64.84059803 | 63.57113022 |
| ENSMUSG00000018341 | Il12rb2 | 4495 | 0.085104913 | 0.214921898 | 0.505796647 | 0.081087509 | 0.246281256 | 0.143311577 | 0.547332437 | 0.331369009 | 0.229068497 | 0.356646031 | 0.903419682 | 0.074503665 | 1.11943394 | 0.888585432 | 0.174155631 | 0.715553064 | 0.264319289 | 0.477275898 |
| ENSMUSG00000018347 | Zkscan6 | 2299 | 7.746702373 | 6.839358883 | 7.722090318 | 8.640546033 | 9.337467575 | 8.104318323 | 7.305785567 | 9.602091601 | 10.87438672 | 7.157718763 | 12.07361935 | 8.375991123 | 7.767792107 | 8.666600039 | 9.390872243 | 9.127121729 | 11.46954862 | 7.728552184 |
| ENSMUSG00000018362 | Kpna2 | 3208 | 10.54679555 | 10.22855387 | 8.896628089 | 8.265750562 | 8.207033119 | 9.360647303 | 6.769488662 | 7.500378278 | 7.241020851 | 7.054968068 | 7.297297274 | 7.933897176 | 6.397156355 | 6.891329675 | 5.843736886 | 6.660273981 | 5.973550016 | 6.944728249 |
| ENSMUSG00000018363 | Smurf2 | 6525 | 6.442546987 | 5.886554335 | 5.233978917 | 5.634906173 | 6.697898343 | 4.738836131 | 4.394097077 | 4.466023454 | 4.241737715 | 4.465767757 | 4.817765928 | 5.611505695 | 4.067520536 | 2.982386608 | 3.22666591 | 4.835472873 | 3.788576475 | 4.21525722 |
| ENSMUSG00000018372 | Ccdc45 | 4280 | 2.850230262 | 3.533657471 | 2.588209919 | 3.417078325 | 3.115080107 | 3.392278257 | 3.47107037 | 3.185674687 | 4.262996842 | 3.073608927 | 4.576573125 | 4.486118675 | 3.100534053 | 4.134391891 | 4.303059692 | 4.634237064 | 4.781832371 | 4.665491995 |
| ENSMUSG00000018377 | Vezf1 | 4595 | 55.62211609 | 59.62971816 | 53.52144251 | 56.32911706 | 62.29391731 | 60.17502794 | 44.30608431 | 45.98880577 | 41.26718576 | 46.16561784 | 42.47240596 | 44.27597174 | 31.01630229 | 25.41032333 | 35.18496307 | 31.68245687 | 33.43854718 | 31.90408475 |
| ENSMUSG00000018378 | Cuedc1 | 4512 | 34.35646653 | 34.87074455 | 32.15253048 | 28.73819419 | 33.00535723 | 29.74042609 | 29.72771382 | 32.25869804 | 34.06650639 | 28.8735357 | 33.44882399 | 33.53640542 | 28.07720824 | 24.11757463 | 24.89260668 | 20.92744633 | 25.19410386 | 25.79771051 |

| ENSMUSG00000018379 | Srsf1 | 3019 | 87.37566162 | 90.51529357 | 98.1410644 | 91.12208867 | 93.37816394 | 83.21707825 | 83.5455089 | 87.94752617 | 95.06049746 | 75.98150757 | 81.14938293 | 85.72945592 | 75.29694429 | 68.16619782 | 80.9974277 | 80.42422707 | 89.37301431 | 77.05647974 |
| --- | --- | --- | --- | --- | --- | --- | --- | --- | --- | --- | --- | --- | --- | --- | --- | --- | --- | --- | --- | --- |
| ENSMUSG00000018381 | Abi3 | 6949 | 0 | 0.004793912 | 0 | 0.006556489 | 0 | 0 | 0.006808559 | 0 | 0 | 0 | 0.027500336 | 0.048193118 | 0 | 0.013367128 | 0 | 0 | 0 | 0 |
| ENSMUSG00000018387 | Shroom1 | 4127 | 8.270331076 | 9.694399311 | 9.423871676 | 7.937579568 | 8.222196798 | 8.789096433 | 11.33807595 | 11.79920355 | 12.21523995 | 11.61915804 | 9.839766103 | 10.8196099 | 11.83392249 | 15.44009884 | 14.47595252 | 12.81301886 | 15.97315984 | 13.78226711 |
| ENSMUSG00000018395 | Kif3a | 5982 | 8.519509538 | 7.345320541 | 8.717262742 | 7.182224141 | 8.384062932 | 7.968861535 | 7.102438261 | 6.67824355 | 6.871304368 | 6.896834729 | 6.852376191 | 6.969959872 | 9.500228803 | 6.560557313 | 5.875113202 | 7.060270806 | 7.630661921 | 8.257803049 |
| ENSMUSG00000018398 | 8-Sep | 3877 | 21.14843553 | 22.64108235 | 23.70639868 | 21.22343205 | 23.88594819 | 23.82416698 | 16.3159793 | 20.24384497 | 18.31456105 | 19.67754394 | 18.82902671 | 20.34241198 | 15.51086076 | 16.19613262 | 16.24894995 | 19.68356475 | 18.64415681 | 14.30208398 |
| ENSMUSG00000018401 | Mtmr4 | 6178 | 7.402973358 | 9.107393709 | 8.730412163 | 10.89984194 | 9.239958153 | 10.96448475 | 8.018189517 | 8.88353549 | 8.239969809 | 8.448661137 | 6.302458912 | 9.730247427 | 7.346287455 | 9.52487192 | 10.59716289 | 9.414605478 | 9.473009934 | 9.046496023 |
| ENSMUSG00000018405 | Mrm1 | 2513 | 7.154671492 | 9.080514341 | 6.641013917 | 7.052624849 | 7.35481842 | 8.518416596 | 7.775621328 | 8.966779713 | 7.063823436 | 6.529424884 | 6.710927315 | 8.462303003 | 8.284150056 | 7.577427708 | 6.705704878 | 7.207105969 | 6.695282268 | 5.560013175 |
| ENSMUSG00000018411 | Mapt | 4731 | 5.50743456 | 6.534425245 | 4.631834549 | 5.681888852 | 5.60572605 | 5.425558232 | 5.12028992 | 8.80742148 | 7.025474153 | 6.458181296 | 6.442702073 | 6.96073577 | 6.464973306 | 4.918302216 | 6.714521157 | 8.206864491 | 6.067722761 | 5.255573079 |
| ENSMUSG00000018412 | 1700081L11R | 8804 | 12.63954462 | 13.0845057 | 13.75271995 | 13.1394243 | 12.48674621 | 13.62643889 | 11.3606319 | 11.43950081 | 11.63458254 | 11.65378581 | 11.78094444 | 13.36431358 | 10.04120806 | 8.071269162 | 11.37675856 | 9.598747221 | 11.87575397 | 12.34018466 |
| ENSMUSG00000018415 | 4933439F18R | 5438 | 4.291162493 | 3.755204883 | 4.127495051 | 3.845626949 | 3.425350213 | 3.335104908 | 3.593257888 | 3.146413438 | 3.650588561 | 2.939332182 | 2.574119683 | 3.151049727 | 1.977631108 | 2.545115491 | 3.121559586 | 2.316588789 | 2.290556545 | 2.002906056 |
| ENSMUSG00000018417 | Myo1b | 11552 | 14.29839976 | 17.01979758 | 18.24057291 | 16.13094454 | 16.69117782 | 17.70291997 | 14.7442559 | 15.12554025 | 14.50726564 | 18.92640331 | 15.50039338 | 14.47090629 | 22.34284896 | 24.52466526 | 23.26147399 | 26.35130963 | 24.73691739 | 25.11885716 |
| ENSMUSG00000018425 | Dhx40 | 5576 | 7.127392322 | 5.96836107 | 6.237548121 | 6.169043844 | 6.439457506 | 6.869487636 | 7.237753789 | 6.232978154 | 6.825025209 | 6.604142619 | 8.285219664 | 6.056039431 | 5.936462786 | 8.504198928 | 6.5836776 | 6.345143694 | 8.756761523 | 5.948796871 |
| ENSMUSG00000018427 | Ypel2 | 4842 | 1.580118068 | 1.479197079 | 1.408646795 | 1.467889904 | 1.670004786 | 1.514623001 | 1.299584118 | 1.301475918 | 1.071768068 | 1.558057109 | 1.095212803 | 1.15273983 | 1.375425203 | 0.968784144 | 1.148742265 | 2.396128468 | 1.456430944 | 1.136082336 |
| ENSMUSG00000018428 | Akap1 | 4448 | 5.752723914 | 5.452290241 | 5.883561134 | 4.89617338 | 4.285126298 | 6.639709805 | 5.009953187 | 4.03561806 | 5.426101222 | 6.826675562 | 5.714091257 | 5.75975475 | 4.780138567 | 6.473771174 | 4.35358167 | 3.701654942 | 6.221130028 | 7.321355931 |
| ENSMUSG00000018433 | Nol11 | 5505 | 7.003123462 | 8.720050966 | 7.908479077 | 7.266593447 | 7.755316311 | 7.489168817 | 7.494397098 | 7.832737932 | 7.668697304 | 7.023356344 | 9.390100831 | 7.320419326 | 4.803252981 | 5.391057555 | 7.409544111 | 7.226873949 | 8.619065498 | 4.996285806 |
| ENSMUSG00000018442 | Derl2 | 6959 | 13.98108175 | 13.68609923 | 13.73560799 | 12.14481064 | 13.12751705 | 15.3165584 | 10.90523595 | 13.05643416 | 11.8724171 | 14.58765112 | 13.07134947 | 14.27674657 | 12.68919844 | 14.77614736 | 12.87733641 | 13.90434903 | 13.87875675 | 14.81348843 |
| ENSMUSG00000018446 | C1qbp | 1652 | 76.33950198 | 66.42413654 | 65.79620019 | 66.6592277 | 71.09073471 | 71.38953143 | 71.39861293 | 70.48948846 | 72.12626519 | 70.06959836 | 73.02165264 | 66.99906714 | 73.25132753 | 74.5860556 | 72.3521269 | 75.09780144 | 63.49822919 | 67.42944377 |
| ENSMUSG00000018449 | Rpain | 3194 | 11.55118747 | 11.22250287 | 9.389977715 | 10.07078813 | 9.689676556 | 9.836077986 | 8.650784125 | 11.12051792 | 9.697013104 | 10.02358407 | 9.872095365 | 12.21513718 | 9.977588779 | 11.28384841 | 10.64223031 | 9.782446975 | 10.0075553 | 7.732964596 |
| ENSMUSG00000018451 | 6330403K07R | 1484 | 0 | 0.291824545 | 0.293370467 | 0.153507562 | 0 | 0.233739309 | 0 | 0 | 0 | 0 | 0 | 0 | 0 | 0.125186219 | 0 | 0.206418369 | 0 | 0 |
| ENSMUSG00000018459 | Slc13a3 | 6763 | 0 | 0 | 0 | 0 | 0 | 0 | 0 | 0 | 0 | 0 | 0 | 0 | 0 | 0.013734759 | 0 | 0 | 0.068004667 | 0 |
| ENSMUSG00000018470 | Kcnab3 | 2851 | 0.954167403 | 0.560862477 | 0.882295187 | 1.534149511 | 0.574004 | 0.225950732 | 0.414877928 | 0.509053417 | 0.202248762 | 0.628455533 | 1.089222133 | 0.626482358 | 0.224943853 | 0.211775916 | 0.216774233 | 0 | 0.067215533 | 0 |
| ENSMUSG00000018474 | Chd3 | 8424 | 4.304002273 | 5.081560906 | 4.369925997 | 5.862793454 | 5.216577197 | 4.62939237 | 3.229438555 | 3.404854488 | 4.419825528 | 3.145619092 | 2.183447176 | 3.160502262 | 2.494705218 | 2.850379057 | 2.724271917 | 1.93634838 | 1.951802157 | 2.017784185 |
| ENSMUSG00000018476 | Kdm6b | 6931 | 4.93062495 | 3.883540402 | 4.299248983 | 3.970404094 | 3.493054048 | 3.581866906 | 3.167375995 | 3.967469302 | 4.45082965 | 3.442253044 | 3.460255253 | 3.929886502 | 3.9858424 | 3.41747 | 3.085212452 | 3.745640141 | 2.549188122 | 3.349277599 |
| ENSMUSG00000018479 | 1700125H20R | 988 | 0 | 0.033717504 | 0 | 0 | 0 | 0 | 0.430985942 | 0 | 0 | 0.095446768 | 0 | 0.169480757 | 0.199724361 | 0.047008186 | 0.417019796 | 0.038755676 | 0.50429338 | 0 |
| ENSMUSG00000018481 | Appbp2 | 6450 | 14.5769689 | 16.38787804 | 16.26700579 | 14.53008809 | 15.74548201 | 15.60334913 | 15.5948458 | 17.53297729 | 14.59088194 | 15.40987325 | 16.45088105 | 17.66197943 | 12.88748537 | 14.44447121 | 13.73385196 | 15.55370409 | 17.38647205 | 12.92927222 |
| ENSMUSG00000018486 | Wnt9b | 4514 | 0 | 0.073799057 | 0.150028672 | 0.201865504 | 0.053314049 | 0.054887831 | 0 | 0.685331102 | 0.346718564 | 0.334253989 | 0.201090875 | 0.012365012 | 0.404359978 | 0.586467209 | 0.392482699 | 0.313857438 | 0.152829717 | 0.804297972 |
| ENSMUSG00000018500 | Adora2b | 1843 | 0.230630364 | 0.054226089 | 0.209977343 | 0.197769048 | 0.104464294 | 0.13443498 | 0.102686227 | 0.787472215 | 0 | 0.051167339 | 0.207379093 | 0.27256699 | 0 | 0.050400529 | 0.245912705 | 0.249314861 | 0 | 1.044665619 |
| ENSMUSG00000018501 | Ncor1 | 13827 | 30.07363398 | 27.4680196 | 26.30859538 | 26.98668931 | 26.76200399 | 26.82090534 | 25.61187544 | 24.88983424 | 24.8274831 | 25.78676633 | 23.49876999 | 26.54140316 | 27.9394116 | 21.13110246 | 22.29180875 | 27.68432792 | 25.75043742 | 22.07207088 |
| ENSMUSG00000018507 | Trpv2 | 3728 | 0 | 0 | 0 | 0 | 0 | 0.026584085 | 0.139602862 | 0.256118578 | 0 | 0 | 0.538237193 | 0.104804087 | 0.185259346 | 0.261621737 | 0.011051919 | 0.174608456 | 0.071964613 | 0 |
| ENSMUSG00000018509 | Cenpv | 1629 | 6.966778391 | 8.895708401 | 9.235216661 | 7.607491749 | 6.441227354 | 8.639027831 | 8.24849651 | 8.229302176 | 6.320828079 | 7.207197735 | 6.334801124 | 8.120510763 | 7.389188346 | 6.300886012 | 7.385423155 | 5.382780292 | 4.634917428 | 6.010817064 |
| ENSMUSG00000018537 | Pcgf2 | 3994 | 1.841110529 | 1.509672973 | 1.635059238 | 1.049478237 | 0.903829313 | 0.843661964 | 1.101672294 | 1.176180645 | 1.814929087 | 0.991652247 | 1.543056996 | 1.621085844 | 1.815671467 | 1.209360313 | 1.702117357 | 1.965341642 | 1.698486368 | 1.432385353 |
| ENSMUSG00000018541 | Cwc25 | 6897 | 4.264692158 | 4.371200416 | 3.99079872 | 3.996582836 | 3.859207451 | 3.951573651 | 5.679991033 | 4.191916575 | 3.475504157 | 4.019807672 | 5.153627535 | 4.507658991 | 3.483346921 | 5.151475548 | 4.342979721 | 4.358145108 | 6.25157083 | 4.801432833 |
| ENSMUSG00000018543 | 1700001P01R | 621 | 0 | 0.053643952 | 0 | 0 | 0.155014247 | 0 | 0 | 0.307507266 | 0 | 0.075927058 | 0.0769323 | 0.089880294 | 0.158878961 | 0.448735144 | 0.132694222 | 0 | 0.061717064 | 0.177162985 |
| ENSMUSG00000018547 | Pip4k2b | 5193 | 0.212812358 | 0.166788995 | 0.23287854 | 0.210565196 | 0.24098402 | 0.209928777 | 0.154884564 | 0.132382677 | 0.230003651 | 0.281469632 | 0.349595306 | 0.257958002 | 0.493984151 | 0.223589868 | 0.333230377 | 0.47190427 | 0.324736581 | 0.169486945 |
| ENSMUSG00000018548 | Trim37 | 7379 | 2.862863869 | 3.765138065 | 4.818341891 | 3.988675246 | 3.939786136 | 4.190392432 | 3.930433959 | 3.105494165 | 3.605703914 | 2.971280257 | 3.897618242 | 3.063469758 | 2.75440438 | 4.110040529 | 3.540017134 | 2.672403146 | 4.077265628 | 2.474999788 |
| ENSMUSG00000018554 | Ybx2 | 1933 | 3.232416387 | 2.9125086 | 2.552560836 | 2.380582244 | 2.191210182 | 2.999311862 | 12.94796024 | 10.27421068 | 14.27571938 | 13.90372522 | 13.76650381 | 13.39806903 | 13.37295636 | 11.653069 | 14.6645993 | 11.70706103 | 14.07742928 | 10.6432519 |
| ENSMUSG00000018559 | Ctdnep1 | 2308 | 13.38876212 | 10.88298191 | 10.98255195 | 10.65986306 | 10.69830019 | 10.77793425 | 9.675729833 | 11.25252757 | 12.40235202 | 11.21565688 | 10.39126047 | 10.66495113 | 11.90549303 | 9.256620537 | 8.64018763 | 11.04919607 | 7.871171894 | 7.412405629 |
| ENSMUSG00000018565 | Rai12 | 2356 | 34.06187281 | 29.69315699 | 33.32353131 | 32.35298267 | 32.40120159 | 30.74961651 | 33.35584028 | 31.91886262 | 29.33402948 | 30.61993887 | 29.36253815 | 30.39537143 | 32.91586331 | 31.32413194 | 25.63730091 | 29.54682693 | 27.39451782 | 29.02220699 |
| ENSMUSG00000018566 | Slc2a4 | 2783 | 1.924416881 | 2.310236645 | 4.032576012 | 2.733990084 | 3.614650394 | 3.418657868 | 7.905280525 | 9.551531467 | 10.46312132 | 13.45226676 | 15.29554007 | 10.91042776 | 24.14303677 | 15.53699079 | 27.64042574 | 24.46306143 | 30.86210254 | 21.03114968 |
| ENSMUSG00000018567 | Gabarap | 2003 | 258.9144547 | 241.2898 | 248.0260393 | 226.690648 | 242.1014134 | 226.6610809 | 298.2848275 | 258.8045321 | 282.5481343 | 263.7661658 | 266.2326942 | 241.7378297 | 309.3158008 | 287.4988715 | 272.7983196 | 268.5121675 | 258.2384938 | 278.9727939 |
| ENSMUSG00000018569 | Cldn7 | 1593 | 292.65334 | 285.34491 | 281.7993482 | 272.1650332 | 265.7377707 | 275.2929643 | 227.9502892 | 282.3073059 | 256.4009271 | 228.6498322 | 256.7491648 | 272.9816869 | 234.4272528 | 238.9552669 | 241.5968196 | 331.1548639 | 267.4901249 | 269.9348319 |

| ENSMUSG00000018570 | 2810408A11R | 2447 | 11.30807911 | 9.325432178 | 9.923789227 | 8.620663477 | 8.812056315 | 9.801194545 | 8.294703416 | 11.81514043 | 7.624639003 | 7.823124447 | 11.3629039 | 11.99797242 | 13.66859007 | 9.22428544 | 11.04545184 | 8.99758858 | 9.475851911 | 10.34090278 |
| --- | --- | --- | --- | --- | --- | --- | --- | --- | --- | --- | --- | --- | --- | --- | --- | --- | --- | --- | --- | --- |
| ENSMUSG00000018572 | Phf23 | 3443 | 15.35766453 | 15.02611813 | 11.75969936 | 13.57700595 | 11.16973773 | 12.0895429 | 12.61488215 | 10.69342896 | 13.18255602 | 13.94116059 | 11.48930166 | 13.08255583 | 11.80641877 | 12.81495295 | 10.41108151 | 14.14628307 | 10.99807765 | 14.87466696 |
| ENSMUSG00000018574 | Acadvl | 2827 | 31.18349313 | 28.25763012 | 26.93312231 | 28.28427054 | 26.67942143 | 29.21981142 | 24.38435557 | 26.0470293 | 25.67054856 | 24.26751795 | 26.44775735 | 23.73202208 | 19.16039801 | 17.11876161 | 19.86477562 | 17.85179362 | 20.17316229 | 19.76980984 |
| ENSMUSG00000018581 | Dnahc11 | 14374 | 1.531771336 | 1.281621713 | 1.662482538 | 1.733817396 | 1.426478328 | 1.603034723 | 1.1290002 | 0.693489427 | 0.406879452 | 0.97752258 | 0.850868886 | 1.133864858 | 2.574018224 | 2.665672193 | 2.204257443 | 2.298928217 | 1.741134818 | 1.637950305 |
| ENSMUSG00000018583 | G3bp1 | 7135 | 24.70483041 | 22.82178373 | 23.59353688 | 24.70577162 | 22.6661897 | 21.84903992 | 19.33619786 | 20.34608571 | 19.9611733 | 20.26125525 | 19.92018241 | 21.05114899 | 17.71385733 | 17.87462705 | 20.42465356 | 18.03173663 | 20.10586255 | 17.91747218 |
| ENSMUSG00000018585 | Atox1 | 677 | 195.5740374 | 132.0706176 | 148.7646827 | 145.4319303 | 177.0998846 | 147.9994495 | 232.6497905 | 164.6165882 | 177.4001181 | 172.932343 | 155.9566591 | 175.5266553 | 306.2659502 | 303.9789531 | 242.3406138 | 240.0374266 | 202.0480852 | 264.2387224 |
| ENSMUSG00000018589 | Glra2 | 3141 | 0 | 0 | 0 | 0 | 0 | 0 | 0 | 0 | 0 | 0 | 0 | 0 | 0 | 0 | 0 | 0 | 0 | 0 |
| ENSMUSG00000018593 | Sparc | 3335 | 2.982372171 | 3.176461878 | 3.278086315 | 5.751484158 | 3.102957598 | 4.472375654 | 0.652588675 | 0.229039894 | 0.382843175 | 2.007616151 | 0.372458447 | 0.652716894 | 3.224695042 | 2.381390988 | 3.409783936 | 1.676290459 | 1.091753585 | 0.709262845 |
| ENSMUSG00000018595 | Glra4 | 2001 | 0 | 0 | 0.024174678 | 0 | 0.024053935 | 0 | 0 | 0.019086658 | 0.082331866 | 0 | 0.071626624 | 0 | 0 | 0 | 0 | 0 | 0 | 0 |
| ENSMUSG00000018599 | Smcr7 | 2837 | 3.236206564 | 3.463977369 | 3.427239902 | 3.998836813 | 2.697559345 | 4.331715871 | 5.570121523 | 3.60788292 | 4.021383457 | 3.972160056 | 3.586910876 | 4.32832068 | 2.312705322 | 1.931759715 | 3.645255738 | 2.739863695 | 3.498946386 | 3.063601996 |
| ENSMUSG00000018604 | Tbx3 | 6735 | 29.39073567 | 31.22063672 | 31.96172384 | 39.6350644 | 28.85056805 | 34.03577513 | 26.67353257 | 30.75239766 | 26.14288862 | 33.21201726 | 26.05455419 | 29.42028243 | 30.13385415 | 34.69342296 | 35.73860275 | 41.22423077 | 38.7587837 | 34.79417886 |
| ENSMUSG00000018620 | Mmp20 | 3266 | 0 | 0 | 0 | 0 | 0 | 0 | 0 | 0 | 0 | 0 | 0 | 0 | 0.468245387 | 0.156425279 | 0.050461183 | 0 | 0.105614413 | 0 |
| ENSMUSG00000018623 | Mmp7 | 1218 | 11.5161807 | 11.59660685 | 17.79256289 | 11.44637074 | 12.44791129 | 13.7104033 | 16.50893969 | 8.654436013 | 21.3371851 | 19.51063583 | 13.96378097 | 14.25178248 | 21.10174787 | 33.05995383 | 28.75313835 | 8.110818317 | 12.33490015 | 28.90462094 |
| ENSMUSG00000018634 | Crhr1 | 2449 | 0 | 0.14962917 | 0 | 0 | 0.019653705 | 0.080935457 | 0.173872646 | 0 | 0 | 0 | 0 | 0 | 0 | 0 | 0.302828912 | 0 | 0 | 0 |
| ENSMUSG00000018648 | Dusp14 | 3203 | 14.21262676 | 17.07766853 | 13.71313318 | 15.49047058 | 15.222491 | 15.98125938 | 13.58965489 | 15.04802118 | 16.22771867 | 16.28119819 | 18.04798619 | 16.41534629 | 15.81763317 | 13.63017238 | 12.91488046 | 23.32343898 | 17.13495384 | 16.26401001 |
| ENSMUSG00000018651 | Tada2a | 3902 | 6.002140439 | 8.230043574 | 7.872166011 | 8.827306386 | 6.685674687 | 6.527448759 | 6.620379983 | 6.753653936 | 9.256938576 | 7.757752817 | 8.472647685 | 6.89470768 | 9.696970925 | 7.581979412 | 10.25287308 | 9.979894343 | 9.861507455 | 9.755587353 |
| ENSMUSG00000018654 | Ikzf1 | 2661 | 0.175707229 | 0.100151505 | 0.036357407 | 0.119852428 | 0.018087908 | 0.05586554 | 0.871221822 | 1.047743471 | 0.510768516 | 0.318945005 | 0.269306418 | 0.272680802 | 1.724114358 | 2.44350704 | 0.356120175 | 0.661919557 | 0.172835612 | 0.640842656 |
| ENSMUSG00000018656 | Fam115e | 3564 | 0 | 0 | 0 | 0 | 0 | 0 | 0 | 0 | 0 | 0.013229715 | 0 | 0 | 0 | 0 | 0 | 0 | 0 | 0 |
| ENSMUSG00000018659 | Pnpo | 2919 | 33.20034304 | 29.99187599 | 30.95640791 | 28.51662487 | 31.16455489 | 31.320587 | 36.29088322 | 34.77745995 | 39.07004527 | 34.19597081 | 39.55877857 | 31.77993533 | 33.17524961 | 32.90386184 | 36.00725217 | 36.55906914 | 35.12259137 | 30.71765809 |
| ENSMUSG00000018661 | Cog1 | 5304 | 14.48894386 | 13.89293401 | 13.99950398 | 15.23855442 | 14.83704661 | 15.38713277 | 9.85680811 | 12.54358314 | 15.89532386 | 15.37020474 | 17.58233766 | 14.8378741 | 13.70021006 | 15.24493894 | 13.57072363 | 13.03786519 | 13.88099856 | 14.90348536 |
| ENSMUSG00000018666 | Cbx1 | 4483 | 23.4475352 | 21.87668092 | 24.09504646 | 20.17369463 | 22.49305824 | 21.77534801 | 22.73288209 | 21.3495785 | 27.3780542 | 21.28775895 | 23.3279911 | 19.87102331 | 20.62190787 | 18.79312392 | 20.97299811 | 20.56742865 | 20.00524978 | 19.1053266 |
| ENSMUSG00000018669 | Cdk5rap3 | 3641 | 39.10803068 | 39.10445204 | 35.97789624 | 42.11987785 | 38.93120439 | 38.05257981 | 43.31039793 | 41.40220057 | 43.81086942 | 37.91740158 | 42.55264772 | 40.48590082 | 41.33800594 | 41.34174327 | 42.78579589 | 39.81550113 | 40.84208514 | 42.99805486 |
| ENSMUSG00000018672 | Copz2 | 2314 | 2.792042678 | 2.864851318 | 2.822137683 | 2.933705966 | 2.080031273 | 3.447705318 | 8.689666614 | 6.651485812 | 7.79589192 | 8.333896989 | 7.8042067 | 6.657356471 | 7.54688795 | 7.366024232 | 4.860857717 | 6.43692579 | 7.569195196 | 6.44229383 |
| ENSMUSG00000018677 | Slc25a39 | 2990 | 111.8213092 | 115.8486535 | 103.1375438 | 112.8816777 | 108.7073179 | 116.2583365 | 127.3011712 | 107.1941943 | 114.3717861 | 128.1426805 | 118.7023968 | 112.4153579 | 128.213488 | 116.8713423 | 109.1083343 | 107.4057941 | 102.9426389 | 111.1036774 |
| ENSMUSG00000018678 | Sp2 | 4634 | 8.145143788 | 11.53802227 | 11.55578293 | 11.34601751 | 11.15530557 | 12.26521049 | 8.984712442 | 11.62090794 | 9.394507372 | 10.30722106 | 12.59840295 | 8.816802981 | 4.449879461 | 8.178328725 | 8.864469405 | 8.419967402 | 7.708266878 | 8.440111109 |
| ENSMUSG00000018697 | Aatf | 3018 | 12.37973815 | 12.46198064 | 11.17175303 | 12.53004201 | 11.54655823 | 13.15166985 | 8.700641751 | 11.78168543 | 11.01309417 | 11.46740099 | 13.26554513 | 11.52192107 | 9.938305402 | 12.18811041 | 11.90449196 | 10.77161222 | 10.71815593 | 10.13421582 |
| ENSMUSG00000018698 | Lhx1 | 4931 | 0.258599732 | 0.175651034 | 0.166771449 | 0.157075188 | 0.165938492 | 0.261279877 | 0.201493867 | 0.255597096 | 0.133641098 | 0.03824839 | 0 | 0.045277358 | 0 | 0 | 0 | 0 | 0.02331756 | 0 |
| ENSMUSG00000018707 | Dync1h1 | 15582 | 14.29114473 | 14.1636455 | 12.28741068 | 11.90926284 | 12.11174899 | 12.16080434 | 10.05948564 | 10.08120595 | 10.25830238 | 10.69076079 | 11.30447131 | 11.96766325 | 10.84972921 | 11.07301917 | 9.778164144 | 10.90577048 | 10.29118382 | 10.02251662 |
| ENSMUSG00000018727 | Cpsf4l | 2237 | 125.6534328 | 106.2230105 | 110.8677202 | 122.9150213 | 116.2094411 | 108.6305791 | 146.2103484 | 137.5063071 | 138.5833277 | 152.6232643 | 150.0095254 | 134.7859675 | 124.0906118 | 121.6639929 | 115.9242704 | 144.6724246 | 128.1883565 | 125.0922333 |
| ENSMUSG00000018733 | Pex12 | 4566 | 5.417873948 | 5.522965599 | 4.078801837 | 3.781786203 | 3.995181575 | 5.382863744 | 7.211919521 | 5.127451308 | 4.122259681 | 5.059974725 | 6.49764547 | 6.332131673 | 6.839050293 | 4.394184352 | 4.096694344 | 4.201400418 | 3.903137989 | 3.252838111 |
| ENSMUSG00000018736 | Ndel1 | 3795 | 50.78220504 | 54.01165695 | 52.01907177 | 52.82439923 | 52.7358468 | 51.70720026 | 38.28649196 | 45.70005252 | 41.96792001 | 43.83338105 | 41.70709814 | 43.87302279 | 48.43497333 | 46.87242548 | 46.83623508 | 57.67302027 | 55.04040003 | 54.63223274 |
| ENSMUSG00000018740 | Slc25a35 | 4562 | 8.590480556 | 9.069402591 | 7.613370187 | 7.29056606 | 8.567102591 | 7.603444433 | 11.80224214 | 12.18104899 | 10.29211486 | 9.239966837 | 8.828209118 | 10.43637882 | 10.08914485 | 7.88999731 | 7.631590252 | 6.790245811 | 8.014749506 | 8.730073053 |
| ENSMUSG00000018750 | Zbtb4 | 4079 | 9.701475578 | 8.967285573 | 9.309443817 | 8.187360995 | 9.970942755 | 7.459028786 | 4.848418679 | 7.733984895 | 9.208654664 | 7.675427056 | 6.816628055 | 7.47128016 | 9.578543389 | 7.526242158 | 8.474651972 | 13.82742454 | 11.06849173 | 9.480608489 |
| ENSMUSG00000018752 | BC096441 | 2515 | 2.197086634 | 2.29150326 | 3.481355465 | 2.699242786 | 2.985518923 | 3.369191826 | 2.765393162 | 2.050089195 | 3.864818179 | 2.455961084 | 2.944381139 | 2.996069363 | 2.687265474 | 2.529956251 | 2.621172542 | 2.085810423 | 2.026798206 | 2.077878783 |
| ENSMUSG00000018761 | Mpdu1 | 3001 | 35.15423089 | 32.09182845 | 32.3511081 | 32.9296585 | 31.72440687 | 31.35642821 | 34.49521543 | 33.29267734 | 36.1634352 | 35.58691865 | 35.46904615 | 36.77026489 | 33.78110297 | 29.9000256 | 32.57957085 | 34.00348841 | 29.38647423 | 27.14711331 |
| ENSMUSG00000018765 | Fxr2 | 3748 | 18.76896113 | 24.22028729 | 22.70251865 | 22.42799541 | 22.53776042 | 23.46747655 | 22.70958095 | 24.24218925 | 22.45038733 | 22.99666103 | 20.86649067 | 23.02321621 | 18.22964392 | 20.19846917 | 21.24936167 | 17.41875283 | 22.9875975 | 22.76390729 |
| ENSMUSG00000018770 | Atp5g3 | 2177 | 369.9531812 | 318.9897073 | 311.2837789 | 310.5350371 | 344.4848427 | 319.9645273 | 418.7508946 | 359.3980504 | 351.5705202 | 354.5724222 | 362.5586077 | 326.2024231 | 397.6691852 | 373.6860977 | 322.1367399 | 339.1453798 | 321.7331538 | 331.7223487 |
| ENSMUSG00000018774 | Cd68 | 1449 | 1.642712117 | 0.804659281 | 0.634297499 | 1.855142592 | 0.498260079 | 1.983477258 | 1.273426141 | 1.159741688 | 2.018110848 | 1.52938789 | 0.197825915 | 1.040043402 | 0.953273764 | 2.435990781 | 2.018847804 | 0.845617279 | 1.481209542 | 1.670393855 |
| ENSMUSG00000018776 | Amac1 | 2878 | 0 | 0 | 0 | 0 | 0 | 0 | 0 | 0 | 0 | 0 | 0 | 0 | 0.188551456 | 0 | 0 | 0.026609178 | 0.03995097 | 0.095568288 |
| ENSMUSG00000018796 | Acsl1 | 9557 | 10.69634282 | 12.13722901 | 12.4919821 | 11.82766046 | 11.86552393 | 11.00250085 | 13.72795424 | 15.21781609 | 16.23413256 | 15.32878885 | 13.25721355 | 14.24447013 | 15.92434496 | 21.25143812 | 16.82637571 | 12.92913991 | 17.61317317 | 15.23585911 |
| ENSMUSG00000018800 | Abca5 | 10710 | 3.936987359 | 5.384184864 | 4.137269263 | 4.462514987 | 4.772745372 | 4.97840722 | 2.107203347 | 4.079561938 | 2.914974692 | 4.14714869 | 3.67568308 | 3.439620664 | 4.638397824 | 4.358198677 | 4.824159765 | 5.856210544 | 5.396457118 | 6.795242595 |

| ENSMUSG00000018809 | Smyd4 | 4021 | 2.494708168 | 3.057064901 | 2.634619037 | 3.297255385 | 2.884803184 | 2.550961415 | 3.235758942 | 3.751802773 | 3.738641694 | 3.834439186 | 5.144629948 | 4.344765578 | 4.085433587 | 4.804959053 | 5.133543771 | 6.47540737 | 4.117622547 | 3.488515845 |
| --- | --- | --- | --- | --- | --- | --- | --- | --- | --- | --- | --- | --- | --- | --- | --- | --- | --- | --- | --- | --- |
| ENSMUSG00000018819 | Lsp1 | 5278 | 0.112745825 | 0 | 0.192467627 | 0.043161277 | 0 | 0.046942718 | 1.586651036 | 1.461702404 | 0.952018743 | 0.33947077 | 3.032324951 | 0.327829773 | 1.831954488 | 1.874306672 | 0.757209345 | 0.827042297 | 0.007261519 | 0.218869125 |
| ENSMUSG00000018820 | Zfyve27 | 8412 | 13.17296647 | 12.09037875 | 11.35731365 | 13.93045721 | 10.88861754 | 10.19683569 | 13.17803218 | 13.31649028 | 12.39218633 | 15.98594932 | 13.62483659 | 11.23346609 | 14.49696856 | 13.41644465 | 12.23507925 | 16.29126057 | 14.57055367 | 14.19041388 |
| ENSMUSG00000018821 | Avpi1 | 1232 | 23.18464147 | 17.90027271 | 15.39157784 | 14.53367729 | 16.09606538 | 16.45054223 | 16.35974125 | 17.88718846 | 15.81268016 | 18.14077382 | 18.30339318 | 19.43581108 | 21.58271381 | 26.99185598 | 20.40012102 | 21.53846666 | 20.99857988 | 20.13726228 |
| ENSMUSG00000018822 | Sfrp5 | 1897 | 0.044813048 | 0.122925809 | 0.025500016 | 0.120087096 | 0.025372653 | 0.052243262 | 0.07482237 | 0.181197481 | 0.065134184 | 0.04971081 | 0.02518448 | 0.264808099 | 0.078015684 | 0.171380396 | 0.130315939 | 0.10092411 | 0.020203636 | 0 |
| ENSMUSG00000018830 | Myh11 | 6590 | 0.316047591 | 2.269725266 | 2.613198301 | 0.622229741 | 1.117478653 | 1.827210055 | 0.488203667 | 0.840348763 | 0.412490143 | 0.550926275 | 1.080192536 | 1.21964422 | 1.265112901 | 4.862886236 | 6.020803994 | 0.90642409 | 6.705648001 | 3.973343672 |
| ENSMUSG00000018841 | Rad51l3 | 4586 | 5.310829887 | 5.121149242 | 6.328852641 | 7.510717296 | 5.971884991 | 5.726765983 | 6.210691826 | 5.946222267 | 4.804794141 | 6.446465535 | 5.865089757 | 5.720314308 | 5.034308174 | 4.283874611 | 5.183885248 | 5.619184209 | 4.680053699 | 5.397753603 |
| ENSMUSG00000018844 | Fndc8 | 2162 | 0 | 0 | 0.156621051 | 0 | 0 | 0 | 0 | 0 | 0 | 0 | 0 | 0 | 0 | 0 | 0 | 0 | 0 | 0 |
| ENSMUSG00000018845 | Unc45b | 4472 | 0 | 0 | 0 | 0 | 0 | 0 | 0 | 0 | 0 | 0 | 0 | 0 | 0 | 0 | 0 | 0 | 0 | 0 |
| ENSMUSG00000018846 | Pank3 | 7369 | 5.912309055 | 5.298237485 | 6.886122044 | 4.896776647 | 5.075112592 | 5.809955464 | 4.038495732 | 6.380085135 | 6.215145274 | 4.670920529 | 4.039055385 | 4.105318106 | 6.158958325 | 4.285789038 | 3.913840294 | 4.74410701 | 3.36505823 | 3.247246767 |
| ENSMUSG00000018848 | Rars | 2934 | 21.25258801 | 21.75443263 | 21.36744899 | 20.69968374 | 23.01604939 | 23.59412707 | 19.23790933 | 20.80145162 | 19.23160416 | 18.64172317 | 21.49384635 | 15.39020826 | 19.52091205 | 14.86400752 | 23.61997853 | 23.50422084 | 23.26487211 | 21.12994658 |
| ENSMUSG00000018849 | Wwc1 | 4937 | 39.04415096 | 52.14544187 | 45.78681534 | 52.37166834 | 48.47314654 | 56.84964209 | 40.76729515 | 43.24398004 | 37.99971223 | 46.89284039 | 33.22086945 | 43.36821586 | 32.20513862 | 30.74322009 | 34.76720313 | 32.90029504 | 33.93239695 | 35.7887889 |
| ENSMUSG00000018858 | Ict1 | 3534 | 19.73712333 | 17.29744224 | 20.06666539 | 16.48912725 | 19.21735832 | 20.3455055 | 21.46073127 | 18.42615906 | 21.4789894 | 20.52002875 | 21.3865264 | 22.64846071 | 21.84619427 | 22.82778149 | 22.17469137 | 21.23644327 | 20.1066651 | 21.29384776 |
| ENSMUSG00000018861 | Fdxr | 2900 | 4.016006287 | 4.227291404 | 3.369466597 | 3.283537333 | 2.456387829 | 3.605388547 | 3.44240526 | 4.62259767 | 3.706786419 | 4.487446241 | 4.480961623 | 5.119643534 | 4.609982614 | 4.96471279 | 4.219607621 | 4.858946043 | 4.599155628 | 5.576785302 |
| ENSMUSG00000018862 | Otop3 | 2587 | 0 | 0 | 0 | 0 | 0 | 0 | 0 | 0 | 0 | 0 | 0 | 0.02157544 | 0 | 0 | 0 | 0 | 0.014814958 | 0 |
| ENSMUSG00000018865 | Sult4a1 | 2476 | 0.961344854 | 0.739987553 | 0.429813275 | 0.772844855 | 0.64149979 | 0.320211526 | 0.439495806 | 1.187728185 | 1.131132097 | 0.57129285 | 0.443790002 | 1.059505711 | 0.159392301 | 1.613162971 | 0.249605549 | 0.154647041 | 0.897788861 | 1.28858166 |
| ENSMUSG00000018868 | Pnpla5 | 1684 | 0 | 0 | 0 | 0 | 0 | 0 | 0 | 0 | 0 | 0 | 0 | 0 | 0 | 0 | 0 | 0 | 0 | 0 |
| ENSMUSG00000018882 | Mrpl45 | 2679 | 13.50201747 | 14.59848369 | 12.4951411 | 11.00335785 | 15.81041166 | 13.04019305 | 13.12180681 | 12.00373379 | 12.77566054 | 12.30247913 | 15.21166091 | 10.47976046 | 11.58259648 | 9.188266635 | 10.42726946 | 12.19182087 | 12.91849425 | 9.137384282 |
| ENSMUSG00000018893 | Mb | 1188 | 1.25225687 | 0.925358173 | 1.343709177 | 1.840850276 | 0.202575436 | 0.750798994 | 1.115113645 | 0.450078816 | 0.589369333 | 1.031917748 | 0.201073058 | 0.328880167 | 2.117784328 | 5.864152448 | 2.774515548 | 0.483467265 | 1.645320827 | 3.380189218 |
| ENSMUSG00000018899 | Irf1 | 4007 | 9.843973884 | 14.44085282 | 11.78252199 | 16.45291519 | 16.03596658 | 12.824104 | 15.05457091 | 18.82455572 | 14.16396774 | 20.39235556 | 20.09004368 | 18.60986404 | 15.42622719 | 22.62512068 | 16.56493799 | 25.27543214 | 24.29468284 | 21.49844301 |
| ENSMUSG00000018900 | Slc22a5 | 3492 | 7.826697648 | 8.37592243 | 6.025912287 | 6.406206406 | 7.649833229 | 6.456613339 | 8.264815048 | 12.29331624 | 13.0919337 | 9.708291994 | 8.988587549 | 11.0448519 | 15.93539596 | 18.02168912 | 12.21180136 | 8.816050501 | 8.363298465 | 15.43783637 |
| ENSMUSG00000018906 | P4ha2 | 4936 | 6.38094316 | 7.984026309 | 8.016521035 | 6.110496627 | 7.17688327 | 6.24428693 | 1.725340804 | 4.24789889 | 3.496181139 | 1.805405777 | 4.161918992 | 3.516748595 | 1.379214867 | 0.404598006 | 1.268767523 | 0.90761772 | 0.986110151 | 1.069869175 |
| ENSMUSG00000018907 | Alox12e | 2815 | 10.04118723 | 7.24244805 | 10.5339162 | 7.898326691 | 9.523794488 | 10.68508323 | 2.050496209 | 2.496412804 | 2.443391876 | 3.735206688 | 1.714128173 | 4.005244704 | 0.473165814 | 0.808440598 | 1.024550235 | 2.067556776 | 0.966666814 | 1.973683758 |
| ENSMUSG00000018909 | Arrb1 | 8623 | 0.566867128 | 0.482907547 | 0.431956609 | 0.375140224 | 0.625162409 | 0.620630317 | 0.318234417 | 0.717519331 | 0.420319307 | 0.399165179 | 0.775657449 | 0.375427163 | 0.755167932 | 0.016158212 | 0.692824493 | 0.577267652 | 0.604473661 | 0.765521606 |
| ENSMUSG00000018914 | Il3 | 624 | 0 | 0 | 0 | 0 | 0 | 0 | 0 | 0 | 0 | 0 | 0 | 0 | 0 | 0 | 0 | 0 | 0 | 0 |
| ENSMUSG00000018916 | Csf2 | 796 | 0 | 0 | 0.121541534 | 0 | 0.362803445 | 0 | 0 | 0 | 0 | 0 | 0 | 0 | 0 | 0 | 0 | 0 | 0 | 0 |
| ENSMUSG00000018919 | Tm4sf5 | 1004 | 0.127007498 | 0.165900868 | 0.144542421 | 0.181518105 | 0 | 0.148065937 | 0 | 0 | 0 | 0 | 0 | 0 | 0 | 0.092518102 | 0 | 0.305104441 | 0 | 0.328739682 |
| ENSMUSG00000018920 | Cxcl16 | 3619 | 16.32555808 | 15.25268464 | 15.15766328 | 15.43460634 | 17.01042563 | 15.56823931 | 3.386013772 | 4.031361624 | 5.974833041 | 3.595908842 | 3.603913696 | 5.058728192 | 1.676658145 | 2.489680286 | 3.005584624 | 10.13606021 | 6.152964493 | 6.444835935 |
| ENSMUSG00000018921 | Pelp1 | 3543 | 3.98298573 | 4.231104262 | 4.177900166 | 3.960711729 | 4.102692901 | 3.94407871 | 3.164861673 | 2.285291931 | 3.859419484 | 4.60461285 | 3.627283046 | 4.095984271 | 2.408812218 | 4.312758891 | 3.186346688 | 3.231411689 | 2.444748264 | 3.586537864 |
| ENSMUSG00000018923 | Med11 | 1188 | 25.65337644 | 25.26508223 | 25.00113438 | 20.8629698 | 24.91677867 | 26.65336427 | 24.57232567 | 23.98277121 | 30.2658487 | 24.72633681 | 30.08052941 | 27.20308808 | 29.48287985 | 21.34551491 | 24.72786982 | 26.13946347 | 23.776499 | 20.32743927 |
| ENSMUSG00000018924 | Alox15 | 2556 | 3.176247504 | 4.36612659 | 6.907800696 | 5.36536555 | 4.914879529 | 5.059962232 | 3.202305733 | 1.972377589 | 1.434115768 | 2.066071504 | 2.635473062 | 2.140037141 | 6.948157363 | 12.17431086 | 7.721261843 | 9.392883741 | 10.93109172 | 15.1296565 |
| ENSMUSG00000018925 | Gm11435 | 1860 | 0 | 0 | 0 | 0 | 0 | 0 | 0 | 0 | 0.022143288 | 0 | 0 | 0 | 0.265225362 | 0 | 0 | 0 | 0.10302768 | 0 |
| ENSMUSG00000018927 | Ccl6 | 2335 | 0 | 0 | 0 | 0 | 0 | 0.042443455 | 0.040524779 | 0.588833613 | 0.917215769 | 0 | 0 | 0 | 0 | 0 | 0.141161648 | 0 | 0.065655327 | 0 |
| ENSMUSG00000018930 | Ccl4 | 657 | 0 | 0 | 0 | 0 | 0.732601578 | 0 | 0 | 5.638756519 | 0.188066282 | 0.071766672 | 0 | 0 | 0 | 0 | 1.881349584 | 0.407966898 | 0.291676537 | 0 |
| ENSMUSG00000018931 | Gm16515 | 4410 | 8.288991244 | 10.82480214 | 8.084193168 | 12.0566301 | 10.44495486 | 10.98924568 | 12.4450584 | 14.66207194 | 12.75754661 | 14.91501838 | 17.18165173 | 14.65635766 | 11.95823573 | 10.49994447 | 13.26671414 | 16.0716359 | 18.67646532 | 14.05788283 |
| ENSMUSG00000018932 | Map2k3 | 3519 | 42.14281306 | 47.61689626 | 42.29762799 | 46.29903227 | 45.4784445 | 44.45523726 | 34.23077026 | 51.55268867 | 44.19445397 | 53.11321049 | 55.67635826 | 46.15617452 | 36.96739581 | 41.58718936 | 39.55068366 | 43.54618097 | 45.78680085 | 37.09480257 |
| ENSMUSG00000018965 | Ywhah | 3150 | 75.09247765 | 76.51390148 | 71.13212464 | 73.59193447 | 73.6494832 | 73.08634927 | 45.54033096 | 50.12298144 | 48.11631048 | 46.25259462 | 49.86795666 | 49.64936082 | 37.86811936 | 35.81355183 | 39.33113605 | 39.27522302 | 40.71104427 | 37.77291995 |
| ENSMUSG00000018973 | Hoxb1 | 2453 | 0 | 0.013580471 | 0 | 0.018573601 | 0 | 0.181807828 | 0 | 0 | 0 | 0.038443297 | 0.019476135 | 0 | 0 | 0.094667932 | 0 | 0 | 0.078121274 | 0.044850474 |
| ENSMUSG00000018974 | Sart3 | 3567 | 10.62927306 | 11.04826293 | 12.49005367 | 11.72555052 | 12.7919999 | 11.58592089 | 9.205214235 | 11.6922073 | 12.10077615 | 11.46051576 | 12.52301267 | 9.357377692 | 8.505502979 | 14.19233397 | 11.25043887 | 11.86182259 | 9.584260397 | 12.52239828 |
| ENSMUSG00000018983 | E2f2 | 4874 | 0.043603997 | 0.075182978 | 0.069473679 | 0.084129955 | 0 | 0.010166749 | 0.271800371 | 0.070523517 | 0.439412971 | 0.154782776 | 0.245050054 | 0.194679168 | 0.212550321 | 1.05771311 | 0.245146722 | 0.086417046 | 0.15726835 | 0.564311723 |
| ENSMUSG00000018986 | Slfn3 | 1795 | 0 | 0 | 0 | 0 | 0 | 0 | 0 | 0 | 0 | 0 | 0 | 0 | 0 | 0 | 0 | 0 | 0 | 0 |

| ENSMUSG00000018995 | Nars2 | 4621 | 2.980237402 | 3.950544478 | 3.171863168 | 3.224077364 | 3.395586911 | 3.249183786 | 2.539178614 | 4.810209522 | 4.32275701 | 3.316160692 | 4.631720709 | 4.988502196 | 2.946463789 | 4.492643814 | 3.718036963 | 3.215052082 | 4.69437006 | 2.737955972 |
| --- | --- | --- | --- | --- | --- | --- | --- | --- | --- | --- | --- | --- | --- | --- | --- | --- | --- | --- | --- | --- |
| ENSMUSG00000018999 | Slc35b4 | 11070 | 5.790226328 | 6.169054485 | 6.476022763 | 6.564576849 | 6.048013173 | 7.166569689 | 8.030760949 | 6.77251002 | 6.656068356 | 7.504926752 | 6.525360182 | 7.08410171 | 6.41270361 | 6.897387504 | 8.199370156 | 6.395603711 | 7.184016809 | 5.600294786 |
| ENSMUSG00000019027 | Dnahc1 | 12789 | 0.073118608 | 0.231827945 | 0.158862169 | 0.220876124 | 0.127960388 | 0.220854313 | 0.066590681 | 0.053754261 | 0 | 0.088483609 | 0 | 0.100380033 | 0.142722726 | 0.083526 | 0.164303648 | 0.032934294 | 0.113879059 | 0.047314112 |
| ENSMUSG00000019039 | Dalrd3 | 1725 | 22.54622381 | 21.62924146 | 22.7145273 | 22.74090387 | 20.81531308 | 21.1999521 | 24.35574428 | 23.57965714 | 20.0799187 | 24.46369821 | 24.39984836 | 24.65596226 | 33.8888823 | 24.52786296 | 28.99634136 | 25.54926905 | 19.90745625 | 30.64565308 |
| ENSMUSG00000019054 | Fis1 | 1890 | 96.74987686 | 90.54409397 | 87.9425663 | 83.19109206 | 87.40146139 | 85.57678434 | 93.09833489 | 88.95394465 | 88.45294576 | 91.00834153 | 91.63186481 | 89.95476626 | 99.44688086 | 97.95033457 | 89.6842333 | 99.21116637 | 87.58268589 | 102.5672445 |
| ENSMUSG00000019055 | Plod1 | 4588 | 2.427279015 | 1.691783861 | 2.519894018 | 2.859978372 | 2.759088039 | 3.488564286 | 4.228029285 | 4.0123666 | 6.131296919 | 4.049123166 | 4.998251977 | 6.362596236 | 1.795647381 | 1.700655336 | 2.263032276 | 0.792852594 | 1.294807328 | 1.870405547 |
| ENSMUSG00000019066 | Rab3d | 5561 | 4.723646608 | 6.715294807 | 5.480187758 | 6.701840364 | 6.621277038 | 6.638515827 | 3.641400212 | 4.615230069 | 4.880761354 | 5.095769225 | 4.768045667 | 4.376124598 | 4.488752049 | 5.537210922 | 4.734363283 | 3.883456676 | 6.485352524 | 7.09252464 |
| ENSMUSG00000019080 | Mfsd3 | 2164 | 2.415959636 | 2.863307913 | 2.749512123 | 2.273841399 | 2.268695108 | 3.114219855 | 3.432574211 | 3.670988771 | 2.283910301 | 3.202935942 | 2.649258326 | 4.075265568 | 3.807037978 | 4.700210323 | 1.827795455 | 2.618766125 | 2.78060472 | 3.762175506 |
| ENSMUSG00000019082 | Slc25a22 | 3753 | 2.480318026 | 3.106718086 | 3.828121107 | 3.435591673 | 2.898431854 | 2.82554889 | 6.303314539 | 6.146605663 | 7.089392268 | 5.502799901 | 4.570000025 | 4.550917333 | 7.715916718 | 7.053858197 | 5.1378439 | 3.836202607 | 3.594685987 | 6.918278277 |
| ENSMUSG00000019087 | Atp6ap1 | 3134 | 205.9751462 | 210.6663212 | 211.120979 | 218.6174169 | 219.5577475 | 223.7146227 | 184.9481907 | 190.5598585 | 185.3261791 | 215.9694784 | 194.3010915 | 179.2904516 | 199.2637973 | 163.1174441 | 180.3981334 | 186.5414786 | 173.654568 | 179.1394203 |
| ENSMUSG00000019088 | Dnase1l1 | 2536 | 10.32460112 | 12.99150331 | 13.1615678 | 7.833050209 | 12.18481664 | 12.81805727 | 6.604372374 | 10.45170634 | 10.26414746 | 9.18471901 | 13.03638457 | 14.04195297 | 10.58224093 | 10.71364003 | 12.05502937 | 9.66324477 | 8.750365105 | 12.55925583 |
| ENSMUSG00000019102 | Aldh3a1 | 1930 | 2.973159981 | 2.295655405 | 1.980056424 | 2.053788009 | 4.538865341 | 0.795924736 | 2.94172097 | 3.00789905 | 1.493811453 | 5.301400314 | 0.792123664 | 7.432448334 | 2.607179048 | 3.032101042 | 3.928023981 | 1.07134342 | 1.25106565 | 1.140085113 |
| ENSMUSG00000019122 | Ccl9 | 3196 | 0 | 0.031269926 | 0.060542591 | 0.085533875 | 0 | 0.01550461 | 0.014803717 | 0.812604306 | 0.786100582 | 0 | 0 | 0.104785349 | 0 | 0 | 0.953978453 | 0.011980791 | 0 | 0 |
| ENSMUSG00000019124 | Scrn1 | 8615 | 0 | 0 | 0.005615035 | 0 | 0 | 0 | 0 | 0.004433245 | 0 | 0 | 0 | 0 | 0 | 0 | 0 | 0.071114303 | 0 | 0 |
| ENSMUSG00000019132 | BC005537 | 3800 | 27.57243655 | 19.94390378 | 25.91802837 | 18.5361512 | 21.93802416 | 18.21714969 | 22.90929717 | 24.79490967 | 28.63546701 | 18.30191771 | 23.27143372 | 23.29569496 | 27.30132159 | 24.23648034 | 21.44649409 | 26.672431 | 21.95693378 | 22.74192281 |
| ENSMUSG00000019139 | Isyna1 | 1825 | 109.6051279 | 112.825753 | 102.5253783 | 125.5237978 | 113.6968345 | 96.60746634 | 124.1018049 | 120.6252096 | 125.9968863 | 148.0144542 | 137.4348121 | 119.4608099 | 186.1637886 | 226.0872725 | 188.2177378 | 192.1663963 | 180.9841242 | 223.4726122 |
| ENSMUSG00000019143 | Hars2 | 4529 | 10.58640728 | 10.51095735 | 12.59271192 | 10.9048735 | 11.27577192 | 8.98273223 | 12.53592729 | 8.466586888 | 15.02321131 | 10.01522997 | 12.7006072 | 9.723682442 | 12.83130902 | 9.608767914 | 11.9902077 | 7.575266998 | 10.68803555 | 9.012311148 |
| ENSMUSG00000019146 | Cacng2 | 5506 | 0 | 0 | 0 | 0 | 0 | 0 | 0 | 0 | 0 | 0 | 0.00867689 | 0 | 0 | 0 | 0 | 0 | 0 | 0 |
| ENSMUSG00000019158 | Tmem160 | 811 | 69.18228404 | 59.06774585 | 61.55546649 | 55.67323667 | 59.58625322 | 61.95619217 | 63.70585128 | 51.09587745 | 58.19944151 | 57.73199548 | 60.61705583 | 59.94505809 | 63.9915869 | 67.86219921 | 61.72612874 | 64.21112955 | 54.81936425 | 60.36757705 |
| ENSMUSG00000019173 | Rab5c | 3337 | 11.17082391 | 9.683400477 | 9.929837667 | 8.369469637 | 8.178244147 | 7.42474282 | 8.350964306 | 8.080262541 | 10.77489609 | 8.322374653 | 7.688088913 | 7.493382331 | 10.24483628 | 8.754369484 | 9.815773729 | 10.958205 | 8.602456212 | 8.835745042 |
| ENSMUSG00000019178 | Styxl1 | 2030 | 0.795663394 | 0.787694051 | 0.548074482 | 0.807979111 | 0.545337066 | 0.976408543 | 0.839042582 | 0.884257593 | 0.649245569 | 0.580673685 | 0.753102794 | 0.769871208 | 0.947756046 | 0.549092659 | 0.588593656 | 0.905393673 | 0.792957867 | 0.867138628 |
| ENSMUSG00000019179 | Mdh2 | 2219 | 228.0415145 | 225.7590371 | 208.7103108 | 220.1466955 | 233.5016486 | 218.2642713 | 244.5803245 | 231.7533566 | 218.5910753 | 244.9329231 | 236.5495578 | 209.1515252 | 244.7254373 | 239.6715839 | 224.8724846 | 247.9139957 | 219.1628578 | 218.1523836 |
| ENSMUSG00000019188 | H13 | 9255 | 25.56281035 | 25.72525716 | 27.0849956 | 24.7767408 | 26.64807962 | 26.46024952 | 22.52400468 | 23.43127616 | 22.50906502 | 21.7642144 | 25.27349505 | 25.78798198 | 22.28598013 | 21.35775645 | 24.27130013 | 23.90111712 | 21.82797525 | 20.91594236 |
| ENSMUSG00000019189 | Rnf145 | 8063 | 38.27205481 | 32.35436868 | 34.83878095 | 33.50259604 | 30.04439685 | 31.55199481 | 28.14233017 | 26.09948372 | 35.5063216 | 29.59564792 | 30.85848738 | 31.78093848 | 26.25977787 | 26.50820912 | 25.64685713 | 29.07293793 | 24.37995496 | 23.16208822 |
| ENSMUSG00000019194 | Scn1b | 1508 | 0.620102038 | 0.706904917 | 0.577402882 | 0.664683671 | 0.446848098 | 0.624338155 | 1.882467331 | 1.089040652 | 1.857216891 | 3.12670446 | 1.900860417 | 1.221430282 | 5.299582625 | 3.326234375 | 3.743112173 | 11.8579003 | 6.836720071 | 2.662907686 |
| ENSMUSG00000019210 | Atp6v1e1 | 1194 | 1256.394621 | 1196.139364 | 1191.674223 | 1118.725878 | 1230.185858 | 1212.962935 | 1559.297511 | 1439.476125 | 1537.181492 | 1472.689428 | 1428.887389 | 1331.769189 | 1551.51772 | 1525.419875 | 1472.455269 | 1495.321978 | 1526.920953 | 1470.687357 |
| ENSMUSG00000019214 | Chtf18 | 4152 | 0.573287538 | 0.625820267 | 0.489327619 | 0.373091404 | 0.347774015 | 0.716079965 | 0.330459463 | 0.542714774 | 0.307510113 | 0.919847535 | 0.782441517 | 0.672153933 | 0.32080004 | 0.302020799 | 0.5755516 | 0.866887547 | 0.636925454 | 0.741934002 |
| ENSMUSG00000019230 | Lhx9 | 7513 | 0 | 0.004434034 | 0 | 0 | 0 | 0 | 0 | 0 | 0 | 0 | 0 | 0 | 0 | 0 | 0 | 0 | 0 | 0 |
| ENSMUSG00000019232 | Agxt2l1 | 2854 | 0 | 0 | 0 | 0 | 0 | 0 | 0 | 0 | 0 | 0 | 0 | 0 | 0 | 0.016273331 | 0 | 0 | 0 | 0 |
| ENSMUSG00000019235 | Rps6kl1 | 2507 | 0.644274706 | 1.288931288 | 0.656043092 | 1.781006121 | 0.998348636 | 1.166179211 | 0.113233376 | 0.106640134 | 1.018573585 | 0.394960019 | 0.838491493 | 0.445278521 | 0.629685422 | 0.092628814 | 0.410865137 | 0.244375636 | 0.107013992 | 0.109711023 |
| ENSMUSG00000019254 | Ppp1r12c | 5151 | 11.90739061 | 14.79064047 | 12.53711183 | 15.60273388 | 14.29661099 | 14.02599215 | 15.45859807 | 21.16115637 | 17.04710767 | 17.46525758 | 16.24033242 | 17.7058421 | 15.14147956 | 15.40923031 | 15.93350793 | 14.78548207 | 16.28737409 | 17.00145562 |
| ENSMUSG00000019256 | Ahr | 6392 | 6.124416008 | 9.005738612 | 7.99918986 | 6.864093507 | 8.215257935 | 6.922808368 | 4.929637698 | 6.572534832 | 6.321021897 | 6.277416845 | 5.91207637 | 4.916179292 | 5.726577381 | 4.061677855 | 5.04061588 | 6.427694265 | 5.486320661 | 6.299541008 |
| ENSMUSG00000019261 | Mtap1s | 3264 | 21.16143108 | 26.03590476 | 19.96297346 | 24.24618076 | 21.64756861 | 24.59418762 | 16.69859256 | 18.68666257 | 15.20519347 | 18.90939662 | 16.56901134 | 16.7412787 | 12.96776502 | 13.14777474 | 13.75909802 | 12.42333126 | 12.22355241 | 12.92646595 |
| ENSMUSG00000019278 | Dpep1 | 2230 | 1.467667513 | 0.597540703 | 1.236453466 | 0.551635963 | 0.798601424 | 0.777733487 | 0.763792126 | 0.291152843 | 0.701833004 | 0.655458207 | 0.642712446 | 0.350412231 | 0 | 0.395711955 | 0.18476034 | 0 | 0.309360244 | 0.394684174 |
| ENSMUSG00000019295 | Tmem129 | 4131 | 21.44294041 | 18.8297284 | 19.70773459 | 19.40011547 | 19.49278826 | 19.85228131 | 20.1116108 | 17.80647955 | 20.53842229 | 20.96728199 | 21.33739976 | 19.34834878 | 17.08883409 | 17.3476705 | 17.85301018 | 20.18807623 | 19.1306764 | 15.61986981 |
| ENSMUSG00000019297 | 2610027L16R | 3684 | 10.31477399 | 14.34154458 | 12.08025188 | 13.17114881 | 13.13045149 | 13.73326302 | 12.98401694 | 12.77226921 | 12.27545991 | 10.53339543 | 15.09501945 | 11.81764843 | 9.480726773 | 13.71638629 | 12.63782795 | 11.01738432 | 14.14868724 | 10.97494393 |
| ENSMUSG00000019301 | Hsd17b1 | 1333 | 0.095660561 | 0.124954592 | 0 | 0 | 0.072215939 | 0 | 0 | 0 | 0 | 0.212231222 | 0 | 0 | 0 | 0 | 0 | 0 | 0 | 0 |
| ENSMUSG00000019302 | Atp6v0a1 | 6364 | 14.13277066 | 14.71441635 | 13.3627697 | 14.73359982 | 15.11874849 | 15.01999105 | 16.18474262 | 17.73390149 | 14.76216883 | 18.93733462 | 14.99160781 | 15.59400504 | 24.3481383 | 27.25780427 | 23.61126247 | 23.58566641 | 24.06534922 | 19.98445235 |
| ENSMUSG00000019303 | Psmc3ip | 2136 | 2.427730091 | 2.729286745 | 1.970270197 | 2.495619002 | 1.960429475 | 1.809509934 | 1.240407313 | 1.5913501 | 2.217438817 | 1.368606555 | 2.505054003 | 1.463331978 | 2.240260288 | 2.783166285 | 1.234503547 | 0.950094659 | 1.561042992 | 1.545199627 |
| ENSMUSG00000019312 | Grb7 | 3171 | 49.47543511 | 49.85903373 | 52.82798348 | 52.37149373 | 49.86230503 | 52.13116342 | 47.86473478 | 47.28583155 | 46.92743029 | 53.96086475 | 53.30425831 | 55.37561479 | 43.23349042 | 42.34306422 | 48.08797173 | 48.94097502 | 48.27348781 | 49.21502232 |
| ENSMUSG00000019320 | Noxo1 | 2672 | 0.715843159 | 1.371414058 | 1.031920371 | 0.630897695 | 0.972726002 | 1.168346638 | 0.920755728 | 1.300714303 | 0.971089256 | 1.147004383 | 0.947632036 | 0.877342002 | 1.08929009 | 1.060287923 | 0.740147711 | 0.673524906 | 0.50202859 | 0.720553419 |

| ENSMUSG00000019326 | Aoc3 | 5375 | 0 | 0 | 0 | 0 | 0 | 0 | 0 | 0 | 0 | 0 | 0 | 0 | 0 | 0 | 0 | 0 | 0 | 0 |
| --- | --- | --- | --- | --- | --- | --- | --- | --- | --- | --- | --- | --- | --- | --- | --- | --- | --- | --- | --- | --- |
| ENSMUSG00000019338 | Zfp687 | 7226 | 7.641049496 | 12.48887772 | 11.14612898 | 11.39341366 | 10.75741167 | 10.61550396 | 7.320173684 | 12.98097707 | 11.11454549 | 10.75996536 | 9.322265632 | 12.13484721 | 10.3224272 | 9.113993346 | 10.70236928 | 9.723673482 | 10.97385944 | 9.873624608 |
| ENSMUSG00000019359 | Gdpd2 | 3489 | 5.335989424 | 7.28510699 | 7.195718617 | 5.68043975 | 7.918522266 | 6.945052084 | 10.03479003 | 8.603963397 | 6.787688897 | 11.70321267 | 12.41957218 | 8.958661808 | 13.19194005 | 9.717450213 | 12.41124541 | 7.155481807 | 13.24778276 | 15.26191324 |
| ENSMUSG00000019362 | D8Ertd738e | 656 | 162.9581063 | 147.0642403 | 145.5630319 | 146.267621 | 162.7387297 | 140.953355 | 171.7969531 | 145.550314 | 153.7588066 | 171.4244318 | 151.5544033 | 149.5791689 | 198.9820932 | 179.1212517 | 150.1093271 | 181.1799472 | 154.0647026 | 165.949729 |
| ENSMUSG00000019368 | Sec14l4 | 3526 | 29.29313039 | 34.30491177 | 37.15131032 | 36.67108447 | 35.38228762 | 37.01698816 | 15.55172855 | 20.92675027 | 17.35767511 | 21.15496669 | 20.16141186 | 23.09559039 | 14.34067363 | 16.74147336 | 19.85293348 | 16.34355194 | 20.06532731 | 18.12835565 |
| ENSMUSG00000019370 | Calm3 | 3368 | 30.9449797 | 29.60376853 | 28.28013102 | 25.45899211 | 30.58263558 | 27.30696957 | 33.04020809 | 27.66908013 | 28.85991009 | 30.19716951 | 22.31294824 | 23.25100196 | 28.65000896 | 25.27672571 | 26.43603393 | 23.12443452 | 19.92557775 | 19.74644003 |
| ENSMUSG00000019373 | Cops3 | 3292 | 25.03570362 | 19.09521002 | 18.57355479 | 19.43125949 | 22.64773687 | 21.43471829 | 23.13894686 | 21.63694729 | 23.78358641 | 23.63264288 | 23.45210598 | 20.43070274 | 24.27633839 | 23.64528872 | 23.81730281 | 21.26222063 | 25.15890875 | 21.53910649 |
| ENSMUSG00000019428 | Fkbp8 | 4230 | 61.39636537 | 63.64889151 | 60.54124579 | 65.96119043 | 61.13778389 | 60.2598727 | 64.38103544 | 56.97259082 | 60.84504421 | 60.01403932 | 60.34553266 | 63.21816536 | 59.47819813 | 57.67630994 | 60.43868896 | 59.81662735 | 58.92101862 | 61.60239682 |
| ENSMUSG00000019429 | Ffar3 | 1623 | 0 | 0 | 0 | 0 | 0 | 0 | 0 | 0 | 0 | 0 | 0 | 0 | 0 | 0 | 0 | 0 | 0 | 0 |
| ENSMUSG00000019432 | Ddx39b | 3524 | 40.96128768 | 43.70190408 | 47.72836693 | 44.86288985 | 47.87241557 | 44.64526933 | 48.41359824 | 52.94264635 | 50.62996205 | 52.95757193 | 51.15065787 | 50.46217395 | 49.16393118 | 52.46705786 | 55.52389016 | 52.19866003 | 49.23471796 | 46.95442481 |
| ENSMUSG00000019433 | Gipc1 | 1608 | 113.0828004 | 105.1800771 | 111.517834 | 119.5977414 | 98.89793271 | 112.6338565 | 101.333872 | 100.7300862 | 108.6524875 | 105.3853405 | 110.5835793 | 103.6824528 | 113.942003 | 110.0736424 | 110.8444843 | 128.7545486 | 95.38671655 | 101.5684315 |
| ENSMUSG00000019437 | Tlcd1 | 3161 | 20.3718259 | 18.31629552 | 19.09843906 | 19.55910698 | 19.47508079 | 17.72987715 | 23.48421172 | 22.21949637 | 21.4857844 | 25.28327808 | 24.81698254 | 23.73180972 | 30.21404361 | 37.2904441 | 32.58585566 | 35.60142207 | 33.36727905 | 33.53449815 |
| ENSMUSG00000019461 | Plscr3 | 3140 | 24.32541443 | 28.10377605 | 28.4078949 | 26.07426646 | 27.0703749 | 30.39444741 | 22.37558223 | 23.14654197 | 21.45896172 | 23.04978647 | 23.18759131 | 23.1617224 | 22.29362758 | 22.24583039 | 22.05726607 | 23.62067087 | 21.65313717 | 23.61537448 |
| ENSMUSG00000019464 | Ptger1 | 5010 | 1.001119914 | 1.336505337 | 1.303478363 | 2.09162479 | 1.248932151 | 1.908917681 | 1.690413079 | 0.983397188 | 1.430429889 | 1.383463748 | 0.791481348 | 0.846704662 | 1.211142879 | 1.149514339 | 0.71547612 | 0.626712536 | 0.841495541 | 0.966227823 |
| ENSMUSG00000019467 | Arhgef25 | 2370 | 0.233150755 | 0.534130793 | 0.040821545 | 0.422929525 | 0.588956028 | 0.501799834 | 0.219594712 | 0.56402282 | 0 | 0.298422173 | 0.645062731 | 0.164856387 | 0.20815155 | 0.058789984 | 0.226000096 | 0.242345616 | 0.113200033 | 0.208895342 |
| ENSMUSG00000019470 | Xab2 | 4620 | 16.91926812 | 19.72094495 | 20.48022194 | 17.56368397 | 18.74227937 | 21.52647972 | 19.52928111 | 21.37782524 | 19.29169266 | 21.13617023 | 22.23293522 | 18.89517236 | 21.32377463 | 22.40776424 | 20.33323537 | 21.4908106 | 22.90452505 | 25.57566261 |
| ENSMUSG00000019471 | Cdc37 | 2448 | 56.79511062 | 58.17509101 | 55.32920139 | 61.45529755 | 51.88731477 | 54.1274549 | 55.39139616 | 53.16981511 | 53.08147763 | 52.92897572 | 52.49780977 | 50.16113468 | 45.38213959 | 49.1002035 | 49.09515463 | 47.97275037 | 43.53980053 | 42.76238974 |
| ENSMUSG00000019478 | Rab4a | 1847 | 31.73505021 | 33.81790832 | 32.1093385 | 38.48144513 | 41.0958547 | 30.10187714 | 35.50372117 | 30.89304234 | 34.96505507 | 35.58639975 | 30.75496786 | 31.42841857 | 35.22945256 | 40.48455912 | 38.88159822 | 39.38936333 | 37.06051233 | 45.32964831 |
| ENSMUSG00000019487 | Trip10 | 2238 | 23.70261827 | 23.33986512 | 25.67817429 | 26.01743282 | 21.50666831 | 27.36692525 | 19.38593681 | 17.85042579 | 20.1699827 | 20.43618506 | 17.59006514 | 19.62775981 | 19.33158689 | 17.99241455 | 17.71040964 | 29.03447755 | 16.21760642 | 16.91075309 |
| ENSMUSG00000019489 | Cd70 | 839 | 0.151985135 | 0 | 0 | 0 | 0 | 0 | 0 | 0 | 0 | 0 | 0.227770958 | 0 | 0 | 0 | 0.245539666 | 0 | 0.091361852 | 0 |
| ENSMUSG00000019494 | Cops6 | 2601 | 121.4036727 | 122.0576247 | 120.6641541 | 112.3348625 | 117.0636483 | 109.2408206 | 125.1121129 | 121.3464104 | 126.9957157 | 115.9823912 | 120.8241741 | 114.3781167 | 137.6020991 | 130.1541534 | 123.2403325 | 132.7731596 | 128.2847908 | 128.1007918 |
| ENSMUSG00000019505 | Ubb | 1529 | 696.7346153 | 669.3516914 | 662.1067647 | 628.6172605 | 679.7965282 | 668.2003014 | 721.7581661 | 700.8018587 | 692.8439319 | 693.5378131 | 670.5366965 | 693.6985194 | 805.8299319 | 921.6523869 | 806.2462735 | 770.5953436 | 751.4611216 | 910.8702185 |
| ENSMUSG00000019518 | Ap4m1 | 3871 | 2.964711322 | 2.624756584 | 2.349321547 | 2.60113428 | 2.31271966 | 1.843346328 | 3.080029731 | 3.038816828 | 2.713139116 | 2.411738374 | 1.875947737 | 1.860041455 | 1.937083809 | 2.567562568 | 2.479969652 | 1.829956696 | 2.930659748 | 2.330533067 |
| ENSMUSG00000019528 | Gyg | 3066 | 9.385519951 | 9.224607695 | 11.12307205 | 8.73773453 | 8.634232882 | 9.002241554 | 10.01497998 | 9.118342649 | 10.06154609 | 8.073750558 | 8.554615853 | 6.644721736 | 11.0699149 | 8.49808644 | 9.80989426 | 9.916093371 | 8.925302019 | 11.59030755 |
| ENSMUSG00000019539 | Rcn3 | 1590 | 0.05346563 | 0.167612046 | 0.334659644 | 0.429821173 | 0.484346402 | 0.592139584 | 0.238051215 | 0.552468714 | 0.310841628 | 0.533781546 | 1.712687191 | 0.842500567 | 0.930790892 | 1.22682495 | 0.932865416 | 1.204107151 | 2.072994675 | 0.588147682 |
| ENSMUSG00000019558 | Slc6a8 | 5343 | 3.134388799 | 2.331840247 | 2.734195427 | 1.858938361 | 1.765647958 | 1.465343797 | 2.302320142 | 1.829918588 | 2.227756514 | 1.491384775 | 2.36058189 | 2.966806696 | 1.763502938 | 1.912352466 | 2.313394491 | 2.665937854 | 2.259551474 | 2.573886708 |
| ENSMUSG00000019564 | Arid3a | 5667 | 0.705044388 | 1.081625646 | 1.16089644 | 1.318512665 | 1.019204313 | 0.909384911 | 0.93506618 | 0.714380564 | 1.061096048 | 1.123230093 | 0.632278434 | 1.457688029 | 0.565832826 | 0.5900784 | 0.785206994 | 0.756758078 | 0.899487822 | 0.844501903 |
| ENSMUSG00000019577 | Pdk4 | 3500 | 0.230742384 | 0.266503154 | 0.069105043 | 0.117156971 | 0.192527695 | 0.325632249 | 0.189250716 | 0 | 0 | 0.55233681 | 0 | 0.191367986 | 0.028189667 | 0.278664524 | 0 | 0.962735272 | 0.19710667 | 0.628675505 |
| ENSMUSG00000019578 | Ubxn6 | 4701 | 18.03825275 | 16.74502639 | 17.99729942 | 19.8003007 | 16.18731575 | 17.86681204 | 16.07282456 | 18.12534562 | 14.74514061 | 17.60252802 | 16.58556312 | 15.09076944 | 19.59214838 | 17.51656391 | 15.25886169 | 17.27597922 | 17.67526307 | 20.7819982 |
| ENSMUSG00000019579 | D17Wsu104e | 2215 | 40.02970528 | 42.08102845 | 41.42870749 | 42.31109174 | 40.43950788 | 42.39387365 | 47.14179792 | 42.29614587 | 43.10173523 | 44.68141135 | 46.65337932 | 46.76924143 | 56.21388677 | 56.32000845 | 50.39052592 | 41.36768556 | 46.51064834 | 49.69445713 |
| ENSMUSG00000019590 | Cyb561 | 3455 | 124.9320007 | 132.9816026 | 131.5395999 | 131.6457035 | 132.456825 | 122.297007 | 141.1027044 | 144.3683865 | 123.2853679 | 111.5376839 | 111.9358289 | 116.817093 | 173.0115316 | 212.7287071 | 134.0749907 | 86.07905866 | 104.7067776 | 141.8614011 |
| ENSMUSG00000019647 | Sema6a | 8597 | 0.143381424 | 0.526992394 | 0.23069847 | 0.4875673 | 0.587862276 | 0.43806069 | 0.011006788 | 0.20435623 | 0.397636479 | 0.170021147 | 0.111143326 | 0.019477374 | 0.109027152 | 0.043218879 | 0.268382823 | 0.142526397 | 0.164949748 | 0.070385038 |
| ENSMUSG00000019659 | Ccdc12 | 811 | 81.02712964 | 75.99118903 | 74.08128816 | 63.93152707 | 75.61044482 | 71.85451873 | 76.77371821 | 77.75050107 | 76.53277343 | 69.88303985 | 72.92897484 | 80.04144955 | 74.69740372 | 72.4436135 | 74.93501226 | 75.68414755 | 66.06678554 | 68.9818268 |
| ENSMUSG00000019689 | 1110001J03R | 686 | 69.70601025 | 47.49272674 | 55.9891882 | 58.17999248 | 71.14543819 | 55.83711816 | 74.4864333 | 61.01876536 | 60.51896194 | 71.27591731 | 74.72672077 | 77.78392629 | 73.56640142 | 53.82368728 | 47.92834053 | 64.85960029 | 53.91381416 | 63.58924436 |
| ENSMUSG00000019699 | Akt3 | 4965 | 3.835310949 | 3.576187839 | 3.799733502 | 4.744221535 | 4.856816467 | 3.393339258 | 3.487702012 | 2.061543574 | 3.119059401 | 3.067407281 | 3.117640795 | 4.170717184 | 3.288794485 | 3.554633073 | 3.86705439 | 3.694098881 | 4.731927493 | 4.464988924 |
| ENSMUSG00000019710 | Mrpl24 | 2296 | 20.78977033 | 20.82055889 | 19.38312192 | 17.83945072 | 18.38488548 | 16.46721939 | 19.20532089 | 20.7097304 | 19.39138656 | 19.87886792 | 18.93519696 | 19.03469678 | 20.94887602 | 22.29154369 | 17.74753431 | 18.91182439 | 18.34520919 | 19.04714279 |
| ENSMUSG00000019715 | Gle1 | 6243 | 7.748020234 | 7.299701953 | 8.120368383 | 8.794018649 | 8.989559987 | 9.127926256 | 8.533409654 | 6.919046471 | 6.207994767 | 8.526853112 | 7.338809095 | 9.199794458 | 8.194329363 | 10.15476202 | 9.991855777 | 11.82513072 | 11.18540973 | 8.000683791 |
| ENSMUSG00000019718 | 2810055F11R | 2592 | 1.000314714 | 1.195254306 | 0.951794 | 0.808567916 | 1.207011975 | 0.516174308 | 0.693627237 | 0.604123649 | 0.635594379 | 0.491153159 | 0.276475454 | 0.516811691 | 0.609036016 | 1.272195295 | 0.349704564 | 0.974992318 | 0.10350466 | 0.891351266 |
| ENSMUSG00000019726 | Lyst | 11757 | 0.903826997 | 0.64886049 | 0.645967872 | 0.697540868 | 0.765558367 | 0.754438999 | 0.663995239 | 0.760144779 | 0.956359514 | 0.617607238 | 0.983375007 | 0.69312637 | 0.881151878 | 0.592550235 | 0.869098057 | 0.752328852 | 1.098576342 | 0.697146968 |
| ENSMUSG00000019731 | Slc35e1 | 10461 | 3.218057491 | 3.515671085 | 3.842692259 | 3.45377193 | 3.496822673 | 3.960050212 | 2.948844916 | 3.789667689 | 2.73631856 | 3.885279247 | 3.46175495 | 2.55041456 | 3.310487136 | 3.396398705 | 3.974034021 | 4.037332948 | 4.099715728 | 3.54422502 |
| ENSMUSG00000019732 | Calr3 | 2522 | 1.702229492 | 1.096340293 | 1.687894794 | 1.535562557 | 1.030580443 | 0.589445681 | 2.570117769 | 2.332129251 | 1.241147977 | 2.131316483 | 2.689946116 | 2.589386409 | 2.542882334 | 1.546908541 | 2.156465257 | 1.867464199 | 2.036369463 | 1.810371078 |

| ENSMUSG00000019734 | Tmc4 | 4062 | 195.3647554 | 212.1298183 | 205.2242857 | 203.7460291 | 208.7375597 | 217.9490739 | 150.0447883 | 167.841599 | 167.9095768 | 164.9113838 | 180.0323214 | 179.0855565 | 161.1727903 | 157.3633616 | 161.4589775 | 183.4876103 | 173.3363861 | 173.5590132 |
| --- | --- | --- | --- | --- | --- | --- | --- | --- | --- | --- | --- | --- | --- | --- | --- | --- | --- | --- | --- | --- |
| ENSMUSG00000019737 | AI428936 | 2801 | 15.90340038 | 19.12428915 | 17.28736304 | 19.84451056 | 17.3041225 | 16.34656402 | 16.89135271 | 18.912125 | 24.05610132 | 17.54053295 | 19.85364215 | 22.91610567 | 20.21886506 | 24.37444072 | 24.09430509 | 28.3932851 | 24.4927067 | 24.27392214 |
| ENSMUSG00000019738 | Polr2i | 1503 | 23.18978333 | 18.64014906 | 20.5981766 | 18.49117568 | 21.9363724 | 21.62780654 | 25.02566849 | 23.45414998 | 21.01800239 | 22.74401854 | 22.82263486 | 23.95282925 | 27.63637681 | 29.75758893 | 25.43915094 | 27.71801787 | 20.3998919 | 28.80383699 |
| ENSMUSG00000019756 | Prl8a1 | 1945 | 0 | 0 | 0 | 0 | 0 | 0 | 0 | 0 | 0 | 0 | 0 | 0 | 0 | 0 | 0 | 0 | 0 | 0 |
| ENSMUSG00000019761 | Krt10 | 2083 | 6.101319075 | 4.87778816 | 6.223766748 | 4.221450579 | 5.407042793 | 4.995714859 | 8.54035875 | 5.977303497 | 6.44589733 | 7.424594656 | 6.146754139 | 7.288459061 | 6.560221356 | 7.781558569 | 7.259227562 | 6.470616325 | 6.605444353 | 6.602149151 |
| ENSMUSG00000019762 | Iyd | 1618 | 0 | 0.0617668 | 0 | 0 | 0 | 0.091877751 | 0 | 0 | 0 | 0 | 0 | 0 | 0 | 0 | 0 | 0 | 0 | 0 |
| ENSMUSG00000019763 | Rmnd1 | 5958 | 3.224628997 | 3.019295549 | 3.783495325 | 3.57881315 | 3.401064093 | 3.734336586 | 3.629052412 | 3.80770175 | 4.140772565 | 3.956923738 | 3.383859093 | 4.122002608 | 4.322131725 | 4.25620539 | 4.868394654 | 3.817492581 | 3.396489554 | 4.080903856 |
| ENSMUSG00000019767 | Gm221 | 3731 | 0 | 0.348218406 | 0.194479484 | 0.158749283 | 0 | 0 | 0 | 0 | 0.099351016 | 0 | 0 | 0 | 0 | 0 | 0 | 0 | 0 | 0 |
| ENSMUSG00000019768 | Esr1 | 16230 | 15.27096004 | 18.1076003 | 17.44785254 | 18.15423744 | 19.32394422 | 18.54792708 | 11.59350118 | 14.16860474 | 13.43954328 | 12.63452917 | 14.16470118 | 13.56014526 | 10.99405698 | 9.248754802 | 12.08883605 | 9.809756351 | 13.4035774 | 9.87655804 |
| ENSMUSG00000019769 | Syne1 | 30771 | 1.976695814 | 1.828522906 | 1.770127561 | 1.640558874 | 1.512579058 | 1.592657161 | 2.666152717 | 3.372290707 | 4.611080134 | 3.844565158 | 3.06172104 | 4.362440887 | 5.869297363 | 4.137117531 | 4.006208938 | 7.688981935 | 4.879998416 | 4.397725213 |
| ENSMUSG00000019772 | Vip | 1520 | 0.838917948 | 1.139651644 | 0 | 0.809308024 | 0.886640699 | 9.617142372 | 0.217887337 | 0 | 0 | 0 | 0 | 0.036720831 | 0 | 0 | 0 | 0 | 0 | 0 |
| ENSMUSG00000019773 | Fbxo5 | 1997 | 3.086254644 | 3.252886516 | 3.463903275 | 1.91643852 | 3.567113021 | 3.846106011 | 2.70087401 | 4.475223917 | 4.289832388 | 3.990219755 | 3.18180745 | 5.729699965 | 2.223271184 | 4.604871959 | 2.228226358 | 1.936580545 | 3.32020499 | 3.93905972 |
| ENSMUSG00000019774 | Mtrf1l | 3273 | 3.441451771 | 3.765893939 | 4.359972947 | 3.090299982 | 3.102913503 | 3.300487602 | 2.934455797 | 3.547354211 | 2.214734731 | 3.94723272 | 4.130862588 | 4.126914252 | 4.461426066 | 3.121814612 | 4.305203824 | 3.310798012 | 3.922795436 | 4.420224585 |
| ENSMUSG00000019775 | Rgs17 | 8450 | 0 | 0 | 0 | 0 | 0 | 0 | 0 | 0 | 0 | 0 | 0 | 0 | 0 | 0 | 0.082890704 | 0 | 0.004535656 | 0 |
| ENSMUSG00000019777 | Hdac2 | 4700 | 25.70206602 | 25.06263701 | 25.65855557 | 28.34478588 | 26.51352136 | 28.87764623 | 26.16290479 | 20.24197327 | 22.51237425 | 23.19413318 | 21.48856643 | 23.26444319 | 21.01329753 | 19.34840917 | 21.88942235 | 22.61589918 | 21.52796252 | 19.37022801 |
| ENSMUSG00000019779 | Frk | 4979 | 20.76171683 | 19.06174646 | 25.07573445 | 21.00987303 | 23.31677041 | 23.806013 | 19.55603399 | 17.15167941 | 20.32441639 | 18.77884004 | 19.49763319 | 19.51698505 | 17.25973085 | 14.54234429 | 20.05048603 | 17.40342329 | 17.18871681 | 19.43382581 |
| ENSMUSG00000019782 | Rwdd1 | 1880 | 25.97789748 | 26.17188551 | 23.31192473 | 21.30221169 | 25.37166827 | 22.4305193 | 33.67253426 | 26.08459824 | 27.8666213 | 24.07695485 | 24.03995251 | 25.20611571 | 34.89970743 | 30.63333424 | 29.80538086 | 33.32097538 | 28.03120119 | 33.73696811 |
| ENSMUSG00000019785 | Clvs2 | 7252 | 0 | 0 | 0 | 0 | 0 | 0 | 0 | 0 | 0 | 0 | 0 | 0 | 0 | 0 | 0 | 0 | 0 | 0 |
| ENSMUSG00000019787 | Trdn | 4562 | 0 | 0 | 0 | 0 | 0 | 0 | 0 | 0 | 0 | 0 | 0 | 0 | 0 | 0 | 0 | 0 | 0 | 0 |
| ENSMUSG00000019789 | Hey2 | 2545 | 0.016701444 | 0.235611826 | 0 | 0 | 0 | 0.097353111 | 0.669255969 | 0.060027352 | 0.210382988 | 0.148214391 | 0.037544172 | 0.285109475 | 0.116303145 | 0.109494902 | 0 | 0 | 0.361426768 | 0.389062444 |
| ENSMUSG00000019790 | Stxbp5 | 11334 | 2.148885289 | 2.080953689 | 1.246256473 | 1.716478378 | 1.685933801 | 1.871234337 | 2.124770917 | 1.607356269 | 1.729731913 | 1.784687815 | 1.635493549 | 1.226230809 | 2.189337779 | 1.5120759 | 1.137823098 | 1.057434279 | 1.298508736 | 2.15978935 |
| ENSMUSG00000019791 | Hint3 | 1040 | 22.35608778 | 23.2549627 | 26.18682461 | 25.01476569 | 25.40810201 | 24.49048563 | 37.8956361 | 33.12456439 | 42.96862462 | 37.76589982 | 39.59809059 | 31.07429677 | 32.87213334 | 31.34975899 | 33.15932912 | 39.39514415 | 35.19385918 | 29.99054184 |
| ENSMUSG00000019792 | Trmt11 | 2870 | 6.812676297 | 6.616149723 | 7.180182554 | 5.492725206 | 6.423180057 | 5.801295638 | 6.660042609 | 5.855281205 | 7.892886296 | 5.552939966 | 6.991457338 | 5.737149987 | 5.397289904 | 4.709139174 | 7.17797141 | 6.017095449 | 6.810596314 | 5.865082459 |
| ENSMUSG00000019794 | Katna1 | 1889 | 31.88450737 | 29.27443325 | 30.67839564 | 28.31586345 | 28.35936 | 30.06216659 | 29.17907409 | 29.37721583 | 30.45927079 | 29.2289431 | 26.75802333 | 29.25225302 | 28.96193555 | 35.18342459 | 34.48367383 | 34.29735719 | 35.93217142 | 40.01191775 |
| ENSMUSG00000019795 | Pcmt1 | 6301 | 23.75190046 | 23.2413082 | 24.39788597 | 22.80582984 | 25.54406487 | 24.91399143 | 26.90387694 | 24.22711195 | 25.08056832 | 25.33012705 | 25.76405474 | 23.36799205 | 25.76596409 | 23.00460193 | 26.05748297 | 26.44063368 | 25.51639668 | 23.91206845 |
| ENSMUSG00000019796 | Lrp11 | 4207 | 0.404137638 | 0.25339021 | 0.20697018 | 0.444022538 | 0.171613705 | 0.259130054 | 0.326139218 | 0.217879168 | 0.63634978 | 0.560383923 | 0.397462217 | 0.504158588 | 1.336781215 | 0.883177321 | 0.332981436 | 0.737232993 | 0.965673276 | 0.614553843 |
| ENSMUSG00000019797 | 1700021F05R | 1608 | 23.71090976 | 22.54006773 | 23.88593476 | 18.02041306 | 21.55160761 | 23.5745281 | 28.27579879 | 20.3075274 | 28.22608232 | 29.52783469 | 25.3433082 | 21.83336553 | 24.66595864 | 21.57570478 | 22.95808089 | 25.86044754 | 22.26166748 | 21.82575254 |
| ENSMUSG00000019802 | Sec63 | 8030 | 14.6200867 | 13.69437906 | 13.8313357 | 12.82858297 | 13.06694814 | 14.1746736 | 10.51130999 | 12.44701334 | 13.30483461 | 12.08420265 | 10.89959202 | 13.51253401 | 14.1360824 | 16.73837969 | 11.65752615 | 14.86324076 | 16.14668274 | 12.83774172 |
| ENSMUSG00000019803 | Nr2e1 | 3953 | 0 | 0 | 0 | 0 | 0 | 0 | 0 | 0 | 0 | 0 | 0 | 0 | 0 | 0 | 0 | 0 | 0 | 0 |
| ENSMUSG00000019804 | Snx3 | 1804 | 147.8492126 | 138.1451007 | 138.8459757 | 137.6933557 | 144.6892583 | 134.0450886 | 152.3499733 | 148.2813672 | 153.033933 | 139.1520755 | 136.5452804 | 129.2670944 | 153.6836891 | 136.9637167 | 128.1953067 | 132.8709547 | 138.0511515 | 134.5344672 |
| ENSMUSG00000019806 | Aig1 | 9222 | 14.30205608 | 13.36200344 | 14.09452137 | 13.03296844 | 14.08153655 | 13.9759987 | 14.01629135 | 13.6212114 | 12.2773511 | 12.62873965 | 14.67129499 | 13.53932305 | 12.44799084 | 11.90060491 | 13.85448111 | 12.08259245 | 12.65906532 | 14.280178 |
| ENSMUSG00000019808 | Adat2 | 1460 | 2.503729547 | 2.144802778 | 2.219881188 | 1.747546906 | 3.626377811 | 1.968533252 | 3.143376614 | 1.412595705 | 0.959138038 | 1.969995136 | 2.257857627 | 2.332024259 | 2.230072973 | 5.471495227 | 2.17295877 | 3.278305428 | 2.835095935 | 1.09264664 |
| ENSMUSG00000019809 | Pex3 | 4633 | 12.30292274 | 11.13785304 | 13.26017344 | 12.45971146 | 11.39665881 | 11.92559852 | 12.20346456 | 10.41161326 | 10.48998243 | 11.33733724 | 10.69342369 | 12.43292981 | 9.987769999 | 11.32782619 | 12.61036181 | 13.81866945 | 15.28750198 | 10.76910421 |
| ENSMUSG00000019810 | Fuca2 | 2704 | 103.7164022 | 99.48284794 | 109.8959308 | 109.5385907 | 105.6979892 | 124.010114 | 84.58191196 | 83.77187081 | 73.75188954 | 81.74648553 | 80.92060275 | 79.67768402 | 83.17463419 | 92.42442095 | 86.27337628 | 98.2896102 | 85.69555883 | 86.21619611 |
| ENSMUSG00000019813 | Cep57l1 | 2966 | 1.84867421 | 1.493801393 | 1.304747953 | 1.843332879 | 1.801295862 | 1.152777686 | 2.791543767 | 2.240552265 | 1.805208041 | 1.70098626 | 1.723506594 | 1.975942202 | 1.230803061 | 3.006495205 | 2.083692982 | 1.897747568 | 2.093344605 | 3.449660772 |
| ENSMUSG00000019814 | Ltv1 | 1755 | 116.0112782 | 111.9730843 | 111.2453382 | 113.0329271 | 127.7758589 | 117.0065686 | 144.3104104 | 139.4729954 | 146.3470725 | 153.5151672 | 164.6398569 | 140.2865457 | 110.3292167 | 109.5604113 | 127.4783182 | 124.4062925 | 145.2905147 | 123.6842935 |
| ENSMUSG00000019815 | Fam164b | 1151 | 0 | 0 | 0 | 0 | 0 | 0.043051897 | 0 | 0 | 0 | 0 | 0 | 0 | 0 | 0 | 0 | 0 | 0 | 0 |
| ENSMUSG00000019817 | Plagl1 | 5750 | 66.18979933 | 75.14230226 | 68.52215735 | 70.04515338 | 69.6866547 | 76.08714518 | 69.12588099 | 69.88877532 | 68.05444978 | 70.73425501 | 77.25418503 | 72.86128059 | 69.87973325 | 60.57116719 | 71.71220371 | 76.23493453 | 89.33026628 | 82.28405686 |
| ENSMUSG00000019818 | Cd164 | 2881 | 219.9321968 | 231.8027041 | 252.3281549 | 248.1740607 | 243.4663393 | 245.9060854 | 238.2877373 | 245.3672947 | 244.8889327 | 255.9165383 | 249.255606 | 259.5496118 | 162.0196464 | 154.9210968 | 186.3437255 | 166.4000016 | 205.2937917 | 179.118511 |
| ENSMUSG00000019820 | Utrn | 13154 | 3.386454651 | 3.051698155 | 2.559523881 | 2.933723928 | 2.674808895 | 2.749999659 | 3.158015212 | 4.006805179 | 2.839909518 | 4.401783762 | 4.423741784 | 4.115948967 | 2.587731711 | 2.778736261 | 3.132245392 | 3.918135743 | 3.327400871 | 4.035562414 |
| ENSMUSG00000019822 | Smpd2 | 4245 | 12.94680627 | 11.84981632 | 11.82843922 | 11.32318063 | 10.98700448 | 13.94947388 | 11.41299958 | 9.833756379 | 11.89509266 | 10.06325964 | 12.44737434 | 13.92433137 | 10.38933664 | 11.76145912 | 11.27825864 | 11.66307547 | 10.81623173 | 10.56123015 |

| ENSMUSG00000019823 | Mical1 | 5962 | 0 | 0.011175074 | 0.024340924 | 0.022925718 | 0 | 0.116359992 | 0.150778413 | 0.160149289 | 0 | 0 | 0.240397309 | 0 | 0.413719534 | 0.124640288 | 0 | 0.01926733 | 0 | 0 |
| --- | --- | --- | --- | --- | --- | --- | --- | --- | --- | --- | --- | --- | --- | --- | --- | --- | --- | --- | --- | --- |
| ENSMUSG00000019826 | Zbtb24 | 2858 | 11.89787992 | 10.15239009 | 10.18924607 | 12.0358952 | 9.885737996 | 12.84764716 | 10.23066325 | 8.940069002 | 12.14843694 | 9.684206722 | 11.48404355 | 7.909497322 | 7.301399933 | 10.23784991 | 8.260493886 | 9.63294147 | 12.67262091 | 6.544120464 |
| ENSMUSG00000019828 | Grm1 | 8849 | 0 | 0 | 0 | 0 | 0 | 0 | 0 | 0 | 0 | 0.005328365 | 0 | 0.006307567 | 0 | 0 | 0 | 0 | 0 | 0 |
| ENSMUSG00000019831 | Wasf1 | 2844 | 1.270372701 | 2.096697632 | 2.160140069 | 1.698126125 | 1.320073856 | 2.108256246 | 1.813319973 | 1.369769707 | 1.882646642 | 2.835010639 | 1.243089637 | 1.47193203 | 2.133553384 | 2.400591015 | 2.042693172 | 1.911837641 | 1.751905273 | 2.920666356 |
| ENSMUSG00000019832 | Rab32 | 2105 | 0.888469238 | 1.23439703 | 1.26391647 | 1.082210079 | 1.440527882 | 1.577212898 | 2.247633203 | 0.943470273 | 0.939169956 | 0.313591376 | 0.431222903 | 1.140177431 | 1.992025163 | 0.904611678 | 2.055184498 | 1.491605609 | 2.294115634 | 1.306629613 |
| ENSMUSG00000019834 | Slc22a16 | 2407 | 0 | 0 | 0 | 0 | 0 | 0 | 0 | 0 | 0 | 0 | 0 | 0 | 0 | 0 | 0 | 0 | 0 | 0 |
| ENSMUSG00000019836 | Amd-ps4 | 1679 | 0 | 0 | 0 | 0 | 0 | 0.059026484 | 0 | 0 | 0 | 0 | 0 | 0 | 0 | 0 | 0 | 0 | 0.022826859 | 0 |
| ENSMUSG00000019837 | Gtf3c6 | 1014 | 43.17586914 | 43.82583914 | 37.49664187 | 40.48372874 | 43.33771825 | 43.98171619 | 50.95211577 | 43.08886425 | 39.03376888 | 40.91974247 | 34.25285485 | 39.30215295 | 39.50445447 | 39.16143463 | 41.52661748 | 44.37027978 | 41.0854879 | 34.23150527 |
| ENSMUSG00000019838 | Slc16a10 | 6139 | 9.04939975 | 7.819495125 | 8.588882243 | 7.800074539 | 7.989319143 | 9.605432963 | 5.98826381 | 5.144993776 | 6.547978398 | 5.560695416 | 4.801620684 | 6.57350123 | 9.908169735 | 9.729124675 | 9.818842852 | 13.69081011 | 10.1949573 | 11.81006722 |
| ENSMUSG00000019841 | Rev3l | 13681 | 5.256834869 | 4.497398993 | 3.889400146 | 5.258452526 | 3.862937811 | 4.411609495 | 4.015058858 | 3.743586845 | 4.506703757 | 3.301686552 | 3.73301883 | 3.761570127 | 4.359497697 | 2.984018187 | 3.22240076 | 3.946331148 | 3.538199911 | 3.791651754 |
| ENSMUSG00000019842 | Traf3ip2 | 3677 | 10.61184433 | 13.74372057 | 12.49792054 | 14.05118963 | 12.9067383 | 12.66781876 | 11.65767939 | 11.57093725 | 11.43634283 | 10.19440008 | 12.47320102 | 12.73574678 | 9.525608177 | 11.16578005 | 11.53016073 | 8.1642198 | 10.24605653 | 11.07063883 |
| ENSMUSG00000019843 | Fyn | 4343 | 12.9482726 | 11.92759625 | 12.69763403 | 12.4734243 | 12.72287552 | 11.67221885 | 11.60211906 | 13.31413706 | 11.10509094 | 13.02805524 | 14.52059525 | 12.11931149 | 18.03801166 | 18.415086 | 17.73099423 | 21.7065336 | 19.82942416 | 20.8611556 |
| ENSMUSG00000019845 | Tube1 | 2917 | 1.180294569 | 0.616693962 | 0.845749074 | 0.937148666 | 0.577517082 | 0.849378361 | 0.25951418 | 1.139094621 | 0.395345369 | 1.050667025 | 2.325692185 | 1.798653508 | 1.978688489 | 1.608108614 | 1.101721412 | 0.682588819 | 1.300755363 | 1.885810995 |
| ENSMUSG00000019846 | Lama4 | 11964 | 0 | 0 | 0.012129772 | 0 | 0 | 0 | 0 | 0 | 0 | 0 | 0 | 0 | 0 | 0 | 0 | 0 | 0 | 0 |
| ENSMUSG00000019848 | Popdc3 | 2077 | 0 | 0.080194738 | 0 | 0 | 0.231737716 | 0 | 0.341690026 | 0.58842411 | 0.773362597 | 0.02270135 | 0.092007623 | 0.295605339 | 1.163824721 | 2.862225896 | 0.555437441 | 0.387146247 | 0.461317969 | 0.847516329 |
| ENSMUSG00000019849 | Prep | 2692 | 30.94730498 | 31.62992482 | 28.17596419 | 32.47832247 | 34.79373085 | 37.53269828 | 27.85683362 | 27.01275415 | 28.59494724 | 27.98916189 | 28.69691971 | 26.06251407 | 25.5272514 | 27.10388607 | 26.76876718 | 32.17435139 | 29.92640271 | 26.23762743 |
| ENSMUSG00000019850 | Tnfaip3 | 8636 | 7.107164681 | 11.67648573 | 12.91678566 | 15.66358738 | 16.18516748 | 14.32186464 | 16.78624922 | 21.8160168 | 21.0081753 | 29.79950653 | 26.43223154 | 28.28915645 | 20.79869277 | 24.59880219 | 26.03484142 | 50.20432464 | 48.22740487 | 42.91933315 |
| ENSMUSG00000019851 | Perp | 1898 | 176.716725 | 192.8918374 | 185.1345229 | 184.6446539 | 195.6722461 | 193.328763 | 199.5209073 | 200.3994392 | 217.5203551 | 225.0216386 | 206.7563272 | 214.8226634 | 200.0827709 | 201.9754991 | 233.9675287 | 210.6588633 | 227.6759734 | 234.3538656 |
| ENSMUSG00000019852 | D10Bwg1379e | 8182 | 0.197408542 | 0.431577461 | 0.283785072 | 0.211601037 | 0.517674075 | 0.230139804 | 0.560905629 | 0.47612137 | 0.619156861 | 0.489832532 | 0.204366114 | 0.402484001 | 0.952632966 | 0.885514255 | 0.619382959 | 1.315040416 | 1.044581302 | 0.726104073 |
| ENSMUSG00000019853 | Hebp2 | 1906 | 40.60961466 | 33.61001867 | 37.84099358 | 39.39380958 | 36.33884478 | 36.00762645 | 46.96515663 | 45.02535583 | 49.83006576 | 48.43708131 | 42.33573183 | 48.61175231 | 64.13666895 | 52.65775071 | 62.60215418 | 59.60557826 | 47.75705936 | 52.72908604 |
| ENSMUSG00000019854 | Reps1 | 5490 | 31.06981264 | 33.67090164 | 32.11685212 | 30.22464908 | 32.01781152 | 30.45372005 | 36.09207638 | 37.9976142 | 36.42268379 | 38.55364425 | 38.12424282 | 39.09129739 | 33.22041861 | 45.03976708 | 38.91258056 | 36.14940222 | 45.72627408 | 37.95528165 |
| ENSMUSG00000019856 | Fam184a | 4682 | 0.072627383 | 0.476711643 | 0.268627037 | 0.136235502 | 0.215884322 | 0.169338688 | 0.040420913 | 0.089730121 | 0.017593556 | 0.281977721 | 0.122447566 | 0.107291961 | 0.042146021 | 0.525744689 | 0 | 0 | 0.327435258 | 0 |
| ENSMUSG00000019857 | Asf1a | 2176 | 27.38614742 | 25.59704004 | 26.38758296 | 26.25622684 | 26.96360981 | 26.23380013 | 27.67878689 | 33.19018058 | 36.45460908 | 28.21241528 | 27.66380868 | 31.93497239 | 34.48246607 | 36.41250601 | 33.21118062 | 36.3373641 | 35.82522422 | 36.58004477 |
| ENSMUSG00000019861 | Gopc | 4198 | 9.96309986 | 10.30017549 | 10.00195911 | 11.61274832 | 9.080629715 | 10.10413052 | 11.16885775 | 9.998439791 | 10.9883034 | 9.44586504 | 7.488309357 | 8.934760662 | 9.495042677 | 11.06338432 | 8.617190584 | 11.51089722 | 10.48997502 | 10.58774612 |
| ENSMUSG00000019863 | Qrsl1 | 3240 | 13.78794445 | 14.52812331 | 12.76523718 | 12.89490051 | 13.78593369 | 15.15640709 | 12.38307152 | 12.96655637 | 15.00002735 | 12.07872954 | 12.96116929 | 10.06060091 | 13.09427434 | 11.1953186 | 12.22058494 | 11.23900236 | 10.71716821 | 10.32269657 |
| ENSMUSG00000019864 | Rtn4ip1 | 3125 | 73.04089449 | 63.84349551 | 67.1501999 | 59.01795439 | 68.55526152 | 62.3492315 | 72.64199472 | 57.96690067 | 48.27718632 | 58.90443057 | 49.54836494 | 48.13542741 | 36.2925049 | 29.54587063 | 37.60218797 | 26.61350377 | 29.70441315 | 41.40205408 |
| ENSMUSG00000019865 | Nmbr | 1265 | 0 | 0 | 0 | 0 | 0 | 0 | 0 | 0.090574867 | 0 | 0 | 0 | 0 | 0 | 0 | 0 | 0 | 0 | 0 |
| ENSMUSG00000019866 | Aim1 | 7523 | 10.93845817 | 11.94712064 | 10.96329513 | 11.24642554 | 11.31160987 | 12.81138732 | 8.333018687 | 8.396947172 | 6.761311573 | 8.110196732 | 9.8814084 | 6.529015429 | 5.318115766 | 6.173612573 | 7.848176205 | 6.168844367 | 6.83688561 | 6.895332665 |
| ENSMUSG00000019867 | Gje1 | 1477 | 0 | 0 | 0 | 0 | 0 | 0 | 0 | 0 | 0 | 0 | 0 | 0 | 0 | 0 | 0 | 0 | 0 | 0 |
| ENSMUSG00000019868 | Vta1 | 2768 | 27.22603941 | 24.31071038 | 27.00039899 | 23.86687655 | 27.24809407 | 26.5128607 | 25.62200351 | 23.01478584 | 24.98271675 | 27.1695707 | 23.07627149 | 23.33046301 | 29.17498142 | 25.94023089 | 24.75368044 | 30.90361883 | 30.89088453 | 27.10708872 |
| ENSMUSG00000019872 | Smpdl3a | 2092 | 28.66864406 | 34.69827749 | 32.55732827 | 33.84410272 | 29.44974297 | 35.67228335 | 32.25042072 | 37.64471021 | 33.0555258 | 35.92649187 | 32.65687888 | 38.47332 | 35.84345806 | 30.01549051 | 33.36301897 | 50.572155 | 40.74459097 | 39.28470624 |
| ENSMUSG00000019873 | Reep3 | 3015 | 52.85303646 | 58.14011588 | 60.0698168 | 59.5089196 | 54.40583611 | 59.15100768 | 31.0082433 | 40.99191184 | 42.57989042 | 35.45295001 | 34.87651198 | 44.87468196 | 34.47507453 | 34.24384951 | 39.39770668 | 34.0487955 | 43.50002919 | 36.94641496 |
| ENSMUSG00000019874 | Fabp7 | 1404 | 0 | 0 | 0 | 0 | 0 | 0 | 0.134793957 | 0 | 0 | 0 | 0.034027748 | 0 | 0 | 0 | 0 | 0 | 0 | 0 |
| ENSMUSG00000019876 | Pkib | 7816 | 3.083474259 | 3.771994803 | 3.051196324 | 3.153598386 | 2.974375529 | 3.810285681 | 1.54359431 | 1.910597408 | 3.372488496 | 2.292383219 | 1.650363202 | 3.120706826 | 1.760952523 | 1.319164202 | 1.402202388 | 1.601973979 | 1.382806516 | 0.520812935 |
| ENSMUSG00000019877 | Serinc1 | 2971 | 155.8713877 | 156.8430711 | 165.8962978 | 163.8878764 | 165.3593217 | 170.0904669 | 135.6795774 | 144.0667296 | 139.5156831 | 144.8959679 | 153.5681095 | 143.6065045 | 133.1347401 | 121.214222 | 146.5976598 | 163.962675 | 167.0825303 | 142.7533534 |
| ENSMUSG00000019878 | Hsf2 | 2654 | 5.044887132 | 5.209062208 | 4.046316405 | 3.776725604 | 5.077972354 | 6.236101362 | 5.383733623 | 4.964724503 | 5.524641903 | 4.174986912 | 6.408396842 | 6.435415505 | 6.542891817 | 5.459892715 | 5.650853935 | 5.468025699 | 6.960540734 | 5.699888602 |
| ENSMUSG00000019880 | Rspo3 | 2389 | 0 | 0.027888568 | 0 | 0 | 0 | 0.04148408 | 0 | 0 | 0.017240065 | 0.118419514 | 0 | 0 | 0.061948829 | 0 | 0.017246361 | 0 | 0 | 0 |
| ENSMUSG00000019883 | Echdc1 | 3325 | 3.234228432 | 2.895466595 | 2.618717433 | 2.904944782 | 2.967532136 | 3.293580187 | 4.183436874 | 3.514849665 | 3.468338169 | 4.169114814 | 2.959892164 | 3.323759757 | 2.462886697 | 3.198705568 | 4.275048656 | 2.982636787 | 3.861446455 | 3.044112974 |
| ENSMUSG00000019888 | Mgat4c | 7138 | 0 | 0 | 0 | 0 | 0 | 0 | 0 | 0 | 0 | 0 | 0 | 0 | 0 | 0 | 0 | 0 | 0 | 0 |
| ENSMUSG00000019889 | Ptprk | 7373 | 6.831497843 | 9.013864637 | 9.80198757 | 9.089962867 | 9.739974246 | 9.254592993 | 4.094057935 | 5.066074807 | 5.418543373 | 5.608458803 | 6.311245023 | 6.200058003 | 5.225583534 | 5.996849477 | 6.387274974 | 7.047382917 | 8.124779881 | 6.871475287 |
| ENSMUSG00000019890 | Nts | 1172 | 0 | 0 | 0 | 0 | 0 | 0 | 0 | 0 | 0 | 0 | 0 | 0 | 0 | 0 | 0 | 0 | 0 | 0 |

| ENSMUSG00000019891 | Dcbld1 | 5328 | 0.127643171 | 0.375145205 | 0.308689946 | 0.359152377 | 0.289080623 | 0.344116205 | 0.452880373 | 0.186374336 | 0.479272518 | 0.194691342 | 0.430405031 | 0.439988331 | 0.092589935 | 0.148188717 | 0.108262347 | 0.06468008 | 0.201414473 | 0.619471923 |
| --- | --- | --- | --- | --- | --- | --- | --- | --- | --- | --- | --- | --- | --- | --- | --- | --- | --- | --- | --- | --- |
| ENSMUSG00000019892 | Lrriq1 | 5523 | 0.230880913 | 0.536818321 | 0.630616365 | 0.255729201 | 0.374737048 | 0.10766482 | 0.676752061 | 0.311181986 | 0.305748171 | 0.230503166 | 0.121102556 | 0.474983911 | 0.321555137 | 0.630691029 | 0.268559843 | 0.464506735 | 0.388606306 | 0.378480184 |
| ENSMUSG00000019893 | Ros1 | 8131 | 0.162053924 | 0.237627335 | 0.220123063 | 0.537924026 | 0.088793366 | 0.255960498 | 0.034912812 | 0.201976793 | 0.101307381 | 0 | 0 | 0.006864551 | 0.036402841 | 0.028559886 | 0 | 0 | 0 | 0 |
| ENSMUSG00000019894 | Slc6a15 | 3594 | 0.070960227 | 0.222456723 | 0.067297622 | 0 | 0.013392299 | 0.082725766 | 0 | 0 | 0.057298992 | 0 | 0 | 0 | 0 | 0 | 0 | 0 | 0 | 0 |
| ENSMUSG00000019897 | Ccdc59 | 4380 | 15.46877867 | 13.2719179 | 11.53012915 | 12.49287997 | 11.81320044 | 11.21158881 | 14.63668492 | 14.27419241 | 16.6720759 | 12.64887587 | 16.62306774 | 17.03779472 | 12.5920282 | 17.02949871 | 15.69986228 | 13.66397702 | 14.22798145 | 16.00036575 |
| ENSMUSG00000019899 | Lama2 | 9727 | 0.004369814 | 0.027398289 | 0.019892477 | 0.014051931 | 0.014844841 | 0.015283047 | 0.024320283 | 0.031411455 | 0.01270274 | 0.009694809 | 0.004911582 | 0.011476439 | 0.040573182 | 0.014324279 | 0.033886342 | 0.01968264 | 0.015760788 | 0.011310601 |
| ENSMUSG00000019900 | Rfx6 | 4206 | 0 | 0 | 0 | 0 | 0 | 0 | 0 | 0 | 0 | 0 | 0 | 0 | 0 | 0 | 0 | 0 | 0 | 0 |
| ENSMUSG00000019905 | Gprc6a | 2850 | 0 | 0 | 0 | 0 | 0.033776789 | 0 | 0 | 0 | 0 | 0 | 0.016763143 | 0 | 0 | 0.293331078 | 0 | 0 | 0 | 0 |
| ENSMUSG00000019906 | Lin7a | 5945 | 0 | 0 | 0 | 0 | 0.048577215 | 0 | 0 | 0 | 0 | 0 | 0 | 0 | 0 | 0.21874423 | 0 | 0 | 0 | 0.009253004 |
| ENSMUSG00000019907 | Ppp1r12a | 3502 | 46.81395315 | 42.10247567 | 42.1576284 | 34.73671855 | 35.84467645 | 37.55367074 | 27.80396723 | 34.38624922 | 37.43480288 | 36.97196777 | 35.96081969 | 35.65380845 | 42.92243059 | 41.0861744 | 38.58969255 | 51.42225203 | 47.62879615 | 38.54721528 |
| ENSMUSG00000019909 | Fam162b | 601 | 0 | 0 | 0 | 0 | 0 | 0.247351416 | 0 | 0 | 0 | 0 | 0 | 0 | 0 | 0 | 0 | 0 | 0 | 0 |
| ENSMUSG00000019913 | Sim1 | 7344 | 2.702875436 | 2.426797169 | 2.529334921 | 2.742099891 | 2.95581394 | 2.321097542 | 1.739436725 | 1.960584927 | 2.792876477 | 1.893989306 | 2.32889912 | 2.698061032 | 1.222550238 | 1.195252249 | 1.284743505 | 1.454667683 | 1.362086532 | 1.198455484 |
| ENSMUSG00000019916 | P4ha1 | 4210 | 7.006791488 | 6.385630792 | 9.019767537 | 6.514904677 | 7.065446277 | 6.073446681 | 14.93552264 | 11.20370949 | 15.11476647 | 11.82687474 | 9.611731551 | 9.784313299 | 9.995279201 | 4.710599837 | 9.649580548 | 7.148792736 | 7.710777548 | 7.330192131 |
| ENSMUSG00000019917 | 10-Sep | 2633 | 12.3172994 | 10.07104588 | 9.167638301 | 11.47245438 | 10.85847423 | 10.20037281 | 11.42835693 | 9.167337 | 8.509481418 | 12.62485598 | 6.132903899 | 7.970637726 | 7.850388659 | 7.990570295 | 9.341940322 | 11.48863648 | 10.72786966 | 9.06720559 |
| ENSMUSG00000019920 | Lims1 | 5169 | 17.29316796 | 14.6489086 | 14.86112714 | 14.4818719 | 15.29904248 | 15.75065608 | 14.52606335 | 14.94003437 | 15.06746011 | 16.52874334 | 16.07286763 | 17.75216663 | 16.83526835 | 12.87567754 | 15.29614737 | 15.27475962 | 15.63748504 | 17.30408348 |
| ENSMUSG00000019923 | Zwint | 3616 | 88.00781331 | 102.1312902 | 91.4093841 | 104.4023267 | 97.86114569 | 103.4769611 | 89.43090721 | 81.12716894 | 70.53874229 | 85.57800483 | 86.23538551 | 78.2592393 | 99.31868301 | 92.4643211 | 93.60364537 | 102.1224054 | 101.4544563 | 103.8114337 |
| ENSMUSG00000019927 | Ube2d1 | 4580 | 9.438376424 | 9.862944227 | 10.4034776 | 11.53947848 | 9.258564356 | 8.493208703 | 10.41292148 | 10.24023366 | 11.46567851 | 8.225635787 | 8.44928305 | 8.165173348 | 8.789267358 | 9.126567391 | 7.196778322 | 9.020865805 | 7.305427335 | 7.362572581 |
| ENSMUSG00000019929 | Dcn | 1928 | 302.1659454 | 363.6427239 | 360.0917571 | 290.6407076 | 324.2663675 | 440.0118251 | 140.1220937 | 195.7558717 | 218.9208576 | 182.317683 | 171.1770815 | 213.5933395 | 75.53310156 | 89.34705401 | 71.67532078 | 86.74967486 | 91.87974289 | 60.6869139 |
| ENSMUSG00000019932 | Kera | 2137 | 0 | 0 | 0 | 0 | 0 | 0 | 0 | 0 | 0 | 0 | 0 | 0 | 0 | 0 | 0 | 0 | 0 | 0 |
| ENSMUSG00000019933 | 2310015B20R | 422 | 0 | 0 | 0 | 0 | 0 | 0 | 0 | 0.724026586 | 0.195196757 | 0.111731524 | 0 | 0.39679381 | 0 | 0 | 0.097634019 | 0 | 0 | 0 |
| ENSMUSG00000019935 | Slc17a8 | 5351 | 0 | 0 | 0 | 0 | 0 | 0.027781387 | 0 | 0 | 0 | 0 | 0 | 0 | 0 | 0 | 0 | 0 | 0 | 0 |
| ENSMUSG00000019936 | Epyc | 2553 | 0 | 0 | 0 | 0 | 0 | 0 | 0 | 0 | 0 | 0 | 0 | 0 | 0 | 0 | 0 | 0 | 0 | 0 |
| ENSMUSG00000019942 | Cdk1 | 3703 | 0 | 0.161931433 | 0.078380011 | 0 | 0.038994267 | 0.160581367 | 0.600511993 | 0.288789432 | 0.300306758 | 0.076398655 | 0 | 0.286388763 | 0.972516868 | 0.990840644 | 0.723224719 | 0.537702291 | 0.900455801 | 0.386237314 |
| ENSMUSG00000019943 | Atp2b1 | 7123 | 24.28099976 | 21.19996483 | 25.06622217 | 22.04814261 | 24.60316357 | 24.13286997 | 27.60515143 | 32.93243515 | 33.28226656 | 32.22372925 | 36.56062034 | 34.27456242 | 42.2953838 | 38.00030062 | 38.65646469 | 48.06887707 | 46.84923026 | 38.89173964 |
| ENSMUSG00000019944 | Rhobtb1 | 6058 | 6.560306964 | 8.385962972 | 5.845068376 | 5.903832916 | 6.213133758 | 6.691009585 | 3.389518431 | 4.696820699 | 5.921666429 | 4.413081668 | 4.802731877 | 4.671267899 | 5.708430837 | 5.351266589 | 4.726820955 | 6.004634703 | 5.852067454 | 4.594686035 |
| ENSMUSG00000019945 | 1700040L02R | 1254 | 12.27023423 | 8.686855191 | 10.99398417 | 12.97072793 | 13.08850556 | 11.30150064 | 11.80930503 | 16.23329385 | 16.19214695 | 18.31131777 | 18.85853624 | 15.8455948 | 20.33859747 | 17.6294941 | 14.81810343 | 23.78658945 | 20.41624109 | 19.12597331 |
| ENSMUSG00000019947 | Arid5b | 6839 | 10.85780707 | 9.215966642 | 9.867096775 | 9.693130503 | 9.662981605 | 10.80320599 | 7.436924967 | 8.399089519 | 7.190627258 | 8.507671855 | 8.564424491 | 9.524327861 | 9.240267003 | 7.809723717 | 8.940358085 | 12.31189438 | 9.330791686 | 11.18038578 |
| ENSMUSG00000019948 | Actr6 | 1692 | 17.48439865 | 12.08872166 | 15.63848765 | 13.2213196 | 16.38533572 | 14.29180496 | 11.38076851 | 14.98803499 | 10.51570615 | 12.76301542 | 12.33904069 | 15.30642284 | 15.27773325 | 14.9598272 | 12.17540068 | 15.23024751 | 14.40633855 | 15.60542035 |
| ENSMUSG00000019951 | Uhrf1bp1l | 6475 | 11.05462802 | 13.76246981 | 12.68544472 | 14.67804455 | 12.54031741 | 14.28037079 | 12.61184306 | 10.94160718 | 8.43448956 | 9.553934004 | 10.13788308 | 9.611500198 | 9.75971985 | 11.5339139 | 10.61377532 | 11.26542195 | 11.47125303 | 14.44254539 |
| ENSMUSG00000019952 | Poc1b | 3876 | 1.94102584 | 2.690127939 | 3.020225579 | 3.996582836 | 2.37182596 | 2.467409077 | 3.002817084 | 4.089227812 | 4.643577757 | 3.625105668 | 3.845662292 | 3.902488277 | 1.463666276 | 2.468390609 | 2.327951687 | 3.220520643 | 3.272962919 | 1.830798444 |
| ENSMUSG00000019960 | Dusp6 | 2793 | 4.276388995 | 3.435056762 | 4.087416099 | 6.590283534 | 5.342247167 | 5.819297033 | 10.62121364 | 12.01973567 | 10.64685441 | 14.48453398 | 14.57367154 | 17.42615746 | 14.2361351 | 10.11027753 | 10.35570793 | 19.49489571 | 17.11166926 | 15.51993416 |
| ENSMUSG00000019961 | Tmpo | 7798 | 12.40052263 | 11.26093731 | 13.56667234 | 13.59002169 | 12.46813103 | 14.03718754 | 11.43076264 | 12.68998649 | 10.67955051 | 12.86697827 | 15.5614766 | 10.56474968 | 9.51464524 | 9.315023426 | 12.07832223 | 11.25443347 | 11.69743353 | 13.14913759 |
| ENSMUSG00000019966 | Kitl | 5922 | 0 | 0 | 0 | 0.02308057 | 0 | 0 | 0.183754072 | 0.077390886 | 0 | 0.031847824 | 0.008067369 | 0.263903842 | 0.58311959 | 0.55682712 | 0.166976924 | 0.006465824 | 0.071190352 | 0.046444703 |
| ENSMUSG00000019969 | Psen1 | 3102 | 40.35388246 | 47.71411638 | 46.58018542 | 46.70667988 | 46.51821636 | 45.5910708 | 35.87344321 | 40.51940565 | 39.20818216 | 46.55789944 | 43.35477373 | 38.38001557 | 43.68625943 | 45.26127536 | 44.42914392 | 51.64664777 | 50.44689563 | 38.4815479 |
| ENSMUSG00000019970 | Sgk1 | 5223 | 4.956088876 | 4.892014142 | 2.472857095 | 4.03009812 | 4.967089193 | 3.434441806 | 7.30117159 | 12.28474747 | 5.740720558 | 7.754634137 | 11.58014502 | 7.865274299 | 15.29166649 | 23.84005328 | 13.16588106 | 10.08033413 | 18.84394609 | 25.15063122 |
| ENSMUSG00000019971 | Cep290 | 77413 | 0.282222114 | 0.266372334 | 0.289317615 | 0.260136949 | 0.282276793 | 0.274606626 | 0.308641996 | 0.253093181 | 0.250056998 | 0.272867801 | 0.346834855 | 0.276147401 | 0.30333397 | 0.377969786 | 0.312951505 | 0.27352907 | 0.34210625 | 0.194702379 |
| ENSMUSG00000019975 | Ikbip | 2943 | 5.517151628 | 5.206908373 | 4.58586985 | 6.393717741 | 4.710157668 | 5.270134424 | 8.745530865 | 5.697065802 | 6.409590289 | 8.667526491 | 5.665463985 | 7.017259652 | 4.073277573 | 4.276706654 | 4.101955785 | 5.204295943 | 3.320831026 | 5.065398546 |
| ENSMUSG00000019977 | Hbs1l | 4874 | 14.08409095 | 17.43561616 | 15.7903748 | 18.39641675 | 17.39029905 | 17.5681419 | 13.2502681 | 15.49166589 | 12.33736418 | 16.12643052 | 14.70300322 | 14.07415866 | 16.14370292 | 16.3612019 | 14.35376321 | 14.61233684 | 15.96273753 | 12.95659715 |
| ENSMUSG00000019978 | Epb4.1l2 | 4993 | 15.70639891 | 17.79400939 | 16.93509535 | 16.91772005 | 20.20518967 | 17.87391812 | 17.03749183 | 20.44628312 | 19.53328046 | 19.21724036 | 20.20843427 | 17.07000136 | 11.44128985 | 11.87844975 | 15.48049647 | 16.26564757 | 16.05820411 | 13.2978153 |
| ENSMUSG00000019979 | Apaf1 | 11277 | 0.772684321 | 0.646938355 | 0.776412964 | 0.852476754 | 0.49937351 | 0.615179809 | 0.654498352 | 0.528333313 | 0.339660013 | 0.539366917 | 0.584636363 | 0.905760952 | 0.419957795 | 0.333596797 | 0.314208904 | 0.716442153 | 0.700116801 | 0.634138856 |
| ENSMUSG00000019982 | Myb | 3761 | 0 | 0 | 0.01286188 | 0.024228154 | 0.166369319 | 0 | 0.49061273 | 0.365574711 | 0.175215169 | 0.025073493 | 0 | 0 | 0.131167023 | 1.457166262 | 0.525837459 | 0.030542893 | 0.346475431 | 0.438785749 |

| ENSMUSG00000019984 | Med23 | 6770 | 4.834414408 | 5.201141682 | 4.101389427 | 4.347479267 | 5.168671862 | 5.379801947 | 4.137238689 | 5.274726182 | 6.223604967 | 3.844488656 | 4.81278016 | 4.254192303 | 4.437686502 | 3.759432775 | 4.05929657 | 3.47273751 | 4.25721939 | 3.63206362 |
| --- | --- | --- | --- | --- | --- | --- | --- | --- | --- | --- | --- | --- | --- | --- | --- | --- | --- | --- | --- | --- |
| ENSMUSG00000019986 | Ahi1 | 5161 | 2.363686402 | 2.181700881 | 2.73688643 | 2.286438768 | 1.846564791 | 2.265926201 | 3.80444909 | 2.989678468 | 3.447505291 | 3.617841211 | 2.462340429 | 3.709508287 | 4.2726927 | 5.33643554 | 4.143307016 | 5.586674554 | 3.616529082 | 4.508593711 |
| ENSMUSG00000019987 | Arg1 | 1468 | 0.318499275 | 0.204234365 | 0.065903992 | 0.310361337 | 0.327874139 | 0.168776341 | 0.064458691 | 0.078049869 | 0 | 0.064238015 | 0.390530996 | 0.228129411 | 0.100814545 | 0.063275323 | 0.252598095 | 0 | 0.026107832 | 0.374721435 |
| ENSMUSG00000019988 | Nedd1 | 3519 | 7.102314153 | 7.611130137 | 10.62595594 | 7.030306074 | 8.658001615 | 9.476837084 | 9.075322045 | 7.662357516 | 7.724666213 | 7.489980995 | 8.688824503 | 8.10508769 | 6.743010006 | 7.681289815 | 8.301194409 | 8.139066308 | 6.60009546 | 7.816014027 |
| ENSMUSG00000019989 | Enpp3 | 2790 | 0 | 0 | 0.034676366 | 0 | 0 | 0.071043346 | 0 | 0.027378066 | 0 | 0 | 0 | 0.68019087 | 0.088408454 | 0 | 0 | 0 | 0 | 0 |
| ENSMUSG00000019990 | Pde7b | 8920 | 0.328795644 | 0.365993681 | 0.097614747 | 0.214525097 | 0.388508801 | 0.194433372 | 0.18564392 | 0.4752642 | 0.34629918 | 0.311871244 | 0.444542775 | 0.337897509 | 0.464560656 | 0.421745637 | 0.083142153 | 0.622437004 | 0.352326945 | 0.228176788 |
| ENSMUSG00000019992 | Fam54a | 2448 | 0.104179353 | 0.054432834 | 0.355687723 | 0.018611538 | 0.039323467 | 0 | 0.057981224 | 0.093608829 | 0 | 0.057782725 | 0.117095486 | 0.045601032 | 0.120911562 | 0.284583869 | 0.033661402 | 0 | 0.234842506 | 0.269652484 |
| ENSMUSG00000019996 | Mtap7 | 4330 | 21.44891677 | 23.40365455 | 23.81809855 | 23.66438538 | 23.41012269 | 24.6161501 | 19.5697478 | 22.26273064 | 20.5076508 | 23.68424471 | 22.13315628 | 25.20083611 | 24.15327128 | 19.83259067 | 19.11638009 | 21.40913638 | 22.13719828 | 21.03812488 |
| ENSMUSG00000019997 | Ctgf | 2915 | 20.88075886 | 40.02118544 | 24.59333516 | 35.97993964 | 45.20933345 | 36.6503237 | 19.96384051 | 41.10105192 | 32.82205013 | 23.16288407 | 32.63119806 | 38.00826423 | 16.75423606 | 14.24391565 | 19.06720374 | 20.53112157 | 25.84888498 | 24.79655788 |
| ENSMUSG00000019998 | Stx7 | 2490 | 440.6369915 | 410.6449699 | 442.744079 | 437.2579424 | 419.4629492 | 436.0640555 | 436.0556902 | 406.7107398 | 409.9629691 | 431.4762749 | 408.8884097 | 413.1031509 | 274.8322717 | 264.4328622 | 286.7562103 | 246.8437068 | 312.7979942 | 266.8052136 |
| ENSMUSG00000020000 | Moxd1 | 3507 | 0.303002395 | 0.161482521 | 0.110347375 | 0.779487499 | 0.507807578 | 0.211944968 | 0.080945558 | 0.653420058 | 0.563716212 | 0.497455381 | 0.231586625 | 0.891268065 | 0.112533601 | 0.331081319 | 0.234967527 | 0 | 0 | 0 |
| ENSMUSG00000020003 | Pex7 | 3238 | 27.04158821 | 26.52274283 | 27.93653544 | 28.88722175 | 27.61862698 | 25.6639698 | 29.69097084 | 29.62918927 | 28.42861727 | 26.29838483 | 30.67453325 | 23.87421022 | 26.47895992 | 23.99658993 | 24.29084935 | 26.07498126 | 28.47840344 | 24.34467262 |
| ENSMUSG00000020007 | Il20ra | 2081 | 0 | 0 | 0 | 0.043787645 | 0 | 0.071435945 | 0 | 0 | 0.41562558 | 0 | 0 | 0 | 0 | 0 | 0.098994608 | 0 | 0 | 0 |
| ENSMUSG00000020009 | Ifngr1 | 3078 | 71.13195637 | 71.50691751 | 73.66040831 | 74.17361701 | 77.48332741 | 70.48143847 | 74.53514624 | 78.97811608 | 83.38998253 | 74.24933681 | 85.16608095 | 90.63245016 | 55.7909045 | 44.60322363 | 49.87556766 | 58.74212091 | 63.3417389 | 58.72642128 |
| ENSMUSG00000020010 | Vnn3 | 1804 | 0 | 0.036932255 | 0 | 0 | 0 | 0 | 0 | 0 | 0.342459943 | 0 | 0 | 0 | 0 | 0 | 0 | 0 | 0 | 0 |
| ENSMUSG00000020014 | Gm872 | 14383 | 0.070925692 | 0.118122617 | 0.057175138 | 0.057018619 | 0 | 0.062014128 | 0.029605375 | 0.002655385 | 0.083043103 | 0 | 0 | 0 | 0.092606672 | 0.251869486 | 0.103125635 | 0.010648851 | 0.042635107 | 0.11473776 |
| ENSMUSG00000020015 | Cdk17 | 5431 | 7.795093965 | 8.918605817 | 8.942556523 | 7.483051288 | 8.676330928 | 7.016397003 | 5.078860581 | 5.562546548 | 6.324719968 | 4.193295834 | 4.354373862 | 5.005013382 | 3.061104055 | 2.702325836 | 4.058706021 | 4.258428811 | 5.342111353 | 5.28719457 |
| ENSMUSG00000020017 | Hal | 6102 | 0.062692 | 0.049134062 | 0.206114682 | 0.037332878 | 0.149869969 | 0.129931783 | 0.170579963 | 0.582086762 | 0.357732766 | 0.10817926 | 0.219223015 | 0.201236411 | 0 | 0 | 0 | 0.006275091 | 0 | 0 |
| ENSMUSG00000020018 | Snrpf | 857 | 6.894071724 | 4.975554796 | 5.983190452 | 4.891033931 | 5.784816964 | 5.377367821 | 4.361378804 | 3.966305501 | 5.334542882 | 5.281759058 | 5.463180783 | 5.405717613 | 5.641222746 | 6.557449911 | 4.471114 | 6.701973291 | 4.293260797 | 5.905295004 |
| ENSMUSG00000020019 | Ntn4 | 3730 | 0.923034654 | 2.384595913 | 1.011564442 | 1.135972419 | 1.483960113 | 1.275351856 | 0.228318558 | 0.276459749 | 0.033125884 | 0.16433221 | 0.0256166 | 0.224459769 | 0.105805721 | 0.323738947 | 0.265103845 | 0.349029665 | 0.123301759 | 0.01474775 |
| ENSMUSG00000020020 | Usp44 | 2928 | 0 | 0 | 0 | 0 | 0 | 0 | 0 | 0 | 0.14066433 | 0.016103382 | 0 | 0 | 0 | 0 | 0 | 0 | 0.013089582 | 0 |
| ENSMUSG00000020021 | Fgd6 | 8219 | 4.126916957 | 5.325847792 | 4.408294712 | 3.525589998 | 6.143130299 | 3.599340789 | 2.434999779 | 3.731414908 | 2.726057254 | 2.495505039 | 4.150300566 | 3.171421636 | 3.187157571 | 2.74629839 | 2.416248928 | 2.282807839 | 3.972990018 | 2.99842801 |
| ENSMUSG00000020022 | Ndufa12 | 1422 | 56.7632414 | 50.92983969 | 47.96531491 | 62.47822535 | 49.1473651 | 65.40821443 | 66.61039607 | 54.92507941 | 56.45043546 | 73.74343463 | 55.06551121 | 58.87728121 | 66.81664745 | 63.13391064 | 49.951816 | 80.53952654 | 50.56268143 | 76.51759008 |
| ENSMUSG00000020023 | Tmcc3 | 7269 | 10.32661176 | 12.60747998 | 10.15518054 | 17.26794293 | 12.60077738 | 16.31306802 | 3.944350451 | 4.744495718 | 4.776479954 | 5.17626375 | 3.52939231 | 7.271623672 | 5.218908294 | 4.676994355 | 3.344190119 | 8.470394374 | 4.265507524 | 5.267070887 |
| ENSMUSG00000020024 | Ccdc41 | 2970 | 5.280946113 | 5.428767947 | 5.798308689 | 6.488997224 | 6.061057054 | 4.921904514 | 6.754402648 | 6.159650085 | 6.864419294 | 4.318178884 | 5.758732369 | 5.243289516 | 5.94640619 | 5.066627715 | 6.658837316 | 5.517973053 | 5.703778866 | 4.204399739 |
| ENSMUSG00000020027 | Socs2 | 10122 | 3.309037612 | 4.712908963 | 3.794564622 | 3.569443604 | 3.737570836 | 3.701034043 | 0.878757522 | 1.331942111 | 1.477050506 | 1.294990664 | 1.548131435 | 1.16351559 | 0.974746439 | 1.367352108 | 1.111245283 | 1.320235327 | 1.78341097 | 1.233656118 |
| ENSMUSG00000020029 | Nudt4 | 3226 | 1.778734893 | 4.047940029 | 3.448825785 | 3.022338341 | 2.402120183 | 3.932269001 | 1.363942697 | 2.569048767 | 2.515109611 | 2.601619709 | 1.332841371 | 2.370348969 | 2.936059553 | 5.499578854 | 4.968197533 | 5.341219259 | 3.813620988 | 4.996177393 |
| ENSMUSG00000020032 | Nuak1 | 5709 | 0.230804074 | 0.250911622 | 0.24572296 | 0.19951412 | 0.185479475 | 0.225673686 | 0 | 0.026759434 | 0.064928821 | 0.049554076 | 0.192472245 | 0.029330353 | 0.259232356 | 0.170840048 | 0.064952532 | 0.241454172 | 0.046993182 | 0.144532598 |
| ENSMUSG00000020033 | 4930463O16R | 2531 | 0.016793827 | 0 | 0 | 0 | 0 | 0 | 0 | 0 | 0 | 0 | 0 | 0 | 0.136437543 | 0.055050281 | 0 | 0.015128648 | 0 | 0 |
| ENSMUSG00000020034 | Tcp11l2 | 3028 | 19.27331793 | 24.36858015 | 16.93393071 | 25.57918605 | 22.17438359 | 20.27603597 | 18.25006902 | 21.75756082 | 21.32775981 | 23.2172056 | 25.37058561 | 22.41474429 | 20.23455788 | 20.04703508 | 21.66211261 | 30.24806239 | 28.1118578 | 21.36417091 |
| ENSMUSG00000020037 | Rfx4 | 4702 | 0 | 0.049593845 | 0 | 0 | 0.061418873 | 0 | 0 | 0.324903466 | 0 | 0.020055595 | 0 | 0.023741243 | 0 | 0.325958078 | 0.140200956 | 0.008143472 | 0.008151063 | 0 |
| ENSMUSG00000020038 | Cry1 | 3001 | 11.95413814 | 13.0321019 | 12.26666331 | 10.80955134 | 12.1091644 | 13.37477981 | 11.44585302 | 16.64633867 | 11.50093309 | 11.34382131 | 11.73280386 | 9.615693954 | 10.84940533 | 11.45239076 | 14.27844656 | 15.1963057 | 17.98181474 | 16.77218682 |
| ENSMUSG00000020042 | Btbd11 | 11664 | 0.069238541 | 0.48838348 | 0.062208758 | 0.121089881 | 0.39614752 | 0.339867857 | 0.146026787 | 0.189914209 | 0.211864793 | 0.088933082 | 0.188412902 | 0 | 0.228388506 | 0.342437544 | 0.388560627 | 0.265906996 | 0.190580009 | 0.391439974 |
| ENSMUSG00000020044 | Timp3 | 8556 | 9.62775025 | 18.8095596 | 13.25805618 | 13.80250431 | 19.57117373 | 16.03687696 | 3.129847624 | 5.42799922 | 4.125390838 | 2.854612469 | 3.439618329 | 4.938342284 | 0.968649147 | 1.346205432 | 1.300189351 | 2.354004148 | 2.772788427 | 2.333836576 |
| ENSMUSG00000020048 | Hsp90b1 | 4304 | 117.2842636 | 125.8211915 | 134.4433481 | 129.0296397 | 123.0137454 | 135.4986342 | 127.2627518 | 131.8720474 | 133.8083294 | 131.4391584 | 133.9785662 | 128.1140638 | 117.3352317 | 120.4913289 | 136.8343494 | 117.8343622 | 129.6273011 | 129.9564584 |
| ENSMUSG00000020051 | Pah | 2333 | 0 | 0 | 0 | 0 | 0 | 0 | 0 | 0 | 0 | 0 | 0 | 0 | 0 | 0 | 0 | 0 | 0 | 0 |
| ENSMUSG00000020052 | Ascl1 | 2479 | 0 | 0 | 0 | 0 | 0 | 0 | 0 | 0 | 0 | 0 | 0 | 0 | 0 | 0 | 0 | 0 | 0 | 0 |
| ENSMUSG00000020053 | Igf1 | 9913 | 0 | 0 | 0 | 0 | 0 | 0 | 0 | 0.026969315 | 0 | 0 | 0.00963885 | 0 | 0 | 0 | 0 | 0 | 0 | 0 |
| ENSMUSG00000020056 | Ccdc53 | 1452 | 31.52759957 | 21.40559938 | 24.22008028 | 22.498119 | 24.89467953 | 23.61604107 | 34.11596063 | 24.93553545 | 32.39325138 | 28.15403562 | 23.95328497 | 25.75516111 | 31.22316252 | 32.49806666 | 27.80821265 | 25.71166819 | 29.72135696 | 30.3838179 |
| ENSMUSG00000020057 | Dram1 | 3036 | 0.686018981 | 0.735165979 | 0.477999312 | 0.810374306 | 0.348782056 | 0.620225256 | 0 | 0.037739528 | 0.271320921 | 0.217427485 | 0 | 0 | 0.487469538 | 0.015297789 | 0.284990999 | 0.138734085 | 0 | 0 |
| ENSMUSG00000020059 | Sycp3 | 1304 | 0.162979969 | 0 | 0.148385062 | 0 | 0 | 0 | 0.036282729 | 0.087865956 | 0 | 0 | 0.18318619 | 0.513641067 | 0.1513249 | 0 | 0.221173996 | 0.088091888 | 0 | 0 |

| ENSMUSG00000020061 | Mybpc1 | 4782 | 0 | 0 | 0 | 0.009527613 | 0 | 0 | 0.00989391 | 0 | 0.189482088 | 0 | 0 | 0 | 0.051580842 | 0 | 0 | 0 | 0 | 0 |
| --- | --- | --- | --- | --- | --- | --- | --- | --- | --- | --- | --- | --- | --- | --- | --- | --- | --- | --- | --- | --- |
| ENSMUSG00000020062 | Slc5a8 | 5306 | 0 | 0.012556688 | 0 | 0.077280324 | 0 | 0.028017 | 0 | 0 | 0 | 0 | 0 | 0 | 0 | 0.297606289 | 0 | 0 | 0 | 0 |
| ENSMUSG00000020063 | Sirt1 | 7273 | 2.933809757 | 2.510032453 | 2.766862179 | 2.493234655 | 2.203757814 | 2.350569653 | 3.089993468 | 3.581358235 | 3.595962809 | 3.137761636 | 2.85086374 | 2.570912549 | 2.306180651 | 3.295084435 | 2.237675591 | 3.369447097 | 3.103834577 | 3.138839446 |
| ENSMUSG00000020064 | Herc4 | 3896 | 10.84449306 | 9.593703109 | 12.34170667 | 12.38427771 | 10.34045691 | 12.66799863 | 8.998638369 | 8.969724898 | 10.9309182 | 10.94051225 | 9.797790509 | 9.369466974 | 7.458033695 | 7.462525335 | 8.915018382 | 9.680761882 | 7.555081114 | 8.457508965 |
| ENSMUSG00000020067 | Mypn | 5226 | 0 | 0 | 0 | 0 | 0 | 0 | 0.009053325 | 0 | 0 | 0 | 0 | 0 | 0.009439709 | 0 | 0 | 0 | 0.022001319 | 0 |
| ENSMUSG00000020068 | Pbld2 | 2469 | 1.222303563 | 0.823040319 | 1.038394941 | 1.605431696 | 1.013714067 | 0.561958907 | 2.069570403 | 1.80984653 | 2.268678065 | 1.81422309 | 1.43189426 | 2.780610165 | 1.138889949 | 0.95935539 | 2.002505754 | 1.969585719 | 1.955898505 | 2.562190066 |
| ENSMUSG00000020069 | Hnrnph3 | 3429 | 3.284885286 | 2.263605819 | 2.736793493 | 2.657395412 | 2.709087567 | 2.716801958 | 2.193851254 | 2.673134027 | 2.834650837 | 2.942621901 | 2.647198049 | 2.929956041 | 2.172388308 | 1.435716904 | 1.694202211 | 2.724674309 | 2.82780785 | 2.261966768 |
| ENSMUSG00000020070 | Rufy2 | 10654 | 4.061410662 | 3.623957612 | 3.995560983 | 4.37906039 | 4.02078206 | 4.404584072 | 3.779152409 | 4.592121971 | 4.720174183 | 4.222054713 | 4.744312565 | 5.511364468 | 3.82931252 | 3.901582337 | 3.940715736 | 3.737772827 | 3.665712084 | 3.376755753 |
| ENSMUSG00000020072 | Pbld1 | 2395 | 0 | 0.055637402 | 0.16158173 | 0.076093602 | 0.261258876 | 0.041380153 | 0.19754772 | 0.494348424 | 0.0687875 | 0.255932836 | 0.478746974 | 0.466101566 | 0.102989389 | 0.969605165 | 0.516094646 | 0.31975455 | 0.848139347 | 0.757954289 |
| ENSMUSG00000020074 | Ccar1 | 4453 | 25.01820489 | 26.24334896 | 24.20305988 | 24.88310349 | 23.42283373 | 26.71819298 | 21.33479885 | 24.34090232 | 22.78068455 | 19.78995383 | 23.84992874 | 21.69703839 | 22.09024097 | 24.94818258 | 25.62028032 | 25.43535071 | 28.56613057 | 21.12408994 |
| ENSMUSG00000020075 | Ddx21 | 4727 | 8.047838491 | 7.653438359 | 9.517110901 | 7.749329309 | 6.262139424 | 7.778322047 | 6.065471426 | 6.108198904 | 8.495208984 | 6.363898599 | 8.014711672 | 6.246347685 | 5.061556987 | 6.219400744 | 5.726554309 | 5.662182055 | 5.351249409 | 7.715472341 |
| ENSMUSG00000020076 | Ddx50 | 3237 | 29.45292241 | 23.37151151 | 27.96010976 | 25.70110193 | 29.26278214 | 28.68761901 | 30.44557004 | 29.15459572 | 25.28192982 | 27.5300677 | 25.96112202 | 31.60645954 | 28.28546137 | 35.55404651 | 33.45001201 | 27.51435058 | 30.215851 | 26.83329611 |
| ENSMUSG00000020077 | Srgn | 2867 | 0.800585806 | 1.278136855 | 1.467909711 | 0.460854651 | 0.688318406 | 0.98517817 | 19.70398976 | 17.27783255 | 13.5325071 | 7.482933374 | 8.548501465 | 13.99771936 | 3.200466206 | 4.033685999 | 3.664596007 | 1.188651573 | 0.414410605 | 1.439024417 |
| ENSMUSG00000020078 | Vps26a | 4823 | 42.8753227 | 41.65665873 | 43.23829346 | 40.1292383 | 39.52945255 | 44.21012079 | 43.84981854 | 40.987741 | 44.31201126 | 43.33797793 | 43.35703779 | 43.59477524 | 41.85490473 | 42.56331459 | 44.12316736 | 45.0944744 | 47.2343995 | 44.6186244 |
| ENSMUSG00000020079 | Supv3l1 | 3467 | 6.546802423 | 7.071903705 | 7.073948627 | 6.675803439 | 6.816491065 | 5.259707516 | 5.854381096 | 7.138354992 | 6.498132138 | 6.895127358 | 7.826990602 | 6.874325908 | 6.659168528 | 8.077815026 | 4.527774097 | 6.217944034 | 7.174435157 | 6.600458147 |
| ENSMUSG00000020080 | Hkdc1 | 5116 | 0 | 0.052092094 | 0.104008763 | 0 | 0.009408116 | 0.048429177 | 0.027743948 | 0 | 0 | 0.064514254 | 0 | 0 | 0 | 0 | 0 | 0.022453445 | 0.037457288 | 0 |
| ENSMUSG00000020081 | Tacr2 | 2253 | 0 | 0.029572032 | 0 | 0.04044478 | 0 | 0.219941117 | 0 | 0 | 0 | 0 | 0.042410083 | 0 | 0.153272712 | 0.206143308 | 0.438898065 | 0.118967711 | 0 | 0 |
| ENSMUSG00000020083 | 2010107G23R | 4407 | 17.29334709 | 14.23375989 | 16.58552402 | 12.93325765 | 16.9504755 | 14.26875855 | 18.14350519 | 14.87137793 | 17.99047943 | 17.043583 | 19.77343414 | 16.65477565 | 18.31336888 | 12.04574356 | 17.21172326 | 17.70734238 | 18.12389443 | 15.06602946 |
| ENSMUSG00000020085 | Aifm2 | 4174 | 9.826903418 | 9.098394684 | 9.236632413 | 10.05311975 | 9.005997217 | 9.568639977 | 8.217942555 | 10.01932933 | 8.337950604 | 9.647029368 | 9.820535308 | 10.44370687 | 9.419630587 | 8.367502088 | 8.06460737 | 8.146157013 | 10.11873004 | 10.87266724 |
| ENSMUSG00000020086 | H2afy2 | 2337 | 18.55168145 | 20.04190384 | 18.36000061 | 20.15837392 | 21.17227964 | 20.0373698 | 11.58016788 | 13.02496565 | 10.29222302 | 13.43704252 | 13.57405752 | 11.249113 | 9.309103774 | 9.221247988 | 8.074588189 | 8.143103072 | 8.019494731 | 10.94532932 |
| ENSMUSG00000020087 | Tysnd1 | 2282 | 25.51800664 | 25.03576406 | 23.99598438 | 25.61560906 | 21.32400299 | 26.20953086 | 22.30869524 | 25.50623193 | 20.82788746 | 26.943259 | 26.94407167 | 24.99719858 | 21.66107849 | 23.30345314 | 22.20767474 | 20.9910385 | 19.66699986 | 26.29927057 |
| ENSMUSG00000020088 | Sar1a | 2604 | 96.84455045 | 91.23946297 | 90.44958498 | 93.65908999 | 87.46553871 | 97.41184454 | 81.87055732 | 93.41298422 | 84.30266091 | 83.21990483 | 89.67895432 | 80.8941285 | 71.55401365 | 77.08577024 | 81.26389825 | 79.84562142 | 90.25224755 | 77.21132299 |
| ENSMUSG00000020089 | Ppa1 | 3056 | 18.79073718 | 16.49293487 | 16.36722199 | 16.6679475 | 16.6477236 | 19.02007738 | 16.81334074 | 14.12219068 | 12.84383165 | 12.00368034 | 11.20898077 | 12.18227976 | 13.64053341 | 14.52897498 | 12.09351951 | 11.32680271 | 11.04890955 | 14.7422966 |
| ENSMUSG00000020090 | Npffr1 | 1303 | 0.81552525 | 1.201616292 | 0 | 0.594426519 | 0.295514497 | 0.076059453 | 0 | 0 | 0 | 0.28949012 | 0 | 0.299853905 | 0 | 0 | 0 | 0 | 0 | 0 |
| ENSMUSG00000020091 | Eif4ebp2 | 2780 | 10.68745253 | 11.83925881 | 11.36255947 | 11.27553903 | 9.349366468 | 13.90328496 | 10.2964643 | 9.328288213 | 10.25218306 | 11.1431698 | 7.750541825 | 10.15925369 | 9.795402279 | 8.988100365 | 10.70054797 | 9.57265185 | 8.685455773 | 9.359463122 |
| ENSMUSG00000020092 | X99384 | 4254 | 7.184114143 | 7.948422104 | 5.890335854 | 8.300378317 | 7.852269635 | 7.268666138 | 5.549841745 | 5.629204586 | 5.00550744 | 6.018531234 | 5.109921511 | 6.835909781 | 7.642156437 | 6.747166433 | 6.159894111 | 8.641039752 | 7.54993813 | 6.982820317 |
| ENSMUSG00000020096 | Tbata | 2477 | 0 | 0 | 0 | 0 | 0 | 0 | 0 | 0 | 0 | 0 | 0 | 0 | 0 | 0 | 0 | 0 | 0 | 0.022207956 |
| ENSMUSG00000020097 | Sgpl1 | 8479 | 14.98885203 | 19.07466699 | 19.38021967 | 20.4296604 | 20.49821634 | 21.88642378 | 15.53467368 | 15.9949547 | 18.99272044 | 17.8949125 | 20.38563507 | 17.20747046 | 20.26454392 | 15.98347057 | 19.90833524 | 20.28106119 | 23.54994774 | 18.2498664 |
| ENSMUSG00000020098 | Pcbd1 | 805 | 54.33270327 | 43.7412785 | 46.99142949 | 47.48536174 | 40.7776049 | 44.56668212 | 58.30332608 | 50.52783672 | 51.93082422 | 48.3807216 | 50.03017391 | 55.81566259 | 53.6829311 | 54.86748709 | 53.17815723 | 56.79377048 | 44.99173984 | 47.2189972 |
| ENSMUSG00000020099 | Unc5b | 5835 | 0.815866275 | 1.216049095 | 0.837313893 | 0.812056317 | 0.81663418 | 0.832248139 | 0.527047837 | 0.81162946 | 0.649384653 | 0.646453515 | 0.704138206 | 0.947000959 | 1.276627165 | 0.986986604 | 0.861454982 | 2.106475574 | 1.037798614 | 1.583809757 |
| ENSMUSG00000020100 | Slc29a3 | 8291 | 2.547952296 | 2.752301597 | 2.748031938 | 2.961934977 | 3.210342997 | 3.167645495 | 1.734779206 | 2.570421004 | 3.502170258 | 2.985661465 | 2.368291875 | 1.810929108 | 2.808426602 | 2.840085913 | 2.420113101 | 2.332258683 | 3.04169622 | 2.49468389 |
| ENSMUSG00000020101 | 4632428N05R | 5097 | 0 | 0.019607354 | 0 | 0.026816389 | 0.113318243 | 0.165272998 | 0.139236842 | 0.044958684 | 0 | 0.018501355 | 0.178089898 | 0 | 0 | 0 | 0 | 0.112685719 | 0 | 0.097132031 |
| ENSMUSG00000020102 | Slc16a7 | 4678 | 0 | 0 | 0 | 0 | 0.061733976 | 0 | 0.182049641 | 0.06531407 | 0.096847301 | 0 | 0 | 0 | 0.147637204 | 0 | 0 | 0 | 0 | 0 |
| ENSMUSG00000020105 | Lrig3 | 4031 | 0.506139531 | 0.942116086 | 0.588018603 | 0.734673253 | 0.38209416 | 0.565474013 | 0.269955747 | 0.435834907 | 0.194131431 | 0.549760122 | 0.734817024 | 0.110772836 | 0.293715211 | 0.44934741 | 0.490616394 | 0.6364353 | 0.294744531 | 0.518567615 |
| ENSMUSG00000020107 | Anapc16 | 1325 | 71.02374319 | 69.84393973 | 67.61341752 | 59.89987867 | 68.3654946 | 62.71693148 | 81.55634617 | 69.29398897 | 73.26537181 | 68.25286705 | 70.45454254 | 66.26266962 | 89.72824199 | 83.77461801 | 82.80735346 | 81.20498626 | 76.76829583 | 74.52177099 |
| ENSMUSG00000020108 | Ddit4 | 1682 | 12.76166105 | 12.00214857 | 10.95738113 | 12.43312684 | 12.10452064 | 12.4618349 | 10.97024066 | 8.855551093 | 10.06400593 | 10.56826107 | 13.34972086 | 11.91309326 | 13.40349952 | 13.58530975 | 12.19879598 | 12.04264644 | 13.28432289 | 16.45040469 |
| ENSMUSG00000020109 | Dnajb12 | 3606 | 8.734424691 | 9.238184753 | 8.491526543 | 9.273934149 | 8.996371756 | 7.915245297 | 9.906010147 | 8.091790193 | 9.445715068 | 9.218315529 | 8.571935146 | 6.779606271 | 6.840254753 | 9.775668976 | 11.28872358 | 8.006410976 | 9.406204315 | 8.435950088 |
| ENSMUSG00000020111 | Micu1 | 5268 | 18.20267219 | 20.67195353 | 20.68822397 | 21.84646887 | 21.78179689 | 23.58175837 | 21.25837339 | 20.4446801 | 19.02179059 | 20.3084559 | 20.35967764 | 21.45533727 | 20.25530316 | 19.84541395 | 19.73263582 | 22.46702116 | 22.58254092 | 22.45056558 |
| ENSMUSG00000020114 | Cand1 | 8341 | 14.76291751 | 14.59760561 | 15.33384657 | 16.94405461 | 13.82617062 | 16.62847396 | 10.85678785 | 13.19174096 | 9.87074032 | 13.25035948 | 13.03630326 | 13.99239305 | 9.392049471 | 11.92701537 | 12.20091632 | 11.7153375 | 13.63315225 | 11.96997107 |
| ENSMUSG00000020115 | Tbk1 | 3010 | 18.64014364 | 20.4304328 | 23.52785662 | 24.03685661 | 20.86782737 | 21.91185661 | 18.64213861 | 19.78138051 | 20.66167402 | 18.93860473 | 19.85597112 | 23.38323938 | 20.19166847 | 18.93252333 | 21.50420077 | 22.0838852 | 23.02124412 | 19.8105886 |
| ENSMUSG00000020116 | Pno1 | 1711 | 42.23191072 | 44.27441347 | 41.19242182 | 36.21450631 | 42.7870578 | 40.25616579 | 50.52031818 | 48.79520261 | 53.17417494 | 47.09558847 | 45.1502676 | 43.68040456 | 47.37132676 | 49.59283908 | 44.78953475 | 50.84527178 | 49.10066792 | 63.75397933 |

| ENSMUSG00000020120 | Plek | 7163 | 0.011867982 | 0 | 0 | 0 | 0 | 0 | 0.792617824 | 1.114367179 | 0.258745387 | 0.151398321 | 0.686977624 | 0.085714406 | 0.413222817 | 0.726195419 | 0.333615837 | 0.277971742 | 0 | 0 |
| --- | --- | --- | --- | --- | --- | --- | --- | --- | --- | --- | --- | --- | --- | --- | --- | --- | --- | --- | --- | --- |
| ENSMUSG00000020121 | Srgap1 | 9643 | 2.816634604 | 2.63932917 | 2.979765325 | 3.203400193 | 2.211183468 | 2.774911971 | 1.835003829 | 2.269443802 | 3.032502975 | 1.985189829 | 1.77861766 | 2.905679002 | 1.33523078 | 1.208904484 | 0.965628086 | 1.767014445 | 1.351336819 | 0.815752594 |
| ENSMUSG00000020122 | Egfr | 11655 | 0.350106984 | 0.354422899 | 0.572762522 | 0.59418951 | 0.569901799 | 0.391149849 | 0.081188638 | 0.104861165 | 0.053007099 | 0.271050804 | 0.204954777 | 0.038311909 | 0.224332185 | 0.179320801 | 0.3570448 | 0.246400305 | 0.174285177 | 0.207670587 |
| ENSMUSG00000020123 | Avpr1a | 2669 | 140.4470391 | 125.6878399 | 131.9080382 | 144.9964446 | 143.0793115 | 150.2548793 | 131.1601766 | 164.6609121 | 154.5304492 | 177.9148492 | 188.719141 | 188.2131747 | 155.185754 | 127.742992 | 181.1540871 | 158.8149209 | 179.6841338 | 159.173972 |
| ENSMUSG00000020124 | Usp15 | 3688 | 19.73125308 | 20.60377216 | 24.9737532 | 20.60624239 | 23.38731215 | 21.29638902 | 23.34844784 | 19.13762463 | 23.69788136 | 23.02560102 | 23.79680009 | 16.87485461 | 20.25176864 | 20.0863122 | 19.89695527 | 15.49066872 | 20.70118857 | 21.28470588 |
| ENSMUSG00000020125 | Elane | 915 | 0 | 0 | 0 | 0 | 0 | 0 | 0 | 0 | 0 | 0 | 0 | 0 | 0 | 0 | 0 | 0 | 0 | 0 |
| ENSMUSG00000020128 | Vps54 | 6209 | 11.59667711 | 14.9422468 | 12.70691384 | 14.04474776 | 11.41863804 | 13.68705719 | 12.4815861 | 14.46747149 | 12.74267946 | 11.86171662 | 12.35731733 | 12.58526778 | 11.54441549 | 7.966331627 | 12.67433915 | 10.02746459 | 13.75894923 | 10.33912507 |
| ENSMUSG00000020130 | Tbc1d15 | 3756 | 17.20124269 | 19.85824552 | 21.3018688 | 18.81394562 | 19.90119208 | 22.38844219 | 15.65752394 | 19.12670632 | 16.96366877 | 20.5499737 | 17.69301577 | 17.32722646 | 16.87739981 | 20.13071732 | 18.88953122 | 20.21572803 | 19.24478061 | 20.5771552 |
| ENSMUSG00000020131 | Pcsk4 | 3508 | 1.405530336 | 1.433935869 | 1.72368623 | 0.922130601 | 0.933001941 | 1.525568765 | 1.793781727 | 1.208482516 | 0.880555497 | 1.680113429 | 2.083685475 | 1.479719675 | 1.237516739 | 1.244510894 | 1.691283936 | 0.895048406 | 0.884957255 | 1.050630031 |
| ENSMUSG00000020132 | Rab21 | 1816 | 46.90549161 | 48.66690989 | 52.10276728 | 50.7042239 | 50.64983818 | 47.99738897 | 49.5272041 | 54.99621822 | 50.34915473 | 56.60161514 | 53.35221134 | 45.79589056 | 51.28780827 | 51.27775067 | 51.59269719 | 51.34230673 | 52.7830774 | 59.52251911 |
| ENSMUSG00000020133 | 2310011J03R | 1647 | 28.00128475 | 26.59772671 | 25.4643903 | 26.25223502 | 25.13263774 | 25.06219009 | 24.64740651 | 26.15727378 | 29.13314564 | 30.803981 | 31.61791423 | 24.29862178 | 26.74766371 | 23.94112337 | 21.7640277 | 29.27011215 | 23.13074628 | 27.48785357 |
| ENSMUSG00000020134 | Peli1 | 5625 | 14.00214954 | 15.43349375 | 12.76201228 | 15.65679977 | 13.56250649 | 14.94950913 | 12.57465867 | 16.07815269 | 16.12314804 | 16.11925375 | 19.26286326 | 18.93267275 | 15.55819044 | 16.55473693 | 15.09625008 | 20.25828402 | 20.91764115 | 18.0625458 |
| ENSMUSG00000020135 | Apc2 | 9834 | 0.458160327 | 0.457315509 | 0.354168618 | 0.643988729 | 0.278983084 | 0.463580587 | 0.442624208 | 0.365068723 | 0.498392859 | 0.445903539 | 0.233190767 | 1.106777934 | 0.260856182 | 0.425052661 | 0.586558656 | 0.494499404 | 0.148098361 | 0.374782403 |
| ENSMUSG00000020137 | Thap2 | 3405 | 2.584015107 | 2.729602786 | 3.849993165 | 3.492344368 | 3.51977944 | 2.837821747 | 3.543240272 | 3.129421519 | 2.769959504 | 2.644870579 | 2.651825889 | 3.425983401 | 2.738247391 | 2.209674642 | 2.504764191 | 2.597688784 | 3.635651659 | 2.649484142 |
| ENSMUSG00000020140 | Lgr5 | 8283 | 0.071842625 | 0.084458624 | 0.087601468 | 0.055005486 | 0.092974862 | 0.191438787 | 0.011424044 | 0.032276568 | 0.004972415 | 0.011384934 | 0.005767833 | 0.175203094 | 0.005955803 | 0.016821473 | 0.009948462 | 0.004622795 | 0.004627103 | 0.006641206 |
| ENSMUSG00000020142 | Slc1a4 | 6213 | 1.977144032 | 2.396726817 | 1.564957283 | 1.737963545 | 2.006465191 | 2.432574238 | 0.784356338 | 1.303201241 | 1.630755332 | 1.191479223 | 1.191874869 | 2.030313817 | 2.469374903 | 3.266709511 | 2.254712539 | 1.69482006 | 1.258420178 | 1.50515824 |
| ENSMUSG00000020143 | Dock2 | 11370 | 0.003738362 | 0.038088621 | 0.021272441 | 0.016028512 | 0.004233239 | 0.0174328 | 0.270477056 | 0.366136487 | 0.220965476 | 0.124408188 | 1.126094008 | 0.073635439 | 0.425200342 | 0.465666312 | 0.311638857 | 0.020206125 | 0.053933223 | 0.217714143 |
| ENSMUSG00000020149 | Rab1 | 4023 | 93.83257974 | 95.55823994 | 104.5387703 | 98.89113575 | 101.5879105 | 102.8622615 | 98.35345112 | 94.1091934 | 101.0159958 | 98.83715649 | 97.93688356 | 91.66643865 | 100.552255 | 82.39409687 | 95.95261674 | 101.6419081 | 104.9757558 | 96.41292854 |
| ENSMUSG00000020150 | Gamt | 1731 | 6.089707484 | 5.715730551 | 5.700866662 | 5.237809256 | 4.031848025 | 3.578331425 | 12.08099601 | 13.15001261 | 7.756674834 | 5.366082346 | 2.953160345 | 6.997110792 | 6.725784216 | 11.53723719 | 5.831531596 | 1.260869221 | 1.682725918 | 4.449032317 |
| ENSMUSG00000020151 | Ptprr | 6461 | 0 | 0.010311993 | 0 | 0.021155105 | 0 | 0 | 0.014645621 | 0 | 0.038247809 | 0 | 0 | 0 | 0.038176689 | 0 | 0 | 0 | 0.083047231 | 0 |
| ENSMUSG00000020152 | Actr2 | 5195 | 28.00677912 | 30.56193529 | 31.60341906 | 28.95033828 | 28.96253963 | 32.10673748 | 27.40401365 | 28.99534843 | 26.86042645 | 28.35395514 | 25.84170035 | 27.05367438 | 26.8073152 | 21.84079028 | 25.68851579 | 22.48052985 | 27.92397185 | 25.1273552 |
| ENSMUSG00000020153 | Ndufs7 | 1549 | 159.5384722 | 159.6610243 | 145.0269044 | 154.9487292 | 152.2261421 | 153.7126435 | 160.936414 | 149.7862134 | 143.0493575 | 160.3245669 | 167.0735636 | 138.3320392 | 178.5694385 | 151.025738 | 139.3245641 | 144.485862 | 135.2925703 | 156.539795 |
| ENSMUSG00000020154 | Ptprb | 13165 | 0 | 0 | 0.003674404 | 0 | 0 | 0 | 0 | 0 | 0.003128486 | 0 | 0 | 0 | 0.029977618 | 0 | 0 | 0 | 0 | 0 |
| ENSMUSG00000020155 | Kcnmb1 | 4187 | 0 | 0.278469381 | 0.150192475 | 0.054407743 | 0.160937887 | 0 | 0 | 0.018243326 | 0.186898447 | 0.123873355 | 0 | 0 | 0 | 0 | 0 | 0.03658047 | 0.045768207 | 0 |
| ENSMUSG00000020156 | Mum1 | 7104 | 2.530924755 | 3.549811504 | 3.261672444 | 3.58511033 | 2.201981305 | 2.936613293 | 3.902763211 | 3.483766436 | 3.530769721 | 3.597365029 | 3.470140565 | 3.496335846 | 3.270739448 | 4.164538804 | 3.207272017 | 2.614153236 | 1.947606022 | 3.910416511 |
| ENSMUSG00000020159 | Gabrp | 3366 | 0 | 0 | 0 | 0 | 0 | 0 | 0 | 0.476554041 | 0 | 0 | 0 | 0 | 1.04057223 | 0.068990029 | 0.465139371 | 0 | 0.148021943 | 0.016342575 |
| ENSMUSG00000020160 | Meis1 | 10398 | 0.085844268 | 0.105724708 | 0.172131239 | 0.017526849 | 0.157384632 | 0.104843252 | 0.222958383 | 0.22772927 | 0.126752116 | 0.149641586 | 0.243515368 | 0.198613148 | 0.260940128 | 0.276931469 | 0.075286551 | 0.150982391 | 0.217469852 | 0.153420282 |
| ENSMUSG00000020163 | Uqcr11 | 795 | 287.4312281 | 212.6577839 | 250.0211776 | 221.3292493 | 253.7369712 | 234.0509617 | 381.060482 | 288.1965057 | 289.8598185 | 346.3056054 | 311.1081258 | 291.2243628 | 450.9992135 | 405.728537 | 340.0294443 | 336.6201951 | 307.622768 | 369.7719073 |
| ENSMUSG00000020164 | 1700058G18R | 822 | 0 | 0 | 0 | 0 | 0 | 0 | 0 | 0.092925553 | 0 | 0 | 0 | 0.203706798 | 0 | 0 | 0 | 0 | 0 | 0 |
| ENSMUSG00000020166 | Cnot2 | 4834 | 37.01836803 | 35.58704711 | 39.06707954 | 36.06994553 | 37.09961679 | 37.46695102 | 36.99667488 | 32.86727224 | 33.39055757 | 36.0896984 | 31.75443558 | 34.75489127 | 23.45154032 | 26.61359579 | 29.209295 | 32.32601754 | 36.81195625 | 30.19014484 |
| ENSMUSG00000020167 | Tcf3 | 4745 | 16.2943973 | 19.80520012 | 20.45043243 | 20.19280848 | 17.71092492 | 20.70876095 | 17.46929683 | 17.61921367 | 16.49196627 | 22.75555542 | 16.3310817 | 19.9383663 | 20.73084153 | 21.74894883 | 23.45319335 | 24.19288535 | 22.37383402 | 24.11358103 |
| ENSMUSG00000020168 | Olfr299 | 992 | 0 | 0 | 0 | 0 | 0 | 0 | 0 | 0 | 0 | 0 | 0 | 0 | 0 | 0 | 0 | 0 | 0 | 0 |
| ENSMUSG00000020169 | Best3 | 2709 | 0.031380713 | 0.08607983 | 0 | 0 | 0.248743792 | 0.036583783 | 0 | 0.056393359 | 0 | 0.052215618 | 0.105813862 | 0.041207577 | 0 | 0.08572183 | 0.076045692 | 0.028269182 | 0.042443297 | 0 |
| ENSMUSG00000020170 | Frs2 | 5672 | 5.44803649 | 6.68372243 | 5.500868667 | 5.622836924 | 6.720818679 | 6.281455473 | 5.446963917 | 6.470891874 | 7.776932014 | 6.641997867 | 7.765957637 | 7.380420829 | 6.183884553 | 7.672445325 | 7.300346204 | 5.960967266 | 7.338215521 | 7.399849864 |
| ENSMUSG00000020171 | Yeats4 | 1983 | 25.22874039 | 27.02997821 | 23.15001528 | 22.97581661 | 24.51499894 | 24.18913066 | 22.97635391 | 24.05562814 | 28.26769942 | 25.08522034 | 24.74275459 | 26.20493286 | 27.4397906 | 29.48719416 | 30.95830473 | 28.57796216 | 29.43567836 | 34.12062596 |
| ENSMUSG00000020173 | Cobl | 9507 | 2.04321715 | 2.344201771 | 2.376190036 | 2.539955138 | 2.333945178 | 3.043946188 | 1.667166029 | 1.57879606 | 1.446965001 | 2.345880156 | 1.371890572 | 2.219240608 | 1.437355747 | 1.39718197 | 1.486497704 | 2.24338575 | 2.096315808 | 1.880504858 |
| ENSMUSG00000020175 | Rab36 | 6066 | 1.660686897 | 2.16923726 | 2.200971708 | 2.230733625 | 2.007480496 | 2.744760713 | 3.424046497 | 2.638083846 | 1.772120114 | 2.370749175 | 2.181612842 | 1.840279017 | 2.146987498 | 2.848203183 | 3.131209572 | 3.0930428 | 2.780014942 | 4.425394672 |
| ENSMUSG00000020176 | Grb10 | 9656 | 2.196570302 | 2.380478149 | 2.114087594 | 2.151598614 | 2.781442979 | 2.06298663 | 0.867268452 | 1.657271811 | 1.083406691 | 1.11333475 | 1.657478365 | 1.641637135 | 1.180164964 | 1.50067888 | 1.373954121 | 1.126194335 | 1.547975952 | 1.088104742 |
| ENSMUSG00000020177 | 9530003J23R | 1682 | 0 | 0 | 0.115038122 | 0 | 0.085847664 | 0 | 0 | 0.022706541 | 0 | 0 | 0 | 0 | 0 | 0 | 0 | 0 | 0 | 0 |
| ENSMUSG00000020178 | Adora2a | 2531 | 0.923660483 | 1.763701235 | 1.299644435 | 1.260084197 | 0.760678367 | 0.95933779 | 0.1495462 | 0.497964946 | 0.716004225 | 0.391214843 | 0.415270283 | 0.838006787 | 0.331348318 | 0.091750469 | 0.406969142 | 0.257187011 | 0.151427487 | 0.434682787 |
| ENSMUSG00000020180 | Snrpd3 | 600 | 202.6788477 | 167.6193794 | 203.24945 | 157.8690186 | 178.4089971 | 163.1936693 | 198.8709604 | 188.1612358 | 197.6952757 | 170.9212993 | 186.1630882 | 175.0751283 | 170.3595543 | 168.5146304 | 189.4584878 | 166.1174184 | 154.5188537 | 170.5282308 |

| ENSMUSG00000020181 | Nav3 | 9264 | 0.229410492 | 0.276888262 | 0.219310047 | 0.378691755 | 0.353299958 | 0.192562436 | 0.526036909 | 0.445248215 | 0.12003842 | 0.223945482 | 0.371307967 | 0.42777548 | 0.431334769 | 0.340910831 | 0.084502327 | 0.268662509 | 0.219267459 | 0.219703902 |
| --- | --- | --- | --- | --- | --- | --- | --- | --- | --- | --- | --- | --- | --- | --- | --- | --- | --- | --- | --- | --- |
| ENSMUSG00000020182 | Ddc | 4746 | 0 | 0.140383035 | 0.091732358 | 0.047999415 | 0 | 0.135732309 | 0.069782712 | 0.056330977 | 0 | 0 | 0 | 0 | 0 | 0.02935783 | 0 | 0 | 0 | 0.092725001 |
| ENSMUSG00000020183 | Cpm | 5981 | 6.3889238 | 7.647317131 | 5.135795314 | 6.269309394 | 6.888635121 | 7.009131016 | 3.116735579 | 3.8249873 | 3.270957196 | 4.036308321 | 2.116763751 | 3.424903556 | 6.095349752 | 8.130239006 | 7.501838215 | 8.130592108 | 7.112889076 | 7.550990908 |
| ENSMUSG00000020184 | Mdm2 | 5734 | 12.84643705 | 14.15246082 | 11.59142496 | 14.36595189 | 14.17768034 | 13.429534 | 11.60951155 | 13.24145378 | 14.22924446 | 13.15680593 | 12.53946852 | 11.73939468 | 9.842293925 | 12.21445479 | 14.55060179 | 9.702869298 | 14.5578435 | 12.39479698 |
| ENSMUSG00000020185 | E2f7 | 7642 | 0 | 0 | 0 | 0 | 0 | 0 | 0 | 0 | 0 | 0 | 0 | 0 | 0.006455367 | 0 | 0 | 0 | 0 | 0.00719826 |
| ENSMUSG00000020186 | Csrp2 | 1066 | 19.65764707 | 23.87528254 | 19.24050363 | 11.7962929 | 19.09925301 | 19.01225894 | 0.399450385 | 0.03582777 | 0.309092051 | 0.884628579 | 0.089634068 | 0.785398629 | 1.48088307 | 1.132782619 | 0.618409845 | 1.329036092 | 1.653855213 | 0.258016448 |
| ENSMUSG00000020189 | Osbpl8 | 7128 | 4.484272218 | 5.510087301 | 4.322943159 | 4.506248072 | 4.058261241 | 6.006391949 | 4.686132341 | 4.361478052 | 4.356710562 | 5.086825309 | 4.048270892 | 4.948863462 | 4.380906795 | 3.498944294 | 4.236916452 | 5.447064521 | 5.253197541 | 4.368007071 |
| ENSMUSG00000020190 | Mknk2 | 4448 | 28.55338713 | 33.19306366 | 30.98385521 | 30.72912163 | 28.09138351 | 34.43513927 | 38.7287464 | 36.67260582 | 37.77899827 | 44.26738687 | 36.56159143 | 31.84805568 | 34.92495672 | 32.72386913 | 37.96878993 | 35.46701945 | 35.22434841 | 37.97953389 |
| ENSMUSG00000020191 | 4930415F15R | 1843 | 0.599638946 | 0.451884078 | 0.813662203 | 1.087729762 | 0.443973251 | 0.40330494 | 0.23104401 | 0.331567248 | 0.446950795 | 0.793093761 | 0.570292505 | 1.211408846 | 1.097454481 | 0.428404496 | 1.274274924 | 0.768720821 | 1.601261455 | 1.641617401 |
| ENSMUSG00000020193 | Zpbp | 4240 | 0.451116255 | 0.644258803 | 0.638895684 | 0.95635211 | 0.794630815 | 1.121948685 | 0.926168007 | 0.52244324 | 1.253080314 | 1.145406235 | 1.250712356 | 1.211094566 | 1.26820259 | 1.380178069 | 2.089229839 | 1.71585269 | 1.527628344 | 2.465030726 |
| ENSMUSG00000020196 | Cabin1 | 8447 | 7.568105214 | 7.978215343 | 6.774699468 | 6.925580789 | 6.330598697 | 6.957445486 | 4.598521297 | 5.240321343 | 5.197682704 | 6.290853862 | 5.028049969 | 5.266376593 | 5.413837731 | 5.844686267 | 6.238521367 | 7.461387449 | 6.338562421 | 5.919649344 |
| ENSMUSG00000020198 | Ap3d1 | 4699 | 26.29549834 | 28.90331341 | 24.91252255 | 29.33010409 | 26.46794866 | 28.0085253 | 21.73825789 | 27.65068163 | 23.90202777 | 24.30283109 | 24.87876642 | 27.45052059 | 23.3904047 | 22.12988118 | 24.66481735 | 25.25273299 | 24.86845696 | 27.15920996 |
| ENSMUSG00000020205 | Phlda1 | 1951 | 118.0384642 | 124.816636 | 125.2356237 | 133.156881 | 125.744959 | 144.2388385 | 181.1510567 | 216.723776 | 213.3845727 | 220.9388668 | 201.5559627 | 225.8374374 | 206.2787192 | 191.4658098 | 243.513655 | 261.8912687 | 217.9746747 | 254.9704521 |
| ENSMUSG00000020211 | Sf3a2 | 2721 | 14.90258652 | 15.23015083 | 16.76451273 | 14.68468794 | 14.15124547 | 15.35205969 | 11.07613983 | 15.9731547 | 13.42610786 | 16.84325012 | 12.27294964 | 15.32315323 | 12.87234887 | 13.89396808 | 12.93132258 | 14.70550707 | 13.5782985 | 15.36454286 |
| ENSMUSG00000020212 | Mdm1 | 3134 | 7.893430902 | 6.951701602 | 7.239050969 | 6.018593604 | 6.465711507 | 7.668498974 | 7.065199023 | 6.54413532 | 4.113394842 | 6.409125586 | 4.161634864 | 6.482738092 | 5.36764001 | 4.001245563 | 4.759082078 | 6.609833632 | 4.879448776 | 3.159425402 |
| ENSMUSG00000020213 | Glipr1l1 | 891 | 0 | 0 | 0 | 0 | 0 | 0 | 0.05310065 | 0 | 0 | 0 | 0 | 0 | 0 | 0 | 0 | 0 | 0 | 0 |
| ENSMUSG00000020214 | Glipr1l2 | 1898 | 0 | 0 | 0 | 0 | 0 | 0 | 0 | 0 | 0 | 0 | 0 | 0 | 0 | 0 | 0 | 0 | 0 | 0 |
| ENSMUSG00000020216 | Jsrp1 | 1318 | 0 | 0 | 0 | 0 | 0.109556731 | 0 | 0 | 0 | 0 | 0 | 0 | 0 | 0.074858752 | 0 | 0 | 0.087156163 | 0 | 0.292157623 |
| ENSMUSG00000020218 | Wif1 | 2883 | 3.818536452 | 7.660925726 | 7.349152374 | 4.946447061 | 7.045324935 | 7.270484323 | 0.935422372 | 0.967063953 | 0.414293776 | 0.261675773 | 0.911419603 | 0.716330044 | 0.119779196 | 0.032219277 | 0.128620882 | 0 | 0 | 0 |
| ENSMUSG00000020219 | Timm13 | 1222 | 122.889351 | 102.3649082 | 97.38042936 | 93.43369649 | 105.1654961 | 95.86142568 | 131.2907482 | 104.2008753 | 116.9198226 | 119.2272284 | 117.5998978 | 107.429164 | 127.0841862 | 126.7520716 | 107.252168 | 108.855622 | 92.39711185 | 111.0085574 |
| ENSMUSG00000020220 | Vps13d | 22805 | 1.27860341 | 1.154009491 | 1.283314443 | 1.244662601 | 1.272683621 | 1.310252065 | 0.890030224 | 1.060109218 | 1.26602708 | 1.102009421 | 1.141738758 | 0.922714527 | 1.648363121 | 1.386907411 | 1.060526784 | 1.279431828 | 1.073909394 | 1.150596093 |
| ENSMUSG00000020224 | Llph | 1513 | 12.19249597 | 11.97766983 | 10.93440012 | 13.03895056 | 12.0568401 | 11.49571017 | 14.41582616 | 14.1107422 | 15.65250929 | 17.07771671 | 14.01988207 | 15.12519607 | 17.41126492 | 17.03666127 | 16.17562736 | 15.91856712 | 18.56786228 | 15.96100321 |
| ENSMUSG00000020225 | Tmbim4 | 2901 | 231.1631031 | 218.1931414 | 223.241925 | 221.7115694 | 218.57634 | 221.5268534 | 239.7925951 | 231.6558127 | 245.1882548 | 274.0299403 | 256.8089288 | 240.3671398 | 241.3879923 | 229.3226845 | 238.5741937 | 221.5338003 | 218.3053189 | 235.0354629 |
| ENSMUSG00000020226 | Slc5a4b | 3187 | 0 | 0 | 0 | 0 | 0 | 0 | 0.029691044 | 0 | 0 | 0 | 0 | 0 | 0.015479108 | 0 | 0 | 0 | 0.012025823 | 0 |
| ENSMUSG00000020227 | Irak3 | 5188 | 3.916244052 | 4.552590979 | 5.268127342 | 3.688442294 | 4.601664252 | 3.916079562 | 3.930563727 | 4.284498496 | 5.739755377 | 5.48031497 | 6.243527727 | 5.31475276 | 2.348686116 | 2.640903196 | 5.781559886 | 4.760493788 | 5.34854259 | 3.827734681 |
| ENSMUSG00000020228 | Helb | 5263 | 1.179128957 | 1.588739587 | 1.305157004 | 1.765999058 | 1.627870495 | 1.252235145 | 1.015833692 | 1.248165156 | 1.322538697 | 1.325917553 | 1.12561179 | 1.505951755 | 1.780935642 | 1.164852657 | 1.377821364 | 1.178620254 | 1.099614447 | 1.850011759 |
| ENSMUSG00000020229 | Slc5a4a | 2040 | 0 | 0 | 0 | 0 | 0 | 0 | 0 | 0 | 0 | 0 | 0 | 0 | 0 | 0 | 0 | 0 | 0 | 0 |
| ENSMUSG00000020230 | Prmt2 | 3639 | 26.88840759 | 28.61668242 | 31.92998624 | 30.6745146 | 25.77881814 | 30.03938727 | 25.11901503 | 26.63707538 | 22.16081282 | 25.83635677 | 21.99039721 | 24.32636462 | 26.82821223 | 22.39883852 | 24.41070473 | 28.75741413 | 23.67615154 | 25.45626153 |
| ENSMUSG00000020231 | Dip2a | 6808 | 3.708589095 | 3.293122475 | 2.678733981 | 2.275375305 | 3.308716257 | 3.391829296 | 2.390652402 | 2.412269835 | 3.478590858 | 3.137377875 | 2.428045775 | 3.476181101 | 1.347787399 | 1.084695931 | 2.196851467 | 1.951651112 | 1.812730259 | 1.680654259 |
| ENSMUSG00000020232 | Hmg20b | 2867 | 19.6736549 | 20.6825782 | 20.95567656 | 20.13458081 | 19.07145633 | 19.09862944 | 18.16925689 | 18.6499349 | 19.33625749 | 19.0773686 | 18.49675756 | 20.42226371 | 19.42648574 | 19.77964098 | 18.68225415 | 19.29889351 | 16.45611144 | 18.70731742 |
| ENSMUSG00000020234 | 4930404N11R | 1987 | 0.085566535 | 0.184419646 | 0.21910507 | 0.045859129 | 0.12111707 | 0.049876934 | 0.619088904 | 0.192211386 | 0.497471756 | 0.332214316 | 0.432787747 | 0.140452095 | 0.645510744 | 0.444105516 | 0.559860095 | 0.674469682 | 0.115731143 | 0.110738011 |
| ENSMUSG00000020235 | Fzr1 | 4634 | 18.87692108 | 22.32840946 | 20.46010347 | 22.02346554 | 19.47504464 | 21.0337132 | 18.05110409 | 22.6154407 | 19.59781339 | 21.79473595 | 21.85647647 | 18.87422168 | 14.78680041 | 18.51148671 | 16.98208281 | 17.6662319 | 18.66690809 | 16.06113971 |
| ENSMUSG00000020238 | Ncln | 4600 | 17.16839501 | 16.69265678 | 14.13348365 | 19.01678372 | 16.34392712 | 15.74915141 | 17.62911558 | 16.84617054 | 16.23285936 | 19.21903665 | 20.31474321 | 16.62336038 | 14.04887209 | 18.06271138 | 15.6207638 | 14.69193958 | 16.58028931 | 15.60584441 |
| ENSMUSG00000020241 | Col6a2 | 8297 | 0 | 0 | 0 | 0 | 0 | 0 | 0 | 0 | 0 | 0 | 0 | 0 | 0 | 0 | 0 | 0 | 0 | 0 |
| ENSMUSG00000020246 | Hcfc2 | 5470 | 4.942100906 | 5.408016466 | 4.819666187 | 5.613920271 | 6.062686546 | 5.725288413 | 6.253577123 | 5.480993764 | 4.585482285 | 4.103059369 | 4.943441773 | 5.65299398 | 4.184645268 | 4.253836889 | 3.939380938 | 5.558088168 | 7.370980684 | 4.927689633 |
| ENSMUSG00000020248 | Nfyb | 5682 | 5.79752049 | 5.974276524 | 7.074692665 | 8.259041828 | 6.505687676 | 5.799466355 | 6.536510552 | 7.380369208 | 6.0163337 | 5.609622563 | 5.498912856 | 7.701421941 | 5.964629914 | 5.566424413 | 6.990197092 | 8.423664071 | 7.446714499 | 7.503002061 |
| ENSMUSG00000020250 | Txnrd1 | 4002 | 69.99228137 | 90.83215935 | 68.83739515 | 85.31595858 | 86.59416552 | 78.43992184 | 59.85609531 | 70.92602066 | 68.20165916 | 63.32709396 | 65.1444149 | 58.64689185 | 52.77109892 | 50.95851767 | 59.12557108 | 53.22604922 | 62.83329185 | 52.49369779 |
| ENSMUSG00000020251 | Glt8d2 | 4383 | 0.077581886 | 0 | 0.022073251 | 0.166319121 | 0 | 0 | 0 | 0 | 0 | 0.021515265 | 0.010900059 | 0.01273458 | 0.168829286 | 0.04238566 | 0 | 0 | 0.008744307 | 0.075303363 |
| ENSMUSG00000020253 | Ppm1m | 3996 | 5.307828538 | 4.068241336 | 5.132726945 | 5.210559875 | 3.746003068 | 4.538613736 | 4.653123829 | 4.759713813 | 4.442289363 | 4.873183295 | 3.80191111 | 3.422131465 | 7.765209201 | 4.730416308 | 3.608744885 | 5.461873428 | 4.143383449 | 3.854491963 |
| ENSMUSG00000020255 | D10Wsu102e | 3505 | 10.34434098 | 13.04002593 | 11.96572063 | 12.80388835 | 13.75982525 | 13.69945759 | 12.35124143 | 15.32054716 | 13.14913299 | 14.3940806 | 12.54007469 | 12.1026829 | 11.11903414 | 11.64746157 | 9.545125074 | 12.93468735 | 14.33545656 | 15.17654956 |
| ENSMUSG00000020256 | Aldh1l2 | 8549 | 2.043470271 | 2.8134179 | 1.459863239 | 2.526135807 | 2.223898684 | 1.680932593 | 1.881659941 | 1.469797683 | 1.112888658 | 1.809033883 | 1.503271006 | 2.226358749 | 1.350294613 | 1.081105789 | 1.720546901 | 1.961783371 | 1.358389047 | 1.299782379 |

| ENSMUSG00000020257 | Wdr82 | 5380 | 8.12970746 | 7.009330159 | 6.464789652 | 6.461538444 | 6.235678587 | 5.609222072 | 5.285301122 | 6.985376202 | 7.142568626 | 4.688778112 | 5.594465398 | 7.220762297 | 7.11553305 | 5.602641769 | 4.786426103 | 6.562070636 | 6.254737673 | 6.76877856 |
| --- | --- | --- | --- | --- | --- | --- | --- | --- | --- | --- | --- | --- | --- | --- | --- | --- | --- | --- | --- | --- |
| ENSMUSG00000020258 | Glyctk | 7339 | 0.10425033 | 0.063548238 | 0.092278161 | 0 | 0.039350258 | 0.101279603 | 0.064467474 | 0.119692772 | 0.084180098 | 0.109219506 | 0.136704473 | 0.205344446 | 0.087384511 | 0.164538258 | 0 | 0.114783126 | 0.172335168 | 0.217368047 |
| ENSMUSG00000020260 | Pofut2 | 2210 | 38.02386109 | 45.62811348 | 44.52115871 | 50.01406948 | 48.85063565 | 45.20285561 | 41.6608476 | 43.9990301 | 34.53330937 | 46.7026649 | 54.02245305 | 44.70304198 | 41.25130458 | 46.59119536 | 40.62361551 | 50.15896309 | 53.86492225 | 47.51691617 |
| ENSMUSG00000020261 | Slc36a1 | 3247 | 3.691549011 | 2.072437521 | 3.858560013 | 3.213267371 | 2.594113533 | 3.097999666 | 2.885097144 | 2.634769984 | 2.37199829 | 1.597344428 | 1.765628278 | 1.272053906 | 4.618695058 | 4.663003538 | 2.093703949 | 4.540155174 | 2.136452029 | 3.591601671 |
| ENSMUSG00000020262 | Adarb1 | 15466 | 0.272081497 | 0.189547051 | 0.272112837 | 0.315209605 | 0.295650637 | 0.240298398 | 0.351801246 | 0.323497007 | 0.226358065 | 0.298769489 | 0.259478631 | 0.144357074 | 0.322159812 | 0.315312891 | 0.359641151 | 0.240151876 | 0.359324522 | 0.551300371 |
| ENSMUSG00000020263 | Appl2 | 5299 | 5.382331216 | 6.644976258 | 7.166110838 | 7.738241159 | 6.049417091 | 7.621339473 | 8.776818907 | 8.432744068 | 7.492696961 | 9.654371209 | 8.34867174 | 7.362737903 | 6.330572513 | 7.143221593 | 7.728693443 | 5.470086772 | 6.053804587 | 9.841221584 |
| ENSMUSG00000020264 | Slc36a2 | 3245 | 0 | 0 | 0 | 0 | 0 | 0 | 0 | 0 | 0.025384601 | 0 | 0 | 0 | 0 | 0 | 0 | 0 | 0 | 0 |
| ENSMUSG00000020265 | Sumo3 | 2500 | 100.7882734 | 89.62501061 | 90.41980294 | 105.9567647 | 97.22648581 | 95.85480783 | 97.48304366 | 92.10479764 | 83.70747464 | 94.20710511 | 92.79807935 | 84.88445966 | 90.19847755 | 88.02083442 | 89.9347562 | 85.75564434 | 84.33318371 | 82.02957995 |
| ENSMUSG00000020267 | Hint1 | 989 | 242.7823452 | 210.0160723 | 207.4339153 | 185.9296005 | 225.6701012 | 213.1919933 | 289.1383533 | 248.3857758 | 243.2461462 | 234.895364 | 244.2367948 | 241.3787552 | 320.632522 | 356.9479355 | 302.5335681 | 290.6833977 | 269.0978824 | 313.8133267 |
| ENSMUSG00000020268 | Lyrm7 | 4112 | 1.891645723 | 1.409640952 | 1.411678901 | 1.617682994 | 1.978184606 | 1.494294495 | 2.25517633 | 1.950487477 | 1.973186674 | 2.820786236 | 2.985934905 | 2.131094121 | 2.41140938 | 1.920110617 | 1.643252715 | 2.402474881 | 1.929363682 | 2.943094231 |
| ENSMUSG00000020270 | 1700008A04R | 685 | 0 | 0 | 0 | 0 | 0 | 0 | 0 | 0 | 0 | 0 | 0 | 0 | 0 | 0 | 0 | 0 | 0 | 0 |
| ENSMUSG00000020271 | Fbxw11 | 4506 | 26.33698435 | 28.93623346 | 34.40682752 | 39.14043556 | 32.36567437 | 39.53441572 | 23.86633804 | 28.66549155 | 18.1801997 | 25.42747646 | 28.74343374 | 26.97880895 | 18.15186836 | 24.78873284 | 24.73373473 | 24.17593832 | 27.28600987 | 19.38625421 |
| ENSMUSG00000020272 | Stk10 | 5168 | 0.082246858 | 0.103135895 | 0.046801016 | 0.070527932 | 0.065194169 | 0.10547215 | 0.485211297 | 0.450800415 | 0.326750609 | 0.072988705 | 0.942926812 | 0.043200977 | 0.496373781 | 0.287579488 | 0.215255807 | 0.400095356 | 0.096409029 | 0 |
| ENSMUSG00000020273 | Papolg | 5029 | 3.76114602 | 2.503931997 | 3.414715306 | 2.871913115 | 2.038596091 | 2.985579295 | 4.525233359 | 3.242822793 | 3.783688662 | 2.362691832 | 2.916466942 | 3.385121712 | 2.638752386 | 3.03839824 | 4.104589282 | 2.878077073 | 3.917223426 | 2.778348202 |
| ENSMUSG00000020275 | Rel | 2789 | 0.167643218 | 0.752496356 | 0.329543592 | 0.653439144 | 1.794808197 | 0.941661843 | 0.712489249 | 1.095515307 | 0.265814731 | 0.896015515 | 1.987054566 | 1.560997376 | 0.442200764 | 0.732714179 | 1.093192949 | 1.098332231 | 1.030646206 | 0.907285374 |
| ENSMUSG00000020277 | Pfkl | 4372 | 69.39660258 | 71.39565847 | 79.43128418 | 75.96981089 | 78.05474355 | 80.99355767 | 111.8644012 | 117.2852687 | 113.0650874 | 105.4635698 | 117.0333498 | 123.5425816 | 79.16576661 | 65.99054686 | 91.14854725 | 64.23223117 | 76.09155012 | 84.42614504 |
| ENSMUSG00000020279 | Il9r | 6988 | 0 | 0 | 0 | 0 | 0 | 0 | 0 | 0 | 0.005893892 | 0 | 0.102550712 | 0 | 0.007059519 | 0 | 0.123816925 | 0.115069083 | 0 | 0 |
| ENSMUSG00000020280 | Pus10 | 4286 | 4.879268923 | 5.114298739 | 5.665775137 | 6.463162612 | 5.367956023 | 6.879112571 | 5.806455697 | 5.435689563 | 6.323081515 | 5.577556358 | 5.918922761 | 6.550461568 | 4.385315092 | 6.902679344 | 3.643348036 | 3.921973087 | 7.547222255 | 7.533911723 |
| ENSMUSG00000020282 | Rhbdf1 | 5288 | 11.23718534 | 14.6279388 | 12.7428759 | 14.39722108 | 14.39952784 | 14.04681309 | 10.45030427 | 16.4094437 | 11.68301317 | 10.35211166 | 8.429280685 | 15.28386525 | 5.476141347 | 8.247163022 | 10.5497176 | 10.369166 | 10.09616709 | 9.268743209 |
| ENSMUSG00000020283 | Pex13 | 4772 | 17.841129 | 15.83968084 | 20.75033878 | 18.57958765 | 17.71157962 | 19.28319913 | 15.36769747 | 17.47150345 | 15.82901716 | 19.8206854 | 16.29874946 | 15.31070879 | 15.99255575 | 14.12203915 | 14.49652396 | 17.45223619 | 18.49653432 | 16.41512321 |
| ENSMUSG00000020284 | 1810043G02R | 2833 | 7.246734918 | 6.984772032 | 5.720131546 | 7.027947891 | 5.572633591 | 7.293854537 | 7.949465291 | 7.980904421 | 9.973155599 | 8.954139906 | 6.425082662 | 9.102307136 | 11.30124049 | 8.721586478 | 10.82031683 | 9.690916169 | 8.157697505 | 8.524178557 |
| ENSMUSG00000020286 | 1700093K21R | 2000 | 0 | 0.133251577 | 0.096747061 | 0 | 0 | 0.024776367 | 0 | 0 | 0 | 0.094301407 | 0 | 0 | 0.024665959 | 0.116110218 | 0 | 0 | 0.057489445 | 0 |
| ENSMUSG00000020287 | Mpg | 2762 | 4.986124921 | 5.367211415 | 6.515189461 | 5.130153797 | 5.349927794 | 5.830788709 | 7.057503157 | 7.881849881 | 7.649776461 | 8.194184491 | 6.538354202 | 5.132939282 | 7.465873071 | 7.550106893 | 10.84487007 | 7.985296837 | 7.590327446 | 9.739121357 |
| ENSMUSG00000020288 | Ahsa2 | 5622 | 10.16888327 | 7.649759238 | 9.318486904 | 7.917669925 | 8.432932197 | 7.024818332 | 6.210735868 | 6.773003415 | 5.999956672 | 6.56688023 | 6.594337919 | 6.701453619 | 6.905766435 | 6.807173249 | 6.742339278 | 6.606526001 | 7.008081326 | 6.751384496 |
| ENSMUSG00000020289 | Nprl3 | 7124 | 5.328063194 | 6.930054637 | 7.333438066 | 6.529734176 | 6.013112305 | 6.204525316 | 6.036949066 | 5.586255377 | 4.023837026 | 6.228075767 | 7.108570464 | 5.891827381 | 7.776493978 | 5.710979864 | 4.696190817 | 4.939509854 | 5.821034987 | 6.501637824 |
| ENSMUSG00000020290 | Xpo1 | 7275 | 10.0960748 | 10.85244801 | 12.28122482 | 10.84697303 | 10.82389376 | 9.48143026 | 8.714637768 | 11.06135971 | 11.83227738 | 10.97914657 | 11.52509308 | 12.11449226 | 7.852420647 | 10.57839901 | 12.13676072 | 10.12135231 | 12.90713779 | 8.922439304 |
| ENSMUSG00000020295 | Hbq1a | 601 | 0 | 0 | 0 | 0 | 0 | 0 | 0 | 0 | 0 | 0 | 0 | 0 | 0 | 0 | 0 | 0 | 0 | 0 |
| ENSMUSG00000020297 | Nsg2 | 4245 | 0 | 0 | 0 | 0 | 0 | 0 | 0.055727537 | 0 | 0 | 0 | 0 | 0 | 0 | 0.109408922 | 0.029117707 | 0.108242 | 0.009028574 | 0.012958565 |
| ENSMUSG00000020299 | 4930524B15R | 1995 | 0 | 0 | 0 | 0 | 0 | 0 | 0 | 0 | 0 | 0 | 0 | 0 | 0 | 0 | 0 | 0 | 0 | 0 |
| ENSMUSG00000020300 | Cpeb4 | 7568 | 5.021092411 | 5.533074529 | 5.16461582 | 5.309836298 | 5.762093399 | 4.118481689 | 4.651246449 | 3.512409101 | 3.521098797 | 3.831617667 | 3.888659411 | 3.245096662 | 4.862792057 | 4.614951601 | 5.068531783 | 5.707162414 | 7.170987898 | 5.24796182 |
| ENSMUSG00000020303 | Stc2 | 7070 | 4.220457365 | 3.444374211 | 2.018414633 | 1.024640177 | 2.314689398 | 2.277883792 | 2.355595896 | 1.48015817 | 1.433081029 | 0.793625698 | 1.108216859 | 1.649996249 | 4.354047579 | 2.50285676 | 3.187729996 | 2.951680486 | 2.878537999 | 3.960344459 |
| ENSMUSG00000020305 | Asb3 | 5427 | 6.015105065 | 5.776199274 | 6.373148001 | 6.539902992 | 7.17500023 | 6.592421899 | 5.283118377 | 5.552571494 | 4.819133501 | 5.516988496 | 6.135829383 | 5.090980833 | 8.13563036 | 6.940511309 | 7.865268455 | 10.44930709 | 8.905373207 | 8.281267771 |
| ENSMUSG00000020307 | Cdc34 | 1467 | 19.1519573 | 17.8940427 | 17.2456417 | 18.44803022 | 17.97975063 | 19.52384459 | 23.28544934 | 24.44626166 | 21.44955558 | 22.82004043 | 22.6011051 | 23.39920415 | 27.642015 | 24.50424243 | 22.07527125 | 23.46506888 | 20.24736203 | 25.38593405 |
| ENSMUSG00000020308 | Gm16517 | 1099 | 42.81458586 | 33.61601428 | 30.41502227 | 33.95313495 | 30.30690735 | 38.46085691 | 36.80831709 | 38.3661622 | 32.60443013 | 33.80778359 | 42.7322877 | 38.29391227 | 38.24458009 | 41.62641135 | 31.79155541 | 36.68790682 | 33.06035438 | 42.39555359 |
| ENSMUSG00000020309 | Chac2 | 2106 | 11.52443282 | 8.636676279 | 8.797275463 | 8.37232866 | 10.12461642 | 10.517603 | 10.60379129 | 10.79035111 | 7.372894797 | 9.470440398 | 13.67915478 | 11.60838567 | 12.32126709 | 11.15893078 | 12.65783792 | 9.14538249 | 12.33866538 | 8.985343168 |
| ENSMUSG00000020310 | Madcam1 | 1415 | 0.150194968 | 0.235426814 | 0.239303684 | 0.386383415 | 0.306139444 | 0.210117598 | 0 | 0.026991097 | 0.17464247 | 0 | 0.101289665 | 0.19722849 | 0.209181275 | 0 | 0 | 0 | 0.162514333 | 0 |
| ENSMUSG00000020311 | Erlec1 | 5095 | 15.62555342 | 13.31861934 | 18.89368507 | 9.183747308 | 13.43348292 | 13.8105754 | 18.47933878 | 15.51683474 | 17.35573098 | 11.54012305 | 14.67474191 | 13.49654491 | 26.52014159 | 18.94225978 | 20.65334127 | 18.54031962 | 17.4217279 | 17.53381537 |
| ENSMUSG00000020312 | Shc2 | 7573 | 0.550047174 | 0.585054131 | 0.338544449 | 0.523413556 | 0.552948285 | 0.700137659 | 0.106208311 | 0.312680437 | 0.348070383 | 0.261498684 | 0.044160136 | 0.53803557 | 0.397365239 | 0.251446928 | 0.223064016 | 0.404496051 | 0.57188321 | 0.559316152 |
| ENSMUSG00000020315 | Spnb2 | 18773 | 16.53293535 | 18.19051183 | 17.18184096 | 18.73601781 | 17.50111921 | 18.82280633 | 14.04032037 | 15.86857395 | 13.60232236 | 16.19494671 | 15.60772494 | 16.17414396 | 16.87843737 | 15.90523826 | 17.19346875 | 21.70610153 | 21.70795904 | 16.49423437 |
| ENSMUSG00000020317 | Theg | 1845 | 0 | 0 | 0 | 0 | 0 | 0.107431401 | 0 | 0 | 0 | 0 | 0 | 0 | 0 | 0 | 0 | 0 | 0 | 0 |
| ENSMUSG00000020319 | Wdpcp | 3716 | 3.969132422 | 4.312029634 | 4.6212603 | 4.646834177 | 4.105979495 | 4.813922718 | 3.068448768 | 2.877791355 | 3.890330203 | 4.440997347 | 5.168335121 | 4.310843693 | 3.584396573 | 4.161970156 | 4.213291507 | 2.854278324 | 4.331820425 | 2.694202751 |

| ENSMUSG00000020321 | Mdh1 | 2558 | 333.7104965 | 327.7504335 | 335.6079137 | 341.298175 | 347.5734535 | 347.9345769 | 392.521373 | 360.3045562 | 384.7831719 | 417.8127407 | 389.6516649 | 353.0482489 | 412.9957031 | 364.308293 | 383.0713854 | 403.5183361 | 394.21548 | 390.0528451 |
| --- | --- | --- | --- | --- | --- | --- | --- | --- | --- | --- | --- | --- | --- | --- | --- | --- | --- | --- | --- | --- |
| ENSMUSG00000020323 | Prss57 | 4795 | 0.044322394 | 0 | 0 | 0 | 0 | 0 | 0 | 0 | 0 | 0 | 0 | 0 | 0 | 0 | 0 | 0.015971056 | 0.015985942 | 0 |
| ENSMUSG00000020325 | Fstl3 | 2560 | 3.337320462 | 4.203150325 | 3.533535225 | 3.363686476 | 4.399558648 | 4.355220725 | 2.088411218 | 1.775349956 | 1.576671307 | 2.154934485 | 1.567615825 | 1.068346667 | 1.849946898 | 1.578373282 | 1.464586557 | 1.061966065 | 0.883301374 | 1.11737248 |
| ENSMUSG00000020326 | Ccng1 | 3992 | 37.13879108 | 39.52150977 | 39.95178597 | 41.87461715 | 41.35583622 | 40.34228059 | 53.3571344 | 41.32088827 | 42.22855937 | 47.81213597 | 36.65698843 | 37.97478447 | 45.38981266 | 44.32664653 | 45.89762501 | 36.18040359 | 51.02812326 | 40.58161806 |
| ENSMUSG00000020327 | Fgf22 | 769 | 0 | 0.043319758 | 0 | 0 | 0 | 0 | 0 | 0 | 0 | 0 | 0 | 0 | 0 | 0 | 0 | 0 | 0 | 0 |
| ENSMUSG00000020328 | Nudcd2 | 2460 | 20.99341011 | 21.01691538 | 20.98152719 | 18.81708173 | 19.17450618 | 18.12904887 | 20.8483512 | 20.29165445 | 21.17924489 | 22.77033962 | 21.11031702 | 20.44305365 | 25.64858626 | 23.56187848 | 22.17514634 | 20.84192004 | 24.02404464 | 17.48663474 |
| ENSMUSG00000020329 | Polrmt | 4482 | 6.638470152 | 6.503521294 | 5.936064635 | 7.197059212 | 6.250062377 | 6.876796137 | 5.721435069 | 6.910762684 | 5.559536376 | 5.386247226 | 5.489536728 | 5.927767825 | 5.316224017 | 3.720086429 | 4.623914015 | 3.981129641 | 4.754444685 | 4.123842003 |
| ENSMUSG00000020330 | Hmmr | 6893 | 0.04933141 | 0.144985758 | 0.056142208 | 0.085926821 | 0.132671776 | 0.201287762 | 0.549109867 | 0.132978044 | 1.027720979 | 0.136807495 | 0.027723754 | 0.016194883 | 0.286272551 | 0.768116344 | 0.68738995 | 0.788809843 | 2.029464438 | 1.117260256 |
| ENSMUSG00000020331 | Hcn2 | 3187 | 0.213392788 | 0.146338412 | 0.182140685 | 0.200142648 | 0.332256768 | 0.559742205 | 0.578975362 | 0.683077169 | 1.240635555 | 1.065218273 | 1.019359014 | 0.73556882 | 0.417935917 | 0.408043441 | 0.284416137 | 0.516628842 | 0.252542276 | 0.414251196 |
| ENSMUSG00000020332 | 4930404A10R | 1705 | 0 | 0 | 0 | 0 | 0 | 0 | 0 | 0 | 0 | 0 | 0 | 0 | 0 | 0 | 0 | 0 | 0 | 0 |
| ENSMUSG00000020333 | Acsl6 | 5812 | 0.511934329 | 0.567442623 | 0.474413495 | 0.337082744 | 0.463762513 | 0.895223163 | 0.04070258 | 0.091998216 | 0.255112623 | 0.032450587 | 0.312362082 | 0.038414083 | 0.076391475 | 0.191785633 | 0 | 0.230586934 | 0.026377355 | 0 |
| ENSMUSG00000020334 | Slc22a4 | 2542 | 1.203923161 | 1.297394385 | 1.141782778 | 1.093321678 | 0.530170678 | 1.03316085 | 1.488990682 | 0.751227427 | 0.194428871 | 0.853238533 | 0.845740807 | 1.075911671 | 0.116440403 | 0.584661997 | 0.794207804 | 0.286200449 | 0.301544429 | 0.389521605 |
| ENSMUSG00000020335 | Zfp354b | 4431 | 0.134297555 | 0.022554431 | 0.196507232 | 0.257058476 | 0.152075588 | 0.156564719 | 0.117454179 | 0.362013293 | 0.148721339 | 0.106410976 | 0.226421604 | 0.088176402 | 0.233800556 | 0.136261146 | 0.04649239 | 0.008641527 | 0.034598327 | 0.024829206 |
| ENSMUSG00000020340 | Cyfip2 | 10944 | 24.67428576 | 22.12032185 | 24.72159679 | 26.3066739 | 23.53380149 | 24.76278325 | 20.52204741 | 19.04385416 | 16.95782529 | 22.79984664 | 20.3645999 | 16.56517471 | 13.12632887 | 9.781946403 | 10.78980804 | 10.47183735 | 13.13614215 | 10.31420751 |
| ENSMUSG00000020346 | Mgat1 | 4814 | 8.493971612 | 11.9370051 | 11.24428344 | 10.8366007 | 10.55822837 | 12.85653599 | 12.22620951 | 11.65447427 | 12.54246616 | 13.74167775 | 12.14718512 | 8.881345563 | 8.720702452 | 9.724894077 | 11.89658552 | 11.08789088 | 10.40558165 | 10.22707738 |
| ENSMUSG00000020349 | Ppp2ca | 7483 | 72.40023702 | 72.29294257 | 77.8126667 | 76.0893186 | 74.18864193 | 73.29937298 | 70.67501337 | 74.5116775 | 70.4623513 | 71.90119937 | 76.19221629 | 68.03349705 | 64.23763223 | 65.63493574 | 71.34702141 | 70.85016037 | 73.57952443 | 71.33614481 |
| ENSMUSG00000020354 | Sgcd | 8248 | 2.705530724 | 2.225437041 | 2.727169206 | 2.695658297 | 2.503466932 | 2.625429749 | 2.414965789 | 2.097618609 | 1.817639638 | 2.023684403 | 2.739761804 | 2.246702228 | 2.410373747 | 2.455094823 | 2.68749237 | 2.692598484 | 2.973912466 | 3.187967145 |
| ENSMUSG00000020357 | Flt4 | 8203 | 0.005181662 | 0.004061062 | 0.005897054 | 0.077758701 | 0 | 0.036244837 | 0 | 0 | 0 | 0 | 0.011648167 | 0.006804299 | 0.018041662 | 0 | 0 | 0 | 0 | 0.067059742 |
| ENSMUSG00000020358 | Hnrnpab | 2724 | 128.6390129 | 144.3803338 | 140.2370665 | 146.7018795 | 135.9143748 | 139.4170889 | 118.8548685 | 121.8539535 | 110.1028663 | 114.5360512 | 123.1380262 | 118.6390185 | 113.5322281 | 117.4742152 | 116.6016132 | 117.9783656 | 127.1633889 | 123.2050514 |
| ENSMUSG00000020359 | Agxt2l2 | 8274 | 4.150855962 | 4.416756703 | 5.320269836 | 4.394212398 | 4.374571743 | 5.054690251 | 5.260776482 | 4.394388094 | 5.226715198 | 4.701393545 | 4.775186205 | 3.980087131 | 5.515110404 | 5.523444766 | 4.835232145 | 5.812545673 | 4.914696763 | 5.026212797 |
| ENSMUSG00000020361 | Hspa4 | 5509 | 35.03648654 | 37.53369657 | 34.15738484 | 37.66291449 | 37.97957382 | 35.5387276 | 27.58546464 | 30.27522623 | 25.41168399 | 31.98442151 | 25.53085456 | 27.45696962 | 29.4343823 | 35.49275874 | 31.74067947 | 31.18714021 | 32.35716226 | 31.44376059 |
| ENSMUSG00000020362 | Cnot6 | 7306 | 14.60860895 | 13.73370345 | 13.81817107 | 12.68425461 | 11.52899896 | 13.58528954 | 11.92206979 | 15.34282796 | 13.02792745 | 15.29525961 | 12.3066619 | 13.02569186 | 15.86102843 | 13.72471731 | 14.30719235 | 16.23131825 | 17.21693217 | 15.9922451 |
| ENSMUSG00000020363 | Gfpt2 | 5629 | 0 | 0 | 0 | 0.097127826 | 0 | 0 | 0.100861991 | 0 | 0 | 0.016752781 | 0 | 0.267724798 | 0 | 0 | 0 | 0 | 0.027234889 | 0 |
| ENSMUSG00000020364 | Zfp354a | 4425 | 0.864512055 | 1.234647379 | 1.104118998 | 1.019331839 | 1.011586192 | 1.075042356 | 1.486206186 | 1.113405629 | 1.424076138 | 1.289318665 | 0.788151857 | 0.592844326 | 0.546274338 | 0.90264215 | 0.810064489 | 0.441315475 | 2.026746548 | 1.131260726 |
| ENSMUSG00000020366 | Mapk9 | 6990 | 7.449047301 | 7.858792928 | 7.820041388 | 7.032956629 | 7.539979459 | 7.755463596 | 10.45752345 | 9.671037519 | 10.32314386 | 8.944468172 | 6.786914702 | 9.366491018 | 8.17258372 | 7.700815062 | 7.751079542 | 7.86628215 | 8.76734603 | 9.907934959 |
| ENSMUSG00000020368 | Canx | 4308 | 109.6767727 | 118.1880862 | 117.5876532 | 121.1259612 | 122.5866914 | 131.0018762 | 129.3081434 | 136.7053958 | 108.8172986 | 119.2558177 | 118.5169409 | 117.8114717 | 100.6563493 | 110.3208789 | 112.7495688 | 97.78859393 | 126.3041869 | 109.8905229 |
| ENSMUSG00000020372 | Gnb2l1 | 6622 | 25.99606809 | 22.87934807 | 22.51392639 | 21.97553241 | 23.36091855 | 21.0572927 | 21.90587037 | 22.68935534 | 24.23175255 | 20.27152706 | 22.24255466 | 21.16477329 | 29.84350054 | 30.49515131 | 23.70551615 | 24.7079078 | 19.93291551 | 24.48910399 |
| ENSMUSG00000020374 | Rasgef1c | 3659 | 0 | 0.009104371 | 0 | 0 | 0 | 0 | 0 | 0 | 0.011256222 | 0 | 0 | 0 | 0 | 0.482338158 | 0.090082658 | 0 | 0 | 0.496119301 |
| ENSMUSG00000020375 | Rufy1 | 3119 | 19.3651347 | 19.42807136 | 20.93756524 | 20.12924626 | 18.42562259 | 21.16198818 | 26.09099319 | 27.84531038 | 23.98035031 | 25.07951802 | 23.42029866 | 24.48086772 | 27.26778627 | 27.41377521 | 25.5214511 | 20.67373609 | 21.67604608 | 22.71616847 |
| ENSMUSG00000020376 | Rnf130 | 1848 | 93.22157924 | 89.39320478 | 83.0045805 | 78.42515261 | 86.52286278 | 79.26292233 | 71.07142679 | 64.97668461 | 74.37197137 | 66.89888741 | 63.31216088 | 65.84315175 | 63.74708791 | 66.24816794 | 72.03583717 | 80.12433919 | 73.39651772 | 78.37607035 |
| ENSMUSG00000020377 | Ltc4s | 860 | 1.828711062 | 1.820588405 | 1.406207278 | 1.80125059 | 1.287249121 | 1.843822645 | 1.485398059 | 1.421112648 | 1.771978004 | 0.986875184 | 1.666568319 | 1.492744465 | 1.548792752 | 1.782156842 | 1.772625079 | 1.870006408 | 1.515225692 | 1.85495825 |
| ENSMUSG00000020380 | Rad50 | 5388 | 9.379854175 | 9.131986778 | 10.10026385 | 8.777350412 | 9.978351659 | 9.481972591 | 8.23669132 | 8.654959788 | 8.844246239 | 8.497277814 | 8.388105182 | 8.121655432 | 6.802823782 | 7.740681232 | 7.524560319 | 9.800064515 | 8.123353946 | 7.779684358 |
| ENSMUSG00000020381 | 3010026O09R | 2029 | 2.178678318 | 2.938890126 | 3.123180126 | 1.95357854 | 3.582020933 | 2.320113204 | 2.938096375 | 2.691726734 | 2.638860054 | 3.648428 | 4.70921227 | 3.356092083 | 3.525445049 | 3.502190917 | 3.837897518 | 3.830947907 | 2.512270817 | 3.985381315 |
| ENSMUSG00000020383 | Il13 | 1201 | 0 | 0 | 0 | 0 | 0 | 0 | 0 | 0 | 0 | 0 | 0 | 0 | 0 | 0 | 0 | 0 | 0 | 0 |
| ENSMUSG00000020385 | Clk4 | 4285 | 20.10688257 | 19.36579218 | 18.94300675 | 20.09576985 | 22.94831273 | 18.94221181 | 20.84628654 | 22.71935443 | 24.09675496 | 22.12836972 | 23.07915147 | 23.19899535 | 20.07814789 | 28.77690829 | 26.18245897 | 28.34487904 | 29.59666662 | 32.64601129 |
| ENSMUSG00000020386 | Sar1b | 1344 | 154.8086582 | 146.4379308 | 150.8433525 | 146.3106156 | 160.5829953 | 142.2797611 | 169.8539255 | 144.8696931 | 169.7411539 | 166.0097677 | 155.1975214 | 149.8384752 | 170.3859821 | 148.6971042 | 162.5070296 | 159.6866477 | 169.5881605 | 155.1224066 |
| ENSMUSG00000020387 | Phf15 | 15011 | 1.138303961 | 0.829992834 | 0.741183931 | 0.925729034 | 0.846501089 | 0.739445228 | 0.873067222 | 0.987186206 | 1.116708541 | 1.033414254 | 0.636532656 | 1.267946236 | 1.160093718 | 0.912731049 | 0.886556695 | 1.124918917 | 0.934476362 | 0.985773746 |
| ENSMUSG00000020388 | Pdlim4 | 2842 | 141.5592861 | 127.132882 | 133.0867818 | 126.7757701 | 126.9856311 | 126.0787532 | 92.04496896 | 86.85344713 | 94.28552834 | 89.04216199 | 94.91112441 | 95.33048072 | 55.35520204 | 43.48617753 | 55.04655444 | 54.66044836 | 51.0837483 | 57.93182844 |
| ENSMUSG00000020389 | Cdkl3 | 3761 | 3.503484331 | 3.622699744 | 3.189746219 | 3.682679468 | 2.52113506 | 3.583712718 | 4.088439418 | 3.747140784 | 4.314673547 | 4.626059426 | 4.738117392 | 4.734165479 | 2.282306197 | 3.914590721 | 5.269329536 | 3.359718278 | 3.974276999 | 4.066081273 |
| ENSMUSG00000020390 | Ube2b | 2262 | 115.4517248 | 108.5540863 | 113.3848178 | 113.1170756 | 112.6482776 | 113.2790386 | 113.7010268 | 116.2825267 | 126.2907486 | 122.0456974 | 123.5559271 | 125.2274481 | 111.5311339 | 126.7870202 | 117.1202495 | 135.0666474 | 130.2958546 | 135.5529447 |
| ENSMUSG00000020392 | Cdkn2aipnl | 2019 | 41.07360001 | 39.05478981 | 38.43048177 | 39.96464067 | 36.87968594 | 40.02996952 | 41.78232121 | 42.07028378 | 45.93959064 | 43.55426527 | 40.10824895 | 41.57838362 | 50.74907983 | 38.0248026 | 39.99754807 | 38.51818902 | 35.68768607 | 37.46286367 |

| ENSMUSG00000020393 | Kremen1 | 7748 | 4.794724296 | 5.976371059 | 6.124733257 | 4.351467838 | 6.920361766 | 5.692040899 | 5.30649432 | 5.363104519 | 4.226029947 | 5.385695971 | 4.809559578 | 5.979220437 | 3.883901592 | 5.310978501 | 6.338700906 | 5.480676769 | 5.357173406 | 5.395833907 |
| --- | --- | --- | --- | --- | --- | --- | --- | --- | --- | --- | --- | --- | --- | --- | --- | --- | --- | --- | --- | --- |
| ENSMUSG00000020395 | Itk | 5336 | 0.127451802 | 0.280937076 | 0 | 0.187845385 | 0.036080902 | 0.148583909 | 0.514268249 | 0.035787484 | 0.007718612 | 0.150217758 | 0.196973217 | 0.083681653 | 0 | 0.217597861 | 0 | 0.007175901 | 0.014365179 | 0.288653484 |
| ENSMUSG00000020396 | Nefh | 5897 | 0.072079322 | 0.045193006 | 0.172264565 | 0.054082976 | 0.032648414 | 0.084030411 | 0.20860262 | 0.025906327 | 0.146670651 | 0 | 0 | 0 | 0 | 0 | 0 | 0 | 0.032496436 | 0 |
| ENSMUSG00000020397 | Med7 | 2928 | 8.768144235 | 6.189280893 | 7.533582596 | 7.080012013 | 7.726094305 | 7.497220278 | 8.774175088 | 7.408908663 | 8.678989148 | 8.969583919 | 9.023071051 | 7.682278696 | 8.491559531 | 10.19929925 | 7.443860337 | 8.657234324 | 8.796199289 | 7.364607184 |
| ENSMUSG00000020399 | Havcr2 | 2803 | 0 | 0.011884729 | 0 | 0.130035089 | 0 | 0 | 0.0168793 | 0.05450218 | 0.235099626 | 0 | 0.051132671 | 0.07965132 | 0 | 0 | 0 | 0 | 0 | 0 |
| ENSMUSG00000020400 | Tnip1 | 3694 | 24.88865613 | 32.19464872 | 30.55101416 | 33.21491402 | 30.07267997 | 29.87383261 | 23.82284321 | 29.03201569 | 25.77780846 | 27.49394012 | 25.43945407 | 26.48750853 | 26.24180223 | 29.77249565 | 28.75409071 | 36.95344217 | 32.05962573 | 27.8917317 |
| ENSMUSG00000020401 | Fam71b | 2489 | 13.20068343 | 7.280921838 | 7.210357478 | 9.280614495 | 7.541763853 | 7.425941995 | 14.44661952 | 8.623595883 | 10.60689297 | 12.9195579 | 7.293886791 | 8.745724552 | 6.302751986 | 2.854939884 | 3.360351887 | 3.784447337 | 2.971866333 | 2.099584226 |
| ENSMUSG00000020402 | Vdac1 | 2627 | 163.5648361 | 151.8291901 | 142.7451874 | 147.7135191 | 143.1865183 | 132.3229639 | 175.8151396 | 160.6785045 | 171.675808 | 166.41847 | 150.3992793 | 152.0004759 | 153.0284712 | 121.6173876 | 129.4704392 | 148.8625708 | 141.7795787 | 127.8380649 |
| ENSMUSG00000020405 | Fabp6 | 475 | 0 | 0 | 0 | 0 | 0 | 0 | 0.29881692 | 0 | 0 | 0 | 0 | 0 | 0 | 0 | 0 | 0 | 0 | 0 |
| ENSMUSG00000020407 | Upp1 | 2044 | 0.415901918 | 0.554128377 | 0.094664443 | 0.289771808 | 0.047095816 | 0.266673224 | 0 | 0.093425642 | 0.100749794 | 0 | 0.397345545 | 0.382299059 | 0.241349889 | 0 | 0 | 0 | 0.393763324 | 0.242212309 |
| ENSMUSG00000020409 | Slu7 | 4090 | 22.33340423 | 28.24672791 | 25.36949208 | 25.96645339 | 27.41989784 | 26.96928727 | 27.79764486 | 25.89426207 | 21.97285511 | 25.3276516 | 24.69346265 | 25.58786488 | 20.90274148 | 20.97365022 | 26.90693295 | 21.20494517 | 28.37457873 | 24.73392354 |
| ENSMUSG00000020411 | Nipal4 | 3438 | 0.210176845 | 0.038758457 | 0 | 0 | 0 | 0 | 0 | 0 | 0 | 0 | 0.013896148 | 0.422107978 | 0.129141145 | 0.486325522 | 0 | 0.022274932 | 0.189513394 | 0 |
| ENSMUSG00000020412 | Ascc2 | 2745 | 23.67592501 | 28.8469033 | 25.37627822 | 28.23290944 | 22.70704596 | 25.86851265 | 22.66527242 | 24.64070844 | 24.60688009 | 22.07236928 | 24.87082538 | 22.77360368 | 22.8598174 | 20.13423096 | 25.17122376 | 23.61602854 | 25.29954462 | 21.70304648 |
| ENSMUSG00000020413 | Hus1 | 9653 | 1.052391699 | 1.204413144 | 1.428204304 | 1.217730181 | 0.952366873 | 1.303884215 | 0.828327229 | 1.218611824 | 1.369612717 | 0.957374685 | 1.331344021 | 0.838420292 | 0.822794849 | 1.578127076 | 0.53780131 | 0.606905929 | 1.060097511 | 1.122634831 |
| ENSMUSG00000020415 | Pttg1 | 1519 | 18.52430976 | 44.30022799 | 34.96651503 | 35.63299583 | 33.36597473 | 51.08596497 | 23.11126252 | 54.40971613 | 44.982508 | 41.28402589 | 42.58544686 | 57.17522777 | 16.40067033 | 54.02679553 | 50.64075369 | 16.334637 | 39.25985384 | 54.79182256 |
| ENSMUSG00000020419 | Hormad2 | 3001 | 0 | 0 | 0 | 0 | 0 | 0.016512074 | 0 | 0 | 0.041172791 | 0.062846655 | 0 | 0 | 0.180823422 | 0 | 0.151022031 | 0 | 0 | 0 |
| ENSMUSG00000020420 | Zfp607 | 2057 | 0.80588326 | 1.45754034 | 1.763740776 | 1.107463401 | 0.982761689 | 1.228580171 | 1.104039178 | 1.856704055 | 2.202487474 | 1.146103628 | 1.57933747 | 1.275321411 | 1.151158011 | 1.964334275 | 1.842753107 | 1.563641722 | 2.030902461 | 1.577801311 |
| ENSMUSG00000020422 | Tns3 | 9539 | 2.473044627 | 2.514444264 | 2.327649694 | 1.896187714 | 1.917405493 | 2.348027632 | 1.954208565 | 2.029934796 | 1.865245289 | 1.843716565 | 2.248763639 | 2.211795834 | 1.241184626 | 1.061412208 | 1.213716029 | 1.071767709 | 1.313837833 | 0.766978844 |
| ENSMUSG00000020423 | Btg2 | 2741 | 195.8401562 | 118.7280787 | 157.3330985 | 179.7674916 | 166.9954739 | 158.999012 | 215.9363785 | 165.5886575 | 152.9205293 | 178.9524867 | 213.6365071 | 165.1866672 | 234.8710399 | 302.6066971 | 286.065965 | 406.5286268 | 330.5905282 | 405.5341188 |
| ENSMUSG00000020424 | Gatsl3 | 1565 | 2.335747692 | 1.958329884 | 1.514570599 | 4.075748375 | 1.783802922 | 2.40639473 | 1.360428468 | 1.244608641 | 1.157959549 | 2.440387836 | 0.946341031 | 1.426598405 | 4.980474715 | 1.12771586 | 3.13289786 | 3.596625743 | 1.297951269 | 0.632692601 |
| ENSMUSG00000020427 | Igfbp3 | 3125 | 0.027203313 | 0.117261388 | 0.030959059 | 0.014579534 | 0 | 0 | 0 | 0.134437256 | 0 | 0.135794025 | 0 | 0.107166072 | 0.015786214 | 0 | 0.210951966 | 0.404348814 | 0.012264415 | 0 |
| ENSMUSG00000020428 | Gabra6 | 3577 | 0 | 0 | 0 | 0 | 0 | 0 | 0 | 0 | 0 | 0 | 0 | 0 | 0 | 0 | 0 | 0 | 0 | 0 |
| ENSMUSG00000020429 | Igfbp1 | 1521 | 0 | 0 | 0 | 0.059909329 | 0 | 0 | 0 | 0 | 0 | 0 | 0.062820458 | 0 | 0 | 0 | 0 | 0 | 0 | 0 |
| ENSMUSG00000020430 | Pes1 | 3060 | 23.01669172 | 20.93486784 | 20.67731298 | 22.54229449 | 20.98300202 | 22.31492381 | 21.77001697 | 19.83259066 | 18.61468997 | 21.34108628 | 20.82738386 | 19.77260727 | 17.87878963 | 21.61319622 | 19.4158966 | 24.71370903 | 23.53435029 | 21.15873157 |
| ENSMUSG00000020431 | Adcy1 | 12500 | 0.044205383 | 0.031980378 | 0.019349412 | 0 | 0.050057201 | 0.047570624 | 0.071915272 | 0 | 0 | 0.018860281 | 0 | 0.053583036 | 0.043412087 | 0 | 0.026368996 | 0.030632486 | 0.003066104 | 0 |
| ENSMUSG00000020432 | Tcn2 | 2166 | 134.1483764 | 138.8037259 | 130.916729 | 139.9014339 | 153.7954957 | 149.2758941 | 130.3392221 | 153.5278152 | 145.445826 | 167.6396887 | 169.6165423 | 139.6680015 | 152.1636839 | 163.1546911 | 161.0018324 | 165.8198972 | 178.5552456 | 158.5765754 |
| ENSMUSG00000020434 | 4921536K21R | 2587 | 0 | 0 | 0 | 0.052834609 | 0 | 0.076618065 | 0.164597646 | 0.177158419 | 0.095523423 | 0.054678048 | 0.073869283 | 0.194178958 | 0.095345801 | 0.125670124 | 0.175190226 | 0.310824413 | 0.074074791 | 0.063791001 |
| ENSMUSG00000020435 | Osbp2 | 7843 | 0.021678019 | 0.016989873 | 0 | 0.005809135 | 0 | 0.082135093 | 0.006032472 | 0.048696165 | 0 | 0.156307317 | 0.012182828 | 0.007116622 | 0 | 0.005921725 | 0.073546064 | 0.009764276 | 0 | 0.098192974 |
| ENSMUSG00000020436 | Gabrg2 | 8302 | 0 | 0 | 0 | 0 | 0 | 0 | 0 | 0 | 0 | 0 | 0 | 0 | 0 | 0 | 0 | 0 | 0 | 0 |
| ENSMUSG00000020437 | Myo1g | 5831 | 0.058316139 | 0 | 0 | 0.039067951 | 0.016508977 | 0 | 0.210963754 | 0.196496668 | 0.473245851 | 0.056603485 | 0.999578963 | 0.162727879 | 0.211507105 | 0.167265621 | 0.042395701 | 0.072234039 | 0 | 0 |
| ENSMUSG00000020439 | Smtn | 4353 | 0.76163651 | 1.278027414 | 0.3667187 | 1.025725326 | 0.984089411 | 0.899302999 | 0.141296767 | 0.122833364 | 0 | 0.097485947 | 0.06585108 | 0.089756407 | 0.067997129 | 0.384099965 | 0.113581133 | 0.703709762 | 0.739583951 | 0.581304597 |
| ENSMUSG00000020440 | Arf5 | 1212 | 99.03846296 | 89.10924345 | 90.36111611 | 79.91978569 | 84.23086642 | 83.8553272 | 95.99164809 | 82.34055073 | 82.61090744 | 81.96908561 | 80.96515241 | 90.72347797 | 99.47789259 | 94.76586478 | 86.95839931 | 79.93006331 | 77.22179624 | 79.83582403 |
| ENSMUSG00000020441 | 2310033P09R | 1278 | 20.75370097 | 19.39342199 | 17.94135633 | 17.86078499 | 16.42059368 | 16.86654077 | 16.99258187 | 19.00654768 | 22.55912444 | 17.93054913 | 18.16794196 | 18.03745591 | 26.09419098 | 24.02155068 | 21.60018971 | 21.45232778 | 14.81470318 | 23.93197444 |
| ENSMUSG00000020444 | Guk1 | 2157 | 45.34279557 | 38.641104 | 42.94636561 | 39.52003428 | 38.82686471 | 41.60407998 | 59.20116849 | 48.2849705 | 51.9557299 | 49.68639245 | 51.14157586 | 49.99344465 | 59.85240033 | 58.37270325 | 53.29269399 | 60.03654809 | 49.62695746 | 55.9527029 |
| ENSMUSG00000020447 | Npc1l1 | 4650 | 0 | 0 | 0.01040291 | 0.068586518 | 0 | 0 | 0.60031141 | 0.02464026 | 0.265719457 | 0.34475783 | 0.092467662 | 0.756212203 | 0.084872116 | 0 | 0 | 0 | 0.008242214 | 0.094639323 |
| ENSMUSG00000020448 | Rnf185 | 5798 | 17.51377467 | 15.23154236 | 17.20355633 | 16.690524 | 14.40305063 | 17.13577627 | 11.84857016 | 15.17024883 | 14.34211372 | 16.67108342 | 14.55166897 | 14.18014332 | 15.06844179 | 16.98998092 | 13.1748335 | 18.84151482 | 16.63141825 | 17.0871682 |
| ENSMUSG00000020451 | Limk2 | 5716 | 24.77733494 | 25.39845924 | 23.66206978 | 24.27903097 | 26.7352795 | 25.44388962 | 27.46386786 | 25.4103755 | 26.48733939 | 30.66115535 | 28.74354132 | 29.11866792 | 29.15381675 | 26.62653505 | 27.18899035 | 29.5151183 | 29.09338738 | 30.39166533 |
| ENSMUSG00000020453 | Patz1 | 6074 | 13.33797588 | 13.26702192 | 14.2874734 | 13.50179121 | 13.32859658 | 12.94701814 | 13.36657179 | 13.30509112 | 13.92774175 | 14.79572611 | 12.88366513 | 15.30014458 | 12.71064382 | 13.2129376 | 12.27771093 | 11.49222544 | 13.10565009 | 14.6624537 |
| ENSMUSG00000020454 | Eif4enif1 | 5250 | 13.69881102 | 19.46742085 | 17.79224516 | 16.70571626 | 16.32818211 | 15.98901537 | 14.7795797 | 16.41909566 | 15.87053741 | 14.9175844 | 18.45478396 | 18.73279952 | 14.79017863 | 13.29627873 | 15.58596 | 15.89971888 | 15.76853358 | 16.50273202 |
| ENSMUSG00000020455 | Trim11 | 4103 | 8.971358138 | 10.36004217 | 8.111379207 | 8.328243542 | 8.117789709 | 8.067566666 | 8.867523786 | 9.894838839 | 10.33929106 | 10.15871842 | 9.711020076 | 9.359292191 | 8.332200505 | 11.35350223 | 7.551442854 | 8.847062105 | 8.07066062 | 10.64519394 |
| ENSMUSG00000020456 | Ogdh | 5411 | 79.91224464 | 88.16127245 | 87.80721034 | 96.3004369 | 92.07420844 | 100.0761916 | 89.99988989 | 95.22337846 | 71.29811368 | 92.26236298 | 100.8474544 | 90.3613388 | 75.74377541 | 80.67417804 | 86.65956525 | 77.4869989 | 96.66216482 | 86.38188502 |

| ENSMUSG00000020457 | Drg1 | 3449 | 66.36427165 | 59.08233515 | 64.22220803 | 58.94270218 | 59.57529201 | 58.71905542 | 60.5915056 | 56.89549538 | 60.79459314 | 61.73748214 | 59.05034735 | 55.29780199 | 62.11902486 | 61.08158318 | 54.27041705 | 62.57056054 | 62.58443148 | 56.61998517 |
| --- | --- | --- | --- | --- | --- | --- | --- | --- | --- | --- | --- | --- | --- | --- | --- | --- | --- | --- | --- | --- |
| ENSMUSG00000020458 | Rtn4 | 7603 | 14.35660819 | 13.53897713 | 16.94314236 | 13.33932457 | 12.99049156 | 15.99400345 | 10.26777854 | 12.91498969 | 13.06072465 | 10.62332693 | 11.29180592 | 12.54622746 | 12.95749557 | 9.083566743 | 11.42892034 | 11.70424459 | 11.33708296 | 9.96284887 |
| ENSMUSG00000020459 | Mtif2 | 3569 | 9.646733702 | 9.595308282 | 10.53130655 | 10.60835748 | 10.04715134 | 12.55132282 | 8.497457885 | 9.352804624 | 9.566719408 | 10.81995684 | 11.48526601 | 7.741314929 | 9.454477842 | 11.06121442 | 9.166162896 | 10.00984497 | 8.515761682 | 10.94325183 |
| ENSMUSG00000020460 | Rps27a | 1678 | 47.19138435 | 40.55914356 | 44.07814536 | 40.2392537 | 46.32482522 | 42.19955678 | 46.94613255 | 40.83264 | 40.89197572 | 41.22174117 | 37.12666618 | 42.24427383 | 53.33021331 | 56.43593217 | 49.69722832 | 44.83971606 | 45.49820228 | 55.33693215 |
| ENSMUSG00000020461 | 1700034F02R | 2205 | 0 | 0.075539443 | 0 | 0.144638236 | 0 | 0.17978316 | 0.600795923 | 0.06928327 | 0 | 0.171068311 | 0.238333126 | 0 | 0.290845771 | 0.147441547 | 0.224226155 | 0 | 0.295486189 | 0.099789763 |
| ENSMUSG00000020462 | Ccdc104 | 2733 | 19.54958151 | 18.42996563 | 18.85027802 | 17.82098661 | 21.38022515 | 18.4938852 | 24.40939528 | 19.32678102 | 25.0615352 | 22.35906016 | 24.22835435 | 20.42285495 | 24.80133712 | 21.88802948 | 19.38719388 | 26.1715531 | 20.65665398 | 20.79195289 |
| ENSMUSG00000020463 | Smek2 | 6219 | 17.42855746 | 17.28049473 | 15.9844999 | 16.38849593 | 16.8643611 | 14.36622908 | 16.46319942 | 17.17706215 | 13.95401008 | 14.71611433 | 16.12472067 | 15.91271423 | 12.06525907 | 18.3939198 | 15.55575707 | 14.4690348 | 21.78540916 | 17.58451587 |
| ENSMUSG00000020464 | Pnpt1 | 3659 | 13.87023235 | 12.21806615 | 13.52449346 | 15.1787136 | 11.94418876 | 13.47498494 | 13.20203476 | 14.24778062 | 11.14365964 | 11.86821473 | 13.04377795 | 11.54754211 | 10.77239735 | 12.75657497 | 13.24215079 | 13.40537526 | 15.7222661 | 13.81617083 |
| ENSMUSG00000020467 | Efemp1 | 2140 | 3.575201722 | 6.584744979 | 5.086002023 | 7.174799973 | 9.333994543 | 6.36775782 | 6.3673138 | 5.746707278 | 10.43135119 | 8.350521745 | 7.166243772 | 6.46835716 | 6.546852573 | 4.362271759 | 4.813265875 | 6.924516385 | 6.895151546 | 5.526615862 |
| ENSMUSG00000020469 | Myl7 | 583 | 0 | 0 | 0 | 0 | 0.082559046 | 0 | 0 | 0.196530373 | 0 | 0 | 0 | 0 | 0 | 0 | 0 | 0 | 0 | 0.283065729 |
| ENSMUSG00000020471 | Pold2 | 2115 | 6.89327441 | 5.197756545 | 8.531123793 | 6.764145589 | 5.165932233 | 5.177850648 | 6.30835719 | 6.031329742 | 7.867306084 | 9.051151547 | 8.741800913 | 6.386472977 | 6.227717216 | 6.609773193 | 8.298753101 | 6.970157849 | 8.806893756 | 6.91840302 |
| ENSMUSG00000020472 | Zkscan17 | 4560 | 7.438405805 | 8.620441925 | 7.521235312 | 10.94064551 | 9.088067166 | 7.780648518 | 7.688310326 | 6.733923582 | 7.78569662 | 8.685655863 | 9.617853483 | 6.830074501 | 6.480223344 | 7.781421659 | 10.26424726 | 7.339033085 | 8.547772796 | 6.140270904 |
| ENSMUSG00000020473 | Aebp1 | 4038 | 0.063157765 | 0.15674715 | 0.287509839 | 0.440039804 | 0.14303692 | 0.147259238 | 0.187469753 | 0.293205665 | 0.234593824 | 0.221858188 | 0.035494026 | 0.539081437 | 1.73480244 | 0.920140414 | 1.224414737 | 2.105129976 | 0.844735122 | 1.198811637 |
| ENSMUSG00000020474 | Polm | 4608 | 3.689685419 | 3.585762919 | 2.162531956 | 3.440808036 | 3.415611771 | 3.279857583 | 4.476633683 | 3.240718173 | 5.023181827 | 3.315283823 | 2.664532189 | 4.251583674 | 2.869130605 | 4.031604808 | 3.370873822 | 2.900048173 | 1.962891942 | 2.936684083 |
| ENSMUSG00000020475 | Pgam2 | 837 | 0.710958739 | 1.3134116 | 1.387054634 | 1.088674895 | 1.725158554 | 0.59202788 | 1.469688951 | 1.642683974 | 1.82067035 | 1.971654258 | 1.198654872 | 2.000561383 | 1.060901447 | 1.054286333 | 0.984505517 | 2.058634807 | 0.641061119 | 0.591495771 |
| ENSMUSG00000020476 | Dbnl | 2453 | 76.51971355 | 74.85555358 | 79.78773087 | 77.91625805 | 72.38429124 | 73.02614427 | 79.75455661 | 77.13214901 | 74.07864148 | 79.13552601 | 77.41763553 | 75.45239997 | 72.64039348 | 62.89737395 | 67.65587735 | 63.84369518 | 66.05934909 | 70.70677273 |
| ENSMUSG00000020477 | Mrps24 | 1868 | 36.36149427 | 26.50051435 | 27.91577394 | 26.41467399 | 30.81679909 | 29.25945671 | 31.83727913 | 28.58296497 | 30.75759609 | 31.09725183 | 29.20717483 | 31.10498113 | 32.43018973 | 32.9186144 | 28.34261206 | 33.49403238 | 25.68764653 | 27.7401384 |
| ENSMUSG00000020481 | Ankrd36 | 5619 | 0 | 0 | 0 | 0 | 0 | 0 | 0 | 0 | 0 | 0 | 0 | 0 | 0 | 0 | 0 | 0 | 0 | 0 |
| ENSMUSG00000020482 | Ccdc117 | 3367 | 6.665498349 | 6.134242476 | 5.890450682 | 6.589910482 | 6.961760269 | 6.99065888 | 6.997836087 | 8.552738763 | 5.308864818 | 8.514293906 | 7.534452911 | 4.625057874 | 9.347717025 | 10.86959931 | 7.501203968 | 5.720277851 | 6.727306644 | 9.230812384 |
| ENSMUSG00000020483 | Dynll2 | 3233 | 25.66350251 | 17.65078497 | 18.65814796 | 17.99611928 | 17.85034843 | 19.20494128 | 23.85390246 | 21.33482176 | 24.06474738 | 20.65122048 | 14.49651539 | 20.47552608 | 21.926992 | 22.84135445 | 19.09060647 | 19.29334966 | 17.98360421 | 19.34591845 |
| ENSMUSG00000020484 | Xbp1 | 3467 | 69.74673967 | 65.69375765 | 66.19095126 | 68.04588623 | 65.94365083 | 69.16229529 | 63.19729337 | 73.27829847 | 66.39499181 | 64.51772029 | 67.0392769 | 66.74696772 | 77.23497173 | 74.49540524 | 66.70445146 | 71.20263799 | 69.68819604 | 75.34994169 |
| ENSMUSG00000020485 | Supt4h1 | 1051 | 45.9023547 | 34.42226748 | 40.27292013 | 37.28115902 | 40.52973591 | 34.79535431 | 42.00069404 | 39.71864494 | 41.18651576 | 38.71651466 | 44.54753502 | 45.30043786 | 47.97071309 | 47.85817949 | 48.14035265 | 48.60101834 | 44.45266977 | 42.1858611 |
| ENSMUSG00000020486 | 4-Sep | 2700 | 0.472279734 | 0.185071635 | 0.197077346 | 0.269991374 | 0.124786469 | 0.183528643 | 2.295541089 | 1.838893449 | 1.800003281 | 0.436580586 | 0.336194152 | 1.633124942 | 0.164439724 | 0.602052985 | 0.289936875 | 0.382906074 | 0.127754323 | 0.264858662 |
| ENSMUSG00000020490 | Btnl10 | 4867 | 0 | 0 | 0 | 0.065528521 | 0 | 0 | 0 | 0 | 0 | 0 | 0 | 0 | 0 | 0 | 0 | 0 | 0 | 0 |
| ENSMUSG00000020491 | 2810021J22R | 1828 | 7.440731033 | 6.815657789 | 3.889993962 | 5.308808777 | 5.476717792 | 7.915425714 | 6.677610046 | 5.494858771 | 11.37811294 | 6.577368343 | 11.47330786 | 10.77840749 | 5.073523221 | 7.037752848 | 7.933778815 | 4.901532895 | 6.604367355 | 2.437493241 |
| ENSMUSG00000020492 | Fam33a | 3320 | 2.739791467 | 2.097106895 | 1.471604387 | 1.509552674 | 2.377601049 | 1.895541315 | 2.337132333 | 3.278564664 | 3.13861099 | 2.584767468 | 2.273627542 | 2.135117213 | 2.719199055 | 1.734658686 | 2.990835834 | 2.122130049 | 2.79366381 | 2.071126006 |
| ENSMUSG00000020493 | Prr11 | 3814 | 0.05572257 | 0.061140603 | 0.063415745 | 0.155294593 | 0.07571881 | 0.051969306 | 0.58303511 | 0.170233571 | 0.226774208 | 0.197800538 | 0.250524166 | 0 | 0 | 0.048709059 | 0.075619007 | 0.060236928 | 0.190928065 | 0.20192121 |
| ENSMUSG00000020495 | 1200011M11R | 3628 | 4.018543379 | 3.865691418 | 4.320017595 | 6.153503783 | 4.98831403 | 4.042891163 | 4.460015489 | 3.600276081 | 2.429414105 | 5.523442361 | 6.044296015 | 5.369257509 | 2.93707115 | 4.92860354 | 6.075753143 | 4.000038645 | 4.215047537 | 4.397089567 |
| ENSMUSG00000020496 | Rnf187 | 1945 | 100.0674555 | 93.94407444 | 97.31908703 | 97.54045687 | 92.15592994 | 94.39222515 | 99.95259523 | 98.80934134 | 83.68591789 | 86.93183644 | 107.3401381 | 95.10185389 | 103.5843446 | 102.7023238 | 87.69894159 | 97.92158417 | 89.77614843 | 106.3980769 |
| ENSMUSG00000020513 | Tubd1 | 2299 | 5.176793949 | 4.593382978 | 4.187182488 | 6.262414097 | 4.899030073 | 4.310807619 | 1.502316469 | 2.724468897 | 4.317507742 | 3.548095547 | 3.574290065 | 3.738848212 | 4.291597849 | 2.84846295 | 3.36924424 | 3.18116834 | 3.534221376 | 3.014853174 |
| ENSMUSG00000020514 | Mrpl22 | 1012 | 24.02466471 | 22.05497937 | 21.22316944 | 18.86371302 | 21.49765761 | 23.50327284 | 25.71341246 | 19.17168026 | 21.65140947 | 21.94464549 | 24.78443992 | 19.80022023 | 24.95844036 | 25.10367174 | 21.78145494 | 16.87510959 | 24.16223066 | 17.88339537 |
| ENSMUSG00000020515 | Cnot8 | 2626 | 45.51582444 | 45.4024556 | 48.63142427 | 44.9364451 | 44.70440283 | 43.77850034 | 39.96173719 | 36.43258493 | 41.31198877 | 38.83738429 | 40.3339996 | 34.22056998 | 30.7901799 | 30.17273918 | 32.74472473 | 31.67067756 | 35.84515811 | 30.2068286 |
| ENSMUSG00000020516 | Rps6kb1 | 9568 | 7.285586192 | 7.301122406 | 7.229739591 | 5.885603135 | 6.630212729 | 6.085332562 | 5.652005854 | 5.488561173 | 5.608907822 | 5.381330263 | 6.466196826 | 5.89774612 | 6.893475481 | 4.23278054 | 5.727222966 | 5.20252818 | 6.164733584 | 7.502809811 |
| ENSMUSG00000020519 | Sap30l | 1147 | 19.30705903 | 19.54627534 | 18.93610735 | 17.91458674 | 18.75760234 | 17.1512077 | 15.63339609 | 16.28254994 | 18.99535034 | 17.3064046 | 15.07806013 | 15.52327495 | 18.66613086 | 18.99065125 | 18.3197851 | 17.55959851 | 16.80743447 | 19.3754831 |
| ENSMUSG00000020520 | Galnt10 | 6205 | 4.452597005 | 3.870845565 | 4.077253243 | 4.295440924 | 4.615389941 | 4.296433629 | 2.668724839 | 2.492815951 | 2.05766638 | 3.761417907 | 2.35602535 | 3.23829791 | 2.671317358 | 5.478980172 | 3.625471316 | 3.702556719 | 4.187788767 | 3.989379214 |
| ENSMUSG00000020521 | Rnft1 | 4823 | 12.45279157 | 10.66454669 | 11.84514604 | 10.96752488 | 12.99352366 | 11.34277791 | 10.61420663 | 9.906426583 | 11.18688278 | 11.1839943 | 9.311312662 | 9.767451632 | 11.8138844 | 10.78527428 | 10.95177165 | 11.36888862 | 13.81908155 | 9.979881481 |
| ENSMUSG00000020522 | Mfap3 | 5823 | 14.01510183 | 12.70615457 | 12.88465492 | 12.58151456 | 13.46503583 | 13.71784416 | 10.48955324 | 10.21218797 | 9.294020558 | 8.712717964 | 10.68229365 | 10.63018544 | 9.12424436 | 8.677857991 | 9.920072361 | 9.580871541 | 10.30722668 | 10.3443194 |
| ENSMUSG00000020523 | Fam114a2 | 4242 | 20.21049978 | 19.04379266 | 18.30257337 | 18.36619185 | 19.03945495 | 18.66696564 | 17.2542938 | 19.51936078 | 21.37970479 | 19.94067931 | 18.32387021 | 18.80258883 | 20.89803285 | 22.0833862 | 20.67848008 | 21.01379869 | 19.18121837 | 22.49900994 |
| ENSMUSG00000020524 | Gria1 | 6022 | 0 | 0 | 0.048196809 | 0 | 0 | 0 | 0 | 0 | 0 | 0 | 0 | 0 | 0 | 0 | 0 | 0 | 0 | 0 |
| ENSMUSG00000020525 | Ppm1d | 3166 | 3.81284586 | 6.050193991 | 5.194883235 | 6.605344078 | 5.655425017 | 5.368473663 | 4.318813711 | 6.212598622 | 5.099530694 | 6.552845683 | 7.982613088 | 7.140032643 | 5.858749493 | 8.391035372 | 6.72811257 | 7.595231032 | 6.331223399 | 8.809102056 |
| ENSMUSG00000020526 | Znhit3 | 2242 | 8.834706525 | 6.805221031 | 6.882763686 | 7.132884283 | 7.964738484 | 6.033852037 | 7.681451887 | 7.836086131 | 9.736330666 | 7.297633377 | 7.756505302 | 8.364880744 | 9.175472571 | 8.431196952 | 9.427474653 | 9.034670524 | 7.282338305 | 7.434326199 |

| ENSMUSG00000020527 | Myo19 | 7428 | 1.556463096 | 1.403733965 | 1.706228454 | 1.423015924 | 1.619948966 | 1.407596498 | 1.605115117 | 2.097805625 | 1.602437137 | 1.459970618 | 1.755864791 | 1.901098901 | 0.949712462 | 1.681941237 | 1.84153427 | 1.149543006 | 1.97100773 | 1.140468825 |
| --- | --- | --- | --- | --- | --- | --- | --- | --- | --- | --- | --- | --- | --- | --- | --- | --- | --- | --- | --- | --- |
| ENSMUSG00000020528 | Prpsap2 | 2597 | 17.49635471 | 16.57306867 | 18.40317597 | 17.15775948 | 16.56909502 | 14.80666972 | 18.83762419 | 18.47118114 | 18.4126087 | 19.84432756 | 21.10045336 | 19.23566346 | 23.06081924 | 21.63933221 | 22.86154872 | 24.60801839 | 21.72364615 | 21.01233495 |
| ENSMUSG00000020530 | Ggnbp2 | 5077 | 33.05306972 | 32.92576387 | 29.71775481 | 27.71174018 | 34.00614737 | 32.18926834 | 32.38360435 | 31.24149048 | 36.14873237 | 30.75893819 | 33.28344064 | 30.32097645 | 32.61911489 | 38.26582973 | 32.76972281 | 35.98276303 | 34.40836346 | 34.49852192 |
| ENSMUSG00000020532 | Acaca | 10128 | 0.780604536 | 0.64797 | 0.659118008 | 0.724262257 | 0.541769283 | 0.606688286 | 0.789478943 | 1.089810851 | 0.825519619 | 0.488825419 | 0.735870214 | 0.573146614 | 1.354094886 | 1.398642047 | 1.049565701 | 1.20981382 | 1.014163465 | 1.064552223 |
| ENSMUSG00000020534 | Shmt1 | 4275 | 4.871938305 | 5.065118419 | 6.291387808 | 5.019708043 | 5.989750501 | 5.26244235 | 6.994529376 | 6.083982699 | 7.668881064 | 5.735290221 | 5.598889871 | 7.82072091 | 2.55025818 | 3.596021737 | 2.852786092 | 2.337742347 | 2.958521164 | 2.123158505 |
| ENSMUSG00000020536 | Llgl1 | 4937 | 18.75152388 | 20.43172852 | 21.46777497 | 22.07454285 | 21.48729183 | 22.26209501 | 13.3207664 | 13.47603099 | 17.08527127 | 16.95209607 | 12.0864742 | 16.14437233 | 11.35123719 | 10.9313408 | 13.31936058 | 12.68080679 | 13.71735196 | 13.60464236 |
| ENSMUSG00000020537 | Drg2 | 2822 | 28.97943256 | 27.87092248 | 24.06677414 | 23.4263201 | 27.63065781 | 22.96774469 | 21.69475781 | 24.80746761 | 28.75174913 | 27.75241606 | 28.11984622 | 27.17601715 | 32.65486232 | 32.50427803 | 31.63847329 | 32.59179269 | 31.22330141 | 27.83593352 |
| ENSMUSG00000020538 | Srebf1 | 5159 | 33.38456547 | 37.1355795 | 33.80239909 | 36.39408097 | 33.61491006 | 40.8697192 | 24.66055314 | 28.05760906 | 24.94821899 | 29.25555362 | 27.19811069 | 24.82980144 | 31.16354301 | 41.75376571 | 43.03042899 | 34.64635692 | 36.32789142 | 35.12308539 |
| ENSMUSG00000020541 | Tom1l1 | 7978 | 4.571251069 | 4.288335719 | 4.153405402 | 3.900500537 | 4.223157003 | 3.54036828 | 4.667219644 | 3.595198572 | 4.078383987 | 4.23162491 | 5.814675944 | 3.512091078 | 4.291345023 | 4.272995756 | 4.570490971 | 5.879417657 | 5.956957654 | 4.06810892 |
| ENSMUSG00000020542 | Myocd | 9063 | 0 | 0 | 0 | 0 | 0 | 0 | 0 | 0 | 0 | 0 | 0 | 0 | 0 | 0 | 0 | 0 | 0 | 0 |
| ENSMUSG00000020544 | Cox11 | 1419 | 7.278899066 | 5.798650368 | 4.840762023 | 6.036276487 | 6.207288253 | 5.552420464 | 6.435057811 | 7.72460852 | 5.311580257 | 4.784849379 | 6.262256714 | 5.742837729 | 6.222983222 | 6.742411559 | 6.010374961 | 5.369859671 | 5.482902235 | 6.473939974 |
| ENSMUSG00000020545 | 1700108M19R | 1956 | 0 | 0.034062264 | 0.148385062 | 0 | 0 | 0 | 0 | 0 | 0 | 0 | 0.024424825 | 0 | 0.07566245 | 0.094977684 | 0 | 0.097879876 | 0 | 0 |
| ENSMUSG00000020546 | Stxbp4 | 11814 | 2.101153107 | 1.595996117 | 1.736107743 | 1.6891601 | 1.804845284 | 1.384154569 | 1.702038983 | 1.312520347 | 1.694315783 | 1.704194201 | 1.427506377 | 2.333920545 | 1.69116527 | 1.387740211 | 1.004405629 | 1.037158826 | 1.216553355 | 1.634348778 |
| ENSMUSG00000020547 | Bzw2 | 1848 | 33.16691766 | 28.6079887 | 24.76264054 | 30.34937531 | 29.27504448 | 27.29690627 | 21.37775265 | 23.0849099 | 20.50410958 | 21.27905115 | 26.70537452 | 25.28014588 | 22.396904 | 15.28030581 | 22.45128073 | 23.0199485 | 19.47423852 | 18.75310456 |
| ENSMUSG00000020548 | 1700086D15R | 1089 | 0 | 0 | 0.133260414 | 0 | 0.044198277 | 0.227514847 | 0 | 0 | 0 | 0 | 0 | 0 | 0 | 0.213241907 | 0 | 0.105483767 | 0 | 0 |
| ENSMUSG00000020549 | Elac2 | 3715 | 9.164642261 | 9.810042066 | 8.346549921 | 8.462212811 | 8.486247645 | 8.163195949 | 7.883323891 | 10.65069418 | 6.496715541 | 10.16627545 | 9.683591853 | 11.41854739 | 9.720313175 | 8.55121286 | 10.38079578 | 11.46141465 | 9.336554158 | 10.7649046 |
| ENSMUSG00000020553 | Pctp | 2717 | 8.181894392 | 5.934280752 | 8.955423544 | 7.864604267 | 5.704261841 | 7.34992699 | 10.83124265 | 10.35988243 | 9.125610044 | 11.03711714 | 10.00513485 | 11.0932859 | 13.19996462 | 11.50418506 | 8.704340479 | 9.752337255 | 10.14229204 | 8.989342431 |
| ENSMUSG00000020561 | Twistnb | 2320 | 10.46140324 | 6.906681948 | 7.110074936 | 5.911152735 | 7.365014182 | 6.493116814 | 8.463259377 | 10.48644842 | 7.420673961 | 7.479077068 | 7.866394025 | 6.784490021 | 7.08083123 | 7.807411242 | 7.370105903 | 9.176542119 | 10.75449107 | 8.441052583 |
| ENSMUSG00000020562 | Efcab10 | 671 | 0 | 0.198586553 | 0.144183399 | 0.475301506 | 0 | 0 | 0 | 0 | 0 | 0 | 0.21359892 | 0 | 0.294079984 | 0.553729805 | 0 | 0.399454921 | 0.799654481 | 0.16396157 |
| ENSMUSG00000020564 | Atxn7l1 | 12068 | 2.856454902 | 2.956422747 | 3.399134384 | 3.424251509 | 2.883607956 | 2.656663791 | 3.367715545 | 3.060329228 | 3.327548299 | 3.328836524 | 2.992862828 | 2.70105626 | 3.64225541 | 2.68242699 | 2.533274318 | 3.750372717 | 2.778878836 | 3.427813081 |
| ENSMUSG00000020566 | Atp6v1c2 | 2189 | 154.1565064 | 148.0437802 | 143.728388 | 141.345387 | 151.2120781 | 145.6021847 | 167.5288142 | 165.2445149 | 187.5689245 | 181.3869676 | 193.7188358 | 174.178525 | 240.8677624 | 246.3721986 | 226.0533057 | 288.8325763 | 243.8070737 | 261.5508373 |
| ENSMUSG00000020570 | Sypl | 6631 | 33.80030059 | 37.55299115 | 37.59143446 | 39.27415615 | 39.69014607 | 36.1613147 | 36.79557913 | 36.90223529 | 39.33557598 | 37.99218934 | 38.19258858 | 35.75704037 | 30.95613147 | 31.5323905 | 30.6075802 | 30.39111246 | 35.5924199 | 30.38732588 |
| ENSMUSG00000020571 | Pdia6 | 3433 | 135.3529229 | 136.1141763 | 122.1244939 | 137.5988662 | 134.4272893 | 138.3377217 | 161.2324587 | 167.6549677 | 154.2124887 | 163.688343 | 150.6866445 | 162.6832275 | 180.155621 | 212.1708775 | 170.8790425 | 164.2603482 | 169.2586622 | 187.7648449 |
| ENSMUSG00000020572 | Nampt | 4536 | 25.03832239 | 27.28337787 | 29.20967805 | 25.33177994 | 28.15123313 | 27.34358292 | 30.39443263 | 31.827002 | 37.7452262 | 32.58762229 | 31.16536643 | 28.81840427 | 34.43228618 | 31.26989482 | 37.58642819 | 34.03609547 | 38.0305693 | 34.50196398 |
| ENSMUSG00000020573 | Pik3cg | 6801 | 0 | 0 | 0 | 0 | 0 | 0 | 0 | 0 | 0 | 0 | 0 | 0 | 0.137818913 | 0.300476378 | 0.012116323 | 0 | 0 | 0.113237391 |
| ENSMUSG00000020576 | Nbas | 8166 | 1.37415705 | 2.468075068 | 2.268805061 | 1.880243931 | 2.481452347 | 2.839906847 | 1.732364805 | 1.70242891 | 1.881284641 | 1.535891142 | 1.562076159 | 2.077879185 | 1.516325158 | 1.899623462 | 1.4228311 | 1.130055888 | 1.267217752 | 1.508944392 |
| ENSMUSG00000020577 | Tspan13 | 2466 | 73.23783169 | 102.6539672 | 85.35005295 | 72.73878003 | 91.28670194 | 94.60432672 | 60.03299772 | 69.95745404 | 72.88643746 | 48.01111755 | 77.78445186 | 65.91046612 | 59.33433359 | 78.70634274 | 69.05354035 | 67.96349903 | 69.17694546 | 69.59789658 |
| ENSMUSG00000020580 | Rock2 | 8161 | 6.515620048 | 6.914844113 | 5.968894139 | 7.86055023 | 5.655987599 | 7.249842408 | 5.211873343 | 6.916844842 | 5.450488546 | 5.858450325 | 5.754537947 | 5.341506247 | 6.286630801 | 6.880394762 | 6.765112718 | 6.498283452 | 7.84277856 | 5.554160512 |
| ENSMUSG00000020581 | Agr2 | 1283 | 1.788994159 | 1.454031236 | 3.544124593 | 1.562498792 | 1.01290876 | 1.583524612 | 0.737532018 | 0.922809411 | 0 | 0 | 0 | 0 | 0 | 0.0361996 | 0 | 0.029844589 | 0.089617218 | 0 |
| ENSMUSG00000020583 | Matn3 | 3006 | 0 | 0 | 0 | 0 | 0 | 0 | 0 | 0 | 0 | 0 | 0 | 0 | 0 | 0 | 0.027412878 | 0 | 0.012749932 | 0 |
| ENSMUSG00000020585 | Laptm4a | 2780 | 559.1265403 | 485.589357 | 513.2988388 | 487.4376189 | 506.8049171 | 490.1620938 | 432.7238037 | 430.2964877 | 461.866773 | 431.632463 | 424.217572 | 430.4230844 | 508.2784556 | 416.9442766 | 448.1336135 | 496.235253 | 471.0274317 | 450.6195638 |
| ENSMUSG00000020589 | Fam49a | 4833 | 0.131921713 | 0.006892798 | 0.050045035 | 0.103697804 | 0.199180317 | 0.061517981 | 0.450317242 | 0 | 0.323833561 | 0 | 0.21747343 | 0 | 0.020414615 | 0 | 0.068200382 | 0 | 0.015860251 | 0 |
| ENSMUSG00000020590 | Snx13 | 7618 | 12.93902641 | 15.69877793 | 14.59206783 | 14.36566402 | 13.37559496 | 16.35929705 | 10.57672515 | 11.96708645 | 12.9755366 | 13.56093343 | 12.54892254 | 13.69381378 | 11.20944458 | 10.88247519 | 12.85047214 | 13.14386169 | 13.07561639 | 11.81345479 |
| ENSMUSG00000020591 | Ntsr2 | 2192 | 8.648407166 | 13.06983989 | 14.80777321 | 7.233231491 | 13.72374648 | 8.567739975 | 1.143965321 | 2.822613681 | 0.206684158 | 0.064531072 | 0.108975726 | 0 | 0.045010873 | 0.042375992 | 0 | 0.034936686 | 0 | 0.351335536 |
| ENSMUSG00000020592 | Sdc1 | 3526 | 54.58407177 | 67.78786614 | 51.95421425 | 57.66844608 | 59.54380347 | 60.59880522 | 16.98747916 | 24.43620528 | 24.763305 | 25.47421716 | 26.39410639 | 24.31448036 | 21.46204229 | 19.25730453 | 23.04295752 | 18.07021291 | 22.18490413 | 19.31403123 |
| ENSMUSG00000020593 | Lpin1 | 5703 | 7.177360076 | 8.166127673 | 6.633017839 | 9.754521328 | 7.283508702 | 7.055377813 | 6.130820573 | 7.801884763 | 6.730814079 | 8.664551467 | 6.559318339 | 5.539481856 | 9.117278767 | 10.04130453 | 11.84102229 | 9.379620996 | 9.845348933 | 10.78382979 |
| ENSMUSG00000020594 | Pum2 | 7257 | 6.14998413 | 5.917089796 | 6.13251315 | 5.210922805 | 5.259558422 | 5.13485678 | 5.339545824 | 5.099688291 | 5.919462028 | 5.145839463 | 5.325884168 | 5.853068655 | 5.037198664 | 4.019138333 | 4.258118633 | 5.777623688 | 4.409874316 | 5.541085437 |
| ENSMUSG00000020598 | Nrcam | 8372 | 0 | 0.067644434 | 0 | 0 | 0 | 0.017756593 | 0 | 0 | 0 | 0 | 0.01711955 | 0.020000835 | 0.088387334 | 0.01664265 | 0 | 0 | 0 | 0 |
| ENSMUSG00000020599 | Rgs9 | 6784 | 25.71989204 | 30.31748362 | 30.49015563 | 30.26209696 | 29.18849263 | 29.05671789 | 19.98095889 | 24.87798146 | 19.39117502 | 21.75437812 | 22.32408879 | 23.39913685 | 14.42725883 | 14.81500665 | 18.17144092 | 13.6816675 | 20.6941665 | 21.21223809 |
| ENSMUSG00000020600 | Slc7a15 | 3211 | 0.026474728 | 0.082996934 | 0.015064942 | 0 | 0.029979398 | 0.030864362 | 0.014734562 | 0.451981094 | 0.025653389 | 0.029368236 | 0 | 0 | 8.44987683 | 20.62574544 | 7.673164255 | 1.335580202 | 4.726631446 | 7.503577934 |
| ENSMUSG00000020601 | Trib2 | 4102 | 17.5222459 | 15.33270217 | 15.44839707 | 18.55985009 | 16.00486199 | 15.92162429 | 22.90662612 | 26.11645264 | 26.83850722 | 29.54103056 | 32.08678952 | 23.18622356 | 22.57338097 | 27.29807282 | 23.23237415 | 20.7601928 | 28.99232065 | 22.22088977 |

| ENSMUSG00000020604 | Arsg | 3891 | 7.373681269 | 6.575251288 | 8.118199774 | 7.997479641 | 9.759981438 | 9.194827459 | 7.794250114 | 10.924735 | 12.83969253 | 8.397696571 | 9.613927651 | 6.971578519 | 8.507251733 | 8.068929086 | 8.905296454 | 7.626630875 | 9.741636504 | 9.387316079 |
| --- | --- | --- | --- | --- | --- | --- | --- | --- | --- | --- | --- | --- | --- | --- | --- | --- | --- | --- | --- | --- |
| ENSMUSG00000020605 | Hs1bp3 | 2951 | 8.051641274 | 7.755323053 | 8.212517353 | 7.704154905 | 7.225497182 | 8.328754949 | 7.872085853 | 5.642794798 | 6.154948645 | 6.375171332 | 6.58909119 | 4.974421979 | 6.335749457 | 6.358323045 | 4.56554008 | 6.721292658 | 5.74050262 | 4.809335661 |
| ENSMUSG00000020607 | Fam84a | 3377 | 23.47413482 | 23.65541023 | 17.6333479 | 22.26109658 | 17.27447186 | 19.14904274 | 2.746012754 | 3.019653966 | 2.146528509 | 2.778498652 | 1.881572247 | 2.280888788 | 5.390428627 | 4.455991802 | 6.234526225 | 2.891354719 | 3.143732378 | 4.430700926 |
| ENSMUSG00000020608 | Smc6 | 5576 | 17.6164745 | 17.15829128 | 15.42470175 | 14.92009809 | 14.26866388 | 16.35169114 | 11.92999042 | 13.30843577 | 12.65288764 | 11.77921981 | 11.01839968 | 12.79275767 | 10.20080714 | 8.670784608 | 10.30038897 | 10.83618696 | 11.3136809 | 9.53977873 |
| ENSMUSG00000020609 | Apob | 13986 | 0 | 0 | 0 | 0.003257618 | 0 | 0.003543024 | 0.00676572 | 0 | 0 | 0.013485115 | 0 | 0 | 0.007054471 | 0 | 0 | 0 | 0 | 0.003933155 |
| ENSMUSG00000020610 | Amz2 | 5626 | 5.704125116 | 7.229833601 | 6.99894307 | 6.753983465 | 7.066649298 | 6.412084971 | 7.778924282 | 6.510222877 | 8.579914055 | 7.634961014 | 8.406898133 | 6.458584491 | 8.06707499 | 7.561817286 | 6.876690541 | 7.41174395 | 6.805540456 | 6.854138609 |
| ENSMUSG00000020611 | Gna13 | 6416 | 17.14516764 | 17.28469839 | 17.46905702 | 17.31972992 | 17.40431155 | 18.35829921 | 12.6393285 | 16.78060823 | 13.46778524 | 14.93301574 | 13.82018749 | 14.13660407 | 15.84680198 | 14.09392739 | 16.18907768 | 20.68504446 | 20.61472111 | 18.93081478 |
| ENSMUSG00000020612 | Prkar1a | 4191 | 171.501438 | 178.1059463 | 167.9396007 | 167.6659477 | 174.9447848 | 169.727867 | 153.2497295 | 157.8181877 | 154.8498995 | 162.1304664 | 164.2542059 | 158.4839859 | 181.7429737 | 168.6770351 | 184.6156092 | 206.4458518 | 190.3786325 | 190.9764979 |
| ENSMUSG00000020614 | Fam20a | 3622 | 0.316852499 | 0.395487148 | 0.093488325 | 0.251579483 | 0.292352932 | 0.164172502 | 0.209001343 | 0.231980357 | 0.159196913 | 0.650893198 | 0.408896663 | 0.277383193 | 14.64158229 | 14.5410257 | 10.74971571 | 16.71381842 | 20.81386693 | 15.56718233 |
| ENSMUSG00000020617 | 1700012B07R | 2198 | 0 | 0 | 0 | 0 | 0 | 0 | 0 | 0 | 0 | 0 | 0 | 0 | 0 | 0 | 0.018745021 | 0 | 0 | 0 |
| ENSMUSG00000020620 | Abca8b | 7722 | 0.440354064 | 0.276097543 | 0.288161408 | 0.141603867 | 0.461248686 | 0.628874371 | 0.006126998 | 0.272025658 | 0.421359071 | 0.14043851 | 0.136110993 | 0.354178641 | 1.303251894 | 1.09464179 | 0.645608426 | 1.586764357 | 1.196145759 | 0.86196606 |
| ENSMUSG00000020621 | Rdh14 | 1470 | 57.10729432 | 45.73157859 | 48.83423064 | 48.81540465 | 51.76637505 | 50.86739794 | 47.79546137 | 42.94696679 | 45.75345595 | 44.00732304 | 46.70245941 | 43.74125395 | 42.55297354 | 39.99878547 | 48.54496245 | 34.04477815 | 40.93352798 | 44.56860279 |
| ENSMUSG00000020622 | Nt5c1b | 2224 | 0.095560198 | 0.014978819 | 0.108753441 | 0.081944324 | 0.151494364 | 0.178247243 | 0 | 0.206074114 | 0.148152934 | 0.021200856 | 0.064444638 | 0.125484853 | 0.044363235 | 0.271480727 | 0.055577638 | 0.051650999 | 0.206796566 | 0 |
| ENSMUSG00000020623 | Map2k6 | 3595 | 1.19416489 | 0.898845825 | 0.524775434 | 0.735059964 | 1.405800274 | 0.951081674 | 2.500531014 | 1.455454556 | 2.142386216 | 3.370717868 | 1.794052683 | 1.506013705 | 1.87996458 | 2.183324275 | 1.524285656 | 1.618963095 | 1.727082086 | 3.917199255 |
| ENSMUSG00000020624 | 4933434M16R | 2145 | 0 | 0 | 0 | 0 | 0 | 0 | 0 | 0 | 0 | 0 | 0 | 0 | 0 | 0 | 0 | 0 | 0 | 0 |
| ENSMUSG00000020627 | Klhl29 | 7027 | 0.024195347 | 0.151702379 | 0.082607423 | 0.090771968 | 0.054796555 | 0.042310574 | 0.161591617 | 0.173922994 | 0.035167083 | 0.073809269 | 0.027195081 | 0.079430287 | 0.940725333 | 1.050890835 | 0.85604485 | 1.874479713 | 0.970838316 | 0.806309662 |
| ENSMUSG00000020628 | Ttc15 | 5426 | 12.97246065 | 12.36494083 | 14.13049127 | 14.90432247 | 13.62525521 | 14.61193766 | 12.86144516 | 14.38022081 | 12.4333787 | 13.49521603 | 11.79846007 | 13.19784281 | 14.17406175 | 12.54829195 | 13.72119637 | 12.37775993 | 14.49420591 | 13.07809577 |
| ENSMUSG00000020629 | Adi1 | 1881 | 79.67743258 | 73.56818851 | 79.18240296 | 79.49566587 | 75.07658907 | 66.14934291 | 83.50775866 | 80.40505769 | 95.13844284 | 85.90401917 | 71.90372539 | 72.34374555 | 84.97363741 | 88.02401464 | 81.30790828 | 76.19444099 | 82.72448986 | 76.97180696 |
| ENSMUSG00000020630 | Rnaseh1 | 2460 | 10.78180075 | 9.953242785 | 8.907808639 | 10.0938086 | 8.648093602 | 8.117785216 | 10.19338205 | 9.144441064 | 10.24640148 | 8.490959977 | 9.962826707 | 10.64127876 | 9.144452959 | 7.929478335 | 8.458042978 | 8.342994133 | 7.63409979 | 6.7531505 |
| ENSMUSG00000020633 | Dcdc2c | 1380 | 0 | 0 | 0 | 0 | 0 | 0 | 0 | 0 | 0 | 0 | 0 | 0 | 0 | 0 | 0 | 0 | 0 | 0 |
| ENSMUSG00000020634 | Ubxn2a | 4234 | 45.1153191 | 47.52240933 | 43.60929744 | 47.83156402 | 50.58740206 | 54.21073735 | 45.5694626 | 45.53501354 | 41.76044217 | 45.85890317 | 43.4420383 | 40.98509565 | 43.44797343 | 40.33417792 | 42.18439886 | 44.81104385 | 50.20256085 | 41.6141164 |
| ENSMUSG00000020635 | Fkbp1b | 975 | 28.64194938 | 19.30439511 | 21.92933376 | 16.30851741 | 16.78446569 | 19.46533022 | 16.93551277 | 10.96807454 | 11.40549667 | 12.08992391 | 15.72898633 | 14.13996786 | 8.955640367 | 12.7185347 | 8.198053172 | 9.071928523 | 10.41689095 | 7.955163126 |
| ENSMUSG00000020636 | Allc | 1488 | 0 | 0 | 0.097527279 | 0 | 0 | 0 | 0 | 0 | 0 | 0 | 0 | 0 | 0 | 0 | 0 | 0 | 0 | 0 |
| ENSMUSG00000020638 | Cmpk2 | 3193 | 9.664502911 | 13.78212755 | 10.39280984 | 12.39979566 | 12.4965126 | 14.66562268 | 10.15007362 | 7.870529528 | 7.700704638 | 7.767388009 | 10.75796904 | 11.90431138 | 5.932808091 | 7.287343496 | 7.626094435 | 10.25320054 | 10.43080238 | 9.251453276 |
| ENSMUSG00000020639 | Pfn4 | 1302 | 1.43642684 | 1.560742356 | 0.668758484 | 1.119779892 | 0.776321349 | 1.10370912 | 2.471015489 | 1.701351259 | 2.562294759 | 1.448562312 | 0.293548132 | 1.286075175 | 1.629241508 | 1.070140262 | 1.392372088 | 0.764635785 | 1.677879358 | 1.182991542 |
| ENSMUSG00000020640 | Itsn2 | 6427 | 13.35933648 | 14.06221908 | 13.38984137 | 12.95870376 | 12.22207864 | 13.23051047 | 13.47163567 | 13.59642395 | 13.07304997 | 11.6501193 | 13.35795867 | 13.96477135 | 8.358870066 | 11.31654595 | 11.98800521 | 11.0099646 | 11.82527567 | 14.09677902 |
| ENSMUSG00000020641 | Rsad2 | 7136 | 8.613016331 | 11.24123448 | 8.459944771 | 8.75977478 | 11.56757975 | 12.18680597 | 8.685483379 | 11.60329714 | 9.638660499 | 8.79450477 | 10.8993319 | 12.10799407 | 10.66692101 | 13.01684064 | 10.54866068 | 21.40432076 | 21.78948802 | 19.2948142 |
| ENSMUSG00000020642 | Rnf144a | 5153 | 0.032994509 | 0.025859029 | 0 | 0.008841654 | 0.102746198 | 0.105779171 | 0.018363159 | 0.007411683 | 0 | 0.036600585 | 0.222510965 | 0.054158415 | 0 | 0.108156229 | 0.191895467 | 0.245214447 | 0.096689668 | 0 |
| ENSMUSG00000020644 | Id2 | 1267 | 13.68753893 | 18.3786212 | 14.31734324 | 16.28978144 | 15.00561156 | 15.76144564 | 20.05280867 | 22.3668213 | 15.99350099 | 24.5987489 | 31.44776272 | 21.54211445 | 317.523114 | 375.4016566 | 259.5340312 | 480.7027635 | 344.3316793 | 412.7636761 |
| ENSMUSG00000020646 | Mboat2 | 2369 | 33.58788076 | 35.01439705 | 35.4480577 | 35.32952235 | 35.57576967 | 39.8262578 | 33.67208808 | 39.77233294 | 37.57030838 | 33.39758551 | 35.73542694 | 37.62668347 | 26.67546899 | 33.0931277 | 30.12287666 | 22.80626722 | 31.79028004 | 33.2051594 |
| ENSMUSG00000020647 | Ncoa1 | 7305 | 9.466929692 | 12.00723484 | 11.6877866 | 11.31385824 | 11.72165533 | 10.60923687 | 7.875731359 | 11.64855203 | 8.068159351 | 10.46287337 | 10.84337867 | 11.53752916 | 6.29395577 | 7.152580193 | 8.950974565 | 6.919041995 | 9.690440846 | 6.988152093 |
| ENSMUSG00000020648 | Dus4l | 1761 | 1.737860689 | 1.740366989 | 1.84044664 | 2.276758604 | 1.284611251 | 2.363673833 | 2.74042774 | 1.800096195 | 2.806576883 | 1.713597393 | 2.007579175 | 1.965117025 | 3.669779195 | 1.160442842 | 2.199288049 | 3.196319868 | 2.306977554 | 1.936720396 |
| ENSMUSG00000020649 | Rrm2 | 2374 | 0.698273743 | 0.04209717 | 0.34639849 | 0.038383357 | 0.101373049 | 0.041746195 | 1.076194045 | 0.611335843 | 1.422617646 | 0.496532258 | 0 | 0.493735853 | 2.036448144 | 1.07600034 | 1.041319862 | 1.290332179 | 2.147176701 | 0.741487538 |
| ENSMUSG00000020650 | Bcap29 | 1947 | 4.519040286 | 4.089256147 | 4.944187232 | 4.329118234 | 4.771166547 | 4.835654535 | 4.179651143 | 3.393572479 | 7.001919218 | 4.552815723 | 4.613093063 | 5.160153706 | 9.349500501 | 8.444379525 | 7.829776928 | 8.358247635 | 5.196786021 | 8.052180491 |
| ENSMUSG00000020651 | Slc26a4 | 3054 | 0 | 0.109079549 | 0 | 0.059673928 | 0 | 0 | 0 | 0 | 0 | 0.138950992 | 0 | 0 | 0.226145004 | 0 | 0.391239398 | 0 | 0 | 0 |
| ENSMUSG00000020652 | Cenpo | 6983 | 1.515650699 | 2.165839034 | 1.932724469 | 2.23140157 | 1.950642188 | 1.738567912 | 2.418820905 | 1.662679412 | 2.512595692 | 2.032416108 | 1.888284196 | 1.694532503 | 2.7269254 | 1.942098456 | 2.324704714 | 2.763635419 | 2.085635518 | 1.536127139 |
| ENSMUSG00000020653 | Klf11 | 4222 | 14.23550898 | 16.54598275 | 15.33012402 | 14.50356314 | 16.55318644 | 14.7179365 | 13.81727454 | 15.37827667 | 14.24261322 | 13.39026367 | 19.50829664 | 15.26932503 | 13.64748446 | 15.75270799 | 16.1507757 | 13.90324518 | 17.92857328 | 16.0649523 |
| ENSMUSG00000020654 | Adcy3 | 6838 | 0.397825573 | 1.183830549 | 1.216766192 | 0.986112103 | 1.062872254 | 0.797133766 | 0.615798249 | 0.943918691 | 0.45173862 | 0.799865688 | 0.712641966 | 0.416291137 | 0.699794673 | 0.794670697 | 0.976111737 | 0.587966332 | 0.605329053 | 1.279094468 |
| ENSMUSG00000020656 | Grhl1 | 3483 | 1.171546626 | 1.578130217 | 1.416623628 | 2.642357438 | 1.575377347 | 1.024345914 | 0.489019937 | 1.129433663 | 0.212850211 | 1.286051338 | 0.123449505 | 0.448704724 | 3.44176167 | 4.520397825 | 3.572457617 | 5.112010463 | 6.118122618 | 4.153716643 |
| ENSMUSG00000020657 | Dnajc27 | 3302 | 5.084659155 | 7.37484121 | 4.365630541 | 6.540258939 | 6.005558011 | 6.017760801 | 7.479472562 | 5.551893748 | 7.147145225 | 5.826010578 | 7.176371716 | 4.597777172 | 3.735002822 | 5.204213305 | 5.502691141 | 4.626878363 | 6.337419173 | 5.730809422 |
| ENSMUSG00000020658 | Efr3b | 6524 | 0.749248198 | 0.786355872 | 0.734055718 | 0.865967121 | 0.855809802 | 0.843095252 | 1.261864827 | 1.457680595 | 0.909083119 | 1.033499474 | 0.930015286 | 1.154983821 | 1.81478543 | 2.18552036 | 1.856722476 | 0.792340895 | 1.051564553 | 1.635770494 |

| ENSMUSG00000020659 | Cbll1 | 4723 | 7.72166865 | 5.424013478 | 7.343599675 | 7.09992137 | 7.184629724 | 7.669499946 | 5.439505538 | 7.520418005 | 7.07225583 | 9.084721568 | 8.729576364 | 8.993376927 | 7.102626243 | 7.611205513 | 8.418272588 | 7.077641385 | 8.317690943 | 4.600592241 |
| --- | --- | --- | --- | --- | --- | --- | --- | --- | --- | --- | --- | --- | --- | --- | --- | --- | --- | --- | --- | --- |
| ENSMUSG00000020660 | Pomc | 1004 | 0.719709156 | 0.331801735 | 0.33726565 | 0.408415736 | 0.623222119 | 0.296131874 | 0.84823528 | 0.684724346 | 1.969076451 | 0.51659137 | 0.9516924 | 1.667798683 | 1.375790522 | 2.405470662 | 1.559421438 | 1.220417766 | 0.954340062 | 1.205378833 |
| ENSMUSG00000020661 | Dnmt3a | 12336 | 7.135880314 | 7.882647392 | 7.109371801 | 7.471627164 | 6.945105717 | 6.419039256 | 5.469186134 | 6.433512662 | 7.528825636 | 7.151342882 | 5.836321533 | 7.773290234 | 5.882559202 | 4.49908272 | 4.341927907 | 5.692685958 | 4.710008601 | 5.025556362 |
| ENSMUSG00000020664 | Dld | 2757 | 78.27304269 | 78.03216208 | 82.49994187 | 79.00883314 | 81.44193831 | 78.41805463 | 84.34596189 | 79.34932208 | 89.03577582 | 82.09045181 | 86.83362965 | 76.91102726 | 64.45178311 | 69.35448233 | 75.78276747 | 66.7759305 | 82.82483749 | 71.0908405 |
| ENSMUSG00000020668 | Kif3c | 6914 | 3.073848425 | 2.375362576 | 3.02946755 | 2.71494797 | 2.373877057 | 2.350780534 | 2.251355419 | 2.3476672 | 2.25769301 | 2.230008673 | 2.446099986 | 1.824463371 | 2.375980395 | 2.599632893 | 1.638765963 | 2.187560012 | 1.912434543 | 2.736929811 |
| ENSMUSG00000020669 | Sh3yl1 | 3080 | 57.54759222 | 51.37540505 | 50.71367841 | 55.01348178 | 54.94540377 | 56.53516422 | 41.47540036 | 31.55833251 | 30.36836925 | 31.30622992 | 31.53457487 | 31.55034694 | 25.21053175 | 22.33236799 | 21.48366843 | 28.78011563 | 24.73788255 | 29.9871072 |
| ENSMUSG00000020671 | Rab10 | 3505 | 84.0159371 | 82.44109685 | 86.18907192 | 85.70155929 | 87.84591033 | 87.27218955 | 74.82287569 | 80.78701039 | 78.43651718 | 86.27031669 | 81.29239725 | 88.09479185 | 70.5988294 | 65.9360282 | 76.25520487 | 74.21058376 | 82.972879 | 69.5578776 |
| ENSMUSG00000020672 | Sntg2 | 4041 | 0 | 0.082437254 | 0 | 0.067648173 | 0 | 0.012262493 | 0 | 0 | 0 | 0.035004234 | 0 | 0.193372748 | 0.146494186 | 0.022986433 | 0 | 0.066328694 | 0 | 0.027225492 |
| ENSMUSG00000020673 | Tpo | 3898 | 0.02180871 | 0 | 0 | 0 | 0 | 0 | 0 | 0 | 0 | 0 | 0 | 0 | 0 | 0 | 0 | 0 | 0 | 0 |
| ENSMUSG00000020674 | Pxdn | 9123 | 2.576494831 | 3.133011426 | 2.720116307 | 3.5408068 | 3.47153412 | 4.220374218 | 0.586027921 | 1.264288669 | 1.277626215 | 1.653866606 | 1.874759962 | 1.786492763 | 1.952079594 | 1.461082219 | 1.46777438 | 2.056603927 | 1.659419848 | 2.755580595 |
| ENSMUSG00000020676 | Ccl11 | 1079 | 0 | 0 | 0 | 0 | 0 | 0 | 0 | 0 | 0 | 0 | 0 | 0 | 0 | 0 | 0 | 0 | 0 | 0 |
| ENSMUSG00000020677 | Ddx52 | 3317 | 18.51672576 | 18.21814595 | 20.4315092 | 16.19429342 | 15.62800175 | 18.27042302 | 13.82152122 | 17.17909689 | 18.02918929 | 17.0862663 | 16.49150662 | 17.46658359 | 16.64227176 | 17.20825547 | 17.07933052 | 16.62299507 | 17.1006691 | 13.10135493 |
| ENSMUSG00000020679 | Hnf1b | 3284 | 22.93519242 | 19.63878295 | 23.40607178 | 22.98862682 | 19.49313596 | 23.11656146 | 21.63935559 | 20.20103623 | 18.71202239 | 21.06275325 | 20.83244231 | 25.08645493 | 18.46191423 | 21.17137601 | 17.2885944 | 25.42990705 | 23.06113358 | 27.21979246 |
| ENSMUSG00000020680 | Taf15 | 2847 | 2.07524393 | 2.152993515 | 2.480693864 | 1.69633674 | 1.420119982 | 1.183556685 | 2.22687003 | 2.079319414 | 2.386995118 | 1.722400119 | 2.248628183 | 1.588011475 | 2.807084861 | 2.169674613 | 1.548495427 | 2.582295968 | 1.103883508 | 1.565064153 |
| ENSMUSG00000020681 | Ace | 5262 | 6.268342189 | 8.274411392 | 8.301272883 | 7.636799906 | 6.823720079 | 6.836808168 | 2.508597001 | 3.832304916 | 2.168123312 | 2.616496646 | 2.169938251 | 2.418466566 | 1.96877663 | 1.774089997 | 2.842296615 | 1.098799262 | 2.104960054 | 1.90263349 |
| ENSMUSG00000020682 | Mmp28 | 4934 | 0.034459 | 0.033758507 | 0.078432964 | 0.046170495 | 0.019510305 | 0.050215579 | 0.124658457 | 0.178035115 | 0.07512741 | 0.23890709 | 0.213021704 | 0.090499656 | 1.209801782 | 0.80952402 | 0.459279606 | 0.34922524 | 0.574816776 | 0.401363567 |
| ENSMUSG00000020684 | Rasl10b | 4004 | 0 | 0 | 0 | 0 | 0 | 0 | 0 | 0 | 0 | 0 | 0 | 0 | 0 | 0 | 0 | 0 | 0.009572002 | 0 |
| ENSMUSG00000020686 | Gas2l2 | 2604 | 0 | 0.243066432 | 0.20434287 | 0 | 0 | 0 | 0 | 0 | 0 | 0 | 0 | 0.064303759 | 0 | 0.249699395 | 0 | 0 | 0.0735912 | 0 |
| ENSMUSG00000020687 | Cdc27 | 6972 | 10.89454239 | 10.5357045 | 9.873140232 | 11.0765878 | 11.53592146 | 10.70373161 | 8.577628538 | 7.964967455 | 8.175866009 | 7.736718951 | 8.61347143 | 7.637427153 | 6.9200538 | 6.874684192 | 8.025202653 | 7.859130693 | 8.663546173 | 7.94523386 |
| ENSMUSG00000020689 | Itgb3 | 5885 | 0.650036677 | 0.435869644 | 0.098637615 | 0.596125814 | 0.760623433 | 0.378907903 | 0.024118613 | 0 | 0.216955308 | 0 | 0 | 0.009484395 | 0 | 0.055243604 | 0 | 0 | 0.019537619 | 0 |
| ENSMUSG00000020690 | Efcab3 | 2867 | 0 | 0 | 0 | 0 | 0 | 0 | 0 | 0 | 0 | 0.016446007 | 0 | 0 | 0 | 0 | 0 | 0 | 0 | 0.249430899 |
| ENSMUSG00000020691 | Mettl2 | 2943 | 10.81766118 | 11.09297871 | 10.35518998 | 9.768609914 | 8.602579629 | 9.243782105 | 9.292126544 | 10.07043978 | 7.697106242 | 9.116123056 | 7.499840577 | 9.103471981 | 8.414754493 | 10.21043987 | 8.791905233 | 9.016442721 | 9.23321254 | 9.177530208 |
| ENSMUSG00000020692 | Nle1 | 3501 | 2.440314305 | 2.102870501 | 2.224546812 | 1.392468364 | 1.457293319 | 1.967389308 | 1.756826124 | 2.269071608 | 2.152851295 | 1.831618293 | 2.606403048 | 1.434849938 | 2.353164863 | 3.435880787 | 2.847979585 | 1.607746155 | 1.992398182 | 2.41970935 |
| ENSMUSG00000020694 | Tlk2 | 8043 | 4.703419951 | 4.133565639 | 4.799462542 | 3.591408941 | 4.542102457 | 4.189463985 | 3.888310428 | 4.739029914 | 5.9708414 | 4.818833529 | 4.062920751 | 4.941035902 | 5.072422677 | 4.532961407 | 4.543799587 | 5.06542413 | 5.136857896 | 4.288289185 |
| ENSMUSG00000020695 | Mrc2 | 6899 | 0.006161063 | 0 | 0 | 0 | 0 | 0 | 0 | 0 | 0 | 0 | 0 | 0 | 0 | 0 | 0 | 0 | 0.005555341 | 0 |
| ENSMUSG00000020696 | Rffl | 8277 | 4.780997811 | 5.175834477 | 6.072260244 | 4.777937234 | 6.233831361 | 4.651742659 | 3.841261356 | 5.112623156 | 3.418525592 | 4.243962055 | 4.900439742 | 5.313850685 | 3.742955666 | 4.791983887 | 5.062460111 | 5.04249874 | 5.440787587 | 4.479417413 |
| ENSMUSG00000020697 | Lig3 | 7271 | 4.647450824 | 6.652499299 | 5.255822993 | 5.564325041 | 5.117037133 | 6.310800618 | 6.149151642 | 5.835752864 | 5.307628286 | 5.978950406 | 4.980527923 | 4.851533318 | 4.803603003 | 5.423054627 | 5.07723753 | 4.628997924 | 6.683777262 | 6.597158721 |
| ENSMUSG00000020698 | Cct6b | 1842 | 1.292231193 | 0.488299752 | 0.131307086 | 0.667832897 | 0.391953776 | 0.269015926 | 0.924677764 | 1.181849586 | 1.25214163 | 1.04949991 | 0.518729191 | 0.303016626 | 0.723106279 | 0.680776525 | 1.677587781 | 1.20567602 | 1.227606687 | 0.447956895 |
| ENSMUSG00000020700 | Map3k3 | 3993 | 5.716323448 | 6.090261152 | 6.772051958 | 7.690494336 | 4.327413122 | 6.515197878 | 5.723021268 | 7.441444796 | 5.332689369 | 7.76988799 | 7.836863963 | 5.773070035 | 7.042121925 | 7.060245691 | 8.223801664 | 9.263392625 | 6.718859963 | 8.265831213 |
| ENSMUSG00000020701 | Tmem132e | 4534 | 0 | 0 | 0 | 0 | 0 | 0 | 0 | 0 | 0 | 0 | 0 | 0 | 0 | 0 | 0 | 0 | 0 | 0 |
| ENSMUSG00000020702 | Ccl1 | 2508 | 0 | 0 | 0 | 0 | 0 | 0 | 0 | 0 | 0 | 0 | 0 | 0 | 0 | 0 | 0 | 0 | 0 | 0 |
| ENSMUSG00000020703 | 5530401A14R | 5200 | 0 | 0 | 0 | 0 | 0 | 0 | 0 | 0 | 0 | 0 | 0 | 0 | 0 | 0 | 0 | 0 | 0 | 0 |
| ENSMUSG00000020704 | Accn1 | 4253 | 0.049970816 | 0.015665598 | 0.034121935 | 0.214253677 | 0 | 0.174768635 | 0.311487188 | 0.682488263 | 0.416416689 | 0.088691659 | 0.101099136 | 0.433086495 | 0.197188477 | 0.720740599 | 0.300317007 | 0.153054391 | 0.045057955 | 0.375091487 |
| ENSMUSG00000020705 | Ddx42 | 4210 | 23.50404983 | 26.05685526 | 24.83021356 | 25.57262417 | 24.99201547 | 24.58804286 | 20.61079647 | 22.39834716 | 21.10197494 | 23.09376487 | 23.44490837 | 23.99675755 | 21.68963869 | 25.80349653 | 24.37840278 | 21.61918617 | 27.42014401 | 22.9705486 |
| ENSMUSG00000020706 | Ftsj3 | 4206 | 10.48986513 | 9.789523836 | 11.2595545 | 10.12828732 | 9.521103303 | 8.682920743 | 7.975437318 | 8.980572035 | 9.576893109 | 10.73950873 | 9.893482842 | 7.789775069 | 10.09861645 | 8.469475755 | 8.904473206 | 9.112909655 | 9.075842063 | 8.697350445 |
| ENSMUSG00000020707 | Rnf135 | 1735 | 6.026670492 | 4.780928335 | 6.022295422 | 3.492575733 | 3.800618756 | 5.083796298 | 6.544693339 | 5.173034332 | 5.815963321 | 6.522287482 | 6.443423795 | 6.627104607 | 6.767144848 | 9.690351375 | 6.126801971 | 6.819479935 | 7.04673701 | 4.8826527 |
| ENSMUSG00000020708 | Psmc5 | 1384 | 163.0184063 | 153.1574754 | 152.3906014 | 139.3496392 | 150.8991451 | 151.0212646 | 136.7076612 | 125.0911561 | 155.9076272 | 125.4740174 | 132.1750838 | 116.1885288 | 140.0826842 | 142.2853544 | 141.9131625 | 129.9501322 | 119.2712144 | 130.8851073 |
| ENSMUSG00000020709 | Adap2 | 2784 | 0.213747293 | 0.095726708 | 0.01737555 | 0 | 0.069155063 | 0 | 0.016994497 | 0.013718535 | 0.10355805 | 0.050808947 | 0 | 0.060146188 | 0 | 0 | 0 | 0.02750762 | 0 | 0 |
| ENSMUSG00000020712 | Tcam1 | 3789 | 0 | 0 | 0 | 0 | 0 | 0 | 0 | 0.131037538 | 0 | 0 | 0 | 0 | 0 | 0 | 0 | 0.020211458 | 0.010115148 | 0 |
| ENSMUSG00000020713 | Gh | 857 | 0 | 0 | 0 | 0 | 0 | 0.346926956 | 0 | 0 | 0 | 0.110036647 | 0.390227199 | 0 | 0 | 0 | 0 | 0 | 0 | 0 |
| ENSMUSG00000020715 | Ern1 | 9454 | 3.713695303 | 3.513111438 | 3.719860014 | 3.52767976 | 3.828560038 | 3.700468575 | 3.853476071 | 3.575235469 | 3.498283495 | 3.895144832 | 4.290346916 | 3.819836439 | 4.14317139 | 4.598229934 | 4.432196144 | 4.353966888 | 3.8310081 | 4.067206008 |

| ENSMUSG00000020716 | Nf1 |  | 15083 | 1.014510599 | 1.223585719 | 1.529813298 | 0.948496183 | 1.244543541 | 1.212289246 | 0.912815061 | 0.967280894 | 1.578320368 | 0.931572603 | 1.080107461 | 1.517232756 | 1.399858158 | 1.317912179 | 0.81403326 | 1.548582544 | 1.331497685 | 0.754948292 |
| --- | --- | --- | --- | --- | --- | --- | --- | --- | --- | --- | --- | --- | --- | --- | --- | --- | --- | --- | --- | --- | --- |
| ENSMUSG00000020717 | Pecam1 |  | 5241 | 0.235193685 | 0.057205886 | 0.175366739 | 0.199943526 | 0.202042038 | 0.11345789 | 0.713165738 | 0.386223493 | 0.086443746 | 0.089965089 | 0.9662556 | 0.095848304 | 0.865967638 | 0.478530952 | 0.18867341 | 1.168955769 | 0.22669628 | 0 |
| ENSMUSG00000020718 | Polg2 |  | 4312 | 2.247490755 | 1.869601206 | 1.581787519 | 2.17661761 | 1.573887114 | 2.034052376 | 2.205437956 | 1.567730804 | 1.56646303 | 2.197887605 | 1.684089445 | 2.174636205 | 2.299562934 | 1.927989713 | 2.044789648 | 2.468643056 | 2.115412491 | 2.946916432 |
| ENSMUSG00000020719 | Ddx5 |  | 5758 | 184.4892948 | 174.669953 | 177.0447688 | 172.6461317 | 178.9106569 | 157.7804476 | 165.3067862 | 179.7987933 | 205.5892386 | 165.2076134 | 183.3171123 | 187.202514 | 171.8734964 | 167.4826382 | 172.9349084 | 190.1163902 | 175.7698024 | 179.5391008 |
| ENSMUSG00000020720 | Psmd12 |  | 2793 | 61.57391415 | 58.10971022 | 52.85929633 | 51.38463647 | 60.71205409 | 56.27686033 | 53.78365757 | 53.95890436 | 55.13654939 | 48.2311347 | 47.74074798 | 48.40155202 | 53.39433796 | 54.22634049 | 52.36860845 | 54.38554943 | 50.78611702 | 49.5731907 |
| ENSMUSG00000020721 | Helz |  | 14191 | 3.02217762 | 3.267673085 | 3.044011177 | 3.367876507 | 3.27301151 | 3.442956473 | 2.790551213 | 2.279540895 | 2.658505281 | 2.957094348 | 3.40023311 | 3.02067711 | 3.723097978 | 2.333495477 | 2.520114898 | 3.00583022 | 3.014033355 | 2.49248507 |
| ENSMUSG00000020722 | Cacng1 |  | 1245 | 0.170703518 | 0.321088137 | 0.233125447 | 0.292761731 | 0.193300898 | 0.517418102 | 0.722040883 | 0.214736399 | 0.066163078 | 0.227232305 | 0 | 0 | 0 | 0.596871806 | 0.430217049 | 0.30755508 | 0.123136697 | 0 |
| ENSMUSG00000020723 | Cacng4 |  | 3692 | 1.036150012 | 0.983506357 | 1.349532402 | 1.406814478 | 0.560583076 | 0.805299029 | 1.102082987 | 0.455164059 | 0.557780549 | 0.804575922 | 1.138731405 | 1.118732132 | 0.200427616 | 0.377389659 | 0.636102028 | 0.093341134 | 0.259522595 | 0.581082113 |
| ENSMUSG00000020728 | Ccdc46 |  | 4970 | 1.496661128 | 1.179692029 | 1.888222312 | 1.576760488 | 1.433304769 | 2.014014524 | 2.227598968 | 2.013362058 | 1.947450947 | 2.390739883 | 1.345773478 | 1.74072992 | 1.379705533 | 1.205490397 | 1.633140143 | 0.770434757 | 2.298035508 | 0.796912612 |
| ENSMUSG00000020732 | Rab37 |  | 5319 | 0.015982394 | 0.162837986 | 0.181889567 | 0.034262865 | 0 | 0.102477922 | 0.09784536 | 0.035901864 | 0 | 0.053187483 | 0.116765268 | 0.115430022 | 0.00927466 | 0 | 0.046476656 | 0.014397672 | 0.021616637 | 0.217182034 |
| ENSMUSG00000020733 | Slc9a3r1 |  | 1919 | 250.7340243 | 261.0175495 | 253.5388058 | 267.7393939 | 251.8460894 | 265.4002062 | 320.5378524 | 298.0161718 | 292.3190957 | 320.669009 | 320.6324597 | 299.4092916 | 422.1362239 | 466.6432772 | 420.6895707 | 427.3822407 | 393.9479971 | 408.2254762 |
| ENSMUSG00000020734 | Grin2c |  | 5021 | 0 | 0 | 0 | 0 | 0 | 0 | 0 | 0 | 0 | 0 | 0.009515029 | 0 | 0 | 0 | 0 | 0.030504368 | 0 | 0 |
| ENSMUSG00000020736 | Nt5c |  | 1430 | 47.17182822 | 42.11868024 | 44.6863172 | 39.12514856 | 39.48164088 | 45.1518964 | 46.32010524 | 43.64082904 | 39.45841021 | 41.2156497 | 40.42496487 | 48.08734007 | 43.08780747 | 43.81333838 | 38.69488781 | 37.406978 | 32.00111783 | 36.42910774 |
| ENSMUSG00000020737 | Hn1 |  | 2410 | 202.1729597 | 201.7434403 | 182.053079 | 172.1300036 | 180.3049821 | 191.3845826 | 111.0375569 | 104.8944031 | 108.3324993 | 107.9575023 | 109.7832863 | 120.5710952 | 98.99134936 | 85.95046878 | 79.36000931 | 114.2521817 | 95.59309987 | 94.93065347 |
| ENSMUSG00000020738 | Sumo2 |  | 1730 | 2.530654989 | 2.118160904 | 2.488580463 | 1.790838737 | 2.810014041 | 2.491958278 | 2.680140194 | 2.472571716 | 2.285494516 | 2.098615116 | 2.07116872 | 2.129383659 | 1.653902429 | 1.906086823 | 2.667383965 | 1.903463721 | 2.547701817 | 2.448382207 |
| ENSMUSG00000020739 | Nup85 |  | 3156 | 14.7475183 | 15.17868881 | 16.89088417 | 14.91272459 | 15.17467175 | 14.91606366 | 14.55659418 | 13.81993775 | 12.84142317 | 14.67109968 | 13.06393827 | 12.78666795 | 14.19308647 | 15.42249797 | 14.46493154 | 13.40656565 | 12.69042467 | 16.41906798 |
| ENSMUSG00000020740 | Gga3 |  | 5654 | 7.179420429 | 8.036573703 | 8.162070739 | 8.485281161 | 8.070227032 | 6.941238945 | 7.397313083 | 6.8157294 | 7.444750461 | 6.996717374 | 7.14849927 | 7.542123491 | 6.334448522 | 7.696871221 | 8.700859168 | 6.91452249 | 6.412571077 | 8.911981209 |
| ENSMUSG00000020741 | 1300001I01Ri | k | 6656 | 5.913430439 | 4.654596097 | 6.075761926 | 8.309962113 | 6.334670242 | 5.486833631 | 8.174516341 | 5.967562876 | 6.070308288 | 8.323629256 | 8.326164639 | 7.488489587 | 6.10719649 | 6.147422024 | 7.019616043 | 8.076924999 | 8.412668235 | 5.925710565 |
| ENSMUSG00000020743 | Mif4gd |  | 2228 | 47.06475281 | 43.240974 | 48.02614414 | 46.91069825 | 45.49633359 | 40.54516756 | 44.29723889 | 42.64932549 | 47.08350792 | 41.83884216 | 45.95230522 | 43.91600382 | 41.60443113 | 44.35952332 | 40.81321088 | 39.66549456 | 36.64049031 | 40.76303196 |
| ENSMUSG00000020744 | Slc25a19 |  | 3916 | 6.273746615 | 6.558795057 | 6.398745844 | 6.794599053 | 5.592447719 | 6.213072571 | 8.880188716 | 6.622227077 | 7.28862498 | 8.211639331 | 5.758370889 | 7.939051088 | 8.188392806 | 6.74838757 | 6.84939042 | 6.92281666 | 5.891836245 | 8.456456141 |
| ENSMUSG00000020745 | Pafah1b1 |  | 13402 | 5.172805708 | 5.396380641 | 6.374246725 | 5.361122737 | 5.40864625 | 5.209655396 | 5.383661794 | 5.685259125 | 6.324567187 | 5.10136694 | 5.65371468 | 6.022194307 | 6.029374757 | 5.024916185 | 4.645243318 | 5.125604363 | 5.519307045 | 5.253817087 |
| ENSMUSG00000020747 | 2310067B10R |  | 5888 | 9.406291502 | 10.37067512 | 10.16271349 | 11.48311647 | 10.50433456 | 10.36837088 | 8.975588037 | 10.93620762 | 8.911620428 | 11.87576307 | 9.752972162 | 11.61246377 | 9.635140093 | 9.875678908 | 10.40535218 | 7.907842833 | 10.49286525 | 7.941192377 |
| ENSMUSG00000020752 | Recql5 |  | 6800 | 5.275642436 | 6.314753037 | 6.068032558 | 6.633152043 | 4.678706109 | 5.428939193 | 3.284056537 | 5.785025658 | 5.015063978 | 5.242048774 | 5.10770512 | 5.54052533 | 4.389089699 | 4.903949227 | 5.713686354 | 5.028457707 | 4.926056396 | 7.442408557 |
| ENSMUSG00000020755 | Sap30bp |  | 3904 | 16.52737121 | 18.56784268 | 19.72609127 | 17.41216141 | 19.91113133 | 18.55688947 | 19.41468024 | 19.85926662 | 21.50054281 | 17.95929707 | 18.06245869 | 18.57186109 | 18.66374022 | 21.35428711 | 18.78554546 | 22.4408068 | 20.95969362 | 21.06521889 |
| ENSMUSG00000020758 | Itgb4 |  | 7058 | 7.62419281 | 8.70817368 | 8.519169486 | 8.59837221 | 8.490258567 | 9.127026587 | 5.302398559 | 5.573558299 | 6.068854689 | 5.865445942 | 6.971976089 | 7.568091399 | 6.912619182 | 6.600087794 | 7.256097191 | 6.027325705 | 6.15240782 | 5.292035062 |
| ENSMUSG00000020766 | Galk1 |  | 1398 | 45.97126334 | 55.54531933 | 59.65376705 | 51.23173226 | 60.49198132 | 57.45706805 | 51.50922556 | 50.15826239 | 52.94146554 | 51.9737008 | 56.967708 | 56.97349822 | 49.75536721 | 52.32434738 | 54.61121822 | 50.45157284 | 48.99076721 | 52.56950399 |
| ENSMUSG00000020770 | Unk |  | 4480 | 9.639566706 | 12.22464243 | 12.37412183 | 13.73950243 | 13.6230534 | 13.09607959 | 9.631509639 | 10.83538916 | 10.79307355 | 11.8718735 | 12.97815278 | 12.12246422 | 6.595941618 | 11.17560853 | 8.957659696 | 9.735045051 | 11.60054879 | 10.67029325 |
| ENSMUSG00000020773 | Trim47 |  | 3048 | 29.88470874 | 36.38406327 | 27.29739902 | 30.4188731 | 31.31417475 | 28.93827617 | 20.0395894 | 19.71018667 | 21.93100889 | 17.23290139 | 21.00342663 | 20.38150671 | 15.99079208 | 13.63761752 | 15.32892532 | 24.12006765 | 18.37097105 | 15.3043709 |
| ENSMUSG00000020774 | Aspa |  | 5612 | 0 | 0 | 0 | 0 | 0 | 0 | 0 | 0 | 0 | 0 | 0 | 0 | 0 | 0.099310237 | 0 | 0 | 0 | 0 |
| ENSMUSG00000020775 | Mrpl38 |  | 2852 | 32.95194397 | 31.11695309 | 29.97055685 | 28.61144124 | 29.68585334 | 27.57369852 | 25.82953755 | 25.5776608 | 30.87544555 | 28.75002558 | 27.0032374 | 23.68055811 | 29.12866364 | 28.04232766 | 26.2349318 | 25.30778224 | 22.37492438 | 24.39920056 |
| ENSMUSG00000020776 | Fbf1 |  | 8490 | 2.363067501 | 3.943399139 | 4.518281457 | 3.697474623 | 3.350526135 | 3.630365169 | 3.711453965 | 3.945198694 | 4.210823991 | 4.409618185 | 4.124740231 | 4.016886907 | 3.771073535 | 3.823841824 | 3.950302296 | 2.936064242 | 3.029086599 | 4.127302825 |
| ENSMUSG00000020777 | Acox1 |  | 5067 | 22.43957882 | 26.9487968 | 27.93387603 | 26.71439959 | 22.63634776 | 25.42670363 | 23.36221091 | 28.92130413 | 24.92975012 | 28.31647721 | 26.73021656 | 23.14361695 | 29.42195658 | 25.55478896 | 27.06926772 | 26.2752007 | 32.9029784 | 26.38092152 |
| ENSMUSG00000020778 | 2310004N24R |  | 1727 | 50.5287356 | 44.69367453 | 42.43537839 | 43.66156825 | 44.39731002 | 41.57609204 | 45.01142529 | 38.01548334 | 39.44556866 | 38.38673351 | 38.12037799 | 40.49625085 | 39.59122023 | 39.12921086 | 32.56521354 | 33.39065127 | 32.44530751 | 33.79540373 |
| ENSMUSG00000020780 | Srp68 |  | 2720 | 31.36319422 | 33.65582107 | 34.39500283 | 34.57279247 | 30.7902747 | 36.18078268 | 35.71063597 | 29.41657465 | 26.45325112 | 29.74654661 | 28.5244605 | 29.07749775 | 28.74672385 | 27.83230237 | 27.62927866 | 29.224743 | 29.33652579 | 22.93394376 |
| ENSMUSG00000020781 | Tsen54 |  | 2426 | 11.56365053 | 12.79786373 | 11.6248014 | 15.2120552 | 12.97538256 | 14.15500592 | 19.48283852 | 18.70262739 | 15.00778233 | 17.18104768 | 18.25531183 | 17.89966426 | 15.39334764 | 15.41116667 | 14.31694626 | 15.78343256 | 13.61799997 | 11.94962871 |
| ENSMUSG00000020782 | Llgl2 |  | 4576 | 35.35286275 | 44.88068026 | 37.31611935 | 48.73717483 | 38.3498675 | 48.90300368 | 36.40470772 | 37.68328166 | 31.00689397 | 48.61385882 | 35.60153156 | 36.76320084 | 28.88116398 | 29.00725563 | 38.3923589 | 30.53374703 | 32.83196763 | 33.26271816 |
| ENSMUSG00000020783 | 1200014J11R |  | 8516 | 3.558735381 | 3.215500123 | 3.544514202 | 3.279581984 | 2.961616722 | 2.816289696 | 3.90568498 | 3.170740782 | 3.496694563 | 3.155930115 | 2.8162317 | 3.32953929 | 3.307717798 | 2.786863393 | 2.027178478 | 3.201375349 | 3.361876912 | 3.178074273 |
| ENSMUSG00000020785 | Camkk1 |  | 3797 | 0.078360872 | 0.07018782 | 0 | 0.119992216 | 0.240849763 | 0.104403969 | 0 | 0.281640049 | 0 | 0.062089417 | 0 | 0 | 0.623632349 | 0.648284601 | 0.292979196 | 0.010084437 | 0 | 0.260775328 |
| ENSMUSG00000020787 | P2rx1 |  | 2749 | 0.01546205 | 0 | 0 | 0.049721038 | 0 | 0 | 0 | 0.013893198 | 0.014982363 | 0 | 0.417096764 | 0 | 0.807543208 | 2.821448743 | 0.80934304 | 0.041786767 | 0.404315246 | 1.080571758 |
| ENSMUSG00000020788 | Atp2a3 |  | 5440 | 34.62921694 | 37.10346068 | 43.6873446 | 47.16908119 | 45.06174398 | 51.78442838 | 27.70922703 | 39.87034067 | 28.96683995 | 42.18427808 | 31.74751377 | 34.32047635 | 10.23818651 | 5.694523215 | 12.99666726 | 7.151334029 | 13.22398149 | 10.54678278 |
| ENSMUSG00000020790 | Ankfy1 |  | 11307 | 5.229035098 | 5.756911232 | 5.304959551 | 6.334302794 | 5.329545276 | 7.607990227 | 5.335072577 | 6.269134064 | 6.480128375 | 6.968145852 | 5.661841723 | 5.701520323 | 5.109018761 | 4.505984246 | 5.746427314 | 5.861947591 | 6.341956443 | 4.188807012 |

| ENSMUSG00000020792 | Exoc7 | 4568 | 24.06269378 | 28.24449197 | 27.33189182 | 32.01640813 | 29.87171707 | 28.49716093 | 22.06129731 | 22.96727877 | 21.67521539 | 24.3907863 | 27.24469502 | 25.23190526 | 21.98769343 | 25.60118477 | 30.26979456 | 23.05986448 | 24.72583121 | 25.50553591 |
| --- | --- | --- | --- | --- | --- | --- | --- | --- | --- | --- | --- | --- | --- | --- | --- | --- | --- | --- | --- | --- |
| ENSMUSG00000020793 | Galr2 | 2818 | 0 | 0 | 0 | 0 | 0 | 0 | 0 | 0 | 0.102308591 | 0 | 0 | 0.079227342 | 0.10503602 | 0 | 0 | 0.013587866 | 0.068002656 | 0 |
| ENSMUSG00000020794 | Ube2g1 | 4888 | 23.93959689 | 27.26777614 | 26.96764939 | 25.93101991 | 24.78478966 | 27.44256338 | 28.77671736 | 24.37812919 | 22.92727279 | 24.9547605 | 24.86487201 | 24.28803484 | 19.79130315 | 18.91779419 | 20.18775089 | 21.65988737 | 24.39302571 | 24.71358395 |
| ENSMUSG00000020798 | Spns3 | 5814 | 0.007310832 | 0.022919088 | 0 | 0 | 0 | 0 | 0.122065735 | 0.052552325 | 0 | 0 | 0.016434454 | 0.086401954 | 0.212125547 | 0.902680061 | 0.46062971 | 0.138304567 | 0.283459024 | 0.624458384 |
| ENSMUSG00000020799 | Tekt1 | 1857 | 0 | 0.771380965 | 0.026049289 | 0.392556117 | 0 | 0.186790057 | 0 | 0 | 0 | 0 | 0 | 0 | 0 | 0 | 0 | 0 | 0 | 0 |
| ENSMUSG00000020801 | Med31 | 1726 | 14.5542057 | 12.2173013 | 16.67569557 | 12.64411369 | 14.97499595 | 12.68963398 | 16.22775546 | 15.6664084 | 18.66040285 | 15.95365626 | 17.32741831 | 18.72379411 | 17.17756795 | 19.40103535 | 15.96978035 | 19.83302608 | 16.67615815 | 18.61258304 |
| ENSMUSG00000020802 | Ube2o | 5270 | 3.331050797 | 5.252944041 | 4.369222097 | 5.610838287 | 2.712558127 | 4.908259379 | 4.928778127 | 3.775757429 | 5.548847475 | 5.824498638 | 4.596186708 | 5.740434368 | 6.346686891 | 3.401787046 | 6.254505638 | 6.103246721 | 4.552611359 | 5.6261686 |
| ENSMUSG00000020803 | Txndc17 | 1473 | 95.48515104 | 84.44694298 | 81.80479573 | 77.63626699 | 86.03622201 | 85.64919191 | 102.141425 | 87.81919005 | 100.7154309 | 95.00562612 | 82.80344127 | 84.31082774 | 105.3284995 | 97.55465471 | 98.48654331 | 98.28702416 | 91.37946689 | 99.59897327 |
| ENSMUSG00000020804 | Aanat | 1656 | 0 | 0.020116482 | 0 | 0.082538124 | 0 | 0.029923148 | 0 | 0 | 0.024871084 | 0 | 0 | 0.03370511 | 0.029789805 | 0 | 0 | 0 | 0.046287798 | 0.03321806 |
| ENSMUSG00000020805 | Slc13a5 | 3531 | 0 | 0 | 0 | 0 | 0 | 0 | 0 | 0 | 0 | 0 | 0 | 0 | 0 | 0 | 0 | 0 | 0 | 0 |
| ENSMUSG00000020806 | Rhbdf2 | 6543 | 7.256347489 | 8.553517085 | 7.585395406 | 9.163737482 | 8.084515377 | 9.754534747 | 6.370544114 | 7.267238422 | 6.307334372 | 7.804403428 | 7.820110122 | 7.268064271 | 4.644423207 | 5.373402744 | 4.162345781 | 7.402967807 | 8.019058606 | 5.355464005 |
| ENSMUSG00000020807 | 4933427D14R | 5712 | 2.321711296 | 1.702970083 | 2.227282648 | 1.746825754 | 1.828541218 | 1.492134134 | 2.882495145 | 2.199807491 | 2.011736327 | 3.112012452 | 2.057534976 | 2.589487147 | 1.692761867 | 2.105920629 | 1.803289386 | 1.722809192 | 1.986096618 | 2.186111209 |
| ENSMUSG00000020808 | Fam64a | 2522 | 0 | 0.013208919 | 0 | 0 | 0.076339292 | 0 | 0 | 0 | 0.195970733 | 0 | 0 | 0.022131508 | 0.1760457 | 1.38116834 | 0.032673716 | 0.258104808 | 0.471100398 | 0.174493598 |
| ENSMUSG00000020810 | Cygb | 2327 | 0.146128667 | 0.200421366 | 0.64442606 | 0.13705514 | 0.06205233 | 0.234241542 | 1.016602469 | 0.426730753 | 1.115062524 | 1.256271423 | 0.349022043 | 0.695596998 | 0.487595229 | 0.199587827 | 0.548881922 | 0.082274618 | 0.592929389 | 0.354592437 |
| ENSMUSG00000020811 | Wscd1 | 4274 | 0 | 0 | 0 | 0 | 0 | 0 | 0 | 0 | 0 | 0 | 0 | 0 | 0 | 0.097799903 | 0 | 0 | 0 | 0 |
| ENSMUSG00000020812 | 1810032O08R | 2858 | 1.383128541 | 1.177257633 | 1.540234871 | 1.131852396 | 1.296766313 | 1.179001359 | 1.556120301 | 1.897593122 | 1.959890183 | 1.781762055 | 2.340270884 | 1.87484381 | 2.036797144 | 1.820062207 | 2.277762712 | 2.264210165 | 1.837194778 | 3.021843861 |
| ENSMUSG00000020814 | Mxra7 | 2385 | 28.28331839 | 31.51106472 | 27.62463243 | 28.29178476 | 28.1324535 | 33.28447765 | 12.49768877 | 14.36418656 | 17.16536548 | 13.16661148 | 12.67989464 | 16.61598341 | 9.638856793 | 5.569395804 | 10.36517129 | 8.830119107 | 10.30069447 | 9.295039836 |
| ENSMUSG00000020817 | Rabep1 | 6059 | 17.69938259 | 14.28402363 | 13.70011191 | 14.2871735 | 13.40131296 | 15.67793205 | 14.57096202 | 14.15747414 | 16.84439446 | 16.24866618 | 14.18503884 | 16.13025667 | 17.15503378 | 14.94734616 | 13.04251266 | 17.34737041 | 17.09154221 | 16.37835097 |
| ENSMUSG00000020818 | Mfsd11 | 6877 | 8.263693522 | 8.370609453 | 10.35420048 | 8.8511786 | 9.24564071 | 8.761978193 | 10.34728357 | 8.580378321 | 9.983702454 | 8.968026736 | 7.739029191 | 9.804467123 | 9.763085562 | 10.35317522 | 9.178534772 | 9.331839175 | 7.763346166 | 8.790898399 |
| ENSMUSG00000020821 | Kif1c | 7178 | 2.356792987 | 1.809978928 | 1.920654242 | 1.751859604 | 1.924472289 | 2.05721853 | 1.608288334 | 1.431283958 | 1.187741538 | 1.16267407 | 1.277903598 | 1.150838404 | 1.55322002 | 1.462296427 | 1.888452478 | 1.44563313 | 0.923718217 | 1.103554105 |
| ENSMUSG00000020823 | Sec14l1 | 6140 | 11.22164338 | 12.72834688 | 12.15640999 | 13.05984333 | 12.30734856 | 13.06610353 | 9.539592266 | 9.504558774 | 9.692917797 | 10.3670113 | 11.15008396 | 9.045046299 | 7.480132734 | 9.409844416 | 10.19302335 | 9.765975764 | 11.64145663 | 10.19549893 |
| ENSMUSG00000020826 | Nos2 | 4165 | 0 | 0.151967585 | 0.127757223 | 0 | 0 | 0.07138449 | 0 | 0 | 0.168108228 | 0 | 0.389999661 | 0.25462127 | 0 | 0 | 0 | 0 | 0 | 0 |
| ENSMUSG00000020827 | Mink1 | 6396 | 4.20001114 | 4.265675479 | 5.997531203 | 4.680050989 | 4.214177182 | 5.384482464 | 3.543272859 | 3.582776961 | 4.043954331 | 3.840762414 | 3.82438692 | 4.057892918 | 4.519778537 | 3.717850648 | 4.01322222 | 3.753628962 | 3.894948872 | 3.509023693 |
| ENSMUSG00000020828 | Pld2 | 6142 | 5.806226423 | 7.956694207 | 6.221929173 | 7.707249278 | 6.175180047 | 6.889943744 | 5.315165819 | 4.358983081 | 6.155848497 | 5.780605593 | 5.41376931 | 4.34384349 | 4.281001613 | 4.786569085 | 5.064665369 | 5.043487689 | 4.424185038 | 5.096089503 |
| ENSMUSG00000020829 | Slc46a1 | 2046 | 8.683853167 | 8.743413585 | 10.19012296 | 10.5329296 | 9.574629977 | 10.51118591 | 7.261085622 | 11.08811683 | 8.434579719 | 8.204130186 | 7.822390562 | 10.4483865 | 10.07856374 | 21.36064821 | 9.887567909 | 7.055502928 | 9.21629427 | 11.18464731 |
| ENSMUSG00000020830 | Vmo1 | 669 | 0 | 0 | 0 | 0 | 0 | 0 | 0 | 0 | 0.123128597 | 0 | 0 | 0 | 0 | 0 | 0 | 0 | 0 | 0 |
| ENSMUSG00000020831 | 0610010K14R | 1919 | 1.018883844 | 0.920053879 | 0.983099366 | 0.807230593 | 0.953107399 | 0.955420085 | 1.750495156 | 1.572277118 | 2.275023799 | 1.154811388 | 1.966764836 | 2.210521291 | 2.030860586 | 2.323414481 | 2.640850117 | 2.613897639 | 1.637705235 | 1.89192342 |
| ENSMUSG00000020832 | Eral1 | 3056 | 13.95048807 | 13.96394552 | 16.17727357 | 13.11967245 | 12.91497952 | 12.55033239 | 11.17793003 | 13.64728515 | 14.08373985 | 12.43568941 | 10.45858875 | 10.90377963 | 13.23696733 | 14.54417265 | 10.77226543 | 14.75992654 | 14.73605984 | 10.98022091 |
| ENSMUSG00000020834 | Dhrs13 | 2024 | 8.022221958 | 7.077344128 | 7.48068923 | 5.447516171 | 7.253081383 | 6.340987153 | 5.025803345 | 5.679798974 | 6.979730685 | 5.614288283 | 6.137099409 | 6.921804007 | 5.142803629 | 7.113471883 | 6.147662984 | 4.767407641 | 4.847594865 | 6.522818979 |
| ENSMUSG00000020836 | Coro6 | 3107 | 0.615620509 | 0.450319137 | 0.544922292 | 0.454583963 | 0.542200621 | 0.446564706 | 0.289327615 | 0.897343217 | 1.524444581 | 1.912130225 | 2.198847461 | 0.844330911 | 3.778885199 | 5.067443072 | 2.294132336 | 4.732408511 | 2.849492946 | 4.001306121 |
| ENSMUSG00000020838 | Slc6a4 | 3815 | 0 | 0.061124577 | 0 | 0 | 0 | 0 | 0 | 0.010011115 | 0 | 0.049437172 | 0 | 0.01463058 | 0 | 0 | 0 | 0.230847699 | 0.040184846 | 0.100934141 |
| ENSMUSG00000020839 | Tmigd1 | 1490 | 0.256742674 | 0.022357647 | 0 | 0.122311528 | 0 | 0.232798077 | 0.254027806 | 0.205059879 | 0.331703483 | 0.031644767 | 0 | 0.187300881 | 0.198652016 | 0 | 0.276520509 | 0 | 0.257223469 | 0.664539544 |
| ENSMUSG00000020840 | Blmh | 5078 | 11.71863853 | 11.04744267 | 10.99311816 | 12.72263901 | 11.0424756 | 9.875416775 | 12.27073615 | 11.43966996 | 9.927588675 | 11.41162156 | 12.81400029 | 10.43108779 | 10.297722 | 8.78029222 | 8.61678857 | 10.99403418 | 11.52506014 | 10.90865901 |
| ENSMUSG00000020841 | Cpd | 8311 | 10.52017171 | 12.63012028 | 13.6314292 | 12.41676879 | 13.37223099 | 12.66394009 | 11.48233346 | 13.16583238 | 13.62311778 | 11.46004339 | 12.86492084 | 11.30282278 | 11.78243963 | 11.4112413 | 10.5445445 | 8.859684518 | 12.17439825 | 9.206794303 |
| ENSMUSG00000020843 | Timm22 | 3179 | 18.03695579 | 17.98204671 | 14.88182217 | 16.12336423 | 16.44267666 | 17.72301292 | 19.92817776 | 17.20400134 | 14.70484284 | 19.19251652 | 15.82479751 | 19.06757143 | 21.04249129 | 19.6792028 | 20.08883663 | 20.99418959 | 19.26562518 | 17.8403237 |
| ENSMUSG00000020844 | Nxn | 3230 | 8.106250289 | 7.786760104 | 6.664464708 | 5.684551352 | 7.435860653 | 7.256483896 | 4.086760811 | 5.628354039 | 6.719905203 | 5.153002554 | 6.981356162 | 5.40876235 | 8.277987357 | 5.291462588 | 4.987556766 | 8.523512917 | 4.580170466 | 6.045892535 |
| ENSMUSG00000020846 | Fam101b | 3509 | 2.628561755 | 2.620221945 | 2.426258576 | 2.882459915 | 2.798208159 | 3.332700235 | 2.130351459 | 1.741460355 | 2.265317054 | 1.881190783 | 2.246471402 | 1.336140113 | 1.363692213 | 1.376513277 | 1.925635556 | 0.556517805 | 0.731793073 | 0.752475669 |
| ENSMUSG00000020847 | Rph3al | 3878 | 2.33460611 | 1.417387196 | 1.359647965 | 2.431958787 | 1.985845226 | 1.903908536 | 2.537657869 | 2.245453262 | 2.644518418 | 2.054788254 | 3.067551486 | 3.036901703 | 1.679167891 | 1.149724701 | 1.944271462 | 1.668672679 | 1.64057898 | 1.390121831 |
| ENSMUSG00000020848 | Doc2b | 4352 | 0.175802658 | 0.244947752 | 0.33345724 | 0.282662729 | 0.188015327 | 0.11386198 | 0.108714795 | 0.184292383 | 0.416407788 | 0.184182435 | 0.263464845 | 0.102602321 | 0.181367343 | 0.181422216 | 0.170410847 | 0.070387146 | 0.290617601 | 0.214879323 |
| ENSMUSG00000020849 | Ywhae | 2739 | 290.7701655 | 293.8929448 | 309.4563705 | 291.5807032 | 302.1995952 | 295.9233527 | 288.2117736 | 272.2411335 | 287.8544984 | 287.6382259 | 267.3409232 | 273.3928183 | 241.0221676 | 236.306974 | 253.9183145 | 259.1850534 | 263.4005889 | 251.4472494 |
| ENSMUSG00000020850 | Prpf8 | 7833 | 16.4203578 | 15.45500544 | 14.56825713 | 14.82057206 | 15.4970907 | 13.93018248 | 13.29442185 | 14.70063746 | 14.93819626 | 11.0156756 | 12.73510958 | 14.24428821 | 13.13124569 | 10.90395464 | 13.88639187 | 13.59944718 | 12.31549717 | 10.87119842 |

| ENSMUSG00000020857 | Nme2 | 1395 | 0.396105583 | 0.382083375 | 0.41611639 | 0.359262715 | 0.241522198 | 0.24865171 | 0.406990786 | 0.109512265 | 0.324768225 | 0.135199149 | 0.479461949 | 0.120033683 | 0.388997197 | 0.299639273 | 0.206746159 | 0.219587713 | 0.494532863 | 0.512629668 |
| --- | --- | --- | --- | --- | --- | --- | --- | --- | --- | --- | --- | --- | --- | --- | --- | --- | --- | --- | --- | --- |
| ENSMUSG00000020859 | Spag9 | 11634 | 9.974138779 | 10.9095863 | 11.58819229 | 10.87528108 | 10.62427196 | 10.89103832 | 9.463349137 | 8.912873692 | 10.12847014 | 8.774391658 | 9.264251876 | 9.556884981 | 8.743545936 | 8.087993902 | 9.664693659 | 9.436064244 | 10.98990257 | 8.009749595 |
| ENSMUSG00000020863 | Luc7l3 | 6613 | 23.18405715 | 20.47234267 | 23.91977079 | 18.9808978 | 20.77249511 | 18.6956102 | 23.08755707 | 23.89263099 | 28.36905781 | 16.77689472 | 21.85381058 | 24.29116542 | 20.76819261 | 17.78261444 | 20.16776598 | 21.65535637 | 19.69924137 | 20.75422974 |
| ENSMUSG00000020864 | Ankrd40 | 2705 | 48.35061983 | 51.39175881 | 46.33486771 | 52.88786662 | 47.43796987 | 51.65941172 | 48.06478436 | 43.69888556 | 50.94642578 | 48.45802405 | 44.54286332 | 41.55739166 | 37.05820921 | 52.67669505 | 40.66844888 | 39.055004 | 49.30702892 | 46.91534536 |
| ENSMUSG00000020865 | Abcc3 | 5782 | 3.190461155 | 6.48166828 | 3.907028481 | 3.017966098 | 4.137247676 | 5.047831214 | 1.661098897 | 1.902354184 | 2.272310365 | 1.427079396 | 2.462285996 | 2.316803705 | 8.583173431 | 6.867812992 | 7.866917624 | 19.01947846 | 10.81117092 | 12.53926023 |
| ENSMUSG00000020866 | Cacna1g | 11174 | 0.095098389 | 0.11627017 | 0 | 0.12639989 | 0.017229971 | 0.031042521 | 0 | 0 | 0 | 0 | 0.123990853 | 0 | 0 | 0 | 0 | 0 | 0 | 0 |
| ENSMUSG00000020867 | Spata20 | 2858 | 0 | 0 | 0 | 0.079707915 | 0 | 0 | 0 | 0 | 0 | 0 | 0 | 0 | 0 | 0 | 0 | 0.013397693 | 0.026820362 | 0 |
| ENSMUSG00000020868 | Xylt2 | 4289 | 6.699346933 | 9.79425498 | 9.158150303 | 9.316165979 | 9.841850561 | 9.554700552 | 7.214383777 | 7.8183561 | 7.480600555 | 9.080550452 | 6.883871378 | 7.378755115 | 5.739479301 | 6.302275323 | 6.637975081 | 5.320867804 | 7.970868929 | 6.143474498 |
| ENSMUSG00000020869 | Lrrc59 | 3361 | 49.6504972 | 53.07662855 | 49.33783459 | 53.66741283 | 50.82421812 | 49.93606326 | 39.86596451 | 44.93090125 | 36.59117407 | 43.26473337 | 43.99389958 | 39.87307524 | 38.80797063 | 52.96643468 | 44.48689269 | 42.20966987 | 40.85781666 | 40.55714563 |
| ENSMUSG00000020870 | Cdc34-ps | 1251 | 0.033976959 | 0.079887036 | 0.07733578 | 0 | 0 | 0.039610498 | 0 | 0 | 0.065845749 | 0 | 0 | 0.044616837 | 0 | 0 | 0 | 0 | 0 | 0 |
| ENSMUSG00000020871 | Dlx4 | 3256 | 0 | 0.102312329 | 0.148567354 | 0.01399295 | 0 | 0 | 0 | 0 | 0 | 0.072405871 | 0 | 0 | 0 | 0.385132174 | 0.278388891 | 0.105840131 | 0.235419514 | 0.202736266 |
| ENSMUSG00000020872 | Tac4 | 1247 | 0 | 0 | 0.193959625 | 0.036536523 | 0 | 0 | 0.151764808 | 0.091882283 | 0.231199367 | 0.189056549 | 0.038311915 | 0 | 0.158241916 | 0.223467943 | 0.16520271 | 0.061412362 | 0 | 0.088226314 |
| ENSMUSG00000020873 | Slc35b1 | 2570 | 53.12320055 | 50.37117001 | 56.24128743 | 50.54262156 | 56.4098654 | 52.34851038 | 63.62358692 | 63.66391125 | 59.8085902 | 66.7815408 | 64.28241495 | 60.50678442 | 73.46040755 | 67.58789371 | 66.80423478 | 73.88448331 | 68.27151254 | 68.57944667 |
| ENSMUSG00000020875 | Hoxb9 | 2868 | 37.08087532 | 34.99712353 | 33.22728549 | 32.66161337 | 35.02486914 | 29.78692911 | 31.21185095 | 26.7133749 | 27.01249517 | 26.00851205 | 27.58554088 | 22.40021884 | 28.08891943 | 26.57417971 | 19.73882071 | 27.83679095 | 23.45280721 | 23.72603383 |
| ENSMUSG00000020876 | Snx11 | 5667 | 2.917683691 | 3.06264653 | 3.516833334 | 4.011815974 | 2.44609035 | 2.67569022 | 3.189243577 | 4.158234036 | 3.524873856 | 2.704072447 | 3.591341506 | 2.964622276 | 3.508163519 | 2.794676866 | 3.955116712 | 3.452708732 | 3.746738748 | 4.038078065 |
| ENSMUSG00000020877 | Scrn2 | 2146 | 8.299006876 | 7.606392436 | 6.965247381 | 6.66643426 | 7.850034149 | 7.920124705 | 11.00141043 | 11.67484435 | 11.59210416 | 8.722660388 | 8.793620037 | 9.623390101 | 11.58587433 | 10.8860093 | 10.27159012 | 10.47371228 | 8.143891607 | 10.61219487 |
| ENSMUSG00000020878 | Lrrc46 | 1328 | 0.96020729 | 0.576955246 | 1.019923833 | 0.651852291 | 0.072487837 | 0.335824249 | 0.534405259 | 1.926875724 | 1.054474048 | 0.497070667 | 0.791452625 | 1.260895992 | 0.445770337 | 1.189080551 | 0.62050536 | 0.432499331 | 0.51948294 | 0.828450402 |
| ENSMUSG00000020882 | Cacnb1 | 4972 | 1.085711455 | 1.22611819 | 1.011835711 | 1.860195495 | 1.03582378 | 1.335492015 | 1.103835631 | 1.759062782 | 1.35852546 | 1.31817131 | 1.921760196 | 2.054357654 | 1.200555509 | 1.746790897 | 0.812098246 | 2.094739584 | 1.117721853 | 1.338717199 |
| ENSMUSG00000020883 | Fbxl20 | 17848 | 3.515107565 | 3.854276477 | 3.38246111 | 3.382361259 | 3.203760943 | 3.187278023 | 3.072355159 | 3.432351718 | 3.246830327 | 3.080329449 | 3.870606788 | 3.771497595 | 2.573286362 | 4.350880441 | 3.243399038 | 3.175151219 | 3.734280049 | 4.841960257 |
| ENSMUSG00000020884 | Asgr1 | 1580 | 0 | 0 | 0 | 0 | 0 | 0 | 0 | 0 | 0 | 0 | 0.120949262 | 0 | 0 | 0 | 0 | 0 | 0 | 0 |
| ENSMUSG00000020886 | Dlg4 | 4632 | 0.29364543 | 0.237332796 | 0.396846752 | 0.472135174 | 0.374082308 | 0.299541567 | 0.541358373 | 0.412266865 | 0.7913644 | 0.264662842 | 0.360993857 | 0.20485023 | 0.298206754 | 0.250669729 | 0.195689601 | 0.851453489 | 0.620568279 | 0.368152485 |
| ENSMUSG00000020887 | A230052G05R | 2301 | 0 | 0.202686014 | 0.105114147 | 0.25740703 | 0.104589143 | 0.064605911 | 0 | 0.099589055 | 0.035798797 | 0 | 0 | 0.16979993 | 0 | 0.121105834 | 0 | 0 | 0.016656365 | 0 |
| ENSMUSG00000020888 | Dvl2 | 6693 | 3.765959866 | 4.076387325 | 4.365398526 | 3.247066808 | 3.631648207 | 3.997979401 | 4.418112107 | 3.098531975 | 4.609099104 | 4.107105931 | 4.047273488 | 3.552588116 | 3.582147288 | 2.511991578 | 3.878228031 | 4.84568123 | 3.830911793 | 5.399818186 |
| ENSMUSG00000020889 | Nr1d1 | 3064 | 8.226360765 | 10.79624803 | 8.241182391 | 10.61703187 | 12.78700583 | 10.31809531 | 11.55022318 | 11.03142171 | 8.441622682 | 13.12648495 | 12.87928058 | 12.11403904 | 11.20594466 | 13.82408867 | 14.18656706 | 13.63415557 | 18.31256484 | 14.2370175 |
| ENSMUSG00000020890 | Gucy2e | 10472 | 0 | 0.028630257 | 0.04157389 | 0 | 0.022981247 | 0.028391559 | 0.045180175 | 0.036470973 | 0.062928214 | 0.06753825 | 0.082118913 | 0.15456973 | 0.02826504 | 0.02661044 | 0.078688991 | 0 | 0.025619182 | 0 |
| ENSMUSG00000020891 | Alox8 | 3726 | 0.068446338 | 0 | 0 | 0 | 0 | 0.159590124 | 0 | 0.010250242 | 0 | 0.063272549 | 0 | 0 | 0.145639047 | 0.04985946 | 0 | 0.010276599 | 0.123434129 | 0 |
| ENSMUSG00000020892 | Aloxe3 | 6718 | 0.03163529 | 0.014876255 | 0.007200585 | 0.006781936 | 0.078810831 | 0 | 0 | 0 | 0.110353868 | 0.112297001 | 0 | 0.083083749 | 0 | 0.01382676 | 0 | 0 | 0 | 0.049129896 |
| ENSMUSG00000020893 | Per1 | 5607 | 5.928134056 | 8.549537147 | 6.867367605 | 8.345138672 | 8.318144112 | 7.865513266 | 7.020536627 | 10.54429087 | 7.044385343 | 10.87316913 | 8.469468295 | 8.75012795 | 6.633897918 | 9.061856894 | 9.133856603 | 12.46305663 | 10.58809237 | 11.89067903 |
| ENSMUSG00000020894 | Vamp2 | 3924 | 74.16741597 | 84.60660137 | 91.23662033 | 90.6460431 | 83.78928914 | 91.38968728 | 110.2515637 | 109.0488472 | 97.71826242 | 96.77669828 | 107.384081 | 116.254182 | 81.60384175 | 78.38919235 | 88.61904478 | 82.44580579 | 86.38067528 | 90.63044724 |
| ENSMUSG00000020895 | Tmem107 | 1555 | 4.674202637 | 3.66334721 | 4.572931808 | 4.717252822 | 3.404830613 | 4.493206067 | 6.085232016 | 6.877088536 | 5.297301063 | 5.670213189 | 6.943498789 | 6.927603138 | 6.725637597 | 6.242324286 | 5.511204904 | 4.432353268 | 4.017483212 | 6.650618691 |
| ENSMUSG00000020897 | Aurkb | 2825 | 0.01504608 | 0 | 0.085616868 | 0.016127803 | 0.204454189 | 0.192948697 | 0.217722062 | 0.040558303 | 0.583171905 | 0.133524115 | 0 | 0.23709308 | 0.261939384 | 0.115082694 | 0.306277053 | 0.189758762 | 0.379871261 | 0.642584256 |
| ENSMUSG00000020898 | 1500010J02R | 6926 | 7.812458718 | 7.758258501 | 7.976114881 | 9.334554131 | 8.478334807 | 9.300975116 | 6.94729923 | 5.608096051 | 7.046783306 | 9.306239006 | 8.656883178 | 8.220036939 | 6.495915183 | 6.015065895 | 6.757864206 | 6.402039181 | 7.099716853 | 5.16256416 |
| ENSMUSG00000020899 | Pfas | 10183 | 0.784736629 | 1.207154863 | 0.783819357 | 1.185669915 | 0.964245549 | 0.856454985 | 1.147621693 | 1.413977777 | 1.464157783 | 1.268711843 | 0.882028105 | 1.205877027 | 1.230512323 | 1.017090395 | 1.306894093 | 1.015267013 | 1.004922054 | 0.993977771 |
| ENSMUSG00000020900 | Myh10 | 10412 | 4.106819735 | 3.60260458 | 3.010569311 | 3.426267548 | 3.078741948 | 3.364750542 | 3.735211494 | 3.326979349 | 2.618658609 | 2.65369882 | 3.317450536 | 3.119930429 | 3.714581554 | 3.729087309 | 2.868913563 | 3.993814794 | 3.011036388 | 1.94951598 |
| ENSMUSG00000020901 | Pik3r5 | 5576 | 0.060983036 | 0.113512373 | 0.164830896 | 0.089880109 | 0.138111689 | 0.071094309 | 0 | 0.35617018 | 0.044318346 | 0.101472102 | 0.068543699 | 0.030029948 | 0.168096562 | 0.249878519 | 0.133003588 | 0.089271502 | 0 | 0.276229374 |
| ENSMUSG00000020902 | Ntn1 | 6268 | 0.081375576 | 0.069091835 | 0.09261046 | 0.007268833 | 0.0998269 | 0.071151021 | 0.015096579 | 0 | 0.137989284 | 0.120359166 | 0.007622042 | 0.07123888 | 0.01574088 | 0.118555424 | 0.013146636 | 0.036653421 | 0.097833559 | 0.026328545 |
| ENSMUSG00000020903 | Stx8 | 7049 | 13.57343612 | 11.04916253 | 10.41036254 | 10.21229253 | 12.21563505 | 11.52879601 | 12.43728104 | 11.06924076 | 11.26508759 | 12.4080798 | 11.30495542 | 10.72921305 | 11.98130832 | 11.70161714 | 11.93553371 | 12.14065932 | 10.87968791 | 10.26201948 |
| ENSMUSG00000020904 | Wdr16 | 2731 | 1.058349312 | 1.110023205 | 1.257605513 | 1.484779548 | 1.268948555 | 1.161250439 | 2.373429884 | 1.440431142 | 1.70416561 | 1.225814695 | 2.36163288 | 1.491960223 | 1.535413024 | 2.363869699 | 2.247906198 | 3.098580826 | 2.483981894 | 2.67894954 |
| ENSMUSG00000020905 | Usp43 | 5915 | 2.46479719 | 3.638229867 | 2.813270404 | 3.389156299 | 2.351669643 | 3.736351509 | 2.247652203 | 3.331746347 | 1.42742785 | 3.013181037 | 2.616920803 | 2.717651872 | 1.86819095 | 2.065054097 | 2.117544039 | 1.696050573 | 2.086401962 | 1.915786303 |
| ENSMUSG00000020907 | Rcvrn | 1037 | 0 | 0 | 0 | 0 | 0 | 0 | 0 | 0 | 0 | 0 | 0 | 0 | 0 | 0 | 0 | 0 | 0 | 0 |
| ENSMUSG00000020908 | Myh3 | 6132 | 0 | 0 | 0 | 0 | 0 | 0 | 0 | 0 | 0 | 0 | 0 | 0 | 0 | 0 | 0 | 0 | 0 | 0 |

| ENSMUSG00000020910 | 2310004I24Ri | k | 2375 | 9.592747095 | 9.369689827 | 8.819258376 | 8.709353317 | 10.51809195 | 8.512638021 | 10.08009075 | 9.230500623 | 10.37033113 | 8.993376242 | 10.41996989 | 10.34058591 | 9.409414116 | 7.548386414 | 7.910698731 | 8.657707863 | 7.923457591 | 7.180136035 |
| --- | --- | --- | --- | --- | --- | --- | --- | --- | --- | --- | --- | --- | --- | --- | --- | --- | --- | --- | --- | --- | --- |
| ENSMUSG00000020911 | Krt19 |  | 2107 | 103.9329221 | 112.982412 | 88.66567358 | 93.21958336 | 85.29881489 | 92.6850133 | 19.10920255 | 25.03260927 | 18.70692719 | 17.36542275 | 14.60231289 | 26.17269798 | 26.0356393 | 42.56456229 | 18.0684564 | 27.56851041 | 22.35549069 | 20.57293597 |
| ENSMUSG00000020912 | Krt12 |  | 1853 | 0 | 0 | 0 | 0 | 0 | 0 | 0 | 0 | 0 | 0 | 0 | 0 | 0 | 0 | 0 | 0 | 0 | 0 |
| ENSMUSG00000020913 | Krt24 |  | 1839 | 0 | 0 | 0 | 0 | 0 | 0 | 0 | 0 | 0 | 0 | 0 | 0 | 0 | 0 | 0 | 0 | 0 | 0 |
| ENSMUSG00000020914 | Top2a |  | 9357 | 0.018170429 | 0.21717287 | 0.015509308 | 0.019476774 | 0.005143948 | 0.047662136 | 0.151691821 | 0.044898624 | 0.189272222 | 0 | 0.362511708 | 0.023860495 | 0.147621426 | 0.079417057 | 0.343456381 | 0.020460942 | 0.434176282 | 0.035273554 |
| ENSMUSG00000020916 | Krt36 |  | 1664 | 0 | 0 | 0 | 0.027380435 | 0 | 0 | 0 | 0 | 0 | 0.056671518 | 0 | 0 | 0.088939755 | 0.083733331 | 0.049521101 | 0 | 0 | 0 |
| ENSMUSG00000020917 | Acly |  | 6232 | 43.4327601 | 54.7321444 | 51.19117983 | 55.76695221 | 50.28673861 | 57.03494191 | 42.79550243 | 48.60461224 | 40.91555184 | 42.54306874 | 46.17274951 | 40.38395741 | 44.20241112 | 58.4277351 | 57.03559414 | 40.92646597 | 50.47853738 | 50.93108886 |
| ENSMUSG00000020918 | Kat2a |  | 4085 | 8.636302596 | 9.092748361 | 10.33784382 | 8.5656994 | 8.801602688 | 8.17589778 | 8.234838364 | 8.423832207 | 8.005408449 | 9.187750255 | 7.882575769 | 9.058943524 | 7.197508616 | 9.015951359 | 8.684097827 | 7.264435921 | 6.792714555 | 7.743019918 |
| ENSMUSG00000020919 | Stat5b |  | 5519 | 12.83858098 | 11.14856235 | 11.07884442 | 11.21896344 | 11.52933558 | 11.19627809 | 6.918161242 | 9.674393651 | 9.358197276 | 9.619802839 | 8.500635845 | 8.343526297 | 10.51174755 | 9.172686221 | 8.615074379 | 9.955974202 | 8.152758211 | 9.369190129 |
| ENSMUSG00000020921 | Tmem101 |  | 2017 | 8.724413919 | 7.613903934 | 8.370035743 | 9.396824216 | 7.158937581 | 7.566803144 | 6.169179255 | 7.782388393 | 9.597254541 | 6.91948843 | 9.450772649 | 9.187307872 | 8.7559873 | 8.243422551 | 7.660180198 | 8.675660676 | 6.517560653 | 9.245457203 |
| ENSMUSG00000020922 | Lsm12 |  | 2530 | 13.30596815 | 14.02301674 | 15.56365759 | 11.65138169 | 12.99371694 | 13.49479582 | 16.56878796 | 12.37856521 | 12.437351 | 12.59836972 | 10.40474392 | 11.36168626 | 16.0474972 | 12.42792378 | 14.83581716 | 12.81902943 | 14.633677 | 11.87153054 |
| ENSMUSG00000020923 | Ubtf |  | 7371 | 16.12901606 | 14.43513555 | 15.63887164 | 14.5812649 | 12.25010023 | 13.96968937 | 14.57058488 | 12.77743377 | 15.1760381 | 13.63794592 | 14.7323946 | 15.08408627 | 13.84720348 | 16.07994994 | 14.28164093 | 12.65444575 | 12.45305672 | 15.9258491 |
| ENSMUSG00000020925 | Ccdc43 |  | 5144 | 11.19644509 | 9.642865376 | 11.55736174 | 10.9119764 | 9.469188715 | 11.70423237 | 9.859873991 | 8.523498826 | 9.656092148 | 9.175321532 | 9.454686573 | 8.626254229 | 7.183049386 | 9.254508082 | 10.44456238 | 10.41379466 | 9.596475585 | 9.025599937 |
| ENSMUSG00000020926 | Adam11 |  | 5643 | 0.037661861 | 0.100357824 | 0.068578459 | 0.032295619 | 0 | 0.052687649 | 0.201223515 | 0.223347382 | 0.138675137 | 0.058489265 | 0.008466234 | 0.049455664 | 0.1398743 | 0 | 0.226342058 | 0.379988307 | 0.0679183 | 0.23395686 |
| ENSMUSG00000020928 | Higd1b |  | 754 | 0.056372913 | 0 | 0.320779379 | 0.181277365 | 0 | 0 | 0.752986933 | 0.202612214 | 0.491616236 | 0 | 0 | 0 | 0 | 0.123193866 | 0 | 0.406266392 | 0.254153162 | 0 |
| ENSMUSG00000020929 | Eftud2 |  | 8075 | 11.16978124 | 11.10979865 | 11.8912022 | 13.07869979 | 11.42052827 | 11.43855051 | 11.38433872 | 11.19995157 | 10.67024037 | 11.59644541 | 10.50753266 | 11.71610503 | 11.45477955 | 11.70448518 | 13.42941116 | 11.83570973 | 12.20745952 | 15.08237304 |
| ENSMUSG00000020930 | Ccdc103 |  | 1955 | 0 | 0.170398436 | 0 | 0.23304882 | 0 | 0.101386667 | 0.701824905 | 0.371179358 | 0 | 0.096472027 | 0.488746378 | 0.456803377 | 0.428973194 | 0.570157595 | 0.569024046 | 0.332961803 | 0.431293367 | 0.056275301 |
| ENSMUSG00000020932 | Gfap |  | 4207 | 0 | 0 | 0 | 0 | 0 | 0 | 0 | 0 | 0 | 0 | 0 | 0 | 0 | 0 | 0 | 0 | 0 | 0 |
| ENSMUSG00000020935 | Dcakd |  | 3671 | 27.51084125 | 26.11672829 | 24.48325236 | 26.05083957 | 26.82591006 | 25.22856417 | 26.49819337 | 26.55053417 | 20.8120367 | 26.88270823 | 30.33599243 | 23.01958953 | 21.12497248 | 24.26580213 | 23.16535314 | 23.44791213 | 20.45252401 | 23.60101964 |
| ENSMUSG00000020936 | Nmt1 |  | 5088 | 14.61115426 | 14.43689696 | 13.63357754 | 13.85277822 | 14.15199642 | 12.14470495 | 14.0877965 | 14.20204901 | 12.07749244 | 12.94605591 | 12.81698473 | 12.88981988 | 11.62519041 | 12.66076046 | 13.71765638 | 14.02784831 | 13.57389682 | 14.14149206 |
| ENSMUSG00000020937 | Plcd3 |  | 3720 | 0.788402459 | 0.573125062 | 0.689192771 | 1.102283331 | 1.048033822 | 0.452901328 | 0.661360028 | 1.108811683 | 1.12930769 | 0.773170134 | 0.423810116 | 1.095307357 | 0.795676085 | 1.460741458 | 1.461990693 | 0.957265185 | 0.473927327 | 0.842881474 |
| ENSMUSG00000020940 | 1700023F06R |  | 1612 | 0.975615083 | 0.392645776 | 0.750209838 | 0.734855554 | 0.716604323 | 0.952937184 | 0.557655645 | 0.947702293 | 0.638748694 | 0.994493741 | 0.711289704 | 0.657876916 | 0.275426337 | 1.584630773 | 1.354641726 | 0.80761827 | 1.141229684 | 1.160241705 |
| ENSMUSG00000020941 | Map3k14 |  | 5633 | 2.844745493 | 3.465534531 | 3.357722416 | 2.652942045 | 3.306773382 | 2.5246999 | 0.974306898 | 2.73238739 | 1.352654965 | 2.159574195 | 2.272978674 | 1.506121199 | 1.357437809 | 1.616020083 | 1.645721654 | 1.774160932 | 1.680560152 | 1.591777809 |
| ENSMUSG00000020945 | Lyzl6 |  | 1294 | 0 | 0 | 0 | 0.035209462 | 0 | 0.03829423 | 0 | 0 | 0 | 0 | 0 | 0 | 0 | 0 | 0 | 0 | 0 | 0 |
| ENSMUSG00000020946 | Gosr2 |  | 3931 | 35.3795309 | 39.21108154 | 36.44935052 | 38.27081363 | 37.5529407 | 35.19492041 | 41.3910717 | 40.54360093 | 38.48335599 | 41.9570491 | 39.71726388 | 38.83384309 | 41.22496776 | 43.69123255 | 53.71609614 | 49.6482386 | 50.43549577 | 49.55157642 |
| ENSMUSG00000020948 | Klhl28 |  | 2849 | 4.565315502 | 5.191619878 | 4.380614543 | 5.277341043 | 6.149533245 | 4.696117257 | 3.454207517 | 5.12091882 | 5.363354632 | 5.163572979 | 5.517009947 | 5.093742461 | 4.051831745 | 4.254793545 | 4.425298737 | 5.080326289 | 4.237551255 | 6.275170125 |
| ENSMUSG00000020949 | Fkbp3 |  | 940 | 116.9344523 | 105.5026448 | 104.6206247 | 108.9102836 | 112.8539997 | 115.3418948 | 140.2267271 | 129.4884962 | 144.5471441 | 126.9056162 | 135.8536851 | 124.9914572 | 150.2524246 | 121.9404337 | 124.4812965 | 129.0882286 | 131.5733618 | 119.0303437 |
| ENSMUSG00000020950 | Foxg1 |  | 3565 | 0 | 0 | 0 | 0 | 0 | 0 | 0 | 0 | 0 | 0 | 0 | 0 | 0 | 0 | 0 | 0 | 0 | 0 |
| ENSMUSG00000020952 | Scfd1 |  | 2153 | 56.6801488 | 48.32149031 | 53.3389508 | 56.7132368 | 53.65379322 | 59.61058057 | 50.4551374 | 50.05989763 | 50.25405337 | 52.25340361 | 63.68515134 | 52.10844487 | 52.79179629 | 53.39020729 | 55.19056535 | 55.89752859 | 60.11514382 | 55.49455634 |
| ENSMUSG00000020953 | Coch |  | 2589 | 1.526837146 | 0.514683572 | 0.728685857 | 2.340524873 | 1.208410598 | 0.689029899 | 2.960468902 | 4.440290894 | 2.863488929 | 4.899010883 | 2.251272667 | 3.600315045 | 46.95011362 | 37.94099839 | 34.29484471 | 62.45702397 | 38.03022663 | 35.35540888 |
| ENSMUSG00000020954 | Strn3 |  | 4204 | 54.54696115 | 49.90594857 | 58.25765561 | 52.02022189 | 54.03964789 | 53.01812456 | 45.43322665 | 45.86903895 | 49.24943262 | 46.50019403 | 47.44539763 | 48.393932 | 45.07228693 | 39.85972105 | 47.96394256 | 47.71752906 | 54.44449218 | 51.59393621 |
| ENSMUSG00000020955 | Ap4s1 |  | 984 | 44.40581398 | 39.3390072 | 36.52594823 | 37.04149946 | 39.91427816 | 35.75451305 | 39.6195604 | 35.6695303 | 40.01454174 | 32.77548885 | 37.14211711 | 35.90580733 | 39.75631138 | 36.01304744 | 37.8099644 | 36.2282068 | 34.74294394 | 31.75322421 |
| ENSMUSG00000020956 | 6530401N04R |  | 3104 | 10.46196987 | 11.50496862 | 10.45703572 | 12.24159503 | 10.82348026 | 10.23302262 | 12.86465883 | 10.96308974 | 9.951638796 | 11.31677639 | 11.91295679 | 12.11976694 | 13.09584402 | 13.46639132 | 12.37108572 | 10.75689744 | 11.10030313 | 11.74969 |
| ENSMUSG00000020961 | Ston2 |  | 6901 | 1.81082767 | 1.583339995 | 1.53511131 | 1.37323536 | 1.443748471 | 1.65869895 | 1.796250091 | 1.494268751 | 1.420430481 | 1.509970355 | 1.654573986 | 1.431585608 | 1.444000477 | 3.089093771 | 1.564238175 | 1.670116335 | 1.416201378 | 1.777572931 |
| ENSMUSG00000020962 | Gtf2a1 |  | 6740 | 6.558662176 | 8.950986859 | 8.849341681 | 9.443439011 | 9.36215904 | 9.167990916 | 7.69357509 | 9.791761329 | 8.225081634 | 7.975044765 | 8.23657296 | 7.560786341 | 6.118914368 | 6.498037746 | 8.105825387 | 7.226357955 | 9.297254516 | 7.786155462 |
| ENSMUSG00000020963 | Tshr |  | 4863 | 0.061183679 | 1.575563576 | 0.228787004 | 0.037475669 | 1.167914249 | 0.560436016 | 0.19458227 | 0.062829369 | 0.042346819 | 0.20361192 | 0.117890089 | 0.229552386 | 0 | 0.286515036 | 0.355843172 | 0.10236025 | 0.441347445 | 0.21492351 |
| ENSMUSG00000020964 | Sel1l |  | 12255 | 11.74051578 | 13.63504508 | 12.86408286 | 13.29096153 | 12.44242629 | 14.27749508 | 10.87168534 | 12.24774716 | 10.84191757 | 11.86173955 | 11.18062676 | 10.83065244 | 11.15452817 | 10.93738362 | 11.73008801 | 10.20457966 | 12.72852129 | 12.32598997 |
| ENSMUSG00000020973 | 1110034A24R |  | 3263 | 8.389008079 | 6.197035486 | 6.181968176 | 8.293981102 | 5.841324478 | 6.423783728 | 6.075394567 | 5.536318216 | 5.932473922 | 6.256896877 | 9.194812726 | 6.277765299 | 7.650000043 | 7.757287045 | 6.490223637 | 10.10365093 | 8.668344197 | 8.76639151 |
| ENSMUSG00000020974 | Pole2 |  | 1676 | 0.152166501 | 0.417404999 | 0.375212348 | 0.13592197 | 0.430774973 | 0.236528561 | 0.028229522 | 0.387393103 | 0.270317228 | 0.225063023 | 0.342064142 | 0.26642321 | 0.088302955 | 0.637359194 | 0.27041594 | 0.388389216 | 0.914708757 | 0.393859953 |
| ENSMUSG00000020978 | Klhdc2 |  | 4848 | 35.94703418 | 32.76317649 | 35.35219019 | 35.5240816 | 36.19822477 | 31.31798178 | 33.09350129 | 30.479869 | 35.40952923 | 33.02883421 | 32.35255542 | 29.89960308 | 37.31438545 | 32.46679294 | 35.209993 | 43.05323864 | 33.78058306 | 39.32788034 |
| ENSMUSG00000020982 | Nemf |  | 3678 | 52.96389933 | 49.77007986 | 44.44104379 | 40.68038869 | 45.17438839 | 42.06189076 | 41.53687881 | 46.08425282 | 46.49440279 | 42.63818353 | 44.1768988 | 43.0226763 | 47.13223418 | 38.74128877 | 42.89308252 | 48.86789319 | 52.01872598 | 45.10806576 |

| ENSMUSG00000020986 | Sec23a | 5202 | 8.898142386 | 9.983622085 | 8.889868324 | 9.424006864 | 8.207038888 | 10.74499875 | 7.339740849 | 8.751507818 | 6.99901575 | 8.592631043 | 7.889021402 | 8.261833947 | 8.895297656 | 8.38350597 | 5.995689698 | 6.46273612 | 9.297921317 | 6.471659614 |
| --- | --- | --- | --- | --- | --- | --- | --- | --- | --- | --- | --- | --- | --- | --- | --- | --- | --- | --- | --- | --- |
| ENSMUSG00000020988 | L2hgdh | 3328 | 7.752596709 | 9.449330572 | 8.881077839 | 8.405793636 | 6.739626331 | 8.829558597 | 4.591945701 | 5.956086793 | 6.324011284 | 7.239786468 | 5.483784297 | 5.450748299 | 5.914493688 | 3.586577662 | 5.521602743 | 5.177516025 | 7.151631724 | 5.917445976 |
| ENSMUSG00000020990 | Cdkl1 | 1679 | 0.734157299 | 0.773795637 | 0 | 0.651259717 | 0.860010548 | 0.796857538 | 0.253611739 | 0.181976902 | 0.490607692 | 0.758230487 | 0.028454412 | 0.565137739 | 0.26443553 | 0 | 0.319011451 | 0 | 0.136961156 | 0 |
| ENSMUSG00000020992 | 4930512B01R | 1855 | 0.252052257 | 0.017958434 | 0 | 0.02456121 | 0.103788515 | 0.080139192 | 0.025505487 | 0.041177792 | 0.044405947 | 0 | 0.025754695 | 0 | 0 | 0 | 0.111055407 | 0 | 0 | 0 |
| ENSMUSG00000020993 | Trappc6b | 1248 | 86.44081471 | 72.07116538 | 74.69214182 | 67.28285634 | 72.27501999 | 69.24676872 | 84.69272815 | 74.54863162 | 89.53446896 | 88.86094075 | 83.49133369 | 71.29019725 | 87.91200643 | 95.38156729 | 89.7322347 | 93.45608185 | 95.14011843 | 87.4944526 |
| ENSMUSG00000020994 | Pnn | 3460 | 36.30138588 | 36.43236755 | 35.31547332 | 32.48528797 | 34.20705211 | 31.03490568 | 38.09627847 | 41.34934665 | 40.06757574 | 28.74012519 | 37.15676684 | 36.5221561 | 34.80323991 | 29.43696059 | 29.4126714 | 33.38808166 | 30.1071893 | 32.3695206 |
| ENSMUSG00000021000 | Ctage5 | 9445 | 14.80593215 | 14.41854013 | 14.39682306 | 14.91044871 | 14.15165188 | 15.47178521 | 17.04659041 | 15.30122294 | 15.99057208 | 13.39888698 | 14.61321917 | 14.67340288 | 13.87770293 | 13.65539764 | 14.03775615 | 12.73380602 | 14.26330689 | 14.16433537 |
| ENSMUSG00000021003 | Galc | 3843 | 4.059172418 | 5.530477885 | 4.64476521 | 4.730381652 | 5.686154917 | 6.202150625 | 3.607237816 | 5.088345051 | 3.954677157 | 3.521272921 | 3.530598024 | 4.72029413 | 3.542963614 | 3.782721663 | 4.342084344 | 4.971900363 | 4.777074217 | 3.163417274 |
| ENSMUSG00000021007 | Spata7 | 4802 | 3.522919629 | 3.316027371 | 3.243706117 | 3.757220649 | 3.718647164 | 2.765541982 | 2.679935166 | 3.618813639 | 1.98127554 | 3.397364291 | 4.855097821 | 4.126314081 | 2.814857421 | 4.091180543 | 2.539704403 | 4.680671917 | 4.748885185 | 2.714943418 |
| ENSMUSG00000021009 | Ptpn21 | 5527 | 4.129777371 | 3.477752843 | 3.1595521 | 2.794498648 | 3.274399005 | 3.944853044 | 2.191432207 | 3.005915514 | 3.003105817 | 2.610478595 | 2.014033893 | 3.413369632 | 2.311736308 | 2.016750674 | 1.692193448 | 2.577185807 | 2.489441033 | 2.090087283 |
| ENSMUSG00000021010 | Npas3 | 5873 | 0.27502072 | 0.079410952 | 0.008236596 | 0.023273137 | 0.040977289 | 0.059061661 | 0.024167893 | 0.026012193 | 0 | 0 | 0.008134677 | 0.076030189 | 0.134396505 | 0 | 0 | 0 | 0 | 0.046832204 |
| ENSMUSG00000021012 | Zc3h14 | 36640 | 2.540565925 | 2.606661237 | 2.647077739 | 2.789121792 | 2.6180929 | 2.980737577 | 2.734941429 | 2.400575948 | 2.371821732 | 2.524827505 | 2.262269459 | 2.260656203 | 2.008821522 | 2.721491693 | 2.545860331 | 2.229090218 | 2.599368117 | 2.824021008 |
| ENSMUSG00000021013 | Ttc8 | 2902 | 8.553763887 | 8.942365471 | 10.58483521 | 9.843823156 | 12.09103113 | 9.510982978 | 8.331074753 | 10.42328832 | 11.7797409 | 10.77219719 | 8.725268089 | 10.57843364 | 10.13157226 | 9.858565759 | 8.064260422 | 12.03343692 | 9.70703936 | 10.10332663 |
| ENSMUSG00000021018 | Polr2h | 1442 | 17.83330894 | 19.19765263 | 18.61810634 | 16.61935459 | 15.75469346 | 18.38468271 | 18.99725458 | 23.22797289 | 18.73672284 | 17.49349947 | 21.96587897 | 21.17279295 | 21.62120091 | 20.54877098 | 21.85796828 | 19.0125346 | 17.14317719 | 16.63243449 |
| ENSMUSG00000021020 | Srp54a | 2500 | 0 | 0 | 0 | 0 | 0 | 0 | 0 | 0.030553922 | 0 | 0 | 0 | 0 | 0 | 0 | 0 | 0 | 0.015330519 | 0.044007285 |
| ENSMUSG00000021022 | Ppp2r3c | 2381 | 13.03182633 | 13.66933963 | 13.71362158 | 15.8439919 | 12.7960973 | 13.15301454 | 11.08797767 | 11.91808273 | 14.96276192 | 13.76301502 | 10.45390734 | 10.00978073 | 12.74217939 | 14.66860719 | 13.68770714 | 12.43117998 | 13.44076351 | 12.70684952 |
| ENSMUSG00000021023 | 1110008L16R | 3110 | 6.081930332 | 5.516443898 | 6.563868106 | 6.870781284 | 6.05131259 | 6.038741488 | 6.632902898 | 6.177099167 | 7.336761972 | 7.140829979 | 7.204648079 | 9.260733728 | 6.836674066 | 4.211328824 | 5.193250775 | 7.104077322 | 5.730459183 | 8.313273363 |
| ENSMUSG00000021024 | Psma6 | 1226 | 255.932471 | 213.7894386 | 224.5857543 | 206.3625442 | 224.2099642 | 214.3378027 | 245.9024389 | 221.0860358 | 243.8940493 | 239.253283 | 255.6310992 | 235.0085239 | 262.6341142 | 245.4793526 | 244.3527838 | 280.4958769 | 249.8712 | 248.6626994 |
| ENSMUSG00000021025 | Nfkbia | 1571 | 235.225971 | 232.5753175 | 261.6666206 | 280.6745939 | 295.6850384 | 272.019589 | 294.2360746 | 319.9555748 | 359.4578724 | 394.4031773 | 348.9001264 | 454.8390276 | 409.8223121 | 358.544807 | 353.5570815 | 615.2089252 | 442.569925 | 476.5588431 |
| ENSMUSG00000021027 | Ralgapa1 | 8004 | 13.03194677 | 14.86677388 | 14.40810799 | 14.72593974 | 14.25195641 | 13.89259547 | 12.27150443 | 14.40088337 | 13.67738117 | 14.04388763 | 14.39098262 | 15.20216697 | 15.55644168 | 12.59746548 | 14.9126571 | 14.15566058 | 15.76338948 | 16.03401374 |
| ENSMUSG00000021028 | Mbip | 1500 | 17.9088475 | 15.61264309 | 15.931016 | 18.497784 | 18.96397793 | 16.91399973 | 16.08631084 | 14.53857452 | 14.22307678 | 17.09998838 | 20.16103248 | 13.35854858 | 14.43780779 | 18.79437403 | 22.13896937 | 17.79236891 | 20.13408131 | 15.84262274 |
| ENSMUSG00000021032 | Ngb | 2239 | 0.056952 | 0.10414929 | 0 | 0.040697672 | 0.064491188 | 0.022131636 | 0.169049322 | 0.068231179 | 0.110370297 | 0.168470579 | 0.341402115 | 0 | 0.352528216 | 0.165945824 | 0 | 0.136813247 | 0.222528745 | 0 |
| ENSMUSG00000021033 | Gstz1 | 1647 | 34.63384713 | 28.74172598 | 34.6867877 | 30.56767091 | 33.43225299 | 29.75570948 | 44.1814816 | 42.20411195 | 41.83669755 | 41.73996682 | 40.92924494 | 37.82044897 | 32.97780262 | 32.20348986 | 38.14958879 | 35.64025571 | 42.46842249 | 34.20116896 |
| ENSMUSG00000021036 | Sptlc2 | 10052 | 17.56531051 | 18.06492105 | 18.31572389 | 17.49558607 | 18.71264999 | 17.71712341 | 13.57909657 | 15.43349966 | 14.91843453 | 14.89759586 | 14.81441958 | 15.55864371 | 17.95226357 | 17.32181492 | 19.24816021 | 22.4098332 | 20.78740258 | 18.79240673 |
| ENSMUSG00000021037 | Ahsa1 | 1338 | 52.79790924 | 50.3677018 | 50.50659335 | 54.073945 | 53.99552872 | 50.73785127 | 51.16700031 | 49.46743899 | 51.40618933 | 55.326311 | 56.16592652 | 50.22575318 | 58.73374008 | 60.7452563 | 48.22244882 | 62.64434948 | 46.97692595 | 53.61126694 |
| ENSMUSG00000021038 | Vipar | 2680 | 24.56735734 | 26.93620962 | 28.93386909 | 30.66870298 | 25.37701796 | 26.01518513 | 30.84151123 | 29.81287531 | 28.76908862 | 28.32560905 | 25.81273876 | 25.7626771 | 29.94889157 | 30.03268785 | 27.28834392 | 26.21763604 | 29.41686296 | 23.44044772 |
| ENSMUSG00000021039 | Snw1 | 2217 | 52.95412548 | 55.341177 | 52.65012573 | 52.3633473 | 54.92727419 | 55.02879119 | 54.37650244 | 56.93544878 | 58.74233777 | 54.9985199 | 54.73540574 | 54.73308546 | 56.29668503 | 56.855775 | 60.67797924 | 62.05600018 | 62.77076412 | 62.77539017 |
| ENSMUSG00000021040 | 1810035L17R | 2811 | 12.79237955 | 12.40789763 | 13.07857811 | 13.06375017 | 13.15023742 | 13.80284254 | 14.7273547 | 12.43189192 | 15.22333329 | 13.1505341 | 12.38987717 | 14.13758512 | 19.74329667 | 16.95184406 | 14.42274315 | 15.69220197 | 15.58411859 | 17.63187661 |
| ENSMUSG00000021041 | 2700073G19R | 1334 | 2.230406538 | 2.247496611 | 2.175721005 | 1.502763082 | 0.829860753 | 2.37734254 | 1.276803929 | 2.6053288 | 3.82843175 | 2.827628381 | 2.900878288 | 2.677812898 | 1.886002834 | 2.43709604 | 1.266314686 | 1.578698206 | 2.41335003 | 2.721589988 |
| ENSMUSG00000021044 | Adck1 | 2318 | 4.419218043 | 4.110219045 | 7.679663145 | 6.604189343 | 5.980152725 | 6.455964426 | 7.572477947 | 5.223033461 | 5.561423397 | 5.268348638 | 7.584635341 | 7.175611497 | 6.150528081 | 4.748597373 | 5.616778111 | 5.401651691 | 5.423220614 | 4.366555495 |
| ENSMUSG00000021047 | Nova1 | 6995 | 0 | 0.038099093 | 0 | 0.006513373 | 0 | 0.049588154 | 0 | 0.060059532 | 0 | 0 | 0 | 0 | 0.014104908 | 0.013279224 | 0.005890144 | 0 | 0 | 0 |
| ENSMUSG00000021048 | Mthfd1 | 3523 | 34.65082758 | 40.1021812 | 46.47868301 | 42.89695829 | 43.33649216 | 46.24734261 | 44.94905943 | 45.02215362 | 47.31246928 | 57.44275288 | 52.3720947 | 52.06081955 | 44.92103055 | 37.95416622 | 37.13168889 | 42.15988252 | 50.11900364 | 41.19046935 |
| ENSMUSG00000021051 | Ppp2r5e | 6144 | 10.78541169 | 10.67053643 | 11.60523824 | 10.75994065 | 11.24958372 | 11.38809567 | 9.471776543 | 8.659182381 | 8.366011014 | 8.748665643 | 9.307718961 | 9.27535669 | 7.611760673 | 8.390777507 | 10.48815648 | 9.242345504 | 8.327735411 | 8.362386992 |
| ENSMUSG00000021054 | Sgpp1 | 5696 | 175.7218899 | 186.665668 | 206.4367584 | 200.4813931 | 200.3525123 | 220.9342033 | 140.7668486 | 141.8670734 | 137.1823345 | 139.1011969 | 118.7663996 | 141.0286194 | 103.713081 | 102.7624356 | 131.4673944 | 98.3011854 | 124.4662641 | 116.7012018 |
| ENSMUSG00000021055 | Esr2 | 4836 | 0.026367975 | 0.179101582 | 0 | 0.122475926 | 0.099528378 | 0.071726455 | 0.039133729 | 0 | 0.008516649 | 0 | 0 | 0.092333602 | 0.020401951 | 0.086434406 | 0.025559278 | 0.007917826 | 0.007925206 | 0 |
| ENSMUSG00000021056 | Tex21 | 2315 | 0 | 0 | 0 | 0.216488764 | 0 | 0.214050685 | 0 | 0.016497798 | 0 | 0.020367474 | 0 | 0 | 0.042619367 | 0.080248963 | 0 | 0 | 0 | 0 |
| ENSMUSG00000021057 | Akap5 | 6906 | 0.055393366 | 0.13024155 | 0.070045656 | 0.039583879 | 0.034847903 | 0.035876581 | 0 | 0.055303218 | 0.190843977 | 0.204824949 | 0.062261023 | 0.080821985 | 0 | 0.013450358 | 0.089490782 | 0.005544542 | 0.00554971 | 0.031861631 |
| ENSMUSG00000021061 | Spnb1 | 12278 | 7.283832087 | 7.067933657 | 6.922323899 | 6.605201085 | 8.377416589 | 7.385690051 | 4.119339776 | 5.664470173 | 4.414518223 | 6.129053787 | 4.895007148 | 5.123330488 | 4.44782802 | 4.134499716 | 5.050361754 | 5.679035354 | 4.791567498 | 4.753596859 |
| ENSMUSG00000021062 | Rab15 | 4707 | 25.18524239 | 23.00833208 | 22.77368669 | 27.22821706 | 22.83377641 | 22.19187644 | 17.99228708 | 18.06978546 | 17.13261055 | 21.89747978 | 19.18306172 | 17.77516002 | 17.23001317 | 21.17463598 | 21.83936307 | 24.41260098 | 18.76824184 | 18.41817552 |
| ENSMUSG00000021065 | Fut8 | 3375 | 67.44154596 | 67.0403489 | 69.51455473 | 74.8551084 | 72.63285548 | 69.50596763 | 58.42940615 | 57.2376804 | 57.02654464 | 67.18451317 | 60.26014763 | 60.89282064 | 25.69644758 | 22.01793773 | 31.14227232 | 21.73771964 | 29.20747723 | 26.24138128 |
| ENSMUSG00000021066 | Atl1 | 2803 | 1.137312951 | 0.879469915 | 0.91466183 | 1.202824574 | 0.566661963 | 0.760173936 | 0.320706707 | 0.463268527 | 0.573055339 | 1.042933857 | 1.244228316 | 0.477907921 | 0.475191497 | 0.795332214 | 1.778590176 | 0.532762643 | 1.093865056 | 0.726128059 |

| ENSMUSG00000021067 | Sav1 | 2519 | 2.480452908 | 1.891124999 | 1.747515388 | 2.206608737 | 2.197368488 | 2.281904365 | 2.1036205 | 2.562332674 | 2.272697773 | 2.021546626 | 2.143140257 | 2.370891583 | 2.036728621 | 1.272188182 | 1.782838266 | 2.112899734 | 2.495241243 | 1.61598805 |
| --- | --- | --- | --- | --- | --- | --- | --- | --- | --- | --- | --- | --- | --- | --- | --- | --- | --- | --- | --- | --- |
| ENSMUSG00000021068 | Nin | 10313 | 1.034500066 | 0.688029329 | 0.72703357 | 0.720105714 | 0.536717853 | 0.56697591 | 0.440416676 | 0.777698488 | 0.850647518 | 0.502916451 | 0.176034948 | 0.784763994 | 0.597933643 | 0.220669086 | 0.623236955 | 0.278463643 | 0.260141645 | 0.36270913 |
| ENSMUSG00000021069 | Pygl | 4143 | 6.555830915 | 7.63068709 | 7.332507136 | 5.058672555 | 3.984853927 | 5.238739393 | 5.675694286 | 6.93234048 | 6.43197579 | 5.679025085 | 4.001426645 | 3.637516027 | 8.311290915 | 7.510870999 | 6.673001208 | 5.249593291 | 4.273895527 | 3.399066213 |
| ENSMUSG00000021070 | Bdkrb2 | 1677 | 2.712017791 | 6.753955894 | 5.884436608 | 5.623814059 | 6.027253411 | 4.254975334 | 3.977989105 | 5.921302698 | 2.431404329 | 4.751621259 | 4.843019047 | 4.393361635 | 5.059683823 | 5.068138338 | 4.348643645 | 8.859126817 | 7.496139168 | 7.118053762 |
| ENSMUSG00000021071 | Trim9 | 7559 | 0 | 0 | 0.006399462 | 0 | 0 | 0.111442846 | 0 | 0 | 0 | 0.006237691 | 0.113764949 | 0.095992011 | 0.045683744 | 0 | 0 | 0 | 0 | 0.007277299 |
| ENSMUSG00000021072 | Tmx1 | 3291 | 57.86180146 | 54.40802383 | 58.60384853 | 58.46377704 | 56.40985402 | 58.81281597 | 55.34907745 | 48.57897188 | 54.96541393 | 56.44903398 | 46.65716091 | 45.24952531 | 50.80093213 | 62.22181139 | 61.58324322 | 60.01305165 | 64.42451366 | 55.59413217 |
| ENSMUSG00000021076 | Actr10 | 4009 | 42.2507175 | 39.20434895 | 38.75674221 | 40.321922 | 41.13244462 | 37.67441555 | 38.43786362 | 39.33560228 | 41.13514819 | 37.17719456 | 40.52946715 | 35.23807483 | 36.79282431 | 39.89858742 | 42.31150052 | 42.14927674 | 39.47350459 | 35.8265846 |
| ENSMUSG00000021078 | Tomm20l | 591 | 0 | 0.225467981 | 0 | 0 | 0 | 0.083845573 | 0.080055294 | 0 | 0 | 0.079781224 | 0 | 0 | 0 | 0 | 0 | 0 | 0.06484991 | 0 |
| ENSMUSG00000021079 | Timm9 | 1402 | 14.9162244 | 14.8743736 | 14.93989918 | 12.25143632 | 12.05014637 | 11.20414875 | 16.1308563 | 17.05309836 | 16.83300537 | 12.51074295 | 14.34611806 | 14.77005051 | 14.81364991 | 12.42263393 | 11.49059084 | 13.98344578 | 14.98060678 | 11.61390556 |
| ENSMUSG00000021081 | Serpina1f | 3466 | 13.47755004 | 13.5423739 | 25.01020381 | 9.490788809 | 12.12324563 | 8.878317239 | 90.27084413 | 87.58026957 | 53.09329268 | 27.95576895 | 28.76697586 | 59.32628418 | 366.2318417 | 589.3548331 | 309.14243 | 66.40647464 | 113.497147 | 260.3489286 |
| ENSMUSG00000021086 | 4930403N07R | 2609 | 0 | 0 | 0 | 0 | 0 | 0 | 0 | 0 | 0 | 0 | 0 | 0 | 0 | 0 | 0 | 0 | 0 | 0 |
| ENSMUSG00000021087 | Rtn1 | 4816 | 0.008825826 | 0.103756938 | 0.070310364 | 0.018920699 | 0 | 0.061735133 | 0.07859249 | 0.111024426 | 0 | 0.009790428 | 0 | 0.046358524 | 0.163893413 | 0.086793353 | 0.136882245 | 0.302126886 | 0.302408489 | 0.205598821 |
| ENSMUSG00000021090 | Lrrc9 | 7006 | 0 | 0.014264728 | 0.027618344 | 0.019509439 | 0.075571105 | 0.049510296 | 0.006753166 | 0.043611079 | 0 | 0.033650231 | 0 | 0 | 0 | 0.033145937 | 0.041166271 | 0.120238847 | 0.092998437 | 0.102072279 |
| ENSMUSG00000021091 | Serpina3n | 2059 | 0.206436018 | 0.016179162 | 0.164455907 | 0.309788548 | 0.187010874 | 0.240664078 | 0.643397285 | 1.910547571 | 0.260041139 | 0.137398844 | 0.301638883 | 0.216865129 | 1.173999003 | 1.939869604 | 0.240125629 | 0.688077938 | 0.018614034 | 0.026716419 |
| ENSMUSG00000021094 | Dhrs7 | 1294 | 65.3016151 | 59.95806077 | 64.67249422 | 53.37754499 | 60.55546502 | 56.33081228 | 48.70207754 | 45.65969592 | 58.34225919 | 41.21131792 | 47.33191404 | 52.96880499 | 47.23512017 | 40.48603599 | 43.2075049 | 42.40374065 | 42.17669767 | 42.59592344 |
| ENSMUSG00000021095 | Gsc | 1262 | 0 | 0 | 0 | 0 | 0 | 0 | 0 | 0 | 0 | 0 | 0 | 0 | 0 | 0 | 0 | 0 | 0 | 0 |
| ENSMUSG00000021096 | Ppm1a | 5394 | 13.60890601 | 16.40941044 | 16.51910325 | 16.41177403 | 13.70608727 | 14.92828922 | 10.71859356 | 12.44049889 | 13.58376187 | 13.66268988 | 13.56900934 | 14.10395775 | 14.98980579 | 11.88224705 | 13.17624841 | 14.19748142 | 12.81806444 | 13.94094344 |
| ENSMUSG00000021097 | Clmn | 11974 | 4.781566069 | 5.411189181 | 5.81742807 | 4.29584258 | 5.466796074 | 5.657139369 | 2.690824649 | 2.33479527 | 3.033782103 | 2.272085834 | 2.469742717 | 2.377316512 | 2.006400845 | 1.854039901 | 2.621979755 | 2.973965666 | 2.522224984 | 2.742645458 |
| ENSMUSG00000021098 | 4930447C04R | 5752 | 0 | 0.023166129 | 0 | 0.01584181 | 0 | 0.00861487 | 0.024676293 | 0.079678169 | 0 | 0.008197271 | 0 | 0.038814786 | 0 | 0 | 0 | 0 | 0 | 0.028690424 |
| ENSMUSG00000021099 | Six6 | 5370 | 0 | 0 | 0 | 0 | 0 | 0 | 0 | 0 | 0 | 0 | 0 | 0 | 0 | 0 | 0 | 0 | 0 | 0 |
| ENSMUSG00000021101 | 4930408O17R | 619 | 0 | 0 | 0 | 0.515229904 | 0.155515101 | 0 | 0 | 0 | 0 | 0 | 0 | 0 | 0 | 0.150061672 | 0 | 0 | 0.123832946 | 0 |
| ENSMUSG00000021102 | Glrx5 | 928 | 94.85799521 | 88.73865788 | 88.98019 | 86.26158933 | 93.92986397 | 85.32895286 | 112.010728 | 100.7900792 | 96.35336823 | 99.38230126 | 98.94770495 | 94.30922299 | 106.2124684 | 107.9524747 | 100.917173 | 104.7627718 | 92.51175115 | 98.51846484 |
| ENSMUSG00000021103 | Mnat1 | 2497 | 16.32457501 | 13.38119059 | 16.25366118 | 14.37809489 | 16.44234317 | 14.62569669 | 15.84036828 | 15.52474507 | 17.30262516 | 16.57922205 | 15.421152 | 15.64716212 | 16.89178585 | 17.33515797 | 17.49044824 | 18.69293169 | 17.42104405 | 16.91910051 |
| ENSMUSG00000021108 | Prkch | 5329 | 2.887384823 | 4.075812916 | 3.313255504 | 3.31726125 | 4.217978674 | 3.254542457 | 1.447169575 | 3.511780292 | 3.207431796 | 1.964243971 | 1.515100016 | 2.503273289 | 2.703118755 | 2.431581982 | 1.755067215 | 3.319621058 | 2.74735324 | 1.847744437 |
| ENSMUSG00000021109 | Hif1a | 5647 | 16.01753402 | 19.42613081 | 21.45841926 | 21.469442 | 20.37956959 | 22.16578803 | 19.34566595 | 20.1073158 | 16.49065205 | 19.68022093 | 21.07445043 | 23.60329418 | 12.78069683 | 16.64650839 | 16.70826332 | 14.53103801 | 19.70271647 | 18.08958937 |
| ENSMUSG00000021111 | Papola | 6574 | 35.7744355 | 39.80419594 | 40.19844787 | 40.07902638 | 37.36191154 | 39.85173447 | 35.54568318 | 34.16053029 | 31.37542911 | 32.23231829 | 30.78410771 | 32.52659011 | 29.61866101 | 27.28431176 | 33.88129163 | 32.89707189 | 37.16030065 | 32.60883616 |
| ENSMUSG00000021112 | Mpp5 | 5497 | 18.41865186 | 19.30172241 | 17.52077477 | 18.15148028 | 17.40699732 | 18.76819926 | 17.23982097 | 13.6733942 | 17.37521012 | 15.55106877 | 15.31371236 | 16.42891433 | 14.8884211 | 11.33854198 | 14.52585325 | 15.85399717 | 18.55307914 | 15.6511266 |
| ENSMUSG00000021113 | Snapc1 | 2115 | 11.99791494 | 14.97898932 | 15.57559062 | 12.64507472 | 11.44697759 | 8.411078655 | 12.34827365 | 14.28377792 | 12.52147973 | 10.92380359 | 8.87733271 | 10.23946907 | 12.82863097 | 15.10805301 | 15.89620312 | 8.63575401 | 11.92373681 | 8.869080564 |
| ENSMUSG00000021114 | Atp6v1d | 4287 | 144.8071136 | 123.227403 | 127.3260827 | 124.8332229 | 131.7312481 | 129.9213262 | 160.5783714 | 151.9767186 | 159.2698993 | 157.8839154 | 152.2624814 | 132.879555 | 169.3303389 | 159.9813013 | 153.6288479 | 177.8499124 | 161.6642003 | 150.66875 |
| ENSMUSG00000021115 | Vrk1 | 3781 | 9.690415693 | 9.541884274 | 8.866135027 | 11.71259854 | 11.04959263 | 11.40197784 | 7.983467113 | 10.37386862 | 9.618538328 | 10.66221933 | 12.59762856 | 8.960620786 | 9.367975828 | 8.279109997 | 10.42842872 | 8.557408954 | 12.29563558 | 8.918429627 |
| ENSMUSG00000021116 | Eif2s1 | 3397 | 32.18231167 | 30.90047974 | 33.90561548 | 30.84792522 | 34.10466302 | 31.17285595 | 30.79432826 | 28.56841169 | 30.62618158 | 31.96587271 | 27.64956384 | 27.34096631 | 30.72897761 | 30.8305614 | 29.60641682 | 30.33265367 | 35.674934 | 27.18878133 |
| ENSMUSG00000021118 | Plek2 | 1574 | 9.127540977 | 8.423463722 | 7.283689132 | 8.278563329 | 7.369627448 | 7.177905499 | 2.88565259 | 4.586000035 | 1.83167478 | 3.804408713 | 5.038528023 | 3.971635457 | 7.427995168 | 3.452324158 | 4.502330123 | 3.649041366 | 2.581059385 | 3.285169016 |
| ENSMUSG00000021120 | Pigh | 4713 | 17.35199632 | 12.28470404 | 14.81073717 | 14.32664284 | 16.22780113 | 13.46850238 | 12.33763683 | 10.52661378 | 12.83746905 | 11.43502097 | 10.92752074 | 9.24931731 | 9.650759119 | 8.129932547 | 9.729966413 | 9.026281524 | 10.18131206 | 8.251949598 |
| ENSMUSG00000021123 | Rdh12 | 3138 | 2.871605264 | 2.569063225 | 2.358556451 | 1.684219612 | 2.162715499 | 2.100227395 | 1.884666943 | 2.994050667 | 4.410028456 | 3.485966589 | 4.171554692 | 3.059367102 | 9.463930593 | 6.615840364 | 6.144782714 | 8.138889464 | 5.447268457 | 6.976935779 |
| ENSMUSG00000021124 | Vti1b | 2004 | 80.47137617 | 69.68445737 | 74.90172889 | 68.02327577 | 66.67376253 | 71.73277451 | 93.42129267 | 68.55193186 | 71.97364182 | 82.67842877 | 74.02257788 | 67.31859105 | 83.84456599 | 91.03411939 | 79.15468572 | 83.05851815 | 76.86296769 | 79.52164779 |
| ENSMUSG00000021125 | Arg2 | 1420 | 5.028781389 | 3.988163392 | 3.849442909 | 3.850229099 | 4.270860832 | 5.02506594 | 4.531355165 | 5.083354968 | 4.930780056 | 3.453290943 | 4.373763804 | 3.576919222 | 4.585783859 | 9.942959554 | 5.68697532 | 1.159504309 | 2.429131494 | 3.292798642 |
| ENSMUSG00000021127 | Zfp36l1 | 4734 | 13.25256439 | 14.11611022 | 16.69673608 | 14.99453043 | 18.42311907 | 18.41217963 | 13.88198374 | 17.5149357 | 19.24473445 | 18.62522499 | 18.36722105 | 22.74364451 | 14.65160027 | 15.01044649 | 19.21694876 | 19.48501758 | 17.8677941 | 26.9584131 |
| ENSMUSG00000021130 | Galntl1 | 4082 | 0 | 0 | 0 | 0.122775965 | 0 | 0.024278654 | 0.092724505 | 0.168413337 | 0.010089788 | 0.069305296 | 0.023407623 | 0.150409674 | 0 | 0.284444435 | 0 | 0 | 0 | 0 |
| ENSMUSG00000021131 | Erh | 1515 | 15.62731554 | 14.68845765 | 13.88942951 | 11.87895215 | 14.36015148 | 15.66716791 | 16.48917127 | 12.93247685 | 16.55616376 | 14.40975288 | 14.97894738 | 16.6157517 | 13.74129973 | 14.34708442 | 15.66474996 | 15.41734027 | 14.7233695 | 15.10481082 |
| ENSMUSG00000021133 | 4933426M11R | 5030 | 16.48660009 | 20.4579583 | 22.02293927 | 21.47619008 | 22.6306162 | 23.67300572 | 16.33839429 | 21.95873315 | 23.81939848 | 24.78458438 | 21.90239647 | 21.5273132 | 17.48684066 | 16.26928071 | 20.24855789 | 21.99997125 | 24.48158886 | 23.53471127 |
| ENSMUSG00000021134 | Srsf5 | 3600 | 322.8150049 | 362.4257819 | 359.5228273 | 358.1730877 | 354.3311781 | 352.622758 | 317.0343761 | 387.1966974 | 376.2388215 | 332.3076786 | 370.8531152 | 378.9108272 | 297.6907141 | 329.3401841 | 350.2132251 | 327.0762412 | 367.7621112 | 375.1773882 |

| ENSMUSG00000021135 | Slc10a1 | 1574 | 0.108018236 | 0.042328963 | 0.430260118 | 0.318406282 | 0.152896835 | 0.094446125 | 0.661295385 | 0.679407413 | 1.360672694 | 0.808811301 | 0.242820628 | 0.39007134 | 1.347695326 | 1.268802896 | 0.654408448 | 0.535192734 | 1.485326627 | 1.293098443 |
| --- | --- | --- | --- | --- | --- | --- | --- | --- | --- | --- | --- | --- | --- | --- | --- | --- | --- | --- | --- | --- |
| ENSMUSG00000021136 | Smoc1 | 3813 | 0.011147437 | 0.305783188 | 0.342534833 | 0.59744354 | 0.441808898 | 0.168944541 | 0.359839415 | 0.020032731 | 0.270040098 | 0.185486638 | 0.125294934 | 0.409871113 | 0.828020641 | 0.791729788 | 0.583499611 | 0.612569381 | 0.412110719 | 0.779043211 |
| ENSMUSG00000021139 | Gm20498 | 3233 | 0.026294572 | 0.216384404 | 0.074812141 | 0.22547996 | 0.238203148 | 0.122617343 | 0.087805776 | 0.082693108 | 0.216569987 | 0.175010343 | 0.251213824 | 0.103586135 | 0.198365272 | 0.129290686 | 0.178416883 | 0.130280446 | 0.17782074 | 0.05104464 |
| ENSMUSG00000021140 | Pcnx | 12132 | 2.883428938 | 3.558647454 | 2.787100043 | 2.839115522 | 2.709701934 | 2.810112158 | 1.665225346 | 2.417718847 | 2.315298694 | 2.429046286 | 2.965260776 | 3.146877119 | 1.776957455 | 2.381159113 | 2.244825952 | 2.827924846 | 2.653650626 | 2.067602429 |
| ENSMUSG00000021143 | Pacs2 | 5832 | 3.687863352 | 4.335474402 | 4.437558083 | 4.781097245 | 4.745517165 | 4.681679733 | 3.496530284 | 4.990159574 | 4.653963287 | 5.25513668 | 4.390839805 | 4.077070004 | 3.62038076 | 3.249174838 | 5.001834974 | 2.764119636 | 3.71959603 | 4.395446456 |
| ENSMUSG00000021144 | Mta1 | 3000 | 8.359351285 | 10.69343904 | 10.0455698 | 12.77227943 | 10.20396782 | 8.506552598 | 8.311260598 | 9.980947829 | 9.665102366 | 9.272971641 | 8.949842222 | 8.893295572 | 9.405952227 | 9.041115678 | 9.72356719 | 7.849574517 | 7.818564568 | 9.534911832 |
| ENSMUSG00000021147 | Wdr37 | 11312 | 2.186882289 | 2.588581508 | 1.988480518 | 2.726735061 | 2.208315805 | 2.527574901 | 3.358564461 | 2.211458944 | 2.508619993 | 2.204978962 | 2.71563811 | 2.190784493 | 1.700799835 | 1.983070563 | 2.469470906 | 2.355928461 | 2.703711539 | 2.728086004 |
| ENSMUSG00000021148 | Gm9745 | 735 | 0 | 0 | 0 | 0 | 0 | 0 | 0 | 0 | 0 | 0 | 0 | 0 | 0 | 0 | 0 | 0 | 0 | 0 |
| ENSMUSG00000021149 | Gtpbp4 | 2960 | 22.12853928 | 21.0231373 | 20.62412004 | 19.56354302 | 19.38285575 | 18.13027381 | 22.95304289 | 22.7993159 | 20.52368606 | 17.26735214 | 20.40119849 | 18.87549941 | 19.4661079 | 22.42182462 | 19.27843071 | 17.58004576 | 21.027671 | 20.53549423 |
| ENSMUSG00000021156 | Zmynd11 | 8077 | 22.21825594 | 22.31720572 | 20.66221118 | 22.24182219 | 22.7460137 | 23.14137812 | 16.78232328 | 18.171772 | 16.79177868 | 17.71143168 | 16.88714949 | 19.15575296 | 16.94887587 | 17.9922681 | 17.51206406 | 15.80074217 | 17.0349642 | 16.48842979 |
| ENSMUSG00000021171 | Esyt2 | 5517 | 17.60455451 | 18.57962217 | 19.68435303 | 22.528644 | 20.06587356 | 21.49350942 | 21.58528419 | 19.66039928 | 20.36556371 | 16.91340253 | 17.04207328 | 22.96566142 | 18.84037515 | 19.63161352 | 22.44913486 | 21.13375014 | 28.44079382 | 22.90301217 |
| ENSMUSG00000021175 | Cdca7l | 3245 | 0.196480012 | 0.492763921 | 0.134163875 | 0.014040383 | 0.059330568 | 0.244327808 | 0.072900892 | 0.282470773 | 0.36807672 | 0 | 0 | 0.103203074 | 0.288846357 | 0.229000123 | 0.025393871 | 0.153398427 | 0.082676141 | 0.084759795 |
| ENSMUSG00000021176 | 2610021K21R | 2072 | 0.205140811 | 0.305475381 | 0.116731492 | 0.065966763 | 0.185837543 | 0 | 0.159840132 | 0.497680919 | 0.079510648 | 0.091024524 | 0 | 0.296318672 | 0.59522101 | 0 | 0.278388891 | 0.221760275 | 0.22196697 | 0 |
| ENSMUSG00000021177 | Tdp1 | 5464 | 1.968120339 | 1.499811855 | 1.478473567 | 2.284722941 | 2.078904463 | 1.850065456 | 1.922294056 | 2.201794795 | 1.545248121 | 2.347179957 | 1.906101931 | 1.777438743 | 1.688336115 | 1.861503503 | 1.892677622 | 2.578869605 | 1.886854661 | 1.349052032 |
| ENSMUSG00000021178 | Psmc1 | 1507 | 143.8463157 | 139.7726809 | 138.7328455 | 139.4947967 | 145.4497547 | 137.0509579 | 124.3880782 | 130.6195368 | 137.0879649 | 132.7228157 | 126.5545018 | 130.2982754 | 137.2586126 | 134.8943401 | 141.4577639 | 127.6268885 | 136.4945159 | 128.5979316 |
| ENSMUSG00000021179 | BC002230 | 3709 | 2.716022302 | 4.409984109 | 4.225673722 | 3.120114657 | 3.322127921 | 2.618586083 | 3.138020765 | 3.871756081 | 3.764418669 | 4.678204044 | 4.44388263 | 4.665099866 | 3.857173365 | 2.366657459 | 3.376994605 | 4.686960302 | 4.019662846 | 4.953637543 |
| ENSMUSG00000021180 | Rps6ka5 | 4388 | 2.65415183 | 4.069214061 | 3.560768073 | 3.675617524 | 3.56492142 | 3.410420588 | 3.644413281 | 2.376145364 | 4.092370299 | 3.578209705 | 3.233628685 | 2.060651171 | 2.259734588 | 1.323042599 | 2.441295472 | 2.321171734 | 2.008898881 | 2.319208009 |
| ENSMUSG00000021182 | Ccdc88c | 9317 | 0.994539913 | 1.440924801 | 1.230495513 | 1.305656202 | 1.539477649 | 1.893396281 | 0.827730671 | 1.20926894 | 0.959265206 | 1.801615365 | 1.338334675 | 1.198146669 | 0.275331083 | 0.618124593 | 0.588151436 | 0.731536773 | 0.575902283 | 1.334341324 |
| ENSMUSG00000021185 | 9030617O03R | 4645 | 5.975431635 | 7.429958754 | 5.217467967 | 6.934695015 | 7.761207928 | 8.107659315 | 6.091061787 | 5.147135394 | 5.860987496 | 8.628223416 | 8.125342777 | 6.596942682 | 6.786457511 | 3.299579944 | 6.040529507 | 3.701287992 | 6.411094229 | 4.204140557 |
| ENSMUSG00000021186 | Fbln5 | 5818 | 0.138810303 | 0 | 0.049886762 | 0.078310492 | 0 | 0.07665428 | 0 | 0 | 0 | 0.024312841 | 0 | 0 | 0.05087513 | 0 | 0 | 0 | 0.013175076 | 0 |
| ENSMUSG00000021187 | Tc2n | 6457 | 0.296226254 | 0.45916797 | 0.412040293 | 0.324579222 | 0.365256971 | 0.191856642 | 0.175856326 | 0.212935804 | 0.172221763 | 0.160649756 | 0 | 0.112374727 | 0.542444808 | 0.575426203 | 0.599805831 | 0.266854163 | 0.552012639 | 0.511157876 |
| ENSMUSG00000021188 | Trip11 | 10354 | 8.148809582 | 8.963658808 | 7.461128837 | 8.008605437 | 8.530237583 | 7.954089551 | 8.252530244 | 7.362568592 | 6.806077696 | 7.422797596 | 6.759736737 | 6.986391608 | 8.923959925 | 6.661142532 | 6.665308684 | 7.803088818 | 8.376705611 | 6.582604137 |
| ENSMUSG00000021189 | Atxn3 | 5519 | 0.007701608 | 0 | 0 | 0 | 0 | 0 | 0 | 0.013840334 | 0 | 0 | 0 | 0 | 0 | 0 | 0.007465402 | 0 | 0 | 0 |
| ENSMUSG00000021190 | Lgmn | 2823 | 26.01804611 | 23.27064378 | 25.1206502 | 22.69175641 | 23.44363976 | 29.0505753 | 31.24011106 | 34.22840173 | 36.83880705 | 35.50917291 | 32.1545948 | 36.04390822 | 54.3122915 | 43.68014595 | 49.33094542 | 56.4118442 | 48.33213495 | 49.72838837 |
| ENSMUSG00000021192 | Golga5 | 2654 | 27.59473184 | 29.58496295 | 26.8296295 | 35.12354812 | 29.63359581 | 27.91308843 | 35.67169199 | 27.67294266 | 29.96652673 | 34.07499957 | 25.32756842 | 28.68596977 | 21.52462706 | 34.61431984 | 34.8055344 | 31.56738847 | 37.05549279 | 27.5460071 |
| ENSMUSG00000021193 | Pitrm1 | 3497 | 13.58901538 | 14.14631053 | 14.49684295 | 16.01215999 | 17.08084566 | 15.92715829 | 12.17655449 | 12.16652453 | 11.33011958 | 14.54835825 | 10.27359702 | 13.55090007 | 11.18679165 | 10.93036429 | 11.8408818 | 11.63938109 | 13.69970578 | 14.25171595 |
| ENSMUSG00000021194 | Chga | 1874 | 0.022681524 | 0.053329073 | 0 | 0.02431219 | 0 | 0.026442227 | 0 | 0.040760301 | 0.483512992 | 0.025160461 | 0 | 0 | 5.844015814 | 1.833971434 | 7.54115991 | 0.408651093 | 0.470386782 | 0.645784604 |
| ENSMUSG00000021196 | Pfkp | 7118 | 53.00905417 | 60.18130188 | 53.2462223 | 61.5951011 | 57.65958319 | 54.49547604 | 20.97763623 | 28.19090787 | 21.28182143 | 22.42937359 | 25.47147618 | 24.37916479 | 17.0908272 | 12.82138687 | 13.5737073 | 15.11612908 | 13.3318223 | 14.48258865 |
| ENSMUSG00000021198 | Unc79 | 8938 | 0.152177851 | 0.216172283 | 0.286842371 | 0.183508346 | 0.210018463 | 0.155233446 | 0.01058686 | 0.115371992 | 0 | 0.105506161 | 0.026725754 | 0.187342792 | 0.066232156 | 0 | 0.046097064 | 0.154224868 | 0.047168188 | 0.135399457 |
| ENSMUSG00000021200 | Asb2 | 3479 | 0 | 0 | 0 | 0 | 0 | 0 | 0.054398021 | 0.021955966 | 0 | 0 | 0 | 0 | 0 | 0 | 0 | 0 | 0 | 0 |
| ENSMUSG00000021203 | Otub2 | 3858 | 1.344124281 | 1.459274008 | 1.379234925 | 1.29904481 | 1.384822065 | 1.20735017 | 1.802738155 | 0.791962725 | 0.715266085 | 2.18765575 | 2.489053047 | 0.62210303 | 1.739020412 | 0.999185913 | 1.078630675 | 1.647548167 | 1.639149557 | 1.91063253 |
| ENSMUSG00000021206 | D830046C22R | 1039 | 0 | 0 | 0 | 0 | 0 | 0 | 0 | 0 | 0 | 0 | 0 | 0 | 0 | 0 | 0 | 0.110559983 | 0 | 0 |
| ENSMUSG00000021207 | Akr1c21 | 1409 | 0.12066764 | 0 | 0 | 0.032335731 | 0 | 0.035168725 | 0.033578906 | 0.216848275 | 0 | 0 | 0 | 0 | 0.10503602 | 0 | 0 | 0.108702931 | 0 | 0.078082479 |
| ENSMUSG00000021208 | Ifi27l2b | 1300 | 0 | 0 | 0 | 0 | 0 | 0 | 0 | 0 | 0 | 0 | 0 | 0 | 0 | 0 | 0 | 0 | 0 | 0.084629395 |
| ENSMUSG00000021209 | Ppp4r4 | 3740 | 0.022730041 | 0.071257528 | 0.194011485 | 0.073092584 | 0.15443398 | 0.13249394 | 0.240358529 | 0 | 0 | 0.075642839 | 0.051096212 | 0.328327427 | 0.079142113 | 0.447055387 | 0.165246882 | 0.092143173 | 0.327925535 | 0.367707933 |
| ENSMUSG00000021210 | Akr1c6 | 1881 | 0 | 0 | 0 | 0 | 0 | 0 | 0.025152939 | 0.020304307 | 0 | 0 | 0 | 0 | 0 | 0 | 0 | 0 | 0.02037549 | 0 |
| ENSMUSG00000021211 | Akr1c12 | 1256 | 0.812200816 | 1.432242267 | 0.693251231 | 0.79804377 | 0.613145524 | 0.946867521 | 9.266655268 | 4.926090119 | 13.54301832 | 4.016819465 | 2.206168465 | 5.777098835 | 14.88598459 | 20.55964378 | 9.808332175 | 7.01181505 | 6.835263142 | 13.13911785 |
| ENSMUSG00000021213 | Akr1c13 | 1512 | 10.73874156 | 10.75178067 | 9.182012706 | 11.96278743 | 9.772817835 | 9.668026723 | 31.72953468 | 24.29966344 | 32.08843622 | 26.38194111 | 23.00275779 | 22.2229027 | 80.06648479 | 76.6081706 | 58.66861761 | 51.0541432 | 47.24882105 | 54.42699977 |
| ENSMUSG00000021214 | Akr1c18 | 1198 | 0 | 0.083421271 | 0 | 0 | 0 | 0 | 0 | 0.031880136 | 0 | 0 | 0 | 0 | 0 | 0 | 0 | 0 | 0 | 0 |
| ENSMUSG00000021215 | Net1 | 3721 | 18.67668982 | 16.41024862 | 18.39520168 | 18.53787184 | 18.92421508 | 16.37996837 | 17.49590062 | 11.45464152 | 12.51866416 | 16.0294651 | 14.8036891 | 12.42014744 | 23.47939411 | 19.55868985 | 21.38140404 | 17.73037263 | 24.00929806 | 26.71364038 |
| ENSMUSG00000021216 | Tubal3 | 3091 | 0 | 0 | 0 | 0 | 0 | 0 | 0 | 0 | 0 | 0 | 0 | 0.126402342 | 0 | 0 | 0 | 0 | 0 | 0 |

| ENSMUSG00000021217 | Tshz3 | 5517 | 0.385220011 | 0.326064217 | 0.359491525 | 0.074324705 | 0.165761564 | 0.278436603 | 0.137212772 | 0.332288439 | 0.141842269 | 0.034185755 | 0.467617864 | 0.68795814 | 0.143068819 | 0.159948824 | 0.17923461 | 0.249857145 | 0.180620576 | 0.099708368 |
| --- | --- | --- | --- | --- | --- | --- | --- | --- | --- | --- | --- | --- | --- | --- | --- | --- | --- | --- | --- | --- |
| ENSMUSG00000021218 | Gdi2 | 2864 | 107.4205531 | 107.7202211 | 113.4007971 | 102.3373597 | 104.1289801 | 102.0467956 | 113.3092405 | 104.4423518 | 106.0437735 | 108.7231998 | 100.9212635 | 101.2828207 | 116.2331626 | 108.796572 | 107.1326769 | 109.5640809 | 113.3730403 | 99.47352085 |
| ENSMUSG00000021219 | Rgs6 | 3373 | 1.549996043 | 1.195037118 | 1.6349192 | 1.755984513 | 2.725525473 | 2.100812601 | 2.384570239 | 2.955297073 | 3.235821725 | 3.606546528 | 2.889443086 | 2.796574852 | 9.901484731 | 8.578317949 | 9.454492813 | 12.44189318 | 9.328754746 | 12.13364228 |
| ENSMUSG00000021221 | Dpf3 | 3938 | 0 | 0 | 0 | 0 | 0.024444857 | 0 | 0.012014393 | 0.07758741 | 0 | 0 | 0 | 0.014173607 | 0 | 0.117938262 | 0 | 0 | 0 | 0.041906379 |
| ENSMUSG00000021222 | Dcaf4 | 2067 | 9.315357881 | 8.316136148 | 10.6482614 | 11.9468244 | 10.501934 | 11.24346011 | 13.23015405 | 10.10703634 | 10.8396055 | 11.26872153 | 13.4518751 | 10.47725065 | 10.62056274 | 13.93097928 | 14.31190959 | 11.35565667 | 12.01521064 | 13.06699149 |
| ENSMUSG00000021223 | Papln | 5231 | 4.265956302 | 7.126195494 | 4.688468723 | 7.098499548 | 6.578919025 | 7.199403082 | 5.951394138 | 7.257359585 | 5.668952657 | 8.355706733 | 6.292668016 | 6.017976238 | 14.47610266 | 13.08709325 | 13.21663368 | 20.700791 | 15.31293453 | 13.02930285 |
| ENSMUSG00000021224 | Numb | 5928 | 9.787376059 | 9.412803275 | 8.576346222 | 9.737827796 | 9.418527573 | 8.47612548 | 8.843193025 | 9.122881546 | 7.316025826 | 8.661794171 | 9.638807412 | 7.786699217 | 13.27334819 | 12.81756528 | 9.202236842 | 14.87572012 | 13.42842758 | 12.33250723 |
| ENSMUSG00000021226 | Acot2 | 2193 | 4.399760583 | 3.949545142 | 3.352839313 | 3.490312562 | 4.148168524 | 4.225426894 | 3.818670392 | 3.657275196 | 3.568371179 | 3.375586143 | 3.158854984 | 3.257822531 | 2.834391964 | 2.964966819 | 2.141804547 | 2.217467916 | 3.932246604 | 1.780960592 |
| ENSMUSG00000021228 | Acot3 | 1923 | 0.088414303 | 0.155910581 | 0.704346776 | 0 | 0.17520721 | 0.128842261 | 0 | 0.496521092 | 0.08567138 | 0.147116079 | 0.322971638 | 0 | 0.436111593 | 0.79701242 | 0.899877976 | 0.657093107 | 1.255619711 | 0.02860588 |
| ENSMUSG00000021234 | Fam161b | 5742 | 0.932715461 | 1.02108488 | 0.497046028 | 0.682384154 | 0.77118373 | 0.552311903 | 0.552063652 | 0.651838286 | 0.308432633 | 0.993597195 | 1.838778444 | 0.709603513 | 0.807593212 | 0.913998933 | 0.337247149 | 0.693525456 | 0.720870788 | 0.105381431 |
| ENSMUSG00000021235 | Coq6 | 2895 | 11.08511499 | 8.975494816 | 10.34307955 | 10.48140087 | 10.840074 | 9.037597005 | 10.03453709 | 9.511821117 | 13.28780854 | 10.66794841 | 11.22175191 | 8.251849253 | 12.03051247 | 9.754060495 | 12.32488684 | 12.7635358 | 11.26621025 | 12.02789795 |
| ENSMUSG00000021236 | Entpd5 | 9526 | 6.313754365 | 8.452368815 | 7.815123159 | 7.853373378 | 7.715457426 | 7.469843105 | 8.93010673 | 9.638309404 | 7.0301569 | 8.939131858 | 8.731493041 | 8.853397666 | 7.327804214 | 5.060777106 | 8.321624347 | 8.622018988 | 8.477168547 | 6.790962577 |
| ENSMUSG00000021238 | Aldh6a1 | 5304 | 29.95557089 | 36.39044289 | 40.94056896 | 37.40919081 | 34.91923874 | 37.36074313 | 62.70000381 | 63.4163816 | 61.89625135 | 64.28994833 | 56.87237704 | 58.30968823 | 57.49809815 | 63.36150384 | 65.29794095 | 47.95710878 | 66.62301234 | 65.91966107 |
| ENSMUSG00000021239 | Vsx2 | 3798 | 0 | 0 | 0 | 0 | 0 | 0 | 0 | 0 | 0 | 0 | 0 | 0 | 0 | 0 | 0 | 0.010081782 | 0 | 0 |
| ENSMUSG00000021240 | Abcd4 | 3196 | 4.82771555 | 3.877470791 | 4.041217961 | 3.90604698 | 3.328271317 | 4.217253922 | 5.655019822 | 3.728419759 | 4.08514565 | 4.16035617 | 5.067494031 | 4.750269156 | 3.256894413 | 3.458602252 | 4.473385449 | 4.576662086 | 3.405715995 | 3.769397488 |
| ENSMUSG00000021241 | Isca2 | 864 | 115.2657725 | 107.341548 | 118.8062864 | 108.260213 | 122.6138472 | 104.2672102 | 133.1764296 | 112.0133654 | 131.3773581 | 117.9859033 | 134.3670707 | 101.8765045 | 142.6857191 | 133.4729965 | 121.2680054 | 131.2251024 | 123.2296907 | 141.0245039 |
| ENSMUSG00000021242 | Npc2 | 3257 | 95.24187125 | 91.32662956 | 89.95961724 | 84.71528538 | 90.44131804 | 93.64356008 | 178.0798683 | 157.6242779 | 143.5521421 | 127.7281716 | 101.5786268 | 133.8753319 | 171.8210837 | 188.9562794 | 133.1428848 | 120.7029985 | 121.3803354 | 155.7212048 |
| ENSMUSG00000021243 | Fcf1 | 1108 | 56.43060825 | 51.53276236 | 54.13644191 | 48.7272902 | 54.86518014 | 49.95523773 | 50.85685975 | 55.08254426 | 47.2455429 | 50.46997659 | 58.51138868 | 52.69240349 | 52.98283534 | 52.22863979 | 51.46475934 | 65.93725534 | 57.69698848 | 53.51969048 |
| ENSMUSG00000021244 | Ylpm1 | 10173 | 4.249264133 | 4.083473812 | 4.232030081 | 3.578420763 | 3.524848435 | 3.955256037 | 4.501983014 | 4.29115462 | 4.55873555 | 3.666195446 | 3.470529275 | 3.341368182 | 4.107356132 | 3.592990934 | 3.017316341 | 3.824167612 | 3.726010777 | 3.14167807 |
| ENSMUSG00000021245 | Mlh3 | 5877 | 5.771504249 | 6.597959651 | 7.383198354 | 5.736799865 | 5.618257552 | 7.209050061 | 7.406442847 | 6.745569797 | 6.56657568 | 7.196559609 | 8.055978195 | 5.707880418 | 7.571446211 | 7.167736475 | 5.966058204 | 5.870314322 | 8.12567057 | 5.260356981 |
| ENSMUSG00000021248 | Tmed10 | 3521 | 61.73572002 | 59.56773133 | 58.19661306 | 58.59142538 | 60.72195539 | 62.07813343 | 67.40141934 | 70.98298249 | 68.73387294 | 71.21483667 | 70.85510741 | 68.51328989 | 77.49356275 | 82.77098789 | 73.35772618 | 80.64843638 | 69.62084493 | 72.39767127 |
| ENSMUSG00000021250 | Fos | 2348 | 205.3028966 | 84.01999981 | 88.25903068 | 141.6506063 | 151.7754527 | 112.6543832 | 358.996034 | 230.1303702 | 204.9327358 | 289.531022 | 347.1619853 | 154.4677068 | 690.6468419 | 896.8970419 | 744.5759879 | 761.164438 | 727.1060032 | 980.8161214 |
| ENSMUSG00000021252 | 0610007P14R | 2245 | 32.1108145 | 27.92644406 | 31.2003884 | 27.51927667 | 28.34316396 | 27.14915915 | 33.02403914 | 30.23069001 | 30.85778152 | 28.96250325 | 26.51563397 | 27.39815598 | 32.89526956 | 36.86564086 | 34.9616766 | 32.16752141 | 32.9315932 | 34.84318475 |
| ENSMUSG00000021253 | Tgfb3 | 3377 | 70.93845783 | 76.90474484 | 73.5844908 | 88.16743403 | 77.59259474 | 84.62262794 | 55.15842965 | 62.85856457 | 65.50570807 | 59.828476 | 68.67031343 | 87.7646338 | 35.95079906 | 37.21578338 | 52.57255088 | 49.87870357 | 60.65020155 | 51.93042115 |
| ENSMUSG00000021254 | 1700020O03R | 4018 | 4.792146526 | 5.156948782 | 4.189643744 | 6.055151238 | 5.174960434 | 5.24139168 | 4.462793757 | 5.322982914 | 3.310912044 | 5.327630483 | 5.517068376 | 4.473032193 | 5.046146839 | 4.981931723 | 5.732122883 | 5.746450041 | 5.971195087 | 6.475686282 |
| ENSMUSG00000021255 | Esrrb | 6037 | 0.028163111 | 0.060699327 | 0.008012843 | 0.128298452 | 0.039864108 | 0.196996125 | 0.141068117 | 0.088569427 | 0.341117407 | 0.234308613 | 0.395684599 | 0.036982384 | 0.441266114 | 0.115398594 | 0.109197432 | 0.025370619 | 0.0571371 | 0.109343926 |
| ENSMUSG00000021256 | Vash1 | 5074 | 0.167541096 | 0.052523286 | 0.066735261 | 0.01795863 | 0.161261865 | 0.078128078 | 0.018649065 | 0 | 0.056820183 | 0 | 0 | 0 | 0.058334944 | 0 | 0.040600666 | 0 | 0 | 0 |
| ENSMUSG00000021257 | Angel1 | 4695 | 1.430419129 | 1.915757495 | 1.90609225 | 1.853495094 | 1.814558145 | 1.720361144 | 1.662745905 | 2.131290613 | 1.298318282 | 1.847865687 | 3.042537292 | 1.783248006 | 1.376460312 | 0.999116683 | 1.597163615 | 2.332505584 | 1.199992683 | 0.878739724 |
| ENSMUSG00000021258 | Ccnk | 2626 | 1.602441899 | 2.004355402 | 1.989467357 | 2.047297499 | 2.309452545 | 1.754914022 | 1.675582308 | 2.385207157 | 2.242830066 | 2.765121212 | 1.237127638 | 1.317810769 | 2.573675806 | 2.493760976 | 2.416237474 | 2.347596265 | 2.305999585 | 2.953649676 |
| ENSMUSG00000021259 | Cyp46a1 | 2104 | 0 | 0.20583062 | 0.068973665 | 0.389780798 | 0.274516675 | 0.023551679 | 0.112435073 | 0.14521826 | 0.117452041 | 0.08964012 | 0.158947105 | 0.265283567 | 0.445487846 | 0.684299767 | 0.097912443 | 0 | 0.054647762 | 0.261450127 |
| ENSMUSG00000021260 | Hhipl1 | 3093 | 0 | 0.010770415 | 0.03127936 | 0.014730373 | 0.062246264 | 0 | 0.030593391 | 0 | 0 | 0 | 0 | 0.108274806 | 0 | 0.060063482 | 0 | 0 | 0 | 0 |
| ENSMUSG00000021262 | Evl | 2678 | 8.60261591 | 9.87693727 | 8.543943188 | 12.67474908 | 10.49628208 | 9.344335496 | 7.932558939 | 11.25235455 | 7.551373876 | 10.21187748 | 8.88421558 | 8.607867307 | 7.957949314 | 10.66583784 | 9.569592145 | 10.00874727 | 8.400872398 | 12.65332701 |
| ENSMUSG00000021263 | Degs2 | 3570 | 3.274208237 | 2.78074019 | 3.143601973 | 2.322719907 | 2.777360301 | 3.039789539 | 3.31321281 | 2.93129363 | 3.172630766 | 3.618849494 | 2.890585723 | 4.627853256 | 3.744803804 | 2.692976496 | 3.035296694 | 2.87447697 | 3.145547617 | 4.715066291 |
| ENSMUSG00000021264 | Yy1 | 3289 | 10.58429284 | 9.014434739 | 9.383494183 | 9.447440631 | 9.907361606 | 9.084918926 | 9.551723566 | 8.65106105 | 10.80692098 | 9.662381878 | 10.70542548 | 11.93019483 | 12.17923893 | 10.61901907 | 11.61260028 | 14.13341362 | 9.753454092 | 8.730542326 |
| ENSMUSG00000021265 | Slc25a29 | 2866 | 8.423914858 | 8.206177013 | 7.932853896 | 8.361866476 | 5.2397628 | 10.01082789 | 11.48975036 | 9.541437586 | 13.00551178 | 9.788788708 | 10.96857037 | 13.5352008 | 18.77917716 | 21.99044891 | 16.07234455 | 13.86798691 | 11.59417286 | 14.41445888 |
| ENSMUSG00000021266 | Wars | 3891 | 52.00903189 | 57.40502589 | 50.07673613 | 53.17211574 | 56.12298578 | 53.36056379 | 60.99213506 | 60.48357328 | 56.74657183 | 55.19698107 | 62.10376253 | 59.71745139 | 79.37988539 | 95.62158419 | 74.83202145 | 73.06804419 | 75.96309476 | 80.90905109 |
| ENSMUSG00000021268 | Meg3 | 16216 | 0.002621187 | 0 | 0 | 0 | 0 | 0 | 0 | 0 | 0 | 0 | 0 | 0 | 0 | 0 | 0 | 0 | 0 | 0 |
| ENSMUSG00000021270 | Hsp90aa1 | 4440 | 57.61174535 | 52.24272127 | 48.16652651 | 48.14694145 | 50.12657996 | 49.78710462 | 43.33798766 | 46.78569295 | 51.99333803 | 44.80378762 | 45.41849093 | 50.14609866 | 62.29821175 | 70.52388225 | 67.23091721 | 63.54171066 | 63.66999233 | 78.32602989 |
| ENSMUSG00000021271 | Zfp839 | 2821 | 4.565426564 | 5.006971694 | 4.475537548 | 4.506037352 | 5.238036358 | 5.23456739 | 6.507380157 | 4.061581255 | 3.547792744 | 6.117390072 | 6.723381253 | 4.768371033 | 4.809031284 | 5.301310223 | 3.738957217 | 3.583381905 | 5.217049986 | 5.557460268 |
| ENSMUSG00000021273 | Fdft1 | 2852 | 54.39827928 | 48.60271839 | 47.77988605 | 54.92246509 | 49.27952914 | 54.7304035 | 42.45201451 | 40.45555669 | 42.15408117 | 44.72042507 | 44.10640451 | 36.92992823 | 52.92975697 | 61.40976632 | 51.05410186 | 59.31553419 | 57.11317035 | 60.23612914 |
| ENSMUSG00000021275 | Tecpr2 | 7809 | 1.039632299 | 1.467490794 | 0.99113393 | 1.277739622 | 1.411475287 | 0.939147723 | 0.527110138 | 1.340084295 | 1.002105006 | 1.587992695 | 0.966633812 | 1.207945573 | 1.44034795 | 0.802913535 | 0.601482568 | 0.946355132 | 1.560732797 | 1.634282592 |

| ENSMUSG00000021276 | Cinp |  | 3139 | 15.49089563 | 13.40369079 | 14.53209274 | 13.73072569 | 13.07949375 | 11.52389153 | 14.87658939 | 13.29859695 | 15.18088523 | 13.56390094 | 11.67358813 | 13.83389152 | 14.78857412 | 12.51726599 | 12.95506074 | 13.60115554 | 13.55278419 | 12.3547054 |
| --- | --- | --- | --- | --- | --- | --- | --- | --- | --- | --- | --- | --- | --- | --- | --- | --- | --- | --- | --- | --- | --- |
| ENSMUSG00000021277 | Traf3 |  | 7546 | 8.229533816 | 8.639455869 | 8.724671987 | 9.370625604 | 9.325321018 | 10.29667191 | 5.498703872 | 5.871082267 | 6.784367757 | 5.779803938 | 4.900320416 | 5.983947924 | 5.00771914 | 4.419143221 | 5.689374667 | 6.210933403 | 5.007915286 | 5.452797751 |
| ENSMUSG00000021278 | Amn |  | 1673 | 0.076219682 | 0.29868106 | 0.231313953 | 0.517429673 | 0.287698289 | 0.385048139 | 0 | 0 | 0 | 0 | 0 | 0.166813098 | 0.088461298 | 0.138804804 | 0.147764098 | 0.068662177 | 0 | 0 |
| ENSMUSG00000021279 | Cdc42bpb |  | 6681 | 4.536175798 | 5.056020766 | 5.698244034 | 4.187169768 | 4.351396781 | 4.242503159 | 4.029473778 | 4.007315385 | 3.409091935 | 4.573215942 | 3.189287754 | 3.959979654 | 2.702511858 | 3.475833512 | 3.188325759 | 3.106347734 | 4.032837408 | 3.540475362 |
| ENSMUSG00000021280 | 1200009I06Ri | k | 4578 | 17.84511759 | 16.60550996 | 17.8785525 | 22.67104827 | 19.07193306 | 19.12618616 | 21.8787552 | 22.52500797 | 20.32335935 | 26.47978551 | 22.34298517 | 18.60522086 | 18.96552521 | 21.6495593 | 24.90273157 | 24.53175 | 22.96396514 | 23.22686835 |
| ENSMUSG00000021281 | Tnfaip2 |  | 4997 | 0 | 0.106665261 | 0.251693374 | 0.118529833 | 0 | 0.386743368 | 0.037872867 | 0.14521826 | 0.098906982 | 0 | 0.019121456 | 0 | 0 | 0.037177576 | 0.008245258 | 0.007662719 | 0.007669861 | 0 |
| ENSMUSG00000021282 | Eif5 |  | 4117 | 77.95884969 | 71.02758937 | 73.07140765 | 74.1349128 | 74.60038983 | 69.97804057 | 83.11034588 | 86.61706613 | 80.30220112 | 81.966865 | 84.2008984 | 80.28670241 | 87.06478283 | 99.03634763 | 89.25836215 | 86.26314881 | 97.15162363 | 94.30514337 |
| ENSMUSG00000021285 | Ppp1r13b |  | 3826 | 12.75377472 | 13.08658652 | 11.49282255 | 11.93208741 | 14.51757447 | 12.87386753 | 11.97037982 | 17.88833903 | 16.15811818 | 13.56845904 | 12.16226073 | 16.77679351 | 11.17897864 | 19.31326269 | 14.11794035 | 10.21816784 | 19.05295784 | 16.66376233 |
| ENSMUSG00000021286 | Zfyve21 |  | 1300 | 75.07068012 | 67.72767648 | 74.2719743 | 68.62194216 | 76.27058673 | 72.46134349 | 133.9312757 | 122.950157 | 136.5491407 | 114.5036694 | 120.833895 | 120.1324799 | 125.9861272 | 117.4320887 | 122.558782 | 104.3566323 | 93.86994565 | 118.608097 |
| ENSMUSG00000021287 | Xrcc3 |  | 4182 | 1.778671881 | 1.760437499 | 1.781330386 | 1.579711006 | 1.772433344 | 2.381659361 | 1.368922561 | 1.835646314 | 2.511372912 | 2.322583661 | 2.364757629 | 1.721716995 | 1.380161244 | 1.810231048 | 1.960571407 | 1.776273992 | 1.832917116 | 1.854682938 |
| ENSMUSG00000021288 | Klc1 |  | 4138 | 18.98249524 | 17.12337506 | 16.06216305 | 17.55058112 | 15.79583189 | 19.42352196 | 14.60084364 | 14.74902345 | 14.37248158 | 15.61055183 | 15.44777536 | 14.23043113 | 14.58021625 | 15.97146843 | 18.41026507 | 19.33962529 | 12.57784224 | 10.95396422 |
| ENSMUSG00000021290 | 2010107E04R |  | 361 | 463.6714216 | 352.8767521 | 412.7159931 | 380.2643396 | 471.1862001 | 379.8127806 | 586.8869148 | 472.0623256 | 450.9980521 | 502.0700923 | 519.0398536 | 402.769532 | 612.7543409 | 534.171332 | 460.863941 | 458.5326753 | 476.4776191 | 472.0726112 |
| ENSMUSG00000021294 | Kif26a |  | 6874 | 0 | 0.004846217 | 0 | 0 | 0 | 0.028834876 | 0 | 0 | 0 | 0 | 0.006950096 | 0 | 0 | 0 | 0 | 0 | 0.01115109 | 0 |
| ENSMUSG00000021298 | Gpr132 |  | 2436 | 0 | 0 | 0 | 0.056109661 | 0.019758589 | 0 | 0.271911948 | 0.266531544 | 0.507223101 | 0 | 0.451077194 | 0.114564168 | 0.162009581 | 0.076262869 | 0.304444994 | 0 | 0 | 0 |
| ENSMUSG00000021301 | Hecw1 |  | 9482 | 0.089654453 | 0.017566386 | 0 | 0 | 0 | 0.036581854 | 0 | 0 | 0 | 0.03978123 | 0 | 0 | 0 | 0 | 0 | 0 | 0.03637805 | 0 |
| ENSMUSG00000021302 | Ggps1 |  | 2386 | 32.796324 | 31.45597262 | 35.1549462 | 36.24260777 | 33.38572241 | 34.26739749 | 33.17439725 | 37.37605181 | 32.90087973 | 33.33748382 | 33.93904217 | 33.19464594 | 27.49851214 | 28.12728679 | 33.43093554 | 33.02685249 | 36.27023403 | 29.34894923 |
| ENSMUSG00000021303 | Gng4 |  | 3224 | 80.13227664 | 93.40836344 | 81.517801 | 94.62678439 | 95.29344565 | 89.94497422 | 150.3175466 | 140.2125542 | 105.585159 | 139.7263706 | 139.9166123 | 103.5636767 | 85.93301719 | 86.78014343 | 91.31052011 | 64.25315944 | 84.02303552 | 77.58263284 |
| ENSMUSG00000021306 | Gpr137b |  | 4203 | 0.75847923 | 0.911487708 | 0.736594324 | 0.791329107 | 0.81307794 | 0.813499552 | 0.630385444 | 0.717868139 | 0.538962257 | 1.020869378 | 1.398125123 | 0.385118776 | 0.915510242 | 1.237624979 | 0.990092112 | 1.056795255 | 1.504601235 | 1.112484909 |
| ENSMUSG00000021311 | Mtr |  | 4271 | 1.044964524 | 1.918747829 | 1.812166906 | 2.485535783 | 1.656612685 | 1.728718638 | 2.426007185 | 2.22662332 | 2.285461071 | 1.744277948 | 1.834486816 | 1.973347003 | 1.617061208 | 1.468028986 | 1.254086225 | 2.304070733 | 2.369033571 | 2.472898265 |
| ENSMUSG00000021313 | Ryr2 |  | 18086 | 0 | 0 | 0 | 0.005038267 | 0.010645123 | 0 | 0.013079918 | 0 | 0 | 0.041712443 | 0.007924631 | 0 | 0 | 0 | 0 | 0 | 0.01059557 | 0 |
| ENSMUSG00000021314 | Amph |  | 3172 | 0 | 0.010502173 | 0 | 0 | 0 | 0.109353447 | 0.044747174 | 0 | 0 | 0 | 0.331352171 | 0.070385451 | 0 | 0 | 0 | 0.374214637 | 0 | 0 |
| ENSMUSG00000021318 | Gli3 |  | 9730 | 0.021842331 | 0.047932222 | 0.049715859 | 0.037460263 | 0.079148076 | 0.137505016 | 0.024312785 | 0.027476548 | 0.038096469 | 0.125993657 | 0.024550338 | 0.00573645 | 0.10140168 | 0.042959588 | 0.004234487 | 0 | 0.133925395 | 0 |
| ENSMUSG00000021319 | Sfrp4 |  | 4573 | 0 | 0 | 0 | 0 | 0 | 0 | 0 | 0 | 0 | 0 | 0 | 0 | 0 | 0 | 0 | 0 | 0 | 0 |
| ENSMUSG00000021322 | Aoah |  | 3541 | 0 | 0 | 0 | 0 | 0 | 0 | 0 | 0 | 0.081419263 | 0 | 0 | 0 | 0 | 0 | 0 | 0 | 0 | 0 |
| ENSMUSG00000021326 | Trim27 |  | 4692 | 12.3837544 | 10.85580035 | 11.25828967 | 11.34170925 | 11.06870112 | 10.44493894 | 13.36089079 | 13.56107042 | 11.56944326 | 15.84754029 | 14.9474934 | 13.71599722 | 9.557270419 | 7.522912706 | 10.00182697 | 6.789809326 | 9.132310302 | 10.62196306 |
| ENSMUSG00000021327 | Zkscan3 |  | 9114 | 10.84315715 | 8.315430585 | 9.750074091 | 9.553122199 | 9.416197048 | 9.308128142 | 9.494721281 | 9.185620592 | 8.9612531 | 9.322533165 | 9.021363127 | 8.206384449 | 8.530513674 | 7.893558412 | 9.22672543 | 8.915149101 | 8.944484695 | 6.844451067 |
| ENSMUSG00000021335 | Slc17a1 |  | 2358 | 0 | 0 | 0 | 0 | 0 | 0 | 0 | 0 | 0 | 0 | 0 | 0 | 0 | 0 | 0 | 0 | 0 | 0 |
| ENSMUSG00000021336 | Slc17a4 |  | 3659 | 0 | 0 | 0 | 0 | 0 | 0 | 0 | 0 | 0 | 0 | 0 | 0 | 0 | 0 | 0 | 0 | 0 | 0 |
| ENSMUSG00000021337 | Scgn |  | 1501 | 0 | 0 | 0 | 0 | 0 | 0 | 0 | 0 | 0 | 0 | 0 | 0 | 0 | 0 | 0 | 0 | 0 | 0 |
| ENSMUSG00000021338 | Lrrc16a |  | 9916 | 5.761088804 | 7.169192846 | 7.307739862 | 7.006917367 | 7.644995944 | 7.700762651 | 5.639732401 | 7.063822712 | 6.894878597 | 8.501962225 | 6.615068373 | 6.698329818 | 6.089377445 | 5.915579102 | 5.979128573 | 6.402362553 | 7.529207984 | 7.622278402 |
| ENSMUSG00000021339 | Mrs2 |  | 2024 | 10.31128529 | 12.64046579 | 11.85438293 | 12.65084334 | 12.74639876 | 14.93437129 | 8.204916158 | 9.68018895 | 9.218128281 | 11.39164718 | 11.11759162 | 9.541610304 | 9.48128252 | 10.1424341 | 9.933971974 | 6.016014404 | 11.15325537 | 11.1703275 |
| ENSMUSG00000021340 | Gpld1 |  | 3214 | 34.00149582 | 41.34571719 | 44.44525051 | 48.89235903 | 46.3797659 | 47.33257377 | 17.22334609 | 24.455496 | 14.96759502 | 20.59725805 | 23.78342675 | 22.28111235 | 15.19558124 | 14.65286391 | 19.31883781 | 13.59352304 | 14.61980087 | 19.58009275 |
| ENSMUSG00000021342 | Prl |  | 970 | 0 | 0 | 0 | 0 | 0 | 0 | 0 | 0 | 0 | 0 | 0 | 0 | 0 | 0 | 0 | 0 | 0 | 0 |
| ENSMUSG00000021345 | Prl8a6 |  | 1422 | 0 | 0 | 0 | 0 | 0 | 0 | 0 | 0 | 0 | 0 | 0 | 0 | 0 | 0 | 0 | 0 | 0 | 0 |
| ENSMUSG00000021346 | Prl8a8 |  | 1542 | 0 | 0 | 0 | 0 | 0 | 0 | 0 | 0 | 0 | 0 | 0 | 0 | 0 | 0 | 0 | 0 | 0 | 0 |
| ENSMUSG00000021347 | Prl7b1 |  | 919 | 0 | 0 | 0 | 0 | 0 | 0 | 0 | 0 | 0 | 0 | 0 | 0 | 0 | 0 | 0 | 0 | 0 | 0 |
| ENSMUSG00000021348 | Prl7d1 |  | 1100 | 0 | 0 | 0 | 0 | 0 | 0 | 0 | 0 | 0 | 0 | 0 | 0 | 0 | 0 | 0 | 0 | 0 | 0 |
| ENSMUSG00000021356 | Irf4 |  | 4706 | 0 | 0 | 0.102791182 | 0 | 0.010227778 | 0 | 0 | 0.1866607 | 0.043759579 | 0 | 0.182734648 | 0.20162904 | 0.167724326 | 0 | 0.026265335 | 0.219686868 | 0 | 0.011689143 |
| ENSMUSG00000021357 | Exoc2 |  | 4397 | 16.62699631 | 17.02389659 | 14.95101837 | 19.43882628 | 16.97807906 | 16.43117707 | 12.89074127 | 14.19294645 | 12.45805457 | 13.75809695 | 14.90726473 | 18.27940735 | 13.56431385 | 11.21756216 | 15.77034764 | 12.95802225 | 16.84892698 | 15.38803759 |
| ENSMUSG00000021359 | Tfap2a |  | 3235 | 4.7826535 | 4.767811445 | 4.695297846 | 3.788538153 | 4.046949996 | 5.376509887 | 4.943333996 | 4.852265035 | 5.805579965 | 4.882684881 | 6.291231008 | 5.348641546 | 5.733786985 | 6.259543154 | 7.323305916 | 5.586759411 | 5.734135302 | 6.461657049 |
| ENSMUSG00000021360 | Gcnt2 |  | 6261 | 6.578424464 | 7.097811993 | 6.914919289 | 7.029543017 | 7.564576408 | 7.225945657 | 3.967282613 | 5.81334603 | 4.183776397 | 5.572835076 | 5.593203093 | 5.313230299 | 4.097204438 | 4.703010926 | 4.599886211 | 6.592760706 | 8.123142627 | 5.693323935 |

| ENSMUSG00000021361 | Tmem14c |  | 939 | 121.6308924 | 122.0408478 | 125.0808644 | 111.8889971 | 128.1467616 | 115.9396759 | 124.9578741 | 123.8100883 | 119.2610611 | 120.4627658 | 119.8189853 | 115.970562 | 115.2128803 | 129.1432505 | 103.815635 | 103.8617434 | 104.3667105 | 101.0550683 |
| --- | --- | --- | --- | --- | --- | --- | --- | --- | --- | --- | --- | --- | --- | --- | --- | --- | --- | --- | --- | --- | --- |
| ENSMUSG00000021362 | Gcm2 |  | 2874 | 0 | 0 | 0 | 0 | 0 | 0 | 0 | 0 | 0 | 0 | 0 | 0 | 0 | 0 | 0 | 0 | 0 | 0 |
| ENSMUSG00000021363 | Mak |  | 3550 | 0.059866445 | 0.356588727 | 0.04087904 | 0.295184231 | 0.013558288 | 0 | 0.239895273 | 0.021516846 | 0 | 0.026563776 | 0.255696961 | 0.047168166 | 0.013896315 | 0.340153879 | 0.011606072 | 0 | 0.12955368 | 0.046486569 |
| ENSMUSG00000021364 | Elovl2 |  | 6751 | 0.00629613 | 0.148035376 | 0.057323099 | 0.101231768 | 0.071295991 | 0.264242099 | 0.932096919 | 0.780706789 | 1.085942794 | 0.467945063 | 0.375066331 | 1.198825518 | 0.036536748 | 0.69483822 | 0.164781811 | 0.323295011 | 0.090834062 | 0.464452538 |
| ENSMUSG00000021365 | Nedd9 |  | 4714 | 59.72725626 | 74.46993621 | 59.92817507 | 77.53302878 | 77.86466905 | 73.34098901 | 25.10161434 | 44.66582827 | 33.09599527 | 42.96975418 | 45.44397832 | 33.31889574 | 24.90665319 | 25.92159396 | 20.58329744 | 34.23735897 | 38.5702063 | 30.9119906 |
| ENSMUSG00000021366 | Hivep1 |  | 8744 | 2.318729296 | 2.727817047 | 1.764770835 | 2.370799997 | 2.377972441 | 1.904130659 | 1.374361899 | 1.528744378 | 1.658011613 | 1.784867655 | 2.458683819 | 1.774560178 | 1.760242245 | 2.09805747 | 1.856520245 | 1.777903317 | 2.081998059 | 2.157836643 |
| ENSMUSG00000021367 | Edn1 |  | 2339 | 0.417964536 | 0.413028616 | 0.55839475 | 0.331140553 | 1.111211577 | 0.444894145 | 0.262960593 | 0.359227385 | 0.334563403 | 0.120950928 | 0.285955288 | 0.167041316 | 0 | 0.575835201 | 0.510835879 | 0.180075537 | 0.098314571 | 0.470364316 |
| ENSMUSG00000021368 | Tbc1d7 |  | 1089 | 25.29233615 | 24.04401732 | 29.53939181 | 27.94745419 | 28.28689729 | 22.9789995 | 26.0675917 | 28.72321173 | 25.22626815 | 25.89175441 | 26.32229117 | 31.00870144 | 22.92190096 | 32.54071504 | 32.2726604 | 23.03062245 | 25.90280489 | 26.72159546 |
| ENSMUSG00000021371 | Ccdc90a |  | 2266 | 11.32971153 | 12.58421776 | 10.69511858 | 12.26488837 | 11.49133747 | 13.84240086 | 9.541877436 | 13.87129178 | 12.28688643 | 15.04411229 | 14.69512183 | 10.66557012 | 10.73284873 | 9.346206465 | 7.98211961 | 8.702410773 | 9.877562838 | 10.26869027 |
| ENSMUSG00000021373 | Cap2 |  | 3394 | 0 | 0 | 0 | 0 | 0.042544423 | 0 | 0.013940094 | 0.168793764 | 0.206296632 | 0.125031329 | 0 | 0.082226963 | 0 | 0.232630962 | 0 | 0.022563705 | 0 | 0.162077509 |
| ENSMUSG00000021374 | Nup153 |  | 5999 | 5.299861922 | 5.503096878 | 4.765587004 | 4.24547821 | 4.749808103 | 5.154343349 | 3.454401295 | 5.207765489 | 4.043816933 | 5.132423608 | 5.502999885 | 6.029096409 | 4.96690749 | 3.948405496 | 6.270548513 | 4.946694572 | 5.264355501 | 4.722402061 |
| ENSMUSG00000021375 | Kif13a |  | 6961 | 2.607342359 | 4.039086729 | 4.621232249 | 4.260916515 | 3.885956199 | 4.897612513 | 3.357630138 | 4.224701889 | 2.911042344 | 4.023490552 | 2.731566366 | 3.88889475 | 3.380451737 | 2.989075011 | 3.699320849 | 2.832877864 | 2.39504944 | 2.315424259 |
| ENSMUSG00000021376 | Tpmt |  | 2201 | 4.847250878 | 5.569806939 | 4.901089173 | 3.72602816 | 4.657928097 | 5.268214292 | 10.33957227 | 9.318182686 | 10.38551397 | 7.883443343 | 6.273040891 | 7.557050182 | 9.25673868 | 11.92226687 | 7.375471613 | 4.540594517 | 4.666718569 | 8.397573766 |
| ENSMUSG00000021377 | Dek |  | 4418 | 29.16097522 | 25.2297293 | 27.11019945 | 23.35802748 | 26.65885405 | 23.98002363 | 25.14490044 | 24.14472157 | 27.47321457 | 20.949939 | 20.65418078 | 26.40442164 | 22.0530866 | 22.41258359 | 21.72920444 | 23.63478642 | 24.09056734 | 25.59952997 |
| ENSMUSG00000021379 | Id4 |  | 1657 | 0.102607546 | 0.201043417 | 0.554675363 | 0.27496104 | 0.435714457 | 0.179430538 | 1.170681856 | 1.198554572 | 0.497121494 | 1.508139573 | 0.864966056 | 0.404217231 | 38.25679765 | 45.68730383 | 39.31180438 | 64.05646573 | 48.27216755 | 41.99548582 |
| ENSMUSG00000021381 | Barx1 |  | 1395 | 0 | 0 | 0 | 0 | 0 | 0 | 0 | 0 | 0 | 0 | 0 | 0 | 0 | 0 | 0 | 0 | 0 | 0 |
| ENSMUSG00000021384 | Susd3 |  | 3331 | 0.102083881 | 0.100008689 | 0.145222247 | 0.095745215 | 0.101147843 | 0.0446287 | 0.241463687 | 0.022931493 | 0.07418766 | 0.240637033 | 0.37290571 | 0.083782141 | 0.385058496 | 0.348574658 | 0.086583876 | 0.149437975 | 0.264636694 | 0.08257146 |
| ENSMUSG00000021385 | Ippk |  | 3035 | 6.834440165 | 7.123581004 | 8.686515336 | 8.166460665 | 6.644901488 | 7.445155358 | 7.841277595 | 9.563830586 | 7.74876458 | 8.529072846 | 8.815148846 | 8.570049017 | 6.729296129 | 7.085210037 | 9.068414937 | 7.683354171 | 7.917821469 | 7.866211637 |
| ENSMUSG00000021388 | Aspn |  | 2338 | 0 | 0.014248458 | 0 | 0.097435937 | 0.082347175 | 0.042388994 | 0 | 0 | 0 | 0 | 0 | 0.119366259 | 0 | 0 | 0 | 0 | 0 | 0 |
| ENSMUSG00000021390 | Ogn |  | 2533 | 0.083902835 | 0.052606229 | 0.019097327 | 0.035973979 | 0.133013607 | 0 | 0 | 0.271402781 | 0.260159594 | 0 | 0.056583054 | 0.066106193 | 0 | 0 | 0 | 0.332567455 | 0.105915546 | 0 |
| ENSMUSG00000021391 | Cenpp |  | 1260 | 4.655328803 | 5.076250548 | 4.530219509 | 3.25436031 | 4.278393215 | 3.696791235 | 2.215434966 | 4.546714572 | 5.099282904 | 3.405328569 | 4.436246144 | 2.835081274 | 2.505748179 | 2.764529011 | 4.512551359 | 4.46723753 | 2.798428028 | 2.53216523 |
| ENSMUSG00000021392 | Nol8 |  | 4224 | 5.232644776 | 3.083650956 | 4.168552332 | 2.53476454 | 2.985455493 | 3.847844842 | 5.062815075 | 4.059751108 | 4.407269206 | 3.739461551 | 4.026487978 | 3.263842012 | 2.943097338 | 3.034698892 | 3.940679115 | 3.390314197 | 4.718199429 | 3.893873795 |
| ENSMUSG00000021395 | Spin1 |  | 1468 | 77.27950601 | 93.76626629 | 97.6697166 | 91.33934161 | 87.05058402 | 96.94513 | 69.61538589 | 64.49520815 | 67.89602735 | 74.83728787 | 68.24529151 | 65.32105472 | 60.52233175 | 55.36590799 | 71.12039689 | 72.43393512 | 77.87966189 | 65.12658548 |
| ENSMUSG00000021396 | Nxnl2 |  | 1161 | 6.516728108 | 5.451722568 | 5.541498309 | 4.434451344 | 4.891961234 | 4.90832417 | 1.100294859 | 0.526338018 | 0.886875878 | 1.218364425 | 1.481394061 | 1.057661134 | 2.46447132 | 2.560225317 | 1.525983552 | 1.022402093 | 1.089377948 | 1.800470332 |
| ENSMUSG00000021400 | Wrnip1 |  | 2626 | 11.99403482 | 11.50601487 | 14.47890132 | 16.48247986 | 12.95859483 | 16.96416888 | 13.5848286 | 13.17681515 | 14.99402478 | 15.63909464 | 15.40951631 | 13.58195293 | 14.61547283 | 18.64130545 | 16.411587 | 19.17446639 | 18.02474359 | 22.66559538 |
| ENSMUSG00000021403 | Serpinb9b |  | 3246 | 0.366649701 | 0.071839267 | 0.178830057 | 0.028072116 | 0.34104567 | 0.122126269 | 0.029151373 | 0.035297969 | 0.317209764 | 0.058103146 | 0.014718102 | 0.068780853 | 0.182373077 | 0.243237673 | 0.431562816 | 0.353887314 | 0.413253355 | 0.016946737 |
| ENSMUSG00000021404 | Serpinb9c |  | 2792 | 0 | 0 | 0 | 0 | 0 | 0 | 0 | 0 | 0 | 0 | 0 | 0 | 0 | 0 | 0 | 0 | 0 | 0 |
| ENSMUSG00000021408 | Ripk1 |  | 6432 | 4.850559578 | 5.930233812 | 5.963962931 | 6.261810198 | 5.096057217 | 5.5315396 | 5.024029805 | 6.911684764 | 4.770515274 | 5.56395892 | 7.47969266 | 5.61454193 | 4.862629906 | 5.155640298 | 4.496811604 | 7.030660345 | 6.447303055 | 5.028817592 |
| ENSMUSG00000021411 | 1300014I06Ri | k | 3143 | 2.745323173 | 4.165436662 | 0.769543913 | 2.580294589 | 1.699854765 | 1.27705104 | 1.565548396 | 4.046474706 | 1.048336386 | 1.770214122 | 1.656847112 | 1.473973909 | 0.847573507 | 4.610421656 | 1.782822654 | 1.729960627 | 2.255922662 | 1.45267447 |
| ENSMUSG00000021413 | Prpf4b |  | 4450 | 20.97558799 | 17.87667222 | 19.71900765 | 21.87953972 | 17.85748449 | 17.82784864 | 20.91326729 | 20.10894356 | 19.9731463 | 20.34367421 | 23.2433225 | 19.17801081 | 19.33367724 | 17.36695762 | 19.86933459 | 19.65297692 | 20.76510154 | 21.65751797 |
| ENSMUSG00000021414 | 1700026J04R |  | 2388 | 0 | 0.013950123 | 0 | 0 | 0 | 0.041501452 | 0 | 0 | 0.137978277 | 0 | 0 | 0 | 0 | 0 | 0 | 0 | 0.032099076 | 0 |
| ENSMUSG00000021415 | 4933417A18R |  | 1166 | 0 | 0 | 0 | 0 | 0.041279523 | 0 | 0 | 0 | 0 | 0 | 0 | 0 | 0 | 0 | 0 | 0 | 0 | 0 |
| ENSMUSG00000021416 | Eci3 |  | 1408 | 0.271695017 | 0.189277808 | 0.240493404 | 0.388304355 | 0.752061307 | 0.211162217 | 0.235219284 | 0.298378144 | 0.263266081 | 0.401852585 | 0.237517549 | 0.118925417 | 0.665700588 | 0.494787863 | 0.292624687 | 0.380730471 | 0.326644576 | 0.273482775 |
| ENSMUSG00000021417 | Eci2 |  | 2920 | 31.57319411 | 25.81749302 | 26.35694753 | 25.5734766 | 29.19234138 | 25.04788862 | 24.38547321 | 25.79295121 | 23.35783223 | 27.22468688 | 27.35607212 | 23.56873698 | 25.93304555 | 25.73511418 | 25.97673438 | 28.27210601 | 27.43217826 | 25.24390514 |
| ENSMUSG00000021418 | Rpp40 |  | 6775 | 1.242217691 | 1.032576795 | 1.542240968 | 1.956939321 | 1.449285967 | 1.682233022 | 1.955357948 | 1.66862747 | 1.392134629 | 1.350145599 | 1.459692458 | 1.853656691 | 1.521825935 | 1.672672668 | 1.435212869 | 2.142013314 | 1.346370282 | 1.485854838 |
| ENSMUSG00000021420 | Fars2 |  | 2118 | 26.65102633 | 29.9312737 | 30.44471953 | 28.65312137 | 30.90623994 | 30.67215945 | 28.25804478 | 29.48280356 | 24.71579865 | 29.07404082 | 30.90259354 | 31.36007482 | 29.09139031 | 32.25649318 | 28.80996425 | 26.8829713 | 26.85374155 | 31.78996536 |
| ENSMUSG00000021423 | Ly86 |  | 992 | 0 | 0.067163093 | 0 | 0 | 0 | 0 | 2.337017407 | 0.308003245 | 0.871891967 | 0.903088066 | 3.756498751 | 1.125315778 | 1.889730702 | 0.842735457 | 1.162947142 | 1.235180884 | 0 | 0.055452729 |
| ENSMUSG00000021427 | Ssr1 |  | 10921 | 20.46444607 | 19.76628098 | 20.10949801 | 20.31703012 | 21.40176003 | 20.64961913 | 21.09380402 | 21.76627713 | 19.93893497 | 20.84025681 | 24.05590117 | 20.53542096 | 24.47397929 | 20.74482724 | 23.00586831 | 22.98628533 | 27.00493129 | 22.60606821 |
| ENSMUSG00000021428 | Riok1 |  | 2472 | 15.81907846 | 20.03894567 | 19.19677722 | 18.04379547 | 19.29560532 | 18.44195586 | 19.31168812 | 20.87295131 | 20.16006637 | 19.83686545 | 20.87255468 | 16.1666725 | 16.82314169 | 21.7189988 | 22.40084592 | 18.43277622 | 24.27952304 | 18.40312753 |
| ENSMUSG00000021431 | Snrnp48 |  | 1780 | 10.55465607 | 8.290793337 | 8.859421852 | 8.574690928 | 9.031495789 | 7.043169436 | 9.515696099 | 8.968777643 | 10.1346595 | 7.575899512 | 10.03810925 | 11.00634695 | 11.25211147 | 10.77607196 | 10.74018086 | 11.12148541 | 9.150941676 | 9.642045673 |
| ENSMUSG00000021432 | Slc35b3 |  | 2203 | 18.50316106 | 19.94539604 | 18.83998595 | 20.16432965 | 20.1004856 | 21.45860546 | 21.11137693 | 18.20336928 | 21.29434474 | 20.80366934 | 24.80914775 | 19.78757716 | 25.34894705 | 28.29231288 | 24.21970716 | 31.28601603 | 25.59145835 | 26.46829466 |

| ENSMUSG00000021439 | Ctsq |  | 2015 | 0 | 0 | 0 | 0 | 0 | 0 | 0 | 0 | 0 | 0 | 0 | 0 | 0 | 0 | 0 | 0 | 0 | 0 |
| --- | --- | --- | --- | --- | --- | --- | --- | --- | --- | --- | --- | --- | --- | --- | --- | --- | --- | --- | --- | --- | --- |
| ENSMUSG00000021440 | Cts7 |  | 1485 | 0 | 0 | 0 | 0 | 0 | 0 | 0 | 0 | 0 | 0 | 0 | 0 | 0 | 0 | 0 | 0 | 0 | 0 |
| ENSMUSG00000021441 | Cts6 |  | 1348 | 0 | 0 | 0 | 0 | 0 | 0 | 0 | 0 | 0 | 0 | 0 | 0 | 0 | 0 | 0 | 0 | 0 | 0 |
| ENSMUSG00000021448 | Shc3 |  | 1525 | 0 | 0 | 0.031720348 | 0 | 0 | 0.097480787 | 0 | 0 | 0 | 0 | 0 | 0 | 0 | 0.243641114 | 0 | 0.125542975 | 0.075395994 | 0 |
| ENSMUSG00000021451 | Sema4d |  | 8707 | 1.703721883 | 1.798215262 | 1.977831263 | 1.360499773 | 2.012176434 | 1.883766488 | 0.94005897 | 1.662446366 | 1.660326982 | 1.272587029 | 1.563783527 | 2.423144649 | 1.252136639 | 0.906798536 | 1.429065106 | 1.429246285 | 1.267712588 | 1.017166209 |
| ENSMUSG00000021453 | Gadd45g |  | 1258 | 75.17807366 | 74.86132906 | 49.64247034 | 67.76209376 | 62.70923918 | 55.1461264 | 65.70369641 | 83.33715787 | 59.25881839 | 74.58656557 | 67.25711563 | 43.52556836 | 162.975714 | 223.1383658 | 190.4507532 | 138.156651 | 140.5703767 | 195.6802485 |
| ENSMUSG00000021456 | Fbp2 |  | 1318 | 0 | 0 | 0 | 0 | 0 | 0 | 0 | 0 | 0 | 0 | 0 | 0 | 0 | 0 | 0.093781994 | 0 | 0 | 0 |
| ENSMUSG00000021457 | Syk |  | 8286 | 0.47193775 | 0.080407662 | 0.507904555 | 0.318916313 | 0.191691224 | 0.233231548 | 1.0677614 | 0.640688382 | 0.347943049 | 0.113808118 | 1.4529676 | 0.067361408 | 0.416755275 | 0.448410209 | 0.333152817 | 0.406658635 | 0.055505137 | 0.205802837 |
| ENSMUSG00000021458 | 2010111I01Ri | k | 6603 | 5.780652442 | 4.994663831 | 5.545776538 | 5.188839216 | 5.598260999 | 5.103113559 | 4.750614286 | 4.2455283 | 6.143982739 | 5.462712099 | 5.904038485 | 5.105658065 | 7.979174262 | 6.246001757 | 8.473680585 | 9.115982862 | 7.626950497 | 9.397284929 |
| ENSMUSG00000021460 | Auh |  | 9564 | 7.466404823 | 6.809567984 | 5.922773321 | 7.107599137 | 6.794029376 | 7.077481605 | 7.395697929 | 7.74709961 | 9.465491598 | 8.745853992 | 7.133274854 | 6.104473344 | 7.964082003 | 7.400751692 | 7.754370619 | 8.671837686 | 8.764074794 | 7.362155647 |
| ENSMUSG00000021461 | Fancc |  | 7791 | 3.104279959 | 1.808670807 | 2.024101001 | 2.397641917 | 2.069592405 | 2.219728407 | 2.993858389 | 1.808881593 | 2.421181391 | 2.130284629 | 2.538677039 | 2.120579659 | 3.349580829 | 3.302531692 | 4.167221928 | 4.590351343 | 4.506082398 | 3.876267436 |
| ENSMUSG00000021464 | Ror2 |  | 6582 | 0 | 0 | 0 | 0.020766201 | 0.014625319 | 0 | 0 | 0 | 0 | 0 | 0 | 0 | 0 | 0 | 0 | 0 | 0 | 0 |
| ENSMUSG00000021466 | Ptch1 |  | 6550 | 8.351780389 | 9.286922877 | 9.977502215 | 10.2738416 | 9.420629945 | 9.653326419 | 7.172746592 | 8.134107077 | 9.224521927 | 7.680885554 | 8.964186866 | 7.976100791 | 7.28304794 | 7.282149275 | 9.221600143 | 10.30046569 | 8.355717859 | 7.172179716 |
| ENSMUSG00000021468 | Sptlc1 |  | 3831 | 31.3879256 | 29.46943318 | 30.10245272 | 34.38187919 | 36.69886427 | 31.37951753 | 27.1575518 | 29.37953795 | 33.20938324 | 30.30149606 | 28.48290398 | 32.66476521 | 27.93028676 | 25.6285045 | 30.05960551 | 33.83286506 | 30.14281717 | 23.85020263 |
| ENSMUSG00000021469 | Msx2 |  | 2454 | 12.95593793 | 10.47985099 | 10.88108751 | 15.35410907 | 12.47428829 | 10.07612635 | 8.637359478 | 7.983986322 | 12.20154725 | 8.780713687 | 10.49335885 | 9.598292425 | 21.08768591 | 12.75603704 | 15.41280697 | 18.67720337 | 16.05518876 | 16.58791319 |
| ENSMUSG00000021470 | 0610007P08R |  | 7582 | 3.481365644 | 4.253083831 | 5.110419126 | 4.002064828 | 4.20249716 | 3.888667433 | 4.474174465 | 4.246398737 | 4.0252187 | 3.656635916 | 4.637611374 | 3.717608758 | 3.500471049 | 4.520672183 | 4.733125189 | 5.23200597 | 5.201498221 | 3.81624771 |
| ENSMUSG00000021474 | Sfxn1 |  | 2800 | 19.59792223 | 16.04967654 | 16.91345936 | 17.21556604 | 18.47922069 | 16.45858651 | 15.86664483 | 17.66398611 | 17.43072185 | 18.91079991 | 20.01429511 | 13.85424482 | 23.94359842 | 21.2979315 | 22.67557058 | 22.38633011 | 20.5593207 | 24.71480581 |
| ENSMUSG00000021476 | Habp4 |  | 2558 | 11.4155809 | 12.08536585 | 10.85473277 | 12.50346096 | 14.58258047 | 11.39054235 | 16.09148971 | 13.15383367 | 13.54099287 | 11.05958638 | 10.88850696 | 13.0047439 | 13.55759884 | 15.03350444 | 17.45992439 | 13.2774702 | 16.36134332 | 18.58008921 |
| ENSMUSG00000021477 | Ctsl |  | 1966 | 1546.098753 | 1449.161732 | 1445.300698 | 1393.621039 | 1577.483403 | 1466.06526 | 1219.492722 | 1192.084731 | 1302.968736 | 1338.44682 | 1379.009839 | 1162.419374 | 1407.967045 | 1101.806834 | 1197.129439 | 1423.471833 | 1339.392958 | 1168.733666 |
| ENSMUSG00000021478 | Drd1a |  | 3524 | 0 | 0 | 0 | 0 | 0 | 0 | 0 | 0 | 0 | 0 | 0 | 0 | 0 | 0 | 0 | 0 | 0 | 0 |
| ENSMUSG00000021481 | Zfp346 |  | 4373 | 4.830796361 | 6.025725892 | 5.984468311 | 6.334579226 | 5.965584868 | 5.28048796 | 8.060358061 | 6.192182325 | 7.026101248 | 6.986886739 | 6.347416162 | 6.713706499 | 9.611432316 | 11.39595375 | 11.97511492 | 10.34100328 | 10.3155846 | 9.472183252 |
| ENSMUSG00000021482 | 1110018J18R |  | 4954 | 24.95055549 | 24.38283295 | 24.75310849 | 24.40835924 | 23.83278336 | 23.91614574 | 19.71202449 | 22.10286994 | 19.7452513 | 22.04300136 | 22.06481732 | 22.03783529 | 19.71683412 | 14.23135338 | 19.977016 | 24.52484806 | 23.33308669 | 19.7317688 |
| ENSMUSG00000021483 | Cdk20 |  | 1946 | 2.053179109 | 2.584916252 | 2.187497776 | 2.200790425 | 2.819650205 | 2.138966917 | 2.479904034 | 1.432705743 | 1.735505803 | 2.737939089 | 2.160429777 | 2.552720437 | 1.723828558 | 1.527452 | 1.079794116 | 2.951485668 | 1.674067439 | 1.017640207 |
| ENSMUSG00000021484 | Lman2 |  | 4307 | 56.47948048 | 64.47557144 | 59.94184618 | 62.27487068 | 58.34612804 | 63.27839208 | 69.3377361 | 73.5117288 | 62.17662537 | 61.09776524 | 67.38631826 | 66.64962913 | 67.58942276 | 72.52912277 | 67.83381305 | 56.95138867 | 61.66734097 | 65.68651866 |
| ENSMUSG00000021485 | Mxd3 |  | 1949 | 0.02180871 | 0.034184602 | 0.049639333 | 0 | 0.098782809 | 0.101698786 | 0.218478251 | 0.05878769 | 0.338114034 | 0.193538033 | 0.122562746 | 0.343657235 | 0.151868396 | 0.357445516 | 0.295957816 | 0 | 0.373627317 | 0.73383108 |
| ENSMUSG00000021486 | Prelid1 |  | 1083 | 139.5253008 | 127.8686069 | 133.2840393 | 125.4927011 | 120.9742347 | 128.6632381 | 168.7179742 | 139.8624819 | 143.2971296 | 140.450756 | 136.7519587 | 143.5333521 | 153.7351993 | 152.1976603 | 133.4199968 | 128.307123 | 132.8503403 | 136.3291251 |
| ENSMUSG00000021488 | Nsd1 |  | 13098 | 8.359545995 | 6.80348084 | 7.183273518 | 6.563879269 | 6.982031987 | 7.539975418 | 7.773468091 | 7.018561047 | 7.483883609 | 6.630905131 | 7.305943032 | 6.958846923 | 7.544039571 | 5.630875765 | 6.291274376 | 7.396185427 | 6.592544428 | 6.984283439 |
| ENSMUSG00000021490 | Slc34a1 |  | 3071 | 0 | 0 | 0 | 0 | 0 | 0 | 0 | 0.012436471 | 0 | 0 | 0.046670425 | 0 | 0.064255184 | 0 | 0 | 0 | 0 | 0 |
| ENSMUSG00000021492 | F12 |  | 2148 | 0 | 0 | 0 | 0 | 0 | 0 | 0 | 0 | 0 | 0 | 0 | 0 | 0 | 0 | 0 | 0 | 0 | 0 |
| ENSMUSG00000021493 | Pdlim7 |  | 4269 | 15.08440892 | 24.15165322 | 15.43329781 | 19.55208087 | 19.21218035 | 15.70504768 | 2.648800718 | 2.603417451 | 2.942583113 | 3.048394026 | 2.227036012 | 1.542808195 | 1.802712367 | 0.881230049 | 1.206417073 | 1.47996023 | 1.337694598 | 1.352999814 |
| ENSMUSG00000021494 | Ddx41 |  | 2188 | 34.42375316 | 32.85613607 | 29.07275705 | 33.08797964 | 38.73871919 | 29.0567446 | 30.6191743 | 31.0356908 | 27.76513288 | 29.78165992 | 36.28975365 | 30.76494016 | 28.18322513 | 30.46035896 | 27.86942537 | 29.40046638 | 31.52986034 | 34.36812107 |
| ENSMUSG00000021495 | Fam193b |  | 4194 | 9.303708058 | 8.657857582 | 6.89732264 | 8.386534627 | 7.436692069 | 7.490804265 | 7.569577387 | 7.294257112 | 8.808847076 | 8.094541332 | 7.780232866 | 7.372884376 | 7.645623803 | 7.485980705 | 6.847278125 | 5.961794619 | 5.464741428 | 8.263170538 |
| ENSMUSG00000021496 | Pcbd2 |  | 542 | 30.58490526 | 25.38417954 | 29.36326843 | 21.35148572 | 30.99269624 | 30.71903779 | 44.51931043 | 29.52512289 | 29.5600639 | 31.75277987 | 31.02727931 | 33.77774415 | 40.59416071 | 37.10385579 | 32.68758124 | 37.79607926 | 28.63865359 | 37.34935659 |
| ENSMUSG00000021497 | Txndc15 |  | 1516 | 81.42152453 | 76.00877382 | 77.72949864 | 74.29215145 | 77.72226196 | 75.11357636 | 63.60372009 | 61.82332157 | 66.50698587 | 64.56784958 | 62.17677644 | 57.14110048 | 65.66742023 | 59.31118284 | 60.38908786 | 63.80084056 | 54.05120236 | 65.56824264 |
| ENSMUSG00000021499 | Catsper3 |  | 1533 | 0.554535891 | 0.28249682 | 0.851979987 | 0.980766121 | 0.753533052 | 0.614156515 | 1.080198149 | 1.395156252 | 1.450797033 | 1.660885829 | 0.966095051 | 0.655369815 | 0.8688596 | 0.878590042 | 1.209439018 | 0.224798087 | 1.825061757 | 1.184149068 |
| ENSMUSG00000021500 | Ddx46 |  | 5830 | 13.29836039 | 12.15949209 | 11.61628516 | 8.901205746 | 11.64908135 | 12.19694214 | 9.779035696 | 11.68045514 | 10.20125708 | 9.155162279 | 10.94808654 | 11.06739382 | 11.11872029 | 11.61500505 | 10.65728067 | 11.02743222 | 9.466529595 | 9.511008504 |
| ENSMUSG00000021501 | Caml |  | 1809 | 30.28699386 | 27.30957553 | 29.41452924 | 29.64364245 | 30.2254645 | 29.20022885 | 29.99869692 | 31.96423286 | 31.30539479 | 30.36515716 | 33.09122331 | 32.98338491 | 34.30599886 | 30.55194251 | 33.07056891 | 31.98292304 | 31.94917398 | 31.56409772 |
| ENSMUSG00000021504 | B4galt7 |  | 3017 | 7.368315235 | 6.503577957 | 5.499542894 | 8.305791974 | 6.971710521 | 6.914716883 | 6.978502527 | 7.595439656 | 7.685783352 | 7.782913564 | 8.503530893 | 5.180107897 | 6.000932595 | 6.819599176 | 6.992110247 | 6.942314302 | 7.228260835 | 9.754680841 |
| ENSMUSG00000021506 | Pitx1 |  | 2649 | 0 | 0 | 0 | 0.068797349 | 0 | 0 | 0 | 0 | 0.062191794 | 0 | 0 | 0 | 0 | 0 | 0 | 0 | 0 | 0 |
| ENSMUSG00000021508 | Cxcl14 |  | 1819 | 0.420611967 | 0.512787816 | 0.026593475 | 0.425804153 | 0.158763904 | 0.272417447 | 0 | 0.986829529 | 0.316993524 | 0.207369778 | 0 | 0.061369613 | 0 | 0.127663792 | 0.226506629 | 0.757813011 | 0.105349909 | 0 |
| ENSMUSG00000021509 | Slc25a48 |  | 3366 | 0.025255601 | 0 | 0 | 0 | 0.085796655 | 0 | 0.267065033 | 0.124811773 | 0.31813708 | 0.056031733 | 0.099353746 | 0.331643866 | 0.351742726 | 0.579516242 | 0.318253254 | 0.045502801 | 0.03415891 | 0.277823771 |

| ENSMUSG00000021510 | A530054K11R | 6990 | 2.152622649 | 2.030227888 | 2.346012416 | 2.431225971 | 2.478897356 | 2.254330369 | 1.800453876 | 1.666478217 | 1.608572075 | 2.225998866 | 2.303313449 | 2.419477219 | 1.40444228 | 2.19928368 | 1.780095834 | 1.78579943 | 1.348822466 | 1.754940029 |
| --- | --- | --- | --- | --- | --- | --- | --- | --- | --- | --- | --- | --- | --- | --- | --- | --- | --- | --- | --- | --- |
| ENSMUSG00000021514 | Zfp369 | 6022 | 2.498643692 | 2.113172632 | 2.682955686 | 3.011174966 | 3.077182101 | 2.764815424 | 2.011299536 | 2.080223844 | 2.968274301 | 2.513347019 | 2.887759031 | 1.955679974 | 2.482160567 | 1.766140155 | 1.97729154 | 2.327194007 | 2.83851352 | 2.48463584 |
| ENSMUSG00000021518 | Ptdss1 | 3411 | 27.5641892 | 28.4785692 | 26.91672841 | 30.12024479 | 27.61482692 | 30.76889174 | 26.02127988 | 29.01099813 | 27.11958792 | 28.6829989 | 29.30085404 | 26.99966088 | 30.54500419 | 30.67678947 | 28.18036643 | 28.95088727 | 30.16889686 | 26.33534778 |
| ENSMUSG00000021519 | Mterfd1 | 4567 | 11.46625287 | 11.05080682 | 9.892900668 | 11.67208712 | 11.74052178 | 10.11235991 | 12.46270041 | 11.37325756 | 13.52743018 | 11.12950692 | 13.77701343 | 11.17046543 | 10.12130643 | 12.86441221 | 11.34012607 | 10.65630874 | 11.04388148 | 11.32221597 |
| ENSMUSG00000021520 | Uqcrb | 872 | 479.7917978 | 373.4329599 | 425.764731 | 383.9771959 | 424.4661617 | 394.7170727 | 499.930073 | 430.7156941 | 432.175022 | 448.8509034 | 462.7377286 | 394.9982269 | 500.3342619 | 472.8562017 | 415.5120212 | 460.5411587 | 439.7854665 | 439.8836023 |
| ENSMUSG00000021532 | Fastkd3 | 1825 | 6.56792309 | 6.753847047 | 5.32771485 | 7.664241431 | 5.960446438 | 6.299306406 | 9.618084319 | 7.512916418 | 4.58129463 | 5.838936403 | 7.251322465 | 7.584813327 | 4.243896445 | 9.721447333 | 7.766211083 | 8.119706885 | 5.229176947 | 7.927339763 |
| ENSMUSG00000021534 | 1700001L19R | 1805 | 0.306131462 | 0.221470765 | 0.267997398 | 0.201932606 | 0.106663543 | 0.192171266 | 0.393180157 | 0.634776771 | 0.433542271 | 0.130611366 | 0.158808726 | 0.742147314 | 0.437291233 | 0.411692741 | 0.273916161 | 0.50912719 | 0.297267677 | 0.335235553 |
| ENSMUSG00000021536 | Adcy2 | 4186 | 1.106799854 | 1.766713454 | 1.467615469 | 1.273445339 | 1.598265024 | 1.905874368 | 0.904207911 | 0.638668937 | 0.216460427 | 0.101375138 | 0.182608537 | 0.840035056 | 0.212129601 | 0.266282393 | 0 | 0.192093348 | 0.009155828 | 0.144553314 |
| ENSMUSG00000021537 | Cetn3 | 994 | 119.775652 | 112.5064244 | 107.8428001 | 106.5230051 | 101.7355851 | 102.2959852 | 127.2301718 | 117.7663112 | 136.2801311 | 109.9076252 | 118.8125728 | 115.8989211 | 106.1082889 | 110.7369086 | 112.5374489 | 125.4267784 | 107.8071692 | 91.58960928 |
| ENSMUSG00000021538 | Il9 | 531 | 0 | 0 | 0 | 0 | 0 | 0 | 0 | 0 | 0 | 0 | 0 | 0 | 0 | 0 | 0 | 0 | 0 | 0 |
| ENSMUSG00000021539 | Lect2 | 954 | 0.089109384 | 0 | 0.101412013 | 0 | 0 | 0.155826206 | 0 | 0 | 0 | 0 | 0 | 0 | 0.10342121 | 0 | 0 | 0 | 0.160697262 | 0 |
| ENSMUSG00000021540 | Smad5 | 5680 | 9.384065271 | 8.674255379 | 8.678279477 | 9.031995761 | 10.49072562 | 9.282413473 | 8.071476388 | 7.759512742 | 8.012517591 | 7.728398721 | 8.326973397 | 8.107028457 | 6.305452807 | 6.11622841 | 7.072450175 | 8.103047552 | 8.717883028 | 6.982670061 |
| ENSMUSG00000021541 | Trpc7 | 4325 | 0 | 0 | 0 | 0 | 0 | 0 | 0 | 0 | 0 | 0 | 0 | 0 | 0 | 0 | 0 | 0 | 0 | 0 |
| ENSMUSG00000021545 | 1700067P10R | 791 | 0 | 0 | 0 | 0 | 0 | 0 | 0 | 0 | 0.624827041 | 0.11921796 | 0 | 0 | 0 | 0 | 0 | 0 | 0.096905934 | 0 |
| ENSMUSG00000021546 | Hnrnpk | 6657 | 144.9019776 | 164.5077489 | 158.8834672 | 160.3499636 | 165.8986013 | 161.6846817 | 128.7470601 | 148.9371739 | 141.0933592 | 143.888619 | 144.2219567 | 144.9178177 | 119.9246533 | 121.0113709 | 137.945062 | 135.5097521 | 148.6705671 | 128.2802122 |
| ENSMUSG00000021548 | Ccnh | 3311 | 15.19967636 | 12.96898842 | 12.18469475 | 13.32017243 | 12.35642861 | 13.9035003 | 19.94820289 | 14.99550683 | 16.60646286 | 18.98275066 | 15.03518778 | 15.54274869 | 17.44719877 | 17.6322011 | 17.03561764 | 16.46808364 | 17.2011106 | 15.4178348 |
| ENSMUSG00000021549 | Rasa1 | 9216 | 10.93530516 | 9.9765178 | 9.264244909 | 11.48419119 | 10.2886165 | 11.59241467 | 8.101064165 | 9.958370549 | 7.9548609 | 11.84906996 | 8.097274864 | 9.738670295 | 7.467233572 | 7.745720738 | 9.674497282 | 7.570039788 | 9.273832692 | 8.22510298 |
| ENSMUSG00000021550 | 2210016F16R | 2818 | 35.68745439 | 31.43328095 | 35.73942165 | 32.10938043 | 34.17742344 | 30.22751917 | 34.61985236 | 29.04411581 | 31.71566331 | 32.46002992 | 36.97558 | 34.26582551 | 31.68586596 | 32.50097244 | 34.46134394 | 33.10004245 | 29.29554419 | 30.88162059 |
| ENSMUSG00000021552 | Gkap1 | 1506 | 10.16059985 | 9.06924743 | 10.21433111 | 8.349832826 | 10.22723478 | 11.05558996 | 13.57176447 | 14.98785513 | 13.94762486 | 11.55286156 | 10.18310868 | 13.86126016 | 14.93715424 | 22.38938077 | 12.99517865 | 9.153133243 | 13.05537205 | 12.89390085 |
| ENSMUSG00000021553 | Slc28a3 | 7858 | 0.048682436 | 0.347627555 | 0.030779798 | 0.046384367 | 0.036751278 | 0.208098779 | 0 | 0 | 0 | 0.012000688 | 0 | 0 | 0.577568897 | 0.419639884 | 0.157297872 | 0.053601003 | 0.009754721 | 0.315017791 |
| ENSMUSG00000021555 | Naa35 | 3719 | 20.03537875 | 18.90029761 | 20.40819283 | 21.19403246 | 18.59789575 | 20.51928199 | 18.07779423 | 20.31314116 | 18.38387098 | 20.69103192 | 22.17251367 | 19.24056989 | 22.80224948 | 21.1052723 | 20.185328 | 23.51593098 | 24.15618175 | 23.13370339 |
| ENSMUSG00000021556 | Golm1 | 3682 | 4.340561159 | 7.075144834 | 5.307687741 | 5.67966305 | 5.411900163 | 5.961939428 | 9.534494233 | 11.1091969 | 7.88606562 | 8.438977036 | 6.474661673 | 8.049461389 | 9.325098975 | 6.786235474 | 7.273495744 | 6.853207571 | 7.400868305 | 7.24590352 |
| ENSMUSG00000021557 | Agtpbp1 | 11598 | 4.976895072 | 4.029831918 | 4.892408269 | 4.961510519 | 4.809872351 | 4.635688561 | 5.666262375 | 5.637643811 | 5.763555361 | 6.293265101 | 5.865799351 | 4.783649647 | 5.031872576 | 3.207597344 | 4.671499051 | 4.041015991 | 5.971341482 | 5.008589127 |
| ENSMUSG00000021559 | Dapk1 | 5907 | 8.275089289 | 10.10609555 | 8.557700901 | 10.8831274 | 9.574235705 | 10.4524642 | 8.714439593 | 16.12524997 | 12.34131249 | 15.42954281 | 15.12428853 | 11.95307486 | 14.84884864 | 12.2970294 | 15.00330908 | 14.61097496 | 14.92305449 | 14.35991918 |
| ENSMUSG00000021565 | Slc6a19 | 6608 | 0 | 0 | 0 | 0 | 0 | 0.067490103 | 0 | 0.005779722 | 0 | 0.064218573 | 0.144597332 | 0.244953725 | 0 | 0 | 0 | 0 | 0 | 0 |
| ENSMUSG00000021566 | 5430425J12R | 1653 | 0 | 0 | 0 | 0 | 0 | 0 | 0 | 0 | 0 | 0 | 0 | 0 | 0 | 0 | 0 | 0 | 0 | 0 |
| ENSMUSG00000021567 | Nkd2 | 6561 | 1.690878059 | 3.802980913 | 1.688391777 | 2.749912141 | 2.105450708 | 2.333759286 | 0.165857585 | 0.058211252 | 0.056497278 | 0.201222328 | 0 | 0.263722838 | 0.075189632 | 0.212364368 | 0.194672799 | 0.233443727 | 0.479005692 | 0.251527694 |
| ENSMUSG00000021569 | Trip13 | 2254 | 1.131459877 | 1.330151056 | 2.274886521 | 1.596860028 | 1.13176218 | 0.417702723 | 2.833723006 | 1.118322342 | 1.132903273 | 1.882681142 | 0.784238449 | 0.693362268 | 1.991661256 | 0.886022963 | 2.010723668 | 2.174444431 | 1.479320244 | 1.317875671 |
| ENSMUSG00000021572 | Cep72 | 3620 | 0.270060511 | 0.349693365 | 0.467699879 | 0.302062172 | 0.106368892 | 0.520166817 | 0.522792032 | 0.917883704 | 1.206013997 | 0.416801797 | 0.382727568 | 0.493398122 | 0.490593652 | 0.307916601 | 1.297507561 | 0.507720761 | 0.688179364 | 0.151958858 |
| ENSMUSG00000021573 | Tppp | 3050 | 0 | 0.218445208 | 0 | 0.179256568 | 0.55233355 | 0.097480787 | 0.698055919 | 0.237919884 | 0.648181232 | 0.742043855 | 0.95549917 | 0.164701955 | 0.097046395 | 0.060910279 | 0.918592066 | 0.514726198 | 0.326715974 | 0.108214636 |
| ENSMUSG00000021575 | Ahrr | 12855 | 0 | 0 | 0 | 0 | 0 | 0 | 0 | 0 | 0 | 0.011003665 | 0 | 0 | 0 | 0 | 0 | 0 | 0 | 0 |
| ENSMUSG00000021576 | Pdcd6 | 1518 | 96.18266117 | 90.34860705 | 79.09295279 | 88.96109052 | 89.76381812 | 87.94141256 | 94.90586781 | 92.43668408 | 87.03975136 | 96.47568137 | 86.3914763 | 86.81210762 | 101.9461294 | 96.68202645 | 88.89004976 | 104.075788 | 100.7390808 | 93.02264622 |
| ENSMUSG00000021577 | Sdha | 4308 | 155.6845804 | 168.7760514 | 163.4683971 | 170.2405132 | 168.9533309 | 181.1067294 | 189.6351038 | 179.3393937 | 181.0657897 | 184.9691005 | 185.8321679 | 168.6000969 | 174.2419808 | 148.2480607 | 172.1417838 | 178.0051055 | 184.2295627 | 179.7372763 |
| ENSMUSG00000021578 | Ccdc127 | 5786 | 11.17358672 | 11.11197474 | 10.8100544 | 11.86665984 | 12.17855794 | 11.75871123 | 10.4748603 | 12.00030895 | 10.10087554 | 11.62876833 | 10.62674932 | 8.981054592 | 9.080278586 | 10.56350138 | 10.21137766 | 11.67380426 | 12.83063206 | 10.99991989 |
| ENSMUSG00000021579 | Lrrc14b | 2753 | 0 | 0 | 0 | 0 | 0 | 0 | 0 | 0 | 0 | 0 | 0 | 0 | 0 | 0 | 0 | 0 | 0 | 0 |
| ENSMUSG00000021583 | Erap1 | 4675 | 16.17469693 | 16.66000999 | 16.91780153 | 16.68460062 | 21.53839237 | 19.52430701 | 13.05526328 | 15.73445284 | 14.22806908 | 13.72665393 | 13.08063034 | 14.80458216 | 12.06125806 | 11.61350549 | 10.77850328 | 11.08175226 | 12.7317089 | 11.82548711 |
| ENSMUSG00000021585 | Cast | 3883 | 38.71769447 | 61.47828998 | 47.99979718 | 58.50305616 | 53.61075711 | 61.57402511 | 31.57021661 | 37.72028412 | 28.71282466 | 39.42784792 | 34.85615693 | 37.47397176 | 38.62194914 | 35.89459342 | 33.09494021 | 43.58601203 | 45.0578278 | 39.2416239 |
| ENSMUSG00000021587 | Pcsk1 | 3552 | 0 | 0.131300822 | 0.136186741 | 0 | 0 | 0.167407884 | 0 | 0 | 0 | 0 | 0.013450157 | 0 | 0 | 0.156905701 | 0 | 0 | 0.226591282 | 0.015486798 |
| ENSMUSG00000021589 | Rhobtb3 | 6119 | 12.07288706 | 16.56661826 | 15.71606118 | 14.77253015 | 17.2815552 | 16.6984371 | 15.44099033 | 16.5215377 | 15.58881688 | 15.85817083 | 16.52096946 | 14.65856672 | 14.26989599 | 16.78180703 | 15.04910564 | 12.22120547 | 21.16433359 | 12.81058622 |
| ENSMUSG00000021590 | Spata9 | 1098 | 0.193557268 | 0.27305651 | 0.044056039 | 0.082989152 | 0.482195957 | 0.135389982 | 0.473988587 | 0.347836087 | 0.18755244 | 0.300596469 | 0.826706932 | 0.355837557 | 0.449288864 | 0.169195218 | 0.750483714 | 0.348730486 | 0.349055527 | 0.050099369 |
| ENSMUSG00000021591 | Glrx | 1342 | 19.38388057 | 25.2204922 | 26.74602051 | 21.96571959 | 30.19901619 | 25.18253674 | 18.1917603 | 21.25910924 | 21.29913706 | 22.87265858 | 23.06868338 | 20.37978738 | 31.39303827 | 40.73374878 | 34.72351692 | 36.35039779 | 35.21335625 | 37.21927638 |

| ENSMUSG00000021592 | Arsk | 5671 | 2.398458178 | 2.989995267 | 3.05373371 | 2.827982297 | 3.106380544 | 2.656327104 | 2.486189088 | 3.74448523 | 5.599506904 | 3.40057091 | 3.403470856 | 3.07079646 | 4.166811563 | 2.293130747 | 2.688163583 | 2.741313103 | 3.811678973 | 3.821828258 |
| --- | --- | --- | --- | --- | --- | --- | --- | --- | --- | --- | --- | --- | --- | --- | --- | --- | --- | --- | --- | --- |
| ENSMUSG00000021594 | Srd5a1 | 3013 | 5.826298606 | 4.488893147 | 5.153635328 | 5.050577102 | 5.655061725 | 3.387939966 | 13.06476896 | 9.405497041 | 12.97245385 | 11.75246537 | 11.86049417 | 12.61548829 | 13.2948944 | 14.39720465 | 12.82677047 | 19.58374577 | 16.94345419 | 15.92032561 |
| ENSMUSG00000021595 | Nsun2 | 5857 | 20.71192972 | 19.79321699 | 18.82248176 | 21.60201812 | 20.33095085 | 20.75343272 | 22.63447607 | 25.63331635 | 20.7796063 | 25.3987483 | 25.40874094 | 22.42346834 | 24.05531087 | 24.47096699 | 28.88400008 | 26.76485346 | 26.39063606 | 27.82857832 |
| ENSMUSG00000021596 | Mctp1 | 8173 | 0.005200682 | 0 | 0.029593497 | 0 | 0.023556551 | 0 | 0 | 0 | 0 | 0.005769082 | 0 | 0.081951297 | 0 | 0 | 0 | 0 | 0 | 0 |
| ENSMUSG00000021597 | Ankrd32 | 5919 | 2.491856071 | 2.656476782 | 1.855176785 | 2.062909255 | 2.374475707 | 2.327362719 | 2.933561947 | 3.071394415 | 2.38671649 | 2.931484845 | 2.381080039 | 3.262750339 | 3.550497848 | 5.500473942 | 2.916616307 | 3.887929557 | 3.360592683 | 3.355007185 |
| ENSMUSG00000021598 | Med10 | 858 | 0 | 0.038826217 | 0 | 0 | 0 | 0.231015075 | 0.110285965 | 0.04451329 | 0 | 0.0549542 | 0.11136354 | 0 | 0 | 0 | 0 | 0.044627748 | 0 | 0 |
| ENSMUSG00000021600 | 8030423J24R | 866 | 0 | 0 | 0 | 0 | 0 | 0 | 0 | 0 | 0 | 0 | 0 | 0 | 0 | 0 | 0 | 0 | 0 | 0 |
| ENSMUSG00000021604 | Irx4 | 2672 | 0 | 0 | 0.036207732 | 0 | 0 | 0 | 0 | 0 | 0 | 0 | 0 | 0.020889095 | 0 | 0 | 0 | 0 | 0 | 0 |
| ENSMUSG00000021606 | Ndufs6 | 1203 | 104.6553046 | 90.91124831 | 96.34495323 | 81.19940071 | 101.9052449 | 93.33873174 | 118.4193485 | 105.9418511 | 112.7750481 | 100.4157122 | 108.8935463 | 96.64507495 | 121.2589189 | 135.6646077 | 103.0552632 | 98.06596958 | 103.8601229 | 121.7242245 |
| ENSMUSG00000021607 | Mrpl36 | 897 | 58.94809251 | 51.47343521 | 53.49670247 | 51.91013078 | 53.98073045 | 47.45350964 | 66.67026328 | 59.73683453 | 74.29184226 | 64.49711583 | 65.56407345 | 62.16259412 | 61.15617838 | 69.22602546 | 54.43014909 | 50.45651945 | 58.27989852 | 54.70247848 |
| ENSMUSG00000021608 | Lpcat1 | 4403 | 3.958010941 | 4.418516971 | 4.669259687 | 4.387436475 | 4.219605391 | 4.490470292 | 6.2646586 | 6.739835719 | 6.781790581 | 7.613933685 | 4.13405841 | 5.996095481 | 3.260410613 | 4.208764676 | 3.780474354 | 5.043959185 | 4.186906384 | 3.510687938 |
| ENSMUSG00000021609 | Slc6a3 | 3441 | 0.07411539 | 0.067768166 | 0 | 0.013240641 | 0 | 0 | 0.164996265 | 0 | 0 | 0 | 0 | 0 | 0 | 0 | 0 | 0 | 0 | 0 |
| ENSMUSG00000021610 | Clptm1l | 2221 | 123.6114057 | 125.1220908 | 122.4257605 | 127.5545131 | 132.5199069 | 121.0148703 | 115.9490821 | 116.8298883 | 106.5545788 | 119.2248309 | 123.0619259 | 124.045975 | 143.3979549 | 134.2926291 | 139.5398935 | 125.2332157 | 137.4121127 | 141.4484505 |
| ENSMUSG00000021611 | Tert | 3454 | 1.009097983 | 1.099499114 | 0.812294372 | 1.081646102 | 0.710691403 | 0.817749222 | 1.260210325 | 1.183146224 | 0.357728857 | 0.682552161 | 0.719252415 | 0.549430379 | 0.557019332 | 0.201696963 | 0.882720074 | 0.421263197 | 0.687964797 | 0.366302679 |
| ENSMUSG00000021612 | Slc6a18 | 4392 | 0.02903359 | 0.015169806 | 0.044056039 | 0 | 0.021917998 | 0.022564997 | 0.075407275 | 0.104350826 | 0.356349635 | 0 | 0.054388614 | 0 | 0.011232222 | 0.042298805 | 0 | 0 | 0.008726388 | 0.46341916 |
| ENSMUSG00000021613 | Hapln1 | 5050 | 0 | 0 | 0 | 0 | 0 | 0 | 0 | 0 | 0 | 0 | 0 | 0 | 0 | 0 | 0 | 0 | 0 | 0 |
| ENSMUSG00000021614 | Vcan | 16350 | 0 | 0.024449831 | 0 | 0 | 0 | 0 | 0 | 0.049054462 | 0.002519053 | 0.00576767 | 0.061362332 | 0.003413802 | 0.05732761 | 0.005681234 | 0 | 0.002341933 | 0.01172058 | 0 |
| ENSMUSG00000021615 | Xrcc4 | 4201 | 5.149996334 | 5.416021602 | 5.492543199 | 5.910680591 | 5.969229286 | 6.440321955 | 6.194233137 | 4.82746148 | 5.460816301 | 5.724079662 | 5.185760787 | 5.194935509 | 4.967246134 | 5.560908345 | 6.433758787 | 7.082076161 | 7.709050436 | 5.329375491 |
| ENSMUSG00000021619 | Atg10 | 1601 | 33.50501696 | 32.39736184 | 32.45045072 | 29.82384414 | 32.13805522 | 29.03214497 | 40.57483333 | 33.08735924 | 38.4853389 | 37.43194493 | 34.82409528 | 35.00245174 | 31.12132195 | 30.40187607 | 32.52889859 | 35.54018901 | 39.54718456 | 34.18742111 |
| ENSMUSG00000021620 | Acot12 | 3404 | 0.012486832 | 0 | 0 | 0 | 0 | 0 | 0 | 0 | 0 | 0 | 0 | 0 | 0.028984675 | 0 | 0 | 0 | 0 | 0 |
| ENSMUSG00000021621 | Zcchc9 | 2246 | 12.58501427 | 15.11390882 | 14.62405481 | 15.27491825 | 18.17269424 | 13.14934515 | 12.89196772 | 13.55269133 | 13.84497747 | 12.74286593 | 12.99666057 | 12.89774216 | 12.40985452 | 11.43525393 | 15.95964097 | 14.66158609 | 15.39195005 | 13.86249083 |
| ENSMUSG00000021622 | Ckmt2 | 1476 | 0.143987724 | 0.067709135 | 0 | 0 | 0 | 0 | 0 | 0 | 0 | 0 | 0 | 0 | 0 | 0 | 0.055828667 | 0 | 0 | 0 |
| ENSMUSG00000021624 | Cd180 | 2840 | 0.044899834 | 0 | 0 | 0 | 0 | 0 | 0.399825456 | 0.013448029 | 0.014502294 | 0.01660236 | 0.050466505 | 0 | 0.469000622 | 0.212596175 | 0 | 0.026965216 | 0 | 0.038738808 |
| ENSMUSG00000021629 | Slc30a5 | 3776 | 16.04075102 | 13.02167158 | 17.75575028 | 16.8802704 | 14.64607529 | 17.72927518 | 17.60442635 | 16.39562614 | 14.45236583 | 17.74394845 | 19.00370435 | 18.22582414 | 13.36508246 | 14.21858184 | 15.4287606 | 16.33637938 | 15.33660874 | 14.46611308 |
| ENSMUSG00000021631 | Mrps36-ps1 | 624 | 0.749289962 | 1.281265162 | 0.697695149 | 0.584115953 | 0.771344931 | 0.635291456 | 0.454929605 | 1.162909689 | 0.726044348 | 0.528934171 | 0.689061901 | 0.805033595 | 1.027748277 | 0.744296272 | 0.660281344 | 0.245452612 | 1.2898273 | 0.705244958 |
| ENSMUSG00000021635 | Rad17 | 2829 | 10.69766184 | 11.06897157 | 12.17460361 | 13.20609981 | 9.119374721 | 11.00004125 | 13.98140671 | 11.13776316 | 10.58416294 | 12.65018868 | 10.77427484 | 10.63437332 | 11.35209549 | 9.686112252 | 10.04915997 | 10.46258025 | 10.30976032 | 9.527911734 |
| ENSMUSG00000021636 | Marveld2 | 3695 | 20.57963732 | 19.47384344 | 19.37559538 | 23.18126207 | 22.47024853 | 17.7021944 | 16.14649205 | 14.85317517 | 14.1672697 | 14.955514 | 15.11472976 | 15.18125599 | 14.97981358 | 14.89478851 | 14.84148062 | 19.95878481 | 17.60209089 | 14.32172143 |
| ENSMUSG00000021638 | Ocln | 5326 | 23.69468037 | 27.37086465 | 22.24310474 | 27.77632575 | 26.67770159 | 27.3163398 | 18.16643417 | 22.03628475 | 16.08485783 | 21.20276649 | 19.34858908 | 23.30717867 | 14.42166639 | 17.08298295 | 20.70908095 | 23.22886829 | 24.58898921 | 17.93011815 |
| ENSMUSG00000021639 | Gtf2h2 | 4354 | 6.765293291 | 5.837790145 | 5.877261727 | 6.843574413 | 6.190601115 | 6.043293875 | 7.573940563 | 6.122714028 | 7.586491879 | 5.815325597 | 6.770130772 | 7.12758576 | 7.13805877 | 7.680234938 | 4.8450153 | 8.891089592 | 6.21459936 | 8.073224436 |
| ENSMUSG00000021640 | Naip1 | 5338 | 0 | 0 | 0 | 0.008535227 | 0 | 0 | 0.017726744 | 0.07154815 | 0.038578602 | 0.061831196 | 0.02684992 | 0 | 0 | 0 | 0 | 0 | 0 | 0 |
| ENSMUSG00000021643 | Serf1 | 1323 | 10.76283747 | 8.334518508 | 9.360259842 | 7.886227629 | 10.33217409 | 9.43861592 | 12.55234339 | 9.122297173 | 11.08269056 | 7.448599381 | 9.533325048 | 9.408082205 | 9.172828156 | 9.408174921 | 7.349635064 | 6.367296771 | 4.055692794 | 6.652650851 |
| ENSMUSG00000021645 | Smn1 | 2479 | 11.19640175 | 10.68323957 | 10.84940818 | 8.693172235 | 9.610852043 | 10.53420355 | 10.63056158 | 9.813860562 | 8.75566511 | 11.16476919 | 10.13700208 | 10.04186588 | 11.18375858 | 11.65317562 | 9.623114506 | 10.48782671 | 10.55944364 | 10.20741795 |
| ENSMUSG00000021646 | Mccc2 | 2213 | 19.28386658 | 21.69176709 | 20.48169812 | 22.48199748 | 20.55339714 | 19.36878199 | 25.76221333 | 23.64373759 | 22.91034835 | 24.5873979 | 27.2012868 | 23.75885863 | 24.07522398 | 22.26713815 | 27.01466679 | 24.65617512 | 23.36293471 | 28.80955928 |
| ENSMUSG00000021647 | Cartpt | 871 | 0 | 0.152986885 | 0 | 0 | 0 | 0 | 0 | 0 | 0 | 0 | 0 | 0 | 0 | 0 | 0.047303738 | 0.175846647 | 0 | 0 |
| ENSMUSG00000021650 | Ptcd2 | 1665 | 22.08226863 | 18.96734157 | 27.04850256 | 20.85136083 | 22.46156438 | 19.91037764 | 22.16449824 | 22.80074954 | 23.15350075 | 23.02313619 | 27.0007423 | 15.85634138 | 21.59938 | 21.08812617 | 21.57823227 | 20.467652 | 21.10823679 | 15.39594218 |
| ENSMUSG00000021660 | Btf3 | 2037 | 87.99426967 | 74.62284552 | 73.37958213 | 68.15144923 | 72.72953414 | 67.23797526 | 95.02021085 | 85.36573792 | 95.59639005 | 86.45452954 | 75.35637781 | 80.20247638 | 104.3792653 | 99.13544034 | 89.46219033 | 104.1760168 | 91.36597828 | 96.94781205 |
| ENSMUSG00000021661 | Ankra2 | 3721 | 11.67435902 | 11.11921919 | 12.02513184 | 10.51785463 | 12.8834711 | 11.42602672 | 9.968594539 | 12.42972301 | 14.84308456 | 12.29136849 | 11.67090493 | 12.49514833 | 11.05692529 | 7.90086195 | 10.9287653 | 10.77405696 | 9.640799221 | 8.840485305 |
| ENSMUSG00000021662 | Rgnef | 6395 | 13.85156948 | 16.54965832 | 12.63990136 | 12.74569325 | 13.18641599 | 13.5524208 | 8.596924615 | 8.803065074 | 9.628434881 | 8.538000684 | 9.749229213 | 10.21177877 | 10.71493872 | 9.680995855 | 9.812348651 | 13.84928458 | 10.99146654 | 8.128789031 |
| ENSMUSG00000021665 | Hexb | 1890 | 346.6533245 | 309.6512834 | 358.0153135 | 378.5664763 | 365.6498201 | 369.2071927 | 490.2745062 | 493.005313 | 537.7128447 | 605.1755342 | 590.3619869 | 531.7253994 | 510.1285668 | 404.260149 | 499.4544164 | 557.1384675 | 582.5394345 | 502.7366669 |
| ENSMUSG00000021666 | Gfm2 | 10405 | 4.297495933 | 4.178118881 | 4.453805101 | 4.418173353 | 3.945846313 | 2.971735296 | 5.597492336 | 5.645354627 | 5.973133329 | 5.628176208 | 6.249083949 | 5.058545874 | 4.826515309 | 4.74037682 | 5.409065387 | 5.163067966 | 5.53990875 | 5.149338774 |
| ENSMUSG00000021668 | Polk | 4510 | 6.47473524 | 8.738171588 | 7.304295825 | 7.69789707 | 7.662687626 | 9.284270482 | 6.609088187 | 7.494517989 | 8.538667902 | 7.893299548 | 9.279570649 | 8.984960319 | 9.450726503 | 7.146828525 | 8.185497578 | 7.938296656 | 11.07298556 | 9.562558685 |

| ENSMUSG00000021669 | Col4a3bp | 5345 | 10.16307109 | 8.843965743 | 9.357947687 | 8.524049455 | 9.104092578 | 8.102729494 | 9.170427571 | 7.659916815 | 8.684228861 | 9.386033352 | 8.679042972 | 9.857808322 | 9.035724418 | 6.73417544 | 8.54864836 | 9.735628711 | 10.23229667 | 10.76511237 |
| --- | --- | --- | --- | --- | --- | --- | --- | --- | --- | --- | --- | --- | --- | --- | --- | --- | --- | --- | --- | --- |
| ENSMUSG00000021670 | Hmgcr | 7397 | 7.004705905 | 8.673872417 | 8.030646921 | 8.629312304 | 8.550134879 | 8.715439555 | 6.767177816 | 8.75167258 | 7.700547695 | 8.484194408 | 7.266030592 | 7.892819125 | 7.336096932 | 10.17161303 | 7.976291474 | 8.732763916 | 10.53882492 | 11.02859339 |
| ENSMUSG00000021671 | Poc5 | 3020 | 7.459517647 | 6.739794162 | 7.288065004 | 6.954848159 | 7.761671134 | 5.988989323 | 9.008208736 | 8.346683969 | 7.869079336 | 8.290405109 | 6.217072411 | 8.372349388 | 6.942405577 | 4.905848966 | 7.503594616 | 10.3460714 | 4.911349967 | 5.28233144 |
| ENSMUSG00000021675 | F2rl2 | 2500 | 0.119014493 | 0.066625788 | 0 | 0.018224418 | 0.077011078 | 0.039642187 | 0 | 0 | 0.115322244 | 0.018860281 | 0.1146599 | 0 | 0 | 0 | 0 | 0 | 0 | 0 |
| ENSMUSG00000021676 | Iqgap2 | 6683 | 15.85596347 | 15.2781716 | 13.85409802 | 15.51652505 | 12.38768647 | 15.9565289 | 3.122084604 | 3.61179086 | 2.052238478 | 3.400664218 | 2.452014179 | 2.371935983 | 2.554069037 | 2.752036302 | 2.891445416 | 2.016802904 | 3.400792169 | 2.255348682 |
| ENSMUSG00000021678 | F2rl1 | 2752 | 32.03333397 | 30.11718053 | 29.35457692 | 34.13767203 | 30.67710542 | 34.78774756 | 25.23801332 | 30.51783899 | 27.74702043 | 30.53145102 | 28.69622324 | 31.19349166 | 20.05901724 | 14.85130701 | 25.90068739 | 20.31405771 | 24.48315042 | 18.98933553 |
| ENSMUSG00000021679 | S100z | 298 | 3.423235653 | 0.782517649 | 1.785600114 | 1.070225874 | 0.161516522 | 0.665137363 | 4.445486611 | 2.050598787 | 1.24388806 | 0.474671509 | 2.565098442 | 1.498407049 | 0.331086693 | 0 | 1.659123056 | 1.027935769 | 0.128611735 | 0 |
| ENSMUSG00000021680 | Crhbp | 1679 | 0.202526151 | 0 | 0.144054587 | 0.217086572 | 0 | 0 | 0.056358164 | 0.272965354 | 0 | 0.252743496 | 0 | 0 | 0 | 0 | 0 | 0.136833618 | 0 | 0 |
| ENSMUSG00000021681 | Aggf1 | 3683 | 21.73153583 | 23.60756283 | 25.32287986 | 23.04649079 | 21.91616508 | 22.34783885 | 18.96104103 | 21.4553572 | 18.30636066 | 21.17492891 | 20.28781837 | 18.11015932 | 20.1319228 | 19.23085345 | 20.30432363 | 19.82627975 | 19.93841566 | 16.05614709 |
| ENSMUSG00000021684 | Pde8b | 7602 | 3.377154212 | 3.496933647 | 3.340713422 | 3.380219548 | 3.311364914 | 3.037565621 | 6.914546999 | 5.963480881 | 7.503725905 | 6.450503997 | 5.85717723 | 6.490534823 | 8.111667534 | 8.290532306 | 7.620282502 | 8.184982508 | 7.280080601 | 7.64859587 |
| ENSMUSG00000021685 | Otp | 1179 | 0 | 0 | 0 | 0 | 0 | 0 | 0 | 0 | 0 | 0 | 0 | 0 | 0 | 0 | 0 | 0 | 0 | 0 |
| ENSMUSG00000021686 | Ap3b1 | 3993 | 27.80453789 | 27.68983118 | 27.45164533 | 28.81082819 | 31.11156899 | 34.31337549 | 24.68126977 | 26.37026132 | 22.45505755 | 25.94292388 | 26.57354941 | 20.59015051 | 24.27678874 | 25.93797017 | 27.66375441 | 27.01343377 | 27.58571933 | 25.65162953 |
| ENSMUSG00000021687 | Scamp1 | 5360 | 62.19554022 | 69.2112668 | 66.90167547 | 73.03367405 | 67.64510839 | 70.60340044 | 48.30130207 | 46.68593667 | 45.81231473 | 54.19691843 | 51.02829054 | 50.40071025 | 35.99573292 | 46.6953707 | 50.12599738 | 43.45555314 | 50.4319724 | 50.42159353 |
| ENSMUSG00000021690 | Jmy | 4547 | 7.291409127 | 7.619399601 | 6.063978954 | 7.324636774 | 7.981409819 | 7.432365142 | 5.67086211 | 6.282805586 | 5.887669946 | 8.01572325 | 5.379542279 | 5.732552106 | 5.674201173 | 6.608604473 | 6.478801949 | 7.090541331 | 6.861140462 | 7.839432847 |
| ENSMUSG00000021692 | Dimt1 | 2533 | 6.628323936 | 6.154928738 | 5.99656081 | 6.025641474 | 4.921503446 | 5.555853271 | 4.389451065 | 6.468432938 | 7.447068385 | 6.422026302 | 6.771105446 | 6.610619335 | 5.161057275 | 5.720708751 | 6.164780767 | 8.631637121 | 6.143101675 | 6.297923786 |
| ENSMUSG00000021693 | Kif2a | 8659 | 3.573596044 | 2.096723334 | 1.99996811 | 1.757407184 | 2.401315513 | 2.146007055 | 1.748476413 | 2.245055182 | 1.612452806 | 1.660811236 | 1.909012084 | 1.985359057 | 2.882775164 | 2.51556496 | 2.050799237 | 2.746098533 | 2.190959345 | 1.658087176 |
| ENSMUSG00000021694 | Ercc8 | 6434 | 1.261810479 | 1.444559759 | 1.511202922 | 1.479990405 | 1.398922867 | 1.28618379 | 0.853010686 | 2.19633026 | 1.952422646 | 1.275135587 | 1.8340713 | 1.474768828 | 1.947514297 | 1.147747885 | 1.127055306 | 1.225965981 | 1.876404029 | 1.633002702 |
| ENSMUSG00000021696 | Elovl7 | 5427 | 37.74321785 | 39.32235127 | 38.66673571 | 37.1238139 | 35.80404935 | 40.96067124 | 29.49305193 | 29.61371463 | 28.31525526 | 30.66924314 | 31.40100919 | 35.34889116 | 23.45243165 | 15.87502895 | 20.08832078 | 31.09392055 | 27.88843679 | 22.70506708 |
| ENSMUSG00000021697 | Depdc1b | 3437 | 0 | 0 | 0 | 0 | 0 | 0 | 0 | 0.1333456 | 0 | 0.041155691 | 0 | 0 | 0 | 0 | 0 | 0.033422119 | 0.089208721 | 0.048014932 |
| ENSMUSG00000021699 | Pde4d | 12032 | 0.480444144 | 0.57865649 | 1.021183237 | 0.458185369 | 0.516042067 | 0.700130046 | 0.232001999 | 0.269810024 | 0.35942355 | 0.419308947 | 0.289858043 | 0.292252888 | 0.147602146 | 0.131241603 | 0.082183955 | 0.178214263 | 0.184751099 | 0.246882627 |
| ENSMUSG00000021700 | Rab3c | 10722 | 0 | 0.003106966 | 0 | 0 | 0 | 0 | 0.119142169 | 0.292088882 | 0.230478544 | 0.074758623 | 0.017823152 | 0.156171412 | 0.920199912 | 1.667690137 | 0.618676599 | 0.221415562 | 0.339582 | 0.69774655 |
| ENSMUSG00000021701 | Plk2 | 2788 | 6.967311852 | 10.59847101 | 9.525490733 | 7.631638345 | 10.16847311 | 7.695958981 | 2.104294185 | 1.794549754 | 1.226140892 | 2.790482797 | 1.182378815 | 1.321317694 | 6.122253722 | 9.562017992 | 6.753626629 | 8.913416142 | 8.660533375 | 11.12809763 |
| ENSMUSG00000021702 | Thbs4 | 3267 | 0 | 0 | 0 | 0 | 0 | 0 | 0 | 0 | 0 | 0.057729664 | 0 | 0 | 0 | 0 | 0 | 0 | 0 | 0 |
| ENSMUSG00000021703 | Serinc5 | 5304 | 13.23077783 | 14.84760217 | 16.63524121 | 16.41537627 | 14.67370297 | 16.83522359 | 4.888263207 | 9.591304676 | 9.753066327 | 7.680657544 | 9.016352458 | 8.629118273 | 9.514809837 | 9.054145241 | 10.71209381 | 7.25529043 | 12.36355468 | 10.0808544 |
| ENSMUSG00000021704 | Mtx3 | 6266 | 1.105704388 | 1.275948709 | 1.806480387 | 0.945249882 | 1.751369071 | 1.178320668 | 1.260966706 | 1.560366265 | 1.866736431 | 1.008329754 | 1.707882333 | 1.888432887 | 1.157323945 | 0.741207906 | 1.643853969 | 1.448272256 | 1.694284111 | 1.79969149 |
| ENSMUSG00000021706 | Zfyve16 | 7840 | 3.757153952 | 3.743451506 | 4.948414712 | 5.009390334 | 4.868445851 | 4.316902683 | 5.244224233 | 4.569448145 | 3.782435121 | 4.41436431 | 5.527026446 | 4.990660647 | 4.605990235 | 5.568551294 | 5.985787265 | 4.444445502 | 5.866269934 | 3.950271312 |
| ENSMUSG00000021707 | Dhfr | 5284 | 1.35945775 | 1.349159607 | 1.107720888 | 1.103674049 | 1.129515241 | 1.247258434 | 1.772882367 | 1.539549908 | 1.138007438 | 1.748966283 | 0.95839243 | 1.066877729 | 2.025932258 | 1.116275378 | 1.52049648 | 1.739164606 | 0.935672275 | 1.301313085 |
| ENSMUSG00000021708 | Rasgrf2 | 8867 | 0.139015462 | 0.172819796 | 0.103653668 | 0.046244434 | 0.043425667 | 0.095003549 | 0.101380501 | 0.090452289 | 0.009289842 | 0.015952646 | 0.021551803 | 0.025179052 | 0.584171796 | 0.141422168 | 0.408902325 | 0.544109229 | 0.566228137 | 0.303986267 |
| ENSMUSG00000021709 | Erbb2ip | 7763 | 19.00495504 | 17.41385093 | 16.65624715 | 16.94967101 | 20.26215723 | 18.21125195 | 15.49866579 | 17.16517996 | 15.88994135 | 17.74756601 | 14.8623631 | 17.13367563 | 15.16878611 | 13.13213601 | 14.37781977 | 16.97255959 | 20.844213 | 13.11630253 |
| ENSMUSG00000021710 | Nln | 5552 | 15.74038939 | 16.15243358 | 16.92899306 | 15.22257515 | 16.54968341 | 17.30597089 | 12.1264305 | 14.20520731 | 12.81885793 | 13.66453198 | 13.67334456 | 14.10471445 | 13.01715757 | 11.59429127 | 11.50259575 | 13.10377415 | 15.8289263 | 12.81101855 |
| ENSMUSG00000021711 | 2410002O22R | 7223 | 13.44653568 | 14.72168882 | 14.25153989 | 14.57095562 | 13.38737846 | 14.159884 | 14.4368191 | 13.35650124 | 14.58605944 | 14.14586376 | 15.1665485 | 13.07491362 | 11.91123684 | 11.67692963 | 11.94462938 | 12.04964013 | 14.95802727 | 12.9621348 |
| ENSMUSG00000021712 | Trim23 | 5858 | 4.629276597 | 7.034491319 | 5.714319393 | 7.349809986 | 5.841891043 | 7.13938326 | 7.018558892 | 6.506660567 | 5.969162151 | 6.157440763 | 7.030046809 | 7.098441213 | 6.955985604 | 7.809393322 | 6.674680188 | 7.621517281 | 10.06245214 | 7.183674743 |
| ENSMUSG00000021713 | Ppwd1 | 2386 | 3.064078071 | 2.708592405 | 2.14903362 | 2.176847885 | 2.057609478 | 2.159884448 | 2.439002309 | 3.393457381 | 2.89997261 | 1.699480503 | 2.402764048 | 3.789663596 | 3.05998481 | 3.873584824 | 3.522681225 | 3.354038955 | 2.570078585 | 2.582154213 |
| ENSMUSG00000021714 | Cenpk | 1865 | 1.481413642 | 0.785934234 | 1.478440338 | 1.245905234 | 1.161360088 | 0.478256946 | 0.989380417 | 1.699715496 | 1.634210277 | 1.137684529 | 1.972478178 | 1.167190799 | 1.666440101 | 2.116754653 | 1.303427237 | 1.375587505 | 2.40438431 | 0.914360487 |
| ENSMUSG00000021715 | Cwc27 | 2856 | 11.19183907 | 12.81893233 | 11.73769487 | 10.25761607 | 9.909513696 | 8.866052822 | 11.49684845 | 10.350462 | 12.9068388 | 10.74758677 | 13.29870168 | 12.29273521 | 10.48475973 | 8.732659289 | 9.10300482 | 11.1278726 | 11.84948185 | 9.380054263 |
| ENSMUSG00000021716 | Srek1ip1 | 3248 | 6.477851642 | 6.482065624 | 7.089224276 | 5.821377278 | 6.579610255 | 6.529732134 | 7.050288362 | 5.92640727 | 7.34205438 | 6.968084225 | 7.869335833 | 7.801819832 | 9.249734489 | 8.365083474 | 7.484272776 | 5.882701076 | 7.457579939 | 5.97851437 |
| ENSMUSG00000021718 | 4933425L06R | 2149 | 0 | 0 | 0 | 0 | 0 | 0 | 0 | 0 | 0.019165433 | 0.043881529 | 0 | 0 | 0.298424814 | 0.151283672 | 0 | 0 | 0 | 0.102390147 |
| ENSMUSG00000021719 | Rgs7bp | 5822 | 3.752603998 | 4.302867821 | 2.86651803 | 2.958102844 | 3.13328737 | 2.842771043 | 1.665939399 | 1.331682873 | 0.834766207 | 1.401077235 | 1.723246527 | 1.725664594 | 2.948730198 | 3.629690787 | 2.611366218 | 3.077980808 | 3.120347773 | 3.316419866 |
| ENSMUSG00000021720 | Rnf180 | 1978 | 37.21889023 | 38.93802398 | 41.42808919 | 37.4300794 | 40.78316687 | 39.83258159 | 35.23335493 | 32.01365176 | 39.04181853 | 33.58712886 | 35.02208786 | 39.22334226 | 26.36189917 | 29.1625665 | 32.09888657 | 23.34604273 | 28.01811189 | 28.03295226 |
| ENSMUSG00000021721 | Htr1a | 4440 | 0 | 0 | 0 | 0 | 0.010840523 | 0 | 0 | 0 | 0 | 0 | 0 | 0 | 0 | 0 | 0.046398149 | 0 | 0 | 0 |
| ENSMUSG00000021725 | Parp8 | 3306 | 10.954147 | 11.10429807 | 9.803468039 | 11.1628693 | 9.900093192 | 9.997481336 | 10.44714326 | 13.47015765 | 10.20319311 | 7.858450543 | 7.50006154 | 11.09222333 | 5.625569514 | 7.853068618 | 8.374907912 | 6.89138276 | 9.656928409 | 8.569174217 |

| ENSMUSG00000021728 | Emb | 2244 | 14.77452643 | 17.33933175 | 9.312551298 | 17.86707621 | 15.97962706 | 19.10120969 | 39.40614836 | 51.80823214 | 36.39610551 | 51.03790473 | 49.49945564 | 39.22517821 | 90.7276393 | 111.0394514 | 72.69026728 | 78.91892123 | 83.96260052 | 83.42067299 |
| --- | --- | --- | --- | --- | --- | --- | --- | --- | --- | --- | --- | --- | --- | --- | --- | --- | --- | --- | --- | --- |
| ENSMUSG00000021730 | Hcn1 | 3517 | 0 | 0.189439262 | 0 | 0.077727116 | 0 | 0 | 0 | 0 | 0 | 0 | 0 | 0 | 0 | 0 | 0 | 0 | 0 | 0 |
| ENSMUSG00000021731 | Mrps30 | 1585 | 32.71691783 | 32.57727826 | 32.99166329 | 34.03424384 | 31.73366576 | 36.10940523 | 38.56654964 | 33.90328718 | 31.52002752 | 35.48945677 | 37.97883135 | 35.0036395 | 33.95654369 | 28.07156828 | 28.07424629 | 34.0387797 | 32.13605589 | 33.73429132 |
| ENSMUSG00000021732 | Fgf10 | 4725 | 0 | 0 | 0 | 0 | 0 | 0 | 0 | 0 | 0 | 0 | 0 | 0 | 0 | 0 | 0 | 0 | 0 | 0 |
| ENSMUSG00000021733 | Slc4a7 | 7756 | 2.263362261 | 2.349426655 | 1.602887739 | 1.539065706 | 1.849318367 | 2.10835509 | 1.067524344 | 0.738635941 | 0.785921136 | 1.404307949 | 0.819245678 | 0.993110036 | 0.540641177 | 0.634743845 | 0.664027139 | 0.62698648 | 0.810406484 | 0.382992749 |
| ENSMUSG00000021737 | Psmd6 | 2318 | 62.03408564 | 58.93709197 | 56.99228274 | 54.5042174 | 56.66609995 | 54.23437666 | 51.66022018 | 54.4711313 | 50.12388307 | 51.93086515 | 48.39327114 | 51.86666834 | 50.22576565 | 53.15623979 | 50.44435531 | 52.82716241 | 56.19977704 | 54.86671904 |
| ENSMUSG00000021738 | Atxn7 | 6835 | 2.717595015 | 3.665149444 | 2.972477359 | 2.659671791 | 3.140722451 | 2.849191558 | 2.118168215 | 3.140326284 | 3.537159437 | 2.524821857 | 2.334577342 | 2.670332358 | 1.6672528 | 3.139307736 | 1.916912183 | 2.089597156 | 2.11397424 | 2.824900726 |
| ENSMUSG00000021741 | Gm5457 | 335 | 0.634405612 | 0.099441475 | 0.433195794 | 0.816018705 | 0.431032152 | 0.443755823 | 0.423695632 | 0.114007171 | 0.983558585 | 0.70374184 | 0.570447265 | 0.499841755 | 1.325335091 | 0.831834401 | 0.245979438 | 0.571501603 | 0.228813713 | 0.656825155 |
| ENSMUSG00000021743 | Fezf2 | 2299 | 0.221862598 | 0 | 0.042082236 | 0.039635532 | 0 | 0.086216152 | 0 | 0 | 0 | 0.082036891 | 0 | 0 | 0.193121903 | 0 | 0 | 0.016655332 | 0 | 0 |
| ENSMUSG00000021745 | Ptprg | 11663 | 1.61084522 | 2.233617704 | 1.410186086 | 2.187617665 | 1.469173011 | 1.538033915 | 0.26773873 | 0.805567263 | 0.81574939 | 1.047074693 | 0.729138524 | 0.875783782 | 0.313003676 | 0.410163852 | 0.65707703 | 0.617219771 | 0.575075191 | 0.749931233 |
| ENSMUSG00000021747 | 4930452B06R | 3262 | 0.104243228 | 0.153186209 | 0.563517521 | 0.432983561 | 0.073776707 | 0.151909055 | 0.188554514 | 0.456622837 | 0.012626154 | 0.086727229 | 0.410085481 | 0.205330457 | 0.181478543 | 0.626468377 | 0.846261264 | 0.281721207 | 0.986943268 | 0.269817813 |
| ENSMUSG00000021748 | Pdhb | 1475 | 143.9412571 | 138.3557898 | 140.3324315 | 141.4709038 | 142.1441759 | 146.8440668 | 146.2362734 | 153.9089084 | 147.9643031 | 153.1518775 | 160.1027931 | 137.9687497 | 144.3502068 | 119.7470267 | 135.3925026 | 141.5843883 | 146.8871569 | 135.4902269 |
| ENSMUSG00000021749 | Oit1 | 1241 | 0.102752239 | 0.590558963 | 0.389794765 | 0.03671317 | 0.426632684 | 1.038171695 | 0.571869608 | 0.584734606 | 0 | 0.531917684 | 0.230982877 | 0.809574477 | 0.159006986 | 0.598795647 | 0.564404875 | 0.092563918 | 0.370600776 | 0.842202279 |
| ENSMUSG00000021750 | Fam107a | 5105 | 0 | 0 | 0 | 0.008924788 | 0.037713554 | 0.048533529 | 0 | 0.052369602 | 0 | 0.018472362 | 0 | 0.032800585 | 0.077307608 | 0.318421755 | 0.064566591 | 0.195015826 | 0.510516785 | 0 |
| ENSMUSG00000021751 | Acox2 | 3686 | 0 | 0.081339134 | 0.052494336 | 0 | 0.091406258 | 0.188208972 | 0.269551345 | 0.093253289 | 0 | 0.243044862 | 0.051844773 | 0.030285221 | 0.133835912 | 0.34020357 | 0.167667753 | 0.103881192 | 0.207956033 | 0 |
| ENSMUSG00000021752 | Kctd6 | 3249 | 10.75384878 | 11.53493567 | 9.990347451 | 12.25618737 | 11.58484589 | 11.39301077 | 11.60609575 | 10.72067436 | 10.38219649 | 11.42123837 | 10.67547549 | 13.77782745 | 10.76525988 | 13.53725785 | 10.24649343 | 12.23319498 | 12.46872756 | 12.41048791 |
| ENSMUSG00000021754 | Map3k1 | 8315 | 8.010295951 | 7.055202251 | 8.580993058 | 7.413601081 | 7.953489251 | 8.35513319 | 7.351531109 | 10.13717764 | 9.693567209 | 8.415110479 | 9.342403185 | 9.390993621 | 7.546626431 | 6.105037585 | 7.170012192 | 8.454787154 | 9.287731601 | 8.031395738 |
| ENSMUSG00000021756 | Il6st | 5192 | 14.56408092 | 18.90853222 | 15.46611333 | 16.53255153 | 17.38200248 | 18.64908348 | 10.5341789 | 16.69814204 | 9.630283154 | 12.89560836 | 15.66120557 | 12.99713715 | 9.89108742 | 11.40527955 | 13.12545713 | 11.69663007 | 15.39843131 | 9.726186435 |
| ENSMUSG00000021758 | Ddx4 | 2827 | 0.33077958 | 0.011783832 | 0 | 0 | 0 | 0 | 0 | 0.04052961 | 0 | 0.116750945 | 0 | 0.059231336 | 0.139602171 | 0.016428754 | 0 | 0 | 0 | 0 |
| ENSMUSG00000021759 | Ppap2a | 1971 | 3.234792696 | 2.873258253 | 1.398930102 | 2.057297284 | 2.759465943 | 2.162118259 | 1.488273005 | 1.666436635 | 1.253775213 | 1.818089014 | 1.672487131 | 1.925654518 | 0.500577547 | 2.521317783 | 1.964965121 | 2.836341289 | 0.855584507 | 1.255915679 |
| ENSMUSG00000021760 | Gpx8 | 1013 | 46.65918632 | 41.17250105 | 48.27802487 | 37.91506454 | 47.08661042 | 43.58488215 | 81.87475435 | 67.75098433 | 90.09804435 | 71.2613294 | 57.11300564 | 73.99845494 | 43.48805738 | 48.09461764 | 47.13978606 | 43.8848916 | 52.81689089 | 47.29805721 |
| ENSMUSG00000021763 | BC067074 | 8397 | 0.870655029 | 0.99577664 | 1.399877084 | 1.24252461 | 1.249584299 | 1.274668388 | 0.602888728 | 0.682250847 | 0.711211717 | 0.875968764 | 0.961530068 | 0.505179273 | 0.264372547 | 0.298675803 | 0.240428277 | 0.369362772 | 0.246471363 | 0.340654228 |
| ENSMUSG00000021764 | Ndufs4 | 1529 | 118.5641437 | 104.2961574 | 102.5366984 | 100.8958117 | 117.5124075 | 106.5594428 | 133.0262961 | 119.7228154 | 110.9530794 | 111.2929287 | 117.1095777 | 101.2639948 | 117.8287521 | 117.5226776 | 118.2692144 | 128.7706365 | 121.9473083 | 118.9045767 |
| ENSMUSG00000021765 | Fst | 2680 | 0.047580421 | 0.024860369 | 0.018049825 | 0 | 0 | 0.018489826 | 0.070615939 | 0.156759861 | 0.015368103 | 0 | 0 | 0 | 0.018407432 | 0.05198965 | 0 | 0.04286262 | 0.028601714 | 0.020525786 |
| ENSMUSG00000021767 | Myst4 | 8404 | 4.501381088 | 3.190966188 | 3.280927214 | 3.138962955 | 3.310358386 | 2.853822353 | 4.047812488 | 4.094640001 | 3.131625842 | 3.085779009 | 2.586578547 | 3.148099008 | 3.69813278 | 3.188748028 | 4.010337068 | 3.722444818 | 4.03602722 | 2.93896822 |
| ENSMUSG00000021768 | Dusp13 | 2758 | 0.046234782 | 0.024157284 | 0.017539351 | 0 | 0 | 0 | 0.017154706 | 0 | 0 | 0.068383906 | 0.01732232 | 0.080950925 | 0.017886845 | 0.033679541 | 0.014938925 | 0.027766938 | 0.027792819 | 0.219398178 |
| ENSMUSG00000021770 | Samd8 | 7863 | 3.405603573 | 3.512195003 | 2.737660054 | 3.691006644 | 2.877019474 | 3.503919607 | 3.231197836 | 2.982339448 | 3.01709692 | 3.747830286 | 3.311376367 | 2.917491711 | 4.2474511 | 3.437677586 | 3.075837887 | 3.686377948 | 4.547683455 | 2.735414057 |
| ENSMUSG00000021771 | Vdac2 | 4828 | 75.73985695 | 69.46166241 | 75.1953905 | 66.47307297 | 71.13116723 | 73.55932924 | 81.88582128 | 78.49693643 | 82.8848772 | 74.89617715 | 79.46000758 | 74.11644839 | 78.24851336 | 73.99501247 | 70.98478498 | 80.15014051 | 75.73174657 | 76.00781917 |
| ENSMUSG00000021772 | Nkiras1 | 5316 | 8.795277207 | 5.903074935 | 6.306030199 | 6.676458529 | 6.410346493 | 6.124180956 | 7.582844704 | 6.509088897 | 6.190373607 | 6.953753076 | 6.156103566 | 5.963750254 | 5.966971935 | 5.09347309 | 6.36314473 | 6.55463746 | 7.21682152 | 7.181399561 |
| ENSMUSG00000021773 | Comtd1 | 2706 | 1.03671161 | 1.071035402 | 0.48266272 | 0.723992944 | 0.853781351 | 0.714174652 | 0.874218014 | 0.719812462 | 0.791462978 | 0.662131088 | 0.441379882 | 0.701305443 | 0.747453292 | 1.613357064 | 0.883107998 | 0.735813594 | 0.835643576 | 1.93121402 |
| ENSMUSG00000021774 | Ube2e1 | 4450 | 9.714104274 | 8.129843398 | 9.739928809 | 7.566204891 | 7.874166389 | 8.774731249 | 7.431811814 | 7.544072295 | 9.375716959 | 8.232830659 | 6.452303381 | 8.315906583 | 8.003965005 | 8.2138195 | 7.286657188 | 7.06440195 | 7.794449145 | 6.58873121 |
| ENSMUSG00000021775 | Nr1d2 | 5458 | 15.87908646 | 20.80682602 | 21.84696818 | 22.11271646 | 22.16114404 | 23.88663284 | 17.86578074 | 21.94418724 | 18.92557375 | 21.23423023 | 20.7800937 | 19.06199983 | 13.42211381 | 17.12083251 | 20.03461512 | 16.23387056 | 22.72332297 | 18.30277351 |
| ENSMUSG00000021779 | Thrb | 6608 | 3.505647841 | 2.787837546 | 2.049725863 | 2.433875401 | 3.204910172 | 2.969564543 | 1.890215986 | 3.213525384 | 3.440520082 | 3.810301988 | 4.034265562 | 2.407303849 | 3.613294183 | 4.870725266 | 4.065286689 | 5.771405111 | 4.686387394 | 5.985403713 |
| ENSMUSG00000021782 | Dlg5 | 10193 | 5.200034779 | 6.078876018 | 4.930844505 | 4.653099888 | 5.642857724 | 5.522604272 | 3.513754336 | 5.073335903 | 5.08315872 | 3.733014572 | 3.2762382 | 4.895438276 | 5.53671278 | 4.720501769 | 4.696969287 | 5.439497647 | 4.643674745 | 4.020581233 |
| ENSMUSG00000021785 | Ngly1 | 4019 | 9.88861398 | 8.446339689 | 9.80951163 | 9.352540825 | 9.377281969 | 10.13494576 | 10.88933267 | 8.828251265 | 12.43076477 | 11.08669186 | 8.725259896 | 8.582751799 | 11.24360195 | 9.845822955 | 9.000986831 | 13.85283483 | 12.25411583 | 12.00372894 |
| ENSMUSG00000021786 | Oxsm | 5229 | 1.284340756 | 1.987688467 | 1.52641662 | 0.914880408 | 1.702888866 | 1.279330471 | 1.112920158 | 2.096236277 | 1.496545801 | 1.262401694 | 2.156223026 | 1.174167696 | 1.169852313 | 1.980690665 | 0.914014244 | 1.025183598 | 0.916195661 | 1.91464093 |
| ENSMUSG00000021789 | Sftpa1 | 2898 | 0 | 0 | 0 | 0 | 0 | 0 | 0 | 0 | 0 | 0 | 0 | 0 | 0 | 0 | 0 | 0 | 0.039675256 | 0 |
| ENSMUSG00000021790 | Dydc1 | 1081 | 0 | 0 | 0.044748872 | 0.295029889 | 0 | 0.183358866 | 0 | 0 | 0.038100385 | 0.043617672 | 0 | 0 | 0 | 0.042964003 | 0 | 0 | 0 | 0 |
| ENSMUSG00000021791 | Dydc2 | 749 | 1.134984674 | 0.62267092 | 0.839593985 | 0.851608305 | 0.321307902 | 0.727743751 | 0 | 0.152973574 | 0.329932035 | 0.188854619 | 0 | 0.074520244 | 0 | 0.496065019 | 0.38506127 | 0 | 0.307019735 | 0.587547201 |
| ENSMUSG00000021792 | 5730469M10R | 3049 | 61.31117224 | 65.57690754 | 58.33698626 | 65.50987811 | 69.6484248 | 57.12622451 | 71.4578178 | 73.00272916 | 78.91493115 | 82.79595449 | 76.38665877 | 63.88870529 | 86.70703335 | 85.80503256 | 78.09242096 | 94.37648889 | 83.55358987 | 85.71605971 |
| ENSMUSG00000021794 | Glud1 | 4074 | 26.36489441 | 34.40042857 | 29.20934823 | 32.12981845 | 34.33268782 | 32.29320266 | 28.85910828 | 30.77703905 | 26.1535386 | 30.12598934 | 30.81801445 | 31.62065519 | 22.20783905 | 27.28048628 | 28.69141239 | 27.17185714 | 28.30727231 | 27.85561786 |

| ENSMUSG00000021795 | Sftpd | 1725 | 0.41889159 | 0 | 0 | 0.0264122 | 0 | 0.315988446 | 0 | 0 | 0 | 0 | 0 | 0 | 0 | 0 | 0.16719472 | 0 | 0 | 0 |
| --- | --- | --- | --- | --- | --- | --- | --- | --- | --- | --- | --- | --- | --- | --- | --- | --- | --- | --- | --- | --- |
| ENSMUSG00000021796 | Bmpr1a | 6520 | 18.39717895 | 20.71837209 | 22.53969098 | 21.06150117 | 21.51910391 | 23.08914181 | 17.37217076 | 17.46189441 | 16.83467247 | 17.65258691 | 16.66261589 | 18.40547156 | 16.22959548 | 14.63131219 | 17.84558188 | 19.60338152 | 19.41591391 | 17.05113569 |
| ENSMUSG00000021797 | 9230112D13R | 777 | 0 | 0 | 0 | 0 | 0 | 0.063774432 | 0.060891479 | 0 | 0 | 0 | 0.245945732 | 0 | 0 | 0.836830403 | 0 | 0.098560122 | 0.197303974 | 0.283187165 |
| ENSMUSG00000021798 | Ldb3 | 5999 | 0 | 0.027765373 | 0 | 0.007594773 | 0.016046649 | 0.008260166 | 0.007886761 | 0.019099384 | 0.006865564 | 0.047158563 | 0 | 0 | 0.008223357 | 0.193549289 | 0 | 0 | 0 | 0.320939946 |
| ENSMUSG00000021799 | Opn4 | 2859 | 0 | 0 | 0 | 0 | 0 | 0 | 0 | 0 | 0 | 0 | 0 | 0 | 0 | 0 | 0 | 0 | 0 | 0 |
| ENSMUSG00000021803 | Cdhr1 | 4286 | 0 | 0 | 0 | 0 | 0 | 0 | 0 | 0.008910966 | 0 | 0 | 0 | 0 | 0 | 0 | 0 | 0 | 0 | 0 |
| ENSMUSG00000021804 | Rgr | 1652 | 0 | 0 | 0 | 0 | 0 | 0 | 0 | 0 | 0 | 0 | 0 | 0 | 0 | 0 | 0 | 0 | 0 | 0 |
| ENSMUSG00000021806 | Nid2 | 4863 | 0 | 0 | 0 | 0 | 0 | 0 | 0 | 0 | 0 | 0 | 0 | 0.022955239 | 0.334763165 | 0.286515036 | 0.076252108 | 0 | 0.102455657 | 0.101805873 |
| ENSMUSG00000021807 | 2700060E02R | 1502 | 54.64546931 | 52.32031788 | 54.10621238 | 42.2546836 | 53.00279743 | 49.51974241 | 53.83313468 | 54.31356294 | 57.11818398 | 49.91319453 | 51.14656008 | 50.35301119 | 58.75818909 | 64.03841876 | 58.15399367 | 56.26322938 | 55.60119904 | 62.55363135 |
| ENSMUSG00000021809 | Nudt13 | 2570 | 5.391707154 | 4.08309793 | 3.76447707 | 6.098443289 | 4.101514118 | 4.318993121 | 6.498589752 | 6.107812213 | 5.673162093 | 5.815864954 | 6.115938264 | 5.950774922 | 4.453309264 | 5.529918576 | 7.166185013 | 6.138416439 | 4.548451578 | 5.286867455 |
| ENSMUSG00000021810 | Ecd | 3111 | 33.76094181 | 39.71633708 | 38.09551571 | 42.99814406 | 42.14444232 | 37.97290802 | 33.22989504 | 38.68346512 | 29.53619951 | 35.6017364 | 40.4344152 | 38.50222176 | 39.75413584 | 36.54616713 | 43.69139598 | 38.93063041 | 49.10595289 | 45.42539221 |
| ENSMUSG00000021811 | Dnajc9 | 1626 | 62.89511287 | 58.28732107 | 53.52028358 | 52.6221656 | 55.02905664 | 53.45356377 | 68.43752819 | 65.25122625 | 74.09013444 | 69.6531299 | 74.27742622 | 68.20770084 | 85.92127289 | 72.95092201 | 79.2357105 | 81.0084191 | 83.34673176 | 79.4012137 |
| ENSMUSG00000021814 | Anxa7 | 2835 | 61.09650523 | 65.61523856 | 68.33720955 | 57.77493982 | 64.37963123 | 63.43099476 | 50.53361263 | 51.05792068 | 59.63691259 | 55.84905169 | 52.25754718 | 57.76478096 | 48.60107406 | 54.91378516 | 56.72298859 | 72.191639 | 70.44738383 | 58.71518763 |
| ENSMUSG00000021815 | Zmynd17 | 1822 | 0.419919412 | 0.164553265 | 0.026549687 | 0.050012123 | 0.264170821 | 0.13598445 | 1.16853488 | 0.565968642 | 0.293866468 | 0.051757084 | 0.131105814 | 0.275708542 | 0.324908346 | 0.688249374 | 0.655787662 | 0.042031402 | 0.231388181 | 0.301916063 |
| ENSMUSG00000021816 | Ppp3cb | 7654 | 5.003548942 | 5.057431811 | 5.864990354 | 5.268032955 | 4.854696247 | 4.052784325 | 4.462993753 | 4.820206523 | 5.413330919 | 4.552439209 | 4.75627363 | 5.068184674 | 4.930613629 | 4.083730182 | 4.010342192 | 5.057721986 | 4.075987154 | 5.368670332 |
| ENSMUSG00000021819 | 2310021P13R | 6046 | 10.95320282 | 11.33938741 | 11.06526505 | 10.85899188 | 9.537222057 | 10.68752309 | 11.1982209 | 10.85887194 | 10.61339589 | 10.89472915 | 10.7545019 | 10.25656651 | 13.38146615 | 12.76713087 | 11.35325705 | 11.87477487 | 9.679830528 | 12.84698291 |
| ENSMUSG00000021820 | Camk2g | 4804 | 2.08809774 | 1.657323422 | 1.903121823 | 1.602792775 | 1.623099841 | 1.609122906 | 1.300015324 | 1.828528841 | 2.126198149 | 2.443906143 | 2.237586523 | 2.149437464 | 2.331046049 | 2.484623326 | 2.169857127 | 1.86511285 | 1.531775397 | 2.46189799 |
| ENSMUSG00000021822 | Plau | 2332 | 0.437446065 | 0.499979116 | 0.518584159 | 0.488433151 | 0.804950696 | 0.382482506 | 1.01442279 | 0.31117309 | 0.883072808 | 0.161751984 | 0.245840267 | 0.359020128 | 0.401932431 | 0.876303536 | 0.017667906 | 0.525428575 | 0.345133891 | 0 |
| ENSMUSG00000021823 | Vcl | 5190 | 6.994107963 | 7.952731394 | 7.801280329 | 8.374804681 | 7.864329725 | 8.048738807 | 4.630990539 | 5.82820476 | 4.721768185 | 5.305589731 | 5.403449061 | 5.205160056 | 4.686056882 | 3.53476195 | 6.779600912 | 4.766037068 | 5.959406859 | 3.932250211 |
| ENSMUSG00000021824 | Ap3m1 | 8568 | 4.534282317 | 5.307201285 | 5.939420394 | 5.179325068 | 5.977166991 | 7.102095802 | 4.649541977 | 5.888441127 | 6.162594912 | 5.90484414 | 5.609431399 | 5.595898011 | 5.262531792 | 4.417825773 | 5.313692724 | 7.132564124 | 6.852927971 | 5.309585815 |
| ENSMUSG00000021830 | Txndc16 | 11246 | 1.810419644 | 2.310515516 | 2.090479793 | 1.58406263 | 1.861763009 | 2.507158581 | 5.755268075 | 4.564341884 | 4.237310932 | 3.245122205 | 3.419779617 | 3.965562369 | 4.627883045 | 5.455507686 | 3.755254738 | 3.115410437 | 4.229320154 | 3.957172981 |
| ENSMUSG00000021831 | Ero1l | 4275 | 7.536590276 | 9.374365321 | 8.237644467 | 9.058921096 | 10.68472411 | 9.516443104 | 10.81274557 | 12.21263487 | 10.27976896 | 12.31984457 | 10.12493857 | 13.34353384 | 9.554813451 | 7.115994676 | 9.522137361 | 7.004270173 | 9.296928629 | 10.461381 |
| ENSMUSG00000021832 | Psmc6 | 2166 | 78.35787991 | 67.33326449 | 68.89766443 | 70.57123903 | 64.79810222 | 69.27316587 | 66.90615677 | 64.30641347 | 62.16007388 | 63.80365247 | 63.34703636 | 62.97944569 | 73.92954916 | 60.83189101 | 62.29690468 | 69.47464778 | 70.67185126 | 65.90426221 |
| ENSMUSG00000021835 | Bmp4 | 3225 | 1.014852265 | 0.237580331 | 0.344989519 | 0.57922568 | 0.83577914 | 0.322668963 | 0.54281213 | 1.065834486 | 0.204336202 | 0.628676043 | 0.592557625 | 0.917280656 | 0.030593437 | 0.014401267 | 0.076654057 | 0.213715018 | 0.1544936 | 0.085285437 |
| ENSMUSG00000021838 | Samd4 | 8714 | 0.009755606 | 0.011468749 | 0.027756214 | 0.005228488 | 0.01657055 | 0.045492526 | 0.005429502 | 0.008765757 | 0.009452953 | 0.010821828 | 0 | 0.025621144 | 0.039628577 | 0.053298241 | 0.028369215 | 0 | 0 | 0.018938182 |
| ENSMUSG00000021840 | Mapk1ip1l | 4726 | 10.9995409 | 13.44921756 | 13.21418275 | 13.0050463 | 11.81401427 | 12.82331637 | 9.400466677 | 10.90172468 | 7.817246009 | 12.03210921 | 10.8064813 | 9.908874505 | 8.528179521 | 9.9452849 | 9.982179749 | 9.536192322 | 12.78083875 | 11.32540433 |
| ENSMUSG00000021843 | Ktn1 | 4442 | 36.33321778 | 37.89510457 | 38.42003942 | 37.19143331 | 41.41382536 | 42.2235697 | 32.35837879 | 34.11694118 | 35.54909983 | 35.96276061 | 31.78185554 | 34.01463274 | 29.36370763 | 32.59032427 | 33.9017755 | 32.3858199 | 38.90438378 | 37.47356077 |
| ENSMUSG00000021846 | Peli2 | 7390 | 1.990093492 | 2.005986188 | 2.160117052 | 2.404439417 | 2.025578926 | 2.165836664 | 1.677391323 | 1.772664956 | 1.432332145 | 2.143793815 | 1.247708659 | 2.092143239 | 1.668874062 | 2.268784242 | 1.711620793 | 2.186554306 | 2.178219851 | 2.754177197 |
| ENSMUSG00000021848 | Otx2 | 4521 | 0 | 0 | 0 | 0 | 0 | 0 | 0 | 0 | 0 | 0 | 0 | 0 | 0 | 0 | 0 | 0 | 0 | 0 |
| ENSMUSG00000021850 | 1700011H14R | 1371 | 0.217021322 | 0.291578943 | 0.635101055 | 0.631407617 | 0.140428661 | 0.108430489 | 3.761547778 | 5.738610427 | 3.725111564 | 1.134918459 | 1.916573827 | 3.745471158 | 2.374840657 | 7.215602198 | 2.013496896 | 1.005442645 | 1.928894592 | 3.932087862 |
| ENSMUSG00000021852 | Slc35f4 | 6477 | 0.039374874 | 0.041146079 | 0.05227956 | 0.03517141 | 0.029724825 | 0 | 0.029218885 | 0.005896619 | 0 | 0 | 0.007376094 | 0.198202909 | 0.08378124 | 0.028682469 | 0 | 0 | 0.011834583 | 0 |
| ENSMUSG00000021866 | Anxa11 | 4095 | 19.01574664 | 20.29686717 | 22.91688618 | 23.48702432 | 21.43898138 | 23.3666248 | 23.67367012 | 22.09470117 | 24.67167843 | 23.75382193 | 27.91830907 | 23.58024329 | 26.53924634 | 25.34860447 | 28.60464552 | 27.76185919 | 27.46015998 | 26.28884539 |
| ENSMUSG00000021867 | Gm9746 | 1597 | 0 | 0 | 0 | 0 | 0 | 0 | 0 | 0 | 0 | 0 | 0 | 0 | 0 | 0 | 0 | 0 | 0 | 0 |
| ENSMUSG00000021868 | Ppif | 3293 | 12.64958169 | 14.68883158 | 15.46836546 | 16.22930611 | 14.32410726 | 16.25173163 | 15.63200567 | 16.5620257 | 17.19752784 | 17.78353278 | 17.13398906 | 16.6616448 | 17.81222279 | 18.70174913 | 19.7812511 | 17.27902903 | 18.92455474 | 21.2819927 |
| ENSMUSG00000021870 | Slmap | 9653 | 10.37420436 | 13.01042279 | 11.14500482 | 11.72891279 | 12.9342391 | 11.99162806 | 8.592056994 | 10.73407104 | 10.7947669 | 9.857051608 | 9.200626521 | 10.63348218 | 8.595906439 | 9.459139729 | 9.249328873 | 8.583950525 | 8.794441898 | 8.889900185 |
| ENSMUSG00000021871 | Pnp | 2936 | 16.08421341 | 15.24949926 | 12.22519057 | 16.85262062 | 12.11494945 | 14.21096787 | 13.76193045 | 15.12866621 | 15.59925256 | 17.52091868 | 16.97182619 | 14.7333578 | 23.00251865 | 21.40287815 | 20.24994726 | 34.56066404 | 24.63273919 | 18.5861832 |
| ENSMUSG00000021872 | Rnase10 | 1662 | 19.48793269 | 70.23368312 | 20.37392975 | 31.58021846 | 13.98779731 | 67.02439536 | 0.085401948 | 0.275757418 | 0.123906485 | 0.085109573 | 0.546163785 | 0 | 0.059364521 | 0 | 0.842871781 | 0.506855212 | 0.922413885 | 0.959846026 |
| ENSMUSG00000021874 | 4933413J09R | 4822 | 0.079333593 | 0.048359656 | 0.090286556 | 0.05669147 | 0.059890407 | 0.020552772 | 0.127553883 | 0 | 0.093955138 | 0 | 0 | 0.05787605 | 0.143228296 | 0.019263412 | 0.076900457 | 0 | 0.047689295 | 0 |
| ENSMUSG00000021876 | Rnase4 | 1993 | 112.6291192 | 91.1967641 | 102.5722726 | 94.3682845 | 106.5036545 | 101.8653033 | 133.2256669 | 115.2479218 | 129.5319021 | 123.2826466 | 117.7234426 | 133.3358603 | 173.9612216 | 155.0855 | 162.5321788 | 242.4815635 | 185.7277348 | 189.5923503 |
| ENSMUSG00000021877 | Arf4 | 2693 | 173.1616362 | 170.1518232 | 172.9807268 | 176.4411925 | 173.0282781 | 174.7134071 | 171.6292464 | 165.6611409 | 172.6988994 | 166.3668705 | 165.1633358 | 170.037763 | 176.5910444 | 167.0814403 | 179.7232369 | 212.4112083 | 205.9060021 | 182.8598304 |
| ENSMUSG00000021879 | Dnahc12 | 11036 | 0 | 0.012074264 | 0.026299491 | 0.037155618 | 0.021806779 | 0 | 0 | 0 | 0.067176267 | 0.004272445 | 0.00432901 | 0 | 0 | 0.092585169 | 0.014933511 | 0.006939218 | 0 | 0 |

| ENSMUSG00000021880 | Rnase6 | 1070 | 3.257406013 | 2.95768687 | 3.345459109 | 2.938048653 | 1.304510081 | 2.454481196 | 4.289093323 | 2.284405378 | 3.156349806 | 3.745616614 | 1.02693836 | 2.243059338 | 4.333831985 | 5.382305454 | 4.197167843 | 3.399633367 | 2.829698556 | 3.136033187 |
| --- | --- | --- | --- | --- | --- | --- | --- | --- | --- | --- | --- | --- | --- | --- | --- | --- | --- | --- | --- | --- |
| ENSMUSG00000021884 | Hacl1 | 4930 | 4.810930674 | 4.284051711 | 5.416265468 | 4.870318532 | 5.184188939 | 5.176401175 | 4.241826387 | 4.17559937 | 4.636615872 | 5.011555478 | 3.740595126 | 4.66451379 | 5.783742025 | 4.74803652 | 6.485275329 | 5.172727085 | 5.193096619 | 4.463213527 |
| ENSMUSG00000021886 | Gpr65 | 2047 | 0 | 0 | 0 | 0 | 0.047026794 | 0 | 0.531603134 | 0.261208419 | 0.020120428 | 0.023034051 | 1.587052846 | 0 | 0 | 0.431088256 | 0.04025555 | 0 | 0 | 0 |
| ENSMUSG00000021890 | Eaf1 | 4232 | 12.53460768 | 15.78269209 | 13.61362823 | 13.10203 | 12.20358084 | 13.81668847 | 10.74373451 | 11.56060196 | 12.48636572 | 12.36703228 | 13.03877057 | 13.20214514 | 13.10233342 | 16.29713369 | 14.73987609 | 11.63556737 | 15.81231436 | 15.76702421 |
| ENSMUSG00000021891 | Mettl6 | 2430 | 5.947226275 | 8.15686093 | 9.017781585 | 6.299798723 | 7.982372526 | 8.299573073 | 8.469553635 | 7.779933823 | 6.864419294 | 6.150935363 | 5.701538255 | 8.016323557 | 8.059576609 | 9.938652445 | 9.410231901 | 9.864164705 | 6.529665398 | 8.149497292 |
| ENSMUSG00000021892 | Sh3bp5 | 3181 | 92.58672263 | 109.6885426 | 104.7003321 | 108.280885 | 109.1101231 | 115.9294069 | 122.4983413 | 161.1020608 | 136.9862737 | 146.1064074 | 141.8980848 | 156.3575509 | 86.27269909 | 76.8422609 | 96.06788433 | 125.3683358 | 121.8344905 | 97.60182281 |
| ENSMUSG00000021893 | Capn7 | 5021 | 16.82090914 | 17.38294819 | 17.75590847 | 18.91949361 | 19.34479625 | 20.09348049 | 13.78578954 | 17.74604159 | 14.56006084 | 17.09107348 | 17.49813755 | 16.6524323 | 14.85557836 | 16.6499417 | 17.71642286 | 15.98428861 | 18.82347105 | 13.45373094 |
| ENSMUSG00000021895 | Arhgef3 | 3758 | 12.6565439 | 13.13722971 | 14.91881897 | 15.32441725 | 14.66499537 | 13.89797264 | 15.12041708 | 15.29525429 | 19.74936174 | 15.23175991 | 12.24249202 | 16.78331525 | 13.02215592 | 13.21147669 | 14.97640376 | 17.43353626 | 17.32740246 | 13.64249267 |
| ENSMUSG00000021898 | Asb14 | 2609 | 0 | 0.051073813 | 0.092705118 | 0.069852119 | 0.055345255 | 0.037985997 | 0 | 0.014638713 | 0 | 0.072289311 | 0 | 0.085574032 | 0 | 0 | 0 | 0.117410831 | 0.029380067 | 0.168674915 |
| ENSMUSG00000021900 | Btd | 2427 | 19.52751185 | 20.65756316 | 17.73895427 | 21.36319261 | 21.75555017 | 19.33516222 | 11.91102053 | 14.60344023 | 9.825707716 | 13.6964342 | 15.15727978 | 13.84467609 | 13.00882862 | 9.702164115 | 13.80175729 | 15.11429827 | 12.71226576 | 13.78060852 |
| ENSMUSG00000021901 | Bap1 | 3389 | 26.04994117 | 24.87900126 | 28.47600857 | 27.4387995 | 26.37385136 | 29.50646691 | 20.94099097 | 25.75085261 | 25.42407524 | 26.25357836 | 27.03817361 | 25.98911642 | 22.82456367 | 23.88670532 | 31.35403147 | 29.28570504 | 30.78317503 | 29.3630788 |
| ENSMUSG00000021902 | Phf7 | 2314 | 4.261538824 | 4.750758467 | 2.884851854 | 4.351335695 | 5.033675682 | 4.43276398 | 4.579965462 | 5.199052186 | 7.84928844 | 4.869930025 | 5.037636071 | 5.813126484 | 3.922676222 | 5.158224053 | 4.807441699 | 5.559915336 | 5.068213851 | 5.206134128 |
| ENSMUSG00000021903 | Galntl2 | 6937 | 0.643367952 | 0.513835906 | 0.655486789 | 0.321823724 | 0.562013236 | 0.614319603 | 0.190969441 | 0.264269182 | 0.124681682 | 0.11554879 | 0.447653496 | 0.072414727 | 0.675584855 | 0.461963677 | 0.225696861 | 0.23734988 | 0.292820201 | 0.491648352 |
| ENSMUSG00000021904 | Sema3g | 4522 | 0 | 0 | 0 | 0 | 0 | 0 | 0 | 0 | 0 | 0 | 0 | 0 | 0 | 0 | 0 | 0 | 0 | 0 |
| ENSMUSG00000021905 | Dph3 | 4252 | 14.66488552 | 12.49625265 | 12.50294211 | 13.27613686 | 14.13846958 | 11.26940107 | 13.49724354 | 16.82369937 | 12.69885281 | 13.37341207 | 16.42685543 | 14.1902002 | 14.53736885 | 14.57112243 | 14.31201742 | 13.50797533 | 13.25015439 | 14.77431794 |
| ENSMUSG00000021906 | Oxnad1 | 5157 | 7.08007489 | 7.693553813 | 6.434795777 | 5.875139516 | 7.298655092 | 7.139360296 | 8.779956125 | 7.08747898 | 6.069759934 | 9.636773557 | 8.031973822 | 7.31653828 | 8.944220022 | 9.582413997 | 7.717801627 | 8.382799255 | 8.331157423 | 5.8667847 |
| ENSMUSG00000021907 | Msmb | 581 | 0 | 0 | 0 | 0 | 0 | 0 | 0 | 0 | 0 | 0.405771973 | 0 | 0 | 0 | 0 | 0 | 0 | 0 | 0 |
| ENSMUSG00000021908 | Gm6768 | 1849 | 0.229881969 | 0.306284046 | 0.078485988 | 0.468177308 | 0.286344597 | 0.348396721 | 0.30705903 | 0.247868485 | 0.400950397 | 0.408010412 | 0.129191343 | 0.513178077 | 0.373524522 | 0.251184897 | 0.289681031 | 0.310632294 | 0.373106189 | 0.178504403 |
| ENSMUSG00000021909 | Tnnc1 | 1081 | 0 | 0 | 0 | 0 | 0 | 0 | 0 | 0 | 0 | 0 | 0 | 0 | 0 | 0 | 0 | 0 | 0 | 0 |
| ENSMUSG00000021910 | Nisch | 10589 | 36.53221334 | 41.7001051 | 40.54806454 | 41.78759681 | 38.64099378 | 41.86876073 | 33.58221691 | 36.9588769 | 34.90099216 | 35.60904466 | 33.97801608 | 34.58894871 | 33.07274348 | 35.60611025 | 35.3183986 | 35.76297169 | 35.92298581 | 37.86064499 |
| ENSMUSG00000021911 | Parg | 5990 | 13.59597951 | 14.65433661 | 15.59420486 | 14.71796674 | 15.78148549 | 16.53688054 | 12.86683706 | 13.14736791 | 10.69199199 | 12.50005288 | 11.26180991 | 11.39608603 | 6.448562809 | 10.1339603 | 10.55145021 | 9.67173439 | 10.51893691 | 11.94772084 |
| ENSMUSG00000021913 | Ogdhl | 3425 | 13.21693795 | 14.26861776 | 14.98520167 | 12.71718493 | 13.25208876 | 17.81004819 | 13.82773478 | 12.79027316 | 8.670212515 | 11.41253518 | 11.18701218 | 10.4134915 | 11.4507662 | 16.42154448 | 16.13176247 | 11.24681782 | 12.74559188 | 14.24615406 |
| ENSMUSG00000021916 | Glt8d1 | 2510 | 15.32557144 | 12.95353974 | 12.87391166 | 15.75577151 | 13.86429514 | 14.7276252 | 14.85354223 | 14.75961368 | 16.99969336 | 13.7882933 | 16.654617 | 12.96435509 | 13.75790522 | 16.98632359 | 14.74063633 | 13.89750731 | 14.75028 | 14.99052948 |
| ENSMUSG00000021917 | Spcs1 | 1765 | 111.6934881 | 94.86153334 | 103.3521717 | 93.98740081 | 104.6630725 | 98.74048952 | 115.426853 | 106.116454 | 113.7354549 | 114.9515445 | 105.9981515 | 95.18705064 | 132.5672995 | 132.8853492 | 124.1882591 | 127.1725442 | 120.9069808 | 125.1340303 |
| ENSMUSG00000021918 | Nek4 | 4375 | 3.478137833 | 4.195521078 | 4.444836389 | 3.644883547 | 4.114591875 | 3.65840753 | 5.028661875 | 5.683029478 | 5.375428686 | 5.216214943 | 4.335236232 | 5.154177757 | 4.194622452 | 3.651832243 | 4.106029341 | 4.524855778 | 3.530399463 | 6.802268968 |
| ENSMUSG00000021919 | Chat | 3059 | 0 | 0 | 0 | 0 | 0 | 0 | 0 | 0 | 0 | 0 | 0 | 0 | 0 | 0 | 0 | 0 | 0 | 0 |
| ENSMUSG00000021922 | Itih4 | 3411 | 0 | 0.019532626 | 0.070908136 | 0.013357093 | 0.084664773 | 0.174327999 | 0.152576801 | 0.033590503 | 0.036223849 | 0.013823132 | 0.322141321 | 0.098180585 | 0.274789337 | 0.13615974 | 0.096632204 | 0.112256252 | 0.134833059 | 0 |
| ENSMUSG00000021927 | Gm5803 | 962 | 0 | 0 | 0 | 0 | 0 | 0 | 0 | 0 | 0 | 0 | 0 | 0 | 0 | 0 | 0.04282906 | 0 | 0.039840225 | 0 |
| ENSMUSG00000021928 | Ebpl | 2073 | 19.35595088 | 15.00928278 | 17.87463784 | 18.72552329 | 16.92627706 | 16.90003986 | 17.80216573 | 18.12895512 | 19.80846899 | 19.60632234 | 20.21159604 | 24.28640987 | 18.6333291 | 17.47534402 | 18.38467882 | 20.02268134 | 16.78739874 | 17.43414526 |
| ENSMUSG00000021929 | Kpna3 | 4152 | 20.4847922 | 17.62727086 | 16.63713905 | 17.65600201 | 19.08120095 | 21.47046428 | 15.96461059 | 19.20658387 | 19.02594827 | 16.17114678 | 16.0285446 | 17.36845763 | 14.47164625 | 12.14794771 | 17.36578102 | 14.06386712 | 19.23699488 | 18.29662244 |
| ENSMUSG00000021930 | 6330409N04R | 4933 | 9.469529385 | 8.434584407 | 8.129263463 | 8.764936362 | 8.72287447 | 8.247069586 | 6.531509092 | 6.480243425 | 5.952966904 | 8.267861001 | 7.941509417 | 8.044787371 | 7.210280226 | 8.360500629 | 6.422865696 | 8.926454188 | 8.45307339 | 9.445107114 |
| ENSMUSG00000021932 | Rnaseh2b | 4460 | 3.840714336 | 4.063276784 | 3.67682215 | 4.116838759 | 3.388660097 | 4.488633266 | 3.967475767 | 3.9819433 | 2.973555622 | 3.774170642 | 3.299257222 | 3.854534546 | 3.849216864 | 3.36355162 | 2.364932356 | 4.155303583 | 3.583422827 | 2.146095195 |
| ENSMUSG00000021933 | Gucy1b2 | 2902 | 0 | 0 | 0 | 0.015699877 | 0 | 0 | 0 | 0 | 0 | 0 | 0 | 0 | 0.118994976 | 0 | 0 | 0 | 0.026413713 | 0 |
| ENSMUSG00000021936 | Mapk8 | 10517 | 1.770207008 | 1.48873826 | 1.665039269 | 1.187004483 | 1.299749579 | 1.281576831 | 1.947930967 | 1.4707543 | 1.578222483 | 1.165653974 | 1.903366702 | 2.191867324 | 1.2711752 | 1.024534399 | 1.257554383 | 1.004869035 | 1.508708464 | 1.705141085 |
| ENSMUSG00000021938 | Pspc1 | 3994 | 13.87750364 | 10.82627358 | 13.21370096 | 11.47581638 | 13.83461401 | 13.25045555 | 18.38489677 | 12.75630065 | 14.70505044 | 13.61160763 | 12.09326065 | 15.62391356 | 14.8341594 | 12.31454395 | 12.29650842 | 17.52509523 | 15.74698378 | 13.63520672 |
| ENSMUSG00000021939 | Ctsb | 4728 | 134.5098231 | 145.8566462 | 143.2278028 | 155.6379922 | 152.1937942 | 160.4175423 | 125.0263559 | 131.5812273 | 129.4744467 | 134.94994 | 141.9708474 | 128.4775499 | 121.0447488 | 120.1377303 | 131.0467423 | 120.9053464 | 134.7256884 | 137.6856533 |
| ENSMUSG00000021940 | Ptpn20 | 3118 | 0 | 0 | 0 | 0 | 0 | 0 | 0 | 0 | 0 | 0 | 0 | 0 | 0 | 0 | 0 | 0 | 0 | 0 |
| ENSMUSG00000021943 | Gdf10 | 4344 | 0 | 0 | 0 | 0.02097654 | 0 | 0 | 0 | 0 | 0 | 0 | 0 | 0 | 0 | 0 | 0 | 0 | 0.061759687 | 0 |
| ENSMUSG00000021944 | Gata4 | 5513 | 0 | 0 | 0 | 0 | 0 | 0 | 0 | 0 | 0 | 0 | 0 | 0 | 0 | 0 | 0 | 0 | 0 | 0 |
| ENSMUSG00000021945 | Zmym2 | 4979 | 16.00666902 | 12.66545667 | 15.77799022 | 13.607004 | 13.19543599 | 15.29675668 | 13.35093671 | 11.79753262 | 12.54042135 | 13.76925538 | 14.08588854 | 13.68767303 | 12.25619234 | 10.91375422 | 12.70222701 | 11.72789241 | 14.19435459 | 10.90459697 |
| ENSMUSG00000021947 | Cryl1 | 1989 | 8.419828736 | 7.352619689 | 8.633787469 | 7.123923976 | 9.413433035 | 7.748064426 | 13.10673006 | 8.371989667 | 10.20862356 | 9.766762062 | 8.262737917 | 8.811522399 | 11.23547437 | 13.05291345 | 12.4081106 | 8.432019126 | 10.34752209 | 6.444002949 |

| ENSMUSG00000021948 | Prkcd |  | 5632 | 172.352753 | 186.4682156 | 186.2879084 | 190.6250797 | 184.4772202 | 185.0572879 | 180.4803967 | 177.3654625 | 176.0811304 | 185.0363713 | 202.7721249 | 180.1422761 | 162.8426268 | 158.472306 | 167.0009087 | 163.0206293 | 173.1828711 | 169.1295618 |
| --- | --- | --- | --- | --- | --- | --- | --- | --- | --- | --- | --- | --- | --- | --- | --- | --- | --- | --- | --- | --- | --- |
| ENSMUSG00000021950 | Anxa8 |  | 1919 | 0 | 0 | 0.050415352 | 0.142452458 | 0.175572416 | 0 | 0 | 0 | 0.042924977 | 0.073711365 | 0 | 0 | 0.025707096 | 0.024202234 | 0.085881305 | 0.718323015 | 0 | 0.286655064 |
| ENSMUSG00000021951 | N6amt2 |  | 831 | 41.43103801 | 32.43096441 | 43.48378721 | 32.07365937 | 41.23938586 | 35.89740748 | 48.67911009 | 41.59341056 | 42.5742684 | 31.43380217 | 36.33426445 | 35.06110213 | 43.27673609 | 39.29024481 | 35.00396926 | 35.94064233 | 33.29914123 | 39.25439264 |
| ENSMUSG00000021952 | Xpo4 |  | 7083 | 3.126527843 | 3.42394282 | 3.681114339 | 3.132603218 | 4.682040859 | 3.386068481 | 2.818855077 | 3.769093503 | 3.727312806 | 2.735978969 | 3.777209763 | 2.82900224 | 2.820757659 | 2.67530533 | 2.86194628 | 3.421989903 | 3.706552785 | 3.642421439 |
| ENSMUSG00000021953 | Tdh |  | 2799 | 0 | 0 | 0 | 0 | 0 | 0 | 0 | 0 | 0 | 0 | 0 | 0 | 0 | 0 | 0 | 0 | 0 | 0 |
| ENSMUSG00000021957 | Tkt |  | 6518 | 20.43091692 | 23.49995207 | 24.23871589 | 23.30477475 | 22.53737819 | 21.2108203 | 18.45179961 | 21.10015972 | 20.04983346 | 21.55708741 | 21.73254862 | 19.16469053 | 22.66028848 | 24.28374499 | 24.53888309 | 21.96510909 | 23.1263157 | 25.74912314 |
| ENSMUSG00000021958 | Pinx1 |  | 1344 | 11.44856676 | 9.840192712 | 11.98540596 | 10.74616894 | 11.88973114 | 10.1022686 | 6.934968563 | 9.519683635 | 8.917616136 | 10.45454581 | 9.597647909 | 7.433782443 | 10.71794631 | 10.4015404 | 11.46531392 | 12.05128492 | 10.72223782 | 13.26112394 |
| ENSMUSG00000021959 | Lats2 |  | 6618 | 3.468237391 | 4.691389757 | 3.844738133 | 3.696929708 | 3.745533498 | 4.477566437 | 2.223367051 | 2.198746648 | 1.655426593 | 3.035086066 | 2.187339441 | 2.884399608 | 3.00404392 | 2.301852624 | 2.994552491 | 3.164847726 | 3.932238682 | 3.017271946 |
| ENSMUSG00000021961 | 4930578I06Ri | k | 1029 | 0.123921796 | 0.194244281 | 0.141030701 | 0.177108044 | 0.280652616 | 0.433405833 | 0 | 0.185580187 | 0.080051537 | 0 | 0.232142655 | 0.108485253 | 0.239708053 | 0.090270335 | 0.280282693 | 0.186057373 | 0.22347695 | 0.213835206 |
| ENSMUSG00000021962 | Dcp1a |  | 5606 | 4.443102596 | 5.24710767 | 4.530164723 | 4.551228118 | 3.597444883 | 4.958809051 | 4.464574947 | 4.993762212 | 2.990173371 | 4.314718297 | 4.618984569 | 3.673917141 | 2.32315843 | 3.463009013 | 4.130444954 | 3.900095759 | 5.599221756 | 4.307705646 |
| ENSMUSG00000021963 | Sap18 |  | 3842 | 24.38350025 | 20.73168404 | 21.71898487 | 21.77253446 | 23.70265476 | 20.85548417 | 24.48141742 | 23.03274242 | 27.79714611 | 23.57535163 | 22.55694292 | 21.15242184 | 25.19240544 | 24.84190515 | 23.36756645 | 24.67661216 | 21.79670972 | 25.50005702 |
| ENSMUSG00000021965 | F630043A04R |  | 2251 | 1.680568932 | 0.887949202 | 0.472775508 | 0.283365003 | 0.85529851 | 0.506314026 | 0.315277736 | 0.610806969 | 0.475721639 | 0.14662591 | 0.573044815 | 0.421530992 | 1.599835611 | 1.052264974 | 0.6223247 | 0.06804195 | 1.958029384 | 1.099693382 |
| ENSMUSG00000021966 | Prss52 |  | 1059 | 0 | 0 | 0 | 0.043022705 | 0 | 0 | 0 | 0 | 0 | 0.044523799 | 0 | 0 | 0.046583491 | 0 | 0.350154866 | 0 | 0 | 0 |
| ENSMUSG00000021967 | Mrp63 |  | 2319 | 34.45870243 | 26.50379036 | 27.09668647 | 27.8199391 | 25.28015654 | 28.20595443 | 28.542664 | 26.91090364 | 30.56575836 | 27.20467484 | 29.91342808 | 26.35539048 | 37.03616558 | 33.52626231 | 30.86119128 | 26.83149505 | 25.84835203 | 29.88851853 |
| ENSMUSG00000021969 | Zdhhc20 |  | 5195 | 27.11494771 | 28.72155018 | 27.11524935 | 29.26606448 | 29.62035803 | 28.95901812 | 22.09442908 | 26.73835756 | 23.38791559 | 24.16984077 | 23.87368474 | 25.19494298 | 21.84088734 | 21.25074027 | 24.36403844 | 23.14388975 | 26.58126039 | 24.22730244 |
| ENSMUSG00000021972 | Hmbox1 |  | 9685 | 1.558011099 | 2.273600731 | 2.09277328 | 1.825264347 | 1.86862192 | 1.719124262 | 1.294564782 | 1.778500102 | 1.667022631 | 1.3242118 | 1.909025187 | 1.619432234 | 1.283597641 | 1.640049343 | 1.761222936 | 1.656558028 | 1.804521568 | 1.90274143 |
| ENSMUSG00000021973 | Efha1 |  | 2298 | 75.26264633 | 75.74406975 | 77.67551219 | 77.81858095 | 87.96957328 | 78.05957161 | 73.97495883 | 74.90563866 | 74.32570969 | 79.83611243 | 82.28602509 | 72.13773624 | 69.59707389 | 74.51668851 | 73.67153749 | 67.6167471 | 79.48787252 | 72.81884363 |
| ENSMUSG00000021974 | Fgf9 |  | 4260 | 0.897996677 | 0.312797129 | 1.226371192 | 0.652399931 | 0.915184464 | 0.569972757 | 1.232795155 | 2.402714518 | 1.904634649 | 2.73385533 | 1.570069058 | 1.650885795 | 2.814003732 | 1.722574133 | 2.137451608 | 3.37065206 | 3.022919192 | 2.104808544 |
| ENSMUSG00000021975 | Ints9 |  | 4178 | 9.583503068 | 10.70828553 | 10.00352566 | 11.01403896 | 9.481228621 | 10.93528491 | 8.946150396 | 7.870669811 | 7.47232622 | 8.249680249 | 8.404642049 | 8.376357214 | 6.564994257 | 7.314554692 | 7.445470248 | 6.928602009 | 8.760558531 | 7.294170686 |
| ENSMUSG00000021977 | 1700129C05R |  | 1350 | 0 | 0 | 0 | 0 | 0 | 0 | 0 | 0 | 0 | 0 | 0 | 0 | 0 | 0 | 0 | 0 | 0 | 0 |
| ENSMUSG00000021978 | Extl3 |  | 6441 | 4.46103074 | 3.713500706 | 4.45357918 | 3.451915795 | 4.707826721 | 4.177477773 | 2.335884474 | 3.783069824 | 3.600063402 | 3.169733661 | 2.929841422 | 3.492270148 | 4.074612636 | 3.518819228 | 3.33271396 | 3.442052738 | 3.344104776 | 3.014782592 |
| ENSMUSG00000021981 | Cab39l |  | 3112 | 41.41249798 | 41.99437147 | 42.38901583 | 44.55085409 | 42.61036302 | 42.65802483 | 32.9455576 | 34.92788472 | 33.92064264 | 44.15075491 | 36.61416323 | 33.41406793 | 35.66735664 | 31.74375767 | 34.82008097 | 33.99644867 | 36.58979052 | 30.5625789 |
| ENSMUSG00000021982 | Cdadc1 |  | 1889 | 25.9666348 | 25.8179339 | 27.06766627 | 31.69254222 | 28.10455892 | 26.17979254 | 30.13083788 | 31.23730107 | 26.4474556 | 34.67036889 | 30.02058005 | 27.8635097 | 23.94778621 | 24.09486799 | 31.1465441 | 32.04735326 | 28.72950578 | 26.67461184 |
| ENSMUSG00000021983 | Atp8a2 |  | 6221 | 0 | 0.021419639 | 0.054430913 | 0.043942496 | 0 | 0.015930794 | 0 | 0.018417812 | 0 | 0 | 0.053757388 | 0.008972137 | 0 | 0 | 0 | 0 | 0 | 0.336014476 |
| ENSMUSG00000021986 | Fam123a |  | 3479 | 0 | 0.162782179 | 0 | 0.052384069 | 0 | 0.085460305 | 0.108796042 | 0 | 0 | 0.081317683 | 0 | 0 | 0 | 0 | 0 | 0 | 0 | 0 |
| ENSMUSG00000021987 | Mtmr6 |  | 3799 | 13.85138403 | 17.51141083 | 13.76461867 | 14.90718034 | 14.91215429 | 17.77846167 | 15.89128147 | 15.07991672 | 14.0721499 | 13.54077843 | 14.68837892 | 12.98790359 | 9.985850062 | 17.8856804 | 17.64541496 | 13.71769325 | 15.25384599 | 16.95595261 |
| ENSMUSG00000021990 | Spata13 |  | 9054 | 5.276763624 | 5.268838582 | 5.706089068 | 7.487832336 | 5.448997323 | 5.659103879 | 5.037488678 | 6.892686716 | 4.644514313 | 7.54077958 | 4.828151867 | 7.015487522 | 3.661480938 | 3.606162297 | 5.073971153 | 5.603606672 | 5.570731911 | 3.694006725 |
| ENSMUSG00000021991 | Cacna2d3 |  | 3653 | 0.139628282 | 0.09119325 | 0.132421381 | 0.149666721 | 0 | 0.054259768 | 0.194275988 | 0.125460944 | 0 | 0.090351745 | 0 | 0 | 0.58069325 | 1.449391175 | 0.225576545 | 0 | 0.146884248 | 0.75293056 |
| ENSMUSG00000021993 | Mipep |  | 3847 | 9.02696356 | 7.793502677 | 6.752426774 | 8.645584186 | 7.506928568 | 7.844454055 | 8.842686808 | 7.565014461 | 8.671972386 | 8.898209141 | 8.929086339 | 6.427433981 | 6.578443869 | 5.130942849 | 9.531943006 | 7.375445824 | 8.239107756 | 6.03427165 |
| ENSMUSG00000021994 | Wnt5a |  | 7797 | 0.223510609 | 0.081178016 | 0.093061813 | 0.309700571 | 0.277791018 | 0.063553589 | 0.03034031 | 0.225323908 | 0.116211793 | 0.241891513 | 0.09191027 | 0.007158607 | 0.54412529 | 1.250898852 | 0.824348175 | 0.23081423 | 0.412903545 | 0.599688864 |
| ENSMUSG00000021996 | Esd |  | 2384 | 72.63678152 | 68.9314208 | 64.78887686 | 60.58242892 | 63.55675155 | 64.58068088 | 75.77173161 | 66.22793269 | 63.17914771 | 63.82354002 | 64.14750088 | 66.07038583 | 79.27457009 | 70.6402101 | 71.04848837 | 70.20480073 | 65.04538476 | 74.18384149 |
| ENSMUSG00000021997 | Lrrc63 |  | 2509 | 0.169410825 | 0 | 0 | 0 | 0 | 0 | 0 | 0.015222161 | 0 | 0 | 0 | 0 | 0 | 0 | 0 | 0 | 0 | 0 |
| ENSMUSG00000021998 | Lcp1 |  | 9526 | 0.629144428 | 1.182002755 | 0.523039432 | 0.468715342 | 0.323372151 | 0.998753396 | 1.316172571 | 1.238867556 | 0.756628203 | 0.193037731 | 1.128423857 | 0.791005086 | 0.761263053 | 1.306636093 | 1.051015965 | 0.426076463 | 0.016093343 | 0.421547847 |
| ENSMUSG00000021999 | Cpb2 |  | 1462 | 0 | 0 | 0 | 0 | 0.032921972 | 0 | 0 | 0 | 0 | 0.032250823 | 0 | 0 | 0.101228284 | 0 | 0.112726555 | 0 | 0 | 0 |
| ENSMUSG00000022000 | Zc3h13 |  | 6396 | 11.47028359 | 8.661560833 | 8.576545251 | 7.700357869 | 8.669164488 | 9.978724335 | 7.759693589 | 7.440233489 | 8.049272155 | 8.094351497 | 7.91020654 | 7.443833675 | 7.828626647 | 8.880725278 | 6.261399676 | 7.609030958 | 8.586864206 | 7.663088507 |
| ENSMUSG00000022002 | 4930564B18R |  | 864 | 0 | 0 | 0 | 0 | 0 | 0 | 0 | 0 | 0 | 0 | 0 | 0 | 0 | 0 | 0 | 0 | 0 | 0 |
| ENSMUSG00000022003 | Slc25a30 |  | 1696 | 6.91711591 | 7.522900051 | 6.246346196 | 5.802585835 | 5.505656363 | 5.113047392 | 7.92264199 | 7.611457555 | 6.945367634 | 4.92079863 | 5.859193021 | 7.569341035 | 2.966895968 | 5.230436728 | 4.42139338 | 5.824868343 | 7.00539625 | 6.422053733 |
| ENSMUSG00000022008 | 1200011I18Ri | k | 5440 | 10.75912268 | 12.74953047 | 11.90664653 | 12.57116315 | 10.89161729 | 12.06936985 | 11.78468381 | 12.54592336 | 10.95531035 | 14.99461703 | 10.25756461 | 10.5167379 | 11.99744973 | 13.45512532 | 13.18601265 | 12.40925383 | 15.11211523 | 12.43772082 |
| ENSMUSG00000022009 | Nufip1 |  | 3964 | 7.130661468 | 7.319760561 | 6.931424128 | 6.78128561 | 6.216837769 | 5.925326871 | 6.612316883 | 7.09122305 | 7.449730525 | 8.659362253 | 7.171064656 | 8.293497796 | 7.379875617 | 7.53369026 | 8.502238325 | 9.408438852 | 7.570507183 | 7.049602981 |
| ENSMUSG00000022010 | Tsc22d1 |  | 8272 | 49.75551493 | 44.99979207 | 42.23327324 | 43.17063171 | 45.70554405 | 39.59066927 | 39.27661584 | 47.53735193 | 51.34373193 | 43.35447884 | 45.74198152 | 50.55258028 | 41.15565294 | 45.93877212 | 46.01305287 | 51.20072636 | 50.76659032 | 51.33828626 |
| ENSMUSG00000022012 | Enox1 |  | 3126 | 0.271946104 | 0.309044764 | 0.479711913 | 0.64129429 | 0.569699672 | 0.237777033 | 0 | 0.256570841 | 0.013175469 | 0.467585349 | 0.320945019 | 0.249974177 | 0.236717453 | 0 | 0.197704203 | 0.477713912 | 0.245209833 | 0 |

| ENSMUSG00000022013 | Dnajc15 | 642 | 85.67242645 | 67.50790557 | 78.96489067 | 75.65120446 | 77.14602719 | 69.00333928 | 112.1649491 | 91.43570482 | 99.63030994 | 112.0012811 | 110.2842499 | 93.54774602 | 134.3180551 | 154.0901965 | 133.2953763 | 134.1364113 | 125.1872969 | 125.3556435 |
| --- | --- | --- | --- | --- | --- | --- | --- | --- | --- | --- | --- | --- | --- | --- | --- | --- | --- | --- | --- | --- |
| ENSMUSG00000022014 | Epsti1 | 1881 | 0.881287541 | 1.08032246 | 0.951526115 | 0.847759996 | 1.023539047 | 1.053752973 | 3.169270359 | 0.832476607 | 1.992542761 | 1.930145747 | 1.981098757 | 1.424323128 | 3.383209638 | 3.234543035 | 2.803720975 | 2.483495004 | 3.64721273 | 2.778237713 |
| ENSMUSG00000022015 | Tnfsf11 | 2077 | 0.839052584 | 1.170843177 | 1.607016656 | 1.140671307 | 1.320904983 | 0.787308718 | 0.182234681 | 0 | 0 | 0 | 0 | 0 | 0 | 0 | 0.158696412 | 0.184355356 | 0 | 0 |
| ENSMUSG00000022016 | Akap11 | 9407 | 6.181256597 | 4.100811258 | 4.859464886 | 4.470377795 | 4.850543599 | 4.066621699 | 3.842552004 | 3.934138188 | 3.537653315 | 4.47598363 | 3.423017116 | 2.88364112 | 3.272362749 | 3.604144126 | 4.051391432 | 4.212903015 | 3.666808463 | 3.403348582 |
| ENSMUSG00000022018 | 1190002H23R | 913 | 2.188108733 | 1.240567802 | 1.218610294 | 1.996102709 | 0.105436854 | 1.465414903 | 3.627478122 | 2.635401261 | 1.308224488 | 6.4038195 | 1.308186158 | 4.096001526 | 4.592785289 | 2.899576102 | 2.346638452 | 2.348602426 | 0.755611549 | 3.193299733 |
| ENSMUSG00000022019 | Tdrd3 | 6027 | 5.028403934 | 4.825312204 | 5.45777346 | 4.301349631 | 5.398569836 | 4.521984979 | 5.196780065 | 5.779238592 | 5.179920184 | 5.797025239 | 4.209142683 | 4.510075938 | 5.03386911 | 5.663913096 | 4.764805783 | 4.790296662 | 6.250995496 | 4.654827348 |
| ENSMUSG00000022020 | Naa16 | 6807 | 2.32913628 | 1.526902159 | 1.996909362 | 1.947739665 | 1.81016196 | 2.038308418 | 2.224189402 | 1.91326711 | 1.452146876 | 1.634723956 | 2.589828071 | 2.213931085 | 1.753830466 | 1.42600474 | 1.319515085 | 1.524424064 | 1.734170625 | 2.440539185 |
| ENSMUSG00000022021 | Diap3 | 5069 | 0.117394449 | 0.046003208 | 0 | 0 | 0 | 0.019551286 | 0.186674606 | 0.037672522 | 0 | 0 | 0.028274783 | 0 | 0 | 0.119110897 | 0.016256286 | 0.067984902 | 0.083170106 | 0 |
| ENSMUSG00000022022 | Mtrf1 | 1851 | 7.738651713 | 6.802957328 | 7.73558346 | 7.162757375 | 5.824716857 | 5.193533395 | 6.13454508 | 6.60268437 | 8.010343422 | 7.259832754 | 7.407570547 | 4.794538277 | 5.410253488 | 8.982703017 | 9.437849648 | 4.923373615 | 8.13734991 | 5.943717636 |
| ENSMUSG00000022023 | Wbp4 | 3442 | 21.53661447 | 23.50872169 | 20.87004433 | 19.85519073 | 20.65393703 | 23.86938764 | 21.99311048 | 23.1018541 | 27.65311968 | 21.35617268 | 22.41619929 | 23.3997679 | 22.81708667 | 22.76327002 | 25.13749778 | 24.98567816 | 23.79526336 | 25.37898358 |
| ENSMUSG00000022024 | Sugt1 | 1938 | 51.6510266 | 43.59210513 | 40.46103855 | 44.12697638 | 46.44308424 | 46.15205578 | 48.55774943 | 42.35060514 | 45.28816568 | 44.81506471 | 38.80174646 | 37.6424515 | 46.124579 | 46.80356175 | 44.02911365 | 48.54490319 | 49.61851338 | 44.3933142 |
| ENSMUSG00000022025 | Lect1 | 2183 | 0 | 0 | 0.044318397 | 0 | 0 | 0 | 0 | 0.017495374 | 0 | 0 | 0 | 0 | 0 | 0 | 0 | 0 | 0 | 0 |
| ENSMUSG00000022026 | Olfm4 | 2563 | 0 | 0 | 0 | 0 | 0.112677153 | 0 | 0 | 0 | 0 | 0 | 0 | 0 | 0.346459037 | 0 | 0 | 0 | 0 | 0 |
| ENSMUSG00000022031 | Elp3 | 2755 | 15.67523007 | 17.32754171 | 15.57434534 | 15.80993045 | 17.59304796 | 16.58352826 | 14.70041857 | 15.70671213 | 13.91820188 | 18.58644782 | 15.83249985 | 18.67950668 | 16.7245048 | 15.03743229 | 15.73286272 | 14.55182067 | 19.43442351 | 13.79720245 |
| ENSMUSG00000022032 | Scara5 | 6390 | 0 | 0 | 0 | 0.014260108 | 0.037661912 | 0.007754731 | 0.037020876 | 0.149422545 | 0.21914578 | 0.140197709 | 0.127100829 | 0.131022682 | 0.262485945 | 0.49424068 | 0 | 0.053930433 | 0.245912077 | 0.008608624 |
| ENSMUSG00000022033 | Pbk | 1979 | 0.214781081 | 0.016833196 | 0.562198685 | 0.138133535 | 0 | 0 | 0.836757839 | 0 | 1.082212643 | 0.262080715 | 0 | 0.084611919 | 0.199421596 | 0.328558476 | 0.874414021 | 1.122210828 | 1.646152217 | 0.19457491 |
| ENSMUSG00000022034 | Esco2 | 3999 | 0 | 0.116624286 | 0.1814461 | 0 | 0 | 0 | 0.082817893 | 0 | 0 | 0 | 0.203094347 | 0 | 0.123360633 | 0.034841776 | 0 | 0.23937614 | 0.086255732 | 0 |
| ENSMUSG00000022035 | Ccdc25 | 2218 | 25.1428273 | 22.16854458 | 20.76266948 | 19.86362934 | 21.13638127 | 19.88364873 | 26.85602469 | 21.30027131 | 20.59325788 | 21.55582196 | 15.74548902 | 21.6417808 | 27.11253704 | 30.34151606 | 23.23856917 | 20.69902537 | 22.58452212 | 26.33889601 |
| ENSMUSG00000022037 | Clu | 3470 | 109.4600153 | 128.1250393 | 144.5908521 | 127.7678739 | 134.8664824 | 110.9152973 | 97.58410464 | 111.3743285 | 65.91029453 | 52.61311902 | 50.00537441 | 76.25995859 | 96.94361496 | 145.221426 | 88.61302928 | 100.6369854 | 90.9227885 | 114.4728414 |
| ENSMUSG00000022039 | Adam2 | 2530 | 0 | 0 | 0 | 0 | 0 | 0 | 0 | 0 | 0 | 0 | 0 | 0 | 0 | 0 | 0 | 0 | 0 | 0 |
| ENSMUSG00000022040 | Ephx2 | 2147 | 1.366025685 | 2.063630615 | 2.636098301 | 3.183119073 | 2.309076916 | 2.792678511 | 10.26907703 | 7.453477693 | 8.766761855 | 5.094999141 | 6.297304727 | 8.579025922 | 4.618404924 | 5.343125098 | 3.972390344 | 1.515929962 | 2.35634429 | 3.663857597 |
| ENSMUSG00000022041 | Chrna2 | 3520 | 0.012075334 | 0.179813918 | 0.206137203 | 0.25886957 | 0.273476839 | 0.295627104 | 2.137135213 | 2.007271149 | 2.106128647 | 0.321482068 | 0.97721506 | 2.806639852 | 0.406427728 | 0.356247261 | 0.433084536 | 0 | 0 | 0 |
| ENSMUSG00000022043 | Trim35 | 5092 | 38.19789582 | 37.31017984 | 35.68165358 | 37.64244178 | 40.02171343 | 40.42459845 | 33.86777586 | 37.96738825 | 36.8025622 | 39.38176374 | 35.1556892 | 33.07067047 | 37.40583908 | 33.13656117 | 38.60420722 | 46.99848709 | 40.11014065 | 44.35730405 |
| ENSMUSG00000022044 | Stmn4 | 2013 | 0 | 0 | 0 | 0 | 0 | 0.02461636 | 0 | 0 | 0 | 0 | 0 | 0.055455204 | 0 | 0 | 0 | 0 | 0.342709063 | 0.027326928 |
| ENSMUSG00000022048 | Dpysl2 | 4506 | 9.008531537 | 8.235810954 | 7.300044528 | 8.048511161 | 8.844481313 | 7.86289492 | 7.937946132 | 11.26447022 | 9.478565656 | 8.29793779 | 9.733114044 | 10.61562868 | 10.57581715 | 9.183684391 | 11.08217615 | 14.23363679 | 13.08163441 | 9.705335074 |
| ENSMUSG00000022051 | Bnip3l | 4351 | 36.55581905 | 36.15340991 | 39.12352409 | 39.71800532 | 40.0785933 | 37.37809046 | 40.81003999 | 39.79874799 | 41.94379484 | 43.75880022 | 44.48089101 | 42.93611185 | 38.19793824 | 35.55625261 | 38.17133344 | 42.73481718 | 38.24701934 | 39.68595403 |
| ENSMUSG00000022052 | Ppp2r2a | 2960 | 40.2219588 | 45.16373125 | 44.68014594 | 40.68170276 | 40.39179 | 43.44234582 | 35.62836531 | 36.83760435 | 33.33881479 | 39.87101698 | 37.33225641 | 37.01558975 | 34.39901259 | 32.62069516 | 36.97004475 | 40.04990558 | 37.13507416 | 32.87537493 |
| ENSMUSG00000022053 | Ebf2 | 7281 | 0 | 0 | 0 | 0 | 0 | 0 | 0 | 0 | 0 | 0 | 0 | 0 | 0 | 0 | 0 | 0 | 0 | 0 |
| ENSMUSG00000022054 | Nefm | 5777 | 0 | 0.023065878 | 0 | 0 | 0 | 0 | 0 | 0 | 0 | 0 | 0 | 0 | 0 | 0 | 0 | 0.066281128 | 0.006634291 | 0.07617671 |
| ENSMUSG00000022055 | Nefl | 3376 | 0 | 0 | 0 | 0 | 0 | 0 | 0 | 0 | 0 | 0 | 0 | 0 | 0 | 0 | 0 | 0 | 0 | 0 |
| ENSMUSG00000022056 | Adam7 | 4268 | 29.94682857 | 67.60144724 | 42.88787227 | 39.38019976 | 23.55847904 | 55.10245397 | 74.06185753 | 69.15437811 | 37.42298691 | 25.60809048 | 14.74217908 | 32.96890473 | 143.6150591 | 218.6289034 | 117.7352801 | 26.05340297 | 31.60931703 | 96.05971706 |
| ENSMUSG00000022057 | Adamdec1 | 2411 | 0 | 0.179621578 | 0 | 0 | 0 | 0.102763861 | 0 | 0 | 0 | 0 | 0 | 0 | 0 | 0 | 0 | 0 | 0 | 0 |
| ENSMUSG00000022061 | Nkx3-1 | 3193 | 0 | 0 | 0.045449606 | 0 | 0 | 0 | 0 | 0 | 0 | 0 | 0 | 0 | 0.13905019 | 0.101819171 | 0 | 0 | 0 | 0.551297029 |
| ENSMUSG00000022064 | Pibf1 | 3274 | 9.061885421 | 7.997536608 | 7.239776993 | 7.333741712 | 7.482941096 | 6.644364704 | 9.956761082 | 10.06720931 | 7.308908264 | 8.828155497 | 9.572502371 | 9.444678397 | 6.780501764 | 8.766782531 | 8.809129238 | 9.59019489 | 7.351531599 | 10.41711856 |
| ENSMUSG00000022066 | Entpd4 | 4302 | 9.168945456 | 9.400709805 | 9.793897011 | 9.955225866 | 11.98263372 | 10.22845826 | 11.85566432 | 11.70985095 | 10.3875801 | 10.02856659 | 12.94876838 | 10.78168657 | 10.92824667 | 13.16023769 | 13.8679342 | 10.72528636 | 11.30545578 | 12.51834391 |
| ENSMUSG00000022070 | 6720463M24R | 2661 | 2.060566594 | 2.591420182 | 1.999657399 | 2.208709026 | 1.808790818 | 2.420840047 | 3.769367882 | 3.358520166 | 6.206611357 | 3.224888385 | 2.621249131 | 3.251194175 | 4.857182385 | 4.537941646 | 2.32252288 | 2.935469339 | 4.076039843 | 3.762366563 |
| ENSMUSG00000022074 | Tnfrsf10b | 3163 | 1.222880499 | 0.789904226 | 0.489393921 | 0.460940063 | 0.928247658 | 0.438658407 | 0.314121485 | 0.652035956 | 0.455747092 | 0.46211565 | 0.302086364 | 0.405867923 | 0.857810765 | 0.161519115 | 0.50801792 | 0.714241491 | 0.230224357 | 0.678265938 |
| ENSMUSG00000022075 | Rhobtb2 | 5498 | 1.066881465 | 1.066400397 | 1.601306389 | 1.342468022 | 0.796654248 | 1.396994126 | 1.781324943 | 1.319853848 | 1.363395029 | 0.866173341 | 1.390322546 | 0.761399544 | 1.148505895 | 2.044283221 | 1.229002395 | 1.406821152 | 1.41510336 | 0.710375878 |
| ENSMUSG00000022076 | Klhl1 | 4111 | 0 | 0 | 0 | 0 | 0 | 0 | 0 | 0.009290295 | 0 | 0 | 0 | 0 | 0 | 0 | 0 | 0 | 0 | 0 |
| ENSMUSG00000022085 | Pebp4 | 1040 | 0 | 0 | 0 | 0.262852179 | 0 | 0.047646859 | 0 | 0 | 0 | 0 | 0 | 0 | 0 | 0 | 0 | 0.110453675 | 0 | 0 |
| ENSMUSG00000022089 | Bin3 | 1703 | 36.46509817 | 32.78473911 | 34.76758729 | 32.85317818 | 31.73937186 | 32.44351024 | 36.44993233 | 34.82842098 | 35.30963771 | 39.31532509 | 36.5816476 | 34.24977534 | 40.38091173 | 44.37142113 | 42.0241354 | 42.11292288 | 32.54246936 | 47.99972554 |

| ENSMUSG00000022090 | Pdlim2 | 4901 | 2.801300114 | 2.542138837 | 1.816104792 | 2.83536391 | 1.581154807 | 2.50746336 | 1.187402471 | 1.301393838 | 1.495857949 | 1.115992977 | 1.978844434 | 1.856346256 | 2.184253428 | 2.880841168 | 2.051250691 | 2.343844567 | 1.376337126 | 2.054002557 |
| --- | --- | --- | --- | --- | --- | --- | --- | --- | --- | --- | --- | --- | --- | --- | --- | --- | --- | --- | --- | --- |
| ENSMUSG00000022091 | Sorbs3 | 2893 | 20.77508431 | 23.27181445 | 20.1653915 | 23.05612475 | 20.97972891 | 20.36577955 | 17.85877822 | 24.51548264 | 20.31564397 | 26.15861691 | 23.03700901 | 21.99441941 | 27.28346617 | 26.92247997 | 25.22224524 | 31.91104544 | 29.55615914 | 30.70850583 |
| ENSMUSG00000022092 | Ppp3cc | 3291 | 7.038991472 | 6.367004091 | 8.77514361 | 8.749492562 | 8.906819056 | 6.760612998 | 10.37974907 | 12.48709358 | 11.41358322 | 10.91730048 | 13.28291918 | 7.784682202 | 10.40302358 | 12.10848586 | 8.888819411 | 11.39061217 | 11.30806268 | 9.3604071 |
| ENSMUSG00000022094 | Slc39a14 | 10975 | 1.270314145 | 1.74835782 | 1.564701893 | 1.681295941 | 1.697225919 | 1.688630739 | 1.664026794 | 2.641278672 | 1.561147203 | 1.894620514 | 2.685845046 | 2.15634086 | 1.636156527 | 1.61231866 | 1.50165124 | 2.581781274 | 2.451486144 | 2.5462074 |
| ENSMUSG00000022095 | Fam160b2 | 4016 | 12.51023856 | 15.44537078 | 13.80380124 | 13.06930355 | 16.03598453 | 13.88118159 | 9.778267806 | 15.95788128 | 14.82960702 | 13.43137563 | 14.14452829 | 14.8573066 | 15.30566955 | 16.45665746 | 16.19951612 | 12.26138474 | 19.13451825 | 13.04000737 |
| ENSMUSG00000022096 | Hr | 6577 | 0.859539062 | 0.628067338 | 1.27976194 | 0.852061495 | 0.900140887 | 1.084931372 | 0.546717895 | 0.516819798 | 0.294323588 | 0.501832025 | 0.581115505 | 0.908054721 | 0.382534253 | 0.46606504 | 0.538746207 | 0.349313736 | 0.804170438 | 0.301098957 |
| ENSMUSG00000022097 | Sftpc | 798 | 0 | 0 | 0 | 0 | 0 | 0 | 0 | 0 | 0 | 0 | 0 | 0 | 0 | 0 | 0 | 0 | 0 | 0 |
| ENSMUSG00000022098 | Bmp1 | 4045 | 3.225979985 | 3.376585075 | 3.252805997 | 2.579352077 | 3.462642716 | 3.613858197 | 1.111175401 | 1.7467477 | 1.221849664 | 1.958298677 | 1.653521431 | 0.745128747 | 0.243915537 | 0.803729572 | 1.77232898 | 0.700495661 | 0.454799073 | 0.421577827 |
| ENSMUSG00000022099 | Epb4.9 | 6685 | 0.782069806 | 0.702635465 | 0.694668499 | 0.633833526 | 1.029598367 | 0.630064675 | 0.771440838 | 0.57131492 | 0.757807246 | 0.895757564 | 0.293010217 | 0.317276766 | 1.483278291 | 0.972651045 | 1.23265687 | 1.162751429 | 1.353030078 | 0.732357591 |
| ENSMUSG00000022100 | Xpo7 | 15093 | 4.001845567 | 3.811791447 | 3.955008047 | 3.571106834 | 3.64186423 | 3.952924616 | 2.736630803 | 2.940407579 | 3.342839847 | 3.395800334 | 3.364790357 | 3.494719482 | 2.503693675 | 2.310972612 | 2.847228702 | 2.770379864 | 3.730294186 | 2.6533247 |
| ENSMUSG00000022101 | Fgf17 | 1682 | 0 | 0 | 0 | 0 | 0 | 0 | 0 | 0 | 0 | 0 | 0 | 0 | 0 | 0 | 0 | 0 | 0 | 0 |
| ENSMUSG00000022102 | Dok2 | 2746 | 0.309578849 | 0.012131425 | 0.08807999 | 0.232285004 | 0.210336156 | 0.090227119 | 0.017229672 | 0.305984287 | 0.569951784 | 0.223218916 | 0.034796037 | 0.223587869 | 0.07186004 | 0.135306882 | 0.015004208 | 0.250994513 | 0 | 0 |
| ENSMUSG00000022103 | Gfra2 | 3869 | 0.054930442 | 0 | 0.012502851 | 0 | 0 | 0 | 0.048914633 | 0.434341097 | 0 | 0.158428313 | 0.111133271 | 0.158690175 | 0.344265124 | 0.252087318 | 0.07454404 | 0.26721282 | 0 | 0 |
| ENSMUSG00000022105 | Rb1 | 5984 | 7.870276577 | 8.266986617 | 9.441892289 | 7.758473292 | 7.5045262 | 9.042711408 | 7.748399958 | 9.771485308 | 6.098137193 | 9.746895042 | 10.28311272 | 8.917074437 | 5.55643587 | 7.606150675 | 8.641034867 | 7.313864348 | 11.02905803 | 7.299002463 |
| ENSMUSG00000022106 | Rcbtb2 | 7949 | 5.940778786 | 5.47322177 | 6.176768563 | 5.949473395 | 6.376011526 | 6.626563819 | 4.571158311 | 5.97702209 | 6.00517949 | 6.358731148 | 5.805838456 | 6.951504084 | 5.182054463 | 4.31195578 | 5.83114233 | 5.428797904 | 5.33260591 | 5.079467145 |
| ENSMUSG00000022108 | Itm2b | 1767 | 2046.502748 | 2038.967819 | 2160.02733 | 2199.461349 | 2150.573574 | 2156.988147 | 3108.633113 | 2997.855025 | 2935.057867 | 3060.606317 | 2785.169226 | 2923.009707 | 3745.065863 | 3974.63611 | 3396.668393 | 3215.28419 | 3229.603258 | 3504.550182 |
| ENSMUSG00000022109 | Med4 | 1297 | 39.81787885 | 35.21355279 | 36.84891904 | 33.51213284 | 34.25271052 | 33.77379837 | 42.67990312 | 35.65998405 | 44.01273003 | 37.95322606 | 42.9127422 | 39.54864604 | 37.80719026 | 41.64570057 | 41.04272183 | 38.32012984 | 40.30614416 | 40.6312446 |
| ENSMUSG00000022110 | Sucla2 | 3414 | 61.03115785 | 57.99019635 | 58.50448355 | 56.504236 | 60.27064255 | 60.96118366 | 60.68606357 | 59.2687018 | 58.66726015 | 61.69367061 | 62.0066904 | 56.60041707 | 54.18707961 | 43.91374168 | 52.85964113 | 53.46553178 | 58.34263767 | 48.2578426 |
| ENSMUSG00000022111 | Uchl3 | 1662 | 18.38821995 | 15.39368397 | 18.01637502 | 16.31096353 | 18.96895909 | 18.60463643 | 24.90890136 | 17.5795354 | 25.35126692 | 17.27724325 | 19.54691442 | 20.1500587 | 21.48995674 | 21.46152775 | 22.90628017 | 25.27364399 | 22.57607982 | 19.82578515 |
| ENSMUSG00000022112 | Gpc5 | 4983 | 0.025590112 | 0.006685309 | 0.067953986 | 0 | 0 | 0.029833073 | 0.075958545 | 0.122632639 | 0.033061622 | 0.151397 | 0.028762769 | 0 | 0.158400698 | 0.270294709 | 0.008268424 | 0 | 0.092296922 | 0 |
| ENSMUSG00000022113 | Trim52 | 3082 | 0 | 0 | 0 | 0 | 0.015617107 | 0 | 0 | 0 | 0 | 0 | 0 | 0 | 0 | 0 | 0 | 0 | 0 | 0 |
| ENSMUSG00000022114 | Spry2 | 2083 | 3.224108407 | 1.727216791 | 2.159739953 | 1.902933681 | 2.218273966 | 2.378911838 | 5.56486142 | 3.135334043 | 4.982718181 | 2.354139769 | 4.724743853 | 3.938983389 | 3.694565096 | 2.274266401 | 3.441704621 | 2.757364911 | 2.318345372 | 2.852128433 |
| ENSMUSG00000022116 | 4930449E01R | 592 | 0 | 0.056271781 | 0 | 0.153922447 | 0 | 0 | 0 | 0.19354258 | 0 | 0 | 0 | 0.282849641 | 0 | 0 | 0 | 0.12936016 | 0 | 0 |
| ENSMUSG00000022119 | Rbm26 | 8072 | 10.0154664 | 9.871709982 | 10.63115001 | 10.19366279 | 9.349709652 | 9.748481284 | 9.776703227 | 10.87291139 | 8.883266098 | 8.75021723 | 11.92598381 | 10.6071886 | 12.60188472 | 10.74220901 | 9.172348357 | 9.847782577 | 11.12469198 | 10.09272635 |
| ENSMUSG00000022120 | Rnf219 | 3450 | 3.523617491 | 2.32707464 | 2.902411821 | 2.165800368 | 2.38566926 | 3.202973794 | 1.535947838 | 3.232516377 | 1.993666125 | 1.763026296 | 2.935736579 | 2.815050809 | 1.37271422 | 2.74625908 | 1.504752476 | 1.675907744 | 2.321795957 | 1.132071472 |
| ENSMUSG00000022122 | Ednrb | 6600 | 0 | 0 | 0 | 0 | 0 | 0 | 0 | 0.017360183 | 0.068644193 | 0 | 0 | 0 | 0 | 0 | 0 | 0 | 0 | 0 |
| ENSMUSG00000022123 | Scel | 3129 | 0.040752805 | 0.287455463 | 0 | 0.291217925 | 0.138442733 | 0.36424189 | 3.114864768 | 2.538836593 | 1.303120825 | 1.717858796 | 0.396979521 | 1.801656095 | 1.150920409 | 2.345211188 | 0.790058598 | 0.354882587 | 0.024497473 | 1.7228803 |
| ENSMUSG00000022124 | Fbxl3 | 7229 | 12.79447545 | 15.8384863 | 12.64711196 | 13.66390152 | 14.13529865 | 14.37433702 | 11.15895941 | 12.85935917 | 11.96454324 | 13.22750397 | 12.14035119 | 13.24936741 | 9.779034093 | 9.611336939 | 11.34196932 | 12.68584932 | 12.80370826 | 13.33946107 |
| ENSMUSG00000022125 | Cln5 | 2392 | 30.01306117 | 28.74490538 | 29.76832637 | 29.25659034 | 30.30379475 | 35.42440402 | 22.90471664 | 25.30725661 | 26.86077115 | 21.52532105 | 27.462612 | 28.39785174 | 21.03618546 | 17.84369411 | 17.51755115 | 23.4193807 | 20.5891687 | 21.52530263 |
| ENSMUSG00000022126 | Irg1 | 2618 | 0 | 0 | 0 | 0 | 0 | 0 | 0 | 0 | 0 | 0 | 0 | 0 | 0 | 0 | 0 | 0 | 0 | 0 |
| ENSMUSG00000022129 | Dct | 2475 | 0 | 0 | 0 | 0 | 0 | 0 | 0 | 0 | 0 | 0 | 0 | 0 | 0 | 0 | 0 | 0 | 0 | 0 |
| ENSMUSG00000022130 | Tgds | 2253 | 7.357753507 | 5.91440643 | 5.582386992 | 5.197154192 | 7.135402798 | 5.894421926 | 6.425956392 | 6.916333858 | 7.03808636 | 5.545910503 | 4.686314169 | 8.596553448 | 7.094336972 | 6.679043193 | 5.541088076 | 6.764164113 | 8.641703873 | 6.250479947 |
| ENSMUSG00000022131 | Gpr180 | 2868 | 18.34777124 | 19.25829101 | 17.08590873 | 15.47296276 | 16.48031696 | 15.94741042 | 17.88247697 | 16.37958681 | 17.72111592 | 19.79408882 | 16.14153932 | 20.39568145 | 20.692572 | 18.16954883 | 16.11860032 | 17.11595491 | 18.65533838 | 19.103581 |
| ENSMUSG00000022132 | Cldn10 | 2622 | 58.39193137 | 37.50559258 | 43.30002889 | 48.56716969 | 45.65373538 | 50.9323482 | 101.7348908 | 67.45576489 | 81.60333691 | 101.1528245 | 70.35053954 | 98.81628746 | 193.1131956 | 142.5737831 | 154.6708294 | 192.9572066 | 138.7318398 | 166.6008072 |
| ENSMUSG00000022136 | Dnajc3 | 5178 | 46.21555447 | 42.55789789 | 42.27312183 | 46.08898228 | 35.34136226 | 48.36607097 | 41.84860361 | 38.74559479 | 35.97655674 | 46.28563628 | 34.2396429 | 45.88784775 | 48.26486924 | 41.87861028 | 45.92008093 | 52.16337285 | 48.8738004 | 51.01462543 |
| ENSMUSG00000022139 | Mbnl2 | 4752 | 59.80421021 | 62.0480696 | 60.88427716 | 66.16514456 | 58.58481618 | 64.96496847 | 49.34377878 | 50.98750018 | 47.738916 | 52.90562916 | 59.51762504 | 52.86748681 | 51.4497016 | 40.54084059 | 42.51945078 | 47.97606828 | 56.43289129 | 46.36184184 |
| ENSMUSG00000022141 | Nipbl | 8768 | 9.976693917 | 11.03717583 | 10.77480666 | 8.677799275 | 8.602044296 | 9.432426134 | 9.08157375 | 10.73725729 | 11.29716816 | 8.087894354 | 9.469990629 | 11.46487321 | 7.893781927 | 8.109705496 | 8.970548608 | 10.07486671 | 9.668997348 | 9.504881009 |
| ENSMUSG00000022142 | Nup155 | 6367 | 4.16573423 | 3.123574344 | 3.198563889 | 5.352546123 | 3.50765079 | 3.891372199 | 2.444773263 | 2.729314134 | 2.859186425 | 2.865921495 | 3.429111988 | 2.78771489 | 3.626093495 | 4.551768577 | 3.132017128 | 2.622067666 | 3.208405254 | 3.956992442 |
| ENSMUSG00000022144 | Gdnf | 3506 | 0.024247106 | 0 | 0 | 0 | 0 | 0 | 0.107958195 | 0 | 0 | 0 | 0 | 0 | 0.211060684 | 0 | 0 | 0 | 0 | 0 |
| ENSMUSG00000022146 | Osmr | 12364 | 1.670779323 | 2.928754126 | 2.320082781 | 3.032735319 | 3.180506441 | 2.593062005 | 1.607192264 | 3.10135652 | 2.791515708 | 1.769486114 | 1.514702663 | 2.293299627 | 1.488256644 | 1.769262792 | 1.352946594 | 1.734288268 | 2.141982462 | 1.38813016 |
| ENSMUSG00000022148 | Fyb | 6328 | 0.040302 | 0.073701093 | 0 | 0.086398946 | 0.022818548 | 0.046984261 | 0.127104226 | 0.090531927 | 0.05206892 | 0 | 0 | 0 | 0 | 0.036697288 | 0.045576942 | 0.012101962 | 0.006056621 | 0.008692969 |

| ENSMUSG00000022149 | C9 | 2673 | 0 | 0 | 0 | 0 | 0 | 0 | 0 | 0.042864649 | 0 | 0 | 0 | 0 | 0.018455637 | 0.121626866 | 0 | 0 | 0 | 0 |
| --- | --- | --- | --- | --- | --- | --- | --- | --- | --- | --- | --- | --- | --- | --- | --- | --- | --- | --- | --- | --- |
| ENSMUSG00000022150 | Dab2 | 7126 | 199.1110414 | 229.5064873 | 238.4802828 | 223.278528 | 242.7399765 | 249.6968225 | 259.0711103 | 239.9432589 | 219.6132528 | 234.5095741 | 247.8920979 | 225.3539753 | 282.5335683 | 309.0949824 | 318.8177089 | 334.8789271 | 374.474705 | 340.8850889 |
| ENSMUSG00000022151 | Ttc33 | 2168 | 20.44875397 | 18.5925286 | 20.50520037 | 20.2376779 | 20.26957856 | 21.37075918 | 20.40468395 | 19.8184745 | 22.01806816 | 22.00946111 | 19.61241377 | 21.42003287 | 15.88269292 | 18.68046319 | 20.71480439 | 18.29754118 | 22.08004835 | 20.93289347 |
| ENSMUSG00000022155 | Heatr7b2 | 5190 | 0 | 0 | 0 | 0 | 0 | 0 | 0 | 0 | 0 | 0 | 0 | 0 | 0 | 0 | 0 | 0 | 0 | 0 |
| ENSMUSG00000022156 | Gzme | 938 | 0 | 0 | 0 | 0 | 0 | 0 | 0 | 0 | 0 | 0 | 0 | 0 | 0 | 0 | 0 | 0 | 0 | 0 |
| ENSMUSG00000022157 | Mcpt8 | 850 | 0 | 0 | 0 | 0 | 0 | 0 | 0 | 0 | 0 | 0 | 0 | 0 | 0 | 0 | 0 | 0 | 0 | 0 |
| ENSMUSG00000022159 | Rab2b | 5219 | 11.55678191 | 12.16600429 | 11.17809496 | 13.71458146 | 12.80997164 | 11.91582308 | 12.31090592 | 11.50382383 | 10.96939201 | 12.88310076 | 12.12910902 | 9.860517514 | 9.546893361 | 8.311702936 | 9.315546264 | 10.07338647 | 10.52339212 | 9.644248448 |
| ENSMUSG00000022160 | Mettl3 | 3506 | 7.965174173 | 7.743870162 | 6.898678031 | 8.225938695 | 7.276075169 | 6.784173451 | 8.353265333 | 8.671178641 | 11.30103484 | 8.20363063 | 6.336382114 | 8.676422165 | 6.908719733 | 11.94882111 | 9.283864562 | 9.41429081 | 8.701577962 | 9.272784391 |
| ENSMUSG00000022174 | Dad1 | 3913 | 66.28330798 | 58.60617526 | 61.46516558 | 55.78404381 | 63.22465823 | 60.38012617 | 71.85771809 | 65.79478268 | 64.14276183 | 67.28584896 | 67.06563939 | 61.17898718 | 88.14944176 | 89.68349714 | 77.88599768 | 76.76713904 | 71.72590652 | 81.00242089 |
| ENSMUSG00000022175 | Lrp10 | 4194 | 107.8034233 | 121.0352604 | 107.577472 | 131.2840298 | 122.2922696 | 131.6562018 | 92.84295364 | 108.9858541 | 100.8156344 | 117.8317885 | 122.1599081 | 112.0971211 | 91.12407323 | 100.8281869 | 98.31787586 | 94.4392091 | 110.1903882 | 104.0634842 |
| ENSMUSG00000022176 | Rem2 | 2051 | 8.890648716 | 11.10971216 | 7.382211117 | 8.485772275 | 10.18491218 | 8.142014245 | 4.959642111 | 5.549164269 | 6.747279033 | 5.839238726 | 6.265950186 | 4.95292569 | 2.068525054 | 4.166607547 | 3.274428869 | 5.731456105 | 3.195415295 | 3.191654656 |
| ENSMUSG00000022177 | Haus4 | 1577 | 6.145326655 | 7.41460106 | 10.52131954 | 7.858341192 | 6.867268754 | 8.106915195 | 9.750552094 | 9.372517267 | 6.685953098 | 11.33171626 | 7.634299009 | 7.645011489 | 7.069761132 | 9.453742582 | 10.24160425 | 11.87324478 | 11.32533567 | 11.12739698 |
| ENSMUSG00000022178 | Jub | 3247 | 5.943132096 | 8.423124778 | 6.674265968 | 5.205773775 | 6.240695985 | 6.119693922 | 9.238139341 | 7.798448659 | 8.739608673 | 6.52006953 | 4.340502849 | 5.46639381 | 5.834141125 | 4.877558916 | 7.588090675 | 5.813757145 | 6.373945281 | 4.557268158 |
| ENSMUSG00000022179 | 4931414P19R | 2578 | 1.797154456 | 1.318043138 | 2.120329298 | 1.820321011 | 1.53096111 | 1.383939805 | 1.76183754 | 0.918513945 | 1.326020484 | 1.66435764 | 2.965086639 | 1.623807096 | 2.277152116 | 2.197897076 | 1.965784086 | 2.198219509 | 1.932668191 | 2.09111422 |
| ENSMUSG00000022180 | Slc7a8 | 4022 | 13.1573705 | 18.74367967 | 18.61815639 | 14.79431226 | 14.77944449 | 16.86665646 | 12.622204 | 17.62434084 | 13.29191881 | 15.79114801 | 18.17396481 | 13.79432337 | 15.87158154 | 16.62841518 | 17.19975444 | 14.97541657 | 17.84812384 | 17.5339818 |
| ENSMUSG00000022181 | C6 | 4118 | 0 | 0 | 0 | 0 | 0 | 0 | 0 | 0 | 0 | 0 | 0 | 0 | 0 | 0 | 0 | 0 | 0.009307017 | 0 |
| ENSMUSG00000022184 | Fbxo4 | 2039 | 17.61494544 | 19.31134917 | 18.00662557 | 20.91473149 | 20.39528301 | 21.72640697 | 15.82503533 | 17.94424792 | 19.6337878 | 19.1701486 | 16.09681043 | 16.94452925 | 17.15366815 | 18.72341336 | 15.96332965 | 19.86829948 | 18.8342077 | 12.9496671 |
| ENSMUSG00000022185 | Acin1 | 8657 | 6.176679155 | 5.94144723 | 5.62690829 | 5.510270696 | 5.665522723 | 4.584930068 | 5.552695136 | 5.289671998 | 7.245819973 | 5.501006156 | 5.342053807 | 5.635080178 | 6.866692886 | 5.423931194 | 4.530886128 | 5.87827391 | 5.85718965 | 7.390041714 |
| ENSMUSG00000022186 | Oxct1 | 3846 | 13.4500258 | 12.73269748 | 7.961634091 | 11.83449904 | 10.16201821 | 11.37677165 | 11.36685266 | 10.50638631 | 8.974077017 | 10.23682715 | 9.16742573 | 8.896256153 | 11.06953838 | 9.853971739 | 8.666681933 | 8.372959133 | 8.061875766 | 11.02756664 |
| ENSMUSG00000022187 | Gm5546 | 2775 | 0.076585903 | 0.108041819 | 0 | 0 | 0 | 0 | 0 | 0.027526056 | 0 | 0 | 0 | 0 | 0 | 0.066946432 | 0 | 0 | 0 | 0 |
| ENSMUSG00000022191 | Drosha | 5849 | 6.867394657 | 9.784843951 | 10.24701729 | 9.479875356 | 10.50031366 | 9.607249081 | 7.951506819 | 7.430835004 | 8.266878014 | 9.463998226 | 7.261401622 | 9.017917788 | 5.229233837 | 6.717677882 | 6.184812117 | 6.919673794 | 7.352043961 | 8.840581351 |
| ENSMUSG00000022193 | Psmb5 | 918 | 44.49615922 | 37.37721247 | 39.15199679 | 41.98762909 | 42.36448183 | 38.21714093 | 40.71570409 | 36.36182974 | 34.95021327 | 35.08053412 | 37.26238592 | 39.94650362 | 41.70105426 | 38.29866466 | 38.64328935 | 39.79219985 | 31.10358518 | 39.54903098 |
| ENSMUSG00000022194 | Pabpn1 | 2115 | 54.76435209 | 57.80535309 | 59.16894706 | 49.41703816 | 49.01946269 | 52.85624915 | 46.37313637 | 56.62949123 | 62.31529572 | 46.99464892 | 48.18155387 | 57.95328371 | 49.79841295 | 43.50153055 | 52.46136644 | 50.85499583 | 45.91907156 | 49.70326381 |
| ENSMUSG00000022195 | 6030458C11R | 3965 | 6.646458799 | 6.124867563 | 7.393281058 | 6.561250017 | 6.919343377 | 7.885945747 | 5.882761844 | 7.87929008 | 7.167388619 | 7.515572373 | 7.205403574 | 8.404022841 | 6.096504279 | 4.978243919 | 7.242745134 | 7.783664455 | 7.520267085 | 8.809781278 |
| ENSMUSG00000022197 | Pdzd2 | 16835 | 2.125890004 | 2.338927292 | 2.476862677 | 2.246252854 | 3.047735707 | 2.757998893 | 1.753674586 | 2.302660436 | 2.037919075 | 1.974531975 | 1.853106498 | 1.836761335 | 2.200669431 | 2.642912725 | 3.122850331 | 2.363168464 | 3.043793226 | 2.231732693 |
| ENSMUSG00000022199 | Slc22a17 | 3429 | 15.19724287 | 17.77853497 | 15.17932886 | 18.52204602 | 15.07544066 | 15.27478547 | 11.57635976 | 11.07123009 | 12.53972658 | 16.47318242 | 14.33666733 | 14.24284187 | 11.23599516 | 5.282354646 | 7.641933376 | 5.5498489 | 6.572138402 | 6.096080652 |
| ENSMUSG00000022200 | Golph3 | 3343 | 82.93785918 | 80.93548515 | 85.99577951 | 94.81549071 | 87.17882076 | 91.50135339 | 80.85472172 | 79.19525381 | 68.67294013 | 81.81909351 | 77.30025439 | 73.91442964 | 66.61210726 | 67.07509839 | 70.07838672 | 84.90824788 | 81.62824826 | 80.21818584 |
| ENSMUSG00000022201 | Zfr | 5984 | 24.70471294 | 25.9310597 | 24.65562627 | 26.0925299 | 28.2083316 | 26.27520449 | 26.54222312 | 26.54448556 | 20.03575324 | 25.54521724 | 28.95718155 | 24.49397225 | 23.71790207 | 23.98265876 | 24.24309463 | 26.99666989 | 29.70544202 | 23.67119816 |
| ENSMUSG00000022203 | Efs | 3565 | 0.727297542 | 1.382975693 | 1.19407312 | 1.712532943 | 0.94508686 | 1.084183231 | 0.477771793 | 0.814199883 | 0.242613417 | 1.058080298 | 0.227818876 | 0.438383886 | 0.733405783 | 0.846806642 | 1.2250673 | 0.25777688 | 0.623541436 | 1.31157758 |
| ENSMUSG00000022204 | Ngdn | 1187 | 44.29562152 | 41.84458743 | 39.08190026 | 35.2359214 | 37.75132345 | 40.0346011 | 41.85199063 | 45.85018823 | 43.68477114 | 40.67592261 | 43.42812148 | 44.24813689 | 45.54994215 | 45.38765069 | 37.00156578 | 50.25843836 | 40.90936662 | 42.35747561 |
| ENSMUSG00000022205 | Sub1 | 4179 | 56.54134327 | 48.00436683 | 54.0338932 | 45.97533476 | 49.1454698 | 51.42622769 | 66.71778342 | 58.75543313 | 65.17502482 | 59.0088964 | 59.74442044 | 55.21463248 | 61.60882418 | 64.65941623 | 62.78332326 | 67.26282578 | 64.55582769 | 63.22295755 |
| ENSMUSG00000022206 | Npr3 | 10083 | 0 | 0 | 0 | 0 | 0 | 0 | 0 | 0 | 0 | 0 | 0 | 0 | 0 | 0.032243242 | 0 | 0 | 0 | 0 |
| ENSMUSG00000022208 | Jph4 | 7984 | 0.037266562 | 0.01251737 | 0.05452928 | 0.079891611 | 0 | 0.043445533 | 0 | 0 | 0 | 0 | 0 | 0 | 0 | 0 | 0.067086702 | 0.019183671 | 0 | 0 |
| ENSMUSG00000022209 | Dhrs2 | 1257 | 0 | 0 | 0.269383224 | 0.108737576 | 0 | 0 | 0 | 0 | 0 | 0 | 0 | 0 | 0 | 0 | 0 | 0 | 0 | 0 |
| ENSMUSG00000022210 | Dhrs4 | 1382 | 51.48600917 | 48.11326835 | 48.05850736 | 47.60502751 | 47.57467998 | 43.2779663 | 42.28014362 | 38.80038566 | 41.27592209 | 41.76010187 | 37.02386435 | 43.61860752 | 46.08357829 | 40.19329126 | 39.44258932 | 38.9555673 | 43.26267957 | 40.56026031 |
| ENSMUSG00000022211 | Lrrc16b | 4540 | 0.046811868 | 0.066038777 | 0.117204589 | 0.090319251 | 0.095415708 | 0.087317592 | 0.281374963 | 0.126186352 | 0.254013754 | 0.020771235 | 0 | 0.331943368 | 0.010866061 | 0 | 0.009075233 | 0.059038381 | 0.067535325 | 0.03634963 |
| ENSMUSG00000022212 | Cpne6 | 3393 | 0 | 0 | 0 | 0 | 0 | 0 | 0 | 0 | 0 | 0 | 0 | 0 | 0 | 0 | 0 | 0 | 0 | 0 |
| ENSMUSG00000022214 | Dcaf11 | 3810 | 61.03564778 | 73.18344478 | 64.129843 | 67.65994458 | 69.25438466 | 68.9446301 | 72.95590255 | 70.79127186 | 68.29826944 | 67.18665826 | 67.51190983 | 67.38898895 | 64.96016509 | 75.70020543 | 67.02552321 | 63.73727878 | 62.42860855 | 66.42963255 |
| ENSMUSG00000022215 | Fitm1 | 967 | 3.164811452 | 2.755978839 | 4.602238669 | 3.627921834 | 2.88691993 | 4.40696493 | 3.033491307 | 2.132771179 | 3.577732496 | 4.388379827 | 3.557184085 | 2.943742287 | 3.111941007 | 2.497510387 | 2.258203167 | 1.702684714 | 2.140248224 | 1.649704338 |
| ENSMUSG00000022216 | Psme1 | 1816 | 152.2790062 | 142.2585323 | 151.8332175 | 136.4573349 | 154.8914989 | 144.9199163 | 121.8249376 | 118.9726985 | 125.6687686 | 118.8374278 | 118.5692389 | 128.1977581 | 119.8254885 | 108.9748108 | 115.8453438 | 123.200451 | 106.5158703 | 107.5040307 |
| ENSMUSG00000022217 | Fam158a | 2682 | 8.241122869 | 7.489811788 | 6.204509486 | 6.40436753 | 5.742809684 | 7.020894392 | 8.714565023 | 6.39388094 | 9.091132487 | 8.245219921 | 8.764086343 | 7.741769755 | 8.074836571 | 7.775315152 | 7.297068996 | 8.351978124 | 7.759574653 | 8.532359581 |

| ENSMUSG00000022218 | Tgm1 | 2990 | 0.014215778 | 0.111414362 | 0.080892191 | 0 | 0.1931716 | 0 | 0 | 0.166053924 | 0 | 0 | 0.287608446 | 0.205341902 | 0.016498969 | 0.217463954 | 0.082678707 | 0.051224893 | 0.230726871 | 0.717510088 |
| --- | --- | --- | --- | --- | --- | --- | --- | --- | --- | --- | --- | --- | --- | --- | --- | --- | --- | --- | --- | --- |
| ENSMUSG00000022219 | Cideb | 1752 | 0.194087562 | 0 | 0.055220925 | 0.104020649 | 0.082417678 | 0.028283524 | 0.43207926 | 0.108996582 | 0.141049712 | 0.242212517 | 0.081806436 | 0.223007784 | 0 | 0.159055094 | 0.141101219 | 0.305975173 | 0.175005922 | 0.219785244 |
| ENSMUSG00000022220 | Adcy4 | 3645 | 0 | 0 | 0 | 0 | 0 | 0 | 0 | 0 | 0 | 0.038807163 | 0 | 0 | 0.081204802 | 0 | 0.022607164 | 0 | 0.021029518 | 0 |
| ENSMUSG00000022221 | Ripk3 | 1954 | 0.891869098 | 0.954719589 | 0.940733958 | 1.282424482 | 0.689710267 | 2.104849993 | 0.556904614 | 1.895938093 | 2.740146903 | 1.713254827 | 1.540338989 | 1.571065221 | 0.908878722 | 0.308993417 | 0.527143755 | 1.097376671 | 0.333442706 | 0.844561516 |
| ENSMUSG00000022223 | Sdr39u1 | 1673 | 86.94125067 | 75.62604438 | 73.93372213 | 74.56433915 | 72.55750836 | 79.1422021 | 74.03741389 | 74.6041668 | 79.78810376 | 75.81314511 | 79.98664596 | 73.49785097 | 84.09720745 | 82.86646794 | 78.36422645 | 70.0125332 | 68.24509174 | 70.82463591 |
| ENSMUSG00000022225 | Cma1 | 1069 | 0 | 0 | 0 | 0 | 0 | 0 | 0 | 0 | 0 | 0 | 0 | 0 | 0 | 0 | 0 | 0 | 0 | 0 |
| ENSMUSG00000022226 | Mcpt2 | 966 | 0 | 0 | 0 | 0 | 0 | 0 | 0 | 0 | 0 | 0 | 0 | 0 | 0 | 0 | 0 | 0 | 0 | 0 |
| ENSMUSG00000022227 | Mcpt1 | 1023 | 0 | 0 | 0 | 0 | 0 | 0 | 0 | 0 | 0 | 0 | 0 | 0 | 0 | 0 | 0 | 0 | 0 | 0 |
| ENSMUSG00000022228 | Zfp187 | 5660 | 2.380590247 | 1.801015129 | 2.299024676 | 1.658228822 | 2.474627171 | 1.996117183 | 2.148296552 | 2.638379742 | 2.415887497 | 2.032645158 | 2.388747924 | 2.139929113 | 2.693208911 | 2.592991451 | 2.591475954 | 3.017245742 | 2.397086414 | 2.565795791 |
| ENSMUSG00000022229 | Atp12a | 3928 | 0 | 0 | 0 | 0.092792351 | 0.085774813 | 0.037845774 | 0 | 0 | 0 | 0.084026202 | 0 | 0 | 0 | 0.106414661 | 0.062935167 | 0.04874059 | 0 | 0.182056616 |
| ENSMUSG00000022231 | Sema5a | 12502 | 0.622176229 | 0.530256435 | 0.804806776 | 0.841833406 | 0.739188077 | 0.546974663 | 1.263992542 | 1.270839818 | 1.106916437 | 1.252122339 | 1.031774019 | 1.147386441 | 0.856265082 | 0.739271588 | 1.196301775 | 1.179162042 | 1.106686385 | 1.091206084 |
| ENSMUSG00000022234 | Cct5 | 2796 | 98.43383933 | 91.7534329 | 97.85432321 | 92.88195734 | 97.69265623 | 98.07754922 | 86.11381369 | 85.63239295 | 93.98067617 | 90.60827203 | 92.79892686 | 86.27871735 | 83.49021193 | 85.16410445 | 83.30200123 | 77.53984947 | 74.56904691 | 86.56654849 |
| ENSMUSG00000022235 | Cmbl | 1255 | 1.287009314 | 0.265441388 | 0.26981252 | 0.471947073 | 0.03835213 | 0.276389749 | 3.355241773 | 0.791237022 | 0.78763058 | 2.216646607 | 1.065895488 | 1.200815052 | 3.18397235 | 4.181818227 | 4.793169052 | 2.959513082 | 4.886221119 | 3.594220519 |
| ENSMUSG00000022236 | Ropn1l | 1000 | 0.467556936 | 0.832822355 | 0.628855895 | 0.364488355 | 0.818242702 | 0.743291004 | 1.135504294 | 1.986004925 | 3.171361714 | 1.555973207 | 1.194373962 | 1.953548191 | 2.515927781 | 5.759066836 | 3.048915136 | 1.110427615 | 2.031293736 | 3.630601044 |
| ENSMUSG00000022237 | Ankrd33b | 15204 | 0.025160917 | 0.024101673 | 0.03499795 | 0.032963134 | 0.028491668 | 0.117330861 | 0.115138721 | 0.065311922 | 0.008126779 | 0.105440931 | 0.311083984 | 0.066080106 | 0.012978668 | 0.042766195 | 0.02438924 | 0.03273993 | 0.063020088 | 0.01809034 |
| ENSMUSG00000022240 | Ctnnd2 | 5891 | 5.923739521 | 6.503111246 | 8.277120794 | 6.952873682 | 6.119641967 | 7.688202086 | 5.091875478 | 4.317796639 | 4.719215437 | 3.673768935 | 2.765449133 | 5.419548294 | 1.783686705 | 1.474290331 | 2.259056621 | 1.488465302 | 1.086486434 | 1.241930266 |
| ENSMUSG00000022241 | Tars | 2617 | 26.10080928 | 31.13616326 | 27.929845 | 28.96965142 | 27.10984161 | 29.72785316 | 22.97837788 | 20.32939112 | 26.70749608 | 23.07988188 | 27.7120317 | 27.34263639 | 18.58665282 | 24.63293592 | 22.7812959 | 21.06934454 | 24.66239357 | 22.23906569 |
| ENSMUSG00000022243 | Slc45a2 | 2976 | 0 | 0 | 0 | 0 | 0 | 0 | 0 | 0 | 0 | 0 | 0 | 0 | 0 | 0 | 0 | 0 | 0 | 0 |
| ENSMUSG00000022244 | Amacr | 2570 | 6.995988117 | 5.651526023 | 7.058394507 | 5.35386591 | 4.213884368 | 5.842209445 | 7.106106641 | 5.394491079 | 5.801369146 | 9.063209109 | 6.915285819 | 4.973457872 | 5.009972922 | 6.072067456 | 5.434757761 | 6.406599682 | 5.309012334 | 4.216651371 |
| ENSMUSG00000022245 | Skor1 | 4575 | 0 | 0 | 0 | 0.039834793 | 0.021041278 | 0 | 0.020683138 | 0 | 0.009002517 | 0.103061646 | 0.093983525 | 0 | 0 | 0.172579123 | 0 | 0 | 0.008377333 | 0 |
| ENSMUSG00000022246 | Rai14 | 4989 | 0.869017429 | 0.641017808 | 0.727202801 | 1.178066691 | 0.771808758 | 0.576079083 | 0.967306725 | 0.918638663 | 1.411684545 | 0.737172751 | 0.737356545 | 0.984521609 | 0.533959417 | 0.279278938 | 0.48725031 | 0.652375552 | 1.221463461 | 0.176417259 |
| ENSMUSG00000022247 | Brix1 | 2720 | 35.25429306 | 29.92036786 | 31.69177614 | 31.35671875 | 31.72813939 | 30.64253598 | 34.00598798 | 30.89091371 | 32.51009167 | 31.35868463 | 33.24926338 | 28.646568 | 32.06574623 | 32.28888575 | 34.06702176 | 29.5626013 | 35.80408839 | 31.93359541 |
| ENSMUSG00000022248 | Rad1 | 7320 | 3.304022563 | 2.211757731 | 3.046475067 | 2.607934095 | 2.610433565 | 2.653643656 | 2.779296713 | 2.113104231 | 1.468535603 | 2.067674282 | 3.067517826 | 1.769021 | 1.846577231 | 2.436411142 | 1.6773311 | 2.008687601 | 2.471313134 | 2.314590829 |
| ENSMUSG00000022249 | Ttc23l | 2171 | 0 | 0 | 0 | 0 | 0 | 0 | 0 | 0 | 0 | 0 | 0 | 0 | 0 | 0 | 0 | 0 | 0 | 0 |
| ENSMUSG00000022253 | 1110020G09R | 3800 | 29.78717994 | 29.02605073 | 30.80630091 | 34.96210665 | 29.52513528 | 32.28751798 | 34.09003024 | 38.32306062 | 32.11464374 | 37.77019493 | 38.84858466 | 38.64500217 | 30.54684246 | 29.38199634 | 32.71186687 | 34.36078191 | 43.09691228 | 38.40504214 |
| ENSMUSG00000022255 | Mtdh | 9938 | 12.59159169 | 12.35573839 | 11.2586009 | 11.45215222 | 11.31860592 | 12.01172622 | 11.03073828 | 10.74905912 | 10.63439318 | 11.81377049 | 10.48472373 | 10.84524496 | 12.08229892 | 11.9685357 | 12.33807912 | 12.49896663 | 11.37293717 | 12.62032233 |
| ENSMUSG00000022257 | Laptm4b | 1890 | 118.22736 | 116.29549 | 120.7034757 | 121.0380971 | 129.3959281 | 126.660982 | 112.5741361 | 121.2255143 | 128.2665777 | 125.4607866 | 133.8204393 | 131.2406373 | 133.8226137 | 113.5791386 | 128.7930117 | 138.1500804 | 133.7364699 | 138.9489288 |
| ENSMUSG00000022261 | Sdc2 | 3720 | 22.72655783 | 20.3011643 | 19.34941214 | 21.37204902 | 23.32845655 | 20.95334675 | 16.87739918 | 16.83751074 | 13.4299042 | 18.17583561 | 16.65702181 | 15.49934932 | 16.65615271 | 15.14426828 | 16.29233568 | 16.64406241 | 19.29708444 | 16.76890511 |
| ENSMUSG00000022262 | Dnahc5 | 15551 | 0 | 0 | 0 | 0.023438258 | 0 | 0.019118796 | 0 | 0 | 0 | 0 | 0 | 0 | 0 | 0 | 0 | 0 | 0 | 0 |
| ENSMUSG00000022263 | Trio | 12310 | 2.783035896 | 3.596493616 | 2.404922874 | 2.013420643 | 3.249198096 | 2.63664017 | 1.979368777 | 1.545070382 | 1.294815727 | 1.796399661 | 1.41267952 | 1.759258902 | 1.743248092 | 1.305414641 | 1.191531592 | 1.670353873 | 1.584734779 | 1.345064267 |
| ENSMUSG00000022265 | Ank | 3596 | 7.789463571 | 9.263874922 | 10.2100972 | 9.882540206 | 10.58741702 | 9.053433248 | 4.065522188 | 5.990132079 | 7.559260401 | 7.919639813 | 8.914626568 | 5.882685239 | 7.39430017 | 6.767714625 | 6.255853592 | 3.620357763 | 6.181660791 | 4.742164373 |
| ENSMUSG00000022269 | 11-Mar | 3572 | 0 | 0 | 0 | 0 | 0.067373913 | 0 | 0 | 0 | 0 | 0 | 0 | 0 | 0 | 0 | 0 | 0 | 0 | 0 |
| ENSMUSG00000022270 | Fam134b | 3437 | 37.86757317 | 37.68418176 | 36.39626113 | 38.60161801 | 36.04642756 | 40.44091291 | 27.60020985 | 28.44706144 | 35.73412569 | 33.50073243 | 24.03343125 | 29.34503995 | 37.54794751 | 33.78243191 | 36.28661905 | 42.61320143 | 39.30759284 | 35.29097478 |
| ENSMUSG00000022272 | Myo10 | 10563 | 4.402221204 | 3.857016911 | 2.683602082 | 2.92008208 | 2.829681397 | 3.532444228 | 2.382878462 | 2.458660763 | 2.608518323 | 2.231880302 | 2.49209525 | 2.150617691 | 2.120296359 | 2.075320387 | 2.06729382 | 2.972479226 | 2.743035166 | 1.98413989 |
| ENSMUSG00000022280 | Rnf19a | 4266 | 15.42381915 | 18.19480627 | 12.84744723 | 15.64625644 | 14.82544484 | 15.43731433 | 11.77826184 | 14.60192413 | 11.90411953 | 11.30688454 | 14.07715021 | 11.97171385 | 9.436203586 | 10.21203797 | 9.918893085 | 12.06342624 | 12.28130517 | 11.79871839 |
| ENSMUSG00000022283 | Pabpc1 | 5945 | 28.4201976 | 24.43692711 | 24.84991786 | 23.56605742 | 22.44267324 | 22.07993124 | 24.07415538 | 24.20029939 | 24.75347701 | 21.99308665 | 21.52886691 | 22.53281585 | 29.5078718 | 30.92887165 | 23.46652115 | 28.36532128 | 23.02156539 | 27.18532474 |
| ENSMUSG00000022285 | Ywhaz | 5198 | 151.2294585 | 176.1071605 | 170.8801009 | 177.695085 | 174.3138636 | 173.1771755 | 147.2991695 | 158.0230855 | 145.9118291 | 154.9950109 | 155.6500427 | 155.6674991 | 130.6944657 | 127.0913234 | 132.7517618 | 150.967239 | 163.701509 | 130.6545655 |
| ENSMUSG00000022286 | Grhl2 | 5635 | 17.4244821 | 17.8062888 | 16.10447967 | 15.62092949 | 16.41695782 | 15.99581586 | 13.24083313 | 15.16173987 | 13.55098496 | 16.24126265 | 17.98237568 | 13.28284002 | 12.79040482 | 15.48685718 | 14.68925036 | 11.5449409 | 14.27629054 | 11.55825774 |
| ENSMUSG00000022288 | 4930447A16R | 583 | 1.093615164 | 1.257090348 | 0.91270812 | 0.312597217 | 0.57791332 | 0.424980562 | 0.24346147 | 0.720611366 | 0.353229123 | 0.970511902 | 0.983361066 | 0.095738701 | 0.930790892 | 0 | 0.494701357 | 1.379250009 | 0.788877466 | 0.377420972 |
| ENSMUSG00000022292 | Rrm2b | 5195 | 8.075574348 | 8.477313986 | 9.590902072 | 7.875614594 | 9.329903202 | 9.471773714 | 9.772185657 | 8.792707079 | 9.577153232 | 9.929330003 | 9.04919329 | 9.809374387 | 8.337521341 | 7.47396671 | 9.548926524 | 8.078249415 | 11.75241405 | 8.619328753 |
| ENSMUSG00000022295 | Atp6v1c1 | 2084 | 73.22148845 | 71.06963901 | 82.37891518 | 83.27352105 | 76.44753711 | 83.79262626 | 72.21767355 | 76.4031313 | 71.6020857 | 70.86180547 | 82.59749299 | 72.87640014 | 82.28298678 | 79.53884256 | 76.78831242 | 80.64178305 | 85.88474402 | 81.51061495 |

| ENSMUSG00000022296 | Baalc | 2492 | 0 | 0.040103805 | 0 | 0.018282923 | 0 | 0 | 0 | 0.030652008 | 0.033054989 | 0 | 0 | 0.022397938 | 0 | 0.018637274 | 0 | 0 | 0 | 0.088297122 |
| --- | --- | --- | --- | --- | --- | --- | --- | --- | --- | --- | --- | --- | --- | --- | --- | --- | --- | --- | --- | --- |
| ENSMUSG00000022297 | Fzd6 | 4074 | 64.6445927 | 65.37471509 | 74.63819632 | 71.30515923 | 66.01894685 | 74.59668999 | 55.03553889 | 52.31065425 | 48.69794954 | 52.31251319 | 49.05317902 | 63.4605177 | 30.00601154 | 22.61783735 | 34.72904834 | 29.57794342 | 40.49943745 | 33.12158537 |
| ENSMUSG00000022299 | Slc25a32 | 2938 | 20.80409909 | 18.5953528 | 21.43714654 | 20.08221661 | 19.34778824 | 20.28997226 | 18.51925826 | 19.14819903 | 18.85495701 | 21.00758018 | 16.37487515 | 20.19470706 | 18.25180193 | 18.30573628 | 15.34189998 | 14.04944683 | 19.38491395 | 14.97865397 |
| ENSMUSG00000022300 | Dcaf13 | 1531 | 73.96067207 | 64.49341507 | 61.80184544 | 62.64271609 | 62.02761946 | 69.61981053 | 54.20407501 | 53.9333599 | 64.3219851 | 63.38089308 | 54.2968176 | 48.0867792 | 57.03298078 | 62.40071049 | 55.94907557 | 64.57632156 | 66.03838748 | 59.60816986 |
| ENSMUSG00000022303 | Dcstamp | 1742 | 0.048800432 | 0.038246721 | 0 | 0 | 0 | 0 | 0.271599764 | 0.131546736 | 0.472864705 | 0 | 0.438805589 | 0.320411381 | 0.424786888 | 0.666533975 | 1.229897191 | 0.219808309 | 0 | 0 |
| ENSMUSG00000022304 | Dpys | 2889 | 0.029425529 | 0.161433201 | 0.117208277 | 0.031541048 | 0.01666041 | 0 | 0 | 0 | 0 | 0.01632077 | 0 | 0 | 0 | 0 | 0 | 0 | 0 | 0 |
| ENSMUSG00000022305 | Lrp12 | 4567 | 3.276072249 | 2.283103983 | 2.563256918 | 2.164822996 | 3.109025067 | 2.875295467 | 3.346178081 | 1.906693179 | 3.652406149 | 1.930628752 | 2.092181234 | 2.395417094 | 1.739093208 | 2.1864416 | 1.813337581 | 2.548794537 | 3.650523134 | 2.541476137 |
| ENSMUSG00000022306 | Zfpm2 | 6359 | 0 | 0 | 0 | 0 | 0 | 0 | 0 | 0 | 0 | 0 | 0 | 0 | 0 | 0 | 0 | 0 | 0 | 0 |
| ENSMUSG00000022307 | Oxr1 | 5060 | 37.09542635 | 37.11161753 | 40.02766237 | 39.61829942 | 40.1796928 | 43.98049931 | 36.8683583 | 41.0077712 | 41.39814608 | 44.15020396 | 45.32956041 | 40.27331702 | 39.39728809 | 35.72339686 | 39.5974637 | 43.92068881 | 55.48981247 | 42.95276298 |
| ENSMUSG00000022309 | Angpt1 | 4247 | 0.010008283 | 0.078438649 | 0.216410896 | 0.06436691 | 0.06799895 | 0.116677027 | 0.011140259 | 0.008992795 | 0.048488952 | 0 | 0 | 0.354844099 | 0.034847128 | 0.01093574 | 0.05820799 | 0.198350215 | 0.279753992 | 0 |
| ENSMUSG00000022311 | Csmd3 | 18781 | 0.04979042 | 0.021285061 | 0.100450864 | 0.03153685 | 0.076883963 | 0.068599706 | 0.030230134 | 0.01016783 | 0.002192988 | 0 | 0 | 0.041606905 | 0 | 0.009891718 | 0.05703852 | 0.016310359 | 0.05101738 | 0.067366458 |
| ENSMUSG00000022312 | Eif3h | 1484 | 91.45486998 | 78.07428982 | 77.61278691 | 84.9817862 | 88.02563399 | 81.77536694 | 87.54758513 | 83.10193219 | 89.72776648 | 85.34150199 | 79.61419968 | 79.09726309 | 94.30906288 | 91.54242292 | 90.67673958 | 88.24385264 | 87.577138 | 88.29628855 |
| ENSMUSG00000022313 | Utp23 | 1295 | 9.354420979 | 8.746242498 | 12.32684557 | 10.20285935 | 10.44406992 | 8.686077623 | 8.439558944 | 10.88262277 | 9.636690561 | 10.70448398 | 9.296748677 | 10.04250918 | 10.5901722 | 12.62418437 | 9.544761982 | 9.520907786 | 13.99871694 | 11.38412402 |
| ENSMUSG00000022314 | Rad21 | 3590 | 21.12234931 | 17.47303059 | 20.02313819 | 16.85377907 | 18.9578106 | 17.15711639 | 16.21019362 | 16.8940209 | 19.37716577 | 16.6800538 | 16.92750618 | 19.15456722 | 15.06066332 | 11.59161624 | 14.093457 | 15.20958389 | 16.59026891 | 19.12294295 |
| ENSMUSG00000022315 | Slc30a8 | 1963 | 0 | 0 | 0 | 0 | 0 | 0 | 0 | 0 | 0 | 0 | 0 | 0 | 0 | 0 | 0 | 0 | 0 | 0 |
| ENSMUSG00000022321 | Cdh10 | 6877 | 0 | 0 | 0.028136414 | 0.006625134 | 0.048992797 | 0 | 0 | 0 | 0 | 0.041137737 | 0 | 0 | 0 | 0.114810162 | 0.017973632 | 0 | 0 | 0 |
| ENSMUSG00000022322 | Shcbp1 | 2119 | 0.260767951 | 0.503073438 | 0.159799298 | 0.473026416 | 0.636004654 | 0.140309769 | 0.424228834 | 0.702927652 | 0.13605739 | 0.511782055 | 0.541103824 | 0.368767945 | 0.651861106 | 0.284933051 | 0.252770282 | 0.072280524 | 0.994783544 | 0.051919874 |
| ENSMUSG00000022323 | Hrsp12 | 2742 | 19.43890982 | 16.93587693 | 18.77076451 | 16.78214981 | 18.67701196 | 17.52959576 | 35.64842986 | 24.7233823 | 23.79264806 | 28.32137428 | 21.69213104 | 23.08350159 | 37.42173156 | 44.19132896 | 33.34290756 | 27.30056071 | 29.40865379 | 31.93818304 |
| ENSMUSG00000022324 | Matn2 | 3813 | 0 | 0.131049938 | 0 | 0.14338645 | 0.126231114 | 0 | 0.024816511 | 0.010016366 | 0.010801604 | 0.173120862 | 0 | 0 | 0.970336689 | 0.682105663 | 0.443027483 | 0.080336968 | 0.050257405 | 0 |
| ENSMUSG00000022325 | Pop1 | 3869 | 0.82395663 | 0.835190163 | 0.762673908 | 1.048057107 | 0.733983845 | 0.858111437 | 0.42800304 | 0.315884434 | 0.415165189 | 0.804328357 | 0.728540333 | 0.432791387 | 0.726781929 | 0.840291062 | 0.97972167 | 0.861019086 | 0.703325686 | 0.924164367 |
| ENSMUSG00000022329 | Stk3 | 4372 | 14.29153906 | 14.02006527 | 14.8152235 | 14.0893257 | 11.94490786 | 14.70034205 | 11.10311267 | 11.45247931 | 12.46334866 | 11.48570425 | 9.987720048 | 12.60065393 | 8.575539142 | 8.455968335 | 9.782071138 | 10.69369434 | 10.28287884 | 8.744585813 |
| ENSMUSG00000022330 | Osr2 | 1719 | 0.024726688 | 0 | 0 | 0 | 0 | 0.028826488 | 0 | 0 | 0 | 0.137145734 | 0 | 0 | 0.028698032 | 0 | 0 | 0 | 0 | 0.704014164 |
| ENSMUSG00000022332 | Khdrbs3 | 1878 | 105.0633797 | 107.5662356 | 94.45459841 | 115.3097144 | 104.2602053 | 101.4801988 | 132.0623338 | 118.0344534 | 115.7082307 | 128.6224988 | 117.4022542 | 129.0774348 | 103.5497433 | 104.4373701 | 101.3145821 | 98.37709303 | 105.3258883 | 102.9882957 |
| ENSMUSG00000022335 | Zfat | 7545 | 0.507019992 | 0.759419192 | 0.589842915 | 0.845400425 | 0.593276196 | 0.709303542 | 0.26964151 | 0.728920603 | 0.376656009 | 0.918641932 | 0.481232186 | 0.628804681 | 0.817294852 | 0.443204015 | 0.300342687 | 0.253748227 | 0.614643065 | 0.262468899 |
| ENSMUSG00000022336 | Eif3e | 1528 | 65.92753088 | 60.67394281 | 68.57137876 | 58.26327266 | 61.77140204 | 61.00045279 | 61.27735052 | 66.38679371 | 72.507675 | 57.08691167 | 57.65512005 | 56.87499126 | 65.79741323 | 74.80294441 | 66.06270414 | 60.64350125 | 66.34362258 | 58.06916829 |
| ENSMUSG00000022337 | Ttc35 | 1234 | 74.60795079 | 71.37706022 | 71.266676 | 77.90421681 | 72.5099239 | 65.73567655 | 81.74281319 | 75.70390298 | 94.02140592 | 84.97825287 | 78.0117839 | 66.76168394 | 75.55698837 | 80.20449775 | 75.02422697 | 90.668683 | 87.67839235 | 77.6992488 |
| ENSMUSG00000022338 | Eny2 | 2424 | 33.00113089 | 26.57885207 | 28.41745348 | 25.28036495 | 28.37480154 | 25.85982177 | 33.27892639 | 31.19676434 | 29.24174655 | 29.48864115 | 27.61250694 | 26.77954438 | 31.42264038 | 28.12702982 | 29.35440884 | 28.76534492 | 31.21126654 | 27.34569868 |
| ENSMUSG00000022339 | Ebag9 | 1554 | 23.52281299 | 21.84416938 | 22.3501897 | 22.42869943 | 24.90223078 | 23.85163753 | 26.91403357 | 21.08692488 | 29.52495403 | 27.76247972 | 25.20943755 | 25.57319933 | 26.41194027 | 29.10974332 | 26.61928064 | 21.51074663 | 28.5844132 | 28.07092769 |
| ENSMUSG00000022340 | Sybu | 4753 | 0.00894281 | 0.014017629 | 0 | 0 | 0 | 0 | 0 | 0 | 0 | 0 | 0 | 0 | 0 | 0 | 0 | 0.016112185 | 0 | 0 |
| ENSMUSG00000022342 | Kcnv1 | 4493 | 0 | 0 | 0 | 0 | 0 | 0 | 0 | 0 | 0 | 0 | 0 | 0 | 0 | 0 | 0 | 0 | 0 | 0 |
| ENSMUSG00000022346 | Myc | 3100 | 0.850103521 | 1.354007959 | 0.390109116 | 1.146374664 | 1.164482024 | 1.32673448 | 2.625090573 | 3.092352582 | 0.558010859 | 1.292841864 | 4.561737971 | 1.566439563 | 1.941449648 | 5.003975867 | 2.179695215 | 2.149214738 | 1.372328696 | 1.366355232 |
| ENSMUSG00000022347 | A1bg | 1816 | 0 | 0 | 0 | 0 | 0 | 0 | 0 | 0 | 0 | 0.051928087 | 0 | 0 | 0.162990916 | 0.25574938 | 0 | 0 | 0 | 0.121165433 |
| ENSMUSG00000022350 | E430025E21R | 4060 | 14.96056565 | 20.43081443 | 18.76559367 | 20.0311488 | 16.72762175 | 20.78529687 | 15.30087375 | 16.08596259 | 15.92680536 | 18.45380972 | 17.62731227 | 19.026817 | 13.46299614 | 13.79595305 | 16.1964737 | 17.2496357 | 18.32299071 | 17.00404654 |
| ENSMUSG00000022351 | Sqle | 6583 | 6.114598465 | 7.104861535 | 5.922689574 | 6.484991424 | 5.841927238 | 5.991793396 | 5.735305755 | 6.683525379 | 4.360777987 | 7.047894882 | 7.177492623 | 6.155578162 | 12.43976647 | 12.33241148 | 11.99808334 | 12.25555367 | 13.21014244 | 12.74326109 |
| ENSMUSG00000022353 | Mtss1 | 5883 | 5.46938947 | 4.161988314 | 5.500916506 | 4.12008764 | 4.69619653 | 4.615824919 | 5.726096787 | 5.790858906 | 8.023074463 | 5.23362387 | 6.033791288 | 5.635645687 | 7.287002569 | 6.418331302 | 8.033007752 | 6.573774176 | 6.202045666 | 5.86277578 |
| ENSMUSG00000022354 | Ndufb9 | 647 | 551.3847641 | 471.7867847 | 487.5483639 | 451.0332132 | 521.044812 | 476.6865746 | 631.6644831 | 574.4798458 | 581.9584654 | 575.3551812 | 595.7470866 | 549.9611267 | 692.3242795 | 686.2526668 | 594.3340357 | 596.3747307 | 584.4316001 | 639.0238735 |
| ENSMUSG00000022357 | Klhl38 | 2208 | 0.327259055 | 0.362096676 | 0.219083018 | 0.041269062 | 0.043597757 | 0.112211806 | 0 | 0.034594567 | 0.018653313 | 0.256253822 | 0 | 0.101115331 | 0.424504723 | 0.231379059 | 0.0746405 | 0.398860494 | 0.138863395 | 0.099654179 |
| ENSMUSG00000022358 | Fbxo32 | 2713 | 21.18208551 | 21.80748254 | 17.79461235 | 22.23473008 | 22.67327624 | 20.73068655 | 14.42225782 | 18.72314604 | 18.88537619 | 17.44906232 | 17.99705402 | 20.34710295 | 14.25588763 | 13.09610278 | 18.83152573 | 17.50105167 | 20.75242541 | 18.65402808 |
| ENSMUSG00000022359 | Wdyhv1 | 1675 | 9.313074389 | 7.597328711 | 10.36781934 | 9.493017596 | 7.67237231 | 8.727197854 | 8.841115525 | 8.527736418 | 8.950383127 | 9.852385755 | 7.016501365 | 8.997151581 | 8.894471055 | 11.39613129 | 10.03596108 | 11.54433238 | 9.633057311 | 8.932822107 |
| ENSMUSG00000022360 | Atad2 | 5671 | 0.269826545 | 0.458191809 | 0.554448858 | 0.867676387 | 0.373444656 | 0.567964677 | 0.775891226 | 0.922651936 | 0.392183363 | 0.573690447 | 0.48019267 | 0.374007261 | 1.017780695 | 0.384918375 | 0.603020479 | 0.94528038 | 0.54742198 | 1.202808893 |
| ENSMUSG00000022361 | Zhx1 | 7757 | 6.411119757 | 6.716690287 | 5.980432591 | 6.760443732 | 6.577264289 | 7.474113483 | 8.20972874 | 6.587783217 | 6.578586184 | 8.631429499 | 7.403055317 | 7.296259103 | 6.474138428 | 7.753653883 | 7.170568577 | 7.547549145 | 8.389590324 | 7.488670452 |

| ENSMUSG00000022362 | 9130401M01R | 1598 | 16.51796891 | 14.40501246 | 16.13460055 | 12.71603615 | 10.99383738 | 13.70607525 | 18.14935681 | 14.7941784 | 14.40754838 | 14.63501177 | 14.05145838 | 17.21972569 | 15.3120344 | 18.77526937 | 16.73329773 | 16.150106 | 14.98994716 | 18.00360627 |
| --- | --- | --- | --- | --- | --- | --- | --- | --- | --- | --- | --- | --- | --- | --- | --- | --- | --- | --- | --- | --- |
| ENSMUSG00000022364 | Wdr67 | 6334 | 0.523429702 | 0.762609672 | 1.046285706 | 0.525095711 | 0.349552966 | 0.453750955 | 0.664797667 | 0.850193991 | 0.897338045 | 0.64018953 | 0.399758888 | 0.590408809 | 0.529613258 | 0.161315112 | 0.286212261 | 0.828199118 | 0.532477759 | 0.312650433 |
| ENSMUSG00000022365 | Derl1 | 3137 | 41.15021345 | 41.98890142 | 47.23242699 | 48.9450811 | 47.74834123 | 44.49794405 | 45.80446474 | 47.74963412 | 43.98304999 | 48.81908963 | 48.20138463 | 47.47089187 | 46.01376792 | 46.2072033 | 45.37818795 | 44.49131781 | 47.01293927 | 43.54083902 |
| ENSMUSG00000022366 | Slc22a22 | 2757 | 0 | 0 | 0 | 0 | 0 | 0 | 0 | 0 | 0 | 0.273634839 | 0 | 0 | 0 | 0.101075272 | 0 | 0 | 0.055605799 | 0 |
| ENSMUSG00000022367 | Has2 | 4233 | 0 | 0 | 0 | 0 | 0 | 0 | 0 | 0 | 0 | 0 | 0 | 0 | 0 | 0 | 0 | 0 | 0 | 0 |
| ENSMUSG00000022369 | Mtbp | 5598 | 0.744106333 | 1.618632945 | 1.823296696 | 0.813880749 | 1.272512451 | 1.389742617 | 1.056463892 | 1.173471457 | 0.65480527 | 1.558213666 | 1.72392669 | 1.525508463 | 1.092739861 | 2.115620272 | 1.74433168 | 1.614252116 | 1.643142418 | 1.788434695 |
| ENSMUSG00000022370 | Mrpl13 | 8171 | 7.64687416 | 6.196989256 | 6.57728457 | 6.367728874 | 6.838962597 | 5.937110044 | 8.158556432 | 6.730762387 | 8.251416755 | 6.612985673 | 7.963467562 | 6.885593916 | 8.041869271 | 7.286907359 | 7.422431804 | 8.655336173 | 7.570510733 | 6.819755857 |
| ENSMUSG00000022371 | Col14a1 | 6961 | 0.024424753 | 0.014356943 | 0.006949221 | 0.052361493 | 0 | 0.007118623 | 0.006796822 | 0.005486626 | 0.005916753 | 0.020320659 | 0 | 0 | 0.170085622 | 0.053376339 | 0.112459354 | 0.170522745 | 0.016517582 | 0.071122247 |
| ENSMUSG00000022372 | Sla | 8555 | 0.00993692 | 0 | 0 | 0 | 0 | 0 | 0.503267537 | 0.437505019 | 0.255159011 | 0.132275497 | 0.279222434 | 0 | 0.288322135 | 0.162866467 | 0.317861214 | 0.09399214 | 0 | 0 |
| ENSMUSG00000022375 | Lrrc6 | 2072 | 1.189816703 | 0.434096595 | 0.210116686 | 0.593700867 | 0.16260785 | 0.382646591 | 0.296845959 | 0.479248293 | 0.417430903 | 0.568903273 | 0.253631536 | 0.377132855 | 0.238088404 | 0.0896604 | 0.278388891 | 0.794640984 | 0.647403664 | 1.008854274 |
| ENSMUSG00000022376 | Adcy8 | 5014 | 0.661229304 | 0.823852988 | 0.627100015 | 0.972283954 | 0.499174318 | 0.711566976 | 0.754889173 | 0.83788677 | 0.558572611 | 0.573632409 | 0.381132497 | 0.567730114 | 0.18693786 | 0.944813904 | 0.443734347 | 0.030546954 | 0.321041976 | 0.482728499 |
| ENSMUSG00000022377 | Asap1 | 8424 | 1.508671371 | 1.747898771 | 3.95647702 | 1.411613553 | 1.999783153 | 2.335284334 | 1.965745207 | 1.786301822 | 2.977515206 | 1.645572977 | 1.979280687 | 2.246143117 | 2.863640497 | 1.990303365 | 3.863868608 | 3.354519024 | 2.138338028 | 3.225842678 |
| ENSMUSG00000022378 | Fam49b | 4591 | 7.175236642 | 7.343203866 | 8.587328957 | 8.53463257 | 6.97184257 | 8.343337629 | 7.502424365 | 7.736644356 | 8.558470058 | 8.627007348 | 7.055417523 | 7.513413086 | 8.295412794 | 6.565500483 | 9.916732577 | 8.006748662 | 8.498403452 | 7.404841637 |
| ENSMUSG00000022382 | Wnt7b | 4407 | 0.453311385 | 0.385513412 | 0.471990426 | 0.589285121 | 0.251199057 | 0.281102414 | 0.021471604 | 0.25132282 | 0.121494147 | 0.192582859 | 0.021681397 | 0.025330457 | 1.712680586 | 2.466057737 | 1.96331444 | 0.894924566 | 1.208839408 | 2.409066848 |
| ENSMUSG00000022383 | Ppara | 3824 | 0.35569185 | 1.454825663 | 1.492697851 | 1.584628373 | 1.183159211 | 1.5550021 | 1.286746498 | 0.858929552 | 1.378628143 | 2.379729532 | 1.998952238 | 1.342845439 | 1.470669082 | 2.064721458 | 1.098995476 | 2.613454114 | 1.934355466 | 1.064506772 |
| ENSMUSG00000022385 | Gtse1 | 2767 | 0.046084398 | 0.252826447 | 0.15734072 | 0.955020084 | 0.260924776 | 0.644704882 | 0.341978164 | 0.400281774 | 1.175907028 | 0.068161479 | 0.224457702 | 0.383266205 | 0 | 0.268559956 | 1.265678443 | 0.152221424 | 0.443238707 | 0 |
| ENSMUSG00000022386 | Trmu | 2418 | 11.46127989 | 11.4211701 | 12.94362041 | 11.60694926 | 12.50076429 | 10.04170366 | 14.4794799 | 14.12076416 | 15.34700194 | 10.82243189 | 16.12256663 | 13.87312374 | 16.93361925 | 14.80909486 | 14.02352378 | 15.15471931 | 15.26394703 | 12.73990892 |
| ENSMUSG00000022387 | Brd1 | 8315 | 13.40842775 | 11.8748549 | 12.24609518 | 12.10395032 | 11.9591767 | 11.69837835 | 9.473915865 | 10.56893782 | 10.79319517 | 12.11798591 | 11.79578951 | 11.69343166 | 11.97255671 | 12.42232716 | 12.49673168 | 12.71441576 | 11.45871005 | 10.94889617 |
| ENSMUSG00000022388 | Ttll8 | 3415 | 0.074679665 | 0 | 0.014165016 | 0.013341448 | 0.183225478 | 0.029020635 | 0.277087431 | 0.145388355 | 0.217088516 | 0.055227764 | 0.013989739 | 0.179786907 | 0.115565253 | 0.12240023 | 0.229232668 | 0.538198874 | 0.101006346 | 0 |
| ENSMUSG00000022389 | Tef | 8068 | 11.69050392 | 14.33181158 | 13.94006669 | 14.22512031 | 12.55796967 | 14.92478218 | 12.79576914 | 15.07246025 | 11.77195158 | 16.21755101 | 13.16949773 | 13.14449168 | 15.61036004 | 17.18339128 | 17.24051223 | 12.32532318 | 16.83544822 | 17.60455052 |
| ENSMUSG00000022390 | Zc3h7b | 5121 | 3.087663636 | 2.894793581 | 2.975524714 | 3.576555326 | 2.838477045 | 3.212557615 | 2.531473155 | 2.050949163 | 2.187606383 | 2.651709146 | 1.931149464 | 3.400602759 | 2.726212183 | 2.639177784 | 1.987265047 | 2.325401074 | 2.162917361 | 2.545822748 |
| ENSMUSG00000022391 | Rangap1 | 4097 | 10.73782211 | 11.50542966 | 10.08323039 | 9.863960538 | 9.468960331 | 8.647840983 | 9.434820279 | 12.34238242 | 11.93272985 | 11.32445497 | 8.232638683 | 9.38662229 | 10.77668195 | 10.51992021 | 8.950301377 | 9.196474904 | 8.634408602 | 8.405101491 |
| ENSMUSG00000022394 | L3mbtl2 | 6009 | 2.94261162 | 3.10454664 | 3.099319219 | 3.586349471 | 3.732647101 | 3.636670912 | 2.952613513 | 2.980901436 | 3.749213533 | 3.978267025 | 5.239423804 | 2.591540999 | 4.260819615 | 3.67131661 | 4.950494817 | 3.84244238 | 4.254225334 | 3.542773224 |
| ENSMUSG00000022400 | Rbx1 | 1746 | 131.897505 | 112.8364584 | 128.6082061 | 114.3723123 | 116.2498981 | 116.7015123 | 150.1757541 | 134.8546167 | 147.3609187 | 131.4062555 | 128.7132902 | 132.4742874 | 152.1208721 | 146.0089322 | 138.2116339 | 128.2274808 | 130.5201383 | 144.3595229 |
| ENSMUSG00000022401 | Xpnpep3 | 5928 | 2.466562172 | 2.427660307 | 3.019265559 | 2.766865041 | 2.476423198 | 4.254780936 | 3.016901592 | 3.427523293 | 3.126506763 | 2.569108831 | 2.812662029 | 2.928250854 | 2.196967976 | 2.185880631 | 1.542973247 | 1.924865217 | 2.793009491 | 3.646858796 |
| ENSMUSG00000022403 | St13 | 3572 | 60.75907861 | 58.53071784 | 62.6878701 | 58.34160604 | 57.64512023 | 59.69356455 | 53.18040479 | 53.54634693 | 54.30808769 | 51.1371289 | 54.40888328 | 48.90902125 | 49.84291418 | 54.37546849 | 51.73263667 | 49.96431162 | 54.76411517 | 49.64987684 |
| ENSMUSG00000022404 | Slc25a17 | 1658 | 58.3741169 | 60.39720146 | 55.93007098 | 58.77869351 | 59.97620886 | 60.3718467 | 55.70226132 | 57.26556838 | 58.77400259 | 53.71995082 | 66.41814191 | 54.53641338 | 44.45228252 | 45.32360277 | 46.51948892 | 49.28356827 | 52.82001714 | 55.80537847 |
| ENSMUSG00000022407 | Adsl | 4573 | 11.27460716 | 11.59722886 | 10.94830613 | 11.24828757 | 10.7462703 | 10.80342781 | 10.23228503 | 11.04097004 | 9.808028792 | 9.681721049 | 9.924821901 | 9.825411848 | 10.14038973 | 8.541324622 | 10.27110731 | 9.177018524 | 8.079280607 | 11.68026298 |
| ENSMUSG00000022408 | Fam83f | 5305 | 3.789811171 | 3.102086662 | 4.659542698 | 3.847567929 | 3.193673352 | 4.707743209 | 2.300974772 | 4.621964627 | 3.330634357 | 3.821828916 | 4.241659839 | 4.282181842 | 2.473570216 | 2.862811796 | 2.93575648 | 3.356292637 | 2.362431496 | 3.131526905 |
| ENSMUSG00000022412 | Smcr7l | 5607 | 6.678626731 | 7.985113221 | 6.76383945 | 8.377641646 | 6.446776293 | 7.3440916 | 7.509949036 | 7.28836643 | 8.146218295 | 8.577442005 | 9.611227602 | 8.322078459 | 7.012223661 | 7.819371945 | 9.302866017 | 8.488536651 | 8.024803382 | 9.810791283 |
| ENSMUSG00000022414 | Tab1 | 2920 | 4.512535811 | 6.662578844 | 5.798197132 | 5.211434523 | 6.313194098 | 6.329852611 | 6.286753227 | 7.363809094 | 5.289364182 | 6.733507965 | 5.890063374 | 5.1992672 | 5.913072276 | 9.336534006 | 6.575316796 | 5.061703581 | 5.079546884 | 6.066072728 |
| ENSMUSG00000022415 | Syngr1 | 4949 | 1.520189161 | 1.299128831 | 1.534581585 | 1.003466121 | 1.468563442 | 1.471863374 | 0.736123717 | 1.034104248 | 0.857185517 | 1.324297384 | 0.752969643 | 1.612778289 | 0.538275113 | 0.778916802 | 0.432911882 | 0.889759517 | 1.04547385 | 0.733602959 |
| ENSMUSG00000022416 | Cacna1i | 10068 | 0 | 0 | 0 | 0 | 0 | 0 | 0.014097938 | 0 | 0 | 0 | 0 | 0 | 0 | 0 | 0 | 0 | 0 | 0 |
| ENSMUSG00000022419 | Deptor | 9058 | 26.17038714 | 25.91697566 | 23.15606399 | 26.28134871 | 26.12769581 | 26.66372527 | 22.43408655 | 24.53117655 | 27.91389051 | 26.63613917 | 29.04578233 | 25.01167746 | 27.06229818 | 24.01166496 | 28.77922766 | 34.0041561 | 36.15568636 | 30.82040369 |
| ENSMUSG00000022420 | Dnalc4 | 2547 | 17.62287628 | 16.45371846 | 18.38460046 | 19.15817765 | 21.2407861 | 20.03899316 | 23.57274816 | 20.97808047 | 20.37495479 | 23.23287499 | 20.70810921 | 20.4460201 | 22.0996143 | 19.51125776 | 17.90739002 | 20.46074467 | 19.68229146 | 19.11388278 |
| ENSMUSG00000022421 | Nptxr | 5639 | 0.007537715 | 0.047260712 | 0.051470328 | 0.024238896 | 0.017071085 | 0.096662541 | 0 | 0.040637421 | 0.131469637 | 0.025084609 | 0 | 0.118777789 | 0.05249007 | 0.057653593 | 0.036532679 | 0.061112869 | 0.027186591 | 0.058530704 |
| ENSMUSG00000022422 | Dscc1 | 2017 | 0.105367318 | 0.115612424 | 0.55160694 | 0.158119638 | 0.143178752 | 0.147405256 | 0.211112598 | 0.340834528 | 0.306295358 | 0 | 0.189489176 | 0.553452281 | 0.122290325 | 0.06907896 | 0.306407208 | 0.189839402 | 0.323027787 | 0.327272821 |
| ENSMUSG00000022425 | Enpp2 | 4081 | 21.7993956 | 22.41539023 | 22.88882311 | 25.32037455 | 24.83847862 | 27.70873267 | 62.23338899 | 56.58203013 | 55.88084729 | 63.67251302 | 52.66835045 | 61.94293944 | 49.71873947 | 44.75976371 | 50.55040195 | 39.38837782 | 51.07042454 | 57.75888809 |
| ENSMUSG00000022426 | Josd1 | 3390 | 9.642029606 | 11.71356045 | 9.360777555 | 13.96398969 | 10.57766464 | 10.94837683 | 11.20710359 | 13.62083024 | 10.32700248 | 13.93657955 | 13.41644852 | 12.4473867 | 10.43391879 | 8.61750176 | 10.68323529 | 7.86143444 | 11.75791999 | 10.90446009 |
| ENSMUSG00000022427 | Tomm22 | 1109 | 135.4493166 | 114.9880605 | 123.6163977 | 107.3087897 | 114.8395582 | 118.6763394 | 119.6255651 | 110.1001898 | 123.96807 | 109.4797664 | 105.2427625 | 112.6379196 | 126.9106943 | 117.3875356 | 117.1773736 | 114.5265507 | 104.1618204 | 127.0318506 |
| ENSMUSG00000022428 | Cby1 | 1114 | 29.76125431 | 25.17904572 | 24.92496088 | 27.19757135 | 26.22628157 | 25.35463029 | 30.06945842 | 27.01581068 | 26.65662286 | 34.02977147 | 26.16043507 | 26.65523563 | 27.76580981 | 29.64250102 | 31.43745288 | 34.33780682 | 31.41104944 | 28.78842838 |

| ENSMUSG00000022429 | Dmc1 | 3166 | 0 | 0.052610383 | 0.076395342 | 0.057562911 | 0 | 0.04695458 | 0.044831976 | 0.036189895 | 0.013009007 | 0.029785662 | 0 | 0 | 0.140236025 | 0 | 0 | 0 | 0.036316769 | 0 |
| --- | --- | --- | --- | --- | --- | --- | --- | --- | --- | --- | --- | --- | --- | --- | --- | --- | --- | --- | --- | --- |
| ENSMUSG00000022431 | Ribc2 | 1645 | 0.025839013 | 0.101254998 | 0 | 0 | 0 | 0 | 0 | 0.023217266 | 0 | 0 | 0.348510335 | 0 | 0.209923052 | 0.112933951 | 0.425791155 | 0.162938755 | 0.372778572 | 0.267521492 |
| ENSMUSG00000022432 | Smc1b | 4013 | 0.042367482 | 0.033204978 | 0.132596271 | 0.034060088 | 0.011994 | 0 | 0 | 0.066620189 | 0 | 0.10574541 | 0.011905048 | 0.069543562 | 0 | 0 | 0.102670212 | 0.114499698 | 0.315167655 | 0.082246359 |
| ENSMUSG00000022433 | Csnk1e | 7727 | 13.95019107 | 13.81318922 | 11.48769616 | 13.58517486 | 12.83185748 | 13.46715938 | 9.919314076 | 12.332088 | 11.22541765 | 11.84411997 | 11.27753647 | 11.08800855 | 7.25262819 | 8.216522255 | 8.840711747 | 9.519380978 | 8.213840775 | 11.22678417 |
| ENSMUSG00000022434 | Fam118a | 2534 | 40.27424137 | 37.41456075 | 37.74051678 | 42.1628449 | 38.31179559 | 35.17970312 | 31.08745478 | 31.10857086 | 27.97237318 | 31.46481421 | 35.55784202 | 28.78889937 | 31.20720734 | 36.94999218 | 34.30753075 | 47.82548241 | 40.14127545 | 42.63531397 |
| ENSMUSG00000022435 | Upk3a | 1100 | 1.197873143 | 2.271333697 | 2.154820898 | 0.704125231 | 1.794008064 | 1.621725826 | 2.580691578 | 2.117942315 | 1.610018343 | 3.814920536 | 2.95336107 | 3.044490687 | 5.695594086 | 3.166642322 | 3.183756592 | 3.306916094 | 3.762945514 | 3.950654028 |
| ENSMUSG00000022436 | Sh3bp1 | 3706 | 3.57841741 | 4.045008742 | 2.375602408 | 4.462671099 | 4.143033904 | 3.222398487 | 5.693862602 | 6.636780127 | 4.612089596 | 7.875414279 | 5.104933502 | 3.855588133 | 8.652387002 | 8.371465293 | 5.469823394 | 7.997012987 | 10.0934878 | 5.996675423 |
| ENSMUSG00000022437 | Samm50 | 1658 | 77.08869983 | 81.81550378 | 75.53623045 | 80.35011679 | 82.7067856 | 76.57062933 | 80.04346465 | 80.94577928 | 68.95884666 | 81.02071989 | 84.83080685 | 71.36863974 | 84.73902316 | 79.19025033 | 82.10490993 | 82.23935643 | 78.94107595 | 81.91645627 |
| ENSMUSG00000022438 | Parvb | 4521 | 0.01880344 | 0 | 0 | 0 | 0 | 0 | 0 | 0 | 0 | 0.062575585 | 0 | 0 | 0.021823454 | 0.09245671 | 0 | 0.279493485 | 0.033909575 | 0 |
| ENSMUSG00000022439 | Parvg | 3075 | 0.262633608 | 0 | 0 | 0 | 0 | 0 | 0.107703659 | 0 | 0.013393989 | 0 | 0.357341153 | 0.127060045 | 0.0641716 | 0.181245219 | 0.16078656 | 0.08716561 | 0 | 0 |
| ENSMUSG00000022440 | C1qtnf6 | 4029 | 1.360925219 | 1.63711915 | 1.092576635 | 1.402226234 | 1.314100671 | 1.795656268 | 0.669352866 | 0.805746886 | 0.429345659 | 1.20539152 | 1.007910516 | 0.235509125 | 0.685675693 | 1.118162441 | 0.582896174 | 0.608239979 | 0.913210351 | 1.215142839 |
| ENSMUSG00000022441 | Efcab6 | 6828 | 0 | 0 | 0 | 0 | 0 | 0 | 0 | 0 | 0 | 0 | 0 | 0 | 0 | 0 | 0 | 0.016823641 | 0 | 0 |
| ENSMUSG00000022442 | Ttll1 | 3389 | 34.71653209 | 36.13402158 | 34.62796832 | 37.99218392 | 40.57624843 | 38.63036946 | 31.04850928 | 27.73428808 | 27.21056618 | 27.93703516 | 31.57742903 | 26.26910057 | 22.19863495 | 23.87300095 | 23.13560368 | 30.91268865 | 26.39527205 | 25.32139466 |
| ENSMUSG00000022443 | Myh9 | 12053 | 4.302357484 | 4.963905917 | 3.736476957 | 4.271466033 | 4.560412912 | 5.266493961 | 2.535799435 | 3.716891066 | 3.495711078 | 4.639569739 | 3.301795438 | 3.700050975 | 3.679531538 | 2.897697977 | 2.628722847 | 3.291219553 | 3.02718283 | 2.980249456 |
| ENSMUSG00000022445 | Cyp2d26 | 1636 | 0 | 0.020362405 | 0 | 0 | 0 | 0 | 0 | 0.023344989 | 0.050350264 | 0 | 0 | 0 | 0 | 0 | 0 | 0 | 0 | 0 |
| ENSMUSG00000022449 | Adamts20 | 8956 | 0 | 0 | 0 | 0 | 0 | 0 | 0 | 0 | 0 | 0 | 0 | 0 | 0 | 0.005185807 | 0 | 0 | 0 | 0 |
| ENSMUSG00000022450 | Ndufa6 | 557 | 279.2979251 | 231.455836 | 252.6366244 | 220.1162842 | 253.7079495 | 221.0745834 | 295.3432399 | 248.0106275 | 278.2492977 | 271.2223577 | 283.3046461 | 266.2517334 | 313.0849687 | 291.8389692 | 304.0926324 | 266.3156429 | 248.1232076 | 298.2540436 |
| ENSMUSG00000022451 | Twf1 | 3369 | 52.96430067 | 53.44499651 | 52.75284967 | 60.72101189 | 51.84646809 | 58.74550843 | 42.62213729 | 40.1309304 | 40.78308596 | 46.85679861 | 40.47187044 | 39.03285873 | 35.43580879 | 34.4229404 | 35.02560287 | 39.58628245 | 51.55677577 | 38.76272465 |
| ENSMUSG00000022452 | 1500032L24R | 1022 | 161.1619933 | 139.4447764 | 131.4889113 | 128.4803618 | 143.124184 | 132.9002375 | 172.9088609 | 158.5246292 | 164.1818642 | 162.5361327 | 163.566125 | 142.2152499 | 191.5835418 | 210.4980556 | 159.9281529 | 175.7921036 | 152.667666 | 176.8149859 |
| ENSMUSG00000022453 | Naga | 1903 | 74.80285576 | 82.17063871 | 68.40418822 | 73.54888502 | 77.74962341 | 75.95655483 | 66.48139961 | 62.99839787 | 77.7630852 | 75.09920209 | 77.77552358 | 76.93351706 | 71.39259074 | 57.15819898 | 74.5655063 | 69.09613548 | 72.74544636 | 73.27802709 |
| ENSMUSG00000022454 | Nell2 | 4002 | 0.127451802 | 0.074916554 | 0.205484762 | 0.034153706 | 0.04810787 | 0.074291954 | 0.283734206 | 0.019086658 | 0.308744496 | 0.07069071 | 0.059688854 | 0.041840827 | 0.295843582 | 0 | 0.144133379 | 0 | 0.047883929 | 0 |
| ENSMUSG00000022455 | Wbp2nl | 1312 | 0 | 0 | 0 | 0.104179217 | 0.073371835 | 0.226613111 | 0.108184479 | 0.087330188 | 0 | 0 | 0 | 0 | 0 | 0 | 0 | 0 | 0 | 0 |
| ENSMUSG00000022456 | 3-Sep | 4960 | 0.591301844 | 0.846254974 | 0.175549102 | 0.293942222 | 0.786025366 | 0.269742703 | 0.133543852 | 0.146301541 | 0 | 0 | 0 | 0.225063156 | 0.228756874 | 0 | 0.024920296 | 0 | 0.054089532 | 0.410350191 |
| ENSMUSG00000022462 | Slc38a2 | 4610 | 37.1297926 | 43.51632299 | 37.59704105 | 42.19091069 | 42.97418608 | 41.3727704 | 26.89946453 | 30.26395791 | 22.84466829 | 23.35033743 | 28.72715508 | 29.73606666 | 21.07040024 | 24.18920907 | 22.49551327 | 26.55424552 | 22.81287607 | 29.01998862 |
| ENSMUSG00000022463 | Srebf2 | 3719 | 30.03592433 | 32.00510111 | 26.48252428 | 30.63946542 | 31.88950253 | 31.67164499 | 25.73636716 | 31.40423946 | 26.41297125 | 39.12532139 | 38.79547583 | 31.8925203 | 53.98787398 | 53.99952511 | 48.2032723 | 59.4693596 | 54.81515817 | 52.98268897 |
| ENSMUSG00000022464 | Slc38a4 | 4338 | 21.79149642 | 29.82648251 | 23.49539614 | 27.73783266 | 27.86059482 | 25.24470752 | 14.14581479 | 14.09544404 | 11.3172722 | 11.9887565 | 11.16731395 | 11.30981268 | 22.51664274 | 23.07215498 | 17.02959652 | 16.86799233 | 19.0217882 | 18.88164129 |
| ENSMUSG00000022466 | Rpap3 | 2243 | 106.1966146 | 118.1174533 | 105.438337 | 98.23148034 | 110.8343227 | 103.3692556 | 91.39805519 | 108.2941058 | 106.9417155 | 106.1574014 | 107.7332769 | 101.3291922 | 86.8312124 | 82.12093205 | 86.48101878 | 97.85187768 | 96.95203238 | 101.9005075 |
| ENSMUSG00000022468 | Endou | 2707 | 0.408250675 | 1.255232808 | 1.179406355 | 0.690063841 | 0.337830273 | 1.208156785 | 0.436947534 | 0.860634114 | 0.745526144 | 0.34836131 | 0.264730099 | 1.010331536 | 0.656058006 | 0.343140653 | 0.441390883 | 0.41020599 | 0.396430112 | 0.548668593 |
| ENSMUSG00000022469 | Rapgef3 | 6622 | 3.081015478 | 3.184394751 | 2.41794602 | 2.490652076 | 1.838927316 | 3.165328647 | 4.394034663 | 3.437431567 | 3.097385208 | 3.382147999 | 4.105096855 | 4.787571179 | 3.426862269 | 4.257257784 | 2.557209222 | 4.828247838 | 3.599963255 | 4.876222538 |
| ENSMUSG00000022471 | Xrcc6 | 3918 | 9.948250745 | 11.25734353 | 11.14887644 | 9.675035449 | 11.22832523 | 10.95269711 | 9.805486291 | 10.96642489 | 13.4660354 | 10.81890817 | 10.25491069 | 9.872448742 | 10.46320144 | 8.997208353 | 9.516949485 | 10.4180162 | 10.72118974 | 8.550420111 |
| ENSMUSG00000022472 | Pppde2 | 4622 | 26.37491234 | 26.73248045 | 23.89371913 | 26.67420726 | 26.47151665 | 24.49761926 | 19.33657518 | 21.67431231 | 16.69916282 | 19.19896658 | 22.84350027 | 22.48566935 | 22.17908354 | 22.72966804 | 20.68992021 | 23.23780912 | 23.87308714 | 23.255689 |
| ENSMUSG00000022474 | Pmm1 | 2978 | 22.29452148 | 21.6791098 | 24.414176 | 22.13795539 | 22.48203688 | 22.92937101 | 31.83835076 | 31.51065571 | 33.15113441 | 33.45514976 | 35.93549597 | 34.2241101 | 45.98569589 | 45.80466241 | 47.39977518 | 47.47109554 | 46.74315324 | 50.37267765 |
| ENSMUSG00000022475 | Hdac7 | 6577 | 5.881056741 | 7.729280307 | 6.273775489 | 6.033703758 | 5.883847747 | 6.193149917 | 4.517616294 | 5.934717236 | 6.537740985 | 4.487812109 | 3.777250784 | 5.43984183 | 4.905439243 | 4.300509232 | 5.161940407 | 5.280459315 | 3.945097005 | 5.52851141 |
| ENSMUSG00000022476 | Polr3h | 2508 | 7.541787613 | 5.738106181 | 5.959896683 | 6.848689679 | 8.137135421 | 7.68581075 | 6.017840741 | 7.583658851 | 8.243871975 | 7.952451147 | 6.838600516 | 7.321911081 | 10.12995909 | 7.092538066 | 7.343339507 | 7.496287175 | 6.433561003 | 7.369641092 |
| ENSMUSG00000022477 | Aco2 | 3938 | 115.9124138 | 121.7807073 | 109.6941661 | 135.9195401 | 118.5453346 | 123.2149231 | 124.481124 | 126.8651132 | 116.0083374 | 139.6800671 | 141.6264259 | 112.2549639 | 121.6386279 | 110.0128104 | 110.4846191 | 109.310058 | 121.8694489 | 118.483302 |
| ENSMUSG00000022479 | Vdr | 5730 | 1.453929232 | 2.232487152 | 3.292439239 | 4.206246502 | 4.107593486 | 3.545658075 | 7.728563259 | 9.278154302 | 7.540079413 | 10.35998873 | 10.12195455 | 10.59815722 | 10.04718106 | 7.562361873 | 8.09650121 | 14.32055352 | 12.70854522 | 9.388988896 |
| ENSMUSG00000022483 | Col2a1 | 6556 | 0 | 0 | 0 | 0 | 0 | 0 | 0 | 0 | 0 | 0 | 0 | 0 | 0 | 0 | 0 | 0 | 0 | 0 |
| ENSMUSG00000022484 | Hoxc10 | 1907 | 0 | 0 | 0 | 0 | 0.025239603 | 0 | 0 | 0 | 0 | 0 | 0 | 0 | 0 | 0 | 0 | 0 | 0 | 0 |
| ENSMUSG00000022485 | Hoxc5 | 2921 | 0.087309502 | 0.01140462 | 0 | 0.093586534 | 0 | 0 | 0 | 0 | 0 | 0.096851838 | 0 | 0 | 0 | 0 | 0.084631748 | 0.104869859 | 0 | 0 |
| ENSMUSG00000022487 | Gtsf1 | 1472 | 0.086627397 | 0 | 0 | 0 | 0.032698318 | 0 | 0 | 0.155675553 | 0 | 0 | 0 | 0 | 0 | 0 | 0 | 0 | 0 | 0 |
| ENSMUSG00000022488 | Nckap1l | 5414 | 0.08636072 | 0 | 0 | 0.008415413 | 0 | 0 | 0.26216852 | 0.112870048 | 0.395585301 | 0 | 0.476514178 | 0.206190109 | 0.082007251 | 0.283091039 | 0.205475066 | 0 | 0 | 0 |

| ENSMUSG00000022489 | Pde1b | 3779 | 0 | 0 | 0 | 0 | 0 | 0 | 0.025039788 | 0.13138429 | 0.010898787 | 0 | 0 | 0.088619734 | 0 | 0.294961127 | 0 | 0 | 0 | 0 |
| --- | --- | --- | --- | --- | --- | --- | --- | --- | --- | --- | --- | --- | --- | --- | --- | --- | --- | --- | --- | --- |
| ENSMUSG00000022490 | Ppp1r1a | 1288 | 0.033000913 | 0.025864048 | 0 | 0.106120445 | 0 | 0 | 0 | 0 | 0 | 0.073215378 | 0.037092359 | 0 | 0 | 0.18029537 | 0.127955143 | 0 | 0.119025767 | 0 |
| ENSMUSG00000022491 | Glycam1 | 621 | 0 | 0 | 0 | 0 | 0 | 0 | 0 | 0 | 0 | 0 | 0 | 0 | 0 | 0 | 0 | 0 | 0 | 0 |
| ENSMUSG00000022494 | Shisa9 | 1270 | 0.033468643 | 0.157383752 | 0.038089394 | 0.215249028 | 0 | 0.117053701 | 0.186270389 | 0.390945851 | 0.291872946 | 0.445518456 | 0.526653086 | 0.219746703 | 0.271908205 | 0.365701476 | 0.42174821 | 0 | 0.241425492 | 0 |
| ENSMUSG00000022496 | Tnfrsf17 | 812 | 0 | 0 | 0 | 0 | 0 | 0 | 0 | 0 | 0 | 0.058067369 | 0 | 0 | 0 | 0 | 0 | 0 | 0 | 0 |
| ENSMUSG00000022498 | Txndc11 | 9040 | 7.208012705 | 8.383499375 | 7.898156062 | 7.922783373 | 7.001491109 | 8.68269137 | 6.568297794 | 7.562433662 | 8.182630786 | 7.797599709 | 8.360617734 | 8.088331636 | 8.905939048 | 9.242578895 | 7.406253133 | 7.560700687 | 8.754845476 | 8.987660468 |
| ENSMUSG00000022500 | Litaf | 2863 | 133.1274584 | 146.5511361 | 139.291437 | 141.6323069 | 138.209062 | 142.2542498 | 110.9030347 | 127.6237212 | 119.7904704 | 119.614236 | 126.6044743 | 111.7288028 | 91.44410932 | 90.55218156 | 98.37716943 | 99.67862276 | 102.2748545 | 103.9466529 |
| ENSMUSG00000022501 | Prm1 | 467 | 0.36407003 | 0.214001462 | 0 | 0.097561123 | 0 | 0.106108637 | 0 | 0 | 0.264581472 | 0 | 0 | 0 | 0 | 0 | 0 | 0.081992735 | 0 | 0 |
| ENSMUSG00000022503 | Nubp1 | 3151 | 9.537022993 | 9.388083169 | 11.22216778 | 9.02256162 | 9.852456606 | 8.96383946 | 7.087142004 | 7.102744465 | 8.548391402 | 7.556682052 | 7.232197777 | 7.581435604 | 7.23305166 | 6.42641133 | 8.342301701 | 7.910880806 | 8.30747724 | 7.21001621 |
| ENSMUSG00000022504 | Ciita | 6091 | 0.104675364 | 0.076568793 | 0.071476239 | 0.149601196 | 0.047412829 | 0.130166432 | 0.085444011 | 0.44519136 | 0.175808473 | 0.100633581 | 0.149027124 | 0.045818144 | 0.153883833 | 0.122000558 | 0.081172003 | 0.119442052 | 0.094384248 | 0.027093633 |
| ENSMUSG00000022505 | Emp2 | 3615 | 70.24230196 | 74.67064505 | 79.68585706 | 78.64479022 | 72.16459925 | 79.03763812 | 41.90738532 | 51.22958762 | 53.69627906 | 58.71990763 | 57.79250164 | 46.5516522 | 49.19545277 | 41.06094144 | 48.50728129 | 47.41044501 | 53.17972483 | 44.17467132 |
| ENSMUSG00000022507 | 1810013L24R | 4062 | 10.45363635 | 13.60563553 | 11.15854208 | 10.22936298 | 10.84212461 | 10.60101563 | 12.54449907 | 11.49909112 | 9.59193597 | 13.69715161 | 12.52593077 | 10.42936679 | 10.52948604 | 15.16121612 | 11.23863218 | 12.45245996 | 14.93612211 | 7.136828962 |
| ENSMUSG00000022508 | Bcl6 | 5350 | 2.685373738 | 2.596537736 | 2.95666251 | 2.324890674 | 3.03186136 | 2.389645844 | 1.715637329 | 2.555678516 | 1.562778075 | 2.626338238 | 2.839707812 | 2.441283186 | 1.069626618 | 2.265776974 | 1.74047694 | 1.36701047 | 1.855422598 | 1.758235 |
| ENSMUSG00000022510 | Trp63 | 8961 | 1.271218299 | 1.029759145 | 1.408937777 | 2.257460516 | 1.455613359 | 1.647886911 | 2.06969871 | 1.457627678 | 1.604072537 | 1.762692288 | 1.295537876 | 2.049252649 | 1.365284621 | 1.192070093 | 1.97248828 | 0.61104306 | 0.96660452 | 1.559235923 |
| ENSMUSG00000022512 | Cldn1 | 3243 | 4.312119307 | 4.550605038 | 3.893151842 | 6.406363311 | 8.845706601 | 5.454926268 | 5.500117162 | 7.254554388 | 6.591366537 | 7.66217102 | 6.997565611 | 7.590103978 | 8.807641105 | 12.40227557 | 10.45602236 | 9.245003267 | 11.4045256 | 11.05949355 |
| ENSMUSG00000022514 | Il1rap | 8400 | 0.52119442 | 0.503659234 | 0.454941535 | 0.650872062 | 0.716248864 | 0.578115225 | 0.895561423 | 1.177599074 | 0.593281953 | 0.420988422 | 0.790561813 | 0.6113144 | 1.098222444 | 1.354619216 | 1.373385196 | 0.724786497 | 0.857779026 | 0.681065131 |
| ENSMUSG00000022515 | Anks3 | 2537 | 10.80640069 | 12.63185031 | 12.31742239 | 12.2657443 | 9.182440306 | 10.33243834 | 11.35728083 | 13.44336435 | 15.893417 | 12.45209743 | 12.84293326 | 12.51837289 | 13.68926676 | 14.88334373 | 12.39132327 | 12.69310241 | 12.8862165 | 11.49185123 |
| ENSMUSG00000022516 | Nudt16l1 | 1065 | 65.81317866 | 64.06085198 | 66.85994055 | 65.83891759 | 61.64501773 | 65.18627206 | 80.72031701 | 66.6305011 | 70.84854167 | 70.96955617 | 72.40261313 | 65.30170477 | 69.48157363 | 70.42929684 | 82.13230343 | 80.24848424 | 69.70707709 | 75.51484885 |
| ENSMUSG00000022517 | Mgrn1 | 3378 | 72.89297949 | 83.29209727 | 79.30509506 | 86.8465259 | 82.48540737 | 87.95683556 | 77.66394454 | 77.08579029 | 59.74361375 | 74.25747227 | 90.04830096 | 76.12278198 | 73.07783128 | 75.19322496 | 77.01202602 | 69.28128847 | 80.37403412 | 86.1288234 |
| ENSMUSG00000022518 | 4930562C15R | 4365 | 0 | 0.06105456 | 0.022164275 | 0 | 0 | 0.011352287 | 0 | 0.052498148 | 0.018871256 | 0.075613957 | 0.021890015 | 0 | 0.033905098 | 0.117041228 | 0.009439074 | 0.017544379 | 0.008780366 | 0.012602315 |
| ENSMUSG00000022519 | Srl | 8373 | 0.248746402 | 0.115379665 | 0.138655766 | 0.527818142 | 0.27592647 | 0.035508946 | 0.423796837 | 0.150525413 | 0.241029413 | 0.551865391 | 0.142645881 | 0.479962702 | 0.265130333 | 0.08320331 | 0.359215763 | 0.050304154 | 0.169362592 | 0.00656982 |
| ENSMUSG00000022521 | Crebbp | 7477 | 10.72151424 | 10.89786536 | 10.28026478 | 9.591157387 | 10.55078546 | 10.66341425 | 8.302024175 | 10.07805403 | 9.105431397 | 9.988861035 | 9.769681893 | 9.428270944 | 9.461277167 | 8.416709698 | 8.441993263 | 9.586734928 | 10.14414091 | 9.630456159 |
| ENSMUSG00000022523 | Fgf12 | 3074 | 10.78530817 | 11.0320515 | 12.47892309 | 12.33142124 | 10.49069254 | 11.18724694 | 14.08298673 | 12.87160992 | 15.71625992 | 13.20649171 | 14.54696198 | 14.83454663 | 9.227668326 | 11.99629323 | 11.28552702 | 10.16432519 | 12.43048732 | 10.62960618 |
| ENSMUSG00000022525 | Hrasls | 3280 | 0.349890169 | 0.55860036 | 0.324456606 | 0.333373495 | 0.616323413 | 0.558979007 | 0.966448015 | 0.966454085 | 0.57761577 | 1.020640223 | 0.509793764 | 0.170169703 | 0.015040219 | 0.637190223 | 0.803932798 | 0.046695863 | 0.350545399 | 0.318650313 |
| ENSMUSG00000022526 | Zfp251 | 3493 | 3.906144147 | 4.24398452 | 4.21000665 | 4.721757243 | 3.858270434 | 4.085653384 | 3.453974557 | 3.619148353 | 4.115114229 | 4.980993273 | 6.660865955 | 6.791198568 | 2.655138977 | 2.712451711 | 5.036663145 | 3.661340645 | 4.904624883 | 5.29145716 |
| ENSMUSG00000022528 | Hes1 | 1901 | 15.65156403 | 12.5120497 | 12.95219724 | 18.02309592 | 10.22898325 | 12.32953339 | 16.87427476 | 17.69989822 | 25.00222998 | 20.36334949 | 13.84744983 | 20.99326604 | 29.66143159 | 34.22838844 | 30.60315461 | 39.39843665 | 28.64895734 | 35.7660473 |
| ENSMUSG00000022529 | Zfp263 | 4098 | 10.66259662 | 9.98248758 | 10.75360814 | 10.07279311 | 10.3357962 | 10.55625584 | 10.58704895 | 12.90787636 | 11.13583686 | 10.33219413 | 12.83558548 | 9.874659682 | 10.70182393 | 12.60269038 | 12.6781752 | 11.52081965 | 11.99917983 | 12.16160339 |
| ENSMUSG00000022533 | Atp13a3 | 9494 | 3.366746616 | 3.645681177 | 4.519414517 | 3.575203079 | 3.604571069 | 3.768387786 | 2.800687335 | 3.974520073 | 3.526926197 | 2.8606283 | 3.512420583 | 3.345177166 | 2.686391535 | 2.494889885 | 2.621207053 | 2.181927386 | 3.116484223 | 2.103255292 |
| ENSMUSG00000022534 | Mefv | 4088 | 0 | 0 | 0 | 0 | 0 | 0 | 0 | 0 | 0 | 0 | 0 | 0 | 0 | 0 | 0 | 0 | 0 | 0 |
| ENSMUSG00000022536 | Glyr1 | 3332 | 55.84863765 | 56.22800633 | 55.42921335 | 67.90400545 | 63.05397563 | 62.75886426 | 57.18131994 | 59.14549712 | 58.2940001 | 63.4666579 | 75.20397876 | 60.30503761 | 56.69024948 | 66.73898272 | 71.50918299 | 67.97387238 | 76.74461373 | 65.8062123 |
| ENSMUSG00000022537 | Tmem44 | 9673 | 0.083489956 | 0.058546387 | 0.08001411 | 0.061231632 | 0.024879522 | 0.28175337 | 0.21032205 | 0.153985702 | 0.234183642 | 0.204727544 | 0.044451011 | 0.236580396 | 0.219298299 | 0.024007075 | 0.221490841 | 0.003958504 | 0.091130449 | 0.119424299 |
| ENSMUSG00000022538 | Lsg1 | 6033 | 9.257716111 | 10.34781432 | 11.21739913 | 9.28137303 | 9.158867623 | 8.172545982 | 8.955922317 | 9.109707784 | 9.755599374 | 10.35549176 | 8.267372228 | 8.363560248 | 8.888412743 | 10.52363127 | 9.315253147 | 9.209294106 | 8.855769581 | 10.95076035 |
| ENSMUSG00000022540 | Rogdi | 1449 | 142.9452883 | 141.1832184 | 140.8140449 | 138.6011618 | 149.7437625 | 144.5544547 | 152.5499213 | 150.6873461 | 127.8231618 | 155.249141 | 162.5469602 | 132.046992 | 157.0858981 | 165.0383754 | 152.0675783 | 140.1610639 | 146.1900918 | 162.9013644 |
| ENSMUSG00000022542 | 12-Sep | 1612 | 0 | 0 | 0 | 0 | 0 | 0 | 0 | 0 | 0 | 0 | 0 | 0 | 0 | 0 | 0 | 0 | 0 | 0 |
| ENSMUSG00000022543 | 4930451G09R | 2200 | 1.275155281 | 1.49908024 | 1.231326227 | 0.476320009 | 1.159541797 | 0.608147185 | 0.881736289 | 1.458255365 | 0.973499463 | 1.350224684 | 1.259521633 | 0.735751916 | 0.852096753 | 0.485551823 | 1.273502637 | 1.392385724 | 2.003420065 | 0.45007451 |
| ENSMUSG00000022544 | Fam86 | 3146 | 8.552376486 | 7.634646132 | 7.98024865 | 8.153486319 | 8.598264813 | 7.765256724 | 10.52729665 | 11.66652852 | 10.7613846 | 9.681930802 | 11.49575447 | 10.39668731 | 11.88607543 | 9.581122923 | 10.59505999 | 9.566566248 | 9.892229207 | 9.074928922 |
| ENSMUSG00000022545 | Ercc4 | 6943 | 7.523961017 | 8.785238271 | 7.35740286 | 6.522782381 | 7.09881749 | 7.351190492 | 7.420928612 | 7.310629814 | 6.958344086 | 7.212162878 | 9.117358488 | 6.921688821 | 5.961324873 | 7.572332842 | 6.159760192 | 5.97825413 | 8.357484302 | 5.379689394 |
| ENSMUSG00000022546 | Gpt | 1769 | 3.027502645 | 2.711733616 | 2.050884554 | 2.652791163 | 2.911314772 | 2.885207212 | 2.24661675 | 2.547599482 | 3.049990709 | 3.358388135 | 3.780946403 | 3.691595547 | 4.294581832 | 4.069436713 | 3.610085451 | 2.770603588 | 2.838182529 | 1.959058069 |
| ENSMUSG00000022548 | Apod | 2790 | 0 | 0.179101582 | 0 | 0.244951851 | 0.034503171 | 0.106565018 | 0.271327191 | 0.191646464 | 0.059048768 | 0.152099043 | 0 | 0.040011228 | 0.194498599 | 0.149819637 | 0.103373079 | 0.054896928 | 0 | 0.13801568 |
| ENSMUSG00000022550 | Adck5 | 3486 | 7.82797562 | 7.434719364 | 10.42126256 | 7.305973547 | 5.826641362 | 9.822415061 | 8.740494902 | 10.38622991 | 13.12628199 | 9.481538435 | 9.346678624 | 10.71161167 | 7.712534399 | 9.645874719 | 14.27753285 | 12.03858454 | 10.9173875 | 11.61408564 |
| ENSMUSG00000022551 | Cyc1 | 1187 | 229.7499658 | 246.3245767 | 226.666871 | 267.8390627 | 240.8218152 | 242.0444392 | 270.6428727 | 242.0568183 | 241.8103019 | 258.514555 | 292.9285154 | 232.4790529 | 237.1838685 | 222.3603611 | 207.1185206 | 231.2275264 | 213.7813073 | 216.5607041 |

| ENSMUSG00000022552 | Sharpin | 3850 | 18.1944234 | 18.47351407 | 17.42703548 | 19.02913228 | 18.09010222 | 18.36668852 | 19.09711768 | 19.84020904 | 20.32581297 | 18.61534258 | 20.81002737 | 19.32526707 | 20.75784571 | 19.18080492 | 19.62692299 | 18.55851257 | 18.5160811 | 19.07458636 |
| --- | --- | --- | --- | --- | --- | --- | --- | --- | --- | --- | --- | --- | --- | --- | --- | --- | --- | --- | --- | --- |
| ENSMUSG00000022553 | Maf1 | 2651 | 76.60872541 | 74.51733788 | 79.08369693 | 76.11839508 | 73.35080031 | 81.16105967 | 76.88533415 | 83.87633603 | 86.33476729 | 84.28788485 | 86.17874442 | 89.50297309 | 75.27257842 | 67.44991944 | 78.30005227 | 73.80799842 | 78.56095714 | 78.14571706 |
| ENSMUSG00000022554 | Fam203a | 1953 | 7.595651016 | 7.556381024 | 7.108654998 | 5.248968241 | 6.530957384 | 5.556604533 | 7.727979815 | 7.118297222 | 5.272211439 | 7.701522959 | 6.482521248 | 7.630712704 | 7.24949322 | 6.729993206 | 5.885936556 | 8.567842004 | 7.535738869 | 5.74595892 |
| ENSMUSG00000022555 | Dgat1 | 3110 | 17.84944048 | 19.29148633 | 17.68511383 | 20.56839429 | 19.02062193 | 18.03655769 | 15.66947244 | 18.66638317 | 18.05055337 | 17.38966451 | 15.33099954 | 20.2264475 | 18.35274221 | 11.21527641 | 15.46051953 | 17.27386565 | 12.6070102 | 13.97337438 |
| ENSMUSG00000022556 | Hsf1 | 2433 | 23.27040463 | 24.38564102 | 26.78139967 | 31.25416482 | 24.55064417 | 21.65004348 | 21.35196114 | 20.29707206 | 25.52785276 | 21.25948273 | 18.75260392 | 18.5363976 | 23.80422149 | 20.8263458 | 22.75992237 | 20.91583117 | 21.0613477 | 28.64633712 |
| ENSMUSG00000022557 | Bop1 | 2697 | 24.36522141 | 28.12512426 | 21.82817443 | 26.74198487 | 28.46511615 | 31.67553307 | 22.68271926 | 20.74596571 | 13.82046599 | 17.22040886 | 16.7575494 | 17.96366152 | 18.87672919 | 17.75448799 | 17.58360802 | 15.98636408 | 20.16500382 | 17.9692336 |
| ENSMUSG00000022558 | Heatr7a | 14972 | 6.844775541 | 8.937877109 | 7.066050687 | 7.814638115 | 6.837870802 | 8.505912723 | 5.763981189 | 5.362037794 | 5.633848803 | 6.862234999 | 5.076807303 | 5.099908257 | 6.197791638 | 6.343718856 | 6.720157593 | 6.764537575 | 5.931206915 | 5.011516268 |
| ENSMUSG00000022559 | Fbxl6 | 1703 | 23.98559845 | 21.4000624 | 18.23594039 | 22.12506381 | 22.80825743 | 21.735697 | 19.41959047 | 18.21035276 | 20.19421061 | 21.06969183 | 23.64902525 | 20.15656635 | 21.78367692 | 24.51746011 | 24.04835382 | 23.56345071 | 18.58926673 | 22.64320835 |
| ENSMUSG00000022560 | Slc52a2 | 2695 | 9.683925076 | 10.24726876 | 11.75683205 | 10.90422026 | 9.59066531 | 9.800225231 | 9.322089985 | 8.913922491 | 8.665215005 | 11.45963289 | 10.0513549 | 8.387882504 | 10.21417805 | 9.219883767 | 8.102717856 | 8.112778043 | 9.016279123 | 7.45021297 |
| ENSMUSG00000022561 | Gpaa1 | 2742 | 35.06444499 | 31.39333285 | 31.61391918 | 32.40118033 | 31.61400238 | 32.6917925 | 36.90803072 | 33.83272992 | 26.6766054 | 28.32137428 | 43.34941528 | 32.48789113 | 29.41563995 | 36.33208149 | 30.06721858 | 30.21913728 | 30.40105608 | 30.01226246 |
| ENSMUSG00000022562 | Oplah | 5583 | 5.679538119 | 7.965737736 | 6.429009408 | 8.862492235 | 7.638345758 | 10.10048555 | 4.576186033 | 5.233241598 | 4.234472514 | 8.58053278 | 5.545078469 | 6.96821007 | 3.410732593 | 4.958029032 | 4.309817059 | 5.04094509 | 5.491856981 | 4.433834502 |
| ENSMUSG00000022564 | Grina | 1686 | 202.1390874 | 226.4723568 | 210.8523574 | 205.1327921 | 210.4558371 | 218.2847878 | 166.2959284 | 173.6100664 | 166.7003461 | 191.930769 | 179.9070056 | 167.8111469 | 202.5651621 | 178.9722632 | 205.3235306 | 182.6639118 | 175.1734543 | 207.4097814 |
| ENSMUSG00000022565 | Plec | 25477 | 6.423241657 | 7.177237366 | 6.429044777 | 6.248392233 | 6.247685032 | 7.387105316 | 5.079097887 | 5.967890802 | 5.069706536 | 5.748325325 | 5.299369339 | 6.046678523 | 6.0452269 | 4.232961923 | 4.956736225 | 6.976684836 | 4.997447043 | 4.320493879 |
| ENSMUSG00000022568 | Scrib | 6070 | 10.46873775 | 13.26478836 | 11.5474869 | 11.95695941 | 10.48276822 | 12.49839129 | 9.478124807 | 10.46987769 | 9.858979803 | 9.896210206 | 9.161458265 | 10.69417061 | 10.14270721 | 9.158743427 | 9.04805173 | 9.083768477 | 8.827044987 | 8.156210223 |
| ENSMUSG00000022570 | Tsta3 | 1300 | 71.89914007 | 77.20903868 | 67.7229425 | 61.54245681 | 66.3850301 | 75.47262499 | 70.93262402 | 76.50231989 | 75.33964191 | 76.56548814 | 68.83269018 | 69.68370798 | 79.15875341 | 75.38232645 | 76.53981344 | 76.5812148 | 80.07247876 | 71.55415344 |
| ENSMUSG00000022571 | Pycrl | 1347 | 38.93941145 | 37.41826945 | 37.70765172 | 43.43012689 | 42.62909052 | 38.95793976 | 39.72579055 | 42.30368551 | 40.97248042 | 53.10142304 | 46.3207838 | 43.67461646 | 42.1170786 | 37.37593967 | 37.71456452 | 39.68350997 | 38.29784382 | 42.26757644 |
| ENSMUSG00000022574 | Naprt1 | 1877 | 43.77331127 | 48.43414402 | 47.80655238 | 45.22121769 | 44.8239758 | 42.63594285 | 28.15570717 | 46.63664694 | 43.8196441 | 43.00586253 | 48.23310939 | 42.37470388 | 30.51377515 | 26.3521327 | 30.81885161 | 38.71900525 | 32.996961 | 28.42772164 |
| ENSMUSG00000022575 | Gsdmd | 1765 | 5.466671364 | 7.32317448 | 10.90802554 | 8.05385033 | 7.853820972 | 7.383778431 | 5.039537472 | 6.772930285 | 5.413751647 | 5.262713055 | 6.956467042 | 7.463170748 | 2.655259004 | 3.105043803 | 1.844148961 | 3.406020035 | 3.170333908 | 5.76582705 |
| ENSMUSG00000022577 | Ly6h | 4896 | 0 | 0 | 0 | 0 | 0 | 0 | 0 | 0 | 0 | 0 | 0 | 0 | 0 | 0 | 0 | 0 | 0 | 0 |
| ENSMUSG00000022579 | Gpihbp1 | 792 | 0 | 0 | 0 | 0 | 0 | 0 | 0 | 0 | 0 | 0 | 0 | 0 | 0 | 0 | 0 | 0 | 0 | 0 |
| ENSMUSG00000022580 | Rhpn1 | 2966 | 0.85984847 | 1.493801393 | 1.027489013 | 1.612916269 | 0.535520391 | 0.568035382 | 1.052810792 | 2.189045317 | 1.721890747 | 0.604088578 | 0.322150765 | 0.225821966 | 0.149692264 | 0.892553264 | 1.611389239 | 0.361475727 | 0.439343929 | 1.001514418 |
| ENSMUSG00000022582 | Ly6g | 334 | 0 | 0 | 0 | 0 | 0 | 0 | 0 | 0 | 0 | 0 | 0 | 0 | 0 | 0 | 0 | 0 | 0 | 0 |
| ENSMUSG00000022583 | Ly6f | 873 | 1.265904441 | 0.763181998 | 0.221642751 | 0.678457705 | 0.716741131 | 0.340568616 | 123.6199549 | 75.5973326 | 83.03352548 | 43.74807519 | 23.58649153 | 48.78276123 | 319.4990381 | 393.4181286 | 372.3242548 | 55.70340367 | 54.04315176 | 254.6297827 |
| ENSMUSG00000022584 | Ly6c2 | 793 | 0 | 0 | 0.061000669 | 0 | 0.060695995 | 0 | 0 | 0 | 0 | 0 | 0 | 0 | 0 | 0 | 0 | 0 | 0 | 0 |
| ENSMUSG00000022586 | Ly6i | 875 | 0 | 0 | 0 | 0 | 0 | 0 | 0 | 0 | 0 | 0 | 0 | 0.063789329 | 0 | 0 | 0 | 0 | 0 | 0 |
| ENSMUSG00000022587 | Ly6e | 2559 | 888.6388892 | 656.1169135 | 1830.670642 | 612.5191904 | 712.894166 | 1323.710557 | 975.928002 | 764.9972529 | 2472.414148 | 727.3995558 | 764.5860295 | 1577.474078 | 1266.552155 | 764.3945403 | 2063.314025 | 876.6588927 | 802.7105154 | 1529.141386 |
| ENSMUSG00000022589 | Cyp11b2 | 1616 | 0 | 0 | 0 | 0 | 0 | 0 | 0 | 0 | 0 | 0 | 0 | 0 | 0 | 0 | 0 | 0 | 0 | 0 |
| ENSMUSG00000022591 | Gm9747 | 2821 | 0 | 0 | 0 | 0 | 0 | 0 | 0 | 0 | 0 | 0 | 0 | 0 | 0 | 0 | 0 | 0 | 0 | 0 |
| ENSMUSG00000022594 | Lynx1 | 3948 | 0.129195064 | 0.270013327 | 0.085768671 | 0.115402848 | 0.13410617 | 0.238475668 | 0.059919806 | 0.164455633 | 0.073025737 | 0.274687481 | 0.157313693 | 0.282754116 | 0.124954198 | 0.694073241 | 0.907937022 | 0.135782296 | 0.398018787 | 0.348335276 |
| ENSMUSG00000022595 | Lypd2 | 472 | 0 | 0 | 0 | 0 | 0 | 0 | 0 | 0 | 0 | 0 | 0 | 0 | 0 | 0 | 0 | 0 | 0 | 0 |
| ENSMUSG00000022596 | Slurp1 | 522 | 0 | 0 | 0 | 0 | 0 | 0 | 0 | 0 | 0 | 0 | 0 | 0 | 0 | 0 | 0 | 0 | 0 | 0 |
| ENSMUSG00000022598 | Psca | 851 | 0 | 0 | 0.056843161 | 0 | 0.226237009 | 0 | 0 | 0.134638316 | 0.193591143 | 0 | 0.280698933 | 0 | 6.724444658 | 1.85558046 | 2.130280211 | 0.26996903 | 0.540441319 | 1.422092066 |
| ENSMUSG00000022601 | Rpl24 | 6107 | 11.44932284 | 10.25515656 | 10.55868937 | 9.04208052 | 10.43501997 | 10.94590431 | 11.24906006 | 11.21319429 | 11.97086384 | 10.3072194 | 10.63925717 | 9.980465621 | 13.39320761 | 13.47616225 | 10.59217992 | 11.71854351 | 12.3005636 | 10.06143314 |
| ENSMUSG00000022602 | Arc | 3054 | 1.155183238 | 0.970807985 | 0.586057833 | 1.626114549 | 0.23640434 | 1.573875297 | 0.325332763 | 3.213964446 | 0.310180047 | 1.590216908 | 0.078217024 | 0.255867477 | 5.766697599 | 2.448427659 | 9.915895082 | 3.309993567 | 3.990753901 | 9.834634011 |
| ENSMUSG00000022603 | 1700016M24R | 3966 | 0 | 0.00839962 | 0 | 0 | 0 | 0 | 0 | 0 | 0 | 0 | 0 | 0 | 0 | 0 | 0 | 0 | 0 | 0 |
| ENSMUSG00000022604 | Cep97 | 9532 | 0.985485093 | 0.712949058 | 1.365136348 | 1.166270963 | 0.843268071 | 0.873359131 | 1.186291467 | 1.246101253 | 1.162313548 | 0.70735948 | 1.222942705 | 1.253100272 | 1.257622314 | 1.724843363 | 0.81261986 | 1.586738347 | 0.731786198 | 1.540855172 |
| ENSMUSG00000022607 | Ptk2 | 5083 | 16.57392463 | 19.05185589 | 19.22379133 | 19.98841803 | 19.72433543 | 20.51130267 | 19.70508387 | 17.15389626 | 15.94630396 | 18.50593213 | 19.12690154 | 18.51371378 | 14.33469247 | 15.56055102 | 16.6086933 | 15.45033165 | 18.32242799 | 14.92377694 |
| ENSMUSG00000022610 | Mapk12 | 1877 | 3.532662472 | 3.851304233 | 2.860661625 | 2.961346515 | 2.589943682 | 2.270396957 | 3.780981268 | 3.337002661 | 4.388547231 | 4.16996097 | 2.494377161 | 2.706033722 | 8.568036778 | 6.458128294 | 7.068141181 | 7.89476029 | 5.063890054 | 5.56831661 |
| ENSMUSG00000022613 | Miox | 1694 | 0 | 0 | 0 | 0 | 0 | 0 | 0 | 0 | 0 | 0 | 0 | 0 | 0 | 0 | 0 | 0 | 0 | 0 |
| ENSMUSG00000022614 | Lmf2 | 2892 | 23.38372582 | 27.49580861 | 23.28352498 | 25.15940104 | 27.39458726 | 27.50073907 | 24.71972955 | 24.15418534 | 22.04589433 | 26.1350544 | 23.19365204 | 24.49172746 | 21.9878428 | 20.42768989 | 21.12790712 | 19.93971464 | 21.61486523 | 24.42313037 |
| ENSMUSG00000022615 | Tymp | 1407 | 2.326153912 | 2.628096132 | 2.68168825 | 2.266718624 | 2.086742959 | 2.394872696 | 2.219358073 | 2.144420604 | 2.078352963 | 2.71443281 | 1.86753569 | 1.54712924 | 2.279015368 | 3.267920861 | 3.484708707 | 2.476506946 | 2.124698762 | 3.440512716 |

| ENSMUSG00000022617 | Chkb | 5434 | 10.73971415 | 11.13290686 | 10.55778561 | 11.96459519 | 11.99312194 | 10.74220099 | 13.3562108 | 13.84597584 | 13.33976219 | 12.33866397 | 12.96799112 | 14.47262948 | 14.91577845 | 14.22211288 | 14.99754832 | 16.48173182 | 14.25422268 | 15.11383812 |
| --- | --- | --- | --- | --- | --- | --- | --- | --- | --- | --- | --- | --- | --- | --- | --- | --- | --- | --- | --- | --- |
| ENSMUSG00000022619 | Mapk8ip2 | 5688 | 0.037363903 | 0.093707157 | 0.059531419 | 0 | 0.042310059 | 0.017423605 | 0.182995594 | 0.167863935 | 0 | 0.049737029 | 0.109190306 | 0.147193203 | 0.121421737 | 0.106148582 | 0 | 0.309663843 | 0.363857249 | 0.415856468 |
| ENSMUSG00000022620 | Arsa | 4599 | 2.375262065 | 4.30988738 | 3.460511304 | 3.427728058 | 2.742023049 | 3.092331928 | 3.775549721 | 3.355018607 | 3.528481672 | 4.295747916 | 4.279904956 | 6.153194375 | 2.606578799 | 3.140706932 | 3.019118142 | 2.198025735 | 3.125105749 | 4.114618985 |
| ENSMUSG00000022621 | Rabl2 | 3118 | 6.448027024 | 6.506591591 | 6.748712542 | 7.043112049 | 7.239856382 | 6.865548719 | 8.634032813 | 8.770288686 | 8.202959041 | 7.394706188 | 9.407897532 | 8.216609726 | 7.214674239 | 8.624479345 | 6.422051367 | 10.53667131 | 6.440981263 | 9.226992565 |
| ENSMUSG00000022622 | Acr | 1498 | 0 | 0 | 0 | 0 | 0 | 0 | 0 | 0 | 0 | 0 | 0 | 0 | 0 | 0 | 0 | 0 | 0 | 0 |
| ENSMUSG00000022623 | Shank3 | 8934 | 0 | 0.003728777 | 0 | 0 | 0 | 0 | 0.0052958 | 0.0170998 | 0 | 0.01055534 | 0 | 0 | 0 | 0 | 0.00461177 | 0 | 0.008579874 | 0 |
| ENSMUSG00000022629 | Kif21a | 6461 | 17.9533548 | 17.01478888 | 15.57296752 | 14.69574623 | 14.03506333 | 16.63517708 | 14.62365266 | 14.84898852 | 15.96208566 | 14.59548158 | 15.28414165 | 17.34682719 | 16.1563747 | 13.61478123 | 15.59167376 | 20.23872841 | 17.95006569 | 15.98082237 |
| ENSMUSG00000022634 | Yaf2 | 3209 | 18.37166692 | 17.33640802 | 21.13421301 | 17.52020215 | 15.47901067 | 17.26393149 | 14.92067033 | 15.85300094 | 17.64769684 | 16.11851713 | 14.76870016 | 17.13257328 | 16.37223182 | 16.19536734 | 18.20623442 | 18.99615051 | 19.9693264 | 20.26200192 |
| ENSMUSG00000022635 | Zcrb1 | 2957 | 19.83670711 | 17.42815264 | 18.53473449 | 16.80997611 | 17.43296931 | 18.63464313 | 20.44829343 | 17.69482289 | 20.4052234 | 19.13454309 | 18.01456838 | 20.00832003 | 20.78713863 | 20.6226198 | 20.3290734 | 23.80051958 | 20.94531478 | 23.06773498 |
| ENSMUSG00000022636 | Alcam | 7664 | 62.52653373 | 61.65319567 | 64.97984016 | 50.31094966 | 62.70213021 | 57.93868027 | 53.52925874 | 68.39649309 | 67.22903343 | 51.56185334 | 62.51762247 | 72.11465174 | 49.94341677 | 45.29268126 | 52.43736641 | 51.72529468 | 57.85950615 | 41.63006511 |
| ENSMUSG00000022637 | Cblb | 6632 | 1.281820749 | 1.632492554 | 1.60467079 | 1.600682046 | 1.57488351 | 1.56159851 | 1.062965796 | 2.027099766 | 1.881712044 | 1.471682083 | 2.305184969 | 1.792632107 | 1.301731834 | 1.28855731 | 1.391608643 | 1.131628325 | 1.727919598 | 1.94091239 |
| ENSMUSG00000022639 | 5330426P16R | 2629 | 13.51629028 | 9.541502224 | 8.463987814 | 7.244015417 | 9.227268744 | 10.15934705 | 16.7367027 | 11.95600882 | 10.80969794 | 10.94025446 | 14.22890547 | 11.63445534 | 15.31184652 | 12.86089601 | 14.16744257 | 12.90432794 | 13.33912578 | 13.66334983 |
| ENSMUSG00000022641 | Bbx | 15633 | 13.16238452 | 12.76642674 | 13.53765722 | 14.21360028 | 14.06426325 | 14.18147061 | 13.85514259 | 14.89046841 | 13.63926259 | 15.18606735 | 14.54671543 | 14.22794188 | 15.47835056 | 13.81171639 | 14.14499779 | 16.70698094 | 16.1660335 | 15.59523834 |
| ENSMUSG00000022650 | Retnlb | 691 | 0 | 0 | 0 | 0 | 0 | 0 | 0 | 0 | 0 | 0 | 0 | 0 | 0 | 0 | 0 | 0 | 0 | 0 |
| ENSMUSG00000022651 | Retnlg | 604 | 0 | 0 | 0 | 0 | 0 | 0 | 0 | 0 | 0 | 0 | 0 | 0 | 0 | 0 | 0 | 0 | 0 | 0 |
| ENSMUSG00000022652 | Morc1 | 3023 | 0.042181782 | 0 | 0 | 0 | 0.04776572 | 0 | 0 | 0 | 0.013624385 | 0.077986608 | 0 | 0.018463666 | 0.359014945 | 0.491634402 | 0.940425854 | 0.215329251 | 0.177495255 | 0.655086947 |
| ENSMUSG00000022656 | Pvrl3 | 12691 | 0.884198761 | 0.955471869 | 0.54125296 | 0.800576226 | 0.853335657 | 1.026898505 | 0.339252524 | 0.225705632 | 0.356986584 | 0.360382808 | 0.436679157 | 0.092359067 | 0.26043956 | 0.644091039 | 0.207777132 | 0.117668717 | 0.24763662 | 0.416111752 |
| ENSMUSG00000022657 | Cd96 | 2169 | 0 | 0 | 0 | 0 | 0 | 0 | 0 | 0 | 0 | 0 | 0 | 0 | 0 | 0 | 0 | 0 | 0 | 0 |
| ENSMUSG00000022658 | Tagln3 | 1236 | 1.100457632 | 3.072548496 | 3.013561357 | 3.94420044 | 3.621576781 | 2.966749421 | 0.306230932 | 0.339900021 | 0.399869085 | 0.228886909 | 0.309223032 | 0.767691152 | 2.634228592 | 0.789098572 | 0.866699396 | 3.252842862 | 2.015541504 | 2.00275874 |
| ENSMUSG00000022659 | Gcet2 | 3403 | 0 | 0 | 0 | 0 | 0 | 0 | 0 | 0.03366947 | 0 | 0 | 0 | 0 | 0 | 0 | 0 | 0 | 0 | 0 |
| ENSMUSG00000022661 | Cd200 | 4583 | 180.7698857 | 168.3453262 | 178.8543469 | 165.4035906 | 163.2158468 | 182.2844284 | 146.1397082 | 127.3191193 | 133.2383336 | 137.2545346 | 112.562514 | 121.6545175 | 113.2814963 | 96.87083381 | 106.9100813 | 97.47687466 | 100.009641 | 116.9438636 |
| ENSMUSG00000022663 | Atg3 | 3142 | 32.52146504 | 27.51335471 | 29.37514192 | 29.74083448 | 33.59430573 | 31.87335282 | 41.6056435 | 35.45742769 | 36.82142673 | 40.33771176 | 36.34059541 | 33.2016147 | 40.35105901 | 43.04429741 | 38.19864173 | 41.02042792 | 39.66808324 | 46.36031655 |
| ENSMUSG00000022664 | Slc35a5 | 4608 | 12.07449553 | 12.60077574 | 11.39003482 | 11.49902226 | 12.847714 | 13.4850538 | 9.887610635 | 10.51782957 | 9.358134115 | 12.3197584 | 11.5497621 | 12.42770612 | 11.23029106 | 12.30647368 | 12.75924919 | 14.4586929 | 13.25783795 | 12.53462718 |
| ENSMUSG00000022665 | Ccdc80 | 4242 | 28.28668362 | 33.51707509 | 34.18761056 | 30.40625095 | 34.56148974 | 31.5749738 | 11.47683796 | 17.59263421 | 18.88443497 | 11.70431177 | 13.50357737 | 19.13153546 | 4.861089444 | 5.550955282 | 7.236011112 | 7.63645777 | 9.821000644 | 10.30934461 |
| ENSMUSG00000022667 | Cd200r1 | 3612 | 0 | 0 | 0 | 0.025227599 | 0 | 0.013718918 | 0 | 0.222048851 | 0 | 0 | 0 | 0 | 0 | 0 | 0 | 0 | 0 | 0 |
| ENSMUSG00000022668 | Gtpbp8 | 3743 | 9.652524612 | 10.24395972 | 10.01589261 | 10.57775782 | 11.21320797 | 7.996219899 | 8.506928378 | 9.530244148 | 8.208693764 | 7.873147084 | 11.48743324 | 9.185799667 | 7.36749713 | 9.157290006 | 7.72735566 | 7.150717225 | 7.362171379 | 5.819825344 |
| ENSMUSG00000022671 | Mzt2 | 1519 | 10.74521896 | 9.276730912 | 11.36889423 | 9.178195896 | 11.37548426 | 10.86310749 | 11.08842245 | 9.629815748 | 9.381523669 | 12.7266546 | 9.561282013 | 14.07333691 | 12.99062996 | 14.43160582 | 13.34507274 | 14.31801514 | 11.75777114 | 13.36297589 |
| ENSMUSG00000022672 | Prkdc | 12588 | 3.835865901 | 4.74499677 | 4.177155028 | 4.62559697 | 4.47747717 | 4.030981823 | 3.623246146 | 3.607464765 | 3.504191165 | 2.813010657 | 3.381592628 | 3.139298468 | 3.013683221 | 3.626830148 | 2.733023448 | 2.886700542 | 3.026401265 | 3.242513282 |
| ENSMUSG00000022673 | Mcm4 | 4237 | 5.989046516 | 7.194075575 | 6.998577793 | 7.215355381 | 7.429378824 | 8.911773185 | 8.039917118 | 7.571776734 | 7.164140221 | 6.521197099 | 6.957080335 | 5.216663296 | 5.530484 | 6.412506755 | 7.312619785 | 8.359408035 | 9.389354776 | 8.906359975 |
| ENSMUSG00000022674 | Ube2v2 | 5308 | 2.754668529 | 2.510391426 | 2.916264076 | 3.004213549 | 2.72941014 | 2.492573449 | 2.781001474 | 2.367238205 | 1.994147429 | 1.945366242 | 3.087191175 | 1.798130803 | 1.3290249 | 1.977461144 | 2.79437832 | 1.998209919 | 2.260009595 | 1.27470236 |
| ENSMUSG00000022676 | Snai2 | 2028 | 0.859325551 | 1.215559257 | 0.310086733 | 0.786309937 | 1.068016058 | 0.781897177 | 0.34994585 | 0.564976367 | 0.467105455 | 0.813744879 | 0.023557672 | 0.385315225 | 3.33258021 | 0.801549832 | 1.137715547 | 0 | 0.737044171 | 1.356240304 |
| ENSMUSG00000022677 | 0610037P05R | 2227 | 81.70842325 | 65.78810452 | 71.74574349 | 78.37645301 | 70.86869228 | 71.06934488 | 82.45195641 | 66.6780694 | 75.82609548 | 82.72016059 | 80.08258644 | 69.1242018 | 91.55312712 | 65.52641337 | 70.17400157 | 84.50755967 | 75.60279403 | 74.81930142 |
| ENSMUSG00000022678 | Nde1 | 3177 | 22.00850317 | 20.38409391 | 21.0121095 | 22.37180647 | 17.42263526 | 20.01138092 | 17.15587225 | 14.81052558 | 16.81426218 | 19.82793187 | 17.89493251 | 18.97416292 | 18.21414509 | 16.09535418 | 22.92865937 | 21.24845478 | 20.91841323 | 16.76072248 |
| ENSMUSG00000022679 | Mpv17l | 3753 | 3.125880251 | 2.369982083 | 3.415663614 | 3.217073474 | 2.449559664 | 3.432909867 | 5.458670391 | 4.894896232 | 2.886238648 | 5.326911319 | 3.640724254 | 3.40575186 | 6.835224349 | 4.219939728 | 4.852408128 | 5.774709243 | 4.963117585 | 3.253935969 |
| ENSMUSG00000022680 | Pdxdc1 | 8955 | 22.89251412 | 22.52851897 | 20.81331239 | 23.33763377 | 22.49918844 | 22.609991 | 26.05235285 | 21.65302368 | 19.74929075 | 21.68774391 | 20.59311443 | 20.51248973 | 21.42398954 | 25.0450584 | 22.32843671 | 20.18220736 | 20.62900626 | 19.43593676 |
| ENSMUSG00000022681 | Ntan1 | 2323 | 28.17820537 | 23.84818902 | 26.21707909 | 28.90959077 | 27.78515266 | 24.95768329 | 23.84982653 | 23.26399027 | 22.25100184 | 24.2756096 | 24.74097075 | 22.29743215 | 25.05883013 | 19.89318422 | 22.43649083 | 28.26878678 | 22.04215784 | 25.71669819 |
| ENSMUSG00000022682 | Rrn3 | 3579 | 14.299031 | 19.8071637 | 20.4766411 | 17.30021214 | 18.854696 | 19.24512425 | 17.13250402 | 18.25850811 | 17.26174173 | 15.9540379 | 21.69161987 | 17.24842772 | 16.85748389 | 17.466818 | 19.35172267 | 16.09082803 | 22.97007736 | 14.46313759 |
| ENSMUSG00000022683 | Pla2g10 | 1773 | 0.023973591 | 0 | 0 | 0.17988004 | 0.081441495 | 0 | 0 | 0.495445716 | 0 | 0.026593741 | 0.458079128 | 0.283328236 | 0 | 0.41912318 | 0 | 0.086386029 | 0.172933094 | 0 |
| ENSMUSG00000022684 | Bfar | 4353 | 23.8841398 | 23.45601213 | 23.04771467 | 23.25675178 | 25.61949624 | 23.92828991 | 23.31396653 | 21.74150544 | 19.89782739 | 21.39274958 | 24.20027187 | 20.41317134 | 23.81032809 | 24.75310883 | 19.46023407 | 25.26318044 | 21.65043972 | 19.39788165 |
| ENSMUSG00000022685 | Parn | 2878 | 36.64188385 | 41.25335476 | 40.72587354 | 44.38956509 | 45.48951785 | 44.06026589 | 49.17033449 | 42.63800866 | 41.08634008 | 42.98938337 | 44.91974901 | 37.60478449 | 43.9667713 | 44.86260005 | 46.87070674 | 37.75842384 | 47.26199712 | 41.91625123 |
| ENSMUSG00000022686 | B3gnt5 | 5433 | 0 | 0 | 0 | 0 | 0 | 0 | 0.008708389 | 0 | 0.174358524 | 0.173571519 | 0.360532541 | 0.071914161 | 0.844444746 | 0.17097032 | 0.015167147 | 0.077525618 | 0 | 0.050624983 |

| ENSMUSG00000022687 | Boc | 5187 | 0.44250617 | 0.526635305 | 0.969895345 | 0.289862052 | 0.862978388 | 0.296150904 | 1.194902822 | 0.927750666 | 0.516121751 | 0.990828351 | 1.326314637 | 0.710205076 | 1.531220104 | 0.062677581 | 0.365388774 | 0.959664346 | 0.472890496 | 1.410489916 |
| --- | --- | --- | --- | --- | --- | --- | --- | --- | --- | --- | --- | --- | --- | --- | --- | --- | --- | --- | --- | --- |
| ENSMUSG00000022696 | Sidt1 | 6769 | 0 | 0.019685563 | 0 | 0.020192515 | 0.063995762 | 0 | 0.027958445 | 0 | 0 | 0.013931365 | 0.077636954 | 0 | 0 | 0.013722585 | 0 | 0 | 0.045296259 | 0 |
| ENSMUSG00000022698 | Naa50 | 4904 | 21.51261152 | 18.78265753 | 19.57036791 | 20.35567866 | 17.67650786 | 18.14777926 | 17.62648132 | 17.18035067 | 19.19909768 | 19.67176567 | 16.60043419 | 16.16195776 | 15.5319087 | 13.95974405 | 14.51800338 | 16.87316529 | 18.74893684 | 19.05800822 |
| ENSMUSG00000022701 | 2610015P09R | 3947 | 3.273770842 | 2.810538073 | 2.684013972 | 2.297098511 | 2.926694117 | 3.452496006 | 4.65095501 | 4.460779708 | 2.744376399 | 3.27319298 | 4.212233481 | 3.139365871 | 3.174640737 | 3.388876911 | 2.661868952 | 3.114082842 | 3.136405852 | 3.135811759 |
| ENSMUSG00000022702 | Hira | 5398 | 1.700836981 | 1.968657513 | 1.855005703 | 1.662750229 | 1.952740141 | 1.533031958 | 2.191213363 | 2.030607538 | 2.136388369 | 1.904196612 | 1.752397514 | 1.230467552 | 2.156786306 | 1.187344213 | 1.923451664 | 1.666967903 | 1.491019331 | 1.049636531 |
| ENSMUSG00000022704 | Qtrtd1 | 6354 | 1.204112635 | 1.620032155 | 1.987014703 | 1.878658108 | 1.742263526 | 1.622122849 | 1.697716524 | 2.133821664 | 1.153792856 | 2.114880458 | 1.89475756 | 1.317650203 | 1.490671721 | 1.520360431 | 1.984210907 | 1.87415469 | 1.839710506 | 1.731479595 |
| ENSMUSG00000022705 | Drd3 | 1448 | 0 | 0 | 0 | 0 | 0.099720836 | 0 | 0 | 0 | 0 | 0 | 0 | 0 | 0 | 0 | 0 | 0.02644379 | 0 | 0 |
| ENSMUSG00000022706 | Mrpl40 | 1646 | 44.62268783 | 37.60349785 | 33.88498208 | 35.20877788 | 35.82114611 | 36.487189 | 45.81798919 | 36.22013375 | 38.65927512 | 39.9606507 | 43.24707663 | 40.149298 | 43.87723384 | 47.71382246 | 41.00130532 | 45.36250573 | 43.12290514 | 44.4150078 |
| ENSMUSG00000022707 | Gbe1 | 5568 | 25.97792996 | 27.47356506 | 29.30386456 | 29.68668622 | 27.61880318 | 27.6331246 | 22.10134301 | 22.71789453 | 18.5442879 | 21.49218478 | 23.0809336 | 23.31667227 | 18.94246393 | 16.16534507 | 21.30374989 | 25.63022518 | 19.98914623 | 20.58889698 |
| ENSMUSG00000022708 | Zbtb20 | 30702 | 28.04328205 | 28.67217868 | 29.28062303 | 27.94179219 | 28.09184875 | 29.14382484 | 18.50005571 | 22.5332935 | 21.58462115 | 18.93891188 | 22.42942647 | 24.62273904 | 15.17781547 | 14.12292358 | 15.50929521 | 16.10595088 | 17.36679834 | 16.46940619 |
| ENSMUSG00000022710 | Usp7 | 9474 | 9.300530917 | 8.512826357 | 8.246068347 | 9.608503967 | 7.834011642 | 8.619685976 | 9.618347014 | 8.542294775 | 9.711914314 | 9.908913889 | 7.286765358 | 9.526485793 | 8.070980766 | 6.49550515 | 7.258327716 | 8.337927282 | 8.798785704 | 7.838536423 |
| ENSMUSG00000022711 | Pmm2 | 4614 | 20.16554623 | 17.37844308 | 18.06406433 | 17.18166822 | 17.49398266 | 17.89225274 | 22.77448199 | 21.80294494 | 20.36117088 | 20.96949352 | 19.11412513 | 20.85526708 | 24.14206095 | 26.71491286 | 21.81521479 | 23.16191705 | 22.75987289 | 22.36607807 |
| ENSMUSG00000022715 | Cldn26 | 1402 | 0 | 0 | 0 | 0 | 0 | 0 | 0 | 0 | 0 | 0 | 0 | 0 | 0 | 0 | 0 | 0 | 0 | 0 |
| ENSMUSG00000022718 | Dgcr8 | 8918 | 2.621422608 | 2.18898363 | 2.310733789 | 2.201929817 | 2.33157558 | 1.766962242 | 1.798497214 | 2.244092718 | 2.276850445 | 2.030261275 | 2.646426271 | 2.722562595 | 2.118650406 | 1.676945071 | 2.444003484 | 1.940721523 | 2.56568729 | 1.967695116 |
| ENSMUSG00000022721 | Trmt2a | 4306 | 13.85909595 | 14.04928377 | 13.52571541 | 14.6756081 | 14.73243739 | 12.91178985 | 13.58069465 | 13.69462827 | 12.95089232 | 12.83340089 | 15.26668436 | 16.17695005 | 13.58747187 | 15.07868885 | 14.08469351 | 13.04512797 | 14.35678516 | 16.44141207 |
| ENSMUSG00000022722 | Arl6 | 2609 | 16.74791911 | 13.41964424 | 16.27901865 | 15.73418971 | 16.65892184 | 16.16304188 | 15.57745926 | 14.63871307 | 15.75474999 | 16.64461391 | 15.08875653 | 14.97545566 | 14.63507243 | 14.41920689 | 14.86035419 | 18.43350054 | 16.46752734 | 15.05423618 |
| ENSMUSG00000022723 | Crybg3 | 9064 | 1.510002944 | 1.859700405 | 1.206136128 | 1.372260051 | 1.683342873 | 1.492486349 | 0.647260833 | 1.175604619 | 1.213239156 | 1.560592561 | 0.996190109 | 1.237748034 | 1.23547498 | 1.152903758 | 1.62733418 | 1.601074603 | 1.704048726 | 0.958896609 |
| ENSMUSG00000022724 | Mina | 5419 | 2.96493016 | 2.545033808 | 2.945795353 | 3.590065682 | 2.327101679 | 3.154768977 | 3.073272372 | 2.896674181 | 3.769793655 | 2.523289158 | 2.47735068 | 3.223897839 | 2.785673867 | 2.691168747 | 2.417806749 | 2.310579188 | 2.588563327 | 2.34491671 |
| ENSMUSG00000022738 | Gsc2 | 1162 | 0 | 0 | 0 | 0 | 0 | 0 | 0 | 0 | 0 | 0 | 0 | 0 | 0 | 0 | 0 | 0 | 0.098949131 | 0 |
| ENSMUSG00000022742 | Cpox | 3179 | 12.92938195 | 12.66916927 | 11.76242182 | 15.76506724 | 12.96034182 | 10.63069025 | 15.28471886 | 13.56376921 | 13.66837815 | 15.66251735 | 12.98444924 | 16.29346803 | 16.38707287 | 10.4166827 | 11.54784092 | 18.63339718 | 15.3353412 | 13.89503388 |
| ENSMUSG00000022744 | Cldn25 | 2716 | 46.80890335 | 43.45640067 | 42.88787227 | 53.46209437 | 50.29396148 | 46.15921059 | 53.28773374 | 48.99202135 | 50.77041781 | 58.67797386 | 56.74595584 | 54.39766527 | 43.64675892 | 37.10738941 | 41.06502643 | 54.23562838 | 44.23893254 | 41.05429283 |
| ENSMUSG00000022747 | St3gal6 | 6595 | 2.642474931 | 2.399336581 | 2.794589093 | 3.530203739 | 2.641964574 | 2.674870835 | 3.386138659 | 2.096383574 | 3.166271947 | 2.566656932 | 2.890403098 | 1.33720617 | 3.261367086 | 2.71129244 | 2.292793178 | 5.428615303 | 3.207902334 | 2.126963186 |
| ENSMUSG00000022748 | 2610528E23R | 1825 | 4.122419812 | 5.238794872 | 4.506027485 | 3.644883547 | 5.221984047 | 5.294675643 | 5.703446227 | 6.236348448 | 5.506580738 | 4.702152325 | 7.722527535 | 7.493061553 | 4.352021195 | 7.329258722 | 5.847234507 | 5.119401757 | 6.678225981 | 7.95748174 |
| ENSMUSG00000022749 | Tbc1d23 | 3630 | 12.07240675 | 12.36156157 | 14.12560391 | 19.22851788 | 14.75780469 | 15.15248878 | 15.58841983 | 12.30994788 | 10.98307087 | 16.92489431 | 11.72658072 | 14.11536591 | 15.1665068 | 15.45577343 | 15.52719792 | 18.23814331 | 17.47383509 | 15.01763768 |
| ENSMUSG00000022750 | Klhl22 | 5629 | 5.217814289 | 4.817320287 | 5.233519273 | 6.151428974 | 5.190278498 | 5.193837771 | 5.47176301 | 5.828259667 | 6.965635638 | 5.846720709 | 6.009001706 | 6.683204225 | 3.558137931 | 5.924117027 | 6.0166422 | 5.135798292 | 5.807839982 | 3.391039267 |
| ENSMUSG00000022751 | Nit2 | 1270 | 15.56291878 | 15.00391771 | 14.20734395 | 11.4081985 | 13.56789659 | 11.58831644 | 15.38593417 | 18.58496432 | 18.64743824 | 13.84819867 | 14.67105024 | 11.73447395 | 14.9549513 | 10.16650102 | 11.71162337 | 12.63288543 | 14.27428223 | 13.51404827 |
| ENSMUSG00000022752 | Tomm70a | 6354 | 17.2255002 | 18.5648345 | 19.87776011 | 17.00830928 | 19.20277408 | 20.05037426 | 16.99950362 | 19.70929926 | 16.71055046 | 17.93567041 | 17.07537468 | 16.47941187 | 14.10703395 | 16.53391968 | 16.17196733 | 16.2587439 | 16.79263623 | 15.90364008 |
| ENSMUSG00000022753 | Tmem30c | 3941 | 0.075497648 | 0.008452904 | 0 | 0.011560783 | 0.024426249 | 0.176031025 | 0 | 0 | 0 | 0 | 0.121225472 | 0.113302538 | 0.025035228 | 0.07070909 | 0.010454594 | 0 | 0.009725018 | 0 |
| ENSMUSG00000022754 | Tmem45a | 3334 | 0 | 0 | 0 | 0 | 0.043310069 | 0 | 0 | 0 | 0 | 0.014142383 | 0 | 0 | 0.029593232 | 0.01393044 | 0.160653937 | 0 | 0.1149559 | 0.065997729 |
| ENSMUSG00000022755 | Gpr128 | 2682 | 0 | 0 | 0 | 0 | 0 | 0 | 0 | 0.042720808 | 0 | 0 | 0 | 0 | 0.423055219 | 0.658044489 | 0.337969511 | 0.128491971 | 0.743090022 | 0.389699115 |
| ENSMUSG00000022756 | Slc7a4 | 4891 | 22.63872082 | 25.30989878 | 24.02357498 | 21.98406556 | 26.02922472 | 27.80059312 | 24.64786463 | 23.91807985 | 21.82691655 | 20.41815365 | 21.80202562 | 23.69112973 | 31.81207669 | 46.4820707 | 28.74252887 | 24.32404768 | 21.94887704 | 32.62756463 |
| ENSMUSG00000022757 | Tfg | 3010 | 56.34407055 | 54.65085437 | 50.60739106 | 57.77624792 | 49.93886964 | 54.01412587 | 52.27972429 | 46.28766909 | 52.59832777 | 57.39540755 | 43.04507887 | 46.82210898 | 48.08632734 | 49.7615222 | 54.28749856 | 57.28325752 | 52.25618687 | 54.20498689 |
| ENSMUSG00000022758 | P2rx6 | 4230 | 0.633055813 | 0.370143269 | 0.560355316 | 0.441608231 | 0.31860375 | 0.281150261 | 0.301995823 | 0.568823015 | 0.759467667 | 0.423576058 | 0.406595391 | 0.197927882 | 0.268235011 | 0.746618899 | 0.087662885 | 0.199147367 | 0.534574827 | 0.221076788 |
| ENSMUSG00000022759 | 4930451C15R | 4142 | 0.133405912 | 0.193024979 | 0.385399928 | 0.406991463 | 0.011620455 | 0.059817399 | 0.479751935 | 0.248960614 | 0.507125134 | 0.295972546 | 0.403699553 | 0.673776709 | 0.488316902 | 0.358814775 | 0.74604459 | 0.45297918 | 0.277592686 | 0.571074744 |
| ENSMUSG00000022760 | Thap7 | 2358 | 16.34953124 | 13.84505358 | 13.78584071 | 13.39007792 | 13.49245188 | 13.44942726 | 17.35600817 | 13.68642071 | 16.13924536 | 13.37736237 | 16.57333165 | 13.75271415 | 19.37292426 | 18.75096319 | 13.38439008 | 13.67289713 | 14.95343221 | 16.2601134 |
| ENSMUSG00000022761 | Lztr1 | 5675 | 15.34680276 | 13.806507 | 16.03358777 | 14.64376121 | 15.31740161 | 13.59534911 | 10.85482079 | 12.2955981 | 11.06048459 | 12.13040119 | 14.40404475 | 13.66131371 | 11.59626038 | 12.65243332 | 13.19175807 | 13.30556437 | 13.58810738 | 13.28942473 |
| ENSMUSG00000022762 | Ncam2 | 8380 | 0 | 0 | 0 | 0 | 0 | 0 | 0 | 0 | 0 | 0 | 0 | 0 | 0 | 0 | 0 | 0 | 0 | 0 |
| ENSMUSG00000022763 | Aifm3 | 4194 | 1.743178416 | 2.319352673 | 1.960777339 | 2.639802998 | 2.444468226 | 2.268508547 | 2.865383988 | 2.121800134 | 0.824908756 | 3.170362022 | 1.594776869 | 1.703482311 | 2.081962174 | 1.904716984 | 3.232072458 | 1.561205023 | 1.635767083 | 2.478951161 |
| ENSMUSG00000022765 | Snap29 | 3424 | 10.55181064 | 8.989811407 | 10.86426543 | 10.76486123 | 10.61320163 | 9.218776662 | 13.26523708 | 9.358477108 | 10.86198123 | 13.1509701 | 10.21357173 | 8.623389459 | 10.97865682 | 12.14002868 | 9.542299597 | 10.48965822 | 13.26421198 | 11.13356044 |
| ENSMUSG00000022766 | Serpind1 | 2250 | 0.264476651 | 0.266503154 | 0.042998694 | 0.080997412 | 0.021391966 | 0.022023437 | 0 | 0.203692813 | 0 | 0 | 0.212333149 | 0 | 0.241178262 | 0.1238509 | 0.164806224 | 0 | 0.289576465 | 0.146690951 |
| ENSMUSG00000022768 | Ccdc116 | 5487 | 0.007746524 | 0.03642744 | 0.061712177 | 0.041517263 | 0.070175941 | 0.009030934 | 0.198321782 | 0.048723677 | 0.247704578 | 0.111711161 | 0.069655489 | 0.111895806 | 0.089906902 | 0.118501408 | 0.022526821 | 0.048848962 | 0.083819129 | 0.180456337 |

| ENSMUSG00000022769 | Sdf2l1 |  | 1105 | 37.61996575 | 47.24100022 | 43.9082814 | 41.19048262 | 40.46566252 | 46.86208741 | 48.93972128 | 44.75942182 | 39.99378408 | 46.68132974 | 42.88937449 | 38.18700535 | 76.34170004 | 107.5569408 | 74.57295184 | 61.47288464 | 50.77800784 | 63.8703022 |
| --- | --- | --- | --- | --- | --- | --- | --- | --- | --- | --- | --- | --- | --- | --- | --- | --- | --- | --- | --- | --- | --- |
| ENSMUSG00000022770 | Dlg1 |  | 8735 | 9.868402631 | 10.6288536 | 10.48323903 | 10.35359737 | 9.714548532 | 10.21688302 | 8.720482321 | 7.975150771 | 10.22708102 | 8.280386811 | 8.729116626 | 8.504939542 | 7.251537697 | 6.827041581 | 7.018650849 | 8.438399456 | 8.226881133 | 7.859343468 |
| ENSMUSG00000022771 | Ppil2 |  | 6433 | 22.92755492 | 24.13152294 | 25.04775837 | 26.99808814 | 23.54596048 | 26.09741355 | 20.15918746 | 22.19232088 | 20.60286145 | 23.53503935 | 24.90120252 | 23.00129357 | 21.94745022 | 22.82135244 | 25.65730342 | 24.66598431 | 25.21325797 | 23.65229119 |
| ENSMUSG00000022772 | Senp5 |  | 4434 | 2.981305761 | 4.673121381 | 4.014763006 | 3.709412947 | 3.549647957 | 3.07329764 | 4.42822773 | 3.324823484 | 2.136422784 | 2.488332107 | 3.167757734 | 3.549845929 | 2.714701808 | 3.687036256 | 4.051393407 | 3.290194276 | 2.748706003 | 4.379389868 |
| ENSMUSG00000022773 | Ypel1 |  | 4831 | 1.31976328 | 0.930913003 | 0.71093369 | 0.952528561 | 1.27528177 | 1.436013807 | 1.596349962 | 1.731346745 | 1.858551115 | 1.268803855 | 1.532833485 | 0.970506242 | 0.71480733 | 0.94214874 | 0.579943242 | 0.578599532 | 0.904408579 | 0.626268033 |
| ENSMUSG00000022774 | Ncbp2 |  | 2426 | 57.08238396 | 47.34660316 | 47.79528123 | 45.07275614 | 45.57255922 | 44.32375592 | 51.1156354 | 45.41841753 | 54.83612775 | 47.53941474 | 43.99392302 | 44.03593495 | 46.62740574 | 48.51167248 | 42.59418886 | 50.85421972 | 44.23480269 | 44.78276413 |
| ENSMUSG00000022779 | Top3b |  | 5444 | 13.42145437 | 14.67382813 | 13.84385751 | 12.71256913 | 12.05066348 | 14.58182939 | 13.94872331 | 12.05263544 | 15.91022091 | 11.80499789 | 15.86648143 | 13.46178637 | 13.97314777 | 14.49458201 | 13.84233022 | 14.06708575 | 15.20661303 | 17.62231486 |
| ENSMUSG00000022780 | Mfi2 |  | 4117 | 0.061945848 | 0.040457729 | 0.011749704 | 0.165998461 | 0 | 0.024072253 | 0 | 0 | 0 | 0.02290537 | 0 | 0.013557363 | 0 | 0 | 0 | 0 | 0.009309278 | 0.106891633 |
| ENSMUSG00000022781 | Pak2 |  | 6779 | 24.58515935 | 24.94904321 | 25.57467662 | 23.10648627 | 24.65172429 | 29.60445066 | 20.1143444 | 21.52724142 | 21.1552512 | 20.14981374 | 21.88246734 | 23.82807605 | 15.66774124 | 17.09367134 | 19.8440891 | 16.22789719 | 19.40908353 | 20.27035678 |
| ENSMUSG00000022783 | Spag6 |  | 4547 | 0.243046971 | 0.358990943 | 0.106385596 | 0.150300344 | 0.010585424 | 0.272447403 | 0.010405252 | 0.109193145 | 0.054347723 | 0.373306646 | 0.084055348 | 0.220954899 | 0.141041329 | 0.030642679 | 0.126857661 | 0.454737805 | 0.623740042 | 0.483915608 |
| ENSMUSG00000022787 | Wdr53 |  | 1534 | 13.60498138 | 9.251168799 | 10.28016356 | 11.37541068 | 11.04461351 | 9.141736377 | 12.76887163 | 11.5025358 | 12.29688671 | 12.57147173 | 13.765666 | 11.13402396 | 11.93099173 | 12.26196571 | 10.74356086 | 13.15459589 | 11.91762948 | 9.036698106 |
| ENSMUSG00000022788 | Fgd4 |  | 17746 | 2.263461701 | 2.021750652 | 2.619574139 | 2.218231844 | 2.270169058 | 2.591284614 | 2.431486712 | 2.85808128 | 2.882545508 | 2.683546168 | 2.140262143 | 2.648303161 | 2.401824441 | 2.242904479 | 2.985754586 | 2.710075673 | 3.019281138 | 4.860491341 |
| ENSMUSG00000022789 | Dnm1l |  | 4164 | 37.71772945 | 40.8970978 | 40.12540198 | 37.70493727 | 39.06962104 | 39.53270436 | 38.05239235 | 35.1472829 | 31.71084763 | 36.54066268 | 32.22859231 | 35.87000794 | 44.33238099 | 49.0986726 | 46.11922479 | 42.4470326 | 45.83692569 | 45.18039025 |
| ENSMUSG00000022790 | Igsf11 |  | 3612 | 0 | 0 | 0.01339245 | 0 | 0 | 0.082313511 | 0 | 0 | 0 | 0 | 0 | 0 | 0.423391317 | 0.038574823 | 0.011406854 | 0.021201887 | 0.010610824 | 0.228443134 |
| ENSMUSG00000022791 | Tnk2 |  | 5300 | 6.840927387 | 6.518013454 | 5.823077805 | 5.209432617 | 5.049311238 | 5.207711812 | 3.374375969 | 5.462234155 | 4.289614472 | 5.008650176 | 3.957208825 | 3.559564897 | 5.324123902 | 5.135138719 | 5.301785117 | 6.979005047 | 4.403908455 | 5.677354977 |
| ENSMUSG00000022792 | Yars2 |  | 3821 | 4.438514848 | 3.478629886 | 4.760127562 | 4.113729468 | 4.3080654 | 4.266644687 | 4.45761958 | 3.738277545 | 5.475726252 | 4.615117252 | 3.600939032 | 4.323851381 | 4.854436246 | 3.938205788 | 2.889823863 | 4.549577535 | 4.132539734 | 3.555940686 |
| ENSMUSG00000022793 | B4galt4 |  | 3068 | 11.55453612 | 10.91247024 | 10.86354707 | 11.24175703 | 10.04055774 | 10.62767233 | 12.6146582 | 10.10828903 | 11.39744194 | 10.81945733 | 8.96948373 | 9.260160449 | 15.11473345 | 15.2593351 | 11.21359165 | 15.5758403 | 15.31552803 | 11.08071315 |
| ENSMUSG00000022797 | Tfrc |  | 6219 | 9.179040265 | 9.036638133 | 9.450689722 | 8.454324677 | 9.945251956 | 10.3742819 | 12.07352009 | 11.44113936 | 9.973772751 | 7.877404838 | 9.364475701 | 8.633971283 | 4.807065745 | 5.899795632 | 5.233836494 | 6.304804949 | 6.581843559 | 5.271816628 |
| ENSMUSG00000022798 | 4930435E12R |  | 1526 | 0 | 0 | 0 | 0 | 0 | 0 | 0 | 0 | 0 | 0 | 0 | 0 | 0 | 0 | 0 | 0 | 0 | 0 |
| ENSMUSG00000022799 | Arhgap31 |  | 11129 | 1.520088064 | 1.358976905 | 1.621289139 | 1.383739149 | 0.977429666 | 1.286794861 | 0.663202257 | 0.658903878 | 0.455201855 | 0.813454491 | 0.377769462 | 0.727223567 | 3.954000378 | 3.359465392 | 4.516659016 | 5.618524745 | 3.619457098 | 3.647831859 |
| ENSMUSG00000022800 | Fyttd1 |  | 5726 | 32.66945157 | 42.27817015 | 39.66359152 | 41.52691065 | 38.03649225 | 42.37857777 | 32.45620028 | 32.66297495 | 33.82099146 | 32.04864427 | 33.95810007 | 34.63378435 | 31.69614454 | 33.68534665 | 40.54676344 | 38.47116039 | 45.64216182 | 42.83716502 |
| ENSMUSG00000022801 | Lrch3 |  | 6868 | 7.513291162 | 9.875517273 | 7.043321251 | 9.413384087 | 9.082552864 | 9.393951532 | 7.233301234 | 8.647231471 | 7.364158613 | 8.066697194 | 7.742214442 | 9.28906557 | 6.328104124 | 8.047242864 | 7.072893768 | 9.734333215 | 9.458805074 | 9.250949078 |
| ENSMUSG00000022802 | Lmln |  | 3685 | 1.684058535 | 1.943628835 | 1.64089316 | 1.285847656 | 1.07104959 | 1.559868954 | 1.091337234 | 1.927757625 | 2.514780474 | 1.688980415 | 1.815059481 | 1.590405583 | 2.235666265 | 1.146923189 | 1.229897191 | 1.735286685 | 1.362481654 | 1.253938932 |
| ENSMUSG00000022803 | Popdc2 |  | 3256 | 0.378578042 | 0.092081096 | 0.653696356 | 0.307844894 | 0.428693423 | 0.243502376 | 0.188901974 | 0.129028387 | 0.227689583 | 0.217217613 | 0.24943928 | 0.188566428 | 0.212115123 | 0.071320773 | 0.303696972 | 0.305760379 | 0.364900247 | 0.405472531 |
| ENSMUSG00000022805 | 4932425I24Ri | k | 4516 | 0.009412129 | 0.243429032 | 0.053557939 | 0.100888052 | 0.021316175 | 0.076808932 | 0.010476678 | 0.22834253 | 0.136801979 | 0 | 0 | 0 | 0.098314273 | 0.143980785 | 0.045617312 | 0.084788767 | 0.152762034 | 0.024361872 |
| ENSMUSG00000022807 | Osbpl11 |  | 4507 | 15.56102517 | 13.68142161 | 13.10496573 | 14.0008978 | 12.76184797 | 14.24902212 | 9.290375161 | 9.05872602 | 10.12528499 | 10.16873387 | 11.78738714 | 10.41512586 | 7.95746702 | 9.480488218 | 9.050264107 | 9.838145856 | 11.53955733 | 9.471281743 |
| ENSMUSG00000022808 | Snx4 |  | 2428 | 64.14290155 | 63.34663617 | 60.02860254 | 60.66674478 | 57.19135082 | 66.43086812 | 49.26130656 | 48.3539724 | 49.58327248 | 51.09287491 | 54.0518167 | 50.5743236 | 47.78775512 | 51.72356355 | 56.37214525 | 60.30612961 | 58.84696658 | 57.95440486 |
| ENSMUSG00000022809 | Nr1i2 |  | 2544 | 0 | 0 | 0 | 0 | 0 | 0 | 0 | 0 | 0 | 0.037068163 | 0.056338394 | 0.065820357 | 0 | 0 | 0 | 0.075256697 | 0 | 0 |
| ENSMUSG00000022811 | Zfp148 |  | 2249 | 50.91549321 | 44.57025287 | 49.47048458 | 53.5428369 | 49.05218721 | 49.86119879 | 40.85425633 | 41.74162966 | 40.93013016 | 44.94935873 | 40.29750833 | 46.60818779 | 39.85597763 | 29.63417759 | 40.26723871 | 35.77081643 | 45.99496458 | 46.17927679 |
| ENSMUSG00000022812 | Gsk3b |  | 10143 | 12.86930845 | 11.43929908 | 11.92288533 | 11.11288807 | 11.39829248 | 9.941813352 | 9.011938843 | 9.801323421 | 8.21050329 | 9.896859631 | 8.129703079 | 9.608020002 | 10.58814788 | 8.722861725 | 8.839060004 | 10.60796676 | 9.272674021 | 8.118764938 |
| ENSMUSG00000022814 | Umps |  | 5619 | 5.272487576 | 4.150031314 | 4.605773045 | 5.42451302 | 4.779785265 | 4.832692294 | 3.923777964 | 4.350086766 | 4.507867449 | 4.472561815 | 5.101437104 | 4.062752447 | 3.959547018 | 5.471789616 | 4.634166813 | 5.424332353 | 4.433545646 | 3.857196663 |
| ENSMUSG00000022816 | Fstl1 |  | 3645 | 0.338175612 | 0.20106548 | 0.331780044 | 0.162494808 | 0.633836032 | 0.299083714 | 0.155761906 | 0.29338471 | 0.54237387 | 0.142292932 | 0.078641907 | 0.015312939 | 0.121807203 | 0.012741862 | 0 | 0.294139097 | 0.189265664 | 0 |
| ENSMUSG00000022817 | Itgb5 |  | 3697 | 76.96230682 | 86.61172281 | 77.2641863 | 91.25765034 | 89.46783323 | 95.08441764 | 50.75522982 | 60.18635013 | 60.4150595 | 67.27615896 | 71.91442898 | 69.62992584 | 45.72909941 | 54.23292541 | 58.93260145 | 56.28108213 | 63.0201674 | 60.35080792 |
| ENSMUSG00000022818 | Cyp2ab1 |  | 2712 | 0 | 0 | 0 | 0 | 0 | 0 | 0 | 0 | 0 | 0 | 0 | 0 | 0 | 0 | 0 | 0 | 0 | 0 |
| ENSMUSG00000022820 | Ndufb4 |  | 1285 | 11.54420734 | 10.11052821 | 10.61582534 | 10.60136362 | 11.64904923 | 12.37854278 | 14.72768216 | 11.948129 | 13.71815467 | 12.54905877 | 13.34724521 | 11.51062302 | 16.16244138 | 14.52958998 | 13.75522761 | 11.94905336 | 11.57245385 | 13.5275313 |
| ENSMUSG00000022821 | Hgd |  | 2686 | 0.047474136 | 0.161231431 | 0.090047525 | 0 | 0 | 0.092242616 | 0.757425612 | 0.042657188 | 0 | 0 | 0.355733123 | 0 | 0 | 0 | 0 | 0.256601241 | 0.099882382 | 0.22527929 |
| ENSMUSG00000022822 | Abcc5 |  | 8382 | 6.166343838 | 7.42008751 | 6.232809924 | 7.365212965 | 6.936693366 | 7.035045688 | 6.683155792 | 7.568310713 | 6.353395953 | 8.032832767 | 9.165131619 | 7.191710283 | 7.645211231 | 6.831968474 | 6.99964395 | 6.62845041 | 9.126615201 | 6.451199226 |
| ENSMUSG00000022824 | Muc13 |  | 3025 | 0 | 0.264300648 | 0.063964999 | 0.150615023 | 0.302316215 | 0 | 0 | 0 | 0.013615377 | 0 | 0 | 0 | 0.17938879 | 0.122827339 | 0 | 0 | 0 | 0 |
| ENSMUSG00000022827 | Rabl3 |  | 3087 | 4.860488221 | 6.097112159 | 5.108445382 | 5.121374274 | 6.408234735 | 6.533191631 | 7.709192582 | 6.829350867 | 7.511502551 | 7.194033442 | 8.496421187 | 7.413158944 | 7.718599302 | 8.304870826 | 7.834568613 | 7.231429904 | 8.88941645 | 8.072279023 |
| ENSMUSG00000022828 | Gtf2e1 |  | 8220 | 2.347609479 | 2.334334193 | 2.818846842 | 2.455418813 | 2.45929537 | 2.881533656 | 2.532552157 | 2.239505834 | 2.775830868 | 2.340326877 | 2.6851619 | 3.143874912 | 2.676646606 | 2.791165349 | 2.280621403 | 2.077568236 | 2.210056537 | 2.308776377 |
| ENSMUSG00000022829 | Stxbp5l |  | 8920 | 0 | 0 | 0 | 0 | 0.010791911 | 0 | 0 | 0.008563319 | 0 | 0 | 0 | 0 | 0 | 0.046860626 | 0.023095043 | 0 | 0 | 0 |

| ENSMUSG00000022831 | Hcls1 | 2511 | 0 | 0.013266784 | 0 | 0.018144582 | 0 | 0 | 0.602949313 | 0.243360589 | 1.049755878 | 0.112665958 | 0.951313391 | 0.04445692 | 0.550097046 | 0.25894752 | 0.328168506 | 0.030498293 | 0 | 0.065721752 |
| --- | --- | --- | --- | --- | --- | --- | --- | --- | --- | --- | --- | --- | --- | --- | --- | --- | --- | --- | --- | --- |
| ENSMUSG00000022832 | Ropn1 | 969 | 0 | 0 | 0 | 0 | 0 | 0 | 0 | 0 | 0 | 0 | 0 | 0 | 0 | 0 | 0 | 0 | 0 | 0 |
| ENSMUSG00000022833 | Ccdc14 | 4094 | 1.962256539 | 2.815403371 | 2.504931225 | 2.38154946 | 2.974444721 | 1.767146825 | 0.92452719 | 1.977720887 | 1.287707381 | 1.980928422 | 1.785434452 | 2.222265022 | 2.289463674 | 1.100409496 | 1.580030355 | 1.07557886 | 1.94720805 | 0.671825925 |
| ENSMUSG00000022836 | Mylk | 10594 | 0.653987511 | 1.018819872 | 0.91322504 | 0.761214352 | 0.79962418 | 0.696937635 | 0.549316548 | 0.966166117 | 1.119663634 | 0.485126171 | 0.608799263 | 0.869320778 | 0.512224929 | 0.758431859 | 0.451140314 | 0.249391345 | 0.372626825 | 0.379050858 |
| ENSMUSG00000022837 | Iqcb1 | 5363 | 7.624460067 | 9.211825867 | 8.604950207 | 9.574360799 | 8.481198558 | 8.879391566 | 5.487317974 | 7.164191091 | 6.696744685 | 7.499449912 | 6.191235529 | 4.412798981 | 7.699200962 | 6.728893511 | 8.143510954 | 7.796633094 | 6.967767943 | 8.68780783 |
| ENSMUSG00000022838 | Eaf2 | 4543 | 3.648914517 | 3.703062201 | 2.960123583 | 2.457067106 | 4.004813371 | 3.370414853 | 1.374702535 | 1.017231057 | 1.160438921 | 1.131284758 | 1.020068451 | 1.118033303 | 1.72656281 | 0.899643339 | 0.906923969 | 1.407556997 | 0.944870186 | 1.404590883 |
| ENSMUSG00000022840 | Adcy5 | 5620 | 32.28729474 | 36.98798219 | 35.98749652 | 41.41028727 | 38.27430015 | 39.44820819 | 24.10252665 | 24.72992035 | 25.92845067 | 30.35431395 | 25.37513364 | 28.41434353 | 18.50385794 | 19.18091225 | 24.72093353 | 21.44137749 | 25.30763128 | 21.66105928 |
| ENSMUSG00000022841 | Ap2m1 | 4126 | 63.53112471 | 66.892227 | 58.83140459 | 64.33316634 | 59.87909941 | 62.18711936 | 53.57363935 | 57.41819489 | 54.94197352 | 58.54412331 | 57.43184538 | 52.89365989 | 53.8394628 | 50.90163916 | 52.72520967 | 54.63325394 | 54.78635461 | 52.7426142 |
| ENSMUSG00000022842 | Ece2 | 6396 | 0.591457265 | 0.609382211 | 0.423533099 | 0.491512204 | 0.353689871 | 0.449352493 | 0.79890077 | 0.453818415 | 0.547350507 | 0.943603818 | 0.478048365 | 0.296706149 | 0.694163939 | 0.501038466 | 0.463807383 | 0.407092136 | 0.503347239 | 0.490231251 |
| ENSMUSG00000022843 | Clcn2 | 6186 | 5.435110611 | 7.011540296 | 6.685963216 | 7.446203657 | 6.738158082 | 7.289522722 | 7.503029103 | 8.217602263 | 9.108172254 | 8.925553429 | 9.020554721 | 8.815373803 | 7.44045891 | 7.643078073 | 8.205676828 | 9.309581884 | 9.572280749 | 8.198900161 |
| ENSMUSG00000022844 | Pdia5 | 1791 | 23.51905608 | 21.39018892 | 22.55270678 | 22.58973052 | 23.89127869 | 25.3435533 | 32.14937479 | 38.32035015 | 37.57608417 | 32.67114614 | 29.92937097 | 36.05735433 | 19.0882293 | 17.03727829 | 20.750309 | 10.06972422 | 19.55903706 | 21.25421656 |
| ENSMUSG00000022847 | Thpo | 1830 | 0 | 0.145630139 | 0 | 0.22407071 | 0.078904793 | 0.027077996 | 1.085864762 | 0.146091157 | 0.157544049 | 0 | 0 | 0.030500362 | 0.350445314 | 0.710619916 | 0 | 0 | 0 | 0 |
| ENSMUSG00000022848 | Dirc2 | 5858 | 15.8686958 | 17.93596251 | 17.22553505 | 16.85400872 | 16.966955 | 17.6539015 | 12.0745058 | 12.21139804 | 10.69387 | 15.84836714 | 12.41268126 | 12.88200338 | 13.28037445 | 13.3671443 | 12.42798724 | 12.97488148 | 13.36644325 | 11.94461996 |
| ENSMUSG00000022849 | Hspbap1 | 4582 | 2.513946465 | 2.820908546 | 3.431121206 | 2.495814519 | 1.995867634 | 2.746921503 | 2.075479805 | 3.050724417 | 3.3528089 | 3.076835503 | 3.524211254 | 2.594660875 | 1.335040974 | 2.655685486 | 2.499788856 | 2.064116113 | 3.078148682 | 3.373539718 |
| ENSMUSG00000022853 | Ehhadh | 3011 | 6.493650273 | 6.605047442 | 6.056732296 | 7.414450921 | 6.474071433 | 7.86655817 | 7.558086537 | 7.978419499 | 9.164717888 | 8.393482878 | 6.965528652 | 7.748570894 | 9.093063463 | 7.033711009 | 8.552299047 | 6.027813985 | 7.79000123 | 7.636601332 |
| ENSMUSG00000022855 | Senp2 | 4500 | 13.19549576 | 15.24249982 | 13.5445885 | 14.54916016 | 14.40748915 | 15.74675756 | 16.24401977 | 15.52308978 | 15.54104528 | 14.31285792 | 14.22632097 | 13.66863559 | 14.52550898 | 16.06965424 | 13.88034638 | 13.04433359 | 16.49734158 | 17.39510197 |
| ENSMUSG00000022856 | Tmem41a | 1334 | 4.588264879 | 5.718630267 | 9.428124357 | 7.923659884 | 6.386319707 | 4.643247149 | 8.086424884 | 5.582847428 | 8.830092585 | 5.231112505 | 8.237061807 | 5.104580836 | 10.72432984 | 9.609121529 | 6.671316396 | 4.477762185 | 4.108441122 | 7.46375436 |
| ENSMUSG00000022857 | Tmprss15 | 4645 | 0 | 0 | 0 | 0 | 0 | 0 | 0 | 0 | 0 | 0 | 0 | 0 | 0 | 0 | 0 | 0 | 0 | 0 |
| ENSMUSG00000022858 | Tra2b | 5430 | 23.07647494 | 16.09816472 | 15.465276 | 16.6218101 | 14.63458674 | 15.50462143 | 18.11474392 | 18.79375676 | 16.58079622 | 18.19166175 | 14.75480762 | 15.70650689 | 19.06047191 | 17.58545924 | 16.85242277 | 21.13387668 | 22.03585616 | 18.78211489 |
| ENSMUSG00000022860 | Chodl | 3813 | 0 | 0 | 0 | 0 | 0 | 0 | 0 | 0 | 0 | 0 | 0 | 0 | 0 | 0 | 0 | 0 | 0 | 0 |
| ENSMUSG00000022861 | Dgkg | 5679 | 0.321838804 | 0.26983503 | 0.076661696 | 0.160454461 | 0.093229646 | 0.17451218 | 0.449882842 | 0.309359132 | 0.203067871 | 0.57288229 | 0.344915178 | 0.186740199 | 0.825238975 | 1.169484856 | 0.602171005 | 0.350609541 | 0.391428988 | 0.610243656 |
| ENSMUSG00000022863 | Btg3 | 1433 | 20.85215544 | 30.05831977 | 24.27115724 | 12.20896094 | 26.26599045 | 27.42171509 | 21.42772409 | 32.14238472 | 26.01102356 | 11.64783598 | 27.83816492 | 29.75796665 | 29.64046111 | 38.24425898 | 29.75827659 | 12.02426611 | 33.05743403 | 27.86923341 |
| ENSMUSG00000022864 | D16Ertd472e | 3043 | 1.857768127 | 1.390317964 | 1.017386113 | 1.676909946 | 1.312832621 | 1.416722912 | 0.777401888 | 1.305294068 | 1.976086527 | 1.472006838 | 0.926297256 | 0.788719517 | 1.426621334 | 2.625166951 | 1.367517958 | 1.27090087 | 1.032782237 | 2.801975531 |
| ENSMUSG00000022865 | Cxadr | 6419 | 83.03044122 | 80.71591273 | 78.29895317 | 84.29396519 | 81.67955203 | 81.57403579 | 45.24150542 | 61.53385662 | 51.50399782 | 54.01873684 | 48.41503426 | 56.75476394 | 29.0734761 | 27.58137734 | 31.31677694 | 33.09491975 | 37.8128117 | 29.77981708 |
| ENSMUSG00000022867 | Usp25 | 4385 | 33.75211469 | 35.61478862 | 39.89023621 | 40.17891869 | 37.3969131 | 42.47861471 | 32.04530363 | 36.49399454 | 37.57976045 | 37.40873355 | 35.0821816 | 36.02242306 | 31.62417776 | 38.05555439 | 41.13578374 | 42.32487436 | 51.68150367 | 39.89257911 |
| ENSMUSG00000022868 | Ahsg | 1467 | 0 | 0 | 0 | 0 | 0 | 0 | 0 | 0 | 0 | 0 | 0 | 0 | 0 | 0 | 0 | 0 | 0 | 0 |
| ENSMUSG00000022871 | Fetub | 1903 | 4.846885548 | 4.25382727 | 4.98224485 | 4.764397174 | 5.513798928 | 4.452715419 | 4.773533554 | 4.435376212 | 4.80473279 | 8.027760302 | 6.627739905 | 2.522410395 | 34.55567301 | 29.14043108 | 28.0811445 | 38.77351574 | 33.5329923 | 37.0870646 |
| ENSMUSG00000022875 | Kng1 | 3146 | 0 | 0 | 0 | 0 | 0 | 0 | 0 | 0 | 0 | 0 | 0 | 0 | 0 | 0 | 0 | 0 | 0 | 0 |
| ENSMUSG00000022876 | Samsn1 | 5447 | 0 | 0 | 0 | 0 | 0.00883641 | 0.009097252 | 0.086860068 | 0.084139679 | 0.015122642 | 0.00865627 | 0.078937879 | 0.040988186 | 0 | 0.196110521 | 0.052948576 | 0 | 0 | 0 |
| ENSMUSG00000022877 | Hrg | 1727 | 0 | 0 | 0 | 0 | 0 | 0 | 0 | 0 | 0 | 0 | 0 | 0 | 0 | 0 | 0 | 0 | 0 | 0 |
| ENSMUSG00000022878 | Adipoq | 1377 | 0 | 0 | 0 | 0 | 0 | 0.071972017 | 0 | 0 | 0 | 0 | 0 | 0 | 0.071651296 | 0 | 0 | 0 | 0 | 0 |
| ENSMUSG00000022881 | Rfc4 | 1588 | 7.2001841 | 4.971760661 | 5.757303046 | 6.828418484 | 5.122352078 | 4.399833397 | 7.001561428 | 6.084809703 | 4.616624563 | 6.591597055 | 8.062776368 | 6.643048003 | 6.927592917 | 6.258837973 | 6.901520067 | 6.196905606 | 5.357958383 | 7.447706515 |
| ENSMUSG00000022883 | Robo1 | 10506 | 0.760610422 | 1.173212532 | 0.98533557 | 1.309674033 | 1.067460329 | 1.414983826 | 0.085564525 | 0.105423536 | 0.109767984 | 0.049367765 | 0.004547398 | 0 | 0.042260352 | 0 | 0.070590901 | 0.11662854 | 0.03283235 | 0 |
| ENSMUSG00000022884 | Eif4a2 | 4742 | 47.20207865 | 38.88377678 | 39.75361615 | 39.68096017 | 38.77350241 | 37.25337309 | 44.53897063 | 43.74971106 | 50.38443229 | 39.74301158 | 47.27121577 | 46.78769692 | 47.11603824 | 35.88594184 | 36.76165816 | 42.87708252 | 43.55554914 | 47.38764255 |
| ENSMUSG00000022885 | St6gal1 | 3496 | 32.60837589 | 31.29277592 | 30.91146076 | 31.10818445 | 29.9860783 | 33.84780543 | 34.80784045 | 28.92834141 | 29.1462929 | 29.36129319 | 25.96465135 | 27.36500162 | 38.67814224 | 27.92490609 | 37.37131972 | 39.67064646 | 41.09991049 | 34.88420755 |
| ENSMUSG00000022886 | Prl2a1 | 879 | 0 | 0 | 0 | 0 | 0 | 0 | 0 | 0 | 0 | 0 | 0 | 0 | 0 | 0 | 0 | 0 | 0 | 0 |
| ENSMUSG00000022887 | Masp1 | 6000 | 0 | 0 | 0 | 0.14427664 | 0 | 0 | 0 | 0 | 0 | 0 | 0 | 0 | 0 | 0 | 0 | 0 | 0 | 0 |
| ENSMUSG00000022889 | Mrpl39 | 2523 | 27.460736 | 25.40388762 | 23.94710242 | 22.53673537 | 26.89893475 | 23.7648861 | 23.66571574 | 22.79736743 | 28.12697846 | 23.13617544 | 21.85188351 | 23.27311813 | 24.83215812 | 23.80190448 | 23.31978669 | 22.68904402 | 20.87210937 | 22.30452484 |
| ENSMUSG00000022890 | Atp5j | 2589 | 157.346314 | 130.4594185 | 145.6998029 | 124.3117872 | 149.062095 | 136.0834051 | 194.860987 | 170.324248 | 177.7908459 | 171.3925332 | 169.6573844 | 159.7720647 | 196.6607641 | 186.3862758 | 171.7288488 | 163.4708705 | 168.1975224 | 176.6283137 |
| ENSMUSG00000022892 | App | 4328 | 696.6802627 | 790.734808 | 733.6726616 | 742.2934194 | 749.0363805 | 798.3165207 | 578.6948439 | 649.3149633 | 647.8787384 | 655.4361738 | 656.1329787 | 695.1165003 | 580.8810463 | 513.2457975 | 580.5440118 | 619.0816204 | 696.576904 | 622.3095849 |
| ENSMUSG00000022893 | Adamts1 | 5916 | 0.431086978 | 1.587937148 | 0.523310673 | 0.808639225 | 1.667857396 | 1.139143301 | 0.495839434 | 2.040026903 | 0.118352057 | 0.462261797 | 0.161511016 | 0.358518455 | 0.875566483 | 0.314023579 | 1.072521908 | 0.310674299 | 0.239701316 | 0.622990222 |

| ENSMUSG00000022894 | Adamts5 | 8083 | 0 | 0 | 0 | 0 | 0.035728262 | 0 | 0 | 0 | 0 | 0 | 0 | 0 | 0 | 0 | 0 | 0 | 0 | 0 |
| --- | --- | --- | --- | --- | --- | --- | --- | --- | --- | --- | --- | --- | --- | --- | --- | --- | --- | --- | --- | --- |
| ENSMUSG00000022895 | Ets2 | 4889 | 85.15815079 | 92.52521796 | 85.44770058 | 94.45832387 | 93.16269046 | 95.62897143 | 44.9513998 | 65.62781195 | 54.44640036 | 63.74844519 | 53.72606294 | 59.68588341 | 49.00902479 | 55.01282677 | 59.2951825 | 67.59008834 | 73.88532385 | 63.83036663 |
| ENSMUSG00000022897 | Dyrk1a | 8298 | 8.036951216 | 9.94007304 | 8.557766035 | 8.16453036 | 7.702963923 | 7.745227218 | 7.377995485 | 8.542431774 | 8.576801547 | 7.307279391 | 8.49217447 | 9.356421627 | 6.022322512 | 6.738814319 | 7.035744119 | 7.23543895 | 7.588588312 | 6.86785184 |
| ENSMUSG00000022898 | Dscr3 | 3662 | 27.56684682 | 26.12633321 | 25.37562857 | 27.14751468 | 27.28614788 | 29.24179608 | 23.29458222 | 21.8808466 | 25.11455207 | 28.36510084 | 27.60562866 | 23.06092231 | 24.38306124 | 26.5956448 | 26.65387381 | 28.82774566 | 27.78708855 | 29.74277207 |
| ENSMUSG00000022899 | Slc15a2 | 7306 | 4.945168303 | 7.496085149 | 8.190262394 | 5.531432566 | 7.062335364 | 7.128377086 | 0.466262371 | 2.770595849 | 5.310388427 | 0.838980485 | 1.667480757 | 4.446306546 | 0.553684262 | 1.754526159 | 4.2239208 | 0.398314558 | 1.757365106 | 4.75852114 |
| ENSMUSG00000022900 | Ildr1 | 4502 | 38.37928489 | 44.78223808 | 43.28044875 | 44.68058879 | 39.35442271 | 44.79778447 | 31.40166251 | 31.54987662 | 30.07109669 | 36.74026367 | 28.60979299 | 30.52380304 | 20.89648295 | 21.66427888 | 27.03451713 | 32.04775848 | 28.74046609 | 23.96109691 |
| ENSMUSG00000022901 | Cd86 | 4140 | 0.061601704 | 0 | 0 | 0 | 0.046504274 | 0 | 0.262848216 | 0.295206975 | 0.198968675 | 0.022778118 | 0.946267294 | 0.026964088 | 0.035747766 | 0.863815152 | 0.169185133 | 0.203476658 | 0.037030239 | 0.013287224 |
| ENSMUSG00000022902 | Stfa2 | 778 | 0 | 0 | 0 | 0 | 0 | 0 | 0 | 0 | 0 | 0 | 0 | 0 | 0 | 0 | 0 | 0 | 0 | 0 |
| ENSMUSG00000022905 | Kpna1 | 8145 | 11.66870149 | 13.98773459 | 14.15868586 | 15.79113912 | 14.18842833 | 14.0597136 | 12.23333356 | 13.91244419 | 11.83259016 | 13.7139369 | 13.32067903 | 12.66388514 | 11.63494329 | 14.27250469 | 13.09142132 | 13.43109458 | 14.86937977 | 12.04864904 |
| ENSMUSG00000022906 | Parp9 | 4908 | 5.031684448 | 4.513666393 | 5.203998376 | 5.458413625 | 5.717382131 | 5.502493847 | 3.63424612 | 4.824631111 | 4.967892692 | 4.217432504 | 5.256413525 | 4.765029059 | 4.492943566 | 4.286709777 | 3.685306242 | 4.150489637 | 4.68536637 | 5.278991293 |
| ENSMUSG00000022911 | Arl13b | 5806 | 3.609206301 | 3.718008173 | 5.023981882 | 4.449382042 | 4.062115501 | 3.994260991 | 4.685633893 | 5.104599426 | 5.313244972 | 3.906215685 | 5.406157031 | 4.777882243 | 4.996067406 | 5.439541754 | 3.853331872 | 5.394715269 | 6.271095773 | 5.0972958 |
| ENSMUSG00000022912 | Pros1 | 4072 | 74.2066052 | 95.52914189 | 79.46231448 | 88.30249555 | 92.74142267 | 95.9293219 | 66.04255084 | 70.56027586 | 65.39067397 | 74.11877494 | 75.05314572 | 64.64309056 | 39.49460961 | 55.47741676 | 48.30457461 | 51.24847995 | 60.89664562 | 51.68586501 |
| ENSMUSG00000022913 | Psmg1 | 1411 | 21.53876744 | 24.08161028 | 21.15270604 | 20.66553393 | 24.04890587 | 23.59988445 | 22.9689476 | 22.27664577 | 23.35167442 | 21.85440108 | 24.17528019 | 20.25345092 | 24.26389128 | 24.91720351 | 23.44780254 | 27.30003618 | 22.13744293 | 28.10883483 |
| ENSMUSG00000022914 | Brwd1 | 21764 | 4.191146285 | 4.146509393 | 3.73625733 | 3.889561679 | 3.671153891 | 3.501750792 | 5.019526541 | 4.353765409 | 4.64587834 | 4.135760546 | 3.435389176 | 4.818858206 | 4.236415796 | 4.063110751 | 3.964163666 | 4.808317866 | 4.367267796 | 3.816566401 |
| ENSMUSG00000022915 | 1700093J21R | 646 | 0.460582403 | 0.206271791 | 0 | 0.28211173 | 0.596060974 | 0 | 0.805633852 | 0.413849562 | 0.127512433 | 0.656898343 | 0.073955044 | 0.345607818 | 0.305460788 | 0.071894872 | 0.829133478 | 0.177820158 | 0.296643165 | 0 |
| ENSMUSG00000022931 | Krtap15 | 377 | 0 | 0 | 0 | 0 | 0 | 0 | 0 | 0 | 0 | 0 | 0 | 0 | 0 | 0 | 0 | 0 | 0 | 0 |
| ENSMUSG00000022935 | Grik1 | 3312 | 0 | 0 | 0 | 0 | 0 | 0 | 0 | 0 | 0 | 0 | 0 | 0 | 0.014894903 | 0.014022973 | 0.024880167 | 0.034683521 | 0 | 0 |
| ENSMUSG00000022938 | Fam3b | 1503 | 0 | 0 | 0 | 0 | 0.032023901 | 0 | 0 | 0 | 0 | 0.1568553 | 0.127145598 | 0 | 0.722090606 | 1.143334154 | 0.575670442 | 0 | 0 | 0.036599539 |
| ENSMUSG00000022940 | Pigp | 1882 | 47.90301719 | 44.26968568 | 49.47877042 | 38.66152381 | 50.8684358 | 46.97241058 | 60.78749078 | 57.75535454 | 67.51349688 | 57.97381899 | 55.61898726 | 61.12437863 | 64.19440245 | 63.17580431 | 59.24091936 | 60.9353715 | 66.12406272 | 58.37044959 |
| ENSMUSG00000022941 | Ripply3 | 1527 | 3.869167955 | 2.770620541 | 3.611383406 | 3.878805346 | 4.381360439 | 4.51069415 | 7.529129653 | 8.103692455 | 10.46520505 | 12.19680929 | 10.9190966 | 7.054631879 | 19.25463176 | 11.0407359 | 12.76249898 | 18.88200876 | 13.85469279 | 13.76128276 |
| ENSMUSG00000022945 | Chaf1b | 3581 | 8.427443446 | 8.391016639 | 7.294528453 | 7.315722003 | 8.198959351 | 7.513860467 | 7.596981397 | 7.625682133 | 8.856078508 | 8.729661061 | 7.737915643 | 9.071408999 | 8.499802558 | 10.37566878 | 8.502638873 | 9.783832944 | 9.803654835 | 10.15387309 |
| ENSMUSG00000022946 | Dopey2 | 7276 | 7.074459616 | 8.126771198 | 7.712107643 | 7.332597982 | 7.316369925 | 8.751410479 | 6.457049227 | 6.955048541 | 5.654938049 | 6.959848172 | 5.48926131 | 7.026820633 | 5.390169631 | 6.69596587 | 5.096399162 | 5.652022038 | 4.477371134 | 5.798788463 |
| ENSMUSG00000022947 | Cbr3 | 1167 | 11.87376811 | 11.87503341 | 9.409418501 | 12.72742113 | 11.71333875 | 12.52618372 | 14.51408659 | 8.934469457 | 8.717283113 | 11.67656662 | 8.146715285 | 12.19622447 | 15.00671005 | 12.49652394 | 14.93424005 | 9.744910367 | 8.341798983 | 12.72704269 |
| ENSMUSG00000022948 | Setd4 | 4273 | 1.084264963 | 0.810798268 | 0.758489711 | 0.54378967 | 0.99124954 | 0.475465031 | 0.586841091 | 1.269206914 | 0.954239424 | 1.467597363 | 0.648478257 | 1.175616577 | 0.854332291 | 0.684759538 | 1.205287734 | 0.914028073 | 0.457440005 | 0.823913604 |
| ENSMUSG00000022949 | Clic6 | 3907 | 160.3708216 | 178.5612057 | 166.5772914 | 181.6844307 | 161.0516094 | 167.6954808 | 105.8995079 | 105.0364381 | 109.9501816 | 111.3176572 | 115.2370639 | 103.445409 | 145.3946832 | 140.651764 | 152.33081 | 147.3507249 | 173.6111601 | 173.4046988 |
| ENSMUSG00000022951 | Rcan1 | 2501 | 16.16250396 | 17.79529255 | 13.53917283 | 17.85278826 | 19.86091372 | 17.39596165 | 13.54493327 | 31.99243624 | 10.72068043 | 13.85676405 | 14.78521306 | 12.72088272 | 12.24914859 | 15.78467584 | 12.15783616 | 13.79441714 | 15.37036217 | 14.67056145 |
| ENSMUSG00000022952 | Runx1 | 6999 | 0.655887843 | 0.709190061 | 0.435423977 | 1.178246753 | 0.818359611 | 0.516838056 | 0.114918637 | 0.69847514 | 0.353077718 | 0.154945874 | 0.08191163 | 0.023924416 | 0.69074552 | 0.398149056 | 0.394414094 | 0.661975067 | 0.399745631 | 0.683782295 |
| ENSMUSG00000022956 | Atp5o | 2249 | 169.9073066 | 149.2340637 | 150.0461306 | 141.4438468 | 156.5946134 | 155.9511998 | 170.8221222 | 153.313032 | 146.3595527 | 167.8682441 | 174.1906001 | 145.7312453 | 170.3475632 | 154.5726964 | 144.727564 | 160.1088804 | 146.6419675 | 163.8777301 |
| ENSMUSG00000022957 | Itsn1 | 14973 | 0.936800113 | 1.012313289 | 1.049983127 | 0.727248353 | 1.076884688 | 0.539444037 | 1.453538523 | 0.803486727 | 0.816963546 | 0.966757891 | 0.995511056 | 0.808922646 | 0.688597523 | 0.576945185 | 1.108944568 | 0.877157439 | 0.952206134 | 1.006644977 |
| ENSMUSG00000022960 | Donson | 2824 | 4.590679422 | 4.883689167 | 5.447160996 | 4.339915342 | 4.755243167 | 5.035989567 | 3.685831928 | 2.785989694 | 2.173084578 | 2.804999344 | 4.838398769 | 2.628712154 | 3.546168274 | 2.138006856 | 4.595782615 | 5.898163676 | 4.112205369 | 3.019267543 |
| ENSMUSG00000022961 | Son | 14067 | 26.90151291 | 22.50935235 | 24.05093277 | 20.88416961 | 23.04802999 | 21.89662273 | 21.90906309 | 24.54934972 | 23.34693088 | 21.16703569 | 23.70578021 | 25.68782253 | 25.94423289 | 25.91782797 | 25.7484096 | 26.55327186 | 25.13945699 | 26.17302561 |
| ENSMUSG00000022962 | Gart | 4325 | 12.09800502 | 12.70126302 | 13.04128009 | 14.14762602 | 13.56596877 | 12.36240452 | 9.965745781 | 10.47310734 | 13.97960812 | 12.48267173 | 13.31071097 | 12.45366807 | 10.42528841 | 11.85532312 | 12.50812552 | 9.995397863 | 12.49481587 | 13.5328762 |
| ENSMUSG00000022964 | Tmem50b | 2838 | 78.98953431 | 78.58697209 | 82.82134743 | 83.92992945 | 83.11999926 | 80.63231983 | 108.1956611 | 100.985126 | 121.6003578 | 112.4771885 | 109.976675 | 118.2198548 | 103.6526507 | 111.8716636 | 100.0567735 | 114.4265826 | 116.4644061 | 97.51614374 |
| ENSMUSG00000022965 | Ifngr2 | 3390 | 49.51414163 | 44.04377342 | 44.24965122 | 48.85380418 | 42.70820838 | 48.74878067 | 33.77483275 | 36.2546168 | 28.69691747 | 32.07360522 | 26.10006581 | 30.8385652 | 23.93834924 | 22.12601803 | 24.74524124 | 32.71079618 | 25.31344506 | 24.92448021 |
| ENSMUSG00000022967 | Ifnar1 | 6035 | 13.63546326 | 15.91401392 | 17.03293322 | 15.94447815 | 17.45829013 | 17.04581192 | 9.885880386 | 14.65676951 | 13.1646709 | 14.46944531 | 15.37348291 | 13.49379482 | 9.784640427 | 9.827522717 | 13.15582406 | 13.61584813 | 12.64418511 | 13.55402514 |
| ENSMUSG00000022969 | Il10rb | 3937 | 15.34159389 | 13.91069294 | 15.08831478 | 16.29412 | 16.94458882 | 14.62541946 | 8.340106471 | 9.51657271 | 9.624484252 | 10.13195198 | 9.720025842 | 11.10075281 | 6.302757021 | 6.806766173 | 6.791925266 | 9.745285399 | 8.264929152 | 5.910300265 |
| ENSMUSG00000022971 | Ifnar2 | 3553 | 8.326372788 | 9.497878369 | 8.931335748 | 10.15602227 | 11.41998639 | 9.609297336 | 8.855314238 | 9.265930446 | 10.37487521 | 10.21841866 | 9.896529536 | 10.2896873 | 9.344323199 | 8.928035938 | 10.12354582 | 10.93863397 | 9.168970551 | 11.96792556 |
| ENSMUSG00000022972 | 1110004E09R | 1481 | 24.71097675 | 24.6079043 | 24.36641029 | 24.73401732 | 23.85471436 | 24.55888349 | 26.70722457 | 24.11201637 | 27.97679599 | 26.96599977 | 28.0327069 | 25.66540596 | 27.94698082 | 27.78491656 | 22.11697295 | 27.63852756 | 25.20581579 | 29.90029096 |
| ENSMUSG00000022973 | Synj1 | 10987 | 2.301863997 | 2.510519379 | 2.641678184 | 2.575171433 | 2.470775002 | 2.408406274 | 2.346901794 | 2.697488329 | 2.440376969 | 2.184373164 | 2.739439687 | 2.83472647 | 2.514414642 | 2.916758014 | 2.280016927 | 2.429102881 | 2.8011301 | 3.634896831 |
| ENSMUSG00000022974 | Gcfc1 | 5491 | 6.014664319 | 7.929330312 | 5.893624441 | 7.782964776 | 6.880998011 | 6.353145955 | 8.487158759 | 9.522017646 | 10.7710502 | 7.152893064 | 7.952342387 | 9.077287687 | 9.577094121 | 8.559718892 | 8.741543 | 7.342926533 | 8.731778806 | 8.154691836 |
| ENSMUSG00000022978 | 2610039C10R | 2847 | 7.703783221 | 5.499494304 | 4.774486136 | 6.417273895 | 5.511418024 | 4.977900177 | 5.417609178 | 6.841631622 | 8.159183312 | 5.200323436 | 6.343145172 | 4.744429345 | 4.713130137 | 6.150130294 | 7.351735298 | 8.0696749 | 6.30021319 | 5.042984494 |

| ENSMUSG00000022982 | Sod1 |  | 641 | 89.18792786 | 79.04666475 | 94.55855466 | 76.12461542 | 86.42721395 | 81.4025405 | 84.36566617 | 84.78594169 | 88.41286379 | 86.65137042 | 85.11544865 | 83.94118367 | 108.3609041 | 113.6104977 | 115.5057659 | 105.7322544 | 100.8681168 | 111.562931 |
| --- | --- | --- | --- | --- | --- | --- | --- | --- | --- | --- | --- | --- | --- | --- | --- | --- | --- | --- | --- | --- | --- |
| ENSMUSG00000022983 | Scaf4 |  | 5446 | 5.088757762 | 7.921446569 | 6.83056277 | 7.596296044 | 7.149968095 | 8.05254668 | 7.30627304 | 5.610341888 | 5.505652493 | 6.233658896 | 7.658380234 | 7.953168228 | 5.009282088 | 5.338596898 | 6.196120873 | 5.097445899 | 7.410501403 | 6.292815551 |
| ENSMUSG00000022986 | Krt75 |  | 3257 | 0 | 0 | 0 | 0 | 0 | 0.015214226 | 0 | 0 | 0.012645538 | 0 | 0 | 0 | 0 | 0 | 0 | 0 | 0 | 0 |
| ENSMUSG00000022987 | Zfp641 |  | 7346 | 1.215094877 | 1.115569286 | 1.126037802 | 1.277644314 | 0.930402009 | 1.207451581 | 1.455576564 | 1.440143679 | 1.603504425 | 1.887055099 | 1.183643131 | 0.425493752 | 2.175815573 | 1.890386895 | 2.064003889 | 2.027640386 | 2.681692977 | 2.066772864 |
| ENSMUSG00000022991 | Lalba |  | 729 | 0.816285959 | 0.776843898 | 1.194408157 | 0.249992013 | 0.528196693 | 0.883656429 | 0.259603177 | 0.471511141 | 0.790961894 | 0.064678605 | 0.720884147 | 0.30625878 | 0.609036016 | 0.700802416 | 0.282589547 | 0.682822903 | 0.368016568 | 0.075458308 |
| ENSMUSG00000022992 | 2310037I24Ri | k | 2856 | 14.01956436 | 14.27695467 | 13.7024461 | 15.07534555 | 14.66203557 | 16.46552667 | 13.74983316 | 13.74712524 | 11.73873383 | 13.3395547 | 14.46965654 | 14.05163214 | 12.62662168 | 13.22095345 | 15.26304136 | 13.62158863 | 13.35247389 | 18.5867605 |
| ENSMUSG00000022993 | 4930415O20R |  | 536 | 4.361538585 | 1.678074895 | 2.436726343 | 2.210050658 | 1.885765666 | 2.773473894 | 3.089447318 | 3.206451694 | 1.844172348 | 1.583419139 | 2.228309631 | 2.915743568 | 2.761114773 | 1.126442418 | 1.38363434 | 2.357444112 | 0.786547138 | 4.002528288 |
| ENSMUSG00000022994 | Adcy6 |  | 5995 | 13.81152342 | 11.49142039 | 11.33691579 | 10.41938145 | 12.33204917 | 8.976525215 | 12.67458922 | 13.09180766 | 14.86012237 | 13.12669287 | 9.833911387 | 15.16659122 | 13.6516515 | 9.17261042 | 8.467108734 | 11.94385919 | 8.413245492 | 9.111600163 |
| ENSMUSG00000022995 | Enah |  | 4272 | 17.72043973 | 17.17891057 | 18.16272067 | 12.89403151 | 15.71723631 | 14.16289506 | 13.74415603 | 10.62092089 | 14.23017258 | 14.13858869 | 11.56350821 | 11.61519757 | 14.49240547 | 13.27439857 | 12.38361371 | 11.55350958 | 13.44829566 | 10.36571416 |
| ENSMUSG00000022996 | Wnt10b |  | 3057 | 1.68240965 | 1.863429804 | 1.629857254 | 1.713941805 | 1.574482292 | 1.507492386 | 2.894167798 | 1.39926368 | 2.802353706 | 2.945954963 | 2.906817886 | 3.8159874 | 0.774593402 | 0.197505115 | 0.458244325 | 0.764058571 | 0.401191201 | 0.647801061 |
| ENSMUSG00000022997 | Wnt1 |  | 1454 | 0.087699813 | 0 | 0.033269278 | 0 | 0 | 0 | 0 | 0 | 0 | 0 | 0 | 0 | 0 | 0 | 0 | 0 | 0 | 0 |
| ENSMUSG00000022999 | Lmbr1l |  | 4094 | 7.236469881 | 7.673194736 | 7.266663695 | 8.902988634 | 6.36037389 | 8.460518019 | 8.470980375 | 10.56028322 | 10.04009349 | 10.22711883 | 10.6892677 | 9.884307615 | 9.169904506 | 12.54693714 | 8.956859976 | 9.427682526 | 9.314769277 | 8.532189245 |
| ENSMUSG00000023000 | Dhh |  | 2360 | 0 | 0.112925065 | 0.020497259 | 0 | 0 | 0 | 0.060143236 | 0 | 0 | 0 | 0 | 0 | 0 | 0 | 0 | 0 | 0 | 0 |
| ENSMUSG00000023004 | Tuba1b |  | 1872 | 40.643304 | 41.83686662 | 41.11233269 | 40.2066481 | 40.28991687 | 42.93511424 | 47.31267893 | 44.06815662 | 42.5065964 | 42.28954635 | 40.27184 | 41.32505788 | 41.26804618 | 41.80464063 | 40.49725579 | 40.88831421 | 41.82725672 | 44.48920277 |
| ENSMUSG00000023007 | Prpf40b |  | 4603 | 14.78400756 | 15.60343253 | 14.912457 | 14.71850379 | 14.37788291 | 12.69230347 | 16.53836637 | 16.17970556 | 19.24662077 | 16.33833841 | 20.72704586 | 19.21960139 | 15.24005787 | 16.36591565 | 14.90345222 | 10.93898517 | 16.11985896 | 16.3605186 |
| ENSMUSG00000023008 | Fmnl3 |  | 5457 | 1.254046791 | 1.593305001 | 1.303080257 | 1.269063357 | 0.793819522 | 2.424515277 | 2.340924191 | 3.12845957 | 2.045362978 | 3.335197262 | 2.862820492 | 1.810403569 | 1.591060554 | 2.604341349 | 1.880005024 | 0.898148753 | 1.657505235 | 0.997966202 |
| ENSMUSG00000023009 | Nckap5l |  | 4988 | 0.153386762 | 0.85486176 | 0.39761723 | 0.529779585 | 0.144743155 | 0.188753396 | 0.218161912 | 0.245019422 | 0.734883701 | 0.378113097 | 0.08620181 | 0.033569965 | 0.197802395 | 0.242090271 | 0.156942575 | 0.176560539 | 0.407236114 | 0.408046702 |
| ENSMUSG00000023010 | Tmbim6 |  | 3846 | 124.0784221 | 126.3741879 | 141.1209076 | 129.8359454 | 130.1288982 | 131.2644955 | 109.1291666 | 117.0697431 | 127.1470363 | 127.2554082 | 132.5550135 | 113.4018688 | 123.4195809 | 111.8957134 | 123.0154619 | 118.1871556 | 113.045635 | 117.5415598 |
| ENSMUSG00000023011 | Faim2 |  | 4508 | 0.594016435 | 1.559232626 | 1.770548471 | 0.919710522 | 0.736713117 | 2.088513616 | 0.703183116 | 1.728316347 | 0.676087437 | 0.857665853 | 0.201358521 | 1.498157758 | 0 | 0.257564815 | 0.036558612 | 0.331263019 | 0.034007362 | 0.29286126 |
| ENSMUSG00000023013 | Aqp2 |  | 1412 | 0 | 0 | 0 | 0 | 0.034087765 | 0 | 0 | 0 | 0 | 0 | 0 | 0 | 0.209625711 | 0 | 0 | 0 | 0 | 0 |
| ENSMUSG00000023015 | Racgap1 |  | 4369 | 2.120880836 | 1.791835693 | 1.140415113 | 2.419355067 | 2.28045507 | 1.928121929 | 2.111689721 | 0.961584863 | 1.875970848 | 1.500102484 | 1.552768163 | 2.376221845 | 2.122774193 | 1.700859231 | 1.216525683 | 2.129690455 | 3.280850319 | 1.145760749 |
| ENSMUSG00000023017 | Accn2 |  | 4484 | 0.24646177 | 0.401181153 | 0.938558684 | 0.375949741 | 0.847997763 | 0.983539984 | 0.685843918 | 0.868783462 | 0.789928714 | 0.715041887 | 0.372908909 | 0.348536698 | 0.297047673 | 0.6939683 | 0.091885718 | 0.247642198 | 0.29915709 | 0.12267865 |
| ENSMUSG00000023018 | Smarcd1 |  | 3140 | 15.60779234 | 17.03837838 | 14.68152052 | 18.4855957 | 14.60819212 | 15.57597071 | 16.21287979 | 19.81382915 | 17.09109237 | 19.28073343 | 16.09742232 | 20.53092047 | 15.25518843 | 14.93902174 | 17.25479172 | 16.19424415 | 18.00359488 | 17.02829673 |
| ENSMUSG00000023019 | Gpd1 |  | 2901 | 19.91193872 | 21.14065435 | 14.62378012 | 23.85633449 | 21.25370363 | 18.34527262 | 13.45500176 | 15.5481652 | 13.95599621 | 17.34222695 | 15.97439149 | 12.77545672 | 11.88659434 | 11.76711625 | 12.7112694 | 13.41029201 | 9.882133776 | 9.462097296 |
| ENSMUSG00000023020 | 2310016M24R |  | 721 | 155.9309162 | 125.858979 | 137.539164 | 121.1380333 | 136.785453 | 134.9126436 | 185.0509772 | 154.1468669 | 172.7434448 | 185.5945851 | 176.1908663 | 165.9761173 | 202.6645478 | 195.3099486 | 164.0061934 | 171.3783496 | 147.3515881 | 158.8473789 |
| ENSMUSG00000023021 | Lass5 |  | 6461 | 23.36764978 | 18.75235974 | 21.60000543 | 21.19741515 | 20.43427745 | 19.53425359 | 17.16466792 | 18.70901619 | 18.83704598 | 22.61569871 | 19.50632108 | 21.78720029 | 15.16378079 | 21.83109324 | 21.87935896 | 20.45207958 | 19.51609918 | 19.27574952 |
| ENSMUSG00000023022 | Lima1 |  | 9973 | 17.76838252 | 20.89696844 | 19.66372125 | 20.34782828 | 20.14948173 | 21.41008012 | 13.07942001 | 13.97413781 | 13.91743288 | 13.53579298 | 14.21320584 | 14.07564338 | 10.32344444 | 11.33043865 | 12.22867797 | 11.39541991 | 11.80187083 | 11.14192275 |
| ENSMUSG00000023025 | Larp4 |  | 6728 | 13.1533556 | 13.82920832 | 14.2431575 | 13.2322058 | 12.46937321 | 13.05104695 | 14.54260107 | 14.44703688 | 15.67144476 | 14.18445651 | 14.58528013 | 14.4184931 | 13.3008469 | 11.96998328 | 13.35621186 | 12.60038716 | 16.11550148 | 12.86107682 |
| ENSMUSG00000023026 | Dip2b |  | 171295 | 0.303475468 | 0.303772677 | 0.321652419 | 0.346837922 | 0.280145526 | 0.277422409 | 0.189200999 | 0.292750077 | 0.253666331 | 0.268103476 | 0.320182447 | 0.268170643 | 0.201019751 | 0.236700945 | 0.281419892 | 0.323456662 | 0.279680499 | 0.326917135 |
| ENSMUSG00000023027 | Atf1 |  | 2580 | 12.1749322 | 11.86610457 | 10.93091791 | 12.64407277 | 11.24943797 | 11.27420721 | 8.894050109 | 11.53173701 | 9.658078309 | 9.74082358 | 10.31420615 | 11.20640047 | 8.795613158 | 8.62275886 | 10.12472342 | 10.03273279 | 11.00766899 | 11.64146212 |
| ENSMUSG00000023030 | Slc11a2 |  | 6334 | 20.42717964 | 24.10898439 | 23.51469845 | 22.05401988 | 22.7589377 | 26.12979636 | 14.08773484 | 16.73857106 | 13.27800208 | 14.37448816 | 14.30080854 | 14.55754256 | 7.624873226 | 11.20406781 | 9.340927417 | 9.521267234 | 11.35750857 | 8.319975409 |
| ENSMUSG00000023031 | Cela1 |  | 1095 | 1.863240593 | 1.186486643 | 1.192771981 | 0.748948674 | 1.802199882 | 1.991160071 | 2.03077252 | 3.348375005 | 2.520088179 | 1.679340116 | 1.178012675 | 1.733089067 | 3.964574174 | 2.672125576 | 2.069484542 | 0.27974873 | 1.505050929 | 3.617037154 |
| ENSMUSG00000023032 | Slc4a8 |  | 13099 | 2.978834384 | 3.097572728 | 2.031104794 | 3.095604967 | 2.314918079 | 2.345422919 | 2.304411723 | 2.11969437 | 1.726192622 | 2.32891862 | 2.804713571 | 2.168881003 | 2.854690686 | 2.290471063 | 1.635606463 | 2.087143575 | 1.928164719 | 1.327038532 |
| ENSMUSG00000023033 | Scn8a |  | 11039 | 0.003850455 | 0.015088728 | 0.013146172 | 0.04952736 | 0.013080512 | 0.013466637 | 0.025715742 | 0.128011495 | 0.048503008 | 0.102510814 | 0 | 0.136518062 | 0.026813253 | 0.143047284 | 0 | 0.124871987 | 0.045134692 | 0.024915802 |
| ENSMUSG00000023034 | Nr4a1 |  | 2467 | 149.8794188 | 115.8456909 | 133.5321207 | 186.4918629 | 186.674603 | 152.8763094 | 136.3763518 | 140.9109229 | 92.65713923 | 178.1864639 | 209.3616036 | 119.9120437 | 130.0986842 | 142.7962719 | 166.5598366 | 257.6661425 | 291.0903223 | 298.3469185 |
| ENSMUSG00000023036 | Pcdhga9 |  | 28969 | 3.515564975 | 3.50044703 | 3.590151205 | 3.461626517 | 3.638679721 | 3.691352793 | 4.13040716 | 3.963076448 | 3.325460332 | 3.566128994 | 3.949774778 | 3.66850846 | 3.518234702 | 3.499859946 | 3.293962631 | 3.464381995 | 3.859222205 | 3.978186288 |
| ENSMUSG00000023039 | Krt7 |  | 7163 | 361.6233327 | 405.8378335 | 359.3539435 | 337.29675 | 382.0988004 | 402.2951439 | 233.5778675 | 244.6275893 | 258.4693922 | 229.2763012 | 247.051827 | 255.7016568 | 115.6886146 | 116.3857837 | 127.7058417 | 109.3016962 | 118.103637 | 122.6358265 |
| ENSMUSG00000023041 | Krt6b |  | 2217 | 0 | 0 | 0 | 0 | 0 | 0 | 0 | 0 | 0 | 0 | 0 | 0 | 0 | 0 | 0 | 0 | 0 | 0 |
| ENSMUSG00000023043 | Krt18 |  | 1393 | 885.2854789 | 1012.348484 | 1031.088918 | 889.7322965 | 968.7197501 | 1073.049683 | 725.3478616 | 854.9011482 | 874.1120976 | 726.4186953 | 735.2473509 | 885.0769353 | 457.8696041 | 448.8039198 | 552.74564 | 520.9501733 | 538.3290202 | 684.3559365 |
| ENSMUSG00000023044 | Csad |  | 2250 | 54.55775483 | 53.62635683 | 50.24397353 | 58.17639128 | 55.57632786 | 48.23132735 | 36.96697314 | 49.87079034 | 44.64618309 | 43.65107328 | 48.77292428 | 47.55494452 | 47.62174414 | 58.18928104 | 62.00376367 | 53.86212108 | 55.83715611 | 65.6197522 |
| ENSMUSG00000023045 | Soat2 |  | 2203 | 0.057882673 | 0.060486417 | 0 | 0.020681364 | 0.087393416 | 0 | 0.064429431 | 0.104019253 | 0.037391299 | 0.042805904 | 0.086745272 | 0 | 0 | 0 | 0.243132195 | 0 | 0 | 0.124850446 |

| ENSMUSG00000023046 | Igfbp6 | 1108 | 0 | 0 | 0 | 0 | 0 | 0 | 0 | 0.103409032 | 0 | 0.170219145 | 0 | 0.050375147 | 0 | 0 | 0 | 0 | 0 | 0 |
| --- | --- | --- | --- | --- | --- | --- | --- | --- | --- | --- | --- | --- | --- | --- | --- | --- | --- | --- | --- | --- |
| ENSMUSG00000023047 | Amhr2 | 1991 | 0.725854337 | 0.552147418 | 1.0204361 | 0.480553456 | 0.362621223 | 0.423102195 | 0.332685839 | 0.345285406 | 0.434413275 | 0.307864964 | 0.263950047 | 0.644781637 | 0.768101173 | 1.586237038 | 1.179552328 | 0.38463694 | 0.750741125 | 0.1657733 |
| ENSMUSG00000023048 | Prr13 | 1557 | 199.2310691 | 191.5116995 | 192.0028372 | 189.1500261 | 193.1150463 | 183.6347288 | 211.8283139 | 173.987611 | 167.8410035 | 201.9879195 | 196.4695306 | 190.4256902 | 241.5584697 | 252.1165232 | 227.1510313 | 278.2401619 | 236.0378041 | 255.4372778 |
| ENSMUSG00000023050 | Map3k12 | 9631 | 2.241992464 | 2.09956669 | 1.843327343 | 2.559290311 | 1.634216493 | 2.114647856 | 2.515220809 | 2.284168185 | 2.159608603 | 1.669441368 | 2.242163974 | 2.555778964 | 1.762037124 | 1.687823755 | 1.813878071 | 2.174744289 | 2.144935524 | 1.764906446 |
| ENSMUSG00000023051 | Tarbp2 | 3557 | 9.440283683 | 9.12194517 | 8.458908035 | 10.08055718 | 8.078368971 | 9.083042534 | 6.584137214 | 7.516075818 | 8.81162173 | 7.754613833 | 9.952556714 | 8.379410689 | 12.89813974 | 10.1975913 | 9.544583091 | 10.4957386 | 9.557330715 | 8.737741158 |
| ENSMUSG00000023052 | Npff | 439 | 3.195149906 | 1.517671718 | 3.085327676 | 0.830269601 | 1.315679007 | 0.564381931 | 0.646642537 | 3.392946911 | 1.407284138 | 3.11473894 | 1.414748201 | 2.03428383 | 3.7083218 | 4.654988258 | 1.595504442 | 3.052781911 | 2.968323678 | 1.879582234 |
| ENSMUSG00000023055 | Calcoco1 | 4328 | 28.70670738 | 37.21530581 | 35.47541251 | 39.88696788 | 37.74485881 | 41.24051441 | 27.72295604 | 32.21822116 | 29.67180133 | 34.58952931 | 30.08012058 | 33.51776965 | 26.62646921 | 24.76731832 | 28.62594237 | 26.22304999 | 32.75623203 | 28.20360624 |
| ENSMUSG00000023057 | Fabp2 | 961 | 0 | 0 | 0 | 0 | 0 | 0 | 0 | 0 | 0 | 0 | 0 | 0 | 0 | 0 | 0 | 0 | 0 | 0 |
| ENSMUSG00000023064 | Sncg | 847 | 0.050183207 | 0.039330454 | 0 | 0.753075113 | 0 | 0 | 0.055859125 | 0.360731073 | 0 | 0.111335781 | 0 | 0.131796134 | 0 | 1.535341732 | 0.145932311 | 0 | 0.09049893 | 0 |
| ENSMUSG00000023066 | Rttn | 7371 | 0.634319545 | 0.546853914 | 0.492201164 | 1.279492088 | 0.43750358 | 0.699156735 | 0.44931319 | 0.922296517 | 1.156642079 | 0.946724201 | 0.259259034 | 0.605786597 | 0.863358748 | 0.132319337 | 0.413636567 | 0.680514119 | 1.175110989 | 0.537329492 |
| ENSMUSG00000023067 | Cdkn1a | 2219 | 34.19186084 | 45.60818956 | 29.71298868 | 25.93222127 | 35.5295588 | 46.60502703 | 21.47087322 | 23.82076833 | 22.55142706 | 17.36012824 | 15.11402472 | 22.58786165 | 22.78738856 | 35.18364619 | 27.14586513 | 33.92489146 | 27.47955763 | 44.62207846 |
| ENSMUSG00000023068 | Nus1 | 4871 | 11.23930748 | 9.157455422 | 11.48014804 | 11.70126595 | 10.70147266 | 9.959377165 | 9.246904196 | 10.44390885 | 11.02591184 | 11.18026324 | 8.719346764 | 10.69103124 | 11.31261581 | 10.72666769 | 9.262102997 | 9.252318808 | 9.819589106 | 10.32197158 |
| ENSMUSG00000023070 | Rgn | 1600 | 2.869099382 | 2.06123533 | 2.418676518 | 1.936344384 | 1.985441851 | 1.827257051 | 11.1776204 | 9.548100601 | 6.924482963 | 9.489079031 | 5.34482348 | 6.104838095 | 11.83966015 | 21.91580374 | 7.776793674 | 3.996582148 | 5.437543373 | 10.27982682 |
| ENSMUSG00000023072 | Ccdc123 | 5217 | 6.746077391 | 6.660024664 | 5.795180462 | 5.990967302 | 7.159358399 | 5.907954818 | 8.370634972 | 8.169967622 | 9.126243477 | 6.07346609 | 8.397476888 | 7.457066671 | 6.505723421 | 7.77183981 | 8.553054443 | 7.376281472 | 7.67701366 | 7.623458724 |
| ENSMUSG00000023073 | Slc10a2 | 1623 | 0 | 0 | 0 | 0 | 0 | 0 | 0 | 0 | 0 | 0 | 0 | 0 | 0 | 0 | 0 | 0 | 0 | 0 |
| ENSMUSG00000023074 | Mospd1 | 2078 | 16.09796609 | 15.82282319 | 16.6444053 | 15.30395041 | 17.23298903 | 18.88631617 | 16.12000803 | 19.62920393 | 16.98596921 | 17.26741346 | 15.51881471 | 18.45301261 | 13.81673525 | 17.88030313 | 16.61544939 | 14.18853114 | 17.00521932 | 16.88922526 |
| ENSMUSG00000023075 | Akirin1 | 3445 | 32.85668614 | 38.92145116 | 33.50340883 | 36.44883547 | 36.87086983 | 37.44144137 | 27.5498502 | 27.07281471 | 24.46084507 | 28.67364973 | 30.63421866 | 27.65670132 | 23.6277688 | 27.00363049 | 23.01068027 | 29.59880624 | 28.03549149 | 30.03545131 |
| ENSMUSG00000023078 | Cxcl13 | 1170 | 0 | 0 | 0 | 0 | 0 | 0 | 0 | 0 | 0 | 0 | 0 | 0 | 0 | 0 | 0 | 0 | 0 | 0 |
| ENSMUSG00000023079 | Gtf2ird1 | 4041 | 18.87015239 | 20.36200166 | 20.0987769 | 20.93710946 | 19.93883698 | 20.36800061 | 11.23983464 | 15.66068072 | 14.4015211 | 13.47663011 | 13.31220075 | 14.77920291 | 12.67174713 | 13.20570562 | 14.68206891 | 14.57336159 | 14.63436677 | 15.08292261 |
| ENSMUSG00000023083 | H2-M10.2 | 1108 | 0 | 0 | 0 | 0 | 0 | 0 | 0 | 0 | 0 | 0.042554786 | 0 | 0 | 0.133570173 | 0 | 0 | 0 | 0 | 0 |
| ENSMUSG00000023084 | 4933430H15R | 5117 | 0.058146616 | 0.214837895 | 0.094534943 | 0.06232701 | 0.009406278 | 0.058103655 | 0.009246175 | 0.126885058 | 0.008048958 | 0.073716167 | 0 | 0.207249871 | 0.028922367 | 0.081687861 | 0 | 0.044898113 | 0.209719819 | 0.118252917 |
| ENSMUSG00000023087 | Ccrn4l | 11459 | 2.247852053 | 2.907137989 | 2.220479707 | 2.278251017 | 2.805840388 | 2.996774969 | 3.608629146 | 4.982776996 | 3.37140691 | 3.579816025 | 3.347874305 | 4.534809483 | 3.956368966 | 3.274877617 | 2.851281423 | 3.732490485 | 3.568737132 | 4.013230929 |
| ENSMUSG00000023088 | Abcc1 | 11188 | 19.47457386 | 22.36144401 | 23.37391 | 24.77595582 | 22.31930819 | 26.23353959 | 16.2811775 | 15.40258488 | 12.12992219 | 18.18513448 | 17.13629857 | 15.34581499 | 15.1417415 | 14.38820226 | 17.48156827 | 17.33823893 | 16.28559309 | 15.19289773 |
| ENSMUSG00000023089 | Ndufa5 | 1184 | 94.73915501 | 84.77343774 | 95.68480413 | 79.73182759 | 99.31274453 | 85.37802069 | 124.0359421 | 101.2227692 | 110.6191893 | 103.6996886 | 107.9375118 | 97.96025917 | 124.4547609 | 123.9162771 | 102.4819106 | 99.08988267 | 97.01343904 | 108.903164 |
| ENSMUSG00000023092 | Fhl1 | 3748 | 3.277480222 | 4.52408302 | 3.213716398 | 3.999355279 | 2.170302855 | 3.516816204 | 8.83641282 | 9.823232636 | 9.274658299 | 7.296533588 | 4.89476629 | 9.113976922 | 16.08420569 | 22.0200489 | 12.10323187 | 3.974131878 | 5.256060995 | 12.10846133 |
| ENSMUSG00000023093 | Gm7257 | 515 | 0 | 0 | 0 | 0 | 0 | 0 | 0 | 0 | 0 | 0 | 0 | 0 | 0 | 0 | 0 | 0 | 0 | 0 |
| ENSMUSG00000023094 | Msrb2 | 1370 | 17.46745555 | 15.39201609 | 12.88783838 | 13.43551964 | 16.72320852 | 13.1296659 | 13.67578165 | 10.34261408 | 18.78946887 | 12.87179782 | 14.47197647 | 10.6742362 | 11.5587923 | 11.08555954 | 11.87928071 | 14.25416772 | 13.34426542 | 13.97311616 |
| ENSMUSG00000023104 | Rfc2 | 1507 | 30.54618821 | 32.40657128 | 34.63506254 | 31.07946488 | 35.83544682 | 32.71729921 | 30.70457863 | 30.74146259 | 33.86203917 | 28.90992025 | 31.35330719 | 29.70415487 | 25.2388243 | 31.71265157 | 30.18348885 | 28.66078643 | 27.1615694 | 26.53723994 |
| ENSMUSG00000023106 | Denr | 2932 | 48.63740299 | 47.35611975 | 46.49270414 | 44.3023661 | 45.25911103 | 44.80364827 | 49.16839451 | 48.35272774 | 51.35671952 | 46.2179813 | 39.95368288 | 41.70945318 | 46.30335482 | 46.00055586 | 46.90681909 | 44.74202625 | 49.4241912 | 47.03541288 |
| ENSMUSG00000023110 | Prmt5 | 3701 | 14.42488546 | 15.49081085 | 14.11602615 | 15.8435731 | 12.67998529 | 15.1161946 | 13.75532087 | 14.49887203 | 15.05683756 | 12.94382991 | 12.40522429 | 13.70884553 | 11.55654479 | 15.61103613 | 12.1901388 | 15.37418065 | 14.59112465 | 14.50658961 |
| ENSMUSG00000023118 | Sympk | 7789 | 6.084641324 | 7.685617013 | 7.632695957 | 7.341007914 | 6.754165178 | 7.500664127 | 5.011292864 | 6.74214319 | 5.779543167 | 7.179449745 | 5.90056619 | 5.496291334 | 5.630514117 | 6.666387174 | 6.992997418 | 4.385058647 | 6.057218062 | 6.377355677 |
| ENSMUSG00000023120 | Gm853 | 2574 | 0 | 0 | 0.037586271 | 0 | 0 | 0.077005025 | 0 | 0 | 0 | 0 | 0 | 0 | 0 | 0 | 0 | 0 | 0 | 0 |
| ENSMUSG00000023122 | Sult1c2 | 1838 | 0.832527931 | 1.250592873 | 1.10537991 | 2.305319436 | 1.675975579 | 1.105365657 | 2.41969087 | 2.576636506 | 2.308058283 | 1.436582907 | 2.625283355 | 1.609483198 | 5.421679701 | 5.508602313 | 6.07487576 | 1.854115375 | 2.04351311 | 3.621382978 |
| ENSMUSG00000023132 | Gzma | 933 | 0 | 0 | 0 | 0 | 0 | 0 | 0 | 0 | 0 | 0 | 0 | 0 | 0 | 0 | 0 | 0 | 0 | 0 |
| ENSMUSG00000023140 | Reg2 | 686 | 0 | 0 | 0 | 0 | 0 | 0 | 0 | 0 | 0 | 0 | 0 | 0 | 0 | 0 | 0 | 0 | 0 | 0 |
| ENSMUSG00000023143 | Nagpa | 2945 | 3.088661348 | 3.800724094 | 2.792359987 | 4.130661745 | 3.203347042 | 4.896382163 | 4.867824012 | 4.331498269 | 3.678116688 | 3.89053341 | 5.191166965 | 4.870840504 | 5.209584473 | 4.699605447 | 4.239073492 | 5.369786369 | 3.435701997 | 5.622322962 |
| ENSMUSG00000023147 | Wrb | 4423 | 8.793180209 | 8.164408169 | 8.301042175 | 8.879407691 | 7.421653163 | 8.514600389 | 10.61138989 | 8.678128966 | 9.432950592 | 9.231858246 | 7.161383105 | 7.48331176 | 8.610499692 | 6.205845726 | 8.961315118 | 8.72641471 | 7.928682267 | 8.531821662 |
| ENSMUSG00000023150 | Ivns1abp | 4460 | 111.6570947 | 152.7314038 | 149.1878722 | 120.0114684 | 141.3956197 | 106.2717257 | 133.2286849 | 167.387195 | 171.0625601 | 151.5482805 | 160.6031284 | 121.0799407 | 118.9385889 | 140.1965804 | 151.9561419 | 132.3000583 | 176.5759526 | 140.0635462 |
| ENSMUSG00000023151 | Lrrc69 | 1388 | 0 | 0 | 0.139404987 | 0 | 0 | 0 | 0.068173889 | 0.05503228 | 0 | 0 | 0 | 0 | 0 | 0 | 0 | 0 | 0 | 0.356687291 |
| ENSMUSG00000023153 | Tmem52 | 1232 | 131.6556426 | 119.1639 | 106.2882684 | 107.6527598 | 117.0871552 | 111.8556429 | 126.3463585 | 126.8223362 | 124.6627575 | 113.6668739 | 115.4820019 | 122.1873717 | 75.95994081 | 76.79107631 | 83.23918232 | 56.2237896 | 74.56829032 | 74.74451676 |
| ENSMUSG00000023156 | Rpp14 | 2461 | 15.2161967 | 12.64292694 | 12.10812462 | 11.9410295 | 12.96686932 | 13.02747608 | 15.34153506 | 15.79840132 | 17.50552438 | 17.5881128 | 14.88963558 | 16.39769364 | 15.07419821 | 17.40001974 | 18.03091251 | 18.12619164 | 16.91278279 | 15.66898977 |

| ENSMUSG00000023159 | Psg29 | 5436 | 0 | 0 | 0 | 0 | 0 | 0 | 0 | 0 | 0 | 0 | 0 | 0 | 0 | 0 | 0 | 0 | 0 | 0 |
| --- | --- | --- | --- | --- | --- | --- | --- | --- | --- | --- | --- | --- | --- | --- | --- | --- | --- | --- | --- | --- |
| ENSMUSG00000023165 | Ssxb2 | 920 | 0 | 0 | 0 | 0 | 0 | 0 | 0 | 0 | 0 | 0 | 0 | 0 | 0 | 0 | 0 | 0 | 0 | 0 |
| ENSMUSG00000023169 | Slc38a1 | 8373 | 4.675417069 | 6.74374247 | 4.887615751 | 4.119158075 | 4.409075057 | 5.687349453 | 0.836292426 | 1.979637244 | 2.070885362 | 1.221987652 | 1.41504714 | 2.353150471 | 1.260842028 | 0.998439715 | 1.072726524 | 2.291125559 | 0.956669778 | 1.405941579 |
| ENSMUSG00000023170 | Gps2 | 1615 | 65.71852913 | 58.56056142 | 60.86378556 | 51.541813 | 52.93021451 | 49.09249147 | 56.5408485 | 55.85786655 | 58.96174888 | 52.87301771 | 66.35246555 | 55.33181164 | 58.70956347 | 62.23220131 | 61.43241275 | 60.74336605 | 51.37859616 | 61.03796857 |
| ENSMUSG00000023175 | Bsg | 1744 | 870.5030804 | 827.1301293 | 885.8957489 | 852.806612 | 913.1818178 | 872.6565977 | 1303.920069 | 1175.664635 | 1173.343377 | 1338.928571 | 1329.535456 | 1212.262424 | 1377.10726 | 1070.637411 | 1144.217292 | 1235.838136 | 1204.025891 | 1209.632593 |
| ENSMUSG00000023176 | Cpn2 | 4581 | 0 | 0 | 0 | 0 | 0 | 0 | 0 | 0 | 0 | 0 | 0 | 0 | 0 | 0 | 0 | 0 | 0 | 0 |
| ENSMUSG00000023185 | Ceacam14 | 1106 | 0 | 0 | 0 | 0 | 0 | 0 | 0 | 0 | 0 | 0 | 0 | 0 | 0 | 0 | 0 | 0 | 0 | 0 |
| ENSMUSG00000023186 | Vwa5a | 4995 | 16.87442724 | 15.55268655 | 16.32788232 | 17.5494393 | 19.50330601 | 17.39057897 | 11.00647306 | 13.84713529 | 15.18830071 | 14.74462432 | 16.42234308 | 15.18588297 | 14.61686438 | 11.5668558 | 11.91092026 | 14.48067215 | 19.5429586 | 14.99948015 |
| ENSMUSG00000023191 | Leprel2 | 4551 | 0.214814117 | 0.351355509 | 0.233842599 | 0.400448643 | 0.095185083 | 0.304872894 | 0.020792212 | 0.01678418 | 0.162899865 | 0.072523604 | 0.346423562 | 0.318876561 | 0 | 0 | 0.09958627 | 0.092550358 | 0.109479644 | 0.253832398 |
| ENSMUSG00000023192 | Grm2 | 3314 | 0 | 0 | 0 | 0 | 0 | 0 | 0 | 0 | 0 | 0 | 0 | 0 | 0 | 0 | 0.012432576 | 0.011554197 | 0 | 0 |
| ENSMUSG00000023206 | Il15ra | 2734 | 2.50304804 | 3.740694413 | 2.317824607 | 2.499691533 | 2.341457881 | 2.446824811 | 3.564890951 | 2.612282096 | 1.74748933 | 3.345733881 | 2.586208433 | 3.613523145 | 2.868973975 | 1.936585941 | 2.622191194 | 2.352897602 | 1.710244412 | 2.333963562 |
| ENSMUSG00000023210 | Lcn9 | 832 | 65.2904026 | 243.7206576 | 58.19940371 | 95.33867571 | 54.49551934 | 168.3125302 | 0.056866201 | 0.321330309 | 0.643539309 | 0.170014555 | 0.11484365 | 0.201258399 | 0.237172679 | 0.111644441 | 0.198084403 | 0.138067094 | 0.138195782 | 0.13223343 |
| ENSMUSG00000023216 | Epb4.2 | 6402 | 0.013278718 | 0.020814054 | 0 | 0 | 0 | 0 | 0.088683559 | 0 | 0 | 0 | 0 | 0 | 0 | 0.072546216 | 0 | 0.065791422 | 0 | 0 |
| ENSMUSG00000023224 | Serping1 | 2638 | 0.24168978 | 0.896594196 | 1.063557529 | 1.208973883 | 1.058245479 | 0.563526159 | 1.273389008 | 1.331956414 | 0.718187917 | 0.857935465 | 1.466933903 | 1.35413283 | 0.635816978 | 1.021136114 | 1.09329375 | 0.841870822 | 1.075112195 | 0.229378383 |
| ENSMUSG00000023232 | Serinc2 | 2872 | 113.2632702 | 131.8132764 | 95.68594218 | 115.6793647 | 122.4079284 | 118.2226081 | 46.93378213 | 47.96657224 | 46.22010456 | 52.63410677 | 52.84855261 | 49.26626207 | 72.48631299 | 56.0822058 | 62.56266896 | 84.44732148 | 75.0512165 | 67.2482499 |
| ENSMUSG00000023235 | Ccl25 | 2718 | 2.955657936 | 2.463536327 | 2.420456265 | 2.380304745 | 3.13441887 | 3.044630797 | 3.081068495 | 3.077312777 | 3.682238164 | 2.411312639 | 2.355351154 | 2.936585633 | 3.466665268 | 4.288986731 | 2.865008853 | 1.662358967 | 3.229110372 | 2.388180498 |
| ENSMUSG00000023236 | Scg5 | 1562 | 248.228051 | 216.427177 | 197.6129944 | 220.2797739 | 250.8591489 | 227.1114082 | 264.4300173 | 285.9539988 | 285.3524159 | 347.2911914 | 353.5408099 | 298.2671803 | 313.8988001 | 247.7414444 | 257.9185746 | 366.4082643 | 350.9481592 | 293.8514638 |
| ENSMUSG00000023243 | Kcnk5 | 3359 | 2.454898526 | 4.155433962 | 3.03864689 | 2.712774298 | 3.940541533 | 3.5405347 | 1.887436432 | 4.332034926 | 0.968661728 | 1.333526886 | 1.422297067 | 1.412423733 | 1.336470519 | 3.055713996 | 2.72305609 | 2.029094408 | 1.540354296 | 2.407364897 |
| ENSMUSG00000023247 | Guca2a | 591 | 0 | 0.056366995 | 0.081850305 | 0 | 0 | 0.419227864 | 0.56038706 | 0 | 0.139379072 | 0.877593462 | 0.161674986 | 0.377770982 | 0.66777553 | 0.785855963 | 0.209144954 | 1.101421871 | 2.075197125 | 0 |
| ENSMUSG00000023249 | Parp3 | 4369 | 0.914508251 | 1.044602085 | 1.018623207 | 0.896829895 | 0.705068234 | 0.873325815 | 0.357362876 | 0.646884362 | 0.518484405 | 0.74465519 | 1.027888784 | 0.536566223 | 1.535623884 | 1.860314784 | 1.782351582 | 1.779124125 | 1.13163019 | 1.775299622 |
| ENSMUSG00000023257 | Cypt1 | 970 | 0 | 0 | 0 | 0 | 0 | 0 | 0 | 0 | 0 | 0 | 0 | 0 | 0 | 0 | 0 | 0 | 0 | 0 |
| ENSMUSG00000023259 | Slc26a6 | 2063 | 1.730700333 | 2.002326167 | 1.571025949 | 1.8772122 | 2.239779288 | 1.200987241 | 2.201656412 | 2.332643094 | 1.517292873 | 2.719793353 | 2.014746189 | 0.784611835 | 2.845612291 | 1.823543906 | 2.676203824 | 1.447730188 | 1.560547232 | 2.933107969 |
| ENSMUSG00000023262 | Acy1 | 1416 | 14.16839201 | 12.84522616 | 13.28905601 | 13.96433139 | 14.75229864 | 15.78268562 | 15.93795752 | 14.13334665 | 13.23436771 | 17.78140928 | 16.43107682 | 14.42692974 | 16.33945565 | 16.17015189 | 14.37398913 | 14.52122611 | 13.28969758 | 18.06443123 |
| ENSMUSG00000023263 | 9530002B09R | 798 | 0 | 0 | 0 | 0 | 0 | 0 | 0 | 0 | 0 | 0 | 0 | 0 | 0 | 0 | 0 | 0 | 0 | 0 |
| ENSMUSG00000023266 | Frs3 | 3121 | 2.465055066 | 2.198800452 | 1.580935628 | 2.934242202 | 2.791373978 | 2.85789556 | 3.153167964 | 4.221845187 | 3.972169575 | 2.371887347 | 2.265521902 | 2.789888934 | 2.718708674 | 3.184567351 | 2.481862386 | 2.343321376 | 2.419186315 | 2.432315517 |
| ENSMUSG00000023267 | Gabrr2 | 2349 | 0 | 0 | 0 | 0 | 0.061471167 | 0 | 0.080566503 | 0.308921092 | 0.070134552 | 0 | 0 | 0.190091656 | 0.147008693 | 0.494296375 | 0.438501021 | 0 | 0.17947606 | 0.515198105 |
| ENSMUSG00000023272 | Creld2 | 1354 | 47.33959044 | 53.78285581 | 51.66035812 | 53.13211891 | 45.53692335 | 53.28565738 | 52.79871334 | 60.22214116 | 57.70370783 | 51.19020221 | 45.83432132 | 45.3862958 | 56.40015358 | 63.45757878 | 48.13948406 | 48.72578771 | 39.8831258 | 53.22151389 |
| ENSMUSG00000023274 | Cd4 | 4055 | 0.041928657 | 0.221812119 | 0 | 0.112357693 | 0.094958172 | 0.109981406 | 0 | 0 | 0 | 0.058138968 | 0 | 0 | 0 | 0 | 0.050803398 | 0 | 0 | 0 |
| ENSMUSG00000023277 | Twf2 | 1585 | 14.61534444 | 9.058585116 | 9.247431986 | 8.824757483 | 10.87143765 | 9.285275629 | 19.37282563 | 14.19263408 | 18.18962841 | 14.81454273 | 16.96990639 | 18.10047355 | 20.35525167 | 19.86693454 | 18.04030271 | 16.83820401 | 14.9436287 | 21.5524639 |
| ENSMUSG00000023279 | Bmp15 | 3058 | 0 | 0 | 0 | 0 | 0 | 0 | 0 | 0 | 0 | 0 | 0 | 0 | 0 | 0 | 0 | 0 | 0 | 0 |
| ENSMUSG00000023284 | Zfp605 | 3576 | 3.102307311 | 2.46865687 | 3.030109284 | 1.592598026 | 3.028434795 | 2.494265113 | 2.804890362 | 3.663309291 | 2.476258638 | 2.66343458 | 2.805576421 | 3.730409216 | 1.986520159 | 1.753342225 | 2.696074966 | 4.272358041 | 3.4403639 | 3.891863535 |
| ENSMUSG00000023286 | Ube2j2 | 4412 | 15.14463661 | 14.03641667 | 15.07561293 | 17.18349451 | 13.13482232 | 14.33120763 | 13.91927861 | 16.50791283 | 16.25243063 | 15.43191648 | 15.18143513 | 14.39669629 | 17.09621521 | 17.85339352 | 17.54708149 | 15.72587956 | 13.80337393 | 19.26316181 |
| ENSMUSG00000023289 | Sva | 647 | 0 | 0 | 0 | 0 | 0 | 0 | 0 | 0 | 0 | 0 | 0 | 0 | 0 | 0.287135007 | 0 | 0 | 0 | 0 |
| ENSMUSG00000023307 | 5-Mar | 2127 | 60.77021171 | 57.36959639 | 58.35753591 | 59.37715792 | 60.89469045 | 62.64565143 | 72.24804004 | 59.18296113 | 59.85308896 | 65.61639946 | 66.01344756 | 59.62067955 | 65.5207646 | 62.689692 | 61.17278491 | 70.7845173 | 68.3999168 | 65.19885193 |
| ENSMUSG00000023328 | Ache | 3962 | 0.096553908 | 0.075672905 | 0.085465601 | 0.022999013 | 0.036445172 | 0.050028 | 0.107474536 | 0.501263232 | 0.50937387 | 0.238014656 | 0.156757814 | 0.338105982 | 0.149415196 | 0.398561073 | 0.395168885 | 0.879466248 | 0.648122638 | 0.055536705 |
| ENSMUSG00000023330 | Dtwd1 | 1345 | 11.94569259 | 7.900976398 | 8.092226268 | 8.705716278 | 7.264520821 | 7.884226755 | 11.43243171 | 9.115064068 | 10.41146123 | 10.55194177 | 12.8228699 | 9.37869126 | 11.92035176 | 12.74190948 | 10.81349385 | 10.16338055 | 8.919056454 | 9.693054493 |
| ENSMUSG00000023333 | Gcm1 | 2028 | 0 | 0.065705906 | 0 | 0 | 0 | 0 | 0 | 0 | 0 | 0.046499707 | 0 | 0 | 0 | 0 | 0 | 0 | 0 | 0 |
| ENSMUSG00000023336 | Wfdc1 | 1206 | 0.140979025 | 0 | 0.040110722 | 0 | 0.638566151 | 0.287619515 | 1.294625543 | 0.126674635 | 0 | 0.156387075 | 0.198071967 | 1.064477811 | 0.572675657 | 0.770217038 | 0.034163811 | 0.444501247 | 0.286017141 | 0.45612858 |
| ENSMUSG00000023341 | Mx2 | 2403 | 0.37145597 | 0.346576095 | 0.785088508 | 1.042803761 | 0.660987716 | 1.154786966 | 0.452847114 | 0.8423626 | 1.131215165 | 0.333567189 | 1.033831811 | 1.695606895 | 0.800642852 | 1.932754365 | 0.7887106 | 1.689057172 | 2.073415979 | 1.167484163 |
| ENSMUSG00000023345 | Poc1a | 2642 | 2.123649976 | 2.761364055 | 3.204151329 | 3.828369358 | 2.714480177 | 2.850876421 | 5.085844366 | 2.789982462 | 4.318192606 | 3.747784892 | 3.218751934 | 3.359080375 | 4.81742417 | 3.884989899 | 5.42700282 | 5.174014702 | 5.715579478 | 5.18443133 |
| ENSMUSG00000023348 | Trip6 | 2035 | 24.1036723 | 25.22612776 | 26.43310357 | 24.06787354 | 24.62178503 | 23.01097456 | 26.15565788 | 26.7252978 | 25.80481946 | 22.70648118 | 21.26983409 | 30.22548411 | 31.00517061 | 28.11750155 | 24.70068707 | 35.52465198 | 27.19566227 | 32.89733803 |

| ENSMUSG00000023349 | Clec4n | 1561 | 0 | 0 | 0 | 0 | 0 | 0.15872112 | 0 | 0.195733004 | 1.055387976 | 0 | 0 | 0 | 0 | 0 | 0 | 0 | 0 | 0 |
| --- | --- | --- | --- | --- | --- | --- | --- | --- | --- | --- | --- | --- | --- | --- | --- | --- | --- | --- | --- | --- |
| ENSMUSG00000023350 | 4921501E09R | 3915 | 0 | 0 | 0 | 0 | 0 | 0 | 0 | 0 | 0 | 0 | 0 | 0 | 0 | 0 | 0 | 0 | 0 | 0 |
| ENSMUSG00000023353 | Agap3 | 3499 | 10.84799148 | 11.33914176 | 10.49314362 | 12.27895536 | 9.340261838 | 10.72061141 | 11.57463651 | 8.961406795 | 10.77040924 | 13.00383785 | 8.246949106 | 12.28295518 | 6.27399348 | 5.827080413 | 9.714570909 | 8.71086696 | 6.287309066 | 6.524372475 |
| ENSMUSG00000023367 | Tmem176a | 1111 | 519.9710598 | 469.9786715 | 500.1936244 | 467.9541646 | 516.4975643 | 497.088403 | 469.6347644 | 476.769063 | 526.0455974 | 502.4881427 | 523.8044728 | 534.393522 | 558.7694392 | 450.1021502 | 500.982735 | 520.4903267 | 511.8682209 | 498.6469071 |
| ENSMUSG00000023387 | Kcnk16 | 874 | 0 | 0 | 0 | 0 | 0 | 0 | 0 | 0 | 0 | 0 | 0 | 0 | 0 | 0 | 0 | 0 | 0 | 0 |
| ENSMUSG00000023391 | Dlx2 | 2909 | 0 | 0 | 0 | 0 | 0 | 0 | 0 | 0 | 0 | 0 | 0 | 0 | 0 | 0 | 0 | 0 | 0 | 0 |
| ENSMUSG00000023393 | Slc17a9 | 3257 | 1.68350252 | 2.649075715 | 1.247582607 | 1.650661109 | 1.477799314 | 2.069134715 | 2.890765461 | 0.914647647 | 1.150743916 | 1.085754604 | 0.616072538 | 0.531251317 | 1.923596406 | 2.352862886 | 0.822260096 | 0.822948271 | 0.364788211 | 1.013370096 |
| ENSMUSG00000023403 | Stk31 | 3262 | 0 | 0 | 0 | 0 | 0 | 0.015190905 | 0 | 0 | 0.012626154 | 0 | 0 | 0 | 0 | 0 | 0 | 0 | 0 | 0 |
| ENSMUSG00000023411 | Nfatc4 | 4010 | 1.494571027 | 2.018711545 | 0.965057962 | 1.88606817 | 2.028502519 | 1.915379976 | 0.365758865 | 0.590505972 | 0.472463772 | 0.03527484 | 0.571869827 | 0.208786768 | 0 | 0.011582067 | 0.020549404 | 0 | 0.08601912 | 0.013717982 |
| ENSMUSG00000023433 | Cela3b | 1740 | 0 | 0 | 0 | 0 | 0 | 0 | 0 | 0 | 0 | 0 | 0 | 0 | 0 | 0 | 0 | 0 | 0 | 0 |
| ENSMUSG00000023439 | Gnb3 | 3158 | 0 | 0.010548732 | 0 | 0.014427183 | 0 | 0 | 0.55432841 | 0.520035878 | 0.612972211 | 0.3583334 | 0.257179954 | 0.371161784 | 0.296803809 | 0.117654433 | 0 | 0.02424991 | 0.012136256 | 0 |
| ENSMUSG00000023443 | Esx1 | 1980 | 0 | 0 | 0 | 0 | 0 | 0 | 0 | 0 | 0 | 0 | 0 | 0 | 0 | 0 | 0 | 0 | 0 | 0 |
| ENSMUSG00000023452 | Pisd | 4935 | 25.64091368 | 19.97423587 | 22.11361388 | 23.40369755 | 21.73982935 | 22.13054201 | 25.69361287 | 19.98232685 | 25.062433 | 22.27118324 | 21.38498141 | 20.2565049 | 20.82236751 | 18.00355404 | 23.35172276 | 21.91138304 | 21.16295016 | 20.30933991 |
| ENSMUSG00000023456 | Tpi1 | 2915 | 327.9241522 | 302.9473457 | 310.7190334 | 299.4056142 | 316.4653341 | 308.3828953 | 453.0493334 | 407.7088089 | 419.1699359 | 410.9956155 | 406.8820306 | 412.0785161 | 379.7965309 | 291.0283706 | 329.2308204 | 306.4430806 | 318.6802005 | 317.8262008 |
| ENSMUSG00000023460 | Rab12 | 3014 | 39.05004394 | 37.57924364 | 35.74248577 | 32.92367437 | 39.31678702 | 38.84973771 | 31.59934396 | 28.37186098 | 29.2432461 | 30.31787091 | 29.54629151 | 25.85224452 | 27.02289165 | 25.31772912 | 23.94994224 | 26.06911957 | 30.11170241 | 22.24820872 |
| ENSMUSG00000023461 | Tspan18 | 664 | 0 | 0 | 0 | 0 | 0 | 0 | 0 | 0 | 0 | 0 | 0 | 0 | 0 | 0 | 0 | 0 | 0 | 0 |
| ENSMUSG00000023467 | Tulp2 | 3142 | 0.162336764 | 0.233253874 | 0.307915534 | 0.319014314 | 0.275739856 | 0.488903482 | 0.587267498 | 0.668549374 | 0.602985272 | 1.800790704 | 0.349721211 | 0.479638094 | 1.868395339 | 1.803366856 | 2.373482373 | 2.059552085 | 2.793355185 | 2.573627845 |
| ENSMUSG00000023473 | Celsr3 | 11506 | 0.018470874 | 0.023162103 | 0.037837804 | 0 | 0.016732809 | 0.004306686 | 0.024672004 | 0.003319347 | 0 | 0 | 0 | 0.014553015 | 0.004287495 | 0 | 0.003580876 | 0 | 0.009992951 | 0 |
| ENSMUSG00000023484 | Prph | 1857 | 0.206002469 | 0.430538213 | 0.052098579 | 0.122673787 | 0.285111018 | 0.106737175 | 0.025478018 | 0 | 0.155253425 | 0.279298727 | 0 | 0 | 0 | 0 | 0 | 0 | 0.144471771 | 0.177735401 |
| ENSMUSG00000023495 | Pcbp4 | 2002 | 6.53925785 | 7.604391937 | 5.267447361 | 7.282484609 | 5.52964158 | 6.559178021 | 5.057399246 | 3.968041808 | 5.163743984 | 5.299154962 | 4.104541887 | 4.265632555 | 4.287589214 | 5.753313523 | 4.939247459 | 5.699600902 | 5.781489343 | 5.083259113 |
| ENSMUSG00000023505 | Cdca3 | 2774 | 0.827425921 | 0.588439732 | 0.732403848 | 0.410607826 | 0.29496853 | 0.607351457 | 1.210958978 | 0.977527243 | 2.420116103 | 1.291800089 | 1.050566859 | 1.388349214 | 1.191506293 | 1.824947918 | 2.970551975 | 1.29751878 | 1.395441956 | 1.804552528 |
| ENSMUSG00000023571 | Fam132a | 1307 | 10.08156432 | 11.54609111 | 9.807946093 | 8.993687405 | 8.138603772 | 12.89053513 | 10.82363504 | 7.422241936 | 11.8801197 | 9.848616671 | 8.918967 | 7.558815821 | 9.058653517 | 11.44223117 | 7.376560121 | 7.968665045 | 9.647552931 | 9.385639479 |
| ENSMUSG00000023572 | Ccndbp1 | 3172 | 33.92910016 | 34.33160504 | 34.19087486 | 37.17275622 | 33.95940894 | 32.68105884 | 34.72380723 | 30.73934532 | 31.7208884 | 30.87389996 | 35.43962084 | 31.07517659 | 40.88701467 | 33.33959237 | 29.53730709 | 36.62474869 | 36.85220856 | 33.660995 |
| ENSMUSG00000023577 | Iqcf3 | 1114 | 0 | 0 | 0 | 0 | 0 | 0 | 0 | 0 | 0 | 0 | 0 | 0 | 0 | 0 | 0 | 0 | 0 | 0 |
| ENSMUSG00000023707 | Ogfod2 | 2303 | 15.17119179 | 12.06380972 | 9.347034741 | 12.48329091 | 11.05592168 | 10.86588383 | 13.600084 | 12.9187501 | 13.16251654 | 13.28736709 | 12.11488309 | 14.17809927 | 15.23013165 | 18.23076639 | 16.7990712 | 16.65965637 | 12.44814159 | 17.65164128 |
| ENSMUSG00000023723 | Mrps23 | 1401 | 38.89481489 | 36.59424997 | 36.5650026 | 32.845578 | 38.06578974 | 38.62354395 | 32.18342828 | 33.33997726 | 31.95556219 | 35.13585596 | 33.69140541 | 36.33396451 | 34.36684601 | 41.83614439 | 38.81945309 | 37.17004002 | 36.52077542 | 35.33775592 |
| ENSMUSG00000023737 | 1700082M22R | 2235 | 0 | 0.044715294 | 0 | 0 | 0.021535536 | 0 | 0.084675935 | 0.034176646 | 0.110567828 | 0 | 0.064127461 | 0.149840705 | 0 | 0.020780352 | 0.055304102 | 0 | 0.034296463 | 0 |
| ENSMUSG00000023755 | Rhebl1 | 2183 | 3.387952647 | 3.067288932 | 4.077292526 | 2.087084028 | 3.373423876 | 3.722697346 | 3.727796965 | 4.233880615 | 4.395995498 | 4.730189653 | 4.989780363 | 4.96025586 | 4.677831826 | 4.723127531 | 4.454222258 | 4.630655224 | 4.652528025 | 3.124658375 |
| ENSMUSG00000023764 | Sfi1 | 6903 | 5.781886178 | 6.254311301 | 6.643214077 | 5.537551238 | 6.484526874 | 5.656606412 | 5.942357327 | 6.550746697 | 8.818436954 | 5.976657301 | 7.640671326 | 7.34991124 | 7.403718137 | 7.643123754 | 7.508555311 | 5.585780335 | 4.913627808 | 4.95663688 |
| ENSMUSG00000023781 | Hes7 | 1805 | 0.023548574 | 0.018455897 | 0 | 0.100966303 | 0.133329428 | 0.082359114 | 0 | 0.105796129 | 0 | 0 | 0 | 0.030922805 | 0 | 0.077192389 | 0.091305387 | 0.021213633 | 0.021233405 | 0 |
| ENSMUSG00000023791 | Pigx | 2119 | 20.7009635 | 22.9841677 | 25.70485851 | 23.04928718 | 21.82858832 | 24.18005027 | 21.32308088 | 24.94491974 | 28.28050044 | 19.0916958 | 23.6958383 | 23.23238056 | 21.90718931 | 23.03574131 | 25.35480363 | 20.99749212 | 20.85428048 | 23.62354276 |
| ENSMUSG00000023795 | Pisd-ps2 | 2130 | 5.368024578 | 4.61375765 | 5.155301122 | 4.77000605 | 5.129552428 | 3.838592038 | 8.307484469 | 7.190212847 | 5.472199043 | 4.427296081 | 6.661578717 | 4.061703146 | 3.636202353 | 4.709822947 | 3.888034147 | 5.123391131 | 4.876256554 | 4.080487729 |
| ENSMUSG00000023800 | Tiam2 | 6852 | 4.782762802 | 4.42907861 | 5.358363914 | 5.246302391 | 4.355194494 | 5.366043245 | 2.75507281 | 2.881709288 | 4.147503776 | 3.915462661 | 3.79996386 | 3.233919738 | 2.771860501 | 3.158631746 | 3.800260263 | 2.805295522 | 3.35606803 | 3.106906641 |
| ENSMUSG00000023802 | Nox3 | 1779 | 0 | 0 | 0 | 0 | 0 | 0 | 0 | 0 | 0 | 0 | 0 | 0 | 0 | 0 | 0 | 0 | 0 | 0 |
| ENSMUSG00000023805 | Synj2 | 7492 | 4.067833851 | 4.06406638 | 4.151652432 | 4.475831371 | 4.638447529 | 3.829555892 | 2.633393902 | 2.73239825 | 3.232470805 | 2.832063063 | 2.404052235 | 2.30206083 | 2.673352698 | 2.640841061 | 2.980678496 | 2.693426335 | 2.573161685 | 1.94573055 |
| ENSMUSG00000023806 | Rsph3b | 2231 | 5.715622057 | 6.107115076 | 5.442293195 | 7.699020042 | 6.903727285 | 6.307923055 | 6.319667557 | 6.094350182 | 6.165977707 | 6.55164411 | 6.402829487 | 5.629100888 | 5.528005074 | 5.287672881 | 5.466454748 | 5.166056848 | 4.638323695 | 5.646385226 |
| ENSMUSG00000023809 | Rps6ka2 | 5654 | 0.488651652 | 1.095896414 | 0.444892745 | 0.644655739 | 0.349028806 | 1.262043445 | 0.736384108 | 0.790327393 | 0.451638482 | 0.908989504 | 1.005521765 | 0.997060828 | 1.230244134 | 2.020736735 | 1.195092884 | 1.686303722 | 0.684640252 | 1.770721157 |
| ENSMUSG00000023826 | Park2 | 1590 | 1.684167352 | 3.058919847 | 2.73812436 | 3.925700047 | 3.602326363 | 3.397011296 | 5.058588313 | 4.707994259 | 3.367450974 | 3.706816294 | 3.33523295 | 4.282711217 | 2.513135408 | 4.001786146 | 4.379284871 | 4.431114316 | 5.76099683 | 5.397119905 |
| ENSMUSG00000023827 | Agpat4 | 2689 | 2.529129105 | 3.134310985 | 2.194708331 | 2.677071404 | 2.702833943 | 1.769082344 | 1.407591787 | 2.002651074 | 1.5010333 | 2.191832617 | 1.474645427 | 1.411478265 | 5.173522005 | 3.385288631 | 3.340252579 | 6.792346497 | 2.266225812 | 4.091417384 |
| ENSMUSG00000023828 | Slc22a3 | 3490 | 0 | 0 | 0 | 0 | 0 | 0 | 0 | 0.109433818 | 0 | 0 | 0 | 0 | 0 | 0 | 0 | 0 | 0 | 0 |
| ENSMUSG00000023829 | Slc22a1 | 1983 | 0 | 0 | 0 | 0 | 0 | 0 | 0 | 0 | 0 | 0 | 0 | 0 | 0 | 0 | 0 | 0.038618868 | 0 | 0 |

| ENSMUSG00000023830 | Igf2r |  | 9388 | 6.266208311 | 8.576615395 | 6.358429533 | 6.97877948 | 6.706066911 | 7.004311617 | 3.467311792 | 5.382248443 | 4.084431846 | 4.8516808 | 3.150053185 | 4.013162787 | 4.760834795 | 4.269412805 | 5.205053823 | 5.41648132 | 5.29497306 | 3.949311667 |
| --- | --- | --- | --- | --- | --- | --- | --- | --- | --- | --- | --- | --- | --- | --- | --- | --- | --- | --- | --- | --- | --- |
| ENSMUSG00000023832 | Acat2 |  | 3123 | 16.14189522 | 18.19782182 | 14.56007638 | 15.62468091 | 14.57982702 | 14.51833212 | 23.39121878 | 23.21139282 | 17.85672185 | 21.28802164 | 16.00147505 | 20.48182815 | 30.56588534 | 37.3426524 | 31.2540781 | 28.16315248 | 24.90043433 | 27.47813208 |
| ENSMUSG00000023845 | Lnpep |  | 5163 | 1.695926063 | 1.129141292 | 1.845745783 | 1.438398262 | 1.277178683 | 1.756372312 | 0.934707196 | 1.546041469 | 1.946447772 | 1.863014423 | 2.146773265 | 1.837819609 | 1.509673238 | 1.151431955 | 2.114741877 | 2.143324722 | 2.100782883 | 2.269405333 |
| ENSMUSG00000023846 | Riok2 |  | 3461 | 6.275684759 | 7.43067158 | 7.351770288 | 9.570320495 | 6.633610976 | 8.075048176 | 8.516555612 | 7.21694053 | 6.96160408 | 7.806227381 | 7.067546588 | 5.708969822 | 7.796752023 | 5.206676078 | 8.154598609 | 6.660198109 | 9.501289437 | 7.184084438 |
| ENSMUSG00000023852 | Chd1 |  | 9023 | 4.753155559 | 6.309623875 | 5.028745592 | 5.448339448 | 5.190331567 | 5.25018434 | 4.991873029 | 5.007382472 | 4.377465213 | 5.706369052 | 4.680601052 | 5.23948423 | 5.062767971 | 4.617128049 | 5.127933865 | 5.389457099 | 5.921185624 | 7.096375953 |
| ENSMUSG00000023861 | Brp44l |  | 1975 | 19.67074982 | 16.51307516 | 20.37811507 | 16.26356266 | 19.17965768 | 19.82109343 | 25.34522243 | 23.16683447 | 24.25312295 | 25.95079212 | 25.35096531 | 22.63713708 | 25.8024661 | 24.45663336 | 24.86696437 | 24.15701105 | 24.54823574 | 25.29026274 |
| ENSMUSG00000023868 | Pde10a |  | 12932 | 0.026294572 | 0.038640072 | 0 | 0.028184995 | 0 | 0.045981503 | 0 | 0 | 0 | 0.003646049 | 0.014777284 | 0 | 0.003814717 | 0.08619379 | 0.006372032 | 0.011843677 | 0 | 0 |
| ENSMUSG00000023873 | 1700010I14Ri | k | 2178 | 2.166241753 | 1.605993523 | 2.398687456 | 2.656681649 | 2.894987144 | 2.457160343 | 2.237468287 | 2.419904285 | 2.723075422 | 1.64529543 | 1.228373588 | 3.485275533 | 2.151759477 | 2.409633551 | 1.343117754 | 2.285481617 | 1.84768649 | 3.081318191 |
| ENSMUSG00000023882 | Zfp54 |  | 946 | 20.39888998 | 17.92416824 | 19.02214936 | 12.76287183 | 19.02890005 | 17.33821862 | 16.40439608 | 19.66141646 | 19.54835685 | 17.04602591 | 22.72593162 | 17.16951143 | 22.05856343 | 25.0385672 | 18.16178521 | 12.99290167 | 22.93095565 | 14.42099204 |
| ENSMUSG00000023883 | Phf10 |  | 5172 | 6.895174534 | 7.761414836 | 7.772313172 | 7.972301841 | 6.979687307 | 7.042006802 | 5.324048557 | 8.115516288 | 7.159063741 | 6.49097075 | 6.013437349 | 5.94633219 | 4.416217653 | 4.786291295 | 5.648086451 | 5.389706533 | 5.342857708 | 5.286064586 |
| ENSMUSG00000023885 | Thbs2 |  | 5904 | 0 | 0 | 0 | 0 | 0 | 0.075537704 | 0 | 0 | 0 | 0 | 0 | 0 | 0 | 0.094398552 | 0 | 0 | 0 | 0 |
| ENSMUSG00000023886 | Smoc2 |  | 2853 | 0.65553023 | 1.52961414 | 1.848129972 | 1.053988407 | 1.214685771 | 1.927919182 | 0.746256765 | 1.472542679 | 1.86227148 | 0.512328006 | 1.624315097 | 1.389033314 | 0.536028544 | 0.325580704 | 0.115531878 | 0.12079056 | 0.429877848 | 0.019281145 |
| ENSMUSG00000023892 | Zfp51 |  | 2595 | 10.30279604 | 7.586867238 | 5.499110387 | 7.233583918 | 8.99575452 | 8.382909457 | 6.417750668 | 8.668718696 | 7.189784832 | 7.5041389 | 8.247854103 | 6.022499239 | 6.007277788 | 6.478905447 | 8.049783752 | 9.768162659 | 9.880652265 | 8.352057052 |
| ENSMUSG00000023902 | Zscan10 |  | 2774 | 0 | 0.024017948 | 0.017438187 | 0 | 0.19086199 | 0.142906225 | 0 | 0.041303968 | 0.07423669 | 0 | 0.017222408 | 0.040242006 | 0 | 0 | 0 | 0 | 0 | 0 |
| ENSMUSG00000023903 | Mmp25 |  | 3363 | 0 | 0.009905707 | 0.043152123 | 0.013547738 | 0 | 0.058938726 | 0 | 0 | 0 | 0 | 0 | 0 | 0 | 0 | 0 | 0 | 0 | 0 |
| ENSMUSG00000023904 | Hcfc1r1 |  | 1240 | 101.0183498 | 76.94203955 | 89.02290022 | 76.71891982 | 84.8519235 | 75.84765188 | 129.8809347 | 109.7107559 | 104.7598957 | 113.1236631 | 114.6984285 | 107.8052515 | 140.1583424 | 135.8489556 | 124.9337501 | 136.9506805 | 118.0079045 | 135.393382 |
| ENSMUSG00000023905 | Tnfrsf12a |  | 959 | 266.3775891 | 251.7398795 | 212.6619437 | 218.2559308 | 239.2543067 | 252.4657521 | 76.02590013 | 118.9181581 | 94.57008103 | 94.00640733 | 99.73458485 | 112.5043543 | 100.4642695 | 90.80569745 | 77.80606644 | 173.6453093 | 117.2169226 | 103.708514 |
| ENSMUSG00000023906 | Cldn6 |  | 1503 | 89.19582514 | 77.26463689 | 76.05233016 | 84.21063284 | 76.56914803 | 100.4572051 | 26.88291936 | 23.0475775 | 30.22536719 | 26.82225634 | 26.3509252 | 30.67447591 | 12.5381187 | 10.0428 | 14.58365119 | 19.15804176 | 14.40742365 | 15.66460258 |
| ENSMUSG00000023908 | Pkmyt1 |  | 2031 | 3.369440345 | 4.756641715 | 3.048651839 | 5.428740881 | 4.55013754 | 4.416073253 | 4.472690475 | 4.494329973 | 3.285187372 | 4.248438551 | 6.068901668 | 3.297823491 | 4.493552288 | 5.465355434 | 4.868721523 | 4.449327103 | 4.019449158 | 2.735558729 |
| ENSMUSG00000023909 | Paqr4 |  | 2504 | 5.448946288 | 6.77167059 | 5.158040178 | 6.877825383 | 3.709848749 | 6.213881128 | 3.778967965 | 3.797886661 | 4.408141463 | 4.330935203 | 2.938236264 | 4.168342214 | 1.792813288 | 3.190248814 | 1.184709275 | 2.400808851 | 2.739779212 | 1.471889038 |
| ENSMUSG00000023911 | Flywch2 |  | 1132 | 16.63409274 | 16.4210203 | 13.16170261 | 17.06526749 | 17.17782435 | 15.67127087 | 21.77553509 | 16.66700246 | 18.6285301 | 17.91060283 | 16.4595705 | 21.39929113 | 21.74613668 | 22.40147678 | 15.94194477 | 17.5216737 | 18.14743387 | 21.33303697 |
| ENSMUSG00000023912 | Slc25a27 |  | 2836 | 4.166586367 | 3.230270067 | 3.394334464 | 3.646811377 | 2.664567001 | 2.323876434 | 3.586821569 | 4.767316802 | 4.400392904 | 3.923683346 | 4.211473773 | 2.912806087 | 3.722507157 | 3.504596157 | 4.213135123 | 3.766953267 | 5.446226252 | 5.760826762 |
| ENSMUSG00000023913 | Pla2g7 |  | 2648 | 1.637283971 | 0.440313934 | 1.205684669 | 1.032349947 | 0.98154225 | 0.823383791 | 0.893366294 | 1.096156564 | 1.835350778 | 0.819083214 | 0.39692186 | 1.201470078 | 1.285461591 | 2.36780657 | 1.960496995 | 0.17352239 | 0.723683854 | 0.768631778 |
| ENSMUSG00000023914 | Mep1a |  | 4113 | 0.020668697 | 0.04859649 | 0 | 0.022154653 | 0 | 0 | 1.575938977 | 1.504295937 | 1.381896225 | 1.043207877 | 0.278774375 | 1.547042435 | 3.250413222 | 5.465338754 | 3.265671583 | 0.660985443 | 0.950469799 | 2.688264151 |
| ENSMUSG00000023915 | Tnfrsf21 |  | 3620 | 223.7744875 | 179.4018986 | 201.5385593 | 265.8902272 | 207.2730823 | 249.2283481 | 310.028745 | 233.7332826 | 236.7542007 | 317.3033928 | 288.5237962 | 285.8933745 | 165.7797717 | 149.8784058 | 176.2333954 | 158.4088775 | 193.7277847 | 174.8894501 |
| ENSMUSG00000023918 | Gpr115 |  | 3736 | 0.056885942 | 0 | 0 | 0 | 0 | 0 | 0 | 0 | 0 | 0.126206379 | 0 | 0 | 0.118840272 | 0 | 0 | 0 | 0.041034579 | 0.103068455 |
| ENSMUSG00000023919 | Cenpq |  | 2594 | 2.408735881 | 4.353535521 | 3.636406484 | 6.164967834 | 4.304782687 | 4.508267203 | 5.61769665 | 4.902879723 | 6.20814482 | 4.780506922 | 6.114605326 | 5.228683889 | 4.868531543 | 4.297062827 | 5.162107041 | 3.498409389 | 6.427116096 | 6.383092183 |
| ENSMUSG00000023921 | Mut |  | 3284 | 20.12653737 | 22.25593481 | 24.93799844 | 24.37599114 | 20.81222034 | 28.7297213 | 35.4845092 | 27.20220135 | 34.62727467 | 30.03631888 | 31.97605321 | 35.72610315 | 31.14039723 | 27.09709849 | 27.33806038 | 27.37708471 | 27.07582485 | 31.05568936 |
| ENSMUSG00000023923 | Tbc1d5 |  | 8068 | 1.622656695 | 2.849020453 | 2.338333768 | 2.586385511 | 2.815848782 | 2.413761068 | 1.636122635 | 2.418978387 | 1.643785086 | 2.524678211 | 1.859362539 | 1.390548857 | 2.341859732 | 2.348684637 | 1.629064989 | 1.988567737 | 2.731484968 | 2.420455241 |
| ENSMUSG00000023926 | Rhag |  | 2329 | 0.054751193 | 0.042910555 | 0 | 0.097812461 | 0.041332695 | 0.042552798 | 0.365662611 | 0.213182152 | 0.300631502 | 0.222695464 | 0.246156935 | 0.335517079 | 0.190634287 | 0.219358077 | 0.053071991 | 0.213730312 | 0.148105055 | 0.070857587 |
| ENSMUSG00000023927 | Satb1 |  | 15177 | 1.971644193 | 1.942538801 | 1.877314975 | 2.167429269 | 2.270702863 | 1.985113133 | 1.546227103 | 2.035820883 | 1.989175466 | 2.202665125 | 1.781684556 | 1.813080428 | 3.305696769 | 4.330130043 | 3.659464805 | 3.827294685 | 3.035396249 | 3.211311099 |
| ENSMUSG00000023930 | Crisp2 |  | 1814 | 0.164022179 | 0.055092989 | 0.026666775 | 0.200930736 | 0.026533585 | 0.24585149 | 0.156491772 | 0.336867937 | 0.613029727 | 0.675809418 | 0.026336802 | 0.30769384 | 0.027195103 | 0.358443894 | 0.022713096 | 0.464384434 | 0.823994255 | 0.424546579 |
| ENSMUSG00000023931 | Efhb |  | 2648 | 0.032103607 | 0.113223583 | 0 | 0.13764666 | 0.2181205 | 0.112279608 | 0.464550473 | 0.057692451 | 0 | 0.089030784 | 0.126293319 | 0 | 0.316707928 | 0.333246851 | 0 | 0.057840797 | 0.318420896 | 0.020773832 |
| ENSMUSG00000023932 | Cdc5l |  | 2973 | 26.44957136 | 21.53628748 | 23.49524986 | 23.0487086 | 21.20848302 | 19.80109233 | 24.96925437 | 21.54344394 | 22.01324371 | 24.28110551 | 20.7940788 | 23.43018732 | 19.71285494 | 21.12089006 | 25.40277563 | 21.89506646 | 21.96704 | 21.55587263 |
| ENSMUSG00000023935 | Spats1 |  | 1369 | 0.186290034 | 0.55967609 | 0.38868432 | 0.432646878 | 0.105475362 | 0.180981496 | 0.138240114 | 0.446368472 | 0.541531982 | 0.275533693 | 0.6630564 | 0.978506868 | 0.252245012 | 0.949915593 | 0.57182583 | 0.755183638 | 0.531920848 | 1.044731026 |
| ENSMUSG00000023938 | Aars2 |  | 3326 | 4.664578849 | 4.677426819 | 3.999615408 | 5.739650504 | 5.889865584 | 5.795554228 | 3.257547647 | 5.305138278 | 5.993467718 | 4.777446482 | 6.305834868 | 5.420462723 | 4.597983871 | 4.636030371 | 4.174661556 | 3.016277552 | 2.604252285 | 3.78745804 |
| ENSMUSG00000023939 | Mrpl14 |  | 1015 | 80.61326488 | 70.85964396 | 73.68027382 | 60.822872 | 69.47120017 | 64.29650258 | 77.51821188 | 75.14308138 | 71.98510243 | 72.79325321 | 70.03855982 | 69.12343633 | 83.15951769 | 79.61843552 | 78.18147453 | 74.0913822 | 60.86895626 | 78.04247653 |
| ENSMUSG00000023940 | Sgol1 |  | 3801 | 0 | 0 | 0 | 0 | 0 | 0 | 0.099579435 | 0 | 0 | 0.086833708 | 0 | 0 | 0 | 0.232159342 | 0 | 0 | 0.191581068 | 0 |
| ENSMUSG00000023942 | Slc29a1 |  | 3962 | 5.954157672 | 7.651371464 | 6.446548215 | 7.014698901 | 8.175866893 | 7.116482939 | 10.10260635 | 8.087689454 | 7.817329594 | 11.3651998 | 9.694867899 | 7.494682609 | 16.02477979 | 18.19314074 | 21.93187314 | 19.44490209 | 20.48841414 | 18.31322861 |
| ENSMUSG00000023943 | Sult1c1 |  | 1548 | 0 | 0 | 0 | 0 | 0 | 0 | 0 | 0 | 0 | 0 | 0 | 0 | 0 | 0 | 0 | 0 | 0 | 0 |
| ENSMUSG00000023944 | Hsp90ab1 |  | 2730 | 432.9611483 | 391.286178 | 381.21177 | 389.3216272 | 386.9066172 | 375.7658207 | 333.8230115 | 307.9175007 | 324.8001824 | 330.6248764 | 309.1022314 | 317.0247854 | 411.6414196 | 392.5971446 | 349.6397236 | 374.4625042 | 337.2714127 | 382.3435165 |

| ENSMUSG00000023945 | Slc5a7 | 5167 | 0 | 0 | 0 | 0 | 0 | 0 | 0 | 0 | 0 | 0 | 0 | 0 | 0 | 0 | 0 | 0 | 0 | 0 |
| --- | --- | --- | --- | --- | --- | --- | --- | --- | --- | --- | --- | --- | --- | --- | --- | --- | --- | --- | --- | --- |
| ENSMUSG00000023947 | Nfkbie | 2416 | 24.6304828 | 28.92816725 | 27.7306869 | 31.94553357 | 30.32151814 | 29.20657807 | 17.09601354 | 27.55354197 | 22.67304055 | 21.76036181 | 26.12198682 | 24.14212227 | 11.18952428 | 14.28309476 | 10.89726582 | 21.33243277 | 14.3089072 | 10.90619293 |
| ENSMUSG00000023949 | Tcte1 | 3069 | 12.81110714 | 16.06486916 | 14.21731 | 12.03975463 | 13.55033628 | 11.85132175 | 4.162405771 | 6.595625049 | 5.609632971 | 3.28779749 | 4.312044151 | 5.910749541 | 0.546524988 | 0.332932526 | 0.201376128 | 0.536492707 | 0.711827606 | 0.645268114 |
| ENSMUSG00000023951 | Vegfa | 6165 | 10.12815954 | 7.213741084 | 10.6869016 | 7.175956861 | 9.228213102 | 9.171073644 | 21.39622852 | 15.84690436 | 19.52100552 | 18.06487609 | 19.55169834 | 19.1755999 | 14.72355682 | 8.16632453 | 13.24598277 | 10.42821246 | 12.93706794 | 14.05342143 |
| ENSMUSG00000023952 | Gtpbp2 | 5507 | 9.678861583 | 12.50972676 | 9.082663953 | 9.141992735 | 11.53697825 | 9.52902576 | 8.058705799 | 9.251618814 | 10.68740349 | 8.998617963 | 9.829131643 | 10.91582869 | 8.187647066 | 10.08664037 | 8.828370429 | 10.63821124 | 9.485880276 | 9.559418038 |
| ENSMUSG00000023953 | Polh | 3429 | 1.01645507 | 0.301166439 | 0.282143659 | 0.624487922 | 0.196514124 | 0.462434376 | 0.055191227 | 0.723973799 | 1.333246792 | 0.72878019 | 0.431911261 | 0.488326007 | 0.791267265 | 0.433423971 | 0.492640359 | 0.256834054 | 0.838282959 | 0.930454415 |
| ENSMUSG00000023959 | Clic5 | 5900 | 0.136881075 | 0.378298968 | 0.090187938 | 0.185333062 | 0.203948829 | 0.587913788 | 0.008019098 | 0.129465771 | 0.286211381 | 0.119874669 | 0.429164881 | 0.245967327 | 0.242478915 | 0.125950067 | 0 | 0.064899335 | 0.02598393 | 0 |
| ENSMUSG00000023960 | Enpp5 | 4824 | 250.9602868 | 142.6363661 | 150.3149295 | 288.827176 | 218.2698927 | 218.9503558 | 263.2993737 | 173.797599 | 182.231549 | 306.7825711 | 230.5557698 | 224.4196914 | 236.8422894 | 127.9523054 | 145.213278 | 208.8758984 | 243.6150999 | 186.2601056 |
| ENSMUSG00000023961 | Enpp4 | 7866 | 11.56382872 | 10.71890863 | 11.7828228 | 13.35090353 | 12.02992143 | 12.57402189 | 12.61912031 | 12.41034586 | 10.76525253 | 13.60093385 | 14.16971499 | 14.02842168 | 14.7694718 | 10.86411401 | 10.88441815 | 18.91164629 | 15.2554838 | 12.04941405 |
| ENSMUSG00000023963 | Cyp39a1 | 3325 | 23.54722834 | 29.16506318 | 28.19485769 | 27.73400112 | 26.1577101 | 27.91346466 | 25.64133757 | 26.77488421 | 26.74336467 | 32.78569201 | 30.14492117 | 28.33589126 | 26.17188032 | 24.24870247 | 31.66014295 | 23.80351444 | 27.88310142 | 28.86944097 |
| ENSMUSG00000023964 | Calcr | 5864 | 0.913310399 | 0.551048898 | 0.461957316 | 0.846888443 | 1.058836656 | 0.684476709 | 1.645939035 | 2.025552038 | 1.98768485 | 2.854450828 | 1.727198362 | 1.951263784 | 0.597299817 | 0.855382237 | 0.597225827 | 1.083942843 | 1.163383501 | 0.412755917 |
| ENSMUSG00000023965 | Fbxl17 | 8141 | 6.025177882 | 7.140523328 | 5.787473107 | 6.665422406 | 7.047445401 | 7.164177303 | 6.474183064 | 6.703960574 | 5.398109854 | 6.926942771 | 6.918889086 | 8.631853482 | 5.52643515 | 5.562336962 | 6.351548046 | 5.855767868 | 6.694508561 | 6.554334052 |
| ENSMUSG00000023966 | Rsph9 | 1596 | 12.9699378 | 11.31302548 | 12.18431028 | 10.04855113 | 11.12824551 | 10.7736833 | 11.62065798 | 12.87438126 | 13.05788031 | 14.35792092 | 13.20097537 | 13.35938791 | 14.28029184 | 7.507878788 | 10.09386488 | 13.50727567 | 11.0704404 | 11.27066284 |
| ENSMUSG00000023967 | Mrps18a | 3522 | 27.90231885 | 23.74087578 | 23.85713294 | 24.43634661 | 28.17945105 | 25.15624294 | 29.72826759 | 26.40502551 | 29.41058692 | 27.93966999 | 30.76479439 | 26.70340473 | 31.99151024 | 32.95393935 | 27.42091056 | 29.41918899 | 27.87960609 | 27.81692762 |
| ENSMUSG00000023968 | Crip3 | 1468 | 0 | 0 | 0.131807985 | 0 | 0 | 0 | 0 | 0 | 0 | 0 | 0 | 0 | 0 | 0 | 0 | 0 | 0 | 0 |
| ENSMUSG00000023971 | Rrp36 | 1220 | 39.36954829 | 30.41849521 | 32.63230777 | 32.49025293 | 32.70603666 | 32.73729776 | 30.21030892 | 27.83036536 | 29.67454701 | 36.21328603 | 31.28868182 | 34.45015896 | 33.76405814 | 30.41707035 | 35.19018134 | 32.9864167 | 27.45670778 | 35.98136653 |
| ENSMUSG00000023972 | Ptk7 | 4688 | 19.33949242 | 25.91555572 | 24.51695992 | 27.83422162 | 23.9735584 | 28.423059 | 14.68428922 | 22.71339972 | 22.78085225 | 24.29950919 | 24.30530631 | 24.66937988 | 23.81359403 | 19.07100431 | 24.03717051 | 25.67136925 | 23.97846178 | 26.19034262 |
| ENSMUSG00000023973 | Cnpy3 | 3804 | 21.31963088 | 19.89666027 | 20.19378712 | 22.90029358 | 20.40925154 | 19.78722458 | 25.67123273 | 23.34314816 | 22.7153812 | 25.7072972 | 30.631736 | 28.31867213 | 25.2106328 | 24.80924963 | 26.01633471 | 25.38614928 | 21.78271659 | 25.75478945 |
| ENSMUSG00000023977 | Ubr2 | 7699 | 8.932767218 | 8.671131318 | 9.562867022 | 9.279090469 | 8.589851035 | 8.128991104 | 7.110114238 | 10.32320943 | 8.366763301 | 8.4637319 | 11.1944441 | 8.953415157 | 9.976593739 | 8.680743181 | 9.595322732 | 9.355063323 | 8.253669342 | 9.367052723 |
| ENSMUSG00000023978 | Prph2 | 3798 | 0.011191463 | 0 | 0 | 0 | 0 | 0 | 0 | 0 | 0 | 0.074487683 | 0 | 0 | 0 | 0 | 0 | 0 | 0 | 0.130353333 |
| ENSMUSG00000023979 | Guca1b | 1716 | 0.049539832 | 0.349435953 | 0 | 0.106202901 | 0.140244533 | 0.057753769 | 0 | 0 | 0 | 0.274770998 | 0.194886194 | 0 | 0.172489221 | 0.216522552 | 0 | 0.044627748 | 0.022334672 | 0 |
| ENSMUSG00000023980 | Taf8 | 2829 | 8.774486674 | 11.16317558 | 10.75537313 | 8.664489874 | 10.08915897 | 8.442706817 | 10.23519247 | 11.23226539 | 13.49589965 | 11.78350909 | 14.810406 | 10.5159944 | 9.451360609 | 11.83675751 | 13.79210796 | 9.136147047 | 9.794949686 | 5.911194218 |
| ENSMUSG00000023982 | Guca1a | 867 | 0 | 0 | 0.055794153 | 0 | 0.055515483 | 0.057154249 | 0 | 0 | 0.095009264 | 0 | 0 | 0.128755854 | 0 | 0 | 0 | 0 | 0 | 0.19034293 |
| ENSMUSG00000023984 | Gm20517 | 6579 | 0.090450291 | 0.030381116 | 0.014705436 | 0 | 0.09510792 | 0.067787597 | 0.028765879 | 0.023220795 | 0 | 0.043001097 | 0 | 0.127258693 | 0 | 0.049416114 | 0 | 0.174603773 | 0.058255505 | 0 |
| ENSMUSG00000023987 | Pgc | 1500 | 0 | 0 | 0 | 0 | 0 | 0 | 0 | 0.076384805 | 0 | 0 | 0.031849972 | 0.037210442 | 0.032887945 | 0 | 0 | 0 | 0 | 0 |
| ENSMUSG00000023988 | Bysl | 3847 | 5.380821608 | 5.60266248 | 5.457268566 | 4.74914967 | 5.542615593 | 5.178112529 | 6.346072869 | 5.390817392 | 5.974025421 | 4.400078625 | 5.576021928 | 5.571410042 | 5.95009348 | 7.026373502 | 6.115437591 | 5.295191874 | 5.519305559 | 5.59099577 |
| ENSMUSG00000023990 | Tfeb | 5648 | 15.5481044 | 15.47091387 | 15.05677521 | 18.82781117 | 13.53284256 | 15.71334027 | 18.78098905 | 17.56796414 | 18.01180842 | 19.45133474 | 15.919347 | 16.8790991 | 11.45080445 | 8.478019493 | 10.59947959 | 10.88109506 | 10.3890865 | 10.07071819 |
| ENSMUSG00000023991 | Foxp4 | 5498 | 6.725991841 | 8.143421213 | 9.185515768 | 9.861339171 | 8.780705609 | 9.084968252 | 7.306013898 | 8.460957826 | 6.966798774 | 8.919012621 | 6.178245813 | 8.172355108 | 6.128355675 | 5.980795539 | 6.93936718 | 6.678918243 | 5.618587724 | 5.773054671 |
| ENSMUSG00000023992 | Trem2 | 3234 | 0 | 0 | 0 | 0 | 0 | 0 | 0 | 0 | 0.012735472 | 0 | 0 | 0 | 0 | 0 | 0 | 0 | 0 | 0 |
| ENSMUSG00000023993 | Treml1 | 1242 | 0 | 0 | 0 | 0 | 0 | 0 | 0 | 0 | 0 | 0 | 0 | 0 | 0 | 0 | 0 | 0 | 0.061717064 | 0 |
| ENSMUSG00000023994 | Nfya | 8289 | 0.866615364 | 0.28132496 | 0.501884861 | 0.456215066 | 0.632932764 | 0.573900642 | 0.542249306 | 0.44693727 | 0.337879487 | 0.602964718 | 0.541783822 | 0.424223277 | 0.517779805 | 0.717196669 | 0.407591698 | 0.660581115 | 0.568721742 | 0.497729883 |
| ENSMUSG00000023995 | Tspo2 | 4104 | 0 | 0 | 0 | 0 | 0 | 0 | 0 | 0 | 0 | 0 | 0 | 0 | 0 | 0 | 0 | 0 | 0 | 0 |
| ENSMUSG00000023999 | Kif6 | 6157 | 0.041421318 | 0.064926869 | 0 | 0.066598895 | 0.046904587 | 0.024144584 | 0 | 0.043421604 | 0.040136283 | 0.030632258 | 0 | 0 | 0.128197284 | 0.075432983 | 0.133836465 | 0.186571093 | 0.130721493 | 0.134016014 |
| ENSMUSG00000024002 | Brd4 | 9921 | 18.45275609 | 21.06358083 | 18.78186059 | 19.09513379 | 19.83301118 | 19.64927466 | 16.03795376 | 19.76411591 | 16.02874079 | 20.14158657 | 17.39372543 | 16.38857223 | 17.37879759 | 18.41659508 | 17.51304921 | 19.51003129 | 19.57071062 | 18.80766959 |
| ENSMUSG00000024006 | Stk38 | 8421 | 16.40949142 | 15.06022899 | 17.91101623 | 16.69651856 | 14.72936317 | 17.18251776 | 14.55169676 | 13.75579581 | 11.02904561 | 14.97221001 | 13.56488846 | 13.19664935 | 13.54416254 | 13.34143776 | 11.22388899 | 12.06329194 | 13.18504716 | 11.14422706 |
| ENSMUSG00000024007 | Ppil1 | 2657 | 61.52612232 | 48.57137832 | 52.61554487 | 53.56895089 | 46.12114327 | 36.34861789 | 63.01828029 | 52.52353722 | 56.03660312 | 66.06777126 | 55.36285177 | 48.23212695 | 68.73419562 | 73.17085051 | 62.6474542 | 69.5485402 | 55.83782285 | 50.16449589 |
| ENSMUSG00000024008 | Cpne5 | 13296 | 0.169432486 | 0.130285086 | 0.192824692 | 0.092520171 | 0.188241579 | 0.260882322 | 0.811318501 | 0.706628384 | 0.765122548 | 0.482287578 | 0.276675075 | 0.818596135 | 1.93676751 | 2.095852319 | 1.375864231 | 0.524134367 | 0.52750544 | 1.601122466 |
| ENSMUSG00000024011 | Pi16 | 4287 | 0.099148999 | 0 | 0 | 0.021255444 | 0.033682242 | 0 | 0.011036314 | 0.160359982 | 0.038429219 | 0.076989718 | 0 | 0 | 0.138087942 | 0 | 0.048054066 | 0.071454364 | 0.071520965 | 0.076989653 |
| ENSMUSG00000024012 | Mtch1 | 3041 | 184.8227368 | 175.1966844 | 161.2344964 | 184.3267111 | 169.2293745 | 184.0017979 | 183.7586487 | 173.8062009 | 168.1726295 | 174.2296786 | 164.4394904 | 178.2577162 | 192.7367672 | 190.6635932 | 169.0605111 | 155.5548056 | 155.8762381 | 177.9429736 |
| ENSMUSG00000024013 | Fgd2 | 4344 | 0.851277697 | 0.812883699 | 1.013349738 | 1.216639305 | 0.819926876 | 1.163531037 | 2.875356178 | 1.450678268 | 1.393742591 | 1.769236794 | 2.980435946 | 1.914487506 | 3.293337019 | 1.881712565 | 1.451159772 | 1.762919309 | 0.926395298 | 0.975069341 |
| ENSMUSG00000024014 | Pim1 | 1966 | 1.318827944 | 2.660287076 | 2.3620849 | 1.784435612 | 1.860644048 | 2.495280073 | 2.743461545 | 3.030526335 | 2.346332536 | 2.206441861 | 3.304880139 | 3.208123028 | 1.380089242 | 1.322924158 | 1.676563821 | 2.960413187 | 2.066422926 | 3.385606263 |

| ENSMUSG00000024018 | 1110021J02R | 4889 | 1.217166014 | 0.878986164 | 1.167534584 | 0.857356531 | 0.826975166 | 1.206131171 | 0.812899372 | 0.874933334 | 1.086737683 | 1.369482484 | 1.11399985 | 1.31290677 | 0.84759277 | 0.62698093 | 0.859594743 | 0.939839004 | 1.034786499 | 1.001393025 |
| --- | --- | --- | --- | --- | --- | --- | --- | --- | --- | --- | --- | --- | --- | --- | --- | --- | --- | --- | --- | --- |
| ENSMUSG00000024019 | Ftsjd2 | 8058 | 1.144654157 | 1.161693134 | 1.446763566 | 1.4418052 | 1.206583343 | 1.334443185 | 1.033386882 | 0.981115326 | 1.042696602 | 1.018146236 | 1.44071915 | 1.122131712 | 1.07136827 | 0.835739969 | 0.961267375 | 0.955126841 | 1.032117948 | 1.153703033 |
| ENSMUSG00000024026 | Glo1 | 2137 | 43.06209925 | 36.41503084 | 45.56664324 | 39.12237546 | 45.47419461 | 58.45692155 | 37.32764468 | 35.24352716 | 40.22286308 | 37.66319628 | 41.58232231 | 53.83064136 | 36.65843923 | 34.92543212 | 41.27867626 | 35.60291853 | 45.67949378 | 52.51220296 |
| ENSMUSG00000024027 | Glp1r | 1579 | 0 | 0 | 0 | 0 | 0 | 0 | 0 | 0 | 0.026083924 | 0 | 0 | 0.070697483 | 0 | 0 | 0.052186898 | 0 | 0 | 0 |
| ENSMUSG00000024028 | Tff2 | 580 | 0 | 0 | 0 | 0 | 0 | 0 | 0.326294337 | 0 | 0 | 0 | 0 | 0.096233901 | 0.17011006 | 0.240228038 | 0.568297323 | 0.132036577 | 0.462558756 | 0 |
| ENSMUSG00000024029 | Tff3 | 490 | 0 | 0 | 0 | 0 | 0 | 0 | 0 | 0 | 0 | 0 | 0 | 0 | 0 | 0 | 0 | 0 | 0 | 0 |
| ENSMUSG00000024030 | Abcg1 | 4133 | 7.435577611 | 8.729219559 | 8.965430499 | 7.870937734 | 9.211795698 | 10.29900754 | 7.498138083 | 5.18411269 | 6.357850727 | 5.30488193 | 5.109250338 | 7.508712412 | 5.430927259 | 4.405052566 | 7.30722005 | 5.827435774 | 7.047661661 | 4.63178542 |
| ENSMUSG00000024032 | Tff1 | 475 | 0 | 0 | 0 | 0 | 0 | 0 | 0 | 0 | 0 | 0 | 0 | 0 | 0 | 0 | 0 | 0 | 0 | 0 |
| ENSMUSG00000024033 | Rsph1 | 1350 | 4.533885443 | 3.380641858 | 3.941546918 | 2.699913738 | 2.103543331 | 3.193398386 | 6.448542906 | 3.28171754 | 4.881364831 | 3.283086005 | 3.857385536 | 3.969113784 | 7.162263545 | 5.401275348 | 4.15067526 | 2.325799857 | 4.230087584 | 4.60446597 |
| ENSMUSG00000024034 | Tmprss3 | 2866 | 0 | 0 | 0 | 0 | 0 | 0 | 0 | 0 | 0 | 0 | 0 | 0 | 0 | 0 | 0 | 0 | 0 | 0 |
| ENSMUSG00000024036 | Slc37a1 | 3605 | 3.8437413 | 4.990003572 | 4.790388443 | 5.459742345 | 4.499433641 | 4.907163908 | 5.512156768 | 6.377760402 | 5.860938304 | 6.971241286 | 6.400916767 | 5.357064981 | 5.117929837 | 7.910310584 | 5.543066465 | 5.799354131 | 6.081176651 | 10.34567943 |
| ENSMUSG00000024037 | Wdr4 | 7758 | 2.481934099 | 2.33593896 | 1.633651064 | 1.908654216 | 1.811616617 | 1.596826939 | 2.09180831 | 1.634426089 | 1.49180342 | 2.011714717 | 2.666480668 | 2.273491799 | 1.735964606 | 1.927687051 | 1.651673612 | 1.993993992 | 1.383264132 | 1.524485427 |
| ENSMUSG00000024038 | Ndufv3 | 2087 | 52.50519588 | 46.44969917 | 46.40335782 | 47.0455441 | 52.81365846 | 50.7873968 | 70.68564106 | 54.95533513 | 64.27622513 | 66.21883625 | 62.37985714 | 60.5761743 | 85.47398796 | 80.9822875 | 69.33390718 | 67.82960017 | 66.05639193 | 69.90136609 |
| ENSMUSG00000024039 | Cbs | 3975 | 0.342180033 | 0.821299027 | 0.23121939 | 0.137542775 | 0.399585781 | 0.162059254 | 4.68960893 | 3.4108938 | 3.056609346 | 2.170711622 | 0.733150306 | 2.31687656 | 19.85687236 | 34.40951883 | 14.29357121 | 3.03435002 | 5.31265147 | 15.34719481 |
| ENSMUSG00000024041 | Cryaa | 1182 | 0 | 0.056366995 | 0 | 0 | 0 | 0 | 0 | 0 | 0 | 0 | 0 | 0 | 0 | 0 | 0 | 0 | 0 | 0 |
| ENSMUSG00000024042 | Sik1 | 4497 | 20.96430519 | 24.51983097 | 20.771467 | 27.14210313 | 24.306782 | 23.17309289 | 17.0123642 | 22.41266399 | 18.50050297 | 22.9305288 | 21.30059857 | 20.97586608 | 14.4803493 | 13.18859231 | 11.87396407 | 20.63963361 | 22.65316815 | 19.33942578 |
| ENSMUSG00000024043 | Arhgap28 | 5904 | 18.20004827 | 22.89697235 | 20.74555875 | 17.06995767 | 20.07127305 | 17.68421573 | 15.98726193 | 21.70316905 | 17.23778463 | 20.17321755 | 20.48076218 | 17.16823226 | 28.21712138 | 30.46713253 | 31.43851783 | 36.60436792 | 34.30800798 | 31.07306418 |
| ENSMUSG00000024044 | Epb4.1l3 | 5813 | 0.116993431 | 0.143268941 | 0.798874749 | 0.266484691 | 0.422282489 | 0.272782982 | 0 | 0.354789219 | 0.014170485 | 0.616455092 | 0.147935533 | 0.297657929 | 0.772269821 | 2.293041989 | 0.538675081 | 0.441333338 | 0.323067013 | 0.681344518 |
| ENSMUSG00000024045 | Akap8 | 3681 | 40.43823049 | 40.0098629 | 42.39418879 | 42.77613943 | 41.22791506 | 41.46221663 | 34.83220861 | 36.23142331 | 32.99620619 | 36.8008613 | 35.19850242 | 39.31812363 | 34.26832721 | 35.76989616 | 36.15349783 | 29.72957239 | 35.90031832 | 40.03515265 |
| ENSMUSG00000024048 | Myl12a | 3570 | 162.7460087 | 162.206734 | 153.5622464 | 142.4133596 | 167.5988636 | 152.4058865 | 105.0951103 | 115.3260779 | 135.0271654 | 103.2428704 | 107.2460833 | 119.6362605 | 110.7411723 | 97.45452623 | 105.1159023 | 125.7476418 | 112.1339415 | 109.4480911 |
| ENSMUSG00000024049 | Myom1 | 5694 | 0.306061155 | 0.228170508 | 0.161415012 | 0.288057182 | 0.228233568 | 0.174052454 | 0.232658063 | 0.603673378 | 0.622065394 | 0.207019245 | 0.125856055 | 0.264668579 | 0.008663842 | 0.236543473 | 0.151955159 | 0.154668769 | 0.343280847 | 0.173896017 |
| ENSMUSG00000024050 | Wiz | 10318 | 5.194710891 | 6.970589128 | 6.296341468 | 6.411575536 | 6.148272475 | 7.352707415 | 5.392489864 | 6.126034695 | 5.312972716 | 6.041212416 | 5.213665754 | 4.798264462 | 4.671184646 | 4.168174542 | 4.895629727 | 4.883740971 | 5.088876406 | 4.393051361 |
| ENSMUSG00000024052 | Lpin2 | 11448 | 9.252524341 | 9.241942002 | 8.911580671 | 10.53459856 | 8.825026885 | 9.293301799 | 9.034374226 | 9.534755946 | 10.39016925 | 9.847775287 | 11.32610389 | 9.648776752 | 10.15251547 | 10.75094615 | 11.8623627 | 9.07428528 | 12.99304318 | 11.63802031 |
| ENSMUSG00000024053 | Emilin2 | 5060 | 0 | 0 | 0 | 0 | 0 | 0 | 0 | 0.037739528 | 0 | 0.046591604 | 0 | 0 | 0.009749391 | 0.009178673 | 0.374559599 | 0 | 0 | 0 |
| ENSMUSG00000024054 | Smchd1 | 8402 | 5.559770109 | 4.341540011 | 6.557659019 | 4.538751977 | 6.312946903 | 5.384628155 | 4.8990753 | 5.691131613 | 5.446110332 | 5.028211154 | 5.816922462 | 6.370771295 | 5.196232658 | 3.963390938 | 4.874356886 | 5.564488412 | 5.291419234 | 4.884169676 |
| ENSMUSG00000024055 | Cyp4f13 | 3534 | 20.29038822 | 17.89130538 | 20.05297735 | 21.15610463 | 21.24668956 | 18.36844397 | 20.34953932 | 20.14449295 | 18.3090029 | 21.1737878 | 22.04894094 | 20.6742225 | 18.8868376 | 16.97955883 | 19.38828168 | 20.15295126 | 22.24313383 | 21.63629268 |
| ENSMUSG00000024056 | Ndc80 | 2184 | 0.642248539 | 0.442341544 | 0.664471571 | 0.146028988 | 0.308537972 | 0.38571267 | 1.559758646 | 0.69949455 | 0.886339854 | 0.410193847 | 1.509373688 | 0.332236087 | 0.293642365 | 1.063280389 | 0.433899169 | 1.262327717 | 0.579106135 | 0.906743517 |
| ENSMUSG00000024059 | Clip4 | 4445 | 5.020296381 | 5.403508826 | 5.267219053 | 5.360725801 | 5.273396361 | 5.183806775 | 4.928182305 | 6.719113314 | 6.198825432 | 5.855385421 | 4.933342169 | 7.207691862 | 5.826604403 | 4.586941364 | 5.709821919 | 4.89292801 | 6.380530868 | 5.865987984 |
| ENSMUSG00000024063 | Lbh | 3203 | 5.8124468 | 6.42752689 | 5.754079009 | 5.490653485 | 6.837347883 | 6.033582921 | 5.908545604 | 7.702869795 | 6.287919515 | 5.829434433 | 5.787288133 | 5.36722575 | 3.634821254 | 2.334529525 | 3.614622918 | 4.36343169 | 3.960663214 | 2.730704954 |
| ENSMUSG00000024064 | Galnt14 | 5332 | 0.127547415 | 0.131202322 | 0.10886766 | 0.256344961 | 0.027080977 | 0.083641148 | 0.079860111 | 0.300840379 | 0 | 0.150330449 | 0.179200895 | 0.146552752 | 0 | 0.200340212 | 0.108181129 | 0 | 0.007187978 | 0 |
| ENSMUSG00000024065 | Ehd3 | 3652 | 2.618747154 | 1.550709753 | 3.059771498 | 2.058480919 | 2.385508813 | 2.33380892 | 7.021761221 | 5.80415754 | 5.086286585 | 5.099815933 | 4.447832936 | 4.768479389 | 3.687736423 | 4.158602567 | 5.70864931 | 3.680176122 | 5.52016215 | 2.666104022 |
| ENSMUSG00000024066 | Xdh | 4655 | 0.00913108 | 0 | 0.12470083 | 0 | 0.010339833 | 0.106450555 | 0 | 0.07384138 | 0.097325816 | 0.060774268 | 0 | 0 | 0.402709529 | 0.069840733 | 0 | 0 | 0 | 0.200892549 |
| ENSMUSG00000024067 | Dpy30 | 968 | 126.9886044 | 105.9267442 | 107.291291 | 102.9359545 | 117.4955947 | 107.1424291 | 136.2194589 | 120.1798117 | 133.7285321 | 133.8047333 | 113.9590693 | 115.7252426 | 148.4544163 | 140.3398302 | 127.6907724 | 139.6736929 | 117.4336742 | 128.3166973 |
| ENSMUSG00000024068 | Spast | 4679 | 8.984316968 | 8.294405165 | 8.746314743 | 8.588339625 | 7.303625947 | 10.4421875 | 10.20271277 | 6.766724 | 6.962712966 | 6.358643675 | 5.47283132 | 7.455607847 | 5.735533874 | 8.367678066 | 7.564038579 | 8.126217814 | 7.363825799 | 6.160455636 |
| ENSMUSG00000024069 | Slc30a6 | 2118 | 22.33628939 | 18.13492306 | 19.98434328 | 20.90903451 | 18.88462161 | 20.07377026 | 19.07697252 | 22.07153 | 18.39586587 | 19.9911858 | 21.60925884 | 19.97557707 | 20.98586283 | 19.80123273 | 17.70227378 | 22.25483367 | 21.09936836 | 17.9208138 |
| ENSMUSG00000024070 | Prkd3 | 12405 | 4.426980043 | 4.621643769 | 4.932891245 | 4.708525501 | 4.780212008 | 4.693628533 | 3.848326726 | 4.963011099 | 3.449640458 | 3.576687768 | 3.211956096 | 3.61755685 | 4.330710029 | 3.50811043 | 4.360954686 | 5.204188962 | 5.456206395 | 3.911167443 |
| ENSMUSG00000024072 | Yipf4 | 2313 | 108.2200526 | 107.6584022 | 109.5881103 | 107.2152029 | 106.9182118 | 113.7806174 | 109.3939502 | 116.608191 | 116.4547398 | 116.8880815 | 107.7570075 | 113.0792023 | 119.8426473 | 113.4095139 | 116.8713395 | 127.3870402 | 136.221568 | 112.872123 |
| ENSMUSG00000024073 | Birc6 | 16139 | 3.605526116 | 4.508046408 | 4.190234552 | 4.211975844 | 3.89194872 | 4.055961401 | 4.453061484 | 3.899936747 | 3.812668353 | 3.409434953 | 3.179274144 | 3.472267503 | 3.713878152 | 3.718059415 | 3.482181191 | 4.652573339 | 3.552645156 | 3.561838808 |
| ENSMUSG00000024074 | Crim1 | 5978 | 50.27669784 | 49.32292184 | 45.6222757 | 50.54547441 | 46.15930385 | 47.7788485 | 44.04400439 | 53.85141517 | 54.64206365 | 57.15188956 | 50.29237432 | 47.52454375 | 47.58244145 | 37.56392816 | 43.99289583 | 50.86412067 | 55.26475064 | 41.56509452 |
| ENSMUSG00000024076 | Vit | 2617 | 0 | 0 | 0 | 0.13927717 | 0 | 0 | 0.144631804 | 0.189721525 | 0.157380649 | 0.090085409 | 0 | 0.063984329 | 0.018850561 | 0.070988288 | 0.062975248 | 0.014631489 | 0.087870761 | 0.042039822 |
| ENSMUSG00000024077 | Strn | 9901 | 1.734379468 | 2.60083499 | 2.828832853 | 2.452685247 | 2.829287908 | 2.107029677 | 1.562594284 | 2.630766432 | 1.921843277 | 1.814404398 | 1.877028467 | 2.345148534 | 2.042832652 | 2.547130538 | 2.292905494 | 3.059071857 | 2.539344588 | 2.072356005 |

| ENSMUSG00000024078 | Ttc27 |  | 2800 | 4.872914823 | 5.532319933 | 6.426769033 | 6.996874666 | 6.308719995 | 6.707316438 | 4.663678351 | 4.896811594 | 7.134092909 | 4.597193568 | 4.999308155 | 5.322422111 | 5.51460361 | 4.113619169 | 4.885327341 | 4.239317248 | 4.366460254 | 5.59914122 |
| --- | --- | --- | --- | --- | --- | --- | --- | --- | --- | --- | --- | --- | --- | --- | --- | --- | --- | --- | --- | --- | --- |
| ENSMUSG00000024079 | Eif2ak2 |  | 4837 | 7.003643848 | 5.26174306 | 5.91043135 | 5.397245897 | 5.39332285 | 6.956027724 | 6.181850958 | 4.879657781 | 4.955665118 | 4.825221855 | 4.197717046 | 4.558029093 | 6.639462094 | 4.272817632 | 5.400410675 | 6.594185645 | 5.41179673 | 5.947852557 |
| ENSMUSG00000024081 | Cebpz |  | 4058 | 8.861355081 | 10.27790625 | 8.833116312 | 8.330777451 | 10.76978479 | 8.877485784 | 9.886927484 | 9.646922737 | 9.855127355 | 8.179915006 | 10.53686245 | 9.243007703 | 10.75868575 | 7.58808032 | 10.27500605 | 9.190500643 | 9.907413862 | 6.777859379 |
| ENSMUSG00000024082 | 2410091C18R |  | 2293 | 23.35652935 | 25.33697668 | 25.90610348 | 27.71812335 | 25.18897008 | 23.03672656 | 27.54575943 | 25.61705839 | 31.16380499 | 25.3129157 | 26.0855857 | 28.57722978 | 28.57077459 | 30.36183471 | 34.28371942 | 24.94817595 | 28.11375115 | 27.06073807 |
| ENSMUSG00000024083 | Pja2 |  | 6551 | 27.79608825 | 32.96201806 | 28.64309636 | 31.56794081 | 29.44048514 | 31.98878191 | 27.89216395 | 30.00706689 | 26.53767105 | 31.46019294 | 28.93772481 | 27.25603795 | 26.40925567 | 27.20286419 | 30.5851269 | 32.01892037 | 37.77635462 | 34.72182206 |
| ENSMUSG00000024084 | Qpct |  | 1917 | 10.31033221 | 9.123249591 | 6.434663662 | 8.056960892 | 8.23540478 | 11.7354935 | 8.490120788 | 10.77834622 | 9.539286906 | 16.50397594 | 12.75992631 | 8.967774688 | 72.98138623 | 70.62311671 | 69.74389612 | 90.74294701 | 68.57548168 | 82.24105366 |
| ENSMUSG00000024085 | Man2a1 |  | 6929 | 8.950072424 | 9.250109464 | 8.559091964 | 7.903647755 | 8.558021354 | 7.923860413 | 5.401115461 | 7.242721426 | 8.511919551 | 6.199204888 | 6.329544218 | 7.974816851 | 7.269142378 | 5.214821762 | 4.810515041 | 6.150591144 | 6.32779386 | 3.636047176 |
| ENSMUSG00000024087 | Cyp1b1 |  | 6752 | 0 | 0 | 0 | 0 | 0 | 0 | 0 | 0 | 0 | 0 | 0 | 0 | 0 | 0.013757135 | 0 | 0 | 0 | 0 |
| ENSMUSG00000024088 | 4930583I09Ri | k | 626 | 0 | 0 | 0 | 0 | 0 | 0 | 0 | 0 | 0 | 0 | 0 | 0 | 0 | 0 | 0 | 0 | 0.183672349 | 0 |
| ENSMUSG00000024091 | Vapa |  | 1634 | 191.5334217 | 192.7418005 | 181.8892108 | 205.331414 | 202.2777967 | 207.0661352 | 216.7291321 | 207.5338684 | 205.6553134 | 219.9981405 | 192.0062132 | 195.0473888 | 174.9864091 | 167.1304981 | 199.9309282 | 190.1173181 | 198.3163037 | 182.2976211 |
| ENSMUSG00000024095 | Hnrpll |  | 2753 | 25.30547894 | 27.03269367 | 31.43488769 | 28.92869797 | 26.33006794 | 27.30530216 | 19.78092751 | 24.74945365 | 22.96451206 | 25.10822048 | 22.26490074 | 29.19526121 | 27.86455916 | 21.79649869 | 19.35111216 | 21.86445145 | 28.35839695 | 18.3230479 |
| ENSMUSG00000024096 | Ralbp1 |  | 5189 | 47.87054321 | 44.62476927 | 44.36493177 | 39.25677958 | 43.11373698 | 41.04406416 | 24.73680824 | 24.08277908 | 28.09795063 | 24.32500147 | 26.29510145 | 27.03117365 | 32.83724075 | 34.29827382 | 33.15022082 | 46.00185904 | 32.57255142 | 41.8531419 |
| ENSMUSG00000024097 | Srsf7 |  | 2656 | 95.95671516 | 98.12001938 | 96.49207975 | 91.49947684 | 95.41211525 | 86.32548881 | 91.49018034 | 110.8241135 | 107.7734505 | 90.99943709 | 96.95294661 | 97.78248419 | 87.68674001 | 107.8391141 | 97.55895519 | 93.11710588 | 109.6253304 | 95.16823998 |
| ENSMUSG00000024098 | Twsg1 |  | 4041 | 162.6472499 | 187.7673327 | 183.6422492 | 201.2758633 | 196.1843392 | 192.4720877 | 98.41880205 | 126.9110565 | 113.0412389 | 117.2408479 | 128.3929842 | 116.8800141 | 110.3345381 | 104.7376809 | 130.0382687 | 145.9420775 | 141.0988169 | 112.6046352 |
| ENSMUSG00000024099 | Ndufv2 |  | 2126 | 166.6618755 | 142.2300756 | 143.18656 | 139.7262507 | 152.1838151 | 138.47262 | 180.6605492 | 164.6261409 | 179.0819152 | 159.8821354 | 162.4483419 | 149.3318386 | 179.4605119 | 167.9065172 | 159.5736647 | 174.8652433 | 165.7981903 | 169.5294766 |
| ENSMUSG00000024101 | ORF19 |  | 2979 | 4.580114637 | 4.137553159 | 4.384307887 | 4.282340522 | 3.683812889 | 4.191772025 | 3.605229311 | 2.743596547 | 4.728361326 | 3.244677465 | 3.800827512 | 3.391283964 | 3.593496492 | 3.991166959 | 5.227991986 | 3.123403021 | 3.718126823 | 3.877781944 |
| ENSMUSG00000024104 | D6Wsu116e |  | 4268 | 44.2081716 | 49.5010251 | 49.5409328 | 48.37925326 | 47.82743399 | 49.7036674 | 59.65077913 | 54.00448653 | 49.17677702 | 52.07788546 | 62.92011284 | 51.42155232 | 51.05414213 | 56.60781223 | 55.74952795 | 48.6348132 | 62.35656178 | 60.83481343 |
| ENSMUSG00000024105 | 9130404H23R |  | 1793 | 0 | 0 | 0 | 0 | 0.080533057 | 0 | 0 | 0 | 0 | 0 | 0 | 0.124519046 | 0.165081709 | 0 | 0 | 0.085422437 | 0 | 0 |
| ENSMUSG00000024107 | Lhcgr |  | 2542 | 0 | 0 | 0 | 0 | 0 | 0 | 0 | 0 | 0 | 0 | 0 | 0 | 0 | 0 | 0 | 0 | 0 | 0 |
| ENSMUSG00000024109 | Nrxn1 |  | 15676 | 0.002711481 | 0 | 0 | 0 | 0 | 0 | 0 | 0 | 0 | 0 | 0 | 0 | 0 | 0 | 0 | 0 | 0 | 0 |
| ENSMUSG00000024112 | Cacna1h |  | 11518 | 0.114400109 | 0.075198407 | 0.008399641 | 0.007911277 | 0 | 0 | 0 | 0.023211219 | 0 | 0 | 0 | 0 | 0 | 0 | 0 | 0 | 0 | 0 |
| ENSMUSG00000024114 | Prss41 |  | 2564 | 1.193593087 | 0.714590165 | 1.377249499 | 0.852936867 | 0.919837855 | 1.101601331 | 1.273235119 | 0.134060695 | 0.754979033 | 0.956254512 | 1.602436205 | 1.436752625 | 1.077452171 | 0.452847966 | 1.333747714 | 0.88110212 | 0.911819076 | 0.879630802 |
| ENSMUSG00000024116 | Prss21 |  | 1088 | 0 | 0 | 0 | 0 | 0 | 0 | 0 | 0 | 0 | 0 | 0 | 0 | 0 | 0 | 0 | 0 | 0 | 0 |
| ENSMUSG00000024118 | 1600002H07R |  | 3309 | 1.464366899 | 0.7047152 | 2.090484993 | 0.950048975 | 1.440030355 | 1.018309424 | 1.10095989 | 0.565556881 | 1.804788391 | 0.698212288 | 1.429350526 | 1.332558883 | 1.013771645 | 1.403568673 | 2.241245107 | 1.272882083 | 1.494134875 | 1.679032874 |
| ENSMUSG00000024120 | Lrpprc |  | 8123 | 6.111786975 | 6.996404966 | 6.622105842 | 7.347650878 | 7.566719996 | 8.05239546 | 7.321437574 | 6.925693554 | 6.33794715 | 6.506947969 | 6.15198899 | 7.482858126 | 6.322113244 | 4.568364622 | 5.959845891 | 5.251229428 | 5.341052332 | 5.072241898 |
| ENSMUSG00000024121 | Atp6v0c |  | 3833 | 150.093284 | 142.2294584 | 129.8626735 | 139.70231 | 134.6262337 | 109.8356443 | 153.1706321 | 145.6950973 | 145.1467401 | 160.1869183 | 145.09478 | 111.8497533 | 164.1351268 | 150.9402548 | 146.9303594 | 155.2303022 | 131.2673169 | 118.5860289 |
| ENSMUSG00000024122 | Pdpk1 |  | 14050 | 6.39544072 | 5.887253832 | 6.551945072 | 6.566627386 | 6.536349492 | 6.67286633 | 6.832556978 | 5.349654657 | 6.367054252 | 5.040594754 | 5.498370666 | 7.381174454 | 5.993564611 | 4.495655434 | 5.205178769 | 5.753129696 | 6.805986532 | 6.569770895 |
| ENSMUSG00000024124 | Prss30 |  | 1920 | 0.132828675 | 0.988976547 | 1.133754618 | 0.688161607 | 0.626717756 | 1.729183932 | 0 | 0.457513154 | 0.643539309 | 0.12278829 | 0.024882791 | 0.930261043 | 0.128468535 | 0 | 0.40772373 | 0.29914537 | 0.33934742 | 0.974119598 |
| ENSMUSG00000024125 | Sbpl |  | 867 | 0 | 0 | 0 | 0 | 0 | 0.228616995 | 0 | 0 | 0 | 0 | 0 | 0 | 0 | 0 | 0 | 0 | 0 | 0 |
| ENSMUSG00000024127 | Prepl |  | 3815 | 14.87402621 | 14.9056646 | 12.85734201 | 15.77618337 | 15.51828732 | 16.53489642 | 16.55633719 | 15.30699431 | 15.02795018 | 13.74353395 | 16.27980236 | 16.73738349 | 12.90517784 | 12.70973191 | 16.16742573 | 16.73143972 | 15.87301418 | 16.10620504 |
| ENSMUSG00000024128 | Sbp |  | 553 | 0.153725772 | 0 | 0 | 0 | 0 | 0 | 0 | 0 | 0 | 0 | 0.08639233 | 0 | 0 | 0 | 0 | 0 | 0 | 0 |
| ENSMUSG00000024130 | Abca3 |  | 8993 | 20.99026874 | 27.5193474 | 26.96502921 | 26.78541548 | 27.89007608 | 36.60940308 | 18.41895796 | 21.63372599 | 17.35301993 | 22.62898201 | 25.42012413 | 23.23118259 | 18.32735857 | 20.37383796 | 23.69559069 | 20.80372598 | 23.95553374 | 24.62044419 |
| ENSMUSG00000024131 | Slc3a1 |  | 2297 | 0 | 0.014502784 | 0 | 0.099175107 | 0.062862765 | 0.021572805 | 0.020597596 | 0 | 0.089652842 | 0 | 0 | 0.072898123 | 0 | 0.040438909 | 0 | 0 | 0.066741484 | 0.095792959 |
| ENSMUSG00000024132 | Eci1 |  | 1084 | 133.6325091 | 123.8477525 | 121.2015761 | 112.0112391 | 115.933997 | 123.2875668 | 119.154625 | 115.3523298 | 123.3315776 | 108.568409 | 112.870543 | 114.6692625 | 109.9045943 | 102.6997025 | 113.5323316 | 106.5001673 | 98.92710215 | 114.7883758 |
| ENSMUSG00000024134 | Six2 |  | 3074 | 0 | 0 | 0 | 0 | 0 | 0 | 0 | 0 | 0 | 0 | 0 | 0 | 0 | 0 | 0 | 0 | 0 | 0 |
| ENSMUSG00000024135 | Srbd1 |  | 4218 | 1.249559466 | 1.911266098 | 2.259266354 | 1.598633135 | 2.544670218 | 1.245279696 | 1.020733471 | 1.684159992 | 1.415847507 | 1.699124442 | 0.917442304 | 2.884735525 | 0.994123511 | 1.398387648 | 1.30891619 | 2.70521598 | 1.308436879 | 1.551940185 |
| ENSMUSG00000024136 | Dnase1l2 |  | 3197 | 38.71600644 | 39.01266061 | 32.83408222 | 33.91782469 | 34.76278128 | 25.55910468 | 26.41636906 | 25.25453196 | 22.45483171 | 24.21690796 | 24.4478674 | 21.38698363 | 36.18496904 | 38.49760137 | 37.25795999 | 45.81219059 | 33.05148594 | 30.93724551 |
| ENSMUSG00000024137 | E4f1 |  | 2547 | 17.62287628 | 25.8446325 | 13.31364145 | 21.14375909 | 20.10693631 | 21.86779448 | 21.84519451 | 26.90112678 | 16.9629584 | 20.71520885 | 29.03637052 | 20.33644872 | 19.03936972 | 20.82416482 | 21.83828051 | 18.77698023 | 21.60838726 | 25.98192202 |
| ENSMUSG00000024140 | Epas1 |  | 5293 | 0.891380038 | 1.189521445 | 0.594044865 | 0.748877925 | 0.945724553 | 0.70214529 | 0.080448538 | 0.151528519 | 0.303471399 | 0.204886865 | 0.054156386 | 0.253084433 | 0.643095086 | 0.307111857 | 0.27244558 | 0.933211478 | 0.441697357 | 0.114320834 |
| ENSMUSG00000024142 | Mlst8 |  | 3350 | 4.136324593 | 4.713525928 | 3.696604111 | 3.508880429 | 4.410895691 | 3.875467522 | 5.027854835 | 4.070056018 | 3.516221943 | 5.052866409 | 4.335399217 | 3.965411252 | 4.299976073 | 3.618479644 | 4.353836055 | 3.394719522 | 3.031781695 | 2.167523011 |
| ENSMUSG00000024143 | Rhoq |  | 4369 | 19.19494445 | 22.34838475 | 19.37598492 | 24.57939609 | 22.03338232 | 21.47020478 | 8.067737652 | 9.642073667 | 7.598153285 | 10.95398576 | 10.15860298 | 9.594315084 | 7.745867533 | 7.919625796 | 8.487388487 | 10.63968812 | 10.33380127 | 7.025653823 |

| ENSMUSG00000024145 | Pigf | 982 | 17.74656026 | 15.60481807 | 18.22627926 | 17.90892374 | 17.84116112 | 15.94568616 | 20.66918458 | 20.49633001 | 19.4608384 | 18.82186932 | 14.59520116 | 18.41575833 | 23.35981826 | 24.02606557 | 19.09033394 | 16.88374033 | 19.35829253 | 18.98990548 |
| --- | --- | --- | --- | --- | --- | --- | --- | --- | --- | --- | --- | --- | --- | --- | --- | --- | --- | --- | --- | --- |
| ENSMUSG00000024146 | Cript | 1272 | 131.3249542 | 109.3144815 | 103.4022241 | 105.1270952 | 113.1402923 | 108.844605 | 129.886694 | 120.7324293 | 119.6092683 | 125.7722768 | 119.0993658 | 106.1901757 | 128.9533215 | 117.9723635 | 112.9479759 | 117.4004472 | 119.8299393 | 114.2995826 |
| ENSMUSG00000024150 | Mcfd2 | 4003 | 35.76253631 | 35.62640523 | 28.30147592 | 32.00541011 | 34.53282158 | 31.14531044 | 35.0915173 | 32.06711578 | 31.08280393 | 33.55791996 | 32.43875522 | 31.45644137 | 38.01872715 | 33.73954687 | 34.30546735 | 37.39145249 | 37.61779179 | 35.93525208 |
| ENSMUSG00000024151 | Msh2 | 5669 | 12.26644346 | 12.19925355 | 11.0928893 | 13.02777436 | 11.10540768 | 11.8965021 | 11.9429253 | 11.88417672 | 11.46451259 | 13.50727502 | 13.80408926 | 12.49428044 | 9.998654604 | 11.83836766 | 13.02402331 | 11.52298046 | 12.84529266 | 13.7692578 |
| ENSMUSG00000024154 | Gtf2a1l | 1647 | 0 | 0 | 0 | 0 | 0 | 0 | 0 | 0 | 0 | 0 | 0 | 0 | 0 | 0 | 0 | 0 | 0 | 0 |
| ENSMUSG00000024155 | 4930528F23R | 1689 | 0 | 0 | 0 | 0 | 0 | 0 | 0 | 0.04522487 | 0 | 0 | 0 | 0 | 0 | 0.054995959 | 0.951368076 | 0 | 0.158841965 | 0 |
| ENSMUSG00000024158 | Hagh | 1896 | 42.46033934 | 46.19177157 | 49.11342085 | 39.84188371 | 44.57787867 | 49.42205654 | 55.82197403 | 52.7361337 | 64.27790092 | 56.227711 | 59.74389586 | 59.05391305 | 51.28333805 | 47.66887872 | 51.91482965 | 46.20723087 | 50.29315754 | 53.73252408 |
| ENSMUSG00000024160 | Spsb3 | 2798 | 2.521750972 | 2.940773721 | 3.215681432 | 2.865884133 | 2.889979691 | 2.886738947 | 2.73933309 | 2.907427345 | 3.326716427 | 3.050134843 | 3.671056495 | 3.610662948 | 2.274416486 | 2.390260395 | 3.151226934 | 3.284398062 | 2.232732807 | 3.361886079 |
| ENSMUSG00000024163 | Mapk8ip3 | 11743 | 3.033240016 | 3.50915866 | 3.596192796 | 3.336668494 | 3.475761839 | 3.671198008 | 4.407738291 | 4.088209982 | 4.854137598 | 3.738167822 | 3.779522816 | 6.67335351 | 2.781038 | 2.895092563 | 4.880470429 | 3.446578559 | 3.593396312 | 3.330620359 |
| ENSMUSG00000024164 | C3 | 5199 | 0.008175645 | 0.461344178 | 0.167479043 | 0.297956435 | 0 | 0.276404938 | 0.036401369 | 0.117537688 | 0.047532044 | 0.036276748 | 0.009189259 | 0.010735846 | 0.123353515 | 0.017866546 | 0.0158498 | 0.022094984 | 0.036859297 | 0 |
| ENSMUSG00000024165 | Hn1l | 2928 | 70.60969132 | 86.53615896 | 78.98697016 | 83.41965802 | 77.86916749 | 77.49384121 | 75.4611375 | 81.44581988 | 73.44084658 | 81.95011232 | 78.93963426 | 72.80055171 | 70.25754612 | 67.08061666 | 75.00146615 | 73.12878299 | 85.39643477 | 61.03981822 |
| ENSMUSG00000024168 | Tmem204 | 2587 | 0.049290888 | 0.231786663 | 0.07479479 | 0.017611536 | 0.353500792 | 0.114927098 | 0.036577255 | 0.221448023 | 0.541299395 | 0.036452032 | 0.240075168 | 0.215754397 | 0.286037402 | 0.305198873 | 0.3026013 | 0.1184093 | 0.014814958 | 0.170109337 |
| ENSMUSG00000024169 | Ift140 | 7231 | 5.643060294 | 5.178217826 | 4.401850778 | 4.54917356 | 4.845808385 | 6.051039102 | 4.148283562 | 5.223660072 | 4.824364383 | 3.755885089 | 5.523422111 | 4.886089672 | 6.631257584 | 6.615600887 | 7.082496746 | 7.588222985 | 5.819703222 | 5.233890945 |
| ENSMUSG00000024170 | Telo2 | 5961 | 4.606331776 | 5.281108042 | 4.885231551 | 6.641930469 | 4.085682499 | 5.07913441 | 6.595677938 | 6.765840788 | 5.824565138 | 6.810393475 | 7.605843918 | 6.254799968 | 10.48541171 | 8.624996601 | 9.0199682 | 8.087212669 | 6.802419413 | 10.63084901 |
| ENSMUSG00000024171 | Prss28 | 1184 | 0.143598568 | 0.02813589 | 0.081712045 | 0 | 0.16260785 | 0 | 0 | 0.064514193 | 0.452216812 | 0.119469687 | 0.807009434 | 0.141424821 | 0.416654707 | 0 | 0 | 0 | 0 | 0 |
| ENSMUSG00000024172 | St6gal2 | 7788 | 0 | 0 | 0 | 0 | 0 | 0 | 0 | 0 | 0 | 0 | 0.012268865 | 0 | 0.0380061 | 0 | 0 | 0 | 0 | 0 |
| ENSMUSG00000024173 | Tpsab1 | 1597 | 0 | 0 | 0 | 0 | 0 | 0 | 0 | 0.023915092 | 0 | 0 | 0 | 0 | 0 | 0 | 0 | 0 | 0 | 0 |
| ENSMUSG00000024174 | Pot1b | 3482 | 1.489268086 | 1.224598064 | 0.903009613 | 0.981355062 | 0.815561027 | 0.69732451 | 1.644122387 | 1.129758026 | 0.99358625 | 0.988512733 | 1.207408485 | 1.538858015 | 1.685955817 | 0.680255157 | 0.757294537 | 0.648806961 | 0.913579162 | 0.42654965 |
| ENSMUSG00000024175 | Tekt4 | 1526 | 0 | 0 | 0 | 0.119426066 | 0.031541234 | 0 | 0.248035014 | 0.450500159 | 0.215918824 | 0.061796466 | 0 | 0 | 0.193965599 | 1.278277635 | 0.296996799 | 0.050184282 | 0.025115529 | 0.324431167 |
| ENSMUSG00000024176 | Sox8 | 3208 | 0 | 0 | 0 | 0 | 0 | 0 | 0.044245024 | 0 | 0 | 0 | 0 | 0 | 0 | 0 | 0.051373511 | 0 | 0 | 0 |
| ENSMUSG00000024177 | Nme4 | 1493 | 2.676146381 | 2.476712162 | 3.240022127 | 2.563380927 | 2.643548386 | 2.555633279 | 5.070347373 | 3.63249909 | 4.910381651 | 3.631835817 | 4.127910009 | 4.37403384 | 6.211922604 | 7.217031664 | 6.291999158 | 5.43711237 | 4.77474295 | 5.637235987 |
| ENSMUSG00000024180 | Tmem8 | 3451 | 31.16143012 | 36.44050295 | 36.0944192 | 36.86074639 | 35.34230501 | 36.09839821 | 38.5932168 | 37.19636757 | 31.01818443 | 32.58604094 | 29.39039028 | 37.97599993 | 27.71793324 | 33.67229981 | 27.45974458 | 32.65408797 | 26.8983747 | 34.19140363 |
| ENSMUSG00000024181 | Mrpl28 | 1395 | 118.7097963 | 103.2102716 | 101.7751338 | 96.54368965 | 98.19602492 | 99.60277058 | 111.4476437 | 94.09841367 | 102.9810516 | 99.10097631 | 107.9816803 | 98.02750777 | 116.0626183 | 95.48504848 | 106.0607794 | 106.1981076 | 93.93376997 | 105.6411447 |
| ENSMUSG00000024182 | Axin1 | 4124 | 17.78950869 | 22.38361539 | 20.98454069 | 22.82471329 | 18.02029344 | 21.67631702 | 18.67726511 | 23.52296365 | 18.55590356 | 19.4708166 | 23.30824841 | 21.76323604 | 16.02928447 | 21.93818677 | 18.79246524 | 17.14906689 | 21.01255027 | 22.02231697 |
| ENSMUSG00000024184 | Pdia2 | 1830 | 0 | 0.145630139 | 0.052867246 | 0.074690237 | 0.105206391 | 0.027077996 | 0 | 0.062610496 | 0.067518878 | 0 | 0 | 0.122001448 | 0.053914664 | 0 | 0.045029023 | 0.020923829 | 0.020943332 | 0.030059621 |
| ENSMUSG00000024186 | Rgs11 | 3770 | 90.27558208 | 87.67388235 | 86.02019828 | 95.91989626 | 84.56919426 | 77.40478728 | 131.9735097 | 142.9125254 | 137.2483283 | 117.7641968 | 131.5395409 | 129.5752464 | 72.16592143 | 70.39297489 | 77.63815731 | 71.98024792 | 70.76640656 | 78.36973799 |
| ENSMUSG00000024187 | Itfg3 | 4972 | 101.1164968 | 117.9150493 | 110.0371336 | 115.1946678 | 122.1304 | 127.9381418 | 95.99563657 | 100.4662975 | 93.10041244 | 102.2673339 | 122.7620413 | 115.2460966 | 69.64214146 | 67.07863868 | 69.0863579 | 68.24076271 | 84.93144394 | 79.32729188 |
| ENSMUSG00000024188 | Luc7l | 7214 | 20.5867875 | 18.96997081 | 21.01506018 | 22.22474564 | 20.45640116 | 18.58055785 | 24.12198962 | 23.44803857 | 25.40044478 | 22.17664765 | 27.70205868 | 26.37587937 | 24.08469818 | 24.98606919 | 27.74565546 | 23.51363887 | 25.54909368 | 26.50563557 |
| ENSMUSG00000024190 | Dusp1 | 3754 | 102.5830833 | 63.91922671 | 63.03764265 | 78.29363266 | 87.03236491 | 81.03469138 | 102.5528152 | 71.25721536 | 75.63661152 | 91.0734548 | 130.2548482 | 78.68313437 | 209.8380541 | 255.9002407 | 200.6737474 | 198.2870026 | 230.1007138 | 322.6692941 |
| ENSMUSG00000024191 | Bnip1 | 5140 | 8.484496227 | 8.749495563 | 8.187737628 | 6.745516486 | 7.4070723 | 7.510034914 | 8.385574028 | 9.399492032 | 9.270972517 | 7.925721326 | 7.249896423 | 8.600390033 | 8.954606774 | 8.213555532 | 8.785390128 | 8.462671208 | 7.695085293 | 7.341682337 |
| ENSMUSG00000024193 | Phf1 | 2414 | 31.20098257 | 33.13349587 | 27.85385551 | 30.7263381 | 30.44633283 | 29.70290202 | 23.98952734 | 29.14266994 | 27.24725173 | 30.31395669 | 24.6791107 | 30.77491587 | 27.24086401 | 31.12946702 | 31.69481744 | 33.59548736 | 30.72136889 | 30.64923303 |
| ENSMUSG00000024194 | Cuta | 806 | 93.55357602 | 87.16736217 | 87.32442514 | 82.07771262 | 85.63421655 | 91.91232841 | 96.26897449 | 85.76705751 | 86.92092222 | 95.0034021 | 96.26120666 | 86.97825336 | 102.5810091 | 106.7176797 | 93.08688992 | 98.19688027 | 77.12810617 | 84.76589398 |
| ENSMUSG00000024197 | Plin3 | 2151 | 123.2077046 | 134.939678 | 130.4353678 | 131.1759868 | 123.3170764 | 143.0832302 | 122.2079238 | 123.9344346 | 126.1061798 | 130.8645739 | 121.8916653 | 133.5839289 | 127.4694543 | 103.4465935 | 116.9197104 | 131.8542673 | 113.3574621 | 109.9414922 |
| ENSMUSG00000024201 | Kdm4b | 5025 | 17.81410959 | 19.95458937 | 20.81265127 | 19.63885016 | 21.04394752 | 20.49165779 | 21.29776711 | 21.34974296 | 26.29379952 | 23.77709096 | 23.62602424 | 20.06031575 | 16.55196269 | 17.27442773 | 20.10471941 | 15.24766277 | 17.66441863 | 19.96748471 |
| ENSMUSG00000024205 | Rpl36-ps2 | 310 | 1.23402124 | 1.611914236 | 1.24834917 | 2.645479994 | 0.776321349 | 0.639390111 | 1.526215449 | 1.601616875 | 1.062877826 | 1.216792342 | 1.541127693 | 0.720202098 | 1.909622604 | 1.947655278 | 1.461990693 | 1.235180884 | 1.607231806 | 2.306833508 |
| ENSMUSG00000024206 | Rfx2 | 3335 | 1.797070411 | 1.87790828 | 1.711567191 | 2.718635029 | 2.641122048 | 1.604706215 | 1.77333879 | 2.966066634 | 2.655202665 | 2.021754293 | 2.764787702 | 2.259404632 | 1.079829074 | 1.559741466 | 1.692537678 | 1.814067757 | 2.206491456 | 1.83088781 |
| ENSMUSG00000024207 | Acsbg2 | 2878 | 0.07384499 | 0 | 0.134464296 | 0 | 0.033448175 | 0 | 0 | 0 | 0 | 0 | 0 | 0 | 0 | 0 | 0.05726415 | 0 | 0 | 0 |
| ENSMUSG00000024208 | 2900010M23R | 1003 | 127.6002842 | 94.09314987 | 99.06204521 | 88.66915109 | 114.1633563 | 87.89064111 | 101.1349787 | 76.68943015 | 80.93581512 | 85.55760711 | 80.49818527 | 74.95882104 | 101.7132651 | 96.87041956 | 87.16819701 | 88.83573621 | 68.74277979 | 76.34364562 |
| ENSMUSG00000024209 | 1700061G19R | 3089 | 0.220162776 | 0.05392181 | 0.062639729 | 0.073747239 | 0.077908585 | 0.016041675 | 0.015316503 | 0.024728004 | 0.039999853 | 0.030528134 | 0.139195412 | 0 | 0.143731711 | 0 | 0.013338153 | 0.024791588 | 0.037222043 | 0 |
| ENSMUSG00000024210 | Ip6k3 | 2301 | 2.253642536 | 3.460139817 | 1.345461079 | 2.118657864 | 2.154536348 | 0.753735626 | 0.822471602 | 0.099589055 | 0.894969921 | 0.368845136 | 0.519067345 | 0.339599859 | 0.128636029 | 0 | 0 | 0.26625368 | 0.083281827 | 0.143439652 |
| ENSMUSG00000024211 | Grm8 | 8399 | 0 | 0.039662929 | 0.034556636 | 0 | 0.040114712 | 0 | 0 | 0 | 0 | 0 | 0 | 0 | 0.005873547 | 0 | 0 | 0 | 0 | 0 |

| ENSMUSG00000024212 | Mllt1 |  | 3621 | 17.76037872 | 21.72100062 | 21.08075971 | 21.86829469 | 21.56033698 | 22.66206208 | 10.94946836 | 11.16977469 | 9.645447491 | 14.33662643 | 10.81896326 | 11.49916716 | 6.689304441 | 8.298625943 | 10.06997431 | 8.829786573 | 8.287625097 | 7.459119415 |
| --- | --- | --- | --- | --- | --- | --- | --- | --- | --- | --- | --- | --- | --- | --- | --- | --- | --- | --- | --- | --- | --- |
| ENSMUSG00000024213 | Nudt3 |  | 3357 | 37.5544689 | 40.00127393 | 39.10806118 | 40.58013779 | 36.23156721 | 37.96533535 | 44.04293168 | 39.29596601 | 40.70803077 | 42.36119185 | 39.02262024 | 40.66878483 | 38.76604045 | 39.74794849 | 35.23671938 | 35.21093388 | 37.8239564 | 32.00261705 |
| ENSMUSG00000024215 | Spdef |  | 2754 | 0.16977376 | 0.060480926 | 0.035129652 | 0.248153836 | 0.279633543 | 0.21591605 | 0 | 0.124811773 | 0.16450678 | 0 | 0 | 0.567479503 | 0.053738472 | 0.303556127 | 0.553543053 | 0.472723548 | 0.180915708 | 0.878867355 |
| ENSMUSG00000024217 | Snrpc |  | 6244 | 6.507839251 | 5.367195962 | 5.748424011 | 5.859307912 | 5.912425601 | 5.59491947 | 6.402820899 | 6.177822939 | 5.639729497 | 5.65597001 | 5.29472634 | 6.248422189 | 5.569987457 | 6.359656425 | 5.569204544 | 6.236634806 | 4.953446765 | 5.726444486 |
| ENSMUSG00000024218 | Taf11 |  | 2945 | 13.45155316 | 12.98580732 | 13.41975358 | 10.96868606 | 11.47321236 | 10.49945866 | 11.82415338 | 12.07372721 | 12.89438626 | 12.904403 | 14.97327222 | 9.931207876 | 13.16634532 | 11.9067185 | 12.82914321 | 13.31394974 | 11.32219976 | 12.55216289 |
| ENSMUSG00000024219 | Anks1 |  | 7392 | 2.938331297 | 3.096044146 | 3.272019099 | 3.574865492 | 3.398926428 | 3.18419216 | 2.765026691 | 2.75902907 | 3.170336508 | 3.278606801 | 3.121659219 | 3.473377271 | 2.649454973 | 2.180208107 | 3.115756188 | 2.621083245 | 2.327990707 | 3.058542054 |
| ENSMUSG00000024220 | Zfp523 |  | 3378 | 15.90483792 | 15.15746549 | 15.68058488 | 18.0328941 | 14.27714254 | 15.71076899 | 12.89963804 | 16.11135981 | 14.13119354 | 13.69296622 | 16.51899097 | 17.49816065 | 11.91676865 | 19.57856141 | 18.67364774 | 15.49534053 | 15.70266279 | 17.01732283 |
| ENSMUSG00000024222 | Fkbp5 |  | 8353 | 20.0185996 | 19.16299015 | 20.8018342 | 21.65417766 | 18.01273715 | 20.02160611 | 22.36208026 | 20.62106247 | 21.14796673 | 19.64377437 | 19.06308352 | 20.40048101 | 19.11146705 | 21.97934604 | 18.30961038 | 13.86218087 | 16.55927278 | 17.16853121 |
| ENSMUSG00000024223 | 4930511I11Ri | k | 1077 | 0 | 0 | 0 | 0 | 0 | 0 | 0 | 0 | 0 | 0 | 0 | 0 | 0 | 0 | 0 | 0 | 0 | 0 |
| ENSMUSG00000024224 | Gm749 |  | 439 | 75.52172506 | 156.7754885 | 61.15560216 | 96.41505738 | 65.78395034 | 117.3914417 | 0.107773756 | 0.086998639 | 0.281456828 | 0.107404791 | 0 | 0.127142739 | 0.112373388 | 0.211590375 | 0.281559607 | 0 | 0.261910913 | 0 |
| ENSMUSG00000024225 | Clps |  | 774 | 0 | 0 | 0 | 0 | 0 | 0 | 0 | 0 | 0 | 0 | 0 | 0 | 0 | 0 | 0 | 0 | 0 | 0 |
| ENSMUSG00000024227 | 2610034M16R |  | 4627 | 0 | 0.014399349 | 0 | 0 | 0 | 0 | 0.020450693 | 0.024762742 | 0.008901343 | 0.02038068 | 0.07227679 | 0.07237821 | 0 | 0.030112873 | 0.097950533 | 0 | 0.01656637 | 0.03566616 |
| ENSMUSG00000024228 | Nudt12 |  | 6574 | 2.831954229 | 3.765056344 | 3.767454752 | 3.763256324 | 3.873104293 | 2.902008279 | 4.519677878 | 3.863393307 | 3.264097157 | 4.446826288 | 4.970804928 | 3.302752167 | 3.361834338 | 3.793805131 | 3.396903452 | 3.424836804 | 4.669815001 | 5.346945421 |
| ENSMUSG00000024231 | Cul2 |  | 4439 | 17.37934095 | 19.06166959 | 18.91787535 | 16.54525872 | 16.4813075 | 19.66927609 | 17.11740535 | 16.53656171 | 12.3494599 | 16.43211037 | 20.56723263 | 15.9814614 | 13.5804467 | 11.43577101 | 13.92258027 | 16.07015129 | 16.90535917 | 15.26722674 |
| ENSMUSG00000024232 | Bambi |  | 5037 | 1.535823315 | 1.640182205 | 1.017985749 | 1.01307067 | 1.127569385 | 1.023125728 | 1.117770259 | 1.13735564 | 1.349171154 | 1.3198827 | 1.564992684 | 1.14135661 | 3.310341084 | 2.886043151 | 2.298518405 | 3.44364605 | 2.427256048 | 1.430651773 |
| ENSMUSG00000024233 | Lyzl1 |  | 926 | 0 | 0 | 0 | 0 | 0 | 0 | 0 | 0 | 0 | 0 | 0 | 0 | 0 | 0 | 0 | 0 | 0 | 0 |
| ENSMUSG00000024234 | Mtpap |  | 2642 | 14.35072559 | 16.25296012 | 15.9475189 | 14.48572189 | 16.43262496 | 15.9611568 | 23.85332639 | 19.51542137 | 15.85415841 | 13.58125858 | 16.41925144 | 17.13342254 | 14.60165 | 14.55552777 | 17.45062114 | 15.85538399 | 19.97551508 | 15.71986207 |
| ENSMUSG00000024235 | Map3k8 |  | 5591 | 4.340986498 | 3.92056598 | 4.481754377 | 3.935965733 | 4.614328579 | 4.130133044 | 4.38346766 | 6.455342564 | 4.412577883 | 6.299691528 | 6.639446027 | 5.770247357 | 5.858771789 | 5.831470103 | 6.499690985 | 7.060922238 | 6.971533527 | 7.133178747 |
| ENSMUSG00000024236 | Svil |  | 10894 | 7.592718244 | 8.146277786 | 6.864831828 | 7.193408873 | 6.521270363 | 6.895717285 | 4.256143322 | 3.754733154 | 3.402594473 | 3.644289714 | 3.547819112 | 4.196165564 | 4.433261154 | 4.651229974 | 4.863704872 | 4.896164504 | 4.49614535 | 4.81721019 |
| ENSMUSG00000024238 | Zeb1 |  | 9794 | 0 | 0 | 0 | 0 | 0 | 0 | 0 | 0 | 0 | 0 | 0 | 0 | 0 | 0 | 0 | 0 | 0 | 0 |
| ENSMUSG00000024240 | Epc1 |  | 5812 | 6.699026366 | 5.754670646 | 6.075821948 | 4.578054008 | 5.250454165 | 4.331174924 | 5.551831904 | 5.973312764 | 4.790448151 | 4.713447796 | 5.663617755 | 4.734535729 | 5.135204741 | 6.576648989 | 5.649972478 | 3.834331299 | 6.805357606 | 6.208873711 |
| ENSMUSG00000024241 | Sos1 |  | 8897 | 7.543629645 | 8.229936101 | 8.15015421 | 7.573877102 | 8.114857312 | 8.153894791 | 5.812381485 | 7.396370613 | 7.40218486 | 8.060719305 | 7.571393892 | 6.800514583 | 5.483788489 | 5.883161345 | 6.228626804 | 7.05817648 | 6.771826316 | 6.096322269 |
| ENSMUSG00000024242 | Map4k3 |  | 4196 | 20.05725656 | 21.22942782 | 19.92122508 | 23.27999977 | 21.714426 | 21.39884491 | 15.12066312 | 19.45118302 | 19.5429821 | 20.1929966 | 20.88161913 | 15.7630029 | 13.93191658 | 14.21215639 | 14.80742285 | 16.59016307 | 16.63302828 | 14.23734268 |
| ENSMUSG00000024245 | Tmem178 |  | 1692 | 0.025121262 | 0.196884718 | 0.028589557 | 0.053854662 | 0 | 0 | 0 | 0 | 0 | 0 | 0 | 0.03298798 | 0 | 0 | 0 | 0 | 0 | 0 |
| ENSMUSG00000024246 | Thumpd2 |  | 2011 | 4.396358336 | 4.207595789 | 3.006808202 | 3.149172135 | 4.284243827 | 4.139661482 | 3.387879545 | 4.178184251 | 4.710541335 | 4.29069055 | 3.088386177 | 5.217973429 | 2.673883134 | 3.879963541 | 4.486892462 | 3.617710297 | 3.582965598 | 4.075761761 |
| ENSMUSG00000024247 | Pkdcc |  | 3383 | 0.075386064 | 0 | 0 | 0 | 0.056910344 | 0 | 0.041956263 | 0.090316057 | 0 | 0.08362525 | 0 | 0 | 0 | 0.054914676 | 0.073073998 | 0 | 0 | 0 |
| ENSMUSG00000024248 | Cox7a2l |  | 1143 | 48.3064074 | 46.25333607 | 41.85601183 | 42.81064008 | 47.92137282 | 43.65669529 | 58.24054178 | 46.24521866 | 46.05106487 | 45.5006349 | 47.90035207 | 43.36334941 | 63.83368823 | 67.97984095 | 46.32020938 | 45.39262732 | 52.20826271 | 50.48517319 |
| ENSMUSG00000024251 | Thada |  | 7849 | 2.924295459 | 4.45643253 | 3.328029863 | 4.237426725 | 4.519458242 | 3.851085168 | 2.218252751 | 3.644554644 | 2.954262756 | 3.261922776 | 3.725095501 | 3.143142166 | 2.922581416 | 2.473388779 | 2.965840117 | 2.180647408 | 3.208100021 | 2.600124678 |
| ENSMUSG00000024253 | Dync2li1 |  | 1311 | 14.03870421 | 12.73055683 | 12.95126556 | 15.74306109 | 12.2991567 | 15.53483867 | 16.38440598 | 16.98411182 | 16.58770429 | 15.75286653 | 23.97858328 | 17.37054945 | 20.54555822 | 14.38314224 | 17.63087174 | 16.44363994 | 18.41767128 | 17.41325651 |
| ENSMUSG00000024254 | Abcg8 |  | 3492 | 0 | 0 | 0 | 0 | 0 | 0 | 0 | 0 | 0 | 0 | 0 | 0 | 0 | 0 | 0 | 0 | 0 | 0 |
| ENSMUSG00000024256 | Adcyap1 |  | 2083 | 0 | 0 | 0 | 0 | 0 | 0 | 0 | 0 | 0 | 0 | 0 | 0 | 0 | 0 | 0 | 0 | 0 | 0 |
| ENSMUSG00000024258 | Polr2d |  | 952 | 69.87458034 | 60.81702747 | 60.16203775 | 58.43491086 | 59.2548472 | 57.98502648 | 69.0307889 | 60.05675042 | 56.02577512 | 62.50439865 | 65.03817877 | 57.75044921 | 80.11254633 | 77.0327878 | 74.18011218 | 75.09302943 | 67.15153701 | 72.40169194 |
| ENSMUSG00000024259 | Slc25a46 |  | 6638 | 13.98483044 | 12.42584003 | 12.67272812 | 12.73897232 | 14.45842962 | 13.70575759 | 13.65638639 | 10.8743056 | 12.39690547 | 14.38387678 | 13.58110073 | 11.36830006 | 10.5010544 | 12.39115378 | 11.59453245 | 14.25947296 | 15.37555418 | 11.69295715 |
| ENSMUSG00000024260 | Sap130 |  | 4009 | 12.0019604 | 10.68605187 | 8.748019333 | 7.909824609 | 7.539747584 | 7.947719554 | 6.561698549 | 11.17477875 | 11.11594164 | 7.186101195 | 8.639771738 | 9.481283667 | 7.715418342 | 8.932000842 | 8.715120827 | 9.751736133 | 9.502703697 | 7.917249833 |
| ENSMUSG00000024261 | Syt4 |  | 3897 | 0 | 0 | 0 | 0 | 0 | 0 | 0 | 0 | 0 | 0 | 0 | 0 | 0 | 0 | 0 | 0 | 0 | 0 |
| ENSMUSG00000024266 | Adad2 |  | 1918 | 0 | 0 | 0 | 0 | 0 | 0.025835628 | 0 | 0.099563093 | 0.214736787 | 0 | 0.074726212 | 0 | 0 | 0 | 0 | 0 | 0 | 0 |
| ENSMUSG00000024268 | Celf4 |  | 3985 | 0.234658437 | 0.225708443 | 0.412722718 | 0.49162482 | 0.181174117 | 0.198957023 | 0.747979617 | 0.661298812 | 0.444421626 | 0.41412161 | 0.599434862 | 0.67230911 | 0.544693691 | 0.990651801 | 0.651367132 | 0.076869475 | 0.163499886 | 0.276080837 |
| ENSMUSG00000024269 | 5730494M16R |  | 6832 | 4.087514732 | 3.296182156 | 3.327804342 | 3.414410817 | 3.691617096 | 3.459697587 | 4.210496017 | 3.689547071 | 3.653253594 | 4.844817577 | 4.482398768 | 3.954154083 | 4.527387607 | 4.37792627 | 3.90787591 | 4.08014669 | 4.734688903 | 3.607154539 |
| ENSMUSG00000024270 | Slc39a6 |  | 4063 | 9.446757065 | 12.6921881 | 13.44172182 | 11.47156002 | 10.1405185 | 11.50091749 | 8.850021163 | 10.189654 | 10.23834135 | 9.283918928 | 10.46510289 | 6.442910596 | 5.803754974 | 7.3615536 | 10.32320549 | 8.038859984 | 7.838826662 | 7.690172931 |
| ENSMUSG00000024271 | Elp2 |  | 3444 | 26.58424772 | 30.45943783 | 27.68415457 | 27.05352371 | 30.08944009 | 30.47406786 | 28.42332541 | 23.90906492 | 29.37110416 | 28.35339908 | 26.67573901 | 22.2517145 | 22.2165516 | 25.31229734 | 28.46065664 | 24.89334204 | 30.08013372 | 26.29064741 |
| ENSMUSG00000024273 | 2700062C07R |  | 1035 | 22.29981699 | 23.33511914 | 25.61226535 | 19.63306838 | 25.62385502 | 24.32153493 | 26.37624709 | 28.41367135 | 34.34199334 | 27.8348596 | 29.21888765 | 27.55729815 | 26.73932907 | 37.15526991 | 30.65236525 | 24.63917346 | 30.99430967 | 36.77903561 |

| ENSMUSG00000024274 | Zscan30 |  | 1686 | 0.806741182 | 0.968168337 | 0.774665077 | 0.621532633 | 0.827891925 | 0.617204867 | 0.112248349 | 1.042022841 | 0.635141999 | 0.783048453 | 1.218459795 | 0.529686003 | 0 | 0.385656716 | 0.562061557 | 0.953858547 | 0.659230492 | 0.391523891 |
| --- | --- | --- | --- | --- | --- | --- | --- | --- | --- | --- | --- | --- | --- | --- | --- | --- | --- | --- | --- | --- | --- |
| ENSMUSG00000024276 | Zfp397 |  | 5483 | 7.914970769 | 7.564208153 | 7.957855988 | 5.517514762 | 7.847882502 | 5.395400683 | 6.532135318 | 6.693944686 | 6.595250928 | 6.879548168 | 8.277623665 | 7.543207364 | 6.406041419 | 6.708684518 | 7.424245344 | 8.114843298 | 8.492878121 | 2.829211763 |
| ENSMUSG00000024277 | Mapre2 |  | 10272 | 5.15176637 | 5.344592063 | 4.431414726 | 5.854807099 | 4.994999088 | 5.615204623 | 5.545606058 | 6.852472511 | 5.71366676 | 6.793520329 | 5.674206517 | 6.118422515 | 6.113661477 | 6.33451776 | 7.52875004 | 7.809464808 | 7.977182903 | 6.833296357 |
| ENSMUSG00000024283 | Wac |  | 6146 | 24.93185154 | 26.47253097 | 29.74350329 | 28.92599984 | 26.61900562 | 30.68625187 | 25.6963427 | 22.15358194 | 22.42272726 | 26.54432692 | 23.84083919 | 21.75049006 | 19.56099616 | 22.94999893 | 23.63089563 | 21.35078288 | 25.0062562 | 20.03992677 |
| ENSMUSG00000024286 | Ccny |  | 4007 | 9.897012537 | 8.696103657 | 8.221206432 | 7.493069183 | 8.75671883 | 7.988786097 | 9.847460501 | 9.788768972 | 9.528300502 | 9.037120065 | 11.24327074 | 7.703035041 | 9.898393184 | 8.22942402 | 7.938008772 | 7.950532907 | 7.804905983 | 7.413256209 |
| ENSMUSG00000024287 | Thoc1 |  | 3834 | 23.88005977 | 20.51427836 | 23.12693822 | 21.78231462 | 20.9525771 | 21.4676814 | 19.65808491 | 18.25943495 | 21.96829023 | 20.51313851 | 20.6850368 | 21.2256745 | 20.17539027 | 20.88409146 | 22.87900691 | 23.20007329 | 25.71080742 | 22.76981022 |
| ENSMUSG00000024290 | Rock1 |  | 6154 | 22.42676415 | 18.77301221 | 20.80674924 | 18.53832548 | 18.45031003 | 21.86150011 | 21.41909709 | 19.82852221 | 20.56648732 | 20.11988085 | 19.33822255 | 22.62012715 | 16.04048854 | 15.87128953 | 19.89779397 | 20.30883045 | 18.83929934 | 16.42943421 |
| ENSMUSG00000024292 | Cyp4f14 |  | 2186 | 0 | 0 | 0 | 0 | 0 | 0.022668222 | 0 | 0 | 0 | 0.064708193 | 0 | 0 | 0 | 0 | 0 | 0 | 0 | 0 |
| ENSMUSG00000024293 | Esco1 |  | 5694 | 10.83904383 | 10.04535289 | 10.39002944 | 9.193825069 | 11.30178819 | 9.590290201 | 10.64410638 | 9.531331896 | 10.83551117 | 12.09820468 | 9.65735462 | 13.37066452 | 12.38063045 | 11.03597651 | 10.73092859 | 13.1333959 | 12.76197031 | 12.67508746 |
| ENSMUSG00000024294 | Mib1 |  | 11016 | 2.859144466 | 3.979644952 | 3.258275193 | 3.304581919 | 3.495419293 | 3.544621828 | 2.697200651 | 2.943477636 | 3.492030294 | 2.546720083 | 2.515384524 | 3.12620405 | 3.103396757 | 2.33991181 | 2.333857196 | 4.021626068 | 3.287795077 | 2.371943146 |
| ENSMUSG00000024295 | Cyp4f41-ps |  | 2164 | 0.137493638 | 0.153941286 | 0.201183814 | 0.252649044 | 0.311389525 | 0.343480131 | 0.45913413 | 0.811853286 | 0.380651717 | 0.217886799 | 0.154540069 | 0.412685121 | 0.113983173 | 0.53655369 | 0.266553504 | 0.230026754 | 0.442771452 | 0.279621152 |
| ENSMUSG00000024298 | Zfp871 |  | 8127 | 3.436188095 | 5.26316675 | 4.815329895 | 5.084762792 | 4.074660205 | 4.133967438 | 3.434782893 | 3.797152842 | 3.091395917 | 4.972087202 | 4.120862052 | 4.230644537 | 4.206597597 | 3.468876713 | 3.026618539 | 3.910570216 | 4.913990571 | 3.309887189 |
| ENSMUSG00000024299 | Adamts10 |  | 4622 | 2.657723036 | 2.328010565 | 2.323436551 | 1.922198971 | 1.968181214 | 2.283585515 | 0.603948087 | 1.462582264 | 1.755461619 | 1.152754104 | 0.930278291 | 1.485358394 | 0.853862696 | 0.663199863 | 1.149935247 | 0.679322762 | 0.572158046 | 1.451992865 |
| ENSMUSG00000024300 | Myo1f |  | 4997 | 0 | 0 | 0 | 0 | 0.038528656 | 0 | 0 | 0.129932127 | 0 | 0 | 0 | 0 | 0 | 0 | 0.206131458 | 0 | 0 | 0 |
| ENSMUSG00000024301 | Kifc5b |  | 3279 | 0.038888542 | 0.060956806 | 0.25079293 | 0.069473993 | 0.102751896 | 0 | 0.072144982 | 0.139770915 | 0.12560694 | 0.086277591 | 0.335109498 | 0 | 0.270806499 | 0.099148707 | 0.263870898 | 0.070065155 | 0.222079793 | 0 |
| ENSMUSG00000024302 | Dtna |  | 9378 | 0.163167662 | 0.234447752 | 0.283700594 | 0.072874351 | 0.19503232 | 0.200789494 | 0.30270428 | 0.122176591 | 0.19324021 | 0.115639387 | 0 | 0.410671862 | 0.326144047 | 0.614103949 | 0.316326725 | 0.518544161 | 0.568069447 | 0.516186969 |
| ENSMUSG00000024304 | Cdh2 |  | 9379 | 0.126894651 | 0.120763237 | 0.020630571 | 0.014573316 | 0.010263765 | 0.031700224 | 0 | 0.057009663 | 0 | 0.035190844 | 0.010187644 | 0 | 0 | 0 | 0.061501416 | 0.00408259 | 0 | 0 |
| ENSMUSG00000024306 | 4921528I01Ri | k | 3723 | 0 | 0 | 0 | 0 | 0.025856526 | 0 | 0.101665708 | 0 | 0 | 0.151976481 | 0.051329528 | 0.01499212 | 0 | 0.024949819 | 0 | 0 | 0 | 0 |
| ENSMUSG00000024308 | Tapbp |  | 3290 | 172.8113175 | 191.706087 | 191.0533901 | 202.7951165 | 193.683446 | 228.9065182 | 149.9337357 | 162.6485563 | 156.1582364 | 179.5452919 | 189.2411121 | 187.6695622 | 186.1117803 | 169.0621248 | 196.4650483 | 187.2864603 | 192.9012615 | 206.3259504 |
| ENSMUSG00000024309 | H2-Ke2 |  | 1369 | 49.05637555 | 40.95368953 | 42.29592099 | 35.67672719 | 39.94000386 | 37.02881406 | 45.48099742 | 45.78066643 | 45.81962272 | 45.63526794 | 35.00239836 | 43.38047114 | 43.386142 | 46.2744596 | 46.76933371 | 44.47192532 | 39.72608861 | 43.43670151 |
| ENSMUSG00000024312 | Wdr46 |  | 2403 | 11.24980939 | 13.58578291 | 13.38676558 | 12.03964343 | 11.2568211 | 14.57918545 | 13.17194432 | 7.930923346 | 10.81510256 | 13.24457957 | 10.5570133 | 12.84480292 | 12.35864095 | 8.426809031 | 11.3334284 | 9.640373482 | 9.713156394 | 13.64354041 |
| ENSMUSG00000024313 | Mep1b |  | 2376 | 0 | 0 | 0 | 0 | 0 | 0 | 0 | 0 | 0 | 0 | 0 | 0.070474321 | 0 | 0 | 0.034681444 | 0 | 0 | 0 |
| ENSMUSG00000024317 | Rnf138 |  | 3102 | 24.15751945 | 22.88516363 | 22.72090062 | 27.02524874 | 26.09861238 | 24.00959335 | 20.36183958 | 21.66944817 | 18.16349244 | 22.19214273 | 20.89961917 | 20.54851279 | 17.38226486 | 16.37970071 | 22.60639849 | 21.910325 | 21.62186318 | 21.67025416 |
| ENSMUSG00000024319 | Vps52 |  | 3664 | 16.27586298 | 14.35618449 | 14.7602639 | 19.64695689 | 15.33022787 | 15.48522924 | 16.05067137 | 16.05248354 | 15.48990686 | 17.83593742 | 20.04096921 | 12.75046659 | 15.29504859 | 16.33909079 | 15.88913714 | 16.14601213 | 18.0962046 | 15.83913963 |
| ENSMUSG00000024325 | Ring1 |  | 3343 | 20.10190945 | 18.47505411 | 18.94135544 | 21.75154854 | 18.52999873 | 18.30619981 | 17.97400605 | 24.4943197 | 20.77190116 | 25.40185958 | 24.53772172 | 20.78686806 | 19.72981555 | 20.3670452 | 23.08421005 | 21.06384296 | 20.9115063 | 22.18135682 |
| ENSMUSG00000024327 | Slc39a7 |  | 3180 | 1.898029873 | 1.927538533 | 1.931898854 | 2.679218645 | 1.952521432 | 2.649045506 | 2.142460933 | 2.197864667 | 1.774387629 | 2.179607981 | 2.313630065 | 2.053595133 | 1.551318153 | 2.073918368 | 2.720857464 | 2.39617323 | 2.000680908 | 2.646664569 |
| ENSMUSG00000024330 | Col11a2 |  | 6764 | 0 | 0.034475201 | 0.050061312 | 0.067358138 | 0.085390758 | 0.007325951 | 0.18186423 | 0.208917636 | 0.140048767 | 0.006970831 | 0.098883711 | 0.189793057 | 0.10939958 | 0.178525469 | 0.042639103 | 0.096236003 | 0.158654097 | 0.00813263 |
| ENSMUSG00000024331 | Dsc2 |  | 5707 | 6.286029187 | 9.409564654 | 6.848749348 | 8.007311629 | 6.780807189 | 9.342691665 | 3.738920308 | 3.058336762 | 5.434281824 | 5.361977644 | 3.365259034 | 5.134610629 | 5.57544886 | 3.507517376 | 4.425539471 | 5.615776835 | 6.205273933 | 5.821885485 |
| ENSMUSG00000024334 | H2-Oa |  | 1416 | 1.170693408 | 1.505667535 | 1.332321811 | 0.546989939 | 0.815795316 | 1.014851182 | 1.503580898 | 1.267685673 | 2.297835272 | 0.599373346 | 0.776005682 | 0.98544602 | 1.916140855 | 1.803972321 | 1.30937148 | 1.784731701 | 1.245063317 | 1.165447176 |
| ENSMUSG00000024335 | Brd2 |  | 7171 | 25.4580576 | 26.77202753 | 24.65559245 | 23.75578647 | 25.61979538 | 22.82424753 | 24.48436083 | 26.0439057 | 26.0409514 | 25.9588588 | 26.2825563 | 25.17970555 | 25.91456315 | 31.96876521 | 28.48658682 | 26.65551696 | 26.44519831 | 27.57742835 |
| ENSMUSG00000024337 | Psmb9 |  | 3965 | 0 | 0 | 0 | 0 | 0 | 0 | 0 | 0 | 0 | 0 | 0 | 0 | 0 | 0 | 0 | 0 | 0 | 0 |
| ENSMUSG00000024338 | Psmb8 |  | 2730 | 10.43167324 | 9.432552099 | 11.19856087 | 10.46402007 | 10.06715327 | 13.68599308 | 7.469510849 | 8.268025576 | 10.19856581 | 8.860187096 | 9.572491681 | 9.83418817 | 7.119697951 | 6.209557471 | 8.270495468 | 9.958363097 | 6.724650629 | 10.29657639 |
| ENSMUSG00000024339 | Tap2 |  | 4062 | 5.430868134 | 6.372259677 | 7.073824971 | 7.391612067 | 7.145137856 | 8.905341091 | 4.379509423 | 6.205560214 | 6.043122451 | 6.535166404 | 7.174476778 | 8.519377352 | 5.683736407 | 5.362451252 | 6.522058207 | 6.994493031 | 6.302798211 | 6.473252834 |
| ENSMUSG00000024340 | Btnl2 |  | 2555 | 0 | 0 | 0 | 0 | 0 | 0 | 0 | 0 | 0.354639275 | 0 | 0 | 0.065536982 | 0 | 0.4362654 | 0 | 0 | 0 | 0 |
| ENSMUSG00000024346 | Pfdn1 |  | 1073 | 54.50803561 | 48.49463259 | 54.91049392 | 42.54628744 | 49.29821446 | 46.13530368 | 57.27788437 | 42.17893462 | 62.45150153 | 51.50104773 | 56.14559426 | 52.79859974 | 61.00974299 | 65.53247745 | 60.20879742 | 57.3466972 | 60.36481059 | 62.0839033 |
| ENSMUSG00000024347 | Psd2 |  | 4926 | 0 | 0 | 0 | 0 | 0 | 0 | 0 | 0.139558109 | 0 | 0.02871541 | 0.00969853 | 0 | 0.370540183 | 0.103711929 | 0 | 0.046638986 | 0 | 0 |
| ENSMUSG00000024349 | Tmem173 |  | 2956 | 8.713848671 | 10.26659223 | 9.622339597 | 7.367448983 | 9.688259331 | 11.09739839 | 7.298572933 | 13.30790747 | 12.53987286 | 9.012228464 | 11.58817498 | 11.385942 | 5.874436699 | 4.666405262 | 6.857633795 | 9.689233537 | 10.34654429 | 6.457428969 |
| ENSMUSG00000024350 | Dnajc18 |  | 5161 | 6.415720234 | 6.209456353 | 6.879707669 | 6.98290759 | 7.721998217 | 6.423324698 | 5.67458792 | 7.303991702 | 6.855108901 | 6.596165036 | 7.572159666 | 6.153674096 | 5.821185356 | 5.561411743 | 5.157179831 | 5.794412784 | 6.965717205 | 5.8728963 |
| ENSMUSG00000024352 | Spata24 |  | 961 | 24.9458057 | 23.67607362 | 17.41648439 | 19.01142433 | 20.28452558 | 17.27384573 | 26.38875745 | 23.44798899 | 24.2576149 | 21.73544385 | 22.47063604 | 20.15404258 | 34.85574592 | 31.22047914 | 29.49705562 | 29.08651759 | 31.98510938 | 33.88698354 |
| ENSMUSG00000024353 | 2010001M09R |  | 901 | 0.094351112 | 0.221839473 | 0 | 0.303403181 | 0.427364472 | 0.054997485 | 4.673505466 | 0.805389174 | 3.565536326 | 0.732641338 | 6.521997661 | 0.743382854 | 2.956629892 | 2.835099674 | 1.143217422 | 1.869907576 | 0.297762573 | 0 |
| ENSMUSG00000024354 | Slc23a1 |  | 3222 | 0.342996455 | 0.351532714 | 0.42037829 | 0.749452312 | 0.448155714 | 0.338348895 | 0.469896253 | 0.806046978 | 0.460184534 | 0.439019583 | 0.919319499 | 0.346465938 | 1.822004394 | 2.335177578 | 1.189244164 | 0.68927847 | 0.951615069 | 1.980464426 |

| ENSMUSG00000024357 | Sil1 | 1685 | 87.6085913 | 91.33861798 | 98.55568529 | 93.47449868 | 102.3196146 | 100.0171198 | 88.50419227 | 81.77933999 | 99.28761248 | 95.89641547 | 95.29473914 | 96.99006531 | 96.81937711 | 82.30388424 | 91.74376125 | 81.69420392 | 83.04410089 | 87.42693638 |
| --- | --- | --- | --- | --- | --- | --- | --- | --- | --- | --- | --- | --- | --- | --- | --- | --- | --- | --- | --- | --- |
| ENSMUSG00000024359 | Hspa9 | 4011 | 112.5947383 | 116.1092648 | 114.2826162 | 111.4206641 | 117.5878135 | 119.2922452 | 105.9256686 | 106.169356 | 106.0930144 | 113.7917745 | 119.4671737 | 100.1370002 | 97.93818431 | 90.20180523 | 104.5087583 | 102.0891936 | 112.2078545 | 110.4845085 |
| ENSMUSG00000024360 | Etf1 | 3687 | 27.31075726 | 29.03923027 | 30.92389776 | 26.12314828 | 29.82952145 | 28.4118467 | 27.92307821 | 24.6639843 | 29.08860511 | 25.7685563 | 25.06014909 | 27.02222884 | 19.06644484 | 27.13332363 | 23.36654553 | 28.06108522 | 27.92092039 | 25.2740512 |
| ENSMUSG00000024365 | Cyp21a1 | 2131 | 0 | 0.015632517 | 0.136199522 | 0.085520496 | 0.338798149 | 0 | 0 | 0 | 0 | 0.110630463 | 0 | 0 | 0.347244842 | 0 | 0.019334376 | 0 | 0 | 0 |
| ENSMUSG00000024366 | Gfra3 | 1984 | 0 | 0 | 0 | 0 | 0 | 0 | 0 | 0 | 0 | 0 | 0 | 0 | 0 | 0 | 0.020766913 | 0 | 0 | 0 |
| ENSMUSG00000024369 | Rdbp | 2186 | 30.41083957 | 37.534153 | 32.39654549 | 34.82731248 | 31.57418963 | 30.85145032 | 37.55146292 | 32.84616492 | 31.33265129 | 36.04246347 | 37.45941392 | 29.56749189 | 36.5814446 | 42.13111862 | 37.31888411 | 38.97374267 | 37.44966614 | 40.76612667 |
| ENSMUSG00000024370 | Cdc23 | 9483 | 7.211940127 | 8.525824557 | 9.549219532 | 9.387986991 | 9.323878918 | 10.5501391 | 7.204419316 | 8.429473609 | 8.16520612 | 9.243188585 | 10.24720717 | 7.11012553 | 7.433861625 | 6.528521406 | 10.01037486 | 8.382505638 | 9.445170923 | 7.79049144 |
| ENSMUSG00000024371 | C2 | 4746 | 0.098516 | 0.070191518 | 0.030577453 | 0.047999415 | 0.030424731 | 0.093968521 | 0.059813753 | 0.064378259 | 0.225631987 | 0.11921796 | 0.080530903 | 0.035281708 | 0.0519721 | 0.127217264 | 0.190989092 | 0.225903289 | 0.25034033 | 0.069543751 |
| ENSMUSG00000024376 | Epb4.1l4a | 3829 | 29.69478868 | 27.38808854 | 29.05696522 | 29.66405942 | 27.81821548 | 28.66526636 | 29.25996963 | 29.42480729 | 32.55982847 | 36.94231125 | 32.11615125 | 26.34079454 | 39.48872459 | 35.22424387 | 43.70872813 | 39.86063231 | 42.60034464 | 41.40408607 |
| ENSMUSG00000024378 | Stard4 | 5989 | 23.3994933 | 29.43037624 | 36.29832116 | 30.84062009 | 28.46606673 | 35.37116982 | 28.39234731 | 32.82289116 | 36.6408008 | 34.04235112 | 35.38649454 | 31.78016521 | 27.81664462 | 29.60820265 | 37.56228004 | 34.46090731 | 41.18685037 | 41.02031568 |
| ENSMUSG00000024379 | Tslp | 1128 | 0.075363787 | 0.295327076 | 0.042884335 | 0.323127974 | 0.08534029 | 0.087859457 | 0.251663186 | 0.033858513 | 0.146051474 | 0.083600538 | 0 | 0 | 1.09334923 | 0.4529122 | 0.292209616 | 0 | 0.033977214 | 0.146300816 |
| ENSMUSG00000024380 | Gypc | 314 | 0 | 0 | 0 | 0 | 0 | 0 | 0 | 0 | 0 | 0 | 0 | 0 | 0 | 0 | 0 | 0 | 0 | 0 |
| ENSMUSG00000024381 | Bin1 | 2432 | 15.41511729 | 15.23188255 | 16.62840106 | 15.43680121 | 12.8246244 | 15.28147623 | 14.64903258 | 11.00858309 | 17.57878428 | 12.17542831 | 18.0727639 | 15.83585822 | 17.22154513 | 12.85233175 | 14.29856627 | 13.65047558 | 12.02424529 | 11.87490996 |
| ENSMUSG00000024382 | Ercc3 | 2888 | 9.787376059 | 12.5154052 | 10.20065096 | 11.3744851 | 11.23300435 | 11.25574558 | 8.764641006 | 11.65079866 | 9.669133548 | 8.097904715 | 8.320915552 | 10.01125804 | 10.12944146 | 10.34056378 | 10.59998477 | 11.57468846 | 11.28024666 | 8.418983785 |
| ENSMUSG00000024383 | Map3k2 | 10774 | 2.939145734 | 3.061051167 | 2.707373195 | 2.791005129 | 2.537491428 | 2.722778752 | 2.107860208 | 2.40696503 | 2.477154466 | 2.393858797 | 2.478763856 | 3.165339692 | 2.747275883 | 2.340740621 | 2.2639058 | 2.875171838 | 3.589310709 | 2.512017868 |
| ENSMUSG00000024384 | Iws1 | 9609 | 5.777058996 | 5.550415615 | 4.908335113 | 4.954864328 | 5.494923537 | 5.156908479 | 4.751455424 | 4.888818291 | 4.903462799 | 4.990349174 | 4.768049244 | 4.571435785 | 4.692410489 | 5.365067853 | 4.360701669 | 5.064768655 | 4.933877539 | 4.104644554 |
| ENSMUSG00000024386 | Proc | 1633 | 0.052057778 | 0.122398876 | 0.444337388 | 0.558004217 | 0.206321779 | 0 | 0 | 1.28633321 | 0.529649009 | 0.230989361 | 0.263303507 | 0 | 0.302093798 | 0.199086719 | 0.151383549 | 0 | 0.02346987 | 0.976891669 |
| ENSMUSG00000024387 | Csnk2b | 2309 | 60.50866765 | 55.40126193 | 53.54817825 | 54.93371478 | 56.44922014 | 52.29970192 | 53.19346665 | 52.30159221 | 58.72066258 | 52.78673362 | 49.24400224 | 45.25202268 | 55.44232366 | 47.97277974 | 50.49822571 | 56.91354032 | 52.13637877 | 49.88699414 |
| ENSMUSG00000024388 | Myo7b | 6574 | 0 | 0.050673706 | 0.088299721 | 0.069304905 | 0.08053714 | 0.045226103 | 0.30946839 | 0.453149892 | 0.125301234 | 0.064550704 | 0 | 0.101884386 | 0.187602363 | 0.254333305 | 0.00626735 | 0 | 0.04663985 | 0 |
| ENSMUSG00000024391 | Apom | 1160 | 0 | 0 | 0.166805277 | 0 | 0 | 0 | 0 | 0.131697939 | 0 | 0 | 0.205926545 | 0 | 0.297692604 | 0.760722121 | 0.284148661 | 0.693192031 | 0.429518845 | 0.331951506 |
| ENSMUSG00000024392 | Bag6 | 6264 | 13.24554655 | 13.16774044 | 14.34062035 | 12.6122048 | 14.6685572 | 15.67904182 | 11.76774485 | 13.66975833 | 12.92667465 | 14.06090576 | 12.60728071 | 15.20139214 | 13.49854825 | 12.82699093 | 12.82615485 | 11.91385597 | 11.64962792 | 12.96191595 |
| ENSMUSG00000024393 | Prrc2a | 7808 | 6.799303901 | 6.809346718 | 7.911500802 | 6.161944521 | 6.287725684 | 5.895810642 | 5.308133548 | 7.332276025 | 6.155822732 | 6.745304244 | 6.10036291 | 6.340739333 | 7.727066448 | 6.204044973 | 6.701585039 | 7.699315269 | 5.232560515 | 5.593907625 |
| ENSMUSG00000024395 | Lims2 | 1678 | 0.633271395 | 0.238232855 | 0 | 0.407279896 | 0.573682046 | 0.442962457 | 0 | 0.113803344 | 0.319085045 | 0.168596078 | 0 | 0.465684908 | 0.205794649 | 0.304460644 | 0.417417432 | 0.433564685 | 0.525330649 | 0.229477799 |
| ENSMUSG00000024397 | Aif1 | 1726 | 0.344769678 | 0.212306974 | 0.392369308 | 0.818303809 | 0.864478351 | 0.574191583 | 0.493411484 | 0.774469342 | 0.286348893 | 0.30049695 | 0.304475402 | 0.35571975 | 0.685959452 | 0.026908509 | 0.167097851 | 0.332768894 | 0.333079058 | 0.509933782 |
| ENSMUSG00000024399 | Ltb | 1335 | 0.636781663 | 0.274488267 | 0.579757667 | 0.204768739 | 1.009508511 | 0.482535982 | 1.063206268 | 0.457736658 | 1.264904229 | 0.423826546 | 0.644156744 | 1.379713008 | 1.219440652 | 0.521843679 | 1.049328015 | 1.003873602 | 0.114835347 | 0.082410647 |
| ENSMUSG00000024400 | Wdr33 | 6390 | 18.45216875 | 18.51759003 | 18.84220924 | 17.36168121 | 18.77822937 | 19.05337502 | 17.29615305 | 19.49067672 | 18.47914565 | 17.78297259 | 20.03707179 | 17.94137261 | 16.43625225 | 18.3595876 | 19.11777985 | 19.96025247 | 20.74658231 | 19.43827277 |
| ENSMUSG00000024401 | Tnf | 2691 | 0.063181235 | 0.111414362 | 0.323568765 | 0.457134224 | 1.019516778 | 0.257799431 | 0.087909102 | 0.46835722 | 0.811179983 | 0.438040721 | 0.088768039 | 1.721553324 | 0.219986253 | 0.17259044 | 0.658367485 | 0.327270149 | 0.541211179 | 0.654140251 |
| ENSMUSG00000024402 | Lta | 1379 | 0.092469564 | 0.072471851 | 0.31570832 | 0.066078382 | 0.104710494 | 0.107801451 | 0 | 0.16617434 | 0 | 0.068383906 | 0.27715712 | 0.647607398 | 1.323626497 | 0.404154495 | 0 | 0.360970193 | 0.667027647 | 0.119671733 |
| ENSMUSG00000024403 | Atp6v1g2 | 2504 | 6.026093247 | 6.745062847 | 5.660321244 | 6.677677025 | 7.304365413 | 5.343146193 | 5.649557108 | 6.787387808 | 6.266798125 | 4.406255816 | 4.426433853 | 7.177573224 | 6.580215803 | 6.36194967 | 7.14116424 | 6.820132149 | 5.984657384 | 5.865587658 |
| ENSMUSG00000024404 | Riok3 | 3648 | 53.00330201 | 61.87723992 | 64.246095 | 59.42419105 | 59.08299183 | 63.27210335 | 57.13057858 | 59.64422053 | 59.98463768 | 67.90838676 | 61.34262761 | 55.47905497 | 62.69264487 | 65.01917607 | 73.69521716 | 66.5887098 | 76.49500212 | 72.32008658 |
| ENSMUSG00000024406 | Pou5f1 | 2250 | 0 | 0 | 0 | 0 | 0 | 0 | 0 | 0 | 0 | 0 | 0 | 0 | 0 | 0 | 0 | 0 | 0 | 0 |
| ENSMUSG00000024409 | Psors1c2 | 841 | 0 | 0 | 0 | 0 | 0.171695328 | 0 | 0 | 0.090826165 | 0.146919795 | 0 | 0 | 0.26547283 | 0.293293206 | 0.110449673 | 0.097982297 | 0.273179125 | 0 | 0.196227491 |
| ENSMUSG00000024410 | 3110002H16R | 3151 | 19.39779217 | 18.87131583 | 16.4110771 | 17.22094694 | 15.39732753 | 19.45310423 | 15.90102411 | 14.41154124 | 15.60669623 | 14.8889082 | 14.26729163 | 16.0308393 | 17.94172555 | 11.46731196 | 13.25876791 | 12.52859925 | 14.64451332 | 14.19308276 |
| ENSMUSG00000024411 | Aqp4 | 5307 | 0 | 0.031385806 | 0.027345127 | 0 | 0.009069516 | 0 | 0 | 0 | 0 | 0.035538499 | 0.009002253 | 0.126208395 | 0.16732137 | 0.21878692 | 0 | 0.331895221 | 0.209433316 | 0.124384639 |
| ENSMUSG00000024413 | Npc1 | 7241 | 10.12000048 | 8.994159398 | 8.905111996 | 7.317703986 | 9.910882225 | 7.732991154 | 7.853865498 | 5.944322264 | 9.009728072 | 6.635349623 | 7.45555905 | 7.076201941 | 9.994465218 | 8.511435432 | 9.707202645 | 9.687666449 | 10.15718323 | 10.9547206 |
| ENSMUSG00000024414 | Mrpl27 | 1045 | 43.19664779 | 36.15006894 | 33.19025958 | 32.61211595 | 34.91291688 | 33.57256974 | 43.5095545 | 41.59134348 | 40.55590882 | 38.08152493 | 42.74601548 | 40.27273645 | 45.74414147 | 40.66635403 | 40.13701808 | 37.22799724 | 38.47300043 | 45.90233594 |
| ENSMUSG00000024420 | Zfp521 | 6173 | 2.086354825 | 2.90873967 | 3.933826703 | 4.649839289 | 4.56914098 | 4.390951769 | 4.414725913 | 9.509431799 | 7.886353739 | 10.67822504 | 11.71736386 | 10.56094183 | 1.75814382 | 1.595034266 | 4.585366742 | 1.532120529 | 3.340279886 | 3.59123117 |
| ENSMUSG00000024421 | Lama3 | 10456 | 0.581316007 | 0.720037691 | 0.786486243 | 0.771260984 | 0.874623709 | 0.928876796 | 0.221721621 | 0.080358919 | 0.263915126 | 0.144302076 | 0.059398858 | 0.080072201 | 0.061334633 | 0.373116234 | 0.193083037 | 0.018310352 | 0.256583855 | 0.336704555 |
| ENSMUSG00000024422 | Dhx16 | 4339 | 9.854852981 | 12.95970626 | 11.82860468 | 13.45095593 | 12.57930432 | 12.91637282 | 9.050362644 | 9.858375362 | 8.504982282 | 11.29052332 | 10.52612591 | 9.660650519 | 7.810792157 | 7.642562433 | 10.21730218 | 9.168919357 | 10.10492825 | 10.94096776 |
| ENSMUSG00000024423 | Impact | 3421 | 14.56184341 | 14.87930499 | 16.19049759 | 17.89945735 | 15.23732047 | 17.4977206 | 19.52806274 | 15.56276204 | 17.13195321 | 17.71080201 | 19.97023988 | 20.72081014 | 21.71700305 | 21.6675719 | 28.15821037 | 27.40000202 | 32.17571608 | 30.40696311 |
| ENSMUSG00000024424 | Ttc39c | 5182 | 1.058118045 | 1.478573074 | 1.222873886 | 1.22211215 | 1.28178415 | 0.831935126 | 2.748189185 | 2.402686836 | 2.32081486 | 1.683303764 | 1.299936153 | 1.421780676 | 6.416385998 | 9.679585948 | 4.985213343 | 2.187190234 | 2.337149715 | 4.564630623 |

| ENSMUSG00000024425 | Ndfip1 |  | 922 | 1268.516728 | 1385.989829 | 1374.028619 | 1425.14156 | 1422.971774 | 1387.315306 | 1317.211665 | 1342.699058 | 1365.0069 | 1425.618389 | 1460.037966 | 1362.640726 | 1312.53933 | 1312.977373 | 1399.333537 | 1557.99581 | 1493.603226 | 1373.55711 |
| --- | --- | --- | --- | --- | --- | --- | --- | --- | --- | --- | --- | --- | --- | --- | --- | --- | --- | --- | --- | --- | --- |
| ENSMUSG00000024426 | Atat1 |  | 4613 | 15.24952663 | 14.89078428 | 17.5331764 | 15.06191039 | 13.5850346 | 13.55637324 | 13.76406137 | 14.828223 | 13.58003262 | 13.8089313 | 12.90431352 | 17.54448531 | 10.04176682 | 9.010938264 | 9.48537879 | 9.877698419 | 8.765281429 | 9.53984075 |
| ENSMUSG00000024427 | Spry4 |  | 4585 | 0.092704855 | 0.225234399 | 0.168806213 | 0.04968489 | 0.188958479 | 0.183728783 | 0.712011962 | 0.274885339 | 0.37728106 | 0.308510599 | 1.167021887 | 0.584329728 | 1.667709308 | 2.015784818 | 0.97050557 | 4.910551178 | 3.66126893 | 2.927420292 |
| ENSMUSG00000024429 | Gnl1 |  | 3357 | 25.69049811 | 28.79774174 | 24.8712283 | 29.2475575 | 25.33485526 | 29.18253032 | 24.67813548 | 28.1238066 | 24.32912146 | 28.21738542 | 24.4211584 | 28.49807735 | 20.83784888 | 26.61853564 | 24.8289388 | 22.70974064 | 23.41591747 | 22.13801108 |
| ENSMUSG00000024430 | Cabyr |  | 2208 | 1.097280359 | 1.433299344 | 0.920148675 | 1.320609981 | 0.871955139 | 0.673270837 | 1.071392186 | 1.764322937 | 1.436305124 | 0.875533892 | 1.882437223 | 2.426767939 | 2.636397753 | 3.723099397 | 0.522483499 | 1.369999087 | 0.902612065 | 2.366786748 |
| ENSMUSG00000024431 | Nr3c1 |  | 11456 | 7.687737843 | 5.969917234 | 7.427465075 | 6.375205488 | 6.247570748 | 6.384412951 | 6.434458255 | 6.557651495 | 8.711149414 | 7.815920521 | 6.801759539 | 7.517769498 | 6.536823536 | 5.404152277 | 6.124847214 | 7.226282577 | 7.376875408 | 7.140235833 |
| ENSMUSG00000024436 | Mrps18b |  | 2651 | 7.583911075 | 6.132286827 | 6.623761418 | 6.599562816 | 7.552953695 | 7.140378811 | 6.35356986 | 7.678819495 | 6.944689757 | 6.509678383 | 6.812121579 | 5.705788216 | 6.438643296 | 6.709953025 | 7.056019002 | 7.597457372 | 7.257563578 | 8.009624744 |
| ENSMUSG00000024437 | Gm8615 |  | 635 | 0 | 0 | 0.076178788 | 0.071749676 | 0 | 0.156071602 | 0.074508156 | 0.060145516 | 0.129721309 | 0 | 0 | 0 | 0.155376117 | 0 | 0.19465302 | 0.180900507 | 0.181069119 | 0.086628515 |
| ENSMUSG00000024440 | Pcdh12 |  | 5368 | 0 | 0 | 0 | 0 | 0.017932907 | 0 | 0.017627675 | 0 | 0 | 0 | 0 | 0 | 0 | 0 | 0 | 0 | 0 | 0 |
| ENSMUSG00000024442 | 0610009O20R |  | 2391 | 8.390816849 | 9.446316303 | 10.35853097 | 9.603833687 | 10.66914243 | 8.600746313 | 9.240912195 | 10.94177986 | 11.19666886 | 9.40647656 | 11.24939424 | 9.827851923 | 8.541787433 | 10.25615982 | 8.288560595 | 7.654918585 | 8.543670535 | 8.926613722 |
| ENSMUSG00000024446 | Rpp21 |  | 1346 | 59.68408818 | 50.71182783 | 54.59093062 | 41.73608296 | 54.92617738 | 54.55954767 | 55.99487187 | 48.83293056 | 52.23282497 | 43.85786068 | 53.31202647 | 51.29567208 | 51.09115355 | 57.69280395 | 52.46617147 | 47.90593081 | 44.87536695 | 49.24663715 |
| ENSMUSG00000024448 | H2-M10.1 |  | 1435 | 0 | 0 | 0 | 0 | 0 | 0 | 0 | 0 | 0 | 0 | 0 | 0 | 0 | 0 | 0 | 0 | 0 | 0 |
| ENSMUSG00000024451 | Arap3 |  | 6216 | 0 | 0 | 0 | 0 | 0 | 0 | 0.007611435 | 0.098307342 | 0 | 0.037926885 | 0.046114825 | 0.062855476 | 0.039681401 | 0.0896604 | 0.059654762 | 0 | 0 | 0 |
| ENSMUSG00000024454 | Hdac3 |  | 4726 | 29.3740806 | 32.57276432 | 32.13984031 | 32.17037769 | 31.3478758 | 31.35054452 | 33.65747494 | 33.10924093 | 32.69822411 | 33.38261809 | 31.11538768 | 29.33688232 | 34.90603711 | 33.89455298 | 38.35074997 | 34.86341169 | 33.11177957 | 35.86184042 |
| ENSMUSG00000024456 | Diap1 |  | 8175 | 7.804314277 | 6.222481893 | 6.822590887 | 5.54535035 | 4.60417912 | 5.709929667 | 5.527046896 | 5.723020544 | 6.791366758 | 5.092852719 | 5.27131652 | 6.254085251 | 6.511209632 | 4.863136245 | 5.125624751 | 6.993012459 | 5.499296179 | 4.764091445 |
| ENSMUSG00000024457 | Trim26 |  | 2217 | 62.96211009 | 55.92719544 | 63.47253036 | 65.9268517 | 55.6437169 | 62.22589548 | 51.53816852 | 60.01909336 | 52.35164701 | 63.46310263 | 58.65739151 | 61.12784337 | 56.34118834 | 46.25554576 | 60.54788863 | 52.66037978 | 51.82780249 | 62.67614054 |
| ENSMUSG00000024459 | H2-M5 |  | 2427 | 0.665511615 | 0.192163378 | 0.27903973 | 0.37545154 | 0.079327439 | 0.183755502 | 1.208646928 | 0.849769151 | 0.916387248 | 1.146226408 | 0.629912926 | 0.896914232 | 0.142284063 | 1.205594357 | 0.441384612 | 0.804623394 | 0.347415959 | 0.725295185 |
| ENSMUSG00000024462 | Gabbr1 |  | 7178 | 2.315341854 | 3.239398184 | 3.019133686 | 3.122880163 | 1.67636959 | 2.540457782 | 2.386067118 | 3.628757097 | 3.155835005 | 3.139876868 | 2.935184827 | 3.37475586 | 3.587525887 | 2.180503964 | 2.760929003 | 3.680763319 | 2.813869945 | 3.188045193 |
| ENSMUSG00000024471 | Myot |  | 2199 | 17.29974195 | 21.58748261 | 18.17032109 | 17.59041684 | 23.28893442 | 21.72298098 | 8.477123919 | 14.22445547 | 16.25734228 | 5.231819734 | 14.4476341 | 15.99084467 | 12.1591174 | 15.7981707 | 12.92818261 | 11.59688246 | 15.07605585 | 16.78540728 |
| ENSMUSG00000024472 | Dcp2 |  | 8632 | 2.457145834 | 1.701921496 | 1.776460742 | 2.15348773 | 1.739708084 | 2.078091932 | 1.95674541 | 1.893691871 | 1.669981524 | 1.982820352 | 2.064418387 | 1.920441588 | 2.566036939 | 2.663325215 | 2.587030039 | 3.122866961 | 2.464213946 | 2.765749805 |
| ENSMUSG00000024474 | Ik |  | 1976 | 60.8538173 | 60.91067146 | 52.90242869 | 65.36718658 | 56.097075 | 57.22638564 | 66.22817303 | 61.34751277 | 61.96736405 | 63.591409 | 62.91014269 | 54.51631012 | 58.294548 | 61.13414539 | 64.15849569 | 64.64446675 | 66.5279344 | 56.7907782 |
| ENSMUSG00000024477 | Pggt1b |  | 2727 | 9.165032455 | 9.55287249 | 6.971322856 | 7.885886662 | 8.754467964 | 9.558026352 | 8.848355795 | 9.159454042 | 7.113622634 | 7.65961186 | 6.412040632 | 6.283611446 | 5.209971462 | 7.306385585 | 6.557196647 | 8.452858693 | 8.067214676 | 7.483820533 |
| ENSMUSG00000024479 | Mal2 |  | 2771 | 9.495021278 | 11.82962393 | 9.863242386 | 10.67091944 | 14.13907826 | 12.0350017 | 9.561566294 | 10.84713847 | 7.342525726 | 6.465993229 | 8.275705547 | 11.0986756 | 4.913608505 | 6.989240145 | 3.895636104 | 4.739689043 | 5.947422471 | 4.843818853 |
| ENSMUSG00000024480 | Ap3s1 |  | 1473 | 12.78329462 | 12.57431717 | 13.20173741 | 10.70205115 | 9.933540255 | 10.1931285 | 14.26125556 | 10.34539617 | 13.84068249 | 10.59530399 | 12.16266764 | 10.34465437 | 10.85101235 | 9.616732284 | 9.118606395 | 11.56776666 | 11.37039494 | 9.597651343 |
| ENSMUSG00000024481 | 4833403I15Ri | k | 3199 | 0 | 0 | 0 | 0 | 0 | 0 | 0 | 0 | 0.01287481 | 0 | 0 | 0 | 0 | 0.043554943 | 0 | 0 | 0 | 0 |
| ENSMUSG00000024483 | Ankhd1 |  | 9714 | 10.25655061 | 10.93968834 | 11.40866358 | 11.05490853 | 11.15348571 | 12.40093631 | 10.77856274 | 11.40975415 | 9.662761933 | 10.57662985 | 11.04617632 | 10.39433124 | 12.08667522 | 11.10182941 | 10.45096085 | 11.65589109 | 13.21337949 | 10.37437549 |
| ENSMUSG00000024485 | Slc4a9 |  | 3419 | 1.442117701 | 3.64405453 | 1.428992853 | 2.158785956 | 2.449533407 | 2.087041133 | 2.532383809 | 2.502222328 | 1.686490847 | 1.999664221 | 2.277659617 | 0.979508557 | 3.289756402 | 4.496341891 | 3.193451978 | 5.218901155 | 3.878589859 | 2.381207311 |
| ENSMUSG00000024486 | Hbegf |  | 2367 | 3.860841929 | 4.447348785 | 2.534143542 | 2.579290216 | 4.656616189 | 3.705464235 | 3.378049319 | 15.36086485 | 4.941685879 | 4.103525505 | 5.045939848 | 5.164186779 | 6.335827145 | 11.36084774 | 8.181128546 | 36.94792873 | 19.54365879 | 18.9406092 |
| ENSMUSG00000024487 | Yipf5 |  | 2323 | 52.80668877 | 47.89714451 | 55.30783324 | 47.0320208 | 48.77423518 | 53.84033558 | 44.95010004 | 45.57440356 | 48.20754901 | 43.8625354 | 46.10910758 | 41.95184971 | 40.41267265 | 39.50646435 | 44.32315462 | 37.77962642 | 46.92207852 | 45.08710254 |
| ENSMUSG00000024491 | Rbm27 |  | 6410 | 4.515760511 | 5.285368708 | 4.943005052 | 4.449487325 | 4.53536379 | 5.852015494 | 6.399390426 | 5.713964728 | 4.394941776 | 4.943100248 | 3.912925616 | 4.893666517 | 4.34828912 | 2.912718117 | 5.007178165 | 4.844568269 | 5.112165968 | 5.071822476 |
| ENSMUSG00000024493 | Lars |  | 3829 | 20.13695203 | 23.05541125 | 18.35642194 | 23.39331762 | 19.43378271 | 21.05570581 | 14.28400544 | 17.17610785 | 14.80089989 | 16.02064898 | 16.10798417 | 14.05231098 | 12.2911071 | 15.55009664 | 13.17072458 | 15.65024826 | 13.49277833 | 18.05861769 |
| ENSMUSG00000024497 | Pou4f3 |  | 2358 | 0 | 0 | 0 | 0 | 0 | 0 | 0 | 0 | 0 | 0 | 0 | 0 | 0 | 0 | 0 | 0 | 0 | 0 |
| ENSMUSG00000024498 | Tcerg1 |  | 4555 | 10.97389396 | 14.10769988 | 14.05009455 | 15.4137364 | 14.99433583 | 15.15410711 | 13.90816182 | 11.89791855 | 12.09825644 | 13.38438185 | 13.9391701 | 11.01608796 | 11.30681046 | 13.33674343 | 14.87055058 | 12.27316944 | 14.8172577 | 16.62953676 |
| ENSMUSG00000024500 | Ppp2r2b |  | 3970 | 0.770874729 | 1.510408302 | 1.389063592 | 2.226408716 | 0.933541089 | 1.173288906 | 3.110480907 | 3.501771908 | 2.562485993 | 2.981064614 | 3.57409639 | 4.133452091 | 8.300685325 | 5.65050232 | 6.994924098 | 7.706346427 | 6.931556971 | 5.556335465 |
| ENSMUSG00000024501 | Dpysl3 |  | 7211 | 10.99322608 | 14.4181865 | 8.083498 | 14.52570253 | 6.721515342 | 10.12219894 | 4.717489412 | 5.487079308 | 3.918173598 | 7.49991078 | 3.166888109 | 6.393528955 | 6.765950102 | 4.888512318 | 3.056834337 | 12.43608411 | 4.443320512 | 4.157531988 |
| ENSMUSG00000024502 | Jakmip2 |  | 3516 | 0 | 0 | 0 | 0 | 0 | 0 | 0 | 0.05431229 | 0 | 0 | 0 | 0 | 0.070153466 | 0 | 0 | 0 | 0 | 0 |
| ENSMUSG00000024503 | Spink3 |  | 418 | 0.101687024 | 0 | 0 | 0 | 0 | 0 | 0 | 0 | 0 | 0 | 0 | 0 | 0 | 0 | 0 | 0 | 0 | 0 |
| ENSMUSG00000024505 | Dtwd2 |  | 5197 | 1.962909803 | 1.378155139 | 1.982598031 | 2.14786528 | 2.3061091 | 2.030927892 | 2.749360984 | 1.616765159 | 2.670743855 | 1.9869163 | 0.937665146 | 0.859198193 | 2.657867392 | 1.876709323 | 2.275321636 | 1.878796399 | 2.264031778 | 1.862921451 |
| ENSMUSG00000024507 | Hsd17b4 |  | 3278 | 42.06430477 | 45.96528998 | 40.40473646 | 45.1023761 | 43.94717741 | 43.49091352 | 40.02381289 | 40.60418621 | 34.52731706 | 38.18948052 | 35.22638031 | 35.38283919 | 32.38629835 | 38.25473946 | 38.09698472 | 39.45871623 | 40.50100441 | 36.02945459 |
| ENSMUSG00000024510 | Ftmt |  | 1450 | 0 | 0 | 0 | 0 | 0 | 0 | 0 | 0 | 0 | 0 | 0 | 0 | 0 | 0 | 0 | 0 | 0 | 0 |
| ENSMUSG00000024511 | Rab27b |  | 12391 | 0.006860653 | 0.024196275 | 0.011711774 | 0 | 0.023306557 | 0.047989089 | 0.06491127 | 0.00308227 | 0.059830303 | 0.053273331 | 0 | 0.01801813 | 0 | 0 | 0 | 0 | 0 | 0 |

| ENSMUSG00000024512 | 2310002L13R | 1038 | 0 | 0 | 0 | 0 | 0 | 0 | 0 | 0 | 0 | 0 | 0 | 0 | 0 | 0 | 0 | 0 | 0 | 0 |
| --- | --- | --- | --- | --- | --- | --- | --- | --- | --- | --- | --- | --- | --- | --- | --- | --- | --- | --- | --- | --- |
| ENSMUSG00000024513 | Mbd2 | 3880 | 50.33795975 | 60.08341076 | 52.01401254 | 57.16267109 | 60.16490458 | 57.87963623 | 47.60533467 | 48.8232773 | 45.4962388 | 46.05700138 | 40.89191678 | 46.40755864 | 42.31356203 | 45.24707483 | 48.27378172 | 40.60975501 | 48.67834823 | 49.97605701 |
| ENSMUSG00000024515 | Smad4 | 4166 | 46.1476023 | 51.8564856 | 50.5216588 | 54.0805003 | 48.89445534 | 55.19075463 | 36.98929315 | 41.44691581 | 36.82663735 | 38.49244882 | 45.19469788 | 39.37643599 | 33.00241321 | 30.27895856 | 35.78186011 | 35.57919857 | 44.35215491 | 41.80480842 |
| ENSMUSG00000024516 | Sec11c | 2463 | 37.93194353 | 38.95295792 | 33.03462365 | 30.35553137 | 31.95115518 | 32.25052047 | 42.3566614 | 40.51837088 | 33.76190634 | 36.9471609 | 33.57631065 | 41.67478826 | 39.89816452 | 45.99152625 | 33.20547643 | 32.27417822 | 35.68095769 | 34.46165314 |
| ENSMUSG00000024517 | Grp | 1054 | 0 | 0 | 0 | 0 | 0 | 0 | 0 | 0.108707028 | 0.078152781 | 0.17894005 | 0.045327285 | 0.052956037 | 0 | 0 | 0 | 0 | 0 | 0 |
| ENSMUSG00000024518 | Rax | 1666 | 0 | 0 | 0 | 0.082042697 | 0.115562842 | 0.208204763 | 0.085196901 | 0.068773834 | 0.074165395 | 0.113206971 | 0.028676446 | 0.134011195 | 0 | 0 | 0 | 0 | 0.06901494 | 0 |
| ENSMUSG00000024519 | Cplx4 | 1748 | 0 | 0 | 0 | 0 | 0 | 0 | 0 | 0.131095203 | 0 | 0 | 0 | 0.191586943 | 0 | 0 | 0 | 0 | 0 | 0 |
| ENSMUSG00000024521 | Pmaip1 | 2643 | 1.077505408 | 3.6552173 | 3.880131833 | 4.137211744 | 5.827550349 | 4.274696654 | 2.792575828 | 3.323591583 | 3.849061443 | 2.46189824 | 1.934135663 | 1.668345571 | 1.138572438 | 1.107066782 | 1.590071396 | 1.825431908 | 1.334097357 | 1.956434367 |
| ENSMUSG00000024524 | Gnal | 7906 | 0.360213356 | 0.324448881 | 0.391589419 | 0.507130268 | 0.505306054 | 0.463812583 | 0.520642938 | 0.695636978 | 0.640771748 | 0.578499648 | 0.404872529 | 1.023687209 | 0.555342858 | 0.963424024 | 0.698331456 | 0.634463644 | 0.615664016 | 0.605336742 |
| ENSMUSG00000024525 | Impa2 | 1653 | 0.925702563 | 0.886731607 | 0.79013026 | 1.185193531 | 0.727948029 | 1.378962943 | 0.286223103 | 1.409399 | 1.345476014 | 0.998351249 | 1.502902505 | 0.742858183 | 0.268594831 | 0 | 0.324029175 | 0.579107795 | 0.556461661 | 1.098185434 |
| ENSMUSG00000024526 | Cidea | 1159 | 0.036674009 | 2.845536262 | 0.166949199 | 0.157242603 | 0.041528838 | 0 | 0 | 0 | 0.035536252 | 0.122046687 | 0 | 0 | 5.150269189 | 0.681233378 | 2.595093684 | 7.63169138 | 2.083310358 | 2.088352628 |
| ENSMUSG00000024527 | Afg3l2 | 3601 | 26.0744054 | 25.02398747 | 23.28001336 | 26.40541503 | 27.26718253 | 25.23735445 | 26.0804409 | 27.21513595 | 26.28342494 | 29.3431619 | 29.65205004 | 26.31907667 | 23.68644404 | 26.6850366 | 27.52872632 | 24.53108339 | 29.55626951 | 30.73544091 |
| ENSMUSG00000024528 | Srfbp1 | 1455 | 10.86730274 | 9.799256856 | 11.43676594 | 10.17686557 | 11.41272417 | 9.229409484 | 14.53523538 | 9.082179541 | 15.42725161 | 13.31885845 | 12.28029861 | 9.973932833 | 11.86678422 | 12.99157633 | 12.62948036 | 9.526597855 | 12.32763364 | 14.13979788 |
| ENSMUSG00000024529 | Lox | 4688 | 0 | 0 | 0 | 0 | 0 | 0 | 0 | 0 | 0 | 0 | 0 | 0 | 0 | 0 | 0 | 0 | 0 | 0 |
| ENSMUSG00000024530 | Slmo1 | 2160 | 0.216461545 | 0.138803726 | 0 | 0.126558456 | 0.22283298 | 0.344116205 | 0.131424108 | 0.035363336 | 0 | 0.152803205 | 0 | 0 | 0 | 0.107509462 | 0.381495888 | 0 | 0.124205592 | 0.407474865 |
| ENSMUSG00000024532 | 1700034E13R | 576 | 0 | 0 | 0 | 0 | 0 | 0 | 0 | 0 | 0 | 0 | 0 | 0 | 0 | 0 | 0 | 0 | 0 | 0 |
| ENSMUSG00000024533 | Spire1 | 8945 | 4.590273901 | 4.219509128 | 4.191110791 | 4.599398886 | 3.868848867 | 4.509326454 | 5.405652081 | 4.133062664 | 6.206754975 | 5.12358676 | 4.609255357 | 3.712724342 | 4.693288049 | 5.228529458 | 4.859434485 | 4.199338833 | 5.462943382 | 5.405590253 |
| ENSMUSG00000024534 | Sncaip | 4000 | 0 | 0.008328224 | 0 | 0.125292872 | 0 | 0 | 0 | 0 | 0 | 0 | 0 | 0 | 0.110996814 | 0.046444087 | 0 | 0.114871822 | 0 | 0.110018213 |
| ENSMUSG00000024535 | Snx24 | 1956 | 41.98364013 | 41.96470928 | 41.72093339 | 42.99881792 | 47.02459414 | 40.40726486 | 32.92052966 | 28.66382757 | 34.51160498 | 29.09555155 | 31.53244959 | 31.61746122 | 27.1880403 | 30.89149166 | 35.32464685 | 33.84686104 | 35.15203305 | 34.76035578 |
| ENSMUSG00000024537 | Psmg2 | 1429 | 34.38487298 | 25.85304387 | 29.34909085 | 28.05718615 | 27.51839163 | 27.77588495 | 33.50624988 | 31.21674318 | 34.55747544 | 30.28995493 | 29.55427802 | 27.61488695 | 37.00756845 | 29.28350087 | 35.49273289 | 33.09230241 | 36.20748833 | 29.79499552 |
| ENSMUSG00000024538 | Ppic | 1281 | 32.28535228 | 30.76436679 | 33.68398835 | 17.96122357 | 33.25273415 | 24.95043962 | 14.44126265 | 23.55347221 | 16.71896034 | 12.88270581 | 17.75244359 | 18.82308059 | 19.71736272 | 23.5302207 | 21.38878584 | 21.19284984 | 18.93875561 | 18.72284975 |
| ENSMUSG00000024539 | Ptpn2 | 3475 | 20.67158201 | 15.39582967 | 17.80424326 | 18.00152917 | 16.56569229 | 17.86750365 | 20.88565453 | 20.49750518 | 21.03771668 | 18.04616844 | 19.79739287 | 20.30244533 | 15.06220553 | 16.19862846 | 17.77298771 | 20.72651296 | 22.41123318 | 18.28360238 |
| ENSMUSG00000024542 | Cep192 | 8050 | 1.557643097 | 2.338109967 | 1.904895543 | 1.448897807 | 2.020942882 | 1.520437912 | 1.592762235 | 1.546673687 | 0.977220436 | 1.376449101 | 1.311585817 | 1.671003066 | 0.692485298 | 0.848109422 | 0.752376238 | 1.598216657 | 1.314044465 | 1.059181558 |
| ENSMUSG00000024544 | D18Ertd653e | 2637 | 11.49267748 | 11.58434736 | 10.93311494 | 13.23464542 | 11.80938741 | 9.508412283 | 3.498662264 | 3.490469844 | 1.70243846 | 3.003912836 | 1.376904378 | 2.857457129 | 1.683683183 | 1.51467255 | 1.218703587 | 2.061913633 | 2.398118691 | 2.002606843 |
| ENSMUSG00000024546 | 4930546C10R | 360 | 0 | 0 | 0 | 0 | 0 | 0 | 0 | 0 | 0 | 0 | 0 | 0 | 0 | 0 | 0 | 0 | 0 | 0 |
| ENSMUSG00000024548 | Setbp1 | 9947 | 2.922845119 | 2.109894778 | 2.543415741 | 2.161939572 | 2.201671385 | 2.22680928 | 2.796808607 | 2.438139693 | 3.726537065 | 2.189969328 | 3.227583402 | 2.940324439 | 2.881456111 | 3.347784323 | 2.530828456 | 3.156559573 | 2.573837974 | 2.411176287 |
| ENSMUSG00000024552 | Slc14a2 | 4870 | 0.052367773 | 0.08208516 | 0.089396668 | 0.093554506 | 0 | 0.091575894 | 0.359459778 | 0.22742909 | 0.02537157 | 0.213001124 | 0.019620106 | 0.160455704 | 0.151946357 | 0.886919944 | 0.473775591 | 0 | 0.015739752 | 0.158137062 |
| ENSMUSG00000024553 | Galr1 | 2868 | 0 | 0.011615375 | 0 | 0 | 0 | 0 | 0 | 0 | 0 | 0 | 0 | 0 | 0 | 0 | 0 | 0 | 0 | 0 |
| ENSMUSG00000024556 | Me2 | 2439 | 10.75264191 | 13.82232429 | 12.9115081 | 14.19696338 | 12.51153735 | 13.22625238 | 15.11134764 | 15.43981499 | 12.4792272 | 14.71163804 | 13.90742949 | 12.19751872 | 9.425450369 | 12.01567001 | 12.82164039 | 14.41196293 | 17.7881788 | 14.02856268 |
| ENSMUSG00000024558 | Mapk4 | 7290 | 2.921137612 | 4.08528497 | 4.379496575 | 3.037402956 | 3.85583586 | 3.589004572 | 0.967021833 | 1.880805551 | 1.768364806 | 0.97664694 | 1.389340356 | 1.79927033 | 0.64287135 | 0.547900071 | 1.017322368 | 1.071506709 | 1.025189012 | 0.860224714 |
| ENSMUSG00000024560 | Cxxc1 | 2603 | 29.18046468 | 28.12975086 | 31.70389658 | 32.55610544 | 28.1062328 | 28.87879248 | 27.68237034 | 30.09320681 | 26.5030403 | 25.34146518 | 26.44783525 | 23.24401777 | 29.56503686 | 26.38909153 | 22.1282271 | 30.4059491 | 30.15453556 | 30.32580413 |
| ENSMUSG00000024561 | Mbd1 | 2880 | 18.90594809 | 18.13702019 | 22.2719796 | 24.33086326 | 20.97415424 | 21.6104977 | 21.04428531 | 17.69050861 | 18.205012 | 20.82489394 | 23.27370373 | 19.32229708 | 19.49295898 | 26.22155767 | 23.04712033 | 23.46629238 | 23.79424266 | 28.13486604 |
| ENSMUSG00000024563 | Smad2 | 3814 | 15.22340599 | 16.48175967 | 18.88520889 | 19.62684736 | 16.21644518 | 18.7219426 | 20.41863385 | 17.5240441 | 17.12685212 | 18.91467645 | 20.806032 | 17.97106284 | 14.42188982 | 14.125627 | 16.77661675 | 17.51890664 | 20.46949837 | 19.2402067 |
| ENSMUSG00000024565 | Sall3 | 6882 | 0 | 0 | 0 | 0 | 0 | 0 | 0 | 0 | 0 | 0 | 0 | 0 | 0 | 0 | 0 | 0 | 0 | 0 |
| ENSMUSG00000024566 | Atp9b | 4543 | 20.78945656 | 27.989284 | 27.67396113 | 28.68249764 | 27.80060922 | 28.80668487 | 23.80734845 | 25.72501681 | 24.668393 | 24.08910021 | 22.95679823 | 21.26720492 | 21.50059349 | 25.58871907 | 25.76570995 | 21.04592707 | 27.15658149 | 28.30977141 |
| ENSMUSG00000024570 | Rbfa | 1371 | 43.4972735 | 41.18552568 | 40.61118413 | 39.14727223 | 42.26902706 | 40.33614199 | 41.72212168 | 34.73809322 | 38.06222864 | 42.1983318 | 42.89640692 | 41.1594711 | 42.78311425 | 39.26236126 | 39.969416 | 37.36895164 | 33.32235296 | 35.14805069 |
| ENSMUSG00000024571 | Gm16286 | 2740 | 80.63573175 | 85.6652017 | 79.4808882 | 82.47546712 | 79.96223231 | 86.44600967 | 75.32040346 | 62.91989214 | 75.18793863 | 76.62849694 | 73.94656894 | 69.5047594 | 71.60329745 | 72.32734339 | 78.25288206 | 79.48794704 | 73.12769351 | 80.64656267 |
| ENSMUSG00000024575 | Pde6a | 3884 | 0 | 0 | 0 | 0 | 0 | 0 | 0.048725725 | 0.019666531 | 0.212082985 | 0.012139728 | 0 | 0 | 0.127013175 | 0.191324768 | 0.360672734 | 0.147878247 | 0.207222512 | 0.552357148 |
| ENSMUSG00000024576 | Csnk1a1 | 10038 | 56.66933361 | 47.89848598 | 51.86251581 | 49.53254402 | 52.09257011 | 47.40041321 | 51.58298049 | 51.46348226 | 60.3806302 | 53.77378471 | 50.8971315 | 55.77673953 | 48.19174943 | 46.73557748 | 51.19205071 | 61.0292317 | 54.75567084 | 51.47445103 |
| ENSMUSG00000024578 | Il17b | 689 | 0 | 0 | 0 | 0 | 0 | 0 | 0 | 0 | 0 | 0 | 0 | 0 | 0 | 0 | 0 | 0 | 0 | 0 |
| ENSMUSG00000024579 | Pcyox1l | 2639 | 1.497908818 | 1.451679741 | 0.76987051 | 1.070399677 | 0.401251353 | 0.319210485 | 0.60956085 | 1.316979393 | 0.952018743 | 0.75040907 | 0.488792679 | 0.655659545 | 0.915977244 | 1.407929894 | 0.593277425 | 1.479970426 | 0.60996759 | 1.208991357 |

| ENSMUSG00000024580 | Grpel2 | 4026 | 11.28614833 | 8.828827156 | 8.663019222 | 9.121262229 | 11.41728443 | 9.046512464 | 10.58835661 | 10.00819288 | 10.33243441 | 10.90345373 | 9.208486781 | 9.524431246 | 9.312532819 | 6.944694637 | 8.473643387 | 9.044800705 | 9.738649213 | 8.49867471 |
| --- | --- | --- | --- | --- | --- | --- | --- | --- | --- | --- | --- | --- | --- | --- | --- | --- | --- | --- | --- | --- |
| ENSMUSG00000024581 | Napg | 5288 | 7.837092781 | 8.328223555 | 8.031762415 | 8.538387847 | 8.474058422 | 8.199440598 | 8.884548066 | 8.861966292 | 10.14085543 | 7.971393478 | 8.628041858 | 8.52856569 | 7.780412067 | 9.169369749 | 9.513445488 | 8.602352797 | 9.088724719 | 7.65633558 |
| ENSMUSG00000024583 | Txnl1 | 2550 | 69.22509649 | 69.18630894 | 73.18630593 | 67.60901638 | 71.83925548 | 72.91053192 | 71.67407008 | 70.49868162 | 63.65335632 | 66.58418919 | 67.61561771 | 71.81615253 | 64.84728891 | 61.19691515 | 61.85080626 | 76.71635812 | 75.07445217 | 64.41458536 |
| ENSMUSG00000024587 | Nars | 2617 | 92.67655117 | 93.58670168 | 88.22577776 | 97.42438063 | 90.59910431 | 97.38238777 | 76.18480283 | 78.60308726 | 87.69249745 | 79.85170685 | 84.52352226 | 78.53009921 | 76.08086287 | 77.87415188 | 79.31732464 | 77.41520969 | 80.84110008 | 77.10103304 |
| ENSMUSG00000024588 | Fech | 3024 | 40.4108403 | 47.54578421 | 41.78295677 | 45.72677565 | 46.4925096 | 47.11114717 | 47.71946783 | 49.50866978 | 46.06243264 | 47.10392677 | 43.01958067 | 45.73783462 | 51.38741383 | 52.18816433 | 53.01430356 | 58.23363209 | 58.17384351 | 50.49777786 |
| ENSMUSG00000024589 | Nedd4l | 9178 | 40.95835441 | 41.95871183 | 36.96796055 | 41.12805103 | 44.03090337 | 38.19307445 | 34.94581068 | 40.51024596 | 34.29367547 | 35.83823338 | 37.53598013 | 35.57047401 | 30.16460228 | 29.90173375 | 34.72369087 | 33.46360449 | 38.20102027 | 34.30127672 |
| ENSMUSG00000024590 | Lmnb1 | 2951 | 2.448620781 | 1.715879336 | 2.065423526 | 3.118716013 | 1.989866041 | 2.350858252 | 2.549209065 | 3.559102223 | 2.177260745 | 2.157012857 | 1.79702487 | 1.967071809 | 1.738569772 | 1.11742806 | 1.689389449 | 0.739601702 | 1.610457749 | 2.740202876 |
| ENSMUSG00000024592 | C330018D20R | 3531 | 5.693839779 | 5.151186702 | 5.356570481 | 5.806420264 | 5.234397816 | 5.655551298 | 9.406259022 | 6.684481644 | 7.721742689 | 6.810212025 | 8.023378753 | 6.923608103 | 6.775978442 | 6.103102959 | 6.779412057 | 6.484787093 | 6.165204373 | 6.839138446 |
| ENSMUSG00000024593 | Megf10 | 7692 | 0 | 0 | 0 | 0 | 0 | 0 | 0 | 0 | 0.010708922 | 0 | 0 | 0 | 0 | 0 | 0 | 0 | 0 | 0 |
| ENSMUSG00000024594 | Prrc1 | 4792 | 16.15232169 | 20.93873902 | 21.52177937 | 20.81242196 | 18.67221329 | 23.1838958 | 17.53491606 | 20.47501707 | 16.48492012 | 20.2692923 | 19.61046397 | 19.16042674 | 15.606675 | 15.77858395 | 19.06173819 | 18.3862036 | 17.31561619 | 16.8976221 |
| ENSMUSG00000024597 | Slc12a2 | 6635 | 1.467021147 | 1.325486673 | 1.377934776 | 1.469489599 | 1.603188415 | 1.762538828 | 0.399323289 | 1.646273864 | 1.837408993 | 0.412168921 | 0.504031212 | 0.883292324 | 0.810426373 | 0.685986521 | 0.807264848 | 0.750230439 | 0.537203559 | 0.489154076 |
| ENSMUSG00000024598 | Fbn2 | 10415 | 0 | 0 | 0 | 0 | 0 | 0 | 0 | 0 | 0 | 0.07243507 | 0 | 0.005359161 | 0 | 0 | 0 | 0.01102946 | 0 | 0.036972035 |
| ENSMUSG00000024600 | Slc27a6 | 2475 | 0 | 0 | 0 | 0 | 0 | 0 | 0 | 0 | 0 | 0 | 0 | 0 | 0 | 0 | 0 | 0 | 0 | 0 |
| ENSMUSG00000024601 | Isoc1 | 2558 | 28.1650795 | 28.50739853 | 26.04001224 | 23.88481644 | 27.58459738 | 25.72557865 | 26.85614144 | 28.95037852 | 29.51324605 | 24.64444498 | 28.29517674 | 28.14785173 | 30.25870923 | 24.92874589 | 25.56171587 | 21.57027571 | 22.44440687 | 22.66598846 |
| ENSMUSG00000024603 | Dctn4 | 5248 | 15.86654723 | 14.09196364 | 14.99690051 | 16.11305227 | 14.63768105 | 14.60710344 | 12.98213751 | 13.82727983 | 15.35076693 | 14.63576517 | 13.30925863 | 15.48544297 | 11.12976182 | 11.86766791 | 14.25724571 | 13.43235675 | 11.80899811 | 12.62022951 |
| ENSMUSG00000024604 | Rbm22 | 4656 | 9.101731207 | 9.92375092 | 12.19727763 | 11.59575548 | 11.43339939 | 10.25962955 | 11.26927855 | 12.25503634 | 9.456268331 | 12.28389241 | 11.23573444 | 10.32158193 | 10.86022663 | 9.526224969 | 9.866781533 | 10.97894349 | 11.58185132 | 13.17335782 |
| ENSMUSG00000024608 | Rps14 | 1397 | 174.9497224 | 140.8346122 | 143.7008955 | 125.6923872 | 155.0075337 | 134.1861067 | 184.7124917 | 154.382603 | 151.5085931 | 148.7424118 | 154.4735057 | 145.5522253 | 220.1397082 | 215.3649235 | 175.8365614 | 162.043 | 139.6152648 | 173.8083014 |
| ENSMUSG00000024610 | Cd74 | 1555 | 58.03118245 | 65.42609578 | 72.01589889 | 41.72278272 | 63.54652044 | 76.83063708 | 124.4125686 | 141.0539981 | 151.794162 | 41.54113406 | 209.9640297 | 62.38432256 | 118.7772979 | 105.2832206 | 116.4506998 | 54.37020009 | 12.61933377 | 21.47300822 |
| ENSMUSG00000024613 | Tcof1 | 5668 | 4.844450197 | 4.596097967 | 5.026818874 | 6.390442881 | 4.551642746 | 5.93618668 | 5.317250613 | 4.656130921 | 4.454363825 | 6.588453948 | 4.737037167 | 5.731248346 | 4.134202665 | 3.220276349 | 4.296069078 | 4.269524326 | 4.131504483 | 3.872377132 |
| ENSMUSG00000024614 | Tmx3 | 4595 | 17.51083748 | 20.6039707 | 20.18107893 | 21.3576691 | 19.77651184 | 22.46318695 | 13.89005525 | 19.49932014 | 17.79221628 | 21.33325616 | 16.65625321 | 18.31774084 | 13.22675127 | 14.81763484 | 15.01906553 | 15.66623328 | 18.97547888 | 15.39536697 |
| ENSMUSG00000024617 | Camk2a | 7653 | 0 | 0.008705839 | 0 | 0 | 0 | 0 | 0 | 0.004990514 | 0.107634956 | 0.018483224 | 0 | 0 | 0.038676533 | 0 | 0 | 0 | 0.025040048 | 0 |
| ENSMUSG00000024619 | Cdx1 | 1747 | 0 | 0 | 0 | 0 | 0 | 0.056728945 | 0 | 0 | 0 | 0 | 0 | 0 | 0 | 0 | 0 | 0 | 0 | 0 |
| ENSMUSG00000024620 | Pdgfrb | 5826 | 0.131323922 | 0 | 0 | 0.023460888 | 0.008261573 | 0.017010894 | 0 | 0 | 0 | 0.064745216 | 0 | 0 | 0 | 0.159437307 | 0.091936187 | 0.203743362 | 0.059206432 | 0.009442003 |
| ENSMUSG00000024621 | Csf1r | 5206 | 0 | 0 | 0 | 0.008751641 | 0.036981885 | 0 | 0.027264317 | 0.396156306 | 0.015822711 | 0.045284963 | 0 | 0 | 0.037903893 | 0 | 0.039571222 | 0.014710183 | 0 | 0.042265929 |
| ENSMUSG00000024622 | Hmgxb3 | 5177 | 4.491081956 | 6.602091843 | 5.204569521 | 6.072459067 | 5.764302236 | 7.150066058 | 4.459839157 | 6.034650409 | 6.674809103 | 5.728760353 | 6.78280751 | 5.315263986 | 4.926521389 | 5.284057847 | 5.069613889 | 5.732126857 | 6.22607991 | 9.010570311 |
| ENSMUSG00000024639 | Gnaq | 6342 | 9.651127953 | 9.292101841 | 9.786067399 | 10.7688435 | 10.07871249 | 8.868235984 | 8.735910915 | 10.03890489 | 10.4427495 | 8.720872741 | 9.951233073 | 7.780045053 | 7.327446559 | 8.480330053 | 8.510570517 | 8.796817248 | 8.375945681 | 7.615578004 |
| ENSMUSG00000024640 | Psat1 | 5266 | 1.662754702 | 1.771289476 | 1.598365796 | 1.271833178 | 1.700064148 | 1.985496921 | 7.088815738 | 5.693322425 | 4.606695363 | 2.829400347 | 2.975728519 | 5.161835868 | 10.42659019 | 13.56456635 | 6.454858262 | 3.381149343 | 4.89813859 | 6.225869276 |
| ENSMUSG00000024642 | Tle4 | 6191 | 6.858774164 | 7.452488854 | 8.047930264 | 8.441046334 | 9.267202877 | 9.180582364 | 5.464152065 | 7.569387458 | 7.610624137 | 5.887173901 | 7.315564632 | 7.65425578 | 5.123634762 | 5.993995449 | 9.643200531 | 9.314433007 | 9.230254996 | 10.78679625 |
| ENSMUSG00000024644 | Cndp2 | 2102 | 49.13776296 | 62.07735807 | 52.63095334 | 55.37986122 | 57.77204729 | 60.53825872 | 51.11660031 | 55.90771751 | 48.76938046 | 39.27729848 | 42.61562662 | 44.42416913 | 45.31871183 | 44.03571178 | 47.06229101 | 47.08906762 | 44.87203458 | 36.74252419 |
| ENSMUSG00000024645 | 1700034H14R | 1333 | 17.69720382 | 17.76854298 | 17.41882563 | 18.59355448 | 15.92361466 | 15.38997127 | 20.23122805 | 22.49139977 | 26.72643371 | 21.3999816 | 24.94476452 | 22.86222939 | 18.76315233 | 19.89465409 | 26.8907379 | 23.72696303 | 23.60531865 | 21.29384776 |
| ENSMUSG00000024646 | Cyb5 | 2366 | 168.5296096 | 149.4574692 | 160.3318787 | 156.9410445 | 162.3587839 | 163.884252 | 211.8272223 | 170.7842846 | 194.2346335 | 200.0018841 | 181.2863809 | 181.9552855 | 210.8386929 | 196.3568073 | 180.6355618 | 174.7838377 | 153.8235483 | 180.0932125 |
| ENSMUSG00000024647 | Cbln2 | 2888 | 0 | 0 | 0 | 0 | 0 | 0 | 0 | 0 | 0 | 0 | 0 | 0 | 0 | 0 | 0 | 0 | 0 | 0 |
| ENSMUSG00000024650 | Slc22a6 | 4016 | 0 | 0 | 0 | 0 | 0 | 0 | 0 | 0 | 0 | 0 | 0 | 0 | 0 | 0 | 0 | 0 | 0 | 0 |
| ENSMUSG00000024653 | Scgb1a1 | 473 | 0 | 0 | 0 | 0 | 0 | 0 | 0 | 0 | 0 | 0 | 0 | 0 | 0 | 0 | 0 | 0 | 0 | 0 |
| ENSMUSG00000024654 | Asrgl1 | 4289 | 8.582299473 | 9.25832826 | 8.684452873 | 8.423853616 | 8.854298851 | 8.722852379 | 10.18176793 | 8.032069706 | 9.804484167 | 9.465319539 | 11.07211675 | 8.276698859 | 15.9302181 | 11.70577255 | 11.42192818 | 14.80201144 | 12.80521881 | 11.95348274 |
| ENSMUSG00000024658 | Gm9750 | 2011 | 0 | 0 | 0.024054466 | 0 | 0.143605938 | 0 | 0 | 0 | 0 | 0 | 0 | 0 | 0.024531038 | 0 | 0 | 0.057121742 | 0.095291638 | 0.109416423 |
| ENSMUSG00000024659 | Anxa1 | 2379 | 190.1566156 | 174.3500824 | 181.1923199 | 166.8656491 | 175.1281762 | 199.1274203 | 145.1001704 | 149.2216815 | 157.9076126 | 122.4055247 | 129.9502969 | 165.1242679 | 72.5981683 | 77.48490242 | 89.8502194 | 70.88349516 | 92.02177718 | 96.93071685 |
| ENSMUSG00000024660 | Incenp | 3680 | 1.605494421 | 1.339757702 | 2.063762029 | 2.216148624 | 1.124822129 | 2.329517095 | 1.09282003 | 0.95481006 | 0.548407411 | 1.089078744 | 2.479624204 | 1.152714771 | 1.286919581 | 1.678549897 | 1.768979845 | 2.882200611 | 2.541200121 | 2.481389053 |
| ENSMUSG00000024661 | Fth1 | 862 | 2642.668096 | 2511.14297 | 2596.120953 | 2376.521156 | 2571.093454 | 2499.308814 | 4191.178843 | 3418.928923 | 3708.267604 | 3740.221331 | 3342.141005 | 3044.608416 | 5066.078864 | 5092.847416 | 4653.720518 | 4567.776287 | 4531.840067 | 4961.604453 |
| ENSMUSG00000024663 | Rab3il1 | 4526 | 0.131478671 | 0.176648135 | 0.374077234 | 0.27179589 | 0.478554257 | 0.361299206 | 2.101159625 | 1.603304564 | 2.329595235 | 0.958432324 | 1.034455575 | 1.455202869 | 1.068167895 | 0.913284087 | 0.892123835 | 0.245344148 | 0.533485794 | 1.39771261 |
| ENSMUSG00000024664 | Fads3 | 3638 | 1.834335524 | 2.426585203 | 2.260445344 | 1.928642339 | 1.865475876 | 2.179339575 | 2.991180911 | 3.023477706 | 4.14355821 | 1.477509668 | 1.654657715 | 1.549582716 | 6.102078827 | 10.26416885 | 4.020492672 | 2.54709373 | 3.38173208 | 6.698470115 |

| ENSMUSG00000024665 | Fads2 | 1496 | 20.9116374 | 30.03950154 | 27.71130716 | 30.12023586 | 30.53288473 | 26.03505922 | 17.7422546 | 18.50901855 | 20.0976982 | 17.14571028 | 13.95565298 | 15.48362298 | 25.22654861 | 38.74480017 | 29.22115695 | 14.87088429 | 18.70200317 | 23.16559976 |
| --- | --- | --- | --- | --- | --- | --- | --- | --- | --- | --- | --- | --- | --- | --- | --- | --- | --- | --- | --- | --- |
| ENSMUSG00000024666 | Tmem138 | 1585 | 12.3090699 | 13.59838647 | 10.040941 | 7.761187363 | 13.2400749 | 10.66087202 | 10.08939147 | 9.807134245 | 13.12251764 | 12.25620804 | 12.02662993 | 11.76178631 | 11.88945893 | 12.65857776 | 14.63500061 | 11.9582654 | 11.96941134 | 15.93008201 |
| ENSMUSG00000024667 | Tmem216 | 1969 | 7.188534087 | 8.933066606 | 8.598646838 | 6.085604195 | 7.015673993 | 6.870439953 | 8.60230526 | 7.176835394 | 8.408826479 | 8.59680166 | 10.06937926 | 8.532511142 | 12.00100984 | 13.89312721 | 9.960355817 | 11.70693025 | 10.78352894 | 9.247340947 |
| ENSMUSG00000024668 | Sdhaf2 | 3092 | 21.21134755 | 17.88467154 | 17.14663301 | 18.34524586 | 18.97568401 | 16.49086768 | 21.57531607 | 19.46676138 | 20.68650032 | 19.73253881 | 17.8306281 | 18.10579223 | 20.83683181 | 23.37225096 | 24.14528437 | 22.71182858 | 20.87370116 | 20.17475001 |
| ENSMUSG00000024669 | Cd5 | 2058 | 0 | 0 | 0 | 0 | 0 | 0 | 0.344844599 | 0.222696224 | 0.14009019 | 0 | 0 | 0.08136394 | 0.287649663 | 0.090270335 | 0 | 0 | 0 | 0.294023408 |
| ENSMUSG00000024670 | Cd6 | 4077 | 0 | 0.016341866 | 0 | 0 | 0 | 0.036462644 | 0 | 0 | 0 | 0 | 0 | 0 | 0 | 0 | 0 | 0 | 0 | 0 |
| ENSMUSG00000024671 | Ms4a14 | 3498 | 0 | 0 | 0 | 0 | 0 | 0 | 0 | 0 | 0 | 0 | 0 | 0 | 0 | 0.053109305 | 0 | 0 | 0 | 0 |
| ENSMUSG00000024672 | Ms4a7 | 2619 | 0 | 0.1144773 | 0 | 0 | 0.055133933 | 0 | 0 | 0.029165638 | 0 | 0 | 0 | 0 | 0 | 0 | 0 | 0 | 0 | 0 |
| ENSMUSG00000024673 | Ms4a1 | 3797 | 0.089555283 | 0.026320433 | 0.012739934 | 0.059996108 | 0 | 0.078302976 | 2.865924718 | 0.764451562 | 1.778927728 | 0.894087605 | 7.247399548 | 0.455698062 | 3.819748138 | 4.158806877 | 1.60596004 | 1.01852814 | 0 | 0.883738612 |
| ENSMUSG00000024675 | Ms4a4c | 1743 | 0 | 0 | 0.027753029 | 0 | 0 | 0 | 0 | 0.17529502 | 0.094518682 | 0 | 0.027409615 | 0 | 0.339634542 | 0 | 0 | 0.06590466 | 0 | 0 |
| ENSMUSG00000024677 | Ms4a6b | 2829 | 0.525868208 | 0.741856605 | 0.410379897 | 1.159559983 | 0.476385247 | 0.963379409 | 0.133793366 | 0.229505423 | 0.684258127 | 0.500007458 | 0.472852187 | 0.197298206 | 1.272969233 | 0.591017019 | 0.131076 | 0.108280261 | 0.365786503 | 0.311115485 |
| ENSMUSG00000024678 | Ms4a4d | 1506 | 0 | 0 | 0 | 0 | 0 | 0 | 0 | 0 | 0 | 0 | 0 | 0 | 0 | 0 | 0 | 0 | 0 | 0 |
| ENSMUSG00000024679 | Ms4a6d | 1927 | 0 | 0 | 0 | 0.047287021 | 0.149865875 | 0 | 0 | 0.317113876 | 0.192360478 | 0 | 0 | 0 | 0 | 0.024101758 | 0 | 0 | 0 | 0 |
| ENSMUSG00000024680 | Ms4a2 | 3363 | 0 | 0 | 0 | 0 | 0 | 0 | 0 | 0 | 0 | 0 | 0 | 0 | 0 | 0 | 0 | 0 | 0 | 0 |
| ENSMUSG00000024681 | Ms4a3 | 1876 | 0 | 0 | 0 | 0 | 0 | 0 | 0 | 0 | 0 | 0 | 0 | 0 | 0 | 0 | 0 | 0 | 0 | 0 |
| ENSMUSG00000024682 | Gif | 1528 | 0 | 0 | 0 | 0 | 0 | 0 | 0 | 0 | 0 | 0 | 0 | 0 | 0 | 0 | 0 | 0 | 0 | 0 |
| ENSMUSG00000024683 | Mrpl16 | 1251 | 40.29667368 | 35.28344112 | 32.09434868 | 34.16167833 | 38.74408244 | 35.92672211 | 38.72756453 | 35.75004254 | 38.22345707 | 36.52200756 | 36.12718683 | 36.36272183 | 38.76360886 | 37.57107629 | 36.85414951 | 36.33169543 | 38.1118412 | 35.61740723 |
| ENSMUSG00000024687 | Osbp | 4767 | 12.77741079 | 16.40836493 | 15.69831161 | 15.66489441 | 15.61990474 | 17.14127495 | 14.48063741 | 15.65511943 | 14.61011079 | 15.43995129 | 16.7969017 | 15.13942767 | 10.74187752 | 13.32819626 | 17.22565574 | 13.22941691 | 15.68588321 | 15.70534807 |
| ENSMUSG00000024691 | Fam111a | 4118 | 0 | 0 | 0.01174685 | 0 | 0 | 0 | 0.402123303 | 0.027823508 | 0.170026898 | 0 | 0.162420937 | 0.054216282 | 0.095836653 | 0.293236103 | 0 | 0.353337319 | 0.111684207 | 0.040074629 |
| ENSMUSG00000024694 | Keg1 | 4928 | 0 | 0 | 0 | 0 | 0 | 0 | 0.249620465 | 0.162751715 | 0.041788267 | 0.14351878 | 0 | 0.113262302 | 0 | 0 | 0 | 0 | 0.015554504 | 0 |
| ENSMUSG00000024695 | Zfp91 | 10034 | 6.294866611 | 5.929522531 | 5.794796042 | 5.83021536 | 5.051117762 | 6.33607307 | 5.879899404 | 6.440257611 | 7.146275059 | 6.226298766 | 5.927827716 | 5.612717117 | 5.717861251 | 5.512747467 | 5.588530752 | 6.815529681 | 5.87843043 | 6.310093865 |
| ENSMUSG00000024696 | Lpxn | 1666 | 0 | 0.179961613 | 0.029035733 | 0.656341575 | 0.808939894 | 0.029743538 | 0.993963843 | 2.13198885 | 1.063037322 | 0.820750537 | 0.946322707 | 1.306609148 | 0.710663874 | 1.031471329 | 1.13761799 | 0.137901347 | 1.058229086 | 1.849045604 |
| ENSMUSG00000024697 | Gna14 | 3376 | 1.674522634 | 2.003115381 | 1.318236017 | 2.172786771 | 0.926710616 | 1.438438356 | 2.662739632 | 2.681160951 | 1.988566964 | 3.156415562 | 3.000086255 | 3.786074269 | 1.271290522 | 0.316414102 | 1.586552804 | 0.567100228 | 1.419072012 | 0.619178334 |
| ENSMUSG00000024712 | Rfk | 2597 | 59.44505173 | 50.62995513 | 54.87424737 | 54.45571106 | 51.96839198 | 49.66721814 | 63.67262721 | 46.73679432 | 47.5143632 | 44.33654885 | 45.78816775 | 42.51189087 | 51.72537957 | 41.43663861 | 37.44153712 | 39.94195435 | 46.2070218 | 48.25211595 |
| ENSMUSG00000024713 | Pcsk5 | 8746 | 0.077759298 | 0.217108961 | 0.243361003 | 0.109396516 | 0.049529764 | 0.237961904 | 0.178517997 | 0.034934738 | 0 | 0.080866745 | 0.032774954 | 0.057436653 | 0.203058429 | 0 | 0.009421806 | 0.008756142 | 0.11831809 | 0.150951128 |
| ENSMUSG00000024725 | Ostf1 | 5111 | 24.59162698 | 21.96526189 | 20.9167486 | 20.13742891 | 19.49385286 | 20.14685587 | 29.36329829 | 25.69823359 | 29.68716965 | 27.39002895 | 25.64947878 | 27.21436728 | 27.05485504 | 23.32654516 | 27.58593705 | 27.00795142 | 25.57086137 | 22.28993544 |
| ENSMUSG00000024726 | 2410127L17R | 2082 | 6.145080684 | 5.936159345 | 6.342926891 | 5.864726168 | 6.334364594 | 5.59312795 | 6.112925375 | 5.136346145 | 6.745726165 | 6.680815303 | 5.621933154 | 5.549395848 | 6.279038462 | 5.443015045 | 5.362930666 | 5.241509659 | 6.1484069 | 5.099307203 |
| ENSMUSG00000024727 | Trpm6 | 6469 | 0.131411891 | 0.056645824 | 0.022433234 | 0.077472791 | 0.05952317 | 0.03830015 | 0 | 0 | 0 | 0 | 0 | 0.138050796 | 0 | 0 | 0 | 0.065110014 | 0.011849218 | 0 |
| ENSMUSG00000024728 | 1700025F22R | 643 | 0 | 0 | 0 | 0 | 0.074855247 | 0 | 0 | 0 | 0 | 0 | 0 | 0 | 0 | 0 | 0 | 0 | 0 | 0 |
| ENSMUSG00000024729 | 1700017D01R | 1187 | 0 | 0 | 0 | 0 | 0 | 0 | 0 | 0 | 0 | 0 | 0 | 0 | 0 | 0 | 0 | 0 | 0 | 0 |
| ENSMUSG00000024730 | Ms4a8a | 1074 | 0 | 0 | 0 | 0 | 0 | 0 | 0 | 0 | 0 | 0 | 0 | 0 | 0 | 0 | 0 | 0 | 0 | 0 |
| ENSMUSG00000024731 | Ms4a10 | 998 | 0 | 0 | 0 | 0 | 0 | 0 | 0 | 0 | 0 | 0 | 0 | 0 | 0 | 0 | 0 | 0 | 0 | 0 |
| ENSMUSG00000024732 | Ccdc86 | 3362 | 7.990265095 | 7.827836536 | 8.935146446 | 7.18243709 | 8.203329049 | 8.40126655 | 6.923806672 | 6.179853334 | 6.835834561 | 8.526956449 | 8.384064694 | 6.341934297 | 8.261115237 | 8.122880245 | 6.152046715 | 6.02490521 | 7.056507371 | 7.67378675 |
| ENSMUSG00000024734 | Zp1 | 2133 | 0 | 0 | 0 | 0 | 0 | 0 | 0 | 0 | 0 | 0.022105346 | 0 | 0 | 0 | 0.457255431 | 0.057948742 | 0 | 0 | 0.180526839 |
| ENSMUSG00000024735 | Prpf19 | 2160 | 74.56116295 | 88.10952068 | 72.85143252 | 75.61867775 | 70.10325549 | 75.15497926 | 40.52243334 | 46.06074457 | 52.01704398 | 47.4781387 | 46.66905666 | 42.4302398 | 45.17524647 | 38.05834939 | 44.02462546 | 39.19469119 | 37.42137045 | 45.5607833 |
| ENSMUSG00000024736 | Tmem132a | 5376 | 3.043990471 | 5.403430759 | 4.13910416 | 4.720517428 | 4.754101835 | 4.894438529 | 2.30578904 | 2.117063973 | 1.447963445 | 2.675030598 | 1.244139544 | 1.785768967 | 0.954337685 | 1.701913173 | 1.563451898 | 1.175213955 | 1.055113828 | 1.340437682 |
| ENSMUSG00000024737 | Slc15a3 | 3387 | 0 | 0 | 0 | 0 | 0 | 0.014630273 | 0 | 0.056380872 | 0 | 0 | 0 | 0 | 0 | 0 | 0 | 0 | 0.022631412 | 0 |
| ENSMUSG00000024738 | Pga5 | 1407 | 0 | 0.023676542 | 0 | 0.194290168 | 0 | 0 | 0.168133187 | 0 | 0 | 0 | 0 | 0 | 0.385679524 | 0.363102318 | 0 | 0.353786707 | 0 | 0 |
| ENSMUSG00000024740 | Ddb1 | 4329 | 64.45980148 | 80.08484411 | 75.42650263 | 83.03918684 | 69.34599397 | 82.2673819 | 62.29666664 | 69.30919688 | 53.54532472 | 64.28354138 | 69.78079521 | 66.10462049 | 57.44564449 | 68.85612217 | 71.21045072 | 60.69534488 | 73.04041336 | 72.53222018 |
| ENSMUSG00000024742 | Fen1 | 4250 | 0.180021922 | 0.156766561 | 0.07967405 | 0.096482212 | 0.124576744 | 0.058297334 | 0.122456345 | 0.053918686 | 0.03876378 | 0.055471416 | 0.067447 | 0.157597165 | 0.185720159 | 0.207632391 | 0.135722772 | 0.180191094 | 0.072143618 | 0.129433192 |
| ENSMUSG00000024743 | Syt7 | 6984 | 2.726563411 | 2.590048906 | 2.472701938 | 2.681212661 | 2.370759127 | 2.09307795 | 2.21524141 | 2.690530066 | 1.981481858 | 1.95786139 | 2.031667049 | 2.149830074 | 1.299695415 | 1.077311305 | 0.902611405 | 1.474824369 | 0.938401599 | 1.283860881 |

| ENSMUSG00000024747 | Aldh1a7 |  | 2053 | 5.652173919 | 4.072838017 | 4.806721964 | 2.441166526 | 3.985595238 | 3.35500729 | 7.397647314 | 8.669098646 | 8.706745173 | 10.33502994 | 8.191322642 | 9.406828668 | 20.71315767 | 19.00293882 | 23.32012077 | 10.29538007 | 14.61738455 | 23.87389873 |
| --- | --- | --- | --- | --- | --- | --- | --- | --- | --- | --- | --- | --- | --- | --- | --- | --- | --- | --- | --- | --- | --- |
| ENSMUSG00000024749 | Tmc1 |  | 4483 | 0.02844424 | 0.007430938 | 0.010790437 | 0.020326141 | 0 | 0 | 0 | 0.119271388 | 0.018374533 | 0 | 0.010656917 | 0.024901032 | 0 | 0 | 0 | 0 | 0 | 0 |
| ENSMUSG00000024750 | Zfand5 |  | 3418 | 83.18230616 | 87.69731486 | 80.91031979 | 86.65662646 | 81.63275644 | 84.63688082 | 77.51638443 | 92.27409569 | 83.71056905 | 83.16898477 | 87.19139584 | 78.31829074 | 81.18549874 | 83.85209575 | 76.02639351 | 85.74497047 | 98.5629461 | 91.04345136 |
| ENSMUSG00000024754 | Tmem2 |  | 6642 | 1.535869052 | 2.066382478 | 1.602254845 | 2.496871445 | 2.202966696 | 1.812904887 | 1.146844521 | 0.902771331 | 1.804467944 | 0.986743112 | 0.949457169 | 1.554636793 | 0.438208841 | 1.300602267 | 1.222027478 | 0.478488469 | 1.055963916 | 0.861328982 |
| ENSMUSG00000024757 | Slc22a19 |  | 5421 | 0 | 0 | 0 | 0 | 0 | 0 | 0 | 0 | 0 | 0 | 0 | 0 | 0 | 0 | 0 | 0 | 0 | 0 |
| ENSMUSG00000024758 | Rtn3 |  | 5894 | 139.8329425 | 141.0400343 | 137.6191647 | 144.2972539 | 145.4573845 | 149.6838936 | 108.2476205 | 114.8234426 | 113.7555633 | 121.0686704 | 118.3188105 | 112.1521703 | 108.6825493 | 93.17973081 | 110.4486907 | 112.5070816 | 114.6342532 | 107.2556251 |
| ENSMUSG00000024759 | Atl3 |  | 6684 | 2.823503614 | 2.277676939 | 2.156689414 | 2.338036835 | 2.059504813 | 2.394603972 | 2.208491296 | 1.977045367 | 2.076579266 | 2.045736676 | 2.387318392 | 2.229620274 | 2.236321205 | 1.903901847 | 2.243771147 | 2.096702919 | 2.700730976 | 2.148021672 |
| ENSMUSG00000024761 | Gm16437 |  | 506 | 2.100058104 | 2.699266132 | 1.720797523 | 1.980914971 | 1.997569955 | 1.371024249 | 2.431086269 | 1.886976403 | 2.523284563 | 1.956847368 | 1.699504452 | 1.764922137 | 2.339853784 | 1.835734679 | 3.094187992 | 2.043174703 | 3.559952479 | 3.15269584 |
| ENSMUSG00000024764 | Naa40 |  | 3267 | 10.29127464 | 13.97979124 | 12.40802523 | 11.92368929 | 12.27238825 | 12.37680765 | 13.39584573 | 13.88814633 | 12.2538394 | 13.66749801 | 17.46045314 | 11.54924638 | 10.37374569 | 18.225075 | 15.9534644 | 16.34998388 | 14.34743224 | 16.55156165 |
| ENSMUSG00000024766 | Lipo1 |  | 3501 | 6.883871696 | 6.099275977 | 7.944810041 | 6.623985023 | 7.533931497 | 7.317555915 | 7.540838287 | 9.196285412 | 7.11734991 | 7.838248871 | 6.263554968 | 7.684418557 | 8.820845534 | 8.158558624 | 7.884902156 | 7.120018686 | 8.96579182 | 8.62610671 |
| ENSMUSG00000024767 | Otub1 |  | 3567 | 41.17055878 | 39.83164952 | 39.12465239 | 38.89357443 | 40.29210095 | 39.75887959 | 39.67261639 | 36.74693722 | 33.64662375 | 34.11717553 | 36.61809265 | 34.26865743 | 32.33474141 | 36.99121174 | 34.2248977 | 35.95044693 | 35.55416777 | 31.93828428 |
| ENSMUSG00000024768 | Lipf |  | 1401 | 0 | 0 | 0 | 0 | 0 | 0 | 0 | 0 | 0 | 0 | 0 | 0 | 0 | 0 | 0 | 0 | 0 | 0 |
| ENSMUSG00000024769 | Cdc42bpg |  | 6062 | 2.895849175 | 2.269585172 | 2.202424015 | 1.826350012 | 1.667387656 | 1.912791102 | 1.404863446 | 2.205104065 | 2.839980797 | 2.286754661 | 2.411602985 | 3.213405847 | 3.979430476 | 1.723840261 | 2.698287313 | 1.901265725 | 2.427795779 | 1.397459986 |
| ENSMUSG00000024770 | Lipn |  | 2017 | 0 | 0 | 0 | 0 | 0 | 0 | 0 | 0 | 0 | 0 | 0 | 0 | 0 | 0 | 0 | 0 | 0 | 0 |
| ENSMUSG00000024771 | Lipk |  | 2088 | 0 | 0 | 0 | 0.021820424 | 0 | 0 | 0 | 0 | 0 | 0 | 0 | 0 | 0.33076956 | 0.155703358 | 0 | 0 | 0.055066519 | 0 |
| ENSMUSG00000024772 | Ehd1 |  | 3714 | 9.933896821 | 8.314769236 | 7.332874957 | 6.747058262 | 6.946341165 | 8.458921133 | 6.687979655 | 7.03381886 | 7.296910978 | 7.261766899 | 3.601773929 | 8.761586238 | 6.999978569 | 7.115424266 | 8.453307913 | 9.72214399 | 6.883047936 | 6.087437497 |
| ENSMUSG00000024773 | Atg2a |  | 7654 | 3.848456622 | 5.41432459 | 5.725949634 | 6.369260183 | 5.590446844 | 5.256966248 | 5.291305613 | 5.289253534 | 2.959574558 | 6.394359809 | 4.244443659 | 4.586889439 | 4.460110629 | 4.793680303 | 4.327939762 | 7.21888509 | 6.058899824 | 5.016508557 |
| ENSMUSG00000024774 | Ankrd22 |  | 2572 | 0 | 0.220186315 | 0 | 0.106285484 | 0 | 0.057798678 | 0 | 0 | 0 | 0 | 0 | 0 | 0 | 0.216690921 | 0 | 0 | 0.01490136 | 0 |
| ENSMUSG00000024776 | Stambpl1 |  | 3088 | 0.165175555 | 0.183393524 | 0.062660013 | 0.221313363 | 0 | 0.128374957 | 0.474965365 | 0.333936161 | 0.320102454 | 0.183228121 | 0.402250298 | 0 | 0.079876809 | 0.210562572 | 0.066712364 | 0.26039597 | 0.322695505 | 0 |
| ENSMUSG00000024777 | Ppp2r5b |  | 2839 | 17.65185014 | 21.00390301 | 17.39675044 | 24.68224241 | 21.66699487 | 21.83531867 | 19.66500921 | 17.44823738 | 15.94363537 | 21.52423791 | 17.08051527 | 19.89624887 | 17.06373468 | 15.85217354 | 13.33717149 | 17.33125414 | 17.88740521 | 13.67961583 |
| ENSMUSG00000024778 | Fas |  | 1791 | 0.569583598 | 0.409203614 | 0.675230742 | 0.559655486 | 0.080622988 | 0.498017423 | 2.219020117 | 5.11790987 | 0.896858802 | 0.63183522 | 0.61352543 | 2.088023112 | 1.184406724 | 0.959481426 | 1.058219749 | 0.791039907 | 0.9415729 | 0.737140459 |
| ENSMUSG00000024780 | Cdc37l1 |  | 5404 | 24.66621614 | 26.34702256 | 26.28139991 | 22.77209118 | 26.80032537 | 23.59348325 | 25.72254824 | 27.75380537 | 27.29254496 | 23.74112945 | 27.06127829 | 24.35479874 | 26.99379707 | 30.21787773 | 30.01675158 | 23.02821504 | 29.04968766 | 25.26510046 |
| ENSMUSG00000024781 | Lipa |  | 2959 | 4.582342397 | 4.266842484 | 5.623688558 | 5.050362468 | 6.229985389 | 5.1746518 | 4.972708059 | 3.394593387 | 6.124388961 | 3.61717122 | 2.131224914 | 3.69715102 | 2.717506764 | 3.704225963 | 2.756981435 | 3.260977717 | 4.727643923 | 2.602661352 |
| ENSMUSG00000024782 | Ak3 |  | 3115 | 103.0085309 | 103.788648 | 102.9898084 | 104.6807044 | 104.7622608 | 113.7247166 | 92.45337934 | 102.4512727 | 100.1301714 | 96.7989558 | 93.06531238 | 98.40758232 | 104.3334417 | 94.49843527 | 104.6770829 | 104.3741725 | 121.4998979 | 103.9433715 |
| ENSMUSG00000024784 | Gpha2 |  | 803 | 0 | 0 | 0 | 0 | 0 | 0 | 0 | 0.09512429 | 0 | 0 | 0 | 0 | 0 | 0 | 0.153928602 | 0 | 0 | 0 |
| ENSMUSG00000024785 | Rcl1 |  | 1794 | 15.49519795 | 11.64280082 | 11.99900837 | 12.77436193 | 15.85616883 | 13.61733426 | 12.7907744 | 18.20206469 | 12.37432107 | 12.24761857 | 14.03422693 | 14.1250339 | 15.61902398 | 14.60115122 | 15.75488704 | 17.62990062 | 14.97588302 | 17.07919311 |
| ENSMUSG00000024786 | 1700123I01Ri | k | 1745 | 0.024358267 | 0 | 0 | 0 | 0 | 0 | 0 | 0 | 0.023602588 | 0 | 0 | 0.127944212 | 0 | 0.026615523 | 0 | 0 | 0 | 0 |
| ENSMUSG00000024787 | Snx15 |  | 2158 | 12.62549483 | 12.62740847 | 13.87544731 | 13.11094001 | 12.31178029 | 12.42262691 | 13.48345576 | 13.46821975 | 12.93997112 | 12.47592751 | 13.34953659 | 11.12174926 | 12.11580915 | 8.544162509 | 9.813530921 | 12.11885304 | 12.07686835 | 9.941404829 |
| ENSMUSG00000024789 | Jak2 |  | 5005 | 21.26532683 | 28.12127434 | 25.16776684 | 29.33930987 | 25.67677047 | 24.68231065 | 18.29171503 | 24.80789215 | 23.91368927 | 25.59609605 | 23.9208883 | 26.27406614 | 22.03919421 | 26.6601125 | 28.6723315 | 24.56566241 | 28.73132187 | 25.90538752 |
| ENSMUSG00000024790 | Sac3d1 |  | 1372 | 11.02903984 | 10.53775225 | 8.955449497 | 11.09139126 | 9.366781064 | 7.981890759 | 14.79383328 | 10.16051522 | 12.60811707 | 12.74993506 | 11.59552564 | 15.62187641 | 12.47680415 | 19.29528412 | 15.16529572 | 9.740103486 | 10.67102436 | 18.1626278 |
| ENSMUSG00000024791 | Cdca5 |  | 2122 | 0.36055286 | 0.863435053 | 0.47872014 | 0.751478111 | 0.54437614 | 1.354410249 | 1.426961099 | 0.647938966 | 1.222785341 | 0.488838582 | 2.18386945 | 0.946919818 | 2.255040516 | 1.160007838 | 0.465993092 | 0.39698085 | 0.740517518 | 1.322085034 |
| ENSMUSG00000024792 | Zfpl1 |  | 2196 | 39.27276965 | 31.75040419 | 34.25356999 | 32.80146225 | 31.67150715 | 34.52444553 | 30.16291006 | 33.75749229 | 33.79694964 | 31.43380217 | 33.43811985 | 34.23665642 | 38.54898454 | 32.25283846 | 35.44159339 | 31.9785856 | 30.08858646 | 35.79599886 |
| ENSMUSG00000024793 | Tnfrsf25 |  | 2169 | 0 | 0 | 0 | 0 | 0 | 0 | 0 | 0.0176083 | 0 | 0.021738452 | 0 | 0 | 0.204696752 | 0.14988871 | 0 | 0.035307153 | 0.035340062 | 0 |
| ENSMUSG00000024795 | Kif20b |  | 5535 | 1.005994229 | 0.752323718 | 0.725384466 | 0.831375877 | 0.89567988 | 1.110124474 | 0.598353663 | 0.779718423 | 1.198017893 | 0.80927133 | 1.027140029 | 0.605047833 | 1.051701217 | 1.501985843 | 2.292697238 | 1.674133151 | 1.689542266 | 0.805011318 |
| ENSMUSG00000024797 | 1110014N23R |  | 5122 | 12.53081136 | 14.34756826 | 12.13588179 | 14.39237988 | 13.60699443 | 15.66299798 | 12.60870885 | 12.06468315 | 13.92718573 | 11.97638909 | 13.21693014 | 12.01965557 | 10.52709597 | 9.729501321 | 12.35564035 | 12.82084961 | 12.63076712 | 10.28870055 |
| ENSMUSG00000024798 | Htr7 |  | 5047 | 0 | 0 | 0.095846107 | 0 | 0 | 0 | 0 | 0.060538779 | 0 | 0 | 0 | 0 | 0 | 0 | 0 | 0 | 0 | 0 |
| ENSMUSG00000024799 | Tm7sf2 |  | 3027 | 5.392133332 | 3.51067501 | 4.570475613 | 3.85319701 | 3.307380285 | 3.372270604 | 9.847039222 | 7.305385197 | 7.660392591 | 5.841266509 | 5.618726534 | 5.937444121 | 7.284891649 | 10.15724673 | 6.438168463 | 5.502614542 | 5.735649983 | 5.797127534 |
| ENSMUSG00000024800 | Rpp30 |  | 1144 | 23.33326097 | 21.86886675 | 23.00279765 | 20.27147864 | 24.10803519 | 21.18119469 | 21.58847762 | 20.66529466 | 21.88933705 | 22.00915694 | 23.42810464 | 20.73571381 | 23.67414562 | 28.37798696 | 23.44599028 | 27.57994799 | 24.75798375 | 30.2934766 |
| ENSMUSG00000024803 | Ankrd1 |  | 1756 | 0.121028406 | 0.626039584 | 1.404925996 | 0.05189185 | 0.767479421 | 0.479724642 | 0 | 0.02174966 | 0 | 0.026851198 | 0 | 0 | 0 | 0 | 0 | 0 | 0 | 0.595201041 |
| ENSMUSG00000024805 | Pcgf5 |  | 1708 | 7.814183415 | 9.615489959 | 7.505260857 | 7.228947901 | 7.749577874 | 9.022775259 | 8.448692666 | 7.490898598 | 6.968912796 | 7.840046677 | 5.734113869 | 8.529208393 | 11.66867364 | 11.82855856 | 10.10740745 | 12.64397106 | 11.17476338 | 9.919674983 |
| ENSMUSG00000024806 | Mlana |  | 618 | 0.275114408 | 0.323426157 | 0.469645926 | 0.147446745 | 0.311533487 | 0.080182417 | 0.765577329 | 0.927000058 | 0.53315878 | 0.228886909 | 0.541140306 | 0.541899637 | 1.037726415 | 0.075152245 | 0.533353474 | 0.619589117 | 0.124033323 | 0.979126495 |

| ENSMUSG00000024807 | Syvn1 | 4954 | 10.00424611 | 13.17318526 | 10.38947039 | 11.88229093 | 9.939232522 | 10.06258579 | 10.23802822 | 10.0993232 | 8.554889931 | 14.17186055 | 8.544330483 | 11.77379237 | 11.01354471 | 12.05633758 | 10.46256708 | 10.99869486 | 11.27972162 | 8.494543125 |
| --- | --- | --- | --- | --- | --- | --- | --- | --- | --- | --- | --- | --- | --- | --- | --- | --- | --- | --- | --- | --- |
| ENSMUSG00000024810 | Il33 | 2922 | 0.290932074 | 0.10260645 | 0.132439508 | 0.405402859 | 0.280028303 | 0.016958499 | 0.599099631 | 0.143777011 | 0 | 0.435684116 | 0 | 0.114611217 | 0.438956143 | 0.174840849 | 0.112803712 | 0.078625477 | 0.354144427 | 0 |
| ENSMUSG00000024811 | Tnks2 | 9688 | 14.89523871 | 15.41168372 | 16.63214591 | 16.3940752 | 17.68679276 | 16.6948929 | 13.2737264 | 14.69272128 | 14.90077805 | 13.71497541 | 13.73382116 | 13.98272224 | 13.47360168 | 13.63887579 | 12.7968086 | 17.03868791 | 17.25237209 | 15.35916946 |
| ENSMUSG00000024812 | Tjp2 | 5077 | 68.38335194 | 82.49157103 | 73.67030465 | 72.34846158 | 71.94665525 | 83.81117259 | 48.96214597 | 59.12036449 | 51.12417221 | 54.03245845 | 52.3764859 | 61.69736034 | 57.83406965 | 59.18716671 | 61.92000619 | 77.72005304 | 76.6450448 | 66.22329334 |
| ENSMUSG00000024815 | Trpd52l3 | 2230 | 0 | 0 | 0 | 0 | 0 | 0 | 0.127298688 | 0 | 0 | 0 | 0 | 0 | 0 | 0 | 0 | 0 | 0 | 0 |
| ENSMUSG00000024816 | Frmd8 | 5071 | 9.295649816 | 9.013072544 | 9.37708151 | 10.86241424 | 8.324136671 | 8.794608602 | 4.739664937 | 6.770847912 | 5.482330534 | 8.805307826 | 5.633883883 | 7.913914691 | 5.710478296 | 5.513575352 | 6.532449406 | 6.712748961 | 6.756795392 | 7.278862278 |
| ENSMUSG00000024817 | Uhrf2 | 8874 | 9.01929755 | 9.264843693 | 8.847221069 | 9.960381565 | 8.624043118 | 8.945625332 | 9.015747134 | 11.43964453 | 8.655944545 | 9.239922579 | 8.226294406 | 10.03851673 | 9.989796636 | 7.918627926 | 8.979469134 | 10.3385503 | 8.78472931 | 7.996590903 |
| ENSMUSG00000024818 | Slc25a45 | 2411 | 1.81585779 | 2.265995293 | 1.865922158 | 2.494424659 | 1.137917731 | 1.870302263 | 3.748121806 | 2.677111575 | 4.612343117 | 5.06513154 | 4.220682769 | 6.134861296 | 4.419615982 | 4.006789787 | 3.862111834 | 5.542688504 | 5.357097493 | 2.509747714 |
| ENSMUSG00000024824 | Rad9 | 2035 | 16.50078087 | 15.82976349 | 16.37806507 | 15.08999699 | 15.37383311 | 14.95104591 | 13.78693789 | 13.73800421 | 20.88672446 | 13.78607786 | 14.57899224 | 13.74134986 | 16.67830913 | 13.60229783 | 15.245588 | 14.3001777 | 14.53950919 | 13.92122357 |
| ENSMUSG00000024826 | Dpf2 | 7731 | 16.36210113 | 15.27110298 | 17.07558716 | 16.91964277 | 16.70391297 | 16.26760287 | 17.79044854 | 18.76274018 | 17.73508097 | 20.7729007 | 23.60630467 | 20.62674273 | 17.95628188 | 17.02529345 | 21.07775883 | 21.34189979 | 22.99776113 | 22.12887361 |
| ENSMUSG00000024827 | Gldc | 4209 | 0.525129283 | 0.633174516 | 1.103316444 | 0.638655646 | 0.251580499 | 0.765247727 | 6.823187481 | 9.409722332 | 5.841850775 | 3.103051747 | 2.054470773 | 6.710079655 | 3.949835144 | 8.728266364 | 3.24013186 | 1.310013653 | 0.855944858 | 3.88161195 |
| ENSMUSG00000024829 | Mrpl21 | 2120 | 22.9969042 | 20.60056808 | 19.12123511 | 15.55952646 | 18.63976855 | 18.23166613 | 19.326783 | 18.32154398 | 20.72925109 | 19.46078554 | 22.28746884 | 19.29852862 | 18.80197602 | 23.50684234 | 20.21208402 | 21.51137425 | 18.44001077 | 20.55057195 |
| ENSMUSG00000024830 | Rps6kb2 | 2843 | 13.29127734 | 17.48253189 | 13.91823701 | 17.16351688 | 15.89724809 | 16.61053644 | 18.0064434 | 18.59243226 | 18.19566085 | 18.65794624 | 21.05593492 | 14.50853839 | 18.75758093 | 22.3480519 | 21.9992831 | 20.39112895 | 18.98115584 | 21.53539774 |
| ENSMUSG00000024831 | Ighmbp2 | 5994 | 1.992651729 | 2.034120668 | 1.88845614 | 2.02189486 | 1.911144116 | 2.041962828 | 1.239254353 | 2.465875831 | 1.628495868 | 2.666681415 | 2.470843698 | 2.085870607 | 2.049323891 | 2.494994351 | 2.976355305 | 2.370005897 | 2.154815158 | 2.624725479 |
| ENSMUSG00000024833 | Pola2 | 2853 | 2.994581276 | 3.211022051 | 3.187600318 | 2.666910061 | 2.63181917 | 3.456359615 | 4.01320305 | 4.538108803 | 2.858370179 | 3.140074875 | 4.839454259 | 3.110652068 | 4.755091921 | 4.086037832 | 2.888296943 | 4.898728252 | 3.627094344 | 4.916691978 |
| ENSMUSG00000024835 | Coro1b | 2465 | 102.788379 | 106.5877471 | 107.5992158 | 121.8411376 | 113.5835294 | 106.5233003 | 98.54088991 | 97.68888323 | 100.1843199 | 101.4935625 | 119.0788417 | 95.59988943 | 86.11571604 | 97.71157696 | 85.36160078 | 89.08585535 | 95.34152236 | 101.6496881 |
| ENSMUSG00000024837 | Dmrt1 | 3082 | 0 | 0 | 0 | 0 | 0 | 0 | 0 | 0 | 0 | 0 | 0 | 0 | 0 | 0 | 0 | 0 | 0 | 0 |
| ENSMUSG00000024841 | Eif1ad | 2463 | 19.27660642 | 20.13922026 | 17.61715661 | 16.370899 | 20.12825066 | 18.99219671 | 19.6319764 | 23.05810084 | 22.79221315 | 21.05796735 | 20.50269229 | 20.78073999 | 26.01792957 | 24.02345405 | 26.04580695 | 23.16402965 | 25.95544589 | 24.90262038 |
| ENSMUSG00000024842 | Cabp4 | 1425 | 0.029828194 | 0.233774696 | 0.509195056 | 0.255781302 | 0.135107154 | 0.312964633 | 0 | 0.45562866 | 0.173416908 | 0.23161749 | 0.167631433 | 0.470026632 | 0.138475557 | 0.228146394 | 0.028913373 | 0.134353008 | 0.080686941 | 0.038602882 |
| ENSMUSG00000024843 | Chka | 12362 | 10.05380478 | 12.48761946 | 11.27359173 | 11.12305709 | 11.10437197 | 10.89101902 | 8.630487865 | 10.30039392 | 10.23166073 | 8.837419467 | 8.239460563 | 9.314650697 | 9.896712089 | 10.11009862 | 8.865566953 | 13.6380476 | 12.37962332 | 13.83014914 |
| ENSMUSG00000024844 | Banf1 | 816 | 184.5016342 | 160.9714974 | 165.33551 | 161.9762128 | 158.7881599 | 153.5163119 | 180.3216072 | 170.8829181 | 157.5788017 | 170.6323857 | 159.0742184 | 158.5547866 | 175.4426764 | 159.5377169 | 157.3333923 | 157.7141317 | 143.9114874 | 154.7131127 |
| ENSMUSG00000024845 | Tmem134 | 4176 | 30.00604859 | 28.82969342 | 27.43020112 | 28.14834636 | 27.61592172 | 29.21427923 | 34.56680637 | 30.00700965 | 30.42634127 | 30.41762322 | 26.77045087 | 28.2553426 | 32.52173569 | 28.13782114 | 28.00048267 | 30.38675118 | 26.55123969 | 28.49250427 |
| ENSMUSG00000024846 | Cst6 | 3948 | 0.387585192 | 0.14344458 | 0.13477934 | 0.103862563 | 0.13410617 | 0.100410808 | 0.563246178 | 0.406302153 | 0.605070394 | 0.668804302 | 0.1815158 | 0.494819704 | 0.762220606 | 0.376446504 | 0.542675002 | 0.746802627 | 0.592174294 | 0.696670551 |
| ENSMUSG00000024847 | Aip | 2251 | 55.76089952 | 53.12896057 | 53.81044869 | 53.17141869 | 57.21947034 | 55.18822882 | 56.72897398 | 53.63224523 | 60.654509 | 54.08401413 | 57.68651139 | 59.73342122 | 52.70691295 | 48.56924998 | 50.77437407 | 55.69233613 | 48.57615507 | 48.55757222 |
| ENSMUSG00000024851 | Pitpnm1 | 6745 | 11.53216785 | 13.78447558 | 11.1162301 | 13.26640342 | 14.61440766 | 13.98055626 | 10.71812801 | 11.69270733 | 9.452442758 | 11.2266908 | 11.33991231 | 11.26243392 | 8.659598229 | 11.91915719 | 11.08685307 | 10.12189073 | 11.25071429 | 10.30860058 |
| ENSMUSG00000024853 | Sf3b2 | 3210 | 81.15707909 | 86.3852123 | 87.52444371 | 97.52334443 | 90.34107168 | 93.11591556 | 85.3691702 | 81.81026758 | 84.70821715 | 88.29373124 | 91.79935947 | 83.75826999 | 87.39894503 | 83.78744862 | 86.89549327 | 87.62704111 | 83.81638762 | 90.96209922 |
| ENSMUSG00000024854 | Pold4 | 906 | 56.11058557 | 44.0127311 | 39.61717387 | 48.37717953 | 45.369385 | 45.50538006 | 54.41480292 | 49.99579388 | 58.32483413 | 56.57043537 | 54.73554846 | 49.90142019 | 64.30573323 | 47.57186876 | 46.06752331 | 61.28187719 | 51.48245401 | 45.65877291 |
| ENSMUSG00000024855 | Pacs1 | 4353 | 15.94554387 | 21.42035169 | 18.8915694 | 21.41463283 | 17.91263872 | 19.52284358 | 12.70584003 | 16.9948733 | 12.19605302 | 16.86506891 | 17.3298092 | 15.95099569 | 19.19785616 | 18.14872333 | 19.28986237 | 17.94459892 | 17.35380914 | 19.42315576 |
| ENSMUSG00000024856 | Cdk2ap2 | 1512 | 67.38419771 | 60.50079863 | 56.9796677 | 60.47686242 | 56.91790973 | 57.84429548 | 72.65875693 | 74.086188 | 70.98681222 | 70.57013325 | 71.69403491 | 71.91065524 | 88.22321714 | 90.18507975 | 82.64835914 | 73.77019799 | 71.81111053 | 87.02498894 |
| ENSMUSG00000024857 | Cabp2 | 1338 | 0 | 0 | 0 | 0 | 0 | 0 | 0 | 0 | 0 | 0 | 0 | 0 | 0.184349467 | 0 | 0 | 0 | 0 | 0 |
| ENSMUSG00000024858 | Adrbk1 | 4504 | 16.57173382 | 21.0572402 | 17.7856497 | 16.73134266 | 16.08315833 | 16.93198423 | 14.65390477 | 19.58802166 | 16.67944155 | 19.55539824 | 17.78832268 | 15.65168336 | 19.37570197 | 16.98343959 | 16.40195153 | 17.53852644 | 16.05722075 | 15.66977885 |
| ENSMUSG00000024862 | Klc2 | 4529 | 2.271197306 | 2.464080275 | 2.915869681 | 2.162866976 | 3.135110948 | 2.680596098 | 2.329593155 | 2.959932269 | 2.919159099 | 2.321617758 | 2.289062926 | 3.179607186 | 2.287415021 | 3.096956147 | 3.038489659 | 1.969907601 | 3.181869648 | 1.943355504 |
| ENSMUSG00000024863 | Mbl2 | 1095 | 0.271722586 | 0.365072813 | 0.08835348 | 0 | 0 | 0.045253638 | 0.043207926 | 0.174394532 | 0.41374582 | 0.086120006 | 0 | 0 | 0.630727709 | 0.127244075 | 0 | 0 | 0 | 0 |
| ENSMUSG00000024866 | Acy3 | 1499 | 5.274157933 | 5.911427527 | 6.00231931 | 7.568178812 | 6.197105582 | 7.966783717 | 11.26792954 | 11.28701419 | 11.62234568 | 11.07207975 | 9.242653742 | 9.122639982 | 11.84755852 | 13.81858771 | 14.01787425 | 12.00572747 | 9.485694564 | 10.01833631 |
| ENSMUSG00000024867 | Pip5k1b | 4175 | 46.81168847 | 45.88801309 | 48.81381638 | 47.14340396 | 48.4662532 | 49.57647141 | 45.02353853 | 44.09278553 | 44.94509361 | 44.2369592 | 49.05658611 | 48.51617713 | 37.75220975 | 38.31220047 | 40.71799272 | 38.37315003 | 39.89606858 | 40.85826107 |
| ENSMUSG00000024868 | Dkk1 | 2423 | 0 | 0.02749723 | 0.019964313 | 0.056410703 | 0 | 0 | 0.039052975 | 0.141861998 | 0 | 0.058378914 | 0.019717275 | 0.046071533 | 0 | 0.01916801 | 0.017004357 | 0.015802975 | 0.015817704 | 0 |
| ENSMUSG00000024869 | Nudt8 | 4511 | 3.345009419 | 4.342270406 | 3.538741968 | 4.110694978 | 3.745135271 | 4.086370404 | 4.468011799 | 3.953857664 | 4.592511068 | 4.504977412 | 4.268079864 | 3.674850762 | 4.057224852 | 3.747649703 | 4.292780153 | 3.420774725 | 3.177573718 | 2.999831579 |
| ENSMUSG00000024870 | Rab1b | 1852 | 195.9323368 | 204.1763835 | 190.1246909 | 204.4342756 | 196.8938735 | 201.6357453 | 236.7677691 | 195.375173 | 198.5714899 | 221.4199062 | 221.2558417 | 185.4092636 | 198.3931533 | 206.3652241 | 206.3414854 | 194.1201474 | 192.7282954 | 191.1655566 |
| ENSMUSG00000024871 | Doc2g | 2817 | 1.508880938 | 0.839622112 | 0.772740102 | 0.841027442 | 0.905570449 | 0.967483261 | 1.360428468 | 1.030394953 | 1.096552603 | 0.78668195 | 1.407639884 | 0.97087947 | 1.033220845 | 0.890301995 | 0.906814507 | 0.910710222 | 0.503398291 | 0.702991779 |
| ENSMUSG00000024873 | Cnih2 | 1231 | 1.173985366 | 2.002562284 | 0.746626382 | 0.259079862 | 1.016596276 | 1.650415984 | 2.613535473 | 1.799479561 | 2.074381785 | 1.647018879 | 2.212162984 | 1.088201383 | 2.484629465 | 1.056404912 | 1.807379381 | 1.866317176 | 1.276505421 | 1.161849533 |
| ENSMUSG00000024875 | Yif1a | 1447 | 52.78631743 | 49.0139266 | 46.70201929 | 48.67821323 | 52.85530526 | 43.97077396 | 52.15184719 | 49.40993594 | 54.96279332 | 52.91827373 | 61.34476355 | 50.49253789 | 54.7185399 | 47.27860451 | 47.03868026 | 61.70953453 | 51.56966142 | 58.316496 |

| ENSMUSG00000024878 | Cbwd1 | 1360 | 18.15846123 | 21.13899096 | 17.17971703 | 21.03848224 | 19.64207179 | 20.25832343 | 19.7600012 | 20.10717656 | 19.59387919 | 23.43667309 | 24.16850841 | 19.20715448 | 26.87864022 | 23.15374357 | 19.66162483 | 24.94520453 | 25.87025041 | 18.97005224 |
| --- | --- | --- | --- | --- | --- | --- | --- | --- | --- | --- | --- | --- | --- | --- | --- | --- | --- | --- | --- | --- |
| ENSMUSG00000024883 | Rin1 | 4166 | 0.510143735 | 0.6157208 | 0.348345154 | 0.437456019 | 0.173302654 | 0.511466045 | 0.386133241 | 0.660070325 | 0.069204419 | 0.350857369 | 0.183485198 | 0.629701426 | 0.094732438 | 0.278709118 | 0.771416553 | 0.625002713 | 0.478388728 | 0.528171932 |
| ENSMUSG00000024885 | Aldh3b1 | 1855 | 7.676136911 | 4.05860598 | 4.354921601 | 8.129760472 | 8.017662756 | 5.743308744 | 13.26285339 | 7.926725027 | 11.27911052 | 11.36192149 | 8.96263372 | 10.0498282 | 11.46202498 | 14.47152696 | 14.32614747 | 15.21303378 | 14.60738108 | 10.97216684 |
| ENSMUSG00000024887 | Asah2 | 4910 | 0.51941152 | 0.766671496 | 0.906387941 | 0.593871046 | 1.048903428 | 0.807376515 | 1.387581622 | 0.894526737 | 1.392456541 | 0.749033575 | 1.235727032 | 1.079936445 | 0.452125515 | 1.021580741 | 0.914657758 | 1.044998247 | 1.537735334 | 1.176365826 |
| ENSMUSG00000024889 | Rce1 | 1438 | 44.27868824 | 38.54843949 | 35.25414451 | 38.84411708 | 37.62189305 | 38.76691605 | 36.98153763 | 39.01574349 | 40.98602507 | 40.23220647 | 38.40601669 | 41.49300647 | 46.3816079 | 46.79936205 | 39.08130893 | 48.80854198 | 35.74100427 | 40.43437121 |
| ENSMUSG00000024891 | Slc29a2 | 2460 | 1.434117728 | 0.690633173 | 0.275296514 | 1.074203484 | 0.997856954 | 1.228746646 | 0.961639816 | 1.009148844 | 0.585987013 | 0.97751458 | 0.699145734 | 0.748746693 | 0.842252246 | 1.510376826 | 0.870927198 | 0.389132189 | 0.498553456 | 0.156529978 |
| ENSMUSG00000024892 | Pcx | 4304 | 3.999673859 | 3.978352144 | 3.06830246 | 4.266055034 | 3.131258975 | 5.411195349 | 10.27819582 | 9.068920831 | 6.507163271 | 9.936265765 | 7.647989403 | 7.67724727 | 10.99193975 | 11.98870378 | 13.91892711 | 12.76650131 | 13.56202374 | 13.50943908 |
| ENSMUSG00000024893 | 2700046G09R | 1639 | 7.157674548 | 6.686969004 | 6.581633477 | 6.504749466 | 6.284461052 | 7.346744515 | 10.62298099 | 7.410118343 | 9.649555859 | 10.70168494 | 8.249123398 | 7.38987113 | 9.029637085 | 11.75979028 | 10.60833227 | 8.036588741 | 7.599784316 | 9.162590687 |
| ENSMUSG00000024896 | Minpp1 | 2614 | 21.31763036 | 21.66485087 | 23.46504839 | 25.90042536 | 21.2855791 | 24.81428013 | 21.59297091 | 19.98745466 | 24.07536193 | 23.93610682 | 16.88755227 | 25.43093119 | 21.60866308 | 20.94781141 | 23.4852021 | 23.75951232 | 25.64372352 | 22.79068959 |
| ENSMUSG00000024897 | Apba1 | 6883 | 0.043227696 | 0.101637481 | 0.126503494 | 0.178722679 | 0.04195722 | 0.107989395 | 0.570529181 | 0.299635294 | 0.448785222 | 0.219210011 | 0.152702177 | 0.218948553 | 0.451534329 | 0.141700688 | 0.263383475 | 0.211396641 | 0.350800044 | 0.119880372 |
| ENSMUSG00000024899 | Papss2 | 3622 | 0.187764444 | 0.174750135 | 0.320531399 | 0.314474354 | 0.358796781 | 0.082086251 | 0 | 0.021089123 | 0.068227249 | 0.260357279 | 0.092331505 | 0.616407096 | 0.299641684 | 0.076936644 | 0.068252163 | 0.211433503 | 0.497331848 | 0.242999919 |
| ENSMUSG00000024900 | Cpt1a | 4281 | 22.7965158 | 25.35234977 | 20.8703365 | 19.7739828 | 22.14900482 | 24.05292697 | 18.23544037 | 16.09415883 | 16.95179649 | 20.08943769 | 20.16569726 | 24.06814135 | 13.24045152 | 15.65494466 | 17.16009674 | 10.6258448 | 16.41916107 | 13.02948709 |
| ENSMUSG00000024901 | Peli3 | 4755 | 3.119727957 | 3.06156357 | 3.163862869 | 3.43025318 | 2.530595356 | 3.949629028 | 0.696506314 | 2.329294784 | 0.701599953 | 1.398159655 | 1.758279229 | 1.291214066 | 2.386191582 | 2.383250751 | 3.413966987 | 2.625181496 | 2.716290653 | 2.880603065 |
| ENSMUSG00000024902 | Mrpl11 | 816 | 80.42646052 | 75.89297837 | 90.52252555 | 72.02665097 | 84.58477762 | 81.13045596 | 102.3368607 | 83.68629351 | 84.08913635 | 87.82974136 | 94.32041435 | 88.92201147 | 95.09694347 | 88.79016707 | 97.0963137 | 78.36435583 | 85.85842002 | 74.55891181 |
| ENSMUSG00000024903 | Lao1 | 2497 | 0 | 0 | 0 | 0 | 0 | 0 | 0 | 0 | 0 | 0 | 0 | 0 | 0 | 0 | 0 | 0 | 0 | 0 |
| ENSMUSG00000024905 | Mtl5 | 4349 | 0 | 0.206817232 | 0.389301808 | 0.052381058 | 0.088538834 | 0.079758366 | 0.087031831 | 0.079036933 | 0.227288199 | 0.21683469 | 0.010985274 | 0 | 0.124776061 | 0.160188851 | 0.0663166 | 0.114458012 | 0.114564695 | 0.088540756 |
| ENSMUSG00000024906 | Mus81 | 3532 | 8.135192241 | 8.535721763 | 9.655531965 | 9.042551551 | 8.367214364 | 10.05926103 | 10.85030293 | 10.21852216 | 7.463015314 | 8.810720314 | 9.481949573 | 7.095479191 | 9.693190994 | 9.388753793 | 8.480614703 | 9.767790846 | 8.724332591 | 12.33499788 |
| ENSMUSG00000024907 | Gal | 706 | 0 | 0.566224831 | 0.137035497 | 0 | 0 | 0 | 0 | 0 | 0 | 0 | 0.203009739 | 0 | 0 | 0 | 0.058359144 | 0 | 0.271432698 | 0 |
| ENSMUSG00000024908 | Ppp6r3 | 4928 | 13.89525945 | 15.1287048 | 15.84311648 | 16.27180155 | 15.72491824 | 14.0473151 | 13.67152086 | 12.0823773 | 14.91841125 | 15.48089243 | 14.46433402 | 13.31964675 | 12.57323216 | 10.56489894 | 14.38041318 | 13.52758675 | 17.15661749 | 16.66570543 |
| ENSMUSG00000024909 | Efemp2 | 2392 | 2.523300584 | 1.14199721 | 2.264981354 | 2.342812899 | 1.408542917 | 1.94730642 | 2.452663958 | 3.289145023 | 2.427800469 | 2.64138555 | 2.496601091 | 3.406808837 | 1.546778343 | 0.427161339 | 1.067933305 | 2.11302683 | 1.650338036 | 2.092737756 |
| ENSMUSG00000024910 | Ctsw | 1222 | 0.034783286 | 0.245348648 | 0.356269863 | 0 | 0.236326957 | 0.081101037 | 0 | 0 | 0 | 0 | 0 | 0 | 0 | 0.038006618 | 0 | 0.344678135 | 0.062727164 | 0 |
| ENSMUSG00000024911 | Fibp | 1199 | 91.35599552 | 81.37903851 | 79.88289416 | 83.82624171 | 81.41079333 | 84.26857695 | 72.17255193 | 78.74196726 | 73.37314234 | 80.22304808 | 77.30060004 | 82.25711075 | 83.35818549 | 76.19309416 | 79.10423825 | 86.6408823 | 72.59301106 | 81.71077487 |
| ENSMUSG00000024912 | Fosl1 | 1703 | 0.124794997 | 0.136929101 | 0.085214675 | 0.026753402 | 0.282630204 | 0.145486593 | 0.111127842 | 1.12132714 | 0.096738733 | 0.166121092 | 0.112213643 | 0.065549809 | 0.405547177 | 1.390868148 | 0.169354604 | 0.562105217 | 0.990227283 | 0.742929803 |
| ENSMUSG00000024913 | Lrp5 | 10175 | 1.867303556 | 1.587887341 | 1.606904497 | 1.191079881 | 1.598878644 | 1.407443735 | 1.399618315 | 1.625288476 | 1.145531593 | 1.524578022 | 1.108097317 | 1.711497467 | 1.532077234 | 1.369358842 | 1.984153552 | 1.249383947 | 1.461484344 | 1.04341598 |
| ENSMUSG00000024914 | Drap1 | 1907 | 91.1398347 | 77.4040033 | 78.68625651 | 76.42883106 | 78.19229131 | 80.03273174 | 103.9787296 | 81.87233405 | 90.83926864 | 90.64209656 | 77.98817291 | 88.12845309 | 107.6661981 | 106.0639751 | 98.75842264 | 107.0008636 | 85.07352637 | 102.6336663 |
| ENSMUSG00000024921 | Smarca2 | 7924 | 10.5351042 | 12.25480649 | 8.815166309 | 12.26997332 | 10.66628697 | 11.2062719 | 11.33259271 | 10.01080512 | 10.59809511 | 12.44221612 | 10.44851124 | 12.71419371 | 10.77034539 | 11.32382343 | 13.2693552 | 12.60729363 | 13.10271811 | 14.24516494 |
| ENSMUSG00000024922 | Ovol1 | 2896 | 0.366930042 | 0.47162592 | 0.267257074 | 0.409042525 | 0.398883345 | 0.290882759 | 1.503027093 | 0.817655024 | 1.592848676 | 1.107129773 | 1.022806432 | 1.676782681 | 3.032141324 | 3.48009909 | 1.749927961 | 3.014592019 | 2.196880278 | 2.906213166 |
| ENSMUSG00000024924 | Vldlr | 9715 | 0.105005067 | 0.133731639 | 0.144398598 | 0.084415728 | 0.163494954 | 0.188723741 | 0.56492751 | 0.314502542 | 0.542653013 | 0.349444224 | 0.177035358 | 0.373444989 | 0.035545386 | 0.191226299 | 0.021205124 | 0.126124492 | 0.007890128 | 0.277452005 |
| ENSMUSG00000024925 | Rnaseh2c | 1610 | 16.76446384 | 14.6700882 | 17.48658054 | 13.69661209 | 13.1540661 | 13.63469626 | 12.6069188 | 11.41027674 | 11.71641256 | 12.41732806 | 13.0268365 | 12.06450347 | 15.41239403 | 15.11596385 | 12.74433219 | 9.941288133 | 9.141178889 | 12.57351011 |
| ENSMUSG00000024926 | Kat5 | 2476 | 14.31717157 | 16.83135326 | 12.23014136 | 15.67770992 | 13.39373805 | 13.28877831 | 13.14665715 | 16.62819459 | 16.86717568 | 15.00595887 | 15.32040268 | 14.60765321 | 13.52842158 | 13.69312754 | 13.89470887 | 15.60388646 | 14.27174707 | 11.30841491 |
| ENSMUSG00000024927 | Rela | 2787 | 76.34765382 | 95.98225353 | 84.73612314 | 102.99052 | 91.56636501 | 93.45144158 | 60.11273631 | 83.23668899 | 65.86591344 | 75.45465968 | 73.38521609 | 63.8064948 | 51.54450775 | 61.59215895 | 62.77065171 | 82.35159697 | 77.61522057 | 78.18122416 |
| ENSMUSG00000024935 | Slc1a1 | 6416 | 6.022008262 | 7.06133668 | 6.416127546 | 7.257385803 | 7.651895596 | 6.827402818 | 16.02407284 | 18.78666178 | 15.00842797 | 18.12979815 | 17.92305565 | 15.97218774 | 22.38235835 | 27.73181091 | 28.15910576 | 13.9650906 | 19.87997445 | 17.55044287 |
| ENSMUSG00000024936 | Kcnk7 | 1223 | 0.938380828 | 0.871637461 | 1.54257374 | 0.409788624 | 0.236133722 | 0.810347238 | 0.967143887 | 0.624569132 | 0.471472789 | 0.693959655 | 2.343824619 | 1.506064485 | 2.17818768 | 1.443070581 | 1.246490244 | 1.346276466 | 2.381683209 | 1.259407186 |
| ENSMUSG00000024937 | Ehbp1l1 | 6420 | 0.456831331 | 0.269824065 | 0.158231174 | 0.255482492 | 0.352367665 | 0.324176762 | 0.397957112 | 0.297448617 | 0.218121735 | 0.345184276 | 0.476261268 | 0.486865593 | 0.238207077 | 0.803005249 | 0.693110286 | 0.793247786 | 0.399978488 | 0.788292495 |
| ENSMUSG00000024939 | Fam89b | 1949 | 0 | 0.017092301 | 0 | 0.023376626 | 0 | 0 | 0 | 0 | 0 | 0 | 0.049025098 | 0 | 0 | 0.023829701 | 0 | 0 | 0.019664596 | 0 |
| ENSMUSG00000024940 | Ltbp3 | 5165 | 18.65619246 | 27.94026288 | 23.66697216 | 25.89878532 | 25.53368264 | 22.6225258 | 11.19382258 | 12.44488156 | 13.19722819 | 15.34565967 | 11.07195069 | 11.72507142 | 11.61425197 | 9.288817478 | 12.99464367 | 15.783292 | 14.26940347 | 12.04555657 |
| ENSMUSG00000024941 | Scyl1 | 2620 | 35.70759253 | 42.11156704 | 38.58804521 | 47.21306694 | 39.99358696 | 42.49808868 | 39.09616408 | 41.23904825 | 38.16826728 | 40.04210487 | 46.80851848 | 30.2086296 | 36.45289765 | 37.03118265 | 35.69753125 | 36.05455285 | 41.42750871 | 40.45898804 |
| ENSMUSG00000024942 | Capn1 | 3628 | 9.302400707 | 11.23896982 | 11.93338194 | 13.86422077 | 10.2684975 | 13.30329727 | 10.49798967 | 12.35884245 | 13.9293977 | 14.59488417 | 12.06225523 | 13.40006673 | 12.29218666 | 11.08615757 | 19.88531543 | 16.28511776 | 14.52553976 | 18.4526138 |
| ENSMUSG00000024943 | Smc5 | 3437 | 9.893552756 | 7.918718236 | 7.543849948 | 7.357107828 | 7.954301031 | 8.837889346 | 11.45305466 | 6.822849891 | 6.578817909 | 9.630431681 | 5.726878933 | 8.736929436 | 9.774523033 | 8.378043114 | 6.017800715 | 8.010167798 | 8.329864355 | 6.834125275 |
| ENSMUSG00000024944 | Arl2 | 3305 | 47.04506321 | 46.85980188 | 47.10015754 | 45.85053963 | 47.66589596 | 47.93346119 | 53.21065887 | 48.03806862 | 48.15270708 | 50.60319046 | 57.43113768 | 47.48975588 | 54.67558638 | 44.04108014 | 42.29860186 | 47.99939076 | 45.42332979 | 47.10310803 |
| ENSMUSG00000024947 | Men1 | 3201 | 13.43806252 | 15.65216898 | 13.37412507 | 15.24394829 | 15.03652723 | 16.6259406 | 14.15980831 | 13.39895904 | 15.22138336 | 15.71690109 | 12.82058398 | 14.73421896 | 11.35820776 | 8.545944227 | 14.14573881 | 10.69409654 | 12.78740553 | 11.35926884 |

| ENSMUSG00000024948 | Map4k2 | 4502 | 5.579866511 | 6.001056689 | 4.35168365 | 4.341556646 | 4.843127815 | 4.083532687 | 4.214212406 | 6.116553117 | 6.84307281 | 5.477525056 | 7.523866184 | 5.690668398 | 5.971989097 | 5.643028832 | 4.777257258 | 5.281756374 | 6.827563331 | 3.763395129 |
| --- | --- | --- | --- | --- | --- | --- | --- | --- | --- | --- | --- | --- | --- | --- | --- | --- | --- | --- | --- | --- |
| ENSMUSG00000024949 | Sf1 | 5763 | 28.92682637 | 29.94865607 | 30.1589614 | 32.58764962 | 29.55732741 | 28.60696592 | 27.17420914 | 31.63150038 | 30.01620097 | 28.4311459 | 27.92882789 | 29.7141164 | 22.5730116 | 26.94933684 | 30.34173229 | 28.22462263 | 31.616036 | 30.03880945 |
| ENSMUSG00000024952 | Rps6ka4 | 3764 | 7.023969046 | 9.292916825 | 9.84434757 | 9.514075677 | 9.526642703 | 9.636716519 | 10.83515655 | 10.17719968 | 5.930683194 | 7.929435484 | 7.298246845 | 7.236462099 | 7.208436371 | 3.911470697 | 5.407430555 | 8.94193515 | 6.394504372 | 6.050417158 |
| ENSMUSG00000024953 | Prdx5 | 1645 | 73.25360124 | 67.27382042 | 64.37061273 | 69.29710208 | 73.58771308 | 69.04247134 | 114.8159357 | 96.60704341 | 87.53073502 | 98.68685186 | 92.90704691 | 95.51434054 | 116.8071841 | 111.9175455 | 85.18327756 | 101.4177364 | 90.81817953 | 96.8093398 |
| ENSMUSG00000024955 | Esrra | 2531 | 13.7037628 | 11.77994481 | 13.07289402 | 10.13467719 | 9.603564383 | 10.55271568 | 11.68329685 | 13.30924493 | 16.82609929 | 16.03980858 | 11.55206424 | 13.49631984 | 11.92853946 | 11.78076021 | 12.61604339 | 16.83818492 | 10.55449583 | 10.58452587 |
| ENSMUSG00000024957 | Kcnk4 | 1708 | 0 | 0.078016146 | 0.028321739 | 0.29342593 | 0 | 0.116048556 | 0.083101895 | 0.022360891 | 0 | 0.496904367 | 0.167827723 | 0.130715837 | 0 | 0.244728798 | 0 | 0.044836777 | 0.291710691 | 0 |
| ENSMUSG00000024958 | Gpr137 | 3048 | 14.79592906 | 13.59620748 | 13.72805241 | 16.33799917 | 15.52286121 | 12.29063864 | 19.79122888 | 16.4648349 | 20.43110624 | 16.93898296 | 19.16954535 | 17.7628585 | 17.51218323 | 19.06218941 | 17.18083253 | 16.84635975 | 14.63642047 | 14.7990379 |
| ENSMUSG00000024959 | Bad | 2112 | 22.64125144 | 16.95613697 | 17.2010044 | 17.36583366 | 18.27736874 | 18.2068756 | 22.31222927 | 17.25168177 | 24.37648897 | 20.91865954 | 19.95147414 | 21.03658495 | 25.62363317 | 21.41881682 | 17.10879002 | 19.65294433 | 17.23957484 | 20.02935751 |
| ENSMUSG00000024960 | Plcb3 | 4200 | 16.73894313 | 18.53624614 | 15.98630003 | 20.06855524 | 15.1959359 | 16.05744533 | 15.80468775 | 15.4133624 | 14.05244693 | 15.94142824 | 14.63961228 | 15.54864886 | 11.82791445 | 13.24762302 | 16.33347394 | 13.26496042 | 12.29179094 | 14.43334181 |
| ENSMUSG00000024962 | Vegfb | 1598 | 55.43228213 | 46.86319537 | 46.28481096 | 45.64657821 | 45.93315619 | 46.3277747 | 53.26377309 | 51.40917245 | 52.86204245 | 57.59585279 | 50.28628295 | 49.2141856 | 53.12905484 | 40.89288545 | 43.1095128 | 50.72666825 | 40.9645276 | 51.60115831 |
| ENSMUSG00000024963 | Dnajc4 | 1080 | 43.60716207 | 37.63123236 | 42.05809723 | 35.30980936 | 45.90359387 | 35.00808863 | 42.537603 | 41.83482597 | 39.88990323 | 40.51467835 | 46.4478763 | 41.7583846 | 42.80000601 | 45.49800413 | 41.23970548 | 41.83603401 | 40.95235799 | 43.29420437 |
| ENSMUSG00000024965 | Fermt3 | 2572 | 0.099156709 | 0.388564085 | 0.338539481 | 0.389713443 | 0.318134799 | 0.520188105 | 0.441486895 | 1.009752474 | 1.809516439 | 0.714960119 | 1.003051228 | 0.347220296 | 1.937217591 | 1.300145526 | 0.849021175 | 0.878361523 | 0.476843507 | 0.406365874 |
| ENSMUSG00000024966 | Stip1 | 2159 | 68.11853129 | 62.95350089 | 72.34744304 | 66.9593301 | 70.31407469 | 62.97948168 | 59.387383 | 58.71263714 | 57.24906614 | 59.42429994 | 70.30154844 | 58.27165515 | 49.26336899 | 62.29832194 | 62.07904646 | 60.81449411 | 58.45692251 | 63.49360535 |
| ENSMUSG00000024968 | Rcor2 | 3142 | 0.40584191 | 0.265061221 | 1.293245242 | 0.855538388 | 0.53616083 | 0.520445642 | 0.31622096 | 0.474062283 | 0.183517257 | 0.765336049 | 0.638621342 | 0.497402467 | 1.193260889 | 0.591267822 | 0.354055382 | 0.463094552 | 0.524516476 | 0.280122759 |
| ENSMUSG00000024969 | Mark2 | 6874 | 18.9090527 | 20.58188271 | 20.38669151 | 23.3969285 | 19.24856825 | 21.13596375 | 17.3172389 | 18.496146 | 17.98090396 | 20.52297122 | 15.41531247 | 18.0503663 | 16.49899299 | 17.18174487 | 19.30011783 | 17.37393141 | 18.70037821 | 17.69350232 |
| ENSMUSG00000024970 | AI846148 | 3177 | 8.763264179 | 7.644035217 | 7.765344816 | 7.672382348 | 8.559816453 | 9.140038362 | 8.697074125 | 8.042403906 | 7.609847262 | 6.099760478 | 9.173032632 | 9.047864725 | 6.84777322 | 7.133998944 | 7.91090623 | 7.62919562 | 8.98743821 | 10.97758063 |
| ENSMUSG00000024972 | Lgals12 | 4265 | 0.657758879 | 1.06226345 | 1.134197664 | 1.185762232 | 1.049535498 | 0.952713752 | 0.022186485 | 0 | 0 | 0.165828968 | 0 | 0 | 0 | 0.174233388 | 0.019320777 | 0.215468834 | 0.20668343 | 0.37403613 |
| ENSMUSG00000024973 | Hrasls5 | 1676 | 0.253610835 | 0 | 0 | 0.027184394 | 0.057436663 | 0 | 0 | 0 | 0 | 0 | 0 | 0 | 0 | 0 | 0 | 0 | 0 | 0 |
| ENSMUSG00000024974 | Smc3 | 5672 | 13.09177407 | 11.11800225 | 11.88017062 | 9.952421356 | 11.091048 | 12.82500227 | 12.12846177 | 13.81713853 | 14.21050976 | 12.24488468 | 13.51040786 | 12.20229577 | 10.7065568 | 12.52811243 | 13.54740863 | 12.29322921 | 13.8655785 | 12.27812572 |
| ENSMUSG00000024975 | Pdcd4 | 2543 | 19.75663314 | 19.24366559 | 19.84018567 | 18.23875074 | 18.22691407 | 20.20691495 | 17.99109734 | 22.84335197 | 15.72634951 | 21.22986836 | 17.03967257 | 18.48084463 | 17.86657326 | 18.95751581 | 15.45665919 | 20.77901621 | 20.43667267 | 16.56978991 |
| ENSMUSG00000024976 | Shoc2 | 4193 | 25.70787656 | 27.09205087 | 29.7301664 | 27.8712327 | 28.03199561 | 31.74305805 | 21.20212824 | 24.09227388 | 22.03228115 | 26.21232752 | 26.34288195 | 22.96256093 | 21.38932163 | 22.07561797 | 21.05769956 | 24.78433305 | 26.89147758 | 20.03312806 |
| ENSMUSG00000024978 | Gpam | 6322 | 13.46019652 | 14.71205325 | 19.38920056 | 16.78451001 | 17.62502425 | 17.49473289 | 18.44760417 | 18.53437054 | 20.4108437 | 21.65113754 | 23.5776448 | 22.62820994 | 10.79184461 | 14.21532887 | 15.17197439 | 9.496942804 | 16.24108659 | 11.57262131 |
| ENSMUSG00000024979 | Tectb | 3888 | 0 | 0.017136262 | 0 | 0 | 0 | 0.012745045 | 0 | 0.039292595 | 0 | 0 | 0 | 0.057423521 | 0.088817752 | 0 | 0 | 0 | 0 | 0 |
| ENSMUSG00000024981 | Acsl5 | 3510 | 50.75190676 | 49.5707255 | 48.33218546 | 50.88298969 | 55.79737818 | 60.19739487 | 58.60841253 | 58.63785087 | 63.0587851 | 60.24812083 | 66.57188659 | 61.52444403 | 53.74508594 | 47.14537988 | 55.6866613 | 59.73771115 | 66.07191526 | 61.49736034 |
| ENSMUSG00000024982 | Zdhhc6 | 2518 | 36.51258767 | 39.25312039 | 36.3281755 | 40.94703865 | 34.3499074 | 39.29976527 | 34.85505139 | 40.89226246 | 36.50846036 | 45.20325562 | 42.89880108 | 36.55283066 | 38.81117082 | 38.84481654 | 41.46336085 | 41.19509748 | 45.98242373 | 42.6003805 |
| ENSMUSG00000024983 | Vti1a | 8152 | 6.136971563 | 6.803970667 | 6.776566691 | 8.327521597 | 7.238682338 | 7.16666743 | 5.943103928 | 7.421094874 | 6.320452799 | 6.240874487 | 7.261184195 | 5.59389062 | 5.252711506 | 5.383913467 | 6.337923342 | 6.449092733 | 8.048898466 | 4.757289038 |
| ENSMUSG00000024985 | Tcf7l2 | 8767 | 4.504084468 | 4.825752898 | 5.208693128 | 5.051367069 | 5.204638261 | 4.923055885 | 4.403689518 | 5.10132351 | 5.355609441 | 5.781567925 | 4.800928302 | 4.596658878 | 5.024911843 | 5.488316931 | 5.319968207 | 7.992678401 | 7.558591006 | 7.065159595 |
| ENSMUSG00000024986 | Hhex | 3833 | 0 | 0 | 0 | 0 | 0.012557246 | 0 | 0.135778625 | 0.129533324 | 0.107452428 | 0 | 0 | 0 | 0.193054725 | 0 | 0.397719167 | 0.049948614 | 0 | 0 |
| ENSMUSG00000024987 | Cyp26a1 | 1766 | 0 | 0 | 0 | 0.103196023 | 0 | 0 | 0.589399171 | 1.275963614 | 0 | 0.10679661 | 0.162315827 | 0.916565241 | 0 | 0 | 0 | 0 | 0 | 0 |
| ENSMUSG00000024989 | Cep55 | 1882 | 0.42911708 | 0.283212703 | 0.179922802 | 0.266297284 | 0.255748797 | 0.052659653 | 0.553070636 | 0 | 0.415804357 | 0.37580263 | 0.863097018 | 0 | 0.209699967 | 0 | 0.547310785 | 0 | 0.407293272 | 0.17537441 |
| ENSMUSG00000024990 | Rbp4 | 1290 | 0 | 0 | 0 | 0 | 0 | 0 | 0 | 0 | 0 | 0.036550933 | 0 | 0 | 0 | 0 | 0 | 0.089047924 | 0 | 0 |
| ENSMUSG00000024991 | Eif3a | 5124 | 40.85440904 | 35.94593914 | 38.19658544 | 36.99834221 | 38.73849594 | 39.35980204 | 37.61745784 | 30.60460661 | 31.77406261 | 36.08998013 | 34.22753173 | 29.97749872 | 30.50992308 | 33.75444603 | 33.61094918 | 34.74850203 | 34.01795439 | 33.26955928 |
| ENSMUSG00000024992 | Pde6c | 2937 | 0 | 0 | 0 | 0 | 0.016388125 | 0 | 0 | 0 | 0 | 0.016054036 | 0 | 0 | 0 | 0 | 0 | 0 | 0 | 0 |
| ENSMUSG00000024993 | Fam45a | 5486 | 16.44886779 | 16.12815294 | 15.19278031 | 15.40571222 | 16.75756001 | 15.61733072 | 13.94542505 | 15.63618954 | 17.25989787 | 13.72578802 | 14.49969119 | 16.1362816 | 14.66648871 | 12.69042782 | 15.55385021 | 15.94860334 | 18.82781082 | 12.66432771 |
| ENSMUSG00000024997 | Prdx3 | 1471 | 107.1731461 | 94.59412587 | 97.43764136 | 95.82996001 | 110.4318439 | 98.26330649 | 131.6456797 | 118.6275232 | 119.6116896 | 126.8357123 | 130.4311579 | 109.4305717 | 123.8160697 | 120.0410743 | 120.2435618 | 121.1451304 | 118.9391879 | 113.6829942 |
| ENSMUSG00000024998 | Plce1 | 10759 | 2.133357659 | 2.696861313 | 2.122158781 | 2.08770284 | 2.241295079 | 2.049536801 | 0.677214663 | 0.798707179 | 0.382809887 | 0.666131322 | 0.528415286 | 0.855988877 | 0.76113935 | 0.461893982 | 0.570595021 | 0.320304365 | 0.723137677 | 0.409027655 |
| ENSMUSG00000024999 | Noc3l | 3956 | 1.729861815 | 2.197842617 | 3.30153013 | 3.362948672 | 3.333707554 | 2.668031409 | 2.403905072 | 2.625969023 | 2.748544025 | 2.860507781 | 2.318703748 | 2.708950257 | 1.621119627 | 2.911560585 | 2.624568272 | 1.645450773 | 2.073262775 | 2.224837481 |
| ENSMUSG00000025001 | Hells | 7057 | 0.283086761 | 0.703361377 | 0.246768753 | 0.419649693 | 0.150049925 | 0.287893167 | 0.402261688 | 0.389663168 | 0.373520902 | 0.374158904 | 0.724375876 | 0.672287278 | 0.643125463 | 0.348807798 | 0.198505441 | 0.363535595 | 0.792920412 | 0.358568642 |
| ENSMUSG00000025002 | Cyp2c55 | 1986 | 0 | 0 | 0 | 0 | 0 | 0 | 0 | 0 | 0 | 0 | 0 | 0 | 0 | 0 | 0 | 0 | 0 | 0 |
| ENSMUSG00000025003 | Cyp2c39 | 1808 | 0 | 0 | 0 | 0.050399385 | 0.186351474 | 0 | 0 | 0.021124116 | 0 | 0 | 0 | 0 | 0.54570705 | 0.35963342 | 0.113942356 | 0 | 0.021198173 | 0.091276173 |
| ENSMUSG00000025004 | Cyp2c40 | 2327 | 0 | 0 | 0.020787937 | 0 | 0.06205233 | 0 | 0 | 0 | 0 | 0 | 0.020530708 | 0 | 0 | 0 | 0.177058685 | 0.016454924 | 0.016470261 | 0.070918487 |

| ENSMUSG00000025006 | Sorbs1 | 4886 | 5.628499568 | 5.720327108 | 5.544244167 | 6.965636537 | 5.083109417 | 6.206768921 | 4.706091273 | 5.057405722 | 6.979622401 | 6.234006202 | 4.830296662 | 4.900720272 | 9.591756327 | 9.353455176 | 13.04519176 | 14.06705696 | 11.92303956 | 10.33531723 |
| --- | --- | --- | --- | --- | --- | --- | --- | --- | --- | --- | --- | --- | --- | --- | --- | --- | --- | --- | --- | --- |
| ENSMUSG00000025007 | Aldh18a1 | 4231 | 6.28887738 | 11.18040884 | 8.712037374 | 9.250044218 | 9.078060763 | 9.943339827 | 3.589546191 | 4.83836627 | 4.711480413 | 3.120349069 | 3.01486975 | 2.334996993 | 3.684444779 | 3.358990957 | 4.46975045 | 2.235353351 | 3.107047941 | 3.419379595 |
| ENSMUSG00000025008 | Tctn3 | 3259 | 8.764491651 | 9.168967817 | 8.683189708 | 9.772068116 | 9.201039719 | 8.256254782 | 9.828377918 | 9.972916369 | 10.26187505 | 9.982812288 | 12.29922988 | 8.974350167 | 11.82210107 | 14.08001176 | 12.51596819 | 13.20608859 | 12.08942412 | 10.87016408 |
| ENSMUSG00000025010 | Ccnj | 4484 | 1.241788149 | 2.696828859 | 2.373366788 | 1.493638162 | 1.899944355 | 1.856569858 | 1.192313274 | 2.589315417 | 1.69008004 | 1.587813602 | 2.727562304 | 1.25722166 | 1.298208349 | 2.361563766 | 1.653942922 | 0.990568791 | 1.324838541 | 0.34350022 |
| ENSMUSG00000025013 | Tll2 | 3421 | 0.024849562 | 0.068164355 | 0.042420518 | 0.079908292 | 0.365808248 | 0 | 0.069150364 | 0.279102619 | 0.012039321 | 0.220523605 | 0 | 0.016315599 | 0.937320849 | 1.194703213 | 1.782470117 | 0.43651964 | 0.795430307 | 1.415025253 |
| ENSMUSG00000025014 | Dntt | 2116 | 0 | 0 | 0 | 0 | 0 | 0 | 0 | 0 | 0 | 0 | 0 | 0 | 0 | 0 | 0 | 0 | 0 | 0 |
| ENSMUSG00000025016 | Tm9sf3 | 10424 | 41.63664164 | 47.5788919 | 46.74932318 | 46.70618954 | 48.53363869 | 50.24202237 | 38.00355532 | 41.40563503 | 41.09563991 | 41.62777457 | 40.8681221 | 44.41050513 | 35.91992058 | 38.84301169 | 47.14226373 | 42.44512361 | 50.97059228 | 41.65261698 |
| ENSMUSG00000025017 | Pik3ap1 | 4604 | 0.923222763 | 0.101298983 | 0.714465696 | 0.851053391 | 1.045437091 | 1.130112299 | 0.719350027 | 1.020344374 | 1.690758358 | 1.04460724 | 0.954669023 | 1.175959876 | 2.025137351 | 2.45132781 | 0.760671644 | 0.931483065 | 2.330878178 | 1.863905441 |
| ENSMUSG00000025019 | Lcor | 7620 | 1.879822089 | 1.871117943 | 2.075871972 | 1.949199535 | 2.387646607 | 1.762308504 | 1.577089298 | 2.285529593 | 2.502540262 | 1.571690109 | 2.000027789 | 2.732184008 | 2.460121859 | 2.072308361 | 2.162811333 | 3.547659951 | 2.866927721 | 2.714360122 |
| ENSMUSG00000025020 | Slit1 | 6526 | 0.045592435 | 0.091883557 | 0.274259979 | 0.328128882 | 0.140132784 | 0.0303725 | 0.057998993 | 0.468187587 | 0.006311142 | 0.281777111 | 0.270866298 | 0.085528138 | 0.597183798 | 1.423355421 | 0.713419524 | 0.428319697 | 0.193804443 | 0.547899469 |
| ENSMUSG00000025024 | Smndc1 | 2119 | 20.38001833 | 18.14208586 | 20.38582473 | 16.66343056 | 20.19314778 | 20.64892107 | 20.74255721 | 19.46568881 | 22.15791787 | 22.11788534 | 25.2289658 | 19.83444735 | 18.53148002 | 18.323387 | 19.28831687 | 21.6299467 | 22.51828204 | 21.18330868 |
| ENSMUSG00000025025 | Mxi1 | 5611 | 17.49901205 | 17.88838893 | 20.48396153 | 20.32423703 | 20.08141745 | 20.63887692 | 21.03816324 | 21.97883931 | 26.11684603 | 21.7560454 | 20.2730665 | 22.81966316 | 18.12031394 | 16.55465599 | 18.7833862 | 23.28418329 | 21.42748055 | 19.7154364 |
| ENSMUSG00000025026 | Add3 | 4920 | 47.55914513 | 51.58758965 | 59.80816771 | 60.35912337 | 56.92676094 | 57.21729257 | 43.36033929 | 50.25561243 | 48.76249071 | 56.95459948 | 59.22346987 | 59.9337694 | 42.1025855 | 46.20809101 | 52.61572676 | 50.70392423 | 65.46630064 | 53.17546983 |
| ENSMUSG00000025027 | Xpnpep1 | 2715 | 142.8580962 | 162.2699912 | 158.1449191 | 159.6902018 | 137.6590745 | 145.153919 | 120.224741 | 131.0639459 | 115.0946943 | 130.2331947 | 130.7960465 | 129.5170071 | 145.4882732 | 145.3366359 | 144.3951397 | 130.8368195 | 145.1176177 | 128.8408507 |
| ENSMUSG00000025034 | Trim8 | 3224 | 57.62720981 | 56.12768033 | 54.79532657 | 69.28839962 | 48.95303279 | 58.23675792 | 62.35470619 | 59.07739169 | 53.29719099 | 73.10991488 | 57.79228848 | 60.33423825 | 52.17798943 | 51.05392237 | 54.58183784 | 68.33875775 | 54.62448333 | 59.56476165 |
| ENSMUSG00000025035 | Arl3 | 816 | 99.12665439 | 77.03606788 | 87.91414892 | 79.50848913 | 76.26786434 | 74.6934587 | 137.4155013 | 116.9642324 | 151.2695928 | 139.0252353 | 120.7839943 | 124.1488083 | 166.3743092 | 142.6903518 | 146.0231613 | 177.938705 | 140.1540073 | 138.4665505 |
| ENSMUSG00000025036 | Sfxn2 | 3198 | 6.977866609 | 7.541755915 | 8.924447439 | 8.092142959 | 8.202594871 | 8.553192287 | 16.836094 | 15.48953906 | 18.95764578 | 14.89124775 | 17.09022905 | 14.97493386 | 16.93760012 | 17.020785 | 17.88235134 | 15.46950118 | 16.07115827 | 18.19876014 |
| ENSMUSG00000025037 | Maoa | 4152 | 0.880405862 | 1.002917095 | 1.293222994 | 1.075381104 | 1.205616585 | 1.336682601 | 1.754853698 | 1.370584768 | 1.626827694 | 1.078833528 | 1.691454455 | 1.666941754 | 0.914874188 | 0.995550043 | 1.726654799 | 1.429442232 | 3.036934413 | 1.298384504 |
| ENSMUSG00000025038 | Efhc2 | 2539 | 0.38504098 | 0.131204782 | 0.190521979 | 0.448612095 | 0.284355595 | 0 | 0 | 0.376057527 | 0.227101702 | 0.241417543 | 0 | 0.021983325 | 0.097148321 | 0.018292275 | 0.584189056 | 1.085830537 | 0.362280869 | 0.389981852 |
| ENSMUSG00000025040 | Fundc1 | 2816 | 33.91659465 | 33.45485258 | 35.95376386 | 36.3711746 | 36.21858887 | 36.35509502 | 36.0053519 | 31.15339074 | 40.36746573 | 37.69042366 | 32.82831841 | 33.59643043 | 33.2324741 | 26.07532037 | 30.79874828 | 33.46348893 | 32.41947416 | 30.47379492 |
| ENSMUSG00000025041 | Nt5c2 | 5677 | 22.74629642 | 24.14113913 | 23.0918209 | 23.65129429 | 23.98541696 | 23.34922711 | 18.25167639 | 19.77232177 | 19.21843936 | 21.63600176 | 17.32757434 | 21.71865398 | 16.840806 | 21.27085207 | 23.34053263 | 24.88855757 | 24.74972775 | 22.92611353 |
| ENSMUSG00000025043 | Dusp21 | 820 | 0 | 0 | 0 | 0 | 0 | 0 | 0 | 0 | 0 | 0 | 0 | 0 | 0 | 0 | 0 | 0 | 0 | 0 |
| ENSMUSG00000025044 | Msr1 | 4002 | 0 | 0 | 0 | 0 | 0 | 0 | 0 | 0 | 0.349910429 | 0 | 0 | 0 | 0 | 0 | 0 | 0 | 0 | 0 |
| ENSMUSG00000025047 | Pdcd11 | 6005 | 2.682674723 | 2.912451202 | 2.142774201 | 2.154760465 | 2.556883206 | 2.318787366 | 2.410937511 | 2.111553305 | 1.844991297 | 2.614685127 | 2.084436157 | 2.491023742 | 2.867083952 | 3.279316079 | 2.826817823 | 2.98418056 | 3.216728333 | 3.517651454 |
| ENSMUSG00000025049 | Taf5 | 3247 | 1.191244539 | 1.446602428 | 1.296118614 | 0.701586762 | 1.571291626 | 1.663457949 | 2.710242772 | 1.482058116 | 1.496768974 | 1.321439481 | 2.839718813 | 1.478332917 | 2.749946728 | 2.503146071 | 1.636895815 | 2.051914286 | 2.053826813 | 0.965666487 |
| ENSMUSG00000025050 | Pcgf6 | 4075 | 7.24934904 | 5.918658997 | 6.422105502 | 7.323308967 | 6.20104538 | 6.55433703 | 8.603360757 | 7.366681789 | 9.844335301 | 9.626842972 | 8.851556724 | 9.218145011 | 6.077207969 | 9.835888937 | 8.371751725 | 8.83267999 | 6.950462187 | 5.38616775 |
| ENSMUSG00000025051 | 4930542N07R | 960 | 0 | 0 | 0 | 0 | 0 | 0 | 0 | 0 | 0 | 0 | 0 | 0 | 0 | 0 | 0 | 0 | 0 | 0 |
| ENSMUSG00000025056 | Nr0b1 | 1806 | 0 | 0 | 0 | 0 | 0 | 0 | 0.130987483 | 0.105737548 | 0 | 0 | 0 | 0 | 0 | 0 | 0 | 0 | 0 | 0 |
| ENSMUSG00000025058 | 5430427O19R | 3752 | 0.06797203 | 0 | 0.025785464 | 0.012143135 | 0 | 0 | 0.226979803 | 0.152688176 | 0.537883601 | 0 | 0.063665989 | 0.357029825 | 0.433889464 | 0.210434298 | 0.02196245 | 0.051026929 | 0.091934081 | 0.27856424 |
| ENSMUSG00000025059 | Gyk | 7721 | 1.15607913 | 1.432441508 | 0.789413913 | 0.867436021 | 1.502369331 | 1.116717477 | 3.995320122 | 4.239203324 | 2.864545909 | 2.503793334 | 1.392224538 | 2.97837754 | 2.875192692 | 6.255906085 | 2.833574171 | 1.215023807 | 1.449459746 | 1.802526098 |
| ENSMUSG00000025060 | Slk | 8590 | 8.817721034 | 9.737913549 | 8.903207391 | 8.136279629 | 9.788879004 | 9.633651348 | 8.988858208 | 8.816709426 | 7.911263214 | 9.292915089 | 9.371455767 | 8.95389791 | 10.38324871 | 9.386139198 | 8.403390678 | 8.808176976 | 9.588266827 | 8.600376057 |
| ENSMUSG00000025064 | Col17a1 | 5711 | 0.394462323 | 0.250823753 | 0.127053573 | 0.207422019 | 0.320261443 | 0.24294809 | 0.546775838 | 0.401250945 | 0.014423574 | 0.454086619 | 0.326251686 | 0.332294262 | 0.889719398 | 0.398487179 | 0.62765459 | 0.864907784 | 0.26172747 | 0.057792793 |
| ENSMUSG00000025066 | Sfr1 | 1619 | 99.08247951 | 83.12791393 | 87.60419457 | 87.12599955 | 93.20171754 | 102.2579882 | 116.3091181 | 96.38922559 | 92.39618607 | 106.0089931 | 90.29732733 | 112.320833 | 115.3921993 | 110.5593038 | 106.6048904 | 136.2994259 | 130.7686622 | 130.0987459 |
| ENSMUSG00000025068 | Gsto1 | 2001 | 3.250020956 | 2.980014026 | 3.191057475 | 2.459066861 | 5.43618928 | 6.067176275 | 8.582959746 | 6.031383888 | 8.603679954 | 5.678820332 | 6.82840486 | 8.172908115 | 11.34067064 | 8.68070399 | 10.15110797 | 5.262327354 | 12.23913229 | 12.67326247 |
| ENSMUSG00000025069 | Gsto2 | 1777 | 1.387338328 | 1.499736375 | 1.633321228 | 1.153768709 | 1.16470046 | 1.310623792 | 2.742378126 | 2.794041819 | 3.198502631 | 2.81259119 | 2.634747288 | 2.638444377 | 2.331953321 | 3.580664025 | 2.017183659 | 3.102896717 | 2.092093866 | 2.259800107 |
| ENSMUSG00000025075 | Habp2 | 3033 | 0.224227767 | 0.01098348 | 0.622013743 | 0.270391955 | 1.666288159 | 2.042232673 | 0 | 0 | 0.230850896 | 0.233188443 | 0.299282628 | 0.496875335 | 1.008433522 | 0.183755044 | 1.209013674 | 0.694356547 | 4.460660339 | 5.803796291 |
| ENSMUSG00000025076 | Casp7 | 2343 | 14.42237087 | 15.54033008 | 14.84445938 | 17.57882376 | 18.96106084 | 17.82883244 | 7.814770271 | 14.57277326 | 15.15270021 | 12.71841419 | 13.94710696 | 14.24573889 | 12.12769285 | 10.42662824 | 8.423194738 | 8.432758608 | 10.14182846 | 12.44337455 |
| ENSMUSG00000025077 | Dclre1a | 4083 | 4.393138448 | 4.193687884 | 4.798256134 | 3.927868628 | 5.516958187 | 4.247723917 | 2.723115246 | 4.461860384 | 5.406801971 | 5.600806045 | 4.680378004 | 6.479702196 | 4.748332963 | 3.378372264 | 4.076764286 | 4.20136961 | 5.247220174 | 6.264813771 |
| ENSMUSG00000025078 | Nhlrc2 | 4369 | 17.95938543 | 14.76930101 | 17.44945842 | 16.58092481 | 18.79447511 | 20.49480192 | 12.9300386 | 10.83968391 | 14.62126023 | 13.51171446 | 16.40248059 | 18.33267929 | 13.97869389 | 12.52257609 | 16.61642057 | 14.73255002 | 15.08840253 | 16.99754957 |
| ENSMUSG00000025081 | Tdrd1 | 5416 | 0.102024979 | 0.32599398 | 0.464443054 | 0.109359966 | 0.40880142 | 0.393420891 | 0.139771577 | 0.162190778 | 0.448671424 | 0.504937369 | 0.26463234 | 0.19580827 | 0.300582214 | 0.600274394 | 0.996566437 | 0.353495268 | 0.459972175 | 0.101567775 |
| ENSMUSG00000025082 | Vwa2 | 4201 | 13.79065816 | 18.3098007 | 13.27652476 | 18.11162677 | 14.80277205 | 12.23189353 | 24.76567031 | 26.69195274 | 19.96090124 | 30.67433278 | 20.49285293 | 17.69732506 | 19.91595613 | 22.74113015 | 16.2217028 | 15.7318706 | 14.26858566 | 14.24658608 |

| ENSMUSG00000025083 | Afap1l2 | 6613 | 9.030662682 | 6.332119769 | 8.273168431 | 8.667139539 | 8.464755365 | 9.67376063 | 4.299851812 | 4.221782271 | 3.824061799 | 4.798491349 | 7.11603419 | 5.114818014 | 7.22858428 | 5.716843662 | 6.211696843 | 5.738090418 | 7.209725291 | 4.691537304 |
| --- | --- | --- | --- | --- | --- | --- | --- | --- | --- | --- | --- | --- | --- | --- | --- | --- | --- | --- | --- | --- |
| ENSMUSG00000025085 | Ablim1 | 15884 | 10.50052321 | 13.18338284 | 11.57870577 | 11.69430734 | 10.91483185 | 14.24438312 | 7.705735356 | 7.838531342 | 7.861843099 | 8.926099515 | 8.544992259 | 8.408894521 | 4.928843661 | 6.035041323 | 6.409534412 | 5.83857033 | 7.229009413 | 7.109902802 |
| ENSMUSG00000025086 | Trub1 | 5700 | 3.989520908 | 3.494931709 | 4.387564069 | 2.941484968 | 4.21365437 | 3.616480205 | 4.523756143 | 4.683594611 | 3.995814599 | 4.375916144 | 4.676916988 | 4.308577463 | 3.332068097 | 4.505891285 | 3.917761981 | 4.910602458 | 4.921903392 | 4.719202314 |
| ENSMUSG00000025089 | Gfra1 | 8272 | 0.688550966 | 0.644349985 | 0.654960759 | 0.947352469 | 1.024083482 | 0.617013003 | 0.08007465 | 0.240087636 | 0.049790275 | 0.108300697 | 0.086632541 | 0.337377071 | 0.131201908 | 0.078604597 | 0.024904229 | 0.050918361 | 0.05096582 | 0 |
| ENSMUSG00000025090 | 1700011F14R | 1081 | 0 | 0 | 0 | 0 | 0 | 0 | 0 | 0 | 0 | 0 | 0 | 0 | 0 | 0 | 0 | 0 | 0 | 0 |
| ENSMUSG00000025091 | Pnliprp2 | 1555 | 0 | 0 | 0 | 0 | 0 | 0 | 0 | 0 | 0 | 0 | 0 | 0 | 0 | 0 | 0 | 0 | 0 | 0 |
| ENSMUSG00000025092 | Hspa12a | 5721 | 0.20060125 | 0.163041608 | 0.25366298 | 0.119457379 | 0.117784816 | 0.008661551 | 0.554090105 | 0.2536814 | 0.194377893 | 0.206042227 | 0.425891082 | 0.156100437 | 0.275934514 | 0.105536294 | 0.108027152 | 0.06692992 | 0.408653052 | 0.086537661 |
| ENSMUSG00000025094 | Slc18a2 | 3842 | 0.962506589 | 0.893083838 | 1.498295188 | 1.340030716 | 1.453228304 | 1.302661658 | 1.095999069 | 1.510995618 | 1.972493207 | 0.908160344 | 1.330536324 | 1.539942799 | 0.667688625 | 0.761576654 | 0.739955064 | 0.996632155 | 0.638439095 | 0.916341184 |
| ENSMUSG00000025102 | 3110040N11R | 1216 | 18.84069891 | 17.97142978 | 18.17985475 | 17.49753923 | 15.91203398 | 21.88307396 | 20.81602239 | 15.86115396 | 21.5416316 | 18.88354645 | 19.2121338 | 19.92105063 | 24.30083754 | 23.56579105 | 16.56871779 | 19.90103937 | 18.84796509 | 23.2069669 |
| ENSMUSG00000025103 | Btbd1 | 2992 | 31.50951886 | 35.36154814 | 34.59871489 | 34.35351471 | 34.92460103 | 38.62198352 | 28.87464964 | 30.4441443 | 31.06883893 | 33.21981366 | 31.32836515 | 30.18373732 | 29.66180454 | 29.43114629 | 30.62575545 | 28.78194387 | 30.40996953 | 27.61486574 |
| ENSMUSG00000025104 | Hdgfrp3 | 9569 | 1.896719633 | 1.841626193 | 1.50140438 | 1.556950726 | 2.449602553 | 1.667465797 | 2.126079208 | 2.454627179 | 2.061693077 | 2.202567077 | 2.341567094 | 2.444013233 | 2.381789715 | 2.188973081 | 1.382140186 | 2.933118949 | 2.050691192 | 1.109494994 |
| ENSMUSG00000025105 | Bnc1 | 5282 | 0 | 0 | 0 | 0 | 0 | 0 | 0 | 0 | 0 | 0 | 0.009044862 | 0.010567146 | 0 | 0 | 0.00780037 | 0 | 0.00725602 | 0 |
| ENSMUSG00000025127 | Gcgr | 2510 | 17.74718107 | 20.51861931 | 17.67272005 | 17.53464894 | 18.96512849 | 19.76186706 | 26.74768582 | 27.29767726 | 28.43674574 | 27.14452837 | 25.14371321 | 32.02173471 | 26.33656141 | 28.31053932 | 22.01246472 | 26.72714907 | 30.30984038 | 27.94287294 |
| ENSMUSG00000025128 | Bhlhe22 | 3184 | 0 | 0 | 0 | 0 | 0 | 0 | 0 | 0 | 0 | 0 | 0 | 0 | 0 | 0 | 0 | 0 | 0 | 0 |
| ENSMUSG00000025129 | Ppp1r27 | 768 | 0.055345281 | 0.477137808 | 0.314931838 | 0.296621382 | 0.250687102 | 0.322608943 | 0.061605051 | 0 | 0.160884827 | 0.491153159 | 0.248827909 | 0.654089796 | 0.192702802 | 0.241896288 | 0.375535015 | 0.049857562 | 0.099808065 | 0.501385087 |
| ENSMUSG00000025130 | P4hb | 3386 | 198.428623 | 212.7263434 | 201.151597 | 212.0082146 | 215.8686925 | 203.2305999 | 225.6085393 | 233.5647007 | 209.3262699 | 241.1982667 | 230.2538533 | 234.1587381 | 237.6411408 | 231.4523126 | 250.7867888 | 237.6935254 | 241.9899296 | 249.0193703 |
| ENSMUSG00000025132 | Arhgdia | 3572 | 150.0056688 | 163.7386573 | 147.7749057 | 161.7085443 | 150.8636667 | 161.6429036 | 162.1240734 | 165.5895118 | 140.9358293 | 176.5907358 | 154.9074941 | 150.8522975 | 149.9292536 | 143.2069369 | 148.5885899 | 159.454867 | 149.4425149 | 144.545206 |
| ENSMUSG00000025133 | Ints4 | 3220 | 26.80994177 | 25.9157764 | 26.71060154 | 29.60052943 | 25.96433274 | 25.74587679 | 20.01238158 | 22.87985846 | 20.40139523 | 21.81818256 | 28.54627954 | 23.48764683 | 23.40968 | 21.20273555 | 20.33207215 | 25.38833752 | 23.62661471 | 24.73701445 |
| ENSMUSG00000025134 | Alyref | 4191 | 26.77491403 | 26.56446969 | 26.68566027 | 26.57999365 | 25.66806237 | 25.39710611 | 26.94711634 | 28.06790728 | 28.34214065 | 26.03357846 | 24.48594865 | 27.00886751 | 26.94363127 | 21.98641598 | 26.29781962 | 27.53707724 | 21.24361434 | 25.59478838 |
| ENSMUSG00000025135 | Anapc11 | 5217 | 19.70050137 | 17.10023591 | 19.42544491 | 15.8507371 | 18.45195464 | 17.5528947 | 19.90633127 | 18.16280436 | 19.83142181 | 21.16675235 | 20.70522927 | 18.39124477 | 23.55487942 | 21.33027284 | 17.98273774 | 19.20769016 | 18.43217921 | 18.59997398 |
| ENSMUSG00000025137 | Pcyt2 | 4396 | 21.01086158 | 21.57457003 | 18.28862772 | 22.12757726 | 20.09146495 | 19.4896898 | 22.69846221 | 25.48187358 | 22.32653937 | 27.24358195 | 29.04969609 | 23.90830133 | 29.51386316 | 31.71636723 | 28.77360705 | 25.10316891 | 28.97140232 | 30.75804921 |
| ENSMUSG00000025138 | Sirt7 | 4358 | 23.91525737 | 21.23525015 | 20.09088801 | 27.19235345 | 22.77375553 | 19.9097835 | 26.38132395 | 23.0924691 | 22.81419207 | 26.9726258 | 27.79016057 | 25.87141772 | 28.14115336 | 31.81174871 | 26.41513244 | 28.85414289 | 29.46147192 | 26.94916082 |
| ENSMUSG00000025139 | Tollip | 4324 | 44.17628609 | 46.61802103 | 47.22124691 | 48.38490185 | 42.08760485 | 50.81332578 | 42.18093831 | 46.07113112 | 37.10024951 | 44.0211353 | 47.46559242 | 36.29825236 | 41.08331039 | 42.73844212 | 47.30937211 | 38.3171735 | 53.16399834 | 41.3331609 |
| ENSMUSG00000025140 | Pycr1 | 3899 | 1.362694794 | 0.828763975 | 0.694772429 | 1.098419638 | 1.098676893 | 1.156526996 | 0.194153081 | 1.126475065 | 0.507040974 | 0.95534895 | 0.673922215 | 0.68713819 | 0.683232504 | 1.155444082 | 0.348717965 | 0.274977432 | 0.933828727 | 0.366821435 |
| ENSMUSG00000025141 | Myadml2 | 1398 | 0.060808549 | 0.047657932 | 0.138407812 | 0.065180321 | 0.17214565 | 0 | 0 | 0.027319315 | 0.088383081 | 0.370999811 | 0.06834758 | 0 | 0.247012461 | 0.033221808 | 0.117887141 | 0.136947809 | 0 | 0.196742156 |
| ENSMUSG00000025142 | Aspscr1 | 4641 | 33.94186218 | 31.97061643 | 32.39494341 | 32.98537146 | 31.26863819 | 32.67213203 | 33.39718513 | 34.40690249 | 27.59966904 | 33.18125336 | 34.6705581 | 30.71605306 | 34.31230962 | 36.35668379 | 38.71579083 | 31.4426858 | 32.5620747 | 39.35148337 |
| ENSMUSG00000025144 | Stra13 | 1347 | 56.35801365 | 44.68923523 | 52.71888832 | 52.29203752 | 48.09619098 | 45.24859859 | 75.09614517 | 63.31375989 | 59.37952013 | 68.9933453 | 59.23101756 | 56.18859574 | 72.58786937 | 69.8902488 | 53.00838631 | 61.54355236 | 61.25947827 | 57.25520982 |
| ENSMUSG00000025145 | Lrrc45 | 2981 | 21.75877176 | 22.89772568 | 20.49505832 | 26.70082001 | 19.9244528 | 21.70944987 | 24.82288824 | 22.06216603 | 23.86082278 | 25.65529711 | 21.63575778 | 29.11551671 | 23.30068417 | 23.10519342 | 22.15568403 | 24.28968084 | 21.13667632 | 30.46629829 |
| ENSMUSG00000025147 | Mob2 | 3221 | 19.3193349 | 16.49613979 | 17.94671493 | 18.31777473 | 19.96406396 | 17.03038686 | 20.81405341 | 22.24431758 | 20.45899572 | 21.22586766 | 20.7504399 | 19.58140289 | 20.12484921 | 20.77799749 | 22.47473881 | 23.10988537 | 23.46459407 | 21.34783713 |
| ENSMUSG00000025150 | Cbr2 | 1640 | 12.46645712 | 12.06576778 | 19.88034113 | 11.64029121 | 21.33652958 | 15.86291776 | 44.8604974 | 62.66814321 | 90.56010722 | 42.26313036 | 61.35003814 | 95.15889792 | 20.00349085 | 4.70104787 | 10.22502027 | 6.654160433 | 9.768531772 | 13.48393957 |
| ENSMUSG00000025151 | Maged1 | 4425 | 59.85305126 | 59.83522785 | 63.5360358 | 67.9348634 | 65.65520706 | 62.76679587 | 65.35030364 | 65.55283531 | 55.36212333 | 54.97185946 | 56.21790029 | 60.53318977 | 57.80474375 | 65.59899349 | 64.49789326 | 53.37321276 | 54.12452641 | 66.80654001 |
| ENSMUSG00000025153 | Fasn | 10251 | 17.87116464 | 19.50807765 | 16.49258498 | 18.9026555 | 15.98293514 | 19.50011972 | 14.65857656 | 17.50345396 | 15.94264897 | 18.33408479 | 16.88039212 | 14.39086881 | 15.61142714 | 18.55316924 | 18.64540218 | 16.88731207 | 18.69393079 | 15.35811759 |
| ENSMUSG00000025154 | Arhgap19 | 8733 | 0.170351673 | 0.125881771 | 0.310193255 | 0.50084281 | 0.281086466 | 0.272361298 | 0.406326683 | 0.28426728 | 0.033013353 | 0.356343343 | 0.087529982 | 0.415437773 | 0.847336264 | 0.329730152 | 0.712405238 | 0.455997157 | 0.456422178 | 0.466125489 |
| ENSMUSG00000025155 | Dus1l | 4099 | 12.72355477 | 13.19837527 | 12.37956412 | 15.89468002 | 11.14349733 | 13.35832413 | 15.3976857 | 12.49475765 | 12.70059915 | 14.80433157 | 16.51551992 | 14.16157333 | 11.86661879 | 14.50315488 | 12.94647572 | 12.06915461 | 13.31462474 | 12.46729938 |
| ENSMUSG00000025156 | Gps1 | 2935 | 48.86284972 | 51.6434987 | 48.76908904 | 49.20903255 | 52.13335105 | 49.50208342 | 46.55503126 | 46.57260927 | 49.53608199 | 46.04221994 | 50.94910393 | 48.66517225 | 44.17181554 | 47.94738834 | 46.52196123 | 44.04398316 | 44.93382884 | 48.0369133 |
| ENSMUSG00000025157 | Zdhhc16 | 1819 | 25.79753399 | 25.73096008 | 24.25324886 | 25.8738641 | 28.02182912 | 27.32346992 | 22.65494412 | 28.40809261 | 25.63119068 | 28.53926569 | 30.09901177 | 26.75715106 | 26.08977703 | 26.12001176 | 25.07428388 | 23.66060622 | 28.33912553 | 25.22132766 |
| ENSMUSG00000025158 | Rfng | 3348 | 13.74943418 | 14.16892514 | 11.86220682 | 14.9692798 | 12.93868916 | 12.62499455 | 17.35363492 | 17.79574306 | 19.1293405 | 16.71681146 | 15.96779526 | 17.37154134 | 13.67384087 | 12.56820286 | 16.79812539 | 12.90077812 | 12.84411742 | 16.4797294 |
| ENSMUSG00000025159 | Mms19 | 10357 | 4.575965171 | 5.73816774 | 4.86210728 | 5.08091206 | 5.809105396 | 5.569120584 | 6.345207206 | 6.235720041 | 4.644766864 | 6.03667399 | 6.084307254 | 5.593961356 | 6.482643566 | 6.031408471 | 6.118373367 | 5.30530285 | 6.209474385 | 6.909997861 |
| ENSMUSG00000025161 | Slc16a3 | 5309 | 5.156024367 | 5.521820854 | 8.063773661 | 5.852822045 | 6.935566322 | 5.945581332 | 11.15755774 | 10.75487693 | 12.65308103 | 12.29168832 | 14.11021565 | 12.02733434 | 8.799646951 | 4.715268997 | 10.1820383 | 7.695625937 | 6.988106122 | 6.247973508 |
| ENSMUSG00000025162 | Csnk1d | 5838 | 32.581477 | 39.86363121 | 33.67420818 | 43.78168186 | 37.90038594 | 42.83026612 | 38.51955515 | 36.91016348 | 31.93753971 | 40.50373019 | 38.81408497 | 38.13783454 | 32.61754037 | 31.20934478 | 37.19290845 | 35.28009202 | 42.98086088 | 38.88704752 |
| ENSMUSG00000025163 | Cd7 | 1129 | 0 | 0 | 0 | 0 | 0 | 0 | 0 | 0.101485569 | 0 | 0 | 0 | 0 | 0 | 0.082274734 | 0 | 0 | 0 | 0 |

| ENSMUSG00000025165 | Sectm1a |  | 2047 | 0.228410814 | 0.504494246 | 0.874362786 | 0.623209204 | 1.41080382 | 1.355619482 | 0.184905438 | 0.317181652 | 0.362167701 | 0.092136206 | 0 | 0.818011665 | 0 | 0 | 0.100638876 | 0 | 0.112338926 | 0 |
| --- | --- | --- | --- | --- | --- | --- | --- | --- | --- | --- | --- | --- | --- | --- | --- | --- | --- | --- | --- | --- | --- |
| ENSMUSG00000025169 | 1110031I02Ri | k | 1299 | 37.72784293 | 39.51899153 | 35.04194924 | 34.37245839 | 38.20170385 | 36.43022369 | 39.95535703 | 43.48465216 | 48.92285898 | 41.81494238 | 39.97796756 | 44.21425466 | 46.9773531 | 42.29665541 | 43.16806587 | 38.23165343 | 37.94273889 | 44.97280319 |
| ENSMUSG00000025170 | Rab40b |  | 3189 | 3.105583573 | 2.945825077 | 2.184317457 | 2.843100603 | 2.822411328 | 2.626030723 | 3.308475196 | 2.634784738 | 2.725103426 | 2.631804697 | 3.25091439 | 2.152814832 | 4.470656661 | 3.072970348 | 2.816537844 | 2.665573798 | 2.607966895 | 5.347388863 |
| ENSMUSG00000025171 | Ubtd1 |  | 1524 | 11.37933846 | 11.21359235 | 9.80801895 | 13.93139545 | 9.380040242 | 11.93297456 | 7.295590255 | 7.417946924 | 7.702202751 | 9.869471351 | 12.85284316 | 10.62109065 | 9.808117411 | 10.23964131 | 6.380293432 | 6.457143112 | 8.952862006 | 14.00494318 |
| ENSMUSG00000025172 | Ankrd2 |  | 1091 | 0.389598314 | 0.366411302 | 0.620741911 | 0.125282432 | 0.132351761 | 0.227097771 | 0 | 0.28005428 | 0.113253481 | 0 | 0 | 0.409280752 | 0 | 0.042570199 | 0.188824729 | 0.210580792 | 0.491813159 | 0.605049753 |
| ENSMUSG00000025173 | Wdr45l |  | 4783 | 29.51279314 | 28.34695368 | 27.82261802 | 30.47246097 | 30.26987798 | 29.90983522 | 29.36887806 | 24.74561051 | 30.1385752 | 27.83892661 | 26.42955052 | 24.24941394 | 30.83889455 | 27.01389319 | 30.93347004 | 32.19837403 | 29.88056892 | 24.16352357 |
| ENSMUSG00000025175 | Fn3k |  | 4031 | 0.400693795 | 0.322302871 | 0.036001139 | 0.180842647 | 0.28657062 | 0.233565353 | 0.598597526 | 0.435834907 | 0.470002413 | 0.304122621 | 0.213333975 | 0.609250596 | 0.477287218 | 0.241956297 | 0.265750547 | 0.322967167 | 0.361299748 | 0.327516388 |
| ENSMUSG00000025176 | Hoga1 |  | 2569 | 12.75651643 | 15.02905582 | 10.67644675 | 10.144382 | 17.21807624 | 15.99035272 | 6.851037976 | 12.59204548 | 8.000027779 | 6.295325503 | 12.58997543 | 14.3612896 | 10.21587388 | 10.59409701 | 7.810493468 | 6.304759413 | 13.7550976 | 13.3400831 |
| ENSMUSG00000025178 | Pi4k2a |  | 3762 | 15.47902476 | 17.55966753 | 17.30748853 | 15.63511649 | 17.18266175 | 19.7051806 | 20.24811618 | 20.27385104 | 19.49845417 | 19.60225941 | 19.82368692 | 21.11262304 | 15.97189666 | 13.58014251 | 16.83327789 | 20.5091903 | 20.73206119 | 20.92451667 |
| ENSMUSG00000025184 | D19Ertd386e |  | 6615 | 4.25373039 | 5.43380391 | 4.84832209 | 4.938362629 | 5.26795355 | 5.917862362 | 4.627559073 | 4.884470512 | 5.06192552 | 5.010875947 | 6.023328098 | 4.733573199 | 5.675221322 | 4.275804871 | 6.919868268 | 4.24293503 | 5.851785317 | 5.629805783 |
| ENSMUSG00000025185 | Loxl4 |  | 4492 | 0.066236917 | 0.489459265 | 0.775354894 | 0.162283328 | 0.600041791 | 0.728067769 | 0.547697975 | 0.416613472 | 0.623482429 | 0.619299086 | 0.659405037 | 0.832513222 | 0.164732582 | 0.175767918 | 0.669571144 | 0.323918763 | 1.245689971 | 0.624546848 |
| ENSMUSG00000025188 | Hps1 |  | 6435 | 4.40574241 | 6.574572752 | 5.961182529 | 6.067725718 | 6.245556529 | 6.121899486 | 5.433421869 | 6.866916796 | 6.861219098 | 6.719066805 | 7.030751465 | 7.702301224 | 7.827177551 | 7.448375786 | 8.425990296 | 6.932176787 | 8.076217343 | 8.103905699 |
| ENSMUSG00000025189 | Cnnm1 |  | 6447 | 0.006593016 | 0 | 0 | 0 | 0 | 0 | 0.080725837 | 0.02962029 | 0 | 0.007313588 | 0 | 0 | 0.068867265 | 0 | 0.006390811 | 0 | 0.053503439 | 0 |
| ENSMUSG00000025190 | Got1 |  | 3066 | 46.76123899 | 50.92548441 | 45.23382633 | 45.14496174 | 51.14605587 | 51.86390152 | 53.02229772 | 52.99102408 | 47.28523662 | 53.36364654 | 53.29104958 | 54.10441918 | 50.47430675 | 50.8370376 | 43.28447864 | 47.5447953 | 51.62674697 | 51.81549257 |
| ENSMUSG00000025192 | Entpd7 |  | 9045 | 0.822377646 | 0.81394689 | 0.984049705 | 0.957058974 | 0.425710768 | 0.914903981 | 1.124624209 | 0.806495175 | 0.910702394 | 0.938322453 | 0.596856861 | 0.796044276 | 1.385329684 | 1.653399241 | 1.024914326 | 1.367370502 | 1.453390806 | 1.423121169 |
| ENSMUSG00000025193 | Cutc |  | 1535 | 8.4179632 | 8.572373431 | 10.68314449 | 9.824563958 | 8.685695672 | 10.168802 | 11.34271391 | 11.86825795 | 14.65005707 | 12.16395993 | 11.42241678 | 11.63583845 | 16.22971871 | 16.73197415 | 12.61546011 | 11.52459975 | 12.10961173 | 15.69640961 |
| ENSMUSG00000025194 | Abcc2 |  | 5818 | 0 | 0 | 0 | 0 | 0 | 0 | 0 | 0 | 0 | 0 | 0 | 0 | 0 | 0 | 0 | 0 | 0.013175076 | 0 |
| ENSMUSG00000025195 | Dnmbp |  | 4884 | 5.735239763 | 6.909492597 | 6.309160286 | 7.155061631 | 6.563444136 | 7.599303327 | 7.032965787 | 8.156158007 | 7.640250467 | 8.061186982 | 7.913583417 | 8.639770867 | 7.131108435 | 6.124077043 | 6.276404091 | 7.557769356 | 7.407867378 | 6.600191757 |
| ENSMUSG00000025196 | Cpn1 |  | 3679 | 0.288836477 | 0.135823162 | 0.223525419 | 0.123840838 | 0.013082882 | 0.228974306 | 2.391997358 | 2.636822563 | 2.944292918 | 1.845529021 | 0.70123614 | 2.139170542 | 0.469316962 | 1.91886417 | 1.534279195 | 0 | 0.197934124 | 0.882179205 |
| ENSMUSG00000025197 | Cyp2c44 |  | 1955 | 0 | 0 | 0 | 0 | 0 | 0 | 0 | 0 | 0 | 0 | 0 | 0 | 0 | 0 | 0 | 0 | 0 | 0 |
| ENSMUSG00000025198 | Erlin1 |  | 4725 | 4.776772163 | 3.793087215 | 4.975563122 | 4.23307904 | 2.363302919 | 2.349166629 | 5.907826575 | 3.516125936 | 4.654730035 | 4.550417076 | 4.357885101 | 3.898236752 | 3.63333486 | 6.29083935 | 4.813388117 | 3.063248592 | 4.785717497 | 3.050240415 |
| ENSMUSG00000025199 | Chuk |  | 6271 | 19.82580767 | 22.43347987 | 20.69624971 | 18.72282112 | 23.64765696 | 23.23944976 | 23.05653752 | 23.03359367 | 25.66686391 | 24.69190073 | 30.87735715 | 24.77927039 | 24.55975853 | 27.52132495 | 30.9915068 | 30.72529683 | 29.75773236 | 32.11423054 |
| ENSMUSG00000025200 | Cwf19l1 |  | 3560 | 6.996638526 | 8.038139364 | 5.965162872 | 6.629387912 | 7.233308753 | 7.238039736 | 6.60516894 | 8.507464918 | 7.010963076 | 7.814301944 | 9.716030882 | 6.506601116 | 6.734638145 | 5.231482877 | 8.541221418 | 8.679921397 | 9.581574226 | 7.880518098 |
| ENSMUSG00000025201 | Gm13540 |  | 869 | 1.320644134 | 0.498351697 | 1.224646338 | 0.7864392 | 0.720040285 | 1.140454168 | 1.25223431 | 1.845892867 | 1.516649602 | 0.596844345 | 1.20949262 | 0.385378568 | 1.646289529 | 1.817144961 | 1.991329514 | 2.291267647 | 1.411325087 | 0.569714569 |
| ENSMUSG00000025202 | Scd3 |  | 1990 | 0.939812937 | 1.255511089 | 1.871739617 | 1.488174815 | 1.209344816 | 1.444250526 | 3.827809702 | 3.704087268 | 3.477052587 | 2.772177026 | 1.344420942 | 1.907268872 | 4.610922921 | 9.568882326 | 5.300300657 | 4.906585371 | 3.601516342 | 4.008201244 |
| ENSMUSG00000025203 | Scd2 |  | 5447 | 111.1985971 | 120.7202532 | 119.7926475 | 126.6123606 | 111.8336012 | 135.3944068 | 112.9094021 | 102.6714427 | 109.5332967 | 111.9948226 | 105.969717 | 116.211755 | 112.4028686 | 102.4037983 | 116.4944304 | 101.2764422 | 114.9648183 | 109.1295038 |
| ENSMUSG00000025204 | Ndufb8 |  | 3113 | 44.08905024 | 37.61478419 | 39.12770621 | 37.62847574 | 40.75674616 | 36.97751369 | 48.66533824 | 44.49850418 | 47.36515465 | 45.46945428 | 47.09969405 | 40.07324314 | 48.85650529 | 49.17433731 | 42.8692064 | 41.79617217 | 42.20447985 | 47.14561411 |
| ENSMUSG00000025207 | Sema4g |  | 4921 | 0.293675266 | 0.324937802 | 0.186770387 | 0.148135889 | 0.107590157 | 0.14097506 | 0.009614444 | 0.085372166 | 0.17576038 | 0.019163058 | 0.087375457 | 0.124765757 | 0.0400991 | 0.075503495 | 0.058608188 | 0.023343187 | 0.046729889 | 0 |
| ENSMUSG00000025208 | Mrpl43 |  | 1715 | 35.19379006 | 27.5632635 | 27.38816208 | 25.60982317 | 26.26908487 | 27.76686704 | 28.24966952 | 30.26441683 | 29.73114082 | 28.53786005 | 29.02711763 | 28.28210542 | 31.58393304 | 30.52040028 | 23.68789161 | 29.2035653 | 22.79464889 | 28.0337955 |
| ENSMUSG00000025209 | Peo1 |  | 3597 | 5.802068788 | 4.899227423 | 3.98069641 | 5.269222809 | 5.272165117 | 3.85731593 | 3.998625075 | 3.949839782 | 4.740399646 | 4.286427569 | 4.688507185 | 4.500011718 | 2.838951036 | 4.235101658 | 5.154489895 | 3.459674008 | 3.718620411 | 5.597256898 |
| ENSMUSG00000025212 | Sfxn3 |  | 3016 | 63.137662 | 67.66405504 | 63.96340818 | 70.54710777 | 68.16029376 | 67.75711979 | 71.58082027 | 64.74726575 | 62.13482981 | 70.08508047 | 76.00273567 | 65.32801357 | 78.02163309 | 62.92126693 | 72.17102778 | 72.62011749 | 74.28896939 | 77.18784471 |
| ENSMUSG00000025213 | Kazald1 |  | 1675 | 1.674830817 | 1.014303048 | 1.184068504 | 1.16962681 | 0.919535258 | 1.094597697 | 3.022362176 | 2.120533387 | 2.213006817 | 2.280123561 | 2.624057421 | 2.066012585 | 4.093812837 | 3.438248857 | 3.345320359 | 5.417835197 | 3.134747866 | 1.510697856 |
| ENSMUSG00000025215 | Tlx1 |  | 3581 | 0 | 0 | 0 | 0 | 0 | 0 | 0 | 0 | 0 | 0 | 0 | 0 | 0 | 0 | 0 | 0 | 0 | 0 |
| ENSMUSG00000025216 | Lbx1 |  | 2477 | 0 | 0 | 0 | 0 | 0 | 0 | 0 | 0 | 0 | 0 | 0.115724566 | 0 | 0 | 0 | 0 | 0 | 0 | 0 |
| ENSMUSG00000025217 | Btrc |  | 6308 | 12.7353809 | 14.25356714 | 12.82956821 | 13.3404009 | 13.56667094 | 14.77626694 | 11.34064213 | 12.43614371 | 12.11828999 | 13.51434234 | 13.88260921 | 13.35222493 | 12.73964699 | 11.64783549 | 12.21415814 | 11.41191216 | 15.43866843 | 12.67965142 |
| ENSMUSG00000025218 | Poll |  | 2315 | 5.232818647 | 4.719926265 | 5.725420007 | 6.081366177 | 5.322579896 | 6.721191509 | 6.13123269 | 5.856718295 | 7.240998667 | 7.984049968 | 5.406928346 | 4.653314419 | 5.220872455 | 5.276369323 | 5.054532127 | 5.921398466 | 5.397137274 | 2.399965347 |
| ENSMUSG00000025219 | Fgf8 |  | 1811 | 0 | 0.036789502 | 0.02671095 | 0 | 0 | 0 | 0 | 0.105445617 | 0 | 0 | 0 | 0 | 0 | 0 | 0.136504327 | 0.105716752 | 0 | 0 |
| ENSMUSG00000025220 | Mgea5 |  | 5059 | 30.73412412 | 38.37666486 | 38.54392189 | 39.69817818 | 38.3418961 | 42.83338682 | 24.2502029 | 27.84972771 | 24.44813339 | 31.45554922 | 33.98736422 | 28.31053374 | 25.27541601 | 34.18813628 | 29.09111636 | 23.16055716 | 34.63685105 | 31.32659349 |
| ENSMUSG00000025221 | Kcnip2 |  | 3422 | 0.01242115 | 0.204433308 | 0.381673092 | 0 | 0.154719801 | 0 | 0.304172687 | 0 | 0.30089506 | 0.013778698 | 0.181494582 | 0.081554153 | 0.302738242 | 0.162866467 | 0.180602963 | 0.123084945 | 0.167999548 | 0.0482254 |
| ENSMUSG00000025223 | Ldb1 |  | 4110 | 16.5056596 | 17.51561178 | 15.24178146 | 14.90987947 | 13.36217151 | 14.9261032 | 16.1277526 | 16.65225915 | 15.7931749 | 17.85072853 | 14.79745064 | 15.67184297 | 18.94057554 | 21.35750004 | 20.77119801 | 23.04889604 | 18.5570148 | 20.0495479 |
| ENSMUSG00000025224 | Gbf1 |  | 8684 | 7.170668227 | 8.032842065 | 7.793018075 | 7.812113659 | 8.746220123 | 8.519372551 | 7.959906024 | 7.890047203 | 6.426500329 | 7.617737986 | 7.608563747 | 8.36849294 | 8.248496532 | 7.738898486 | 7.909142523 | 8.708423494 | 8.248715904 | 8.355252391 |

| ENSMUSG00000025225 | Nfkb2 |  | 3266 | 67.85731409 | 80.84445792 | 80.60280226 | 90.29903245 | 80.90761204 | 96.67789907 | 58.85836328 | 77.83485316 | 63.39394205 | 70.00421313 | 66.89371865 | 70.7009786 | 39.22687972 | 39.63247751 | 50.72610418 | 51.69114759 | 51.92708628 | 44.29698429 |
| --- | --- | --- | --- | --- | --- | --- | --- | --- | --- | --- | --- | --- | --- | --- | --- | --- | --- | --- | --- | --- | --- |
| ENSMUSG00000025226 | Fbxl15 |  | 1344 | 7.87484288 | 9.022242184 | 7.270426437 | 9.898679275 | 7.413175743 | 8.811832831 | 9.610387907 | 8.951344314 | 10.81758933 | 9.963392653 | 10.13085057 | 10.09167114 | 8.111870326 | 8.984719287 | 8.522345638 | 10.51282301 | 9.068275607 | 8.677031715 |
| ENSMUSG00000025227 | Tmem180 |  | 2794 | 6.861071721 | 7.726111472 | 7.583252074 | 9.947114189 | 9.405880574 | 10.69445181 | 9.702993925 | 7.258470178 | 9.817544559 | 9.112877509 | 10.94344074 | 8.82982207 | 11.33539402 | 9.209028065 | 10.23402999 | 10.52512043 | 10.93273394 | 11.005759 |
| ENSMUSG00000025228 | Actr1a |  | 2731 | 62.13133017 | 64.60091094 | 64.36814695 | 58.80727985 | 62.86582634 | 62.99783632 | 47.8324081 | 57.78506288 | 60.89752862 | 56.00764605 | 60.28286595 | 64.19516521 | 52.80014434 | 60.47424927 | 60.94993988 | 53.92371881 | 59.74186961 | 55.89537574 |
| ENSMUSG00000025229 | Pitx3 |  | 1576 | 0 | 0 | 0 | 0 | 0 | 0 | 0 | 0 | 0 | 0 | 0 | 0 | 0 | 0 | 0 | 0 | 0 | 0 |
| ENSMUSG00000025231 | Sufu |  | 7297 | 4.25809013 | 5.135947101 | 4.885746454 | 4.058473183 | 4.267692834 | 5.602440072 | 4.668374514 | 4.516930694 | 4.594466744 | 5.531177469 | 5.250858805 | 4.543580043 | 3.887330743 | 5.085490725 | 5.928687637 | 4.722700653 | 5.436167918 | 5.616251132 |
| ENSMUSG00000025232 | Hexa |  | 2144 | 192.5222782 | 144.2678278 | 170.2098475 | 175.6777768 | 158.3594168 | 158.5271453 | 231.7306163 | 167.7686781 | 181.4780851 | 184.5123136 | 206.5420197 | 195.901521 | 175.1237045 | 126.5081485 | 141.9570398 | 134.356455 | 129.0294828 | 145.5021662 |
| ENSMUSG00000025234 | Arih1 |  | 7687 | 17.05847119 | 18.22740121 | 17.23625597 | 16.64901438 | 14.98369889 | 18.13989753 | 18.40313646 | 16.6442758 | 15.24335077 | 16.4693168 | 16.25852626 | 14.34056636 | 13.94539563 | 12.74236767 | 13.36759209 | 13.80793076 | 15.94477657 | 15.71484303 |
| ENSMUSG00000025235 | Bbs4 |  | 2526 | 10.66836168 | 9.864625842 | 8.904866039 | 10.67780612 | 11.68047079 | 10.43628435 | 12.71785787 | 11.38514609 | 10.32108649 | 10.78903661 | 12.63407453 | 13.41255233 | 11.83497303 | 11.01346332 | 11.56449055 | 15.56787561 | 11.74368717 | 8.79797273 |
| ENSMUSG00000025236 | Adpgk |  | 2803 | 10.11450318 | 11.7658813 | 13.15042102 | 12.02824574 | 10.68071942 | 12.71081536 | 11.83238956 | 13.06689758 | 11.9166123 | 12.81799354 | 15.59546451 | 11.52952859 | 11.0878016 | 16.05576906 | 13.69955408 | 11.80274163 | 12.27863525 | 17.6625744 |
| ENSMUSG00000025237 | Parp6 |  | 2679 | 13.54961565 | 14.61091852 | 12.74793297 | 15.23803499 | 13.42088353 | 14.33496399 | 12.96286164 | 9.922326269 | 17.03421406 | 13.90408943 | 12.59019063 | 15.31336767 | 11.01175309 | 13.07160952 | 14.13371775 | 15.02180977 | 16.6524112 | 12.34060214 |
| ENSMUSG00000025239 | Limd1 |  | 4841 | 18.2277929 | 21.45622891 | 20.4945488 | 22.67228998 | 21.36655731 | 22.57049732 | 22.19522699 | 19.89697147 | 18.72578418 | 21.95366353 | 23.73451253 | 21.24938363 | 24.93600528 | 20.77080132 | 22.83490487 | 24.92330178 | 25.43738731 | 19.26057341 |
| ENSMUSG00000025240 | Sacm1l |  | 3413 | 38.5448344 | 39.32541776 | 51.08063387 | 44.50645233 | 41.46142853 | 46.66348834 | 44.52631841 | 43.4965919 | 45.48256018 | 44.02850609 | 45.80124938 | 44.05725022 | 36.48220311 | 37.54445857 | 43.83324039 | 44.69668324 | 54.49678256 | 47.67563366 |
| ENSMUSG00000025241 | Fyco1 |  | 8041 | 12.34822674 | 16.60466112 | 14.23954065 | 13.55895773 | 16.01803603 | 15.5480098 | 15.71009237 | 14.59110312 | 13.59907964 | 16.77042797 | 12.85723295 | 12.19601034 | 13.26397278 | 17.48367772 | 14.83378986 | 14.33338245 | 17.12552976 | 12.80649767 |
| ENSMUSG00000025243 | Slc6a20b |  | 5737 | 0.007408955 | 0.081293449 | 0 | 0.01588323 | 0.008389737 | 0.043186974 | 0 | 0.046600456 | 0 | 0 | 0 | 0.009729068 | 0 | 0 | 0 | 0.006674326 | 0 | 0.047942397 |
| ENSMUSG00000025245 | Lztfl1 |  | 12326 | 1.555235631 | 1.194572387 | 1.263692745 | 1.463749274 | 1.230046727 | 1.382941778 | 2.414382204 | 1.416025304 | 1.747569994 | 1.805543724 | 1.496116662 | 1.666409527 | 2.181234376 | 2.196730728 | 2.012277839 | 1.863894568 | 1.604443388 | 1.615552218 |
| ENSMUSG00000025246 | Tbl1x |  | 5599 | 55.99538817 | 65.40608076 | 62.15380914 | 65.12324305 | 62.66864146 | 67.34180207 | 64.41588703 | 67.33295305 | 65.14516585 | 68.19546258 | 67.33205882 | 67.70851586 | 63.83456701 | 58.91157504 | 59.93689458 | 61.49475652 | 81.34155851 | 64.33285066 |
| ENSMUSG00000025255 | Zfhx4 |  | 16081 | 0.0343615 | 0.041431371 | 0.048129873 | 0.01416611 | 0.053875669 | 0.077036151 | 0 | 0.011875009 | 0 | 0.005864151 | 0.00594179 | 0.038179982 | 0 | 0 | 0 | 0.004762217 | 0.01191664 | 0.023945261 |
| ENSMUSG00000025257 | Ribc1 |  | 1539 | 4.584703847 | 3.138641756 | 3.017452186 | 3.167661952 | 4.284648176 | 3.702770865 | 2.490140996 | 2.829066845 | 3.639614128 | 3.799017027 | 4.097657257 | 3.155271374 | 2.532307644 | 2.655672313 | 2.1417313 | 2.388497928 | 2.166593782 | 1.71568364 |
| ENSMUSG00000025260 | Hsd17b10 |  | 1213 | 86.65729622 | 75.74357977 | 77.72465842 | 74.59541142 | 87.65327237 | 79.45594955 | 86.47337938 | 82.24118309 | 85.46286906 | 76.34293586 | 75.97518129 | 77.6725791 | 88.41515924 | 90.70572385 | 80.26321234 | 88.32408863 | 74.18808337 | 90.0190246 |
| ENSMUSG00000025261 | Huwe1 |  | 22312 | 8.180227047 | 7.75339995 | 7.063506718 | 6.920328938 | 7.146874467 | 6.260718714 | 5.723239531 | 6.840123701 | 7.832304875 | 6.698983925 | 6.190274514 | 6.53917811 | 7.351587708 | 7.112739629 | 6.839842373 | 8.584152843 | 6.901894091 | 6.713418681 |
| ENSMUSG00000025262 | Fam120c |  | 5808 | 0.644017819 | 0.89476782 | 1.607453746 | 1.490461161 | 0.986174056 | 1.083539457 | 2.117991825 | 1.795200733 | 1.255167577 | 1.152789233 | 0.806120167 | 1.528011424 | 1.223105387 | 0.591746292 | 1.347847042 | 0.896612019 | 0.989832048 | 0.985011553 |
| ENSMUSG00000025264 | Tsr2 |  | 7119 | 4.215290669 | 4.024313672 | 4.029428782 | 3.865552856 | 3.779427635 | 3.661292016 | 5.057585147 | 4.114843748 | 4.686202805 | 4.430934187 | 3.174259778 | 4.202443482 | 4.989321596 | 5.127834343 | 3.958682993 | 5.30334863 | 4.382301683 | 5.38577713 |
| ENSMUSG00000025265 | Fgd1 |  | 4797 | 1.116458657 | 1.60418565 | 1.260515175 | 1.424673056 | 1.033476785 | 1.497841645 | 0.749585908 | 1.138526901 | 0.266162599 | 1.120529533 | 0.388414297 | 1.163553525 | 0 | 0.164592346 | 0.206136615 | 0.183590571 | 0.159792774 | 0.194945761 |
| ENSMUSG00000025266 | Gnl3l |  | 5553 | 12.66046482 | 13.59991558 | 12.68356928 | 14.14500998 | 12.99293239 | 13.8048044 | 10.01975696 | 13.24663552 | 10.88071648 | 13.6281071 | 13.16327867 | 11.84059977 | 11.95763743 | 10.32927209 | 12.37604812 | 13.14968275 | 12.46484643 | 10.34206869 |
| ENSMUSG00000025268 | Maged2 |  | 3178 | 74.23024763 | 71.64683197 | 64.14287505 | 71.3522082 | 76.09024056 | 69.74492678 | 60.39884783 | 61.62701055 | 56.01262465 | 62.28403155 | 62.34196124 | 66.88044151 | 60.97412557 | 62.19824919 | 68.97176757 | 89.64195062 | 71.32212709 | 67.81802396 |
| ENSMUSG00000025269 | Apex2 |  | 9756 | 0.68837821 | 0.491703236 | 0.89250056 | 0.793909137 | 0.730168584 | 0.624742336 | 1.023265196 | 0.559810736 | 0.641692332 | 0.734615303 | 0.901044727 | 0.743751141 | 0.576449218 | 0.499859489 | 0.819301132 | 0.592648803 | 0.577487253 | 0.665341799 |
| ENSMUSG00000025270 | Alas2 |  | 2418 | 0 | 0.316872029 | 0.020005596 | 0 | 0.119434054 | 0.040986546 | 0.606572807 | 0.726571758 | 1.039031208 | 0.311998036 | 0.158064379 | 0.277000807 | 0.040803902 | 0.307322332 | 0.204474223 | 0.285041742 | 0.095102474 | 0.227498374 |
| ENSMUSG00000025271 | Pfkfb1 |  | 4037 | 0.515916182 | 0.354831422 | 0.191720705 | 0.090286935 | 0.202685832 | 0.355964645 | 1.289173813 | 0.64332013 | 1.265079999 | 0.350389175 | 0.603547903 | 0.84338752 | 0.794296414 | 1.760204451 | 0.602153034 | 0.455275986 | 0.123418841 | 0.258898446 |
| ENSMUSG00000025272 | Tro |  | 6920 | 0 | 0 | 0 | 0 | 0 | 0 | 0 | 0.005519133 | 0 | 0.006813685 | 0 | 0 | 0 | 0.006711573 | 0 | 0 | 0 | 0 |
| ENSMUSG00000025277 | Abhd6 |  | 2162 | 25.42053312 | 19.614854 | 21.99407046 | 22.19046239 | 23.06414104 | 22.43854125 | 23.21866436 | 25.49104379 | 23.83179057 | 26.19241194 | 24.94816287 | 24.80982967 | 23.45661932 | 19.33380141 | 25.99395108 | 30.3739092 | 28.32813249 | 18.52295546 |
| ENSMUSG00000025278 | Flnb |  | 9054 | 23.22902706 | 23.85694788 | 23.60440215 | 25.08524476 | 23.39613387 | 25.77792966 | 17.82978358 | 20.80460886 | 18.04582594 | 18.23223019 | 17.68739351 | 19.79501796 | 13.02767994 | 12.98833988 | 14.85786172 | 14.45096151 | 16.67409802 | 13.93758458 |
| ENSMUSG00000025279 | Dnase1l3 |  | 5142 | 0.057863911 | 0.136050326 | 0 | 0.062023981 | 0.102965998 | 0.067458019 | 0.046006106 | 0.103985537 | 0.09611789 | 0.036678882 | 0 | 0.314790785 | 0 | 0 | 0.224356975 | 0.07446637 | 0.156525133 | 0.128375978 |
| ENSMUSG00000025280 | Polr3a |  | 4652 | 4.504525319 | 4.411165701 | 6.582210816 | 6.366010064 | 5.090478599 | 5.091617939 | 4.220714049 | 4.457969584 | 5.922996355 | 6.253650883 | 5.822958181 | 4.475331501 | 4.029692297 | 4.74224882 | 5.969442964 | 5.967474283 | 4.918486511 | 6.231685134 |
| ENSMUSG00000025283 | Sat1 |  | 2906 | 96.97498867 | 96.12134137 | 90.3549631 | 92.14117604 | 96.89323932 | 95.55867824 | 123.9312154 | 105.9688027 | 107.8417751 | 85.45862149 | 83.36779575 | 97.78304825 | 167.8914872 | 208.4230364 | 143.8509931 | 114.7927638 | 121.4017767 | 154.6350285 |
| ENSMUSG00000025287 | Acot9 |  | 2425 | 40.57710619 | 39.21992288 | 39.93559083 | 36.73065636 | 45.01575376 | 41.05214093 | 35.46987641 | 43.32672124 | 45.22873875 | 35.23178322 | 28.84228421 | 37.93163379 | 43.1679705 | 53.93259797 | 38.02436375 | 30.61669598 | 30.26592106 | 43.32676035 |
| ENSMUSG00000025288 | 4933436I01Ri | k | 1424 | 0 | 0 | 0 | 0 | 0 | 0 | 0 | 0 | 0 | 0 | 0 | 0 | 0 | 0 | 0 | 0 | 0 | 0 |
| ENSMUSG00000025289 | Prdx4 |  | 2097 | 11.37119874 | 14.10674777 | 14.32520856 | 12.81879645 | 14.32251806 | 12.50042731 | 17.41792472 | 14.73421724 | 16.38033102 | 16.52635522 | 18.47662915 | 14.50621655 | 17.94957219 | 15.72498905 | 16.46490407 | 17.32846277 | 17.52738137 | 11.38481274 |
| ENSMUSG00000025290 | Rps24 |  | 933 | 59.13367469 | 51.98668165 | 59.77993623 | 49.07700917 | 53.75505301 | 53.00496046 | 66.63329058 | 57.59561649 | 62.41986419 | 53.87207896 | 56.68475781 | 57.96932159 | 80.42212881 | 88.45781703 | 69.41998485 | 58.64660019 | 66.75266074 | 86.31654045 |
| ENSMUSG00000025314 | Ptprj |  | 8639 | 15.16390236 | 16.08381546 | 15.98079125 | 15.97458077 | 15.17111103 | 15.85985743 | 11.10662263 | 13.29373238 | 13.62077504 | 13.2735861 | 12.21056932 | 13.0768493 | 10.30151392 | 9.499560414 | 11.79435672 | 11.13392821 | 12.7103647 | 10.70382086 |
| ENSMUSG00000025316 | Banp |  | 6614 | 5.025559065 | 3.863167503 | 4.593071827 | 4.739340566 | 3.456707551 | 3.925859147 | 3.748388836 | 3.482010682 | 4.452427997 | 3.571590918 | 2.780973543 | 4.109801584 | 3.759190551 | 3.272292822 | 3.993074891 | 4.434624323 | 3.447860093 | 2.528389544 |

| ENSMUSG00000025317 | Car5a |  | 1331 | 0 | 0 | 0 | 0 | 0.036162227 | 0.037229702 | 0 | 0 | 0 | 0 | 0 | 0 | 0 | 0 | 0 | 0 | 0 | 0.041329156 |
| --- | --- | --- | --- | --- | --- | --- | --- | --- | --- | --- | --- | --- | --- | --- | --- | --- | --- | --- | --- | --- | --- |
| ENSMUSG00000025318 | Jph3 |  | 4450 | 0.066862075 | 0.022458131 | 0 | 0.061430622 | 0.010816163 | 0 | 0 | 0 | 0.046276984 | 0 | 0 | 0 | 0 | 0.104368736 | 0 | 0 | 0 | 0 |
| ENSMUSG00000025321 | Itgb8 |  | 11046 | 0.527178084 | 1.767278292 | 0.845201101 | 0.808434247 | 1.16778522 | 0.946553212 | 0.265560936 | 0.829818629 | 0.53319498 | 0.55491503 | 0.315731665 | 0.560885257 | 0.040194392 | 0.142956633 | 0.331969806 | 0.32238154 | 0.357379013 | 0.01992001 |
| ENSMUSG00000025323 | Sp4 |  | 5764 | 2.241771949 | 2.444717948 | 2.803046346 | 2.466177278 | 3.089661998 | 2.441529552 | 2.692324547 | 3.611183087 | 1.407658502 | 2.102312758 | 2.983862778 | 2.875997881 | 2.901374038 | 2.900741058 | 2.651939146 | 2.305142396 | 2.899074506 | 3.931948642 |
| ENSMUSG00000025324 | Atp10a |  | 5448 | 15.67417376 | 19.3102276 | 17.7937876 | 16.30764252 | 18.90644579 | 21.98402295 | 20.14783684 | 24.31891409 | 22.76295869 | 19.17011889 | 22.3967041 | 21.83244805 | 19.40497407 | 23.07711905 | 31.55155859 | 19.1453037 | 28.06936943 | 29.10999535 |
| ENSMUSG00000025326 | Ube3a |  | 5084 | 28.09990099 | 31.33404016 | 34.31057247 | 31.22239938 | 30.22919596 | 31.73558232 | 29.61230219 | 31.07076639 | 33.88733191 | 30.56819786 | 30.64400858 | 29.57659225 | 31.34187506 | 27.49738455 | 30.75529202 | 31.63268117 | 37.56489722 | 31.74601084 |
| ENSMUSG00000025328 | Padi3 |  | 4696 | 0.009051358 | 0 | 0 | 0 | 0 | 0 | 0 | 0 | 0 | 0 | 0 | 0 | 0 | 0 | 0 | 0 | 0 | 0 |
| ENSMUSG00000025329 | Padi1 |  | 3738 | 0 | 0.071295654 | 0 | 0.024377231 | 0.012876384 | 0 | 0.037971652 | 0.020434672 | 0 | 0.025227771 | 0 | 0.044795877 | 0.039592229 | 0.062124247 | 0 | 0.020487216 | 0 | 0.029432374 |
| ENSMUSG00000025330 | Padi4 |  | 4814 | 0.26488477 | 0.318320136 | 0.221067235 | 0.179821322 | 0.269954703 | 0.174988881 | 0.029484428 | 0.166605827 | 0.436334089 | 0.127328447 | 0.178635075 | 0.266672256 | 0.194704285 | 0.009647712 | 0.154056503 | 0.174988235 | 0.039807122 | 0 |
| ENSMUSG00000025332 | Kdm5c |  | 6418 | 5.324736916 | 7.002040246 | 5.637800048 | 6.367755807 | 6.172105512 | 6.61681095 | 5.750060699 | 6.450695576 | 5.46757735 | 4.973671877 | 4.726877319 | 6.183380508 | 5.334426705 | 3.893256313 | 4.750568925 | 5.524634225 | 3.941314421 | 6.291163031 |
| ENSMUSG00000025333 | Gpr143 |  | 2292 | 0 | 0 | 0 | 0.019878292 | 0 | 0.064859599 | 0 | 0 | 0 | 0 | 0 | 0 | 0 | 0 | 0 | 0.016706199 | 0 | 0 |
| ENSMUSG00000025337 | Sbds |  | 2084 | 87.86578615 | 102.6881154 | 89.66744134 | 91.90913166 | 97.46483583 | 93.96948325 | 87.72370028 | 93.04358302 | 89.44825832 | 92.01627166 | 94.77047906 | 97.91845605 | 108.2272197 | 99.41798169 | 106.7404992 | 134.5683343 | 120.8087545 | 108.434458 |
| ENSMUSG00000025340 | Rabgef1 |  | 3477 | 16.74779728 | 18.11753896 | 19.40784436 | 20.8215414 | 18.05120174 | 18.95459754 | 17.88002879 | 22.07843797 | 19.63970179 | 17.98154516 | 22.90504912 | 18.01126644 | 20.33150344 | 21.4788877 | 19.26768187 | 25.33985839 | 22.97152797 | 19.12741157 |
| ENSMUSG00000025347 | Mettl7b |  | 1225 | 0 | 0 | 0 | 0 | 0 | 0 | 0 | 0.155887357 | 0 | 0 | 0 | 0 | 0 | 0 | 0 | 0 | 0 | 0 |
| ENSMUSG00000025348 | Itga7 |  | 4231 | 0.13059969 | 0 | 0 | 0 | 0 | 0 | 0 | 0 | 0.126548028 | 0 | 0 | 0 | 0.011659635 | 0 | 0 | 0 | 0.181168976 | 0 |
| ENSMUSG00000025350 | Rdh5 |  | 2476 | 1.356182918 | 0.995619617 | 1.328513758 | 1.104064079 | 0.894211829 | 0.960634577 | 0.898100125 | 1.234003309 | 0.815080482 | 0.742680706 | 1.215598701 | 0.788993615 | 0.478176904 | 1.425585881 | 1.131545154 | 0.278364674 | 0.572727377 | 0.333253878 |
| ENSMUSG00000025351 | Cd63 |  | 1022 | 556.5599468 | 541.7419784 | 559.1828646 | 494.974829 | 492.1983701 | 559.6743676 | 439.5634896 | 424.7503383 | 478.6018211 | 422.4186292 | 407.7700223 | 415.4498486 | 302.7010306 | 257.4420304 | 292.2004668 | 324.0089754 | 261.4963486 | 277.9520813 |
| ENSMUSG00000025352 | Gdf11 |  | 4059 | 0.136133848 | 0.344701049 | 0.190681568 | 0.112246968 | 0.035574223 | 0.085456796 | 0.233124804 | 0.272870084 | 0.142057458 | 0.197477693 | 0.153011693 | 0.165013045 | 0.194459393 | 0.228844974 | 0.121808 | 0.150936122 | 0.066096102 | 0.501438026 |
| ENSMUSG00000025353 | Ormdl2 |  | 2571 | 17.70635688 | 13.20336025 | 14.61930497 | 14.37184246 | 15.9503691 | 14.35892124 | 15.71568565 | 14.15688817 | 14.65797819 | 13.66288367 | 16.29663111 | 12.8521479 | 18.03656252 | 15.49942706 | 14.24666791 | 16.32302828 | 13.83384034 | 14.52788155 |
| ENSMUSG00000025354 | Dnajc14 |  | 4084 | 18.44250047 | 17.65974926 | 19.14094639 | 20.2369575 | 18.66820938 | 19.65607943 | 17.86389591 | 18.01140231 | 15.01633741 | 18.36845467 | 19.20824726 | 15.88095003 | 17.51500491 | 18.27513429 | 20.09635145 | 19.10780066 | 23.75216882 | 21.02576288 |
| ENSMUSG00000025355 | Mmp19 |  | 3401 | 0.087484926 | 0.029385088 | 0.12800993 | 0.066981835 | 0 | 0.014570048 | 0 | 0.10106781 | 0.096880954 | 0.041591329 | 0.014047327 | 0.1477039 | 0.043515364 | 0.013656009 | 0.339207164 | 0.045034528 | 0.022538252 | 0.016174392 |
| ENSMUSG00000025357 | Dgka |  | 2784 | 16.82496551 | 16.35730115 | 15.18623043 | 14.43421017 | 15.36971269 | 17.65672116 | 14.0714433 | 16.72289459 | 14.42415692 | 17.05487004 | 16.04510998 | 15.57786273 | 25.69370691 | 26.00802164 | 24.25623208 | 33.22920527 | 29.66708686 | 22.07082335 |
| ENSMUSG00000025358 | Cdk2 |  | 2424 | 22.09427467 | 25.34198719 | 26.10254856 | 23.0248677 | 26.25016629 | 24.61282641 | 23.40259985 | 23.7914718 | 21.476797 | 20.81322297 | 25.87810251 | 19.22692998 | 19.37458137 | 17.26325195 | 19.30897999 | 17.40769363 | 17.80338709 | 16.225871 |
| ENSMUSG00000025359 | Pmel |  | 1971 | 1.186090655 | 0.676060765 | 0.122713167 | 0.739702394 | 1.001222156 | 0.42739547 | 0.648118889 | 0.155017361 | 0.083585014 | 1.05257785 | 0.266628383 | 0.877871913 | 1.676934783 | 1.413823056 | 0.501693222 | 0.446820888 | 0.466682458 | 0.111636949 |
| ENSMUSG00000025362 | Rps26 |  | 443 | 162.2488773 | 119.4902684 | 145.5573724 | 120.7419098 | 141.1362728 | 132.2151943 | 186.6875006 | 162.4254766 | 184.5490605 | 156.4594442 | 148.070018 | 169.2109139 | 240.646215 | 299.5276697 | 215.7733853 | 155.5826034 | 168.791434 | 229.101133 |
| ENSMUSG00000025364 | Pa2g4 |  | 2492 | 18.54058039 | 16.16183351 | 20.61504384 | 16.34493324 | 15.25851513 | 17.22017146 | 17.41000264 | 20.13836949 | 18.82481599 | 19.65874024 | 18.53867771 | 18.0079425 | 18.54895926 | 19.21502973 | 18.23648321 | 19.16066911 | 15.30283524 | 15.71688764 |
| ENSMUSG00000025366 | Esyt1 |  | 3795 | 34.60895756 | 37.32448649 | 28.92214503 | 36.08866911 | 33.16600274 | 37.39631857 | 12.00582604 | 16.23302901 | 19.30719672 | 17.70480952 | 16.31524274 | 20.09069699 | 14.71508046 | 13.32743377 | 13.60357036 | 13.65143394 | 12.85622555 | 15.46632856 |
| ENSMUSG00000025369 | Smarcc2 |  | 4998 | 44.66530302 | 52.89538386 | 54.34521267 | 54.17552751 | 49.76906393 | 50.58384288 | 40.79984918 | 51.29763852 | 44.7794171 | 49.33937136 | 38.20658444 | 46.13335377 | 29.85775305 | 32.33802003 | 41.92699345 | 37.33295916 | 38.57935168 | 37.59726062 |
| ENSMUSG00000025370 | Cdh9 |  | 3208 | 0 | 0 | 0 | 0 | 0 | 0 | 0 | 0 | 0 | 0 | 0 | 0 | 0 | 0 | 0 | 0 | 0 | 0 |
| ENSMUSG00000025371 | Chmp6 |  | 2423 | 18.26161298 | 20.62292255 | 19.70477691 | 24.63267358 | 20.2817557 | 22.18931735 | 19.350749 | 19.62424308 | 25.78619249 | 22.08668931 | 21.8664585 | 22.18344328 | 28.36127147 | 20.37559426 | 24.29922549 | 24.68424629 | 23.55256132 | 23.61100743 |
| ENSMUSG00000025372 | Baiap2 |  | 4685 | 47.79450743 | 43.38853373 | 40.84646448 | 42.55605763 | 43.60125593 | 41.99025663 | 38.97110523 | 37.19785105 | 42.47881412 | 40.81026717 | 43.62460456 | 41.72176102 | 44.65670463 | 43.30155255 | 38.6863702 | 41.72327658 | 42.52296506 | 46.75480533 |
| ENSMUSG00000025373 | Rnf41 |  | 4367 | 15.87495812 | 13.21988681 | 13.31462869 | 17.31883068 | 15.2430617 | 14.8306441 | 11.67920034 | 9.847640281 | 12.83600823 | 15.3749946 | 10.99469505 | 12.29555013 | 17.0125641 | 13.25150742 | 11.25565747 | 16.56307703 | 13.77886103 | 15.27960762 |
| ENSMUSG00000025374 | Obfc2b |  | 2812 | 21.55490079 | 17.71080258 | 19.43886533 | 19.92890631 | 17.90398014 | 19.52504581 | 27.67758067 | 25.52045666 | 22.43874187 | 25.67131106 | 21.96764627 | 21.23853448 | 32.77098914 | 25.76556768 | 21.75828965 | 28.94944215 | 22.65231346 | 26.33081709 |
| ENSMUSG00000025375 | Aatk |  | 6998 | 0.091108551 | 0.199934489 | 0.062212314 | 0.052084646 | 0.199460673 | 0.049566896 | 0.020282657 | 0.163728504 | 0.00588547 | 0.148230276 | 0 | 0.111663229 | 0.035247154 | 0.185829444 | 0.247279987 | 0.388487157 | 0.383372504 | 0 |
| ENSMUSG00000025377 | 2410002I01Ri | k | 3317 | 4.587534826 | 6.879509358 | 5.527153453 | 5.837637577 | 5.717207695 | 5.303352554 | 6.661145933 | 6.229149744 | 5.413723507 | 5.956027936 | 9.347587595 | 4.173133651 | 6.722347485 | 7.729013036 | 5.254223136 | 6.799266735 | 4.899110608 | 6.617013441 |
| ENSMUSG00000025380 | Fscn2 |  | 2106 | 0.141280262 | 0.20563515 | 0.068908163 | 0.151437469 | 0.182837317 | 0.376469011 | 0.516710169 | 0.290160702 | 0.176010751 | 0.358219968 | 0.158796158 | 0.8746044 | 0.702733864 | 0.815969247 | 0.391277834 | 0.381815174 | 0.127390351 | 0.28732202 |
| ENSMUSG00000025381 | Cnpy2 |  | 1011 | 157.4499349 | 144.2899741 | 150.6708675 | 150.0675348 | 145.2527192 | 145.91344 | 196.5043905 | 173.5091041 | 193.9147527 | 180.7676813 | 156.7925937 | 180.6969967 | 185.0312862 | 148.979402 | 174.8722812 | 171.9479304 | 138.558472 | 176.6711865 |
| ENSMUSG00000025383 | Il23a |  | 1355 | 4.70537004 | 4.204062665 | 4.783803002 | 4.673789788 | 4.617822935 | 5.558683029 | 1.396684249 | 2.085784338 | 3.495534548 | 1.705080782 | 1.974463229 | 5.43739296 | 0.582517104 | 0.514141189 | 0.425698732 | 5.65175017 | 1.329399228 | 0.487165521 |
| ENSMUSG00000025384 | 2310003H01R |  | 4987 | 11.36994282 | 10.34723745 | 11.95030868 | 11.90415897 | 11.55281986 | 11.34734745 | 12.58951773 | 14.96449856 | 12.17343578 | 13.21770266 | 14.40816875 | 16.45258151 | 14.224844 | 13.69946913 | 12.58270897 | 11.60158568 | 13.90260098 | 13.45721079 |
| ENSMUSG00000025386 | Pde6g |  | 2864 | 0.103888349 | 0.023263194 | 0.050670597 | 0.159081859 | 0.016805839 | 0.017301932 | 0 | 0 | 0.0862846 | 0.115242641 | 0.066724802 | 0.214375799 | 0.103348989 | 0 | 0.115088145 | 0.240653259 | 0.200731304 | 0.134449632 |
| ENSMUSG00000025389 | Mip |  | 2140 | 0 | 0 | 0 | 0 | 0 | 0 | 0 | 0 | 0 | 0 | 0 | 0 | 0 | 0 | 0 | 0 | 0 | 0 |

| ENSMUSG00000025393 | Atp5b | 3852 | 698.0691708 | 733.8782167 | 705.158483 | 768.6947745 | 740.3219661 | 713.7548987 | 769.408316 | 714.0650646 | 679.1818971 | 751.6816034 | 784.6900501 | 695.2614544 | 619.5305032 | 586.9654886 | 591.5931606 | 611.377341 | 639.7964356 | 609.7413888 |
| --- | --- | --- | --- | --- | --- | --- | --- | --- | --- | --- | --- | --- | --- | --- | --- | --- | --- | --- | --- | --- |
| ENSMUSG00000025395 | Prim1 | 3397 | 4.041557803 | 2.343768536 | 2.862254813 | 2.87019826 | 2.592917878 | 1.779638945 | 4.05298486 | 3.743912276 | 4.498144642 | 3.747627283 | 4.584820861 | 2.464630376 | 2.831829223 | 5.250082295 | 3.238391352 | 1.904949264 | 3.632931293 | 2.02417967 |
| ENSMUSG00000025396 | Hsd17b6 | 1387 | 0 | 0 | 0 | 0 | 0 | 0.035726556 | 0 | 0 | 0 | 0 | 0 | 0 | 0 | 0 | 0 | 0 | 0 | 0 |
| ENSMUSG00000025400 | Tac2 | 673 | 0 | 0 | 0.143754919 | 0 | 0 | 0 | 0 | 0 | 0 | 0 | 0 | 0 | 0 | 0 | 0 | 0 | 0 | 0 |
| ENSMUSG00000025401 | Myo1a | 3359 | 0 | 0 | 0 | 0 | 0 | 0 | 0 | 0 | 0 | 0 | 0 | 0 | 0 | 0 | 0 | 0 | 0 | 0 |
| ENSMUSG00000025402 | Nab2 | 3470 | 2.180380788 | 2.188858756 | 2.091074799 | 1.838197754 | 1.553537594 | 2.898906316 | 1.881599335 | 2.498465518 | 1.614226554 | 1.372397991 | 0.468111985 | 1.190305196 | 1.919253266 | 2.998119762 | 2.018520029 | 2.791793566 | 2.054377874 | 2.8693511 |
| ENSMUSG00000025403 | Shmt2 | 2268 | 55.69902255 | 56.62310724 | 57.75816587 | 56.69015305 | 57.97901916 | 58.03000899 | 85.80116774 | 81.1167559 | 80.93840421 | 74.28106823 | 85.37562024 | 84.04342493 | 75.08544023 | 75.85048487 | 77.78882819 | 66.63713731 | 69.58892886 | 85.06037799 |
| ENSMUSG00000025404 | R3hdm2 | 5919 | 10.80043669 | 12.5169584 | 12.55309049 | 14.18634984 | 13.75081651 | 15.01902417 | 11.72625443 | 13.46638686 | 11.30037195 | 13.51032146 | 11.30811232 | 11.27817747 | 10.58481753 | 10.2633665 | 13.35796347 | 12.05840382 | 14.41364029 | 12.48137022 |
| ENSMUSG00000025405 | Inhbc | 1964 | 0 | 0 | 0 | 0 | 0 | 0 | 0 | 0 | 0 | 0 | 0 | 0 | 0 | 0 | 0 | 0 | 0 | 0 |
| ENSMUSG00000025407 | Gli1 | 3945 | 0 | 0 | 0.134881834 | 0 | 0 | 0 | 0 | 0 | 0 | 0 | 0 | 0 | 0 | 0 | 0 | 0 | 0.058290946 | 0 |
| ENSMUSG00000025408 | Ddit3 | 1807 | 22.76978993 | 23.33930497 | 18.73905437 | 21.50723344 | 20.11046064 | 21.1428653 | 23.72179696 | 16.54933652 | 20.65023978 | 23.24918462 | 27.3113072 | 16.43272413 | 30.27620158 | 32.59053836 | 25.6512177 | 45.26216791 | 39.13227326 | 49.37729447 |
| ENSMUSG00000025409 | Mbd6 | 4466 | 7.204751064 | 6.370177264 | 6.813020733 | 5.049869446 | 6.649663435 | 5.658731331 | 5.604211185 | 7.106557635 | 5.358120389 | 6.155141066 | 5.252464087 | 6.448921159 | 7.157877831 | 8.153194024 | 7.057588503 | 9.182543781 | 5.921438617 | 7.821491887 |
| ENSMUSG00000025410 | Dctn2 | 1627 | 209.7566431 | 185.8519612 | 190.2533625 | 191.7092253 | 181.5818976 | 194.8607188 | 169.0112415 | 153.6381523 | 186.7695841 | 172.2290286 | 174.0100823 | 180.6890564 | 186.0483371 | 173.1876326 | 161.9193291 | 177.4735528 | 163.8113514 | 162.9310911 |
| ENSMUSG00000025413 | Ttc4 | 4031 | 17.66216072 | 16.8671836 | 18.24057706 | 19.18062323 | 16.66885771 | 15.26779834 | 13.9790128 | 14.53414669 | 15.35681796 | 15.51025366 | 18.0030171 | 13.56967237 | 17.92886599 | 16.29172403 | 14.66738593 | 15.50242403 | 17.22829322 | 15.69349361 |
| ENSMUSG00000025417 | Pip4k2c | 3364 | 40.48352675 | 47.51345614 | 47.53950397 | 45.18182042 | 45.01279187 | 48.43323068 | 42.88238943 | 44.16422276 | 47.12452413 | 50.44452468 | 46.85183948 | 40.94918409 | 33.62606609 | 39.30627729 | 44.37373276 | 34.69374892 | 40.43401537 | 41.45305753 |
| ENSMUSG00000025418 | Bsnd | 2752 | 107.2977681 | 79.94126214 | 98.50481981 | 102.2805712 | 96.59615349 | 112.5740154 | 119.8977554 | 108.9010835 | 122.9010419 | 131.7718237 | 135.6517171 | 136.7199787 | 77.5292668 | 61.61604762 | 79.15429724 | 66.22939362 | 71.06798441 | 92.28817069 |
| ENSMUSG00000025420 | Katnal2 | 4277 | 0.079504655 | 0.07788846 | 0.271443706 | 0.074567994 | 0.011253665 | 0.081101037 | 0.188056007 | 0.250032094 | 0.645194425 | 0.143315207 | 0.25691467 | 0.45675665 | 0.576711682 | 0.217180675 | 0.71286302 | 0.367059832 | 0.295713771 | 0.643080509 |
| ENSMUSG00000025421 | Hdhd2 | 4805 | 10.47369998 | 8.423551816 | 9.080733482 | 8.846712667 | 8.764918462 | 9.188654655 | 11.96355981 | 10.88145659 | 9.625901603 | 11.10813654 | 10.64869522 | 9.502021227 | 11.38586811 | 9.366143742 | 11.29291345 | 11.84179867 | 10.25756874 | 8.196986559 |
| ENSMUSG00000025422 | Agap2 | 5614 | 0 | 0.035603378 | 0.034466356 | 0.081156117 | 0.017147105 | 0.017653272 | 0 | 0.068030642 | 0.337474123 | 0.016797543 | 0.068079741 | 0.069595589 | 0.043936513 | 0 | 0.036695365 | 0.020461671 | 0.081922972 | 0.019597117 |
| ENSMUSG00000025423 | Pias2 | 5439 | 16.6535264 | 16.2491466 | 17.05836206 | 17.67490413 | 16.85811998 | 20.10716731 | 15.09238793 | 15.65193325 | 13.70612126 | 15.64754907 | 15.26620295 | 14.95190667 | 16.49839263 | 17.95769733 | 18.34715359 | 18.38850276 | 16.68627892 | 20.4805003 |
| ENSMUSG00000025425 | St8sia5 | 2463 | 0.120802368 | 0.202879989 | 0.667762904 | 0.129487337 | 0.273587873 | 0.844991803 | 2.996661759 | 4.264275543 | 2.357815153 | 1.780355421 | 1.163823593 | 3.285940347 | 4.105985807 | 5.487303869 | 2.375404359 | 4.057591771 | 3.143285414 | 3.640472755 |
| ENSMUSG00000025427 | Rnf165 | 7289 | 0.081639795 | 0.045702969 | 0.033182556 | 0.031253289 | 0.013206729 | 0.013596579 | 0 | 0 | 0 | 0 | 0 | 0 | 0 | 0.114692492 | 0 | 0 | 0 | 0 |
| ENSMUSG00000025428 | Atp5a1 | 2845 | 496.4963127 | 498.3702649 | 499.956164 | 536.0348949 | 534.2220575 | 494.6216268 | 583.3511956 | 576.2555591 | 549.3803468 | 569.9351614 | 647.9930045 | 564.0030468 | 519.5717291 | 499.5392176 | 535.6057445 | 533.6055225 | 566.313135 | 552.0632742 |
| ENSMUSG00000025429 | Pstpip2 | 4670 | 0.673529556 | 0.620604239 | 0.58006803 | 0.468293389 | 0.87606285 | 0.933756007 | 1.185349772 | 0.703329038 | 1.569850065 | 0.878396399 | 0.951407096 | 0.896397151 | 0.253525913 | 0.397808029 | 0.538178781 | 0.483757138 | 0.599104855 | 0.200247284 |
| ENSMUSG00000025431 | Crisp1 | 1408 | 60.73893052 | 70.34036542 | 61.01661215 | 40.6748812 | 19.24593255 | 53.21287867 | 2015.459637 | 1798.976079 | 1385.0136 | 774.068541 | 410.2946008 | 1250.421481 | 4510.436817 | 6658.591171 | 3544.357993 | 855.6373442 | 1192.171041 | 3127.002048 |
| ENSMUSG00000025432 | Avil | 3470 | 0.171490624 | 0.182404896 | 0.097583491 | 0 | 0.027741743 | 0 | 0.040904333 | 0.198116208 | 0.118693129 | 0.353290572 | 0.068839998 | 0.482556161 | 0.071083454 | 0.548762992 | 0.249346592 | 0.176556115 | 0.110450423 | 0 |
| ENSMUSG00000025433 | Crisp3 | 2171 | 0 | 0.015344493 | 0 | 0 | 0 | 0 | 0.108965175 | 0.052776235 | 0.132798531 | 0.086873705 | 0.022005969 | 0.025709656 | 0.068169393 | 0.406465988 | 0.075912586 | 0.035274627 | 0.07061501 | 0.025338142 |
| ENSMUSG00000025436 | Xrcc6bp1 | 1355 | 4.924953975 | 3.909040724 | 4.069802554 | 3.227941149 | 5.292735518 | 4.059301422 | 6.7390015 | 8.484068726 | 6.079190518 | 4.384493439 | 8.215177362 | 7.455819135 | 10.66734447 | 8.226259021 | 7.845019498 | 7.855932736 | 7.75011465 | 9.093756388 |
| ENSMUSG00000025437 | Usp33 | 6085 | 22.61820213 | 21.37828298 | 20.5577567 | 27.76341042 | 21.23025195 | 26.53127461 | 18.17864311 | 19.01146867 | 16.3663098 | 23.55597993 | 14.60335625 | 20.67518545 | 16.40884151 | 14.509484 | 16.40614132 | 18.21714189 | 18.17743515 | 20.19561946 |
| ENSMUSG00000025439 | Clns1a | 1682 | 50.81920868 | 46.52317986 | 44.60603186 | 48.48648595 | 49.59133396 | 49.52327298 | 45.76559371 | 43.57385268 | 48.3855857 | 42.18894673 | 44.28130813 | 47.75192536 | 42.73282014 | 48.81875535 | 44.70442301 | 47.30551854 | 46.77995692 | 44.11848096 |
| ENSMUSG00000025450 | Gm9752 | 2657 | 1.535753443 | 1.868129936 | 1.383661388 | 1.93767332 | 2.300622622 | 2.070136781 | 1.531385167 | 2.242384183 | 1.643120313 | 2.040771876 | 2.894907157 | 2.583863944 | 2.228012824 | 1.870349022 | 1.58169315 | 2.060804237 | 1.918478543 | 2.422305415 |
| ENSMUSG00000025451 | Paip1 | 3192 | 7.310570689 | 6.846258962 | 6.910504336 | 6.56581466 | 6.483937086 | 7.296298491 | 7.173977632 | 7.657624542 | 7.561183658 | 5.952924001 | 7.199171375 | 8.550707708 | 6.831048696 | 5.339906038 | 6.273169224 | 7.425402876 | 6.471765047 | 7.238040358 |
| ENSMUSG00000025453 | Nnt | 8686 | 5.280115696 | 4.303139226 | 4.394049672 | 4.217255313 | 4.022999837 | 3.782346576 | 5.011243911 | 4.726782476 | 3.940363182 | 4.820380439 | 5.186712623 | 4.260394232 | 3.748453304 | 2.662808602 | 5.744310866 | 4.342188383 | 4.483020683 | 4.173497735 |
| ENSMUSG00000025461 | Cd163l1 | 3821 | 0 | 0 | 0 | 0 | 0 | 0 | 0 | 0 | 0 | 0 | 0 | 0 | 0 | 0 | 0 | 0 | 0 | 0 |
| ENSMUSG00000025464 | Paox | 1932 | 1.210033479 | 2.224308154 | 2.128235031 | 2.193155861 | 1.395128222 | 1.590201595 | 1.665249569 | 1.660539235 | 1.321720485 | 2.050030576 | 1.755704996 | 2.224537277 | 1.863990663 | 1.105811605 | 1.471484139 | 1.704447327 | 1.269608179 | 1.622939484 |
| ENSMUSG00000025465 | Echs1 | 3429 | 48.24442843 | 47.32199118 | 49.03656795 | 46.70372436 | 47.61256491 | 46.76367625 | 66.79518189 | 60.79152299 | 60.45253247 | 66.89652124 | 64.56376716 | 60.27570678 | 66.40890351 | 61.05860191 | 60.70290474 | 57.37449426 | 61.10523919 | 53.37279034 |
| ENSMUSG00000025466 | 1810014F10R | 3265 | 13.83859177 | 13.26393951 | 12.3859943 | 11.16350244 | 11.35117342 | 11.88354989 | 16.86736854 | 15.96711463 | 15.30145287 | 17.0262417 | 17.55894339 | 15.18049261 | 17.05841795 | 13.96878831 | 13.50250071 | 14.97614262 | 14.54403732 | 12.06325281 |
| ENSMUSG00000025467 | Prap1 | 615 | 0 | 0.216669231 | 0 | 0 | 0 | 0 | 0 | 0 | 0 | 0 | 0.077682859 | 0.090757175 | 0 | 0 | 0 | 0 | 0.373915092 | 0.089445702 |
| ENSMUSG00000025468 | Caly | 1442 | 0 | 0.13861121 | 0 | 0 | 0 | 0 | 0 | 0 | 0 | 0 | 0 | 0 | 0 | 0 | 0 | 0 | 0 | 0 |
| ENSMUSG00000025469 | Msx3 | 4287 | 0 | 0 | 0 | 0 | 0 | 0.011558837 | 0.033108943 | 0.106906655 | 0 | 0 | 0 | 0.065098743 | 0.011507328 | 0.173339258 | 0 | 0.107181546 | 0.035760482 | 0 |
| ENSMUSG00000025470 | Zfp511 | 1291 | 26.20768406 | 20.97860806 | 19.8215318 | 21.73942937 | 19.42426974 | 20.57340449 | 24.40762523 | 16.0638842 | 21.78961291 | 19.35698894 | 24.83113644 | 23.69247335 | 24.34115515 | 21.6211437 | 20.96779413 | 21.62188443 | 23.09671494 | 24.4579607 |

| ENSMUSG00000025473 | Adam8 | 4746 | 0.205988 | 0.217593704 | 0.203849685 | 0.16319801 | 0.010141577 | 0.219259883 | 0.328975644 | 0.072425542 | 0.10413784 | 0.04967415 | 0.161061807 | 0 | 0 | 0 | 0.008681322 | 0.024203924 | 0.048452967 | 0.428853129 |
| --- | --- | --- | --- | --- | --- | --- | --- | --- | --- | --- | --- | --- | --- | --- | --- | --- | --- | --- | --- | --- |
| ENSMUSG00000025474 | Tubgcp2 | 3141 | 10.40639298 | 13.02395228 | 14.92325722 | 13.35935111 | 14.25109488 | 14.67177403 | 13.36082337 | 11.67294056 | 7.487265361 | 12.45943448 | 14.96674917 | 12.33240046 | 10.42865109 | 12.1248493 | 12.71069967 | 13.104872 | 13.20250024 | 12.1717062 |
| ENSMUSG00000025475 | Gpr123 | 5151 | 0.552872606 | 0.562652261 | 0.20660409 | 0.229972268 | 0.214916374 | 0.240500551 | 0 | 0 | 0 | 0 | 0 | 0 | 0 | 0 | 0 | 0 | 0 | 0 |
| ENSMUSG00000025477 | Inpp5a | 3865 | 39.0629716 | 42.35434986 | 41.20198239 | 47.23495593 | 38.79196435 | 38.94985889 | 43.35873448 | 43.49882416 | 46.74909305 | 44.54003043 | 48.38064359 | 46.44325249 | 44.31576113 | 41.42115633 | 48.01340433 | 47.92022458 | 51.94130483 | 43.95035487 |
| ENSMUSG00000025478 | Dpysl4 | 6129 | 1.934890539 | 1.929528055 | 1.625868372 | 2.899136448 | 3.840187742 | 1.608907486 | 2.470232869 | 4.256063117 | 1.847983657 | 4.385038482 | 4.606787479 | 3.160064434 | 2.0283314 | 2.682526829 | 2.487285965 | 1.530624704 | 2.745185893 | 1.489886069 |
| ENSMUSG00000025479 | Cyp2e1 | 2000 | 0 | 0 | 0 | 0 | 0 | 0 | 0 | 0 | 0 | 0 | 0 | 0 | 0 | 0 | 0 | 0 | 0 | 0 |
| ENSMUSG00000025480 | Syce1 | 1449 | 0 | 0 | 0.93475421 | 0.188658569 | 0.431825402 | 0.205187303 | 0.065303905 | 0.131788828 | 0 | 0.130160672 | 0 | 0 | 0 | 0.096157531 | 0.966772188 | 0 | 0.185151193 | 0.265744477 |
| ENSMUSG00000025481 | 1190003J15R | 1574 | 11.12587835 | 8.613944058 | 9.803784106 | 8.596969611 | 12.62927857 | 11.14464275 | 9.107840988 | 6.454370419 | 8.687371813 | 8.148024959 | 10.68410761 | 9.78724452 | 21.3750747 | 14.69450795 | 19.37049006 | 21.26174769 | 21.42766282 | 21.8428791 |
| ENSMUSG00000025482 | Odf3 | 1383 | 0 | 0 | 0 | 0 | 0 | 0 | 0 | 0 | 0 | 0 | 0 | 0 | 0 | 0 | 0 | 0 | 0 | 0 |
| ENSMUSG00000025484 | Bet1l | 1548 | 14.71755449 | 15.86020868 | 15.90576676 | 16.33487055 | 14.17839612 | 15.23714547 | 13.75368573 | 22.18021302 | 17.16104823 | 17.51398861 | 19.84450795 | 21.52580786 | 21.51101044 | 23.31205162 | 20.65400993 | 24.68606343 | 20.64866384 | 26.22527181 |
| ENSMUSG00000025485 | Ric8 | 4314 | 19.49878149 | 21.40550366 | 19.70150506 | 20.08741454 | 18.43160359 | 18.45879529 | 17.67919296 | 18.93684486 | 19.0179739 | 18.6132702 | 19.15871075 | 17.05262941 | 15.94081889 | 19.79848788 | 19.55020512 | 19.81099576 | 17.89271256 | 17.63504743 |
| ENSMUSG00000025486 | Sirt3 | 1562 | 17.30684504 | 13.6066751 | 16.07289517 | 12.834097 | 16.57808895 | 14.71989013 | 21.2937345 | 18.14261369 | 19.67038461 | 19.04743518 | 23.64279315 | 16.25872374 | 18.25470434 | 18.1970432 | 18.12129891 | 17.30676621 | 16.68494359 | 16.12943078 |
| ENSMUSG00000025487 | Psmd13 | 3060 | 63.95223217 | 64.32872286 | 59.89781259 | 56.65352082 | 59.12676505 | 54.89665583 | 53.79111438 | 59.0484496 | 59.22244097 | 59.26196233 | 57.12698466 | 54.28346793 | 61.35858731 | 59.75502355 | 63.52579761 | 56.03442482 | 57.8401435 | 53.69679797 |
| ENSMUSG00000025488 | Cox8b | 360 | 0 | 0.092535817 | 0 | 0 | 0 | 0.412939446 | 0 | 0 | 0 | 0 | 0 | 0 | 0 | 0 | 0 | 0 | 0 | 0 |
| ENSMUSG00000025489 | Ifitm5 | 706 | 0.842878845 | 0.566224831 | 0.342588742 | 0.580806514 | 0.613579763 | 0.070188008 | 0 | 0.540968873 | 0.408364887 | 0.667856987 | 0.270679651 | 0.316236049 | 0.209625711 | 0 | 1.575696897 | 0.054235988 | 0.325719237 | 0.857082399 |
| ENSMUSG00000025491 | Ifitm1 | 1091 | 0.389598314 | 0.305342752 | 0.133016124 | 0.375847295 | 0.264703522 | 0.090839108 | 0.173465367 | 1.820352819 | 0.151004641 | 0.907575406 | 0.175160251 | 0.460440846 | 1.130428902 | 0.127710598 | 0.528709241 | 1.08800076 | 0.702590227 | 1.361361945 |
| ENSMUSG00000025492 | Ifitm3 | 676 | 144.8697124 | 131.5267969 | 135.8179891 | 136.683133 | 170.1705585 | 158.2608754 | 98.19480553 | 126.7806967 | 156.7646524 | 100.8578653 | 119.5080692 | 145.9794252 | 133.6194387 | 85.12459212 | 125.7378843 | 172.3077333 | 109.8195815 | 152.2515365 |
| ENSMUSG00000025494 | Sigirr | 1980 | 11.0127047 | 10.59955725 | 11.31158816 | 10.42381469 | 9.918093362 | 11.3370648 | 10.92014862 | 10.24250792 | 12.31435219 | 8.382347246 | 13.51210947 | 10.34563039 | 11.18688427 | 9.757949674 | 11.3408323 | 12.18337508 | 11.13011147 | 12.27981069 |
| ENSMUSG00000025495 | Ptdss2 | 4859 | 18.24774587 | 17.38660213 | 18.23838395 | 16.71853098 | 18.5930481 | 19.25407718 | 18.63685274 | 18.64424336 | 18.42755408 | 21.00869985 | 22.86984018 | 17.89685168 | 17.86873316 | 13.85006846 | 19.40941787 | 17.26584088 | 18.59129076 | 18.05711571 |
| ENSMUSG00000025496 | Drd4 | 2770 | 0.122758631 | 0.060131578 | 0.087316842 | 0 | 0 | 0 | 0 | 0.068939355 | 0.014868778 | 0 | 0 | 0 | 0 | 0.134134548 | 0.07437104 | 0.13823324 | 0 | 0 |
| ENSMUSG00000025497 | Cdhr5 | 2625 | 0.032384896 | 0.152287516 | 0.608124382 | 0.034713177 | 0.165023738 | 0.226526782 | 0 | 0.116395893 | 0 | 0.125735209 | 0 | 0 | 0 | 0 | 0 | 0 | 0 | 0 |
| ENSMUSG00000025498 | Irf7 | 3035 | 6.106180148 | 8.418777551 | 6.885458027 | 8.031353779 | 8.801719155 | 9.388079673 | 5.191144014 | 6.178737918 | 4.328994573 | 5.484085091 | 3.415870178 | 6.620638725 | 5.981596559 | 5.784469533 | 5.620245186 | 3.835368913 | 3.763175116 | 8.736207394 |
| ENSMUSG00000025499 | Hras1 | 4894 | 12.00289196 | 12.74248835 | 12.10820897 | 11.19941486 | 12.32311 | 11.79585914 | 17.74950521 | 14.37482739 | 13.7007862 | 12.68849125 | 14.88696602 | 14.35879019 | 12.8521035 | 14.85185876 | 13.74788328 | 12.77657578 | 10.99512073 | 14.20750121 |
| ENSMUSG00000025500 | 1600016N20R | 2066 | 6.336686455 | 5.111416974 | 5.432071172 | 5.557300664 | 6.87266093 | 5.732383023 | 7.259496234 | 7.986020251 | 6.997321894 | 7.8051987 | 6.867939336 | 6.240763823 | 11.50918883 | 12.52146983 | 10.13087628 | 8.859104713 | 11.7613128 | 8.680042978 |
| ENSMUSG00000025503 | Taldo1 | 2066 | 119.4506544 | 110.2582627 | 111.6384282 | 101.5089482 | 109.4500375 | 104.5020621 | 107.7016113 | 105.5005036 | 109.6645804 | 107.4698265 | 105.7015175 | 106.5252457 | 114.041257 | 115.4133324 | 100.69054 | 99.34058842 | 94.77688813 | 95.66685404 |
| ENSMUSG00000025504 | Eps8l2 | 4451 | 2.664332535 | 2.522229915 | 2.35835904 | 3.408633512 | 2.411462363 | 2.961363094 | 2.753085563 | 3.26064096 | 3.432980757 | 3.495783436 | 3.885539198 | 4.752670438 | 2.615666699 | 2.086905747 | 2.767752238 | 3.544311447 | 2.815704131 | 1.680799486 |
| ENSMUSG00000025505 | Tmem80 | 3611 | 5.438214158 | 5.406080314 | 5.733556076 | 7.292795244 | 6.544662011 | 7.01230874 | 10.41638874 | 8.302696175 | 9.079054707 | 8.787710687 | 8.004389332 | 6.8938758 | 6.940075873 | 5.260490651 | 7.439328286 | 6.977352443 | 5.678363014 | 6.459114165 |
| ENSMUSG00000025507 | Lrdd | 4743 | 0.304696602 | 0.217731335 | 0.489548694 | 0.624387072 | 0.304439745 | 0.323874076 | 0.847897472 | 0.740818263 | 0.720742317 | 0.775406885 | 0.03021819 | 0.670776464 | 0.457643761 | 0.538567322 | 0.399593416 | 0.460165427 | 0.339385298 | 0.197165257 |
| ENSMUSG00000025508 | Rplp2 | 2142 | 39.54846677 | 32.67525245 | 36.58502296 | 28.18318569 | 36.40229521 | 32.96575413 | 43.88902569 | 35.83880898 | 37.86006047 | 35.70422067 | 35.53011618 | 36.92380667 | 54.76717987 | 58.23941117 | 44.72157677 | 41.11503129 | 36.55491343 | 48.1008669 |
| ENSMUSG00000025509 | Pnpla2 | 3276 | 88.63029837 | 89.11911018 | 84.68321019 | 98.82685624 | 95.20600286 | 92.25339502 | 98.49587005 | 113.6328896 | 100.4267057 | 114.8686699 | 117.8186476 | 101.6812803 | 106.0726573 | 106.7817052 | 96.03634945 | 115.4211685 | 117.8919701 | 109.3801347 |
| ENSMUSG00000025510 | Cd151 | 2001 | 87.4319363 | 67.42489834 | 79.70391283 | 60.47482946 | 70.5742449 | 52.82157957 | 39.46269922 | 51.47671629 | 63.31320464 | 49.03578884 | 45.45903096 | 60.92024342 | 59.46455996 | 41.05926566 | 48.40822484 | 81.36514876 | 42.67415767 | 39.50429103 |
| ENSMUSG00000025511 | Tspan4 | 2068 | 0.698827846 | 1.304808719 | 0.514612025 | 1.013446828 | 1.070632731 | 0.83865845 | 0.8007465 | 1.80989141 | 1.254714939 | 1.140007332 | 1.524732717 | 1.835331265 | 2.146940307 | 2.223387162 | 2.709580078 | 6.647160569 | 4.336727986 | 2.048211421 |
| ENSMUSG00000025512 | Chid1 | 12684 | 3.374543698 | 2.986184226 | 4.046382506 | 3.897329951 | 3.999609551 | 3.203503748 | 3.610743709 | 3.694581973 | 4.292697401 | 4.003572013 | 3.807985101 | 3.762408665 | 2.850858985 | 2.852408079 | 3.170349933 | 3.695025501 | 3.417458357 | 3.426142724 |
| ENSMUSG00000025515 | Muc2 | 6990 | 0.109455389 | 0.233523865 | 0.089965078 | 0.071698353 | 0 | 0.049623624 | 0 | 0.092885671 | 0.023568822 | 0 | 0 | 0.09582088 | 0.098804985 | 0.039866169 | 0.064837928 | 0.098602422 | 0.016449054 | 0 |
| ENSMUSG00000025519 | Tktl2 | 2038 | 0 | 0 | 0 | 0 | 0 | 0 | 0 | 0 | 0 | 0 | 0 | 0 | 0 | 0 | 0 | 0 | 0 | 0 |
| ENSMUSG00000025521 | Tmem192 | 1117 | 138.7031035 | 129.613105 | 123.6837983 | 122.4070672 | 138.0183988 | 122.5731449 | 177.2213506 | 139.4347511 | 140.1890178 | 162.0937337 | 154.2741945 | 126.7220415 | 209.1196314 | 175.8388949 | 171.9620891 | 182.5745524 | 190.5335064 | 190.1926322 |
| ENSMUSG00000025525 | Apool | 3599 | 16.68791712 | 15.20785918 | 16.00802297 | 15.97611502 | 14.63082092 | 15.69605898 | 19.0354985 | 15.67384783 | 17.00560223 | 17.04447484 | 15.95596001 | 14.60915648 | 18.40865932 | 15.61471124 | 16.21044822 | 16.1822767 | 16.99604608 | 17.59252066 |
| ENSMUSG00000025527 | Satl1 | 2732 | 0 | 0.012193592 | 0 | 0 | 0.017617834 | 0 | 0 | 0.013979649 | 0 | 0 | 0 | 0 | 0 | 0 | 0 | 0.042046787 | 0 | 0 |
| ENSMUSG00000025528 | 2010106E10R | 1424 | 0 | 0 | 0 | 0 | 0 | 0 | 0 | 0 | 0 | 0 | 0 | 0 | 0 | 0 | 0.434005153 | 0.134447357 | 0 | 0.309039925 |
| ENSMUSG00000025529 | Zfp711 | 5526 | 0.307674094 | 0.337589952 | 0.070030446 | 0.206121265 | 0.209042014 | 0.286950321 | 0.488024375 | 0.476886675 | 0.156517704 | 0.332768264 | 0.449565299 | 0.030301663 | 0.142835808 | 0.32778129 | 0 | 0.034645863 | 0.13871262 | 0.029863793 |
| ENSMUSG00000025531 | Chm | 7219 | 4.781022709 | 6.358937281 | 4.931835205 | 5.478180701 | 5.780631364 | 5.457047125 | 4.030654875 | 5.34873512 | 4.541413845 | 5.545220538 | 5.367154914 | 5.435435766 | 4.209511157 | 2.978752246 | 3.869601731 | 5.53752516 | 4.629523604 | 4.907309147 |

| ENSMUSG00000025532 | Crcp | 1637 | 57.30539004 | 50.46791549 | 48.63948135 | 46.28467729 | 46.75000527 | 46.04136089 | 44.33576633 | 37.91243366 | 35.19849453 | 40.64119871 | 39.69086349 | 38.18786933 | 45.65537854 | 44.79854245 | 43.69328102 | 41.65887097 | 41.93182514 | 49.46451136 |
| --- | --- | --- | --- | --- | --- | --- | --- | --- | --- | --- | --- | --- | --- | --- | --- | --- | --- | --- | --- | --- |
| ENSMUSG00000025533 | Asl | 3821 | 233.3724386 | 247.1832444 | 235.6009945 | 196.6720401 | 239.2235965 | 240.1511438 | 143.6715555 | 165.3438159 | 152.1346463 | 137.3552677 | 158.3788011 | 158.872323 | 122.9618373 | 143.0881436 | 131.4654199 | 113.9599025 | 125.7114575 | 131.4690297 |
| ENSMUSG00000025534 | Gusb | 4093 | 28.18447294 | 30.47238601 | 26.82822231 | 25.60234595 | 29.66939736 | 29.74616819 | 25.82372947 | 26.03391222 | 29.31256301 | 25.98875801 | 29.2859445 | 26.60551129 | 23.29797119 | 22.61496852 | 19.70990629 | 19.89839285 | 20.4506798 | 20.15970195 |
| ENSMUSG00000025535 | 4930579G22R | 618 | 7.359310413 | 7.115375464 | 6.575042961 | 6.635103544 | 7.243153562 | 8.49933618 | 9.26348568 | 10.07340063 | 10.72982045 | 10.83398036 | 11.44125219 | 9.844510068 | 9.10006241 | 10.89707552 | 11.33376133 | 9.231877836 | 9.798632542 | 12.10556394 |
| ENSMUSG00000025537 | Phkg1 | 3221 | 0 | 0.103424074 | 0.060072686 | 0 | 0.029886323 | 0.03076854 | 0.220332252 | 0.047429249 | 0 | 0.073192647 | 0 | 0.03465735 | 0.015315715 | 0.014419152 | 0.025583083 | 0.142653613 | 0.202280983 | 0.375721934 |
| ENSMUSG00000025538 | Sumf2 | 2745 | 5.884140944 | 6.662578844 | 5.815397092 | 5.07893609 | 4.55894359 | 5.560015279 | 7.239098415 | 4.77231112 | 5.866640314 | 5.204613146 | 8.249664961 | 6.201740287 | 5.085949941 | 4.83898324 | 5.493540785 | 7.365187872 | 3.867535243 | 4.689300901 |
| ENSMUSG00000025544 | Tm9sf2 | 3443 | 135.1943603 | 154.3152338 | 157.0488883 | 155.73858 | 154.7966282 | 164.9790836 | 124.7746514 | 144.6829813 | 135.8090367 | 139.5485525 | 139.7865035 | 143.9891708 | 117.1615125 | 107.7939885 | 132.3643363 | 128.1395232 | 143.1865109 | 134.1436131 |
| ENSMUSG00000025545 | Clybl | 1231 | 50.93024747 | 49.06277597 | 48.60930711 | 55.07297642 | 51.41631164 | 56.43617585 | 68.91278092 | 54.23259091 | 54.10121526 | 71.01332562 | 66.20965001 | 63.29704709 | 60.51274987 | 50.36787706 | 50.17151282 | 72.5064223 | 66.502819 | 54.47286848 |
| ENSMUSG00000025551 | Fgf14 | 6367 | 0 | 0.005232118 | 0 | 0 | 0 | 0 | 0 | 0 | 0 | 0 | 0 | 0 | 0 | 0 | 0 | 0 | 0 | 0 |
| ENSMUSG00000025555 | Farp1 | 5496 | 11.01298593 | 12.40747716 | 10.7819459 | 12.10318863 | 10.50915364 | 10.68413197 | 8.617174601 | 9.381321551 | 10.39404974 | 9.025207392 | 8.484053762 | 7.494897924 | 6.687095773 | 7.36040759 | 7.74403334 | 6.960028529 | 6.694549677 | 4.874351342 |
| ENSMUSG00000025557 | Slc15a1 | 3101 | 0.095948479 | 0.096683666 | 0 | 0 | 0 | 0.07989799 | 0.030514466 | 0.012316157 | 0 | 0 | 0 | 0 | 0 | 0.179725588 | 0.053146154 | 0 | 0.012359335 | 0.141913207 |
| ENSMUSG00000025558 | Dock9 | 10450 | 3.762419888 | 3.267532687 | 3.97172144 | 3.291730954 | 3.187300591 | 3.101195001 | 2.707462392 | 3.165035453 | 3.058443659 | 2.883186544 | 3.739704884 | 3.445081566 | 3.51224368 | 2.933310782 | 3.307952669 | 3.539591077 | 4.144374689 | 3.316338491 |
| ENSMUSG00000025571 | Tnrc6c | 12934 | 2.990545089 | 2.81771349 | 3.208944568 | 2.902605577 | 2.165824925 | 2.065016499 | 2.264307117 | 2.645770261 | 2.235420911 | 2.387792689 | 2.01678733 | 3.072580158 | 2.364758676 | 2.255055426 | 1.978209851 | 2.646652468 | 1.763116333 | 2.466776086 |
| ENSMUSG00000025572 | Tmc6 | 4338 | 4.370057286 | 4.745820338 | 4.538503194 | 5.408929883 | 4.715552688 | 5.882816458 | 3.882737137 | 5.097602811 | 5.373805422 | 5.282443933 | 4.856790385 | 5.699939725 | 3.832378083 | 3.811455765 | 5.822165459 | 6.973162713 | 6.917817076 | 5.972634686 |
| ENSMUSG00000025573 | 6030468B19R | 1065 | 0 | 0 | 0 | 0 | 0.135582884 | 0.139585165 | 0 | 0 | 0.502746202 | 0 | 0 | 0.052409073 | 0 | 0 | 0 | 0.107860866 | 0.071974266 | 0 |
| ENSMUSG00000025574 | Tk1 | 5706 | 0.722573094 | 0.607175079 | 0.279762794 | 0.558933246 | 0.379589303 | 0.234476657 | 0.364836641 | 0.388215797 | 0.461958817 | 0.553644781 | 0.51911101 | 0.665170883 | 0.596547896 | 0.634882372 | 0.353816376 | 0.369082266 | 0.503763103 | 0.829089236 |
| ENSMUSG00000025575 | Cant1 | 6122 | 16.1494674 | 15.84022134 | 15.5898359 | 21.08370444 | 17.38315636 | 18.24434196 | 15.28658591 | 16.5196801 | 17.33708774 | 17.60642072 | 18.9320564 | 15.15283097 | 13.01389193 | 12.1989697 | 17.91547563 | 14.21668583 | 16.96574071 | 16.93762923 |
| ENSMUSG00000025576 | Rbfox3 | 6525 | 0.130284064 | 0.582018382 | 0.2075799 | 0.26533635 | 0.154907341 | 0.151885773 | 0.072509853 | 0.052679176 | 0.050496878 | 0 | 0.014643665 | 0.034216498 | 0.128527601 | 0.284714712 | 0.353607223 | 0.134970723 | 0.070485144 | 0.016861029 |
| ENSMUSG00000025577 | Cbx2 | 4252 | 5.488085992 | 9.479915806 | 6.484692451 | 8.92576433 | 8.150278702 | 8.752140855 | 4.03915862 | 5.883354557 | 5.966814137 | 5.433641721 | 6.314564126 | 4.856960291 | 3.329788396 | 4.620378402 | 4.350775775 | 3.926318163 | 2.866359928 | 3.7000481 |
| ENSMUSG00000025578 | Cbx8 | 4255 | 2.487376929 | 3.04552664 | 2.614785425 | 1.574024328 | 2.613037454 | 1.863322532 | 2.190504759 | 2.52222446 | 1.974629663 | 2.349224228 | 2.717165676 | 2.334944287 | 4.023072925 | 2.903437661 | 2.44013915 | 2.807677911 | 2.143750567 | 2.275347305 |
| ENSMUSG00000025579 | Gaa | 4958 | 23.61874949 | 28.07877873 | 29.57244262 | 33.44941537 | 27.89088679 | 33.41161524 | 29.34378534 | 34.19444822 | 29.19108842 | 37.62165835 | 26.67226403 | 34.84257275 | 26.81515068 | 26.30395268 | 36.57282119 | 27.25444807 | 32.41269926 | 28.436535 |
| ENSMUSG00000025580 | Eif4a3 | 2240 | 69.08988657 | 67.30989252 | 68.86749478 | 65.06686644 | 66.69709423 | 68.31198272 | 62.01340417 | 68.71222397 | 71.26738174 | 62.83252197 | 66.54369217 | 69.89416677 | 58.73581401 | 65.66447534 | 65.37068287 | 66.32480211 | 62.65665145 | 70.750552 |
| ENSMUSG00000025582 | Nptx1 | 5212 | 7.79642139 | 8.07895209 | 7.100105664 | 7.919859203 | 6.73218963 | 8.033779715 | 7.824928863 | 5.569114702 | 4.891491415 | 5.952640588 | 3.969024755 | 6.136295983 | 4.74199742 | 6.050563188 | 7.723315446 | 6.406256649 | 7.750559658 | 7.409131412 |
| ENSMUSG00000025583 | Rptor | 12980 | 5.743765697 | 7.075781923 | 6.40259824 | 7.05529269 | 6.782225608 | 5.485922816 | 6.047128994 | 5.761226803 | 5.245093263 | 5.514234788 | 4.295329472 | 5.82667356 | 4.488520362 | 4.780377562 | 4.126504057 | 5.259796071 | 4.721398209 | 3.915902512 |
| ENSMUSG00000025584 | Pde8a | 4251 | 1.449835456 | 1.810227844 | 1.695520118 | 2.089956162 | 1.607794204 | 1.620284631 | 1.981100176 | 3.809357473 | 3.952975401 | 2.462351476 | 1.753209485 | 2.639131541 | 2.518237132 | 2.687660668 | 1.773673189 | 2.062702681 | 2.587543451 | 3.092725596 |
| ENSMUSG00000025586 | Cpeb1 | 5590 | 9.124545838 | 9.439646591 | 9.094915993 | 9.299669336 | 8.989217944 | 9.591423566 | 11.47692176 | 11.49871436 | 10.04980456 | 12.0871123 | 12.25568702 | 9.53559173 | 14.04944764 | 13.61750612 | 12.2204257 | 12.46670939 | 10.54487382 | 10.96245884 |
| ENSMUSG00000025588 | Nat1 | 1346 | 0 | 0 | 0 | 0 | 0.14303692 | 0 | 0 | 0 | 0 | 0 | 0 | 0 | 0.219904535 | 0.207031593 | 0 | 0 | 0 | 0 |
| ENSMUSG00000025591 | 1810029B16R | 3797 | 2.37321499 | 2.570628919 | 2.917444944 | 2.927810065 | 2.966254975 | 2.061978379 | 2.10583164 | 2.403998993 | 3.416842893 | 3.191396033 | 2.390635268 | 3.322185869 | 3.092177064 | 2.70322447 | 3.038302778 | 2.067309591 | 3.068526273 | 2.883016128 |
| ENSMUSG00000025592 | Dach2 | 10047 | 0.063459504 | 0.026525645 | 0.067406134 | 0.072556655 | 0.153301638 | 0.014796278 | 0.014127405 | 0.045616485 | 0.012298153 | 0.018772053 | 0.066572053 | 0.044443645 | 0 | 0 | 0.004100881 | 0 | 0.019073503 | 0.054751773 |
| ENSMUSG00000025597 | Klhl4 | 3898 | 0 | 0 | 0 | 0 | 0 | 0 | 0 | 0 | 0 | 0 | 0 | 0 | 0 | 0 | 0 | 0 | 0 | 0 |
| ENSMUSG00000025602 | Zfp202 | 3990 | 2.471478907 | 2.279303289 | 1.697316855 | 1.370256973 | 1.507892345 | 1.949819341 | 1.280643941 | 2.153706903 | 2.828347198 | 1.252625199 | 3.125128863 | 1.720633208 | 2.250227806 | 0.826448673 | 2.447310463 | 1.458689806 | 1.815456169 | 1.447608072 |
| ENSMUSG00000025607 | Copg2 | 4632 | 20.09635913 | 17.04480987 | 16.59446022 | 16.67227335 | 19.0470242 | 14.6775368 | 16.86382403 | 16.8864508 | 18.98385388 | 16.63304169 | 14.02718988 | 11.97771343 | 15.24049517 | 10.30753926 | 13.65379713 | 14.60697394 | 15.99411311 | 12.29154263 |
| ENSMUSG00000025608 | Podxl | 5706 | 0.536342915 | 0.280234652 | 0.635824531 | 0.375283751 | 0.438636528 | 0.399478749 | 0.331669674 | 0.180721147 | 0.267069941 | 0.594961555 | 0.234437231 | 0.645607033 | 0.31124238 | 1.001160664 | 0.433244541 | 0.348950506 | 0.214938924 | 0.15424916 |
| ENSMUSG00000025609 | Mkln1 | 22326 | 7.674387018 | 8.479672931 | 8.190089794 | 7.613914558 | 8.026299015 | 7.632887699 | 7.217906675 | 7.961454842 | 8.275674537 | 7.349478068 | 7.483159984 | 6.90508197 | 7.106129444 | 5.939168212 | 8.867395685 | 6.946025261 | 8.725815962 | 8.510322253 |
| ENSMUSG00000025610 | ORF63 | 1647 | 0.025807636 | 0 | 0.205594847 | 0.165978304 | 0 | 0 | 0 | 0 | 0 | 0.171769411 | 0 | 0 | 0 | 0 | 0 | 0 | 0 | 0 |
| ENSMUSG00000025612 | Bach1 | 5520 | 1.778749214 | 1.6777146 | 1.191811617 | 1.493940041 | 1.212017643 | 1.373472507 | 1.157103561 | 1.923457947 | 2.357778801 | 2.212324664 | 0.943382332 | 1.668402958 | 1.179676283 | 0.950757586 | 1.298744697 | 1.824353215 | 1.52749734 | 1.594466862 |
| ENSMUSG00000025613 | Cct8 | 3135 | 84.21041413 | 82.44808493 | 79.43442879 | 83.31785237 | 77.14925564 | 78.14632051 | 72.71211709 | 73.19296767 | 75.72538337 | 72.04206335 | 69.39941337 | 62.88386611 | 62.69166138 | 67.27355688 | 72.95369593 | 74.15064674 | 71.84805324 | 65.95828777 |
| ENSMUSG00000025616 | Usp16 | 7419 | 10.34699392 | 8.459562301 | 8.202439842 | 9.101154833 | 9.471978509 | 7.580853567 | 8.36692745 | 8.154301848 | 7.355726295 | 7.098980393 | 8.963841785 | 7.922077464 | 8.943446386 | 10.12267008 | 8.369152814 | 8.149463416 | 8.80280495 | 9.090330886 |
| ENSMUSG00000025626 | Phf6 | 6963 | 9.992958948 | 9.726427394 | 10.96966888 | 10.84880246 | 10.69367886 | 11.43634107 | 8.391664294 | 7.103139611 | 7.890681032 | 7.191447201 | 8.748107434 | 8.360726135 | 6.503906371 | 6.976951804 | 6.278163263 | 5.944585179 | 8.641718532 | 8.579619403 |
| ENSMUSG00000025630 | Hprt | 1280 | 210.4338285 | 193.9174803 | 228.2248045 | 199.6499201 | 197.303284 | 202.275807 | 205.9580055 | 207.6711881 | 243.4830975 | 221.6083053 | 212.0013782 | 195.9216969 | 236.4077973 | 182.9098785 | 226.1257267 | 203.2692791 | 235.8863805 | 227.1274446 |
| ENSMUSG00000025644 | Gm7628 | 2008 | 0.084671665 | 0.116130607 | 0 | 0.249587394 | 0.191760652 | 0.123388281 | 0.141372547 | 0.228241449 | 0.348690621 | 0.234814259 | 0.16654617 | 0.027796645 | 0.171973815 | 0 | 0.061556109 | 0.07627611 | 0.076347205 | 0.08218492 |

| ENSMUSG00000025645 | Ccdc51 | 2395 | 4.436865974 | 4.951728744 | 4.160729542 | 4.717803338 | 5.164887007 | 4.531126786 | 6.973434514 | 3.938840666 | 4.161643764 | 5.315528133 | 5.385903461 | 4.800846135 | 4.840501278 | 4.964378443 | 4.231976096 | 6.411078734 | 4.800788756 | 5.810982881 |
| --- | --- | --- | --- | --- | --- | --- | --- | --- | --- | --- | --- | --- | --- | --- | --- | --- | --- | --- | --- | --- |
| ENSMUSG00000025646 | Atrip | 6784 | 3.759302125 | 3.736897479 | 3.75065993 | 2.793837624 | 4.107957518 | 3.54991576 | 3.00586153 | 3.86202654 | 3.296621176 | 3.301383262 | 3.105653993 | 3.669484893 | 2.217900172 | 3.601059842 | 3.437511886 | 2.692308333 | 3.604389361 | 2.513682648 |
| ENSMUSG00000025647 | Shisa5 | 3054 | 180.333846 | 190.2565491 | 175.658956 | 208.9035049 | 189.0289104 | 204.7660438 | 162.6818734 | 154.0576965 | 154.8068155 | 178.9997556 | 175.7067235 | 161.8179033 | 193.5801233 | 151.7112691 | 167.3425341 | 197.9225699 | 181.9306897 | 182.5711545 |
| ENSMUSG00000025648 | Pfkfb4 | 4325 | 2.201424146 | 1.1707653 | 0.827662716 | 1.811907428 | 1.268679606 | 1.558190004 | 1.553387378 | 1.200963405 | 1.076087001 | 2.202183135 | 1.458102779 | 1.084049863 | 1.904839349 | 1.771855357 | 1.686167721 | 2.523196095 | 1.338097302 | 2.365709561 |
| ENSMUSG00000025650 | Col7a1 | 9920 | 1.405413078 | 1.776463815 | 1.165450983 | 2.043817009 | 0.883065535 | 0.904137578 | 0.228932317 | 0.169401785 | 0.568805712 | 0.404013082 | 0.192640962 | 0.433246575 | 0.08951356 | 0.491595683 | 0.290736786 | 0.490212413 | 0.142950906 | 0.105360184 |
| ENSMUSG00000025651 | Uqcrc1 | 1718 | 302.5091893 | 310.2675322 | 293.0568707 | 326.4058985 | 332.6649951 | 307.3827018 | 286.6575524 | 298.4253841 | 307.0768803 | 296.5169953 | 345.3534397 | 296.1024149 | 284.3046059 | 284.8282333 | 281.1682429 | 283.2123098 | 284.5917169 | 269.1539878 |
| ENSMUSG00000025652 | Tmem89 | 536 | 0 | 0 | 0 | 0 | 0 | 0 | 0 | 0 | 0 | 0 | 0 | 0 | 0 | 0 | 0 | 0 | 0 | 0 |
| ENSMUSG00000025656 | Arhgef9 | 9209 | 0.147699602 | 0.170019115 | 0.152332759 | 0.291899404 | 0.125438828 | 0.31209236 | 0.899089892 | 0.622093643 | 0.43382474 | 0.660488731 | 1.011631763 | 0.545489155 | 1.258877246 | 0.872497244 | 0.738218777 | 0.844064861 | 0.990515655 | 1.134947364 |
| ENSMUSG00000025658 | Cnksr2 | 9521 | 0.366077558 | 0.489844049 | 0.106695109 | 0.129203676 | 0.156715643 | 0.333092632 | 0.332942914 | 0.120341568 | 0.051910323 | 0.123807119 | 0.045160658 | 0.02931187 | 0.113990356 | 0.024390341 | 0.05625672 | 0.104564205 | 0.177119742 | 0.028888303 |
| ENSMUSG00000025665 | Rps6ka6 | 6702 | 0.088790281 | 0.33800012 | 0.281493238 | 0.095173772 | 0.179543135 | 0.02218117 | 0 | 0.125370464 | 0.012290813 | 0.351765915 | 0.092670018 | 0.00832821 | 0.125133183 | 0 | 0 | 0.005713311 | 0.068623629 | 0.139533694 |
| ENSMUSG00000025666 | Tmem47 | 1993 | 1.962105466 | 2.607532111 | 4.1990069 | 4.252059331 | 6.279127021 | 6.26557394 | 2.184027326 | 4.369226166 | 3.099838116 | 2.460448138 | 3.931306167 | 4.536947987 | 1.881197046 | 0.069910819 | 1.736543249 | 0.211338024 | 1.173057758 | 1.324855556 |
| ENSMUSG00000025701 | Alox5 | 3878 | 0.076724144 | 0.197575185 | 0.324319698 | 0.25846905 | 0.235819121 | 0.191668645 | 0.122002782 | 0.364393731 | 0.23365223 | 0.133743614 | 0.147833807 | 0.30225088 | 0.203535502 | 0 | 0.07437104 | 0.019747606 | 0.158128094 | 0.014184917 |
| ENSMUSG00000025702 | 8-Mar | 6538 | 23.34599069 | 29.2927239 | 28.20432819 | 29.37286661 | 28.24014365 | 30.87003424 | 21.70246621 | 22.46680631 | 22.76030613 | 25.01049845 | 25.77275597 | 25.50036466 | 22.40233441 | 25.35315814 | 27.45089897 | 25.13663 | 24.85523078 | 22.86857633 |
| ENSMUSG00000025716 | Myo3a | 8046 | 0 | 0.004140305 | 0 | 0 | 0 | 0 | 0 | 0 | 0 | 0.017580426 | 0 | 0 | 0 | 0.155852642 | 0 | 0 | 0.052397373 | 0 |
| ENSMUSG00000025722 | Wdr73 | 3712 | 8.507905654 | 11.72053875 | 8.88759367 | 8.260394084 | 9.271100599 | 8.863958808 | 9.508420927 | 11.05028022 | 11.20646038 | 11.17796844 | 9.794381304 | 9.42791499 | 11.2299219 | 10.42239353 | 11.02186018 | 12.46095198 | 11.31616956 | 11.52938713 |
| ENSMUSG00000025723 | Nmb | 1060 | 17.48326108 | 17.28499228 | 19.0299643 | 15.43058011 | 19.29817694 | 17.99792682 | 24.54903152 | 17.54688677 | 22.22517643 | 26.33322295 | 26.63679289 | 15.05969764 | 37.97626839 | 33.12427365 | 32.0283793 | 22.75762515 | 26.93687848 | 29.9436364 |
| ENSMUSG00000025724 | Sec11a | 3888 | 81.67596968 | 73.90869998 | 74.48876704 | 72.22034883 | 76.06032382 | 75.36144898 | 74.75354595 | 68.93885805 | 74.33276263 | 77.33539986 | 74.7958262 | 71.16209862 | 78.85747579 | 72.49721357 | 77.63441318 | 79.33876883 | 79.93517016 | 78.87751286 |
| ENSMUSG00000025726 | Slc28a1 | 2959 | 0 | 0 | 0.032695864 | 0 | 0 | 0 | 0.335777715 | 0 | 0 | 0.09560805 | 0 | 0.075452062 | 0 | 0 | 0.125317338 | 0 | 0.129524491 | 0 |
| ENSMUSG00000025727 | A930017K11R | 2536 | 0.067042864 | 0.065679996 | 0.209822095 | 0.053897134 | 0.227753582 | 0.156317772 | 0.783569604 | 0.361442294 | 0.01624074 | 0.241703132 | 0.37677412 | 1.716727792 | 0.31124238 | 0 | 0.194960044 | 0 | 0.272032076 | 0.34706061 |
| ENSMUSG00000025728 | Pigq | 5356 | 20.6256446 | 20.36962819 | 18.00008327 | 20.23706581 | 20.21073494 | 20.06719177 | 17.60533404 | 19.11759351 | 17.73265596 | 19.79871763 | 19.18669449 | 15.89224896 | 14.55273138 | 15.92071405 | 18.03914275 | 17.10065962 | 15.94305256 | 18.78484992 |
| ENSMUSG00000025730 | Rab40c | 4876 | 6.973777855 | 6.982317041 | 7.033804968 | 7.643341728 | 6.406402473 | 7.957299096 | 6.180922165 | 6.407174973 | 7.154425523 | 7.223456795 | 7.191718524 | 6.570588664 | 7.891487997 | 9.982240276 | 8.238621205 | 7.601580797 | 7.915213491 | 9.171944989 |
| ENSMUSG00000025731 | 0610011F06R | 1329 | 112.8993765 | 100.0138811 | 96.41947473 | 96.19585433 | 103.4347434 | 98.62075268 | 102.6354051 | 91.09850687 | 95.20314253 | 95.86245312 | 104.177449 | 92.39613069 | 93.02165891 | 82.68375528 | 85.93733102 | 90.78533026 | 74.86461005 | 94.0411516 |
| ENSMUSG00000025732 | Fam195a | 811 | 21.75049082 | 13.76056666 | 14.67310539 | 16.12332888 | 10.92018983 | 13.07556718 | 29.05266844 | 21.99241914 | 20.5170806 | 23.54628214 | 26.74455136 | 20.16521472 | 24.02725934 | 28.92017772 | 21.03260683 | 18.83841227 | 14.98080902 | 23.06177101 |
| ENSMUSG00000025733 | Rhot2 | 4186 | 4.457661796 | 3.875628162 | 3.628592578 | 4.321006833 | 4.047404952 | 3.350077306 | 4.837512322 | 6.258955578 | 4.486634302 | 5.034965207 | 5.272821504 | 3.560148569 | 5.173605276 | 4.770892876 | 5.059149481 | 6.156134444 | 4.220836807 | 4.914812689 |
| ENSMUSG00000025735 | Rhbdl1 | 1561 | 0 | 0.298770352 | 0.030988809 | 0 | 0.030834032 | 0 | 0 | 0.024466625 | 0.052769399 | 0.060410895 | 0 | 0.10726905 | 0.252822126 | 0.029752779 | 0.026394334 | 0.024529537 | 0.098209601 | 0.317156925 |
| ENSMUSG00000025736 | Jmjd8 | 2537 | 21.89762123 | 20.9830528 | 18.62867133 | 19.6287826 | 18.74432029 | 18.63354664 | 18.53717101 | 20.05214032 | 19.12405028 | 18.41795307 | 18.36049843 | 19.40457801 | 16.78101877 | 19.35017752 | 18.44894265 | 14.94193982 | 16.23995631 | 16.91253577 |
| ENSMUSG00000025737 | Wdr24 | 4461 | 7.498671494 | 6.556539145 | 6.744751374 | 8.39524287 | 6.851327399 | 6.653684693 | 8.930122317 | 9.297679671 | 8.170828615 | 6.510834613 | 7.443083646 | 8.745830116 | 8.371275808 | 7.662597695 | 8.40471102 | 7.081316096 | 6.701302754 | 10.76506392 |
| ENSMUSG00000025738 | Fbxl16 | 3472 | 0 | 0 | 0 | 0 | 0 | 0 | 0.054507695 | 0 | 0 | 0.08148163 | 0 | 0.032151879 | 0 | 0 | 0 | 0 | 0 | 0 |
| ENSMUSG00000025739 | Gng13 | 769 | 0.055273311 | 0 | 0 | 0 | 0 | 0 | 0 | 0 | 0 | 0 | 0 | 0 | 0 | 0 | 0 | 0 | 0 | 0 |
| ENSMUSG00000025742 | Prps2 | 3865 | 15.30846184 | 24.36624889 | 21.42703337 | 20.35806561 | 21.61889249 | 22.0647489 | 13.44096286 | 13.36981124 | 15.15322779 | 12.95576892 | 10.24720325 | 10.81654108 | 18.53297384 | 27.42183892 | 19.59339191 | 18.03076468 | 21.91490716 | 23.05685715 |
| ENSMUSG00000025743 | Sdc3 | 6080 | 7.277613198 | 12.72793639 | 8.648359786 | 10.06389516 | 9.800546299 | 11.27976699 | 2.684683262 | 2.305363764 | 1.944166122 | 1.768151372 | 1.932999965 | 2.570458145 | 1.176501317 | 0.725688865 | 1.416303484 | 1.058029941 | 1.178785776 | 1.0042781 |
| ENSMUSG00000025745 | Hadha | 5857 | 15.93671958 | 16.77873279 | 16.39430728 | 15.61226156 | 16.78084141 | 16.47245557 | 18.65203614 | 16.24986628 | 18.20588856 | 17.20352618 | 16.48509324 | 17.6204815 | 17.69615131 | 16.9774272 | 15.3142884 | 18.59941575 | 17.00046429 | 14.23831412 |
| ENSMUSG00000025746 | Il6 | 1082 | 0 | 0 | 0 | 0 | 0 | 0 | 0 | 0 | 0 | 0 | 0 | 0 | 0 | 0 | 0 | 0 | 0 | 0.254201048 |
| ENSMUSG00000025747 | Tyms | 8387 | 1.783930125 | 1.66822649 | 1.972546486 | 1.684025724 | 1.624100918 | 2.026539599 | 2.431353835 | 2.09928431 | 2.214751235 | 1.872085869 | 2.6430882 | 1.657100272 | 1.905751901 | 1.77757864 | 1.71939246 | 2.588622201 | 2.508779897 | 2.052921236 |
| ENSMUSG00000025754 | Agbl1 | 4573 | 0.083653309 | 0 | 0 | 0 | 0 | 0 | 0.041384368 | 0 | 0.090064544 | 0 | 0 | 0 | 0 | 0.132029988 | 0 | 0 | 0 | 0 |
| ENSMUSG00000025757 | Hspa4l | 11771 | 2.975470652 | 3.144221007 | 2.831480963 | 3.75062883 | 2.878674222 | 3.468817642 | 2.946240222 | 2.76441482 | 2.981132566 | 2.832006389 | 3.234783953 | 3.006297688 | 3.273148194 | 3.75624596 | 2.968220151 | 3.851506173 | 4.242559244 | 3.701232905 |
| ENSMUSG00000025758 | Plk4 | 6100 | 1.769887658 | 1.63833906 | 1.601877563 | 1.180105739 | 1.483410106 | 1.437841614 | 1.900263334 | 1.458824551 | 0.918256745 | 1.808731895 | 1.660375606 | 1.866622159 | 2.070323086 | 1.111612583 | 1.195520556 | 1.56301004 | 1.633579868 | 1.821613042 |
| ENSMUSG00000025759 | Mfsd8 | 3172 | 4.944643743 | 5.524143241 | 6.023816044 | 6.880119873 | 7.32904134 | 4.60846671 | 4.340475904 | 6.550021093 | 3.739507106 | 6.272886751 | 9.21761494 | 4.627843399 | 4.61903513 | 5.490710205 | 5.312558751 | 5.130361963 | 5.908436061 | 5.601494789 |
| ENSMUSG00000025762 | Larp1b | 4635 | 0.284284888 | 0.071872479 | 0.093929185 | 0.127787179 | 0.103844496 | 0.224510767 | 0.020415395 | 0.288400018 | 0.213263512 | 0.315355297 | 0.391682507 | 0.252886497 | 0.468307305 | 0.050101497 | 0.231119839 | 0.520454858 | 0.339024417 | 0.391650598 |
| ENSMUSG00000025764 | Phf17 | 5936 | 14.86535469 | 16.22993431 | 16.71396745 | 16.04921558 | 16.12776216 | 17.92279633 | 15.27938098 | 19.1605415 | 14.29316416 | 17.21286623 | 18.70434695 | 19.66148087 | 12.83992793 | 11.84574601 | 15.84621835 | 14.01064678 | 17.15515008 | 18.57113374 |
| ENSMUSG00000025766 | D3Ertd751e | 3783 | 5.730277498 | 7.599531512 | 6.70043085 | 7.129827715 | 5.852679061 | 8.252239533 | 10.89329721 | 9.46985817 | 12.06308736 | 11.21745518 | 10.68401133 | 10.19524791 | 6.676696179 | 7.63632629 | 9.529833837 | 9.625791076 | 11.59008291 | 9.45173655 |

| ENSMUSG00000025774 | Crisp4 | 3281 | 1.969151709 | 3.919164026 | 1.592301517 | 2.346789544 | 0.865523772 | 2.567499149 | 4.095337036 | 4.260414288 | 3.414426177 | 1.753241633 | 0.53876058 | 1.548072324 | 1.864418696 | 5.393232946 | 2.134795642 | 0.595190789 | 0.911140249 | 1.458638307 |
| --- | --- | --- | --- | --- | --- | --- | --- | --- | --- | --- | --- | --- | --- | --- | --- | --- | --- | --- | --- | --- |
| ENSMUSG00000025776 | Crispld1 | 4575 | 0 | 0 | 0 | 0 | 0 | 0 | 0 | 0 | 0 | 0 | 0 | 0 | 0 | 0 | 0 | 0 | 0 | 0 |
| ENSMUSG00000025777 | Gdap1 | 5402 | 0 | 0 | 0 | 0.067472854 | 0 | 0 | 0 | 0.091910631 | 0 | 0 | 0 | 0 | 0.027396474 | 0.146158735 | 0 | 0.014176456 | 0 | 0 |
| ENSMUSG00000025779 | Ly96 | 598 | 18.83590576 | 13.25830907 | 18.03895864 | 13.63783769 | 14.7293345 | 18.97587958 | 18.11806601 | 19.35166878 | 22.38397596 | 16.55793927 | 19.17389638 | 15.30730546 | 31.26554623 | 19.88241868 | 15.84675227 | 18.95321035 | 21.14996317 | 24.10097987 |
| ENSMUSG00000025780 | Itih5 | 7886 | 9.933684938 | 13.35726243 | 14.7095772 | 13.43837042 | 16.29014764 | 16.60764327 | 13.37306129 | 14.689013 | 15.37574213 | 15.03728363 | 16.15721713 | 20.75923641 | 14.82584884 | 14.6823624 | 21.06054677 | 15.3288673 | 22.80818049 | 19.2176121 |
| ENSMUSG00000025781 | Atp5c1 | 22595 | 25.18142449 | 23.81661842 | 24.10219981 | 23.09407571 | 25.25781009 | 24.28840559 | 29.65234991 | 28.19763916 | 27.14901375 | 27.6435214 | 28.49159396 | 25.14977919 | 27.73023595 | 25.25390828 | 23.67425537 | 24.30635902 | 24.31205194 | 25.39256398 |
| ENSMUSG00000025782 | Taf3 | 8292 | 5.249071425 | 5.499921875 | 4.206140302 | 4.851712753 | 4.307029349 | 5.605458767 | 5.859879554 | 5.195493236 | 4.500118582 | 5.828445591 | 5.122037878 | 5.203269076 | 3.99200633 | 5.074571053 | 4.437167078 | 4.474625973 | 5.736002708 | 5.081642034 |
| ENSMUSG00000025783 | 4930412O13R | 1376 | 0.278013506 | 1.065237897 | 0.773414003 | 1.192004067 | 0.314816361 | 0.468158093 | 0.378226358 | 0.804927086 | 1.047622131 | 0.582530491 | 0.590242946 | 1.379166081 | 1.254808942 | 1.316365849 | 2.515211261 | 1.196581481 | 1.420524086 | 1.079393809 |
| ENSMUSG00000025784 | Clec3b | 987 | 0.258390128 | 0.033751666 | 0.343074683 | 0 | 0 | 0 | 0.047935845 | 0 | 0 | 0 | 0 | 0.50895741 | 0 | 0.564669755 | 0 | 0.155179767 | 0 | 0.055733644 |
| ENSMUSG00000025785 | Exosc7 | 1898 | 20.69272005 | 19.14877112 | 18.47777108 | 18.65170255 | 20.56638045 | 18.53658632 | 21.93633164 | 21.08832335 | 20.35455837 | 18.38330896 | 22.30169295 | 19.14436056 | 23.9381959 | 19.40472144 | 21.42567738 | 19.56896163 | 19.18334144 | 21.88191548 |
| ENSMUSG00000025786 | Zdhhc3 | 11248 | 8.767070447 | 9.980836906 | 9.710831396 | 10.77862189 | 10.88616766 | 10.51145291 | 9.363266651 | 8.339308349 | 9.454443785 | 10.62232237 | 9.459000047 | 10.94181508 | 9.363766304 | 11.82573491 | 10.49819161 | 11.18965385 | 10.09949716 | 9.384999627 |
| ENSMUSG00000025787 | Tgm4 | 4004 | 0.19108221 | 0.024959711 | 0.072487808 | 0.113788822 | 0.1202096 | 0.061879038 | 0.059081767 | 0.09538562 | 0.154295139 | 0.129534899 | 0.023863616 | 0.083639854 | 0.012320659 | 0.04639769 | 0.030870297 | 0 | 0.019144004 | 0.054954153 |
| ENSMUSG00000025789 | St8sia2 | 5370 | 0 | 0.031017592 | 0 | 0 | 0.026889343 | 0 | 0 | 0 | 0 | 0 | 0.053379842 | 0 | 0 | 0 | 0 | 0 | 0 | 0.030731345 |
| ENSMUSG00000025790 | Slco3a1 | 8140 | 0.673607826 | 0.761203725 | 0.65963905 | 1.007492381 | 0.869212872 | 0.937484149 | 0.023249474 | 0.290900362 | 0.293466574 | 0.254868666 | 0.234766381 | 0.397703738 | 0.296961173 | 0.427924638 | 0.25308081 | 0.216384268 | 0.211877563 | 0.229767768 |
| ENSMUSG00000025791 | Pgm2 | 3193 | 42.66491988 | 38.90503681 | 43.55587215 | 39.39681911 | 39.32859031 | 41.18789694 | 43.47491387 | 39.06557665 | 40.74794967 | 43.94628653 | 41.70022214 | 41.27177556 | 46.33461319 | 46.67681692 | 42.72419404 | 38.65036882 | 41.31509989 | 42.86334404 |
| ENSMUSG00000025792 | Slc25a10 | 3633 | 24.40566947 | 25.84889865 | 22.08964681 | 25.45800165 | 24.01959197 | 23.51470209 | 40.12396471 | 34.27118961 | 40.6423504 | 34.35395308 | 31.27136011 | 31.57203045 | 43.66954197 | 59.48371556 | 48.86801661 | 45.78431009 | 44.76148576 | 40.74580407 |
| ENSMUSG00000025793 | Hgs | 3358 | 33.61934113 | 34.60255361 | 36.86356884 | 35.01877767 | 31.27571693 | 36.61117689 | 32.39185493 | 33.04018135 | 29.90253884 | 31.12957389 | 35.55378834 | 27.25958506 | 25.57679213 | 35.91878944 | 30.41651491 | 25.2686081 | 30.98746161 | 35.44958515 |
| ENSMUSG00000025794 | Rpl14 | 732 | 195.2799276 | 172.5262049 | 192.965449 | 163.1359251 | 177.7988 | 168.0189682 | 197.8471451 | 172.387565 | 179.7127477 | 164.3833261 | 168.3218824 | 169.1245077 | 257.5773058 | 259.1859248 | 191.7110647 | 175.0801407 | 175.2433275 | 212.3712235 |
| ENSMUSG00000025795 | Rassf3 | 3420 | 19.06518714 | 20.01695837 | 18.44417649 | 24.64559416 | 19.68905299 | 21.95099163 | 19.85195739 | 16.75105369 | 17.12491971 | 19.06708263 | 18.4953348 | 13.59486752 | 14.97266963 | 20.96774004 | 17.2757401 | 15.78647849 | 18.34507252 | 16.72791549 |
| ENSMUSG00000025804 | Ccr1 | 2829 | 0 | 0 | 0 | 0.016105 | 0 | 0 | 0 | 0 | 0 | 0 | 0.185763359 | 0 | 0.139503478 | 0 | 0 | 0.311305751 | 0 | 0 |
| ENSMUSG00000025808 | Ccdc7 | 2082 | 0 | 0 | 0 | 0 | 0 | 0 | 0 | 0 | 0 | 0 | 0 | 0 | 0 | 0 | 0 | 0 | 0 | 0 |
| ENSMUSG00000025809 | Itgb1 | 4266 | 128.880556 | 169.6568073 | 162.345015 | 159.3461748 | 168.2360792 | 178.4641816 | 110.8509672 | 134.2374312 | 113.7794393 | 126.6525806 | 139.2820344 | 136.6868793 | 89.04260737 | 95.50106296 | 112.623381 | 103.6521177 | 126.3078781 | 103.4805629 |
| ENSMUSG00000025810 | Nrp1 | 5890 | 1.392784206 | 1.357401462 | 0.878772113 | 0.874091343 | 1.601673521 | 1.01797636 | 0.578355328 | 0.622490769 | 0.524446296 | 1.00865681 | 0.55156149 | 0.113716121 | 3.911376124 | 3.343343472 | 4.316020371 | 3.497512187 | 2.993225225 | 2.764464447 |
| ENSMUSG00000025812 | Pard3 | 13443 | 4.056694253 | 4.616672017 | 4.994603893 | 6.171737093 | 5.893412658 | 5.647161187 | 3.889050823 | 5.096866021 | 5.459672029 | 5.590881573 | 4.847507503 | 4.928452837 | 4.535762088 | 3.72437151 | 4.137625564 | 4.358002628 | 4.404829927 | 3.678731455 |
| ENSMUSG00000025813 | Homer2 | 11139 | 1.587409393 | 1.082616726 | 1.16819101 | 0.965293695 | 1.235813823 | 0.840781636 | 2.616447636 | 1.947507367 | 1.615809982 | 1.811697728 | 2.045843899 | 1.458152241 | 2.214378188 | 1.892954096 | 1.368576684 | 1.887202035 | 1.954334917 | 0.800024714 |
| ENSMUSG00000025815 | Dhtkd1 | 6159 | 1.159420291 | 0.903272176 | 1.162409887 | 1.14660852 | 1.156604108 | 1.335566451 | 1.106190253 | 0.923959727 | 1.631678819 | 0.50526813 | 1.303164966 | 1.069369733 | 0.576700446 | 1.274403437 | 1.966757174 | 0.540880475 | 1.207225459 | 0.643067979 |
| ENSMUSG00000025816 | Sec61a2 | 4778 | 2.713285619 | 2.837661772 | 2.429813161 | 3.089530842 | 3.13290671 | 3.017966821 | 3.109288653 | 4.04465375 | 3.542833399 | 2.950619563 | 3.209661296 | 2.885405747 | 4.295118792 | 4.13117144 | 3.225069465 | 4.519862406 | 3.778084102 | 4.363426423 |
| ENSMUSG00000025817 | Nudt5 | 6500 | 4.342067213 | 4.089798398 | 5.001078831 | 4.037409467 | 4.783572721 | 4.543604494 | 7.264315934 | 4.829869966 | 6.50746949 | 5.019736408 | 5.262595426 | 5.031996658 | 6.716730275 | 5.337497428 | 6.402087915 | 5.543301779 | 5.796115362 | 6.440296956 |
| ENSMUSG00000025821 | Zfp282 | 6312 | 2.235696838 | 2.844684725 | 2.414078273 | 3.269827802 | 3.217945467 | 3.132373368 | 2.121275053 | 3.085888027 | 1.944483792 | 2.704143628 | 1.816538345 | 2.909276456 | 1.422434877 | 2.751915191 | 2.382536106 | 2.402262442 | 2.70202822 | 2.257186097 |
| ENSMUSG00000025823 | Pdia4 | 2470 | 93.23605009 | 97.32220433 | 109.6336125 | 94.31320635 | 87.06616799 | 105.1842033 | 100.4101469 | 116.1853084 | 104.7171332 | 91.45709284 | 94.93097013 | 108.8970356 | 98.92347622 | 105.5051718 | 116.0816305 | 83.52623185 | 96.8863959 | 102.9137984 |
| ENSMUSG00000025825 | Iscu | 2311 | 111.3668719 | 104.4505545 | 102.5033224 | 99.34318581 | 107.4481152 | 103.1365852 | 124.843235 | 114.709418 | 116.6802764 | 135.2292779 | 123.3755743 | 114.1189986 | 140.4604135 | 128.8215492 | 125.0487724 | 131.5235143 | 123.8846551 | 125.7997097 |
| ENSMUSG00000025838 | Pramel6 | 2124 | 0 | 0 | 0 | 0 | 0 | 0 | 0 | 0 | 0 | 0 | 0 | 0 | 0 | 0 | 0 | 0 | 0 | 0 |
| ENSMUSG00000025839 | Pramel7 | 2130 | 0 | 0 | 0 | 0 | 0 | 0 | 0 | 0 | 0 | 0 | 0 | 0 | 0 | 0 | 0 | 0 | 0 | 0 |
| ENSMUSG00000025854 | Fam20c | 5352 | 14.2319274 | 12.56080354 | 15.21163146 | 15.92763714 | 11.88908877 | 13.99920276 | 8.636862353 | 10.83259844 | 11.04309606 | 9.479476215 | 10.41729756 | 10.42893546 | 4.86682592 | 4.790197354 | 4.772975458 | 5.151202789 | 4.805109346 | 4.39908402 |
| ENSMUSG00000025855 | Prkar1b | 5311 | 5.570250897 | 5.896087472 | 4.463006943 | 5.601838063 | 5.700429295 | 6.792391272 | 5.647945479 | 5.551597563 | 5.8239641 | 6.170163578 | 5.136415231 | 4.519061366 | 4.300635982 | 6.147654573 | 5.158921986 | 8.327179731 | 5.989611453 | 5.717384092 |
| ENSMUSG00000025856 | Pdgfa | 3818 | 7.091617897 | 5.313659659 | 5.536729378 | 5.346083777 | 6.328503321 | 7.397867506 | 7.757395759 | 7.222345347 | 7.324684181 | 6.532928765 | 7.044866847 | 7.528828243 | 6.137417681 | 7.310868654 | 6.280593381 | 5.505904522 | 5.691726125 | 7.088653878 |
| ENSMUSG00000025857 | Heatr2 | 3500 | 7.420189301 | 12.33528883 | 10.26900944 | 11.70267967 | 10.91907068 | 11.3829708 | 8.462210574 | 9.668133866 | 10.15541803 | 11.55865811 | 10.90634052 | 9.791661951 | 8.259572435 | 7.324896068 | 10.99492949 | 8.073848075 | 10.57805795 | 11.6933644 |
| ENSMUSG00000025858 | Get4 | 2588 | 36.31334784 | 32.5920588 | 30.93438668 | 38.62478024 | 36.06174652 | 36.83904923 | 42.15727883 | 43.09189143 | 40.75682646 | 50.30258566 | 53.6083769 | 42.59502844 | 53.39207894 | 57.17575828 | 59.36653861 | 49.19484915 | 50.72162554 | 52.13962086 |
| ENSMUSG00000025860 | Xiap | 7291 | 4.523935893 | 3.883686749 | 4.624379462 | 3.455665549 | 4.066556711 | 4.009890661 | 3.919470316 | 3.761095176 | 4.925886949 | 4.171197861 | 3.531847843 | 4.133926457 | 4.776893924 | 4.510000531 | 2.633373343 | 4.222417823 | 3.947750511 | 3.342344573 |
| ENSMUSG00000025862 | Stag2 | 8508 | 8.268225943 | 8.508335583 | 8.13616854 | 9.44101095 | 8.451938641 | 10.51277434 | 7.129155429 | 8.071219972 | 8.418353422 | 8.97791952 | 7.541345703 | 7.537869806 | 6.343337741 | 4.541781936 | 7.51100296 | 7.533906534 | 7.923831247 | 6.782387512 |
| ENSMUSG00000025867 | Cplx2 | 4921 | 0.362775329 | 0.324937802 | 0.098300204 | 0.129618903 | 0.17605662 | 0.21146259 | 0.259589988 | 0.139699907 | 0.075325877 | 0.038326115 | 0.087375457 | 0.090738732 | 0.050123874 | 0.084941432 | 0 | 0 | 0.01557663 | 0 |

| ENSMUSG00000025868 | Higd2a | 646 | 227.1329221 | 197.2989679 | 201.0571656 | 190.4959454 | 206.0880818 | 192.9948571 | 201.921139 | 183.7492054 | 180.047555 | 180.8660103 | 186.4406661 | 189.1338783 | 199.5422598 | 188.2926701 | 165.8266955 | 175.7455897 | 156.6275911 | 180.4400885 |
| --- | --- | --- | --- | --- | --- | --- | --- | --- | --- | --- | --- | --- | --- | --- | --- | --- | --- | --- | --- | --- |
| ENSMUSG00000025869 | Nop16 | 2210 | 21.48338535 | 17.30461654 | 22.01980613 | 16.53391745 | 17.64111229 | 18.61030266 | 16.18478971 | 13.66976937 | 17.3877915 | 17.0041224 | 14.15954652 | 15.86074032 | 13.81740126 | 15.11008997 | 14.13157437 | 14.65785243 | 10.85622709 | 14.76036212 |
| ENSMUSG00000025870 | Arl10 | 1905 | 10.21909219 | 8.49872262 | 9.598527283 | 8.299045871 | 6.417589822 | 6.997210148 | 5.091390646 | 5.292805373 | 5.275333252 | 4.677943788 | 4.639562897 | 8.49687252 | 5.230995953 | 4.266517214 | 3.698407379 | 5.447115279 | 3.058056236 | 2.512226921 |
| ENSMUSG00000025871 | 4833439L19R | 4804 | 112.6953429 | 129.9924053 | 118.9904264 | 134.1129742 | 124.3074057 | 121.1380731 | 103.0557603 | 98.90751006 | 92.38673894 | 104.0574013 | 103.6748422 | 87.72028571 | 75.07611307 | 77.72907215 | 85.03610047 | 77.57753577 | 87.28726362 | 85.33052941 |
| ENSMUSG00000025872 | Thoc3 | 2422 | 21.1472903 | 20.06751142 | 18.57447697 | 25.41410855 | 23.80761542 | 20.76631899 | 22.56240469 | 22.97536346 | 22.39580564 | 23.69215766 | 26.68848837 | 23.29877575 | 23.64754581 | 23.10698815 | 20.22652764 | 28.6310033 | 23.24580105 | 22.2353078 |
| ENSMUSG00000025873 | Faf2 | 5091 | 25.05559483 | 24.72783878 | 26.27240452 | 25.27291086 | 24.12741489 | 24.86883408 | 21.57926546 | 23.38356282 | 23.07286249 | 25.22854364 | 24.3801497 | 20.06337901 | 21.63782583 | 24.15712893 | 22.37719545 | 23.09771269 | 25.1518676 | 22.31267047 |
| ENSMUSG00000025875 | Tspan17 | 1910 | 24.36815065 | 26.40613709 | 21.62879315 | 22.35115107 | 24.36836135 | 25.96978341 | 26.85180312 | 25.89484875 | 23.50434669 | 26.34020962 | 24.51280592 | 23.08605939 | 25.41497728 | 25.24029461 | 20.90277888 | 24.31754282 | 24.66126644 | 24.16368615 |
| ENSMUSG00000025876 | Unc5a | 3620 | 0.281802272 | 0.496932676 | 0.080177122 | 0.012585924 | 0.026592223 | 0.191640406 | 0.013069801 | 0 | 0 | 0 | 0 | 0 | 0 | 0.410555469 | 0 | 0 | 0 | 0 |
| ENSMUSG00000025877 | Hk3 | 3989 | 0 | 0.083511893 | 0 | 0 | 0 | 0 | 0 | 0 | 0 | 0 | 0 | 0 | 0.296807725 | 0 | 0 | 0 | 0 | 0 |
| ENSMUSG00000025878 | Uimc1 | 3797 | 16.97072606 | 14.55519924 | 15.38984058 | 14.9030332 | 15.66791089 | 13.49421294 | 16.54760011 | 15.07780121 | 14.07956214 | 14.88904219 | 14.84710324 | 13.21524379 | 15.73372447 | 18.82471701 | 14.98534335 | 16.94185421 | 18.59284669 | 16.80552115 |
| ENSMUSG00000025880 | Smad7 | 4744 | 2.428099221 | 3.188038359 | 2.48801463 | 2.564249333 | 2.82054696 | 3.133604569 | 0.747987125 | 0.97413168 | 0.972362935 | 0.934267729 | 0.523671552 | 0.917711147 | 4.669062154 | 4.738393401 | 5.08939961 | 7.449879981 | 3.42545318 | 5.241171214 |
| ENSMUSG00000025885 | Myo5b | 9817 | 3.533077686 | 4.109393389 | 3.587239493 | 3.935598003 | 3.427138091 | 3.492970524 | 4.149558578 | 2.777770736 | 2.890649828 | 4.000869493 | 2.627939042 | 3.758190177 | 1.331665282 | 2.994713998 | 1.792101901 | 2.453375986 | 2.904631241 | 1.910777772 |
| ENSMUSG00000025887 | Casp12 | 3878 | 0.05480296 | 0.163214283 | 0.024947669 | 0.058742966 | 0.161349925 | 0.063889548 | 0 | 0 | 0.084964447 | 0.048634042 | 0 | 0 | 0 | 0 | 0 | 0 | 0 | 0 |
| ENSMUSG00000025888 | Casp1 | 1487 | 0 | 0 | 0 | 0 | 0 | 0 | 0.127270152 | 0.154105188 | 0.083093172 | 0 | 1.124494651 | 0 | 0.464456518 | 0.312334145 | 0.166247031 | 0 | 0.154645448 | 0 |
| ENSMUSG00000025889 | Snca | 1456 | 0 | 0.068639205 | 0 | 0 | 0 | 0 | 0.064989944 | 0 | 0 | 0 | 0 | 0.345014398 | 0 | 0 | 0 | 0 | 0 | 0 |
| ENSMUSG00000025892 | Gria4 | 6446 | 0 | 0 | 0 | 0 | 0 | 0 | 0.102757913 | 0 | 0 | 0.021944168 | 0 | 0 | 0 | 0 | 0 | 0 | 0 | 0 |
| ENSMUSG00000025893 | Kbtbd3 | 2307 | 7.332925903 | 6.858961749 | 7.422726374 | 5.88521509 | 6.947520841 | 7.689587612 | 8.880099686 | 7.201428282 | 9.854771002 | 9.360651102 | 7.786469176 | 6.677556512 | 7.569787046 | 8.757337674 | 8.322464259 | 7.518701843 | 7.675227208 | 7.248707605 |
| ENSMUSG00000025894 | Aasdhppt | 3083 | 30.30372264 | 24.31203762 | 33.95404466 | 28.50705628 | 28.72615619 | 27.30784766 | 29.75649836 | 26.53523385 | 29.57734217 | 28.98170051 | 26.43661342 | 25.92540669 | 37.37896813 | 30.06889342 | 38.11444612 | 34.53979215 | 30.63120194 | 36.89874561 |
| ENSMUSG00000025898 | Cwf19l2 | 3935 | 5.865390237 | 6.611784088 | 6.798109857 | 6.402860869 | 6.482825805 | 6.170480166 | 7.899473966 | 7.124072012 | 5.620624896 | 6.158948278 | 8.09806793 | 5.730502588 | 7.584703927 | 6.63318351 | 5.727382738 | 6.840736214 | 8.210690798 | 8.751131082 |
| ENSMUSG00000025899 | Alkbh8 | 3645 | 5.329181192 | 5.099751708 | 6.144566407 | 5.512323883 | 6.17990131 | 6.865330715 | 6.347297667 | 6.737370301 | 6.395491885 | 5.49768145 | 6.986022735 | 6.048610898 | 4.953492928 | 7.708826576 | 6.296095098 | 7.300952577 | 5.089143402 | 6.806339405 |
| ENSMUSG00000025900 | Rp1 | 12436 | 1.544897038 | 1.392948295 | 0.886873988 | 1.234647149 | 1.43203697 | 1.382663119 | 1.118521036 | 0.9981128 | 0.864400178 | 1.046445328 | 1.336899771 | 0.812370129 | 1.400302895 | 1.019559011 | 1.023744031 | 0.702014996 | 1.198852484 | 1.185464828 |
| ENSMUSG00000025902 | Sox17 | 4104 | 0 | 0.008117177 | 0 | 0 | 0 | 0 | 0 | 0 | 0 | 0 | 0 | 0 | 0 | 0 | 0 | 0 | 0 | 0 |
| ENSMUSG00000025903 | Lypla1 | 3478 | 34.365887 | 28.27477393 | 32.04502988 | 29.64480832 | 34.91571116 | 37.3283962 | 39.04180234 | 36.31462759 | 40.61809921 | 38.90814214 | 36.79963018 | 39.78350418 | 33.98484008 | 30.64668791 | 32.2694187 | 38.08142927 | 43.09779965 | 33.08770882 |
| ENSMUSG00000025905 | Oprk1 | 6943 | 0 | 0 | 0 | 0 | 0 | 0 | 0 | 0 | 0 | 0 | 0 | 0 | 0 | 0 | 0 | 0 | 0 | 0 |
| ENSMUSG00000025907 | Rb1cc1 | 14517 | 4.798924262 | 4.635396178 | 5.058277818 | 6.016430529 | 5.785644885 | 5.652636688 | 4.852833156 | 5.030231687 | 4.98198813 | 6.258827938 | 5.518950566 | 6.236343922 | 5.175484605 | 4.383026777 | 4.033024104 | 5.227800776 | 5.721091506 | 5.051122082 |
| ENSMUSG00000025909 | Sntg1 | 10053 | 0.012684326 | 0.096098073 | 0.048118502 | 0.11330211 | 0.11011979 | 0.103512126 | 0.183546651 | 0.05318747 | 0.057357129 | 0.145396578 | 0.190092344 | 0.088834239 | 0.225730448 | 0.184796926 | 0.22131543 | 0.156163822 | 0.205870888 | 0.519831407 |
| ENSMUSG00000025911 | Adhfe1 | 3407 | 14.58425324 | 12.496002 | 13.06241501 | 14.0012954 | 13.30797537 | 12.85723994 | 29.28747868 | 24.68437631 | 28.77132595 | 32.07963902 | 24.73584583 | 20.75680789 | 24.28224986 | 16.60372716 | 22.8077882 | 20.80302738 | 22.91478333 | 17.32455871 |
| ENSMUSG00000025912 | Mybl1 | 6531 | 0.572723242 | 0.443763864 | 0.577727051 | 0.711564312 | 0.198983607 | 0.424889463 | 0.166619448 | 0.210523118 | 0.315315539 | 0.339317571 | 0.212138079 | 0.529868486 | 0.725136129 | 0.248896503 | 0.328047911 | 0.45730629 | 0.316891752 | 0.151609849 |
| ENSMUSG00000025915 | Sgk3 | 5835 | 10.87578883 | 11.86361511 | 12.18664775 | 12.43070824 | 12.37324516 | 12.52618372 | 10.28959547 | 11.14681428 | 11.15247556 | 11.61192126 | 11.82297173 | 11.30661751 | 10.55965119 | 15.87138136 | 12.73823596 | 15.89371913 | 16.17783535 | 13.26440671 |
| ENSMUSG00000025916 | Ppp1r42 | 1620 | 0 | 0.329016239 | 0.029860204 | 0.281241014 | 0.029711064 | 0.061176214 | 0.146026787 | 0.047151114 | 0.279661527 | 0.407475213 | 0.294907151 | 0.206724676 | 0.609036016 | 0.286691897 | 0.228897533 | 0.259997951 | 0.946328319 | 0.033956239 |
| ENSMUSG00000025917 | Cops5 | 3326 | 70.60766613 | 63.80130372 | 64.1683752 | 58.65676243 | 66.5974482 | 61.17363923 | 69.6318591 | 73.8470655 | 74.70783211 | 74.75215222 | 69.66582941 | 68.93888813 | 79.64894641 | 71.23009314 | 71.84877455 | 82.08419446 | 79.31446229 | 74.87258753 |
| ENSMUSG00000025920 | Stau2 | 7792 | 10.08079637 | 12.58211598 | 11.43532378 | 11.75863195 | 12.07004349 | 13.28486402 | 8.561457558 | 9.136375524 | 9.334623567 | 10.61374917 | 10.36186856 | 9.462588588 | 8.838213105 | 10.0374542 | 7.497921747 | 9.449799542 | 9.414339357 | 7.928032193 |
| ENSMUSG00000025921 | Rdh10 | 4154 | 1.88275214 | 0.94629791 | 1.024764244 | 0.811631507 | 1.170275467 | 1.145176318 | 1.173135756 | 0.827471405 | 1.61612953 | 1.36208098 | 0.793565752 | 1.048055292 | 1.235079296 | 1.24104326 | 1.051363728 | 0.387146247 | 0.968767736 | 0.834273886 |
| ENSMUSG00000025922 | Gm5045 | 880 | 0 | 0.113566685 | 0.109939842 | 0 | 0 | 0.056309925 | 0.268822039 | 0.260402744 | 0.187211435 | 0.160741034 | 0 | 0.063426889 | 0.056058997 | 0 | 0 | 0.043512054 | 0.08710522 | 0.062510349 |
| ENSMUSG00000025925 | Terf1 | 1957 | 6.581026232 | 6.723859589 | 7.464898399 | 6.448855024 | 5.656792255 | 7.089813695 | 8.340763531 | 6.967137281 | 4.019736592 | 5.710126046 | 7.201641672 | 8.870041423 | 7.511962876 | 7.214615517 | 8.547691207 | 9.274270776 | 6.580294205 | 10.70948884 |
| ENSMUSG00000025927 | Tfap2b | 6020 | 1.108523694 | 0.924128461 | 1.599058562 | 1.294175844 | 1.271258449 | 0.963068078 | 3.127981099 | 3.425896561 | 2.538238762 | 1.809271172 | 1.595143962 | 4.005376451 | 1.65532347 | 3.001121262 | 2.313309949 | 1.183065279 | 0.999539637 | 2.183916363 |
| ENSMUSG00000025929 | Il17a | 1168 | 0 | 0 | 0 | 0 | 0 | 0 | 0 | 0 | 0 | 0 | 0 | 0 | 0 | 0 | 0 | 0 | 0 | 0 |
| ENSMUSG00000025930 | Msc | 1715 | 0 | 0 | 0 | 0 | 0 | 0 | 0 | 0 | 0 | 0 | 0 | 0 | 0.028764966 | 0 | 0 | 0 | 0 | 0 |
| ENSMUSG00000025931 | Paqr8 | 4652 | 1.799982734 | 1.181562241 | 1.653351532 | 0.989180025 | 2.069300244 | 0.734982506 | 0.742438857 | 0.97697676 | 1.150955943 | 1.337896137 | 0.842120936 | 0.347948026 | 4.316012539 | 6.489393122 | 4.95977457 | 3.926186528 | 5.00087322 | 5.013727698 |
| ENSMUSG00000025932 | Eya1 | 5682 | 0 | 0 | 0 | 0.008018487 | 0 | 0.008721002 | 0.091594415 | 0.275587557 | 0.065237353 | 0 | 0 | 0.117878907 | 0 | 0 | 0 | 0 | 0 | 0 |
| ENSMUSG00000025933 | Tmem14a | 1573 | 11.21401656 | 9.487715582 | 10.88635076 | 9.239652348 | 9.638624256 | 10.99421743 | 14.01634353 | 12.89266731 | 15.238749 | 13.60865816 | 16.91713406 | 13.16440612 | 13.57960596 | 13.78854978 | 13.69892799 | 11.19750757 | 14.81397871 | 14.96751283 |

| ENSMUSG00000025934 | Gsta3 | 1435 | 18.09802268 | 23.53956428 | 19.78763925 | 21.01840512 | 24.48522946 | 17.50748148 | 31.25604155 | 34.06709065 | 29.47634264 | 34.40194168 | 29.99668125 | 27.92727926 | 55.65740353 | 55.18269617 | 46.42711908 | 32.12675353 | 42.35923825 | 56.35079225 |
| --- | --- | --- | --- | --- | --- | --- | --- | --- | --- | --- | --- | --- | --- | --- | --- | --- | --- | --- | --- | --- |
| ENSMUSG00000025935 | Tram1 | 2877 | 98.6321012 | 104.7208256 | 114.0485424 | 113.4354399 | 103.541354 | 112.1438472 | 119.8522086 | 107.3821144 | 111.1763926 | 116.7049558 | 108.1371323 | 105.2873135 | 95.33731667 | 110.0807202 | 117.9908304 | 117.9462505 | 124.8502101 | 118.4885069 |
| ENSMUSG00000025936 | Gm4956 | 1213 | 0.035041365 | 0.027463227 | 0 | 0 | 0 | 0.040851388 | 1.248149815 | 0.251887238 | 1.01862776 | 0.738551823 | 0.118157358 | 0.552174733 | 12.64816675 | 11.21856357 | 9.95223073 | 0.947005954 | 1.20065893 | 3.945418207 |
| ENSMUSG00000025937 | Lactb2 | 3044 | 28.38798386 | 33.64120789 | 35.54914172 | 32.50939169 | 33.37926769 | 31.51579902 | 38.11126437 | 36.0469028 | 36.61324299 | 38.47646087 | 36.55317947 | 35.51739107 | 29.62508041 | 31.69000312 | 32.59308364 | 29.28401249 | 36.04736795 | 25.01071081 |
| ENSMUSG00000025938 | Slco5a1 | 13692 | 0.040356945 | 0.19220849 | 0.286171192 | 0.242912375 | 0.172251261 | 0.126668542 | 0.076020957 | 0.025104559 | 0.162435864 | 0.010331004 | 0.268673079 | 0.004076516 | 0.043235686 | 0 | 0.02407336 | 0.022372543 | 0.008397523 | 0 |
| ENSMUSG00000025939 | Ube2w | 2790 | 45.36932409 | 40.90680129 | 42.20113723 | 37.96753695 | 41.42105689 | 39.74875188 | 44.29416393 | 38.6030734 | 50.05859317 | 45.86631134 | 42.43238248 | 41.73171045 | 44.00972835 | 41.25033998 | 43.04750374 | 45.90755619 | 52.00837279 | 43.67210444 |
| ENSMUSG00000025940 | Tmem70 | 2111 | 25.02791748 | 22.44004528 | 26.00850637 | 23.17980181 | 25.24018925 | 26.80683171 | 29.49478232 | 25.16609746 | 30.8069675 | 29.34913505 | 24.55510656 | 24.32515849 | 29.65523592 | 22.74902244 | 24.35790704 | 26.30098566 | 28.66756173 | 23.608835 |
| ENSMUSG00000025945 | Crygf | 649 | 0 | 0 | 0 | 0 | 0 | 0 | 0 | 0 | 0 | 0 | 0 | 0 | 0 | 0 | 0 | 0 | 0 | 0 |
| ENSMUSG00000025946 | Pth2r | 2568 | 0 | 0 | 0 | 0 | 0 | 0 | 0 | 0.089234585 | 0 | 0 | 0 | 0 | 0 | 0 | 0 | 0 | 0 | 0 |
| ENSMUSG00000025949 | Pikfyve | 6631 | 1.384575181 | 1.788476903 | 1.575732553 | 1.408537791 | 1.473500302 | 1.24049974 | 1.034585801 | 1.733662061 | 2.000008758 | 1.05948647 | 0.907803464 | 1.927580566 | 1.35400527 | 1.723004901 | 1.342110703 | 1.126024498 | 1.543224442 | 1.783585876 |
| ENSMUSG00000025950 | Idh1 | 3453 | 12.59275849 | 14.88612678 | 11.9077616 | 13.7883844 | 12.25252271 | 12.51375143 | 10.24902515 | 11.64685773 | 9.315571622 | 11.27902719 | 12.8949497 | 12.46275003 | 15.3581845 | 15.92522429 | 12.01562896 | 13.57303894 | 13.34150272 | 10.97633204 |
| ENSMUSG00000025952 | Crygc | 1120 | 0 | 0 | 0 | 0 | 0 | 0 | 0 | 0 | 0 | 0 | 0 | 0 | 0.969019804 | 0.663486963 | 0.11036131 | 0 | 0.342199079 | 0.245576369 |
| ENSMUSG00000025955 | Akr1cl | 3887 | 0 | 0.008570336 | 0 | 0 | 0 | 0 | 0 | 0 | 0 | 0 | 0 | 0 | 0 | 0 | 0 | 0 | 0 | 0 |
| ENSMUSG00000025956 | Mettl21a | 4657 | 6.808860064 | 6.9744625 | 7.104894731 | 5.752607702 | 7.152091728 | 7.341933899 | 5.811220173 | 6.741282967 | 8.54330561 | 5.447085753 | 5.139629418 | 7.562740625 | 6.377026884 | 5.485129496 | 5.564908451 | 7.737054233 | 7.653737625 | 4.949284028 |
| ENSMUSG00000025958 | Creb1 | 8640 | 6.021566604 | 5.960848896 | 5.526003988 | 5.826962267 | 5.503974605 | 5.913063463 | 3.7674911 | 6.599682499 | 4.929034409 | 6.013897568 | 4.04207114 | 5.852892396 | 4.145251382 | 5.020691854 | 5.317098937 | 4.555873195 | 7.106334217 | 5.246238882 |
| ENSMUSG00000025959 | Klf7 | 8208 | 1.486231179 | 1.716782926 | 1.066716495 | 0.999145709 | 1.806119943 | 1.068571375 | 1.38917588 | 1.451757986 | 1.264498291 | 1.102940427 | 1.775263442 | 1.421232149 | 1.634780884 | 2.167164409 | 1.626377206 | 1.642092325 | 1.596929038 | 1.454309961 |
| ENSMUSG00000025961 | 4933402D24R | 1755 | 0 | 0 | 0 | 0 | 0 | 0 | 0 | 0 | 0 | 0 | 0 | 0 | 0 | 0 | 0 | 0 | 0 | 0 |
| ENSMUSG00000025962 | Fastkd2 | 5449 | 6.349644575 | 7.507475519 | 6.924454703 | 7.215852682 | 6.660207459 | 8.530090677 | 8.41365129 | 6.42731382 | 6.885835173 | 7.73586506 | 7.224548685 | 7.682464111 | 6.880575726 | 6.60564649 | 7.236169754 | 5.438966807 | 7.786237965 | 6.238874648 |
| ENSMUSG00000025963 | Mdh1b | 1918 | 0 | 0.017368558 | 0 | 0 | 0 | 0 | 0 | 0 | 0 | 0 | 0 | 0 | 0 | 0 | 0 | 0 | 0 | 0 |
| ENSMUSG00000025964 | Adam23 | 10350 | 0 | 0 | 0 | 0 | 0 | 0 | 0 | 0.007380174 | 0 | 0 | 0 | 0 | 0.061962795 | 0.004487351 | 0.027865787 | 0 | 0.070357453 | 0 |
| ENSMUSG00000025967 | Eef1b2 | 2383 | 32.78410136 | 27.39961086 | 30.18524534 | 28.96558211 | 31.32715636 | 27.26128146 | 31.38957003 | 30.46737598 | 30.98926679 | 28.1163027 | 27.58637972 | 27.94296495 | 43.22494472 | 39.33032663 | 36.34312651 | 33.55047766 | 27.93654122 | 36.42650877 |
| ENSMUSG00000025968 | Ndufs1 | 4073 | 38.35171174 | 42.031663 | 43.13593475 | 41.64590426 | 44.79944086 | 42.93434736 | 39.18135675 | 38.79252854 | 41.3483091 | 45.36793667 | 45.56977994 | 39.13811253 | 36.10568264 | 38.45064146 | 38.98620093 | 40.51866386 | 39.85069172 | 40.65244568 |
| ENSMUSG00000025969 | Nrp2 | 10066 | 0 | 0.013237788 | 0 | 0.013578694 | 0.06694287 | 0.009845566 | 0.235012313 | 0.064501375 | 0.004091647 | 0.004684155 | 0 | 0.016634908 | 0.009801692 | 0.143032655 | 0.130980508 | 0 | 0 | 0 |
| ENSMUSG00000025971 | 9430016H08R | 3535 | 7.118264274 | 7.020680677 | 7.485239351 | 5.232753607 | 7.515932635 | 6.658429547 | 7.321084971 | 6.644505652 | 9.041226657 | 7.442741844 | 8.419745158 | 8.478928093 | 7.047416754 | 9.43333939 | 8.799766534 | 8.037236405 | 8.196514981 | 6.629103102 |
| ENSMUSG00000025977 | Boll | 3501 | 0 | 0 | 0.027634122 | 0 | 0 | 0 | 0.040542141 | 0 | 0 | 0.107742253 | 0 | 0 | 0 | 0 | 0 | 0 | 0 | 0.109986789 |
| ENSMUSG00000025978 | Rftn2 | 6193 | 0.679478835 | 0.419571411 | 0.374928057 | 0.4414117 | 0.606215089 | 0.216038076 | 0.099316135 | 0.129507581 | 0 | 0.068521933 | 0.115715223 | 0.315444565 | 0 | 0 | 0.113099701 | 0.074194621 | 0.154716199 | 0 |
| ENSMUSG00000025979 | Mob4 | 3836 | 38.43859636 | 39.07925547 | 38.28520286 | 36.45121091 | 41.83311614 | 39.55434573 | 38.28429494 | 43.4393774 | 45.56714622 | 38.26385277 | 41.9089508 | 44.08797123 | 31.66193439 | 39.99082916 | 39.63340491 | 38.80966673 | 42.51261557 | 40.46811762 |
| ENSMUSG00000025980 | Hspd1 | 2630 | 52.26682102 | 48.91802185 | 50.76461741 | 45.71695665 | 49.33979704 | 47.64975788 | 53.68100149 | 53.00466493 | 51.78852001 | 51.81198951 | 52.02565821 | 43.5913958 | 48.28150383 | 46.47940609 | 45.69769526 | 41.81392565 | 50.61111374 | 48.10682337 |
| ENSMUSG00000025981 | Coq10b | 1727 | 35.71222375 | 39.52409974 | 35.85299297 | 33.00339691 | 38.48881678 | 34.86194053 | 35.20370146 | 38.67892982 | 32.02865702 | 32.95361832 | 27.82953574 | 33.5152531 | 33.36402975 | 36.30545337 | 29.98862522 | 35.27524978 | 39.94634304 | 26.78787421 |
| ENSMUSG00000025982 | Sf3b1 | 6167 | 64.18853646 | 66.59877944 | 64.57909444 | 72.71888429 | 71.08570792 | 68.80493881 | 63.19353597 | 68.77976668 | 64.43449069 | 70.21761938 | 67.14213801 | 67.50011538 | 66.57049061 | 72.50157764 | 69.58232603 | 77.4753039 | 82.69953509 | 81.16229319 |
| ENSMUSG00000025983 | Ccdc150 | 5069 | 0.07546786 | 0.006571887 | 0.143145187 | 0.107858065 | 0.123439536 | 0.078205143 | 0.438685324 | 0.143155582 | 0.089376933 | 0.223242627 | 0.376997108 | 0.121122961 | 0.282230341 | 0.229059417 | 0.105665856 | 0.264385729 | 0.196583886 | 0.15192888 |
| ENSMUSG00000025986 | Slc39a10 | 5949 | 1.993434882 | 2.189501032 | 2.854111473 | 2.374167716 | 2.297775479 | 2.248989421 | 2.982392772 | 2.670707581 | 2.547762279 | 2.361894364 | 1.614181653 | 2.608296218 | 1.459475112 | 1.467723723 | 1.627561882 | 1.873015087 | 1.146760943 | 2.099019537 |
| ENSMUSG00000025991 | Cps1 | 5517 | 0 | 0 | 0 | 0 | 0 | 0.008981826 | 0 | 0 | 0 | 0 | 0 | 0 | 0 | 0 | 0 | 0 | 0 | 0 |
| ENSMUSG00000025993 | Slc40a1 | 3372 | 48.90868416 | 54.61259764 | 48.45960425 | 61.12638333 | 49.54504954 | 55.48965658 | 40.22700192 | 36.91252652 | 38.90244742 | 41.24987385 | 43.34033155 | 44.69225652 | 18.81401116 | 16.88625479 | 20.49080938 | 17.14674293 | 22.85711242 | 14.388503 |
| ENSMUSG00000025995 | Wdr75 | 4853 | 9.152670297 | 7.859728803 | 8.143658417 | 7.54813098 | 7.488069724 | 7.433420574 | 6.33695473 | 7.869854194 | 6.696097452 | 6.150091729 | 6.832025795 | 6.843255561 | 6.414267422 | 9.034250669 | 5.085458887 | 6.998509945 | 8.181752234 | 8.455964067 |
| ENSMUSG00000025997 | Ikzf2 | 9409 | 0.87187788 | 0.775377174 | 0.863721235 | 0.934560693 | 0.762212416 | 0.442388099 | 0.744210488 | 0.982310701 | 1.479545364 | 0.876965997 | 1.106912631 | 1.435581926 | 1.069583497 | 0.972418452 | 1.064085246 | 1.302263191 | 1.315697088 | 1.274522826 |
| ENSMUSG00000026000 | Lancl1 | 4596 | 12.88287864 | 13.85862788 | 13.87213077 | 12.9466327 | 12.8288961 | 11.91378821 | 12.20905944 | 13.19615644 | 14.01560284 | 11.4388673 | 11.68386713 | 11.93794524 | 13.40634567 | 9.963853387 | 11.73473383 | 12.53025969 | 13.47591292 | 12.4716034 |
| ENSMUSG00000026003 | Acadl | 1993 | 38.94352806 | 49.47625032 | 38.66484388 | 42.65775651 | 39.29284486 | 32.02404458 | 33.11650131 | 36.08444241 | 32.8996152 | 39.62740991 | 32.84078932 | 25.2052666 | 30.29717348 | 22.60449813 | 26.00680246 | 35.40872526 | 28.9610653 | 20.70086806 |
| ENSMUSG00000026004 | 1110028C15R | 8136 | 3.777193002 | 3.586786546 | 4.126257383 | 3.567156495 | 4.164807554 | 3.983221209 | 4.134625703 | 4.243600267 | 3.366400317 | 3.737979794 | 4.145663801 | 4.219104288 | 2.564823133 | 2.414681535 | 2.992885881 | 3.863887497 | 3.613110831 | 3.820076856 |
| ENSMUSG00000026005 | Rpe | 3292 | 16.02336678 | 16.39334406 | 15.22326168 | 13.53544999 | 15.64433728 | 13.93858788 | 12.1443541 | 11.1375171 | 13.97489007 | 11.95954958 | 12.30655067 | 10.10514426 | 12.99233666 | 11.90729336 | 11.9274249 | 12.93412984 | 12.72498254 | 12.66613089 |
| ENSMUSG00000026009 | Icos | 3425 | 0 | 0 | 0 | 0 | 0 | 0 | 0 | 0 | 0 | 0 | 0 | 0 | 0 | 0 | 0 | 0 | 0 | 0 |

| ENSMUSG00000026011 | Ctla4 | 2323 | 0 | 0.043021387 | 0 | 0 | 0.020719726 | 0.042662706 | 0.305505891 | 0 | 0.017729882 | 0.08118933 | 0.349623028 | 0.048054811 | 0.339780747 | 0 | 0 | 0.675813562 | 0 | 0 |
| --- | --- | --- | --- | --- | --- | --- | --- | --- | --- | --- | --- | --- | --- | --- | --- | --- | --- | --- | --- | --- |
| ENSMUSG00000026012 | Cd28 | 6115 | 0.45876396 | 0.354102719 | 0.395531728 | 0.171366152 | 0.598205429 | 0.559139594 | 0.046422907 | 0.281056109 | 0.255942371 | 0.323847839 | 0.242195211 | 0.337723551 | 0.056471532 | 0.592418449 | 0.384053751 | 0.61991335 | 0.570350453 | 0.323847562 |
| ENSMUSG00000026014 | Raph1 | 3026 | 2.373884583 | 3.269639651 | 1.566624579 | 2.680061432 | 2.54497944 | 2.210713494 | 4.065200437 | 2.612632947 | 4.57325489 | 2.633334055 | 1.957731279 | 2.213443328 | 3.17902309 | 3.131062071 | 1.974297952 | 3.948007108 | 2.938433867 | 2.036027744 |
| ENSMUSG00000026017 | Carf | 3339 | 1.1711519 | 1.336905608 | 0.811296107 | 1.228060494 | 0.850489217 | 0.920116646 | 1.714535535 | 1.486975835 | 0.678424189 | 0.564848197 | 0.972955129 | 1.002976866 | 0.709174013 | 0.751117316 | 1.098214578 | 0.688061229 | 0.82644306 | 1.268553824 |
| ENSMUSG00000026018 | Ica1l | 3637 | 0.093495026 | 0 | 0.013300393 | 0.037581285 | 0 | 0 | 0.065043551 | 0.136513949 | 0 | 0.142605921 | 0.013135815 | 0 | 0 | 0.051079557 | 0 | 0 | 0 | 0.045374572 |
| ENSMUSG00000026019 | Wdr12 | 4858 | 5.302210101 | 5.170424504 | 5.924711931 | 4.089052147 | 4.924159338 | 5.344922272 | 5.151998179 | 4.158868026 | 5.578577063 | 5.745824687 | 5.172834121 | 5.89407882 | 5.534354655 | 4.894889408 | 6.072522441 | 5.391267078 | 5.625082275 | 4.744507146 |
| ENSMUSG00000026020 | Nop58 | 2572 | 22.31025958 | 17.14862828 | 15.53520065 | 15.96053692 | 14.50320406 | 14.02581261 | 16.77650201 | 18.97740679 | 15.21274882 | 18.69895697 | 15.04576842 | 14.90877146 | 18.50905916 | 18.52707374 | 13.82462781 | 18.05851741 | 14.72254329 | 14.47945772 |
| ENSMUSG00000026021 | Sumo1 | 1731 | 63.45082314 | 54.34755244 | 55.22015943 | 57.37901597 | 61.8680128 | 56.68076978 | 57.86305101 | 59.37363078 | 60.41164848 | 65.37359204 | 56.60683988 | 59.04013761 | 61.50102685 | 64.63535905 | 65.57497775 | 68.90539691 | 68.34966987 | 64.79697782 |
| ENSMUSG00000026023 | Cdk15 | 2682 | 0 | 0 | 0 | 0 | 0 | 0 | 0 | 0 | 0 | 0 | 0 | 0 | 0 | 0 | 0 | 0 | 0 | 0 |
| ENSMUSG00000026024 | Als2 | 6990 | 1.422920056 | 1.59654071 | 1.695495699 | 1.903265371 | 1.569968326 | 1.963677711 | 1.556783427 | 1.010814656 | 1.219686518 | 1.740326386 | 1.592498616 | 1.381417686 | 1.517362262 | 1.561424969 | 1.815461976 | 1.703630744 | 0.904697996 | 1.329976972 |
| ENSMUSG00000026027 | Stradb | 5966 | 9.197818011 | 9.877725423 | 8.724424851 | 9.859086195 | 8.65664668 | 9.701239158 | 8.604468092 | 8.751091869 | 9.506173685 | 9.697270013 | 9.128972958 | 8.878488735 | 10.16240803 | 8.656692118 | 7.589760296 | 7.823709424 | 8.621168361 | 7.044411253 |
| ENSMUSG00000026028 | Trak2 | 7749 | 5.205499039 | 4.982532507 | 5.193802717 | 4.709562074 | 5.528121314 | 4.789649948 | 5.01884399 | 4.494963349 | 4.315840857 | 5.421509434 | 5.869371593 | 6.136913734 | 3.762442007 | 3.979723064 | 3.636838848 | 4.279218739 | 5.435617537 | 5.317050063 |
| ENSMUSG00000026029 | Casp8 | 2767 | 26.42172127 | 24.15094536 | 22.95426287 | 26.36184748 | 23.10054016 | 27.05969658 | 20.87776689 | 22.15352579 | 18.32331077 | 22.16952112 | 20.23572509 | 24.77109998 | 18.50615473 | 20.98124656 | 22.67797962 | 22.08594485 | 22.59132282 | 16.56038522 |
| ENSMUSG00000026031 | Cflar | 9545 | 4.257197306 | 5.158350724 | 4.338160501 | 4.424838984 | 4.039148335 | 4.101273916 | 3.786997035 | 3.56915903 | 3.214662571 | 3.576438885 | 3.924103452 | 3.315608244 | 4.470624248 | 5.50808873 | 3.14245497 | 3.205258807 | 4.019342399 | 5.094609779 |
| ENSMUSG00000026032 | Ndufb3 | 760 | 124.9987742 | 107.4340839 | 126.0894258 | 111.5046611 | 122.166422 | 108.5596071 | 156.6921222 | 133.1206236 | 129.8459102 | 126.9966968 | 133.8327455 | 122.6475744 | 147.6711997 | 156.565463 | 135.5856464 | 126.1574749 | 118.458515 | 131.0809109 |
| ENSMUSG00000026034 | Clk1 | 6474 | 33.32001365 | 46.66069274 | 36.67989813 | 31.89413854 | 49.01664701 | 37.6965111 | 43.74632315 | 51.17097597 | 47.70103417 | 29.40182098 | 49.91501686 | 40.56420953 | 50.90163846 | 50.46866771 | 50.2832087 | 50.47452017 | 67.12133987 | 56.41109971 |
| ENSMUSG00000026035 | Ppil3 | 2458 | 8.940266886 | 6.437601609 | 9.426737608 | 7.766508371 | 7.676043156 | 8.144550189 | 9.720464955 | 10.25507957 | 11.997374 | 9.111710353 | 8.901924729 | 9.8097503 | 12.56378365 | 12.20621662 | 10.69430133 | 10.39049436 | 8.653822124 | 8.795190782 |
| ENSMUSG00000026036 | Nif3l1 | 4179 | 10.33387386 | 7.851926491 | 10.84613495 | 8.351222771 | 9.110397612 | 8.549299093 | 8.740222094 | 8.581638157 | 6.524401396 | 8.349251115 | 7.522355271 | 8.240790576 | 7.401558299 | 10.41352235 | 8.173268685 | 9.180949657 | 9.134479951 | 6.028755894 |
| ENSMUSG00000026037 | Orc2 | 3925 | 10.95929634 | 12.68860557 | 9.440541211 | 13.15176133 | 10.86493869 | 11.56440865 | 12.52429896 | 11.33608887 | 12.72846971 | 11.89278885 | 12.0502443 | 10.65119268 | 12.61891591 | 9.655637022 | 11.60991613 | 12.72126167 | 13.52405636 | 14.54763128 |
| ENSMUSG00000026039 | Sgol2 | 4930 | 0.146569573 | 0.060814614 | 0 | 0.221798187 | 0.048815338 | 0.130666437 | 0.067178246 | 0.147191815 | 0.183793782 | 0.114768446 | 0 | 0.226432708 | 0.070045319 | 0.018841415 | 0.167146271 | 0.023300572 | 0.101063258 | 0 |
| ENSMUSG00000026042 | Col5a2 | 8545 | 0 | 0 | 0 | 0 | 0 | 0 | 0 | 0 | 0 | 0 | 0 | 0 | 0 | 0 | 0 | 0 | 0 | 0 |
| ENSMUSG00000026043 | Col3a1 | 7122 | 0 | 0.037419707 | 0 | 0 | 0 | 0 | 0 | 0 | 0 | 0 | 0 | 0 | 0 | 0.013042428 | 0 | 0.043011073 | 0.021525581 | 0 |
| ENSMUSG00000026047 | Kdelc1 | 3550 | 10.76398683 | 8.42675465 | 10.7648138 | 10.01059566 | 7.877365535 | 7.830727757 | 7.290150809 | 10.26353574 | 9.420690366 | 6.813608668 | 9.554991696 | 9.795255716 | 7.406735749 | 10.51860458 | 10.44546487 | 8.477864061 | 10.33190595 | 7.871725694 |
| ENSMUSG00000026048 | Ercc5 | 4771 | 5.336533314 | 5.571932422 | 6.458591246 | 5.576954494 | 5.367047452 | 6.013630841 | 5.206278859 | 5.571559856 | 7.665819744 | 6.591808651 | 6.348632085 | 5.182632263 | 3.629323614 | 4.322191323 | 5.708285149 | 4.662930811 | 4.779741492 | 6.998643425 |
| ENSMUSG00000026049 | 1700029F09R | 2364 | 5.034454013 | 4.185249401 | 4.03112753 | 3.546206497 | 3.542705042 | 4.401892577 | 5.403732365 | 5.961504436 | 6.620505918 | 4.986326487 | 6.264905723 | 6.44571738 | 5.488280137 | 5.500991738 | 4.339757791 | 4.907806279 | 3.890994609 | 5.282177338 |
| ENSMUSG00000026051 | 1500015O10R | 898 | 0 | 0 | 0 | 0 | 0 | 0.331087307 | 0 | 0 | 0 | 0 | 0.106403026 | 0.062155526 | 0.05493532 | 0.103438947 | 0 | 0 | 0 | 0 |
| ENSMUSG00000026058 | Khdrbs2 | 1350 | 0 | 0 | 0 | 0 | 0 | 0 | 0 | 0 | 0 | 0 | 0 | 0 | 0 | 0 | 0 | 0 | 0 | 0 |
| ENSMUSG00000026062 | Slc9a2 | 3481 | 33.62805366 | 36.21258021 | 40.88334568 | 45.99296461 | 34.51229 | 50.1079064 | 28.88234494 | 24.52169474 | 31.25963075 | 31.1403237 | 26.65296448 | 27.32257657 | 21.32850776 | 15.33015122 | 21.16299395 | 15.6638394 | 19.11360282 | 17.58837569 |
| ENSMUSG00000026063 | 4930521A18R | 1448 | 0 | 0 | 0 | 0 | 0 | 0 | 0 | 0 | 0 | 0 | 0 | 0 | 0 | 0 | 0 | 0 | 0 | 0 |
| ENSMUSG00000026064 | Ptp4a1 | 7131 | 9.686006317 | 9.889692478 | 9.395223607 | 9.871240148 | 9.382046543 | 10.95149462 | 8.611955862 | 9.501237115 | 8.680876902 | 9.197395629 | 9.218670995 | 8.813411313 | 8.024824574 | 9.645728989 | 9.458273313 | 9.439754286 | 12.50670213 | 11.11598973 |
| ENSMUSG00000026065 | Slc9a4 | 3810 | 15.90876142 | 15.10009667 | 18.39717729 | 18.73862375 | 14.59117371 | 21.31677962 | 9.785404461 | 10.30493167 | 8.529176099 | 10.81619807 | 9.266586435 | 9.463758013 | 3.716078808 | 1.79193723 | 3.784919832 | 3.487359782 | 3.661619967 | 4.028225925 |
| ENSMUSG00000026068 | Il18rap | 6810 | 0 | 0.034242329 | 0.049723159 | 0 | 0 | 0.021829398 | 0 | 0 | 0 | 0 | 0 | 0 | 0.007244041 | 0 | 0 | 0 | 0 | 0 |
| ENSMUSG00000026069 | Il1rl1 | 6881 | 0.02470872 | 0.140397316 | 0.126540263 | 0 | 0.25881139 | 0.122423553 | 0.116889339 | 0.255319069 | 0 | 0 | 0.034715128 | 0 | 0 | 0 | 0 | 0 | 0 | 0 |
| ENSMUSG00000026070 | Il18r1 | 4906 | 5.016407852 | 4.135253278 | 3.441166346 | 6.129288476 | 5.15068486 | 4.423990483 | 1.369425277 | 0.926395411 | 1.334825929 | 1.374347853 | 1.158830016 | 2.013732629 | 1.176484778 | 1.287483874 | 1.318516974 | 0.577559101 | 1.453055692 | 1.300663755 |
| ENSMUSG00000026072 | Il1r1 | 5044 | 17.87340967 | 17.49521348 | 19.17103235 | 19.18549924 | 13.68381811 | 19.38293881 | 8.563932566 | 9.949408557 | 10.21497447 | 10.9370188 | 10.96816057 | 12.02847447 | 7.794912385 | 9.806295216 | 9.426367069 | 10.19513992 | 13.11482721 | 9.302689936 |
| ENSMUSG00000026073 | Il1r2 | 1355 | 0 | 0 | 0.035700022 | 0 | 0 | 0.146281132 | 0 | 0.366421573 | 0.303959526 | 0 | 0.035258272 | 0.535500822 | 0 | 0 | 0.030407052 | 0 | 0 | 0 |
| ENSMUSG00000026074 | Map4k4 | 6152 | 4.55313898 | 4.115377049 | 3.129496925 | 3.110474418 | 3.270342018 | 3.197730044 | 2.222588461 | 2.607413688 | 2.731485441 | 2.797465327 | 2.182178695 | 2.99401311 | 3.239612253 | 3.389693634 | 3.094135049 | 3.572628194 | 2.828371065 | 2.360598858 |
| ENSMUSG00000026077 | Npas2 | 4766 | 6.465852417 | 4.172848898 | 6.363869814 | 7.112340954 | 5.604955442 | 5.541671632 | 7.028404675 | 8.935066866 | 6.550436204 | 10.18993377 | 7.187294425 | 10.36442748 | 8.187483543 | 4.979632534 | 7.408672555 | 7.070024027 | 7.494777321 | 10.37624569 |
| ENSMUSG00000026078 | Pdcl3 | 1700 | 67.78325424 | 58.86584367 | 54.178354 | 54.91445873 | 62.42993629 | 63.01941764 | 61.31166569 | 58.29957897 | 64.29941931 | 64.09722072 | 47.83116431 | 55.58583339 | 52.46594496 | 60.70515422 | 57.75488688 | 67.48156457 | 60.2173759 | 52.58223437 |
| ENSMUSG00000026080 | Chst10 | 4695 | 0.126745999 | 0.035476991 | 0.051516007 | 0.213491582 | 0.102517409 | 0.021108726 | 2.499157481 | 1.46424546 | 1.587808169 | 1.024360326 | 0.59019118 | 1.462263365 | 5.23265065 | 5.885885409 | 3.141673484 | 0.481181222 | 0.873464061 | 2.69480182 |
| ENSMUSG00000026082 | Rev1 | 4238 | 4.543380071 | 4.244682133 | 5.410347661 | 5.04203157 | 5.462825692 | 5.109614104 | 4.353927509 | 4.88444599 | 4.412146804 | 4.650541284 | 5.230670301 | 5.070559249 | 3.270945907 | 3.923308939 | 4.676250208 | 4.174200241 | 4.223308318 | 4.802588364 |

| ENSMUSG00000026083 | Eif5b | 7773 | 13.17862784 | 12.68144269 | 13.54185026 | 10.89643399 | 11.56701832 | 12.70533874 | 11.376225 | 10.48043154 | 11.53519167 | 10.33032904 | 11.1063103 | 11.65429311 | 9.741990611 | 13.03159071 | 11.12065667 | 10.69457207 | 10.61085693 | 12.95081247 |
| --- | --- | --- | --- | --- | --- | --- | --- | --- | --- | --- | --- | --- | --- | --- | --- | --- | --- | --- | --- | --- |
| ENSMUSG00000026085 | Lyg1 | 1146 | 0 | 0 | 0 | 0 | 0 | 0 | 0 | 0 | 0 | 0 | 0 | 0 | 0 | 0.081054254 | 0 | 0 | 0.13377416 | 0 |
| ENSMUSG00000026087 | Mrpl30 | 2013 | 37.09964942 | 34.30582698 | 36.91095014 | 29.71766081 | 35.60279898 | 36.75222615 | 48.74639647 | 38.15445665 | 42.02538667 | 46.33089471 | 38.5664717 | 38.51363901 | 53.52255704 | 58.39542235 | 48.77461882 | 41.27700848 | 39.67809376 | 50.39085584 |
| ENSMUSG00000026088 | Mitd1 | 2920 | 6.75424715 | 6.662578844 | 6.60994473 | 5.461084081 | 6.247259955 | 6.36379284 | 6.027505672 | 5.101040047 | 7.207640258 | 4.989577845 | 7.068076049 | 6.097669988 | 7.670099467 | 6.966613108 | 8.268531422 | 9.651331181 | 8.794047577 | 9.325173914 |
| ENSMUSG00000026090 | 2010300C02R | 5783 | 0.345451024 | 0.253461412 | 0.585534692 | 0.212718001 | 0.224721068 | 0.428434494 | 1.538091585 | 1.882212465 | 0.313367922 | 0.391359806 | 0.090874035 | 1.56357208 | 0.017061012 | 1.027986026 | 0.220862568 | 0.145667191 | 0.033137037 | 0.380488374 |
| ENSMUSG00000026094 | Stk17b | 3285 | 3.040705134 | 3.326218966 | 3.121822964 | 3.162227735 | 3.531139605 | 3.5750374 | 7.230126278 | 6.778134125 | 8.651048973 | 8.095280559 | 9.525904963 | 7.696954384 | 12.46438093 | 16.18827399 | 12.25385696 | 13.20647129 | 13.45212187 | 13.1954874 |
| ENSMUSG00000026095 | Asnsd1 | 4607 | 25.96257116 | 20.08029243 | 23.76151491 | 24.57547106 | 22.16972911 | 23.93202349 | 27.20453364 | 25.6080597 | 29.86849341 | 28.69775818 | 24.02747532 | 24.9213844 | 29.74692125 | 22.63229351 | 30.67534984 | 26.12272174 | 30.09866338 | 24.82394896 |
| ENSMUSG00000026096 | Osgepl1 | 4704 | 5.981808361 | 7.025168169 | 7.280709926 | 5.976012831 | 6.159740231 | 6.341570071 | 6.447157141 | 6.511544798 | 6.619261462 | 7.327203249 | 7.840618186 | 6.54979714 | 7.80249712 | 8.017134133 | 9.188017034 | 7.049248727 | 7.593560526 | 6.759197212 |
| ENSMUSG00000026097 | Ormdl1 | 4707 | 10.28540376 | 9.327893474 | 8.889548281 | 9.001863444 | 7.628301478 | 8.369327214 | 8.573978144 | 8.324921366 | 11.14757193 | 8.825105071 | 8.028466572 | 8.395472511 | 10.86832339 | 9.11713124 | 9.611070399 | 10.60780796 | 11.96933428 | 8.659814761 |
| ENSMUSG00000026098 | Pms1 | 5772 | 1.649542521 | 1.644867438 | 1.097874649 | 1.041936565 | 1.642756231 | 1.244827853 | 1.508235087 | 2.143856268 | 2.040773304 | 1.887008394 | 1.109120658 | 1.605232153 | 1.41021593 | 2.373701625 | 1.848787764 | 2.063128708 | 1.739689846 | 1.74405172 |
| ENSMUSG00000026100 | Mstn | 2679 | 0 | 0 | 0 | 0 | 0 | 0 | 0 | 0 | 0 | 0 | 0 | 0 | 0 | 0 | 0 | 0 | 0 | 0 |
| ENSMUSG00000026102 | Inpp1 | 2518 | 4.659026443 | 3.307475598 | 3.669318625 | 3.546451426 | 4.300906603 | 3.778445134 | 2.893626908 | 4.535158189 | 5.741249814 | 4.774991791 | 4.686423647 | 3.502333077 | 4.40813399 | 2.914283482 | 3.91071162 | 4.805334368 | 4.825034201 | 3.014796159 |
| ENSMUSG00000026103 | Gls | 13846 | 6.198031951 | 7.646134467 | 7.584785093 | 7.387299186 | 6.931608825 | 6.971596491 | 3.755354192 | 5.792578727 | 5.027098919 | 4.597244648 | 4.878939137 | 5.696051656 | 2.864571825 | 3.508631764 | 4.597458028 | 4.433037965 | 3.742391551 | 3.4326064 |
| ENSMUSG00000026104 | Stat1 | 5219 | 12.44451216 | 7.755349009 | 7.952575023 | 10.38851174 | 9.738898523 | 14.94462592 | 8.322099877 | 6.373938014 | 6.455368824 | 8.293609042 | 7.43308417 | 8.363258889 | 7.32558649 | 5.908962258 | 7.665589341 | 8.642716137 | 7.182049889 | 10.64556386 |
| ENSMUSG00000026107 | Obfc2a | 2822 | 2.078566368 | 2.69147409 | 3.102625441 | 4.262266373 | 3.240632706 | 2.423202422 | 2.933989657 | 5.900739705 | 3.721673111 | 2.806987295 | 4.418591128 | 3.065707903 | 2.971802246 | 6.714807815 | 4.453038464 | 3.826347019 | 3.368150826 | 3.274815709 |
| ENSMUSG00000026109 | Tmeff2 | 5636 | 0 | 0 | 0 | 0 | 0 | 0 | 0 | 0 | 0 | 0 | 0 | 0 | 0 | 0.082406117 | 0 | 0 | 0.020400797 | 0 |
| ENSMUSG00000026110 | Mgat4a | 8324 | 0.025531701 | 0 | 0.133660644 | 0.021893822 | 0.034693842 | 0.005952995 | 0 | 0 | 0.044531312 | 0 | 0.212358657 | 0.087170064 | 0.011852935 | 0 | 0 | 0.00920005 | 0.013812937 | 0 |
| ENSMUSG00000026111 | Unc50 | 1792 | 77.39642264 | 75.10273027 | 71.58850586 | 70.65521329 | 77.24743999 | 74.55031793 | 68.59282358 | 75.06341589 | 70.58247205 | 68.96316587 | 72.56888224 | 77.36947872 | 76.91594691 | 63.52369319 | 59.54912375 | 75.49147095 | 70.40746061 | 66.06004334 |
| ENSMUSG00000026112 | 6330578E17R | 3860 | 30.86580529 | 35.71211303 | 33.69855521 | 38.41999982 | 37.8571296 | 38.17871235 | 37.87465748 | 34.48200062 | 41.70934977 | 35.4485339 | 39.9527373 | 34.08225822 | 36.32158262 | 34.26755463 | 30.58094757 | 29.35282573 | 41.17594644 | 37.77957045 |
| ENSMUSG00000026113 | Inpp4a | 8036 | 2.940875793 | 2.802329081 | 2.973683922 | 3.260029927 | 3.180444433 | 2.966011057 | 3.332376341 | 3.702324723 | 2.783011207 | 3.150812301 | 3.287649582 | 3.514525295 | 2.185435857 | 1.508450325 | 1.230509384 | 1.896423811 | 1.540492023 | 2.471165695 |
| ENSMUSG00000026114 | Cnga3 | 3709 | 0 | 0 | 0 | 0 | 0 | 0 | 0 | 0 | 0 | 0 | 0 | 0 | 0 | 0 | 0 | 0 | 0 | 0 |
| ENSMUSG00000026115 | Vwa3b | 1688 | 0 | 0 | 0 | 0 | 0 | 0 | 0 | 0 | 0 | 0 | 0 | 0 | 0 | 0 | 0 | 0 | 0 | 0 |
| ENSMUSG00000026116 | Tmem131 | 6505 | 8.814678318 | 8.946598955 | 7.793306658 | 9.462409054 | 8.982652626 | 10.27619333 | 7.673309188 | 8.988865193 | 6.192223277 | 7.328110837 | 8.144877625 | 7.276200134 | 8.023546269 | 7.996522348 | 7.891950598 | 6.692762585 | 9.338536601 | 6.773604072 |
| ENSMUSG00000026117 | Zap70 | 2315 | 0 | 0.215850287 | 0.146269854 | 0 | 0 | 0.064215206 | 0 | 0 | 0.195702667 | 0 | 0.082548524 | 0 | 0 | 0 | 0.017797648 | 0.033080438 | 0 | 0 |
| ENSMUSG00000026121 | Sema4c | 4574 | 9.748126291 | 13.03674697 | 11.48527634 | 12.55070307 | 11.60680188 | 11.87358898 | 8.275064089 | 11.62304857 | 11.90392957 | 13.40094321 | 10.63290506 | 13.27665957 | 9.501840647 | 6.031435922 | 9.845496412 | 7.64305303 | 8.00210178 | 5.832841443 |
| ENSMUSG00000026123 | Plekhb2 | 6040 | 20.42218888 | 26.15944657 | 22.8092408 | 27.11033002 | 24.37674743 | 25.22759201 | 16.24610863 | 16.78189337 | 20.47051661 | 22.43561609 | 13.4624138 | 18.01071629 | 15.46931313 | 13.87940029 | 15.28004722 | 17.24345234 | 18.30651764 | 13.03278671 |
| ENSMUSG00000026124 | Cfc1 | 1032 | 0 | 0 | 0 | 0 | 0 | 0 | 0 | 0 | 0 | 0 | 0 | 0 | 0 | 0 | 0 | 0 | 0 | 0 |
| ENSMUSG00000026125 | Prss39 | 1241 | 0 | 0 | 0 | 0 | 0 | 0 | 0 | 0 | 0 | 0 | 0 | 0 | 0 | 0 | 0 | 0 | 0 | 0 |
| ENSMUSG00000026126 | Ptpn18 | 1564 | 1.820873909 | 3.961763635 | 1.917620769 | 3.087896867 | 1.692618799 | 3.231700016 | 1.210042939 | 0.805849923 | 2.4754044 | 0.241180068 | 1.282959243 | 2.747957813 | 1.766360209 | 0.772088409 | 0.342468176 | 0.269307341 | 0.563622013 | 0.492408884 |
| ENSMUSG00000026127 | Imp4 | 2518 | 19.07499957 | 17.29148242 | 16.69443919 | 18.11223407 | 18.96221933 | 16.82588849 | 19.01526254 | 18.11029725 | 17.48545884 | 21.21594392 | 19.77025684 | 18.88599862 | 21.84475288 | 23.18515403 | 21.28801597 | 19.41902528 | 22.35954335 | 19.1810944 |
| ENSMUSG00000026131 | Dst | 29746 | 3.452313094 | 4.439330084 | 3.475231438 | 3.499865068 | 3.491854074 | 3.983076245 | 3.446734863 | 4.153581046 | 4.139974522 | 3.628318421 | 3.49004857 | 3.833503619 | 3.980252856 | 3.058695865 | 4.138716096 | 4.425573464 | 4.387179485 | 4.077693818 |
| ENSMUSG00000026134 | Prim2 | 2154 | 2.82183852 | 3.835467858 | 4.087271367 | 2.390156922 | 4.35734685 | 3.427742481 | 3.18492964 | 2.251826883 | 1.587038444 | 3.589932839 | 3.482204495 | 2.332130749 | 3.046028318 | 1.983684327 | 3.825585505 | 3.697514549 | 3.754340133 | 4.035022684 |
| ENSMUSG00000026135 | Zfp142 | 11572 | 0.727274875 | 0.739838733 | 1.099398417 | 0.87011673 | 0.711247749 | 0.993452661 | 1.013959936 | 0.996725331 | 0.946734635 | 0.766015573 | 0.920654661 | 0.491980434 | 0.976236524 | 0.377267907 | 0.704969587 | 0.780900739 | 1.265178484 | 0.803364936 |
| ENSMUSG00000026141 | Col19a1 | 7657 | 0 | 0 | 0 | 0 | 0 | 0 | 0 | 0 | 0.010757873 | 0 | 0 | 0 | 0 | 0 | 0.010761801 | 0 | 0 | 0 |
| ENSMUSG00000026142 | Rhbdd1 | 3876 | 13.81747208 | 14.92892086 | 15.41313467 | 17.13828758 | 15.78319788 | 16.2746723 | 9.582160206 | 12.20856207 | 11.21046804 | 14.11114958 | 13.86657077 | 13.26270001 | 12.16752139 | 8.160067986 | 13.29802539 | 13.54396872 | 12.70621556 | 11.31121209 |
| ENSMUSG00000026147 | Col9a1 | 5229 | 0 | 0 | 0 | 0 | 0 | 0 | 0 | 0 | 0 | 0 | 0 | 0 | 0 | 0 | 0 | 0 | 0 | 0 |
| ENSMUSG00000026149 | Tm4sf20 | 1813 | 0.164112649 | 0 | 0.026681484 | 0 | 0 | 0 | 0 | 0 | 0.022717328 | 0 | 0 | 0 | 0.136050516 | 0 | 0.045451248 | 0 | 0.063419134 | 0 |
| ENSMUSG00000026150 | Mff | 3024 | 53.80615537 | 57.40525522 | 55.52398276 | 54.39000332 | 55.66049506 | 58.58168735 | 53.88388433 | 51.55469134 | 51.07454832 | 48.47603718 | 52.05637837 | 53.4346373 | 54.74798756 | 52.91001358 | 47.22374098 | 50.53499145 | 54.15617284 | 51.37093829 |
| ENSMUSG00000026153 | Fam135a | 6446 | 7.391917829 | 7.364392532 | 7.797098672 | 7.195337129 | 7.272803855 | 7.472115581 | 5.886560425 | 6.493774905 | 5.143522369 | 7.197687249 | 6.055249934 | 5.411850623 | 6.818916893 | 4.50318874 | 4.979213782 | 7.199537104 | 8.080272648 | 6.886805635 |
| ENSMUSG00000026154 | 1110058L19R | 871 | 67.14939405 | 62.83936303 | 58.20374261 | 56.07513149 | 58.24460109 | 63.54810954 | 74.14673563 | 75.20088418 | 64.68789158 | 74.38009905 | 74.98090498 | 67.15822548 | 74.13947154 | 68.35972449 | 77.10509311 | 74.5150167 | 71.37227736 | 73.64020487 |
| ENSMUSG00000026155 | Smap1 | 3560 | 15.98719964 | 14.12054982 | 15.31375526 | 13.01561295 | 13.91228917 | 13.44605074 | 15.03107861 | 13.13132038 | 14.60038845 | 13.36378078 | 13.37967236 | 13.56195172 | 15.76958479 | 13.34615208 | 15.18439363 | 13.62758415 | 13.59722275 | 15.7610362 |

| ENSMUSG00000026156 | B3gat2 | 6532 | 0.14315889 | 0.214198034 | 0.274008056 | 0.292952214 | 0.272639494 | 0.098619954 | 0.31870145 | 0.20464392 | 0.353099339 | 0.223770943 | 0.248675534 | 0.290528556 | 0.347407868 | 0.312850558 | 0.586611252 | 0.287238176 | 0.088012011 | 0.126322199 |
| --- | --- | --- | --- | --- | --- | --- | --- | --- | --- | --- | --- | --- | --- | --- | --- | --- | --- | --- | --- | --- |
| ENSMUSG00000026158 | Ogfrl1 | 4837 | 13.52397476 | 16.00561633 | 14.39105028 | 13.52608221 | 13.52311025 | 14.49599297 | 12.01157116 | 11.69380772 | 11.39292084 | 9.874645938 | 11.00295715 | 10.61616903 | 9.56653678 | 8.38240403 | 9.386833697 | 9.309438558 | 9.547899063 | 10.94040948 |
| ENSMUSG00000026159 | Agfg1 | 3130 | 22.56984107 | 22.45693508 | 19.51941496 | 22.16915991 | 20.88279627 | 19.9160827 | 18.59252239 | 15.74063869 | 12.96125177 | 17.95643396 | 16.86623934 | 15.40726277 | 18.39308226 | 12.73131852 | 19.90311582 | 14.82690612 | 20.69375136 | 14.42890627 |
| ENSMUSG00000026162 | Nhej1 | 1122 | 4.318707725 | 2.137725832 | 3.061070103 | 3.289166302 | 4.032442803 | 3.533171735 | 4.469825282 | 4.833619556 | 4.808764318 | 3.361903976 | 4.513498751 | 4.427445606 | 2.989813168 | 3.559885486 | 3.561988344 | 2.55953258 | 2.186170233 | 1.863053525 |
| ENSMUSG00000026163 | Sphkap | 11441 | 0 | 0 | 0 | 0 | 0 | 0 | 0 | 0 | 0 | 0 | 0 | 0 | 0 | 0 | 0 | 0 | 0 | 0 |
| ENSMUSG00000026166 | Ccl20 | 1546 | 0 | 0 | 0 | 0 | 0 | 0 | 0 | 0 | 0 | 0 | 0 | 0 | 0 | 0 | 0 | 0 | 0 | 0 |
| ENSMUSG00000026167 | Wnt10a | 2464 | 23.15014052 | 24.21403959 | 25.22726725 | 23.79750976 | 29.76990733 | 22.72507668 | 1.478521217 | 1.131511922 | 3.15919297 | 1.473459477 | 1.318464763 | 2.197288665 | 0.500526758 | 0.245037799 | 0.451478088 | 0.124320154 | 0.124436029 | 0.022325124 |
| ENSMUSG00000026170 | Cyp27a1 | 1881 | 0 | 0.035420409 | 0 | 0 | 0.435004095 | 0.210750595 | 0.025152939 | 1.258867065 | 1.313764458 | 0.576537041 | 0.609568848 | 1.216609339 | 3.173398187 | 2.271587475 | 1.708517469 | 3.155260046 | 1.79304313 | 1.666942628 |
| ENSMUSG00000026171 | Rnf25 | 2767 | 18.90996447 | 15.26590165 | 13.1466913 | 14.01244985 | 14.68136739 | 15.29383248 | 11.79824664 | 15.23831307 | 13.15825079 | 14.60359693 | 14.71061244 | 12.36537809 | 14.85127831 | 15.72754242 | 15.56039968 | 14.82083142 | 14.01742409 | 15.36756035 |
| ENSMUSG00000026172 | Bcs1l | 2277 | 9.949608618 | 7.417495551 | 9.432519753 | 8.323844727 | 6.996779418 | 7.116268722 | 7.584158019 | 6.323467592 | 8.193891805 | 8.759221554 | 6.273479396 | 6.667483629 | 8.666125125 | 6.323086118 | 5.898861318 | 5.784790929 | 6.934753765 | 6.667770512 |
| ENSMUSG00000026173 | Plcd4 | 5268 | 0.048411362 | 0.012647264 | 0.06427766 | 0 | 0.073093278 | 0.018812731 | 0.17064178 | 0.043499319 | 0 | 0.214809582 | 0.163240177 | 0.042380913 | 0.084280041 | 0.105795188 | 0.195527505 | 0.007268528 | 0.203708488 | 0.1775161 |
| ENSMUSG00000026174 | Rqcd1 | 3973 | 11.04086123 | 10.83318886 | 10.23965241 | 10.45851307 | 9.740263435 | 10.4767924 | 11.3488505 | 10.44931825 | 11.25813142 | 10.57419497 | 12.2654059 | 11.36543444 | 11.94495455 | 13.17454983 | 10.56742649 | 9.6858949 | 10.06149702 | 12.26732156 |
| ENSMUSG00000026175 | Vil1 | 3531 | 0.072226297 | 0.009434408 | 0.013699669 | 0.219353654 | 0.027262489 | 0.014033626 | 0.013399229 | 0.010816313 | 0 | 0.013353357 | 0.040590449 | 0.031614649 | 0 | 0.013153239 | 0.046674093 | 0 | 0 | 0 |
| ENSMUSG00000026176 | Ctdsp1 | 3496 | 2.030424599 | 2.753840512 | 2.310749305 | 1.993947306 | 2.47818829 | 2.239511415 | 2.977342495 | 3.32107847 | 3.145537674 | 2.522077091 | 2.186496956 | 3.145218973 | 4.388508659 | 2.617129638 | 2.380639099 | 4.030590253 | 2.071988019 | 2.659193088 |
| ENSMUSG00000026177 | Slc11a1 | 3780 | 0 | 0 | 0 | 0 | 0 | 0 | 0 | 0.060622861 | 0 | 0 | 0.025277756 | 0.044298145 | 0 | 0.11058116 | 0 | 0 | 0 | 0 |
| ENSMUSG00000026179 | Pnkd | 4442 | 56.99253229 | 57.34887485 | 55.7569733 | 65.85940245 | 58.90255224 | 58.94566507 | 71.54418378 | 73.75381086 | 70.16172102 | 82.30674315 | 82.68660081 | 71.05753533 | 71.75450642 | 78.86712093 | 81.68890201 | 77.3570263 | 79.75010407 | 76.6684781 |
| ENSMUSG00000026180 | Cxcr2 | 2535 | 0 | 0 | 0 | 0 | 0 | 0 | 0 | 0.045198109 | 0 | 0 | 0 | 0 | 0 | 0 | 0.032506158 | 0 | 0 | 0 |
| ENSMUSG00000026181 | Ppm1f | 4967 | 0.949884143 | 1.696831536 | 1.538759371 | 1.385085101 | 1.569835239 | 1.087426608 | 1.162099221 | 1.683941238 | 1.376477072 | 0.825872998 | 1.365823254 | 1.505798024 | 1.330879186 | 1.449332302 | 2.057174524 | 1.21802214 | 1.280886911 | 1.904885516 |
| ENSMUSG00000026182 | Tnp1 | 416 | 0.204351808 | 0 | 0 | 0 | 0 | 0 | 0.341197204 | 0 | 0.198012095 | 0 | 0 | 0 | 0 | 0 | 0 | 0 | 0 | 0 |
| ENSMUSG00000026185 | Igfbp5 | 9460 | 0.004493148 | 0.035214476 | 0.046021329 | 0.02408089 | 0.030527647 | 0.031428795 | 0.015004021 | 0 | 0 | 0.069779053 | 0 | 0.047201406 | 0.354605748 | 0.216019011 | 0.008710688 | 0.287381937 | 0.117490762 | 0.116298323 |
| ENSMUSG00000026187 | Xrcc5 | 2534 | 18.87068786 | 25.38563486 | 26.17210344 | 22.78052217 | 21.31176731 | 21.41288211 | 24.68325238 | 20.4376076 | 26.39577806 | 25.2500017 | 26.99831908 | 25.50692079 | 18.84502602 | 23.36867065 | 23.42992977 | 27.19932649 | 25.03157907 | 16.08593058 |
| ENSMUSG00000026188 | Tmem169 | 3121 | 0.013619089 | 0 | 0 | 0 | 0 | 0.047631593 | 0 | 0 | 0 | 0 | 0 | 0 | 0 | 0 | 0 | 0 | 0 | 0 |
| ENSMUSG00000026189 | Pecr | 1767 | 6.326463665 | 6.127159378 | 9.006729761 | 5.698353593 | 6.210570796 | 6.337814255 | 9.505376921 | 7.997277244 | 7.435483038 | 7.951844692 | 9.273803485 | 7.549486903 | 9.799379153 | 8.226937947 | 9.933142507 | 7.281066263 | 7.5047531 | 5.385724654 |
| ENSMUSG00000026192 | Atic | 5591 | 3.101790703 | 2.478655696 | 2.604262679 | 2.338762247 | 3.013087692 | 2.410721433 | 2.250969879 | 2.780237485 | 1.672212319 | 2.782996257 | 2.666032382 | 1.946709749 | 2.408802257 | 3.530448424 | 2.579242454 | 3.081876825 | 2.46094448 | 2.361328137 |
| ENSMUSG00000026193 | Fn1 | 9058 | 23.66925457 | 31.44460649 | 25.73548717 | 26.04494232 | 29.45410167 | 29.57955735 | 9.083323985 | 16.46093387 | 10.69448941 | 10.12454381 | 10.63834083 | 14.11720943 | 14.76471933 | 19.39699521 | 19.48181319 | 22.63280106 | 19.7132057 | 19.73720433 |
| ENSMUSG00000026196 | Bard1 | 5648 | 0.030102816 | 0.011796351 | 0.017129437 | 0.008066757 | 0 | 0.026320503 | 0.016753781 | 0 | 0.02187669 | 0 | 0 | 0 | 0.087344046 | 0.065784826 | 0 | 0.081353982 | 0.020357452 | 0 |
| ENSMUSG00000026197 | Zfand2b | 1660 | 23.35224129 | 20.69011683 | 22.72973113 | 18.41654262 | 21.97831213 | 20.86587998 | 23.48532978 | 29.288511 | 25.53067754 | 24.3706647 | 26.42012764 | 23.43585351 | 23.47723774 | 23.52980572 | 21.96588974 | 25.53476048 | 21.05637517 | 25.88079057 |
| ENSMUSG00000026198 | Abcb6 | 3731 | 3.508870066 | 4.982201816 | 5.302807268 | 5.849300519 | 4.902206109 | 5.737545137 | 3.639436841 | 7.165553922 | 6.413660053 | 6.470426177 | 6.466457795 | 6.298149008 | 7.615970075 | 5.053953224 | 6.99023985 | 6.506632295 | 5.11565153 | 5.351998322 |
| ENSMUSG00000026199 | Ankzf1 | 4052 | 5.842888215 | 5.105455901 | 6.924147978 | 5.779461202 | 7.329071299 | 4.708243442 | 5.231016821 | 6.202023885 | 6.830537658 | 6.365112212 | 6.590869148 | 6.033381099 | 6.330848219 | 4.974514789 | 8.07355266 | 5.641533223 | 5.911632666 | 3.774070007 |
| ENSMUSG00000026200 | Glb1l | 4953 | 4.222197982 | 4.230529066 | 4.639092857 | 4.240589832 | 4.441003254 | 4.111886433 | 5.330198837 | 6.739382132 | 4.415513804 | 4.112478055 | 5.604128988 | 3.876557224 | 4.501923402 | 6.376333419 | 7.744528273 | 5.821285559 | 5.880877377 | 5.26434819 |
| ENSMUSG00000026201 | Stk16 | 3261 | 19.27787652 | 16.35508851 | 15.26414067 | 17.19891002 | 15.68973777 | 14.8612614 | 18.67262121 | 17.31014129 | 18.730329 | 15.22529608 | 16.61355502 | 16.34589321 | 16.45910027 | 20.82221888 | 15.86910586 | 17.9534924 | 14.52661851 | 17.99899321 |
| ENSMUSG00000026202 | Tuba4a | 2049 | 27.29956645 | 31.47572631 | 24.55269476 | 30.04049336 | 25.72203827 | 31.46320468 | 31.14924542 | 27.53058973 | 22.89479817 | 30.76646669 | 29.26187062 | 27.26768094 | 26.7485408 | 29.26272173 | 23.20478062 | 24.01338727 | 25.40122557 | 31.06175524 |
| ENSMUSG00000026203 | Dnajb2 | 1400 | 17.63964805 | 20.74917411 | 15.85960745 | 16.20671434 | 17.91195159 | 20.17504153 | 17.57328074 | 15.60432441 | 15.91564645 | 20.10640703 | 17.3696099 | 17.66167038 | 18.53470606 | 21.26475715 | 18.6289892 | 16.82051682 | 19.84754661 | 15.40254988 |
| ENSMUSG00000026204 | Ptprn | 4077 | 0.010425601 | 0.032683732 | 0.225434652 | 0.089401117 | 0.035417162 | 0.02430843 | 0.011604778 | 0.18735542 | 0.030306487 | 0.08095534 | 0.02343633 | 0.06845188 | 0.07260032 | 0.079742117 | 0.121270216 | 0.122094161 | 0.103406737 | 0.053970181 |
| ENSMUSG00000026205 | Slc23a3 | 2519 | 0 | 0.039673951 | 0 | 0.108521741 | 0 | 0 | 0.056346978 | 0.045485195 | 0.179853781 | 0 | 0.037931686 | 0.044315731 | 0.430846439 | 0.05531253 | 0 | 0.212810045 | 0 | 0 |
| ENSMUSG00000026207 | Speg | 12490 | 0 | 0 | 0 | 0 | 0 | 0 | 0 | 0 | 0 | 0 | 0 | 0 | 0 | 0 | 0 | 0 | 0 | 0 |
| ENSMUSG00000026208 | Des | 3651 | 0 | 0 | 0 | 0 | 0 | 0 | 0 | 0.23013773 | 0 | 0 | 0 | 0 | 0 | 0.152651068 | 0 | 0 | 0 | 0 |
| ENSMUSG00000026209 | Dnpep | 3342 | 22.48628103 | 23.474526 | 20.77080074 | 25.83428457 | 21.68961013 | 23.18984899 | 21.77345906 | 22.11319529 | 20.61790571 | 21.65659171 | 25.48855505 | 22.24609891 | 20.42949537 | 19.84504991 | 19.15835364 | 17.91936265 | 18.4865322 | 21.13455806 |
| ENSMUSG00000026211 | Obsl1 | 8213 | 0.129383831 | 0.296096588 | 0.100127848 | 0.149780616 | 0.228557777 | 0.265471847 | 0.656720492 | 0.753338511 | 0.466375985 | 0.470760705 | 0.680588109 | 0.931053911 | 0.324354503 | 0.667283856 | 0.396313517 | 1.29142801 | 0.709314152 | 0.683176535 |
| ENSMUSG00000026213 | Stk11ip | 5809 | 5.136621376 | 5.356213324 | 5.895758219 | 6.172584247 | 5.26145146 | 6.073600673 | 7.305813909 | 7.094095747 | 5.962792177 | 6.866843683 | 5.987290372 | 5.601744067 | 5.460565125 | 7.755339089 | 6.440181227 | 5.99835647 | 7.283909757 | 6.865485002 |
| ENSMUSG00000026219 | Trip12 | 9591 | 13.25544589 | 13.10494733 | 15.03004071 | 14.6264681 | 15.39659491 | 14.64210687 | 10.21630258 | 12.14543086 | 11.92953193 | 11.7102466 | 11.99977844 | 12.06982423 | 12.31883452 | 14.1399995 | 11.59021977 | 14.25667386 | 12.01218314 | 12.88191572 |

| ENSMUSG00000026220 | Slc16a14 | 3193 | 0.332799687 | 0.302559954 | 0.590844874 | 0.470878316 | 0.361780823 | 0.186230129 | 0.014817626 | 0 | 0.064495014 | 0.014766897 | 0 | 0 | 0.015450021 | 0.130910362 | 0 | 0 | 0.048012899 | 0 |
| --- | --- | --- | --- | --- | --- | --- | --- | --- | --- | --- | --- | --- | --- | --- | --- | --- | --- | --- | --- | --- |
| ENSMUSG00000026222 | Sp100 | 5591 | 3.70238253 | 3.872899525 | 4.230845349 | 3.487770877 | 4.39910803 | 4.68849867 | 1.06624889 | 1.065643852 | 1.561713708 | 1.433664739 | 2.196058725 | 1.467519657 | 1.799984104 | 1.603238932 | 1.960224265 | 2.554533457 | 2.680304434 | 1.839868173 |
| ENSMUSG00000026223 | Itm2c | 2485 | 297.3138711 | 315.8089183 | 289.7934593 | 349.0874383 | 326.2317869 | 323.1595977 | 253.2799066 | 256.5269169 | 235.7658699 | 271.7094852 | 283.1891905 | 261.7833189 | 297.3017276 | 271.1886149 | 306.0023805 | 320.8706674 | 295.9530428 | 323.8121582 |
| ENSMUSG00000026224 | 4933407L21R | 1578 | 0 | 0.084443331 | 0 | 0.028872652 | 0.091505558 | 0 | 0 | 0 | 0 | 0 | 0 | 0 | 0 | 0 | 0 | 0 | 0 | 0 |
| ENSMUSG00000026226 | Spata3 | 2032 | 0.79488026 | 0.901677747 | 0.690370266 | 1.121088689 | 0.686922139 | 0.463337568 | 0.256121786 | 0.225545683 | 0 | 0 | 0 | 0 | 0 | 0 | 0.060829069 | 0 | 0 | 0 |
| ENSMUSG00000026227 | 2810459M11R | 3632 | 1.064969994 | 1.008925761 | 0.306330176 | 0.802837786 | 1.245705073 | 0.764028932 | 0.130266186 | 0 | 0.0113399 | 0.077892131 | 0 | 0.015367749 | 0.013582576 | 0 | 0 | 0 | 0 | 0 |
| ENSMUSG00000026228 | Htr2b | 2258 | 0.225891104 | 0 | 0.021423176 | 0.040355221 | 0 | 0.109727045 | 0.020953356 | 0.101485569 | 0 | 0.020881622 | 0.042316172 | 0.049438142 | 0 | 0.164549468 | 0.054740774 | 0.576563619 | 0.203682712 | 0 |
| ENSMUSG00000026229 | Psmd1 | 5173 | 48.3883591 | 53.43715373 | 55.53651591 | 50.5989174 | 49.94629156 | 53.31732362 | 39.92264518 | 45.39085443 | 42.89034611 | 41.20787346 | 43.46200553 | 42.6952594 | 35.39920296 | 35.1585243 | 39.24223195 | 34.18249081 | 42.56419403 | 44.93881404 |
| ENSMUSG00000026234 | Ncl | 8243 | 41.22635962 | 37.56440031 | 37.57560535 | 38.94493733 | 38.36306424 | 40.43931078 | 40.56274439 | 38.10436945 | 40.79178875 | 38.14728133 | 37.86989915 | 38.59629707 | 35.15415287 | 37.7615278 | 37.22285415 | 34.99249612 | 39.69326661 | 34.35483215 |
| ENSMUSG00000026235 | Epha4 | 6310 | 0.006736161 | 0.010558762 | 0.045997018 | 0.057763606 | 0.114418202 | 0.039265241 | 0 | 0 | 0 | 0 | 0 | 0 | 0 | 0 | 0.039177391 | 0 | 0.054665083 | 0 |
| ENSMUSG00000026237 | Nmur1 | 1320 | 0.257607127 | 0.302844493 | 0.696285664 | 0.51773914 | 0.328172207 | 0.750798994 | 0.071685877 | 0.868009146 | 0 | 0.03572023 | 0.434317804 | 0 | 0.523217304 | 0 | 0.218493099 | 0 | 0.377455954 | 0 |
| ENSMUSG00000026238 | Ptma | 1187 | 314.9393624 | 335.0373473 | 310.0062725 | 300.5800659 | 301.6050954 | 325.4115908 | 350.0819368 | 359.8515826 | 325.848837 | 326.4004454 | 318.6070525 | 341.3360528 | 320.9691635 | 324.2086842 | 324.2322945 | 344.8090164 | 317.3624029 | 328.4326363 |
| ENSMUSG00000026239 | Pde6d | 2298 | 77.18629224 | 71.30814911 | 74.24431748 | 75.95490028 | 74.18767347 | 62.08107919 | 91.80471512 | 76.4180445 | 82.05042174 | 98.24089086 | 82.97208846 | 78.40424666 | 104.8464247 | 82.37863368 | 85.37937735 | 96.9428868 | 94.33139046 | 89.57531652 |
| ENSMUSG00000026240 | Cops7b | 3267 | 13.46582712 | 12.66440607 | 10.43873245 | 11.85396011 | 13.50994001 | 11.78526905 | 12.58485399 | 13.31531874 | 15.35511974 | 12.07993224 | 11.71341957 | 14.50489671 | 11.46095048 | 12.43911125 | 13.36812037 | 14.15826561 | 12.49388007 | 12.19057033 |
| ENSMUSG00000026241 | Nppc | 1040 | 0.040870362 | 0 | 0 | 0 | 0 | 0 | 0 | 0 | 0 | 0 | 0.597186981 | 0 | 0 | 0.178631105 | 0 | 0 | 0.626487546 | 0.211573487 |
| ENSMUSG00000026245 | Farsb | 2426 | 40.73558708 | 31.74749029 | 33.13965682 | 35.70137893 | 33.56933835 | 33.51856381 | 34.94819482 | 32.66662613 | 34.00519005 | 29.8724777 | 30.93753494 | 27.03355463 | 30.15632041 | 24.428135 | 25.47499334 | 30.68299291 | 28.38926444 | 30.38425516 |
| ENSMUSG00000026246 | Alppl2 | 2102 | 0.141549111 | 0.332811978 | 0.322183361 | 0.065025277 | 0.320574182 | 0.047148177 | 3.128669063 | 2.289363798 | 2.410057773 | 0.829960048 | 0.022728334 | 0.982483119 | 2.088744357 | 3.579420627 | 1.626893025 | 0.87438114 | 0.820496366 | 0.968285894 |
| ENSMUSG00000026247 | Ecel1 | 3908 | 8.135586404 | 15.86368888 | 6.758430802 | 7.659571681 | 11.45411694 | 9.662022259 | 0.024213244 | 0.527735345 | 0.03161708 | 0.108586574 | 0.183373689 | 0.242800989 | 0.858385459 | 0.011884362 | 0.147600251 | 0.019596012 | 0.147107076 | 0.140760253 |
| ENSMUSG00000026248 | Mrpl44 | 1420 | 34.60280527 | 27.35410892 | 32.49883659 | 21.08000432 | 28.0656569 | 30.22018822 | 28.78743281 | 31.28011549 | 28.30847844 | 28.32362668 | 33.50975961 | 30.10901235 | 34.21967501 | 28.61871582 | 31.5104857 | 29.39208596 | 26.63947538 | 32.23068788 |
| ENSMUSG00000026249 | Serpine2 | 3821 | 2.146950791 | 2.458580521 | 2.152185334 | 2.158217489 | 2.897236965 | 1.815593484 | 1.225845384 | 1.729203249 | 0.355706627 | 1.32036777 | 1.250326053 | 0.832633543 | 24.3367349 | 53.34810247 | 29.62069459 | 8.728374522 | 12.12679742 | 22.34340058 |
| ENSMUSG00000026251 | Chrnd | 3027 | 0 | 0 | 0 | 0 | 0 | 0 | 0 | 0 | 0 | 0 | 0 | 0 | 0.081486484 | 0 | 0 | 0 | 0 | 0 |
| ENSMUSG00000026253 | Chrng | 1974 | 0 | 0 | 0 | 0 | 0 | 0 | 0 | 0 | 0 | 0 | 0 | 0 | 0 | 0 | 0 | 0 | 0 | 0 |
| ENSMUSG00000026254 | Eif4e2 | 7300 | 17.53775208 | 16.09058425 | 15.7710962 | 15.23486428 | 15.38902875 | 16.93617401 | 15.79465734 | 16.79942522 | 17.5014482 | 16.08291111 | 15.0196616 | 15.12375077 | 16.48226661 | 17.6360288 | 16.78540099 | 17.99658548 | 17.36758769 | 18.62774135 |
| ENSMUSG00000026255 | Efhd1 | 2117 | 54.09019566 | 55.65786814 | 48.00792975 | 59.93741543 | 55.08911245 | 51.54233318 | 44.07208448 | 42.0350957 | 38.13205994 | 45.34663762 | 39.06398353 | 37.80805864 | 51.07962337 | 44.27216266 | 50.03741152 | 66.88647434 | 60.81154053 | 49.94213657 |
| ENSMUSG00000026258 | 3110079O15R | 459 | 0 | 0 | 0 | 0 | 0 | 0 | 0 | 0 | 0 | 0.102724844 | 0 | 0 | 0 | 0 | 0 | 0 | 0 | 0 |
| ENSMUSG00000026259 | Ngef | 3889 | 1.071099833 | 1.027911367 | 0.298525258 | 1.347266675 | 0.754962032 | 0.828214884 | 1.167913905 | 0.795470454 | 1.186137764 | 1.527639139 | 1.265317748 | 0.5166788 | 3.247356859 | 3.128915119 | 3.496146424 | 3.327905709 | 3.833615195 | 4.611203083 |
| ENSMUSG00000026260 | Ndufa10 | 2327 | 103.6782893 | 98.19215361 | 96.76784865 | 99.149604 | 96.94642296 | 98.65827876 | 129.7794712 | 110.7694559 | 111.488553 | 126.5794771 | 122.1782458 | 111.8711862 | 121.7080091 | 99.57436699 | 112.1489708 | 105.5912452 | 108.4237269 | 109.1671916 |
| ENSMUSG00000026269 | Rnpepl1 | 2701 | 23.40066522 | 22.90338562 | 22.06449071 | 21.25394886 | 22.50670848 | 26.91368388 | 21.05510554 | 24.22198642 | 22.99491513 | 21.96060152 | 19.82811124 | 22.25600467 | 18.95835992 | 22.0269811 | 20.04400016 | 18.98227446 | 20.33379617 | 18.79800278 |
| ENSMUSG00000026270 | Capn10 | 4582 | 5.677251796 | 5.365542543 | 7.094502924 | 5.588238087 | 6.092648566 | 6.121092799 | 9.943716676 | 8.585371994 | 7.137078462 | 7.614910609 | 7.736582102 | 6.651102526 | 10.1743042 | 10.40988166 | 8.479499608 | 8.189610487 | 6.59961769 | 8.403835597 |
| ENSMUSG00000026271 | Gpr35 | 4494 | 0 | 0 | 0 | 0 | 0 | 0 | 0 | 0.050991191 | 0.018329558 | 0 | 0 | 0 | 0 | 0 | 0.119185631 | 0 | 0 | 0 |
| ENSMUSG00000026272 | Agxt | 1523 | 0 | 0 | 0 | 0 | 0 | 0 | 0 | 0 | 0 | 0 | 0 | 0 | 0 | 0 | 0 | 0 | 0 | 0 |
| ENSMUSG00000026273 | Mterfd2 | 5511 | 3.347350099 | 3.717552158 | 3.686605471 | 3.406124164 | 2.934553865 | 3.425801396 | 3.588586426 | 3.700733603 | 3.400447228 | 3.559189359 | 3.953072231 | 4.517108603 | 3.759645301 | 3.918798881 | 3.274592902 | 5.586218173 | 5.028109719 | 3.663281105 |
| ENSMUSG00000026274 | Pask | 10512 | 0.258783416 | 0.345424797 | 0.266901138 | 0.416082597 | 0.196886674 | 0.325260523 | 0.14402642 | 0.392387696 | 0.231164805 | 0.29603752 | 0.381763367 | 0.292034003 | 0.511527681 | 0.167891488 | 0.215571307 | 0.38975409 | 0.229695273 | 0.062795784 |
| ENSMUSG00000026275 | Ppp1r7 | 10489 | 6.196054357 | 7.149139564 | 7.549572809 | 7.571064952 | 7.19982727 | 6.779309056 | 8.277125163 | 7.59915567 | 7.040463606 | 8.271264562 | 6.636296549 | 6.917757781 | 7.47339275 | 6.827798909 | 8.174319555 | 7.268242858 | 9.628162107 | 8.228552622 |
| ENSMUSG00000026276 | 2-Sep | 5052 | 25.53507705 | 21.90527209 | 24.77084779 | 27.50617285 | 24.8281459 | 24.63904726 | 20.95917962 | 20.90300725 | 21.53079733 | 23.4260224 | 20.32232497 | 21.88654544 | 18.46529208 | 15.49974888 | 18.90443519 | 21.71468531 | 21.62871585 | 19.18567816 |
| ENSMUSG00000026277 | Stk25 | 4008 | 44.41409112 | 45.87172235 | 43.83549457 | 48.94856709 | 47.54348448 | 45.89315046 | 43.91296547 | 47.20687663 | 43.84801965 | 48.23300483 | 49.06230766 | 43.40753999 | 51.15355494 | 43.535538 | 46.1153143 | 43.4972893 | 47.93655848 | 49.54662557 |
| ENSMUSG00000026278 | Bok | 1427 | 0.863805259 | 1.634129359 | 1.322051635 | 1.117474809 | 1.113071816 | 1.284829112 | 0.729417615 | 1.579083211 | 1.38539086 | 1.255589855 | 1.037858243 | 0.664937816 | 2.973051777 | 3.417399563 | 3.89783465 | 2.629628259 | 1.799482756 | 2.58276815 |
| ENSMUSG00000026279 | Thap4 | 2165 | 29.88123691 | 33.95914898 | 38.22961221 | 34.11291126 | 35.85995052 | 40.6721513 | 25.65592843 | 31.96518853 | 38.58025587 | 33.90930484 | 30.67306803 | 31.50426775 | 24.99635716 | 23.40438769 | 29.36443452 | 30.6855445 | 28.32428409 | 30.05809388 |
| ENSMUSG00000026280 | Atg4b | 6024 | 16.17934406 | 16.39653575 | 14.43818187 | 14.67271348 | 15.60452306 | 14.1320711 | 12.66855098 | 15.81206036 | 15.71842625 | 14.84026118 | 15.33017841 | 15.09357808 | 16.38664782 | 14.00878267 | 14.95129502 | 15.45862503 | 14.245116 | 14.36409775 |
| ENSMUSG00000026281 | Dtymk | 2845 | 18.82478798 | 15.77239667 | 17.88715428 | 17.42369639 | 16.95191125 | 17.08654891 | 22.96618967 | 21.08972379 | 20.39782099 | 19.29118404 | 17.78336767 | 19.85428841 | 26.63403337 | 21.25490397 | 18.31984823 | 20.48446554 | 20.71910181 | 21.11421601 |
| ENSMUSG00000026283 | Ing5 | 4531 | 6.810584373 | 8.727081315 | 6.779340493 | 5.872357072 | 8.009594007 | 6.48527279 | 5.137461495 | 8.184688311 | 8.026416557 | 6.441466633 | 8.371952556 | 6.565823915 | 5.77045159 | 6.590939791 | 9.175098189 | 6.50712154 | 6.014124277 | 5.778930656 |

| ENSMUSG00000026285 | Pdcd1 | 1923 | 0 | 0 | 0 | 0 | 0 | 0 | 0 | 0 | 0 | 0 | 0 | 0 | 0 | 0 | 0 | 0 | 0 | 0 |
| --- | --- | --- | --- | --- | --- | --- | --- | --- | --- | --- | --- | --- | --- | --- | --- | --- | --- | --- | --- | --- |
| ENSMUSG00000026288 | Inpp5d | 7848 | 0.476612575 | 0.768301969 | 0.215733125 | 0.673430319 | 0.153325445 | 0.801885469 | 0.120572576 | 0.364988555 | 0.27814543 | 0 | 0.35307691 | 0.007112087 | 0.094288833 | 0.497107969 | 0.188997963 | 0.214677208 | 0 | 0.007009315 |
| ENSMUSG00000026289 | Atg16l1 | 5129 | 9.38113848 | 9.839936585 | 11.01584782 | 11.2459119 | 8.64291318 | 8.917366561 | 10.6358976 | 9.054778968 | 10.89688085 | 11.07751948 | 12.31399788 | 8.172657945 | 10.22418172 | 12.26978717 | 11.7443312 | 10.35466416 | 11.32826401 | 10.42481024 |
| ENSMUSG00000026295 | Spp2 | 958 | 0.488055257 | 0.660694144 | 0.252471453 | 0.285351008 | 0.301452549 | 0.413801533 | 3.802793609 | 1.475071909 | 5.11607033 | 5.118656721 | 2.543343301 | 2.330507832 | 9.011571528 | 3.151216785 | 8.859624753 | 7.873955802 | 6.761110832 | 9.474428611 |
| ENSMUSG00000026301 | Iqca | 3532 | 0.012034308 | 0.132044315 | 0.02739158 | 0.051598012 | 0.231665544 | 0 | 0 | 0.032439753 | 0.116609614 | 0.080097457 | 0 | 0 | 0 | 0.039448545 | 0.01166522 | 0 | 0.0542558 | 0.062297969 |
| ENSMUSG00000026303 | Mlph | 5341 | 22.97555574 | 23.55167358 | 26.98085507 | 25.36950868 | 23.36754879 | 27.29528968 | 21.95109874 | 22.6680239 | 24.65330292 | 24.9922563 | 21.20846781 | 22.15487824 | 19.32266822 | 19.02633649 | 19.04636613 | 19.70808458 | 17.2579562 | 19.4349718 |
| ENSMUSG00000026304 | Rab17 | 2832 | 13.55302753 | 11.64539734 | 13.54527174 | 13.51386908 | 11.4551259 | 11.51331169 | 26.56326253 | 23.57355911 | 27.83580197 | 31.86668292 | 31.20892415 | 30.96271396 | 36.58087086 | 32.01230882 | 27.88961252 | 29.59680071 | 31.7491146 | 34.51666054 |
| ENSMUSG00000026305 | Lrrfip1 | 5821 | 0.839734623 | 0.972889884 | 0.747915776 | 0.907933541 | 0.76071757 | 0.987479315 | 0.162558594 | 0.505207866 | 0.297171218 | 0.348304456 | 1.075162268 | 0.03835469 | 1.110201196 | 1.061168807 | 0.120327512 | 0.302588548 | 0.210692579 | 0.160651918 |
| ENSMUSG00000026307 | Scly | 3980 | 5.991307475 | 6.470067144 | 6.466009585 | 8.093381494 | 6.784424416 | 6.847739565 | 5.361311105 | 5.709668199 | 6.12623551 | 6.78827964 | 7.274277598 | 7.22237845 | 7.982350434 | 7.655105861 | 7.236152655 | 7.061634631 | 6.008947052 | 8.444865379 |
| ENSMUSG00000026308 | Klhl30 | 3081 | 0 | 0.010812364 | 0 | 0 | 0.062488703 | 0 | 0 | 0 | 0 | 0 | 0 | 0 | 0 | 0 | 0 | 0 | 0.024879128 | 0 |
| ENSMUSG00000026309 | Ilkap | 1487 | 41.99065473 | 40.5265808 | 42.06252505 | 42.80348281 | 42.66165124 | 38.7890934 | 43.71729714 | 36.75408732 | 40.13400225 | 47.02386882 | 42.2809989 | 41.51454124 | 44.62100117 | 47.19368934 | 45.02523761 | 46.99418864 | 45.28534207 | 55.34204684 |
| ENSMUSG00000026311 | Asb1 | 6221 | 1.305013442 | 1.194144898 | 1.181928406 | 1.245037379 | 1.802722748 | 1.2505673 | 1.003901884 | 1.774249204 | 2.26423218 | 1.811448011 | 2.042780735 | 1.570123927 | 2.212442521 | 1.829095228 | 2.563093093 | 1.754191144 | 1.823594902 | 2.051456801 |
| ENSMUSG00000026312 | Cdh7 | 8320 | 0 | 0 | 0 | 0 | 0 | 0 | 0 | 0 | 0 | 0 | 0 | 0 | 0.005929317 | 0 | 0 | 0 | 0 | 0 |
| ENSMUSG00000026313 | Hdac4 | 4492 | 15.52782588 | 14.94333968 | 16.69166787 | 13.34780373 | 14.86174936 | 14.21938415 | 14.58772491 | 12.20932543 | 12.66219463 | 13.82401518 | 14.40055516 | 14.46336404 | 16.51718691 | 14.08211198 | 15.69364696 | 18.11387817 | 17.54204507 | 17.26688345 |
| ENSMUSG00000026315 | Serpinb8 | 1656 | 0.718686551 | 0.40232964 | 0 | 0.055025416 | 0.290651713 | 0.538616669 | 0 | 0.253693494 | 0.572034941 | 0 | 0.028849613 | 0.101115331 | 0.417057272 | 0.392643251 | 0.696644665 | 0.832404509 | 1.758936331 | 1.361940444 |
| ENSMUSG00000026317 | Cln8 | 7599 | 9.945282666 | 8.596734513 | 9.542297934 | 9.263299579 | 7.879472699 | 10.02922809 | 9.806220465 | 8.48887586 | 9.165205705 | 9.102524237 | 10.7130582 | 9.930619268 | 8.822486588 | 7.627611668 | 10.95780293 | 12.1336732 | 8.508548872 | 9.37449575 |
| ENSMUSG00000026319 | 2310035C23R | 7374 | 4.876509211 | 5.976804862 | 5.516970305 | 7.093040263 | 7.349680777 | 6.189051753 | 6.069676467 | 7.872586338 | 6.931443729 | 6.649950012 | 6.200248883 | 7.455712998 | 6.4290714 | 8.987755995 | 8.029107108 | 6.859491779 | 8.243220354 | 6.400571409 |
| ENSMUSG00000026321 | Tnfrsf11a | 5012 | 11.23690308 | 13.45277292 | 12.76899055 | 13.91739004 | 13.18538132 | 14.6522648 | 8.561971227 | 11.31598515 | 8.685983073 | 12.48383544 | 9.789481715 | 12.27231847 | 9.61637733 | 11.86121944 | 10.81828562 | 13.39254485 | 12.75504055 | 11.93034697 |
| ENSMUSG00000026322 | Htr4 | 4650 | 0 | 0 | 0 | 0 | 0 | 0 | 0 | 0 | 0 | 0 | 0 | 0 | 0.169744231 | 0.06991583 | 0 | 0.008234539 | 0 | 0 |
| ENSMUSG00000026327 | Serpinb11 | 2076 | 0 | 0 | 0.023301315 | 0 | 0 | 0 | 0 | 0 | 0 | 0 | 0 | 0 | 0 | 0 | 0 | 0 | 0 | 0 |
| ENSMUSG00000026331 | Slco6c1 | 2780 | 0 | 0 | 0 | 0 | 0 | 0 | 0 | 0 | 0 | 0 | 0 | 0 | 0 | 0 | 0 | 0 | 0 | 0 |
| ENSMUSG00000026333 | Gin1 | 3935 | 5.476524586 | 3.547167135 | 6.367849739 | 4.816618664 | 4.183257406 | 4.029701333 | 4.857515194 | 4.707322787 | 4.741421001 | 4.193836376 | 5.572733403 | 5.773055825 | 4.212331437 | 5.193239759 | 3.664687309 | 5.34219656 | 3.886198847 | 4.095976689 |
| ENSMUSG00000026335 | Pam | 7944 | 5.414808426 | 5.258606414 | 3.519624943 | 4.089252846 | 4.029170347 | 4.116918953 | 3.990369446 | 3.84135568 | 3.691439983 | 5.169720863 | 3.746701804 | 2.459149283 | 5.613803275 | 3.788490512 | 3.66166899 | 7.601244697 | 5.437278022 | 4.064746431 |
| ENSMUSG00000026336 | Slco6d1 | 3524 | 0 | 0 | 0 | 0 | 0 | 0 | 0 | 0 | 0 | 0 | 0 | 0 | 0 | 0 | 0 | 0 | 0 | 0 |
| ENSMUSG00000026339 | Ccdc93 | 11602 | 1.28226268 | 1.22317643 | 0.954795591 | 1.452989692 | 0.535167915 | 0.832854943 | 0.827829152 | 0.711046278 | 0.933636756 | 0.800610976 | 0.366486063 | 1.529855258 | 0.731347162 | 0.680528776 | 0.365778336 | 1.165021928 | 0.981116202 | 1.152147296 |
| ENSMUSG00000026341 | Actr3 | 2496 | 76.81925043 | 80.1458052 | 85.27385159 | 78.6548638 | 85.73498903 | 87.15404664 | 59.65264447 | 72.63595121 | 62.14279582 | 67.13685872 | 69.17415863 | 58.61091812 | 57.21790886 | 57.85042776 | 62.67720662 | 69.95399429 | 70.11132679 | 57.5656197 |
| ENSMUSG00000026342 | Slc35f5 | 2581 | 80.33330053 | 87.08333874 | 82.35307725 | 76.73531954 | 81.21446245 | 90.10111534 | 42.54658186 | 55.60908494 | 52.38873741 | 54.09268979 | 56.58583807 | 62.30334131 | 54.39699208 | 58.28454051 | 64.17289992 | 55.33667788 | 62.96144861 | 70.24797201 |
| ENSMUSG00000026343 | Gpr39 | 3012 | 1.552313866 | 0.873744569 | 1.027857219 | 0.72607242 | 1.166543967 | 0.888395622 | 0.267037032 | 0.342362173 | 0.546965681 | 0.297431395 | 0.41240004 | 0.296497544 | 0.147406127 | 0.462590512 | 0.068395677 | 0.063563425 | 0.623502174 | 0.310476366 |
| ENSMUSG00000026344 | Lypd1 | 8319 | 0 | 0 | 0 | 0 | 0 | 0 | 0 | 0 | 0 | 0.005667833 | 0.045942982 | 0 | 0 | 0.005582893 | 0.029716232 | 0 | 0 | 0 |
| ENSMUSG00000026347 | Tmem163 | 2830 | 1.321715721 | 0.812222509 | 1.247797779 | 0.450780651 | 1.734790182 | 0.542803795 | 1.153560016 | 1.619465826 | 1.076961896 | 1.332882071 | 1.97514846 | 1.360876579 | 0.66240737 | 0.443106134 | 0.407647903 | 0.216483999 | 1.774114804 | 0.544259713 |
| ENSMUSG00000026348 | Acmsd | 2293 | 0.055610784 | 0.029056166 | 0 | 0 | 0.062972425 | 0.086441751 | 0.061900583 | 0 | 0 | 0 | 0 | 0 | 0 | 0.020254726 | 0 | 0.050096739 | 0 | 0 |
| ENSMUSG00000026349 | Ccnt2 | 8542 | 9.608697601 | 10.71300871 | 10.69744777 | 9.088740289 | 10.00165822 | 8.428953628 | 7.998069348 | 11.88426663 | 10.30386141 | 9.234737362 | 10.16797875 | 9.8013924 | 9.679266373 | 9.678117016 | 12.04887457 | 9.552480025 | 11.10933167 | 11.28903817 |
| ENSMUSG00000026353 | Ubxn4 | 4080 | 53.71487441 | 49.9285167 | 46.28682905 | 44.5560213 | 47.7898095 | 47.37872885 | 52.28746796 | 47.0852412 | 46.39539864 | 47.86720904 | 45.22227687 | 45.19974245 | 52.60862061 | 58.3169264 | 48.40172975 | 53.76639456 | 52.78320154 | 52.71706061 |
| ENSMUSG00000026354 | Lct | 6389 | 0 | 0 | 0 | 0 | 0 | 0 | 0 | 0.077711885 | 0 | 0 | 0 | 0 | 0.138984898 | 0.138118275 | 0.025795308 | 0.017979625 | 0 | 0.034439885 |
| ENSMUSG00000026355 | Mcm6 | 4808 | 4.756194822 | 5.833913671 | 6.68053747 | 6.083650263 | 5.916382464 | 5.792145647 | 3.94600338 | 6.045012111 | 3.700618721 | 3.775586679 | 5.822821478 | 4.771264002 | 5.663730935 | 4.018456812 | 4.961668232 | 4.682794749 | 5.484295397 | 3.466672075 |
| ENSMUSG00000026356 | Dars | 2490 | 62.93838715 | 64.79291032 | 72.17175312 | 66.38372243 | 63.267384 | 64.89617036 | 62.81755684 | 57.45732506 | 66.75854523 | 62.90547639 | 57.92473881 | 61.30756513 | 44.7355298 | 53.45733111 | 61.9181615 | 55.62133613 | 61.18354626 | 58.56592045 |
| ENSMUSG00000026357 | Rgs18 | 2026 | 0 | 0 | 0.119381862 | 0 | 0 | 0 | 0 | 0 | 0 | 0 | 0 | 0 | 0 | 0 | 0 | 0 | 0 | 0 |
| ENSMUSG00000026358 | Rgs1 | 4720 | 0.009005334 | 0 | 0.040994517 | 0 | 0 | 0 | 0 | 0.202290267 | 0.139615308 | 0 | 0 | 0.011825352 | 0 | 0 | 0.497561162 | 0 | 0 | 0 |
| ENSMUSG00000026360 | Rgs2 | 3817 | 43.78578783 | 44.30082553 | 56.40832686 | 44.7493726 | 55.53392503 | 57.47182383 | 75.90852653 | 94.77559224 | 126.7426812 | 74.98160041 | 104.3864694 | 122.7739856 | 43.10242183 | 40.4940851 | 52.95646665 | 29.12172735 | 59.72355619 | 58.07877917 |
| ENSMUSG00000026361 | Cdc73 | 14343 | 3.262790128 | 3.523367533 | 3.918987818 | 3.576778632 | 3.641019116 | 3.700130911 | 3.879224041 | 3.549499575 | 3.71003126 | 4.023737069 | 3.494103845 | 3.482884893 | 3.501352004 | 3.976388434 | 2.924296444 | 4.244723264 | 4.382285918 | 3.543778471 |
| ENSMUSG00000026365 | Cfh | 7473 | 0 | 0.057950973 | 0 | 0 | 0.025763106 | 0.112725341 | 0.012662299 | 0.081771503 | 0.374773595 | 0.018928424 | 0.166218242 | 0 | 0.151831138 | 0.006214919 | 0.170915059 | 0.266440731 | 0.02564318 | 0.191387231 |

| ENSMUSG00000026368 | F13b | 2518 | 0 | 0 | 0 | 0 | 0 | 0 | 0 | 0 | 0 | 0 | 0 | 0 | 0 | 0 | 0 | 0 | 0 | 0 |
| --- | --- | --- | --- | --- | --- | --- | --- | --- | --- | --- | --- | --- | --- | --- | --- | --- | --- | --- | --- | --- |
| ENSMUSG00000026374 | Tsn | 3034 | 49.56602267 | 48.33334224 | 50.06357459 | 48.90979611 | 51.22543887 | 50.12437025 | 51.63226102 | 50.52877497 | 48.08261003 | 50.86494784 | 44.72013252 | 52.30188818 | 52.19362375 | 49.44443054 | 48.88780528 | 54.28078525 | 50.88276992 | 44.98272702 |
| ENSMUSG00000026377 | Mki67ip | 2100 | 18.25698513 | 24.3025495 | 20.54723289 | 23.14934967 | 21.77396547 | 20.26942769 | 23.09309329 | 26.28001023 | 27.45767717 | 29.61513219 | 25.98047742 | 23.78810382 | 25.60561421 | 29.65786723 | 27.38922477 | 26.52992084 | 26.02538066 | 25.33038391 |
| ENSMUSG00000026380 | Tfcp2l1 | 9254 | 109.6572912 | 124.9035542 | 107.1755449 | 123.7740063 | 121.2660255 | 123.8443508 | 110.8427576 | 123.314328 | 103.771861 | 134.9914342 | 119.4528841 | 121.0465239 | 117.1513091 | 99.22693449 | 99.1170345 | 138.6472523 | 144.8770469 | 110.9277005 |
| ENSMUSG00000026383 | Epb4.1l5 | 7744 | 15.79673252 | 15.46915904 | 13.92987767 | 16.26759912 | 16.40239496 | 17.53286286 | 14.3147736 | 14.70683677 | 14.35996805 | 13.07847503 | 12.25837326 | 13.39172276 | 11.79150037 | 11.34713499 | 13.418172 | 13.18711905 | 15.2236169 | 13.00641457 |
| ENSMUSG00000026384 | Ptpn4 | 5869 | 2.991078156 | 2.548558443 | 2.192427854 | 2.616130847 | 1.673012852 | 2.701801797 | 1.338201517 | 1.34054096 | 1.642127226 | 1.799583835 | 1.823409559 | 2.501195989 | 3.3958246 | 1.843835809 | 1.242575463 | 1.461423762 | 2.377027104 | 1.246585652 |
| ENSMUSG00000026385 | Dbi | 1660 | 48.26641977 | 44.95233919 | 42.8076603 | 38.91901257 | 46.0732691 | 41.46310057 | 40.89981582 | 40.33209724 | 43.84130925 | 43.31615811 | 49.78956516 | 40.21417618 | 98.42609037 | 118.6562498 | 85.60491944 | 87.03039863 | 83.46359235 | 84.07114684 |
| ENSMUSG00000026387 | Sctr | 2201 | 0 | 0 | 0 | 0.041400313 | 0 | 0.022513736 | 0.021495992 | 0.017352296 | 0.261976929 | 0.064267201 | 0.108530119 | 0 | 0.022413411 | 0.316520359 | 0.205914182 | 1.200387056 | 1.323397803 | 0.824761709 |
| ENSMUSG00000026388 | 3110009E18R | 2209 | 3.290350883 | 1.417569967 | 3.000078614 | 2.39252202 | 1.743120821 | 1.83944054 | 2.998540086 | 2.437812919 | 2.050935597 | 1.963723268 | 2.400643002 | 2.071925909 | 3.461497138 | 1.871219456 | 2.014381184 | 3.154771637 | 2.255508645 | 2.06688812 |
| ENSMUSG00000026389 | Steap3 | 5542 | 0.023008937 | 0 | 0.017457066 | 0.024663142 | 0.052109625 | 0.017882618 | 0.102445353 | 0 | 0.074317062 | 0.008507886 | 0 | 0 | 0.1513249 | 0.075423455 | 0.111516301 | 0.158910857 | 0.041493645 | 0.05955515 |
| ENSMUSG00000026390 | Marco | 1968 | 0 | 0 | 0 | 0 | 0 | 0 | 0 | 0 | 0 | 0 | 0 | 0 | 0 | 0 | 0 | 0 | 0 | 0 |
| ENSMUSG00000026393 | Nek7 | 4759 | 27.97356825 | 34.27189117 | 31.49006032 | 29.80280122 | 34.10398122 | 32.42429342 | 29.0199012 | 32.22960455 | 30.72328871 | 35.21193515 | 30.48803297 | 32.3587756 | 23.64490509 | 26.90614602 | 27.16757352 | 26.83319514 | 34.44454126 | 29.64874107 |
| ENSMUSG00000026394 | Atp6v1g3 | 1882 | 1957.203002 | 1846.971644 | 1876.414897 | 1735.919365 | 1929.343353 | 1781.028458 | 2010.160365 | 2024.522024 | 2260.969017 | 2071.574415 | 2052.927028 | 2012.448247 | 2190.971469 | 1845.325759 | 1928.21968 | 2192.391595 | 2133.646535 | 1961.8843 |
| ENSMUSG00000026395 | Ptprc | 7127 | 0.322054091 | 0.906791284 | 0.692310944 | 0.709593927 | 0.648332352 | 0.618800798 | 0.770067456 | 0.975307596 | 0.837946511 | 1.038678323 | 1.783097931 | 0.532547363 | 0.823698352 | 0.938396041 | 0.861376712 | 0.725302652 | 0.220482415 | 1.088295783 |
| ENSMUSG00000026398 | Nr5a2 | 3826 | 0 | 0 | 0 | 0 | 0 | 0 | 0 | 0 | 0 | 0 | 0 | 0 | 0 | 0 | 0 | 0 | 0 | 0 |
| ENSMUSG00000026399 | Cd55 | 5068 | 13.360447 | 9.629713897 | 10.33712182 | 13.88946596 | 10.73186143 | 14.68591275 | 7.160383729 | 3.941323295 | 5.599350702 | 5.200718848 | 5.580658133 | 6.784224182 | 8.595122919 | 5.434361449 | 7.097268803 | 7.608256048 | 8.174965855 | 11.38606017 |
| ENSMUSG00000026401 | Daf2 | 1867 | 0.045533129 | 0.071372028 | 0.025909765 | 0.146420067 | 0.103121422 | 0.132706839 | 0.202732422 | 0 | 0.066180797 | 0.101019182 | 0.051178317 | 0.029895909 | 0.105692378 | 0.024876319 | 0.022068321 | 0.102545815 | 0.041056558 | 0.265175126 |
| ENSMUSG00000026404 | Ddx59 | 2944 | 4.793382623 | 5.431450144 | 4.567880923 | 6.298690572 | 4.496018685 | 5.87428805 | 5.785517804 | 6.343778796 | 5.14831447 | 6.806742148 | 5.225386084 | 5.70669648 | 5.127570206 | 3.943961225 | 4.6743613 | 4.721294323 | 4.204957167 | 5.026307646 |
| ENSMUSG00000026405 | C4bp | 3051 | 13.12352534 | 12.76393751 | 17.39290816 | 10.96093299 | 10.06495159 | 8.315634085 | 8.622041784 | 18.73943835 | 6.574183276 | 4.110811887 | 1.064797501 | 5.817561686 | 3.217650455 | 6.743602331 | 3.4435912 | 0.815761875 | 0.615532136 | 2.542210439 |
| ENSMUSG00000026407 | Cacna1s | 6481 | 0 | 0 | 0 | 0 | 0 | 0.007645847 | 0.007300213 | 0.047143839 | 0.050839705 | 0 | 0.272747024 | 0 | 0.015223551 | 0 | 0.012714567 | 0.053173193 | 0 | 0 |
| ENSMUSG00000026409 | Pfkfb2 | 12172 | 1.564436318 | 2.153898106 | 1.76452904 | 2.103623638 | 2.10764996 | 1.681340697 | 2.145629212 | 2.070899243 | 1.583576189 | 2.254494684 | 2.563017408 | 1.279376426 | 1.994027547 | 2.709111243 | 2.38977148 | 2.381366234 | 2.166323716 | 2.336486048 |
| ENSMUSG00000026411 | Tmem9 | 1931 | 67.92903844 | 66.74654984 | 57.84281802 | 61.25140916 | 65.53020576 | 66.05320364 | 75.09754579 | 66.13950991 | 69.9808173 | 75.42647455 | 89.38939668 | 84.80743347 | 78.35369771 | 66.02227855 | 68.2140726 | 76.42257945 | 70.49974448 | 72.6712746 |
| ENSMUSG00000026413 | Pkp1 | 5705 | 0.149010258 | 0.046713962 | 0.067833171 | 0.103820083 | 0 | 0.034743371 | 0 | 0.227614668 | 0 | 0 | 0 | 0.136970951 | 0.06052996 | 0.24422833 | 0.180550201 | 0 | 0.020154056 | 0 |
| ENSMUSG00000026414 | Tnnt2 | 1090 | 0 | 0 | 0 | 0.167196493 | 0 | 0.227306117 | 0.34724902 | 0.525583519 | 0 | 0 | 0 | 0.409656239 | 0 | 0.213046272 | 0.037799593 | 0 | 0 | 0 |
| ENSMUSG00000026415 | Fcamr | 1904 | 0 | 0.017496268 | 0 | 0.07178736 | 0 | 0 | 0 | 0 | 0 | 0.074292074 | 0.07527567 | 0.087944847 | 0 | 0 | 0.173115781 | 0 | 0 | 0 |
| ENSMUSG00000026416 | Il20 | 896 | 0 | 0 | 0 | 0 | 0 | 0 | 0 | 0 | 0 | 0 | 0 | 0 | 0 | 0 | 0 | 0 | 0 | 0 |
| ENSMUSG00000026417 | Pigr | 4012 | 1.059451048 | 0.78051148 | 0.819890345 | 0.249836235 | 0.539864548 | 0.58050311 | 0.023585583 | 0.161832214 | 1.529608885 | 0.129276604 | 0.785929028 | 1.349481374 | 0.172145275 | 0.150491809 | 0.256739506 | 0.286320594 | 0.124187901 | 0 |
| ENSMUSG00000026418 | Tnni1 | 1695 | 0 | 0 | 0.057077912 | 0 | 0.113585661 | 0 | 0 | 0 | 0 | 0 | 0 | 0 | 0.407461264 | 0.575413472 | 0.121538513 | 0 | 0 | 0 |
| ENSMUSG00000026420 | Il24 | 1842 | 0 | 0 | 0 | 0 | 0 | 0 | 0 | 0.020734203 | 0 | 0 | 0 | 0 | 0 | 0 | 0 | 0 | 0 | 0 |
| ENSMUSG00000026421 | Csrp1 | 1949 | 115.8914856 | 190.5620614 | 171.6776344 | 150.3818359 | 157.4844932 | 132.462669 | 75.56919933 | 121.1026408 | 127.4689908 | 98.70439676 | 111.8997873 | 70.13471405 | 98.33478636 | 119.815737 | 116.8610574 | 177.7792234 | 136.5509521 | 113.8002659 |
| ENSMUSG00000026424 | Gpr37l1 | 2267 | 0.937476313 | 0.881682247 | 1.792402536 | 0.844095219 | 0.679409597 | 1.15848914 | 0.104350858 | 0.404330683 | 0.0726714 | 0.395175722 | 0.779741272 | 0.714007153 | 0.282891453 | 0 | 0.163570359 | 0.15201388 | 0.439560529 | 0.024265155 |
| ENSMUSG00000026425 | Srgap2 | 8655 | 7.346937416 | 8.244508309 | 6.511283982 | 7.611931844 | 7.168347731 | 7.54598531 | 5.340784207 | 5.798361266 | 6.35285945 | 5.398769142 | 5.094891586 | 6.352215788 | 5.591520606 | 5.779350909 | 6.364700199 | 6.322042516 | 6.327935098 | 7.506153095 |
| ENSMUSG00000026426 | Arl8a | 2292 | 62.73778818 | 70.05591193 | 66.52415693 | 69.19633304 | 64.67989742 | 74.2426209 | 68.47127225 | 62.52089608 | 61.79774333 | 56.09946238 | 56.48784358 | 60.71049164 | 54.60518068 | 62.20913974 | 59.35756438 | 58.65546361 | 60.11476325 | 62.73726221 |
| ENSMUSG00000026427 | Eif2d | 7169 | 8.549653206 | 9.223893852 | 9.655812797 | 8.369911479 | 7.868686642 | 9.234544854 | 7.411373753 | 8.188272074 | 9.680468553 | 8.76059935 | 8.263488424 | 9.078122831 | 8.250658225 | 8.946754733 | 9.304689495 | 7.317356973 | 7.832058162 | 8.217987627 |
| ENSMUSG00000026429 | Ube2t | 1777 | 1.076383186 | 0.337440684 | 0.734994552 | 0.640982616 | 0.893839888 | 0.892339603 | 0.505875577 | 1.504484056 | 1.645606426 | 1.406295595 | 1.586225408 | 0.565380938 | 2.05433983 | 1.385220389 | 1.99399764 | 0.94810733 | 1.121534856 | 2.445537102 |
| ENSMUSG00000026430 | Rassf5 | 3831 | 0.410517231 | 1.21738585 | 0.795492668 | 0.963311039 | 0.992540321 | 1.177055274 | 0.90154674 | 0.638035436 | 0.279522164 | 0.72615283 | 0.910355513 | 0.50993166 | 1.210441197 | 0.945611803 | 0.677551037 | 0.279858263 | 1.020433903 | 0.402050375 |
| ENSMUSG00000026432 | Avpr1b | 4021 | 0 | 0 | 0 | 0 | 0 | 0 | 0 | 0 | 0 | 0 | 0 | 0 | 0 | 0.080852676 | 0 | 0 | 0 | 0 |
| ENSMUSG00000026433 | Rab7l1 | 1779 | 6.379360314 | 5.299353043 | 5.057603511 | 4.43061308 | 4.788842321 | 4.707370419 | 5.824326411 | 5.302711295 | 4.81551168 | 7.182597292 | 6.49889823 | 3.294347707 | 5.934249745 | 3.472211143 | 4.446710922 | 5.337869947 | 4.373377331 | 3.617799599 |
| ENSMUSG00000026434 | Nucks1 | 6089 | 15.21781635 | 15.98625011 | 19.39231197 | 16.7309074 | 18.03860237 | 16.81326122 | 17.87143398 | 18.39056066 | 17.82336493 | 17.15204594 | 19.88988664 | 16.44494969 | 17.96171138 | 17.74165664 | 18.0125705 | 17.8970387 | 20.12933117 | 19.54092591 |
| ENSMUSG00000026435 | Slc45a3 | 3334 | 0.522709123 | 0.519577234 | 0.435274718 | 0.382636245 | 0.461974072 | 0.326982645 | 0 | 0.584226911 | 0 | 0 | 0.315251676 | 0.033482701 | 0 | 0 | 0 | 0 | 0.10346031 | 0 |
| ENSMUSG00000026436 | Elk4 | 5538 | 5.395655245 | 5.690501613 | 5.083675454 | 4.467252993 | 4.867077872 | 5.144966018 | 3.237902729 | 4.799911895 | 5.042336166 | 4.078220813 | 4.175348484 | 4.716816557 | 4.1956181 | 3.555849233 | 3.623177631 | 4.397404534 | 4.221567553 | 2.532922032 |

| ENSMUSG00000026437 | Cdk18 | 3153 | 1.941244957 | 3.275292486 | 2.914992314 | 3.034512943 | 3.053087451 | 2.923186948 | 3.886452218 | 3.87617151 | 2.756217832 | 4.261957002 | 3.030444559 | 2.655359781 | 3.176143104 | 1.443552351 | 3.25378605 | 2.683864331 | 3.026719927 | 3.64633787 |
| --- | --- | --- | --- | --- | --- | --- | --- | --- | --- | --- | --- | --- | --- | --- | --- | --- | --- | --- | --- | --- |
| ENSMUSG00000026439 | Rbbp5 | 3618 | 8.106293936 | 7.780374686 | 8.302919389 | 8.81501687 | 8.793588044 | 7.395930385 | 9.572382801 | 8.33941346 | 9.403002217 | 10.47793406 | 7.42109637 | 8.376977552 | 5.835837644 | 8.857495937 | 8.25625429 | 11.15486462 | 9.692803114 | 8.636034444 |
| ENSMUSG00000026442 | Nfasc | 10344 | 0.008218325 | 0.022543529 | 0 | 0 | 0 | 0 | 0 | 0 | 0 | 0 | 0 | 0 | 0.004769134 | 0 | 0.047797629 | 0 | 0 | 0 |
| ENSMUSG00000026443 | Lrrn2 | 3913 | 0 | 0.025540169 | 0.321418806 | 0.232870147 | 0.147606206 | 0.063318085 | 0.133002675 | 0 | 0 | 0.240995161 | 0 | 0 | 0 | 0 | 0.515940771 | 0 | 0 | 0 |
| ENSMUSG00000026447 | Pik3c2b | 9203 | 1.057664382 | 1.158331647 | 1.124843583 | 1.000035962 | 0.951864621 | 0.500750214 | 0.807137954 | 1.09974863 | 1.14121064 | 0.763387459 | 0.970761408 | 0.527649967 | 1.238256318 | 0.837739705 | 0.85957826 | 1.281485068 | 0.841237854 | 1.117755401 |
| ENSMUSG00000026450 | Chit1 | 1803 | 1.060861299 | 4.415852312 | 5.687847163 | 1.541444096 | 2.936501167 | 1.154306606 | 0.708509335 | 2.88084677 | 3.289438863 | 1.725982482 | 1.192386651 | 0.804884763 | 0.191527133 | 0.824298833 | 2.719348392 | 0.934435233 | 1.16913274 | 0.915292957 |
| ENSMUSG00000026452 | Syt2 | 6870 | 0.142302627 | 0.130924038 | 0.035206354 | 0.053055073 | 0.168146458 | 0.050490413 | 0.089529087 | 0.088948827 | 0.125897646 | 0.027453102 | 0.069541424 | 0 | 0.14361548 | 0.216333449 | 0.131940936 | 0 | 0.089260662 | 0.024021444 |
| ENSMUSG00000026455 | Klhl12 | 6653 | 6.388873595 | 7.250423994 | 8.36884615 | 7.108426878 | 7.53847354 | 7.604590558 | 6.286548651 | 7.996695709 | 6.803544389 | 6.87452009 | 7.238412468 | 7.701605028 | 5.857840771 | 7.211294495 | 6.310594537 | 4.949635107 | 7.36800447 | 8.888439761 |
| ENSMUSG00000026456 | Cyb5r1 | 3957 | 9.023085131 | 8.620774243 | 7.212631516 | 7.679862667 | 7.991577925 | 7.651432958 | 8.297952786 | 9.005183585 | 7.681488156 | 7.530766858 | 7.630471003 | 9.803357467 | 7.305661745 | 5.751226389 | 5.851724643 | 5.177021723 | 7.196471721 | 5.588491509 |
| ENSMUSG00000026457 | Adipor1 | 4990 | 57.30106596 | 62.16626673 | 56.86555693 | 56.44456435 | 59.82248308 | 59.80091415 | 69.51834908 | 61.72014689 | 58.08206642 | 60.6533796 | 57.75121233 | 55.40179896 | 56.94425722 | 60.59138455 | 55.09779208 | 54.65044207 | 55.64609641 | 54.00593464 |
| ENSMUSG00000026458 | Ppfia4 | 6043 | 0.056270298 | 0.165379253 | 0.160097734 | 0.022618423 | 0 | 0.057400155 | 0 | 0.012640213 | 0.054524595 | 0.078025324 | 0.071152511 | 0.184728322 | 0.016326963 | 0.107598415 | 0.040908379 | 0 | 0.031711316 | 0 |
| ENSMUSG00000026459 | Myog | 1476 | 0 | 0 | 0 | 0 | 0 | 0 | 0 | 0 | 0 | 0 | 0 | 0 | 0 | 0 | 0 | 0 | 0 | 0 |
| ENSMUSG00000026463 | Atp2b4 | 11734 | 0.905598603 | 0.985135017 | 1.484103539 | 1.04447937 | 0.93933957 | 0.907945945 | 1.75396415 | 1.900832027 | 1.681297175 | 1.583209228 | 1.339522869 | 1.622050532 | 4.048631016 | 4.749693614 | 2.942466664 | 3.76249108 | 2.626073181 | 1.908019979 |
| ENSMUSG00000026464 | Zc3h11a | 4378 | 35.43716138 | 30.83991783 | 31.63394642 | 31.40777337 | 29.61795394 | 31.22795613 | 22.74855851 | 25.56047032 | 28.91899257 | 23.79075 | 27.07393147 | 29.60346472 | 27.30270342 | 30.29792453 | 26.99087764 | 24.73408811 | 29.93968374 | 28.63539384 |
| ENSMUSG00000026466 | Tor1aip1 | 6829 | 8.116378612 | 8.175781331 | 7.614811119 | 7.825904965 | 8.394365367 | 6.791822907 | 6.595646557 | 8.534427599 | 7.351934795 | 7.871108759 | 9.038694736 | 6.481449763 | 7.68621467 | 9.351386756 | 9.146516142 | 9.930101876 | 8.255671803 | 8.12771836 |
| ENSMUSG00000026468 | Lhx4 | 5596 | 0 | 0 | 0 | 0.024425149 | 0.02580339 | 0.026565082 | 0.016909464 | 0 | 0 | 0.008425787 | 0 | 0 | 0 | 0 | 0 | 0 | 0 | 0 |
| ENSMUSG00000026469 | Xpr1 | 14767 | 2.017750958 | 2.813108898 | 2.689420222 | 2.860099418 | 2.555388918 | 2.181165899 | 2.191499451 | 1.761293833 | 1.807331361 | 2.017961973 | 2.48467313 | 1.761366477 | 1.279482923 | 1.537967003 | 1.286240757 | 1.861763128 | 2.09189377 | 1.642785675 |
| ENSMUSG00000026470 | Stx6 | 2527 | 62.63920678 | 65.4525207 | 65.31479445 | 74.96748015 | 63.33147851 | 62.80862907 | 44.4668035 | 44.49482892 | 40.77905122 | 44.9489688 | 42.59476908 | 49.9624174 | 38.18489521 | 35.04902044 | 38.95153028 | 44.18496683 | 44.72665199 | 42.60103754 |
| ENSMUSG00000026471 | Mr1 | 2503 | 32.85957476 | 41.76422775 | 37.89871875 | 33.78397854 | 43.32450021 | 41.37643355 | 22.60725689 | 25.19203211 | 30.44149187 | 33.66292718 | 28.53518295 | 30.46112469 | 28.77530932 | 30.05969699 | 31.22626909 | 28.01042835 | 35.23324378 | 33.09776857 |
| ENSMUSG00000026473 | Glul | 3319 | 235.4497322 | 160.4318473 | 193.9605128 | 170.3151181 | 181.3757063 | 154.5107682 | 113.6416621 | 94.39357545 | 104.5981143 | 95.76465522 | 97.04693283 | 89.80284369 | 130.7541055 | 117.3486342 | 113.3880722 | 126.4547598 | 108.673932 | 115.3716155 |
| ENSMUSG00000026475 | Rgs16 | 2334 | 0.327803414 | 0.699370958 | 1.367889033 | 0.507535198 | 1.979719225 | 0.467078037 | 0.750029615 | 0.63817639 | 0.688206562 | 1.656537132 | 1.412370238 | 0.741339135 | 0.866584665 | 1.472520337 | 1.041513195 | 1.558529436 | 2.495971349 | 0.565646342 |
| ENSMUSG00000026478 | Lamc1 | 9472 | 2.683498233 | 3.007023285 | 2.022373102 | 2.659972289 | 2.94218579 | 3.029036396 | 2.192806804 | 2.173321885 | 2.169771048 | 2.827449266 | 2.537035907 | 2.710642398 | 2.874917476 | 1.912288227 | 2.422853318 | 2.518480618 | 3.192509318 | 1.782917627 |
| ENSMUSG00000026479 | Lamc2 | 5140 | 17.37419744 | 24.75783189 | 21.67397673 | 22.37277741 | 25.28330621 | 26.77197299 | 19.17360121 | 28.44368023 | 22.25193663 | 28.51975417 | 29.25054364 | 26.62646005 | 21.03804721 | 22.06545942 | 28.88116846 | 25.11983038 | 32.96508922 | 27.52595768 |
| ENSMUSG00000026480 | Ncf2 | 2581 | 0.905767021 | 1.548836616 | 0.449811983 | 1.288630855 | 0.74594225 | 1.075146486 | 0.366622851 | 0.414330596 | 0.015957581 | 0.16441547 | 0.518287035 | 0 | 0 | 0.071978438 | 0.159634079 | 0.074177852 | 0.029698797 | 0.021313098 |
| ENSMUSG00000026482 | Rgl1 | 8004 | 0.074346885 | 0.104050769 | 0.084611372 | 0.096768835 | 0.21047193 | 0.167156897 | 0.147778233 | 0.472394782 | 0.283015788 | 0.053018032 | 0.214879873 | 0.223151075 | 0.345150846 | 0.098644364 | 0.066919069 | 0.157869821 | 0.335187504 | 0.17181755 |
| ENSMUSG00000026483 | Fam129a | 7363 | 19.32735697 | 29.98296211 | 23.1585895 | 21.22428115 | 23.93195335 | 25.02874048 | 5.661071593 | 6.229672048 | 5.224528823 | 5.231851767 | 5.061044087 | 5.313972494 | 9.534064686 | 9.417495922 | 8.074676783 | 16.12126619 | 13.27340175 | 9.562903247 |
| ENSMUSG00000026484 | Rnf2 | 3021 | 24.42535107 | 27.96474669 | 26.72473425 | 30.49468111 | 27.65872873 | 33.05155848 | 17.77553478 | 25.49952852 | 23.25858851 | 20.7425636 | 26.66288646 | 28.36049059 | 21.76744314 | 30.68599749 | 27.37223524 | 30.53362239 | 28.44341531 | 28.67902783 |
| ENSMUSG00000026489 | Adck3 | 5708 | 5.927491261 | 5.322592769 | 5.474649721 | 4.789177751 | 7.057887195 | 8.004138115 | 7.294176149 | 7.313296396 | 8.687555182 | 8.582617499 | 9.282135416 | 9.133116478 | 8.677162744 | 6.794115797 | 7.918378909 | 5.990454171 | 9.077987634 | 8.683112326 |
| ENSMUSG00000026490 | Cdc42bpa | 19461 | 2.282406297 | 2.31089909 | 2.448380217 | 2.081278887 | 2.164093994 | 2.783060367 | 2.163726645 | 1.899709446 | 1.390449661 | 1.722632445 | 1.939377072 | 2.102301047 | 1.569110364 | 1.56555785 | 1.549742506 | 1.977393784 | 1.658226298 | 1.475502477 |
| ENSMUSG00000026491 | Ahctf1 | 10810 | 2.701300271 | 2.742689718 | 3.016073956 | 2.794354523 | 2.319771714 | 2.910822 | 3.26943304 | 3.745045934 | 3.387124192 | 3.03578996 | 2.603094407 | 2.519707987 | 2.569275618 | 3.196521833 | 2.831892324 | 2.493671379 | 3.0490856 | 3.124476552 |
| ENSMUSG00000026492 | Tfb2m | 3215 | 2.816050542 | 2.71476774 | 2.693269653 | 3.528678084 | 2.245657403 | 2.974705313 | 2.840232359 | 3.005498541 | 2.792740416 | 2.478528414 | 3.150323856 | 2.499986131 | 2.685252107 | 2.990334709 | 3.203853491 | 3.025136635 | 3.135246061 | 2.82317344 |
| ENSMUSG00000026494 | Kif26b | 7804 | 1.06753133 | 1.447087537 | 0.991768946 | 1.582142877 | 0.968312662 | 0.755609341 | 0.339506666 | 0.934744856 | 0.554149687 | 0.580018902 | 0.673404079 | 0.493500861 | 1.314843515 | 1.67232042 | 1.42547669 | 1.275699375 | 1.17375512 | 1.226497254 |
| ENSMUSG00000026495 | Efcab2 | 2529 | 5.76483803 | 7.007694632 | 5.508729435 | 5.080353698 | 7.44151133 | 5.858547782 | 10.32684807 | 9.091271747 | 10.78112829 | 9.210141324 | 7.178522824 | 7.879079297 | 7.978155068 | 9.017021316 | 8.227277867 | 7.509743484 | 8.713966279 | 13.18130434 |
| ENSMUSG00000026496 | Parp1 | 3823 | 12.3190518 | 12.26033015 | 10.22385889 | 13.64566983 | 13.23218723 | 11.34152286 | 14.05890747 | 12.34784446 | 14.61943549 | 12.69109957 | 13.49645701 | 14.48316434 | 11.04580727 | 9.706729224 | 14.36611928 | 10.68691554 | 11.86982358 | 11.56874753 |
| ENSMUSG00000026497 | Mixl1 | 2292 | 0 | 0.029068843 | 0 | 0 | 0 | 0 | 0 | 0 | 0 | 0 | 0 | 0 | 0.301329338 | 0 | 0 | 0 | 0 | 0 |
| ENSMUSG00000026499 | Acbd3 | 3462 | 24.5184392 | 26.62529703 | 25.89143609 | 26.95234512 | 23.52374779 | 28.29744491 | 25.21429596 | 25.78152641 | 27.9930305 | 24.92368196 | 23.1974885 | 26.39232803 | 21.57380784 | 25.78438358 | 23.0762036 | 24.92981776 | 26.04904004 | 20.11598183 |
| ENSMUSG00000026500 | Fam36a | 550 | 2.859439115 | 2.11991145 | 1.143374354 | 2.070956561 | 1.837764358 | 3.063259894 | 1.634437999 | 4.305325362 | 2.770729242 | 2.657585093 | 2.866497509 | 2.131143481 | 3.049609432 | 1.942207291 | 2.247357594 | 3.063248592 | 2.36926199 | 2.000331154 |
| ENSMUSG00000026502 | Pppde1 | 4869 | 19.9998682 | 20.38866806 | 16.87957036 | 19.19197 | 20.30457408 | 18.96010324 | 17.04383628 | 19.64920272 | 14.56627236 | 18.15723323 | 15.89554996 | 15.48715551 | 12.26965533 | 14.72780261 | 12.47301158 | 14.43860242 | 17.48258481 | 15.44412588 |
| ENSMUSG00000026504 | Sdccag8 | 4282 | 4.983091631 | 5.6480993 | 4.586560795 | 4.160291531 | 5.024514218 | 4.501637871 | 6.110208185 | 5.164269265 | 6.280895561 | 4.789947669 | 4.797578736 | 5.148805867 | 5.541488139 | 4.392773328 | 5.388339864 | 4.962934869 | 4.869104511 | 5.061557228 |
| ENSMUSG00000026509 | Capn2 | 4178 | 30.14428831 | 39.39660372 | 38.54367701 | 41.91877799 | 37.03784935 | 39.19860806 | 21.31222158 | 24.59927115 | 19.67646852 | 24.19605261 | 23.5673024 | 22.47054679 | 14.81846725 | 13.49524224 | 16.9421429 | 17.00073641 | 16.13593974 | 17.98526563 |
| ENSMUSG00000026510 | Trp53bp2 | 4343 | 15.41461023 | 16.31511075 | 15.13691636 | 17.70827604 | 14.23011513 | 16.00794041 | 14.36919722 | 12.97996453 | 10.5645357 | 14.64570543 | 14.79560653 | 16.82309517 | 13.23317606 | 11.3570391 | 12.52269256 | 13.33073875 | 15.71704692 | 15.41470939 |

| ENSMUSG00000026511 | Srp9 | 1602 | 30.03486845 | 34.51897903 | 33.48704442 | 34.41252412 | 23.82560266 | 22.48741405 | 28.32263364 | 39.4082654 | 34.60490026 | 40.67557547 | 25.88555803 | 23.34362917 | 28.33043938 | 43.31302532 | 31.17121457 | 32.84100785 | 21.77086778 | 21.73580809 |
| --- | --- | --- | --- | --- | --- | --- | --- | --- | --- | --- | --- | --- | --- | --- | --- | --- | --- | --- | --- | --- |
| ENSMUSG00000026514 | Cnih3 | 3597 | 0 | 0 | 0 | 0 | 0 | 0.013776128 | 0.013153372 | 0 | 0 | 0 | 0 | 0 | 0 | 0 | 0 | 0.12774181 | 0 | 0 |
| ENSMUSG00000026516 | Nvl | 3037 | 10.46884151 | 8.994591129 | 11.1496448 | 10.26136132 | 11.69620009 | 8.745559828 | 14.00530074 | 10.38752861 | 12.99199279 | 9.082371223 | 8.825074648 | 8.582783809 | 11.38678762 | 12.40242176 | 13.24093466 | 12.26761969 | 13.0993402 | 11.80966005 |
| ENSMUSG00000026519 | Tmem63a | 4524 | 12.91879245 | 12.70956132 | 12.53178107 | 13.06204123 | 15.61840494 | 19.08065029 | 14.81920116 | 13.54969183 | 14.99429519 | 16.37350902 | 20.30752545 | 13.06560271 | 12.74734998 | 7.69961661 | 11.43880508 | 11.13847023 | 12.72460167 | 14.05626158 |
| ENSMUSG00000026520 | Pycr2 | 1591 | 230.8530648 | 124.9181187 | 159.9883826 | 129.6957698 | 172.0164161 | 220.8849695 | 244.9494257 | 154.0658948 | 195.7330268 | 156.3885991 | 230.1065411 | 275.2543612 | 170.6617679 | 118.1390457 | 125.4951225 | 101.9959737 | 145.6447461 | 170.4210478 |
| ENSMUSG00000026523 | Wdr64 | 3967 | 0 | 0 | 0 | 0 | 0 | 0 | 0 | 0 | 0 | 0 | 0 | 0 | 0 | 0 | 0 | 0 | 0 | 0 |
| ENSMUSG00000026525 | Opn3 | 2208 | 0.539014913 | 0.60349446 | 0.701065657 | 1.09363014 | 0.893754017 | 0.583501392 | 0.814258061 | 1.608647384 | 1.380345184 | 0.960951833 | 1.557879081 | 1.137547471 | 1.005405923 | 1.283102052 | 1.175587872 | 1.005822115 | 0.746390746 | 0.722492797 |
| ENSMUSG00000026526 | Fh1 | 3571 | 34.62547103 | 33.35953787 | 35.23370273 | 35.7496629 | 37.38951449 | 35.06574006 | 39.45593891 | 34.42770746 | 34.71616142 | 37.72320336 | 36.36301796 | 34.85548238 | 37.61714107 | 34.98588493 | 33.47121637 | 31.50316565 | 32.85264487 | 35.44552074 |
| ENSMUSG00000026527 | Rgs7 | 1613 | 0.158109768 | 0 | 0 | 0 | 0 | 0 | 0 | 0 | 0 | 0 | 0 | 0 | 0 | 0.201555246 | 0 | 0 | 0 | 0.2728288 |
| ENSMUSG00000026531 | 1810030J14R | 797 | 0 | 0 | 0 | 0 | 0 | 0 | 0 | 0 | 0 | 0 | 0 | 0 | 0 | 0 | 0 | 0 | 0 | 0 |
| ENSMUSG00000026532 | Spna1 | 9640 | 0 | 0 | 0 | 0 | 0 | 0 | 0 | 0 | 0 | 0 | 0 | 0 | 0 | 0 | 0 | 0 | 0 | 0 |
| ENSMUSG00000026535 | Ifi202b | 1789 | 0 | 0.018620958 | 0 | 0.025467325 | 0.134521866 | 0.110794262 | 0.132232194 | 0.234833106 | 0.046044176 | 0 | 0.106819359 | 0.062398728 | 0.110300542 | 0.129804604 | 0.04606099 | 0 | 0.021423307 | 0.061497045 |
| ENSMUSG00000026536 | Mnda | 1644 | 0 | 0 | 0 | 0 | 0 | 0 | 0.028779002 | 0.023231388 | 0.075157875 | 0 | 0 | 0 | 0.060014498 | 0 | 0 | 0.232911237 | 0 | 0 |
| ENSMUSG00000026542 | Apcs | 981 | 0 | 0 | 0 | 0 | 0 | 0 | 0 | 0 | 0 | 0 | 0 | 0 | 0 | 0 | 0 | 0 | 0 | 0 |
| ENSMUSG00000026544 | Dusp23 | 1415 | 6.248110681 | 6.568408118 | 6.632130664 | 5.924545695 | 4.898231101 | 4.097293165 | 7.957892286 | 7.422551704 | 10.01283493 | 9.363496548 | 6.651354643 | 6.035191785 | 6.728664335 | 10.83148328 | 6.522366445 | 7.60400048 | 3.873258274 | 6.880997802 |
| ENSMUSG00000026546 | Ccdc19 | 2610 | 1.514552249 | 2.271913859 | 0.518949751 | 1.501245139 | 1.936341757 | 1.272043352 | 1.957766025 | 1.565742168 | 1.56224715 | 1.553624705 | 2.434509378 | 1.368659925 | 1.549891654 | 0.747376119 | 1.057664462 | 0.718865809 | 1.732759783 | 1.791484319 |
| ENSMUSG00000026547 | Tagln2 | 2077 | 377.3076218 | 431.6080806 | 371.7099396 | 404.7628262 | 372.1475985 | 373.9954991 | 369.4352561 | 425.2835257 | 419.7772519 | 422.2451038 | 388.9392262 | 388.9091329 | 382.565812 | 358.2925432 | 350.0842842 | 441.97353 | 371.4163236 | 338.0001059 |
| ENSMUSG00000026548 | Slamf9 | 1309 | 0.4870723 | 1.145210267 | 1.034727922 | 0.382865919 | 0.478009937 | 1.400650223 | 0.831315214 | 2.509202908 | 0.251712854 | 0.360203997 | 0.766443184 | 0.213199628 | 1.319035221 | 0.425767035 | 0.786889914 | 0.263266208 | 0.702697575 | 0 |
| ENSMUSG00000026553 | Copa | 7807 | 27.59270297 | 34.28151558 | 32.83972216 | 35.35985239 | 29.84586172 | 30.25718983 | 28.44699819 | 33.44220095 | 27.21148307 | 38.21822085 | 29.07987738 | 22.492132 | 25.69910434 | 35.96784326 | 34.97408875 | 35.67152397 | 33.55453303 | 28.32542706 |
| ENSMUSG00000026554 | Dcaf8 | 3901 | 66.96662698 | 73.5855446 | 72.31848988 | 74.15203037 | 72.10534784 | 70.02927973 | 70.86592745 | 61.53274779 | 61.29938747 | 74.39440619 | 69.54985624 | 73.78656036 | 63.15395921 | 67.51715447 | 71.31322876 | 76.75789538 | 75.15923387 | 61.19957016 |
| ENSMUSG00000026556 | Vangl2 | 7411 | 1.147083417 | 1.955351368 | 1.18796148 | 2.342296302 | 1.305426617 | 0.909347155 | 0.88100792 | 0.752407334 | 1.028134586 | 1.132482145 | 0.70266772 | 0.956495635 | 0.539183012 | 0.72069492 | 0.328011307 | 0.645840767 | 0.444752602 | 0.178143106 |
| ENSMUSG00000026558 | Uck2 | 1324 | 4.911851913 | 5.636018357 | 5.589992556 | 5.024103076 | 5.12583175 | 4.491184312 | 7.540011521 | 7.153863895 | 7.15475727 | 6.481441082 | 5.665157463 | 5.269605607 | 8.308925644 | 6.559701164 | 7.65527398 | 7.31686078 | 6.773680873 | 5.193457961 |
| ENSMUSG00000026560 | Fmo9 | 4319 | 0 | 0 | 0 | 0 | 0 | 0 | 0 | 0 | 0 | 0 | 0 | 0 | 0 | 0 | 0 | 0 | 0 | 0 |
| ENSMUSG00000026563 | Tada1 | 4260 | 7.044285041 | 5.943145448 | 6.688265112 | 7.753933602 | 6.123826908 | 6.397653396 | 6.452738605 | 7.539861602 | 6.738732743 | 7.083673729 | 5.75318162 | 6.144963792 | 5.419562743 | 6.74856575 | 4.942252362 | 6.022231681 | 4.993214737 | 4.442049933 |
| ENSMUSG00000026564 | Dusp27 | 4014 | 0.116481549 | 0 | 0 | 0 | 0 | 0 | 0 | 0 | 0 | 0 | 0 | 0 | 0 | 0 | 0 | 0 | 0 | 0 |
| ENSMUSG00000026565 | Pou2f1 | 17172 | 2.047040565 | 1.864296025 | 1.907109251 | 2.279113504 | 1.83591952 | 1.731402291 | 1.857019893 | 1.614703246 | 1.743687221 | 1.43604809 | 2.551225071 | 2.301274698 | 1.646120929 | 1.806699882 | 1.398818256 | 1.910962645 | 1.999788145 | 1.870796548 |
| ENSMUSG00000026566 | Mpzl1 | 2350 | 112.1413155 | 117.5023745 | 120.0075242 | 128.1137791 | 117.6467104 | 123.185987 | 98.71236799 | 102.7944448 | 105.4900589 | 108.366361 | 109.0285542 | 114.2202219 | 78.07038313 | 66.24705571 | 93.2031792 | 87.286291 | 97.59343007 | 103.0659987 |
| ENSMUSG00000026567 | Adcy10 | 6165 | 0.013789189 | 0.081053271 | 0.039232385 | 0.01478055 | 0.062458295 | 0 | 0.023023201 | 0.055755332 | 0.0334035 | 0.053536889 | 0.007749385 | 0.108643625 | 0.008001933 | 0.030134039 | 0.073514536 | 0.055898697 | 0.049734043 | 0.008922807 |
| ENSMUSG00000026568 | Brp44 | 4086 | 35.46259057 | 28.69833276 | 31.50317285 | 30.36287903 | 31.8052359 | 30.80370614 | 45.92317294 | 36.85576179 | 41.15627603 | 39.04992164 | 39.54354126 | 39.13653287 | 44.4904834 | 43.57969433 | 35.31273831 | 35.48862708 | 36.46907547 | 39.01526953 |
| ENSMUSG00000026571 | Dcaf6 | 3278 | 8.571055935 | 9.07517222 | 7.688410407 | 9.089970407 | 9.088975214 | 9.629375012 | 5.614591856 | 7.27030479 | 5.79224642 | 6.400873383 | 6.004662262 | 5.806327316 | 5.508078622 | 7.792632112 | 5.920052723 | 6.226020056 | 7.085337377 | 4.581295343 |
| ENSMUSG00000026572 | Tbx19 | 3549 | 0 | 0 | 0 | 0 | 0 | 0 | 0 | 0 | 0 | 0 | 0 | 0 | 0 | 0 | 0 | 0 | 0 | 0 |
| ENSMUSG00000026573 | Xcl1 | 520 | 0 | 0 | 0 | 0 | 0 | 0 | 0 | 0 | 0 | 0 | 0 | 0 | 0 | 0 | 0 | 0 | 0 | 0 |
| ENSMUSG00000026574 | Dpt | 1632 | 0 | 0 | 0 | 0 | 0 | 0 | 0 | 0.046804415 | 0 | 0 | 0 | 0 | 0 | 0 | 0 | 0 | 0 | 0 |
| ENSMUSG00000026575 | Nme7 | 1202 | 12.6596115 | 14.02356445 | 11.95252788 | 9.362377663 | 11.61252734 | 9.935281857 | 11.41487262 | 10.83494943 | 13.12349046 | 9.492903651 | 10.96995719 | 11.42317221 | 7.18226749 | 7.264133469 | 8.020935173 | 10.92652108 | 11.67007044 | 12.31069027 |
| ENSMUSG00000026576 | Atp1b1 | 2416 | 283.6024162 | 303.373468 | 292.9641954 | 318.8141621 | 324.1135208 | 316.801127 | 381.3801416 | 412.6549968 | 410.8764085 | 434.1924032 | 456.630522 | 429.4987513 | 366.7223652 | 303.9242639 | 366.3971144 | 435.4764154 | 441.4504099 | 379.4171169 |
| ENSMUSG00000026577 | Blzf1 | 6350 | 5.395145178 | 7.507204981 | 5.142068187 | 4.792878365 | 5.821309823 | 6.188239012 | 6.936709304 | 8.354212117 | 8.548634295 | 7.603514982 | 8.012650517 | 7.68234474 | 7.085150954 | 7.584648602 | 7.876958874 | 7.043059755 | 8.431785319 | 7.69261209 |
| ENSMUSG00000026578 | 4930455F23R | 1903 | 15.43409177 | 14.10940239 | 13.09110254 | 13.19187358 | 14.34093574 | 13.43626407 | 17.67699144 | 13.60717227 | 17.81214003 | 15.41131762 | 16.26808886 | 14.87048919 | 14.38739574 | 13.05706083 | 15.67521096 | 15.63415762 | 15.2862109 | 15.69624012 |
| ENSMUSG00000026579 | F5 | 7405 | 0 | 0.085475353 | 0.104520795 | 0.073832888 | 0.006499922 | 0.100376908 | 0.14056434 | 0.072207108 | 0.161297631 | 0.114613458 | 0.058065446 | 0.188439104 | 0.046633818 | 0.081535873 | 0.011128037 | 0.046538213 | 0.005175732 | 0.037143219 |
| ENSMUSG00000026580 | Selp | 4396 | 0 | 0.015156003 | 0.077027915 | 0 | 0 | 0.04508893 | 0.182965319 | 0.139007834 | 0.10305998 | 0.160887295 | 0.032603475 | 0.139666126 | 0.168330018 | 0.137346027 | 0.018745021 | 0.026130988 | 0.087184479 | 0 |
| ENSMUSG00000026581 | Sell | 2365 | 0.125808132 | 0 | 0 | 0 | 0.020351765 | 0 | 1.620434247 | 0.985089449 | 0.783675776 | 0 | 2.24229192 | 0.212406327 | 0.604915688 | 1.610323537 | 0.958175718 | 0.615240203 | 0 | 0 |
| ENSMUSG00000026582 | Sele | 2898 | 0 | 0 | 0 | 0 | 0 | 0 | 0 | 0 | 0 | 0 | 0 | 0 | 0 | 0 | 0 | 0 | 0 | 0 |

| ENSMUSG00000026584 | Scyl3 | 5445 | 7.322287441 | 6.999072725 | 6.591948489 | 7.254623588 | 7.646301923 | 8.964084954 | 6.65592508 | 7.07032904 | 6.951406425 | 7.481764484 | 7.457982499 | 6.919297015 | 7.610433519 | 6.91756747 | 7.816566985 | 7.229154161 | 10.13588017 | 8.718615078 |
| --- | --- | --- | --- | --- | --- | --- | --- | --- | --- | --- | --- | --- | --- | --- | --- | --- | --- | --- | --- | --- |
| ENSMUSG00000026585 | Kifap3 | 3991 | 12.13064282 | 13.37190091 | 12.54487695 | 13.21966659 | 14.74952209 | 11.94430712 | 12.82694027 | 11.52183726 | 15.43849351 | 11.53071571 | 15.64567044 | 12.53090295 | 12.70689325 | 13.10349346 | 10.4371769 | 12.35737969 | 11.47580175 | 14.80325247 |
| ENSMUSG00000026586 | Prrx1 | 8165 | 0.156173335 | 0.248877716 | 0.148112463 | 0.12834097 | 0.017684724 | 0.200274367 | 0 | 0.046775753 | 0.010088553 | 0.011549468 | 0 | 0 | 0.012083752 | 0 | 0 | 0.079723249 | 0 | 0 |
| ENSMUSG00000026587 | Astn1 | 7342 | 0 | 0.004537305 | 0 | 0 | 0 | 0 | 0 | 0 | 0 | 0 | 0 | 0.114033634 | 0 | 0 | 0 | 0 | 0 | 0 |
| ENSMUSG00000026589 | Sec16b | 5431 | 0 | 0.0736061 | 0 | 0.058723497 | 0 | 0.264597546 | 0.052269577 | 0 | 0 | 0 | 0.052780289 | 0 | 0 | 0.025654992 | 0 | 0 | 0 | 0 |
| ENSMUSG00000026592 | 1700057K13R | 1321 | 0.80441287 | 0.126089683 | 0.146475489 | 0.655306467 | 0.255051829 | 0.375115319 | 0.680500303 | 0.289117354 | 0.249426288 | 0.356931894 | 0.036165752 | 0.338020667 | 0 | 0.281266237 | 0.280707042 | 0 | 0.145065469 | 0.124926056 |
| ENSMUSG00000026594 | Ralgps2 | 7007 | 8.844376573 | 7.079049449 | 8.588079315 | 8.992568049 | 8.42841021 | 7.651785034 | 7.812297634 | 7.554541135 | 7.564870242 | 7.48274326 | 8.454538107 | 6.969987835 | 12.12352812 | 10.91671356 | 11.25442814 | 10.61776084 | 10.54561159 | 12.2861784 |
| ENSMUSG00000026596 | Abl2 | 10870 | 1.25521265 | 1.379098657 | 1.027993147 | 1.073010796 | 1.275252439 | 1.071287248 | 0.618068115 | 1.672454788 | 0.935884949 | 1.184189695 | 1.182287381 | 1.36586626 | 0.857749068 | 1.12371619 | 0.587510687 | 1.359721661 | 0.588821673 | 1.03236962 |
| ENSMUSG00000026600 | Soat1 | 4769 | 27.32666591 | 21.48678184 | 28.56361113 | 24.99217697 | 24.5352708 | 27.7740526 | 39.63391745 | 34.17214952 | 31.74703962 | 40.98126756 | 31.21551072 | 39.27812196 | 35.78089785 | 33.26756186 | 38.37645445 | 33.49725605 | 44.49836569 | 34.84640625 |
| ENSMUSG00000026601 | 9430070O13R | 2143 | 0 | 0.062179924 | 0 | 0.170083227 | 0 | 0 | 0 | 0.17821933 | 0.076876371 | 0.132013168 | 0 | 0 | 0.276240321 | 0.476794178 | 0 | 0 | 0.017884413 | 0 |
| ENSMUSG00000026602 | Nphs2 | 3108 | 0 | 0 | 0 | 0 | 0 | 0 | 0 | 0 | 0 | 0 | 0 | 0 | 0 | 0 | 0 | 0.073920092 | 0 | 0 |
| ENSMUSG00000026603 | Smyd2 | 3076 | 14.48160744 | 15.43266393 | 13.06840173 | 12.35302698 | 13.34737675 | 16.01281443 | 18.50362573 | 15.0360856 | 18.37057855 | 16.86143485 | 18.34272626 | 13.86318798 | 19.30937204 | 21.168599 | 19.78371198 | 25.00839736 | 19.86089638 | 16.52419201 |
| ENSMUSG00000026604 | Ptpn14 | 10969 | 0.658754665 | 0.883768094 | 0.837904163 | 0.826570136 | 1.123326872 | 0.790566905 | 0.634056323 | 1.096782456 | 0.698394743 | 0.623288538 | 0.744782378 | 0.81415863 | 0.899478845 | 0.995018738 | 1.03670612 | 0.82732008 | 1.23340166 | 0.661974846 |
| ENSMUSG00000026605 | Cenpf | 13304 | 0.565500312 | 0.803776236 | 0.821739166 | 0.236298261 | 0.369021062 | 0.379914223 | 0.697029846 | 0.614339606 | 1.114487799 | 0.393395074 | 0.53865332 | 0.318850748 | 0.689697581 | 0.666778464 | 1.121088637 | 0.457622262 | 0.656824691 | 0.442421409 |
| ENSMUSG00000026608 | Kctd3 | 4701 | 3.236939591 | 4.379359419 | 5.217055922 | 5.475854084 | 4.812168501 | 4.796105888 | 3.774144777 | 3.63969289 | 3.110234651 | 3.991912337 | 4.308994341 | 4.143728195 | 3.400030036 | 3.200996451 | 3.058783877 | 3.168484637 | 3.391563393 | 4.949766463 |
| ENSMUSG00000026609 | Ush2a | 17277 | 0 | 0 | 0.00559976 | 0 | 0 | 0.014340665 | 0 | 0.030948292 | 0 | 0.008187307 | 0 | 0 | 0 | 0.045699455 | 0 | 0 | 0.035493474 | 0 |
| ENSMUSG00000026610 | Esrrg | 6196 | 13.19197119 | 19.19416274 | 17.87853204 | 20.3318734 | 19.1642117 | 20.78559628 | 19.11291726 | 21.83303572 | 19.07103191 | 22.09903845 | 22.49956889 | 19.25982676 | 11.4014373 | 12.61546144 | 17.03653747 | 11.04346601 | 17.56725036 | 14.10740326 |
| ENSMUSG00000026611 | Spata17 | 2857 | 0.327306221 | 0.396443263 | 0.253973733 | 0.446520561 | 0.033694031 | 0.294853507 | 0.38088611 | 0.481248333 | 1.153280105 | 0.544617854 | 0.384607646 | 0.859604184 | 0.43167586 | 0.406406085 | 0.634535688 | 0.428876247 | 0.563424736 | 0.442845451 |
| ENSMUSG00000026614 | Slc30a10 | 5832 | 0 | 0 | 0 | 0 | 0 | 0 | 0 | 0 | 0 | 0 | 0 | 0 | 0 | 0 | 0 | 0 | 0 | 0 |
| ENSMUSG00000026615 | Eprs | 7273 | 21.51265681 | 25.81943967 | 21.28355522 | 23.76090213 | 25.78330463 | 25.018237 | 17.66175214 | 19.0778218 | 19.97882959 | 21.17340806 | 21.96610218 | 19.91497929 | 17.1606972 | 19.87267977 | 20.0484403 | 16.32602414 | 19.11835627 | 19.02968686 |
| ENSMUSG00000026616 | Cr2 | 7309 | 0 | 0.004557791 | 0.079420217 | 0 | 0.019755886 | 0.020339062 | 0 | 0.041803149 | 0.29302214 | 0.070961518 | 0 | 0.084002229 | 0 | 0 | 0 | 0.01571649 | 0 | 0 |
| ENSMUSG00000026617 | Bpnt1 | 2286 | 67.45790841 | 68.19962596 | 61.15463811 | 67.48455648 | 64.36539399 | 65.46336632 | 70.782748 | 69.81891936 | 71.34672022 | 68.84910213 | 68.54849685 | 65.67984793 | 49.95773775 | 52.07182676 | 59.15288995 | 53.68390058 | 58.78040111 | 55.9475823 |
| ENSMUSG00000026618 | Iars2 | 9583 | 8.902002326 | 11.14137806 | 11.59490757 | 10.46910358 | 11.76803685 | 11.12777655 | 9.637310787 | 10.71682877 | 10.16446935 | 9.53051364 | 12.667867 | 8.334781714 | 10.56340334 | 7.536320139 | 9.678044695 | 8.358963861 | 9.378604428 | 11.19354671 |
| ENSMUSG00000026620 | Mark1 | 4528 | 8.176238587 | 11.16070241 | 9.198235344 | 9.921199142 | 10.25779734 | 9.597559043 | 5.537923991 | 7.329769808 | 5.730456036 | 6.154166437 | 6.161787931 | 5.657992298 | 6.656758272 | 7.005590037 | 8.890000023 | 6.849689045 | 8.371181015 | 6.900435649 |
| ENSMUSG00000026621 | Mosc1 | 2714 | 0.062645801 | 0 | 0.142589625 | 0.13429932 | 0.035469362 | 0.018258192 | 0 | 0 | 0 | 0 | 0.176031535 | 0 | 0.090884151 | 0.017112781 | 0.060724474 | 0 | 0.042365103 | 0 |
| ENSMUSG00000026622 | Nek2 | 4667 | 0.054645609 | 0.463967886 | 0.342045533 | 0.068336685 | 0.010313247 | 0.010617685 | 0.182478727 | 0.270055556 | 0.123550722 | 0 | 0.01023676 | 0.011959645 | 0.073992591 | 0.746369521 | 0.626807471 | 0.295363588 | 0.607702158 | 0.011786824 |
| ENSMUSG00000026623 | Lpgat1 | 7779 | 16.34856494 | 14.86425708 | 13.86098459 | 16.9792348 | 10.61142166 | 13.82304048 | 11.95741442 | 15.34275068 | 12.18811663 | 12.86203783 | 10.07210849 | 10.58337862 | 9.893018505 | 9.91093779 | 9.76146902 | 10.10056902 | 7.045456302 | 10.47287403 |
| ENSMUSG00000026626 | Ppp2r5a | 3068 | 58.39612678 | 63.29232738 | 67.73555619 | 76.3013839 | 62.56522542 | 64.46054749 | 65.63323387 | 67.94463244 | 66.12932746 | 75.8591497 | 76.7700604 | 71.1886205 | 59.15649402 | 55.46647203 | 67.45613274 | 68.294069 | 69.60695122 | 65.8746604 |
| ENSMUSG00000026627 | Tmem206 | 2362 | 21.77445512 | 20.33750782 | 22.118295 | 22.39473856 | 22.88406024 | 22.4896403 | 21.39286245 | 20.64847497 | 17.7161304 | 23.57535163 | 24.15042355 | 18.4555599 | 19.42365922 | 17.02818784 | 22.67655489 | 17.86462716 | 21.59707925 | 23.80156946 |
| ENSMUSG00000026628 | Atf3 | 2234 | 99.94614578 | 47.13610503 | 69.2906433 | 85.02416913 | 122.4627816 | 86.88364254 | 116.4603498 | 135.9471728 | 55.52989502 | 81.70115144 | 228.4173373 | 59.66329555 | 148.6361393 | 218.9982169 | 172.6629213 | 580.0838482 | 519.103443 | 469.4734238 |
| ENSMUSG00000026630 | Batf3 | 709 | 1.258968542 | 0.563828957 | 0.818733941 | 0.385565961 | 0.271548229 | 0.279564082 | 1.401362846 | 1.993115499 | 1.800820858 | 1.529571474 | 0.943370125 | 2.204285687 | 1.669909753 | 1.768674696 | 0.348673252 | 3.942474387 | 2.432557631 | 1.629324741 |
| ENSMUSG00000026632 | Tatdn3 | 3228 | 5.727927996 | 5.098069933 | 6.45879541 | 5.659844727 | 6.00903508 | 5.833345341 | 6.00935513 | 7.039801559 | 5.933001806 | 5.594398807 | 5.831268166 | 6.691654715 | 6.097718399 | 7.553638748 | 6.38190147 | 7.39003978 | 6.38771615 | 7.276607364 |
| ENSMUSG00000026634 | Angel2 | 5579 | 14.86424062 | 14.87406695 | 14.43662449 | 14.52824841 | 15.80528485 | 14.45991222 | 16.12990058 | 13.65041233 | 15.69502286 | 17.41846198 | 18.15431295 | 15.63718957 | 14.01525164 | 15.70058233 | 16.74205542 | 18.43494739 | 15.5462287 | 16.24932925 |
| ENSMUSG00000026637 | Traf5 | 1967 | 0.36735536 | 0.779050907 | 0.393480674 | 0.208464361 | 0.440454817 | 0.025192035 | 0.817809397 | 1.067911608 | 0.481591186 | 0.647213517 | 0.194305881 | 0.510768646 | 0.501595498 | 0.779183978 | 1.319622787 | 0.136265507 | 0.175361806 | 0.783047783 |
| ENSMUSG00000026638 | Irf6 | 4427 | 50.1958573 | 68.84564473 | 53.33435773 | 64.73435032 | 62.74437124 | 62.8391792 | 40.00256545 | 51.05548621 | 43.679849 | 50.49502691 | 49.18867421 | 54.61790159 | 32.34934625 | 32.36503492 | 36.74356057 | 46.19609987 | 49.25193203 | 49.87724292 |
| ENSMUSG00000026639 | Lamb3 | 4339 | 0.323270525 | 0.483685719 | 0 | 0.388513169 | 0.11092861 | 0.41113123 | 0.010904051 | 0.255261505 | 0.009492168 | 0.54333606 | 0.033031776 | 0.540276061 | 0.568471045 | 0 | 1.186954249 | 0.388289174 | 0.105995751 | 0.22820095 |
| ENSMUSG00000026640 | Plxna2 | 15059 | 0.053628949 | 0.02875806 | 0.077094411 | 0.030255026 | 0.019177339 | 0.003290573 | 0.003141821 | 0.010144738 | 0.128545471 | 0.018786388 | 0 | 0.011119396 | 0.062242276 | 0.052430406 | 0.049248158 | 0.162733174 | 0.027995834 | 0.215521435 |
| ENSMUSG00000026641 | Usf1 | 2911 | 7.271582845 | 9.166481714 | 8.358600401 | 10.01685619 | 7.655472572 | 7.421845359 | 8.012762182 | 8.501778344 | 9.493697038 | 8.001527794 | 6.548336803 | 6.768440018 | 10.42223604 | 10.6896388 | 10.55869485 | 10.79924036 | 6.688340373 | 5.706887747 |
| ENSMUSG00000026643 | Nmt2 | 5046 | 5.534264893 | 6.291555329 | 5.052090863 | 5.471659308 | 5.007780405 | 4.595854006 | 5.475744054 | 4.836493289 | 5.599276618 | 4.999133223 | 5.444035102 | 5.055045145 | 6.149380889 | 5.393625686 | 5.323704804 | 6.214825101 | 5.28638578 | 4.6331491 |
| ENSMUSG00000026644 | Acbd7 | 527 | 3.710129217 | 2.52849292 | 3.396244067 | 1.123896729 | 1.095983082 | 2.444726894 | 121.8279038 | 78.70388807 | 69.94673929 | 45.45077278 | 28.28422589 | 58.25164027 | 493.9744354 | 579.8019942 | 269.6473743 | 75.34603392 | 141.5236694 | 319.4077165 |
| ENSMUSG00000026645 | Olah | 2093 | 0 | 0 | 0 | 0 | 0 | 0 | 0.113025989 | 0.109486103 | 0.019678221 | 0 | 0.091304268 | 0 | 0.49496907 | 0.221901994 | 0 | 0.109767628 | 0 | 0.394236312 |

| ENSMUSG00000026646 | Suv39h2 | 5552 | 0.091869977 | 0.348009342 | 0.322374032 | 0.188743519 | 0.554834855 | 0.160653675 | 0.323825972 | 0.440258241 | 0.422844272 | 0.178343798 | 0.378619988 | 0.422236641 | 0.284333817 | 0.317881002 | 0.400735594 | 0.310352546 | 0.276126058 | 0.128803744 |
| --- | --- | --- | --- | --- | --- | --- | --- | --- | --- | --- | --- | --- | --- | --- | --- | --- | --- | --- | --- | --- |
| ENSMUSG00000026648 | Dclre1c | 7507 | 2.117615004 | 1.295772627 | 1.256539019 | 2.045300645 | 1.352848794 | 1.161753178 | 1.777297916 | 1.663636018 | 1.656897264 | 1.130561687 | 1.78193531 | 1.241669862 | 1.445720235 | 1.286848299 | 1.267824618 | 1.285364735 | 1.347827678 | 1.267693548 |
| ENSMUSG00000026649 | 1700009P17R | 1871 | 0.090871568 | 0.676584704 | 0.387815583 | 0.267862901 | 0.308702878 | 0.026484625 | 0.202299001 | 0.571559202 | 0.308183442 | 0.201606428 | 0.025534451 | 0.11932798 | 0.158199628 | 0.695047807 | 0.286274841 | 0.368375699 | 0.307265876 | 0.705621893 |
| ENSMUSG00000026650 | Meig1 | 1373 | 0 | 0 | 0.105695988 | 0.232285004 | 0.17528013 | 0.036090847 | 0.447971468 | 0.361617794 | 0.209982236 | 0.137365487 | 0.139184147 | 0.406523398 | 0.826390457 | 1.319242104 | 0.450126248 | 0.167329675 | 1.032828103 | 0.681103288 |
| ENSMUSG00000026655 | Fam107b | 3751 | 29.20176983 | 25.41762284 | 24.82512555 | 24.71786863 | 22.26309986 | 24.02997131 | 18.70559927 | 14.66197266 | 15.39416025 | 16.34121947 | 17.06703395 | 11.51728148 | 14.86138804 | 17.63166634 | 18.31058216 | 17.83356202 | 22.95899471 | 20.6925752 |
| ENSMUSG00000026656 | Fcgr2b | 4205 | 0.566061797 | 0.316888411 | 0.41413724 | 0.335884037 | 0.171695328 | 0.542075088 | 1.428944167 | 0.908261651 | 1.6455017 | 0.672780546 | 1.431544535 | 0.530945661 | 0.809489249 | 1.535250451 | 0.607490241 | 0.97433888 | 0.236975914 | 0.510191477 |
| ENSMUSG00000026657 | Frmd4a | 20281 | 0.299701207 | 0.295661997 | 0.350619248 | 0.505460035 | 0.422438855 | 0.280980443 | 0.606542898 | 0.278707931 | 0.363511973 | 0.481248241 | 0.287389425 | 0.45685124 | 0.398916939 | 0.389305007 | 0.562735121 | 0.477664991 | 0.472440916 | 0.149179077 |
| ENSMUSG00000026659 | Dusp12 | 2896 | 2.891408728 | 2.634203307 | 2.822902842 | 2.753170842 | 2.127377842 | 1.865071809 | 3.283787453 | 2.66397282 | 2.133279477 | 2.409635387 | 3.26638183 | 2.717544346 | 2.265588742 | 3.175389953 | 3.514082978 | 2.803041702 | 3.54677057 | 3.077166882 |
| ENSMUSG00000026662 | Sephs1 | 5915 | 14.17078734 | 13.56733088 | 14.00910524 | 14.65810099 | 14.52501838 | 13.56312353 | 10.83032414 | 11.76442218 | 11.51690568 | 11.1838439 | 10.92806743 | 12.03127131 | 9.607839172 | 13.293295 | 13.24161585 | 12.1118726 | 12.41473962 | 12.76880871 |
| ENSMUSG00000026663 | Atf6 | 7447 | 8.025013763 | 8.311448564 | 7.612968655 | 7.311057873 | 8.260050819 | 8.79000417 | 8.208403542 | 8.062099715 | 5.619108368 | 7.06595741 | 6.973463122 | 7.397617695 | 5.6506144 | 6.529738082 | 6.921330256 | 7.434969559 | 7.956553647 | 5.938944784 |
| ENSMUSG00000026664 | Phyh | 1948 | 117.5656511 | 111.3793019 | 120.9586583 | 118.2295067 | 121.3181444 | 116.4540115 | 147.3785091 | 129.379704 | 142.3133663 | 155.6118436 | 144.0606294 | 126.6167418 | 144.52631 | 139.8329428 | 146.0667069 | 136.0810447 | 145.1598658 | 153.816532 |
| ENSMUSG00000026667 | Uhmk1 | 8092 | 8.819351279 | 8.571112922 | 10.02501364 | 9.543496064 | 10.19502189 | 9.467193044 | 11.37796258 | 9.095002402 | 8.174189856 | 6.980541585 | 8.855961161 | 7.532217442 | 7.157151617 | 7.599106735 | 8.615055928 | 6.62940447 | 8.62010138 | 6.444467767 |
| ENSMUSG00000026668 | Ucma | 1063 | 0 | 0 | 0 | 0 | 0 | 0 | 0 | 0 | 0 | 0 | 0 | 0 | 0 | 0 | 0 | 0 | 0 | 0 |
| ENSMUSG00000026669 | Mcm10 | 7218 | 0.005888775 | 0.027691516 | 0.026807166 | 0.088369995 | 0.040009912 | 0.075516773 | 0.072103002 | 0.026456361 | 0.262479873 | 0.032661889 | 0.033094319 | 0.007732843 | 0.157195081 | 0.379634408 | 0.011416336 | 0.153841454 | 0.069027689 | 0.152422019 |
| ENSMUSG00000026670 | Uap1 | 4337 | 28.99015695 | 27.26019405 | 28.1295927 | 30.41254862 | 30.95225619 | 28.3697112 | 24.87270186 | 28.39993031 | 26.64730533 | 25.63554491 | 27.96878478 | 23.5771948 | 22.44221185 | 23.40944778 | 23.65503209 | 21.78070751 | 26.14883849 | 22.55157753 |
| ENSMUSG00000026672 | Optn | 4250 | 10.3412593 | 12.62754649 | 11.52997324 | 11.81371079 | 12.25382151 | 14.50437661 | 13.62605153 | 14.02784474 | 12.96648426 | 15.23245072 | 11.33109603 | 11.6753233 | 15.39155819 | 16.03140616 | 16.73267893 | 21.1544344 | 16.46678074 | 16.07560248 |
| ENSMUSG00000026673 | Sh2d1b1 | 2484 | 0 | 0 | 0 | 0 | 0 | 0.059846297 | 0 | 0 | 0.066322892 | 0.056945294 | 0 | 0 | 0 | 0 | 0 | 0 | 0 | 0 |
| ENSMUSG00000026674 | Ddr2 | 8591 | 0.01484292 | 0.034898853 | 0.005630722 | 0.026516729 | 0 | 0.005767982 | 0.110144754 | 0.026673777 | 0.023970734 | 0.021953534 | 0 | 0 | 0 | 0.108122657 | 0.014387693 | 0 | 0.013383645 | 0 |
| ENSMUSG00000026675 | Hsd17b7 | 5352 | 7.187441014 | 8.514955025 | 10.43935493 | 8.572490965 | 8.75044128 | 8.906900169 | 10.50214174 | 11.41062247 | 10.36588878 | 10.76572485 | 11.9705193 | 8.854166206 | 11.77993092 | 12.48748912 | 12.34814941 | 14.82401692 | 14.70177271 | 11.71718641 |
| ENSMUSG00000026676 | Ccdc3 | 2667 | 0 | 0 | 0 | 0.239165587 | 0 | 0.204379479 | 0.12418026 | 0 | 0 | 0 | 0 | 0 | 0.092485784 | 0 | 0 | 0.043071549 | 0.071852825 | 0 |
| ENSMUSG00000026678 | Rgs5 | 4642 | 0 | 0 | 0 | 0 | 0 | 0 | 0 | 0 | 0 | 0 | 0 | 0 | 0 | 0 | 0 | 0 | 0 | 0 |
| ENSMUSG00000026679 | Enkur | 1556 | 0.437071219 | 1.391605478 | 0.963740001 | 0.907707181 | 0.618662258 | 0.605078366 | 0.15203303 | 0.441814424 | 1.244065707 | 0.424235119 | 0.36844441 | 1.327236193 | 0.158521585 | 0.567119319 | 0.238312341 | 0.590600628 | 0.788201479 | 0.070705793 |
| ENSMUSG00000026683 | Nuf2 | 3128 | 0.244595003 | 0.340796872 | 0.123717469 | 0.072827756 | 0 | 0.09505 | 0.166380904 | 0 | 0.724187457 | 0 | 0 | 0 | 0.220795026 | 0.311804935 | 0 | 0.012241243 | 0.14703183 | 0 |
| ENSMUSG00000026686 | Lmx1a | 3384 | 0 | 0 | 0 | 0 | 0 | 0 | 0 | 0 | 0 | 0 | 0 | 0 | 0 | 0 | 0 | 0 | 0 | 0 |
| ENSMUSG00000026687 | Aldh9a1 | 2822 | 13.73661252 | 12.60743126 | 13.9532437 | 18.09848714 | 16.9706818 | 18.68324186 | 20.65528719 | 13.62854331 | 13.76289311 | 15.87284482 | 19.85826587 | 19.44252173 | 14.57931219 | 17.98844349 | 16.55654301 | 17.62561978 | 18.45692328 | 16.41306444 |
| ENSMUSG00000026688 | Mgst3 | 1091 | 24.11613562 | 20.12208734 | 19.68638632 | 20.1287107 | 19.94099862 | 16.89607414 | 25.7162407 | 21.07408455 | 23.40571931 | 21.39284887 | 20.97544007 | 18.8780747 | 20.30250308 | 19.92285325 | 16.35222154 | 17.12723777 | 13.5951209 | 14.6220357 |
| ENSMUSG00000026691 | Fmo3 | 2284 | 10.99849345 | 4.798573642 | 5.951384426 | 7.859479627 | 10.22065805 | 11.56375087 | 27.75787643 | 6.738852088 | 9.521226061 | 12.32441762 | 11.6718156 | 10.70370412 | 15.18403583 | 13.99016293 | 13.22274101 | 3.772060712 | 15.57215566 | 21.6038392 |
| ENSMUSG00000026692 | Fmo4 | 2976 | 0.185674492 | 0 | 0 | 0 | 0.177906976 | 0 | 0 | 0 | 0 | 0 | 0 | 0 | 0 | 0 | 0 | 0 | 0 | 0 |
| ENSMUSG00000026694 | Mettl13 | 7405 | 0.918410286 | 1.214649755 | 1.659267618 | 1.544337897 | 1.624980542 | 1.612722322 | 1.514261297 | 1.604029325 | 1.090149506 | 1.267115456 | 2.096807766 | 1.824090526 | 2.11184575 | 0.821630715 | 1.246340111 | 0.81183327 | 1.920196644 | 1.701159411 |
| ENSMUSG00000026696 | Vamp4 | 4743 | 16.74935146 | 15.42380681 | 18.79663007 | 16.45500083 | 16.92684982 | 17.16532601 | 18.51409068 | 17.18376275 | 21.46596394 | 17.24783263 | 17.56684115 | 18.64052489 | 16.78720525 | 16.8522611 | 19.17179714 | 17.03419389 | 21.47824102 | 14.79899224 |
| ENSMUSG00000026697 | Myoc | 2076 | 0 | 0.160466735 | 0.116506576 | 0 | 0.023184934 | 0.047738664 | 0.022790308 | 0.036794222 | 0 | 0.068136854 | 0 | 0.053772315 | 0.071288898 | 0.089487644 | 0 | 0.055333248 | 0.073846429 | 0.079492929 |
| ENSMUSG00000026698 | Pigc | 4088 | 9.576141664 | 7.928925165 | 9.359946798 | 7.99101487 | 7.135016082 | 7.442607245 | 8.830619868 | 8.370937513 | 9.742505074 | 8.800388108 | 8.870154962 | 8.779224815 | 7.916276354 | 7.668727737 | 6.440262773 | 7.118606073 | 5.475185272 | 8.073743648 |
| ENSMUSG00000026700 | Tnfsf4 | 1594 | 0 | 0 | 0 | 0 | 0 | 0 | 0.207772116 | 0 | 0 | 0 | 0 | 0 | 0.185691031 | 0 | 0.206783217 | 0 | 0 | 0 |
| ENSMUSG00000026701 | Prdx6 | 3948 | 195.6336255 | 185.0181944 | 190.6882606 | 182.6711679 | 186.8708526 | 178.1915295 | 206.3757963 | 169.215173 | 189.9921036 | 182.2252863 | 152.521676 | 167.5883648 | 247.0719352 | 231.96163 | 223.5299204 | 196.0502376 | 215.6679362 | 238.3031287 |
| ENSMUSG00000026705 | Klhl20 | 10239 | 1.13745661 | 1.711356808 | 1.351189538 | 1.931193793 | 1.527773727 | 1.384127532 | 1.326178226 | 1.9620279 | 1.351564537 | 1.266377908 | 1.572434906 | 1.313758637 | 1.132239531 | 1.691927395 | 1.372178002 | 1.256533264 | 1.250218104 | 0.913326315 |
| ENSMUSG00000026707 | Nsun6 | 5024 | 2.944227957 | 3.016991813 | 2.628577585 | 2.430406027 | 3.554327962 | 3.619795626 | 2.429671808 | 3.709771571 | 2.205249351 | 3.359863011 | 3.784720039 | 2.58858467 | 3.584026633 | 3.549866552 | 4.133277104 | 3.216289077 | 4.340697241 | 4.324959625 |
| ENSMUSG00000026708 | Cenpl | 5497 | 1.012948528 | 1.127196348 | 1.02079853 | 1.234963727 | 0.998187975 | 1.253016185 | 0.852092999 | 0.694786291 | 1.476031218 | 1.132234461 | 1.633926177 | 1.025537915 | 1.085894486 | 1.901022314 | 1.821353117 | 1.560323096 | 1.373527468 | 1.160825246 |
| ENSMUSG00000026709 | Dars2 | 5868 | 7.040734679 | 4.870903756 | 5.308887789 | 5.986292635 | 6.143628294 | 5.995644315 | 4.482932768 | 4.712218702 | 5.664198742 | 5.070227293 | 6.692402159 | 4.489603398 | 2.816324521 | 3.996977528 | 3.763468636 | 4.287138559 | 4.872429702 | 4.180992092 |
| ENSMUSG00000026712 | Mrc1 | 5917 | 0.172405649 | 0.016890093 | 0.040876737 | 0.146300463 | 0.146421265 | 0.133994209 | 0.495755635 | 0.258187611 | 0.556856728 | 0.055780788 | 0 | 0 | 0.025011957 | 0.243327144 | 0 | 0 | 0.084205148 | 0.176639011 |
| ENSMUSG00000026715 | Serpinc1 | 2149 | 0.35602288 | 0.232523692 | 0.315137006 | 0.169608355 | 0.515139248 | 0.253643587 | 0.220161372 | 0.835292188 | 0.459970395 | 0.197466882 | 0.600243778 | 0.311674244 | 0.642761137 | 0.41062711 | 0.249241613 | 0.338539572 | 0.552868871 | 0.383963053 |
| ENSMUSG00000026718 | Stam | 5563 | 15.18969799 | 13.7251759 | 13.01728832 | 13.52171206 | 12.71001184 | 14.28771969 | 14.44129585 | 13.06491853 | 10.60943324 | 12.80688704 | 12.69304128 | 11.21731274 | 10.53502026 | 12.48137887 | 11.27247314 | 11.99721889 | 14.91577077 | 15.56432392 |

| ENSMUSG00000026721 | Rabgap1l | 10583 | 3.470138154 | 3.664009153 | 4.063504534 | 3.663653853 | 4.029564809 | 3.85352922 | 4.162156674 | 4.319786987 | 3.28464701 | 5.168176867 | 4.532368734 | 3.818438979 | 4.409713101 | 3.063212038 | 3.531116998 | 5.195626215 | 5.493810111 | 4.30903803 |
| --- | --- | --- | --- | --- | --- | --- | --- | --- | --- | --- | --- | --- | --- | --- | --- | --- | --- | --- | --- | --- |
| ENSMUSG00000026723 | Trdmt1 | 3971 | 3.981849781 | 2.910998311 | 2.86269948 | 3.476453396 | 3.102939426 | 1.759490162 | 2.347166394 | 3.270062155 | 2.966341855 | 3.657118258 | 3.741629838 | 3.064168835 | 2.919415905 | 2.549688001 | 2.73916161 | 3.403823826 | 3.474557261 | 3.213828446 |
| ENSMUSG00000026725 | Tnn | 5810 | 0 | 0.005733717 | 0 | 0 | 0 | 0 | 0 | 0.059162069 | 0 | 0 | 0 | 0 | 0 | 0.031975275 | 0 | 0 | 0 | 0 |
| ENSMUSG00000026726 | Cubn | 12165 | 0.013976219 | 0.019168949 | 0.055670319 | 0.078650385 | 0.031652724 | 0.008146771 | 0.093341907 | 0.043953443 | 0.030470912 | 0.034883381 | 0.054981457 | 0.013764652 | 0.004055234 | 0.053449833 | 0.098219903 | 0.031476044 | 0.037806458 | 0.085916402 |
| ENSMUSG00000026727 | Rsu1 | 8979 | 17.20278535 | 15.87918335 | 16.5555027 | 16.04455086 | 16.71939747 | 16.20300989 | 14.66434853 | 13.53471706 | 12.28852609 | 14.08902292 | 14.29151782 | 13.81249608 | 12.64751905 | 11.99508171 | 12.32054545 | 14.14095714 | 12.21626704 | 12.27734156 |
| ENSMUSG00000026728 | Vim | 2179 | 1.853139845 | 1.941595946 | 1.553991338 | 2.341825133 | 1.457873778 | 2.114917037 | 2.757554027 | 3.260113285 | 1.228601893 | 0.995379692 | 5.854021989 | 1.562531169 | 4.278904252 | 3.218475078 | 1.380318302 | 2.7413193 | 0.035177877 | 0.858333928 |
| ENSMUSG00000026729 | 4930562F07R | 1251 | 0.339769593 | 0.106516049 | 0.54135046 | 0.327777297 | 0.153899037 | 0.237662991 | 0.189099436 | 0.305294983 | 0.526765989 | 0.414594513 | 0.343704737 | 0.223084183 | 0.23660392 | 0.556883542 | 0.09880469 | 0.826415987 | 0.214455698 | 0.263832646 |
| ENSMUSG00000026730 | Pter | 3835 | 1.917443404 | 1.468025847 | 1.261369762 | 2.281022298 | 1.24251902 | 1.485935948 | 1.011640071 | 0.856465869 | 1.116922461 | 1.131125085 | 0.697626512 | 1.39721085 | 1.054815441 | 1.223168925 | 0.913202673 | 1.527630491 | 1.489079071 | 1.635212038 |
| ENSMUSG00000026734 | 4921504E06R | 2276 | 0 | 0 | 0 | 0 | 0 | 0 | 0 | 0 | 0 | 0 | 0 | 0 | 0 | 0 | 0 | 0 | 0 | 0 |
| ENSMUSG00000026735 | Ptf1a | 1506 | 0 | 0 | 0 | 0 | 0 | 0 | 0 | 0 | 0 | 0 | 0 | 0 | 0 | 0 | 0 | 0 | 0 | 0 |
| ENSMUSG00000026736 | 4930426L09R | 1458 | 0.02915307 | 0.02284835 | 0 | 0 | 0.066024587 | 0 | 0 | 0 | 0 | 0 | 0.065534922 | 0 | 0 | 0 | 0.028258955 | 0 | 0 | 0 |
| ENSMUSG00000026737 | Pip4k2a | 3556 | 1.099683969 | 1.077329256 | 0.421704005 | 0.614997224 | 0.906872578 | 0.641008365 | 1.383722893 | 1.063286794 | 1.378288913 | 0.875125539 | 2.042124209 | 0.361012441 | 0.138728676 | 0.927314456 | 0.81105425 | 0.829127326 | 0.937679367 | 1.639754025 |
| ENSMUSG00000026739 | Bmi1 | 4149 | 14.01472181 | 16.63637426 | 14.67878398 | 16.28513588 | 16.03237371 | 14.78579999 | 13.55863467 | 10.82532302 | 13.46081354 | 14.39863606 | 14.41654568 | 13.21064851 | 10.7248468 | 11.52986503 | 11.59879906 | 13.47415927 | 17.55120851 | 10.7790802 |
| ENSMUSG00000026740 | Dnajc1 | 10556 | 5.19838786 | 4.528609625 | 5.205791065 | 5.11892749 | 4.555114792 | 4.764685921 | 4.845112346 | 3.961792406 | 5.349252852 | 4.194250697 | 4.136634336 | 3.664290846 | 3.822802987 | 4.289786397 | 3.680661918 | 4.60677069 | 4.415003508 | 4.34611548 |
| ENSMUSG00000026743 | Mllt10 | 5846 | 10.65174185 | 8.86102472 | 8.9779816 | 9.056095367 | 9.213072628 | 8.713686301 | 12.25306122 | 10.75998747 | 9.581536339 | 10.39638325 | 13.17366286 | 10.74112563 | 10.02502869 | 10.32002558 | 12.13634609 | 10.86625345 | 10.79115373 | 12.62781752 |
| ENSMUSG00000026748 | Plxdc2 | 7283 | 11.90588481 | 15.29108957 | 13.38358694 | 16.07116887 | 18.16099495 | 15.00257827 | 9.068858957 | 12.08228685 | 9.087838917 | 10.25493807 | 14.14946937 | 10.44579817 | 13.24910479 | 12.84976467 | 12.08948578 | 16.51390881 | 19.54993725 | 12.14535818 |
| ENSMUSG00000026749 | Nek6 | 4261 | 0 | 0.085999023 | 0 | 0.096233138 | 0 | 0 | 0 | 0.098595735 | 0 | 0 | 0 | 0.091694353 | 0 | 0 | 0.154711311 | 0.269588881 | 0.008994672 | 0.077459432 |
| ENSMUSG00000026750 | Psmb7 | 1206 | 106.3334296 | 99.82819213 | 98.47182174 | 90.32873715 | 104.1661035 | 94.38028941 | 97.68538187 | 102.0047497 | 105.1519752 | 94.26230975 | 96.5006624 | 91.08227527 | 96.0867941 | 102.8624854 | 95.38535991 | 93.63101263 | 91.36658673 | 97.06416178 |
| ENSMUSG00000026751 | Nr5a1 | 5837 | 0 | 0 | 0 | 0 | 0 | 0 | 0 | 0 | 0.00705611 | 0 | 0 | 0.105186275 | 0 | 0.007956842 | 0 | 0 | 0.006566095 | 0 |
| ENSMUSG00000026753 | Ppp6c | 4303 | 35.26457783 | 37.86506289 | 39.26766013 | 39.9281195 | 39.194576 | 38.1635508 | 34.30526569 | 34.71310384 | 36.87930403 | 37.69426428 | 35.69521067 | 31.89651738 | 35.0585645 | 33.17897389 | 37.67792759 | 40.1415129 | 46.75220368 | 32.98245302 |
| ENSMUSG00000026754 | Golga1 | 6316 | 9.724505915 | 9.836692166 | 9.359159926 | 9.39209622 | 9.39624159 | 8.622301172 | 9.805620126 | 10.31001363 | 10.79222349 | 7.957987599 | 9.86360764 | 9.45578831 | 10.7396115 | 10.45653772 | 10.54175338 | 9.336215231 | 10.64956477 | 12.13231249 |
| ENSMUSG00000026755 | Arpc5l | 4619 | 19.62839153 | 18.93909076 | 18.7776012 | 18.16918027 | 17.20411474 | 16.63916211 | 22.06354415 | 19.74528187 | 21.24863976 | 20.27306704 | 20.67593462 | 17.65461854 | 27.29863186 | 23.54883149 | 19.64187618 | 20.87372796 | 21.08402694 | 23.96153339 |
| ENSMUSG00000026761 | Orc4 | 8159 | 11.1329282 | 10.58712277 | 11.17589223 | 10.19106581 | 10.71892454 | 11.24183231 | 12.33411792 | 10.78505885 | 10.10606748 | 11.75444668 | 10.37593166 | 10.44619644 | 11.60893261 | 11.58398307 | 14.68995295 | 13.16400953 | 16.19202665 | 14.96080498 |
| ENSMUSG00000026764 | Kif5c | 7113 | 2.659187872 | 2.482192315 | 2.482263262 | 2.363563245 | 2.361597267 | 2.793567576 | 2.660631459 | 2.555825045 | 2.536158288 | 2.538833031 | 3.190370487 | 1.86758438 | 2.64240974 | 2.050252136 | 2.160576458 | 2.912304035 | 2.963512343 | 1.778728321 |
| ENSMUSG00000026765 | Lypd6b | 2633 | 0.936308473 | 0.809732332 | 1.396273569 | 0.865192638 | 1.224777397 | 1.242111818 | 2.120355531 | 3.133140494 | 4.270383138 | 4.19037773 | 4.518026837 | 3.60374578 | 3.859618291 | 2.645884204 | 4.506664678 | 5.366211216 | 7.263510123 | 2.319031844 |
| ENSMUSG00000026766 | Mmadhc | 2056 | 50.79533925 | 49.56427209 | 45.47958861 | 46.93496688 | 47.52325148 | 50.9747235 | 48.23315906 | 52.49597724 | 50.56165651 | 48.68722909 | 52.88706493 | 50.35897573 | 49.61984675 | 51.30083777 | 45.38984635 | 46.89481977 | 49.39916673 | 51.07606261 |
| ENSMUSG00000026767 | Fam188a | 4678 | 9.213392153 | 10.06935281 | 11.93310261 | 10.63545541 | 11.0195148 | 10.55034687 | 12.90529677 | 12.29537367 | 12.87188671 | 12.08501351 | 10.93778977 | 10.71450727 | 9.2484163 | 10.26575168 | 10.72755346 | 11.39387035 | 11.20786109 | 10.81837926 |
| ENSMUSG00000026768 | Itga8 | 8962 | 0 | 0 | 0 | 0.076257048 | 0 | 0 | 0 | 0 | 0 | 0 | 0 | 0 | 0 | 0 | 0 | 0 | 0 | 0 |
| ENSMUSG00000026770 | Il2ra | 5296 | 0 | 0 | 0 | 0 | 0 | 0 | 0.035734652 | 0.223558247 | 0 | 0.044515392 | 0.135314271 | 0.010539211 | 0.093149391 | 0.08769654 | 0 | 0 | 0 | 0 |
| ENSMUSG00000026771 | Spopl | 7496 | 3.124379935 | 3.648597406 | 4.001012383 | 3.276067622 | 3.460926742 | 3.146625024 | 3.458824447 | 3.240443961 | 3.225251434 | 3.333761036 | 3.231310559 | 3.648569193 | 3.718320873 | 3.841426652 | 3.61118226 | 3.871969105 | 3.880690948 | 3.390369171 |
| ENSMUSG00000026773 | Pfkfb3 | 7251 | 1.184118819 | 1.493130688 | 1.948016944 | 0.904811803 | 1.573198995 | 1.271108598 | 3.021068865 | 4.50344836 | 1.800596538 | 1.137963463 | 2.167695675 | 1.262414655 | 1.176999268 | 1.46038504 | 1.562602106 | 0.908286371 | 1.707267122 | 0.940715658 |
| ENSMUSG00000026774 | 4931423N10R | 2599 | 0 | 0 | 0 | 0.017530221 | 0 | 0 | 0.036408372 | 0.132255337 | 0 | 0 | 0 | 0.021475822 | 0 | 0 | 0 | 0 | 0.073732776 | 0 |
| ENSMUSG00000026775 | Yme1l1 | 8202 | 11.01237492 | 11.12460586 | 12.8217575 | 12.78175604 | 12.52298526 | 12.40328725 | 11.34073723 | 12.10683324 | 10.57532336 | 10.74428973 | 11.59716439 | 9.608841206 | 9.839919125 | 10.77013585 | 11.26236141 | 12.88024699 | 14.18194478 | 11.05949975 |
| ENSMUSG00000026778 | Prkcq | 6732 | 0 | 0 | 0 | 0.040606991 | 0 | 0.06624697 | 0 | 0 | 0 | 0 | 0 | 0 | 0 | 0 | 0 | 0 | 0.005693152 | 0 |
| ENSMUSG00000026779 | Mastl | 5765 | 0.294918828 | 0.99967575 | 0.805526264 | 0.837722584 | 1.001878723 | 0.747803612 | 0.098482593 | 0.775110335 | 0.785865869 | 0.400760531 | 1.011022538 | 0.706772484 | 0.770142681 | 1.297050836 | 0.643215964 | 0.770461485 | 1.150121312 | 0.734727011 |
| ENSMUSG00000026781 | Acbd5 | 4225 | 30.54336436 | 29.9382389 | 31.7261663 | 30.37762412 | 30.33735212 | 32.82795297 | 29.77619249 | 29.17085978 | 29.03040093 | 33.1449914 | 31.49189571 | 32.65711667 | 23.58591075 | 28.94373541 | 30.37700511 | 28.74740986 | 40.58505381 | 26.56061011 |
| ENSMUSG00000026782 | Abi2 | 6000 | 5.086452731 | 5.513283993 | 6.207936396 | 6.211489044 | 4.548466787 | 5.607717683 | 7.0732455 | 7.415691467 | 8.216709895 | 6.451787896 | 5.613557621 | 5.693197584 | 6.067825825 | 7.098204689 | 8.226577326 | 6.917836404 | 9.262188418 | 6.848633787 |
| ENSMUSG00000026784 | Pdss1 | 4401 | 2.211698548 | 1.877209218 | 2.297220596 | 1.697798516 | 2.045141951 | 2.173069207 | 2.494101684 | 2.438551483 | 2.667156781 | 2.806972109 | 2.844135224 | 2.473086618 | 2.634174179 | 2.469419779 | 2.21876136 | 1.931496215 | 2.420974901 | 2.07487201 |
| ENSMUSG00000026785 | Pkn3 | 4099 | 0.342198294 | 0.292574821 | 0.118013004 | 0.122266769 | 0.281816582 | 0.265957584 | 0.057712465 | 0.195667346 | 0.291390329 | 0.356592291 | 0.046621087 | 0.367656231 | 0.180526655 | 0 | 0.482477356 | 0.130780313 | 0.158952683 | 0.187881799 |
| ENSMUSG00000026786 | Apbb1ip | 7531 | 0 | 0 | 0.006423255 | 0 | 0.012782346 | 0 | 0.219883649 | 0.010142717 | 0.010937861 | 0 | 0.076125282 | 0 | 0 | 0.012334109 | 0.032825566 | 0.020337595 | 0 | 0 |
| ENSMUSG00000026787 | Gad2 | 5860 | 0 | 0 | 0 | 0.015549844 | 0 | 0 | 0 | 0 | 0 | 0 | 0 | 0 | 0 | 0 | 0 | 0 | 0 | 0 |

| ENSMUSG00000026788 | Zbtb43 | 6822 | 3.919049505 | 4.726895574 | 4.353759548 | 5.422979771 | 4.825892083 | 4.438100296 | 3.12782442 | 5.077764436 | 4.443458751 | 5.114558841 | 5.329337936 | 6.177195141 | 4.194153037 | 3.68992896 | 3.521035926 | 5.276043823 | 4.775337492 | 4.354282854 |
| --- | --- | --- | --- | --- | --- | --- | --- | --- | --- | --- | --- | --- | --- | --- | --- | --- | --- | --- | --- | --- |
| ENSMUSG00000026790 | Odf2 | 8427 | 6.738686979 | 7.637416831 | 6.899843774 | 6.114814423 | 7.031019109 | 8.291130218 | 6.271302048 | 7.024827783 | 7.888339438 | 8.773264828 | 8.107059526 | 7.299022211 | 6.983858705 | 8.531558951 | 8.028118521 | 7.801705578 | 8.309261237 | 8.388121768 |
| ENSMUSG00000026791 | Slc2a8 | 2218 | 19.98778115 | 21.92823515 | 19.69400266 | 22.98593716 | 19.31353114 | 20.71027233 | 23.1870523 | 25.69119224 | 24.82703858 | 27.59315276 | 25.26601727 | 28.53695283 | 28.91410841 | 25.98607414 | 27.80826383 | 28.03604437 | 27.57834529 | 25.66926306 |
| ENSMUSG00000026792 | Lrsam1 | 6096 | 5.452599681 | 3.573922707 | 3.943839335 | 5.074794801 | 5.274298722 | 5.121099435 | 4.299741491 | 3.928253988 | 5.364516653 | 4.82644994 | 5.50948094 | 4.184343471 | 3.989605515 | 3.207506691 | 3.791678618 | 3.900667191 | 3.677966484 | 4.945044371 |
| ENSMUSG00000026796 | Fam129b | 4653 | 17.86806884 | 18.24942346 | 18.81712657 | 23.59813386 | 15.52676068 | 21.20341555 | 14.89642696 | 13.74860822 | 10.06427432 | 16.09183683 | 13.88174164 | 16.25407754 | 14.39775277 | 14.8824703 | 14.37140022 | 14.21188029 | 12.40477179 | 16.1492456 |
| ENSMUSG00000026797 | Stxbp1 | 6315 | 2.954833298 | 3.328651821 | 3.960271606 | 3.6434406 | 3.963357135 | 3.648776107 | 3.805992224 | 2.261909501 | 2.471843147 | 4.278282338 | 3.207693174 | 2.739961267 | 3.085686037 | 1.573877229 | 3.536222216 | 3.080226217 | 3.441173451 | 2.369355032 |
| ENSMUSG00000026798 | Coq4 | 3529 | 5.877734741 | 4.908672427 | 4.619404854 | 4.905978138 | 5.742006196 | 4.689887508 | 6.703411579 | 4.999968805 | 6.395639172 | 5.451257277 | 6.484614653 | 6.057636376 | 5.955057172 | 5.51433058 | 5.183760503 | 6.087002197 | 5.180403407 | 5.050425213 |
| ENSMUSG00000026799 | Med27 | 3508 | 5.5494215 | 6.201060412 | 6.453481245 | 6.62375502 | 6.928911474 | 5.523123954 | 6.190570019 | 6.434352857 | 7.92499947 | 6.303785584 | 6.523433612 | 5.664303273 | 5.639138776 | 6.063680737 | 6.401039898 | 6.123440921 | 7.385569187 | 8.138462483 |
| ENSMUSG00000026803 | Ttf1 | 6049 | 5.530099774 | 4.681097716 | 4.86214357 | 3.675614091 | 4.53549289 | 5.259192421 | 4.231469549 | 6.452741818 | 5.290448462 | 5.573277042 | 5.568084928 | 5.527125457 | 5.496728756 | 5.904364887 | 6.361754538 | 5.342581029 | 6.278948625 | 5.965609855 |
| ENSMUSG00000026805 | Barhl1 | 6138 | 0.020774768 | 0 | 0 | 0 | 0 | 0 | 0 | 0 | 0.020130262 | 0 | 0 | 0 | 0 | 0 | 0 | 0 | 0 | 0 |
| ENSMUSG00000026806 | Ddx31 | 3913 | 2.194236023 | 2.247534903 | 1.656543079 | 2.957450872 | 2.004984298 | 2.507396179 | 1.596032103 | 2.274170652 | 2.305097611 | 1.91591153 | 2.380811884 | 2.567549007 | 2.054971254 | 2.860471521 | 4.043290944 | 2.074523069 | 3.02653354 | 1.616674488 |
| ENSMUSG00000026807 | Ak8 | 1925 | 0.309128553 | 0.501856588 | 0.653356774 | 0.875718774 | 0.600086321 | 0.772250393 | 2.998517833 | 2.360984876 | 2.417701964 | 1.984003618 | 0.595635846 | 2.609563446 | 3.228998222 | 2.678074649 | 1.327011151 | 1.631080419 | 1.811788581 | 0.885860939 |
| ENSMUSG00000026809 | 1700026L06R | 759 | 0.280007747 | 0.263343037 | 0.382399449 | 0.600277264 | 0 | 0 | 2.057072997 | 0.553513078 | 0.976755315 | 0.683343525 | 0.440612265 | 1.323691603 | 0.714955323 | 1.407396588 | 0.434271999 | 0.706282613 | 0.353470459 | 0.434854599 |
| ENSMUSG00000026810 | Dpm2 | 2014 | 23.5108074 | 25.04256298 | 22.64957255 | 21.76252465 | 21.69999339 | 22.21753646 | 26.96869683 | 25.42999584 | 24.51967845 | 23.90311223 | 28.32338651 | 24.08332214 | 32.82262619 | 30.1402295 | 20.94855672 | 25.64748232 | 24.73892054 | 26.30276553 |
| ENSMUSG00000026811 | St6galnac6 | 4786 | 0.204266412 | 0.30626146 | 0.313326252 | 0.514061094 | 0.080454532 | 0.310610532 | 1.037992329 | 0.646382072 | 1.230604211 | 0.975327961 | 0.279502473 | 1.131240968 | 2.350120589 | 2.202843259 | 0.68870131 | 0.288019613 | 0.408408095 | 1.425225498 |
| ENSMUSG00000026812 | Tsc1 | 5873 | 6.665633768 | 7.005180378 | 6.325705996 | 5.926892197 | 6.351479796 | 5.484297093 | 7.427599178 | 7.218383564 | 7.132076712 | 6.406650979 | 7.719808547 | 7.004281173 | 7.249011512 | 6.682318039 | 6.78391019 | 5.789555487 | 6.06903731 | 8.279933653 |
| ENSMUSG00000026814 | Eng | 4137 | 0 | 0 | 0 | 0 | 0 | 0 | 0 | 0 | 0 | 0.068383906 | 0.057741067 | 0 | 0.047698252 | 0 | 0 | 0 | 0 | 0.053187437 |
| ENSMUSG00000026815 | Gfi1b | 2571 | 0.049597638 | 0.012957174 | 0.018815064 | 0.017721137 | 0 | 0 | 0.33124396 | 0.133695691 | 0.400491207 | 0.055018324 | 0.204404723 | 0.065129128 | 0.095939162 | 0.722584012 | 0.192305978 | 0.044679822 | 0 | 0 |
| ENSMUSG00000026816 | Gtf3c5 | 4545 | 7.173040707 | 5.958940154 | 5.151328645 | 5.343022361 | 5.125599792 | 5.941966953 | 5.350652798 | 6.571278038 | 6.270862246 | 6.141521744 | 8.104398897 | 6.963142065 | 6.078300038 | 6.080139053 | 5.14906133 | 6.891466855 | 5.860676864 | 5.906368335 |
| ENSMUSG00000026817 | Ak1 | 2783 | 4.230662508 | 4.75214481 | 5.336210499 | 6.31928247 | 4.341039468 | 5.733374133 | 4.692166505 | 6.518645757 | 4.484194853 | 4.777757211 | 4.102844081 | 4.973871477 | 1.878973493 | 2.453209072 | 2.975750174 | 2.476575398 | 3.621924573 | 4.704336112 |
| ENSMUSG00000026818 | Cel | 2093 | 0 | 0.095498025 | 0 | 0 | 0 | 0 | 0 | 0 | 0 | 0 | 0 | 0 | 0 | 0 | 0.078741626 | 0.146356837 | 0.036623313 | 0.105129683 |
| ENSMUSG00000026819 | Slc25a25 | 6166 | 3.894814216 | 5.96455323 | 4.409004875 | 5.999929938 | 4.238669243 | 4.741503862 | 4.250928337 | 4.83753913 | 3.252973269 | 4.182847013 | 6.640145867 | 4.634709576 | 4.024319557 | 5.97310433 | 4.637346706 | 9.817945232 | 9.093638075 | 8.047066861 |
| ENSMUSG00000026820 | Ptges2 | 2128 | 19.69459754 | 16.484247 | 18.41285695 | 21.75282944 | 19.33871933 | 19.90958046 | 17.29758656 | 16.17074933 | 16.78040844 | 18.63427699 | 21.61996476 | 22.00626922 | 16.89989083 | 15.69233968 | 14.44377852 | 20.5308191 | 17.38011114 | 16.90599427 |
| ENSMUSG00000026821 | Ralgds | 4745 | 13.5801574 | 16.15447199 | 15.48564649 | 18.37804823 | 15.07355924 | 15.99890155 | 20.67000704 | 21.06417642 | 14.74728984 | 17.73740892 | 13.34074183 | 13.84510745 | 12.45513949 | 13.75214811 | 16.0204153 | 22.45790525 | 16.17862438 | 18.80395598 |
| ENSMUSG00000026822 | Lcn2 | 2185 | 80.01088742 | 129.2265865 | 66.90380262 | 86.68066879 | 66.4374745 | 104.7977949 | 8.856240587 | 13.58146301 | 9.537929966 | 9.948043113 | 9.970426117 | 12.03165999 | 15.46561251 | 11.414405 | 15.70750392 | 10.14657285 | 9.419323312 | 17.62305479 |
| ENSMUSG00000026824 | Kcnj3 | 5211 | 0.415997693 | 0.070320828 | 0.278488949 | 0.069945952 | 0.249388206 | 0.190185122 | 1.579813113 | 1.004098854 | 1.541234038 | 0.877685323 | 0.85263311 | 0.449867171 | 1.486300329 | 0.971484461 | 1.336223939 | 1.961925192 | 1.794591526 | 1.203423175 |
| ENSMUSG00000026825 | Dnm1 | 5017 | 0.169444592 | 0.13944006 | 0.308541553 | 0.345090629 | 0.182281553 | 0.414832531 | 0.179178971 | 0.022837793 | 0 | 0.469909341 | 0.2571106 | 0.233631436 | 0 | 0.240690905 | 0.188884948 | 0.190804302 | 0.244457146 | 0.317971715 |
| ENSMUSG00000026826 | Nr4a2 | 4745 | 2.72319779 | 3.046953865 | 2.365154698 | 3.495093812 | 2.972108248 | 3.738646706 | 0.388871334 | 2.921779151 | 1.171797603 | 1.619718573 | 1.913012036 | 2.870183703 | 1.413939041 | 2.055481212 | 1.562967347 | 0.847315865 | 1.70428844 | 2.179496747 |
| ENSMUSG00000026827 | Gpd2 | 6837 | 12.06085147 | 14.456539 | 14.18588977 | 12.48147375 | 11.88338265 | 15.27821594 | 4.684903267 | 6.384951872 | 6.43369882 | 7.172258503 | 5.792809797 | 10.74351499 | 3.362391904 | 3.831280574 | 4.724589706 | 3.309894541 | 4.691986326 | 4.095310125 |
| ENSMUSG00000026828 | Galnt5 | 4451 | 0 | 0.007484362 | 0 | 0.010236137 | 0 | 0 | 0.021259348 | 0.008580634 | 0.009253317 | 0 | 0.010733534 | 0 | 1.928500024 | 0.980845701 | 2.610388398 | 3.94003554 | 2.083793271 | 1.754952405 |
| ENSMUSG00000026829 | Gbgt1 | 1330 | 0 | 0 | 0 | 0 | 0 | 0 | 0 | 0 | 0 | 0 | 0 | 0 | 0 | 0 | 0 | 0 | 0 | 0 |
| ENSMUSG00000026830 | Ermn | 3632 | 0 | 0 | 0 | 0 | 0 | 0 | 0 | 0 | 0 | 0 | 0 | 0 | 0 | 0 | 0 | 0 | 0 | 0.015145679 |
| ENSMUSG00000026831 | 1700007K13R | 1388 | 0.70433649 | 0.720019328 | 0.453066206 | 0.525199358 | 0.242740249 | 0.178504083 | 0 | 0 | 0.445099234 | 0.339702473 | 0.275359991 | 0.603195201 | 0.675292816 | 0.86999011 | 0.801471188 | 0.441390287 | 0.690315146 | 0.158527685 |
| ENSMUSG00000026832 | Cytip | 6848 | 1.961395389 | 2.383662116 | 2.210998102 | 2.647969574 | 2.185897818 | 1.989924319 | 2.646138439 | 1.901812094 | 1.978733015 | 1.280670386 | 3.125464573 | 1.475267951 | 3.465048512 | 3.479237271 | 2.815760537 | 2.818117134 | 2.076380863 | 3.068557063 |
| ENSMUSG00000026833 | Olfm1 | 3914 | 23.96753283 | 24.43569732 | 31.50335434 | 28.6240695 | 26.75908684 | 32.99295445 | 36.91694467 | 29.43957027 | 25.56005283 | 30.26125871 | 25.86487916 | 29.7046564 | 25.2835529 | 27.86170598 | 31.07485769 | 30.27834183 | 26.74223731 | 31.39763526 |
| ENSMUSG00000026834 | Acvr1c | 12502 | 0.020399221 | 0 | 0 | 0 | 0 | 0 | 0 | 0 | 0 | 0 | 0 | 0 | 0 | 0 | 0 | 0 | 0 | 0 |
| ENSMUSG00000026835 | Fcnb | 1330 | 0 | 0 | 0 | 0 | 0 | 0 | 0 | 0 | 0 | 0 | 0 | 0 | 0 | 0 | 0 | 0 | 0 | 0 |
| ENSMUSG00000026836 | Acvr1 | 4812 | 4.337082591 | 3.717586076 | 4.252286646 | 4.222823311 | 3.170785495 | 3.593911891 | 2.664533664 | 3.222384742 | 2.379437112 | 2.959167162 | 2.333149469 | 3.32898902 | 3.331852268 | 2.54805467 | 2.243309984 | 3.087438835 | 2.429243673 | 2.263466985 |
| ENSMUSG00000026837 | Col5a1 | 8424 | 0.015137171 | 0.114681141 | 0.028711735 | 0.05408481 | 0.022854665 | 0.047058626 | 0.24150584 | 0.23575557 | 0.45469444 | 0 | 0.119097119 | 0.278283218 | 0.058561155 | 0.066159669 | 0.053800702 | 0.018181675 | 0.009099311 | 0 |
| ENSMUSG00000026839 | Upp2 | 7250 | 0.515924895 | 0.542196071 | 0.333610554 | 0.458752584 | 0.331944301 | 0.36224757 | 0.5416486 | 0.284467549 | 0.57945167 | 0.71538998 | 0.47445476 | 0.423429164 | 0.877767907 | 0.582953373 | 0.33529542 | 0.433079973 | 0.512779421 | 0.751158837 |
| ENSMUSG00000026840 | Lamc3 | 6461 | 0 | 0 | 0 | 0 | 0 | 0.023008544 | 0.014645621 | 0 | 0 | 0 | 0.096126677 | 0 | 0 | 0 | 0 | 0.082969897 | 0.05931945 | 0 |

| ENSMUSG00000026841 | Fibcd1 | 4744 | 0 | 0 | 0.010196781 | 0.01920786 | 0.010145852 | 0.104453486 | 0.728040801 | 0.402533752 | 0.234408922 | 0.029817055 | 0.271906383 | 0.741228234 | 0 | 0 | 0 | 0 | 0.105025687 | 0 |
| --- | --- | --- | --- | --- | --- | --- | --- | --- | --- | --- | --- | --- | --- | --- | --- | --- | --- | --- | --- | --- |
| ENSMUSG00000026842 | Abl1 | 12870 | 4.627020327 | 4.477957032 | 4.352490144 | 4.156073508 | 4.207335985 | 5.093882403 | 3.124769005 | 3.798467372 | 3.171393716 | 4.029974637 | 3.081057928 | 3.322051091 | 3.319459236 | 2.345660979 | 2.785186762 | 3.451212478 | 4.02321889 | 3.804048561 |
| ENSMUSG00000026843 | Fubp3 | 4563 | 13.72126327 | 16.07604494 | 14.66153681 | 14.76764948 | 14.546115 | 15.74654037 | 10.67982891 | 13.29996218 | 12.89842889 | 15.91323318 | 11.61131469 | 14.60528186 | 8.108467665 | 10.38197877 | 10.31167583 | 13.89639401 | 13.54817377 | 10.84992243 |
| ENSMUSG00000026848 | Tor1b | 3436 | 20.75776641 | 18.43067867 | 18.17526999 | 20.99043457 | 21.22231209 | 19.80090314 | 13.06744246 | 18.22920254 | 23.85365727 | 17.94910357 | 15.25294804 | 13.17418579 | 17.32934347 | 15.28762015 | 14.61719925 | 19.31246293 | 18.2038756 | 21.54896905 |
| ENSMUSG00000026849 | Tor1a | 2235 | 23.22094851 | 18.58665731 | 19.93379036 | 20.38525474 | 20.28647521 | 18.18042127 | 20.99963199 | 20.47181122 | 22.35312914 | 20.63238827 | 20.75592156 | 23.07546856 | 21.18954836 | 22.21419661 | 21.56859973 | 22.5803224 | 20.26920937 | 16.83500179 |
| ENSMUSG00000026851 | BC005624 | 2615 | 36.14971759 | 34.37024421 | 31.74339506 | 35.83903182 | 35.78143771 | 31.49393622 | 34.75627389 | 36.67346938 | 37.75299361 | 31.82447084 | 34.65739818 | 34.72737401 | 33.29668604 | 36.74677507 | 28.95925802 | 35.61099702 | 35.43900035 | 30.88082932 |
| ENSMUSG00000026853 | Crat | 7628 | 8.614709247 | 9.769394909 | 10.63482045 | 10.41071058 | 9.622598793 | 10.08854159 | 8.187301545 | 10.77979056 | 9.184355442 | 11.70731935 | 9.663969708 | 10.22945678 | 10.48335578 | 9.936647957 | 9.484783973 | 10.64687707 | 10.57138551 | 9.04318561 |
| ENSMUSG00000026854 | Usp20 | 7440 | 34.55830777 | 41.59186484 | 42.26832269 | 43.90761934 | 44.03682855 | 46.11601173 | 35.68800459 | 38.63900711 | 33.27029029 | 36.52278264 | 37.06412101 | 36.52775015 | 21.43020923 | 24.67654267 | 28.03256397 | 21.03410114 | 25.78782827 | 24.52489341 |
| ENSMUSG00000026856 | Dolpp1 | 3319 | 6.621023202 | 5.972031353 | 4.853385239 | 4.639841213 | 4.350580627 | 5.65847726 | 5.160346421 | 5.120704752 | 5.435279875 | 6.478071915 | 6.074429792 | 5.4991629 | 5.930531785 | 7.780329192 | 6.41795854 | 6.772095976 | 7.806139411 | 5.701456075 |
| ENSMUSG00000026857 | Mettl11a | 2931 | 28.49630532 | 27.10721689 | 22.77566424 | 27.88690329 | 25.53570157 | 27.62509951 | 24.01953813 | 24.8621985 | 25.1250393 | 26.17338594 | 27.12300611 | 23.46124064 | 25.4486042 | 23.67364946 | 27.00381742 | 29.23724987 | 23.6940464 | 27.06350457 |
| ENSMUSG00000026858 | Fam73b | 4450 | 5.836103944 | 6.976992677 | 6.815776075 | 7.218098035 | 7.192748144 | 6.324933185 | 8.813979962 | 7.973200412 | 6.960058394 | 7.469942876 | 8.127110914 | 5.242909429 | 6.551722047 | 8.683478811 | 7.101481656 | 5.756498057 | 8.156180487 | 9.122858598 |
| ENSMUSG00000026860 | Sh3glb2 | 4467 | 11.18056455 | 13.229701 | 12.76749324 | 13.14712025 | 13.52262463 | 14.15475443 | 21.35266638 | 23.85422044 | 18.99355775 | 21.82844288 | 22.30995375 | 17.19327327 | 36.03538081 | 32.6678582 | 32.13481547 | 30.20732046 | 26.85500544 | 34.54232021 |
| ENSMUSG00000026864 | Hspa5 | 3478 | 263.6836337 | 289.3279459 | 294.7892398 | 301.0592067 | 288.7500251 | 305.4944547 | 321.8432061 | 344.39049 | 301.7581353 | 317.704638 | 324.8504868 | 309.5859537 | 364.6420153 | 426.9704572 | 373.5741993 | 346.1567792 | 387.6818434 | 412.9320754 |
| ENSMUSG00000026866 | Kynu | 7523 | 0 | 0.008856279 | 0.006430085 | 0.006056233 | 0.006397969 | 0.006586832 | 0.012578141 | 0.081228026 | 0.005474746 | 0 | 0.012701039 | 0 | 0.144264546 | 0.012347225 | 0 | 0.015269417 | 0.00509455 | 0.007312124 |
| ENSMUSG00000026867 | Gapvd1 | 9538 | 11.80946912 | 12.68181159 | 12.30385664 | 12.17126661 | 12.49472038 | 12.94148242 | 8.99823858 | 10.3949965 | 12.87240548 | 10.73719097 | 11.32013065 | 11.61607153 | 10.24084674 | 9.957869439 | 10.04768494 | 11.03193428 | 11.18285639 | 10.3351142 |
| ENSMUSG00000026869 | Psmd5 | 4149 | 68.60861987 | 69.86153112 | 68.82355982 | 72.92622209 | 72.94614028 | 72.60329415 | 62.18270383 | 64.48247261 | 63.41274107 | 66.41328268 | 62.01878196 | 63.64519154 | 65.49052792 | 62.36201756 | 69.8410563 | 77.78096871 | 75.87664564 | 70.62750338 |
| ENSMUSG00000026870 | D730039F16R | 2405 | 27.94207206 | 23.89386384 | 22.64806452 | 23.64248787 | 18.93255709 | 22.60263981 | 30.31552525 | 23.07424561 | 24.35227668 | 29.42752831 | 21.67254873 | 28.10510079 | 26.03002205 | 21.43573264 | 20.42089589 | 28.14877085 | 24.06349618 | 22.87280945 |
| ENSMUSG00000026872 | Zeb2 | 24976 | 0.032334975 | 0.016005555 | 0 | 0.01824193 | 0.017344143 | 0.015872112 | 0.005682977 | 0.024466625 | 0.067610792 | 0.030205447 | 0.193196301 | 0.022347719 | 0.150113137 | 0.11157292 | 0.103927691 | 0.030661921 | 0.004603575 | 0.04845453 |
| ENSMUSG00000026873 | Phf19 | 7299 | 0.302818078 | 0.511171962 | 0.722388657 | 0.505609617 | 0.619865848 | 0.400549566 | 1.011203989 | 0.95755715 | 0.603775474 | 0.981902575 | 0.490905862 | 0.489409838 | 1.595058567 | 1.858018019 | 0.846723302 | 0.907559266 | 0.945161453 | 1.582670559 |
| ENSMUSG00000026874 | Hc | 6328 | 0 | 0 | 0 | 0 | 0 | 0 | 0 | 0 | 0 | 0 | 0 | 0 | 0 | 0 | 0 | 0 | 0 | 0 |
| ENSMUSG00000026875 | Traf1 | 5897 | 0.245069694 | 0.434982678 | 0.434762949 | 0.602638877 | 0.424429376 | 0.260494275 | 0.312903931 | 0.479267047 | 0.579698289 | 0.223879887 | 0.891172704 | 0.255557553 | 0.543763714 | 0.212648865 | 0.845410931 | 0.655816745 | 0.370459373 | 0.093283206 |
| ENSMUSG00000026878 | Rab14 | 6602 | 92.9486862 | 90.83087077 | 93.64769638 | 91.5637589 | 98.71345751 | 100.5690817 | 100.2366616 | 93.4562649 | 93.65222275 | 95.72264098 | 94.90587405 | 89.81908502 | 86.49898128 | 86.24024043 | 90.44122205 | 80.46131422 | 90.2775285 | 90.5042286 |
| ENSMUSG00000026879 | Gsn | 3170 | 493.6902448 | 532.6490083 | 493.3031911 | 547.6236313 | 564.844067 | 550.1760287 | 347.1138719 | 363.0446882 | 303.2340982 | 330.7688767 | 404.0525668 | 360.6008737 | 350.3966717 | 392.2840504 | 387.5805668 | 393.3182203 | 411.8807434 | 390.7728941 |
| ENSMUSG00000026880 | Stom | 3096 | 21.06038115 | 23.66119328 | 22.68681075 | 24.70833121 | 19.58857359 | 22.34354524 | 7.900728361 | 7.056219049 | 6.731387912 | 9.229110521 | 5.848420305 | 7.806260304 | 3.728575143 | 4.14036438 | 3.260459042 | 3.277458321 | 3.280513139 | 3.091597083 |
| ENSMUSG00000026882 | 4930568D16R | 1221 | 0 | 0 | 0 | 0 | 0 | 0 | 0 | 0 | 0 | 0 | 0 | 0 | 0 | 0 | 0 | 0 | 0 | 0 |
| ENSMUSG00000026883 | Dab2ip | 8306 | 12.83445479 | 12.08825706 | 10.76261547 | 11.17907637 | 12.06485253 | 11.00111254 | 7.769623653 | 8.01001264 | 9.307379013 | 8.151746915 | 7.287608041 | 7.855587475 | 6.966811819 | 6.357684488 | 6.979363007 | 8.28415862 | 6.335420858 | 7.364570994 |
| ENSMUSG00000026885 | Ttll11 | 4841 | 0.2985284 | 0.639970907 | 0.479638392 | 0.348225292 | 0.248563952 | 0.440150288 | 0.273653173 | 0.891497928 | 0.638088966 | 0.409074476 | 0.54278511 | 0.299774267 | 0.550283729 | 0.988172072 | 0.927694607 | 0.474578898 | 0.506689321 | 0.988595804 |
| ENSMUSG00000026887 | Mrrf | 2909 | 6.604448114 | 6.722127503 | 7.798963815 | 7.063606392 | 6.899626047 | 6.762610942 | 10.45790737 | 9.216592124 | 10.19398121 | 9.449590924 | 10.29731831 | 8.845314697 | 8.80139741 | 9.850808497 | 7.648277821 | 9.740477648 | 8.761425727 | 9.492805629 |
| ENSMUSG00000026888 | Grb14 | 5445 | 0.655727233 | 0.312021599 | 0.133260414 | 0.418375063 | 0.185632763 | 0.491432069 | 0.40839227 | 0.427867134 | 0.294999837 | 0.207826791 | 0.193030135 | 0.01025081 | 0.172140758 | 0.255890289 | 0.083235466 | 0 | 0.140776114 | 0.131334874 |
| ENSMUSG00000026889 | Rbm18 | 6083 | 13.59075577 | 14.84649946 | 14.86275329 | 13.75145116 | 15.75384843 | 14.67927436 | 15.77348559 | 16.85162059 | 17.94250646 | 15.0063721 | 15.48778861 | 17.12181923 | 14.96258218 | 13.36136001 | 17.12272453 | 16.47950192 | 17.03670998 | 17.96861665 |
| ENSMUSG00000026890 | Lhx6 | 6971 | 0.060974288 | 0.023893914 | 0.173481317 | 0.117644355 | 0 | 0.113734577 | 1.445646336 | 1.145059834 | 0.384037229 | 0.270553454 | 0.541417547 | 0.240205118 | 1.910718357 | 1.652292881 | 0.63832564 | 0.137321071 | 0.087967401 | 0.939045144 |
| ENSMUSG00000026893 | Gca | 3401 | 6.923806974 | 7.620532697 | 8.007732311 | 7.408190978 | 7.37334085 | 8.538048185 | 8.597246568 | 7.714842826 | 6.164050727 | 8.151900475 | 6.349391718 | 7.97601059 | 7.702219369 | 9.559206461 | 7.498901234 | 8.849284745 | 10.48028701 | 5.224328571 |
| ENSMUSG00000026894 | Morn5 | 709 | 0.239803532 | 0.281914479 | 0.409366971 | 0.449826954 | 0 | 0.069891021 | 0 | 0.269339932 | 0.232363982 | 0.199509323 | 0.269534321 | 0.236173467 | 0.626216157 | 0.327532351 | 0.348673252 | 0.054006498 | 0 | 0 |
| ENSMUSG00000026895 | Ndufa8 | 857 | 224.2805204 | 184.8729579 | 185.1966781 | 166.2419902 | 189.8880209 | 185.3746369 | 217.185623 | 199.1620144 | 221.9362075 | 237.7891943 | 210.9456743 | 195.5176419 | 240.5002922 | 218.1300487 | 192.7867434 | 212.0057551 | 202.7224082 | 204.1819936 |
| ENSMUSG00000026896 | Ifih1 | 8789 | 1.5330687 | 1.368296144 | 2.074959716 | 1.29596781 | 1.785300616 | 1.618117481 | 1.523437039 | 1.16458799 | 1.84165442 | 1.013935933 | 1.516579067 | 1.403488614 | 1.021550682 | 1.342223028 | 1.237593669 | 2.004059552 | 1.735563337 | 1.558455749 |
| ENSMUSG00000026904 | Slc4a10 | 6502 | 0 | 0.046111358 | 0 | 0 | 0.007402634 | 0.045726915 | 0 | 0 | 0.082347694 | 0.007251723 | 0.029390931 | 0 | 0.007587191 | 0.021429139 | 0.05703076 | 0 | 0.00589454 | 0.008460336 |
| ENSMUSG00000026914 | Psmd14 | 2853 | 46.25957643 | 45.23454336 | 45.5589471 | 43.10173805 | 49.5659277 | 44.37687848 | 47.16342757 | 45.54172905 | 47.00430961 | 49.2330687 | 42.56710286 | 40.75149848 | 49.90252831 | 47.66501503 | 48.82665982 | 49.10807307 | 50.69871872 | 46.27474803 |
| ENSMUSG00000026915 | Strbp | 16113 | 9.549353765 | 8.559261594 | 8.697208306 | 9.042650021 | 8.453630235 | 9.133719278 | 8.066029232 | 8.604134595 | 7.63252877 | 7.971111256 | 8.868298939 | 8.434874846 | 9.821683772 | 9.114144127 | 10.02870367 | 10.82203352 | 9.666608987 | 10.00631117 |
| ENSMUSG00000026917 | Wdr5 | 3582 | 15.29569288 | 12.00640604 | 15.30072861 | 13.62252331 | 11.36784127 | 11.95241815 | 14.60854911 | 13.7757074 | 15.05113041 | 14.72965856 | 13.05742165 | 12.1074176 | 10.53571098 | 12.79740766 | 11.00778587 | 12.79560499 | 13.05362429 | 10.21246677 |
| ENSMUSG00000026918 | Brd3 | 9407 | 11.20126835 | 10.5849092 | 9.744641227 | 10.71340811 | 10.7755747 | 10.50895115 | 9.42531735 | 10.48291517 | 11.76444859 | 10.88671494 | 11.34572736 | 11.24976042 | 12.04061678 | 12.01216803 | 12.47828561 | 12.57358204 | 12.0189833 | 12.4263157 |
| ENSMUSG00000026919 | Lcn4 | 767 | 0 | 0 | 0 | 0 | 0 | 0 | 0 | 0 | 0 | 0 | 0 | 0 | 0 | 0 | 0 | 0 | 0 | 0 |

| ENSMUSG00000026921 | Egfl7 | 2904 | 0.644017819 | 0.848882291 | 0.916165348 | 0.188268778 | 0.480656263 | 0.238890589 | 1.629224481 | 1.433530256 | 0.666586171 | 0.470857574 | 0.723863007 | 1.172436439 | 0.543602394 | 0.927602296 | 0.1560665 | 0.039556413 | 0.250757452 | 0.435678187 |
| --- | --- | --- | --- | --- | --- | --- | --- | --- | --- | --- | --- | --- | --- | --- | --- | --- | --- | --- | --- | --- |
| ENSMUSG00000026922 | Agpat2 | 1707 | 32.71927434 | 30.36605941 | 30.46370541 | 29.33309181 | 29.18367955 | 31.38049502 | 33.0107795 | 30.69711547 | 28.78471781 | 26.24087176 | 32.15783673 | 33.28666931 | 25.17170471 | 28.65004336 | 34.37083514 | 34.11834438 | 30.91699522 | 29.71200726 |
| ENSMUSG00000026923 | Notch1 | 17230 | 0.759813942 | 0.958978267 | 0.808565104 | 1.015405747 | 0.759830716 | 0.773632347 | 0.851243788 | 0.942064482 | 0.807953704 | 0.692346368 | 0.831833287 | 0.952397261 | 1.125214364 | 1.078214449 | 0.655207563 | 0.395573309 | 0.51605925 | 0.833277821 |
| ENSMUSG00000026924 | Sec16a | 8477 | 9.602148413 | 11.1095378 | 9.016182371 | 10.65258816 | 9.885298939 | 9.31196232 | 9.538441464 | 10.41651933 | 9.071042223 | 10.37905064 | 8.459385712 | 9.527576237 | 10.89992699 | 8.010057304 | 8.884799359 | 10.68722155 | 9.562359083 | 6.677406018 |
| ENSMUSG00000026925 | Inpp5e | 5514 | 4.887247298 | 7.352519453 | 7.693795089 | 5.866583511 | 6.424573054 | 6.650167365 | 4.539065497 | 6.497002803 | 6.207108197 | 6.584338322 | 6.186311272 | 5.982418678 | 6.620532968 | 6.729928514 | 7.614143172 | 5.777617947 | 6.241750928 | 5.616635307 |
| ENSMUSG00000026926 | Pmpca | 4354 | 22.77551118 | 27.38329086 | 22.49802457 | 23.63858501 | 23.56850282 | 24.90155743 | 25.26457935 | 24.83295618 | 22.06893461 | 26.29350195 | 23.71191669 | 24.26712202 | 24.85857292 | 19.05124944 | 27.02610097 | 24.10531807 | 22.1736201 | 26.56962596 |
| ENSMUSG00000026927 | Sdccag3 | 3780 | 28.291805 | 25.98053232 | 26.74621123 | 29.48209377 | 25.84862567 | 25.58913649 | 24.82038156 | 24.10769104 | 21.60657692 | 26.15741395 | 23.17970208 | 25.6486259 | 23.68715076 | 25.64254243 | 23.51104658 | 23.92656473 | 21.61684259 | 23.09509322 |
| ENSMUSG00000026928 | Card9 | 2504 | 0.475297496 | 0.478939374 | 0.676147589 | 0.655030989 | 1.018766755 | 0.57389348 | 1.681640745 | 0.930405969 | 0.625034984 | 0.734375969 | 0.45790695 | 0.691008602 | 1.989825737 | 2.095919279 | 1.003712024 | 0.504628612 | 0.979585863 | 0.527243834 |
| ENSMUSG00000026930 | Gpsm1 | 4405 | 9.977378436 | 7.804519145 | 8.060424808 | 11.7289953 | 9.014491947 | 10.70924004 | 5.467004217 | 7.335022119 | 5.638017935 | 7.503437681 | 8.958482566 | 7.007051398 | 6.97702258 | 6.136313022 | 8.670565791 | 6.736712993 | 7.656558746 | 8.167072826 |
| ENSMUSG00000026931 | 1700019N19R | 1380 | 0 | 0 | 0 | 0 | 0 | 0 | 0 | 0 | 0 | 0 | 0 | 0 | 0 | 0 | 0 | 0 | 0 | 0 |
| ENSMUSG00000026932 | Nacc2 | 7724 | 14.42886737 | 14.2110288 | 13.30209457 | 14.99432609 | 13.74663693 | 14.10751698 | 11.8097935 | 11.54573532 | 11.15512571 | 13.41145715 | 10.14382857 | 8.924435952 | 8.973503855 | 8.141545096 | 10.40707348 | 7.396373154 | 7.348685359 | 7.321253458 |
| ENSMUSG00000026933 | Camsap1 | 11307 | 3.70280343 | 3.76232121 | 3.837539288 | 3.40085977 | 2.881870728 | 4.316745607 | 1.945732352 | 2.95892319 | 3.311094263 | 2.714699551 | 1.799958637 | 2.349717466 | 2.094217767 | 2.768489865 | 2.718347987 | 2.614340578 | 2.501530655 | 2.734157423 |
| ENSMUSG00000026934 | Lhx3 | 3968 | 0 | 0 | 0 | 0.011482118 | 0 | 0 | 0 | 0 | 0 | 0 | 0 | 0 | 0 | 0 | 0 | 0 | 0 | 0 |
| ENSMUSG00000026936 | Lcn3 | 677 | 0.062784603 | 0 | 0 | 0 | 0 | 0 | 0 | 0 | 0 | 0 | 0 | 0 | 0 | 0 | 0 | 0 | 0 | 0 |
| ENSMUSG00000026937 | Lcn5 | 1011 | 554.7535091 | 425.5878751 | 524.7403634 | 295.0426877 | 284.1259352 | 274.4760713 | 299.1789875 | 370.590967 | 123.6410241 | 34.46525194 | 58.50187794 | 138.7938435 | 0.878313067 | 0.643142654 | 6.642782997 | 1.098345811 | 1.13727884 | 0.816158853 |
| ENSMUSG00000026938 | Fcna | 1798 | 0.141841522 | 0.444665996 | 0.107616308 | 0.07601954 | 0.187387912 | 0.248039267 | 0.263140595 | 0.573523284 | 1.626386329 | 0.052447946 | 0 | 0.465647908 | 0 | 0 | 0 | 0 | 0 | 0 |
| ENSMUSG00000026939 | Tmem141 | 1543 | 24.81993752 | 22.06466487 | 20.87930733 | 24.035444 | 21.7108353 | 22.03057371 | 21.43328747 | 18.63828841 | 21.70099632 | 21.02377436 | 19.78496336 | 22.2466834 | 21.06917271 | 19.98630851 | 16.34177071 | 22.5078295 | 18.77814677 | 17.86102558 |
| ENSMUSG00000026940 | 4921530D09R | 2904 | 0.058547074 | 0.286784558 | 0.28317838 | 0.298092232 | 0.480656263 | 0.34127227 | 0.896073465 | 0.236729767 | 0.255288321 | 0.292256425 | 0.16451432 | 0.172982425 | 0.186863323 | 0.495787434 | 0.411448044 | 0.408749597 | 0.541108186 | 0.265195418 |
| ENSMUSG00000026941 | Mamdc4 | 4716 | 0.612882097 | 0.240169297 | 0.512866098 | 0.434742789 | 0.377625355 | 0.294206221 | 0.591910105 | 0.477809954 | 0.209600589 | 0.339932975 | 0.344433543 | 0.579933729 | 0.251053014 | 0.649980867 | 0.454300447 | 0.487157855 | 0.162537307 | 0.711525766 |
| ENSMUSG00000026942 | Traf2 | 5853 | 9.331821493 | 10.67742859 | 9.644953003 | 9.932665739 | 9.892824906 | 9.6091496 | 11.57556061 | 9.318084851 | 8.824174059 | 7.926924997 | 8.342048106 | 9.183407325 | 8.386341652 | 10.48225516 | 7.109784973 | 13.29344169 | 11.07946256 | 11.09956519 |
| ENSMUSG00000026943 | Lcn12 | 889 | 11.28371377 | 11.80378142 | 12.51508659 | 10.65995188 | 8.229530256 | 10.36761355 | 21.60736518 | 7.69003378 | 8.987833586 | 9.812013614 | 5.750191853 | 6.717970638 | 118.1968322 | 141.526471 | 72.29969313 | 22.26799103 | 24.83233635 | 54.8853517 |
| ENSMUSG00000026944 | Abca2 | 8248 | 15.59931715 | 19.10806287 | 15.77066236 | 17.88635567 | 15.64520943 | 17.34465832 | 14.69630012 | 16.67907777 | 14.76083179 | 17.56146465 | 14.20853215 | 13.49374772 | 15.61060913 | 20.37841322 | 19.82150506 | 13.40728348 | 13.91698099 | 18.0607009 |
| ENSMUSG00000026946 | Nmi | 2267 | 18.97452057 | 20.42563871 | 18.56416913 | 17.82648713 | 19.21455268 | 19.01670852 | 16.44569519 | 17.55469047 | 16.27839353 | 16.16060716 | 15.65804771 | 17.60396945 | 15.27613848 | 17.51640702 | 15.8481503 | 20.91035376 | 17.68385821 | 17.39811624 |
| ENSMUSG00000026950 | Neb | 26653 | 0.059006172 | 0.046245341 | 0.061707876 | 0.129915558 | 0.041535071 | 0.037183607 | 0.063904868 | 0.008597697 | 0.020088722 | 0.01769058 | 0.030472153 | 0.025129927 | 0.027763432 | 0.006970185 | 0.010820954 | 0.037352485 | 0.014379731 | 0.010319496 |
| ENSMUSG00000026955 | 2010317E24R | 4084 | 0.041630927 | 0.122353921 | 0.082912515 | 0.011155985 | 0.023570971 | 0 | 0 | 0.056110288 | 0 | 0 | 0 | 0 | 0 | 0 | 0 | 0.084381848 | 0.131382996 | 0 |
| ENSMUSG00000026956 | Uap1l1 | 3999 | 50.88078961 | 47.09122056 | 47.79290283 | 47.90802486 | 47.55419615 | 52.01859854 | 52.35273925 | 49.471529 | 49.78635089 | 46.34894086 | 48.58733587 | 51.90758919 | 54.50072781 | 55.97912008 | 54.99722564 | 64.23898101 | 50.98672156 | 54.87154957 |
| ENSMUSG00000026958 | Dpp7 | 2454 | 4.7458917 | 5.104176131 | 3.666453401 | 5.309885363 | 4.27577806 | 7.148193841 | 6.362340687 | 5.120334308 | 3.33990083 | 5.09166111 | 5.820991273 | 4.503464218 | 6.010694077 | 5.469576714 | 6.41360813 | 6.147717733 | 4.997724128 | 3.877985111 |
| ENSMUSG00000026959 | Grin1 | 7778 | 0 | 0 | 0 | 0 | 0.012376427 | 0 | 0.030414425 | 0.039282492 | 0 | 0 | 0.006142319 | 0 | 0.019027482 | 0 | 0 | 0.054152312 | 0.059130311 | 0 |
| ENSMUSG00000026960 | Arl6ip6 | 2698 | 5.640049302 | 9.346872099 | 7.135902551 | 7.447153652 | 9.187524347 | 7.548618792 | 11.15302587 | 7.290247309 | 8.747173288 | 9.000226901 | 9.491244531 | 7.220039378 | 8.264650337 | 9.674417017 | 9.086332748 | 8.799175904 | 9.389800687 | 7.931261125 |
| ENSMUSG00000026961 | Lrrc26 | 1188 | 0.679796586 | 1.598345935 | 1.628738396 | 1.687446087 | 0.89133192 | 2.252396981 | 1.712495954 | 1.253790988 | 2.49615247 | 1.706633199 | 2.050945187 | 1.832332358 | 1.744057681 | 1.211924839 | 0.520221665 | 0.032231151 | 0.774268624 | 1.203903009 |
| ENSMUSG00000026963 | Tmem210 | 575 | 0 | 0 | 0 | 0 | 0 | 0 | 0 | 0 | 0.214886169 | 0.410006115 | 0 | 0 | 0 | 0.484633955 | 0.358274399 | 0 | 0 | 0 |
| ENSMUSG00000026965 | Anapc2 | 3252 | 55.1312523 | 60.33608455 | 58.50341171 | 56.4889701 | 58.04840241 | 62.29138219 | 46.27971454 | 49.50214518 | 54.64938976 | 47.61467082 | 48.89147042 | 48.41819931 | 48.96784409 | 47.41524297 | 54.37794523 | 43.33008464 | 49.29855472 | 52.11656144 |
| ENSMUSG00000026966 | Ssna1 | 1741 | 30.12716095 | 27.72566555 | 25.53433337 | 25.90777363 | 25.37915332 | 26.98218922 | 31.14321083 | 29.06659 | 28.76668763 | 30.35952806 | 34.13673081 | 25.968229 | 34.03080566 | 33.82602114 | 29.88946875 | 31.82453125 | 24.96382578 | 25.15063122 |
| ENSMUSG00000026969 | Fam166a | 1118 | 0 | 0.059593728 | 0.086535832 | 0.244513655 | 0 | 0.088645319 | 0.042319033 | 0.034161362 | 0.221036757 | 1.265224595 | 0 | 0.49924564 | 0.529501795 | 1.495516231 | 0.958175718 | 0.308242815 | 0.89130923 | 0.393625093 |
| ENSMUSG00000026970 | Rbms1 | 6493 | 14.6702756 | 15.03774726 | 13.38783829 | 13.68304889 | 14.78131153 | 14.72921235 | 10.28156322 | 11.53477608 | 13.35881753 | 11.86573989 | 12.44223853 | 13.16950486 | 11.14583746 | 11.08706845 | 11.33947026 | 15.49197992 | 14.49705609 | 13.86876755 |
| ENSMUSG00000026971 | Itgb6 | 7365 | 2.937560706 | 2.876714287 | 2.92277271 | 2.684792022 | 3.568232223 | 3.061302618 | 2.858675102 | 2.670616054 | 3.249065262 | 2.695240471 | 2.672543502 | 4.403109296 | 2.645771531 | 1.122477604 | 2.987322586 | 2.859447939 | 4.340004293 | 3.495487026 |
| ENSMUSG00000026972 | Arrdc1 | 2727 | 102.0467134 | 99.25458949 | 100.7914923 | 99.10821966 | 103.2356515 | 102.8123823 | 79.13206036 | 92.12674112 | 95.98104425 | 85.60437544 | 95.30464764 | 102.6254977 | 95.44378275 | 91.38942902 | 94.03684777 | 101.2798501 | 92.14052163 | 97.41069907 |
| ENSMUSG00000026974 | Zmynd19 | 4161 | 4.80111337 | 4.675493925 | 3.743397446 | 3.350559857 | 3.562757147 | 2.870033356 | 4.84383591 | 4.708652351 | 4.375015613 | 2.991537049 | 4.718939662 | 3.541296545 | 4.517053709 | 5.268351177 | 4.049852526 | 4.233039992 | 4.384358886 | 3.833847861 |
| ENSMUSG00000026975 | Wdr85 | 1863 | 21.85719733 | 21.35029291 | 27.73104156 | 24.1622715 | 24.54392243 | 22.66179764 | 21.76434023 | 19.27045532 | 17.68610446 | 21.81637478 | 26.6955082 | 24.14783899 | 22.69321154 | 24.58071399 | 25.10132364 | 32.53571203 | 24.41938509 | 26.01343157 |
| ENSMUSG00000026976 | Pax8 | 6290 | 23.48934688 | 19.00264936 | 20.44916013 | 21.02761712 | 20.63780574 | 20.67191938 | 24.32578754 | 21.58569007 | 23.18624043 | 22.23354306 | 21.41138441 | 24.72216788 | 24.03852567 | 24.53635968 | 22.28421195 | 24.0214208 | 24.19004749 | 27.31215267 |
| ENSMUSG00000026977 | 7-Mar | 10175 | 15.91176565 | 16.80868785 | 18.91202985 | 20.08268146 | 19.65485433 | 20.57108075 | 14.67971768 | 16.93603142 | 14.62880275 | 16.00111827 | 18.42446556 | 18.81001543 | 12.83357101 | 13.91268583 | 15.7072074 | 18.31175387 | 20.86381902 | 16.9541581 |

| ENSMUSG00000026979 | Psd4 | 6185 | 2.563367932 | 3.08622286 | 2.448005659 | 3.859981767 | 2.887137216 | 3.076515714 | 1.583463951 | 2.272401631 | 1.345137621 | 2.485227043 | 1.815216692 | 2.030480854 | 1.49949886 | 2.004942819 | 3.324102892 | 3.813583534 | 3.31520919 | 1.814366657 |
| --- | --- | --- | --- | --- | --- | --- | --- | --- | --- | --- | --- | --- | --- | --- | --- | --- | --- | --- | --- | --- |
| ENSMUSG00000026980 | Ly75 | 8375 | 1.619003123 | 1.829723145 | 2.0908917 | 2.143409131 | 2.080448522 | 2.550116797 | 1.525304276 | 1.618901833 | 1.893350277 | 1.463783027 | 1.602956816 | 1.966044234 | 0.577257062 | 1.003746844 | 1.175781714 | 1.170435283 | 1.116610919 | 1.195421782 |
| ENSMUSG00000026981 | Il1rn | 3336 | 0 | 0.738955088 | 0 | 0.027314775 | 0.144280347 | 0.207955117 | 0 | 0.263316923 | 0 | 0 | 0 | 0 | 0 | 0 | 0.123505863 | 0 | 0 | 0.01648954 |
| ENSMUSG00000026983 | Il1f5 | 1832 | 10.78870461 | 9.746567304 | 6.997262852 | 6.913739261 | 9.878604421 | 8.520257138 | 6.327296036 | 3.252191471 | 2.225690535 | 3.526007831 | 4.250719559 | 2.25456279 | 5.089373562 | 7.022386576 | 4.947785096 | 2.487217402 | 5.439321613 | 6.93619195 |
| ENSMUSG00000026984 | Il1f6 | 907 | 0 | 0 | 0 | 0 | 0 | 0 | 0 | 0 | 0 | 0 | 0 | 0 | 0 | 0 | 0 | 0 | 0.042256116 | 0 |
| ENSMUSG00000026985 | Il1f8 | 1212 | 0 | 0 | 0 | 0 | 0 | 0 | 0 | 0 | 0 | 0 | 0 | 0 | 0 | 0 | 0 | 0 | 0 | 0 |
| ENSMUSG00000026986 | Hnmt | 2775 | 6.816145345 | 9.639731192 | 10.12793554 | 10.06447574 | 10.19876437 | 8.374858396 | 14.54332077 | 12.84090502 | 17.11281177 | 15.10521629 | 12.86050234 | 12.12859263 | 12.74630079 | 11.8327819 | 11.95216306 | 12.00462286 | 10.8142308 | 9.415973223 |
| ENSMUSG00000026987 | Baz2b | 18996 | 8.167187434 | 7.083111168 | 7.025824924 | 6.993893729 | 7.510161179 | 6.93884351 | 7.783329209 | 8.197011192 | 7.111590424 | 7.195719559 | 8.063093119 | 8.667940863 | 7.014398219 | 7.630658915 | 7.211790553 | 7.427926315 | 7.683014245 | 6.643024364 |
| ENSMUSG00000026988 | Wdsub1 | 3425 | 17.99489204 | 18.89838057 | 17.17436873 | 19.02256742 | 17.41181122 | 18.57684961 | 17.14307578 | 17.8194041 | 17.88156173 | 16.32722162 | 18.63572103 | 15.79135096 | 16.29033531 | 15.05195241 | 12.64316357 | 14.92495208 | 17.14332463 | 14.63161934 |
| ENSMUSG00000026989 | Dapl1 | 771 | 6.50533174 | 4.191116393 | 5.583974321 | 4.254727876 | 4.744521659 | 4.241868244 | 34.11913033 | 25.26345684 | 33.6543514 | 21.09856371 | 14.87158241 | 25.55503099 | 38.45458143 | 31.14344122 | 21.21532774 | 7.896506585 | 10.09110022 | 22.40319003 |
| ENSMUSG00000026991 | Pkp4 | 5912 | 9.777916001 | 6.94206456 | 7.674961345 | 8.600494837 | 9.590562598 | 7.635747681 | 11.79615845 | 9.787126124 | 11.87107964 | 11.8833809 | 12.4286005 | 10.79115398 | 12.99218457 | 9.482072645 | 9.68013551 | 12.35765882 | 12.19414149 | 12.56128114 |
| ENSMUSG00000026994 | Galnt3 | 4569 | 24.15975972 | 25.78121995 | 25.21903027 | 28.02069043 | 26.5994982 | 25.94224966 | 30.21632679 | 27.87736092 | 29.07124389 | 28.29661377 | 29.72733792 | 30.6014959 | 30.10229489 | 34.60181078 | 32.81515909 | 23.68335665 | 35.46587509 | 31.44753486 |
| ENSMUSG00000026999 | Nup35 | 3354 | 1.850255128 | 1.668624397 | 1.168233738 | 1.222568274 | 1.262853096 | 1.167163373 | 2.539141982 | 1.913036256 | 1.363060003 | 1.377689004 | 1.43866154 | 1.913774955 | 2.118007182 | 1.066247683 | 1.904067133 | 2.32894571 | 1.542650591 | 1.754911872 |
| ENSMUSG00000027001 | Dusp19 | 3493 | 1.752288963 | 1.144445264 | 1.814180497 | 2.073920999 | 1.694883084 | 1.900963727 | 2.383919694 | 2.011852861 | 2.169573118 | 2.200276161 | 1.983214709 | 1.518032621 | 2.55627742 | 2.526303064 | 1.89906971 | 2.203381645 | 2.040850622 | 1.96854805 |
| ENSMUSG00000027002 | Nckap1 | 5826 | 36.91661358 | 47.19040783 | 48.48977296 | 47.20330649 | 51.14739777 | 49.38262464 | 39.33790194 | 43.81047464 | 38.18907624 | 42.16532166 | 41.85434056 | 43.79220626 | 29.45858912 | 36.11254993 | 38.47175833 | 32.51349722 | 44.68769908 | 34.26502717 |
| ENSMUSG00000027004 | Frzb | 3589 | 0 | 0 | 0 | 0 | 0 | 0 | 0 | 0 | 0 | 0 | 0 | 0 | 0 | 0 | 0 | 0 | 0 | 0 |
| ENSMUSG00000027006 | Dnajc10 | 4982 | 27.5063604 | 29.96956883 | 31.93507454 | 31.3678005 | 28.19127926 | 29.74954358 | 37.89192873 | 38.66769926 | 36.31721472 | 28.02353118 | 25.11493702 | 34.92120038 | 31.98355937 | 44.51435514 | 29.52419802 | 27.27686987 | 28.07928215 | 31.35806164 |
| ENSMUSG00000027007 | Ssfa2 | 6076 | 5.988220981 | 5.861007887 | 7.029925494 | 6.846154292 | 7.59685892 | 6.402056593 | 6.392973897 | 7.706366911 | 8.086819174 | 7.03070065 | 8.177412248 | 8.947408716 | 7.818735408 | 7.536844991 | 10.21223555 | 7.03927065 | 8.566015667 | 9.116881249 |
| ENSMUSG00000027009 | Itga4 | 14127 | 0.00601758 | 0 | 0.047938658 | 0 | 0 | 0 | 0.056934632 | 0.062180594 | 0.049562594 | 0.050064455 | 0.067636382 | 0.071117854 | 0.048888429 | 0.11177879 | 0.078745807 | 0.046077747 | 0 | 0 |
| ENSMUSG00000027010 | Slc25a12 | 7983 | 8.998339908 | 8.400207449 | 7.883497807 | 9.074540961 | 8.465141028 | 8.690195041 | 11.83557808 | 10.91758265 | 10.59715688 | 11.3107349 | 15.48212672 | 10.80934666 | 15.14007232 | 13.24148101 | 12.95451651 | 12.51891336 | 15.07990712 | 13.09248438 |
| ENSMUSG00000027011 | Ube2e3 | 4546 | 21.70138882 | 21.08986132 | 21.23923594 | 20.37518767 | 22.96483556 | 20.04563947 | 19.98247768 | 22.38951879 | 24.28065601 | 20.49489433 | 17.91823674 | 20.12359678 | 23.9823003 | 20.72922422 | 22.19591077 | 28.35155841 | 24.38179733 | 20.38942913 |
| ENSMUSG00000027012 | Dync1i2 | 8366 | 13.35209211 | 13.14835964 | 14.83701636 | 12.62922087 | 13.60650245 | 14.39315594 | 13.34663188 | 12.23015133 | 13.36617144 | 12.40481686 | 11.60395836 | 13.53026102 | 12.78997473 | 13.21818935 | 13.72071895 | 13.0213696 | 13.9268399 | 12.68378758 |
| ENSMUSG00000027014 | Cwc22 | 6797 | 2.038647548 | 3.568013387 | 2.661718457 | 2.942665656 | 2.683830965 | 2.609957131 | 2.874817772 | 2.067795216 | 3.205482836 | 2.663803156 | 3.416011449 | 2.603143304 | 2.852353022 | 2.767376106 | 3.12785094 | 2.546322575 | 3.106927997 | 1.351555219 |
| ENSMUSG00000027015 | Cybrd1 | 5335 | 0.007967231 | 0.174838058 | 0.181344069 | 0.076860243 | 0.054131498 | 0.01857647 | 0.106420271 | 0.257718179 | 0.007720059 | 0.026513985 | 0 | 0 | 0.101715293 | 0.008705546 | 0.007722878 | 0 | 0.107759035 | 0.020621971 |
| ENSMUSG00000027016 | Zfp385b | 9245 | 0.036781115 | 0.025223392 | 0.047091593 | 0.039425458 | 0.036443858 | 0.112558941 | 1.028647752 | 0.578359798 | 0.614790608 | 0.214205466 | 0.325562183 | 0.229420787 | 0.677680205 | 1.014786982 | 0.450119756 | 0.484586378 | 0.223863714 | 0.618815262 |
| ENSMUSG00000027018 | Hat1 | 2602 | 22.00402464 | 21.62393479 | 19.98522103 | 20.38165089 | 22.10516863 | 20.62475421 | 17.710434 | 21.00435351 | 23.36329641 | 25.73174428 | 25.08093516 | 20.48576394 | 23.22505713 | 21.57990071 | 22.92846 | 27.63634154 | 24.02390094 | 20.80282898 |
| ENSMUSG00000027022 | Xirp2 | 11970 | 0 | 0 | 0 | 0 | 0 | 0 | 0 | 0 | 0 | 0 | 0 | 0 | 0 | 0 | 0 | 0 | 0 | 0 |
| ENSMUSG00000027030 | Stk39 | 6479 | 30.17808454 | 30.44384113 | 29.58856317 | 30.54045617 | 29.15848131 | 32.11482147 | 28.72790228 | 27.87650424 | 28.31374305 | 29.52468022 | 25.19633171 | 25.93072147 | 30.0757946 | 27.5195017 | 28.24141221 | 33.37352369 | 37.94770715 | 30.27665914 |
| ENSMUSG00000027035 | Lass6 | 8299 | 8.778632572 | 8.032184761 | 9.011384255 | 9.805039786 | 7.533843697 | 7.821916012 | 5.039692514 | 5.191110967 | 4.818906712 | 5.698536615 | 5.480390465 | 5.582238818 | 4.285855206 | 6.077633318 | 6.756876439 | 5.158320168 | 5.527964501 | 5.183410225 |
| ENSMUSG00000027048 | Abcb11 | 5684 | 0.007478039 | 0 | 0 | 0 | 0 | 0 | 0 | 0 | 0 | 0 | 0 | 0 | 0 | 0 | 0 | 0 | 0 | 0 |
| ENSMUSG00000027067 | Ssrp1 | 3758 | 43.6814768 | 47.53159628 | 45.7862287 | 47.1735028 | 46.5437495 | 45.75518508 | 39.07891309 | 42.4506825 | 42.18384757 | 46.17205641 | 48.99539382 | 42.43356786 | 35.57463965 | 34.25838484 | 40.23675096 | 38.09701466 | 40.49859633 | 39.08310669 |
| ENSMUSG00000027068 | Dhrs9 | 4276 | 0 | 0 | 0 | 0 | 0 | 0.081120004 | 0 | 0 | 0 | 0 | 0 | 0 | 0 | 0 | 0 | 0 | 0 | 0 |
| ENSMUSG00000027070 | Lrp2 | 16621 | 0.002557318 | 0.036076776 | 0.040745408 | 0.002741173 | 0.037646051 | 0.005962666 | 0.714486638 | 0.866285765 | 0.634363037 | 0.326233733 | 0.132221171 | 0.362679235 | 1.926262819 | 3.705243961 | 1.745135392 | 0.23267862 | 0.396614107 | 1.893099636 |
| ENSMUSG00000027071 | P2rx3 | 3258 | 0.065232007 | 0.061349713 | 0.02969523 | 0 | 0.029546914 | 0.015209556 | 0.304962019 | 0.422015496 | 0.088491593 | 0.072361423 | 0.10264724 | 0 | 0 | 0.114043186 | 0.164401543 | 0.15278634 | 0 | 0 |
| ENSMUSG00000027072 | Prg3 | 1049 | 0 | 0 | 0 | 0 | 0 | 0 | 0 | 0 | 0 | 0 | 0 | 0 | 0 | 0 | 0 | 0 | 0 | 0 |
| ENSMUSG00000027073 | Prg2 | 832 | 0.459791568 | 0.200197682 | 0 | 0.109521741 | 0.05785087 | 0 | 0 | 0 | 0 | 0 | 0 | 0 | 0.533638528 | 0 | 0 | 0 | 0 | 0 |
| ENSMUSG00000027074 | Slc43a3 | 4477 | 9.275755412 | 13.85494953 | 11.8529736 | 11.5098372 | 11.98728945 | 14.8093718 | 1.035658373 | 2.951657635 | 3.302648907 | 2.064225561 | 3.126661343 | 2.156825916 | 4.859364646 | 4.834251669 | 4.555454583 | 11.28961398 | 6.489017881 | 4.12844759 |
| ENSMUSG00000027075 | Slc43a1 | 5098 | 0.525269927 | 0.725329788 | 0.673699619 | 0.607718912 | 0.811954774 | 0.505441771 | 1.577708007 | 2.135118612 | 2.383291909 | 1.79427941 | 2.417798997 | 1.47805305 | 1.480538121 | 3.999402582 | 3.717676679 | 2.283315938 | 3.728882555 | 3.550018853 |
| ENSMUSG00000027076 | Timm10 | 811 | 27.83014608 | 22.14013562 | 23.56047409 | 23.87600967 | 23.79889197 | 23.27939765 | 28.11924938 | 25.14764844 | 21.68513222 | 23.83697699 | 23.68129878 | 26.42813124 | 28.89353971 | 25.54138468 | 27.12901461 | 25.63723775 | 24.3379074 | 29.03069997 |
| ENSMUSG00000027077 | Smtnl1 | 1753 | 4.606950054 | 3.496618674 | 5.601726561 | 4.158452421 | 5.793403248 | 5.766547433 | 6.234585757 | 5.032769513 | 3.829664615 | 6.213241559 | 4.033481948 | 4.553131631 | 5.90969916 | 2.437453531 | 2.914428369 | 2.948791785 | 5.487678564 | 4.487337285 |
| ENSMUSG00000027078 | Ube2l6 | 2089 | 35.85166116 | 32.19661725 | 35.10496506 | 30.20681972 | 35.20611746 | 36.29281109 | 35.08249864 | 38.81401164 | 42.92151499 | 32.27645077 | 42.12612423 | 39.86451344 | 45.69519384 | 38.59594816 | 36.78358149 | 44.7426389 | 42.61942925 | 45.95064204 |

| ENSMUSG00000027079 | Clp1 | 2668 | 50.69395432 | 55.25095837 | 58.88951521 | 53.1260903 | 51.83021615 | 49.47844162 | 62.26192493 | 66.04937957 | 72.91001272 | 67.08548335 | 69.44201236 | 68.51435344 | 76.54952681 | 78.17855939 | 85.52256991 | 74.2562229 | 84.84074569 | 71.87471741 |
| --- | --- | --- | --- | --- | --- | --- | --- | --- | --- | --- | --- | --- | --- | --- | --- | --- | --- | --- | --- | --- |
| ENSMUSG00000027080 | Med19 | 2054 | 47.65794566 | 46.83916188 | 49.315566 | 45.60540757 | 46.18696278 | 42.96661076 | 42.24510864 | 41.0930912 | 45.35730217 | 43.7532816 | 45.75138429 | 41.52207032 | 49.37995225 | 41.35649262 | 41.44226605 | 55.03109691 | 47.26412369 | 40.7881546 |
| ENSMUSG00000027082 | Tfpi | 5497 | 0.108254041 | 0.07878254 | 0.105599848 | 0.281803803 | 0.411533639 | 0.234377128 | 0.791843999 | 0.625307662 | 0.449552655 | 0.780555575 | 0.921256249 | 0.81230726 | 1.453842205 | 1.098368448 | 0.70455635 | 1.553357368 | 0.829694257 | 1.691202299 |
| ENSMUSG00000027086 | Fastkd1 | 6042 | 2.026065987 | 1.295685227 | 1.457130507 | 1.591092412 | 0.629331673 | 1.369630339 | 2.69373743 | 1.042990135 | 1.622375166 | 2.122640067 | 1.684221476 | 1.505785005 | 2.424955219 | 0.899364155 | 1.541137311 | 1.584351514 | 0.634331296 | 1.16537002 |
| ENSMUSG00000027087 | Itgav | 8531 | 12.95433802 | 17.08794105 | 13.93198501 | 15.83501306 | 16.64390749 | 16.65311068 | 10.149127 | 15.2751702 | 12.94350589 | 13.61848937 | 13.9723973 | 12.90217872 | 12.54260093 | 13.1149697 | 13.175225 | 16.09094216 | 17.07184717 | 12.57387857 |
| ENSMUSG00000027088 | Phospho2 | 3268 | 29.81085173 | 24.53614944 | 27.48765173 | 30.46232615 | 26.83487299 | 21.06142807 | 32.64690667 | 29.38056905 | 30.51179763 | 34.61277145 | 29.8228015 | 30.11108113 | 31.50419564 | 25.28275137 | 34.72126222 | 29.03431002 | 33.91666176 | 30.90474906 |
| ENSMUSG00000027091 | Zc3h15 | 3025 | 27.14710747 | 24.91033611 | 26.78534325 | 26.43293647 | 26.57200414 | 28.37201142 | 31.84417002 | 26.6147386 | 30.04913728 | 29.95823195 | 26.24858876 | 26.45939178 | 36.95409076 | 33.96175911 | 31.69455225 | 35.06280413 | 31.45923809 | 35.93322145 |
| ENSMUSG00000027099 | Mtx2 | 3878 | 46.46194976 | 43.22601437 | 44.36942947 | 44.16296175 | 47.10176646 | 42.56321701 | 41.5053465 | 41.0386129 | 42.65215248 | 41.2295087 | 40.2723928 | 42.60298124 | 40.16009872 | 41.44997072 | 38.92792697 | 43.8594322 | 44.85896381 | 38.95178109 |
| ENSMUSG00000027102 | Hoxd8 | 2115 | 109.5488012 | 112.9173232 | 121.3569513 | 114.7535145 | 116.9730911 | 126.3770425 | 116.7940883 | 112.6269569 | 105.7217939 | 114.387829 | 111.1360736 | 126.0404749 | 106.6175858 | 105.690493 | 117.5656689 | 100.8771936 | 111.390897 | 112.6191168 |
| ENSMUSG00000027104 | Atf2 | 10919 | 7.73104493 | 10.43106377 | 9.272442443 | 10.23131062 | 9.50383986 | 9.312410413 | 7.968496799 | 9.010314919 | 8.128669426 | 8.826452739 | 8.1207366 | 9.042760978 | 8.019429725 | 8.111445613 | 8.591990362 | 7.918325077 | 10.26340252 | 9.919674983 |
| ENSMUSG00000027107 | Chrna1 | 4377 | 0 | 0 | 0 | 0 | 0 | 0 | 0 | 0.017451406 | 0 | 0.053861895 | 0 | 0 | 0 | 0 | 0 | 0 | 0 | 0 |
| ENSMUSG00000027108 | Ola1 | 4071 | 11.88182027 | 11.34160437 | 10.18327573 | 10.66560434 | 12.54433088 | 11.44180043 | 12.07513471 | 10.91077475 | 11.78636474 | 9.879525885 | 11.41857887 | 11.85962863 | 11.83917543 | 10.42738783 | 9.462897263 | 10.22399662 | 11.94695917 | 10.80994482 |
| ENSMUSG00000027109 | Sp3 | 6610 | 10.87386576 | 10.67927728 | 10.62607656 | 9.925552775 | 10.67494706 | 9.385782517 | 8.775392491 | 9.741662701 | 9.776345421 | 10.49299312 | 8.803313075 | 10.34405245 | 8.642414555 | 10.42005773 | 11.15122292 | 10.7341143 | 10.48899714 | 5.900371659 |
| ENSMUSG00000027111 | Itga6 | 8994 | 2.145580378 | 2.30753092 | 2.533236913 | 2.426477678 | 2.038944065 | 2.236870117 | 1.441369138 | 2.263347841 | 1.584449016 | 2.112712187 | 2.145995466 | 1.66317518 | 2.55051607 | 2.587112273 | 2.762345809 | 3.772012248 | 2.735766357 | 2.562687983 |
| ENSMUSG00000027115 | Kif18a | 7225 | 0.194141288 | 0.212095936 | 0.267811933 | 0.271159157 | 0.419697051 | 0.260623374 | 0.47803814 | 0.428177799 | 0.034203335 | 0.261041956 | 0.138861471 | 0.293563346 | 0.273117881 | 0.347125359 | 0.262321325 | 0.27028664 | 0.2864526 | 0.144660627 |
| ENSMUSG00000027120 | Fshb | 1597 | 0 | 0 | 0 | 0 | 0 | 0 | 0 | 0 | 0 | 0 | 0 | 0 | 0 | 0 | 0 | 0 | 0 | 0 |
| ENSMUSG00000027122 | 2700007P21R | 5748 | 4.636524942 | 4.88565933 | 4.670721877 | 5.21558232 | 4.49666719 | 4.741475899 | 5.144471874 | 4.704283386 | 4.664652359 | 3.814383614 | 3.482551775 | 4.884355999 | 2.832208194 | 2.609853902 | 4.687859699 | 4.603133214 | 4.22736121 | 4.134296121 |
| ENSMUSG00000027130 | Slc12a6 | 11024 | 4.137160185 | 4.862159543 | 4.690793174 | 4.818956612 | 4.30061183 | 4.162357701 | 4.154451488 | 4.954203142 | 4.102212836 | 4.435348265 | 4.273050532 | 4.298575611 | 3.857412254 | 4.954485375 | 4.996959382 | 4.331312358 | 5.124542964 | 5.399115881 |
| ENSMUSG00000027131 | Tmem85 | 1047 | 114.037287 | 105.4431055 | 111.6241159 | 97.0402377 | 98.79226739 | 99.20012376 | 112.9268239 | 119.0639937 | 115.1806286 | 109.0275574 | 111.3836425 | 109.125751 | 135.4625236 | 133.6986432 | 120.4171548 | 140.4354655 | 116.772576 | 146.6384115 |
| ENSMUSG00000027132 | 2410042D21R | 4172 | 8.639594744 | 8.982983221 | 7.32791735 | 6.126497093 | 7.983530963 | 7.637202227 | 7.269277851 | 7.717208827 | 8.08527239 | 6.125522811 | 5.691312168 | 8.455296921 | 7.106539378 | 9.362290866 | 7.288290567 | 7.268974368 | 8.846650028 | 7.040954696 |
| ENSMUSG00000027133 | Nop10 | 1107 | 79.48122347 | 68.37117946 | 76.9519304 | 74.0418417 | 78.56764776 | 69.15896421 | 111.3365209 | 81.42192383 | 96.9948027 | 93.70510132 | 93.30574547 | 89.54707927 | 117.6924964 | 105.5166032 | 95.20648597 | 94.49859026 | 81.8112372 | 94.61367588 |
| ENSMUSG00000027134 | Lpcat4 | 2811 | 14.89420078 | 22.76558869 | 19.58344986 | 18.96350831 | 17.80761317 | 18.77398124 | 7.203780356 | 7.662936662 | 10.16842474 | 9.225502238 | 6.475367905 | 6.036272297 | 5.738718232 | 6.625428333 | 6.302621499 | 8.840485309 | 6.503608546 | 5.205415293 |
| ENSMUSG00000027157 | 4930430A15R | 2415 | 0 | 0 | 0 | 0 | 0 | 0 | 0 | 0 | 0 | 0 | 0 | 0 | 0 | 0 | 0 | 0 | 0 | 0 |
| ENSMUSG00000027160 | Ccdc34 | 4504 | 5.181026119 | 5.317933158 | 5.477464583 | 5.391659998 | 5.215004163 | 4.906864826 | 6.145185873 | 4.511180746 | 4.864837119 | 6.134616365 | 4.720216812 | 5.056125741 | 7.261780896 | 6.424215463 | 5.644174064 | 5.024367001 | 5.718310728 | 6.741791055 |
| ENSMUSG00000027162 | Lin7c | 4567 | 29.72663285 | 35.81482606 | 38.27938224 | 33.26046022 | 34.84215889 | 34.95925281 | 27.98150773 | 33.93579351 | 24.00667943 | 31.11719282 | 26.80084161 | 31.88593468 | 22.10052611 | 28.06780845 | 30.15914196 | 30.60230283 | 38.77946529 | 29.73888428 |
| ENSMUSG00000027163 | Commd9 | 1661 | 36.49149971 | 26.49387915 | 27.89996513 | 28.38993431 | 31.527714 | 27.92375595 | 29.65231714 | 26.74157977 | 24.02753388 | 28.95467629 | 31.20760382 | 25.94201777 | 30.68023513 | 30.98136594 | 29.93996265 | 29.04645715 | 21.85129631 | 26.39509817 |
| ENSMUSG00000027164 | Traf6 | 8055 | 7.820319165 | 7.216759828 | 8.095284782 | 7.664210438 | 7.768032373 | 7.874301549 | 6.725390115 | 5.263012622 | 7.020370719 | 7.38723619 | 7.259658495 | 7.088693088 | 4.519795773 | 6.394350455 | 8.475602497 | 6.193998938 | 9.002278553 | 5.975539216 |
| ENSMUSG00000027165 | B230118H07R | 4804 | 10.59090252 | 6.518343165 | 7.10901591 | 7.008661899 | 7.834963428 | 7.241053075 | 10.99103865 | 9.261896087 | 13.66596714 | 10.52155576 | 10.76030497 | 9.899030917 | 13.56525036 | 10.43155085 | 9.948752047 | 10.76823701 | 10.53893385 | 10.397225 |
| ENSMUSG00000027166 | Dnajc24 | 1673 | 15.34556265 | 12.42513209 | 14.54386478 | 12.77234297 | 14.47122391 | 12.11420683 | 14.11179126 | 12.9438686 | 13.44162439 | 16.12086208 | 15.39193223 | 13.17823474 | 19.4909727 | 17.26731761 | 20.73622837 | 17.71484168 | 16.21937729 | 18.0185239 |
| ENSMUSG00000027167 | Elp4 | 7013 | 1.727359927 | 1.596054821 | 1.883070553 | 1.741103648 | 2.2785967 | 1.773525756 | 1.713591965 | 2.085796395 | 1.808847405 | 1.815298714 | 1.737147357 | 1.249544992 | 2.321336475 | 2.21193857 | 2.015133847 | 1.916441351 | 2.442871056 | 2.266880343 |
| ENSMUSG00000027168 | Pax6 | 17502 | 0 | 0.003806753 | 0.011055543 | 0.005206381 | 0.027500813 | 0.005662522 | 0.029736 | 0.006546521 | 0.007059739 | 0.018858126 | 0.035485914 | 0.003189102 | 0.036642379 | 0.013268223 | 0 | 0.013126708 | 0.008759295 | 0.003143018 |
| ENSMUSG00000027170 | Eif3m | 2330 | 68.00828164 | 58.30471357 | 58.46345986 | 49.19810624 | 56.58083216 | 52.78535826 | 49.93213626 | 52.0760783 | 56.84799772 | 56.17611684 | 54.58237746 | 55.00118511 | 56.8481536 | 49.89251105 | 52.78396753 | 54.32993479 | 56.65054358 | 48.06804347 |
| ENSMUSG00000027171 | Prrg4 | 3159 | 6.727631533 | 5.673421048 | 3.537285695 | 2.956636305 | 4.403332048 | 4.517628132 | 2.950489949 | 3.373118161 | 3.076928686 | 2.985166398 | 2.918824624 | 3.07436698 | 2.092585285 | 0.793916024 | 1.121663123 | 2.47270779 | 2.280893896 | 1.253768814 |
| ENSMUSG00000027173 | Depdc7 | 2087 | 12.70878287 | 9.226091451 | 14.20842075 | 11.59219671 | 9.732473305 | 12.79775918 | 11.4711143 | 11.80359346 | 10.81466729 | 8.92406698 | 9.866318689 | 12.46291268 | 8.556853883 | 10.74867954 | 6.139762282 | 3.907953702 | 4.756354048 | 6.853075107 |
| ENSMUSG00000027175 | Tcp11l1 | 6862 | 3.790901738 | 3.708984434 | 3.884263367 | 3.22685333 | 3.668463433 | 4.116155369 | 4.778153089 | 3.806995518 | 3.541248076 | 3.573064077 | 3.613407673 | 2.383268601 | 3.076954327 | 3.370614328 | 2.719951154 | 3.074631985 | 2.764721213 | 3.944109663 |
| ENSMUSG00000027176 | Cstf3 | 6020 | 12.913948 | 12.3512259 | 13.37102234 | 11.6097412 | 13.41617407 | 11.79552612 | 10.05983871 | 11.93988394 | 13.06748258 | 11.67802302 | 11.96754774 | 12.04394447 | 8.342174715 | 11.04782943 | 10.85476207 | 11.48718222 | 10.0781608 | 12.04352204 |
| ENSMUSG00000027177 | Hipk3 | 8519 | 12.74806019 | 17.38965144 | 19.47660631 | 16.61675839 | 16.56565327 | 19.5209501 | 10.52441913 | 12.94300572 | 12.46376777 | 14.18008003 | 13.54904328 | 15.47559514 | 10.64930108 | 12.39199561 | 11.14797351 | 12.7875077 | 15.5302708 | 13.72806208 |
| ENSMUSG00000027180 | Fbxo3 | 7315 | 20.02943838 | 19.73726364 | 20.11651119 | 19.63204535 | 20.04893663 | 20.26818576 | 17.3145648 | 18.91085188 | 16.94193109 | 17.5066726 | 20.13536528 | 17.38182903 | 14.6748123 | 13.55545134 | 17.2410065 | 17.46240141 | 20.82666168 | 16.01016927 |
| ENSMUSG00000027184 | Caprin1 | 11660 | 25.44477947 | 27.65884468 | 28.17778887 | 28.65344238 | 27.60361694 | 26.33179565 | 22.56887823 | 21.6281675 | 22.34527433 | 23.44595004 | 21.46185528 | 23.60437669 | 19.36045055 | 18.03592004 | 19.03540151 | 19.93344656 | 21.31612654 | 19.71081028 |
| ENSMUSG00000027185 | Nat10 | 7839 | 1.827305054 | 1.971830963 | 2.721358194 | 2.842116428 | 2.38234295 | 2.370490508 | 1.877056148 | 2.494515886 | 1.50265895 | 2.129270182 | 2.35858004 | 2.484968267 | 1.447421989 | 1.777423934 | 2.2758354 | 1.592389082 | 2.209910218 | 0.898222434 |
| ENSMUSG00000027186 | Elf5 | 5134 | 0.091070693 | 0.032443411 | 0.103644105 | 0.097618132 | 0.131251837 | 0.144778146 | 0 | 0.185977807 | 0.096267664 | 0.165312166 | 0.093056016 | 0.097845922 | 0.067262061 | 0.153788369 | 0.128403758 | 0.268496663 | 0.13437346 | 0.18214936 |

| ENSMUSG00000027187 | Cat | 2826 | 48.70196744 | 51.56072163 | 45.22394451 | 52.30008062 | 53.36069245 | 51.84976404 | 49.70677415 | 45.72006275 | 48.88166096 | 49.75350216 | 46.33799051 | 51.66800188 | 51.5663424 | 40.64268511 | 39.43744115 | 46.23055642 | 51.84764086 | 39.5341457 |
| --- | --- | --- | --- | --- | --- | --- | --- | --- | --- | --- | --- | --- | --- | --- | --- | --- | --- | --- | --- | --- |
| ENSMUSG00000027188 | Pamr1 | 6227 | 20.21845694 | 24.60350097 | 17.73514851 | 22.76954713 | 19.74127719 | 21.94739669 | 5.508542191 | 8.61736396 | 6.111504828 | 8.889501465 | 5.600725821 | 14.33262317 | 3.850218692 | 1.7676648 | 2.692955395 | 6.438134888 | 3.914489293 | 2.826869143 |
| ENSMUSG00000027189 | Trim44 | 5649 | 19.56336656 | 22.09065352 | 22.4869695 | 25.35739483 | 22.34909467 | 25.84215846 | 26.37415931 | 22.75047514 | 21.48639794 | 22.57791685 | 20.34812358 | 19.67232859 | 13.66692344 | 18.07118146 | 15.25092111 | 16.38314712 | 20.41491014 | 14.83074341 |
| ENSMUSG00000027193 | Api5 | 4103 | 30.14624963 | 36.20331351 | 31.9975046 | 33.55726931 | 33.51520983 | 38.2122469 | 29.49691267 | 31.35072174 | 26.15941021 | 34.05009353 | 32.97555019 | 35.8183377 | 25.57372363 | 27.30273916 | 35.04592495 | 27.07312992 | 31.67547473 | 28.97262482 |
| ENSMUSG00000027194 | Ttc17 | 8957 | 3.611302775 | 4.481638666 | 3.818252312 | 3.926886929 | 4.218327574 | 5.609743424 | 2.535456725 | 3.944174637 | 3.453284954 | 3.521694817 | 3.424307619 | 3.657898173 | 2.720773377 | 2.914098148 | 2.612759154 | 2.466638436 | 3.949444238 | 3.813850092 |
| ENSMUSG00000027195 | Hsd17b12 | 3354 | 77.52062068 | 68.86055326 | 72.63240873 | 69.97165754 | 69.80133474 | 73.97451909 | 91.1410908 | 85.00485508 | 86.58500973 | 89.11398567 | 91.67550172 | 86.35285425 | 109.4156627 | 94.77141744 | 109.1583261 | 110.2824292 | 105.7001331 | 101.948899 |
| ENSMUSG00000027196 | 4921507L20R | 1546 | 0.082480937 | 0.043095594 | 0.031289476 | 0 | 0.280198779 | 0.128208884 | 0.275429567 | 0.098816048 | 0.106562783 | 0.060997029 | 0.123609207 | 0.288826197 | 0 | 0.33045599 | 0.133252121 | 0.44581561 | 0.148743714 | 0.355815697 |
| ENSMUSG00000027198 | Ext2 | 6813 | 10.98658667 | 11.47101862 | 11.92120321 | 13.9565389 | 11.68504356 | 11.37538167 | 11.17365337 | 13.67818316 | 12.62859701 | 10.73399982 | 12.72736675 | 13.17357778 | 9.072786195 | 8.930244309 | 11.96193711 | 11.03812607 | 10.7502647 | 10.73053029 |
| ENSMUSG00000027199 | Gatm | 2834 | 0 | 0.199830346 | 0.221896928 | 0.112536101 | 0.118886191 | 0.262276289 | 0.500839932 | 0.431248016 | 1.206238817 | 0.332750199 | 0.775458042 | 0.768105448 | 1.288130515 | 0.44248072 | 1.177602691 | 1.540271434 | 0.662663567 | 0.970520584 |
| ENSMUSG00000027200 | Sema6d | 8279 | 0 | 0.012071347 | 0 | 0 | 0.017441209 | 0.053868173 | 0.102866073 | 0.013839498 | 0.074622266 | 0.182246951 | 0.005770619 | 0 | 0 | 0.084148002 | 0.069672881 | 0 | 0 | 0 |
| ENSMUSG00000027201 | Myef2 | 6362 | 6.487350821 | 6.016428082 | 7.208127441 | 5.406882816 | 6.839242219 | 5.841645738 | 4.499240923 | 5.871136365 | 6.668046406 | 5.254613111 | 6.127690368 | 5.465758848 | 5.427906648 | 4.256036301 | 5.070861091 | 4.923250056 | 6.74716324 | 4.90257207 |
| ENSMUSG00000027202 | Slc12a1 | 5411 | 0 | 0 | 0 | 0 | 0 | 0 | 0 | 0 | 0 | 0 | 0 | 0 | 0 | 0 | 0 | 0 | 0 | 0 |
| ENSMUSG00000027203 | Dut | 2401 | 11.45391457 | 8.53287378 | 9.44905695 | 10.74033782 | 9.62237541 | 8.750670153 | 11.1926704 | 8.701059607 | 11.1157277 | 9.524402782 | 9.312236805 | 7.903925564 | 9.985552602 | 8.762670382 | 8.408480794 | 9.648403781 | 9.226405502 | 10.40155537 |
| ENSMUSG00000027204 | Fbn1 | 11803 | 0 | 0 | 0 | 0.023160744 | 0.057091157 | 0.008396634 | 0 | 0 | 0 | 0.003994807 | 0 | 0.004728939 | 0.004179608 | 0.106243358 | 0.118686173 | 0.103812542 | 0.09092064 | 0 |
| ENSMUSG00000027206 | Cops2 | 4273 | 16.97023878 | 16.06004261 | 20.42261848 | 17.37994436 | 16.26550381 | 15.28446123 | 19.68685774 | 16.68738949 | 15.48952278 | 16.36426233 | 13.69630801 | 15.92307341 | 14.85845484 | 13.89083634 | 17.63577012 | 18.38809417 | 17.43653667 | 20.8295658 |
| ENSMUSG00000027207 | Galk2 | 6317 | 6.250959083 | 5.827251561 | 5.368029884 | 5.964695847 | 6.202214004 | 6.008769023 | 6.928008233 | 6.227350717 | 6.096152008 | 6.91175419 | 6.443606874 | 6.503138778 | 6.528650126 | 7.388999181 | 6.124467466 | 8.746770062 | 5.891219613 | 7.593468587 |
| ENSMUSG00000027208 | Fgf7 | 3244 | 0 | 0 | 0 | 0 | 0 | 0 | 0 | 0 | 0 | 0 | 0 | 0 | 0 | 0 | 0 | 0 | 0 | 0 |
| ENSMUSG00000027209 | 4930525F21R | 2655 | 0 | 0.012547229 | 0 | 0 | 0.054386354 | 0.01866393 | 0 | 0 | 0.07756406 | 0 | 0 | 0.147159939 | 0 | 0 | 0 | 0 | 0 | 0 |
| ENSMUSG00000027210 | Meis2 | 13113 | 0.019448719 | 0.002540448 | 0 | 0.010423483 | 0 | 0 | 0 | 0 | 0.037690703 | 0.007191444 | 0 | 0 | 0.007524124 | 0.007083671 | 0 | 0 | 0.02630494 | 0 |
| ENSMUSG00000027215 | Cd82 | 3438 | 45.43528851 | 48.47714071 | 45.48912846 | 49.76198996 | 48.94392238 | 49.65362629 | 55.76235457 | 57.53299943 | 59.07234125 | 66.36482055 | 64.4642328 | 62.45574577 | 80.34014102 | 69.07173322 | 75.69197993 | 91.5276939 | 73.70956228 | 71.55343958 |
| ENSMUSG00000027217 | Tspan18 | 10339 | 0 | 0 | 0 | 0 | 0 | 0 | 0 | 0 | 0 | 0 | 0 | 0.01079711 | 0 | 0 | 0 | 0 | 0 | 0 |
| ENSMUSG00000027219 | Slc28a2 | 3549 | 0 | 0 | 0 | 0 | 0 | 0 | 0.319950491 | 0.182944728 | 0.232102089 | 0.19928446 | 0.148076794 | 0.110090064 | 0 | 0.157038334 | 0.058046712 | 0 | 0 | 0 |
| ENSMUSG00000027220 | Syt13 | 3755 | 0.57730066 | 0.558911408 | 0.141706747 | 0.424670187 | 0.833175776 | 0.844573888 | 0.327597777 | 0.345816693 | 0 | 0.238578791 | 0.368967722 | 0.862132738 | 0 | 0.568955531 | 0.384035807 | 0.183550182 | 0.132687579 | 0 |
| ENSMUSG00000027221 | Chst1 | 2677 | 0 | 0 | 0 | 0 | 0 | 0 | 0 | 0 | 0 | 0 | 0 | 0 | 0 | 0 | 0 | 0 | 0 | 0 |
| ENSMUSG00000027222 | Pex16 | 1764 | 23.7103703 | 21.96309296 | 18.53770211 | 21.5149376 | 24.25696153 | 21.04024799 | 23.46859074 | 25.26674241 | 27.15414843 | 24.59106973 | 28.51872521 | 23.09831842 | 29.22440678 | 24.85443225 | 22.65618436 | 29.2389162 | 23.96479903 | 31.65206538 |
| ENSMUSG00000027223 | Mapk8ip1 | 5372 | 2.104686676 | 2.238636413 | 1.854979012 | 2.730948674 | 2.39226054 | 2.398308029 | 1.726225813 | 2.260830969 | 2.315399806 | 2.273274785 | 2.587958473 | 1.724758002 | 2.139675489 | 1.383293742 | 1.549276673 | 1.461201511 | 1.89776526 | 2.047993549 |
| ENSMUSG00000027224 | Duoxa1 | 3547 | 9.730533672 | 7.513480512 | 7.473553604 | 8.195079303 | 8.956038799 | 8.088816674 | 9.763992381 | 7.569568337 | 6.67669765 | 9.065909112 | 10.6136643 | 10.00810866 | 17.16255594 | 14.67826951 | 13.95068188 | 22.35687847 | 16.35918058 | 16.28406035 |
| ENSMUSG00000027225 | Duoxa2 | 1586 | 0 | 0.021004347 | 0 | 0 | 0.091043992 | 0 | 0 | 0 | 0 | 0 | 0 | 0 | 0.031104614 | 0.52710818 | 0 | 0.193143039 | 0.024165383 | 0 |
| ENSMUSG00000027227 | Sord | 2343 | 12.57195347 | 12.21330608 | 9.951362199 | 11.99793613 | 12.36680241 | 14.08541211 | 16.53823476 | 12.71450016 | 13.30695366 | 15.07293075 | 15.49678551 | 14.84129654 | 22.21304854 | 21.05148135 | 20.39855093 | 20.93481352 | 19.02410725 | 20.6372193 |
| ENSMUSG00000027229 | 4933406J08R | 2108 | 0 | 0 | 0 | 0 | 0 | 0 | 0 | 0 | 0 | 0 | 0 | 0 | 0 | 0 | 0.273634622 | 0 | 0 | 0 |
| ENSMUSG00000027230 | Creb3l1 | 2591 | 2.805243188 | 5.155720024 | 3.080522003 | 3.411363412 | 2.712180955 | 4.647361737 | 1.278227528 | 1.149751983 | 0.286127859 | 0.655123627 | 0.885062913 | 0.366216235 | 0.456953306 | 1.344386937 | 0.699679066 | 1.123151742 | 0.931901469 | 1.422466287 |
| ENSMUSG00000027233 | Patl2 | 2416 | 0 | 0.01378845 | 0 | 0 | 0 | 0.061530712 | 0 | 0.395202839 | 0.426184973 | 0.117096117 | 0 | 0 | 0.469633318 | 1.191859858 | 0.648037717 | 0 | 0.380724804 | 0.04553734 |
| ENSMUSG00000027236 | Eif3j | 5894 | 2.307712306 | 2.707308505 | 2.470382192 | 2.458162894 | 2.351882256 | 2.496973511 | 2.737296151 | 2.423474465 | 2.292021916 | 2.575929157 | 2.123691741 | 2.784154191 | 3.406530426 | 2.939199965 | 2.530533294 | 2.709057225 | 2.796115994 | 2.995915042 |
| ENSMUSG00000027238 | Frmd5 | 9079 | 1.231287729 | 1.15580589 | 1.454562594 | 0.988602901 | 0.92775489 | 1.091590122 | 0.385630382 | 0.652034626 | 0.848317926 | 0.347956506 | 0.826155797 | 0.95905313 | 0.114106208 | 0 | 0.027228696 | 0.278354454 | 0.067542764 | 0 |
| ENSMUSG00000027239 | Mdk | 1612 | 2.003966115 | 0.785291551 | 1.320369315 | 1.271865382 | 2.179671481 | 1.690695004 | 1.262062775 | 1.658479013 | 2.325045245 | 2.252235826 | 1.333668196 | 1.835130346 | 1.162911201 | 1.699876648 | 1.738030894 | 1.543976105 | 1.188780921 | 1.842736826 |
| ENSMUSG00000027242 | Wdr76 | 5807 | 0.541653698 | 0.831818437 | 0.841351225 | 0.886584813 | 0.795705988 | 0.588796042 | 0.611064391 | 0.848427744 | 0.8723853 | 0.738886516 | 0.353766698 | 0.692048856 | 0.637143757 | 0.791796909 | 0.879799024 | 0.573666755 | 0.574201452 | 0.568373756 |
| ENSMUSG00000027243 | Harbi1 | 3141 | 4.939315266 | 7.477106152 | 7.715739799 | 8.238991781 | 8.703894506 | 7.115021599 | 7.290460554 | 8.888457866 | 10.38513864 | 8.436388166 | 9.795311449 | 8.938324827 | 7.303196922 | 7.230550377 | 8.539386464 | 6.461006661 | 7.260154937 | 7.250483981 |
| ENSMUSG00000027244 | Atg13 | 5926 | 14.73981383 | 15.63895912 | 14.31778134 | 14.20034575 | 13.43405362 | 16.18031378 | 13.95588302 | 13.41825545 | 10.75186296 | 14.16271546 | 13.28604988 | 16.88786416 | 16.37460028 | 15.70603293 | 20.52429851 | 18.15670044 | 17.48807068 | 17.23791954 |
| ENSMUSG00000027245 | 2310003F16R | 4934 | 1.878015479 | 1.917483169 | 1.970628213 | 1.782181102 | 2.62413609 | 1.827847084 | 2.04248087 | 2.329937804 | 2.161999915 | 1.720131047 | 1.917195334 | 1.776055741 | 1.619734617 | 2.31561522 | 2.037531341 | 2.553224531 | 2.237124748 | 2.553118249 |
| ENSMUSG00000027246 | Ell3 | 2701 | 0.157368293 | 0.111001869 | 0.071637957 | 0.118077494 | 0.160380345 | 0.165114625 | 0.157650541 | 0.19796136 | 0.076243087 | 0.034913516 | 0.08843939 | 0.185983326 | 0.127850211 | 0.103170872 | 0.167796044 | 0.085058735 | 0.22703471 | 0.0407324 |
| ENSMUSG00000027247 | Arhgap1 | 4394 | 40.02877069 | 43.80562194 | 41.69106952 | 46.90900423 | 42.72064231 | 38.94073488 | 40.49681053 | 37.01029801 | 40.44602083 | 41.39904715 | 42.33857267 | 41.3472876 | 42.89878378 | 41.41282075 | 43.16130218 | 47.91990216 | 49.60438108 | 48.24877044 |

| ENSMUSG00000027248 | Pdia3 | 3448 | 254.8214889 | 247.5859128 | 265.0723557 | 257.787243 | 257.1037616 | 265.4117992 | 254.4840323 | 265.5634709 | 256.5795704 | 255.9101684 | 242.699006 | 242.1376685 | 261.8826606 | 243.225315 | 259.8159599 | 227.9446078 | 250.1324418 | 252.9334043 |
| --- | --- | --- | --- | --- | --- | --- | --- | --- | --- | --- | --- | --- | --- | --- | --- | --- | --- | --- | --- | --- |
| ENSMUSG00000027249 | F2 | 4243 | 0 | 0 | 0 | 0.032213795 | 0 | 0 | 0 | 0.090012733 | 0.029120798 | 0.022225172 | 0.135116545 | 0 | 0.023253319 | 0 | 0 | 0 | 0.072262639 | 0 |
| ENSMUSG00000027253 | Lrp4 | 9176 | 1.315548168 | 1.775283923 | 1.302120967 | 1.420058705 | 1.384789434 | 1.549872988 | 1.000289855 | 1.252824011 | 1.427344378 | 1.664867901 | 1.286008582 | 1.721429001 | 0.935457019 | 1.472888996 | 1.356023309 | 1.068264548 | 0.59728209 | 0.815304982 |
| ENSMUSG00000027254 | Mtap1a | 12260 | 0.027735841 | 0.067930045 | 0.035510748 | 0.011148706 | 0.019629659 | 0.036376395 | 0.057886638 | 0.006230408 | 0 | 0.01538359 | 0.105214019 | 0.009105328 | 0 | 0.121224372 | 0.026885191 | 0.003123214 | 0 | 0.224343828 |
| ENSMUSG00000027255 | Arfgap2 | 4222 | 13.65159135 | 13.14525859 | 13.19903055 | 15.8416895 | 14.75194439 | 13.43862623 | 12.79750813 | 14.75409956 | 8.711406579 | 12.83187069 | 13.05833777 | 12.83680918 | 11.40406235 | 11.9905389 | 15.58476664 | 12.56098798 | 14.56980259 | 13.902112 |
| ENSMUSG00000027257 | Pacsin3 | 4189 | 10.22802994 | 12.06389604 | 11.99811364 | 13.49755455 | 11.22580316 | 12.1131533 | 12.17547574 | 12.35395208 | 9.83206392 | 12.67407302 | 11.84964952 | 12.24506897 | 13.97875049 | 11.84108029 | 11.59623643 | 15.90490735 | 11.9946945 | 12.96108578 |
| ENSMUSG00000027259 | Adal | 5539 | 4.358718177 | 3.62057453 | 4.087165952 | 4.343063984 | 4.483854958 | 3.703706752 | 5.927965188 | 5.722999458 | 4.617589148 | 4.758484044 | 4.855985128 | 4.161738337 | 4.292829776 | 3.228195278 | 5.01351303 | 5.495763294 | 4.795111709 | 4.558436538 |
| ENSMUSG00000027261 | Hao1 | 2275 | 0 | 0.073215152 | 0.191367812 | 0.020026833 | 0 | 0 | 0 | 0 | 0.036207926 | 0 | 0 | 0 | 0 | 0 | 0.018110574 | 0.286127616 | 0 | 0 |
| ENSMUSG00000027263 | Tubgcp4 | 8376 | 2.86209638 | 3.245382245 | 3.199490905 | 3.611811538 | 3.758151633 | 3.472714256 | 3.394808982 | 3.497322427 | 2.512692163 | 4.238834713 | 2.994490592 | 3.871645172 | 3.386563089 | 3.404568966 | 3.876173119 | 3.972604803 | 3.935125995 | 2.686094156 |
| ENSMUSG00000027270 | 6330527O06R | 2927 | 0 | 0 | 0 | 0 | 0 | 0 | 0 | 0 | 0 | 0 | 0 | 0 | 0 | 0 | 0 | 0 | 0 | 0 |
| ENSMUSG00000027272 | Ubr1 | 10058 | 6.634830619 | 8.343127912 | 8.416551812 | 7.528579806 | 7.053733892 | 7.601895795 | 6.082252322 | 9.337355092 | 5.892562754 | 6.684917761 | 7.390916225 | 8.041051411 | 6.817594454 | 8.256316191 | 7.455441611 | 7.956588732 | 8.16977337 | 6.776325634 |
| ENSMUSG00000027273 | Snap25 | 6928 | 0 | 0 | 0 | 0 | 0 | 0 | 0 | 0 | 0.10106391 | 0 | 0 | 0 | 0 | 0.046926763 | 0.005947107 | 0 | 0 | 0 |
| ENSMUSG00000027274 | Mkks | 4136 | 14.12043324 | 9.769956646 | 10.98228844 | 9.385398882 | 9.891715454 | 9.572687169 | 13.18943878 | 11.56114309 | 12.89568131 | 12.14107809 | 11.16982226 | 10.64762035 | 10.50808006 | 9.848033037 | 8.337935755 | 10.78543463 | 8.358394537 | 8.179545608 |
| ENSMUSG00000027276 | Jag1 | 6390 | 6.379102318 | 5.333191046 | 6.252979041 | 8.114001323 | 7.713159599 | 7.227409655 | 6.982137125 | 8.678461392 | 7.032001361 | 9.496550093 | 9.121353575 | 9.119178676 | 8.677476529 | 8.263994892 | 9.839369998 | 10.14491367 | 10.12438015 | 8.221235825 |
| ENSMUSG00000027281 | 2210009G21R | 5892 | 1.413953581 | 1.356940701 | 1.896518247 | 1.144438996 | 1.405073128 | 1.320397008 | 1.614027234 | 1.931659185 | 2.551438943 | 1.664519056 | 1.791966365 | 1.136775205 | 1.18892265 | 1.245445656 | 1.971968561 | 2.047104775 | 1.444066177 | 1.297736903 |
| ENSMUSG00000027282 | Mtch2 | 6694 | 42.84171238 | 42.25571927 | 41.85531638 | 39.25164964 | 43.1274691 | 39.87758826 | 42.27324958 | 42.50573617 | 40.79273969 | 39.62095994 | 40.23116835 | 40.91536545 | 44.79963028 | 39.88058176 | 36.94229735 | 42.82667727 | 40.4332699 | 34.49779355 |
| ENSMUSG00000027284 | Cdan1 | 13193 | 0.631472334 | 0.638835916 | 0.597656746 | 0.524920696 | 0.598319979 | 0.631005779 | 0.54151554 | 0.419760354 | 0.421449225 | 0.407426679 | 0.677171018 | 0.575375586 | 0.448709927 | 0.707592023 | 0.371635348 | 0.5775662 | 0.505478334 | 0.750522187 |
| ENSMUSG00000027285 | Haus2 | 3211 | 9.544139494 | 10.13600051 | 10.89195342 | 10.69854482 | 12.96608968 | 11.63586456 | 7.529361237 | 11.07353679 | 13.01909483 | 8.63426145 | 11.63501013 | 12.9153028 | 9.187320626 | 8.374689068 | 10.32925957 | 10.50576927 | 14.09634277 | 12.66014633 |
| ENSMUSG00000027286 | Lrrc57 | 2932 | 10.24937225 | 11.62315511 | 9.618611254 | 11.45241803 | 10.37495763 | 10.63051481 | 13.89366185 | 12.27054675 | 8.147400799 | 12.02889701 | 8.961878296 | 9.537396643 | 10.76822205 | 11.08828826 | 9.401037139 | 10.85248798 | 10.71881428 | 9.418339555 |
| ENSMUSG00000027287 | Snap23 | 4395 | 20.41602425 | 22.09490026 | 20.29597042 | 19.5513378 | 20.79693357 | 19.78726904 | 18.64517859 | 21.70753611 | 20.0637839 | 18.60280306 | 20.66443597 | 18.64332029 | 16.46642153 | 13.97021241 | 21.58042814 | 18.23486719 | 20.77206808 | 17.57287505 |
| ENSMUSG00000027288 | Zfp106 | 11534 | 6.080591334 | 6.232809583 | 7.012358484 | 7.13792328 | 6.777028267 | 6.478717031 | 4.799361396 | 5.675548615 | 4.320763315 | 5.338895306 | 5.069927968 | 6.092588798 | 4.093171912 | 5.798464179 | 4.725997784 | 4.823673709 | 5.206979994 | 4.926686947 |
| ENSMUSG00000027291 | Vps39 | 8115 | 6.515888968 | 8.665868108 | 9.132254899 | 10.3305387 | 8.956155852 | 9.098407028 | 7.975815745 | 7.751433982 | 6.851730903 | 7.169928259 | 9.413697918 | 7.772236441 | 9.933253583 | 7.543229474 | 9.631466608 | 9.470024529 | 11.33494918 | 9.327483777 |
| ENSMUSG00000027293 | Ehd4 | 3998 | 32.78788466 | 33.37122095 | 31.96670015 | 33.89158225 | 32.40898912 | 34.89018135 | 32.76858628 | 36.14808672 | 31.75008544 | 37.88095519 | 32.00133537 | 31.77499951 | 31.47716883 | 25.04588605 | 29.83454335 | 33.13794437 | 32.17184903 | 29.40331693 |
| ENSMUSG00000027296 | Itpka | 1811 | 0 | 0.073579004 | 0 | 0.251579483 | 0 | 0 | 0.026125168 | 0 | 0 | 0 | 0 | 0.092461064 | 0.136200766 | 0 | 0 | 0 | 0.232793631 | 0 |
| ENSMUSG00000027297 | Ltk | 4058 | 0.031423245 | 0 | 0 | 0.011227463 | 0.023721993 | 0 | 0 | 0 | 0 | 0.011619197 | 0.082411215 | 0.068772379 | 0 | 0 | 0 | 0 | 0 | 0 |
| ENSMUSG00000027298 | Tyro3 | 5849 | 0.036335421 | 0.13669165 | 0.02481118 | 0.085684987 | 0.197498063 | 0.025416003 | 0.032356081 | 0.19589196 | 0.091541239 | 0 | 0.179697228 | 0.076342161 | 0 | 0.039702588 | 0.112707282 | 0 | 0 | 0.037619495 |
| ENSMUSG00000027300 | Ubox5 | 5578 | 4.122499145 | 4.759838059 | 4.596265882 | 3.642923229 | 4.53016492 | 4.45956835 | 5.318223322 | 5.279014388 | 4.467164223 | 5.004162124 | 5.464400055 | 4.042582948 | 4.634264011 | 4.229759124 | 4.904595395 | 4.839526393 | 4.30810114 | 6.63698975 |
| ENSMUSG00000027301 | Oxt | 534 | 0 | 0 | 0 | 0 | 0 | 0 | 0.088600522 | 0.071521353 | 0.308513227 | 0 | 0 | 0 | 0 | 0 | 0 | 0 | 0 | 0 |
| ENSMUSG00000027303 | Ptpra | 3604 | 55.40769062 | 58.05723879 | 62.66815017 | 63.29748862 | 64.45190445 | 63.70083648 | 49.62317601 | 61.65466071 | 57.64283727 | 63.83137658 | 69.51495068 | 63.12559398 | 49.87944133 | 40.49037807 | 52.18787532 | 47.36390893 | 56.88328583 | 48.6290272 |
| ENSMUSG00000027304 | Rtf1 | 5884 | 7.722303395 | 6.074904061 | 6.585179778 | 5.358300931 | 6.118742166 | 6.65306926 | 6.472927692 | 5.997583246 | 7.251738669 | 6.402687271 | 6.390022489 | 6.507400499 | 7.637895418 | 7.530193639 | 6.841250868 | 7.216907479 | 6.526673946 | 7.40435291 |
| ENSMUSG00000027305 | Ndufaf1 | 1831 | 23.88739822 | 16.19250456 | 19.62945552 | 20.45394781 | 22.50187147 | 20.35153231 | 19.19897348 | 14.95573595 | 20.22210687 | 19.05599149 | 20.71726763 | 17.77199961 | 14.33346805 | 16.36069709 | 16.69664362 | 19.28123431 | 22.3761941 | 17.21475595 |
| ENSMUSG00000027306 | Nusap1 | 4249 | 0.610217872 | 0.282246221 | 0.136616231 | 0.310960293 | 0.294523421 | 0.128284319 | 0.167025226 | 0.359542503 | 0.513740959 | 0.410585084 | 0.865773547 | 0.302132323 | 0.174153627 | 1.475629983 | 0.795134757 | 0.747969031 | 0.883967309 | 0.608479175 |
| ENSMUSG00000027309 | 4930402H24R | 10803 | 6.291379845 | 6.173508676 | 5.086765758 | 5.950813067 | 5.939077502 | 6.628131077 | 4.99272924 | 5.925249137 | 4.10988281 | 5.09348058 | 4.815970544 | 5.647183116 | 4.342743065 | 3.44364658 | 3.768132669 | 4.678663498 | 4.690119828 | 2.688587277 |
| ENSMUSG00000027312 | Atrn | 13645 | 1.946920851 | 2.875968442 | 2.112907592 | 2.146995347 | 2.243452067 | 2.418623713 | 1.945211644 | 2.082458585 | 1.566566631 | 2.36358234 | 1.887189639 | 2.347980236 | 1.955922847 | 1.490839888 | 2.095557331 | 1.318914289 | 1.474628499 | 1.459384143 |
| ENSMUSG00000027313 | Chac1 | 1634 | 31.21555155 | 25.97345608 | 23.80190845 | 20.35468933 | 20.82574665 | 27.56636158 | 5.993711468 | 5.329172429 | 4.940365416 | 4.963231922 | 4.181039818 | 7.173371569 | 3.683288805 | 4.775157088 | 3.404045315 | 2.69487139 | 2.462828136 | 3.871509959 |
| ENSMUSG00000027314 | Dll4 | 3841 | 5.53308722 | 5.706817078 | 6.372560885 | 5.242900702 | 4.135260299 | 4.489547328 | 5.345926231 | 6.015725971 | 7.430943875 | 6.592014488 | 4.452859968 | 7.469213895 | 5.021811938 | 5.054316201 | 6.993859526 | 7.357060209 | 3.48239459 | 4.94093773 |
| ENSMUSG00000027315 | Spint1 | 2756 | 327.4718442 | 333.6124385 | 321.1854978 | 341.3276061 | 341.3594267 | 335.7934878 | 284.5627598 | 299.4278805 | 306.8514979 | 304.460782 | 314.7149387 | 313.5884323 | 277.8589811 | 262.7730968 | 273.3265771 | 313.1188023 | 291.507923 | 284.1671453 |
| ENSMUSG00000027316 | Gfra4 | 2165 | 11.70119395 | 14.30992685 | 10.9482817 | 13.72092421 | 11.33823606 | 11.14650404 | 12.39089559 | 14.80065848 | 15.71365451 | 16.74775557 | 14.36558798 | 20.08332617 | 11.80320238 | 8.280562463 | 9.153786782 | 13.0170379 | 8.745122711 | 11.28131334 |
| ENSMUSG00000027317 | Ppp1r14d | 1130 | 4.100056803 | 2.594278488 | 3.082207244 | 3.910992301 | 3.066812835 | 4.253641732 | 6.406052988 | 4.900794999 | 6.05040851 | 7.510731492 | 6.088136302 | 5.63096065 | 12.70406011 | 7.726980911 | 7.948618747 | 5.929961323 | 5.833737228 | 6.133758803 |
| ENSMUSG00000027318 | Adam33 | 5926 | 0.466222822 | 0.489068815 | 0.824456052 | 0.461299808 | 0.917804147 | 0.560248591 | 1.381217255 | 2.79063622 | 2.175393087 | 1.519707108 | 1.249598138 | 1.299790995 | 0.058272599 | 0 | 0.076479432 | 0.08399897 | 0.077609781 | 0.25991478 |
| ENSMUSG00000027322 | Siglec1 | 7776 | 0.147587417 | 0.008568131 | 0.180405399 | 0.023436751 | 0.02475922 | 0.057352701 | 0.261631326 | 0.265225017 | 0.471399164 | 0 | 0.135165778 | 0.229694085 | 0 | 0 | 0 | 0.064014647 | 0 | 0 |

| ENSMUSG00000027323 | Rad51 | 4882 | 0.356966861 | 0.736950548 | 0.753049633 | 0.23331137 | 0.670415979 | 0.609005329 | 0.726843695 | 0.586732933 | 0.556802548 | 0.637432695 | 0.401223535 | 1.028965513 | 1.091324676 | 0.846686558 | 1.122451236 | 0.862754366 | 1.562255855 | 1.014096601 |
| --- | --- | --- | --- | --- | --- | --- | --- | --- | --- | --- | --- | --- | --- | --- | --- | --- | --- | --- | --- | --- |
| ENSMUSG00000027324 | Rpusd2 | 2703 | 1.682594834 | 1.700769294 | 1.145359209 | 1.297891385 | 1.24647971 | 2.4932193 | 3.535760689 | 1.836852502 | 1.340885456 | 2.163036331 | 1.184210957 | 1.135723804 | 1.295806928 | 1.907248872 | 2.19497745 | 1.189941185 | 1.502992035 | 1.6484416 |
| ENSMUSG00000027326 | Casc5 | 8594 | 0.054405043 | 0.062020748 | 0.045030049 | 0.042411957 | 0.005600643 | 0.023063874 | 0.269760445 | 0.088881551 | 0.177321513 | 0.043891741 | 0.055591062 | 0.019484174 | 0.034441646 | 0.102680668 | 0.378743649 | 0.040099542 | 0.249740822 | 0.108814849 |
| ENSMUSG00000027327 | 1700037H04R | 2194 | 34.8333211 | 35.74227575 | 35.49744023 | 34.97028198 | 32.24882762 | 35.61743886 | 36.72447229 | 35.52903068 | 33.433539 | 35.30930057 | 34.51381458 | 33.35202081 | 35.68357006 | 32.23990205 | 30.72276456 | 46.61541129 | 37.88957976 | 34.12369838 |
| ENSMUSG00000027329 | Spef1 | 4213 | 0.322849664 | 0.672109188 | 0.436314777 | 0.248731075 | 0.354163217 | 0.423427109 | 0.280754088 | 0.54392218 | 0.430146379 | 0.872953917 | 0.26081748 | 0.66242182 | 0.175641766 | 0.705535626 | 0.547658944 | 0.408990585 | 0.627703415 | 0.705077561 |
| ENSMUSG00000027330 | Cdc25b | 4089 | 1.496880741 | 1.433863874 | 1.691713094 | 1.448504711 | 1.659721506 | 1.926848775 | 2.776973085 | 1.615868334 | 3.273567528 | 1.948757361 | 2.032976957 | 3.412549681 | 1.60458425 | 1.05632187 | 2.841486613 | 2.106966658 | 2.0714384 | 1.910318698 |
| ENSMUSG00000027331 | D2Ertd750e | 1885 | 0.699024115 | 1.661226555 | 1.257455166 | 0.604257882 | 0.331944301 | 0.473182602 | 1.480874301 | 0.162089771 | 0.43699221 | 0.875477249 | 0.253448056 | 0 | 1.360880477 | 1.552242708 | 2.338762059 | 0.95472602 | 2.175551071 | 0.9338416 |
| ENSMUSG00000027332 | Ivd | 5629 | 9.204798291 | 7.746771813 | 7.708484052 | 8.377274984 | 8.448097457 | 9.709835697 | 10.66615554 | 10.95767097 | 11.78743594 | 12.75724304 | 11.02499042 | 12.06744739 | 14.27637116 | 14.46375647 | 13.40935342 | 12.91772312 | 12.04462946 | 10.68128507 |
| ENSMUSG00000027333 | Smox | 3724 | 6.003684906 | 7.460513904 | 6.806586978 | 7.805570968 | 6.100501603 | 5.109626663 | 10.46875602 | 8.25587646 | 7.896662797 | 8.407107133 | 3.989799164 | 6.819582835 | 6.636759011 | 8.82986409 | 5.97444688 | 6.529144925 | 4.744474456 | 6.455150279 |
| ENSMUSG00000027335 | Adra1d | 2486 | 0 | 0 | 0 | 0 | 0 | 0 | 0 | 0 | 0 | 0 | 0 | 0 | 0 | 0 | 0 | 0 | 0 | 0.044255114 |
| ENSMUSG00000027338 | Prnd | 3453 | 0 | 0.221893011 | 0.168109576 | 0.554174301 | 0.125452451 | 0.373116442 | 0.205528579 | 0.320758665 | 0.465182194 | 0.464269884 | 0.498088186 | 1.002192609 | 0.628614063 | 0.174854659 | 1.205142527 | 0.643166878 | 0.832456492 | 1.162949548 |
| ENSMUSG00000027339 | Rassf2 | 13121 | 0.106902737 | 0.114250457 | 0.283877893 | 0.309041456 | 0.084371179 | 0.32478737 | 0.472369556 | 0.349294131 | 0.483402441 | 0.661209466 | 0.538883763 | 0.476438854 | 0.447412404 | 0.743331937 | 0.386235148 | 0.145913449 | 0.578355826 | 0.913953606 |
| ENSMUSG00000027340 | Slc23a2 | 8331 | 3.173474912 | 4.046650936 | 3.605805107 | 3.089903979 | 4.055768865 | 3.782923845 | 2.697578021 | 2.984426115 | 3.223335527 | 2.795876535 | 2.569101116 | 3.122086036 | 3.41077714 | 2.620180179 | 2.403547733 | 3.18513875 | 4.071392721 | 3.671235547 |
| ENSMUSG00000027341 | 5730494N06R | 3225 | 11.6114915 | 10.87704732 | 13.91957711 | 11.65515088 | 12.98442592 | 11.41633521 | 16.28436391 | 13.06239375 | 14.31630517 | 16.38943825 | 15.08059154 | 16.97834574 | 16.41337899 | 16.47504991 | 15.96959531 | 16.2067222 | 13.99949698 | 12.72458717 |
| ENSMUSG00000027342 | Pcna | 2382 | 25.33893785 | 21.64918566 | 22.29812608 | 26.22342224 | 23.70224453 | 23.88183801 | 30.0918172 | 26.82447071 | 26.54126855 | 31.47339134 | 28.96181362 | 28.39990892 | 31.64532729 | 34.88181039 | 30.70225093 | 34.73803635 | 34.36816548 | 37.50410971 |
| ENSMUSG00000027344 | Fsip1 | 1963 | 0 | 0.118792797 | 0 | 0 | 0.196156592 | 0 | 0 | 0 | 0 | 0 | 0 | 0 | 0 | 0 | 0 | 0 | 0 | 0 |
| ENSMUSG00000027345 | 4921508D12R | 1071 | 0 | 0 | 0 | 0 | 0 | 0 | 0 | 0 | 0 | 0 | 0 | 0 | 0 | 0 | 0 | 0 | 0 | 0 |
| ENSMUSG00000027346 | Gpcpd1 | 7258 | 38.99138358 | 48.39503398 | 40.96220964 | 40.9660203 | 51.24876083 | 42.88243588 | 48.79894109 | 65.03443116 | 59.29428261 | 54.53058737 | 62.14430738 | 61.99091432 | 71.0548172 | 76.09714622 | 80.04727743 | 86.33587629 | 107.3379219 | 80.81594172 |
| ENSMUSG00000027347 | Rasgrp1 | 5715 | 0.699122755 | 0.781089733 | 0.634823233 | 1.052328583 | 0.429523728 | 1.274584748 | 1.308032068 | 1.276421498 | 0.36754371 | 0.899287254 | 1.596678402 | 1.123149816 | 0.42296832 | 0.74765635 | 1.002102584 | 0.368501034 | 0.395669557 | 0.644901164 |
| ENSMUSG00000027349 | Fam98b | 3382 | 4.926679185 | 3.388419755 | 4.033511402 | 3.192775727 | 4.483014771 | 3.63367177 | 3.931065281 | 3.986374349 | 4.822548859 | 4.600748692 | 3.531561094 | 4.109432284 | 4.419743032 | 4.133551243 | 4.032440863 | 4.14380908 | 3.9890173 | 5.009699843 |
| ENSMUSG00000027350 | Chgb | 2732 | 1.18242803 | 0.438969324 | 0.442656757 | 0.300182576 | 0.880891722 | 0.380895829 | 0.588810792 | 1.006534763 | 0.768855162 | 0.949227189 | 0.559589558 | 1.573135439 | 1.354280306 | 1.734003263 | 0.452432898 | 2.088323757 | 1.795668376 | 1.268511612 |
| ENSMUSG00000027351 | Spred1 | 6895 | 7.268107692 | 8.208645 | 6.307150658 | 8.649660194 | 7.776499342 | 8.451633747 | 5.681638601 | 7.01809628 | 7.783325604 | 6.735814751 | 8.481007858 | 8.677939129 | 6.374871399 | 9.032852964 | 8.724332357 | 8.846546424 | 9.544053921 | 8.879642608 |
| ENSMUSG00000027353 | Mcm8 | 3940 | 1.585853014 | 0.786319584 | 1.718856409 | 1.260445135 | 1.099460185 | 1.069031054 | 1.092754767 | 2.43306929 | 2.027965497 | 2.955894341 | 1.018552414 | 1.062480887 | 1.502494942 | 1.88605431 | 1.683616878 | 1.321706245 | 1.955224791 | 0.907510644 |
| ENSMUSG00000027355 | Tmco5 | 1295 | 0 | 0 | 0 | 0 | 0 | 0 | 0 | 0 | 0 | 0 | 0 | 0 | 0 | 0 | 0 | 0 | 0 | 0 |
| ENSMUSG00000027356 | Fermt1 | 8486 | 4.928717088 | 4.832450246 | 3.728056664 | 3.382448495 | 4.594256207 | 3.235000519 | 1.639161867 | 1.444704357 | 1.946240021 | 1.383516982 | 2.190014005 | 1.565416886 | 1.610292374 | 1.340891045 | 1.835280241 | 1.95378659 | 1.427187111 | 2.30771176 |
| ENSMUSG00000027357 | Crls1 | 2523 | 14.69065141 | 14.98618111 | 13.57449841 | 13.74235226 | 12.95345865 | 15.57483858 | 16.20220158 | 14.13860636 | 14.13694913 | 15.5673943 | 14.82671125 | 13.60548256 | 14.2736027 | 17.52468141 | 17.11424121 | 15.85956589 | 13.42863514 | 16.52671538 |
| ENSMUSG00000027358 | Bmp2 | 3552 | 0.765859028 | 1.284872328 | 1.184824646 | 2.077953036 | 2.683029529 | 1.743832122 | 0.919080756 | 0.817179781 | 0.16233424 | 0.690269304 | 1.829221383 | 1.194254042 | 0 | 0 | 0.359585651 | 0.237160294 | 0.474762687 | 0.433630346 |
| ENSMUSG00000027359 | Slc27a2 | 3782 | 16.50981057 | 17.85437244 | 15.86017389 | 19.43150833 | 19.89164376 | 16.14197984 | 11.9720343 | 16.3999105 | 16.99951113 | 17.86540396 | 16.69976074 | 17.03100863 | 5.843653871 | 9.21022357 | 8.508306491 | 3.310689746 | 8.755663809 | 10.26875445 |
| ENSMUSG00000027360 | Hdc | 4494 | 0.491826692 | 0.615258171 | 0.624313476 | 0.557600676 | 0.66403633 | 0.352845455 | 0.33689491 | 0.416428064 | 0.366591151 | 0.272790005 | 0.106308319 | 0.235980772 | 0.120750131 | 0.14468563 | 0.137521882 | 0.289693069 | 0.272906431 | 0.1101651 |
| ENSMUSG00000027361 | Gabpb1 | 9209 | 5.224873413 | 6.207506405 | 6.2719074 | 6.550420534 | 6.256261552 | 6.586224986 | 4.320769137 | 4.81915209 | 4.848103278 | 4.316217054 | 5.478375085 | 4.491194047 | 4.542671935 | 3.918672592 | 4.81408124 | 3.326363983 | 6.413380772 | 4.683151229 |
| ENSMUSG00000027363 | Usp8 | 4345 | 26.05092837 | 33.13655439 | 28.30046356 | 29.37088267 | 29.56596186 | 30.45012627 | 28.34405138 | 25.42937173 | 21.80183803 | 26.84714381 | 28.48904889 | 22.85294908 | 22.31006155 | 20.54442715 | 28.21998397 | 28.0327784 | 27.1327248 | 26.11824791 |
| ENSMUSG00000027364 | Usp50 | 3103 | 0.46573509 | 0.62267092 | 0.654749686 | 0.660730582 | 0.511876726 | 0.319385972 | 0.442174569 | 0.369246559 | 0.292008813 | 0.486246376 | 0.492684071 | 0.449691126 | 0.890295639 | 0.808243867 | 0.544396968 | 0.654012953 | 0.444649271 | 0.602742388 |
| ENSMUSG00000027365 | Trpm7 | 10385 | 7.076692282 | 7.409993803 | 9.101769698 | 8.147025453 | 8.620643045 | 7.157351985 | 7.986434873 | 8.160706879 | 6.817488743 | 8.299613438 | 7.213397679 | 7.390133467 | 9.93763803 | 6.475786089 | 8.093516997 | 8.47665926 | 8.920044258 | 7.707101616 |
| ENSMUSG00000027366 | 2010106G01R | 8672 | 10.63609686 | 12.18886224 | 12.4280703 | 12.96115041 | 11.26149367 | 12.35961286 | 7.332592306 | 8.178423808 | 8.586856607 | 8.666769026 | 6.770690033 | 9.847551171 | 7.719489362 | 8.386928142 | 9.877540901 | 8.11114458 | 9.453407412 | 7.878379907 |
| ENSMUSG00000027367 | Stard7 | 3763 | 28.98439588 | 32.6046743 | 33.80876557 | 35.17269035 | 32.14337608 | 36.04194308 | 34.29949193 | 32.10272889 | 30.74486388 | 37.9912124 | 32.36204336 | 33.99667782 | 32.66944934 | 31.9789079 | 37.07373591 | 32.11404649 | 33.79395512 | 29.80695419 |
| ENSMUSG00000027368 | Dusp2 | 1991 | 1.152827476 | 0.652537858 | 0.558810245 | 0.594970946 | 0.94281518 | 0.423102195 | 1.378269903 | 2.915743428 | 1.427357904 | 0.449956485 | 0.287945506 | 1.822208974 | 0.842433545 | 0.373232244 | 1.738287642 | 2.346285336 | 0.423494993 | 0.74597985 |
| ENSMUSG00000027371 | Fahd2a | 1403 | 41.74777802 | 38.67904824 | 35.68539125 | 37.66985847 | 37.32539768 | 35.67231712 | 41.61357505 | 38.08351459 | 46.67609413 | 47.75563031 | 41.71370216 | 45.5516277 | 45.99155224 | 39.19444011 | 41.55395694 | 43.50337006 | 44.36343989 | 49.2455011 |
| ENSMUSG00000027374 | Mrps5 | 6472 | 5.385390041 | 5.728870715 | 5.396429066 | 5.666971676 | 5.272795717 | 5.57391688 | 4.612839988 | 4.313758677 | 4.912853858 | 5.427576317 | 5.639689165 | 4.268966777 | 4.497192706 | 4.19805178 | 4.609073928 | 4.579253728 | 4.701959169 | 4.436766642 |
| ENSMUSG00000027375 | Mal | 2954 | 69.11048086 | 62.4870504 | 56.11919043 | 62.46520974 | 58.38073206 | 63.86163058 | 70.85690304 | 53.19009597 | 57.30419085 | 66.04929251 | 63.17162756 | 61.84314951 | 68.15286202 | 72.62194978 | 72.7094485 | 87.02882129 | 82.62080524 | 65.94016483 |
| ENSMUSG00000027376 | Prom2 | 6812 | 3.363225173 | 4.019847189 | 4.310442297 | 4.481194907 | 3.384496688 | 4.284580164 | 17.20397911 | 13.51759868 | 9.450165023 | 11.31699939 | 6.522417995 | 7.439903351 | 26.34608266 | 45.38730032 | 22.25803372 | 5.40745219 | 10.1216982 | 23.30538491 |
| ENSMUSG00000027377 | Mall | 2002 | 0 | 0 | 0 | 0 | 0 | 0 | 0 | 0 | 0 | 0 | 0 | 0 | 0 | 0 | 0 | 0 | 0 | 0 |

| ENSMUSG00000027378 | Nphp1 |  | 3120 | 9.522794245 | 7.388629102 | 9.659201734 | 10.93757122 | 9.117297079 | 9.27525526 | 10.5240382 | 8.972766334 | 8.250503959 | 8.689632812 | 8.559680061 | 8.569135378 | 7.510468175 | 7.978856039 | 7.223477908 | 7.523122544 | 8.930518541 | 7.704801166 |
| --- | --- | --- | --- | --- | --- | --- | --- | --- | --- | --- | --- | --- | --- | --- | --- | --- | --- | --- | --- | --- | --- |
| ENSMUSG00000027379 | Bub1 |  | 4329 | 0.019637411 | 0 | 0 | 0.010524612 | 0 | 0 | 0.02185848 | 0 | 0.161739609 | 0 | 0.066216159 | 0 | 0 | 0.429143797 | 0.009517569 | 0.061915974 | 0.28330827 | 0 |
| ENSMUSG00000027380 | Acoxl |  | 3158 | 0 | 0 | 0 | 0 | 0 | 0 | 0 | 0 | 0.026083924 | 0.014930558 | 0 | 0 | 0.031242506 | 0 | 0 | 0 | 0 | 0 |
| ENSMUSG00000027381 | Bcl2l11 |  | 6027 | 3.935272644 | 3.764075156 | 3.659918673 | 4.233314224 | 2.851019869 | 3.461373957 | 2.928095112 | 4.708305125 | 3.772184619 | 3.950739198 | 4.510361934 | 3.546938572 | 3.077617537 | 4.569660498 | 3.848759908 | 4.07238748 | 3.961719424 | 3.413540055 |
| ENSMUSG00000027384 | 2310003L22R |  | 3627 | 5.285314141 | 5.501633206 | 4.854691218 | 4.924160292 | 4.910066655 | 4.658804012 | 5.009117372 | 4.454200777 | 4.519501867 | 4.835969565 | 4.307254321 | 5.001403458 | 5.549330645 | 4.673860462 | 4.975539421 | 5.785291661 | 5.262336878 | 3.928138583 |
| ENSMUSG00000027386 | Fbln7 |  | 3219 | 0.079226796 | 0 | 0.090165015 | 0 | 0 | 0 | 0 | 0 | 0 | 0 | 0 | 0 | 0 | 0 | 0.012799489 | 0 | 0 | 0 |
| ENSMUSG00000027387 | Zc3h8 |  | 2986 | 2.718850844 | 1.985832274 | 1.490410178 | 1.296948683 | 1.998780487 | 1.54333698 | 1.647862896 | 2.09764032 | 2.579329688 | 1.973823814 | 2.255950819 | 1.981399944 | 3.304214151 | 2.379753975 | 2.180122515 | 2.423618486 | 3.414197916 | 3.021263732 |
| ENSMUSG00000027394 | Ttl |  | 6255 | 2.466727242 | 2.055759739 | 1.353376149 | 1.23826979 | 1.76983892 | 1.624030437 | 1.701894925 | 1.544792615 | 1.810758087 | 1.696068462 | 1.489387195 | 1.418815404 | 1.672001033 | 0.683110478 | 1.4359615 | 1.389603178 | 0.943605072 | 0.993769634 |
| ENSMUSG00000027395 | Polr1b |  | 6024 | 1.862776639 | 1.415687404 | 1.614057039 | 2.004262409 | 1.262424293 | 1.702758275 | 2.458311505 | 1.546969819 | 2.256233433 | 1.549774111 | 2.24440791 | 1.890171841 | 1.662413547 | 1.881201415 | 2.352811293 | 1.652649058 | 1.934129193 | 1.808068249 |
| ENSMUSG00000027397 | Slc20a1 |  | 4703 | 1.825652893 | 3.109581238 | 2.725703922 | 3.371304152 | 3.346616849 | 2.381228533 | 2.012021217 | 3.183376939 | 1.716469719 | 2.115415349 | 3.555440244 | 2.266806624 | 2.590895932 | 2.942874344 | 3.924792056 | 4.127862631 | 2.94190797 | 5.134807113 |
| ENSMUSG00000027398 | Il1b |  | 2017 | 0 | 0 | 0 | 0 | 0.310220628 | 0 | 0 | 4.998906413 | 0.510492263 | 0 | 0 | 0.083017842 | 0 | 0.828947519 | 6.475405661 | 0 | 0 | 0.136363676 |
| ENSMUSG00000027399 | Il1a |  | 1967 | 2.052868186 | 1.168576361 | 2.114958623 | 1.204460755 | 1.076667332 | 3.400924775 | 0.553224004 | 0.33008177 | 0.020938747 | 0.191766968 | 0.607205878 | 0.227008287 | 0.075239325 | 0.613902528 | 0.209463934 | 0.214131511 | 1.11062477 | 0.055931984 |
| ENSMUSG00000027400 | Pdyn |  | 3001 | 0 | 0 | 0 | 0 | 0 | 0 | 0 | 0 | 0 | 0 | 0 | 0 | 0 | 0 | 0 | 0 | 0 | 0 |
| ENSMUSG00000027401 | Tgm3 |  | 2284 | 7.741748348 | 6.271691994 | 7.878701091 | 9.335625547 | 8.071158829 | 8.287716387 | 17.35903018 | 15.7184143 | 15.99493848 | 18.93047062 | 19.18110198 | 17.30187789 | 10.36747824 | 7.706790333 | 10.39058502 | 6.47117971 | 10.10176477 | 9.53748085 |
| ENSMUSG00000027403 | Tgm6 |  | 3531 | 0 | 0 | 0 | 0 | 0 | 0 | 0 | 0 | 0 | 0.013353357 | 0 | 0 | 0 | 0 | 0 | 0 | 0 | 0 |
| ENSMUSG00000027404 | Snrpb |  | 2277 | 114.056489 | 110.1798008 | 105.3298039 | 103.6878927 | 109.2639058 | 106.0911621 | 106.8846818 | 121.7057847 | 110.1382057 | 112.1718751 | 118.314884 | 116.9015788 | 117.3176689 | 111.6738597 | 107.518509 | 113.5601545 | 99.08955198 | 114.1493321 |
| ENSMUSG00000027405 | Nop56 |  | 3319 | 19.42764448 | 19.53205548 | 19.87993233 | 20.13800905 | 18.3014425 | 19.33437481 | 20.42755918 | 22.08232004 | 21.96448716 | 23.14205954 | 21.30368742 | 20.41585248 | 22.1317339 | 24.4884462 | 22.08423258 | 22.24293193 | 23.70710682 | 21.99369829 |
| ENSMUSG00000027406 | Idh3b |  | 3597 | 44.07681584 | 47.93648609 | 47.09594198 | 48.85430859 | 51.65116079 | 47.77561302 | 57.30924162 | 54.91551438 | 53.33522113 | 52.70863992 | 58.73251776 | 47.59150324 | 51.56741978 | 46.00508295 | 48.36056963 | 50.3409181 | 47.52162474 | 46.70497969 |
| ENSMUSG00000027408 | Cpxm1 |  | 2784 | 0.198479629 | 0.179487577 | 0.156379947 | 0.098191906 | 0.138310125 | 0.231388483 | 0 | 0.164622424 | 0.073970035 | 0.050808947 | 0.171605454 | 0.220536023 | 0.354395957 | 0.717347614 | 0.118395276 | 0.288830013 | 0.165199556 | 0.2173492 |
| ENSMUSG00000027409 | 1700020A23R |  | 730 | 0 | 0.410706915 | 0 | 0 | 0.131868284 | 0 | 0.129623778 | 0.418546876 | 0.282099423 | 0.258360018 | 0 | 0 | 0 | 0.190866113 | 0 | 0 | 0 | 0.301419763 |
| ENSMUSG00000027411 | Vps16 |  | 3659 | 20.09673532 | 22.24197884 | 19.36792074 | 24.81638736 | 22.21776963 | 23.02258734 | 21.95597945 | 17.53572999 | 18.5952785 | 19.97365128 | 23.30645009 | 17.96962299 | 19.6437834 | 19.97895424 | 20.02087083 | 19.23425428 | 24.39517504 | 23.54311591 |
| ENSMUSG00000027412 | Lpin3 |  | 3673 | 3.656856963 | 4.797854898 | 4.912422222 | 5.358663532 | 5.228597208 | 5.517851357 | 4.830453198 | 6.072519195 | 4.608673558 | 6.713808278 | 5.150798682 | 5.136317439 | 3.559204486 | 5.298141197 | 5.294618672 | 4.764173042 | 5.478166587 | 5.541347533 |
| ENSMUSG00000027416 | Otor |  | 945 | 0 | 0 | 0 | 0 | 0 | 0 | 0 | 0 | 0 | 0 | 0 | 0 | 0 | 0 | 0 | 0 | 0 | 0 |
| ENSMUSG00000027419 | Pcsk2 |  | 8291 | 0.025633323 | 0 | 0 | 0 | 0.005805322 | 0 | 0.051358595 | 0.05988436 | 0.049676174 | 0.005686974 | 0 | 0 | 0 | 0.022406989 | 0 | 0.013855002 | 0 | 0 |
| ENSMUSG00000027420 | Bfsp1 |  | 2382 | 0 | 0.041955786 | 0.040615895 | 0 | 0.060619551 | 0.04160599 | 0.258213613 | 0.208438804 | 0.414977489 | 0.039589172 | 0.601699729 | 0.328051753 | 0.331364684 | 0 | 0.138376342 | 0 | 0.06435986 | 0 |
| ENSMUSG00000027422 | Rrbp1 |  | 8760 | 12.15473356 | 11.49979362 | 11.02761874 | 11.05739501 | 10.60990235 | 11.18896199 | 10.36990223 | 10.74706301 | 11.81526417 | 11.35707578 | 11.20202794 | 13.3486088 | 14.49547432 | 14.48991905 | 12.66618607 | 13.91749931 | 10.74536361 | 12.18238208 |
| ENSMUSG00000027423 | Snx5 |  | 4242 | 51.94409065 | 46.74171296 | 55.91122568 | 49.77130586 | 52.54571865 | 49.7045925 | 67.25493964 | 58.28798047 | 63.77016396 | 62.81202831 | 61.40242188 | 60.39459955 | 67.8226642 | 63.49110391 | 69.85421734 | 62.25608657 | 68.30429151 | 66.18728918 |
| ENSMUSG00000027424 | 8430406I07Ri | k | 3457 | 3.282870118 | 2.476544349 | 2.294839161 | 3.031252588 | 2.868144714 | 2.537122894 | 7.116746613 | 5.236678837 | 3.967344446 | 5.482957104 | 3.966275118 | 5.263496096 | 8.476470599 | 10.64035789 | 6.137923426 | 3.411485994 | 4.478977133 | 4.821451934 |
| ENSMUSG00000027425 | Csrp2bp |  | 4612 | 4.368485134 | 5.807364907 | 6.408548796 | 5.769221572 | 5.207682114 | 5.060567545 | 6.462920148 | 6.492377165 | 7.796146631 | 6.563476039 | 5.417672004 | 5.530736731 | 5.04871313 | 9.184086665 | 7.05750849 | 5.230503613 | 7.329530327 | 8.062913301 |
| ENSMUSG00000027427 | Polr3f |  | 7401 | 6.0016091 | 5.711939301 | 5.359585852 | 5.497366922 | 5.625471419 | 5.697794389 | 4.564417343 | 8.024481251 | 4.674594412 | 6.319888884 | 5.067334469 | 5.890019251 | 5.059171087 | 4.656331961 | 4.809910051 | 4.645989116 | 6.747623951 | 5.284620305 |
| ENSMUSG00000027428 | Rbbp9 |  | 2544 | 50.49160454 | 49.99553071 | 49.98978432 | 50.3607141 | 52.33211512 | 52.04595288 | 55.47709168 | 54.67638741 | 57.35999424 | 60.0133558 | 59.62480077 | 55.81566259 | 35.40883685 | 41.16800985 | 45.13708187 | 45.96679049 | 46.34107283 | 35.72132245 |
| ENSMUSG00000027429 | Sec23b |  | 6346 | 25.98803703 | 30.18423286 | 29.38543327 | 31.7764233 | 29.9516178 | 33.25639652 | 27.78669309 | 29.79681442 | 26.59665011 | 28.88779298 | 29.54127593 | 28.12771651 | 30.0531346 | 29.80150076 | 33.00149836 | 37.47000819 | 34.25571321 | 33.65905474 |
| ENSMUSG00000027430 | Dtd1 |  | 2342 | 11.77876142 | 10.22714387 | 11.21555379 | 10.64128576 | 10.93346857 | 12.50455403 | 13.97966431 | 15.00299326 | 18.90499763 | 15.36122399 | 17.11494029 | 19.47113422 | 20.32677206 | 19.47399394 | 20.72392521 | 25.47257316 | 18.47582801 | 17.85094838 |
| ENSMUSG00000027431 | 1700010M22R |  | 772 | 0 | 0 | 0 | 0 | 0 | 0 | 0 | 0 | 0 | 0 | 0 | 0 | 0 | 0 | 0 | 0 | 0 | 0 |
| ENSMUSG00000027433 | Xrn2 |  | 3952 | 44.3443423 | 49.84290069 | 45.12985992 | 47.57855009 | 48.76523845 | 49.96650893 | 40.18943906 | 36.24992445 | 43.1770584 | 34.09835777 | 38.38195677 | 38.16141708 | 37.09880014 | 33.91640592 | 40.05475145 | 45.16005089 | 45.22153908 | 39.54475511 |
| ENSMUSG00000027434 | Nkx2-2 |  | 2827 | 0 | 0 | 0 | 0 | 0 | 0 | 0 | 0 | 0 | 0 | 0 | 0 | 0 | 0 | 0 | 0 | 0 | 0 |
| ENSMUSG00000027435 | Cd93 |  | 6675 | 0.006367817 | 0 | 0 | 0 | 0 | 0 | 0.021264125 | 0 | 0 | 0 | 0 | 0 | 0 | 0 | 0 | 0 | 0 | 0 |
| ENSMUSG00000027438 | Napb |  | 7041 | 0.609717764 | 0.648184421 | 0.357253739 | 0.452957407 | 1.148439593 | 0.541906048 | 1.014659071 | 0.47191294 | 0.432864958 | 0.395098919 | 1.438473398 | 0.737232868 | 0.182165864 | 0.408966541 | 0.596869578 | 0.72872339 | 0.783835642 | 0.804706433 |
| ENSMUSG00000027439 | Gzf1 |  | 4561 | 7.520648335 | 6.339748341 | 6.045365556 | 5.484107288 | 6.289547579 | 5.388764713 | 7.064225904 | 6.003936094 | 5.147185701 | 7.401864401 | 6.253376499 | 5.555867313 | 7.138580443 | 6.802159697 | 5.962075617 | 5.960607598 | 6.646810097 | 5.354975528 |
| ENSMUSG00000027442 | Cst8 |  | 945 | 24.73846224 | 56.82580474 | 10.28897312 | 28.10803053 | 11.81651459 | 38.27883123 | 0.350464288 | 0 | 0.043583615 | 0.049894924 | 0.151666535 | 0 | 0.104406174 | 0.19658873 | 0 | 0.040519161 | 0.081113856 | 0 |
| ENSMUSG00000027443 | Cst12 |  | 874 | 19.06410641 | 107.0281544 | 25.23836366 | 34.24897726 | 18.00816824 | 94.90992678 | 0.108267 | 0 | 0.04712416 | 0.107896346 | 0 | 0 | 0 | 0.159419064 | 0 | 0.043810764 | 0.131554795 | 0.125878963 |

| ENSMUSG00000027444 | 8030411F24R |  | 599 | 0 | 0 | 0 | 0.152123687 | 0 | 0.082725766 | 0 | 0 | 0 | 0 | 0 | 0 | 0 | 0 | 0 | 0 | 0 | 0 |
| --- | --- | --- | --- | --- | --- | --- | --- | --- | --- | --- | --- | --- | --- | --- | --- | --- | --- | --- | --- | --- | --- |
| ENSMUSG00000027445 | Cst9 |  | 684 | 0 | 0 | 0 | 0 | 0 | 0 | 0 | 0 | 0 | 0 | 0 | 0 | 0 | 0 | 0 | 0 | 0 | 0 |
| ENSMUSG00000027446 | 9230104L09R |  | 994 | 66.58003931 | 218.5446511 | 66.96376033 | 106.1104805 | 49.63301988 | 155.6873108 | 0.237991343 | 0.15369176 | 0.207175633 | 0.047435315 | 0.192253354 | 0.449220624 | 0.049629695 | 0.186897736 | 0.16580103 | 0.192608689 | 0.501249356 | 0.332046922 |
| ENSMUSG00000027447 | Cst3 |  | 1262 | 255.0984178 | 234.9060584 | 235.236415 | 227.4441991 | 276.701352 | 269.2024892 | 336.3624052 | 333.4118045 | 337.9448262 | 358.5994056 | 397.872277 | 365.720851 | 514.0761047 | 508.640041 | 427.1315022 | 524.7512322 | 488.037711 | 553.9268845 |
| ENSMUSG00000027452 | Acss1 |  | 4037 | 65.6898175 | 80.4724658 | 77.31137425 | 77.83862343 | 78.95209277 | 80.36208738 | 113.3183781 | 128.777553 | 126.9671015 | 125.4743634 | 149.0053263 | 121.1989344 | 94.31353419 | 93.33685434 | 110.7247163 | 92.00368883 | 139.1974647 | 111.4898467 |
| ENSMUSG00000027454 | Gins1 |  | 1507 | 1.720514756 | 1.237904497 | 1.316068178 | 1.269783585 | 1.245617135 | 1.282386602 | 1.663949558 | 1.343196634 | 0.765243823 | 1.282799491 | 1.775313653 | 0.962977589 | 1.080260962 | 0.523921357 | 1.284985486 | 1.600735412 | 1.068151606 | 0.87605744 |
| ENSMUSG00000027455 | Nsfl1c |  | 4150 | 26.88580411 | 29.07453081 | 26.70452001 | 25.83256321 | 26.66392591 | 26.41220402 | 25.7426576 | 27.70099548 | 25.23790592 | 24.81376769 | 24.17528019 | 25.67520479 | 25.93789 | 25.09099854 | 26.9051124 | 25.29948084 | 26.28352795 | 26.88155866 |
| ENSMUSG00000027456 | Sdcbp2 |  | 1390 | 20.27405159 | 23.99007706 | 17.6789593 | 19.20774963 | 20.46472438 | 20.21323737 | 5.412025863 | 5.055684923 | 7.200232612 | 6.207610573 | 6.427278587 | 6.384669317 | 6.601249362 | 5.880690202 | 7.499276 | 9.531331051 | 7.858269509 | 9.102226292 |
| ENSMUSG00000027457 | Snph |  | 11759 | 1.106096085 | 0.68557874 | 1.180644886 | 1.108126429 | 0.728589371 | 0.703742368 | 1.166823445 | 0.636594172 | 0.6794952 | 0.669629003 | 0.637866186 | 1.006286289 | 0.499234472 | 0.454211247 | 0.6096667 | 0.416803958 | 0.541046457 | 0.626857752 |
| ENSMUSG00000027459 | Fam110a |  | 3725 | 11.71887672 | 13.62027863 | 12.81736227 | 12.98948432 | 12.99884972 | 13.84815991 | 7.595431409 | 9.453260407 | 6.954716353 | 11.84780087 | 9.657595633 | 9.260155565 | 10.85964353 | 8.752684388 | 8.395162663 | 10.89611915 | 10.02142636 | 13.11358034 |
| ENSMUSG00000027460 | Angpt4 |  | 2785 | 0.030524363 | 0 | 0 | 0.032718883 | 0 | 0 | 0 | 0.013713609 | 0.014788695 | 0 | 0 | 0 | 0 | 0 | 0 | 0 | 0 | 0.039503847 |
| ENSMUSG00000027463 | Slc52a3 |  | 2877 | 1.462639008 | 1.065271557 | 0.60529965 | 1.631139231 | 1.154363133 | 1.412347638 | 4.999323738 | 3.438245472 | 3.908209525 | 2.835269956 | 1.610765023 | 2.134071215 | 9.808083657 | 15.98180275 | 6.029146691 | 3.513632379 | 3.783339701 | 10.17200027 |
| ENSMUSG00000027465 | Tbc1d20 |  | 4352 | 10.2844555 | 13.57163636 | 11.84884728 | 12.28012523 | 13.37120765 | 14.11888549 | 10.89322249 | 10.52221748 | 10.94016825 | 11.54932208 | 15.29193869 | 11.85056807 | 11.06340792 | 11.23750552 | 12.13703921 | 11.44670961 | 12.02100075 | 10.93356556 |
| ENSMUSG00000027466 | Rbck1 |  | 3819 | 25.57656311 | 29.94584076 | 30.36170523 | 34.81202602 | 30.60076215 | 31.41324116 | 30.93473665 | 29.44185197 | 25.16055022 | 33.00178838 | 27.99695132 | 26.68745742 | 26.97173168 | 29.7709154 | 26.61540675 | 26.66004846 | 28.46121446 | 32.20747909 |
| ENSMUSG00000027468 | Defb22 |  | 626 | 9.981247406 | 2.341481383 | 0.23182203 | 0.509468547 | 0.538216399 | 0.237473164 | 399.9659695 | 282.416343 | 285.0817457 | 161.035469 | 37.54836992 | 272.5694577 | 15.91860589 | 113.7360798 | 20.27169204 | 0.978673672 | 4.346912269 | 22.23211502 |
| ENSMUSG00000027469 | Tpx2 |  | 4977 | 0.495338599 | 0.307894944 | 0.767833815 | 0.201395012 | 0.241771768 | 0.647162484 | 0.266175409 | 0.368341434 | 0.802710876 | 0.653686664 | 0.25917699 | 0.067288321 | 0.525334864 | 0.363937996 | 0.97685023 | 0.084628628 | 0.600653237 | 0.541580516 |
| ENSMUSG00000027470 | Mylk2 |  | 2941 | 0 | 0.033981191 | 0.11513591 | 0.247866953 | 0 | 0 | 0 | 0 | 0.140042556 | 0.224450815 | 0.162444606 | 0.037956928 | 0 | 0.126335498 | 0 | 0 | 0 | 0 |
| ENSMUSG00000027472 | Pdrg1 |  | 2668 | 57.99056999 | 46.9352209 | 53.97601194 | 45.83427399 | 49.4849575 | 46.71106632 | 60.93192084 | 54.46854991 | 65.51558204 | 55.54522501 | 57.28339287 | 49.30941406 | 66.0840601 | 57.96807009 | 66.14177807 | 63.03311383 | 59.58676145 | 59.71003488 |
| ENSMUSG00000027474 | BC020535 |  | 2256 | 0 | 0 | 0 | 0 | 0 | 0 | 0 | 0 | 0 | 0 | 0 | 0 | 0 | 0 | 0 | 0 | 0 | 0 |
| ENSMUSG00000027475 | Kif3b |  | 5676 | 5.474151458 | 4.595489107 | 4.636222782 | 5.233580146 | 5.503456388 | 4.539714845 | 6.026615022 | 4.676483381 | 5.253530199 | 4.186743207 | 3.728736189 | 4.611970711 | 6.127378731 | 4.655864293 | 5.023163615 | 4.506364648 | 4.463298494 | 3.566481902 |
| ENSMUSG00000027478 | Dnmt3b |  | 5369 | 0.427505961 | 0.521192608 | 0.234254384 | 0.161232975 | 0.510992671 | 0.230735396 | 0.361300025 | 0.754031413 | 0.905198149 | 0.403973245 | 0.685168896 | 0.602963015 | 0.441037815 | 0.337366252 | 0.291610938 | 0.613334371 | 0.207014828 | 0.225405168 |
| ENSMUSG00000027479 | Mapre1 |  | 7802 | 31.97966974 | 34.66641735 | 35.48342915 | 34.39561025 | 34.52271787 | 34.01113667 | 29.453689 | 30.01251207 | 28.3427843 | 29.27428949 | 27.25536275 | 31.57784346 | 25.29825699 | 25.78176653 | 27.57156156 | 26.12909431 | 29.64122988 | 27.44814823 |
| ENSMUSG00000027480 | Sun5 |  | 1362 | 0 | 0 | 0 | 0 | 0 | 0 | 0 | 0 | 0 | 0 | 0 | 0 | 0 | 0 | 0 | 0 | 0 | 0 |
| ENSMUSG00000027481 | Bpifb2 |  | 2120 | 0 | 0 | 0 | 0 | 0.022703738 | 0 | 0 | 0 | 0 | 0 | 0 | 0 | 0 | 0 | 0 | 0 | 0 | 0 |
| ENSMUSG00000027482 | Bpifa3 |  | 1179 | 0 | 0 | 0 | 0 | 0 | 0 | 0 | 0 | 0 | 0 | 0 | 0 | 0 | 0 | 0 | 0 | 0 | 0 |
| ENSMUSG00000027483 | Bpifa1 |  | 1550 | 0 | 0 | 0 | 0 | 0 | 0 | 0.061048618 | 0 | 0 | 0.030419809 | 0 | 0.036010105 | 0 | 0 | 0.026581649 | 0 | 0 | 0 |
| ENSMUSG00000027484 | Bpifa5 |  | 1080 | 0 | 0 | 0 | 0 | 0 | 0 | 0 | 0 | 0 | 0 | 0 | 0 | 0 | 0 | 0 | 0 | 0 | 0 |
| ENSMUSG00000027485 | Bpifb1 |  | 2702 | 0.015731005 | 0.049315906 | 0 | 0 | 0.213761319 | 0.036678559 | 0 | 0.042404592 | 0.015242974 | 0.261754459 | 0 | 0.020657166 | 0.273863345 | 0.24064294 | 0.01524854 | 0.085027256 | 0.099290925 | 0.040717325 |
| ENSMUSG00000027487 | Cdk5rap1 |  | 3649 | 10.05260809 | 4.774635154 | 8.020275655 | 11.03753445 | 8.283597715 | 6.423251023 | 9.20581037 | 4.375013485 | 10.44053907 | 12.83109025 | 12.2023188 | 7.479818856 | 10.91007324 | 4.582042055 | 8.524849191 | 13.90382428 | 10.06209713 | 8.200864362 |
| ENSMUSG00000027488 | Snta1 |  | 3085 | 3.678729984 | 3.779420738 | 3.747576582 | 5.198537312 | 3.947285798 | 5.509428725 | 4.754272437 | 5.509114772 | 5.313527803 | 8.436689854 | 6.209970292 | 3.962278803 | 9.754447176 | 8.551131811 | 10.75113533 | 10.92244231 | 11.71529918 | 8.113174573 |
| ENSMUSG00000027489 | Necab3 |  | 2656 | 0.096020729 | 0.100340043 | 0 | 0 | 0.036243918 | 0.093284514 | 0 | 0 | 0 | 0 | 0.179875597 | 0 | 0.037147528 | 0.104918872 | 0 | 0 | 0.158730898 | 0 |
| ENSMUSG00000027490 | E2f1 |  | 4449 | 0.047769359 | 0.067389537 | 0.097856096 | 0 | 0.032455781 | 0.044551795 | 0.106344524 | 0.06009144 | 0 | 0.042392181 | 0.246982253 | 0 | 0.487885898 | 0 | 0 | 0.172131299 | 0.284281366 | 0 |
| ENSMUSG00000027495 | 2010011I20Ri | k | 3614 | 18.95914326 | 19.75360606 | 16.98561428 | 19.46492874 | 17.80641448 | 18.66111522 | 15.46106082 | 17.26795394 | 16.53614675 | 18.47409956 | 14.89827842 | 18.96558762 | 19.04206547 | 23.05497863 | 17.97865347 | 20.13064584 | 21.42200325 | 21.73575108 |
| ENSMUSG00000027496 | Aurka |  | 3307 | 0.604095335 | 0.735361741 | 1.228719852 | 0.495977501 | 0.465745859 | 1.228704008 | 1.101625726 | 0.923916599 | 1.917968983 | 0.883986575 | 0.057786463 | 0.624487304 | 0.656366604 | 1.123534016 | 1.98096383 | 2.084157645 | 1.228481243 | 0.848341229 |
| ENSMUSG00000027498 | Cstf1 |  | 3620 | 9.264249692 | 9.285279079 | 10.36957446 | 9.829606527 | 11.088957 | 9.924235316 | 10.56039905 | 7.786185905 | 8.510362925 | 11.46204941 | 9.739756729 | 8.804072745 | 7.140863164 | 10.90537964 | 10.09551936 | 10.01690752 | 8.861632737 | 9.725366934 |
| ENSMUSG00000027499 | Pkia |  | 3680 | 0.866273968 | 1.99153172 | 0.946438637 | 1.03998036 | 1.804947138 | 1.225352923 | 0.964252967 | 0.581188732 | 1.611646269 | 1.678462534 | 1.960331177 | 1.98691625 | 0.978595098 | 0.89606799 | 0.358274399 | 0.749164058 | 1.135208251 | 1.27059078 |
| ENSMUSG00000027500 | Stmn2 |  | 1293 | 0 | 0 | 0 | 0 | 0 | 0 | 0 | 0 | 0 | 0 | 0.036948924 | 0.043167566 | 0 | 0 | 0 | 0 | 0 | 0 |
| ENSMUSG00000027502 | 2410001C21R |  | 2771 | 57.16953845 | 57.93390012 | 55.26907149 | 62.28265462 | 55.49675067 | 53.415379 | 58.63289045 | 56.66148188 | 47.96423182 | 54.11015386 | 53.24037235 | 52.7136734 | 58.85648449 | 50.04765245 | 55.13365906 | 54.11260144 | 50.58075344 | 61.61972835 |
| ENSMUSG00000027505 | 1700029J11R |  | 696 | 1.160342449 | 0.095726708 | 0.625519789 | 0.392767624 | 0.207465188 | 0.142392913 | 0.747757857 | 0.054874141 | 1.065168511 | 0.067745263 | 0.41185309 | 0 | 1.063187872 | 0.133460021 | 0.118395276 | 0.825228608 | 0.550665185 | 0.079036073 |
| ENSMUSG00000027506 | Tpd52 |  | 6016 | 63.9838554 | 65.76195674 | 62.98368738 | 67.12226329 | 65.39733113 | 67.99498266 | 73.77663581 | 64.94485981 | 66.14762555 | 67.78697348 | 58.59103119 | 59.62886693 | 53.54677856 | 59.3289248 | 59.2820259 | 50.6637691 | 55.39347599 | 50.94011528 |
| ENSMUSG00000027508 | Pag1 |  | 11801 | 2.917480941 | 3.079770154 | 3.344869144 | 3.451535771 | 3.528015759 | 3.300436284 | 1.266910138 | 1.297784371 | 2.027740501 | 1.178667694 | 1.319772601 | 1.593922404 | 0.539260853 | 0.488015154 | 0.942667578 | 0.68462996 | 1.159434624 | 1.156025631 |

| ENSMUSG00000027509 | Rae1 | 2595 | 51.92347129 | 44.8151498 | 45.57737253 | 44.52516703 | 49.3931841 | 47.62409154 | 48.04196878 | 47.36152252 | 48.85244529 | 45.93332479 | 51.97252708 | 50.20183294 | 43.78088843 | 41.66544718 | 48.56861637 | 46.02250655 | 41.22107694 | 41.69669091 |
| --- | --- | --- | --- | --- | --- | --- | --- | --- | --- | --- | --- | --- | --- | --- | --- | --- | --- | --- | --- | --- |
| ENSMUSG00000027510 | Rbm38 | 2530 | 2.604072049 | 3.002110625 | 3.823994494 | 3.403572087 | 2.834646888 | 3.016253348 | 1.402549771 | 1.35862301 | 1.644204779 | 1.584114536 | 1.378486944 | 1.875229771 | 1.130929329 | 1.285014276 | 1.677375596 | 1.64967439 | 1.423980992 | 1.826389314 |
| ENSMUSG00000027513 | Pck1 | 3176 | 0.040149725 | 0 | 0 | 0 | 0 | 0.031204492 | 0 | 0 | 0 | 0 | 0 | 0.035148402 | 0 | 0 | 0 | 0 | 0 | 0 |
| ENSMUSG00000027514 | Zbp1 | 4132 | 0.072007801 | 0.032248688 | 0 | 0.01102639 | 0 | 0.071954599 | 0 | 0.212590817 | 0.229256985 | 0 | 0.04624875 | 0 | 0.04775597 | 0 | 0 | 0.083401613 | 0 | 0 |
| ENSMUSG00000027517 | Ankrd60 | 1281 | 0 | 0 | 0 | 0.071133559 | 0.112721133 | 0 | 0 | 0 | 0 | 0.036807731 | 0 | 0 | 0 | 0.217536709 | 0 | 0 | 0 | 0 |
| ENSMUSG00000027518 | 1700021F07R | 642 | 0 | 0 | 0 | 0 | 0 | 0 | 0 | 0 | 0 | 0 | 0 | 0 | 0 | 0 | 0 | 0 | 0 | 0 |
| ENSMUSG00000027519 | Rab22a | 7484 | 17.24288007 | 18.8553474 | 17.08974001 | 19.54790398 | 17.53818223 | 17.55930645 | 13.92070003 | 14.07464535 | 12.80061941 | 16.92233952 | 16.4569539 | 16.91474115 | 17.21080118 | 15.35931538 | 14.78169128 | 17.48249673 | 15.45035245 | 16.80262132 |
| ENSMUSG00000027520 | Zdbf2 | 12855 | 0 | 0 | 0 | 0 | 0 | 0 | 0.018402442 | 0.041594215 | 0.009611789 | 0 | 0.011149349 | 0.004341942 | 0.092101596 | 0 | 0.019230598 | 0 | 0 | 0.042791993 |
| ENSMUSG00000027522 | Stx16 | 4020 | 9.590098173 | 10.97999623 | 9.807071453 | 11.54893139 | 9.626384734 | 9.836587412 | 7.96783175 | 11.76173985 | 13.75957479 | 11.79940485 | 8.176410804 | 10.98263411 | 9.301888879 | 8.549409121 | 7.14365285 | 10.5537296 | 7.693861095 | 9.154500596 |
| ENSMUSG00000027523 | Gnas | 10102 | 4.468471287 | 5.048707291 | 4.917800007 | 4.767177179 | 4.50254087 | 5.145596666 | 6.332086905 | 6.555694493 | 5.393957667 | 6.721145584 | 6.493270441 | 6.077730038 | 5.454736844 | 6.574445156 | 6.109674394 | 5.242121366 | 5.618812682 | 6.109702806 |
| ENSMUSG00000027524 | Edn3 | 4326 | 147.6283564 | 125.0119105 | 146.7308057 | 141.6963224 | 143.2831514 | 146.6536403 | 291.4552891 | 249.1158157 | 278.718273 | 286.8170958 | 268.5822908 | 256.1249926 | 224.5366276 | 214.2161063 | 243.3139501 | 229.9914801 | 257.7501054 | 244.031384 |
| ENSMUSG00000027525 | Phactr3 | 6514 | 0 | 0.010228092 | 0.051982609 | 0.048960287 | 0 | 0 | 0.00726323 | 0.052768134 | 0.36039782 | 0 | 0.11734715 | 0 | 0 | 0 | 0.246678029 | 0 | 0.035302085 | 0 |
| ENSMUSG00000027528 | Fabp9 | 579 | 0 | 0 | 0 | 0.393445979 | 0 | 0 | 0.081714471 | 0 | 0 | 0.162869441 | 0 | 0 | 0 | 0 | 0 | 0 | 0 | 0 |
| ENSMUSG00000027530 | Fabp12 | 2945 | 0.14432997 | 0.033935037 | 0.065702588 | 0.108294503 | 0 | 0.033652111 | 0.048196277 | 0 | 0 | 0 | 0 | 0 | 0.067004302 | 0 | 0 | 0 | 0.013014023 | 0 |
| ENSMUSG00000027531 | Impa1 | 4793 | 38.42581426 | 39.54038289 | 40.80412752 | 39.0305963 | 39.60615626 | 41.70576408 | 29.92948936 | 31.97707299 | 28.6406545 | 30.81076624 | 33.00289746 | 25.22360216 | 29.96144611 | 25.16488524 | 32.99219101 | 31.56398703 | 29.08256663 | 27.4184761 |
| ENSMUSG00000027533 | Fabp5 | 3349 | 0.266529918 | 0.139259635 | 0.245550916 | 0.163252473 | 0.143720286 | 0.295925551 | 0.339057717 | 0.467568975 | 0.405839062 | 0.689872935 | 0.17118528 | 0.149997302 | 2.047517618 | 1.830582125 | 1.340988233 | 1.852218095 | 1.201630688 | 1.62612767 |
| ENSMUSG00000027534 | Snx16 | 2930 | 4.381079577 | 4.968510162 | 3.665161686 | 4.011859877 | 3.811128427 | 5.158219707 | 5.296436412 | 5.839657433 | 5.735187178 | 4.940363788 | 7.321145514 | 6.515002254 | 5.977075301 | 7.434224227 | 6.299760082 | 5.867741544 | 5.59851709 | 3.435722365 |
| ENSMUSG00000027536 | Chmp4c | 1851 | 12.07872641 | 13.28196431 | 12.98846277 | 9.230357442 | 10.2972673 | 11.85946028 | 2.990590726 | 5.818615601 | 6.920046679 | 3.591706731 | 3.81993185 | 4.161297373 | 9.381326246 | 5.745918969 | 5.54251076 | 6.102500909 | 5.031491166 | 3.952572228 |
| ENSMUSG00000027540 | Ptpn1 | 5522 | 18.49691018 | 17.9474575 | 18.22986539 | 19.57920286 | 16.48269968 | 18.59349221 | 17.99286957 | 20.76997183 | 19.04186431 | 21.18451553 | 21.75049721 | 20.9131847 | 19.67201772 | 21.22869913 | 15.03461339 | 19.45040814 | 21.01630334 | 17.96114079 |
| ENSMUSG00000027544 | Nfatc2 | 14020 | 0.0515398 | 0.180583449 | 0.100059371 | 0.220980814 | 0.037763991 | 0.095429658 | 0.053994498 | 0.182517187 | 0.164511047 | 0.17151825 | 0.085190725 | 0.043792603 | 0.038705499 | 0 | 0.132244652 | 0.114707954 | 0.057407435 | 0.01177085 |
| ENSMUSG00000027546 | Atp9a | 5878 | 57.62568012 | 56.19779841 | 57.74703343 | 69.75226914 | 59.73500904 | 69.97911559 | 51.65115017 | 49.17970293 | 37.40279366 | 60.38626984 | 45.4585476 | 47.83928345 | 43.8179551 | 37.1758134 | 44.66422493 | 49.20844646 | 45.90288026 | 48.51432617 |
| ENSMUSG00000027547 | Sall4 | 4892 | 0 | 0 | 0.039553173 | 0 | 0 | 0 | 0 | 0 | 0.025257471 | 0 | 0 | 0 | 0 | 0 | 0 | 0 | 0 | 0 |
| ENSMUSG00000027550 | Lrrcc1 | 7671 | 3.202710435 | 3.230972924 | 3.512456838 | 3.694299254 | 3.582756931 | 3.850010326 | 3.410756283 | 3.350734822 | 2.904693655 | 3.276147156 | 3.643377749 | 2.852266945 | 4.141540181 | 4.213933623 | 4.103505241 | 3.249535317 | 3.382466824 | 4.453220607 |
| ENSMUSG00000027551 | Zfp64 | 6481 | 5.830443063 | 5.04242389 | 4.441019991 | 4.365593046 | 4.968408723 | 4.816883529 | 5.905872127 | 4.767420698 | 5.020420838 | 4.481535752 | 4.695671741 | 5.175931679 | 5.510925491 | 3.819580092 | 4.278451954 | 6.333518151 | 5.428720962 | 5.152064154 |
| ENSMUSG00000027552 | E2f5 | 1953 | 30.57848045 | 26.45586223 | 28.83092132 | 28.20445602 | 28.24331005 | 25.85470329 | 39.58469911 | 39.619973 | 43.18995611 | 35.07935066 | 36.98706463 | 36.92464724 | 49.45821507 | 44.47027313 | 39.38725287 | 47.01529777 | 43.50711738 | 52.8402889 |
| ENSMUSG00000027555 | Car13 | 2292 | 0.463625393 | 0.27615401 | 0.063316139 | 0.337930957 | 0.2519996 | 0.064859599 | 0.04128506 | 0.333267037 | 0 | 0.02057186 | 0 | 0.170466683 | 0.473517531 | 1.114495989 | 0.557263627 | 0.735072742 | 0.568540181 | 0 |
| ENSMUSG00000027556 | Car1 | 1173 | 0 | 0 | 0 | 0 | 0 | 0 | 0 | 0 | 0 | 0 | 0 | 0 | 0 | 0 | 0 | 0 | 0 | 0 |
| ENSMUSG00000027559 | Car3 | 1500 | 0 | 0 | 0 | 0 | 0 | 0.066070311 | 0 | 0 | 0 | 0 | 0 | 0 | 0 | 0 | 0 | 0 | 0 | 0.036672738 |
| ENSMUSG00000027560 | Dok5 | 1760 | 0.120753341 | 0.208205589 | 0.522214248 | 0.025886957 | 0.027347684 | 0.112619849 | 0.053764408 | 0.542505716 | 0.163810006 | 0.187531206 | 0 | 0.158567223 | 0 | 0 | 0 | 0 | 0.04355261 | 0.062510349 |
| ENSMUSG00000027562 | Car2 | 1782 | 158.5237934 | 164.0220729 | 173.1891828 | 165.0629081 | 167.3813305 | 176.6324151 | 285.1770395 | 220.8601048 | 240.2546753 | 281.1314378 | 218.1508626 | 186.3967497 | 337.5166866 | 334.0221234 | 310.7688624 | 308.6025272 | 358.2282835 | 324.0351253 |
| ENSMUSG00000027564 | Cypt12 | 508 | 0 | 0 | 0 | 0 | 0.284243644 | 0 | 0 | 0 | 0 | 0 | 0 | 0 | 0 | 0 | 0 | 0 | 0 | 0 |
| ENSMUSG00000027566 | Psma7 | 3167 | 108.8599567 | 107.3438855 | 108.6915194 | 96.11409448 | 105.5497979 | 108.3838919 | 111.461919 | 113.2989014 | 115.8216323 | 112.5691403 | 107.1202969 | 114.3806916 | 127.7769869 | 131.4131566 | 122.40778 | 110.9665913 | 100.4204647 | 117.9386911 |
| ENSMUSG00000027568 | Ntsr1 | 4056 | 0.03143874 | 0.041066191 | 0.035779238 | 0.056164995 | 0 | 0.085520004 | 0 | 0 | 0 | 0 | 0 | 0 | 0 | 0 | 0 | 0 | 0 | 0 |
| ENSMUSG00000027569 | 1600027N09R | 3050 | 12.70974444 | 14.05694913 | 11.13384207 | 12.92141093 | 13.76099588 | 12.38006 | 11.30850588 | 12.84767373 | 11.34317155 | 11.68719071 | 11.5913014 | 15.15257988 | 10.8530218 | 10.67452631 | 10.33416074 | 11.73826817 | 12.13875502 | 8.711278212 |
| ENSMUSG00000027570 | Col9a3 | 3820 | 0.100143085 | 0.244178282 | 0 | 0.059634875 | 0.28979954 | 0.155663037 | 0.383951059 | 0.149970167 | 0.366581554 | 0 | 0.200104538 | 0.10228001 | 0.309938747 | 0 | 0.053928738 | 0.170403227 | 0.090297558 | 0.259205215 |
| ENSMUSG00000027573 | 2310003C23R | 2458 | 10.60035513 | 10.95069916 | 10.09585886 | 9.28644557 | 10.35874191 | 8.809822853 | 14.05136518 | 8.810045632 | 9.685030964 | 11.24097319 | 9.737694954 | 8.515408247 | 11.50007673 | 9.069634641 | 10.62725241 | 11.96386757 | 10.9771005 | 12.59972623 |
| ENSMUSG00000027574 | Nkain4 | 1230 | 0 | 0 | 0 | 0 | 0.195658226 | 0 | 0 | 0 | 0 | 0 | 0.03884143 | 0.090757175 | 0 | 0.075518841 | 0.267977599 | 0.093391725 | 0.062319182 | 0 |
| ENSMUSG00000027575 | Arfgap1 | 4038 | 15.16838996 | 16.62344771 | 14.79477711 | 17.36464766 | 15.04271611 | 15.79355327 | 18.46577068 | 20.00419294 | 19.53248581 | 17.22320141 | 18.87099029 | 17.02944436 | 18.32537789 | 22.86548928 | 19.57022888 | 15.85485279 | 16.23030404 | 18.84041471 |
| ENSMUSG00000027577 | Chrna4 | 4930 | 0.034486958 | 0.013514359 | 0.01962415 | 0 | 0 | 0.010051264 | 0 | 0 | 0.016708526 | 0.009564037 | 0 | 0 | 0.030019422 | 0 | 0 | 0.03106743 | 0.031096387 | 0 |
| ENSMUSG00000027579 | Srms | 3371 | 0 | 0 | 0 | 0 | 0 | 0.029399427 | 0 | 0 | 0 | 0.041961468 | 0 | 0 | 0.058536835 | 0 | 0 | 0 | 0 | 0.03263667 |
| ENSMUSG00000027580 | BC006779 | 11012 | 1.389562602 | 1.524672965 | 1.027915556 | 1.336380069 | 0.987814634 | 1.309466534 | 0.936629496 | 1.255507598 | 1.133265009 | 0.650826997 | 1.093288189 | 1.540861009 | 1.007962349 | 0.843517751 | 1.036399472 | 1.7524942 | 1.360841091 | 1.004071054 |

| ENSMUSG00000027581 | Stmn3 | 1150 | 0 | 0.115870936 | 0 | 0 | 0 | 0 | 0 | 0 | 0 | 0 | 0 | 0 | 0 | 0 | 0 | 0 | 0 | 0 |
| --- | --- | --- | --- | --- | --- | --- | --- | --- | --- | --- | --- | --- | --- | --- | --- | --- | --- | --- | --- | --- |
| ENSMUSG00000027582 | Zgpat | 3968 | 5.452402873 | 6.556796972 | 6.912245895 | 6.717039046 | 6.392521112 | 6.406389195 | 9.431534534 | 6.987823626 | 6.300457426 | 8.401095565 | 9.933049583 | 6.287701909 | 7.33513891 | 7.619733087 | 8.867471958 | 6.832094264 | 7.591852159 | 9.537869311 |
| ENSMUSG00000027583 | Zbtb46 | 8502 | 1.139870634 | 1.637825192 | 1.570347492 | 1.430816142 | 1.641761687 | 2.150665572 | 2.609932536 | 2.82108077 | 2.77580258 | 2.462351476 | 1.545296822 | 2.816386644 | 1.253316176 | 1.911953727 | 1.32298574 | 2.305903433 | 2.30354478 | 1.889280071 |
| ENSMUSG00000027584 | Oprl1 | 3933 | 0.021614633 | 0.101641172 | 0.172191565 | 0 | 0 | 0.01259922 | 0.024059333 | 0.077686046 | 0 | 0.08391938 | 0.036441616 | 0 | 0 | 0 | 0.125710315 | 0 | 0 | 0 |
| ENSMUSG00000027589 | Pcmtd2 | 4245 | 25.81350855 | 26.50121173 | 27.20085205 | 26.35993519 | 25.19414238 | 27.84058176 | 18.95850809 | 27.33298434 | 28.70928154 | 24.94710943 | 24.56837087 | 29.89983904 | 15.06105178 | 17.49448663 | 23.31357768 | 20.89070595 | 27.69966523 | 27.25186135 |
| ENSMUSG00000027593 | Raly | 3720 | 16.54502551 | 14.59677892 | 18.20509207 | 11.58622256 | 14.06435511 | 12.06848834 | 10.55632352 | 11.262652 | 12.41131295 | 10.26668539 | 11.03190573 | 12.10839777 | 11.97492508 | 11.09913809 | 10.90955176 | 21.3274566 | 10.4264012 | 12.37704901 |
| ENSMUSG00000027596 | a | 1162 | 0 | 0 | 0 | 0 | 0.041421621 | 0 | 0 | 0 | 0 | 0.040577197 | 0 | 0.096068266 | 0 | 0 | 0 | 0 | 0 | 0 |
| ENSMUSG00000027597 | Ahcy | 2795 | 2.965477397 | 1.787811854 | 2.630689307 | 1.988711059 | 2.100928332 | 2.375694562 | 3.26702935 | 2.842225295 | 2.534554816 | 2.311143594 | 1.521277748 | 2.855685063 | 2.841659636 | 2.808247144 | 1.695233963 | 2.301546348 | 2.495665845 | 3.188363252 |
| ENSMUSG00000027598 | Itch | 9757 | 7.71514469 | 9.48480272 | 8.765440368 | 9.208402114 | 9.175502513 | 9.040060036 | 7.899185608 | 8.732935304 | 8.311596755 | 9.268735149 | 8.162432693 | 9.1815249 | 7.225100931 | 6.959234986 | 7.769894726 | 7.012946134 | 10.29550314 | 7.633732756 |
| ENSMUSG00000027599 | Armc1 | 2922 | 20.90346952 | 19.32421482 | 22.38227688 | 18.72649358 | 19.17370265 | 22.14779947 | 24.28782286 | 18.92628292 | 19.84620609 | 18.91191794 | 23.18442544 | 19.96145359 | 18.53745557 | 24.3505619 | 18.03449349 | 17.76935785 | 18.7827711 | 20.72725069 |
| ENSMUSG00000027601 | Mtfr1 | 4319 | 24.52486656 | 22.56854098 | 23.41955359 | 27.22691721 | 20.95114992 | 24.03634565 | 25.81986206 | 23.44247714 | 23.02020122 | 23.87557028 | 20.55241326 | 20.97448955 | 19.34898538 | 23.81885936 | 22.38945397 | 21.45479739 | 27.46466518 | 26.61936398 |
| ENSMUSG00000027602 | Map1lc3a | 1280 | 202.663351 | 220.54177 | 221.5356523 | 205.4518343 | 205.0871185 | 214.6639904 | 215.0509109 | 211.4605905 | 220.7983368 | 224.5552243 | 234.1719449 | 214.628665 | 267.2402456 | 240.8924189 | 224.0978376 | 229.4744136 | 204.2671855 | 241.2235282 |
| ENSMUSG00000027603 | Ggt7 | 3247 | 6.375121874 | 6.484061948 | 6.674265968 | 10.2712302 | 9.798337402 | 9.065082767 | 7.110744477 | 8.633576645 | 6.672037971 | 11.80582746 | 6.944804559 | 9.849822809 | 6.198774946 | 5.750084118 | 6.712541751 | 9.056725126 | 7.070357821 | 8.538524727 |
| ENSMUSG00000027605 | Acss2 | 7912 | 0.644668999 | 1.250496661 | 1.681334789 | 1.727542126 | 1.259265445 | 1.647164931 | 1.949435456 | 1.737773618 | 2.576760023 | 1.221675198 | 2.6870395 | 2.377386033 | 0.997612078 | 1.54383152 | 1.463300961 | 1.785798045 | 1.511350434 | 2.238742715 |
| ENSMUSG00000027606 | Dnajc5b | 1336 | 0 | 0 | 0 | 0 | 0 | 0 | 0 | 0 | 0 | 0 | 0 | 0 | 0 | 0 | 0 | 0 | 0 | 0 |
| ENSMUSG00000027610 | Gss | 6146 | 4.709733953 | 4.520493618 | 5.123847748 | 6.597678077 | 5.787421346 | 4.668244833 | 6.150802223 | 5.536841937 | 7.076628807 | 6.781845376 | 5.736726221 | 6.066524993 | 6.56581652 | 5.478679362 | 5.718341551 | 6.392151512 | 7.121482438 | 7.41987463 |
| ENSMUSG00000027611 | Procr | 2184 | 0.058386231 | 0 | 0 | 0 | 0.176307413 | 0 | 0.281623089 | 0 | 0 | 0 | 0 | 0 | 0 | 0 | 0.207516994 | 0 | 0.070194683 | 0.327435159 |
| ENSMUSG00000027612 | Mmp24 | 4674 | 0.145503384 | 0.092654605 | 0.10349493 | 0.058486578 | 0.195658226 | 0 | 0.010122524 | 0.040856229 | 0 | 0.040351479 | 0 | 0 | 0 | 0 | 0.008815053 | 0.098307079 | 0.016399785 | 0.058845857 |
| ENSMUSG00000027613 | Eif6 | 2811 | 66.9256169 | 67.41979908 | 63.53435577 | 61.59088171 | 69.17572807 | 66.56389968 | 59.36386289 | 62.73010384 | 62.84203703 | 59.44578169 | 63.81891465 | 65.08848878 | 59.08949323 | 59.16623157 | 60.08010587 | 70.12452754 | 61.84563598 | 66.78978344 |
| ENSMUSG00000027615 | Hps3 | 10164 | 2.203879158 | 2.336884453 | 2.470076963 | 2.801618724 | 2.628218972 | 2.949567475 | 2.955878701 | 1.82620106 | 1.831592397 | 2.602473881 | 2.519419298 | 1.987925015 | 2.38796766 | 2.184206392 | 2.983505031 | 2.177486332 | 2.575448719 | 2.267691432 |
| ENSMUSG00000027618 | Nfs1 | 4170 | 20.54926496 | 21.06621152 | 18.10817287 | 20.90126566 | 19.55287259 | 20.27265311 | 19.62852147 | 18.08872776 | 22.67727582 | 21.47222674 | 22.18041238 | 20.66651871 | 22.19344768 | 20.69379241 | 22.23105534 | 20.38492768 | 25.39461831 | 19.2333999 |
| ENSMUSG00000027620 | Rbm39 | 7105 | 70.98155012 | 63.44219165 | 65.34000997 | 61.16273622 | 66.28724463 | 58.98902472 | 68.80149172 | 72.75101677 | 77.94424925 | 62.6065777 | 71.71959284 | 76.61789686 | 72.54326132 | 72.56520959 | 70.19631725 | 77.52433321 | 82.78588016 | 78.39088045 |
| ENSMUSG00000027624 | Epb4.1l1 | 11759 | 1.879640406 | 2.206883629 | 2.476474638 | 1.76292841 | 2.157115722 | 2.465205302 | 1.613435177 | 2.89390514 | 2.94914927 | 2.313628351 | 2.628658741 | 2.330597018 | 2.278019481 | 3.207126368 | 3.409228156 | 3.490733152 | 3.950290998 | 2.96120117 |
| ENSMUSG00000027628 | 0610011L14R | 3897 | 14.28837069 | 17.82329598 | 16.28587935 | 16.56658964 | 17.69901119 | 15.71649441 | 13.28202996 | 16.36677239 | 13.89793899 | 16.75743495 | 17.54322956 | 14.49459855 | 12.08929459 | 14.24190003 | 13.98759519 | 13.82470737 | 15.48000804 | 12.87357078 |
| ENSMUSG00000027630 | Tbl1xr1 | 8111 | 4.758315847 | 6.197652247 | 4.693622043 | 6.313723811 | 5.9044833 | 6.646945399 | 3.2723971 | 4.845269643 | 3.249829872 | 3.970401963 | 4.494179919 | 4.569300944 | 3.314744781 | 1.763627039 | 4.028212775 | 3.370668683 | 4.536215636 | 3.329980446 |
| ENSMUSG00000027634 | Ndrg3 | 6066 | 4.715790219 | 4.448309317 | 4.808644709 | 4.882076957 | 4.197459219 | 4.582770118 | 4.484798942 | 5.62875169 | 6.64035813 | 5.308923562 | 5.402839023 | 5.870490064 | 7.831624849 | 6.492678224 | 5.888847504 | 7.347554734 | 7.973588311 | 7.508661452 |
| ENSMUSG00000027635 | Dsn1 | 3580 | 0.569901801 | 0.251242498 | 0.513462054 | 0.254530974 | 0.4705635 | 0.262989368 | 0.290748306 | 0.384057678 | 0.06902768 | 0.223899988 | 0.320279051 | 0.218273541 | 1.474445572 | 0.428115889 | 0.897687531 | 0.492002218 | 0.256936069 | 0.614626891 |
| ENSMUSG00000027636 | Sla2 | 4706 | 0 | 0 | 0 | 0 | 0 | 0 | 0.030161079 | 0 | 0 | 0.020038548 | 0.030455775 | 0.023721064 | 0 | 0 | 0 | 0.024409652 | 0 | 0 |
| ENSMUSG00000027637 | 1110008F13R | 3144 | 8.111674814 | 7.459375805 | 7.862237281 | 7.173256026 | 8.971153712 | 6.525073696 | 10.11263366 | 7.713796287 | 7.480121024 | 9.013222856 | 7.947297482 | 7.86461149 | 10.71682554 | 9.350861106 | 8.583657477 | 10.68093597 | 9.264626478 | 9.098198313 |
| ENSMUSG00000027639 | Samhd1 | 11107 | 3.095947367 | 3.131238099 | 3.527735625 | 2.457105029 | 2.77342497 | 2.587610109 | 2.560090037 | 3.10504541 | 3.111144929 | 2.924897321 | 3.630329068 | 3.331753335 | 3.193449943 | 3.943168669 | 2.96761004 | 4.102441956 | 2.974454662 | 4.016600842 |
| ENSMUSG00000027641 | Rbl1 | 5971 | 3.075236316 | 3.453472035 | 3.694243819 | 3.006372713 | 4.264241772 | 4.13285234 | 2.448437077 | 3.025457434 | 2.931547345 | 1.903084824 | 2.704393898 | 3.131510127 | 2.164622731 | 1.983460439 | 2.560002887 | 1.840462958 | 3.350582312 | 2.809877332 |
| ENSMUSG00000027642 | Rpn2 | 4560 | 89.70829256 | 86.7523287 | 92.50376857 | 94.189466 | 91.69342564 | 93.70465387 | 101.9816494 | 96.55307345 | 96.2463842 | 98.98545664 | 109.7147731 | 95.43743886 | 113.604049 | 116.4666972 | 124.264254 | 112.8733212 | 121.2489384 | 126.3520579 |
| ENSMUSG00000027643 | Ghrh | 661 | 0 | 0 | 0 | 0 | 0 | 0 | 0 | 0 | 0 | 0.21399714 | 0 | 0 | 0 | 0 | 0 | 0 | 0.173946885 | 0 |
| ENSMUSG00000027646 | Src | 4353 | 11.82489505 | 14.54808452 | 14.19090243 | 13.30302949 | 13.1801638 | 14.44576589 | 10.81463715 | 12.18682448 | 13.83291662 | 13.11727582 | 11.99587172 | 11.71962224 | 9.995578001 | 11.61902393 | 13.26059724 | 12.55242287 | 12.40564033 | 13.69856919 |
| ENSMUSG00000027649 | Ctnnbl1 | 2686 | 22.31284371 | 23.03128986 | 23.538423 | 24.23928978 | 24.28099649 | 25.34827101 | 23.49780852 | 20.93046029 | 19.41255732 | 21.34596246 | 25.82622476 | 21.23738167 | 17.87042275 | 19.38340356 | 21.84326716 | 18.19017686 | 17.97882878 | 13.18907845 |
| ENSMUSG00000027650 | Tti1 | 4764 | 10.62629401 | 11.89446538 | 10.65151833 | 8.635941023 | 10.63873124 | 11.5768666 | 11.00387243 | 9.411813691 | 6.094981867 | 10.40205481 | 10.03835714 | 8.02555182 | 8.398023701 | 8.062397202 | 11.73604352 | 9.580689341 | 10.20103788 | 10.29977397 |
| ENSMUSG00000027651 | Rprd1b | 6344 | 8.817278003 | 8.527764851 | 9.676231088 | 9.171099246 | 9.058877185 | 9.490317039 | 8.412468763 | 9.602282761 | 7.868546202 | 8.628935448 | 8.547379867 | 9.229292253 | 8.328260309 | 7.445403038 | 7.118049378 | 8.371543579 | 8.077279155 | 9.191307239 |
| ENSMUSG00000027652 | Ralgapb | 13129 | 4.998704531 | 4.564696222 | 4.557701047 | 4.889596425 | 4.556933591 | 4.653707099 | 4.436126724 | 4.465331533 | 5.085181053 | 3.57338333 | 3.518804543 | 4.659453591 | 4.24594916 | 3.268667587 | 4.129884039 | 4.126834448 | 4.545208643 | 4.11447504 |
| ENSMUSG00000027654 | Fam83d | 3987 | 0 | 0.091909164 | 0 | 0.045709601 | 0.012072216 | 0 | 0.94933893 | 0.58433322 | 0.485519499 | 0.260174435 | 0.167757567 | 0.209991206 | 0.098985538 | 0.908612695 | 0.609704489 | 0.172869559 | 0.096128159 | 0.496696223 |
| ENSMUSG00000027655 | Dhx35 | 4791 | 2.989823486 | 3.984197117 | 3.796378087 | 3.204773939 | 3.214822704 | 3.588979034 | 5.41167774 | 4.480093958 | 3.232337701 | 3.041028451 | 3.799260944 | 3.390181134 | 3.902483124 | 5.050588505 | 5.452261832 | 3.46062054 | 4.767788135 | 4.615667064 |
| ENSMUSG00000027656 | Wisp2 | 2138 | 0 | 0.327208035 | 0 | 0.213101236 | 0.112562965 | 0 | 0.398329383 | 0 | 0.23116847 | 0 | 0 | 0.13053242 | 0.046147724 | 0 | 0.019271074 | 0.358190902 | 0 | 0 |

| ENSMUSG00000027660 | Skil | 6807 | 9.066771793 | 11.3685696 | 9.479989642 | 12.37584413 | 10.79733325 | 10.01682994 | 3.73246784 | 6.334541254 | 5.572613636 | 4.855684293 | 5.102452595 | 4.304865999 | 4.138170231 | 4.987605095 | 3.946439612 | 4.927658599 | 6.103379733 | 5.826585272 |
| --- | --- | --- | --- | --- | --- | --- | --- | --- | --- | --- | --- | --- | --- | --- | --- | --- | --- | --- | --- | --- |
| ENSMUSG00000027661 | Slc2a10 | 3564 | 0.894469193 | 0.102817575 | 1.452291736 | 1.08661301 | 1.228957647 | 1.279139026 | 1.08856332 | 2.454001165 | 0.057781307 | 0.820242313 | 0.026809741 | 1.879315239 | 1.038129572 | 2.358692429 | 1.965281847 | 0.064462302 | 0.376380581 | 0.864340622 |
| ENSMUSG00000027663 | Zmat3 | 13706 | 0.570622529 | 0.522564735 | 0.405877425 | 0.39890014 | 0.604019471 | 0.444694749 | 0.607546439 | 0.540589965 | 0.444739846 | 0.564184688 | 0.331141183 | 0.557912285 | 0.770248818 | 0.457460368 | 0.471957119 | 0.567123399 | 0.640356194 | 0.301012914 |
| ENSMUSG00000027665 | Pik3ca | 8905 | 6.104898387 | 5.809649042 | 7.43121724 | 6.160078313 | 6.502268857 | 6.716468212 | 5.153655088 | 5.536933353 | 4.393845028 | 5.670792048 | 6.749118222 | 6.192686652 | 4.941501427 | 3.828181936 | 6.102734668 | 5.564070295 | 6.67536064 | 6.20821474 |
| ENSMUSG00000027667 | Zfp639 | 1454 | 19.67399138 | 17.3208721 | 18.16502584 | 15.85549411 | 16.75017426 | 17.82398876 | 14.74047012 | 16.57455703 | 19.85677273 | 15.66285397 | 17.11881249 | 16.4683076 | 18.9659847 | 18.17516212 | 17.28538452 | 16.5908409 | 14.31305311 | 21.82961113 |
| ENSMUSG00000027668 | Mfn1 | 5916 | 10.99990272 | 10.74954616 | 9.705777642 | 9.149175232 | 10.0640956 | 10.10152074 | 8.533236717 | 10.09684202 | 9.864992028 | 9.372756428 | 8.002870834 | 10.88763939 | 8.013518002 | 8.572843717 | 9.200009353 | 8.821855627 | 10.37194073 | 9.196079538 |
| ENSMUSG00000027669 | Gnb4 | 10840 | 2.627164939 | 2.34788295 | 1.990276249 | 2.96735215 | 2.122422464 | 2.450208967 | 0.859833741 | 0.887867657 | 0.744700839 | 0.878638566 | 0.956380626 | 0.890785021 | 1.11042323 | 1.109688066 | 0.878003636 | 1.020847374 | 0.887444698 | 1.755825731 |
| ENSMUSG00000027670 | Ocstamp | 3654 | 0.023265012 | 0.100285122 | 0.06619257 | 0.074812881 | 0.09220675 | 0 | 0.116533692 | 0.04180887 | 0.169074367 | 0 | 0.130747013 | 0.030550445 | 0 | 0 | 0.112757405 | 0 | 0.020977721 | 0 |
| ENSMUSG00000027671 | Actl6a | 2750 | 29.39812538 | 30.50249732 | 27.51134599 | 27.98276505 | 29.99931533 | 26.93866789 | 21.9186738 | 28.72068661 | 23.51375627 | 26.66157948 | 26.61499505 | 27.29893316 | 22.92588737 | 27.41045594 | 23.14778322 | 21.24780614 | 23.49750421 | 22.76376853 |
| ENSMUSG00000027673 | Ndufb5 | 1952 | 63.97551597 | 63.53683667 | 58.80655099 | 58.74853924 | 61.0525835 | 57.5238188 | 74.79863072 | 67.83455898 | 73.59557735 | 69.37337078 | 70.70791754 | 62.47808543 | 75.66586082 | 74.40095352 | 60.36703373 | 63.94845293 | 62.51584495 | 65.97147481 |
| ENSMUSG00000027674 | Pex5l | 6260 | 0.020369893 | 0 | 0 | 0 | 0.05382164 | 0 | 0 | 0 | 0 | 0 | 0 | 0 | 0 | 0 | 0 | 0 | 0 | 0 |
| ENSMUSG00000027676 | Ccdc39 | 7024 | 0.21785113 | 0.336733413 | 0.22726744 | 0.214053881 | 0.301509772 | 0.148150257 | 0.26943439 | 0.239246257 | 0.158319465 | 0.127543189 | 0.516927227 | 0.317856848 | 0.182606755 | 0.092570789 | 0.18184058 | 0.109027925 | 0.158237843 | 0.031326371 |
| ENSMUSG00000027677 | Ttc14 | 6558 | 21.53128923 | 19.67380898 | 20.79368437 | 22.61378459 | 22.64945356 | 19.1546477 | 23.07917961 | 22.35169875 | 21.47878681 | 19.60658246 | 24.42656843 | 26.94607925 | 16.42140522 | 18.27169037 | 18.69085526 | 21.46913135 | 19.43782609 | 22.50525059 |
| ENSMUSG00000027678 | Ncoa3 | 7992 | 6.520438665 | 6.573377651 | 7.481191631 | 9.885241601 | 7.365533364 | 8.463398565 | 8.791207233 | 6.676024294 | 6.045017892 | 8.961701482 | 6.019692591 | 8.415649827 | 7.049180372 | 7.717435943 | 5.835856015 | 6.774639498 | 8.727960506 | 8.060018015 |
| ENSMUSG00000027679 | Dnajc19 | 3992 | 16.37599217 | 14.67870264 | 14.80772898 | 13.44461679 | 16.2650213 | 14.42391694 | 19.74522122 | 18.82831862 | 19.42740711 | 17.38623126 | 18.56186388 | 19.00137411 | 20.30369241 | 19.35945927 | 17.77281544 | 15.56754905 | 17.19498942 | 17.81732841 |
| ENSMUSG00000027680 | Fxr1 | 2446 | 15.17049005 | 12.31187914 | 12.20215381 | 13.24362327 | 12.79057661 | 10.53451409 | 10.27106807 | 14.86474534 | 13.45381279 | 11.02624786 | 11.56286812 | 11.18138784 | 10.16487584 | 11.60152796 | 11.28579004 | 12.13214257 | 13.55365774 | 13.22377545 |
| ENSMUSG00000027684 | Mecom | 12100 | 0.555026265 | 0.83144579 | 0.37979218 | 1.114551167 | 0.342094664 | 0.790386577 | 0.172046105 | 0.407175199 | 0.02382691 | 0.245495397 | 0.477749585 | 0.456673603 | 1.121179938 | 0.491309354 | 0.568649573 | 1.063276371 | 0.886889515 | 0.418251059 |
| ENSMUSG00000027690 | Slc2a2 | 2755 | 0.293139145 | 0.048367179 | 0 | 0.049612752 | 0.104824516 | 0.107918839 | 0.051520159 | 0.207944115 | 1.181028946 | 0.034229186 | 0.433529569 | 0.243117224 | 0.286501153 | 0 | 0.55334213 | 0.041695761 | 0.528638578 | 0.459241181 |
| ENSMUSG00000027692 | Tnik | 15796 | 0.201816169 | 0.120209861 | 0.006124782 | 0.04903379 | 0.04265934 | 0.156852157 | 0.074880791 | 0.108803375 | 0.041718426 | 0.032834752 | 0.090734917 | 0 | 0.262337367 | 0.388112151 | 0.224318423 | 0.070298026 | 0.03154228 | 0.10447412 |
| ENSMUSG00000027694 | Gm8325 | 1707 | 0.373507698 | 0.33176286 | 0.311721637 | 0.240216402 | 0.366558294 | 0.435437026 | 0.138584297 | 0.357983854 | 0.217152104 | 0.303841673 | 0.251889061 | 0.392377241 | 0.520195964 | 0.217664147 | 0.313778692 | 0.224315216 | 0.157167005 | 0.580060879 |
| ENSMUSG00000027695 | Pld1 | 8573 | 0.188405073 | 0.097144798 | 0.225701763 | 0.005314481 | 0.230188834 | 0.22542361 | 0.331128046 | 0.196018396 | 0 | 0.098998327 | 0.061299958 | 0.117191406 | 0.07480636 | 0.086679739 | 0.158596914 | 0 | 0.017882327 | 0.012833105 |
| ENSMUSG00000027698 | Nceh1 | 5455 | 4.379085046 | 3.346556559 | 3.662377275 | 4.309715651 | 4.694075782 | 5.177829173 | 4.119802473 | 3.920759917 | 2.287720307 | 4.278569773 | 2.933934205 | 2.885429303 | 2.794420245 | 3.618466937 | 4.652641325 | 4.204596486 | 4.166360048 | 4.144590809 |
| ENSMUSG00000027699 | Ect2 | 6205 | 0.287706268 | 0.574452809 | 0.561304462 | 1.027968768 | 0.51195922 | 0.39131087 | 0.495620327 | 1.070987594 | 0.969094489 | 0.326749434 | 0.415769179 | 0.881536653 | 0.44521956 | 0.112274184 | 0.278882409 | 0.370255672 | 0.568254523 | 0.425533783 |
| ENSMUSG00000027702 | Lrrc34 | 1880 | 0 | 0.407551365 | 0 | 0.145407588 | 0.025602087 | 0 | 0 | 0.020315108 | 0 | 0.025080161 | 0 | 0 | 0 | 0.049408604 | 0.021915721 | 0 | 0.122317969 | 0.087780489 |
| ENSMUSG00000027703 | Lrriq4 | 2621 | 0 | 0.012709994 | 0 | 0 | 0.018363954 | 0 | 0.144411076 | 0.029143382 | 0 | 0 | 0 | 0 | 0 | 0 | 0 | 0 | 0.102359435 | 0 |
| ENSMUSG00000027706 | Sec62 | 3958 | 42.53739319 | 36.94044788 | 41.48048561 | 36.73200922 | 40.17884684 | 39.72481648 | 38.92119318 | 37.0537709 | 43.71514723 | 38.10891858 | 38.66174634 | 43.65975022 | 41.1930234 | 41.19888601 | 38.64077197 | 40.9800437 | 38.99444104 | 43.86274402 |
| ENSMUSG00000027708 | Dcun1d1 | 6567 | 6.809113017 | 6.148199755 | 6.961015104 | 6.438350715 | 6.860283319 | 6.338403564 | 6.340057478 | 5.559911177 | 6.378359453 | 6.31117225 | 5.747254027 | 5.847597969 | 5.235929091 | 5.155739258 | 5.62780503 | 4.886177707 | 7.33026175 | 5.042710865 |
| ENSMUSG00000027709 | Mccc1 | 10075 | 3.978400099 | 3.498267204 | 3.303323959 | 4.169457358 | 3.382372403 | 3.615013318 | 4.87919339 | 5.966733636 | 5.257157248 | 5.21816716 | 5.04541497 | 5.429215815 | 3.505871243 | 5.204503689 | 5.610772673 | 4.218617788 | 5.515943475 | 3.663633808 |
| ENSMUSG00000027710 | Acad9 | 8505 | 5.592391767 | 5.608943506 | 6.774000547 | 5.517680854 | 5.902598046 | 5.476727756 | 5.446103783 | 6.277834046 | 6.246984754 | 5.438546725 | 7.414808371 | 5.998296955 | 4.506866517 | 3.99730417 | 5.023634739 | 5.262988836 | 5.272400632 | 4.93497336 |
| ENSMUSG00000027712 | Anxa5 | 1665 | 349.0785447 | 362.0401327 | 354.4486909 | 348.7814241 | 355.626982 | 354.9642363 | 308.5127658 | 320.5179813 | 339.3620479 | 297.2335023 | 311.0393693 | 313.9421502 | 279.6660463 | 252.4996271 | 283.5607381 | 281.8556663 | 280.0927212 | 278.5806534 |
| ENSMUSG00000027713 | 1810062G17R | 387 | 4.283467352 | 3.615352861 | 5.499832909 | 1.883660748 | 2.736181707 | 3.073037741 | 4.401179435 | 2.861962971 | 3.086328055 | 5.604476356 | 3.086237628 | 4.038342513 | 7.13846865 | 5.400475278 | 5.642590342 | 2.572495588 | 2.079721537 | 4.406414234 |
| ENSMUSG00000027714 | Exosc9 | 3387 | 13.40287216 | 11.15347566 | 11.82559289 | 12.5908289 | 11.53915619 | 8.953726882 | 10.30905139 | 10.81385117 | 11.57648745 | 11.4013658 | 12.11653066 | 9.113097552 | 8.709916307 | 10.01009265 | 11.11845943 | 11.70084991 | 9.935190045 | 6.96749536 |
| ENSMUSG00000027715 | Ccna2 | 4148 | 0.215190139 | 0.248963289 | 0.303209207 | 0.186725592 | 0.150847398 | 0.179192624 | 0.171092137 | 0.119696536 | 0.426957613 | 0.113670934 | 0.115175888 | 0.551697726 | 0.380574097 | 0.526246892 | 0.506576507 | 0.49847946 | 0.776135231 | 0.556987098 |
| ENSMUSG00000027716 | Trpc3 | 5822 | 0 | 0 | 0 | 0 | 0 | 0 | 0 | 0 | 0 | 0 | 0 | 0 | 0 | 0 | 0 | 0 | 0 | 0 |
| ENSMUSG00000027718 | Il21 | 3179 | 0 | 0 | 0 | 0 | 0 | 0 | 0 | 0.084097772 | 0 | 0 | 0 | 0 | 0 | 0 | 0 | 0 | 0 | 0 |
| ENSMUSG00000027719 | Adad1 | 3460 | 0 | 0 | 0 | 0 | 0 | 0 | 0.041022554 | 0.011038267 | 0.071421704 | 0 | 0 | 0 | 0 | 0 | 0 | 0 | 0 | 0 |
| ENSMUSG00000027720 | Il2 | 1197 | 0.745704843 | 0.417454815 | 0.444535367 | 0.228376162 | 0.402104625 | 0.496769259 | 0.276682333 | 0.350974458 | 0.344081168 | 0.236344377 | 0.19956123 | 0.466296262 | 0.206064817 | 0.27160285 | 0.172103408 | 0.447843361 | 0.512298037 | 0.413602306 |
| ENSMUSG00000027722 | Spata5 | 3937 | 7.978492528 | 6.388426501 | 8.195363156 | 9.825076617 | 8.936864669 | 7.753234413 | 9.962461476 | 10.32174655 | 5.157468192 | 7.664833651 | 9.610812069 | 8.222779858 | 5.613588758 | 6.441064698 | 7.723329502 | 4.697577692 | 8.732204298 | 8.076013128 |
| ENSMUSG00000027737 | Slc7a11 | 11233 | 1.664940573 | 0.951966442 | 1.128270716 | 1.484495881 | 1.088356504 | 0.705816556 | 1.461541849 | 0.952005045 | 0.894641667 | 1.183699663 | 0.850617973 | 0.834775333 | 1.339467174 | 1.041922017 | 0.975662233 | 1.441905718 | 1.163470778 | 0.597446009 |
| ENSMUSG00000027739 | Rab33b | 3163 | 14.49986877 | 16.37734762 | 17.75582319 | 17.60214865 | 17.16497309 | 16.62202034 | 15.12270578 | 18.24493204 | 16.432938 | 17.62002253 | 11.69074229 | 14.7700631 | 13.11670643 | 13.69975768 | 17.25958317 | 15.74963017 | 16.17629035 | 13.65227593 |
| ENSMUSG00000027742 | Cog6 | 3134 | 22.28334531 | 22.27945957 | 28.36967096 | 26.82199324 | 22.26907764 | 25.1558389 | 18.38763335 | 22.65465095 | 24.81178742 | 23.13904965 | 19.63437987 | 18.14810471 | 15.96125211 | 23.08866884 | 24.12407628 | 21.97983494 | 24.25049354 | 19.34270441 |

| ENSMUSG00000027744 | Stoml3 | 2069 | 0 | 0 | 0.2338015 | 0.330312066 | 0 | 0 | 0 | 0 | 0 | 0 | 0 | 0 | 0 | 0 | 0 | 0.074027274 | 0.018524068 | 0 |
| --- | --- | --- | --- | --- | --- | --- | --- | --- | --- | --- | --- | --- | --- | --- | --- | --- | --- | --- | --- | --- |
| ENSMUSG00000027746 | Ufm1 | 4289 | 13.49779663 | 13.7010831 | 13.55676929 | 13.60776353 | 12.68106173 | 13.14782253 | 17.01006083 | 13.60643294 | 12.90619659 | 15.01698779 | 13.41129634 | 14.14586739 | 16.63183781 | 16.15634842 | 16.15784962 | 15.83761658 | 14.65496081 | 15.40357593 |
| ENSMUSG00000027748 | Trpc4 | 3294 | 0 | 0 | 0 | 0 | 0 | 0.075216657 | 0 | 0 | 0 | 0 | 0 | 0 | 0 | 0 | 0 | 0 | 0 | 0 |
| ENSMUSG00000027750 | Postn | 5358 | 0.126928484 | 0.273566134 | 0.144452498 | 1.394552309 | 0 | 0.268202552 | 0 | 0 | 0 | 0 | 0 | 0.041669028 | 0 | 0.008668176 | 0 | 0 | 0 | 0 |
| ENSMUSG00000027751 | Fam48a | 3832 | 31.62376223 | 33.4867611 | 35.06828479 | 34.32534838 | 37.84485543 | 33.59551196 | 32.24966528 | 36.83693092 | 31.20158018 | 30.61350462 | 34.07331981 | 31.09771389 | 29.21036542 | 32.80901476 | 38.11573999 | 33.20451159 | 33.82555744 | 31.16512805 |
| ENSMUSG00000027752 | Exosc8 | 3604 | 8.503393983 | 8.799632435 | 8.684149317 | 8.495289066 | 8.413738044 | 6.764690599 | 8.28365716 | 7.248502565 | 8.948124817 | 7.614236764 | 7.860863035 | 7.01567568 | 8.240237015 | 8.466638572 | 7.739581947 | 8.818314135 | 9.209371009 | 7.08219354 |
| ENSMUSG00000027761 | Aadac | 1433 | 0 | 0 | 0 | 0 | 0 | 0 | 0 | 0 | 0 | 0 | 0 | 0 | 0 | 0 | 0 | 0 | 0 | 0 |
| ENSMUSG00000027762 | Sucnr1 | 1569 | 0 | 0 | 0 | 0 | 0 | 0 | 0 | 0 | 0 | 0 | 0 | 0 | 0 | 0 | 0 | 0 | 0 | 0 |
| ENSMUSG00000027763 | Mbnl1 | 5633 | 11.63553727 | 10.43800076 | 10.16763003 | 11.49338612 | 11.7659611 | 10.30112747 | 9.549887439 | 8.990435929 | 8.20366957 | 10.37934884 | 11.34793084 | 7.263071308 | 11.38496227 | 7.634870393 | 9.83775833 | 10.16233944 | 9.783989872 | 7.304600005 |
| ENSMUSG00000027765 | P2ry1 | 3884 | 0.328309805 | 0.18011606 | 0.286454994 | 0.152495771 | 0.285024265 | 0.051032681 | 0.219265762 | 0.186832041 | 0.339332777 | 0.242794558 | 0.381314035 | 0.531714602 | 1.651171279 | 1.219695395 | 1.082018203 | 0.384483442 | 0.552593364 | 0.439053118 |
| ENSMUSG00000027770 | Dhx36 | 4467 | 15.37684452 | 15.44459457 | 18.97255992 | 17.4920956 | 17.57402451 | 16.02948288 | 14.35161852 | 16.71505411 | 13.60897633 | 14.2391535 | 19.80730313 | 18.54274531 | 14.86473263 | 17.2176872 | 16.12274898 | 15.82369851 | 20.48873898 | 15.94734569 |
| ENSMUSG00000027774 | Gfm1 | 4606 | 12.25507463 | 12.54115471 | 12.8127892 | 11.20726514 | 11.59931291 | 13.0605772 | 12.43935175 | 9.925381172 | 12.95685222 | 13.27713029 | 14.16860471 | 10.6153974 | 9.917793182 | 9.196050304 | 11.81659907 | 9.136208757 | 12.48142539 | 9.554338988 |
| ENSMUSG00000027775 | Mfsd1 | 2962 | 31.05374778 | 30.91733498 | 34.09990931 | 35.43978601 | 39.03203263 | 42.57653847 | 31.40335137 | 36.94167214 | 30.10425612 | 35.30731257 | 36.51671371 | 37.51822559 | 32.42715832 | 35.09178514 | 33.95442199 | 32.57674904 | 35.49258353 | 36.1774949 |
| ENSMUSG00000027776 | Il12a | 1362 | 0 | 0 | 0 | 0 | 0 | 0 | 1.111604791 | 0 | 0 | 0.034618725 | 1.157543047 | 0 | 0.217321221 | 0.340999173 | 0.242006202 | 0.11245406 | 0 | 0 |
| ENSMUSG00000027777 | Schip1 | 2547 | 0.634156533 | 0.523170698 | 0.569770676 | 0.321986179 | 0.737002365 | 0.875489993 | 0.334365222 | 0.014995054 | 0 | 0.462806275 | 0.281360179 | 0 | 0.077474546 | 0.419400868 | 0.469118618 | 0.045100833 | 0.105333364 | 0.518342584 |
| ENSMUSG00000027778 | Ift80 | 11708 | 0.820479141 | 0.70279167 | 1.189919435 | 0.708243088 | 0.764651333 | 0.884567929 | 1.01834601 | 1.347237973 | 0.714115366 | 1.02694135 | 0.942604664 | 1.072644694 | 0.653095933 | 1.043286213 | 0.7390098 | 0.523274443 | 0.854387042 | 0.704763069 |
| ENSMUSG00000027782 | Kpna4 | 8922 | 7.560604613 | 6.702157041 | 7.547181602 | 6.327071725 | 6.711064004 | 6.142717254 | 6.220328669 | 7.671013798 | 6.615139777 | 5.718119359 | 6.95580263 | 5.986952376 | 5.800177227 | 5.569958922 | 5.726286629 | 7.188608765 | 5.928075513 | 6.54165683 |
| ENSMUSG00000027783 | Arl14 | 1195 | 0 | 0 | 0 | 0 | 0 | 0 | 0 | 0 | 0 | 0 | 0 | 0 | 0 | 0 | 0 | 0 | 0 | 0 |
| ENSMUSG00000027784 | Ppm1l | 9987 | 1.974807417 | 2.54174681 | 3.322743749 | 2.322075855 | 2.669979545 | 2.341933538 | 2.927729606 | 3.116732548 | 2.507399778 | 2.785512659 | 3.458625711 | 3.867471564 | 2.030181035 | 2.055500814 | 2.665085121 | 2.768180489 | 1.903458823 | 2.329914103 |
| ENSMUSG00000027787 | Nmd3 | 2876 | 20.05546727 | 16.6911963 | 17.54297363 | 18.31313187 | 17.43861768 | 16.04088837 | 15.90798349 | 17.94086775 | 19.56216291 | 16.50930396 | 23.63830526 | 19.64028183 | 15.64350089 | 17.05318369 | 18.0220992 | 18.85239919 | 23.02776114 | 19.85377357 |
| ENSMUSG00000027788 | Otol1 | 2156 | 0 | 0 | 0 | 0 | 0 | 0 | 0 | 0 | 0 | 0 | 0 | 0 | 0 | 0 | 0 | 0 | 0 | 0 |
| ENSMUSG00000027790 | Sis | 6890 | 0 | 0 | 0 | 0 | 0 | 0 | 0 | 0 | 0 | 0 | 0 | 0 | 0 | 0 | 0 | 0 | 0 | 0 |
| ENSMUSG00000027792 | Bche | 6735 | 4.266295322 | 5.638708153 | 6.550357785 | 6.047746642 | 8.139905131 | 6.967548433 | 8.099705836 | 7.933358718 | 9.20963515 | 9.290123715 | 8.795983447 | 8.801222519 | 3.88209594 | 3.654842809 | 6.313289633 | 3.553322884 | 7.3465849 | 5.406982725 |
| ENSMUSG00000027793 | Ccna1 | 1557 | 0 | 0 | 0 | 0 | 0.030913246 | 0 | 0 | 0 | 0.105809931 | 0 | 0 | 0 | 0 | 0 | 0 | 0 | 0 | 0 |
| ENSMUSG00000027794 | Sohlh2 | 2353 | 0 | 0 | 0 | 0 | 0 | 0 | 0 | 0 | 0 | 0 | 0 | 0 | 0 | 0 | 0 | 0 | 0 | 0 |
| ENSMUSG00000027796 | Smad9 | 5362 | 0 | 0.006212774 | 0 | 0 | 0 | 0 | 0 | 0 | 0 | 0 | 0 | 0.052047429 | 0.322009904 | 0.944126357 | 0.583983261 | 1.078306922 | 0.357388073 | 0.492435122 |
| ENSMUSG00000027797 | Dclk1 | 11213 | 0 | 0.071302012 | 0.017256231 | 0.056885278 | 0.004292511 | 0.039772996 | 0.438822671 | 0.252050101 | 0.150597266 | 0.256505208 | 0.119298924 | 0.243910414 | 0.171581626 | 0.04970383 | 0.044093344 | 0.095615536 | 0.006836047 | 0.137363327 |
| ENSMUSG00000027799 | Nbea | 11562 | 5.290170239 | 5.027763398 | 5.430621207 | 4.40951467 | 5.170372703 | 4.830127205 | 4.002058467 | 3.745907657 | 3.084892116 | 3.723282483 | 3.297389454 | 4.470273617 | 4.727535404 | 4.703859742 | 4.45085135 | 3.99067479 | 4.282786334 | 2.830861313 |
| ENSMUSG00000027800 | Tm4sf1 | 4054 | 9.373366396 | 11.02760337 | 4.677460261 | 8.361474343 | 8.310889632 | 6.832752361 | 0.735248834 | 1.724028032 | 0.589249609 | 1.314264792 | 0.836709929 | 1.266660325 | 2.299884649 | 3.104673824 | 2.530633305 | 6.84772826 | 3.866663896 | 1.261925734 |
| ENSMUSG00000027801 | Tm4sf4 | 1491 | 0.171046986 | 0.446853041 | 0.875979423 | 1.038950709 | 0.387379667 | 0.265876505 | 0.856768834 | 1.383225842 | 0.110493671 | 0.758965042 | 0.480633385 | 0.861006197 | 4.235067345 | 2.959214153 | 1.132973703 | 0.282492744 | 0.334166237 | 2.619481273 |
| ENSMUSG00000027803 | Wwtr1 | 8100 | 9.41935691 | 10.0432207 | 8.139891589 | 11.02464777 | 10.46423674 | 12.34536004 | 5.82938933 | 7.223550677 | 5.53729823 | 8.824748906 | 6.393587036 | 7.793520294 | 6.723757614 | 7.843890315 | 6.719414239 | 9.383562431 | 8.578466208 | 8.679214616 |
| ENSMUSG00000027804 | Ppid | 3333 | 20.82536827 | 18.15067984 | 22.52496823 | 20.72323529 | 19.59646577 | 17.36501435 | 19.22033221 | 19.13630724 | 20.51293614 | 17.23059003 | 19.60880384 | 18.43775713 | 12.46248855 | 18.14287482 | 17.88738415 | 16.37087175 | 17.36354885 | 18.73247409 |
| ENSMUSG00000027805 | Pfn2 | 4421 | 14.55617202 | 14.95727099 | 16.91596425 | 17.61226527 | 17.50647665 | 16.77910929 | 17.05867682 | 16.13739147 | 17.40249071 | 19.62390726 | 17.89534749 | 20.50319748 | 20.58751128 | 23.65801511 | 25.41430512 | 23.73134229 | 23.41536483 | 21.38897409 |
| ENSMUSG00000027806 | Tsc22d2 | 9489 | 9.697935093 | 10.50397086 | 10.96548254 | 11.76837598 | 10.97665537 | 11.34767521 | 9.318937392 | 11.83727005 | 10.06984051 | 9.74914952 | 9.258947059 | 10.22904808 | 6.966463184 | 8.795449999 | 10.04312349 | 9.002670999 | 10.62263261 | 10.97979219 |
| ENSMUSG00000027808 | Serp1 | 2437 | 148.3582385 | 159.2512177 | 170.1509652 | 160.6321268 | 159.4851389 | 168.8696973 | 179.81536 | 192.1530759 | 177.7594472 | 193.4591226 | 170.8881055 | 179.2651584 | 184.0685777 | 183.0891865 | 184.2488946 | 160.4528858 | 190.6406939 | 201.4818575 |
| ENSMUSG00000027809 | Etfdh | 7119 | 14.91472534 | 15.42341612 | 14.16755314 | 15.27661369 | 15.1650379 | 15.22290045 | 13.81033106 | 14.37781127 | 18.20098028 | 14.49160538 | 15.51562073 | 12.78765918 | 13.03460267 | 13.1066402 | 13.02777108 | 14.69447105 | 14.50897179 | 12.03879594 |
| ENSMUSG00000027810 | Eif2a | 6147 | 6.94935772 | 5.62531059 | 6.704774339 | 5.707174905 | 5.809970288 | 5.175346504 | 6.165195351 | 5.840386898 | 6.867769425 | 7.14892719 | 7.266892171 | 7.236877026 | 6.765382506 | 5.145343015 | 7.15179114 | 7.381547059 | 10.89247449 | 7.13230162 |
| ENSMUSG00000027811 | 4930579G24R | 1965 | 4.391119966 | 3.475391 | 2.19096397 | 3.315638341 | 1.984064029 | 4.312222617 | 2.913401603 | 3.381922656 | 3.437449662 | 4.199172046 | 4.813965282 | 4.005093346 | 3.238583882 | 5.058032927 | 5.074186527 | 3.799831269 | 4.661569954 | 2.547495528 |
| ENSMUSG00000027820 | Mme | 4355 | 56.76466218 | 79.07892088 | 81.40748656 | 77.21838536 | 86.42747258 | 94.01934272 | 102.2410152 | 135.1072678 | 126.3399917 | 143.3034922 | 143.4674873 | 133.9191408 | 81.61572078 | 56.39410657 | 88.26877531 | 53.29911873 | 108.9945321 | 73.89282993 |
| ENSMUSG00000027822 | Slc33a1 | 2848 | 9.55172495 | 8.363314384 | 10.07215713 | 9.566539506 | 8.145922475 | 9.273738412 | 14.63569878 | 10.31248506 | 12.35017011 | 11.10889111 | 8.756505732 | 9.955883636 | 8.019900877 | 10.37164311 | 11.61687127 | 13.36406733 | 11.92313871 | 10.44941248 |
| ENSMUSG00000027823 | Gmps | 10283 | 8.85403939 | 9.262040705 | 9.606024408 | 9.242471894 | 9.988673063 | 10.28352946 | 8.668393183 | 9.939012821 | 9.204182945 | 9.679581307 | 10.43030067 | 9.314371001 | 8.472251863 | 8.94284674 | 9.57616635 | 10.40768722 | 12.19896623 | 11.24468952 |

| ENSMUSG00000027824 | Vmn2r1 | 2733 | 0 | 0 | 0.088498958 | 0 | 0.123279717 | 0 | 0.086558139 | 0.027949069 | 0.316471581 | 0.155271251 | 0.454500154 | 0.102114275 | 0 | 0.20392574 | 0.180906941 | 0.126094207 | 0.140235261 | 0 |
| --- | --- | --- | --- | --- | --- | --- | --- | --- | --- | --- | --- | --- | --- | --- | --- | --- | --- | --- | --- | --- |
| ENSMUSG00000027827 | Kcnab1 | 8098 | 0.624612984 | 0.64585384 | 0.812398139 | 0.42759192 | 0.618142759 | 0.715938482 | 0.432346041 | 0.58953449 | 1.205384568 | 0.652121359 | 0.843642759 | 0.868458074 | 0.554359653 | 0.596466422 | 0.849673973 | 1.115903105 | 0.728852769 | 1.236312352 |
| ENSMUSG00000027828 | Ssr3 | 4522 | 142.1789678 | 148.3757193 | 157.3155987 | 148.9449178 | 156.1999071 | 159.5396391 | 133.4317988 | 140.4808114 | 139.4348452 | 140.659661 | 140.0814185 | 144.4887541 | 128.8389967 | 124.6040841 | 152.2963305 | 136.3118527 | 164.7386627 | 154.6507682 |
| ENSMUSG00000027829 | Ccnl1 | 6034 | 20.06210496 | 23.24835889 | 21.42096008 | 21.27792889 | 23.90642612 | 23.97156601 | 26.76138104 | 29.22978361 | 25.82860054 | 22.41885443 | 27.1891295 | 29.60061655 | 29.80027154 | 31.18850549 | 26.22724166 | 35.41595623 | 38.35805552 | 42.46476949 |
| ENSMUSG00000027831 | Veph1 | 9103 | 0.042024232 | 0.003659551 | 0.058454228 | 0.045045523 | 0.031724876 | 0.076209851 | 0.467773383 | 0.474101008 | 0.176455467 | 0.150210963 | 0.288654588 | 0.220736444 | 0 | 0.132653661 | 0.104101481 | 0 | 0.088416152 | 0.024171858 |
| ENSMUSG00000027832 | Ptx3 | 1919 | 0 | 0.086797536 | 0.075623028 | 0 | 0 | 0 | 0 | 0 | 0 | 0 | 0 | 0.058171613 | 0 | 0 | 0 | 0 | 0.019972015 | 0.114662025 |
| ENSMUSG00000027833 | Shox2 | 4414 | 0 | 0 | 0 | 0 | 0 | 0 | 0 | 0 | 0 | 0 | 0 | 0 | 0 | 0 | 0 | 0 | 0 | 0 |
| ENSMUSG00000027834 | Serpini1 | 5294 | 1.67001825 | 1.925528075 | 2.476242298 | 1.557716098 | 1.9092754 | 2.648927768 | 0.947326023 | 1.3129991 | 1.50151068 | 0.52548007 | 1.209264155 | 1.001603314 | 0.615018236 | 0.377237582 | 1.253012939 | 1.012596342 | 0.593644946 | 0.488369478 |
| ENSMUSG00000027835 | Pdcd10 | 3255 | 25.56839775 | 22.75101808 | 23.22821135 | 22.33960884 | 21.11594071 | 21.35867441 | 20.13150855 | 23.40824664 | 18.73954834 | 21.91674778 | 24.23239829 | 23.21794382 | 24.43104475 | 23.41466894 | 24.60701218 | 25.89174405 | 25.66861052 | 24.96112155 |
| ENSMUSG00000027840 | Wnt2b | 3568 | 0.023825771 | 0.158721749 | 0.013557604 | 0 | 0.013489889 | 0.055552392 | 0.14586308 | 0.021408297 | 0.05771653 | 0.158578598 | 0.026779685 | 0.156434032 | 0.20739315 | 0.013016841 | 0.023095043 | 0.04292669 | 0.032225025 | 0.154173505 |
| ENSMUSG00000027843 | Ptpn22 | 3041 | 3.172862532 | 4.98433636 | 3.563193292 | 4.239978805 | 5.096507537 | 6.143170194 | 4.511896379 | 5.124136857 | 6.79895788 | 4.86856982 | 3.142055803 | 5.249352022 | 6.099572804 | 6.170145118 | 7.031768335 | 7.038622012 | 6.087340153 | 6.855788046 |
| ENSMUSG00000027845 | Dclre1b | 11082 | 1.047095565 | 0.85972638 | 1.017057623 | 0.805808039 | 1.085813113 | 1.019493165 | 1.033177071 | 1.257916159 | 1.222736301 | 1.250884927 | 1.534730664 | 1.571420928 | 1.331009138 | 0.989063763 | 1.416512615 | 1.637768265 | 1.597793645 | 1.4395092 |
| ENSMUSG00000027848 | Olfml3 | 6963 | 0.189237463 | 0.411447494 | 0.166733409 | 0.15703936 | 0.331801283 | 0.426994688 | 0.462051151 | 0.323617944 | 0.697976283 | 0.311493947 | 0.65181977 | 0.665331035 | 0.276309748 | 0.146742772 | 0.25444017 | 0.230964457 | 0.280718245 | 0.537213738 |
| ENSMUSG00000027849 | Syt6 | 6014 | 0 | 0 | 0 | 0 | 0 | 0 | 0 | 0 | 0 | 0 | 0 | 0 | 0 | 0 | 0 | 0 | 0 | 0 |
| ENSMUSG00000027852 | Nras | 3011 | 28.85439382 | 30.35887803 | 30.89415439 | 25.76899984 | 25.7843882 | 27.23672337 | 20.09728208 | 23.45325541 | 23.97873203 | 21.07766409 | 21.53125827 | 23.82036746 | 23.36343874 | 21.19368186 | 19.7729154 | 22.39447348 | 23.9427979 | 19.34727467 |
| ENSMUSG00000027854 | Sike1 | 3797 | 22.44479271 | 23.81121804 | 22.47324402 | 22.67852878 | 19.85109098 | 22.08143935 | 21.56919864 | 19.0207619 | 22.45353901 | 23.01033793 | 21.30181846 | 23.85799852 | 14.29157467 | 16.02363826 | 19.0870521 | 19.61423002 | 23.7205156 | 20.00726268 |
| ENSMUSG00000027855 | Sycp1 | 3433 | 0.037144051 | 0.145555903 | 0.070453729 | 0.199072434 | 0.028040736 | 0.216513546 | 0.04134519 | 0.14462605 | 0.35991712 | 0.054938192 | 0.069581938 | 0.048775703 | 0.27302838 | 0.027057435 | 0.240032368 | 0.055768435 | 0.033492249 | 0.096141754 |
| ENSMUSG00000027857 | Tshb | 640 | 0.464900363 | 0.780770958 | 0.604669129 | 1.139026108 | 0.752061307 | 0.077426146 | 0.369630304 | 0.537080659 | 1.930617926 | 0 | 0.447890236 | 0.436059864 | 0.693730087 | 1.306239958 | 1.158793759 | 0.29914537 | 0.898272584 | 0 |
| ENSMUSG00000027858 | Tspan2 | 4576 | 0 | 0.021839747 | 0 | 0.069695653 | 0 | 0.010828832 | 0 | 0.083462418 | 0 | 0 | 0 | 0 | 0 | 0 | 0 | 0 | 0 | 0 |
| ENSMUSG00000027859 | Ngf | 1183 | 3.77264876 | 3.097564129 | 2.167199585 | 3.543208858 | 3.010788125 | 2.513240925 | 5.479152167 | 9.007337507 | 6.928247368 | 11.4787849 | 6.784609488 | 11.22918656 | 5.045783593 | 4.122256277 | 2.577273995 | 2.783594452 | 4.794836806 | 2.231984043 |
| ENSMUSG00000027860 | Vangl1 | 4344 | 15.36213774 | 20.69019075 | 16.91514563 | 22.38196791 | 20.09928857 | 20.61275082 | 14.16984238 | 14.53315865 | 14.83814392 | 15.76031794 | 20.79706411 | 16.883467 | 19.26034339 | 18.60329467 | 25.66561009 | 23.7377085 | 23.41574401 | 19.86862073 |
| ENSMUSG00000027861 | Casq2 | 3085 | 0 | 0 | 0.031360473 | 0 | 0 | 0 | 0 | 0 | 0 | 0 | 0 | 0.072370389 | 0.255854352 | 0.240876952 | 0.480796114 | 0.012411866 | 0.12423435 | 0.12481807 |
| ENSMUSG00000027863 | Cd2 | 2110 | 0.181301699 | 0.031576203 | 0 | 0.194336208 | 0 | 0.023484708 | 2.71319154 | 0.923133897 | 0.858865732 | 0.78212067 | 5.637897944 | 0.238076286 | 2.244485336 | 1.562810524 | 1.171608224 | 0.653299463 | 0 | 0.677837334 |
| ENSMUSG00000027864 | Ptgfrn | 6849 | 1.706661324 | 1.814237048 | 1.638583595 | 1.337095913 | 1.658509853 | 1.273365617 | 0.739152671 | 1.092964064 | 1.226755616 | 0.812349684 | 0.864957636 | 0.651956929 | 1.577411284 | 0.678114869 | 0.860245655 | 1.079002369 | 0.884151688 | 0.714821215 |
| ENSMUSG00000027865 | Gdap2 | 41108 | 0.857175998 | 1.05186671 | 1.080249608 | 1.122733724 | 1.170865128 | 1.048722346 | 0.990955935 | 1.081442941 | 0.78048788 | 0.872863802 | 1.287697139 | 0.985749028 | 0.984046224 | 1.057498925 | 1.307969992 | 1.234189326 | 1.144903488 | 1.36224751 |
| ENSMUSG00000027867 | Spag17 | 8598 | 0.084041404 | 0.061991894 | 0.095644338 | 0.079485423 | 0.016794112 | 0.017289858 | 0 | 0.084398191 | 0.033531706 | 0.021935661 | 0.02222608 | 0.006491703 | 0.063113642 | 0.124239824 | 0.028751958 | 0.031174023 | 0.057948576 | 0 |
| ENSMUSG00000027868 | Tbx15 | 3737 | 0.432217471 | 0.418974051 | 0.284778611 | 0.12191877 | 0.128798297 | 0.01326003 | 0.746975664 | 0 | 0.022042556 | 0 | 0.051137231 | 0.492886504 | 0 | 0 | 0.25358196 | 0 | 0 | 0 |
| ENSMUSG00000027869 | Hsd3b6 | 2964 | 0 | 0 | 0 | 0 | 0 | 0 | 0.015962442 | 0.06442713 | 0 | 0 | 0 | 0.018831195 | 0 | 0 | 0.069503299 | 0 | 0 | 0.055677234 |
| ENSMUSG00000027870 | Hao2 | 1984 | 0 | 0 | 0 | 0 | 0 | 0 | 0 | 0 | 0 | 0 | 0 | 0 | 0 | 0 | 0 | 0 | 0 | 0 |
| ENSMUSG00000027871 | Hsd3b1 | 3016 | 0 | 0 | 0 | 0 | 0 | 0 | 0 | 0 | 0 | 0 | 0 | 0 | 0 | 0 | 0 | 0 | 0 | 0 |
| ENSMUSG00000027875 | Hmgcs2 | 3274 | 5.855172385 | 7.132663057 | 6.456699073 | 6.99975727 | 6.292139074 | 6.931934019 | 1.343945981 | 4.129538928 | 2.742413084 | 2.70749304 | 3.38539718 | 5.301976501 | 1.250625881 | 3.560618795 | 3.058026292 | 3.485217167 | 2.142245673 | 2.637883248 |
| ENSMUSG00000027876 | Reg4 | 1015 | 0 | 0.098461756 | 0 | 0.044887728 | 0 | 0.048820427 | 0.512748245 | 0.150511931 | 0 | 0 | 0.470689246 | 0 | 0 | 0.457577216 | 0 | 0.528146309 | 0.302079187 | 0 |
| ENSMUSG00000027878 | Notch2 | 10467 | 0.158374115 | 0.28962199 | 0.198725691 | 0.226347025 | 0.114961125 | 0.468684496 | 0.479138623 | 0.346639747 | 0.373814751 | 0.531554694 | 0.237345738 | 0.677232172 | 0.768233736 | 0.585709328 | 0.531404418 | 0.351189291 | 0.486996989 | 0.494015098 |
| ENSMUSG00000027879 | Sec22b | 3574 | 32.63408031 | 31.48599795 | 30.38572346 | 33.65449274 | 32.87353824 | 30.5579812 | 32.08895739 | 35.27507564 | 30.06592603 | 34.69679619 | 28.32544404 | 34.12345348 | 32.78212186 | 36.25601674 | 32.68226384 | 36.59784972 | 41.03937836 | 33.15322213 |
| ENSMUSG00000027880 | 4930443G12R | 1998 | 0 | 0 | 0 | 0 | 0 | 0 | 0 | 0 | 0 | 0 | 0 | 0 | 0 | 0 | 0 | 0 | 0 | 0 |
| ENSMUSG00000027881 | Prpf38b | 6690 | 13.76811905 | 14.39575145 | 14.02760073 | 15.09167029 | 12.87115268 | 15.6213326 | 17.42577592 | 18.30837586 | 20.13152564 | 15.24468926 | 17.44607228 | 19.00569198 | 20.24894542 | 18.69565431 | 18.13730674 | 20.8337535 | 20.55526955 | 21.72407473 |
| ENSMUSG00000027882 | Stxbp3a | 5869 | 38.71020035 | 42.07108058 | 48.85157853 | 40.29773064 | 42.72743607 | 45.44092897 | 28.74714825 | 30.59166531 | 35.63556433 | 29.66099786 | 32.66670785 | 35.44470514 | 31.42818857 | 30.89414162 | 38.60408176 | 42.8771293 | 43.04769964 | 32.21439765 |
| ENSMUSG00000027883 | Gpsm2 | 3772 | 0.405669761 | 1.192269544 | 1.15419346 | 0.760961239 | 0.676296913 | 0.906452444 | 0.852932706 | 0.901146292 | 0.240218279 | 1.787526661 | 0.823269433 | 0.739868274 | 1.281688201 | 0.664894146 | 1.332605996 | 1.664809017 | 1.41234233 | 4.156308435 |
| ENSMUSG00000027884 | Clcc1 | 3949 | 18.10425578 | 19.41916498 | 20.02803802 | 20.80186451 | 22.01221933 | 20.85506285 | 19.64871953 | 17.0023407 | 22.45494263 | 20.60828408 | 22.57484744 | 19.7595078 | 16.77709924 | 19.86428554 | 20.19909147 | 17.84115412 | 21.10906451 | 21.03412285 |
| ENSMUSG00000027886 | 1700013F07R | 1446 | 0.088185013 | 0.046075926 | 0.133813362 | 0.094524988 | 0 | 0.102806501 | 0 | 0.079237349 | 0 | 0 | 0.066078781 | 0 | 0 | 0 | 0 | 0 | 0 | 0 |
| ENSMUSG00000027887 | Sypl2 | 4879 | 0 | 0 | 0.00991464 | 0 | 0 | 0 | 0.126063707 | 0.086107077 | 0.00844159 | 0 | 0.029375871 | 0.13727976 | 0 | 0 | 0.143559428 | 0.047088265 | 0 | 0 |

| ENSMUSG00000027889 | Ampd2 | 6110 | 5.210536308 | 5.163225994 | 5.898245518 | 6.46504508 | 5.348866804 | 5.798724142 | 7.356308508 | 6.794622163 | 5.736452523 | 8.095104373 | 6.09893087 | 6.431133627 | 2.406041137 | 3.200157249 | 2.569196857 | 3.114636969 | 3.073631012 | 3.09707573 |
| --- | --- | --- | --- | --- | --- | --- | --- | --- | --- | --- | --- | --- | --- | --- | --- | --- | --- | --- | --- | --- |
| ENSMUSG00000027890 | Gstm4 | 1859 | 14.74762697 | 13.49360374 | 12.56827066 | 14.16583304 | 12.35014932 | 11.86173558 | 23.87266963 | 19.39409783 | 21.60131946 | 17.22180071 | 18.91466888 | 20.38667827 | 23.5116077 | 18.2378611 | 18.26255086 | 19.40277148 | 20.03935481 | 18.76050224 |
| ENSMUSG00000027893 | Ahcyl1 | 7685 | 92.27245955 | 99.96035663 | 105.2132153 | 102.6887247 | 103.7419888 | 104.9408899 | 104.8943947 | 117.0173464 | 107.2617991 | 125.7024864 | 117.227377 | 117.0131607 | 92.55913015 | 84.13722637 | 113.5522386 | 101.7179896 | 125.4718678 | 106.3604836 |
| ENSMUSG00000027894 | Slc6a17 | 12212 | 1.5314672 | 1.83040878 | 1.525041696 | 1.413989175 | 1.332180658 | 1.692064355 | 1.712430731 | 1.845194679 | 2.438409015 | 1.420853161 | 2.163409109 | 2.011045819 | 1.252284176 | 0.916559537 | 1.035774456 | 2.103913983 | 2.043106722 | 0.761262614 |
| ENSMUSG00000027895 | Kcnc4 | 3009 | 1.214837201 | 1.948510928 | 1.01280572 | 1.287035151 | 2.25543411 | 2.058521667 | 4.654221656 | 5.305558061 | 4.777033566 | 5.484461994 | 4.810838291 | 5.935863087 | 7.148127595 | 7.547743015 | 5.107404569 | 4.619305579 | 4.763720519 | 4.972574619 |
| ENSMUSG00000027896 | Slc16a4 | 3989 | 1.427849984 | 1.068952234 | 0.812490984 | 1.336335469 | 0.880829889 | 1.602482485 | 1.150496329 | 0.880847586 | 0.722751593 | 0.839232873 | 0.790460581 | 1.595132999 | 1.063561014 | 0.884871056 | 0.371836554 | 1.171084007 | 1.162567542 | 1.985788761 |
| ENSMUSG00000027900 | Dram2 | 2929 | 49.81914281 | 49.79305254 | 55.97060238 | 47.17878029 | 52.07581635 | 54.93264798 | 37.10386599 | 55.62608012 | 59.95879249 | 53.84742315 | 50.92298408 | 49.18409871 | 48.47294569 | 46.36480421 | 52.44090145 | 48.69665844 | 55.37619955 | 51.60977305 |
| ENSMUSG00000027901 | Dennd2d | 4532 | 9.575857177 | 7.402048649 | 8.20812993 | 9.088081218 | 8.783120228 | 8.594097218 | 8.309993916 | 8.435700531 | 12.23235883 | 8.812145997 | 9.413732992 | 8.325548965 | 8.392521673 | 7.880737688 | 7.654834523 | 8.820695968 | 7.721074376 | 7.173517668 |
| ENSMUSG00000027902 | BC051070 | 1653 | 0.02571396 | 0 | 0 | 0.248063762 | 0 | 0 | 0 | 0 | 0 | 0.057048643 | 0 | 0 | 0 | 0 | 0 | 0 | 0 | 0 |
| ENSMUSG00000027905 | Ddx20 | 4994 | 3.855595663 | 4.415926306 | 4.504143295 | 3.932080518 | 4.365991474 | 4.336112249 | 3.931670355 | 3.885010096 | 2.968991925 | 5.136159906 | 4.0561839 | 4.291793038 | 3.556165442 | 5.170787463 | 5.898901174 | 4.224694569 | 5.870968588 | 4.053534496 |
| ENSMUSG00000027907 | S100a11 | 508 | 1158.182375 | 1068.242218 | 1042.601937 | 969.1587502 | 1160.945789 | 1197.459365 | 1050.844402 | 922.3314817 | 857.6200073 | 898.6478522 | 924.5583008 | 979.0814356 | 1130.069924 | 1176.735923 | 872.9376891 | 1092.412939 | 1026.963688 | 1161.363523 |
| ENSMUSG00000027908 | Tchhl1 | 2063 | 0 | 0.016147792 | 0 | 0.198763645 | 0 | 0 | 0 | 0 | 0 | 0 | 0 | 0 | 0 | 0 | 0 | 0 | 0 | 0 |
| ENSMUSG00000027912 | Lce1m | 875 | 0 | 0 | 0 | 0 | 0 | 0 | 0 | 0 | 0 | 0 | 0 | 0 | 0 | 0 | 0 | 0 | 0 | 0 |
| ENSMUSG00000027913 | Crct1 | 1120 | 0 | 0 | 0 | 0 | 0 | 0 | 0 | 0 | 0 | 0 | 0 | 0 | 0.044046355 | 0 | 0 | 0 | 0 | 0 |
| ENSMUSG00000027919 | Lce1g | 923 | 0 | 0 | 0 | 0 | 0 | 0 | 0 | 0 | 0 | 0 | 0 | 0 | 0 | 0 | 0 | 0 | 0 | 0 |
| ENSMUSG00000027923 | Lce1b | 645 | 0 | 0 | 0 | 0 | 0 | 0 | 0 | 0 | 0 | 0 | 0 | 0 | 0 | 0 | 0 | 0 | 0 | 0 |
| ENSMUSG00000027925 | Sprr2j-ps | 671 | 0 | 0 | 0 | 0 | 0 | 0 | 0 | 0 | 0 | 0 | 0 | 0 | 0 | 0 | 0 | 0 | 0 | 0 |
| ENSMUSG00000027927 | Lelp1 | 868 | 0 | 0 | 0.111459747 | 0 | 0.277257625 | 0 | 0 | 0 | 0 | 0 | 0 | 0 | 0 | 0 | 0 | 0 | 0 | 0 |
| ENSMUSG00000027931 | Npr1 | 5233 | 3.630768906 | 4.564369416 | 4.945507116 | 5.014936277 | 4.608082124 | 4.819862677 | 2.468251738 | 3.525115682 | 2.943580526 | 4.225813076 | 2.446720595 | 2.762517984 | 6.042759215 | 4.17135889 | 5.495640362 | 6.709819795 | 5.8738181 | 6.20205866 |
| ENSMUSG00000027932 | Slc27a3 | 2752 | 3.258936098 | 2.602569861 | 2.548750691 | 2.963454555 | 3.235612601 | 2.44882695 | 1.03152643 | 1.304537001 | 1.990482048 | 0.856662487 | 1.440887192 | 1.115501978 | 2.635098779 | 1.637019069 | 1.706750498 | 1.794872222 | 0.93308935 | 0.199887742 |
| ENSMUSG00000027933 | Ints3 | 4945 | 15.86745297 | 15.7638367 | 16.3364602 | 19.71701413 | 14.22057441 | 14.48002023 | 20.38894212 | 13.50057015 | 18.20702193 | 15.71372274 | 16.77195711 | 16.37786176 | 19.60307734 | 14.61415571 | 21.71309498 | 17.99542398 | 20.21334324 | 20.39063552 |
| ENSMUSG00000027935 | Rab13 | 1642 | 5.487879 | 3.124351833 | 4.124417935 | 4.939016986 | 4.015879137 | 3.198897539 | 5.301786189 | 5.326467814 | 4.088551808 | 5.857943645 | 3.258706059 | 3.807158471 | 3.755474823 | 8.626946805 | 4.767536918 | 5.060329967 | 2.35746406 | 5.561212982 |
| ENSMUSG00000027936 | Crtc2 | 4180 | 17.12409484 | 15.55664342 | 19.61558229 | 18.40971385 | 16.82314366 | 18.43409108 | 15.52942476 | 19.90938872 | 14.11968351 | 17.30363129 | 17.01840028 | 15.60969128 | 17.159954 | 17.48875444 | 17.20017106 | 18.04605181 | 20.25425595 | 18.87154523 |
| ENSMUSG00000027937 | Jtb | 1536 | 74.02431372 | 68.44758734 | 74.51287293 | 67.51102663 | 70.44307578 | 68.39309582 | 79.84014569 | 74.02264451 | 80.79099739 | 73.97994456 | 75.83030519 | 74.89328163 | 90.73090254 | 82.30521216 | 74.46322862 | 81.09332414 | 70.4894458 | 83.87456246 |
| ENSMUSG00000027938 | Creb3l4 | 2019 | 3.578940032 | 3.200941792 | 3.713668749 | 3.023863269 | 2.264751237 | 2.773382315 | 1.804396373 | 2.496977275 | 3.6923028 | 2.709005239 | 2.082316169 | 1.852228526 | 2.076876161 | 2.875438793 | 2.326388 | 1.687897008 | 1.575573374 | 2.724571903 |
| ENSMUSG00000027939 | Nup210l | 5641 | 0.075350427 | 0.035432967 | 0.034301387 | 0.080767673 | 0.102390194 | 0 | 0.176133001 | 0.135410042 | 0.219038375 | 0.125378576 | 0.093161592 | 0.217682074 | 0.323574001 | 0.238765916 | 0.277549924 | 0.244364805 | 0.251386808 | 0.390066348 |
| ENSMUSG00000027940 | Tpm3 | 4925 | 47.18290301 | 52.48894602 | 48.3047761 | 48.73412824 | 50.14515743 | 51.30342914 | 42.48054136 | 46.15658459 | 41.96425098 | 42.48828854 | 42.63369391 | 43.56454964 | 39.22513463 | 39.9089092 | 37.80504184 | 37.00777487 | 41.47800254 | 39.86345216 |
| ENSMUSG00000027942 | 4933434E20R | 3764 | 30.61411589 | 25.98476552 | 27.70811144 | 26.52078856 | 29.98654649 | 29.59472505 | 27.26386838 | 24.11884711 | 28.0777363 | 25.90532793 | 26.57831112 | 24.42305959 | 27.91630813 | 25.10991441 | 25.70171446 | 28.9722768 | 30.59790707 | 28.73217423 |
| ENSMUSG00000027944 | Hax1 | 1364 | 40.41731181 | 32.8488583 | 34.40053112 | 34.70522365 | 32.88779535 | 33.85861268 | 39.09192753 | 33.09634871 | 36.11368977 | 31.97536695 | 35.27080879 | 28.3579576 | 41.19432095 | 36.05886257 | 36.21749671 | 38.51518574 | 36.05032179 | 38.1918065 |
| ENSMUSG00000027947 | Il6ra | 3333 | 1.46657523 | 1.46924556 | 1.654540192 | 1.107244102 | 0.996430463 | 0.787967261 | 0.39746625 | 0.469813531 | 0.308929761 | 0.254639262 | 0.329680182 | 0.083731867 | 0.429230603 | 0.139346197 | 0.531553226 | 0.482509904 | 0.471460598 | 0.396105179 |
| ENSMUSG00000027950 | Chrnb2 | 5378 | 0 | 0 | 0 | 0.076245705 | 0.044748906 | 0.036855882 | 0.351897947 | 0.078117595 | 0.176141663 | 0.061371313 | 0.044417031 | 0.083028133 | 0.220149873 | 0.172718808 | 0.076611298 | 0.02135958 | 0.078391459 | 0 |
| ENSMUSG00000027951 | Adar | 6769 | 6.059609228 | 6.845654581 | 4.795189669 | 5.451979009 | 6.428018761 | 6.032124755 | 5.682553992 | 5.817161601 | 5.488290326 | 4.137615266 | 3.938310952 | 4.955711806 | 5.837622358 | 6.799540642 | 5.946804566 | 6.216779071 | 5.673356404 | 5.981194054 |
| ENSMUSG00000027952 | Pmvk | 1632 | 43.15629698 | 39.4161757 | 41.73402619 | 36.9066793 | 38.54682857 | 36.92164463 | 44.44260833 | 39.71354588 | 50.67556596 | 45.38833016 | 47.97987558 | 39.63869665 | 55.10544436 | 45.96029481 | 50.71931727 | 55.04274814 | 48.7767884 | 44.05447456 |
| ENSMUSG00000027953 | Slc50a1 | 1566 | 52.00505573 | 48.13989758 | 43.18403285 | 42.79712402 | 40.47876339 | 49.39451923 | 51.30072083 | 52.55723319 | 49.76046477 | 45.61514396 | 52.47313447 | 53.53456271 | 54.1517023 | 48.78705221 | 52.38333191 | 51.07859442 | 44.34690293 | 57.46800677 |
| ENSMUSG00000027954 | Efna1 | 2692 | 17.32101713 | 22.52209045 | 21.40151362 | 20.9188153 | 21.08006612 | 22.23614493 | 20.75641672 | 21.19593209 | 22.36801116 | 25.01159148 | 23.08886366 | 27.67975838 | 23.38318219 | 16.56252745 | 24.64134658 | 36.61219296 | 25.72645561 | 31.55054264 |
| ENSMUSG00000027955 | Fam198b | 7170 | 0 | 0.041815348 | 0 | 0.006354399 | 0.00671296 | 0.034555602 | 0.0461909 | 0.106533898 | 0.045954271 | 0.098641639 | 0 | 0.365876728 | 0 | 0.006477558 | 0.057463816 | 0 | 0 | 0.038360604 |
| ENSMUSG00000027956 | Tmem144 | 3460 | 6.670609995 | 5.045074153 | 4.865314614 | 4.727287548 | 4.228932022 | 4.625876574 | 4.444110015 | 3.609513175 | 3.809157527 | 3.175177416 | 3.465755658 | 3.323129044 | 3.065422605 | 4.389368953 | 3.953444091 | 3.242528315 | 2.97970343 | 4.864967242 |
| ENSMUSG00000027957 | Slc35a3 | 10727 | 4.762862085 | 5.459501076 | 5.361809228 | 5.105283424 | 5.842058788 | 5.857450003 | 4.935479418 | 6.205775836 | 5.766956901 | 5.511977429 | 5.834361481 | 6.20231843 | 4.212551154 | 4.407577231 | 4.954787648 | 4.483360016 | 5.83094216 | 4.169143634 |
| ENSMUSG00000027959 | Sass6 | 4392 | 2.409787985 | 1.486640999 | 1.53094734 | 1.888003203 | 1.709603846 | 1.782634769 | 2.272990723 | 1.486999274 | 1.753615311 | 1.191650287 | 1.414103962 | 1.461475682 | 1.415259922 | 1.533331665 | 1.41653801 | 2.266748161 | 1.090798523 | 1.402782321 |
| ENSMUSG00000027961 | Lrrc39 | 5366 | 0.158424063 | 0.074497713 | 0.027044463 | 0.084906903 | 0.170426118 | 0.120049485 | 0.167525326 | 0.071174809 | 0.038377298 | 0.131804053 | 0.035613089 | 0.145624166 | 0.101127672 | 0.060586771 | 0.023034787 | 0.142715644 | 0.085709199 | 0.071759923 |
| ENSMUSG00000027962 | Vcam1 | 4907 | 0.199229478 | 0.346231426 | 0.80836142 | 0.575664306 | 0.55910325 | 0.454426943 | 0.038567499 | 0.630443162 | 0.193048678 | 0.442007815 | 0.029208249 | 0.580109801 | 0.120640515 | 0.056789184 | 0.016792972 | 0.21849134 | 0.124968565 | 0.123313669 |

| ENSMUSG00000027963 | Extl2 |  | 3794 | 11.41612398 | 9.842845129 | 11.38575714 | 10.37552512 | 10.65651447 | 10.94496329 | 11.70970098 | 10.69065138 | 12.37549498 | 12.57683492 | 9.721209263 | 12.71078874 | 12.27446756 | 16.33010347 | 11.92390837 | 14.25048436 | 14.21325771 | 9.87380118 |
| --- | --- | --- | --- | --- | --- | --- | --- | --- | --- | --- | --- | --- | --- | --- | --- | --- | --- | --- | --- | --- | --- |
| ENSMUSG00000027965 | Olfm3 |  | 4930 | 0 | 0 | 0 | 0 | 0 | 0 | 0 | 0 | 0 | 0 | 0 | 0 | 0 | 0 | 0 | 0 | 0 | 0 |
| ENSMUSG00000027966 | Col11a1 |  | 8860 | 0 | 0 | 0 | 0 | 0 | 0 | 0 | 0 | 0 | 0 | 0 | 0 | 0 | 0 | 0 | 0 | 0 | 0 |
| ENSMUSG00000027967 | Neurog2 |  | 2224 | 0 | 0 | 0 | 0 | 0 | 0 | 0 | 0 | 0 | 0 | 0 | 0 | 0 | 0 | 0 | 0 | 0 | 0 |
| ENSMUSG00000027968 | Larp7 |  | 1867 | 15.11699887 | 12.77559307 | 11.47802568 | 11.9332355 | 11.7558421 | 13.53609755 | 9.477740717 | 14.27868071 | 11.51545861 | 12.87994572 | 13.6134322 | 11.33054961 | 15.56320261 | 13.01031479 | 13.35133439 | 10.15203571 | 12.3169674 | 11.07842749 |
| ENSMUSG00000027971 | Ndst4 |  | 8184 | 0 | 0.00407049 | 0 | 0 | 0 | 0 | 0 | 0 | 0 | 0 | 0 | 0 | 0 | 0 | 0 | 0 | 0 | 0 |
| ENSMUSG00000027973 | 1700006A11R |  | 2336 | 0 | 0 | 0 | 0 | 0 | 0 | 0 | 0 | 0 | 0 | 0 | 0 | 0 | 0 | 0 | 0 | 0 | 0 |
| ENSMUSG00000027977 | Ndst3 |  | 8874 | 0 | 0 | 0 | 0 | 0 | 0 | 0 | 0 | 0 | 0 | 0 | 0 | 0 | 0 | 0 | 0 | 0 | 0 |
| ENSMUSG00000027978 | Prss12 |  | 2596 | 0.163733344 | 0.256647875 | 0.391311301 | 0.315908628 | 0.241030434 | 0.13361677 | 0.054675669 | 0 | 0.111057631 | 0.326930916 | 0 | 0.064501921 | 0.342054896 | 0 | 0.349165728 | 0.014749849 | 0.324799126 | 0.063569846 |
| ENSMUSG00000027981 | Rnpc3 |  | 7115 | 7.091165699 | 6.479978299 | 6.622040557 | 7.197556407 | 7.617223619 | 5.515919184 | 10.16075522 | 9.731950184 | 10.55277839 | 7.952332243 | 10.41447092 | 10.14331015 | 7.190048941 | 7.38274952 | 9.230538312 | 8.772127908 | 9.345906553 | 7.035599033 |
| ENSMUSG00000027983 | Cyp2u1 |  | 2326 | 1.297449483 | 0.773386194 | 0.873468734 | 1.07732478 | 1.013957119 | 0.681722904 | 0.183067115 | 0.673210876 | 0 | 1.317624984 | 1.211832567 | 0.479928311 | 1.357370037 | 0.998368173 | 0.425123535 | 0.790175905 | 1.03807253 | 0.141897954 |
| ENSMUSG00000027984 | Hadh |  | 1840 | 89.30707094 | 80.2587283 | 79.76374516 | 84.68411502 | 76.43558748 | 81.89666457 | 124.2986358 | 102.4345141 | 101.7575547 | 105.2690701 | 111.6739654 | 107.5664889 | 101.7202686 | 99.45092626 | 94.98750005 | 86.75735992 | 94.27435858 | 98.92670334 |
| ENSMUSG00000027985 | Lef1 |  | 8641 | 23.43908851 | 24.86997808 | 26.64714784 | 27.43364352 | 27.51111804 | 25.73691335 | 27.99009544 | 27.7570058 | 27.29720817 | 31.01546086 | 28.66718663 | 28.84767378 | 30.27510211 | 33.07683298 | 35.25567692 | 34.39552073 | 30.6752308 | 37.59157218 |
| ENSMUSG00000027993 | Trim2 |  | 18905 | 11.48910074 | 12.35951547 | 12.33326719 | 13.27668835 | 12.80887109 | 12.62080995 | 12.28052449 | 10.99407849 | 13.01716116 | 13.17625841 | 12.84023084 | 12.01047952 | 14.57646601 | 13.33501753 | 12.66231366 | 16.19325079 | 16.45973047 | 13.05611541 |
| ENSMUSG00000027994 | Ccdc109b |  | 1514 | 0.786093084 | 1.100161632 | 2.012901197 | 1.023167442 | 1.367022931 | 1.505565221 | 1.812506855 | 1.135177086 | 0.924928359 | 1.401440982 | 1.041330006 | 1.880184143 | 3.29096674 | 7.331662408 | 4.272552361 | 3.515452067 | 3.164324381 | 5.413709975 |
| ENSMUSG00000027995 | Tlr2 |  | 3010 | 0 | 0.320954795 | 0.369631627 | 0.272458072 | 0.799533616 | 0.395104852 | 0.298651462 | 0.735933335 | 0.547329113 | 0.093988113 | 0 | 0.129803866 | 0 | 0.648057033 | 0.232699817 | 0.445239621 | 0.598450483 | 0.018275451 |
| ENSMUSG00000027996 | Sfrp2 |  | 2626 | 0 | 0 | 0 | 0 | 0 | 0 | 0 | 0 | 0 | 0 | 0 | 0 | 0 | 0 | 0 | 0.189557462 | 0 | 0 |
| ENSMUSG00000027997 | Casp6 |  | 1716 | 52.68561154 | 49.93051511 | 52.48922699 | 47.68510235 | 53.88194951 | 50.0725175 | 47.86410875 | 54.1504167 | 62.28380443 | 51.46460793 | 50.5033652 | 55.197657 | 46.42834872 | 46.49821803 | 46.84396011 | 46.25666034 | 48.17588719 | 50.32884473 |
| ENSMUSG00000027998 | Plrg1 |  | 2923 | 17.36270276 | 21.89328423 | 19.92532691 | 21.0269753 | 21.70300219 | 17.95290621 | 17.41650446 | 19.52085159 | 16.34497375 | 15.92122618 | 21.34591029 | 14.87526553 | 20.55637196 | 19.46425147 | 17.94374979 | 18.09077278 | 21.73965113 | 15.50718575 |
| ENSMUSG00000027999 | Pla2g12a |  | 1794 | 39.78048526 | 44.71429726 | 40.55395187 | 38.62784194 | 41.39774706 | 45.38190706 | 51.29496127 | 53.3288562 | 45.66331095 | 44.10193984 | 40.71789939 | 53.10888296 | 58.79132616 | 71.37477644 | 50.93927033 | 40.2969157 | 42.70583473 | 51.17625369 |
| ENSMUSG00000028001 | Fga |  | 2820 | 0.150727575 | 0.259887827 | 0.240152278 | 0.080781994 | 0.017068058 | 0 | 2.348856401 | 5.322558208 | 1.679591955 | 0.484883119 | 0.897898156 | 4.690890792 | 1.277031901 | 3.952688288 | 2.060077795 | 0.149360525 | 0.190272396 | 2.633414684 |
| ENSMUSG00000028003 | Lrat |  | 5349 | 1.819720567 | 0.678838188 | 0.904347174 | 0.562166559 | 0.269949095 | 1.315477317 | 0.141522315 | 0.114241622 | 0 | 0.017629726 | 0.241152342 | 0.062608707 | 0.101449073 | 0.408089756 | 0.616213212 | 0.250546132 | 0.150467795 | 0 |
| ENSMUSG00000028004 | Npy2r |  | 4443 | 0 | 0 | 0 | 0 | 0 | 0 | 0.127785763 | 0.206306022 | 0.528388791 | 0.212247145 | 0 | 0.364315601 | 0.08882632 | 0.083626536 | 0 | 0 | 0 | 0 |
| ENSMUSG00000028005 | Gucy1b3 |  | 3237 | 0 | 0 | 0 | 0 | 0 | 0 | 0 | 0 | 0 | 0 | 0 | 0 | 0.045720035 | 0 | 0 | 0 | 0 | 0 |
| ENSMUSG00000028007 | Snx7 |  | 2667 | 13.19620763 | 11.11678885 | 12.51508659 | 11.36036539 | 11.64045398 | 14.36230336 | 10.50210196 | 9.737845383 | 9.698212185 | 9.635220576 | 9.279125794 | 8.538729035 | 10.95031685 | 8.289233445 | 12.40526786 | 12.28974876 | 11.9131984 | 15.59313261 |
| ENSMUSG00000028008 | Accn5 |  | 2294 | 0 | 0 | 0 | 0 | 0 | 0 | 0 | 0 | 0 | 0 | 0 | 0 | 0 | 0 | 0 | 0 | 0 | 0 |
| ENSMUSG00000028009 | 1700061I17Ri | k | 1953 | 0 | 0 | 0 | 0.093314991 | 0 | 0 | 0 | 0.215113378 | 0 | 0.04828541 | 0.073387033 | 0.028579448 | 0 | 0.023780895 | 0 | 0 | 0.13737024 | 0.056332931 |
| ENSMUSG00000028010 | Gar1 |  | 1545 | 16.20423863 | 16.66722798 | 13.6823513 | 11.3533994 | 14.51746047 | 15.84404556 | 13.74976883 | 14.68368092 | 12.52923133 | 10.89501687 | 10.63727231 | 13.33073106 | 10.72849463 | 17.01446826 | 10.21371903 | 12.81310293 | 13.02349895 | 11.50028574 |
| ENSMUSG00000028011 | Tdo2 |  | 4066 | 0.08363045 | 0 | 0.035691242 | 0.08964298 | 0.059188298 | 0.012187096 | 0.116361729 | 0.037572457 | 0.141812892 | 0 | 0.199747736 | 0.068637067 | 0 | 0.0228451 | 0 | 0.094172669 | 0 | 0 |
| ENSMUSG00000028012 | Rrh |  | 2119 | 0.060177219 | 0.06288418 | 0.479397894 | 0.516028817 | 0.181715616 | 0.210464654 | 0 | 0 | 0 | 0 | 0 | 0 | 0 | 0 | 0.233326414 | 0 | 0 | 0 |
| ENSMUSG00000028013 | Ppa2 |  | 2094 | 78.19003728 | 63.49176735 | 63.18128248 | 62.31462797 | 66.405505 | 58.37946215 | 65.34301216 | 56.52256688 | 63.76632479 | 61.60664953 | 59.63884502 | 54.93604613 | 65.32827441 | 57.35645176 | 62.96321817 | 64.20168223 | 61.25984515 | 66.01618204 |
| ENSMUSG00000028015 | Ctso |  | 4697 | 20.16213161 | 20.64590911 | 19.55744819 | 22.32947074 | 23.65094312 | 21.26853543 | 14.1424316 | 14.77445075 | 16.0203884 | 18.32021151 | 14.2907849 | 17.87241996 | 14.22086779 | 16.58223005 | 14.72799922 | 16.13308751 | 18.16358035 | 16.88804171 |
| ENSMUSG00000028016 | Ints12 |  | 4866 | 19.68899851 | 14.69847593 | 18.6097922 | 16.97537472 | 17.83433177 | 14.67437096 | 15.01248998 | 12.98981216 | 13.55955574 | 15.54248418 | 12.16464726 | 13.52377028 | 12.31777219 | 16.5980822 | 15.64744663 | 16.60361316 | 14.65787886 | 12.525707 |
| ENSMUSG00000028017 | Egf |  | 5725 | 0.274705941 | 0.384043846 | 0.456274347 | 0.628702621 | 0.596919927 | 0.363530971 | 1.842921817 | 1.64110585 | 1.143870045 | 0.790649347 | 1.109881131 | 1.052941757 | 1.34424089 | 2.29583873 | 1.28822332 | 0.541753572 | 0.917153306 | 1.585415303 |
| ENSMUSG00000028018 | Gstcd |  | 5163 | 0.987918095 | 0.761363842 | 1.396020923 | 1.226609561 | 0.755120243 | 1.132524223 | 0.339060453 | 0.55479957 | 0.845587966 | 1.086758413 | 1.313973291 | 1.005395433 | 1.261245997 | 0.728640534 | 0.550630904 | 1.023456096 | 1.11348916 | 1.310501671 |
| ENSMUSG00000028019 | Pdgfc |  | 5464 | 8.028063994 | 10.43161823 | 10.37587437 | 11.68210526 | 11.45159238 | 10.77391059 | 6.58948548 | 7.891512137 | 8.766456411 | 7.775033608 | 7.694356417 | 6.578566381 | 5.922719204 | 6.035011355 | 6.477330987 | 3.60200809 | 5.667578312 | 5.990193723 |
| ENSMUSG00000028020 | Glrb |  | 3622 | 8.566752761 | 9.703231199 | 10.73780188 | 4.541009665 | 5.860347415 | 9.344151583 | 6.008788599 | 6.695796667 | 6.947808153 | 4.517198794 | 5.579460916 | 6.117840433 | 4.167743425 | 6.821715762 | 7.792121973 | 2.484343661 | 3.110969434 | 6.393935376 |
| ENSMUSG00000028023 | Pitx2 |  | 4478 | 0 | 0 | 0 | 0 | 0 | 0 | 0 | 0 | 0 | 0 | 0 | 0 | 0 | 0 | 0 | 0 | 0 | 0 |
| ENSMUSG00000028024 | Enpep |  | 5342 | 0 | 0.043652239 | 0 | 0.093817201 | 0 | 0 | 0 | 0 | 0 | 0 | 0 | 0.198520702 | 0 | 0 | 0 | 0 | 0.107617831 | 0 |
| ENSMUSG00000028028 | Alpk1 |  | 4169 | 2.232821672 | 2.868632532 | 1.926122821 | 3.12555977 | 2.666940362 | 2.472287979 | 2.065461157 | 3.050628448 | 2.736547101 | 2.793529313 | 3.277437785 | 2.222451425 | 3.538077061 | 6.182890022 | 2.539889629 | 3.113580213 | 3.45662932 | 3.166751167 |
| ENSMUSG00000028029 | Aimp1 |  | 1115 | 39.26487113 | 41.67846407 | 38.1782123 | 30.27868507 | 34.62045093 | 39.46441921 | 43.15425513 | 32.54061191 | 41.37120866 | 32.01173306 | 37.87718681 | 27.88280185 | 47.82762563 | 39.2796183 | 35.65874568 | 36.19578493 | 38.77315059 | 38.58037799 |

| ENSMUSG00000028030 | Tbck | 4081 | 2.541353333 | 2.489691923 | 2.323257032 | 2.154687958 | 2.146535189 | 2.076333605 | 4.069284564 | 2.451950363 | 2.018452132 | 2.95775056 | 1.955015454 | 2.721714495 | 2.768196289 | 2.230590818 | 1.484104072 | 3.509112269 | 2.441763586 | 3.35635079 |
| --- | --- | --- | --- | --- | --- | --- | --- | --- | --- | --- | --- | --- | --- | --- | --- | --- | --- | --- | --- | --- |
| ENSMUSG00000028031 | Dkk2 | 3690 | 0.057595089 | 0.108334615 | 0.209749725 | 0.049388666 | 0.456535861 | 0.161147101 | 0.012821864 | 0 | 0 | 0 | 0 | 0 | 0 | 0 | 0 | 0 | 0 | 0 |
| ENSMUSG00000028032 | Papss1 | 2587 | 78.0767671 | 81.83356891 | 93.94225609 | 86.03235469 | 88.74730417 | 81.98132962 | 78.11072737 | 88.10678684 | 82.15014354 | 80.06688806 | 85.1158808 | 74.60787059 | 76.21943307 | 80.03391634 | 77.7526076 | 66.76804406 | 75.65999161 | 68.12878931 |
| ENSMUSG00000028033 | Kcnq5 | 12414 | 0 | 0 | 0 | 0 | 0 | 0 | 0 | 0 | 0 | 0 | 0 | 0 | 0.047686725 | 0 | 0 | 0 | 0 | 0 |
| ENSMUSG00000028034 | Fubp1 | 5751 | 25.62431669 | 24.87316428 | 27.74055001 | 25.5335152 | 23.99482267 | 24.04828368 | 22.7884056 | 25.86670272 | 21.77134549 | 21.57896904 | 21.62375533 | 21.08979913 | 23.17768049 | 21.32018618 | 21.82228121 | 23.77599705 | 21.19908719 | 20.40243864 |
| ENSMUSG00000028035 | Dnajb4 | 4309 | 17.12438746 | 16.52888555 | 16.97395635 | 17.35104984 | 17.23545097 | 18.90570759 | 14.33983724 | 16.22002701 | 14.42340503 | 14.92540247 | 15.14518294 | 14.83150004 | 16.48595054 | 18.4741624 | 16.17847124 | 18.81207145 | 19.21206807 | 19.53212655 |
| ENSMUSG00000028036 | Ptgfr | 4940 | 0.258128599 | 0.026974003 | 0.048961063 | 0.193680553 | 0 | 0.020061835 | 0.229859169 | 0.386562777 | 0.466891677 | 0.505867869 | 0.25144715 | 0.067792303 | 0.04993109 | 0.037606548 | 0 | 0.294543134 | 0.224992431 | 0 |
| ENSMUSG00000028037 | Ifi44 | 3116 | 1.827886261 | 2.640656249 | 1.738714827 | 2.968193838 | 2.934873394 | 4.03927931 | 0.713637968 | 0.968292616 | 2.220582365 | 1.165148957 | 1.333896466 | 0.931455216 | 0.522449702 | 0.685631585 | 1.388370786 | 2.383946674 | 0.811789344 | 0.829726578 |
| ENSMUSG00000028039 | Efna3 | 2428 | 0.367631259 | 0.109762419 | 0.498080008 | 0.206413298 | 0.535239678 | 0.367359639 | 0.662533395 | 0.534819474 | 0.474968057 | 0.563167378 | 0.373856759 | 0.528731565 | 0.650173539 | 0.306056589 | 0.305448108 | 0.220786039 | 0.331487741 | 0.113280698 |
| ENSMUSG00000028040 | Efna4 | 1595 | 20.99942239 | 16.6460042 | 18.46989341 | 14.65380297 | 19.07170894 | 15.47163719 | 14.89088704 | 15.73191748 | 13.91820188 | 16.7318483 | 14.01798155 | 16.79718999 | 14.93875614 | 23.96456672 | 18.83130674 | 16.63660873 | 14.46547382 | 21.96915422 |
| ENSMUSG00000028041 | Adam15 | 5858 | 10.12200761 | 9.91197928 | 9.570659215 | 9.589752077 | 9.670753526 | 10.46376433 | 7.487001257 | 10.91397774 | 9.238491245 | 11.14778491 | 10.59400325 | 13.01539691 | 11.7982274 | 9.973824161 | 13.91906437 | 13.66121022 | 13.68702853 | 13.40013576 |
| ENSMUSG00000028042 | Zbtb7b | 5835 | 4.909766691 | 4.966961092 | 4.816627444 | 4.973844943 | 4.66058901 | 4.959519522 | 4.086647846 | 4.444325833 | 4.559809629 | 4.694868653 | 4.666962525 | 5.050671782 | 5.579959795 | 5.165760534 | 4.886285634 | 4.331071274 | 4.486180083 | 4.440323782 |
| ENSMUSG00000028044 | Cks1b | 1528 | 22.75473429 | 18.66219729 | 19.72297736 | 18.337724 | 20.44346758 | 18.1931175 | 24.55428952 | 17.7464697 | 17.25089665 | 20.2427103 | 16.6962224 | 18.8487447 | 23.60054419 | 22.15820661 | 19.52220318 | 23.55574016 | 18.05951163 | 19.2603875 |
| ENSMUSG00000028047 | Thbs3 | 4835 | 0.246152002 | 0.26181799 | 0.300146 | 0.10365491 | 0.2687822 | 0.081990045 | 1.585243844 | 0.545041523 | 0.766656964 | 0.390078207 | 0.602745081 | 1.235217352 | 0.591778946 | 0.124875519 | 0.426076069 | 0.419731581 | 0.412195954 | 0.250299969 |
| ENSMUSG00000028048 | Gba | 2084 | 23.00664038 | 20.86052157 | 22.3530277 | 21.70926921 | 20.23203701 | 26.2981398 | 27.31149364 | 21.25872687 | 24.2692137 | 20.38521288 | 20.12783759 | 24.82779233 | 20.38137273 | 20.83743844 | 21.88585526 | 25.77817763 | 20.65279819 | 26.92384302 |
| ENSMUSG00000028049 | Scamp3 | 2328 | 29.96176712 | 29.70685928 | 29.19450608 | 25.32473856 | 27.78750232 | 29.90618156 | 27.51776945 | 24.92021446 | 23.31779544 | 25.01336706 | 28.56647002 | 26.61313809 | 28.692189 | 25.71581986 | 26.14033421 | 20.90522423 | 26.98316178 | 26.67752641 |
| ENSMUSG00000028051 | Hcn3 | 4146 | 0.153781389 | 0.008034948 | 0.035002555 | 0.38462049 | 0.174138653 | 0.059759688 | 0.034234934 | 0.221084819 | 0 | 0.341177303 | 0.057615724 | 0.188475465 | 0.202277519 | 0.011202144 | 0.18881562 | 0.15700442 | 0.092441623 | 0.013267995 |
| ENSMUSG00000028053 | Ash1l | 11241 | 14.0473916 | 13.30322766 | 14.22244621 | 12.23634844 | 13.32073788 | 13.43182806 | 13.30024601 | 13.63116435 | 15.39960197 | 12.4200011 | 11.95967736 | 13.94274358 | 12.80145918 | 9.841634744 | 12.11745786 | 14.42923343 | 12.00829265 | 9.958502996 |
| ENSMUSG00000028057 | Rit1 | 3294 | 22.34941253 | 20.52980427 | 18.97346728 | 18.24378187 | 18.48417834 | 20.91023062 | 21.9327446 | 20.37160019 | 19.04282438 | 21.92922811 | 21.81346009 | 21.45192132 | 26.59790075 | 24.08211939 | 21.38878584 | 25.68981249 | 24.50369802 | 21.99362282 |
| ENSMUSG00000028059 | Arhgef2 | 9201 | 15.4618872 | 13.91748347 | 14.78386777 | 14.65222587 | 13.37608182 | 14.42258673 | 9.512928597 | 10.03686871 | 11.12810327 | 11.6839043 | 9.818763883 | 8.844607766 | 7.361452279 | 6.390415676 | 7.66176091 | 8.477118495 | 7.972669522 | 6.426995949 |
| ENSMUSG00000028060 | 2810403A07R | 3823 | 38.22463907 | 32.35437516 | 36.3402509 | 46.70513543 | 42.39084148 | 37.6279324 | 37.44916726 | 40.34028797 | 37.36197663 | 47.9893765 | 54.74812793 | 44.12109138 | 41.96374444 | 40.10251961 | 37.56961126 | 57.3407605 | 57.85536475 | 38.5193248 |
| ENSMUSG00000028062 | Lamtor2 | 1398 | 87.22986411 | 71.29626574 | 84.91319277 | 67.95048458 | 70.8207203 | 71.56435558 | 87.07834255 | 84.93575041 | 91.77109914 | 77.37032423 | 78.2579792 | 84.96118025 | 94.74692267 | 83.32029411 | 90.30155025 | 77.26595385 | 78.40715962 | 80.54623853 |
| ENSMUSG00000028063 | Lmna | 4442 | 18.43932332 | 26.52582324 | 16.72709199 | 21.75483454 | 18.65897626 | 21.86478114 | 19.81125232 | 29.78353938 | 22.97617876 | 24.55190829 | 23.88747924 | 22.96961531 | 23.49985073 | 23.57753648 | 26.68547418 | 38.98021762 | 34.84051934 | 36.4704681 |
| ENSMUSG00000028064 | Sema4a | 6291 | 5.114674655 | 5.422413556 | 5.982293215 | 4.852312781 | 4.812427275 | 6.458947947 | 3.985967228 | 5.172456978 | 3.338916395 | 3.635048654 | 5.247575315 | 4.524874888 | 4.775574252 | 5.743681448 | 5.213231361 | 3.627595297 | 5.020007733 | 5.561244933 |
| ENSMUSG00000028066 | Pmf1 | 1418 | 17.29582974 | 15.50529632 | 17.05695711 | 18.76421008 | 19.4156984 | 16.87868147 | 16.3492332 | 21.22398667 | 14.58083985 | 18.85363099 | 19.33908756 | 18.18535692 | 20.00412725 | 23.94261487 | 15.66124022 | 19.98240443 | 18.81177902 | 16.33204085 |
| ENSMUSG00000028068 | Iqgap3 | 5766 | 0.066345228 | 0.202211463 | 0.15100998 | 0 | 0.025042624 | 0.103127437 | 0.032821838 | 0.039742354 | 0 | 0.008177368 | 0 | 0 | 0.145446166 | 0.136931926 | 0.142912091 | 0.092970604 | 0.312406513 | 0 |
| ENSMUSG00000028069 | Gpatch4 | 1557 | 4.886593776 | 4.300508502 | 3.697142012 | 4.535620984 | 3.060411331 | 3.628138489 | 4.953093555 | 2.600125019 | 2.565890834 | 4.057928219 | 2.209246635 | 3.979151283 | 7.097205825 | 5.607892376 | 3.942859234 | 5.311991778 | 4.430785769 | 3.745000201 |
| ENSMUSG00000028070 | Apoa1bp | 899 | 163.2595916 | 143.1453953 | 155.6669892 | 134.6052656 | 169.2380542 | 156.8710565 | 160.8841596 | 140.067131 | 165.1583863 | 159.0746196 | 162.7218274 | 151.7391317 | 175.7072293 | 152.4543959 | 142.7617871 | 150.4791056 | 149.4682947 | 159.8262367 |
| ENSMUSG00000028071 | Sh2d2a | 4751 | 0 | 0 | 0 | 0 | 0 | 0 | 0 | 0 | 0 | 0.019848749 | 0 | 0 | 0 | 0 | 0.078049674 | 0.016118968 | 0 | 0 |
| ENSMUSG00000028072 | Ntrk1 | 2571 | 0.198390553 | 0.129571739 | 0.018815064 | 0.141769099 | 0.037442181 | 0.578211594 | 0.257634191 | 0.311956612 | 0 | 0.091697206 | 0.111493485 | 0.542742732 | 0 | 0 | 0.064101993 | 0 | 0 | 0 |
| ENSMUSG00000028073 | Pear1 | 7750 | 0.14259801 | 0.412650045 | 0.386988243 | 0.546732532 | 0.223580549 | 0.441179176 | 0.036629171 | 0.423812465 | 0.281662624 | 0.03650377 | 0.129454726 | 0.194454566 | 0.012730817 | 0.005992785 | 0.01063266 | 0.148221706 | 0 | 0.085175391 |
| ENSMUSG00000028076 | Cd1d1 | 2515 | 0.878834653 | 1.854395702 | 0.673190283 | 1.684762276 | 1.779828589 | 1.714150227 | 1.091107506 | 0.637805527 | 1.244602464 | 0.599929425 | 0.322932125 | 1.531324341 | 0.980753823 | 1.108010037 | 1.097616002 | 0.959168297 | 1.386756667 | 1.11549282 |
| ENSMUSG00000028078 | Dclk2 | 5477 | 0 | 0 | 0 | 0 | 0.017576017 | 0 | 0.025915289 | 0.048812638 | 0.05263933 | 0.241047963 | 0.008722833 | 0.050954594 | 0.234184745 | 0.067838725 | 0.248247461 | 0.006991164 | 0.048983765 | 0 |
| ENSMUSG00000028080 | Lrba | 9951 | 1.862351196 | 1.606892697 | 1.341683688 | 2.293848177 | 1.794487356 | 1.359450936 | 1.859035018 | 1.834586308 | 1.312041553 | 1.416748093 | 1.617944026 | 1.3854355 | 1.100561314 | 1.582207379 | 1.072374935 | 1.762345311 | 1.151599113 | 1.210631532 |
| ENSMUSG00000028081 | Rps3a | 971 | 67.85069294 | 33.31289422 | 43.34188611 | 100.6472092 | 89.52240386 | 57.82002795 | 61.39441344 | 42.32237383 | 39.82918466 | 95.9038506 | 75.72158713 | 52.71159896 | 82.05051123 | 56.4886377 | 44.08693777 | 100.6360763 | 89.91483455 | 75.91370032 |
| ENSMUSG00000028082 | Sh3d19 | 6416 | 12.56075651 | 11.9990802 | 11.06046899 | 12.60455949 | 12.54310729 | 11.67763921 | 10.02149792 | 8.327800961 | 6.68253786 | 10.2884951 | 8.875896276 | 9.84777588 | 8.534667733 | 11.21288831 | 9.16375004 | 6.803505679 | 12.20993623 | 10.80291061 |
| ENSMUSG00000028085 | Pet112l | 4477 | 5.126824895 | 3.794857282 | 5.43486392 | 4.711807802 | 4.676655527 | 4.028857499 | 4.935229185 | 4.563979291 | 4.700984935 | 5.83459674 | 5.420969155 | 5.473101603 | 5.432350953 | 7.541847561 | 4.95118094 | 6.457317085 | 5.504536276 | 3.378937759 |
| ENSMUSG00000028086 | Fbxw7 | 7321 | 2.572024721 | 2.475374191 | 2.907301374 | 2.420877783 | 2.577204491 | 2.11179523 | 2.02279313 | 2.994459634 | 2.841031343 | 2.988379499 | 2.766914683 | 2.759905731 | 2.446043706 | 1.960281793 | 2.504397264 | 2.641272605 | 2.554737452 | 3.103231946 |
| ENSMUSG00000028088 | Fmo5 | 5611 | 3.393747792 | 3.348506922 | 3.301918041 | 4.092455239 | 2.856519441 | 3.29409546 | 5.160463287 | 5.159479776 | 6.143844893 | 6.310849785 | 5.619581642 | 5.550728876 | 5.908224632 | 7.060560781 | 6.828987164 | 5.302406336 | 8.804597524 | 7.548923931 |
| ENSMUSG00000028089 | Chd1l | 2985 | 9.341171013 | 12.49931039 | 11.24664324 | 11.69171188 | 12.43206471 | 11.52080305 | 12.60086424 | 9.864771274 | 10.251786 | 11.21507515 | 11.73167322 | 14.71588826 | 9.783750428 | 10.82917415 | 7.991859585 | 10.54434817 | 12.85247008 | 7.500404166 |
| ENSMUSG00000028093 | Acp6 | 5122 | 21.73390395 | 20.26610277 | 19.53074205 | 19.92517363 | 21.00250867 | 19.16516306 | 19.79521104 | 20.80374906 | 23.6006294 | 22.54433274 | 25.77161466 | 20.77013918 | 26.93896381 | 20.71939471 | 21.01102382 | 27.79470487 | 24.00444367 | 25.57139459 |

| ENSMUSG00000028096 | Gpr89 | 8353 | 4.060712375 | 4.398913244 | 4.499728611 | 3.567212139 | 3.319045616 | 4.057712171 | 4.486010022 | 4.457990224 | 3.48603695 | 4.419849234 | 4.215269292 | 2.99358516 | 3.401793889 | 3.736433225 | 5.253161382 | 4.125649068 | 4.051492897 | 4.247680335 |
| --- | --- | --- | --- | --- | --- | --- | --- | --- | --- | --- | --- | --- | --- | --- | --- | --- | --- | --- | --- | --- |
| ENSMUSG00000028098 | Rnf115 | 2256 | 57.03154603 | 56.09737816 | 62.3538237 | 56.06270349 | 52.2282576 | 57.54794414 | 63.25134736 | 56.39135302 | 57.8728967 | 59.08468001 | 55.4621526 | 55.93936751 | 57.61950444 | 59.53736734 | 56.26861423 | 59.48961833 | 62.603016 | 55.7406108 |
| ENSMUSG00000028099 | Polr3c | 3909 | 16.6367151 | 15.50170238 | 15.2706412 | 13.59022197 | 14.25857447 | 15.617541 | 20.93909812 | 16.60964034 | 15.88878633 | 15.33091426 | 17.07383397 | 13.1650143 | 19.20777132 | 15.70710758 | 17.83398121 | 16.03523262 | 18.95234892 | 17.53424072 |
| ENSMUSG00000028100 | Nudt17 | 1353 | 0.439817047 | 0.56629458 | 0.572044705 | 0.134696362 | 0.177871115 | 0.146497365 | 0.034968721 | 0.790382311 | 0.852344746 | 0.766678102 | 0.176551953 | 0.660052181 | 0.546917043 | 0.068653492 | 0.395875999 | 0.650912025 | 0.424903513 | 1.016428432 |
| ENSMUSG00000028101 | Pias3 | 3848 | 5.578252051 | 5.670464063 | 5.342710603 | 5.162322071 | 4.940776988 | 6.258479345 | 6.098515787 | 6.471269846 | 4.805807063 | 7.351980757 | 8.231496224 | 5.120303766 | 5.461381694 | 5.250306137 | 5.503534233 | 5.462979071 | 5.936193595 | 5.618133821 |
| ENSMUSG00000028102 | Pex11b | 5029 | 13.06681291 | 12.85086792 | 13.84162064 | 13.33582368 | 12.83454159 | 12.51381421 | 15.08097521 | 13.73832888 | 11.13813113 | 16.46383673 | 16.28281544 | 13.22972158 | 15.35184938 | 13.94523205 | 12.44485253 | 15.19746518 | 14.15230331 | 12.66664259 |
| ENSMUSG00000028104 | Polr3gl | 1718 | 6.878020335 | 6.263134361 | 6.785809555 | 7.186870207 | 5.827380747 | 3.691938241 | 5.700654562 | 5.513222233 | 6.089275322 | 6.751497672 | 5.78416261 | 3.086430702 | 5.455799932 | 4.001004036 | 5.03627866 | 3.387760375 | 5.064068334 | 7.108278052 |
| ENSMUSG00000028106 | Rprd2 | 9812 | 3.92475433 | 5.340520037 | 5.48713201 | 4.685190964 | 5.548023407 | 5.125970707 | 3.529645432 | 4.242735286 | 5.0832522 | 4.757357952 | 4.76191494 | 4.766971591 | 3.534482047 | 3.30864422 | 3.74559599 | 3.941450619 | 4.734149189 | 3.700164129 |
| ENSMUSG00000028107 | Tars2 | 4420 | 10.12623311 | 12.20216646 | 13.54896619 | 11.62734344 | 10.91135464 | 13.27385442 | 11.79605705 | 10.45538618 | 12.98959343 | 10.24087673 | 11.23035789 | 10.51910564 | 11.12758406 | 9.026124676 | 9.741091834 | 10.04911868 | 11.90543114 | 11.33784982 |
| ENSMUSG00000028108 | Ecm1 | 2602 | 49.82351533 | 57.88147378 | 53.67193779 | 55.57676969 | 56.82600673 | 50.63824696 | 12.85552037 | 17.7311384 | 21.59047175 | 14.53299924 | 16.10247448 | 21.19364898 | 26.1447786 | 24.11451271 | 28.21720699 | 45.51608328 | 25.87982462 | 31.98646367 |
| ENSMUSG00000028109 | Hormad1 | 1758 | 0 | 0 | 0 | 0 | 0 | 0 | 0 | 0 | 0 | 0 | 0 | 0 | 0 | 0 | 0 | 0 | 0 | 0 |
| ENSMUSG00000028111 | Ctsk | 1474 | 0 | 0 | 0 | 0 | 0.032653951 | 0 | 0 | 0.129553604 | 0 | 0.031988265 | 0 | 0 | 0 | 0.094526636 | 0 | 0.441614875 | 0 | 0.074639222 |
| ENSMUSG00000028114 | Mettl14 | 4425 | 6.464629032 | 6.632465493 | 6.635645859 | 3.881698015 | 6.787417032 | 4.781698811 | 4.137854632 | 4.954223498 | 4.672458963 | 5.167930188 | 5.257944583 | 3.935477226 | 4.158374044 | 3.411147661 | 4.124811132 | 3.66032247 | 4.018847856 | 3.070564827 |
| ENSMUSG00000028115 | Bnipl | 2854 | 0.491475406 | 1.062184083 | 1.050861556 | 1.069583031 | 0.455347561 | 0.972303112 | 0.198932077 | 0.187348856 | 0.577246191 | 0.280855626 | 0.385011929 | 0.664937816 | 0.795118498 | 0.5207466 | 0.750694081 | 0.509825887 | 0.711735717 | 0.48185973 |
| ENSMUSG00000028116 | Myoz2 | 3392 | 0 | 0.166957312 | 0.042783193 | 0.026863823 | 0.07094918 | 0.014608707 | 0.013948313 | 0.022519105 | 0.048569004 | 0.027801122 | 0.140845986 | 0 | 0.058174431 | 0 | 0 | 0 | 0.101691236 | 0 |
| ENSMUSG00000028121 | Bcar3 | 3286 | 33.74802321 | 33.93130157 | 32.66611803 | 40.34772946 | 35.60824907 | 33.99021956 | 25.97445916 | 29.41721561 | 24.10276014 | 29.81715197 | 29.00518629 | 30.71050455 | 25.50667299 | 23.54712404 | 31.39643821 | 25.71739822 | 34.02246137 | 26.06487497 |
| ENSMUSG00000028124 | Gclm | 5934 | 27.94998262 | 30.47787373 | 27.41492797 | 29.64462288 | 25.74498243 | 30.17923477 | 26.0163922 | 26.54931916 | 25.95153058 | 26.61861407 | 23.19508854 | 26.47810755 | 29.37967571 | 30.9079375 | 27.12748211 | 26.05619695 | 28.27000363 | 26.89272305 |
| ENSMUSG00000028125 | Abca4 | 11003 | 0.096576334 | 0.012110477 | 0 | 0 | 0.087488728 | 0.058546354 | 0 | 0.111074877 | 0.134755482 | 0 | 0.004341994 | 0.020291071 | 0 | 0 | 0 | 0 | 0.006966518 | 0 |
| ENSMUSG00000028126 | Pip5k1a | 5908 | 8.597441664 | 11.29976981 | 9.096647296 | 8.29013586 | 9.238591984 | 8.613855346 | 6.710904696 | 7.983686014 | 9.397329595 | 7.725436823 | 9.744215465 | 9.277417173 | 7.289567329 | 6.910012325 | 9.428656663 | 8.892131577 | 9.516702362 | 9.869609534 |
| ENSMUSG00000028127 | Abcd3 | 4284 | 37.23667732 | 39.88371486 | 46.02486221 | 43.31702465 | 37.80670475 | 40.55366105 | 34.83291068 | 34.67984252 | 33.90869307 | 35.90233287 | 35.93158642 | 32.48096331 | 26.80782059 | 28.81614946 | 30.28564414 | 28.22636279 | 28.55684867 | 25.70687014 |
| ENSMUSG00000028128 | F3 | 2063 | 58.61717198 | 77.81620807 | 65.34998987 | 73.3437849 | 99.43686799 | 75.30190003 | 25.04384168 | 47.24527917 | 48.05426245 | 30.67195529 | 42.35598597 | 48.10482214 | 19.56059541 | 14.25065793 | 17.49518321 | 16.77882166 | 31.47103585 | 18.61190329 |
| ENSMUSG00000028132 | Tmem56 | 7661 | 0.011096509 | 0.095664231 | 0.018942774 | 0.208149922 | 0.062827208 | 0.032340904 | 0.030878919 | 0.209382705 | 0.032256767 | 0.018463922 | 0.293097905 | 0.072856889 | 0.135226506 | 0.048499243 | 0 | 0.019992485 | 0.01000556 | 0.201051428 |
| ENSMUSG00000028133 | Rwdd3 | 5583 | 0.860305372 | 1.223203173 | 0.788463418 | 1.452600016 | 1.4483545 | 1.162709672 | 1.228790694 | 1.710209672 | 1.6082143 | 1.477946099 | 1.24079688 | 1.259676426 | 0.88360948 | 1.655449291 | 1.475964746 | 1.460845312 | 1.84663691 | 0.571472002 |
| ENSMUSG00000028134 | Ptbp2 | 3350 | 18.47389143 | 21.70807405 | 20.34576247 | 18.86363239 | 20.5746014 | 19.09629225 | 20.59160772 | 16.7020506 | 25.64629011 | 21.05595584 | 18.78197621 | 18.27754682 | 16.19854 | 18.02307869 | 17.69822058 | 20.29973694 | 24.11696533 | 20.96914307 |
| ENSMUSG00000028136 | Snx27 | 9023 | 7.984736049 | 7.328615208 | 7.859425413 | 8.008402562 | 6.177188031 | 7.375520469 | 7.514027364 | 6.019017646 | 5.911175652 | 5.486893319 | 6.539075 | 5.691057251 | 7.17316585 | 4.282553553 | 6.000093588 | 6.170291828 | 5.899947512 | 5.224737278 |
| ENSMUSG00000028137 | Celf3 | 2731 | 0.560302577 | 0.451328116 | 0.318829567 | 0.467121656 | 0.387734281 | 0.163300843 | 0.814242369 | 0.237741062 | 0.180973339 | 0.36256491 | 0.349871538 | 0.592696527 | 0.252891557 | 0.255093852 | 0.211212663 | 0.350518193 | 0.28067592 | 0.261852211 |
| ENSMUSG00000028138 | Adh5 | 2874 | 105.0206176 | 101.8744006 | 96.39360068 | 94.94262162 | 103.0632771 | 99.70892773 | 146.728569 | 128.6103238 | 133.1897973 | 129.2624882 | 113.7855222 | 114.0200957 | 169.9153268 | 193.6463115 | 159.3728938 | 129.3273925 | 135.5155982 | 166.1565955 |
| ENSMUSG00000028139 | Riiad1 | 1154 | 0.220997449 | 0.43300989 | 0.377263235 | 0.394809743 | 0.291961409 | 0.128819931 | 0.327990842 | 0.132382677 | 0.249831551 | 0.204292475 | 0.496793329 | 0.145101376 | 0.085497257 | 0.32196941 | 0.142813019 | 0.132723076 | 0.365328653 | 0.572018441 |
| ENSMUSG00000028140 | Mrpl9 | 2864 | 10.81922951 | 11.10817527 | 10.94484905 | 12.51974228 | 11.14227144 | 11.60959645 | 13.67838623 | 12.29517982 | 16.17836251 | 13.86204335 | 14.04557089 | 14.59704304 | 13.64206651 | 17.95167763 | 10.90460173 | 11.71179193 | 11.46844848 | 10.04530825 |
| ENSMUSG00000028141 | Oaz3 | 1126 | 0.415237066 | 0.295851636 | 0.214802533 | 0.242776435 | 0.683934972 | 0.396069807 | 0.840367299 | 0.678373044 | 0.073155445 | 0.502494173 | 0.339431321 | 0.09913972 | 0.569551443 | 0.123740908 | 0.695230517 | 0.374064548 | 0.374413202 | 0 |
| ENSMUSG00000028145 | Them4 | 4177 | 4.355330462 | 5.471066659 | 4.34284747 | 4.548469114 | 5.715449878 | 5.006285269 | 8.902984352 | 9.116070193 | 11.85209288 | 8.443554234 | 7.525957069 | 7.483067045 | 10.1451082 | 14.34351753 | 10.40642602 | 7.956966058 | 9.863722569 | 10.89119733 |
| ENSMUSG00000028148 | Them5 | 1535 | 0 | 0 | 0.063027401 | 0 | 0 | 0 | 0.277403329 | 0 | 0 | 0 | 0 | 0 | 0 | 0 | 0 | 0 | 0 | 0 |
| ENSMUSG00000028149 | Rap1gds1 | 3648 | 24.7597311 | 34.30826304 | 28.12507069 | 28.2383556 | 26.53325068 | 31.24212917 | 22.22969618 | 23.53523043 | 20.90938245 | 24.68690878 | 20.78367848 | 23.28712677 | 17.72865777 | 15.65960183 | 15.92494348 | 17.01454896 | 17.27204828 | 16.01416429 |
| ENSMUSG00000028150 | Rorc | 3552 | 18.69174689 | 21.71152875 | 23.09727125 | 24.6404184 | 22.35857941 | 19.97734079 | 22.95037888 | 26.48307633 | 24.55885146 | 26.65501468 | 26.2278066 | 25.39361226 | 28.16585817 | 23.71891175 | 24.67221548 | 25.46239152 | 30.64377342 | 26.96251543 |
| ENSMUSG00000028152 | Tspan5 | 4258 | 3.204359207 | 3.559738579 | 3.635399181 | 3.509634228 | 5.369343293 | 2.339149706 | 4.789047585 | 5.426586063 | 5.494114832 | 7.341690056 | 7.214489972 | 4.561730996 | 6.592264192 | 7.94065186 | 5.534826202 | 5.188746001 | 6.606740706 | 6.601609567 |
| ENSMUSG00000028156 | Eif4e | 2874 | 45.09334785 | 39.60687528 | 41.3716554 | 38.18947662 | 38.16724218 | 37.6559395 | 47.01635735 | 42.11263856 | 42.23265899 | 45.90385098 | 43.33657507 | 36.82202375 | 41.21292045 | 39.73765167 | 40.34139815 | 41.63470707 | 46.00755891 | 39.85002095 |
| ENSMUSG00000028158 | Mttp | 4389 | 1.220244288 | 1.502837333 | 1.509950708 | 1.131500076 | 1.952035182 | 1.479017566 | 1.724772984 | 1.035519682 | 0.788258675 | 1.042063845 | 2.590667605 | 2.530716987 | 1.303828299 | 2.148131634 | 1.333019124 | 0.584522829 | 1.737738228 | 0.814671209 |
| ENSMUSG00000028159 | Dapp1 | 5454 | 5.533310411 | 6.529424995 | 6.891498549 | 5.997951931 | 6.186373027 | 5.315062182 | 3.287771418 | 4.712777194 | 5.36919925 | 3.440773725 | 5.737550019 | 4.666656058 | 2.939654036 | 3.26147515 | 3.958491985 | 2.63976318 | 3.604948719 | 2.62236299 |
| ENSMUSG00000028161 | Ppp3ca | 5625 | 16.33710055 | 20.20686135 | 18.89362599 | 19.4555784 | 19.92019881 | 21.46844653 | 17.82321185 | 19.71067452 | 18.23921969 | 20.53675075 | 22.13360743 | 19.77610943 | 18.81190445 | 18.38773024 | 18.76593532 | 18.33184102 | 21.23106509 | 24.65385921 |
| ENSMUSG00000028163 | Nfkb1 | 5626 | 5.772121309 | 5.240297082 | 5.571640183 | 3.984364346 | 4.67117496 | 4.412712322 | 5.693331609 | 4.785930269 | 5.270936962 | 4.877658958 | 5.935779591 | 4.930747299 | 6.260751677 | 4.763284469 | 3.925352641 | 5.778301757 | 4.618775204 | 3.598178328 |
| ENSMUSG00000028164 | Manba | 6433 | 16.22768729 | 19.2845046 | 17.11458963 | 20.53186189 | 18.06911171 | 20.62835699 | 12.77508523 | 15.95852288 | 10.82002357 | 12.78265607 | 13.01130534 | 12.08630779 | 8.036662413 | 8.735791348 | 8.300515535 | 8.315246159 | 9.061759302 | 8.78196061 |

| ENSMUSG00000028165 | Cisd2 | 4357 | 34.58362383 | 36.77645655 | 39.12515974 | 34.81127303 | 36.598821 | 36.53049811 | 29.22157884 | 31.93365205 | 35.61872651 | 31.06946731 | 31.79880183 | 31.04001617 | 21.75042761 | 27.04352759 | 23.99089908 | 26.60217319 | 26.76771207 | 22.37230597 |
| --- | --- | --- | --- | --- | --- | --- | --- | --- | --- | --- | --- | --- | --- | --- | --- | --- | --- | --- | --- | --- |
| ENSMUSG00000028167 | Bdh2 | 1267 | 0.536766232 | 0.236634608 | 0.458154984 | 0.107879347 | 0.07597778 | 0.039110287 | 0.970899489 | 1.899069733 | 0.260056927 | 0.707074477 | 0.188535748 | 0.044053404 | 0.350424037 | 1.282985839 | 1.463354392 | 1.450630746 | 1.39148355 | 1.910339934 |
| ENSMUSG00000028172 | Tacr3 | 3759 | 0 | 0.035448677 | 0 | 0 | 0 | 0 | 0 | 0 | 0 | 0 | 0 | 0 | 0.262473622 | 0.432440292 | 0.175372411 | 0 | 0.101958757 | 0.248777551 |
| ENSMUSG00000028173 | Wls | 3292 | 135.0042894 | 137.673732 | 137.0828265 | 135.0223416 | 143.3723097 | 149.6968212 | 129.3625222 | 126.6544523 | 122.708793 | 129.3779777 | 117.0573557 | 130.7904074 | 123.8243112 | 124.5610108 | 135.6447335 | 147.9515572 | 146.9019485 | 130.6884033 |
| ENSMUSG00000028174 | Rpe65 | 1588 | 0 | 0 | 0 | 0 | 0 | 0 | 0 | 0 | 0 | 0 | 0 | 0 | 0 | 0 | 0 | 0 | 0 | 0 |
| ENSMUSG00000028175 | Depdc1a | 3047 | 0 | 0.098397128 | 0 | 0 | 0 | 0 | 0.155276268 | 0 | 0 | 0 | 0.062717372 | 0 | 0 | 0 | 0.121698065 | 0 | 0.11320534 | 0 |
| ENSMUSG00000028176 | Lrrc7 | 8314 | 0 | 0 | 0 | 0 | 0 | 0 | 0 | 0 | 0 | 0 | 0 | 0 | 0.017800788 | 0 | 0 | 0 | 0 | 0 |
| ENSMUSG00000028177 | 1810013D15R | 649 | 0 | 0 | 0 | 0 | 0 | 0 | 0 | 0.117696155 | 0.063461503 | 0 | 0 | 0.688020494 | 0 | 0 | 0 | 0 | 0 | 0.423798973 |
| ENSMUSG00000028179 | Cth | 2654 | 0.944915368 | 1.016708527 | 1.184732281 | 0.37767256 | 0.308305464 | 0.616141751 | 7.665580987 | 6.072793449 | 5.214268763 | 4.246050519 | 1.854114817 | 6.582630893 | 4.256597233 | 7.524852139 | 5.852670147 | 1.84672108 | 2.38275772 | 5.057355705 |
| ENSMUSG00000028180 | Zranb2 | 4405 | 33.74360966 | 29.38038457 | 35.25063165 | 31.81515831 | 35.60997485 | 28.98919283 | 35.05756731 | 34.9150521 | 32.95856256 | 28.25831024 | 36.32198319 | 38.74785385 | 32.4661131 | 26.75938565 | 32.83965102 | 32.32752984 | 32.47947022 | 25.88737304 |
| ENSMUSG00000028182 | Lrriq3 | 4049 | 0.3779171 | 0.559465746 | 0.394252038 | 0.247553217 | 1.343271764 | 0.220288764 | 1.133447729 | 0.801766907 | 0.508601084 | 0.570606189 | 0.707951966 | 0.124065439 | 1.084351849 | 0.103234573 | 0.946343467 | 0.756544478 | 1.031752621 | 1.154797252 |
| ENSMUSG00000028184 | Lphn2 | 10475 | 10.40413092 | 9.690156438 | 10.51056373 | 10.4431568 | 10.29724496 | 10.94654181 | 6.192427953 | 6.329547549 | 6.825755739 | 7.368563363 | 6.576753234 | 8.163207168 | 8.062648437 | 7.324642708 | 7.780112415 | 9.705161112 | 9.941054767 | 8.927492261 |
| ENSMUSG00000028185 | Dnase2b | 2573 | 1.354615015 | 1.009873979 | 0.658015376 | 0.956197588 | 1.346870775 | 1.656251492 | 2.114635863 | 1.662475348 | 0.912410182 | 1.814193402 | 1.225475033 | 1.214798719 | 1.054510474 | 0.974730167 | 1.681369362 | 1.131009002 | 1.251227726 | 0.555863496 |
| ENSMUSG00000028186 | Uox | 3149 | 0.202469876 | 0.359681932 | 0.245784848 | 0.0868105 | 0.183417937 | 0.047208066 | 0.105172675 | 0.072770535 | 0.013079237 | 0.149732306 | 0.01517147 | 0.07089954 | 0 | 0.176986043 | 0.248596241 | 0 | 0.255589786 | 0.174687541 |
| ENSMUSG00000028187 | Rpf1 | 1254 | 37.48861618 | 39.84795959 | 40.079823 | 28.59373357 | 39.84125739 | 37.61898115 | 40.40819707 | 42.02991652 | 53.47021345 | 37.33704014 | 41.22209336 | 36.85436094 | 36.42851308 | 46.6663079 | 39.29590179 | 40.97766757 | 38.35686014 | 49.65734355 |
| ENSMUSG00000028188 | Spata1 | 4004 | 1.348191148 | 2.09661572 | 1.800113892 | 1.786484506 | 2.308024312 | 2.289524404 | 2.197841728 | 2.365563386 | 2.417290511 | 2.390507683 | 1.968748289 | 2.481315669 | 2.488773049 | 1.86750701 | 2.068309874 | 2.70635412 | 2.804596651 | 3.022478391 |
| ENSMUSG00000028189 | Ctbs | 3022 | 12.3352215 | 10.85810748 | 8.355719009 | 9.37755446 | 9.954484544 | 9.822001129 | 10.66179164 | 8.176864446 | 9.676514292 | 10.09480642 | 12.48915195 | 8.182110697 | 11.62287396 | 9.78983576 | 8.902917272 | 8.501984635 | 11.4395499 | 7.790833116 |
| ENSMUSG00000028191 | Bcl10 | 1913 | 60.90260716 | 62.58575108 | 59.98014323 | 65.92418752 | 65.61843018 | 65.56088274 | 52.16018811 | 59.47473433 | 54.21204741 | 53.36187797 | 56.69061984 | 56.51591136 | 45.85057445 | 47.05104096 | 43.16148354 | 60.76857505 | 64.95235471 | 54.83657424 |
| ENSMUSG00000028194 | Ddah1 | 3975 | 0.352873159 | 0.662067583 | 0.340744365 | 0.733561469 | 0.411694441 | 0.536042149 | 2.868517138 | 1.652602066 | 1.264089289 | 1.672515512 | 0.973527456 | 1.19354247 | 3.810037385 | 3.528582237 | 1.876096004 | 1.367865724 | 2.111562018 | 1.978943965 |
| ENSMUSG00000028195 | Cyr61 | 2023 | 13.95127874 | 10.37425772 | 9.206035189 | 10.11216456 | 11.87238256 | 13.39858886 | 4.58392737 | 9.269633999 | 3.847875175 | 11.74688801 | 3.117298329 | 3.697132371 | 40.69944139 | 75.48599077 | 30.54984372 | 26.55547315 | 41.2249244 | 79.42738544 |
| ENSMUSG00000028197 | Col24a1 | 7766 | 0.405019705 | 0.969445544 | 0.554370873 | 0.42827147 | 1.041226265 | 0.567884791 | 0.042645989 | 0.38359611 | 0.037124081 | 0.121428543 | 0.123036205 | 0.179679573 | 0.031761471 | 0.155491408 | 0.143245172 | 0.069027621 | 0.074027099 | 0.014166651 |
| ENSMUSG00000028199 | Cryz | 2975 | 12.00146147 | 11.45515657 | 9.723486102 | 13.33904861 | 9.982318287 | 12.57556768 | 14.61524435 | 17.80600408 | 15.75470754 | 15.34180866 | 15.769751 | 13.52708999 | 15.70330274 | 12.06765699 | 12.15965246 | 18.57255344 | 16.02618936 | 12.94331923 |
| ENSMUSG00000028201 | Lhx8 | 2535 | 0 | 0 | 0 | 0 | 0 | 0 | 0 | 0 | 0 | 0 | 0 | 0 | 0 | 0 | 0 | 0 | 0 | 0 |
| ENSMUSG00000028207 | Asph | 16079 | 3.005683509 | 3.337712083 | 2.963363853 | 2.978086796 | 3.508340975 | 4.197451529 | 4.228392289 | 4.448931507 | 4.246983216 | 3.589306598 | 4.180568853 | 5.241722153 | 3.37183762 | 4.017894493 | 3.843667755 | 4.731851291 | 4.049777874 | 3.065374689 |
| ENSMUSG00000028211 | Trp53inp1 | 6576 | 18.77053394 | 26.27645716 | 20.81768414 | 24.86596535 | 28.06231681 | 24.01529226 | 9.619381345 | 22.92938028 | 16.53473261 | 16.66335681 | 20.56008706 | 17.44239456 | 12.85060436 | 11.6816485 | 13.42057979 | 20.33897379 | 27.98122741 | 15.91048069 |
| ENSMUSG00000028212 | Ccne2 | 3664 | 0.835254551 | 1.036481971 | 1.056190619 | 0.945043496 | 0.893278059 | 0.635638231 | 0.503601113 | 0.542031912 | 0.966714071 | 0.990885412 | 0.951848245 | 0.624574827 | 1.036724244 | 0.887305163 | 0.888352324 | 0.679282064 | 0.763597073 | 0.630562905 |
| ENSMUSG00000028214 | Gem | 2099 | 13.38538416 | 16.87070346 | 13.34362748 | 22.6394327 | 17.72557265 | 19.35838091 | 27.43188673 | 41.1400771 | 33.65167915 | 45.51087413 | 42.33512281 | 53.71493017 | 40.89449074 | 45.38200249 | 42.16338354 | 119.7972457 | 76.10481443 | 68.19137479 |
| ENSMUSG00000028217 | Cdh17 | 4544 | 0.121603717 | 0.014662365 | 0.234202832 | 0.020053277 | 0.084739302 | 0.447108732 | 0.33318788 | 0.210125453 | 0.290045886 | 0.508447284 | 0.599289752 | 0.073700259 | 0.238842909 | 0.194198429 | 1.668372862 | 0.514024439 | 0.472331124 | 0.23001167 |
| ENSMUSG00000028218 | Fam92a | 2513 | 10.79120665 | 7.383717501 | 6.795008442 | 9.22824177 | 8.235864376 | 8.656446495 | 11.71049992 | 8.708414874 | 9.931965202 | 9.062391434 | 11.4637087 | 9.617262992 | 14.50707794 | 12.60440414 | 12.82117656 | 12.99716996 | 13.13129392 | 11.01057727 |
| ENSMUSG00000028221 | Tmem55a | 2332 | 29.49115558 | 30.84156888 | 30.70018222 | 33.09623032 | 37.04837178 | 32.99974067 | 34.65268251 | 31.8051653 | 38.13108385 | 32.7749957 | 34.35617726 | 30.13375609 | 35.814295 | 38.4976076 | 39.82345925 | 41.09836635 | 45.45906399 | 35.50116021 |
| ENSMUSG00000028222 | Calb1 | 5047 | 0 | 0 | 0 | 0 | 0 | 0 | 0 | 0.015134695 | 0 | 0.009342323 | 0 | 0 | 0.205264566 | 0.06441621 | 0 | 0 | 0 | 0 |
| ENSMUSG00000028223 | Decr1 | 3866 | 21.21960417 | 20.55128109 | 21.09616456 | 20.68278138 | 23.19445778 | 24.34056934 | 21.51466362 | 21.97100438 | 22.0101763 | 20.91656909 | 23.78861745 | 22.68142936 | 24.34694727 | 16.44644481 | 24.90637251 | 22.93870842 | 22.81138365 | 23.57736416 |
| ENSMUSG00000028224 | Nbn | 2660 | 34.57939884 | 29.36794622 | 32.60666915 | 31.15621791 | 31.3219398 | 32.33967876 | 28.29867375 | 26.03113743 | 26.72478429 | 31.80013596 | 29.88628982 | 25.30589815 | 34.64361709 | 29.47278929 | 28.94951427 | 31.52497376 | 33.05282898 | 31.37173492 |
| ENSMUSG00000028226 | Mmp16 | 6075 | 0.258879261 | 0.005483604 | 0 | 0.067497843 | 0.071306554 | 0.016313657 | 0.233642859 | 0.106875859 | 0.332203995 | 0.139705787 | 0.062913526 | 0.284820665 | 0.105566243 | 0.313449808 | 0.020346447 | 0.018908942 | 0.290207351 | 0.190154937 |
| ENSMUSG00000028228 | Cpne3 | 7115 | 11.91220253 | 13.8355028 | 14.05313946 | 11.26379157 | 11.30406809 | 14.12409609 | 14.51631456 | 12.49640377 | 11.91890878 | 11.80921338 | 13.4965097 | 13.54010311 | 13.4024731 | 10.28101724 | 13.25514567 | 9.563233922 | 12.07157152 | 11.57394698 |
| ENSMUSG00000028229 | Fam82b | 2072 | 16.59589161 | 14.32518762 | 13.89104757 | 14.27080974 | 13.42676249 | 12.72299916 | 17.83359182 | 18.69068342 | 20.43423658 | 17.47670854 | 15.56375337 | 15.51632319 | 24.18978183 | 20.93570349 | 17.73734935 | 19.90298464 | 18.60823103 | 20.20363427 |
| ENSMUSG00000028232 | Tmem68 | 3702 | 11.45871574 | 8.872640113 | 8.872400624 | 8.122714549 | 7.995984078 | 11.33742986 | 8.5244616 | 8.779506874 | 11.22560627 | 9.425046032 | 9.033622619 | 7.34257906 | 5.916631894 | 7.32667397 | 8.280377521 | 6.59897556 | 7.919939798 | 5.527657402 |
| ENSMUSG00000028233 | Tgs1 | 5479 | 5.601156615 | 5.107288035 | 5.394456479 | 5.363546924 | 5.560778916 | 5.18228077 | 4.283097052 | 4.537918227 | 5.171805593 | 5.808856488 | 5.336425368 | 4.777796268 | 5.357271542 | 5.552268158 | 4.654820788 | 6.045149736 | 7.009116535 | 5.230834934 |
| ENSMUSG00000028234 | Rps20 | 4291 | 17.21603261 | 14.40116961 | 14.55377014 | 13.44215384 | 15.47938818 | 14.89699984 | 18.19294342 | 13.51998584 | 16.05803796 | 14.83417604 | 14.90810287 | 13.72303054 | 21.28020948 | 24.47217003 | 17.21612438 | 15.42867868 | 15.45199106 | 21.40881105 |
| ENSMUSG00000028236 | Sdr16c5 | 1561 | 0 | 0 | 0 | 0 | 0 | 0 | 0 | 0 | 0 | 0 | 0 | 0 | 0 | 0 | 0 | 0 | 0 | 0 |
| ENSMUSG00000028238 | Atp6v0d2 | 2707 | 437.2992805 | 440.918983 | 441.2051956 | 469.9334758 | 454.0261067 | 450.8255347 | 462.8672612 | 470.4000327 | 461.2220313 | 493.0880157 | 491.9391181 | 441.6179761 | 489.4192724 | 489.9705385 | 501.6026878 | 535.9411983 | 603.3383141 | 507.2542749 |

| ENSMUSG00000028240 | Cyp7a1 |  | 4168 | 0 | 0 | 0 | 0 | 0 | 0 | 0 | 0 | 0 | 0 | 0 | 0 | 0 | 0 | 0 | 0 | 0 | 0 |
| --- | --- | --- | --- | --- | --- | --- | --- | --- | --- | --- | --- | --- | --- | --- | --- | --- | --- | --- | --- | --- | --- |
| ENSMUSG00000028243 | Ubxn2b |  | 6634 | 3.530351521 | 2.852234537 | 3.026080057 | 3.797898329 | 2.677220355 | 3.159603 | 3.722824601 | 2.803693092 | 3.408410786 | 2.693716692 | 3.175875292 | 2.482004893 | 3.718112547 | 3.584469814 | 2.844484866 | 2.424186782 | 3.108162155 | 3.374842695 |
| ENSMUSG00000028244 | 1700025O08R |  | 454 | 0 | 0 | 0 | 0 | 0 | 0 | 0 | 0 | 0 | 0 | 0 | 0 | 0 | 0 | 0 | 0 | 0 | 0 |
| ENSMUSG00000028245 | Nsmaf |  | 8624 | 7.861288934 | 8.899919791 | 10.68547951 | 10.27025396 | 9.381929926 | 10.08981913 | 9.227727963 | 8.99452334 | 8.734941713 | 9.289082193 | 8.376129084 | 8.336105451 | 9.404182746 | 10.29158755 | 11.13168196 | 11.50405424 | 11.99474436 | 10.51832293 |
| ENSMUSG00000028246 | 6230409E13R |  | 8853 | 0.244862078 | 0.598300032 | 0.316916837 | 0.545517983 | 0.516495284 | 0.442185243 | 0.395474782 | 0.254529735 | 0.214004261 | 0.500640021 | 0.026982355 | 0.422416062 | 0.144880814 | 0.267553197 | 0.260622064 | 0.129754685 | 0.415602 | 0.248544479 |
| ENSMUSG00000028247 | Coq3 |  | 2990 | 4.676990941 | 4.612554584 | 4.756460844 | 3.321842028 | 4.3946539 | 3.778603096 | 4.588855147 | 4.100254573 | 5.49612702 | 4.46275887 | 3.563149077 | 3.882828702 | 4.982688635 | 5.871526767 | 4.368191712 | 4.891977267 | 4.306901592 | 3.495561966 |
| ENSMUSG00000028248 | Sfrs18 |  | 8234 | 38.95877623 | 40.71665866 | 40.54822765 | 39.76769804 | 39.62086211 | 36.63195158 | 44.57183027 | 43.21112713 | 46.51365194 | 38.77306434 | 42.38475488 | 49.23356295 | 39.06292211 | 38.00586116 | 39.0098773 | 42.65259304 | 44.47972956 | 41.11319441 |
| ENSMUSG00000028249 | Sdcbp |  | 6810 | 77.31448098 | 87.12226814 | 77.13482616 | 81.52148682 | 84.88464071 | 86.16063411 | 67.83568239 | 75.31923117 | 76.43394805 | 75.75961748 | 72.85505783 | 76.15846355 | 74.81645252 | 65.54004109 | 76.58891278 | 89.00738842 | 95.03345808 | 78.83830861 |
| ENSMUSG00000028251 | 2610029I01Ri | k | 1584 | 0.295174834 | 0.336493881 | 0.305388449 | 0.862898567 | 0.212704208 | 0.344116205 | 0.836335234 | 0.626895494 | 0.598036529 | 0.922772602 | 0.754023966 | 0.246660125 | 0.716309405 | 0.557094483 | 0.364155166 | 0.338427086 | 1.258186514 | 1.736398571 |
| ENSMUSG00000028252 | Ccnc |  | 5289 | 8.623190372 | 7.866856661 | 7.966221391 | 6.202297584 | 7.47141413 | 7.242250531 | 9.509052316 | 7.416070575 | 7.904010871 | 7.604376986 | 5.681688199 | 8.843547976 | 5.754923986 | 6.261038818 | 7.322643702 | 6.964561225 | 6.297135944 | 7.103652844 |
| ENSMUSG00000028255 | Clca3 |  | 2921 | 0 | 0.045618479 | 0.033121212 | 0 | 0 | 0.016964305 | 0.097184551 | 0.06537556 | 0 | 0 | 0.147201173 | 0 | 0 | 0 | 0 | 0 | 0.183693309 | 0 |
| ENSMUSG00000028256 | Odf2l |  | 3402 | 7.958788104 | 8.685637029 | 9.370416374 | 9.495232344 | 10.10176176 | 11.1282447 | 9.220548539 | 10.62022712 | 9.079919701 | 7.58125652 | 8.959560114 | 8.367427372 | 8.00447335 | 5.542710018 | 6.321931856 | 5.132427094 | 6.635564043 | 6.354667531 |
| ENSMUSG00000028259 | Fhl5 |  | 1164 | 0 | 0 | 0 | 0 | 0 | 0 | 0 | 0 | 0 | 0 | 0 | 0 | 0 | 0 | 0 | 0 | 0 | 0 |
| ENSMUSG00000028261 | Ndufaf4 |  | 2322 | 24.16314916 | 17.93329792 | 21.43684872 | 19.56260172 | 19.94096062 | 23.26118846 | 24.51212435 | 17.46784296 | 18.42928074 | 18.11301779 | 19.83422049 | 17.98023928 | 15.02052778 | 20.62181486 | 20.17492207 | 16.58929847 | 16.19211768 | 16.48851778 |
| ENSMUSG00000028262 | Clca2 |  | 3853 | 135.3921685 | 132.758757 | 175.6286831 | 158.3580342 | 156.4755972 | 188.7839544 | 102.2877279 | 118.0068909 | 153.7786701 | 101.1421133 | 107.0317782 | 158.2771683 | 8.309476852 | 9.558827225 | 13.27047258 | 9.649670331 | 13.38883873 | 16.38994407 |
| ENSMUSG00000028264 | Spaca1 |  | 1858 | 0 | 0 | 0 | 0 | 0 | 0 | 0 | 0 | 0 | 0 | 0 | 0 | 0 | 0.399949084 | 0 | 0 | 0 | 0 |
| ENSMUSG00000028266 | Lmo4 |  | 3948 | 1.754899618 | 1.518824964 | 1.862405424 | 1.765663572 | 1.243529943 | 1.418302658 | 2.384808284 | 2.437812919 | 2.076017385 | 2.245271584 | 2.00877485 | 2.304446049 | 4.385892341 | 3.540949925 | 3.089073086 | 2.715645915 | 2.320158298 | 2.730948561 |
| ENSMUSG00000028268 | Gbp3 |  | 3365 | 0.833682502 | 1.752268136 | 0.920031484 | 1.042555844 | 1.301635974 | 2.591762588 | 0.492108102 | 0.363196694 | 1.101571001 | 1.050907205 | 0.681485292 | 0.066348485 | 0.982240255 | 0.634896886 | 0.979531789 | 0.933084638 | 1.480659316 | 1.275099651 |
| ENSMUSG00000028269 | Gbp1 |  | 2812 | 0 | 0 | 0 | 0 | 0 | 0 | 0 | 0 | 0 | 0 | 0.016989672 | 0.019849098 | 0 | 0 | 0 | 0 | 0 | 0 |
| ENSMUSG00000028270 | Gbp2 |  | 2678 | 2.872829298 | 3.719400811 | 4.226813332 | 4.661585567 | 4.726921555 | 8.08608834 | 4.505128128 | 4.121584875 | 1.630235501 | 3.310056838 | 2.426211484 | 3.272240114 | 0.828953054 | 1.491483015 | 2.061616314 | 2.730958183 | 3.864115072 | 3.348201791 |
| ENSMUSG00000028271 | Gtf2b |  | 1264 | 86.22094251 | 74.42690924 | 77.99466366 | 78.32606752 | 74.52070777 | 74.4467097 | 69.92095272 | 73.27260746 | 75.56291935 | 76.88101219 | 70.52853839 | 72.59568773 | 71.9683983 | 81.38738415 | 68.51714437 | 81.79164556 | 77.71384412 | 76.59495873 |
| ENSMUSG00000028273 | Pdlim5 |  | 5350 | 27.82301429 | 34.0476459 | 34.21410072 | 34.06433221 | 34.13318101 | 36.4652546 | 30.66038464 | 34.42313353 | 32.93381578 | 33.73698917 | 29.56689486 | 36.50448661 | 19.94484805 | 19.89716791 | 26.0455443 | 23.00299293 | 29.0635115 | 23.16551728 |
| ENSMUSG00000028274 | Rngtt |  | 4600 | 9.942515088 | 9.675223191 | 10.23205327 | 10.88512777 | 9.270627037 | 10.61074839 | 10.67620885 | 7.298069938 | 8.362653418 | 8.302623834 | 9.960040257 | 10.65351125 | 6.885019759 | 5.280490805 | 7.64020156 | 8.174212276 | 10.09814605 | 9.73422019 |
| ENSMUSG00000028277 | Ube2j1 |  | 3610 | 26.12714283 | 30.71061273 | 30.69910195 | 39.86906899 | 32.67904291 | 31.99651578 | 24.77034991 | 26.7452613 | 26.13803535 | 25.82186713 | 31.21914877 | 25.65046655 | 19.92408183 | 22.16708104 | 26.82095744 | 25.06390728 | 27.78391108 | 29.11977921 |
| ENSMUSG00000028278 | Rragd |  | 5617 | 26.67451406 | 31.61077554 | 31.06343671 | 31.78809556 | 28.46612968 | 34.2378777 | 44.76889594 | 39.89226009 | 36.17843489 | 41.97982321 | 38.52105874 | 36.92558344 | 22.7733063 | 29.9319203 | 25.68037158 | 21.20060335 | 32.87450569 | 29.84026138 |
| ENSMUSG00000028280 | Gabrr1 |  | 2495 | 0 | 0.09346303 | 0 | 0.01826094 | 0 | 0.099304075 | 0 | 0 | 0 | 0 | 0 | 0 | 0 | 0 | 0 | 0 | 0 | 0 |
| ENSMUSG00000028282 | Casp8ap2 |  | 6837 | 4.370504424 | 3.439944905 | 3.127263481 | 2.752188286 | 3.069404522 | 3.660103914 | 3.778371025 | 3.234371946 | 3.837327854 | 3.848192543 | 3.78034994 | 4.302304254 | 4.834340292 | 4.687204958 | 3.477153393 | 4.502800695 | 5.028329431 | 3.910256818 |
| ENSMUSG00000028284 | Map3k7 |  | 6408 | 7.422486263 | 11.26025732 | 10.79496698 | 12.47098498 | 11.31190341 | 10.11469655 | 11.67311882 | 8.797126397 | 9.345379825 | 9.926076575 | 9.095731795 | 9.807808376 | 8.383654481 | 10.48760837 | 6.944082454 | 7.977209875 | 9.252618806 | 9.485808822 |
| ENSMUSG00000028287 | 1700009N14R |  | 1256 | 0 | 0 | 0 | 0 | 0 | 0 | 0 | 0 | 0 | 0 | 0 | 0 | 0 | 0 | 0 | 0 | 0 | 0 |
| ENSMUSG00000028289 | Epha7 |  | 7704 | 0 | 0.142695419 | 0.075348178 | 0.177418397 | 0.031238268 | 0.115777415 | 0.202663344 | 0 | 0 | 0 | 0.179838239 | 0 | 0 | 0.03014284 | 0.288795953 | 0.044732018 | 0.044773711 | 0 |
| ENSMUSG00000028291 | Akirin2 |  | 2028 | 28.25294738 | 33.00079117 | 32.48754849 | 41.40483467 | 29.61964533 | 31.49579565 | 33.45482327 | 38.96453677 | 31.09297615 | 34.50278286 | 36.89131409 | 35.42147818 | 25.22544291 | 31.05433062 | 30.08851296 | 32.13541114 | 34.03632186 | 38.3816006 |
| ENSMUSG00000028292 | Rars2 |  | 2816 | 17.40357527 | 16.50265889 | 13.72530211 | 14.88500028 | 15.02413384 | 15.59081035 | 19.32158408 | 17.23811913 | 21.33917844 | 17.32989271 | 18.06829928 | 19.18663401 | 20.95205009 | 18.48857314 | 21.31770843 | 18.57420799 | 20.7011 | 18.42101834 |
| ENSMUSG00000028293 | Slc35a1 |  | 2620 | 16.15845623 | 17.21590029 | 17.68772598 | 18.20702802 | 19.50996295 | 18.15672681 | 20.15303423 | 16.57433646 | 20.4203374 | 17.78049421 | 19.91231094 | 18.32117169 | 13.63217867 | 15.29818604 | 19.98747234 | 17.77151855 | 16.03267993 | 17.95144513 |
| ENSMUSG00000028294 | 1700003M02R |  | 2512 | 0 | 0.145876527 | 0.308111658 | 0.10882415 | 0.019160798 | 0 | 0 | 0 | 0 | 0 | 0.228224324 | 0 | 0 | 0 | 0.09841136 | 0 | 0.030514568 | 0 |
| ENSMUSG00000028295 | 1810030N24R |  | 1977 | 16.40437497 | 12.57026762 | 14.3628034 | 14.54173949 | 14.1936831 | 13.10879093 | 26.15718668 | 15.39673481 | 19.54119969 | 18.60270538 | 13.99175567 | 18.2381983 | 19.98728667 | 16.70295707 | 17.46429127 | 21.53725616 | 13.14376798 | 14.71917929 |
| ENSMUSG00000028298 | Cga |  | 1022 | 0 | 0 | 0 | 0 | 0 | 0 | 0 | 0 | 0 | 0 | 0 | 0 | 0 | 0 | 0 | 0 | 0 | 0 |
| ENSMUSG00000028300 | 3110043O21R |  | 6481 | 2.046229736 | 3.19713319 | 2.373519928 | 2.945545067 | 2.079453576 | 2.660754711 | 2.978486808 | 2.3689779 | 2.541985235 | 1.513245838 | 2.543181712 | 2.204722978 | 2.473827051 | 2.307513677 | 2.987923356 | 2.168284665 | 2.779410514 | 1.884280465 |
| ENSMUSG00000028307 | Aldob |  | 2382 | 0 | 0.013985262 | 0 | 0 | 0 | 0 | 0.198625856 | 0.112236279 | 0 | 0 | 0 | 0 | 0 | 0 | 0 | 0 | 0 | 0 |
| ENSMUSG00000028309 | Rnf20 |  | 7267 | 6.492465308 | 9.997993985 | 7.462051401 | 8.394968813 | 8.994516629 | 8.6190526 | 7.181214374 | 9.607226035 | 9.521583388 | 7.688672542 | 8.868641666 | 9.577835592 | 7.908584509 | 8.065561894 | 7.74478125 | 9.010174578 | 10.32125541 | 9.166922835 |
| ENSMUSG00000028310 | Ppp3r2 |  | 3024 | 0 | 0 | 0 | 0 | 0 | 0 | 0 | 0 | 0 | 0 | 0 | 0 | 0 | 0 | 0 | 0.025324476 | 0 | 0 |
| ENSMUSG00000028312 | Smc2 |  | 5649 | 2.580859511 | 3.119582411 | 2.842983197 | 3.024498429 | 2.675415831 | 2.377197876 | 2.303237158 | 2.886910219 | 2.981994149 | 2.754422389 | 3.9326174 | 2.578843678 | 3.641601966 | 2.672035476 | 4.813777109 | 2.996007872 | 3.650123515 | 2.522102787 |

| ENSMUSG00000028314 | 4930547C10R | 2771 | 0 | 0 | 0 | 0 | 0 | 0 | 0.017074226 | 0.055131581 | 0 | 0 | 0.017241053 | 0 | 0 | 0 | 0 | 0 | 0 | 0 |
| --- | --- | --- | --- | --- | --- | --- | --- | --- | --- | --- | --- | --- | --- | --- | --- | --- | --- | --- | --- | --- |
| ENSMUSG00000028318 | Polr1e | 4011 | 3.28511707 | 3.12282429 | 3.931630739 | 3.066936418 | 3.815993948 | 3.681554377 | 4.836249903 | 4.294383816 | 3.819841402 | 3.949797132 | 3.85915895 | 3.284092837 | 3.259276509 | 2.153727313 | 3.040553613 | 4.524993246 | 2.761480879 | 2.509764779 |
| ENSMUSG00000028322 | Exosc3 | 1553 | 24.38642102 | 22.30869928 | 22.58262042 | 22.50181649 | 23.27564371 | 22.718317 | 23.00133457 | 25.25666276 | 31.16172313 | 23.77269842 | 21.56487179 | 20.30640654 | 24.3641858 | 26.28741327 | 22.3385126 | 26.0366268 | 27.88713168 | 26.88468255 |
| ENSMUSG00000028327 | 1300002K09R | 7972 | 0.101304358 | 0.045966111 | 0.145630297 | 0.011430267 | 0.030188111 | 0.068374319 | 0 | 0 | 0 | 0 | 0 | 0 | 0 | 0 | 0.072355969 | 0 | 0 | 0 |
| ENSMUSG00000028328 | Tmod1 | 4032 | 70.23052646 | 82.10901358 | 69.42897326 | 69.14385672 | 68.1033295 | 70.01535794 | 25.78024693 | 29.55364472 | 25.16953741 | 28.0191183 | 27.18148679 | 25.91441477 | 26.59910421 | 21.43661871 | 26.72174322 | 32.48813689 | 26.11169087 | 24.43484875 |
| ENSMUSG00000028329 | Xpa | 1885 | 13.9804823 | 15.90536064 | 13.37008452 | 11.19085598 | 15.19283532 | 13.72229545 | 13.22747045 | 13.53449592 | 13.19716473 | 12.33172239 | 10.18861183 | 11.93300373 | 16.22588261 | 16.13839641 | 14.27300583 | 16.12877574 | 16.24547015 | 13.77416359 |
| ENSMUSG00000028330 | Ncbp1 | 3363 | 17.84635996 | 17.98876476 | 19.10200663 | 19.25133631 | 19.89395596 | 21.7925938 | 16.67128294 | 20.06719448 | 17.5253952 | 18.21253748 | 18.86563927 | 16.58038862 | 16.32662026 | 18.46439038 | 18.49965786 | 18.13765614 | 20.61619717 | 23.11265769 |
| ENSMUSG00000028331 | 5830415F09R | 2515 | 3.211126618 | 2.357731678 | 2.500421052 | 2.916631466 | 2.755863622 | 2.758402664 | 3.593129891 | 2.050089195 | 3.602796607 | 2.512204468 | 1.671648647 | 1.864220937 | 3.079567003 | 3.08396127 | 1.949497078 | 2.11626021 | 1.447713004 | 2.449709723 |
| ENSMUSG00000028332 | Hemgn | 2506 | 0 | 0 | 0 | 0 | 0 | 0.158189094 | 0 | 0.091442304 | 0 | 0.056445375 | 0 | 0 | 0 | 0 | 0.295940944 | 0 | 0 | 0 |
| ENSMUSG00000028333 | Anp32b | 1797 | 35.05435218 | 32.36745315 | 34.51022032 | 35.39411124 | 34.36463999 | 31.93214551 | 38.51889219 | 33.45288892 | 40.26973188 | 36.31417546 | 30.6004325 | 34.16651577 | 31.37806424 | 36.15763954 | 27.81162343 | 32.8996649 | 32.84501794 | 25.25459825 |
| ENSMUSG00000028334 | Nans | 4744 | 10.99364481 | 13.77738163 | 12.05259546 | 11.58233969 | 12.22575211 | 11.86591597 | 13.95245317 | 12.81667467 | 14.91535288 | 12.5927363 | 11.81282173 | 13.98921223 | 14.11117448 | 16.76059815 | 14.93816951 | 12.55098957 | 13.25747328 | 13.52036645 |
| ENSMUSG00000028337 | Coro2a | 8632 | 23.11883705 | 25.58671092 | 26.85986226 | 25.43015658 | 22.93403638 | 25.62023285 | 25.47605788 | 24.58702273 | 26.27596644 | 25.98423255 | 27.99971211 | 25.34724251 | 28.63788664 | 27.94070271 | 29.35467664 | 22.51658053 | 27.93219808 | 25.62460822 |
| ENSMUSG00000028339 | Col15a1 | 5937 | 0.007159369 | 0 | 0.016295614 | 0.007674085 | 0.064856896 | 0 | 0 | 0.012865893 | 0 | 0 | 0 | 0.056407946 | 0 | 0 | 0 | 0 | 0 | 0 |
| ENSMUSG00000028341 | Nr4a3 | 6463 | 0.052613555 | 0.185558439 | 0.134724361 | 0.162138948 | 0.201077199 | 0.222347095 | 0.029282178 | 0.342744753 | 0.031863311 | 0.036477412 | 0.044352429 | 0.164087512 | 0.099228675 | 0.610822749 | 0.082874861 | 0.201435967 | 0.28464525 | 0.383012502 |
| ENSMUSG00000028343 | Erp44 | 3623 | 38.34030231 | 34.57258688 | 40.58943756 | 36.45638077 | 32.93376726 | 35.72501797 | 37.80574262 | 36.60061307 | 38.2649219 | 33.98026117 | 35.59056388 | 36.58906946 | 36.66874226 | 30.66360945 | 31.4100738 | 30.18437062 | 34.22179699 | 29.75927386 |
| ENSMUSG00000028344 | Invs | 11702 | 0.719195424 | 0.762934169 | 0.545659375 | 0.96167988 | 0.876098081 | 0.618244668 | 1.006738767 | 1.285917497 | 1.252982363 | 1.1120829 | 1.175797987 | 0.958720576 | 1.222547941 | 0.603273054 | 0.982330721 | 0.798402684 | 0.815522811 | 0.531193733 |
| ENSMUSG00000028345 | Tex10 | 4493 | 7.085772723 | 7.436642088 | 6.545984077 | 7.818287377 | 7.670255363 | 6.959219873 | 7.666065048 | 7.658881497 | 7.98429896 | 7.786739777 | 6.294853198 | 7.267340889 | 6.423140798 | 6.63634634 | 9.096804684 | 6.38318828 | 10.21920848 | 6.807269828 |
| ENSMUSG00000028347 | Tmeff1 | 2986 | 0.868324092 | 0.591287138 | 0.583203983 | 0.442488374 | 0.32238395 | 0.51444566 | 0.221827698 | 0.01279049 | 0.372416586 | 0.047371772 | 0.67198535 | 0 | 0.297379274 | 0.12443158 | 0.579526238 | 0.051293513 | 0.385059915 | 0.478980835 |
| ENSMUSG00000028348 | Murc | 2052 | 0.041428047 | 0 | 0 | 0 | 0 | 0 | 0 | 0 | 0 | 0 | 0.139692861 | 0 | 0 | 0 | 0 | 0 | 0 | 0 |
| ENSMUSG00000028351 | Dbc1 | 6173 | 0 | 0 | 0 | 0 | 0 | 0 | 0 | 0 | 0 | 0 | 0 | 0 | 0 | 0 | 0 | 0 | 0 | 0 |
| ENSMUSG00000028354 | Fmn2 | 8157 | 0 | 0 | 0.023721236 | 0 | 0 | 0 | 0.01160051 | 0 | 0 | 0 | 0.005856928 | 0 | 0 | 0 | 0 | 0 | 0 | 0 |
| ENSMUSG00000028356 | Ambp | 1398 | 0 | 0 | 0 | 0 | 0 | 0 | 0 | 0 | 0 | 0 | 0.13669516 | 0 | 0 | 0 | 0 | 0 | 0 | 0 |
| ENSMUSG00000028357 | Kif12 | 3193 | 0 | 0.146063426 | 0.151498686 | 0.04280712 | 0.060296804 | 0 | 0.93351042 | 0.77748392 | 0.851334181 | 0.103368281 | 0.179548857 | 0.891512306 | 0 | 0 | 0 | 0 | 0 | 0 |
| ENSMUSG00000028358 | Zfp618 | 9465 | 0.202084831 | 0.29212575 | 0.229985089 | 0.221427157 | 0.345797233 | 0.287945097 | 0.794793022 | 0.504389889 | 0.609203614 | 0.169373894 | 0.242281881 | 0.312544122 | 0.547263742 | 0.049069295 | 0.213299127 | 0.121364841 | 0.327990496 | 0.17435533 |
| ENSMUSG00000028359 | Orm3 | 767 | 0 | 0.130298152 | 0 | 0.297008112 | 0 | 0 | 0 | 0.099589055 | 0 | 0 | 0 | 0 | 0 | 0 | 0 | 0.049922565 | 0.099938193 | 0 |
| ENSMUSG00000028360 | Slc44a5 | 7119 | 0 | 0 | 0 | 0 | 0 | 0 | 0 | 0 | 0 | 0 | 0 | 0 | 0 | 0 | 0 | 0 | 0 | 0 |
| ENSMUSG00000028362 | Tnfsf8 | 3803 | 0 | 0 | 0 | 0 | 0 | 0 | 0 | 0 | 0 | 0 | 0 | 0 | 0 | 0 | 0 | 0 | 0 | 0 |
| ENSMUSG00000028364 | Tnc | 7483 | 6.827638859 | 6.793462058 | 7.401803054 | 7.68991033 | 8.168855146 | 8.82055875 | 1.258215035 | 2.337581224 | 1.733763526 | 1.726485727 | 2.209025208 | 2.521140446 | 8.121999477 | 5.312860992 | 9.833753685 | 12.33711806 | 5.316410021 | 4.535696759 |
| ENSMUSG00000028367 | Txn1 | 1046 | 218.1336376 | 197.2021425 | 197.8412647 | 178.4546832 | 208.034825 | 186.6991616 | 215.8019036 | 196.7675493 | 197.3487734 | 202.8922709 | 200.1432773 | 184.3090809 | 242.5564537 | 222.0968691 | 204.4321941 | 218.3223542 | 176.0591363 | 219.826067 |
| ENSMUSG00000028369 | Svep1 | 14892 | 0 | 0 | 0 | 0 | 0 | 0 | 0.006354107 | 0 | 0 | 0 | 0.012832382 | 0.01499212 | 0 | 0 | 0 | 0 | 0 | 0 |
| ENSMUSG00000028370 | Pappa | 11091 | 2.24578786 | 2.360827234 | 2.468615831 | 2.193634269 | 2.369491509 | 2.367951384 | 1.446127325 | 1.880177776 | 1.919883568 | 1.730262035 | 1.326723218 | 2.148885396 | 0.04003131 | 0.25125284 | 0.430924216 | 0.217501422 | 0.317917168 | 0.178552686 |
| ENSMUSG00000028373 | Astn2 | 5201 | 0 | 0.03202547 | 0.130211387 | 0.026280164 | 0 | 0.057165238 | 0.181936854 | 0.044059684 | 0.126703375 | 0 | 0 | 0.032195152 | 0.009485083 | 0.22324595 | 0.023765558 | 0.007362163 | 0.140011467 | 0.095189764 |
| ENSMUSG00000028378 | Ptgr1 | 2286 | 0.706560231 | 0.451749659 | 0.338572391 | 0.956662348 | 1.305415253 | 1.799158743 | 0.641598008 | 0.534626805 | 1.026960367 | 0.123755127 | 0.438877571 | 0.366244505 | 2.913302201 | 7.639097488 | 3.226193572 | 2.026755685 | 3.654913703 | 1.876951148 |
| ENSMUSG00000028381 | Ugcg | 5386 | 4.308916842 | 5.566580913 | 5.55047192 | 5.075496955 | 6.130430679 | 5.934183654 | 5.244275774 | 4.51699969 | 3.35701456 | 3.641792157 | 6.386552192 | 3.834347411 | 3.856059631 | 3.871777801 | 3.205245436 | 6.796476174 | 5.956017547 | 4.207900477 |
| ENSMUSG00000028382 | Rod1 | 12431 | 35.89227196 | 43.41047168 | 37.50495419 | 39.68953014 | 42.5292454 | 38.50691066 | 26.75253963 | 31.93402225 | 27.13187817 | 29.79396461 | 28.71647412 | 31.70415844 | 24.65603749 | 25.64493732 | 27.07881197 | 32.9340499 | 35.92146128 | 27.66164638 |
| ENSMUSG00000028383 | Hsdl2 | 2967 | 42.99225927 | 41.2958628 | 42.50414346 | 42.4745024 | 39.82604402 | 45.81164415 | 38.35085703 | 37.69037892 | 37.20251508 | 44.36965403 | 35.95601021 | 37.15400526 | 45.47448391 | 45.61444916 | 43.85389737 | 61.43016219 | 46.91645108 | 49.92906114 |
| ENSMUSG00000028385 | Snx30 | 8129 | 2.917688304 | 2.405544213 | 1.660255255 | 2.370810894 | 2.55195708 | 2.657767479 | 4.487400105 | 2.847163963 | 1.854381199 | 2.702943501 | 1.962951917 | 3.446852333 | 2.791571158 | 2.902398375 | 2.589985861 | 3.0900035 | 2.465820308 | 2.781245278 |
| ENSMUSG00000028386 | Slc46a2 | 2829 | 2.839688324 | 2.178467809 | 2.581973518 | 2.882794958 | 2.43296751 | 3.71338972 | 5.117596236 | 5.251624085 | 3.319379849 | 4.60006861 | 4.593421246 | 6.767328479 | 5.353445954 | 11.0815691 | 4.208995989 | 1.813694377 | 2.262457258 | 5.502855144 |
| ENSMUSG00000028389 | Zfp37 | 3820 | 4.384041716 | 5.171347192 | 4.330823922 | 4.448761659 | 5.065191967 | 5.396318631 | 5.462013457 | 3.739256152 | 4.086306145 | 3.616532999 | 5.915590408 | 4.149646119 | 3.422240335 | 3.063850791 | 3.580868208 | 3.909250494 | 5.12689469 | 4.464089813 |
| ENSMUSG00000028391 | Wdr31 | 4717 | 1.531880417 | 0.918099692 | 1.240872837 | 1.487471026 | 0.969373065 | 1.229101087 | 1.584779154 | 1.0525784 | 1.344651988 | 1.629333184 | 1.306544338 | 2.03525418 | 1.621040318 | 1.427685536 | 0.97828583 | 1.298812208 | 1.03189309 | 1.667649409 |
| ENSMUSG00000028392 | Bspry | 2320 | 42.61510321 | 50.09857238 | 40.99239684 | 48.34969446 | 49.75015214 | 48.95468335 | 42.17354311 | 44.02003622 | 38.27505517 | 37.11085524 | 51.15215382 | 34.90884759 | 41.69822835 | 50.58802105 | 43.52802306 | 42.00413614 | 40.95296984 | 52.02154317 |

| ENSMUSG00000028393 | Alad |  | 1860 | 349.7079079 | 415.5156698 | 351.3062624 | 267.7323734 | 449.5159387 | 320.5209342 | 279.9842242 | 288.496373 | 267.6902092 | 212.8879602 | 329.1848752 | 238.8370207 | 338.798877 | 408.5331794 | 278.0661995 | 184.8036466 | 287.8593375 | 290.2174001 |
| --- | --- | --- | --- | --- | --- | --- | --- | --- | --- | --- | --- | --- | --- | --- | --- | --- | --- | --- | --- | --- | --- |
| ENSMUSG00000028394 | Pole3 |  | 2037 | 30.90337049 | 29.63326673 | 27.83199685 | 32.0068014 | 29.55966446 | 32.67026077 | 33.84122396 | 33.03633433 | 36.27324952 | 32.29024597 | 27.13580116 | 33.51131828 | 36.61750561 | 36.73118546 | 34.68859517 | 30.94076573 | 32.51244038 | 27.8961253 |
| ENSMUSG00000028396 | 2310002L09R |  | 1177 | 0 | 0 | 0 | 0 | 0 | 0 | 0 | 0 | 0 | 0 | 0 | 0 | 0 | 0 | 0 | 0 | 0 | 0 |
| ENSMUSG00000028397 | Kdm4c |  | 5797 | 6.753021756 | 6.533855568 | 8.795187337 | 6.821974553 | 8.917316891 | 8.539448136 | 8.316649962 | 7.701728206 | 7.722915755 | 7.141334735 | 9.263248806 | 7.481243717 | 6.722824676 | 7.202559007 | 7.064748414 | 7.391269567 | 6.981640423 | 7.724238895 |
| ENSMUSG00000028398 | 3110001D03R |  | 739 | 48.71702855 | 42.73426755 | 40.58401735 | 32.49075827 | 42.20498855 | 41.17101274 | 49.87358171 | 53.54171704 | 48.54324118 | 49.12860827 | 44.73649698 | 36.10268838 | 47.06224855 | 61.90450078 | 41.42456837 | 41.9694073 | 39.67471872 | 42.95027683 |
| ENSMUSG00000028399 | Ptprd |  | 26192 | 0.590710296 | 0.789833052 | 0.622399196 | 0.462707613 | 0.84716008 | 0.56378721 | 0.30889081 | 0.450574692 | 0.50634003 | 0.232225134 | 0.580041112 | 0.375059431 | 0.17327949 | 0.52664531 | 0.303600347 | 0.30700319 | 0.380453467 | 0.065106991 |
| ENSMUSG00000028402 | Mpdz |  | 12517 | 0.186768769 | 0.266141202 | 0.139126555 | 0.036399332 | 0.346079183 | 0.431512979 | 0.412006232 | 0.17086958 | 0.18426499 | 0.094173331 | 0.156489039 | 0.182826729 | 0.122176994 | 0.311680382 | 0.243581939 | 0.214136176 | 0.088796246 | 0.05273702 |
| ENSMUSG00000028403 | Zdhhc21 |  | 15374 | 1.761142001 | 2.010821246 | 1.529174955 | 1.659567115 | 1.621720857 | 1.373062606 | 1.969566444 | 1.860680981 | 1.615420125 | 1.757340508 | 1.855187343 | 1.967743536 | 1.158372716 | 2.2173774 | 1.066620219 | 2.112035585 | 1.6353617 | 1.445536563 |
| ENSMUSG00000028405 | Aco1 |  | 5450 | 18.98304559 | 23.33736333 | 20.92044239 | 20.8243232 | 19.95929312 | 21.30312932 | 17.31904486 | 21.78018104 | 21.13737332 | 18.99870539 | 16.71685244 | 20.24725962 | 16.91767952 | 18.83329048 | 18.81663718 | 14.39586322 | 16.83544125 | 16.25039666 |
| ENSMUSG00000028407 | 2010003O02R |  | 1052 | 7.393961229 | 5.034933632 | 6.023700073 | 4.937223436 | 8.738780818 | 5.935023223 | 9.939260497 | 9.148750386 | 9.396163292 | 7.305669801 | 9.264345561 | 8.22379059 | 11.67647091 | 12.44984092 | 11.55366824 | 8.298724798 | 9.800165273 | 9.673654699 |
| ENSMUSG00000028409 | Smu1 |  | 3106 | 49.88131572 | 51.71757113 | 55.41307824 | 52.08862474 | 55.39975116 | 58.88575004 | 46.30732258 | 47.67287319 | 51.84780316 | 49.09381015 | 52.14330626 | 48.35800001 | 40.67580172 | 45.83101347 | 48.36473689 | 48.38719705 | 51.02357942 | 51.39614543 |
| ENSMUSG00000028410 | Dnaja1 |  | 8533 | 16.92137384 | 15.7409574 | 15.43667211 | 14.48043504 | 15.63596536 | 15.16256737 | 14.94288875 | 15.50880984 | 16.08750229 | 14.40547092 | 13.50441812 | 13.24583578 | 17.47689979 | 21.58101564 | 16.52794162 | 20.05848061 | 19.10251268 | 22.4600642 |
| ENSMUSG00000028411 | Aptx |  | 7664 | 6.455639992 | 6.359181138 | 6.248668457 | 6.158825931 | 6.581713988 | 5.890206196 | 7.025290791 | 6.024871413 | 7.867570339 | 7.204263246 | 7.536524635 | 5.403865036 | 6.037752923 | 6.399394087 | 5.107186599 | 6.155275094 | 7.696264475 | 4.55777437 |
| ENSMUSG00000028412 | Slc44a1 |  | 6978 | 18.25566245 | 19.49697048 | 20.24229129 | 20.58016792 | 19.76181733 | 21.58079068 | 13.04522704 | 14.15384818 | 13.5458661 | 15.07498122 | 14.58306987 | 18.13329136 | 13.60197891 | 12.19340328 | 17.7193851 | 14.78836156 | 16.31806078 | 14.37110942 |
| ENSMUSG00000028413 | B4galt1 |  | 4227 | 7.280280919 | 8.464170426 | 9.441249714 | 9.495926203 | 6.729587624 | 9.413495402 | 4.085433596 | 6.442200832 | 4.238498783 | 5.331922441 | 5.14256118 | 6.826755987 | 4.749962227 | 3.472044385 | 4.357013362 | 5.93336831 | 5.313274186 | 5.348649838 |
| ENSMUSG00000028414 | Fktn |  | 6518 | 12.05769722 | 11.10600171 | 10.36787694 | 14.07093928 | 12.33205163 | 12.7645044 | 10.23486918 | 11.79522952 | 9.054967335 | 10.19259602 | 9.726506582 | 12.46818115 | 7.916720692 | 7.602921332 | 10.20241043 | 10.0631805 | 10.98397094 | 9.908053282 |
| ENSMUSG00000028415 | Spink4 |  | 574 | 0 | 0.058036401 | 0 | 0 | 0.251560577 | 0.172657608 | 0.41213135 | 0.332686432 | 0 | 0.082144082 | 0.249694905 | 0.583438982 | 0 | 0 | 0 | 0.133416751 | 0 | 0 |
| ENSMUSG00000028416 | Bag1 |  | 1604 | 108.6213944 | 100.0632945 | 93.88204488 | 97.5699422 | 96.11381017 | 95.12024108 | 125.8623448 | 102.3623054 | 121.6850986 | 102.9437424 | 92.33314918 | 94.82399037 | 119.1162816 | 128.2713885 | 114.5629297 | 104.2488046 | 98.08569126 | 119.8265704 |
| ENSMUSG00000028417 | Tal2 |  | 2969 | 0.114530619 | 0.011220241 | 0.130342958 | 0 | 0.19453792 | 0.10014025 | 0 | 0.025727452 | 0.013872184 | 0.015881005 | 0.112638838 | 0 | 0 | 0.078215034 | 0.041631751 | 0 | 0 | 0 |
| ENSMUSG00000028419 | Chmp5 |  | 1511 | 163.0443417 | 154.2843374 | 157.6381625 | 140.964846 | 145.6062363 | 148.1662808 | 156.2163833 | 142.7854938 | 162.1293155 | 149.2221462 | 146.1388869 | 135.8636711 | 165.9197906 | 139.9777458 | 149.0727373 | 150.3495524 | 155.4358355 | 134.3008569 |
| ENSMUSG00000028420 | Tmem38b |  | 3962 | 3.711961359 | 3.808869531 | 3.259902222 | 3.702841059 | 2.101671579 | 3.039200974 | 2.686863392 | 2.62199229 | 3.056243219 | 2.463451685 | 4.304810746 | 2.831637602 | 1.780531088 | 2.871984203 | 3.650112599 | 3.40189142 | 2.940735552 | 2.846256153 |
| ENSMUSG00000028423 | Nfx1 |  | 8539 | 13.69384462 | 12.74152858 | 15.13124483 | 14.58764436 | 14.17070572 | 14.39750931 | 13.76328545 | 15.77521997 | 15.99420146 | 15.67640784 | 17.23233073 | 16.75973286 | 16.24562026 | 16.79580066 | 15.2618013 | 17.88300062 | 17.92211073 | 18.22470581 |
| ENSMUSG00000028426 | Rad23b |  | 5473 | 10.7175485 | 7.778892529 | 12.01162575 | 10.25602167 | 7.572005532 | 9.588223069 | 9.604309572 | 8.953197933 | 11.08491462 | 10.2261803 | 7.768995623 | 9.790432319 | 10.18546803 | 7.908987657 | 9.7790647 | 8.395528756 | 7.429965469 | 10.90533177 |
| ENSMUSG00000028427 | Aqp7 |  | 2818 | 0 | 0 | 0 | 0 | 0 | 0 | 0.016789453 | 0.013553017 | 0 | 0.016731974 | 0 | 0.099034178 | 0 | 0.131849787 | 0.043862551 | 0.027175733 | 0 | 0 |
| ENSMUSG00000028430 | Nol6 |  | 5217 | 6.93346843 | 7.892416572 | 8.029802049 | 7.03894992 | 8.404865337 | 8.567484318 | 5.033263716 | 6.164079514 | 5.747322882 | 7.167051501 | 6.163033747 | 4.546991873 | 5.834347893 | 5.475007427 | 7.755401166 | 3.875300117 | 5.950603889 | 4.924142772 |
| ENSMUSG00000028431 | Ikbkap |  | 8069 | 3.402942584 | 4.995489157 | 4.849942503 | 5.335876428 | 4.676592906 | 4.34177502 | 4.303817863 | 4.330901995 | 4.405002243 | 4.867584064 | 6.583932808 | 3.852933952 | 3.723278922 | 6.066683369 | 3.936844664 | 4.180694649 | 5.129805509 | 4.990291997 |
| ENSMUSG00000028433 | Ubap2 |  | 5701 | 10.0205151 | 14.40968201 | 12.41369495 | 15.04049911 | 10.83200457 | 14.16785752 | 11.50243343 | 11.56289889 | 10.80775786 | 11.38034866 | 7.793494367 | 10.73039225 | 7.779230597 | 8.757655489 | 9.084433565 | 9.450076235 | 10.31267136 | 9.996392662 |
| ENSMUSG00000028434 | Epb4.1l4b |  | 8953 | 7.059666788 | 7.586841429 | 7.12122339 | 8.213506708 | 7.107159956 | 7.997732606 | 5.073179021 | 5.793061819 | 6.527830433 | 6.957006479 | 5.821789311 | 6.888898376 | 6.253962483 | 5.623298417 | 6.171259516 | 8.177330655 | 6.30138602 | 6.463708285 |
| ENSMUSG00000028435 | Aqp3 |  | 1823 | 0.256476652 | 0.091368333 | 0.610307843 | 0.049984689 | 0 | 0.054363942 | 0 | 0.817061818 | 0.338890695 | 0.465558233 | 0.026206779 | 0 | 0.811825298 | 0 | 0.045201926 | 0.273054249 | 0.168190003 | 0 |
| ENSMUSG00000028436 | Dcaf12 |  | 3433 | 31.80768926 | 35.0789725 | 32.54962281 | 38.03610634 | 33.91527042 | 35.23397106 | 32.42841058 | 28.03520363 | 29.53719831 | 29.7352964 | 29.62798911 | 29.73692015 | 26.87174055 | 23.33703779 | 27.01564297 | 30.82879081 | 29.31688193 | 27.06390366 |
| ENSMUSG00000028437 | Ubap1 |  | 3758 | 37.76604118 | 37.26647347 | 36.69849256 | 39.6446554 | 36.74573949 | 41.53568674 | 30.26601388 | 35.08253675 | 29.41580849 | 33.1987123 | 34.59171422 | 31.24964185 | 31.26892681 | 32.15740165 | 32.145546 | 37.68945098 | 33.4819672 | 35.80422433 |
| ENSMUSG00000028438 | Kif24 |  | 9499 | 0.214785604 | 0.126251626 | 0.050924866 | 0.081538873 | 0.136810395 | 0.146065537 | 0.164366607 | 0.040206761 | 0.09972522 | 0.163803896 | 0.115677866 | 0.176278543 | 0.140221262 | 0.136902247 | 0.177835961 | 0.161240583 | 0.201738588 | 0.034746251 |
| ENSMUSG00000028439 | 2310028H24R |  | 6420 | 0.357520172 | 0.010377849 | 0.143161538 | 0.049677151 | 0.134949319 | 0.061747955 | 0.140021947 | 0.154673281 | 0.064153451 | 0.066099117 | 0.126506899 | 0.11302237 | 0.176734283 | 0.224262727 | 0.057759191 | 0.232606494 | 0.214913814 | 0.042841983 |
| ENSMUSG00000028441 | 1110017D15R |  | 1354 | 0.062784603 | 0.93492613 | 0.357263887 | 0.471089085 | 0.497671293 | 0 | 0 | 0.225656735 | 0.152092008 | 0 | 0 | 0.164891175 | 0.109302623 | 0.205808364 | 0.182577057 | 0.113118486 | 0.25475382 | 0.04062711 |
| ENSMUSG00000028443 | Nudt2 |  | 842 | 29.88487436 | 32.60074209 | 29.4147833 | 27.10936248 | 31.8974031 | 27.3658208 | 31.80400983 | 36.24195905 | 39.81689291 | 38.02295429 | 34.38435254 | 34.53676984 | 38.90307966 | 36.57058188 | 34.98706943 | 32.87898949 | 30.95235379 | 35.93231437 |
| ENSMUSG00000028444 | Cntfr |  | 2769 | 0.153503705 | 0.132337247 | 0.052409025 | 0 | 0 | 0.017895534 | 0 | 0.068964251 | 0 | 0.017028062 | 0 | 0 | 0 | 0.26836598 | 0 | 0.013828316 | 0 | 0 |
| ENSMUSG00000028445 | Enho |  | 2035 | 1.19056267 | 0.965828383 | 1.069684946 | 1.791097566 | 1.348166904 | 2.2645721 | 1.697211578 | 0.412890837 | 1.315539815 | 2.131628845 | 1.549458113 | 0.795407477 | 1.915096543 | 1.323713547 | 1.498238396 | 2.088578586 | 1.487851329 | 2.351740681 |
| ENSMUSG00000028447 | Dctn3 |  | 1475 | 99.01544734 | 74.93707323 | 74.57722578 | 80.06388266 | 71.72472422 | 78.98201806 | 98.85944167 | 73.53655785 | 71.20380691 | 81.77050775 | 71.28995499 | 76.62828253 | 99.63375021 | 80.92291837 | 68.5482157 | 87.92561849 | 64.18030736 | 82.49501293 |
| ENSMUSG00000028451 | 1700022I11Ri | k | 4475 | 0 | 0 | 0.021619455 | 0 | 0 | 0 | 0 | 0 | 0 | 0.01053647 | 0 | 0.012472774 | 0 | 0.020757134 | 0.147312826 | 0 | 0.171290712 | 0 |
| ENSMUSG00000028452 | Vcp |  | 4760 | 46.4163666 | 51.65598156 | 51.77796999 | 50.14586371 | 50.33628488 | 53.52944455 | 43.93320186 | 43.45589316 | 42.09504185 | 45.75401226 | 49.52135402 | 45.25055503 | 40.7091956 | 39.40917415 | 46.8105072 | 39.63399636 | 43.12513576 | 41.16437776 |
| ENSMUSG00000028453 | Fancg |  | 3277 | 3.074069795 | 6.068903829 | 5.830803934 | 4.74101804 | 4.053833058 | 3.553522243 | 5.601867386 | 5.873961188 | 5.781445606 | 5.942398671 | 4.257030173 | 4.15594192 | 4.410818359 | 4.81873351 | 6.009869916 | 5.398309618 | 4.561262067 | 4.431615555 |

| ENSMUSG00000028454 | Pigo | 3682 | 14.68402605 | 19.64212204 | 19.99579392 | 20.28097547 | 21.58223954 | 20.14677951 | 14.80288053 | 20.25794728 | 16.67818984 | 19.72082646 | 18.54166639 | 19.2368484 | 13.41152884 | 15.02306032 | 19.78390842 | 15.27672522 | 17.28951091 | 13.52070657 |
| --- | --- | --- | --- | --- | --- | --- | --- | --- | --- | --- | --- | --- | --- | --- | --- | --- | --- | --- | --- | --- |
| ENSMUSG00000028455 | Stoml2 | 3284 | 26.79223946 | 26.07007861 | 27.95766158 | 31.67352747 | 26.58687866 | 26.79831146 | 30.03865273 | 32.20303357 | 31.07801037 | 31.81667431 | 33.75088419 | 31.40905739 | 33.33359453 | 32.81068293 | 31.7543051 | 29.04442845 | 32.16421263 | 36.41589465 |
| ENSMUSG00000028456 | Unc13b | 11170 | 5.54812414 | 5.591913757 | 6.153875258 | 6.905536979 | 7.053890693 | 7.31978607 | 6.412837591 | 6.079327796 | 5.228512028 | 6.171381214 | 6.120498262 | 5.831412555 | 5.198179645 | 5.704681101 | 5.138206567 | 5.786440939 | 6.399153422 | 6.426757768 |
| ENSMUSG00000028457 | Atp8b5 | 6515 | 0.300113292 | 0.731196297 | 0.638545451 | 0.629392784 | 0.413720296 | 0.486780499 | 0.595493426 | 0.709329346 | 1.125280093 | 1.034927178 | 0.344654344 | 1.207982874 | 1.461405991 | 0.869712765 | 1.751777587 | 0.581852668 | 1.205969434 | 0.987884188 |
| ENSMUSG00000028458 | Tesk1 | 4737 | 10.26513012 | 11.68096206 | 9.762527975 | 12.34966876 | 10.41486632 | 12.67847015 | 9.438564827 | 11.02959816 | 9.520632206 | 12.51180789 | 10.0754029 | 9.980127974 | 11.11191804 | 9.402550096 | 10.73310533 | 9.756546999 | 9.215463832 | 11.7984489 |
| ENSMUSG00000028459 | Cd72 | 3727 | 1.699294668 | 1.215603331 | 1.84304838 | 1.210234341 | 1.330181953 | 1.887976434 | 1.866102442 | 1.11697662 | 0.983525598 | 2.542873988 | 1.43568429 | 1.857027679 | 2.50167222 | 2.093535466 | 1.558738766 | 1.530802389 | 1.7070473 | 1.564519805 |
| ENSMUSG00000028460 | Sit1 | 1918 | 0 | 0.069474232 | 0 | 0.071263364 | 0 | 0 | 0 | 0 | 0 | 0 | 0 | 0 | 0 | 0 | 0.02148152 | 0.059891461 | 0 | 0 |
| ENSMUSG00000028461 | Ccdc107 | 1542 | 40.35510876 | 32.40553912 | 32.68820923 | 27.41935742 | 28.96655328 | 32.52098987 | 45.1035266 | 36.48340385 | 36.37875128 | 34.61387554 | 32.65551637 | 36.59509395 | 52.75507885 | 49.87769696 | 36.92642687 | 29.79813806 | 22.49370862 | 40.52551572 |
| ENSMUSG00000028463 | Car9 | 2647 | 0 | 0.012585151 | 0.146198807 | 0.017212333 | 0.03636715 | 0.018720338 | 0.053622228 | 0.028857123 | 0 | 0 | 0 | 0 | 0 | 0.070183736 | 0.062261512 | 0 | 0 | 0.249380159 |
| ENSMUSG00000028464 | Tpm2 | 2886 | 0.103096408 | 0.069257576 | 0.184375895 | 0.236803765 | 0.033355456 | 0.085850197 | 0 | 0.026467361 | 0.185524846 | 0.13070188 | 0.231756555 | 0 | 0 | 0.370136525 | 0 | 0 | 0 | 0.019060675 |
| ENSMUSG00000028465 | Tln1 | 9742 | 7.652851442 | 8.042694232 | 8.327079697 | 8.067419573 | 7.134315107 | 8.825086508 | 5.619048401 | 6.876357403 | 7.102581244 | 6.572639604 | 6.056464147 | 6.984119554 | 5.499328902 | 6.626696928 | 6.728769803 | 5.801368972 | 6.892596199 | 5.968448553 |
| ENSMUSG00000028466 | Creb3 | 2343 | 51.97495916 | 44.84373383 | 42.22102841 | 37.08276208 | 43.14000841 | 42.48887826 | 36.30738229 | 39.18674152 | 36.07116105 | 41.97881647 | 39.78187964 | 41.97490289 | 47.79490065 | 44.77899847 | 41.53567009 | 50.59655165 | 41.18890977 | 41.27443859 |
| ENSMUSG00000028467 | Gba2 | 4639 | 5.60749466 | 6.376773026 | 5.756022582 | 7.719547499 | 5.91403244 | 8.182236241 | 7.200420634 | 5.878287415 | 4.199444267 | 6.891178445 | 5.952560035 | 7.363480384 | 7.252504329 | 9.100598283 | 7.202944994 | 7.048971485 | 6.245889299 | 7.019916185 |
| ENSMUSG00000028468 | Rgp1 | 6452 | 2.674690246 | 2.184028868 | 2.451665286 | 1.885430694 | 1.656120126 | 1.436199811 | 2.500561611 | 1.681128686 | 1.346924183 | 1.93659894 | 1.443911485 | 2.318443517 | 2.133230769 | 2.267496517 | 1.647551367 | 2.617197437 | 2.043435545 | 2.532192296 |
| ENSMUSG00000028469 | Npr2 | 5096 | 0.316953824 | 1.261653961 | 0.882797944 | 0.411265314 | 0.972839117 | 0.476468592 | 0.204254108 | 0.239826703 | 0.064657011 | 0.305332262 | 0.10312491 | 0.580500415 | 0 | 0 | 0.145531398 | 0.187846386 | 0.082729448 | 0.334631536 |
| ENSMUSG00000028470 | Hint2 | 1721 | 53.81683821 | 41.22978773 | 43.45466469 | 44.21089137 | 48.94297874 | 44.48516756 | 59.54634663 | 48.37852251 | 53.36776883 | 56.16440539 | 56.18623821 | 51.43732764 | 63.75025219 | 61.90745873 | 60.83274464 | 62.25282947 | 52.69030707 | 60.41093068 |
| ENSMUSG00000028475 | 5430416O09R | 4084 | 0 | 0 | 0 | 0 | 0 | 0 | 0 | 0 | 0 | 0 | 0 | 0 | 0 | 0 | 0 | 0 | 0 | 0 |
| ENSMUSG00000028476 | Reck | 4756 | 1.286952344 | 1.702067556 | 1.37309222 | 1.302838947 | 1.528158216 | 1.510754073 | 0.845579838 | 1.051977442 | 1.576103 | 1.685369965 | 1.486689204 | 1.150112475 | 2.956180913 | 2.226503769 | 2.04448427 | 2.085211799 | 2.014628727 | 1.827468222 |
| ENSMUSG00000028478 | Clta | 1788 | 60.38207333 | 58.44661586 | 53.54094887 | 51.49824978 | 54.16187384 | 57.97780685 | 61.49589811 | 46.95016806 | 57.05760601 | 57.19791687 | 54.26786391 | 53.34953432 | 61.08549488 | 57.01609162 | 58.18452384 | 61.09793228 | 52.75224646 | 60.1469819 |
| ENSMUSG00000028479 | Gne | 6708 | 7.508740846 | 7.086687545 | 8.689639845 | 7.783684676 | 6.938516724 | 9.241274554 | 11.32739451 | 9.940956261 | 8.307298126 | 11.91419827 | 9.102026973 | 9.252685867 | 10.27380567 | 11.34792177 | 8.887694003 | 7.683237561 | 8.48457825 | 9.553609024 |
| ENSMUSG00000028480 | Glipr2 | 2396 | 125.1915806 | 137.589483 | 138.4379372 | 137.1965505 | 136.7018951 | 148.2652533 | 112.5354579 | 107.5157572 | 109.6358921 | 104.6525208 | 104.9613445 | 103.6876103 | 148.0163411 | 164.6865469 | 167.1964641 | 174.3533083 | 167.5415834 | 170.2159922 |
| ENSMUSG00000028483 | Snapc3 | 7069 | 8.412044315 | 6.847310129 | 7.055185995 | 6.290504919 | 5.617320275 | 6.357947285 | 7.917795894 | 7.985340325 | 7.883060663 | 6.336563601 | 7.11656971 | 7.887939868 | 7.425259582 | 7.397940642 | 7.606172081 | 8.233374346 | 7.232604338 | 5.960811395 |
| ENSMUSG00000028484 | Psip1 | 5029 | 15.57706093 | 15.69925617 | 16.02511465 | 16.20773679 | 15.16983476 | 15.81470221 | 11.17666784 | 11.79415409 | 12.84161 | 11.23216196 | 11.2573724 | 10.854587 | 12.4580503 | 9.484604842 | 12.19906875 | 12.58587672 | 12.34611274 | 9.767972222 |
| ENSMUSG00000028487 | Bnc2 | 12061 | 0.045814384 | 0.093909162 | 0.040107396 | 0.079328574 | 0.071832736 | 0.049302114 | 0 | 0 | 0 | 0 | 0.007922222 | 0.027766684 | 0 | 0 | 0 | 0 | 0.009533114 | 0 |
| ENSMUSG00000028488 | Sh3gl2 | 4942 | 51.43281114 | 48.78296549 | 57.2318964 | 53.03777581 | 53.70283835 | 55.08755934 | 53.44934975 | 52.65945568 | 51.25396033 | 52.07376333 | 53.14988299 | 51.09471015 | 30.23601324 | 35.49561272 | 37.44989661 | 27.10249791 | 36.24789796 | 39.77084952 |
| ENSMUSG00000028492 | Fam154a | 2189 | 0 | 0 | 0 | 0 | 0 | 0 | 0.345821317 | 0 | 0.395119612 | 0.043079674 | 0 | 0 | 0.02253628 | 0.042434068 | 0 | 0 | 0 | 0 |
| ENSMUSG00000028494 | Plin2 | 3499 | 2.648221885 | 2.618189743 | 2.129043634 | 3.007888323 | 1.77451219 | 2.520830688 | 0.987089329 | 1.440810837 | 1.141781089 | 1.010660973 | 0.928464469 | 1.004969061 | 0.944623737 | 0.955694282 | 1.024445659 | 0.262639205 | 0.788652008 | 0.927561388 |
| ENSMUSG00000028495 | Rps6 | 1601 | 63.95688261 | 55.30647897 | 58.4954121 | 52.9031739 | 54.50542012 | 54.56681405 | 60.78837012 | 53.53138726 | 58.16534175 | 49.35951197 | 52.63899235 | 50.96970562 | 75.18418373 | 78.6155383 | 64.02840166 | 56.61078558 | 56.54385589 | 64.38917302 |
| ENSMUSG00000028496 | Mllt3 | 13461 | 0.868354759 | 0.692935917 | 1.04214574 | 0.680318692 | 0.815249877 | 0.905577034 | 1.047409429 | 0.885226176 | 1.138205472 | 1.124387174 | 0.883735581 | 1.061496889 | 0.710971841 | 0.807363231 | 0.829479359 | 0.503486926 | 0.671941614 | 1.340389793 |
| ENSMUSG00000028497 | Ptplad2 | 6875 | 0.265850556 | 0.290730713 | 0.189976046 | 0.351234233 | 0.315045319 | 0.021623011 | 0.206455326 | 0.26665241 | 0.293547531 | 0.260614796 | 0.403046922 | 0.535830361 | 1.686254627 | 4.438365878 | 3.380025821 | 1.609597897 | 2.12397369 | 2.832468913 |
| ENSMUSG00000028514 | Usp24 | 12307 | 6.489577131 | 6.515327568 | 6.654455748 | 6.112073145 | 6.187105163 | 6.659642589 | 7.577256047 | 7.298978667 | 7.185134423 | 7.735213283 | 7.868679681 | 7.651013471 | 6.437560668 | 6.034297208 | 5.410068605 | 5.435418147 | 5.739446266 | 5.426265992 |
| ENSMUSG00000028517 | Ppap2b | 3153 | 36.29049599 | 29.61498335 | 32.92407109 | 31.71788529 | 30.77512151 | 30.64631477 | 36.11849609 | 29.4346774 | 30.8670272 | 31.68802417 | 31.74390676 | 32.41309172 | 29.47711137 | 26.55841724 | 32.47252343 | 36.8333055 | 28.50465152 | 28.26348014 |
| ENSMUSG00000028518 | Prkaa2 | 7960 | 3.583036823 | 3.649352231 | 3.524704473 | 4.155441983 | 3.924323927 | 4.276724619 | 2.448847201 | 2.744479168 | 2.178332052 | 3.441527461 | 3.541108731 | 3.22552824 | 3.445797237 | 2.999027754 | 2.976243045 | 3.145987091 | 3.644850095 | 3.973647785 |
| ENSMUSG00000028519 | Dab1 | 8006 | 0 | 0.029126937 | 0 | 0 | 0.024047926 | 0 | 0.005909653 | 0.014311417 | 0 | 0.058894208 | 0 | 0 | 0.018485605 | 0.01740348 | 0 | 0.04304465 | 0 | 0 |
| ENSMUSG00000028520 | 4921539E11R | 2744 | 0 | 0 | 0 | 0 | 0 | 0 | 0 | 0 | 0 | 0 | 0.052232097 | 0.020340985 | 0 | 0 | 0 | 0 | 0 | 0 |
| ENSMUSG00000028521 | Slc35d1 | 11382 | 1.430283115 | 2.004861407 | 1.559750979 | 1.128818705 | 1.611163497 | 1.362678405 | 1.720914521 | 1.375758653 | 0.720094591 | 1.379474977 | 1.171078318 | 1.5986563 | 1.187572073 | 1.264950544 | 1.364697467 | 1.271643788 | 1.559047221 | 1.493394305 |
| ENSMUSG00000028522 | Mier1 | 10032 | 4.775053168 | 4.273693666 | 4.624224044 | 4.119205264 | 4.418809978 | 4.588764902 | 3.57014533 | 4.282939863 | 4.34363374 | 3.727123972 | 4.762256627 | 4.556721258 | 4.115910562 | 3.89811818 | 3.856485345 | 3.202334491 | 3.740175904 | 3.279051617 |
| ENSMUSG00000028523 | Tctex1d1 | 2536 | 0 | 0 | 0 | 0 | 0 | 0 | 0 | 0 | 0 | 0 | 0 | 0.044018661 | 0 | 0 | 0 | 0 | 0 | 0 |
| ENSMUSG00000028524 | Sgip1 | 11673 | 0.185707528 | 0.188353553 | 0.169906172 | 0.089771611 | 0.222661173 | 0.106126817 | 0.012159517 | 0 | 0 | 0.004039296 | 0 | 0 | 0.012678468 | 0 | 0 | 0.111529226 | 0.045966603 | 0 |
| ENSMUSG00000028525 | Pde4b | 10597 | 8.278822622 | 9.77664443 | 6.16252392 | 9.131986238 | 9.238495116 | 8.795762184 | 2.826170214 | 5.027687209 | 3.431885762 | 2.967775698 | 3.705861647 | 3.291949525 | 1.908661516 | 1.893351491 | 1.434686621 | 2.290860158 | 2.180877327 | 1.02781949 |
| ENSMUSG00000028527 | Ak4 | 5344 | 0.691981721 | 1.147000849 | 1.312530296 | 0.375128359 | 1.089813391 | 1.502159963 | 2.50551799 | 2.815832064 | 2.651227811 | 2.258716324 | 1.922083846 | 1.702461265 | 1.513928599 | 2.659410693 | 1.218159774 | 0.55171721 | 1.090119223 | 1.091123749 |

| ENSMUSG00000028528 | Dnajc6 | 5966 | 0.919069344 | 1.831483289 | 1.127040013 | 1.573177193 | 1.395712839 | 1.536583257 | 0.293424258 | 0.288075446 | 0.497054833 | 0.181774418 | 0.168165291 | 0.280668769 | 0.049613058 | 0.303607008 | 0.110496965 | 0.263143631 | 0.244116541 | 0.341156042 |
| --- | --- | --- | --- | --- | --- | --- | --- | --- | --- | --- | --- | --- | --- | --- | --- | --- | --- | --- | --- | --- |
| ENSMUSG00000028530 | Jak1 | 7174 | 24.8667722 | 26.28718904 | 27.6863278 | 30.43330422 | 28.31289628 | 30.32980947 | 20.89302577 | 22.47144279 | 19.81247085 | 24.15372658 | 22.81530635 | 22.67170906 | 21.79846359 | 21.16332892 | 20.9970572 | 23.46860897 | 27.46522057 | 21.0252259 |
| ENSMUSG00000028532 | Cachd1 | 5991 | 18.68070914 | 18.0493498 | 16.92387241 | 21.26334167 | 18.43811798 | 20.92612518 | 15.92094153 | 15.22341127 | 17.13870536 | 19.59693726 | 18.81971324 | 17.88784379 | 20.61054731 | 18.0706339 | 21.11313246 | 21.14260212 | 21.68688808 | 18.46491631 |
| ENSMUSG00000028533 | Izumo3 | 1670 | 0 | 0 | 0 | 0 | 0 | 0 | 0 | 0 | 0 | 0 | 0 | 0 | 0 | 0 | 0 | 0 | 0 | 0 |
| ENSMUSG00000028536 | 2610528J11R | 1200 | 25.82189444 | 25.70645004 | 23.25960585 | 25.77995759 | 23.54453266 | 25.31318807 | 34.26226499 | 27.84862675 | 23.54495818 | 29.86211206 | 24.92260334 | 23.62863049 | 39.21887423 | 36.26509157 | 26.16298799 | 28.59032019 | 30.30971313 | 25.85428016 |
| ENSMUSG00000028538 | St3gal3 | 3732 | 4.669646884 | 5.141539944 | 4.640333298 | 5.249530832 | 4.552671237 | 4.155950056 | 5.552774215 | 3.479479319 | 3.15630855 | 5.836984165 | 6.746356677 | 5.189719967 | 5.062734276 | 6.13529879 | 4.261468535 | 3.785968417 | 4.51864165 | 4.171376528 |
| ENSMUSG00000028539 | Artn | 3305 | 1.414695723 | 1.038193072 | 0.746459924 | 2.095394475 | 0.932055405 | 1.574292595 | 1.775120178 | 1.941398972 | 1.669892016 | 3.281289485 | 2.847705543 | 2.12791936 | 3.015142902 | 2.866745485 | 2.368621973 | 4.286694323 | 1.867029895 | 3.112466855 |
| ENSMUSG00000028540 | Dph2 | 3593 | 4.022198678 | 4.255668925 | 3.823568778 | 4.488897772 | 4.742193426 | 4.689098084 | 5.912438864 | 4.241238118 | 2.968913883 | 4.632395839 | 5.651087491 | 3.169049587 | 4.887882703 | 3.929586019 | 6.364281525 | 2.781477465 | 3.669425587 | 4.562402951 |
| ENSMUSG00000028541 | B4galt2 | 4981 | 5.307813589 | 6.948824954 | 7.341977303 | 6.860225507 | 7.218339084 | 7.12301892 | 9.128183989 | 7.981989742 | 8.318336651 | 8.595229584 | 8.507606539 | 7.216479965 | 5.605674599 | 7.72980294 | 9.363613987 | 6.265176678 | 6.40951723 | 7.631257327 |
| ENSMUSG00000028542 | Slc6a9 | 5119 | 15.31979874 | 20.0697335 | 18.03021995 | 19.83004626 | 16.97169803 | 19.40871866 | 10.49955133 | 10.0796905 | 8.842348276 | 9.809630586 | 9.892841568 | 11.81953082 | 7.767439993 | 5.561677197 | 6.889730776 | 7.659617499 | 7.546963719 | 6.383162609 |
| ENSMUSG00000028544 | Slc5a9 | 7485 | 0 | 0.031154343 | 0.012925459 | 0.01826094 | 0.019291352 | 0 | 0.164345979 | 0 | 0 | 0 | 0 | 0.022371007 | 0.12522464 | 0.30404279 | 0.225686545 | 0.184163242 | 0.08192662 | 0.20577889 |
| ENSMUSG00000028545 | Bend5 | 2450 | 21.26993707 | 19.48464384 | 20.47483713 | 19.82370337 | 19.54745471 | 18.76936194 | 25.25917716 | 20.31212259 | 22.50969168 | 23.76780348 | 24.19947897 | 21.16438798 | 30.90795633 | 24.81441241 | 25.35997807 | 27.67863906 | 28.56482373 | 23.82231112 |
| ENSMUSG00000028546 | Elavl4 | 8251 | 0 | 0 | 0 | 0 | 0 | 0 | 0 | 0 | 0 | 0 | 0 | 0 | 0 | 0 | 0 | 0 | 0 | 0 |
| ENSMUSG00000028549 | Itgb3bp | 6295 | 2.079681369 | 1.693427506 | 1.867318169 | 1.896265864 | 2.117957562 | 1.889222567 | 3.577575087 | 2.299431376 | 3.853670816 | 2.591603388 | 2.929489114 | 2.571333145 | 2.617452005 | 3.024952475 | 2.866764334 | 2.731133078 | 3.117246071 | 2.700208734 |
| ENSMUSG00000028550 | Atg4c | 3340 | 3.76692578 | 4.22894226 | 4.837353036 | 3.983181122 | 4.582620277 | 4.80691188 | 5.212893966 | 5.557337595 | 5.043498487 | 5.067994751 | 5.807375192 | 5.765390297 | 4.770721341 | 4.227246277 | 4.773952733 | 4.493987456 | 3.970328961 | 5.006816899 |
| ENSMUSG00000028551 | Cdkn2c | 2683 | 0.221793688 | 0.360072282 | 0.30650392 | 0.067925523 | 0.053818774 | 0.387852182 | 0.176342448 | 0.612103356 | 0.905704223 | 0.316329727 | 0.249291621 | 0.35365869 | 0.625152884 | 1.69642958 | 0.460695742 | 0.528047884 | 0.271412464 | 1.189164439 |
| ENSMUSG00000028552 | Eps15 | 7048 | 12.26667537 | 14.36405867 | 13.69942772 | 14.07298432 | 14.97634912 | 16.18478898 | 11.66034667 | 12.96742607 | 10.78165743 | 11.28593025 | 13.44179096 | 12.10078496 | 10.28914847 | 10.27331615 | 12.98363445 | 10.34411414 | 12.35490161 | 10.06835239 |
| ENSMUSG00000028553 | Angptl3 | 3571 | 0.607046759 | 0.289190625 | 0.447025063 | 0.293448339 | 0.593056466 | 0.679945098 | 0.60946044 | 0.68448999 | 0.784285374 | 0.277279409 | 0.802715628 | 0.859664364 | 0.345364865 | 0.403183061 | 0.161529483 | 0.707695348 | 0.482969297 | 0.169448383 |
| ENSMUSG00000028555 | Ttc39a | 4883 | 2.167476721 | 2.926731849 | 2.387470984 | 2.174016656 | 2.72054289 | 1.755605757 | 1.928163651 | 2.017947946 | 1.948409818 | 1.863625994 | 1.682836956 | 1.634577053 | 3.081350557 | 3.79504216 | 3.189471253 | 1.340916213 | 2.244792323 | 1.701080302 |
| ENSMUSG00000028556 | Dock7 | 8687 | 9.267273328 | 13.81677885 | 12.65719287 | 13.35310451 | 12.93195693 | 14.53440372 | 9.623748551 | 11.41331606 | 12.46926861 | 12.06584705 | 11.49414795 | 11.23123958 | 12.45933308 | 13.53171235 | 12.77736751 | 12.14350448 | 12.74601954 | 11.49954389 |
| ENSMUSG00000028557 | Rnf11 | 5781 | 57.31324116 | 56.610599 | 63.66964063 | 63.63256737 | 60.11286782 | 63.43881356 | 57.21552299 | 59.45224516 | 61.82608265 | 60.14345024 | 54.74988755 | 56.26944846 | 60.53634685 | 62.95379152 | 62.73954273 | 60.04889235 | 65.64729146 | 61.24175936 |
| ENSMUSG00000028558 | Calr4 | 2503 | 0 | 0.013309187 | 0 | 0 | 0 | 0 | 0 | 0 | 0 | 0 | 0 | 0 | 0 | 0 | 0 | 0 | 0 | 0 |
| ENSMUSG00000028559 | Osbpl9 | 6363 | 25.53784189 | 25.18230728 | 26.93500205 | 26.14228711 | 25.88518667 | 25.3487581 | 21.00555052 | 20.4197003 | 22.37004534 | 22.46753611 | 21.16574068 | 19.41223657 | 21.04921506 | 24.70010871 | 22.63074616 | 24.25733749 | 26.51459358 | 24.70784646 |
| ENSMUSG00000028560 | Usp1 | 4088 | 10.35595776 | 10.03950237 | 11.08757288 | 7.032538888 | 12.50393907 | 8.315355977 | 10.10371054 | 9.669553936 | 8.704782196 | 9.700034599 | 9.793399023 | 9.024988496 | 6.878471832 | 10.69077452 | 7.357420695 | 6.903174573 | 8.456536156 | 7.172175607 |
| ENSMUSG00000028563 | Tm2d1 | 1191 | 97.57275505 | 86.42891951 | 97.31568323 | 86.22551967 | 98.60780332 | 98.19013539 | 105.2717037 | 101.8784739 | 115.8824637 | 101.6649924 | 104.0138265 | 99.63400391 | 102.8058931 | 93.31712941 | 98.45476748 | 96.96429213 | 103.8124549 | 106.7389132 |
| ENSMUSG00000028565 | Nfia | 10900 | 8.259262645 | 8.077612791 | 8.68948371 | 7.55310157 | 8.102943113 | 8.669455315 | 7.083880001 | 7.126912522 | 7.364451305 | 7.544112521 | 7.324032625 | 7.50182988 | 6.68017522 | 5.355983289 | 6.255832569 | 6.48481296 | 6.156820723 | 5.60689152 |
| ENSMUSG00000028567 | Txndc12 | 2467 | 23.12198874 | 23.23935588 | 21.82397215 | 20.55510432 | 22.24174827 | 19.92554184 | 20.38645225 | 17.66417963 | 23.22271724 | 19.81973218 | 23.6066779 | 20.0230488 | 19.71676953 | 17.90365914 | 21.42748124 | 20.23954279 | 20.21180068 | 18.44042613 |
| ENSMUSG00000028568 | Btf3l4 | 4706 | 8.725031883 | 8.586600231 | 8.274690169 | 8.016265344 | 8.253816915 | 8.834412128 | 7.55032339 | 6.825289083 | 6.54643302 | 7.013491772 | 7.756070607 | 7.258645453 | 7.044421677 | 5.408278769 | 8.702581079 | 7.200847333 | 7.272712098 | 7.106998914 |
| ENSMUSG00000028571 | Cyp2j13 | 4680 | 0.090823026 | 0.597923742 | 0.413448977 | 0.116823191 | 0.082276793 | 0.211763819 | 1.688294312 | 1.232276231 | 0.924056443 | 0.987343786 | 0.653332766 | 1.311906599 | 2.972564246 | 7.224635816 | 3.512696752 | 1.120900259 | 0.737044171 | 2.374324692 |
| ENSMUSG00000028572 | Hook1 | 5559 | 12.24919806 | 13.6151998 | 15.54148682 | 12.43319019 | 16.13921671 | 13.62945307 | 15.54109583 | 14.37969027 | 17.86304902 | 14.8178231 | 13.75066263 | 14.86007926 | 16.82556488 | 16.39203084 | 15.0827786 | 15.02970055 | 22.19326313 | 15.51615027 |
| ENSMUSG00000028573 | Fggy | 5898 | 5.296931906 | 6.794745972 | 6.50393516 | 4.866642579 | 6.406164815 | 5.335026419 | 6.473606629 | 8.664197087 | 8.973325323 | 7.282857014 | 7.241575599 | 5.999852506 | 6.3902314 | 8.40214331 | 7.782050401 | 5.115801735 | 7.23248024 | 6.332855793 |
| ENSMUSG00000028575 | 4930579C15R | 1472 | 0.173254794 | 0.045262085 | 0 | 0.185710779 | 0.098094953 | 0 | 0.224992359 | 0.129729628 | 0 | 0.032031728 | 0 | 0.227509494 | 0 | 0 | 0.027990187 | 0 | 0.130184432 | 0.224221902 |
| ENSMUSG00000028576 | Ift74 | 2635 | 11.96919947 | 11.77013454 | 13.73184087 | 11.94788677 | 12.00101474 | 12.50571834 | 15.24419902 | 11.69687618 | 11.09769495 | 11.79214932 | 12.4015452 | 11.67151047 | 13.62946329 | 14.0125425 | 15.83953553 | 16.85658618 | 14.90871132 | 12.25440062 |
| ENSMUSG00000028577 | Plaa | 7169 | 10.1267737 | 13.02962134 | 12.69223191 | 11.99877971 | 13.40765122 | 11.63995025 | 9.76743825 | 10.45243319 | 11.5246409 | 10.3851249 | 10.28937591 | 10.58854807 | 8.773635727 | 10.67002538 | 8.48858949 | 10.15350044 | 10.22177147 | 11.60186489 |
| ENSMUSG00000028578 | 5830433M19R | 2827 | 10.19402524 | 10.53474617 | 10.95120602 | 11.18477707 | 11.25405078 | 11.37591938 | 12.18380978 | 12.5506692 | 12.87898123 | 11.77516678 | 12.64084504 | 12.12268017 | 11.37757696 | 10.44868751 | 9.560743072 | 12.5423072 | 11.34740379 | 11.46104134 |
| ENSMUSG00000028580 | Pum1 | 6096 | 27.03290149 | 29.12145887 | 26.9799874 | 31.45774862 | 28.07695547 | 29.21465297 | 18.88316073 | 25.19846497 | 21.20132652 | 24.86704576 | 20.72912815 | 25.13352917 | 16.26593729 | 15.53469393 | 22.44592636 | 20.31990075 | 20.97383964 | 15.95408476 |
| ENSMUSG00000028581 | Laptm5 | 3898 | 0.250800166 | 0.051276902 | 0.086868833 | 0 | 0.098782809 | 0.114411134 | 0.424818821 | 0.862219449 | 1.479248899 | 0.314499303 | 0.870195498 | 0.114552412 | 0.37967099 | 1.54893057 | 0.877303525 | 0.284871117 | 0.098322978 | 0 |
| ENSMUSG00000028582 | Cc2d1b | 4011 | 17.59127205 | 19.84156178 | 17.90942223 | 19.24218626 | 19.82396856 | 18.61779344 | 12.65681987 | 16.02538351 | 13.21541904 | 16.55153084 | 16.53244636 | 16.3369703 | 17.24341761 | 19.395125 | 15.92181791 | 17.44127143 | 13.73096202 | 16.40261572 |
| ENSMUSG00000028583 | Pdpn | 1891 | 9.036002519 | 12.66629875 | 13.07185299 | 13.99839596 | 12.98111109 | 10.77005473 | 12.08462397 | 13.95608147 | 17.16286326 | 14.68628462 | 14.45123017 | 13.37096518 | 11.19164067 | 10.63473815 | 12.78969503 | 16.30033789 | 16.17365676 | 12.18869155 |
| ENSMUSG00000028584 | Lrrc38 | 2322 | 0 | 0 | 0 | 0 | 0 | 0 | 0 | 0 | 0 | 0 | 0 | 0 | 0 | 0 | 0 | 0 | 0 | 0 |
| ENSMUSG00000028587 | Orc1 | 4797 | 0.584811678 | 0.381948964 | 0.332776006 | 0.693340887 | 0.200675104 | 0.413197695 | 0.286026202 | 0.485665321 | 0.128788354 | 0.383339051 | 0.139430773 | 0.383972663 | 0.771293266 | 0.464731331 | 0.506752511 | 0.590682707 | 0.391492297 | 0.275217545 |

| ENSMUSG00000028589 | 1700012P22R | 970 | 0 | 0.10302957 | 0 | 0.328791042 | 0 | 0 | 0 | 0.039373611 | 0 | 0 | 0 | 0 | 0 | 0 | 0 | 0 | 0 | 0 |
| --- | --- | --- | --- | --- | --- | --- | --- | --- | --- | --- | --- | --- | --- | --- | --- | --- | --- | --- | --- | --- |
| ENSMUSG00000028590 | D530049N12R | 765 | 0.055562322 | 0.304823869 | 0.379400238 | 0.655126128 | 0.69209302 | 0.388648891 | 0.061846639 | 0.049924709 | 0.107677165 | 0.184904719 | 0.062450926 | 0.510731553 | 1.031778662 | 0.789245929 | 0.215432972 | 1.551645529 | 0.601196814 | 0 |
| ENSMUSG00000028591 | Pramef12 | 2568 | 0.215174178 | 0.064861554 | 0.169533401 | 0.337094954 | 0.787204359 | 0.347332245 | 0.276359106 | 0.312321048 | 0 | 0.624269436 | 0.074415823 | 0.021735071 | 1.709712086 | 3.074569648 | 1.588377739 | 1.520888612 | 1.850646735 | 1.092470577 |
| ENSMUSG00000028593 | 9430007A20R | 1402 | 0 | 0 | 0 | 0 | 0 | 0 | 0 | 0 | 0 | 0 | 0 | 0 | 0 | 0 | 0 | 0 | 0 | 0 |
| ENSMUSG00000028597 | Gpx7 | 1867 | 2.071757375 | 1.944887772 | 1.554585871 | 1.415393986 | 1.521040973 | 1.512857961 | 0.55751416 | 1.002371568 | 0.529446373 | 1.18697539 | 0.665318115 | 0.627814094 | 0.4756157 | 0.348268465 | 0.617912997 | 0.902403174 | 1.170111903 | 1.090164407 |
| ENSMUSG00000028599 | Tnfrsf1b | 3808 | 6.250761181 | 4.295333787 | 5.436940912 | 8.040184295 | 6.168166689 | 5.088003894 | 4.646780966 | 4.453105743 | 4.272235747 | 5.101389113 | 4.805096927 | 4.661076865 | 7.332422578 | 8.269194131 | 4.1114998 | 10.14580432 | 7.387474245 | 3.539188852 |
| ENSMUSG00000028600 | Podn | 5100 | 0.066674786 | 0 | 0 | 0 | 0 | 0.009716222 | 0 | 0.044932238 | 0 | 0 | 0 | 0 | 0 | 0 | 0 | 0 | 0.00751496 | 0 |
| ENSMUSG00000028601 | Echdc2 | 2334 | 243.1572881 | 176.8837609 | 212.4994887 | 207.3866902 | 181.0824429 | 224.8980749 | 133.7485242 | 120.5007932 | 145.1762918 | 128.7856612 | 133.4178146 | 157.6182657 | 107.4564985 | 83.65507429 | 108.6704276 | 93.06881568 | 95.65153362 | 103.6075549 |
| ENSMUSG00000028602 | Tnfrsf8 | 5541 | 0 | 0.006012073 | 0 | 0.049335186 | 0 | 0 | 0.051231921 | 0 | 0 | 0 | 0.043110412 | 0 | 0.071224569 | 0 | 0 | 0 | 0 | 0 |
| ENSMUSG00000028603 | Scp2 | 4037 | 79.25104284 | 70.82600225 | 76.29285801 | 77.80476583 | 76.7940848 | 78.11582772 | 96.03172929 | 85.18315364 | 79.52660153 | 89.55947302 | 78.11803342 | 79.30607892 | 81.54369185 | 82.00481915 | 87.85310704 | 71.60163371 | 80.76339058 | 82.34333216 |
| ENSMUSG00000028607 | Cpt2 | 2966 | 17.95650221 | 17.95931148 | 18.0218311 | 18.95560644 | 20.20372386 | 21.83594834 | 20.3383903 | 18.4008574 | 21.14870651 | 19.15596677 | 19.57065899 | 16.67318849 | 20.54110514 | 17.99199474 | 15.57213222 | 18.26743408 | 17.47038214 | 16.93301229 |
| ENSMUSG00000028608 | 0610037L13R | 4789 | 23.30728592 | 19.51193742 | 23.53525281 | 22.12883465 | 20.51310424 | 19.72176033 | 17.06180758 | 19.21183909 | 23.8054449 | 18.20456261 | 23.34378844 | 21.37521929 | 15.6885592 | 15.52662015 | 17.07769648 | 15.63126696 | 17.87066632 | 19.23997782 |
| ENSMUSG00000028609 | Magoh | 765 | 76.23150524 | 69.71757339 | 72.90807908 | 65.27438509 | 75.186469 | 70.86364776 | 76.75167915 | 75.93548243 | 72.25137798 | 72.05120537 | 71.94346689 | 69.67837617 | 80.7366803 | 79.83526133 | 74.37823358 | 85.94114106 | 76.00129726 | 85.06637027 |
| ENSMUSG00000028610 | Dmrtb1 | 2856 | 0 | 0 | 0 | 0 | 0 | 0 | 0 | 0.05349076 | 0.014421049 | 0 | 0.10036756 | 0 | 0 | 0 | 0 | 0 | 0 | 0 |
| ENSMUSG00000028612 | 4930430J20R | 792 | 0.053668152 | 0 | 0 | 0 | 0 | 0.062566583 | 0 | 0 | 0 | 0 | 0 | 0 | 0.311438872 | 0 | 0 | 0 | 0 | 0 |
| ENSMUSG00000028613 | Lrp8 | 8058 | 1.571921377 | 1.69086296 | 1.470776239 | 1.204331403 | 1.618733099 | 1.426685802 | 0.992286267 | 1.729985962 | 1.017140312 | 1.100066048 | 1.0553416 | 0.526432161 | 2.093759706 | 2.1441053 | 1.932760999 | 2.086073052 | 1.864471133 | 1.631568194 |
| ENSMUSG00000028614 | Tmem48 | 5173 | 3.927599873 | 3.413074413 | 4.282829481 | 4.007399028 | 3.042555391 | 4.176491753 | 3.475510921 | 3.300213392 | 2.627401933 | 3.563874922 | 2.650572798 | 3.981438139 | 2.813244848 | 2.100892412 | 4.149625096 | 4.722483573 | 3.600730774 | 3.16889886 |
| ENSMUSG00000028617 | Lrrc42 | 2596 | 9.987733966 | 7.18614051 | 8.19890345 | 7.458953714 | 7.379239453 | 9.105028473 | 7.527017102 | 6.944073165 | 6.758650121 | 7.410434102 | 6.901236298 | 6.579195975 | 7.867262616 | 7.066800662 | 6.856345202 | 8.436913495 | 8.060923771 | 5.636526344 |
| ENSMUSG00000028618 | Tmem59 | 2437 | 433.1097789 | 407.6147693 | 419.8989172 | 427.492343 | 424.1415924 | 434.0185056 | 381.0058777 | 382.1747743 | 413.1833965 | 397.4821698 | 398.6670314 | 413.315325 | 452.0844106 | 408.8680553 | 438.6773782 | 514.118574 | 465.042511 | 472.6900714 |
| ENSMUSG00000028619 | 2210012G02R | 7628 | 2.942151671 | 1.690100952 | 2.168818482 | 2.138287083 | 2.30942371 | 2.026802947 | 1.860750351 | 2.203022687 | 2.3001384 | 2.960826804 | 2.467663036 | 2.853711118 | 2.845574672 | 2.745973179 | 2.760093742 | 3.292952079 | 2.748359256 | 2.848531352 |
| ENSMUSG00000028621 | Cyb5rl | 4262 | 5.235855799 | 8.253969098 | 6.9575256 | 7.312002423 | 7.182520754 | 10.05704236 | 8.014958748 | 7.921652681 | 6.464988044 | 7.445430148 | 5.459034439 | 8.47318951 | 4.734104685 | 5.078119363 | 5.403958175 | 5.264733913 | 4.262474128 | 6.569600029 |
| ENSMUSG00000028622 | Mrpl37 | 4672 | 22.28974342 | 25.09162559 | 21.27731269 | 20.9666621 | 21.4801072 | 23.74755361 | 17.84352317 | 18.76921146 | 19.6059099 | 17.17690431 | 18.62118994 | 18.79238811 | 19.22804396 | 19.29537107 | 18.31670197 | 17.5717171 | 18.40843541 | 16.77824852 |
| ENSMUSG00000028626 | Col9a2 | 3102 | 0.164430081 | 0.139609163 | 0.062377215 | 0.073438176 | 0.031032833 | 0.14377002 | 0.3202986 | 0 | 0 | 0.015200098 | 0.138612065 | 0 | 1.2404544 | 0.449169124 | 0.25236285 | 0.432034577 | 0 | 0.443335805 |
| ENSMUSG00000028629 | Dem1 | 1956 | 9.409376902 | 11.93882354 | 9.52137484 | 11.04086657 | 11.98376627 | 11.12149798 | 9.772148408 | 11.61783202 | 7.93830084 | 9.425319516 | 9.574531556 | 9.730644653 | 10.13876827 | 7.099581866 | 9.8159126 | 7.928269937 | 9.542385783 | 5.7371461 |
| ENSMUSG00000028630 | Dyrk2 | 2134 | 7.05099921 | 9.007282083 | 10.22327188 | 8.902978204 | 9.788779228 | 9.682984959 | 7.11685564 | 8.608503073 | 8.086761998 | 10.14159925 | 10.43258231 | 9.285173486 | 5.941097816 | 7.356186287 | 7.317427218 | 8.325605358 | 8.584803149 | 9.563438891 |
| ENSMUSG00000028631 | Kcnq4 | 4009 | 0.106024385 | 0.149571488 | 0.37405324 | 0.454587621 | 0.132065642 | 0.259567824 | 0.413056563 | 0.304853299 | 0.339025947 | 0.105850918 | 0.190670825 | 0.111380719 | 0.209189971 | 0 | 0 | 0 | 0.038240256 | 0 |
| ENSMUSG00000028633 | Ctps | 2775 | 8.608255469 | 8.811410579 | 8.036107205 | 7.634553375 | 9.262143149 | 7.964151054 | 7.484780558 | 8.216527653 | 8.474774955 | 6.405699145 | 6.146183848 | 5.068665575 | 8.301983916 | 8.033571873 | 7.304924504 | 4.898438064 | 5.869793219 | 5.609937731 |
| ENSMUSG00000028634 | Hivep3 | 11885 | 0.175242207 | 0.089693952 | 0.158735186 | 0.130338705 | 0.113394519 | 0.062540261 | 0.195062791 | 0.16067481 | 0.166340156 | 0.234067437 | 0.100494233 | 0.150281969 | 0.215840109 | 0.230559626 | 0.166400899 | 0.086987497 | 0.041921907 | 0.286963788 |
| ENSMUSG00000028635 | Edn2 | 1457 | 0.291730789 | 0.022864032 | 0.166003879 | 0.093811347 | 0 | 0 | 0 | 0.131065211 | 0 | 0 | 0 | 0 | 0 | 0.223135629 | 0 | 0 | 0.131524698 | 0.49081564 |
| ENSMUSG00000028636 | Ppcs | 1559 | 21.89330106 | 26.8383548 | 20.29268047 | 19.14206802 | 19.69734913 | 21.48662469 | 18.29990083 | 17.7855575 | 23.93520416 | 19.96117008 | 20.9915629 | 19.83443045 | 22.46674873 | 24.27962234 | 21.67111984 | 21.90841681 | 20.72422597 | 19.44196139 |
| ENSMUSG00000028637 | Ccdc30 | 6765 | 0.364419839 | 0.329928147 | 0.407581852 | 0.121226726 | 0.519383655 | 0.322294202 | 0.335699717 | 0.51939409 | 0.45052508 | 0.717889495 | 0.685021578 | 0.338276743 | 0.481286998 | 0.405055603 | 0.596859198 | 0.469788679 | 0.402241993 | 0.520411357 |
| ENSMUSG00000028639 | Ybx1 | 2900 | 108.8718785 | 112.0232222 | 109.591067 | 103.0622244 | 106.9026622 | 109.2210597 | 102.7337721 | 103.4223918 | 109.5135252 | 108.3002877 | 104.5942108 | 98.65899532 | 109.5508784 | 101.0238977 | 99.19629767 | 87.81752753 | 90.06679772 | 89.19062752 |
| ENSMUSG00000028640 | Tfap2c | 3670 | 0.034745375 | 0.308620818 | 0.118627186 | 0.086901174 | 0.23606938 | 0.39156111 | 0.399643882 | 0.176913036 | 0 | 0 | 0.169230098 | 0.288963921 | 0 | 0.303721552 | 0.05613291 | 0 | 0.114874459 | 0 |
| ENSMUSG00000028641 | Lepre1 | 4327 | 2.377225005 | 1.247212587 | 1.229740776 | 1.25300769 | 1.4015767 | 1.236814242 | 0.92941477 | 0.697296 | 1.522958752 | 0.915336047 | 0.728714412 | 1.160945143 | 1.231302765 | 0.568878353 | 0.580840053 | 1.362781035 | 0.752885426 | 1.029752171 |
| ENSMUSG00000028642 | 4930538K18R | 1445 | 0.147076734 | 0.046107812 | 0 | 0.063060269 | 0.166546449 | 0 | 0 | 0.211445826 | 0.399038907 | 0.2936722 | 0.39674706 | 0 | 0.136558941 | 0.224988659 | 0 | 0 | 0 | 0 |
| ENSMUSG00000028643 | Ccdc23 | 1139 | 35.04158059 | 28.45781043 | 24.54776167 | 26.2806024 | 31.22870201 | 26.36431655 | 33.1479524 | 29.64186455 | 31.53173112 | 33.65541857 | 34.64628244 | 29.64747662 | 37.16135647 | 31.39767102 | 28.43232917 | 31.66791235 | 30.68795678 | 33.32421742 |
| ENSMUSG00000028644 | Ermap | 7424 | 0.022901496 | 0.040384705 | 0.019547493 | 0 | 0.019449861 | 0.013349336 | 0 | 0.025722254 | 0.022191011 | 0 | 0 | 0 | 0 | 0 | 0 | 0 | 0.010324972 | 0 |
| ENSMUSG00000028645 | Slc2a1 | 3009 | 41.05019659 | 39.30235111 | 38.26154943 | 43.47150491 | 46.99620862 | 44.62874975 | 61.29106762 | 64.31402559 | 60.66421996 | 63.8548075 | 73.75030978 | 66.14777427 | 54.57824946 | 46.16625636 | 56.22252858 | 58.39667576 | 58.38741941 | 50.21934735 |
| ENSMUSG00000028646 | Rragc | 3625 | 36.03266371 | 41.44583529 | 41.15419796 | 42.00414074 | 39.39514966 | 44.50860704 | 46.25548527 | 38.09757989 | 37.83478551 | 44.78341278 | 41.75201889 | 36.72285663 | 34.2533616 | 34.70814696 | 47.00955453 | 37.95259376 | 44.59595044 | 44.88743109 |
| ENSMUSG00000028647 | Mycbp | 3102 | 17.47069614 | 15.55031297 | 19.10302214 | 16.71452884 | 19.0231265 | 20.11182836 | 17.29612441 | 14.50375565 | 18.76097575 | 19.09132279 | 17.38811351 | 16.44600761 | 17.87526596 | 16.05031002 | 16.41686753 | 16.83700467 | 18.9778182 | 16.8999609 |
| ENSMUSG00000028648 | Ndufs5 | 914 | 43.06323086 | 32.62039423 | 35.56565908 | 26.46927193 | 35.49334415 | 33.88452788 | 39.80683818 | 30.42020673 | 33.43588041 | 37.24596034 | 38.41859353 | 30.96120452 | 40.10242291 | 38.41545959 | 33.35793365 | 31.04304167 | 33.04280302 | 33.64342523 |

| ENSMUSG00000028649 | Macf1 | 28023 | 3.535651619 | 3.269116765 | 2.672170181 | 3.214294853 | 2.913026533 | 3.096272223 | 2.669283995 | 2.817103657 | 2.761640906 | 2.619763943 | 2.802770287 | 2.927917211 | 3.344775464 | 3.530168295 | 2.672962517 | 4.153853852 | 3.558684814 | 2.343820199 |
| --- | --- | --- | --- | --- | --- | --- | --- | --- | --- | --- | --- | --- | --- | --- | --- | --- | --- | --- | --- | --- |
| ENSMUSG00000028651 | Ppie | 1835 | 19.41108311 | 19.82434904 | 20.85202317 | 19.04377167 | 20.77410547 | 17.47172677 | 19.36339067 | 19.48124722 | 21.95117843 | 18.62902445 | 18.433063 | 19.13245328 | 14.24845567 | 20.4252744 | 18.18706282 | 19.55220661 | 18.46345856 | 16.21794373 |
| ENSMUSG00000028653 | Trit1 | 2076 | 16.2158475 | 13.76804588 | 14.84293778 | 14.55056474 | 13.12267283 | 14.13064464 | 15.04160313 | 11.25903192 | 14.54225243 | 11.67411439 | 9.711479999 | 11.48038917 | 14.11520176 | 12.48352638 | 11.11409985 | 13.29842386 | 11.72312068 | 11.95043696 |
| ENSMUSG00000028654 | Mycl1 | 4033 | 1.665216418 | 2.544103997 | 1.631242283 | 2.485353274 | 1.754374604 | 3.182285643 | 1.184869966 | 0.615548266 | 0.357433189 | 0.268898134 | 0.746298632 | 0.650467677 | 0.746155952 | 0.598832766 | 1.144203883 | 0.199380797 | 0.294598364 | 0.491030955 |
| ENSMUSG00000028655 | Mfsd2a | 2235 | 3.689487315 | 6.513527863 | 2.68381108 | 4.301288749 | 4.436320481 | 5.121557699 | 1.651180741 | 1.674655676 | 2.48777612 | 1.603334876 | 2.500970981 | 2.447398181 | 2.825273115 | 3.553440243 | 3.613201322 | 0.702422776 | 2.932347548 | 3.79033666 |
| ENSMUSG00000028656 | Cap1 | 3470 | 82.58498466 | 76.13964382 | 90.93387277 | 85.93574501 | 69.78435388 | 84.11112414 | 83.33576185 | 60.35940484 | 81.06740711 | 83.41733928 | 65.32915792 | 72.06172 | 82.79800756 | 62.06375597 | 83.11553062 | 73.49148279 | 67.08758714 | 74.23851492 |
| ENSMUSG00000028657 | Ppt1 | 3797 | 75.29360389 | 66.23975543 | 72.5666655 | 77.76695505 | 73.06621228 | 71.12520358 | 86.83751895 | 79.43255249 | 87.28677702 | 86.95001954 | 76.61356918 | 79.32086261 | 70.97715672 | 70.76087584 | 80.73204525 | 73.24326615 | 81.46735378 | 77.74002283 |
| ENSMUSG00000028661 | Epha8 | 4696 | 0 | 0 | 0 | 0 | 0 | 0 | 0 | 0 | 0 | 0 | 0 | 0 | 0 | 0 | 0 | 0 | 0 | 0 |
| ENSMUSG00000028664 | Ephb2 | 14278 | 0.101216976 | 0.160988213 | 0.111803229 | 0.29676265 | 0.091018486 | 0.173528273 | 0.019882061 | 0.06152299 | 0.06057689 | 0.184930619 | 0 | 0.179823538 | 0.0380061 | 0.136619391 | 0.011542669 | 0.026817907 | 0.026842903 | 0.061643487 |
| ENSMUSG00000028668 | Tceb3 | 4697 | 6.090266865 | 8.333542837 | 7.641720146 | 7.081022432 | 7.736768654 | 7.690854328 | 7.071215799 | 8.058052115 | 7.909354315 | 6.24392962 | 7.954016932 | 8.959976494 | 6.774342483 | 6.16024408 | 7.613998406 | 7.980946273 | 9.767208302 | 6.218828118 |
| ENSMUSG00000028669 | 1110049F12R | 2868 | 20.51156333 | 20.89605882 | 17.08590873 | 21.28723829 | 18.10820978 | 19.99041588 | 21.80870347 | 19.90852217 | 20.26296156 | 19.44884308 | 20.58920805 | 17.98245196 | 25.78401116 | 29.1975905 | 26.84996791 | 26.08781271 | 23.96061728 | 25.58652314 |
| ENSMUSG00000028670 | Lypla2 | 2334 | 46.23849269 | 43.44663668 | 40.74651272 | 41.53980392 | 44.79114747 | 43.33210336 | 40.72458096 | 46.12869853 | 42.84527004 | 46.50424974 | 52.33957533 | 47.37396212 | 51.91053506 | 50.20498393 | 47.34471847 | 54.22041879 | 49.16406724 | 45.86449087 |
| ENSMUSG00000028671 | Gale | 2281 | 7.472413673 | 7.097793332 | 7.189227001 | 7.84984236 | 7.25882584 | 8.733099035 | 10.35029671 | 10.83318034 | 12.56721393 | 10.06680951 | 10.43048195 | 11.01141962 | 12.76011889 | 15.1895174 | 10.04299214 | 10.47494039 | 7.225036242 | 7.283099619 |
| ENSMUSG00000028672 | Hmgcl | 2497 | 25.39756613 | 27.78965104 | 23.770253 | 20.36288565 | 21.0878352 | 23.47652536 | 26.4890369 | 27.10329878 | 26.11063454 | 26.6815954 | 31.18669696 | 24.72251615 | 22.04822574 | 24.36594092 | 22.32507213 | 19.53633714 | 22.16386573 | 22.33849989 |
| ENSMUSG00000028673 | Fuca1 | 3268 | 91.86476692 | 87.68589843 | 91.75872542 | 89.65822403 | 93.37711018 | 89.7498868 | 113.1276846 | 106.0926037 | 103.2939667 | 110.4896223 | 94.99439418 | 100.6833938 | 118.2728188 | 103.4759487 | 102.0835367 | 97.15597202 | 97.95019331 | 108.1497891 |
| ENSMUSG00000028675 | Pnrc2 | 2339 | 71.65365929 | 64.13337438 | 62.43680553 | 66.16967405 | 64.47084944 | 60.84457069 | 83.29782464 | 65.88556807 | 87.99017497 | 70.49423228 | 73.38838214 | 69.29828309 | 78.5429927 | 70.29160725 | 77.80558887 | 76.85951336 | 76.9966948 | 74.50570761 |
| ENSMUSG00000028676 | Srsf10 | 7385 | 17.92872354 | 17.08723674 | 18.64853635 | 17.48408932 | 17.25840675 | 15.52674685 | 19.45041208 | 17.65590546 | 16.27940955 | 16.60649684 | 16.72932195 | 18.17693548 | 15.97859799 | 16.2381359 | 18.065083 | 15.73622119 | 17.05351545 | 17.81743849 |
| ENSMUSG00000028677 | Rnf220 | 9127 | 4.698994479 | 5.544240859 | 4.669341541 | 5.985284558 | 4.588010693 | 5.581265489 | 5.017932859 | 5.53616176 | 5.550500097 | 5.078244911 | 5.752676604 | 5.185896995 | 5.188850727 | 5.897742663 | 6.915830352 | 4.115600511 | 4.707327581 | 4.592630582 |
| ENSMUSG00000028678 | Kif2c | 4615 | 0.27630667 | 0.252643835 | 0.104818051 | 0.049361912 | 0 | 0 | 0.123023217 | 0 | 0.07139591 | 0 | 0 | 0.060472007 | 0 | 0.110700967 | 0.669581082 | 0.331879587 | 0.307274753 | 0.107276698 |
| ENSMUSG00000028680 | Plk3 | 3038 | 2.098675577 | 4.353264979 | 3.48709781 | 3.644283665 | 3.358778083 | 3.963566247 | 2.67866385 | 6.549783296 | 2.874108407 | 3.911118243 | 5.64555961 | 5.107555694 | 3.458755227 | 6.772459089 | 3.973685278 | 4.751665237 | 9.095872306 | 8.655152407 |
| ENSMUSG00000028681 | Ptch2 | 6194 | 1.146006522 | 0.99497666 | 1.312036342 | 1.691805004 | 1.515293044 | 1.248018476 | 0.763846931 | 1.319530855 | 0.379017016 | 0.647047106 | 0.725031659 | 0.540674807 | 0.892020461 | 0.719830867 | 0.179599937 | 0.17927472 | 0.123752977 | 0.683839396 |
| ENSMUSG00000028683 | Eif2b3 | 6606 | 6.563015372 | 5.784118933 | 5.880100647 | 6.910712447 | 6.907215204 | 6.796060645 | 5.529123241 | 6.359618929 | 7.014052412 | 6.331013289 | 7.072950256 | 5.272324168 | 6.033937202 | 6.988407942 | 8.25153776 | 7.442497715 | 9.201560231 | 7.319558706 |
| ENSMUSG00000028684 | Urod | 2688 | 42.29960784 | 44.54112419 | 39.66341551 | 38.79807682 | 41.14849724 | 41.18333587 | 39.83206563 | 44.03208893 | 35.10353706 | 40.43242969 | 41.25211259 | 42.25627729 | 43.42236469 | 40.88047275 | 44.22116397 | 47.16525339 | 42.67507687 | 43.03725872 |
| ENSMUSG00000028687 | Mutyh | 2071 | 4.351085137 | 4.825624464 | 5.278811135 | 5.169891559 | 6.042636482 | 4.378633629 | 4.683292699 | 4.960770761 | 3.778579428 | 3.733807501 | 4.198475347 | 6.791669228 | 5.978904508 | 4.238499525 | 3.89932639 | 5.750062241 | 4.645050953 | 7.277882782 |
| ENSMUSG00000028688 | Toe1 | 2151 | 16.32230377 | 17.94962548 | 16.82166467 | 17.13569729 | 17.2746839 | 16.0568365 | 20.9618703 | 17.72013835 | 18.4200038 | 20.60514229 | 21.29994662 | 20.44757886 | 15.61833364 | 24.57153484 | 19.3269967 | 20.80972573 | 17.97825829 | 17.31341946 |
| ENSMUSG00000028689 | Ccdc163 | 2857 | 4.790572867 | 3.323127355 | 4.317553462 | 4.018685045 | 3.992742705 | 3.989194513 | 3.229251799 | 2.954330043 | 3.906736357 | 3.234699978 | 2.224035519 | 2.715567763 | 4.00595198 | 2.812330108 | 2.379508828 | 2.921719431 | 2.414677439 | 2.002431606 |
| ENSMUSG00000028690 | Mmachc | 2102 | 19.14957264 | 19.01782734 | 21.70135068 | 17.14499813 | 17.74607081 | 17.09121401 | 25.659588 | 18.96901432 | 16.94878027 | 21.15276554 | 17.27353399 | 20.95078867 | 16.94464523 | 15.64339385 | 18.93468268 | 12.93355436 | 19.491347 | 16.25150108 |
| ENSMUSG00000028691 | Prdx1 | 3093 | 530.3321203 | 518.8432115 | 516.0625222 | 482.5964871 | 527.4281568 | 480.9002276 | 501.3185989 | 473.7808884 | 491.8413017 | 490.8673278 | 483.3565392 | 436.7444765 | 550.6258584 | 550.2115261 | 523.1918232 | 488.8397072 | 514.8090304 | 519.4652406 |
| ENSMUSG00000028692 | Akr1a1 | 2900 | 370.8063615 | 340.6071126 | 333.0100552 | 331.2287923 | 348.1265858 | 329.030151 | 331.4334732 | 303.2345053 | 324.9616094 | 330.4613944 | 307.2424054 | 306.2932601 | 373.1704377 | 348.8591571 | 337.185009 | 334.5938904 | 328.985003 | 322.0498669 |
| ENSMUSG00000028693 | Nasp | 7697 | 5.665884189 | 5.332140532 | 5.662537463 | 5.274118553 | 4.314801524 | 5.221159794 | 4.634761584 | 4.85282182 | 6.506795267 | 5.984959995 | 4.562114393 | 5.039870794 | 4.281372059 | 4.815172371 | 4.801584466 | 4.138987314 | 5.103865704 | 3.544824858 |
| ENSMUSG00000028696 | Ipp | 2222 | 10.13849833 | 9.160296295 | 9.230592651 | 8.11979008 | 9.076181826 | 8.65277256 | 8.751355103 | 9.608259651 | 11.02879247 | 10.27045021 | 10.83644423 | 10.65069349 | 8.814028424 | 12.26943263 | 9.011681441 | 8.357760841 | 10.31463796 | 10.17495178 |
| ENSMUSG00000028698 | Pik3r3 | 5702 | 11.78545831 | 12.70119818 | 11.5461899 | 13.76739379 | 12.75470653 | 13.37454524 | 5.733612967 | 9.008905864 | 9.505691817 | 8.682609334 | 9.090815494 | 7.645042525 | 5.917753668 | 7.705385246 | 8.345807972 | 7.628574186 | 9.705922962 | 7.22585425 |
| ENSMUSG00000028699 | Tspan1 | 2320 | 112.6753589 | 92.38584543 | 102.4184401 | 93.75363175 | 104.7076805 | 100.6433106 | 90.48549845 | 117.9190424 | 122.4588732 | 108.6295297 | 119.1079137 | 121.9042941 | 94.75130318 | 70.78719526 | 95.278598 | 87.40821414 | 101.4655671 | 115.40057 |
| ENSMUSG00000028700 | Pomgnt1 | 5063 | 18.52832777 | 19.48899717 | 20.7710952 | 19.41946152 | 18.82307108 | 18.9382855 | 19.11944323 | 20.99337466 | 22.0453205 | 23.88733041 | 21.06136823 | 22.86424732 | 17.73337733 | 18.34646944 | 20.10030477 | 20.25325827 | 20.44624293 | 21.31697954 |
| ENSMUSG00000028701 | 1520402A15R | 6197 | 2.455519286 | 1.92448219 | 2.583772559 | 2.808507171 | 2.361159399 | 2.302918714 | 2.649265707 | 2.218696928 | 2.193246765 | 1.613030352 | 2.551801074 | 2.693058434 | 1.44087091 | 1.453954002 | 0.704754708 | 2.341800904 | 2.566631146 | 1.828606743 |
| ENSMUSG00000028702 | Rad54l | 7657 | 0.277557634 | 0.256688097 | 0.227432035 | 0.279661628 | 0.176008079 | 0.090601838 | 0.395456635 | 0.364117197 | 0.145231282 | 0.307892799 | 0.305729785 | 0.44465919 | 0.534745871 | 0.624753951 | 0.737183382 | 0.445065177 | 0.325350568 | 0.402312913 |
| ENSMUSG00000028703 | Lrrc41 | 3841 | 12.72610061 | 15.05628335 | 17.50565144 | 15.31354029 | 14.98718581 | 18.12590229 | 15.40957077 | 16.25737515 | 12.43852077 | 16.62120599 | 14.55264291 | 14.67685999 | 15.48927161 | 13.69985707 | 15.36074669 | 9.570159621 | 16.70352018 | 17.2431566 |
| ENSMUSG00000028706 | Nsun4 | 5781 | 2.588102744 | 2.408716448 | 2.752965142 | 2.774171874 | 2.522742237 | 2.511494713 | 2.226093871 | 2.807779108 | 2.358196976 | 2.618124156 | 2.801541416 | 2.375134578 | 2.551503765 | 1.671055211 | 2.530107653 | 2.53018717 | 2.075096165 | 2.331297552 |
| ENSMUSG00000028707 | Dmbx1 | 9711 | 0 | 0 | 0 | 0 | 0 | 0 | 0 | 0 | 0 | 0 | 0 | 0 | 0 | 0 | 0 | 0 | 0 | 0 |
| ENSMUSG00000028708 | Mknk1 | 4546 | 7.610913613 | 8.148688599 | 5.778008575 | 7.576803677 | 8.120806303 | 7.423099773 | 9.023337578 | 7.905642469 | 8.38951025 | 11.53356402 | 8.407383806 | 7.391339392 | 8.30156549 | 5.813173279 | 5.764229993 | 6.35088385 | 9.13895861 | 6.159180669 |
| ENSMUSG00000028709 | Mob3c | 3114 | 3.412425821 | 5.241913349 | 4.800070931 | 4.184476134 | 4.482420637 | 4.869343762 | 4.755577554 | 6.733342621 | 5.224365358 | 5.405523784 | 6.535687961 | 5.126293995 | 4.911012959 | 7.725766624 | 5.676129569 | 3.77495712 | 5.243096493 | 6.218113541 |

| ENSMUSG00000028710 | Atpaf1 | 4086 | 2.912738445 | 2.225751376 | 2.000765206 | 1.850987117 | 2.320604249 | 1.770606731 | 1.72530327 | 2.224618642 | 1.743824578 | 2.215598391 | 2.022777243 | 2.691063517 | 1.943817592 | 2.080094956 | 2.208306593 | 1.69618207 | 2.523194779 | 2.436771492 |
| --- | --- | --- | --- | --- | --- | --- | --- | --- | --- | --- | --- | --- | --- | --- | --- | --- | --- | --- | --- | --- |
| ENSMUSG00000028712 | Cyp4a31 | 2716 | 0 | 0 | 0 | 0 | 0 | 0 | 0 | 0 | 0 | 0 | 0 | 0 | 0 | 0 | 0 | 0 | 0 | 0 |
| ENSMUSG00000028713 | Cyp4b1 | 2771 | 0.015339291 | 0.084153829 | 0 | 0 | 0.017369875 | 0.035765235 | 0 | 0.110263161 | 0.05945365 | 0 | 0.241374745 | 0.241713443 | 0.783328892 | 0.553105335 | 0.609622444 | 0.096728348 | 0.304286731 | 0 |
| ENSMUSG00000028715 | Cyp4a14 | 2589 | 0 | 0 | 0 | 0 | 0 | 0 | 0.127921496 | 0.059007188 | 0.206807534 | 0.036423873 | 0.018453055 | 0 | 0.019054429 | 0.215268076 | 0 | 0 | 0 | 0.021247241 |
| ENSMUSG00000028716 | Pdzk1ip1 | 2262 | 57.06817842 | 46.71463327 | 55.98669428 | 47.63566307 | 51.60031605 | 45.06188018 | 65.38436531 | 50.02833259 | 54.13240997 | 50.63176756 | 48.42969929 | 46.41435072 | 59.45128492 | 63.01366233 | 55.60935019 | 50.44474361 | 47.30637531 | 49.44010343 |
| ENSMUSG00000028717 | Tal1 | 4372 | 0.009722135 | 0 | 0 | 0 | 0 | 0 | 0 | 0 | 0 | 0 | 0 | 0 | 0 | 0 | 0 | 0 | 0 | 0 |
| ENSMUSG00000028718 | Stil | 6002 | 0.155800379 | 0.133207174 | 0 | 0.273275174 | 0.008019314 | 0.066048295 | 0 | 0 | 0 | 0 | 0 | 0.018599021 | 0.164384929 | 0.185714445 | 0.006864638 | 0.006379641 | 0.166025278 | 0 |
| ENSMUSG00000028719 | Cmpk1 | 3411 | 55.19068444 | 53.54869511 | 54.58508307 | 56.23336167 | 58.87023909 | 54.52108154 | 54.8860365 | 56.26409325 | 59.25014156 | 56.38455543 | 50.88432253 | 49.63028573 | 55.36282009 | 48.17331609 | 46.60088028 | 53.54623199 | 56.96696725 | 51.3644107 |
| ENSMUSG00000028729 | Ebna1bp2 | 3694 | 24.53195325 | 24.14147748 | 22.62844084 | 22.49684485 | 21.74666502 | 23.98491815 | 24.02777089 | 22.05316576 | 25.82240674 | 23.51153097 | 23.03389308 | 23.76774154 | 22.18200179 | 27.84885045 | 24.2257118 | 22.49339958 | 22.80487293 | 22.30742877 |
| ENSMUSG00000028730 | Wdr65 | 4211 | 0 | 0.039554612 | 0.022974842 | 0.010819531 | 0.011430046 | 0.01176745 | 0.056177486 | 0 | 0.039122789 | 0.055985162 | 0 | 0 | 0.023430025 | 0 | 0.009784269 | 0.009092996 | 0 | 0 |
| ENSMUSG00000028736 | Pax7 | 6079 | 0 | 0 | 0 | 0 | 0 | 0 | 0 | 0 | 0 | 0 | 0 | 0 | 0 | 0 | 0 | 0 | 0 | 0 |
| ENSMUSG00000028737 | Aldh4a1 | 5194 | 4.705521027 | 5.182290822 | 4.479720466 | 5.149082215 | 5.272827217 | 5.571581904 | 6.075771437 | 6.095783903 | 6.232691835 | 6.399931805 | 6.916975121 | 5.362344172 | 6.37306825 | 8.664674755 | 6.084249782 | 5.529063448 | 6.5746497 | 6.566354672 |
| ENSMUSG00000028738 | Tas1r2 | 3251 | 0 | 0 | 0 | 0 | 0 | 0 | 0 | 0 | 0 | 0 | 0 | 0 | 0 | 0 | 0 | 0 | 0 | 0 |
| ENSMUSG00000028741 | Mrto4 | 2750 | 14.88453982 | 12.10166594 | 14.03711899 | 12.14408927 | 12.30426994 | 12.79361485 | 13.36798237 | 12.88819979 | 9.061033468 | 12.29347427 | 12.49098005 | 12.07647972 | 11.24767714 | 14.59188782 | 14.54789483 | 11.66819236 | 13.18424613 | 15.90263267 |
| ENSMUSG00000028743 | Akr7a5 | 1786 | 68.75557309 | 68.21122853 | 67.41417528 | 64.03035906 | 65.40659504 | 67.36508238 | 85.45951972 | 74.28914107 | 67.17599127 | 66.84522993 | 74.39090679 | 71.44154797 | 81.04134675 | 87.68726914 | 82.10321244 | 73.64402935 | 63.92723319 | 83.12966352 |
| ENSMUSG00000028744 | Pqlc2 | 2249 | 6.860550866 | 5.924925606 | 5.828913618 | 6.118023739 | 6.784268476 | 6.147271084 | 7.973101518 | 6.588996057 | 5.036145769 | 5.136470564 | 8.029761807 | 5.857045963 | 8.379187372 | 7.702821074 | 6.796699527 | 8.85331963 | 7.157423166 | 8.536317584 |
| ENSMUSG00000028745 | Capzb | 3389 | 169.3559728 | 161.7970607 | 161.4211433 | 165.3722061 | 161.6516834 | 156.1006148 | 172.5816869 | 152.1948641 | 160.1157702 | 160.0814375 | 157.4642332 | 161.2049883 | 168.4476089 | 160.6151384 | 169.7299858 | 179.1715704 | 164.6933791 | 163.4366172 |
| ENSMUSG00000028747 | Htr6 | 2791 | 0.106605601 | 0 | 0 | 0 | 0 | 0 | 0 | 0 | 0 | 0.016893839 | 0 | 0 | 0 | 0 | 0 | 0 | 0 | 0 |
| ENSMUSG00000028749 | Pla2g2f | 2460 | 0.051835581 | 0.379171154 | 0.176976331 | 0.055562249 | 0 | 0.040286775 | 0 | 0.155253668 | 0.301364749 | 0 | 0 | 0.385717993 | 0.581555122 | 0 | 0 | 0 | 0.031159591 | 0.201252829 |
| ENSMUSG00000028750 | Pla2g2c | 3605 | 0 | 0.101648221 | 0.013418455 | 0.025276585 | 0 | 0 | 0 | 0.052971432 | 0 | 0.013079252 | 0.238543482 | 0 | 0 | 0 | 0 | 0 | 0 | 0 |
| ENSMUSG00000028751 | Pla2g2e | 938 | 0.181258746 | 0.177574063 | 0.257854639 | 0.145717626 | 0.051313351 | 0.052828074 | 0 | 0 | 0 | 0 | 0 | 0 | 0.157777987 | 0 | 0 | 0 | 0 | 0 |
| ENSMUSG00000028753 | Vwa5b1 | 7781 | 0 | 0 | 0 | 0.076120496 | 0 | 0 | 0.097288634 | 0 | 0 | 0 | 0 | 0 | 0 | 0 | 0 | 0 | 0 | 0 |
| ENSMUSG00000028755 | Cda | 1503 | 8.172984612 | 5.496738367 | 4.248373923 | 4.213562982 | 4.323226677 | 3.791459988 | 2.39239095 | 2.007451623 | 1.863395257 | 1.097987102 | 1.493960777 | 2.636668026 | 0.295400702 | 0.185405538 | 0.30154166 | 0.076428358 | 0.356998108 | 0.512393542 |
| ENSMUSG00000028756 | Pink1 | 3399 | 24.76029672 | 36.10612012 | 31.69398414 | 36.55338862 | 32.13043095 | 37.0734338 | 34.13082928 | 40.64192983 | 36.25479705 | 37.93973916 | 33.00253089 | 28.86847156 | 31.18984708 | 33.51790125 | 34.60736748 | 28.57995616 | 30.94067629 | 28.7426224 |
| ENSMUSG00000028757 | Ddost | 2109 | 183.8867833 | 182.7233572 | 173.057509 | 180.6671474 | 203.4823288 | 185.3822039 | 185.908094 | 212.7836549 | 199.8200234 | 186.5459782 | 213.9566064 | 206.3247537 | 188.2986885 | 200.4869472 | 186.9015103 | 187.5313816 | 204.6249896 | 193.1187417 |
| ENSMUSG00000028758 | Kif17 | 4566 | 0.698179633 | 1.203816808 | 0.731006043 | 1.097616048 | 1.813116704 | 0.368986628 | 0.632079153 | 1.764913909 | 1.199694831 | 1.290809879 | 1.46484761 | 0.611209621 | 0.853530763 | 1.881768762 | 1.20915648 | 1.408852835 | 1.410165983 | 0.590329879 |
| ENSMUSG00000028759 | Hp1bp3 | 8500 | 30.22868107 | 34.27308941 | 35.31267716 | 33.28100286 | 37.74109073 | 33.36939376 | 29.09451444 | 32.91286443 | 29.13098032 | 28.71200471 | 30.85138201 | 29.19487481 | 20.45243253 | 24.76289459 | 24.71123905 | 22.09593286 | 27.54984401 | 22.34016899 |
| ENSMUSG00000028760 | Eif4g3 | 11152 | 10.63772833 | 13.40640865 | 10.56219032 | 10.95311692 | 12.54658376 | 12.34374945 | 10.26691921 | 10.72277725 | 10.411118 | 11.11965114 | 12.75341207 | 9.739713002 | 10.3025498 | 11.97751034 | 11.5343667 | 11.42331876 | 12.77428673 | 11.74961372 |
| ENSMUSG00000028763 | Hspg2 | 14408 | 1.277397364 | 1.976854842 | 1.584696441 | 1.173177918 | 1.095729523 | 1.74713969 | 0.876768828 | 1.457927632 | 0.914747713 | 0.939217923 | 1.12407766 | 1.22028968 | 0.92445986 | 0.522205869 | 0.806415794 | 1.129477245 | 1.292794301 | 0.729229552 |
| ENSMUSG00000028766 | Alpl | 4015 | 1.259804719 | 0.68866008 | 1.18072378 | 1.009945939 | 1.018982195 | 0.666462668 | 0.212111637 | 0.884655896 | 0.041032643 | 0.035230912 | 0.142789415 | 0.361446383 | 0.589771365 | 1.584767116 | 0.944095428 | 0.944151963 | 0.630021319 | 0.095906288 |
| ENSMUSG00000028771 | Ptpn12 | 4242 | 3.537087963 | 3.526046559 | 3.774549398 | 3.157696142 | 2.972787365 | 2.850275104 | 1.137645745 | 1.296488908 | 2.194283961 | 1.533898409 | 1.700617334 | 2.328942074 | 1.581598479 | 1.598499943 | 1.252946891 | 2.581592107 | 1.165509736 | 2.152643026 |
| ENSMUSG00000028772 | Zcchc17 | 4004 | 12.1974144 | 10.18356207 | 11.69469965 | 9.933764162 | 10.36206748 | 10.50706064 | 18.09083702 | 16.44448096 | 16.91074723 | 14.3665979 | 11.81248973 | 13.01993728 | 24.13617032 | 30.43688444 | 19.59234826 | 16.19030927 | 14.39629134 | 15.86801155 |
| ENSMUSG00000028773 | Fabp3 | 819 | 0 | 0 | 0 | 0 | 0 | 0 | 0 | 0 | 0 | 0 | 0 | 0 | 0.120468662 | 0 | 0 | 0 | 0 | 0 |
| ENSMUSG00000028776 | Tinagl1 | 4345 | 12.09123074 | 17.53431279 | 14.53989198 | 15.18351949 | 14.1681773 | 16.41113547 | 13.60035351 | 14.35401453 | 13.36547462 | 14.09637826 | 13.11749723 | 12.29357632 | 17.40525413 | 14.78300871 | 16.51855244 | 19.66965149 | 15.46283049 | 15.45825531 |
| ENSMUSG00000028777 | Gnat3 | 1281 | 0 | 0 | 0 | 0 | 0 | 0 | 0 | 0 | 0 | 0 | 0 | 0 | 0 | 0 | 0 | 0 | 0 | 0 |
| ENSMUSG00000028778 | Hcrtr1 | 3345 | 0.025414156 | 0 | 0 | 0.013620641 | 0 | 0 | 0 | 0.022835517 | 0 | 0 | 0 | 0.016686297 | 0 | 0 | 0.357203325 | 0 | 0 | 0 |
| ENSMUSG00000028779 | Pef1 | 1909 | 85.7228537 | 77.7242697 | 80.63099717 | 82.3631032 | 82.1949037 | 83.11569037 | 72.220611 | 73.64391475 | 71.5424234 | 73.30711748 | 77.6812316 | 84.81992518 | 83.93403212 | 72.28150007 | 76.27359797 | 81.45529422 | 80.18608163 | 76.76493468 |
| ENSMUSG00000028780 | Sema3c | 7438 | 24.28132468 | 31.15861859 | 26.87924186 | 27.27659726 | 30.34291343 | 29.39986734 | 12.24481137 | 14.74705293 | 15.91423048 | 14.32017191 | 16.30180756 | 15.51852517 | 8.277256354 | 7.574170207 | 7.838155631 | 8.211013553 | 9.434720305 | 6.804030409 |
| ENSMUSG00000028782 | Bai2 | 6602 | 0 | 0.141284617 | 0.117233639 | 0.020703292 | 0.007290506 | 0 | 0 | 0 | 0.006238491 | 0.035709409 | 0 | 0.016908713 | 0 | 0 | 0 | 0.034799098 | 0.023221022 | 0 |
| ENSMUSG00000028784 | Spocd1 | 2037 | 0.041733113 | 0.0490617 | 0.047494875 | 0.08946695 | 0 | 0 | 0.046453293 | 0.018749338 | 0 | 0.092588519 | 0.093814351 | 0.027400914 | 0.048435854 | 0 | 0.101132931 | 0.037595098 | 0.037630139 | 0 |
| ENSMUSG00000028785 | Hpca | 3654 | 0.023265012 | 0 | 0 | 0 | 0 | 0 | 0 | 0 | 0 | 0 | 0.078448208 | 0 | 0 | 0 | 0 | 0.010479093 | 0 | 0 |

| ENSMUSG00000028786 | Tmem54 |  | 1372 | 0 | 0.072841605 | 0.176288376 | 0.664155165 | 0.456060501 | 0.072234306 | 0.310360139 | 0 | 0.060038653 | 0 | 0 | 0 | 0.970817614 | 0.270811005 | 0.42042404 | 0.083725818 | 0.530757756 | 0.801882022 |
| --- | --- | --- | --- | --- | --- | --- | --- | --- | --- | --- | --- | --- | --- | --- | --- | --- | --- | --- | --- | --- | --- |
| ENSMUSG00000028788 | Ptp4a2 |  | 4502 | 43.74199923 | 51.36786132 | 46.36423445 | 44.46806504 | 52.10906174 | 50.57636846 | 43.34017945 | 50.7818127 | 50.39904827 | 44.69995591 | 52.76257076 | 47.55861432 | 37.43177019 | 41.27560943 | 39.05087494 | 50.38506403 | 53.19880705 | 44.23211158 |
| ENSMUSG00000028789 | Adc |  | 4012 | 2.076524053 | 3.545514913 | 3.339847435 | 3.100240554 | 3.071229426 | 3.940010471 | 3.856242774 | 2.617874043 | 2.638318682 | 3.419953801 | 2.857923738 | 2.462455702 | 4.549553687 | 3.727566336 | 2.834404144 | 3.846239976 | 3.8880366 | 2.495428072 |
| ENSMUSG00000028790 | Khdrbs1 |  | 6642 | 16.30580977 | 20.33280234 | 20.06460044 | 20.18075767 | 20.55860696 | 19.68829628 | 15.98459071 | 18.0209258 | 18.32372088 | 14.04866633 | 18.06126479 | 18.08420745 | 13.2205379 | 15.02685092 | 15.86774766 | 14.82737763 | 16.97620679 | 18.12103666 |
| ENSMUSG00000028792 | Ak2 |  | 2131 | 33.56931546 | 32.92208129 | 29.28289731 | 31.15084073 | 29.04629462 | 34.11255756 | 30.70550678 | 35.30691353 | 31.23294673 | 28.85242471 | 33.62854891 | 31.40449528 | 26.04336318 | 29.57514152 | 27.04879244 | 25.87446019 | 25.82663649 | 22.97423979 |
| ENSMUSG00000028793 | Rnf19b |  | 2929 | 9.461718938 | 8.49598224 | 8.274202358 | 10.11084972 | 12.53829217 | 10.15078189 | 5.669767943 | 9.362289152 | 5.188741658 | 8.065040059 | 6.801692621 | 9.299434395 | 6.972828184 | 5.740102299 | 5.879907942 | 7.307766998 | 7.550110383 | 8.601628842 |
| ENSMUSG00000028794 | A3galt2 |  | 3582 | 0.011866325 | 0.102300904 | 0.243083067 | 0 | 0.147808811 | 0.041501452 | 0.118876078 | 0.181259308 | 0.540414919 | 0.144795571 | 0.21340015 | 0.062329048 | 0.302987767 | 0.02593193 | 0.172535829 | 0.149656199 | 0.192594457 | 0.47606988 |
| ENSMUSG00000028795 | Ccdc28b |  | 2161 | 29.54316261 | 26.36050399 | 25.92158637 | 27.32397661 | 25.30211258 | 26.07193803 | 27.17030752 | 27.99480121 | 27.15908605 | 29.08463093 | 27.39156574 | 27.99823149 | 29.5854534 | 26.84343598 | 25.43400072 | 31.55741406 | 25.21980296 | 29.40098022 |
| ENSMUSG00000028796 | Phc2 |  | 6206 | 19.20472343 | 21.17617913 | 19.43203532 | 21.47374391 | 19.28069002 | 21.95778558 | 20.99566835 | 18.56079369 | 16.92987459 | 19.16114625 | 22.87901653 | 20.31704508 | 20.29397113 | 16.83841389 | 17.91862703 | 20.0831336 | 18.16268759 | 20.38687487 |
| ENSMUSG00000028797 | 2510006D16R |  | 3897 | 43.35593398 | 36.61255477 | 37.40042262 | 33.83465802 | 35.17570403 | 35.9597461 | 46.45068247 | 41.44615596 | 41.86291736 | 39.6370808 | 43.3615674 | 41.34970949 | 48.44579097 | 53.4756531 | 42.7134426 | 40.93371066 | 40.5883057 | 46.68080984 |
| ENSMUSG00000028798 | Eif3i |  | 2326 | 63.6846683 | 60.30980119 | 58.70957704 | 57.68584504 | 61.9548493 | 54.49522463 | 63.72769694 | 63.00268617 | 65.53366072 | 62.71894922 | 61.35159113 | 64.86231125 | 67.57157715 | 73.49986487 | 64.19365372 | 66.17723205 | 59.7962732 | 65.58050427 |
| ENSMUSG00000028799 | Zfp362 |  | 4575 | 7.24678411 | 6.822771996 | 7.644603813 | 7.279808396 | 6.796332828 | 6.368744775 | 6.866801925 | 7.496563357 | 8.858476833 | 8.080033083 | 9.085074071 | 7.820292835 | 9.003748836 | 9.035024651 | 9.321007726 | 7.248014428 | 7.88307003 | 8.897647863 |
| ENSMUSG00000028800 | Hdac1 |  | 3759 | 22.08369481 | 23.16571043 | 20.46126982 | 21.2109145 | 21.97243443 | 23.43835071 | 20.01254576 | 23.0129799 | 19.8098485 | 20.48339942 | 24.12260473 | 18.87249459 | 21.77218695 | 20.7818449 | 21.35159108 | 23.9481825 | 25.65282336 | 22.52168535 |
| ENSMUSG00000028801 | 4930555I21Ri | k | 3045 | 0.041877021 | 0.065641171 | 0.238293253 | 0 | 0.142261843 | 0.016273476 | 0.046613477 | 0.100341287 | 0.094681645 | 0.154846316 | 0.031379283 | 0.073321067 | 0.032401916 | 0 | 0.081185332 | 0.025149824 | 0.176212859 | 0 |
| ENSMUSG00000028803 | Nipal3 |  | 7489 | 6.311357423 | 4.964239544 | 4.896132462 | 5.219839236 | 4.788127004 | 5.37278938 | 6.216541069 | 5.400688229 | 5.703086773 | 6.00637881 | 5.256585096 | 7.751140218 | 7.614861313 | 6.027994785 | 5.556625911 | 6.657013064 | 6.903748768 | 5.567753091 |
| ENSMUSG00000028804 | Csmd2 |  | 17103 | 0 | 0 | 0 | 0 | 0 | 0 | 0.002766338 | 0.008932328 | 0 | 0 | 0 | 0.003263501 | 0 | 0.008146656 | 0 | 0.020149416 | 0 | 0.016081713 |
| ENSMUSG00000028807 | Zbtb8a |  | 2316 | 6.478552305 | 8.313839749 | 7.393881583 | 8.419743945 | 7.689469671 | 9.414163547 | 8.028446813 | 9.515119252 | 9.443022396 | 8.062037345 | 7.591185112 | 10.26661151 | 5.069514815 | 7.840949123 | 6.08416758 | 6.216437126 | 5.609937241 | 6.436730536 |
| ENSMUSG00000028809 | Srrm1 |  | 7188 | 48.42444164 | 48.81534708 | 50.18383637 | 46.20757001 | 42.99597689 | 45.91969371 | 48.75417541 | 47.57578897 | 51.81547886 | 46.02900456 | 48.14051534 | 45.59676832 | 51.1506371 | 47.01914635 | 46.32595641 | 47.77269994 | 47.50264039 | 49.15463168 |
| ENSMUSG00000028811 | Yars |  | 5103 | 15.14293749 | 16.05259786 | 13.72621437 | 17.31194689 | 14.40279197 | 14.69200194 | 12.94307266 | 10.52293117 | 9.297837773 | 11.36495493 | 12.15204705 | 11.67064707 | 11.2429982 | 12.78736876 | 13.51585431 | 13.01865645 | 13.52648652 | 14.45565591 |
| ENSMUSG00000028813 | CK137956 |  | 2312 | 0 | 0 | 0 | 0 | 0 | 0 | 0 | 0 | 0 | 0 | 0 | 0 | 0 | 0 | 0 | 0 | 0 | 0 |
| ENSMUSG00000028820 | Sfpq |  | 4731 | 20.38559383 | 17.26552877 | 20.14285665 | 17.28642456 | 17.37673338 | 16.56994811 | 17.85101076 | 19.38278549 | 20.51055403 | 18.02908945 | 16.65206226 | 16.75295728 | 19.59304007 | 16.96372501 | 21.24087821 | 19.59449599 | 19.86389347 | 16.68528179 |
| ENSMUSG00000028821 | Syf2 |  | 2154 | 67.70439134 | 68.58991916 | 61.48873079 | 65.02072901 | 66.47747118 | 67.6116453 | 70.68347298 | 69.25697483 | 77.70752092 | 72.12700429 | 70.04332353 | 73.07343013 | 77.08970917 | 76.39340837 | 76.81775694 | 73.8258554 | 69.98018836 | 80.59830122 |
| ENSMUSG00000028822 | Tmem50a |  | 1348 | 140.3175321 | 138.4412711 | 137.0822596 | 134.0468115 | 134.0052741 | 130.9030299 | 139.7268359 | 140.048253 | 153.4717127 | 133.5120433 | 143.0413445 | 139.3321251 | 137.1998204 | 136.6448446 | 128.4033652 | 151.8557768 | 146.7374023 | 139.8080116 |
| ENSMUSG00000028825 | Rhd |  | 1584 | 0 | 0.063092603 | 0.06107769 | 0.086289857 | 0 | 0.125133166 | 0.149345577 | 0.024111365 | 0 | 0 | 0 | 0 | 0 | 0 | 0.052022167 | 0.07252009 | 0 | 0 |
| ENSMUSG00000028826 | Tmem57 |  | 8537 | 9.350441675 | 9.732032117 | 8.465509471 | 8.843229526 | 7.724110979 | 8.770549424 | 6.994096381 | 7.216158496 | 7.921782697 | 8.262557347 | 7.174358296 | 8.218377401 | 8.74880201 | 7.175793753 | 8.392820159 | 8.777640703 | 9.311085835 | 7.880536198 |
| ENSMUSG00000028830 | AU040320 |  | 6175 | 21.52448347 | 26.13780931 | 24.68420958 | 28.4212377 | 26.16661826 | 28.39953427 | 20.31188856 | 22.33405103 | 19.08252981 | 24.16712159 | 26.82360827 | 23.60076033 | 22.40907335 | 23.94785008 | 28.01705801 | 23.23480258 | 28.17836242 | 30.94763348 |
| ENSMUSG00000028832 | Stmn1 |  | 1948 | 2.814767817 | 4.32657199 | 2.657067632 | 2.315474019 | 3.508589918 | 3.942850978 | 3.23028044 | 3.33301253 | 7.209754556 | 2.880356102 | 3.433518576 | 4.670407085 | 3.773334535 | 6.461164108 | 3.637919719 | 2.81086081 | 5.764684288 | 3.106263727 |
| ENSMUSG00000028833 | Ncdn |  | 4243 | 20.62648066 | 22.07774182 | 21.9807133 | 22.66777388 | 20.56638643 | 22.96032859 | 17.00491053 | 18.60563181 | 20.43309349 | 21.29171516 | 19.09647174 | 19.57428846 | 21.26516066 | 15.45582168 | 23.35369831 | 20.03420892 | 23.68407977 | 19.38218585 |
| ENSMUSG00000028834 | Trim63 |  | 3092 | 0.206202342 | 0.086191188 | 0 | 0.073675686 | 0.062266395 | 0 | 0 | 0.06176003 | 0 | 0 | 0.015451151 | 0.19856801 | 0.207411036 | 0 | 0 | 0 | 0.111557785 | 0 |
| ENSMUSG00000028836 | Slc30a2 |  | 3685 | 44.7082937 | 49.57609549 | 45.52493983 | 45.02939579 | 51.31894928 | 44.77899669 | 29.05524896 | 31.74581508 | 30.87032742 | 30.69593952 | 36.24933078 | 32.06560589 | 22.89215157 | 38.60467834 | 29.092659 | 27.02683035 | 34.45726503 | 31.00513288 |
| ENSMUSG00000028837 | Psmb2 |  | 1364 | 232.9994143 | 208.9627001 | 221.7947645 | 207.8305116 | 218.3227359 | 217.0656768 | 209.3343236 | 203.3101421 | 209.2238766 | 210.4151985 | 220.9066445 | 191.5492057 | 227.3463578 | 212.0628858 | 203.5912659 | 226.7399091 | 210.5698453 | 220.1574147 |
| ENSMUSG00000028838 | Extl1 |  | 4320 | 2.843517563 | 2.760651882 | 2.743406235 | 2.140947222 | 2.974820282 | 3.16586909 | 2.069929703 | 2.139481801 | 2.36441109 | 2.717714146 | 2.28921676 | 2.325652608 | 1.986980001 | 2.064181662 | 1.010964103 | 1.382716378 | 1.224312262 | 1.400694847 |
| ENSMUSG00000028840 | Zfp593 |  | 3709 | 0.275040233 | 0.5478799 | 0.404308288 | 0.528208387 | 0.272518306 | 0.227122262 | 0.293392185 | 0.442780616 | 0.399761275 | 0.38137533 | 0.540994407 | 0.331071603 | 0.319214347 | 0.313049928 | 0.355473116 | 0.443919148 | 0.733665969 | 0.756393757 |
| ENSMUSG00000028841 | Cnksr1 |  | 3176 | 9.127370923 | 11.81055381 | 6.930087 | 8.65028644 | 9.289946224 | 9.049302732 | 7.061149185 | 9.896834754 | 6.782288332 | 6.294678269 | 8.423796205 | 7.943538882 | 8.294472237 | 7.83817092 | 7.394485786 | 8.608152923 | 7.807655572 | 6.148687937 |
| ENSMUSG00000028842 | Eif2c3 |  | 16549 | 0.929776646 | 0.988375797 | 1.040605282 | 0.977350338 | 0.805640393 | 0.883319621 | 0.920580253 | 1.153918738 | 0.793914951 | 0.880389589 | 1.085466456 | 1.143362718 | 0.897269146 | 0.867195782 | 0.736942446 | 0.981039189 | 1.107013712 | 0.531842231 |
| ENSMUSG00000028843 | Sh3bgrl3 |  | 1211 | 52.22766468 | 65.74551378 | 52.20826109 | 45.71153499 | 64.14940116 | 51.84418947 | 93.0235248 | 114.6087451 | 100.2284574 | 73.93822088 | 92.98561283 | 81.67246416 | 141.1520176 | 223.1310491 | 102.340446 | 107.5994525 | 123.3024383 | 93.2565616 |
| ENSMUSG00000028845 | Tekt2 |  | 2025 | 0.335843366 | 0.098704872 | 0.191105305 | 0.269991374 | 0.047537702 | 0.048940971 | 0.327100002 | 0.018860446 | 0.406780403 | 0.023284298 | 0.306703437 | 0.275632902 | 0.487228813 | 0.160547463 | 0.142425131 | 0.226907303 | 0.094632832 | 0 |
| ENSMUSG00000028847 | Trappc3 |  | 1869 | 91.15074667 | 78.46086696 | 79.43197681 | 74.86247574 | 81.89380271 | 79.5388982 | 89.89155852 | 73.17656127 | 82.74765473 | 77.17175028 | 80.31509874 | 76.86972472 | 86.86535032 | 82.12825512 | 89.34719424 | 84.05904878 | 75.05310148 | 79.58513889 |
| ENSMUSG00000028848 | Gpn2 |  | 1927 | 13.74194326 | 12.9828664 | 12.80254306 | 12.76749556 | 12.01424768 | 11.31458369 | 12.47267301 | 11.65393493 | 11.92634966 | 13.11508923 | 12.27223894 | 15.64113015 | 10.54735336 | 12.8703387 | 13.36324465 | 11.48519516 | 10.58100153 | 14.95836633 |
| ENSMUSG00000028849 | Mtap7d1 |  | 4218 | 5.371090285 | 6.973751919 | 5.780051991 | 7.118238079 | 5.899526917 | 6.70806327 | 3.981982224 | 4.862332881 | 4.120604469 | 4.415487858 | 4.088847798 | 5.518049146 | 4.25718774 | 3.094070307 | 3.829068257 | 3.731019356 | 3.934397003 | 4.4210733 |
| ENSMUSG00000028850 | Gpatch3 |  | 2079 | 4.109447033 | 5.079455251 | 2.652516816 | 4.996593607 | 4.398780903 | 2.884021531 | 4.392182315 | 3.912930117 | 2.931988616 | 4.830735832 | 4.067420707 | 4.080798804 | 5.505052818 | 3.127547972 | 3.824867863 | 3.794066919 | 1.732886921 | 5.60939424 |

| ENSMUSG00000028851 | Nudc | 1417 | 107.0278533 | 113.409599 | 102.7894848 | 100.4143553 | 99.59266069 | 108.8621451 | 109.0829372 | 105.3862339 | 106.7594018 | 104.6832127 | 96.7299618 | 95.12690554 | 92.98909742 | 99.83676089 | 98.22078744 | 104.1169445 | 96.26202588 | 105.2432522 |
| --- | --- | --- | --- | --- | --- | --- | --- | --- | --- | --- | --- | --- | --- | --- | --- | --- | --- | --- | --- | --- |
| ENSMUSG00000028854 | Slc9a1 | 6325 | 5.22158447 | 6.430836971 | 6.424310751 | 7.275360439 | 6.78032316 | 6.761108155 | 5.834607046 | 6.340240715 | 6.8307755 | 7.745388251 | 5.098513355 | 6.609633404 | 7.347140881 | 6.924391211 | 6.631333422 | 6.326274266 | 6.853287241 | 4.783400585 |
| ENSMUSG00000028857 | Tmem222 | 2087 | 47.08766985 | 40.68738254 | 43.76100877 | 41.84979492 | 43.21956155 | 42.66711176 | 39.85418762 | 36.27088719 | 42.27192945 | 43.82959479 | 41.15925987 | 45.27835015 | 51.12838384 | 48.24666098 | 46.23576612 | 47.44586044 | 47.34316886 | 51.45077926 |
| ENSMUSG00000028859 | Csf3r | 4263 | 0 | 0 | 0 | 0 | 0 | 0 | 0 | 0 | 0 | 0 | 0 | 0 | 0 | 0 | 0 | 0 | 0 | 0 |
| ENSMUSG00000028860 | Sytl1 | 2357 | 8.60202332 | 12.72023963 | 11.39045793 | 11.46274896 | 10.43505006 | 10.1964683 | 9.394286694 | 9.057935063 | 8.544847776 | 10.24232502 | 10.11442693 | 8.880302703 | 11.88821766 | 11.33022921 | 8.547968108 | 8.398915582 | 8.666913979 | 9.942248394 |
| ENSMUSG00000028861 | Mrps15 | 1907 | 33.03233921 | 31.61842608 | 35.36061946 | 29.07592604 | 33.74534973 | 30.60992142 | 36.96690698 | 30.84231762 | 39.99865086 | 33.15631519 | 35.42411709 | 41.8251609 | 42.16624287 | 29.49333499 | 31.28466331 | 34.77678659 | 34.12587947 | 33.25930784 |
| ENSMUSG00000028862 | Map3k6 | 4497 | 1.020804761 | 1.859358783 | 1.850177278 | 1.702080375 | 2.033592505 | 1.311268689 | 2.041062867 | 2.352522897 | 1.91416095 | 2.579291306 | 1.848530748 | 2.44511575 | 2.007502971 | 4.389312239 | 2.840222888 | 0.987705239 | 1.764185781 | 1.859324933 |
| ENSMUSG00000028863 | Meaf6 | 1815 | 18.61796967 | 20.46494604 | 20.46879962 | 18.92728784 | 18.00637806 | 15.78042976 | 19.7853021 | 21.37932829 | 18.90268189 | 18.18484423 | 16.58304344 | 15.77599719 | 12.9649171 | 14.43221228 | 12.62152346 | 13.6706962 | 15.92177844 | 14.30539855 |
| ENSMUSG00000028864 | Hgf | 2736 | 0 | 0 | 0.212164607 | 0 | 0 | 0 | 0 | 0 | 0 | 0 | 0 | 0 | 0 | 0 | 0 | 0 | 0 | 0 |
| ENSMUSG00000028865 | Cd164l2 | 1652 | 10.08597397 | 11.27234132 | 13.20609091 | 11.50057838 | 11.59594771 | 9.808561671 | 21.59428566 | 18.26392125 | 18.00040217 | 19.09432232 | 22.44150592 | 20.10309881 | 21.62004123 | 26.37079538 | 26.13754877 | 24.4299638 | 23.29273734 | 24.74077863 |
| ENSMUSG00000028868 | Wasf2 | 6463 | 20.9796552 | 18.47337349 | 19.1233746 | 16.60161835 | 16.75643327 | 18.63115312 | 15.12424488 | 16.92449953 | 18.56993763 | 17.60399922 | 15.24245155 | 16.05466761 | 16.14374208 | 15.64424853 | 15.28722436 | 16.34593622 | 16.43826319 | 15.26092037 |
| ENSMUSG00000028869 | Gnl2 | 3542 | 23.00463649 | 25.15866517 | 24.15945093 | 25.46890677 | 25.15307473 | 24.5525261 | 22.37400825 | 26.26671154 | 21.55838516 | 23.4022971 | 24.72374333 | 26.07987622 | 20.90547934 | 26.04120767 | 22.85744137 | 23.01544414 | 25.92597611 | 25.03520046 |
| ENSMUSG00000028871 | Rspo1 | 1932 | 0.044001217 | 0.137941591 | 0 | 0 | 0.049826008 | 0.076945238 | 0.024488964 | 0 | 0.063954217 | 0 | 0.024728239 | 0.028890095 | 0.051068237 | 0 | 0.021325857 | 0.019819155 | 0.039675256 | 0.028472623 |
| ENSMUSG00000028873 | Cdca8 | 5164 | 0.485632336 | 0.787020351 | 0.412167958 | 0.405849737 | 0.438071342 | 0.393427978 | 0.92536417 | 0.89490331 | 1.443601734 | 0.83088962 | 0.629104798 | 0.767411318 | 1.394744369 | 0.52164157 | 1.579765311 | 0.748906148 | 1.625379361 | 1.757649615 |
| ENSMUSG00000028874 | Fgr | 4017 | 0.010581323 | 0.041464892 | 0 | 0 | 0 | 0 | 0.247340368 | 0.380307716 | 0 | 0 | 0.190291097 | 0.041684587 | 0.135088646 | 0.127180722 | 0 | 0.00953214 | 0 | 0 |
| ENSMUSG00000028876 | Epha10 | 6469 | 0 | 0 | 0 | 0 | 0 | 0 | 0 | 0.005903911 | 0 | 0 | 0.007385215 | 0 | 0 | 0.007179485 | 0 | 0 | 0 | 0 |
| ENSMUSG00000028878 | Fam76a | 4135 | 15.04899098 | 18.30396509 | 17.02139944 | 17.40905684 | 14.96920286 | 16.87309525 | 17.18588724 | 15.65565226 | 17.62005958 | 14.92630485 | 17.73508132 | 17.10240496 | 15.55715118 | 16.81421979 | 17.1681453 | 17.11270677 | 19.98343316 | 19.0104023 |
| ENSMUSG00000028879 | Stx12 | 3179 | 40.39261931 | 40.52247938 | 40.82610316 | 38.83939294 | 39.89544475 | 41.04194637 | 36.90954506 | 34.67230995 | 35.65438546 | 39.08213372 | 37.81119708 | 40.08403828 | 36.17260119 | 35.69138266 | 42.04399098 | 41.63907826 | 43.08845081 | 37.51486108 |
| ENSMUSG00000028882 | Ppp1r8 | 3293 | 9.513001741 | 9.32720573 | 9.430855295 | 9.29760759 | 7.922108299 | 8.8180692 | 6.939576047 | 9.835152517 | 10.95638864 | 7.94674774 | 7.848846807 | 10.42412162 | 8.898620972 | 9.449601746 | 8.933468238 | 10.30230129 | 9.334251478 | 8.168676947 |
| ENSMUSG00000028883 | Sema3a | 9870 | 0.521086758 | 0.114755664 | 0.156834141 | 0.346208544 | 0.209693284 | 0.215883236 | 0.349931668 | 0.046434532 | 0.070939288 | 0.487271705 | 0.188776432 | 0.458061669 | 2.70400884 | 0.903471609 | 1.494443466 | 2.172516732 | 1.27366012 | 1.437928018 |
| ENSMUSG00000028884 | Rpa2 | 3115 | 6.768079393 | 7.229379291 | 7.79567006 | 9.828899452 | 9.116479924 | 8.542477667 | 9.416969803 | 9.808642672 | 9.678500654 | 10.42916037 | 9.340272781 | 10.32097003 | 9.407113599 | 9.512464769 | 8.888425541 | 8.321907291 | 8.698777499 | 8.81205273 |
| ENSMUSG00000028885 | Smpdl3b | 1998 | 0.170190895 | 0.083365601 | 0.411586595 | 0.159623278 | 0.168630363 | 0.768836207 | 0.44992037 | 1.567455955 | 0 | 0.755166419 | 0.334759469 | 0.111743068 | 0 | 0.092981156 | 0 | 0 | 0.019182331 | 0 |
| ENSMUSG00000028886 | Eya3 | 5736 | 2.134151098 | 1.81199843 | 2.437229824 | 2.478215801 | 2.08101762 | 2.047419431 | 2.169322622 | 2.010827323 | 2.261811797 | 1.824870314 | 1.774070111 | 2.724613934 | 2.683326044 | 2.388599334 | 1.953769735 | 2.403176197 | 2.52568693 | 2.26327566 |
| ENSMUSG00000028889 | Yrdc | 2383 | 31.83874914 | 31.09017069 | 28.66278844 | 29.15677407 | 27.65111351 | 28.71688001 | 27.53784544 | 32.71115959 | 30.41891219 | 32.64736063 | 32.31776461 | 33.91568587 | 29.12715384 | 34.08758239 | 27.73281395 | 29.56555502 | 28.20995584 | 31.94821809 |
| ENSMUSG00000028890 | Mtf1 | 8894 | 4.84599151 | 4.640732622 | 4.345676946 | 4.43623391 | 5.141143185 | 5.34861977 | 5.351535305 | 6.222261197 | 5.468998776 | 6.091315274 | 5.822808072 | 5.56650469 | 4.326388068 | 4.642319956 | 6.059324838 | 5.751771024 | 4.589330583 | 5.943754392 |
| ENSMUSG00000028893 | Sesn2 | 3465 | 28.0914439 | 28.265486 | 26.10634972 | 31.04462501 | 28.85137243 | 28.63046829 | 16.42630671 | 18.02152321 | 18.00795711 | 21.82676133 | 16.5316523 | 20.68320657 | 20.71513409 | 27.66539578 | 22.81840859 | 21.93560049 | 20.3743258 | 25.81379727 |
| ENSMUSG00000028894 | Inpp5b | 4673 | 16.22709909 | 18.85568269 | 22.96008781 | 22.01515902 | 23.32951147 | 21.96099106 | 15.07566423 | 11.25421316 | 17.12505888 | 13.59126841 | 15.53989662 | 15.02591559 | 15.18067559 | 14.97779578 | 15.30620608 | 14.82296358 | 17.02661938 | 12.27787253 |
| ENSMUSG00000028896 | Rcc1 | 4911 | 4.050584887 | 4.29384281 | 4.048365094 | 4.42529386 | 3.214675415 | 3.803987082 | 2.100216658 | 2.698587586 | 2.549521644 | 2.582679531 | 2.188834384 | 3.693767123 | 1.878450118 | 3.376204276 | 3.355858757 | 3.508607887 | 1.990064286 | 2.901111513 |
| ENSMUSG00000028898 | Trnau1ap | 2485 | 28.56484667 | 24.51882637 | 25.15034254 | 23.00970247 | 27.62017028 | 23.76935953 | 24.00856665 | 23.94517624 | 25.45774173 | 24.13508835 | 24.18547194 | 20.59676563 | 21.837066 | 23.43697609 | 25.26807693 | 24.43819048 | 25.06246779 | 21.33954885 |
| ENSMUSG00000028899 | Taf12 | 4104 | 10.48129584 | 8.709730872 | 9.618128843 | 9.614002046 | 7.576321318 | 7.812041575 | 7.804747474 | 6.821401307 | 7.607061147 | 8.616722087 | 8.369930591 | 8.336988588 | 9.676460382 | 7.016406964 | 8.744287325 | 8.294432256 | 7.965967656 | 8.390765304 |
| ENSMUSG00000028901 | Gmeb1 | 11341 | 1.079401349 | 0.919401807 | 0.703785602 | 0.879805018 | 1.039795547 | 0.67287902 | 0.876083465 | 1.111321118 | 0.512062316 | 0.719255062 | 0.758265808 | 0.880963196 | 1.104867912 | 0.700285596 | 0.559477966 | 0.98250302 | 0.557608587 | 0.465644497 |
| ENSMUSG00000028902 | Sf3a3 | 2875 | 27.26245029 | 25.09764483 | 24.22882912 | 27.24154268 | 26.08331724 | 27.76676654 | 22.46323713 | 24.08446106 | 23.10742606 | 25.12517474 | 22.89874532 | 21.45262858 | 20.1617401 | 24.10246205 | 23.33082887 | 22.34839625 | 20.62288046 | 25.16068708 |
| ENSMUSG00000028906 | Epb4.1 | 11566 | 9.308802601 | 8.84522723 | 8.151479393 | 9.745635802 | 8.323002536 | 10.35526172 | 8.925839998 | 9.622398462 | 10.72216833 | 11.37797102 | 9.455030257 | 10.04257253 | 9.903917682 | 7.657692776 | 8.955620916 | 10.39865103 | 11.01143737 | 9.226843078 |
| ENSMUSG00000028907 | Utp11l | 4894 | 10.20506372 | 8.011703411 | 8.935364005 | 8.890641059 | 7.700714391 | 8.019159173 | 9.522474202 | 7.226433312 | 9.274119405 | 9.672927272 | 8.287891244 | 8.336994146 | 9.233354357 | 10.04991593 | 10.48981174 | 11.06312196 | 8.825855458 | 9.284332275 |
| ENSMUSG00000028909 | Ptpru | 7604 | 0.083847664 | 0.39866825 | 0.18448611 | 0.107850973 | 0.215213757 | 0.091233334 | 0.335992196 | 0.150680178 | 0.319569231 | 0.111613974 | 0.257597751 | 0.058722423 | 0.402232887 | 0.69629484 | 0.400962012 | 0.327313188 | 0.231852927 | 0.224261219 |
| ENSMUSG00000028910 | Mecr | 2942 | 16.52818538 | 15.04855079 | 15.06123515 | 13.56610294 | 14.7569664 | 15.12520556 | 14.47362714 | 14.38381437 | 12.03956613 | 14.07148792 | 16.82354078 | 14.58947809 | 16.08066236 | 14.36577822 | 14.01861232 | 19.22339467 | 16.03659806 | 15.57531812 |
| ENSMUSG00000028911 | Srsf4 | 3008 | 7.574060622 | 9.479999153 | 7.863915008 | 10.73895626 | 8.784716122 | 10.01597806 | 7.864474556 | 9.433828121 | 7.476009842 | 10.39259184 | 9.466048954 | 10.27987935 | 8.659325905 | 8.152419595 | 8.355368715 | 7.115840937 | 9.173847663 | 12.30755613 |
| ENSMUSG00000028914 | Casp9 | 3844 | 11.94214103 | 11.83803681 | 12.96168998 | 13.55927022 | 10.38016772 | 11.51160018 | 12.6528184 | 12.10154686 | 8.357305488 | 12.87935443 | 10.83760765 | 11.28219819 | 9.791949241 | 12.62592906 | 11.71521868 | 11.91351885 | 10.94752185 | 12.263997 |
| ENSMUSG00000028917 | Plekhm2 | 5166 | 5.010772784 | 5.951955355 | 4.354179562 | 6.067750388 | 5.021894475 | 5.831990324 | 3.315367745 | 4.243600267 | 4.791540064 | 4.326255003 | 4.032110317 | 5.801976541 | 2.635619274 | 3.964739167 | 4.298807322 | 4.291572142 | 4.228801633 | 5.377390417 |
| ENSMUSG00000028919 | Arhgef19 | 4191 | 1.024343302 | 1.557940173 | 0.992632692 | 1.239312588 | 1.056582433 | 1.241478651 | 0.067734687 | 0.145807311 | 0.746880267 | 0.360014914 | 0.250787184 | 0.226405694 | 0.153021934 | 0.332455887 | 0.393238424 | 0.74918392 | 0.16460829 | 0.603774495 |
| ENSMUSG00000028920 | Fbxo42 | 6181 | 5.253835057 | 5.621315106 | 4.421783635 | 6.486607186 | 5.232915823 | 5.684013607 | 4.676920696 | 4.992632445 | 3.291722826 | 5.751760274 | 4.645324389 | 4.397707115 | 4.17417776 | 4.876591606 | 3.22626647 | 4.392176128 | 4.278457347 | 5.286427664 |

| ENSMUSG00000028923 | Necap2 | 2792 | 54.94311615 | 61.82930439 | 54.1603352 | 55.62951294 | 59.26846475 | 59.49167728 | 47.0246003 | 46.56408947 | 47.27893375 | 49.26167672 | 55.45796577 | 48.5588268 | 48.51914213 | 44.78061721 | 45.71719919 | 48.17869047 | 47.79805366 | 48.74374285 |
| --- | --- | --- | --- | --- | --- | --- | --- | --- | --- | --- | --- | --- | --- | --- | --- | --- | --- | --- | --- | --- |
| ENSMUSG00000028926 | Cdk14 | 18653 | 0.041017164 | 0.003571854 | 0.072613459 | 0.024425585 | 0.041286162 | 0.031878668 | 0 | 0.055283057 | 0 | 0.012638906 | 0 | 0.068823258 | 0.050249634 | 0.05477778 | 0.039759181 | 0.032844568 | 0.012328193 | 0 |
| ENSMUSG00000028927 | Padi2 | 4996 | 0.17015683 | 0.346731485 | 0.66808919 | 0.227987612 | 0.43353414 | 0.119021778 | 0.132581566 | 0.267560866 | 0.296780338 | 0.056626145 | 0.133876985 | 0.368678316 | 0.009874283 | 0 | 0 | 0 | 0 | 0 |
| ENSMUSG00000028931 | Kcnab2 | 7933 | 0.214320817 | 0.197366195 | 0.04878208 | 0.413512567 | 0.133480691 | 0.356045104 | 0.369770086 | 0.298490981 | 0.347850316 | 0.130759545 | 0.475762224 | 0.309578867 | 0.298491369 | 0.06439997 | 0.15581075 | 0.482674996 | 0.188418704 | 0.839039697 |
| ENSMUSG00000028932 | Psmc2 | 3466 | 60.446628 | 59.81135047 | 59.67836578 | 56.62924957 | 61.62997035 | 58.23089552 | 52.32241729 | 48.15372144 | 46.39127453 | 52.17049827 | 47.38900959 | 53.51282365 | 50.29976793 | 47.54287999 | 50.78276017 | 52.4424447 | 51.45189252 | 50.15256124 |
| ENSMUSG00000028933 | Xrcc2 | 5988 | 1.490662486 | 1.201667527 | 1.332937961 | 1.643484565 | 0.739501833 | 1.183373564 | 1.627657291 | 0.854673 | 1.045482698 | 0.787419894 | 1.539840846 | 0.745700235 | 0.766177072 | 1.116891881 | 0.749994922 | 1.489931784 | 1.792144812 | 0.845163296 |
| ENSMUSG00000028936 | Rpl22 | 2801 | 24.8111256 | 18.04200662 | 19.96422745 | 19.73064862 | 17.99147593 | 18.92946267 | 21.5364747 | 18.12127911 | 19.92421594 | 20.97457203 | 19.37606313 | 16.67893952 | 21.87441673 | 23.51221561 | 21.19651626 | 18.15420444 | 21.05825453 | 23.29196736 |
| ENSMUSG00000028937 | Acot7 | 2112 | 11.69294852 | 9.732033965 | 9.436503081 | 11.95114515 | 11.09860172 | 10.04193654 | 9.99121913 | 9.96402165 | 8.502519352 | 7.858450543 | 7.396975105 | 7.822649681 | 13.99139131 | 9.653860968 | 10.72957185 | 11.40378412 | 9.291223492 | 8.048207376 |
| ENSMUSG00000028938 | Galntl5 | 1760 | 0 | 0 | 0 | 0 | 0 | 0 | 0 | 0 | 0 | 0 | 0 | 0 | 0 | 0 | 0 | 0 | 0 | 0 |
| ENSMUSG00000028940 | Hes2 | 2633 | 0 | 0.063260338 | 0.220464248 | 0 | 0.01828026 | 0 | 0 | 0 | 0.093854574 | 0 | 0 | 0 | 0 | 0 | 0 | 0 | 0.203785855 | 0 |
| ENSMUSG00000028943 | Espn | 6421 | 3.157603016 | 3.834017883 | 3.186731559 | 3.21432068 | 3.110846959 | 3.017461272 | 3.441056075 | 4.847657368 | 5.407293691 | 4.17093902 | 3.958305234 | 5.545926293 | 3.595598394 | 4.491789171 | 3.721679243 | 1.604138513 | 1.975705385 | 3.692403831 |
| ENSMUSG00000028944 | Prkag2 | 7504 | 9.397133133 | 9.588999402 | 8.657469519 | 8.991991826 | 9.73029427 | 10.16940428 | 9.041362151 | 8.005950024 | 8.721398394 | 9.066959595 | 9.244301667 | 8.925745616 | 8.671215204 | 7.266172521 | 8.482996249 | 7.261131974 | 9.081044229 | 6.810162599 |
| ENSMUSG00000028945 | Rheb | 5621 | 78.17443689 | 76.96754265 | 76.63516951 | 72.27678924 | 78.57294638 | 76.8547823 | 76.74737706 | 76.77209656 | 76.4306255 | 77.02148324 | 71.02923466 | 72.72618979 | 78.00428407 | 66.84445241 | 74.87527013 | 86.24072046 | 82.89144128 | 76.45101258 |
| ENSMUSG00000028946 | Hes3 | 1883 | 0 | 0 | 0 | 0 | 0 | 0 | 0 | 0 | 0 | 0 | 0 | 0 | 0 | 0 | 0 | 0 | 0 | 0 |
| ENSMUSG00000028948 | Nol9 | 4913 | 3.175127133 | 3.505549829 | 3.790720372 | 2.33693938 | 4.036302168 | 4.044503596 | 3.072001746 | 2.907380114 | 2.39758671 | 3.973212121 | 4.11333349 | 3.64682021 | 2.831589797 | 5.804329261 | 1.903674575 | 2.657662757 | 3.198408657 | 4.00839817 |
| ENSMUSG00000028949 | Smarcd3 | 4816 | 27.70426652 | 25.48270397 | 27.45117493 | 25.81729443 | 27.19413711 | 24.64260733 | 37.49844175 | 31.3009577 | 31.32603971 | 26.93346858 | 26.83373395 | 28.80023288 | 35.12440705 | 42.71197322 | 29.9857669 | 18.19916951 | 21.84503426 | 31.77643997 |
| ENSMUSG00000028950 | Tas1r1 | 5437 | 0.203261831 | 0.324734853 | 0.177941991 | 0.125697198 | 0.2567272 | 0.364559379 | 0.226251545 | 0.625183707 | 0.477239377 | 0.164771632 | 0.123018102 | 0.533826458 | 0.499035396 | 0.375857981 | 0.378899723 | 0.408470706 | 0.204425715 | 0.647523051 |
| ENSMUSG00000028952 | Zbtb48 | 3422 | 5.042986986 | 4.312569298 | 5.131382676 | 4.19395937 | 4.078976582 | 3.924252133 | 5.281543936 | 5.569260316 | 5.355932061 | 4.491855424 | 3.699697252 | 4.664897574 | 6.559328568 | 6.270358964 | 4.033466167 | 4.53176388 | 4.838386985 | 4.40458657 |
| ENSMUSG00000028953 | Abcf2 | 3147 | 24.74403638 | 25.75477332 | 26.50014818 | 26.81253705 | 25.40423426 | 22.39084434 | 20.95769762 | 23.36205104 | 18.27021799 | 24.28703209 | 26.44549656 | 20.14826587 | 21.19375664 | 22.90474218 | 20.77752437 | 18.03199243 | 25.36818445 | 21.20306529 |
| ENSMUSG00000028954 | Nub1 | 3440 | 21.0054649 | 24.23900414 | 23.63834434 | 24.1447046 | 24.23386389 | 23.72481169 | 27.79619887 | 30.57612681 | 29.02212622 | 29.03057834 | 28.17889266 | 32.09400599 | 29.16893016 | 31.87630533 | 32.9971763 | 33.4820195 | 34.90589773 | 35.18024267 |
| ENSMUSG00000028955 | Vamp3 | 2884 | 36.32984012 | 32.95481526 | 33.81450666 | 33.83902807 | 34.48008124 | 34.05461788 | 41.11150256 | 42.78767412 | 38.63020983 | 43.45581458 | 39.21058485 | 41.39726154 | 33.629178 | 42.06378511 | 40.18723187 | 41.80898846 | 49.78166026 | 33.89430744 |
| ENSMUSG00000028957 | Per3 | 11758 | 3.723450528 | 4.1959854 | 4.426766343 | 3.413784662 | 4.081266193 | 3.940449557 | 4.257255852 | 3.81339347 | 4.946024856 | 3.28426824 | 4.567022736 | 4.865713059 | 4.900466013 | 4.93354866 | 4.278542247 | 3.52685217 | 4.446085131 | 4.631656375 |
| ENSMUSG00000028958 | Tmub1 | 2644 | 20.94714235 | 17.1478249 | 17.78330995 | 18.64487518 | 16.8388916 | 19.19137639 | 19.48695437 | 20.33846543 | 19.53399802 | 18.51075264 | 22.31545905 | 19.73814089 | 19.3110947 | 17.65367164 | 18.21657293 | 17.21918767 | 17.16275928 | 16.51937622 |
| ENSMUSG00000028959 | Fastk | 4934 | 29.87595267 | 33.33990102 | 30.77513413 | 35.63438794 | 32.81633385 | 30.00883014 | 33.17832774 | 32.27854034 | 31.59524973 | 32.01355004 | 37.46277145 | 28.04358078 | 30.78495608 | 32.95515808 | 31.28111641 | 29.6996665 | 29.41663688 | 30.76005785 |
| ENSMUSG00000028960 | Ube4b | 7432 | 6.817299492 | 7.548293039 | 6.00763839 | 8.772585367 | 6.430974193 | 7.140874282 | 5.18835217 | 6.459613808 | 6.567010385 | 6.686873148 | 5.213333063 | 6.47377572 | 5.854514402 | 4.380692312 | 6.491795203 | 4.920281226 | 5.486972538 | 6.09896447 |
| ENSMUSG00000028961 | Pgd | 6435 | 21.22286411 | 22.15165103 | 19.96582698 | 21.58042939 | 22.22221371 | 19.99820499 | 19.56473017 | 24.36360713 | 25.59516341 | 20.56019788 | 28.70209627 | 20.36599468 | 26.08037025 | 22.61217184 | 24.17029891 | 21.76644007 | 23.29357377 | 23.67913374 |
| ENSMUSG00000028962 | Slc4a2 | 6461 | 4.966941325 | 5.01678472 | 5.577817693 | 7.143373768 | 5.952237581 | 5.867178639 | 5.162581434 | 5.893487881 | 4.870221025 | 6.035231635 | 5.213023638 | 5.986728706 | 3.145759158 | 3.536680235 | 3.628491766 | 4.57519717 | 3.855764276 | 4.205927677 |
| ENSMUSG00000028963 | Uts2 | 534 | 0 | 0.062383697 | 0.181174271 | 0.170640615 | 0 | 0 | 0 | 0 | 0 | 0.441485986 | 0 | 0 | 0 | 0.434869732 | 0.231469415 | 0.215115772 | 0.071772092 | 0 |
| ENSMUSG00000028964 | Park7 | 2769 | 124.5682569 | 110.5256624 | 117.8678979 | 109.6986214 | 112.5164109 | 105.2257398 | 127.6707609 | 114.4254859 | 117.7883779 | 121.3930529 | 118.3935591 | 111.7925839 | 129.9483585 | 121.7375176 | 121.4620082 | 116.0195724 | 106.2866139 | 125.6329328 |
| ENSMUSG00000028965 | Tnfrsf9 | 2708 | 0.156961507 | 0.172223235 | 0 | 0 | 0.355479495 | 0.073194584 | 0.017471447 | 0.211553189 | 0.015209201 | 0.0522349 | 0.158779404 | 0.164891175 | 0.109302623 | 0.017150697 | 0.213006567 | 0.070699054 | 0.05661196 | 0 |
| ENSMUSG00000028967 | Errfi1 | 3576 | 47.43796351 | 43.41109817 | 40.43302076 | 49.71453999 | 54.43106804 | 42.95678806 | 36.45034408 | 60.03341553 | 24.69348149 | 30.02297296 | 37.6348037 | 34.54140417 | 27.48019553 | 35.72921823 | 39.07004363 | 76.67758378 | 71.86180669 | 62.05445652 |
| ENSMUSG00000028969 | Cdk5 | 2038 | 37.29109653 | 32.56098395 | 33.23009936 | 31.18628893 | 37.10267521 | 34.42918094 | 36.40151156 | 36.80563215 | 40.47919091 | 31.71914336 | 39.26548354 | 29.93450403 | 41.39233491 | 35.16350679 | 35.48024072 | 38.42212568 | 31.25530199 | 33.8206137 |
| ENSMUSG00000028970 | Abcb1b | 4316 | 0 | 0.061747719 | 0 | 0.158344686 | 0.033455925 | 0.057405854 | 0 | 0.008849027 | 0 | 0.03277389 | 0 | 0 | 0.194310147 | 0 | 0 | 0 | 0.062160352 | 0.140199299 |
| ENSMUSG00000028971 | Cort | 675 | 0 | 0.049352436 | 0 | 0 | 0.213919661 | 0.073411457 | 0 | 0 | 0.122034121 | 0 | 0.070777716 | 0 | 0.365421609 | 0.412836332 | 0 | 0 | 0 | 0 |
| ENSMUSG00000028972 | Car6 | 2103 | 0 | 0.015840653 | 0.023002154 | 0.021664786 | 0.045774535 | 0 | 0 | 0 | 0.156677188 | 0 | 0 | 0 | 0 | 0 | 0 | 0 | 0 | 0 |
| ENSMUSG00000028973 | Abcb8 | 4070 | 15.38332292 | 19.44752744 | 17.0555322 | 17.17214791 | 16.9466945 | 17.34954431 | 15.89101526 | 17.10681808 | 15.9281513 | 17.58593797 | 16.97360933 | 17.23839997 | 12.27843543 | 12.42693149 | 14.09153951 | 14.0555694 | 14.633677 | 17.31367708 |
| ENSMUSG00000028974 | Dffa | 3533 | 4.559711779 | 4.393945289 | 4.792169721 | 5.50652871 | 4.577505336 | 4.530295201 | 7.338621017 | 5.124030212 | 7.134488437 | 5.258244292 | 6.477272887 | 5.466238113 | 8.601319287 | 6.11279384 | 6.553997852 | 6.968825519 | 7.669598616 | 5.760931075 |
| ENSMUSG00000028975 | Pex14 | 6262 | 4.262735635 | 3.824971406 | 4.264163008 | 4.969369735 | 4.427337597 | 4.27315173 | 4.359536209 | 3.470373198 | 4.169953848 | 4.630738183 | 4.013035477 | 3.422742643 | 3.615993298 | 3.152145822 | 3.717483085 | 4.732821803 | 4.18639206 | 4.322018606 |
| ENSMUSG00000028976 | Slc2a5 | 3388 | 0.727656496 | 0.560458964 | 0.585393962 | 0.416880866 | 0.284131781 | 0.672793903 | 1.522161158 | 1.217467373 | 0.607829335 | 0.556678905 | 0.197417184 | 1.169690686 | 0.611552694 | 1.082964257 | 0.267542571 | 0.429469623 | 0.067874198 | 0.162364542 |
| ENSMUSG00000028977 | Casz1 | 12264 | 3.226705714 | 2.724464522 | 2.697936625 | 2.90514813 | 2.527475444 | 3.058661066 | 1.898062462 | 2.469551134 | 2.988910554 | 2.572066249 | 2.107489607 | 2.703400487 | 2.152036509 | 2.116947558 | 2.274417265 | 2.791242907 | 2.54383608 | 2.157483719 |
| ENSMUSG00000028978 | Nos3 | 3959 | 0 | 0.042072359 | 0.012218623 | 0.034524661 | 0.072945578 | 0.025032955 | 0.250963945 | 0.077175857 | 0.187258723 | 0.178646261 | 0.072404585 | 0 | 0.112146314 | 0.516175763 | 0.364247147 | 0.038687151 | 0.232339259 | 0.361262131 |

| ENSMUSG00000028979 | Masp2 | 3322 | 0.038385168 | 0.09025167 | 0.160177253 | 0.027429888 | 0.130399552 | 0.089499218 | 0.113937818 | 0.149458528 | 0.086786758 | 0.014193469 | 0.186957995 | 0.470451099 | 0 | 0.041942282 | 0.012402636 | 0.011526372 | 0.092296922 | 0.264944524 |
| --- | --- | --- | --- | --- | --- | --- | --- | --- | --- | --- | --- | --- | --- | --- | --- | --- | --- | --- | --- | --- |
| ENSMUSG00000028980 | H6pd | 5228 | 8.723805256 | 10.3481523 | 9.946737783 | 8.915254085 | 9.326250703 | 11.53513328 | 7.466136212 | 8.364632891 | 8.941602026 | 7.332349225 | 6.661810392 | 6.384423532 | 5.21816187 | 5.205859834 | 6.998274987 | 5.068305341 | 6.348617658 | 4.166718872 |
| ENSMUSG00000028982 | Slc25a33 | 1880 | 24.77961326 | 22.13181111 | 19.58098755 | 25.34938956 | 25.1156474 | 23.88020033 | 20.13305486 | 22.87481123 | 17.43854604 | 23.22422937 | 21.01589929 | 25.94834527 | 24.90212207 | 29.29930194 | 27.0659157 | 31.14166953 | 34.45289456 | 35.31701692 |
| ENSMUSG00000028986 | Klhl7 | 3408 | 15.98932971 | 15.71805572 | 18.96333232 | 16.65758839 | 15.38018336 | 18.33509303 | 18.26980207 | 16.529869 | 16.66555318 | 17.64000782 | 17.59318453 | 16.57436929 | 16.97955956 | 11.40660245 | 17.10686666 | 18.69588343 | 16.02552027 | 17.48088691 |
| ENSMUSG00000028988 | Ctnnbip1 | 2786 | 10.8322595 | 11.35938604 | 10.26157805 | 10.10650589 | 10.98776147 | 12.73501696 | 10.1384312 | 9.966215559 | 9.520501131 | 9.172893455 | 9.174301072 | 10.83857626 | 10.5180039 | 12.78629398 | 9.745809523 | 9.277157213 | 10.79904633 | 9.260327011 |
| ENSMUSG00000028989 | Angptl7 | 2152 | 1.599869544 | 3.854512389 | 4.203461978 | 1.841919543 | 3.422018737 | 3.500007204 | 0.505665249 | 0.230716186 | 0.363635595 | 0.372473028 | 0.310803633 | 0.622479508 | 0 | 0.38847285 | 0.076582818 | 0.088965166 | 0.124667323 | 0.332304083 |
| ENSMUSG00000028990 | Lzic | 1783 | 12.20563664 | 11.97620033 | 13.86364218 | 12.72540666 | 13.49745476 | 11.39462746 | 13.58614224 | 14.11598047 | 14.02143302 | 12.42895711 | 13.58491528 | 13.96174174 | 12.5335718 | 13.75349307 | 12.29345358 | 12.34834507 | 13.45611994 | 13.29720976 |
| ENSMUSG00000028991 | Mtor | 9487 | 6.169455823 | 8.293987156 | 7.357753168 | 7.002003019 | 5.418456254 | 6.999121218 | 5.874811403 | 6.638481244 | 5.565628039 | 6.316911968 | 5.378270861 | 5.848083547 | 5.314347998 | 4.924923781 | 4.820673241 | 4.8352638 | 6.576916977 | 6.378203583 |
| ENSMUSG00000028992 | Nmnat1 | 3826 | 4.854877659 | 4.919442037 | 6.346971312 | 4.751400076 | 3.786646373 | 5.530323376 | 5.960457722 | 4.88136037 | 5.31786168 | 5.139007647 | 5.893826556 | 5.018449537 | 4.680472026 | 5.195522583 | 5.782863438 | 3.22257595 | 3.365822206 | 7.562673846 |
| ENSMUSG00000028995 | Fam126a | 7349 | 0.803948764 | 0.453298329 | 0.862285002 | 0.526968127 | 0.39951658 | 0.917018882 | 1.094455765 | 0.514498277 | 0.257801024 | 0.635177524 | 0.858116005 | 0.683550093 | 0.892794257 | 0.49926288 | 0.549428831 | 1.292158203 | 0.662326807 | 0.232042769 |
| ENSMUSG00000028996 | Rbp7 | 933 | 0 | 0 | 0 | 0 | 0.051588343 | 0.212444731 | 0.050710267 | 0 | 0 | 0 | 0 | 0 | 0 | 0.099558601 | 0 | 0 | 0 | 0 |
| ENSMUSG00000028998 | Tomm7 | 1122 | 61.25745957 | 39.75576235 | 50.96035016 | 48.44414072 | 55.72492767 | 47.9628063 | 67.00521107 | 56.40357467 | 60.38486491 | 54.546892 | 52.20329688 | 60.79032057 | 71.53567683 | 75.29571743 | 60.92101713 | 63.6811706 | 60.52958834 | 72.51200432 |
| ENSMUSG00000028999 | Rint1 | 7059 | 4.088541511 | 4.299199126 | 4.378904363 | 3.988769712 | 4.063837159 | 3.966184229 | 3.967857476 | 4.182281776 | 3.845008342 | 3.907516845 | 4.460078997 | 3.700485917 | 3.97646422 | 5.276690478 | 4.062371852 | 4.757169952 | 3.952619938 | 3.467779075 |
| ENSMUSG00000029001 | Fbxo44 | 3855 | 9.04130852 | 6.593447027 | 5.935325514 | 9.419494769 | 5.968108823 | 5.27019994 | 14.89950512 | 8.668833231 | 8.568504706 | 15.48451111 | 9.926781256 | 7.5144822 | 20.16786034 | 15.04764336 | 14.30030656 | 24.93110884 | 13.1830531 | 10.40250034 |
| ENSMUSG00000029003 | Mad2l2 | 2611 | 12.6489934 | 10.27073146 | 10.57881495 | 9.562409918 | 9.825475034 | 9.546160471 | 11.16224061 | 8.981284978 | 11.15238094 | 10.85314924 | 11.93001644 | 11.43675966 | 14.37824169 | 13.80337488 | 12.8133525 | 13.77054016 | 12.74118181 | 11.1450852 |
| ENSMUSG00000029004 | Mll5 | 7800 | 30.3203589 | 29.71681 | 27.65973659 | 27.24900921 | 26.60831472 | 29.35046527 | 26.43444292 | 27.94410776 | 31.75057939 | 25.43115495 | 27.44610115 | 30.46247123 | 24.19161328 | 25.14530526 | 25.59250491 | 27.79996278 | 28.10103743 | 28.11106403 |
| ENSMUSG00000029005 | 2610109H07R | 5193 | 0 | 0 | 0 | 0 | 0 | 0.076337737 | 0 | 0 | 0 | 0 | 0 | 0 | 0 | 0.107323137 | 0 | 0 | 0.007380377 | 0 |
| ENSMUSG00000029007 | Agtrap | 4317 | 6.340823109 | 6.728942265 | 7.115402311 | 8.485309161 | 6.199061746 | 6.680493617 | 12.60356284 | 9.811298186 | 10.12251407 | 16.63435744 | 11.37657107 | 8.830692042 | 15.58692035 | 18.38613513 | 15.66174501 | 17.09196212 | 16.69062733 | 15.29092612 |
| ENSMUSG00000029009 | Mthfr | 6794 | 2.396155787 | 3.383264205 | 2.976175403 | 2.917140754 | 3.173844834 | 3.398818641 | 2.381650897 | 2.799501972 | 2.934099739 | 2.505358239 | 1.842366665 | 3.154726882 | 2.991573435 | 2.830122487 | 1.995188679 | 2.87433173 | 2.070319541 | 4.080746216 |
| ENSMUSG00000029012 | Orc5 | 3537 | 11.87308847 | 11.4151054 | 12.00790491 | 9.40332552 | 7.811061404 | 9.778854407 | 11.19613013 | 10.92754064 | 12.41301267 | 11.01116225 | 10.98135178 | 10.71496604 | 12.44107159 | 11.85722672 | 13.86198799 | 11.26958505 | 13.38223825 | 9.564772585 |
| ENSMUSG00000029014 | Dnajc2 | 4795 | 8.430119375 | 7.87143048 | 6.779356079 | 8.209539584 | 7.658948438 | 8.608431089 | 7.390447657 | 8.036732852 | 8.872924042 | 7.040647243 | 5.888425539 | 8.753572109 | 6.851940961 | 7.680951262 | 7.896606854 | 8.584442744 | 7.217652993 | 6.367060319 |
| ENSMUSG00000029015 | Slc26a5 | 3375 | 0 | 0 | 0.057331592 | 0 | 0 | 0 | 0 | 0 | 0 | 0.125735209 | 0 | 0.198455689 | 0 | 0.165134533 | 0 | 0 | 0 | 0 |
| ENSMUSG00000029016 | Clcn6 | 6636 | 2.241834179 | 3.16261654 | 2.288922322 | 2.608980839 | 2.262983753 | 3.987516536 | 2.509653855 | 3.614350318 | 1.992295292 | 2.181323975 | 3.210914931 | 3.641980395 | 2.245063143 | 2.603556436 | 3.46450696 | 2.002236403 | 2.9628376 | 3.796589946 |
| ENSMUSG00000029017 | Pmpcb | 5128 | 18.41780443 | 17.01374219 | 17.38541662 | 18.26706458 | 17.29858333 | 18.17642197 | 17.95446045 | 16.9586779 | 19.30015549 | 17.91138259 | 17.21687271 | 16.47911723 | 15.77697822 | 12.58011649 | 17.30658178 | 15.29234867 | 18.7595564 | 17.03480138 |
| ENSMUSG00000029019 | Nppb | 778 | 0 | 0 | 0 | 0 | 0 | 0 | 0 | 0 | 0 | 0 | 0 | 0 | 0 | 0 | 0 | 0 | 0 | 0 |
| ENSMUSG00000029020 | Mfn2 | 6616 | 10.20869479 | 13.26272119 | 11.35491122 | 14.32390753 | 11.56010077 | 12.47806139 | 12.52899236 | 14.14896891 | 8.796334155 | 12.94933915 | 12.22536347 | 9.575389516 | 9.260919174 | 10.84584568 | 11.61440474 | 8.970743874 | 11.28470773 | 10.18533188 |
| ENSMUSG00000029022 | Miip | 3648 | 7.072544366 | 6.967581768 | 6.935130585 | 8.442781242 | 5.924132052 | 6.778183677 | 7.729812676 | 7.307647171 | 7.09022256 | 8.91830736 | 6.364755982 | 4.819609023 | 12.18422628 | 8.313593494 | 6.279623102 | 8.879894151 | 6.78694841 | 3.558703176 |
| ENSMUSG00000029026 | Trp73 | 8594 | 0.004945913 | 0 | 0 | 0 | 0 | 0.023063874 | 0 | 0.031108543 | 0.023962367 | 0 | 0 | 0 | 0.022961097 | 0 | 0 | 0 | 0 | 0 |
| ENSMUSG00000029027 | Dffb | 2343 | 1.215470249 | 2.488158979 | 1.775554251 | 2.83905782 | 2.711657671 | 2.199523812 | 4.543471088 | 2.526599391 | 2.830144702 | 3.964442399 | 2.915842536 | 2.69192056 | 2.610822767 | 3.905029968 | 3.165292386 | 3.644389864 | 2.273732509 | 2.653021366 |
| ENSMUSG00000029028 | Lrrc47 | 2924 | 9.012725423 | 11.10809571 | 12.14301343 | 11.06304428 | 8.576173814 | 12.1339799 | 9.870292116 | 10.61915977 | 8.69086191 | 9.836501022 | 9.983071009 | 7.94094242 | 7.474021667 | 10.00676301 | 11.63901681 | 9.742890529 | 10.66949579 | 9.481733854 |
| ENSMUSG00000029029 | Wdr8 | 5306 | 4.614206821 | 4.32577914 | 4.421628764 | 4.207484305 | 4.15462138 | 4.408007962 | 4.101739975 | 4.556311859 | 4.416722101 | 4.514239965 | 2.746204737 | 4.46020372 | 4.090848775 | 3.772597374 | 3.401108458 | 2.475250347 | 3.026520619 | 3.027263318 |
| ENSMUSG00000029030 | Tprgl | 2663 | 97.09312271 | 96.44852213 | 98.6725561 | 103.5260531 | 100.1859756 | 95.38389498 | 86.31054984 | 97.42432953 | 96.44728212 | 100.781751 | 108.5924986 | 104.6936668 | 98.4600602 | 88.42340333 | 96.06476174 | 99.5296975 | 96.1539578 | 101.6728589 |
| ENSMUSG00000029032 | Arhgef16 | 3972 | 25.86480626 | 30.5368197 | 28.83900299 | 29.29579739 | 27.44682958 | 32.66089036 | 31.38693579 | 34.16354626 | 31.86459288 | 31.35070677 | 28.27767557 | 24.57744055 | 21.54855903 | 22.89464328 | 24.95743793 | 20.44672163 | 26.34209228 | 30.73142191 |
| ENSMUSG00000029033 | Acap3 | 5505 | 2.154213281 | 2.608148485 | 2.135289351 | 2.23460163 | 2.194570907 | 1.80928237 | 1.796248846 | 1.85238355 | 1.907822256 | 1.695883605 | 1.388554652 | 0.922656729 | 1.164968074 | 1.434240664 | 1.90103455 | 1.912791469 | 1.942422677 | 1.838633177 |
| ENSMUSG00000029034 | Cpsf3l | 3136 | 16.90177121 | 13.92640444 | 12.88006947 | 15.08047322 | 15.10261891 | 15.23240917 | 12.68812595 | 12.73891993 | 14.45993426 | 12.10341713 | 13.07108239 | 12.6724336 | 12.12847839 | 14.85440678 | 13.7426108 | 15.42124909 | 13.41909247 | 14.48900579 |
| ENSMUSG00000029036 | Atad3a | 3932 | 15.73945481 | 14.99588575 | 15.55039226 | 15.14452822 | 14.89739346 | 18.05927447 | 16.2441802 | 16.45420388 | 12.88387955 | 14.79755031 | 19.18531522 | 14.59270121 | 13.1108478 | 17.35156775 | 13.42298909 | 17.73326452 | 13.15882523 | 17.5575862 |
| ENSMUSG00000029038 | Ssu72 | 3021 | 61.47140485 | 55.46635482 | 53.5935803 | 51.00544586 | 58.39241981 | 53.29256253 | 55.33124616 | 52.5287759 | 51.08436761 | 52.80067167 | 53.24670149 | 51.19669018 | 56.23936547 | 53.53138441 | 52.71235138 | 53.29758495 | 51.01292282 | 45.41301297 |
| ENSMUSG00000029044 | 4930584F24R | 758 | 0 | 0 | 0 | 0 | 0 | 0 | 0 | 0 | 0 | 0 | 0 | 0 | 0 | 0 | 0 | 0 | 0 | 0 |
| ENSMUSG00000029047 | Pex10 | 3335 | 6.844161777 | 6.292990512 | 7.513489872 | 7.144955379 | 6.379103527 | 4.294074964 | 6.554260169 | 7.306372634 | 6.001993002 | 7.804254332 | 9.927450143 | 7.213358493 | 7.4996348 | 6.503564861 | 8.227956889 | 7.141456613 | 6.918270086 | 5.888531065 |
| ENSMUSG00000029048 | Rer1 | 1806 | 256.4196527 | 221.36658 | 238.1177657 | 221.4226391 | 235.5159521 | 221.8623498 | 264.3327411 | 241.8006252 | 267.6439363 | 255.9348527 | 243.8744974 | 240.1989644 | 271.4074917 | 255.9310951 | 245.7036306 | 286.1194612 | 251.9646307 | 241.1750316 |
| ENSMUSG00000029049 | Morn1 | 4168 | 4.844039974 | 5.211134124 | 5.00215729 | 5.159504061 | 5.427544176 | 4.482097063 | 6.731386421 | 5.95610882 | 6.541620306 | 5.486586152 | 6.923242543 | 5.436938342 | 6.16648966 | 5.058928905 | 6.880106262 | 7.817971905 | 6.078138736 | 6.361417812 |

| ENSMUSG00000029050 | Ski | 7523 | 10.46385564 | 11.16333993 | 11.41983117 | 12.43950353 | 9.43700484 | 12.5149799 | 8.439932889 | 9.305685712 | 6.323331876 | 9.188213608 | 7.023674608 | 7.011272251 | 7.809957926 | 9.025821582 | 8.330129803 | 8.281113684 | 8.319399554 | 9.483824461 |
| --- | --- | --- | --- | --- | --- | --- | --- | --- | --- | --- | --- | --- | --- | --- | --- | --- | --- | --- | --- | --- |
| ENSMUSG00000029053 | Prkcz | 6887 | 10.64018055 | 9.64995266 | 10.63416944 | 10.96852207 | 10.35042529 | 11.38986166 | 13.14889901 | 10.59760142 | 9.837638802 | 11.8304654 | 14.06818873 | 9.936111853 | 10.63712751 | 10.61463533 | 11.00782094 | 12.06484944 | 12.75502723 | 10.46347173 |
| ENSMUSG00000029054 | Gabrd | 2271 | 0.355613538 | 0.308045257 | 0.383409752 | 0.140434747 | 0.741795389 | 0.26183743 | 0.479168479 | 0.891324231 | 0.199494352 | 0.228382975 | 0.483850306 | 0.368663557 | 0.586508924 | 0.061352823 | 0.63498655 | 0.910476794 | 0 | 1.283788048 |
| ENSMUSG00000029055 | Plch2 | 8882 | 0.669975754 | 1.30146074 | 0.642656674 | 1.072084921 | 0.563580282 | 1.389172558 | 1.145263 | 1.277093393 | 0.695561514 | 0.955542286 | 1.242514682 | 1.878955541 | 0.588739387 | 0.70068765 | 0.760758294 | 1.202778593 | 1.363556611 | 1.734130813 |
| ENSMUSG00000029056 | Pank4 | 2934 | 10.45969226 | 9.537433928 | 11.4751115 | 11.16509573 | 9.810119411 | 11.2819448 | 8.75623199 | 10.77822399 | 14.03766727 | 10.4297227 | 11.70763297 | 8.066067122 | 8.507822134 | 9.038709577 | 6.600112907 | 11.15830584 | 7.132296561 | 12.93670199 |
| ENSMUSG00000029059 | 2810405K02R | 1019 | 29.11541989 | 22.4592329 | 22.73888228 | 17.57162946 | 24.46745482 | 20.71586311 | 24.8403172 | 21.92596213 | 31.16271212 | 28.22564179 | 31.45927099 | 30.34531607 | 52.33346671 | 42.6155267 | 39.34162304 | 34.38263569 | 30.27739844 | 39.62383154 |
| ENSMUSG00000029060 | Mib2 | 4964 | 8.083176102 | 8.475863295 | 6.792173783 | 11.33519133 | 8.678096632 | 10.80097859 | 11.39926753 | 8.65561094 | 8.05642764 | 11.37923902 | 7.728302107 | 9.107712872 | 6.31058974 | 8.532837973 | 8.109170045 | 7.435968077 | 7.481503163 | 4.665357359 |
| ENSMUSG00000029061 | Mmp23 | 2473 | 0.120313883 | 0.080823844 | 0.078242669 | 0 | 0 | 0.160299987 | 0.325238796 | 0 | 0 | 0.266926747 | 0.42500974 | 0.112850106 | 0.07979283 | 0.206585104 | 0.18326612 | 0.557404718 | 0.464936881 | 0.71180405 |
| ENSMUSG00000029062 | Cdk11b | 3370 | 27.42025302 | 28.6767674 | 27.53128523 | 25.25460855 | 25.47992636 | 26.6731925 | 26.54844981 | 30.78058307 | 29.64940274 | 27.22708265 | 25.40436961 | 27.54345605 | 27.65222307 | 31.78043487 | 26.67709049 | 27.12156673 | 26.88527177 | 26.34560779 |
| ENSMUSG00000029063 | Nadk | 4267 | 93.71659153 | 107.1992853 | 100.9983084 | 107.8964971 | 102.039227 | 109.5920194 | 77.83806095 | 84.51199051 | 76.76502457 | 79.45009525 | 77.10947716 | 81.20544489 | 68.07272766 | 77.5845922 | 78.74354073 | 78.67207759 | 83.53282428 | 87.80572437 |
| ENSMUSG00000029064 | Gnb1 | 4470 | 85.31463967 | 85.71921908 | 83.15485621 | 79.26806304 | 84.25778584 | 86.60088472 | 76.11308147 | 73.06112594 | 75.9140276 | 74.39684789 | 69.80274135 | 71.41158262 | 71.77959507 | 63.34890398 | 69.23151819 | 72.46947173 | 71.1137151 | 66.23244125 |
| ENSMUSG00000029066 | Mrpl20 | 1168 | 76.49476028 | 67.53847047 | 65.68529036 | 61.35918043 | 64.69787685 | 64.91068696 | 80.85283146 | 74.22667248 | 71.01852975 | 73.63260509 | 72.60321173 | 69.72179085 | 94.18679411 | 95.51258383 | 76.15938285 | 78.64654722 | 76.39008492 | 74.22461661 |
| ENSMUSG00000029068 | Ccnl2 | 5381 | 46.6048204 | 45.87410261 | 46.05418993 | 53.28296822 | 44.54506223 | 45.17853762 | 46.32791401 | 48.84596048 | 49.63660188 | 50.45414409 | 55.88098656 | 51.36576122 | 52.95319721 | 50.10368469 | 54.39432318 | 53.44033852 | 58.52577247 | 56.01094513 |
| ENSMUSG00000029070 | Mxra8 | 2680 | 1.205370663 | 1.57863342 | 1.028840011 | 1.59803663 | 1.275136784 | 2.551595983 | 1.377010805 | 1.895369224 | 1.214080129 | 2.251973887 | 2.584839171 | 1.8327531 | 2.392966137 | 1.975606702 | 2.490541811 | 2.900370635 | 2.102225987 | 2.298888042 |
| ENSMUSG00000029071 | Dvl1 | 4819 | 25.40255384 | 29.766616 | 24.64349803 | 30.44336227 | 25.59911192 | 29.59385085 | 26.56736028 | 27.85770792 | 26.07596173 | 35.68346437 | 27.73901926 | 24.53155704 | 20.13610319 | 23.61237064 | 24.25582363 | 18.64869383 | 20.59074137 | 23.8916913 |
| ENSMUSG00000029072 | Tas1r3 | 3517 | 0.205455784 | 0.303102819 | 0.302592456 | 0.453408175 | 0.19159708 | 0.324058252 | 0.067262836 | 0.130312434 | 0.140528345 | 0.040219536 | 0.163008104 | 0.285664466 | 0.210400557 | 0.369756738 | 0.351449155 | 0.359280649 | 0.163461602 | 0.046922752 |
| ENSMUSG00000029073 | Gltpd1 | 3437 | 14.71665972 | 14.10249086 | 12.65283788 | 12.16905403 | 13.8640106 | 15.9601036 | 16.76661127 | 16.90155486 | 15.11090962 | 14.11640199 | 14.90100538 | 17.5550571 | 17.61136529 | 13.24271331 | 14.14542798 | 16.15402407 | 12.96871786 | 12.13177274 |
| ENSMUSG00000029074 | Ttll10 | 3635 | 3.776938613 | 4.848011291 | 5.349699918 | 5.151468837 | 3.482447297 | 3.994209337 | 4.139045914 | 4.822644485 | 4.928785243 | 3.333626062 | 4.49492044 | 4.406903759 | 5.252118841 | 3.718082374 | 3.547754331 | 4.07660662 | 4.006600502 | 5.735474951 |
| ENSMUSG00000029075 | Tnfrsf4 | 1508 | 0.535542669 | 0.508087909 | 0.641558758 | 0.151064471 | 0.287259491 | 0.328599029 | 0.752986933 | 0.202612214 | 0.901296432 | 0.187602268 | 0.475215104 | 0.62922166 | 0.785123352 | 0.092395399 | 1.256811387 | 0.126958247 | 0.60996759 | 0.218869125 |
| ENSMUSG00000029076 | Sdf4 | 6201 | 38.82427302 | 39.35122563 | 41.18887926 | 35.44371519 | 40.89001353 | 38.96454264 | 50.65455175 | 52.45065294 | 53.70652579 | 49.94887433 | 51.6117476 | 51.68416942 | 64.12115034 | 62.26997945 | 57.98516018 | 61.22421745 | 57.30711577 | 58.22041081 |
| ENSMUSG00000029082 | Bst1 | 2634 | 0.451839381 | 0.290887079 | 0.018365046 | 0.46702665 | 0.438559669 | 0.206940042 | 0.017962293 | 0.014499773 | 0.547277165 | 0 | 0.126964582 | 0.233095022 | 0.299662368 | 0 | 0 | 0.348889361 | 0 | 0 |
| ENSMUSG00000029084 | Cd38 | 3006 | 0.452483577 | 0.343546148 | 0.225292557 | 0.030313403 | 0.288215112 | 0.016484609 | 2.360912122 | 1.308655172 | 1.589366543 | 0.501936961 | 4.815639527 | 0.464202117 | 1.854459964 | 3.043740923 | 0.808679906 | 0.292975373 | 0.203998919 | 0.073199077 |
| ENSMUSG00000029086 | Prom1 | 4677 | 27.79149632 | 28.21303699 | 38.27890439 | 41.74237224 | 23.99906906 | 17.71481089 | 15.30555553 | 20.61099501 | 23.55653831 | 22.7839618 | 16.70131647 | 11.14642481 | 50.67148398 | 74.4575085 | 42.31154008 | 11.57642054 | 17.37262122 | 25.00520866 |
| ENSMUSG00000029088 | Kcnip4 | 5101 | 0.024998143 | 0.248165062 | 0.227595516 | 0.259021816 | 0.264201894 | 0.048571588 | 0.009275177 | 0.119795812 | 0.242226127 | 0.120164505 | 0.03746321 | 0.196957837 | 0.009671029 | 0.227622463 | 0.008077153 | 0.150129807 | 0.1728102 | 0.075487894 |
| ENSMUSG00000029089 | Pacrgl | 1455 | 12.44481442 | 11.03561169 | 9.940677372 | 11.33546258 | 12.63669749 | 10.69385453 | 10.2104338 | 12.07457396 | 13.67222482 | 10.01344832 | 12.90416404 | 11.776913 | 10.51058031 | 13.40654069 | 17.98143505 | 16.50048855 | 16.35782156 | 17.23996747 |
| ENSMUSG00000029090 | Gpr125 | 4428 | 5.193157234 | 8.523827721 | 6.784092672 | 7.706689749 | 8.63070176 | 8.728801308 | 3.71834062 | 4.674860457 | 4.01819666 | 6.772323232 | 8.663796671 | 6.340397082 | 5.258506087 | 5.370228713 | 6.987888093 | 5.629445668 | 5.703936237 | 5.652471445 |
| ENSMUSG00000029092 | 4930435H24R | 404 | 0 | 0 | 0 | 0 | 0 | 0 | 0 | 0 | 0 | 0 | 0 | 0 | 0 | 0 | 0 | 0 | 0 | 0 |
| ENSMUSG00000029093 | Sorcs2 | 8772 | 0.164748744 | 0.205072536 | 0.424619453 | 0.171399278 | 0.279836766 | 0.350235919 | 0.026968011 | 0.169802063 | 0.046952252 | 0.064501646 | 0.119818637 | 0.076355216 | 0.005623794 | 0.052945836 | 0.093938796 | 0 | 0.052429955 | 0.137961736 |
| ENSMUSG00000029094 | Afap1 | 8599 | 3.727049974 | 4.292439446 | 3.90971085 | 4.646707278 | 4.472311549 | 3.734175062 | 2.52547036 | 2.074177454 | 3.453364096 | 2.993869517 | 2.533478435 | 2.778125781 | 2.512778203 | 2.052419259 | 2.357386382 | 2.832053298 | 2.246360465 | 1.311416081 |
| ENSMUSG00000029095 | Ablim2 | 6779 | 0.388747738 | 0.668321819 | 0.235481118 | 0.40325456 | 0.475710117 | 0.57015979 | 0.537406148 | 0.332401791 | 0.358460603 | 0.570343364 | 1.028934052 | 0.642221815 | 0.392967035 | 0.486433132 | 0.425447546 | 0.892449619 | 0.791515204 | 0.14606342 |
| ENSMUSG00000029096 | Htra3 | 4935 | 0.111969055 | 0.182258996 | 0.225449078 | 0.12925119 | 0.312101633 | 0.220903777 | 0.153394704 | 0.147042684 | 0 | 0.085989124 | 0 | 0.203582964 | 0 | 0.272923715 | 0.125232693 | 0.225010662 | 0.30288259 | 0 |
| ENSMUSG00000029097 | 2310079F23R | 2952 | 2.706969205 | 2.697080528 | 2.261364224 | 2.53116913 | 3.032702508 | 2.433992672 | 1.169995109 | 3.079197755 | 1.757961039 | 3.338244235 | 3.058762586 | 2.836161717 | 3.041466445 | 3.25675003 | 2.763518993 | 2.334793134 | 3.609319288 | 2.459756805 |
| ENSMUSG00000029098 | Acox3 | 10373 | 4.12635807 | 5.835296327 | 4.766002894 | 4.809538566 | 4.018340489 | 5.221357158 | 5.400386759 | 7.529496088 | 5.133921226 | 5.072802934 | 4.099075778 | 4.923487059 | 6.458376907 | 8.735410633 | 5.461499986 | 4.769253327 | 4.600042384 | 5.181133449 |
| ENSMUSG00000029101 | Rgs12 | 7964 | 3.415785115 | 3.032627864 | 2.611830494 | 3.369595067 | 3.493251115 | 3.944805762 | 1.348565811 | 1.342776579 | 1.566990743 | 1.7820645 | 1.19977294 | 1.56289462 | 1.567174168 | 1.183846025 | 1.608950764 | 1.64432292 | 1.400420944 | 2.362269525 |
| ENSMUSG00000029102 | Hgfac | 2089 | 4.191510896 | 5.756799815 | 3.51975903 | 6.259463726 | 3.663463793 | 5.337178102 | 0.33972723 | 0.383935113 | 0.61119291 | 0.631986449 | 0.548874583 | 0.347344956 | 1.700286282 | 4.802260831 | 2.0314793 | 1.81463386 | 0.954029411 | 2.422612646 |
| ENSMUSG00000029103 | Lrpap1 | 4435 | 33.17991418 | 33.35045103 | 30.87834598 | 33.0998162 | 33.80630039 | 35.0276933 | 29.85979444 | 31.50980843 | 32.03911598 | 34.76500556 | 35.36531876 | 31.46316944 | 35.43889255 | 46.28700479 | 40.00313408 | 33.17080353 | 36.41646339 | 38.53738322 |
| ENSMUSG00000029104 | Htt | 15106 | 1.547586841 | 1.135716968 | 1.425011321 | 1.284854024 | 1.15980539 | 1.154677758 | 1.108744098 | 1.400674628 | 1.660438773 | 1.423323228 | 1.236595311 | 1.333870925 | 2.148841624 | 1.239041918 | 1.797419921 | 1.728729925 | 1.52229433 | 1.347369885 |
| ENSMUSG00000029106 | Add1 | 6013 | 40.63899168 | 43.97213395 | 46.15315876 | 50.82712214 | 47.10732925 | 49.76699785 | 35.90350141 | 38.46552286 | 38.03570963 | 38.90148659 | 42.04641282 | 39.66411546 | 32.13589223 | 32.06982385 | 34.5824468 | 39.7870805 | 41.09257212 | 37.23383741 |
| ENSMUSG00000029108 | Pcdh7 | 9373 | 0.562321757 | 0.693056052 | 0.541899145 | 0.938150171 | 0.390272719 | 0.660844094 | 0.272579181 | 0.619358281 | 0.307591604 | 0.226371668 | 0.336407475 | 0.452575521 | 0.678952025 | 1.010838987 | 1.094546828 | 0.302305019 | 0.425257108 | 0.340395625 |
| ENSMUSG00000029110 | Rnf4 | 2632 | 76.6126729 | 76.13110134 | 75.28038767 | 75.23111652 | 81.70723212 | 82.34941363 | 74.56420676 | 89.4154953 | 72.81187608 | 84.4305716 | 81.9180804 | 83.99917914 | 59.37823478 | 62.89009402 | 68.39270429 | 74.44264364 | 80.38037952 | 73.65201067 |
| ENSMUSG00000029111 | Whsc2 | 4916 | 7.85948027 | 7.657357703 | 10.0663388 | 9.360588848 | 9.937734443 | 9.202938518 | 8.450067555 | 10.08416158 | 11.14281244 | 8.421138621 | 9.883468831 | 8.867378454 | 10.53671952 | 9.93880796 | 7.475953591 | 10.07893531 | 7.679292606 | 8.291649325 |

| ENSMUSG00000029112 | Nkx1-1 | 1386 | 0 | 0 | 0 | 0 | 0 | 0 | 0 | 0 | 0 | 0 | 0 | 0 | 0 | 0.234566098 | 0 | 0 | 0 | 0 |
| --- | --- | --- | --- | --- | --- | --- | --- | --- | --- | --- | --- | --- | --- | --- | --- | --- | --- | --- | --- | --- |
| ENSMUSG00000029119 | Man2b2 | 3509 | 1.635280354 | 2.17402473 | 2.288402975 | 2.882459915 | 2.263256599 | 2.443038732 | 2.009002326 | 2.11152068 | 2.31226663 | 2.43211094 | 2.11032162 | 2.815438096 | 4.006724544 | 2.964797827 | 3.311153822 | 3.033567643 | 3.058239707 | 2.476899077 |
| ENSMUSG00000029120 | Ppp2r2c | 4440 | 0.019146476 | 0.075029041 | 0.087159514 | 0.123137958 | 0 | 0 | 0.021312018 | 0.154834064 | 0.009276242 | 0.223010083 | 0.139881635 | 0.251421904 | 0.055553961 | 0.543939762 | 0.111355556 | 0.034496043 | 0.233065319 | 0.086726069 |
| ENSMUSG00000029121 | Crmp1 | 3750 | 0 | 0 | 0 | 0.048598447 | 0 | 0 | 0 | 0 | 0 | 0 | 0 | 0 | 0 | 0 | 0 | 0 | 0 | 0 |
| ENSMUSG00000029122 | Evc | 7677 | 0.293444618 | 0.256019377 | 0.365463692 | 0.658756796 | 0.426334611 | 0.29046151 | 0.456055522 | 0.009949825 | 0.252151393 | 0.331657936 | 0.348495203 | 0.138139584 | 0.359852464 | 0.272239668 | 0.268344118 | 0.139655726 | 0.089862361 | 0.265121395 |
| ENSMUSG00000029123 | Stk32b | 3425 | 0.434359463 | 0.350150129 | 1.002779753 | 0.811452176 | 0.309168561 | 0.607653959 | 0.372975863 | 0.301078793 | 0.444934623 | 0.178966173 | 0.13948893 | 0.407413596 | 1.368330552 | 1.356031749 | 0.024059303 | 0.19005557 | 0.089521277 | 0 |
| ENSMUSG00000029125 | Stx18 | 3500 | 6.995137541 | 6.871974179 | 8.099111082 | 7.094505475 | 7.274796463 | 6.951540625 | 8.746086648 | 8.664219288 | 9.649412264 | 10.62911568 | 9.664191601 | 6.873300164 | 7.977675765 | 10.43001505 | 8.793589214 | 10.65572903 | 11.79354908 | 7.889877593 |
| ENSMUSG00000029126 | Nsg1 | 2135 | 0.099543738 | 0.062412917 | 0.090629565 | 0 | 0 | 0.139258268 | 0 | 0.071554852 | 0.270075513 | 0.132507831 | 0 | 0 | 0 | 0 | 0 | 0 | 0.017951427 | 0.02576539 |
| ENSMUSG00000029127 | Zbtb49 | 7736 | 1.027464829 | 1.102391536 | 1.625790834 | 1.419365523 | 1.095038594 | 1.306716346 | 0.807300106 | 1.490965037 | 0.974293224 | 1.602977631 | 1.389525033 | 1.277064669 | 1.173354806 | 1.422860485 | 1.272902257 | 0.747399395 | 1.486283489 | 1.230167459 |
| ENSMUSG00000029128 | Rab28 | 2167 | 28.75523214 | 19.96928915 | 21.54151213 | 23.37973295 | 23.2996714 | 24.81066725 | 31.63639675 | 23.52877582 | 26.38065707 | 24.15195229 | 23.87645594 | 22.84655871 | 22.94719548 | 21.15381368 | 21.6560093 | 22.19335621 | 24.5840114 | 25.68953207 |
| ENSMUSG00000029130 | Rnf32 | 2848 | 2.074515263 | 1.403633183 | 1.902329845 | 1.775728905 | 1.960429475 | 2.070496944 | 3.040105423 | 2.038358555 | 3.109234863 | 1.970131211 | 1.794915926 | 3.566871696 | 3.187174431 | 2.071067099 | 1.750487452 | 2.204936662 | 1.910931938 | 3.013139273 |
| ENSMUSG00000029131 | Dnajb6 | 5883 | 11.56736135 | 12.24247447 | 11.00183301 | 11.18309502 | 11.61776842 | 12.12075193 | 12.36901244 | 13.16576442 | 11.99960701 | 12.03012164 | 11.71838604 | 11.00563804 | 13.55935921 | 14.2971685 | 13.60778907 | 16.20663138 | 16.03280923 | 14.57746003 |
| ENSMUSG00000029134 | Plb1 | 8879 | 43.55835079 | 32.81764678 | 45.9327891 | 40.71194118 | 37.77805789 | 31.81108023 | 47.97342588 | 43.99073087 | 56.25746854 | 51.08037106 | 47.9094752 | 43.37516295 | 25.81883315 | 18.38095766 | 29.81416788 | 23.51170082 | 27.15516768 | 20.02978286 |
| ENSMUSG00000029135 | Fosl2 | 5851 | 3.319922312 | 3.837445001 | 2.637353646 | 3.114752646 | 3.825216972 | 3.015001394 | 1.455525928 | 2.650164993 | 2.154003388 | 1.925998646 | 3.05380866 | 1.650334922 | 1.534508451 | 1.68281431 | 1.175980146 | 2.624258002 | 1.984766358 | 1.964946728 |
| ENSMUSG00000029136 | Rbks | 1479 | 14.88687031 | 11.17186987 | 15.20871644 | 11.18232528 | 14.93749355 | 14.00476176 | 24.98390956 | 19.9612759 | 18.54646349 | 17.11962654 | 17.57239852 | 16.56732649 | 26.28367195 | 28.54474336 | 22.34188494 | 21.59186381 | 18.94288238 | 25.55189744 |
| ENSMUSG00000029138 | 4930548H24R | 1639 | 0 | 0 | 0 | 0 | 0 | 0 | 0 | 0 | 0.075387155 | 0.02876797 | 0 | 0.068109411 | 0 | 0 | 0 | 0.046724353 | 0 | 0 |
| ENSMUSG00000029141 | Slc4a1ap | 5432 | 4.295902364 | 5.004293388 | 3.980664225 | 4.302801131 | 4.767116151 | 5.190630598 | 4.328866242 | 5.209972419 | 4.155046141 | 5.277545578 | 4.555859443 | 4.644454987 | 4.132183792 | 4.950501951 | 5.203289275 | 5.702706441 | 4.917788833 | 3.068438759 |
| ENSMUSG00000029142 | Mrpl33 | 5572 | 18.46061127 | 15.33517115 | 17.5627516 | 15.92019981 | 18.45114662 | 15.33182209 | 20.2598801 | 17.41003268 | 19.83191346 | 18.07500039 | 18.2542869 | 15.97738367 | 21.35473519 | 22.01343051 | 20.60077795 | 18.29317963 | 18.30335177 | 20.99863065 |
| ENSMUSG00000029144 | Gtf3c2 | 6832 | 19.69099563 | 22.91236679 | 21.60948692 | 22.13365137 | 20.71955321 | 24.69658341 | 18.24086597 | 21.77391794 | 24.61424822 | 20.56631963 | 22.013403 | 18.65968579 | 16.86036693 | 18.66057083 | 19.88915857 | 21.59451263 | 20.82926528 | 20.15336565 |
| ENSMUSG00000029145 | Eif2b4 | 2823 | 26.25895395 | 21.9489845 | 20.04854074 | 22.4658072 | 22.25014537 | 22.46811866 | 25.59208669 | 25.6780658 | 22.40966639 | 19.84237884 | 27.39909946 | 22.53979998 | 22.78739643 | 28.97202901 | 25.6579296 | 24.0893088 | 20.24247562 | 29.98902418 |
| ENSMUSG00000029146 | Snx17 | 2467 | 60.59615083 | 60.00912116 | 59.00119693 | 61.57297196 | 65.43756465 | 60.05783275 | 51.51271001 | 62.97798662 | 61.77142615 | 57.24213873 | 57.18664466 | 60.52164468 | 47.95214334 | 64.85605231 | 60.10717456 | 54.41705292 | 54.26581073 | 56.43617719 |
| ENSMUSG00000029147 | Ppm1g | 2155 | 52.11075409 | 45.58687937 | 46.95936218 | 48.49978455 | 47.50654368 | 48.4490068 | 48.65192413 | 42.92436131 | 43.65197309 | 45.57536654 | 46.62215206 | 50.22114606 | 38.36672545 | 49.26737066 | 46.22986642 | 40.10297026 | 44.37313725 | 49.39332785 |
| ENSMUSG00000029148 | Nrbp1 | 3379 | 50.32944933 | 55.00226009 | 52.31041134 | 52.85566552 | 50.93807607 | 53.17496654 | 50.47712564 | 54.98846928 | 46.61060559 | 49.04845278 | 53.38805659 | 51.75213699 | 48.80633307 | 49.66039886 | 42.94521452 | 51.35632813 | 49.39657384 | 49.92984028 |
| ENSMUSG00000029149 | Krtcap3 | 1621 | 66.10459517 | 60.37833635 | 56.66954605 | 56.18539644 | 61.28580534 | 60.49652051 | 44.1896335 | 38.33376848 | 45.35344271 | 41.15869532 | 40.37735541 | 41.80148944 | 48.51022587 | 35.18404646 | 35.48264776 | 48.63686653 | 40.05228066 | 43.84439598 |
| ENSMUSG00000029151 | Slc30a3 | 2081 | 0.22467897 | 0.336170485 | 0.139471976 | 0.437876447 | 0.323809193 | 0.190495852 | 0 | 0 | 0 | 0.385181141 | 0.321407698 | 0.053643116 | 0 | 0.446363166 | 0.11879353 | 0.165600897 | 0 | 0 |
| ENSMUSG00000029152 | Ociad1 | 4123 | 86.80416739 | 73.55823162 | 81.71759372 | 72.65677092 | 77.70217898 | 72.49626217 | 83.6434918 | 80.49769025 | 93.10170432 | 84.55791896 | 82.98914689 | 82.75555309 | 94.94270279 | 87.95426494 | 97.8124737 | 104.2102609 | 98.03277398 | 85.04197096 |
| ENSMUSG00000029153 | Ociad2 | 2502 | 66.3739899 | 73.18983994 | 66.29609736 | 68.2323074 | 72.44797144 | 72.1307177 | 70.89337862 | 66.23374662 | 73.40154827 | 90.34391344 | 78.32649068 | 62.82050593 | 81.78608827 | 93.11092819 | 89.61585418 | 104.5875344 | 109.4796339 | 90.05487655 |
| ENSMUSG00000029154 | Cwh43 | 3153 | 2.318709254 | 1.690473541 | 1.503522352 | 1.488356348 | 1.5570746 | 1.933075239 | 21.66809653 | 17.33375447 | 15.20491733 | 15.43277062 | 11.56114599 | 12.26776219 | 44.38777333 | 52.08572566 | 26.99727703 | 15.22880485 | 16.43423832 | 31.54343957 |
| ENSMUSG00000029155 | Spata18 | 1983 | 0 | 0.100795444 | 0.146364691 | 0.06892745 | 0 | 0.074966314 | 0 | 0 | 0 | 0 | 0 | 0 | 0 | 0 | 0 | 0 | 0 | 0 |
| ENSMUSG00000029156 | Sgcb | 4089 | 12.78585633 | 14.82745597 | 14.76404155 | 15.48785806 | 14.06643404 | 13.18497778 | 9.66155219 | 14.48677333 | 11.21070972 | 12.16531962 | 11.98755378 | 9.159283344 | 9.639570042 | 7.814510179 | 11.33571787 | 10.5067404 | 12.48486854 | 7.318404025 |
| ENSMUSG00000029158 | Yipf7 | 1208 | 0 | 0.110307597 | 0 | 0 | 0 | 0 | 0 | 0 | 0 | 0 | 0 | 0 | 0 | 0 | 0 | 0 | 0 | 0 |
| ENSMUSG00000029161 | Cgref1 | 1322 | 0.321521755 | 0.755965829 | 0.109773518 | 0.654810773 | 0.291267314 | 1.012045239 | 0.322098419 | 0.288898657 | 0.685403439 | 0.392328091 | 0.252968767 | 1.266618667 | 0.559741875 | 0 | 0.249328629 | 0.260677357 | 0.144955737 | 0.457715714 |
| ENSMUSG00000029162 | Khk | 1836 | 8.218593404 | 7.838328052 | 6.613156928 | 7.916107376 | 6.632558109 | 6.261565463 | 12.31778896 | 11.21225757 | 8.412278546 | 11.60790734 | 10.564615 | 7.660973296 | 6.771047469 | 6.147011566 | 10.50235738 | 11.74161872 | 7.723709071 | 9.587643873 |
| ENSMUSG00000029163 | Emilin1 | 4873 | 0.052335534 | 0.102543282 | 0.009926848 | 0.028049073 | 0.039509069 | 0.142363692 | 0.077673185 | 0.227289076 | 0.177491654 | 0.203194083 | 0 | 0.125994724 | 0.202470418 | 0.142963536 | 0.04227535 | 0.220015803 | 0.117975468 | 0.011288551 |
| ENSMUSG00000029165 | Agbl5 | 5270 | 2.782596913 | 3.824345541 | 4.525265743 | 4.339970447 | 3.726342477 | 3.460228834 | 3.932249216 | 3.377164994 | 3.368384875 | 4.661388311 | 4.124782943 | 5.094370722 | 4.043906691 | 2.573372584 | 3.408705573 | 3.313191077 | 3.330824285 | 3.601165431 |
| ENSMUSG00000029166 | Mapre3 | 3566 | 7.092142383 | 7.921854823 | 8.152689777 | 6.59268056 | 8.557386317 | 8.073510434 | 6.7001971 | 7.93622271 | 6.895218707 | 7.973043765 | 8.89584196 | 6.840001276 | 8.466386252 | 8.101015804 | 9.578264115 | 8.600890782 | 8.383205716 | 8.731114528 |
| ENSMUSG00000029167 | Ppargc1a | 6928 | 9.77962624 | 10.67474382 | 9.419153067 | 13.44208641 | 11.25486668 | 13.16065672 | 14.36180193 | 13.68818348 | 11.42616676 | 14.34666317 | 15.99164675 | 10.01427087 | 11.82029196 | 11.26242304 | 11.50170455 | 12.48534673 | 13.14977017 | 14.47482701 |
| ENSMUSG00000029168 | Dpysl5 | 6793 | 0.043800417 | 0 | 0 | 0.020121174 | 0.028342072 | 0 | 0 | 0 | 0 | 0 | 0 | 0 | 0 | 0 | 0 | 0 | 0 | 0 |
| ENSMUSG00000029169 | Dhx15 | 2995 | 44.27918171 | 42.12218044 | 45.87005883 | 41.834014 | 46.29985712 | 42.32250167 | 40.48827916 | 39.2253188 | 38.55992995 | 37.9567097 | 36.94384101 | 44.37298585 | 35.21590622 | 38.45787537 | 41.91690845 | 38.62301334 | 42.88194355 | 40.27878833 |
| ENSMUSG00000029171 | Pgm1 | 2594 | 4.555296429 | 5.432287685 | 4.550170164 | 4.496386796 | 4.249117394 | 4.202621969 | 4.340947411 | 4.82926291 | 3.874136409 | 5.725702207 | 5.709420635 | 3.614892565 | 5.020673154 | 4.440298255 | 6.813981294 | 7.218237093 | 7.815964172 | 4.220050978 |
| ENSMUSG00000029173 | Sepsecs | 9354 | 1.694935927 | 2.026409751 | 1.737599551 | 2.406153079 | 1.759794515 | 2.150781466 | 2.483485712 | 1.596453853 | 2.382072378 | 2.293518279 | 2.502643752 | 2.136199188 | 1.861681291 | 3.172735925 | 2.34329995 | 2.014002442 | 2.114215224 | 1.381990601 |

| ENSMUSG00000029174 | Tbc1d1 | 6634 | 15.1529607 | 16.97280411 | 16.46479221 | 18.72851491 | 16.16489688 | 18.82316681 | 13.07267719 | 13.15490496 | 14.78219687 | 13.99453342 | 14.02138139 | 13.41124 | 13.90574093 | 14.33087834 | 13.66346442 | 14.14108956 | 16.25719015 | 12.04827134 |
| --- | --- | --- | --- | --- | --- | --- | --- | --- | --- | --- | --- | --- | --- | --- | --- | --- | --- | --- | --- | --- |
| ENSMUSG00000029175 | 4930471M23R | 2335 | 22.82719089 | 22.79828906 | 20.13664733 | 21.42442256 | 20.28343164 | 22.36770072 | 23.70699544 | 19.90584742 | 20.49624467 | 24.95857354 | 29.40154832 | 22.73263174 | 25.24695552 | 29.11954772 | 22.39176635 | 31.64919584 | 29.10172352 | 23.72341348 |
| ENSMUSG00000029176 | Anapc4 | 6138 | 21.43956093 | 19.67946472 | 22.78394856 | 22.75825382 | 21.94872179 | 22.81460168 | 16.07151117 | 14.09348183 | 14.27906574 | 16.78466709 | 15.24004052 | 14.2312662 | 20.22142455 | 16.6466263 | 21.04380543 | 17.91012282 | 19.21310127 | 17.87034194 |
| ENSMUSG00000029177 | Cenpa | 2723 | 0.187316237 | 0.746267553 | 0.284236682 | 0.200783155 | 0.406549484 | 0.200176302 | 1.198889036 | 0.420775642 | 0.877274298 | 0.467524417 | 0.263174578 | 0.245974275 | 0.724670103 | 1.228047849 | 2.027546269 | 0.984334381 | 1.632703063 | 1.393914199 |
| ENSMUSG00000029178 | Klf3 | 2874 | 25.36408382 | 29.40668498 | 28.59659989 | 25.76085492 | 24.8698353 | 25.89707927 | 21.49977685 | 25.86026969 | 25.43704436 | 26.38076925 | 22.75710444 | 26.12110862 | 20.28890961 | 24.62797118 | 22.72250038 | 22.70257307 | 22.71039792 | 19.81016891 |
| ENSMUSG00000029179 | Zcchc4 | 8851 | 1.527132073 | 1.588299781 | 1.448309292 | 1.719284692 | 1.419323475 | 1.293264203 | 1.737274958 | 2.381901043 | 1.15867613 | 1.72600021 | 2.493733004 | 1.589147777 | 1.956332953 | 1.332820946 | 1.815456649 | 1.146425371 | 2.351279993 | 1.006832594 |
| ENSMUSG00000029182 | 1700001C02R | 1159 | 0.330066078 | 0.977254878 | 0.751271395 | 1.415183431 | 0 | 0.34203785 | 0.163287934 | 0.13181157 | 0.533043776 | 0 | 0 | 0.096316933 | 0 | 0.360652965 | 0 | 0.1321505 | 0 | 0 |
| ENSMUSG00000029184 | Olfr109 | 944 | 0 | 0 | 0 | 0 | 0 | 0 | 0 | 0 | 0 | 0 | 0 | 0 | 0 | 0 | 0 | 0 | 0 | 0 |
| ENSMUSG00000029185 | Fam114a1 | 2949 | 5.015870213 | 4.654090342 | 6.085649292 | 4.279555538 | 7.344647559 | 6.334818772 | 9.786617208 | 7.913040987 | 8.016636164 | 6.33152882 | 5.977944957 | 6.832639605 | 7.895783301 | 8.142283208 | 10.33881023 | 5.284597224 | 10.76099486 | 8.505985984 |
| ENSMUSG00000029186 | Pi4k2b | 4102 | 6.870046735 | 9.761603816 | 7.700613194 | 9.985221565 | 8.413112938 | 8.915143194 | 8.558266154 | 9.478270514 | 11.41615515 | 9.345080875 | 8.804941153 | 10.57259138 | 7.456311241 | 9.782469894 | 8.316647557 | 9.325284445 | 9.147109865 | 12.739798 |
| ENSMUSG00000029188 | Slc34a2 | 2920 | 0.262018208 | 0.114085254 | 0.298192995 | 0.031206195 | 0.032967071 | 0.356372399 | 0.842554556 | 2.079654788 | 1.382287173 | 0.775080054 | 0.016361287 | 1.376276612 | 0.15205043 | 1.54283441 | 0.719616216 | 0.406509873 | 0.249383439 | 0.772388142 |
| ENSMUSG00000029189 | Sel1l3 | 4487 | 11.54753946 | 13.92058762 | 13.59461149 | 13.24082947 | 10.49097868 | 13.39591394 | 8.003192179 | 8.094712433 | 7.646170633 | 7.702577555 | 8.134626316 | 11.91696117 | 13.46815214 | 12.76254953 | 12.30445395 | 15.45448852 | 14.27306488 | 7.821664829 |
| ENSMUSG00000029190 | D5Ertd579e | 8199 | 7.797022167 | 7.581639299 | 6.867519128 | 6.77942116 | 6.281395088 | 7.228328986 | 5.99559378 | 6.116187871 | 5.636208158 | 5.043440268 | 5.850232749 | 4.316030013 | 5.559542818 | 4.843236336 | 6.110634463 | 6.678322794 | 5.749645712 | 4.830657012 |
| ENSMUSG00000029191 | Rfc1 | 7265 | 9.618514713 | 8.831470649 | 9.621438591 | 8.56660517 | 9.043369003 | 8.389519931 | 7.482762297 | 9.031596329 | 10.31221916 | 7.801121172 | 7.621634808 | 9.265476818 | 9.730576388 | 7.780103834 | 7.627817298 | 8.438164136 | 8.783659235 | 7.647515181 |
| ENSMUSG00000029192 | Tbc1d14 | 12277 | 5.80953697 | 6.360301706 | 7.005631422 | 7.177246864 | 6.045404113 | 6.897908421 | 6.343297998 | 6.669749186 | 6.756507514 | 4.96201911 | 5.350701956 | 5.446539365 | 5.794300294 | 4.149968548 | 5.282336806 | 5.093146688 | 5.644208259 | 4.023635891 |
| ENSMUSG00000029193 | Cckar | 5503 | 0 | 0 | 0 | 0 | 0 | 0 | 0 | 0 | 0 | 0 | 0 | 0 | 0 | 0 | 0 | 0 | 0 | 0 |
| ENSMUSG00000029195 | Klb | 3459 | 0 | 0 | 0 | 0 | 0.111319858 | 0 | 0 | 0 | 0 | 0 | 0 | 0 | 0 | 0.040281082 | 0 | 0 | 0 | 0 |
| ENSMUSG00000029196 | Tada2b | 4836 | 5.669114669 | 7.405161556 | 7.001958488 | 8.969006246 | 6.857505254 | 7.029192563 | 9.000757778 | 7.399975404 | 5.288839183 | 8.404447107 | 7.330235565 | 7.940689797 | 6.549026239 | 8.086418855 | 8.221567708 | 6.571795729 | 7.410067743 | 9.509432014 |
| ENSMUSG00000029198 | Grpel1 | 3524 | 44.61596088 | 41.84908703 | 41.42772832 | 38.23042228 | 43.55638609 | 41.62204637 | 40.70716303 | 40.43582672 | 43.55906477 | 45.03668194 | 44.49415827 | 40.45210052 | 47.21809222 | 44.16405699 | 43.41128746 | 46.35293166 | 44.65600882 | 47.48515966 |
| ENSMUSG00000029199 | Lias | 4074 | 17.016677 | 15.34813511 | 15.38833955 | 15.80210006 | 16.59925203 | 16.40810938 | 18.48841062 | 15.11196678 | 18.86451606 | 18.27465892 | 16.0422541 | 16.13913857 | 17.10996542 | 20.40621414 | 15.62503776 | 16.25048115 | 19.53944984 | 14.77171398 |
| ENSMUSG00000029201 | Ugdh | 5815 | 12.53592036 | 12.56322907 | 14.00877474 | 12.95140263 | 11.81156579 | 13.42141967 | 11.17929851 | 11.25083153 | 12.6853052 | 12.92488753 | 10.58196845 | 14.3210608 | 15.99151575 | 17.52335817 | 12.45611956 | 16.04715567 | 14.84938038 | 19.42109993 |
| ENSMUSG00000029202 | Pds5a | 4128 | 10.48213886 | 12.04041622 | 12.35118726 | 11.34611279 | 10.13242288 | 11.35583478 | 10.33818711 | 12.39772752 | 8.899799433 | 8.749379529 | 10.91370781 | 12.79108934 | 8.042727792 | 9.68710253 | 10.39021795 | 12.05857307 | 9.507298457 | 10.78061224 |
| ENSMUSG00000029203 | Ube2k | 5435 | 15.43012186 | 15.83818191 | 15.16623656 | 14.21739304 | 14.88680105 | 14.6697973 | 14.82492957 | 15.61426277 | 18.36153223 | 16.3010435 | 16.27952587 | 14.59320267 | 17.11867451 | 17.61200812 | 15.8817405 | 18.95156098 | 17.42489046 | 15.3843316 |
| ENSMUSG00000029204 | Rhoh | 4957 | 0.05144867 | 0.127687107 | 0.019517261 | 0.073530029 | 0.145648347 | 0.159944268 | 0.572677171 | 0.161799566 | 0.041543792 | 0.019023887 | 0.607186279 | 0.101339714 | 0.567262313 | 0.477839108 | 0 | 0.030898211 | 0.007731752 | 0 |
| ENSMUSG00000029205 | Chrna9 | 3114 | 0 | 0 | 0 | 0 | 0.015456623 | 0 | 0 | 0 | 0.079357448 | 0 | 0 | 0 | 0 | 0.089487644 | 0 | 0 | 0 | 0 |
| ENSMUSG00000029206 | Nsun7 | 5653 | 0.007519048 | 0.053036626 | 0.008557143 | 0.008059622 | 0.18731688 | 0.09642315 | 0 | 0.02026839 | 0.189430287 | 0.200179883 | 0.177476407 | 0 | 0.200713621 | 0.139669111 | 0.218653224 | 0 | 0 | 0.126502457 |
| ENSMUSG00000029207 | Apbb2 | 11371 | 4.911775684 | 4.743081149 | 4.203064637 | 4.591764736 | 4.292126515 | 4.458046474 | 3.112293012 | 4.561189206 | 3.104110809 | 4.416102275 | 3.693095462 | 4.241028271 | 4.429503785 | 4.043588648 | 4.438651479 | 4.1418914 | 5.504075529 | 4.726400252 |
| ENSMUSG00000029208 | Guf1 | 7620 | 10.64860644 | 7.886674694 | 9.192240414 | 10.00907982 | 8.224116091 | 7.816586058 | 11.0458341 | 10.2347619 | 10.37770476 | 9.250695718 | 10.84654569 | 9.983825211 | 7.594007738 | 9.538713486 | 10.21928355 | 7.150595057 | 11.58842363 | 10.59755494 |
| ENSMUSG00000029209 | Gnpda2 | 2979 | 16.7794854 | 16.65085582 | 16.1407483 | 18.29171166 | 16.91645656 | 19.71130893 | 15.54854403 | 16.32055329 | 15.99623993 | 14.4032024 | 17.11174243 | 16.30064668 | 15.66565752 | 14.62388519 | 16.49998529 | 20.88695436 | 18.69355804 | 14.23699942 |
| ENSMUSG00000029211 | Gabra4 | 4117 | 9.601606438 | 7.85689101 | 7.402313365 | 8.676186242 | 7.786218402 | 8.497505442 | 1.022790484 | 0.157704844 | 0.840336975 | 0.469560076 | 0 | 0.48806506 | 0.215684846 | 0 | 0.040030659 | 0.018601218 | 0.027927834 | 0 |
| ENSMUSG00000029212 | Gabrb1 | 3080 | 17.07107232 | 15.69383426 | 15.34446077 | 17.6770935 | 18.2213711 | 16.49076361 | 2.365633946 | 1.401214764 | 2.019209052 | 1.240002261 | 1.364998814 | 1.141684007 | 1.393466494 | 0.814279454 | 1.64538681 | 0.348096431 | 0.435526101 | 0.642963585 |
| ENSMUSG00000029213 | Commd8 | 3476 | 18.31782327 | 19.20570771 | 18.1470321 | 18.21917481 | 19.5380162 | 18.16173262 | 19.0557395 | 19.0962012 | 17.9983652 | 17.78324855 | 21.16612084 | 16.04138289 | 19.98254877 | 22.62078249 | 17.55451792 | 17.87849707 | 22.59222737 | 19.62350182 |
| ENSMUSG00000029217 | Tec | 4334 | 2.432229731 | 3.189859506 | 2.578284613 | 2.954004028 | 2.720886317 | 3.818784729 | 2.423492091 | 3.225292866 | 2.185717265 | 2.893882572 | 2.965266228 | 1.674212306 | 2.390332863 | 2.614757112 | 2.661844866 | 2.995042896 | 2.988991314 | 3.376193444 |
| ENSMUSG00000029219 | Slc10a4 | 3810 | 0 | 0 | 0 | 0 | 0 | 0.013005967 | 0 | 0 | 0 | 0.074253076 | 0 | 0 | 0 | 0.036570148 | 0.108140567 | 0.010050028 | 0.010059396 | 0 |
| ENSMUSG00000029221 | Slc30a9 | 10065 | 14.39643766 | 14.44055216 | 13.32735218 | 13.34918229 | 14.55673876 | 14.97167042 | 16.11874576 | 15.77784493 | 15.15287311 | 16.45238647 | 15.11805691 | 16.29828439 | 15.65975914 | 14.46619115 | 15.26893229 | 13.76027094 | 14.14626855 | 14.94236441 |
| ENSMUSG00000029223 | Uchl1 | 1686 | 14.01712804 | 18.6520594 | 11.16091537 | 14.32227372 | 16.7005785 | 12.63800441 | 4.770554815 | 4.825018809 | 3.908566146 | 6.488115751 | 7.225749948 | 4.866490154 | 4.623038511 | 6.583711083 | 4.374305163 | 10.67413136 | 6.160395291 | 4.078373867 |
| ENSMUSG00000029227 | Fip1l1 | 9663 | 10.63179245 | 11.76621215 | 10.60783513 | 10.61817985 | 11.6108366 | 10.98436876 | 11.15370823 | 11.15377829 | 10.8560546 | 11.36925785 | 9.388869518 | 9.946659523 | 9.735689356 | 9.372448557 | 10.07973488 | 10.599956 | 11.48638682 | 10.80485197 |
| ENSMUSG00000029228 | Lnx1 | 4499 | 18.75367291 | 24.70885264 | 22.12774516 | 24.91224029 | 23.45080611 | 23.35003227 | 11.48376203 | 11.74889641 | 10.09751653 | 12.85928271 | 10.6083982 | 10.25995843 | 12.56598737 | 11.8510363 | 13.41637683 | 14.6898396 | 14.286108 | 13.64529075 |
| ENSMUSG00000029229 | Chic2 | 6706 | 9.292065025 | 9.160300766 | 8.749939207 | 9.586435067 | 9.833095053 | 9.80561847 | 7.612642494 | 9.442738322 | 8.764264538 | 10.20215783 | 8.207090988 | 7.690675847 | 8.673177821 | 9.446724605 | 8.988648415 | 10.94017354 | 11.62476706 | 10.58182936 |
| ENSMUSG00000029231 | Pdgfra | 6713 | 0.164626036 | 0.416845392 | 0.20176655 | 0.176461664 | 0.050189701 | 0.051671255 | 0.035239594 | 0 | 0.006135337 | 0.02107137 | 0 | 0 | 0.007348714 | 0 | 0.006137577 | 0.005703949 | 0.205533545 | 0 |
| ENSMUSG00000029233 | Srd5a3 | 3193 | 19.91473327 | 18.27879445 | 18.21014203 | 15.79582715 | 21.39029116 | 18.716128 | 17.82560374 | 16.47069781 | 15.45300529 | 17.18866852 | 20.09450963 | 18.87908412 | 14.9556204 | 13.74558803 | 15.89737452 | 14.10264776 | 14.89600202 | 13.69628558 |

| ENSMUSG00000029234 | Tmem165 | 2982 | 38.68512667 | 36.15041103 | 36.54780815 | 38.10504513 | 32.12023075 | 39.88147572 | 38.93538367 | 38.2180177 | 33.60388761 | 38.15380515 | 32.01018345 | 39.84961289 | 37.32153098 | 30.49548059 | 33.29837347 | 37.78982481 | 33.69937977 | 31.24930476 |
| --- | --- | --- | --- | --- | --- | --- | --- | --- | --- | --- | --- | --- | --- | --- | --- | --- | --- | --- | --- | --- |
| ENSMUSG00000029235 | Pdcl2 | 1658 | 0 | 0 | 0 | 0 | 0 | 0.029887053 | 0 | 0 | 0 | 0 | 0.288148121 | 0 | 0 | 0 | 0 | 0 | 0.046231962 | 0 |
| ENSMUSG00000029236 | Nmu | 948 | 0 | 0 | 0 | 0 | 0 | 0 | 0 | 0 | 0 | 0 | 0 | 0 | 0 | 0 | 0 | 0 | 0.12128575 | 0 |
| ENSMUSG00000029238 | Clock | 13508 | 2.599146831 | 3.181346872 | 2.506771635 | 2.600500828 | 2.640343162 | 2.87235641 | 2.861597474 | 3.220398752 | 3.0703895 | 2.405006999 | 2.588930234 | 2.917223703 | 2.392094005 | 2.599328551 | 2.305920658 | 3.084111701 | 2.902561573 | 2.284580165 |
| ENSMUSG00000029245 | Epha5 | 8633 | 0 | 0 | 0 | 0 | 0 | 0 | 0 | 0 | 0 | 0 | 0 | 0 | 0 | 0 | 0 | 0 | 0 | 0 |
| ENSMUSG00000029246 | Ppat | 6539 | 7.689803218 | 8.568938381 | 8.832848332 | 7.796728645 | 7.831834651 | 8.805670044 | 9.565279331 | 6.448143792 | 8.578687027 | 8.292293737 | 6.940848838 | 8.458987861 | 6.540873571 | 4.60960586 | 5.651903293 | 8.584497699 | 6.253885731 | 11.10445341 |
| ENSMUSG00000029247 | Paics | 3350 | 72.95664542 | 68.89305407 | 72.51697595 | 84.7843434 | 70.30134403 | 69.2259084 | 66.32248963 | 67.02481604 | 68.03766514 | 76.55303732 | 73.73030906 | 71.57733925 | 61.18630337 | 47.88593368 | 55.78813657 | 66.40848627 | 65.53224736 | 56.71685213 |
| ENSMUSG00000029248 | 1700023E05R | 2233 | 0 | 0 | 0.043326046 | 0 | 0 | 0 | 0 | 0 | 0 | 0 | 0 | 0 | 0 | 0 | 0 | 0 | 0 | 0 |
| ENSMUSG00000029249 | Rest | 7542 | 12.61854801 | 12.12020442 | 12.12224508 | 12.02154312 | 10.33859936 | 11.57675902 | 9.9932508 | 11.62179309 | 10.70347002 | 10.92180835 | 9.609468578 | 10.94555356 | 11.62982616 | 11.19535281 | 10.64182324 | 13.78400943 | 10.73766446 | 11.31982679 |
| ENSMUSG00000029250 | Polr2b | 4782 | 16.586085 | 20.01420851 | 20.32254757 | 18.36923745 | 19.73791349 | 20.19621032 | 14.52426028 | 14.0246463 | 14.49537976 | 16.6733248 | 16.94402542 | 18.4418124 | 13.40070275 | 16.71482108 | 17.17162294 | 13.39610752 | 16.06145943 | 13.91907552 |
| ENSMUSG00000029253 | Cenpc1 | 3154 | 8.166815686 | 10.57267188 | 10.73604035 | 8.580615198 | 10.77398165 | 9.725156019 | 11.56565487 | 11.74591957 | 11.87017845 | 10.10585777 | 9.724642785 | 13.60882832 | 11.16772002 | 8.143177022 | 11.10378012 | 7.85479486 | 11.44685215 | 11.45877715 |
| ENSMUSG00000029254 | Stap1 | 4281 | 824.4475115 | 759.2320633 | 850.6328814 | 801.8573521 | 815.4768875 | 830.6593833 | 886.6071109 | 833.3544735 | 833.1201866 | 848.2721008 | 809.6968261 | 799.3074073 | 519.4313252 | 463.3885318 | 575.7438628 | 417.708399 | 590.4362549 | 568.62018 |
| ENSMUSG00000029255 | Gnrhr | 3620 | 0.140901136 | 0.138036854 | 0.200442805 | 0.289476249 | 0.079776669 | 0.054754402 | 0.15683761 | 0.084403099 | 0.193417339 | 0.078150337 | 0.079185014 | 0.092512148 | 0.122648413 | 0.089809009 | 0.40973923 | 0.095197643 | 0.127048498 | 0.106371201 |
| ENSMUSG00000029260 | Ugt2b34 | 6021 | 0 | 0 | 0 | 0 | 0 | 0 | 0 | 0 | 0 | 0 | 0 | 0 | 0 | 0 | 0 | 0 | 0 | 0 |
| ENSMUSG00000029263 | Pigg | 5002 | 3.747457543 | 5.228033179 | 5.309289917 | 5.337619348 | 4.994096038 | 5.587313422 | 3.234893281 | 4.413276407 | 5.088617901 | 4.854580603 | 6.131851928 | 5.813666575 | 3.215154944 | 3.556194616 | 3.006510976 | 3.460086874 | 4.988088621 | 4.157025658 |
| ENSMUSG00000029265 | Dr1 | 3474 | 14.43756699 | 15.03587166 | 14.14723858 | 14.63619041 | 13.23142979 | 13.96434259 | 15.51213048 | 17.87589128 | 18.26955118 | 15.03827841 | 17.89154317 | 15.58468414 | 11.44545922 | 15.16050521 | 13.16457313 | 15.19940921 | 17.06700671 | 13.57017975 |
| ENSMUSG00000029267 | Mtf2 | 5494 | 8.873941919 | 8.373699839 | 7.739412665 | 6.675762776 | 7.017413698 | 6.746531619 | 7.983045753 | 7.653774126 | 8.186326031 | 8.839684084 | 8.513229769 | 8.513564843 | 8.557210987 | 10.50782683 | 10.91908725 | 9.450685045 | 12.16619254 | 9.902440216 |
| ENSMUSG00000029268 | Ugt2a1 | 3643 | 0 | 0 | 0 | 0 | 0 | 0 | 0 | 0 | 0 | 0 | 0 | 0 | 0 | 0 | 0 | 0 | 0 | 0 |
| ENSMUSG00000029269 | Sult1b1 | 4466 | 0.076140038 | 0 | 0 | 0 | 0 | 0 | 0 | 0.051310885 | 0 | 0 | 0 | 0.074987455 | 0 | 0 | 0 | 0 | 0.042908975 | 0 |
| ENSMUSG00000029270 | Fam69a | 3399 | 1.000416029 | 0.842868168 | 0.654657957 | 0.549574233 | 1.175330881 | 0.845560032 | 2.714319621 | 3.51698204 | 3.768463196 | 2.427588429 | 2.628395774 | 1.855595726 | 1.8867753 | 2.814793175 | 1.333383686 | 0.856159506 | 1.296712016 | 1.14905754 |
| ENSMUSG00000029272 | Sult1e1 | 1252 | 0.441347674 | 0 | 0.15454802 | 0.03639061 | 0 | 0 | 6.688773299 | 4.24021081 | 3.454140699 | 2.749202346 | 1.030290638 | 2.496547208 | 2.994589228 | 5.230524219 | 1.908698276 | 0.244668418 | 0.887749689 | 3.514958896 |
| ENSMUSG00000029273 | Sult1d1 | 2600 | 0 | 0 | 0 | 0 | 0 | 0 | 0 | 0.014689386 | 0.364342255 | 0.072539543 | 0.202124824 | 0.064402688 | 0 | 0 | 0.316935045 | 0 | 0 | 0 |
| ENSMUSG00000029275 | Gfi1 | 4115 | 0 | 0 | 0 | 0 | 0 | 0.048167906 | 0 | 0 | 0 | 0 | 0 | 0 | 0.023976631 | 0 | 0 | 0 | 0 | 0 |
| ENSMUSG00000029276 | Glmn | 2850 | 4.146118925 | 4.523540373 | 5.60114562 | 4.412227451 | 4.830080761 | 4.346731016 | 3.270385175 | 3.886244455 | 4.176457212 | 4.996320135 | 5.297153291 | 5.150708512 | 4.500455611 | 3.585157623 | 4.35146257 | 3.331954609 | 5.917042329 | 3.918192514 |
| ENSMUSG00000029279 | Brdt | 5228 | 0.081302938 | 0.114696269 | 0.074022235 | 0.043574067 | 0.101272219 | 0.018956669 | 0 | 0.021916069 | 0.047268381 | 0.063132158 | 0.091382859 | 0 | 0.264210728 | 0.248744156 | 0 | 0 | 0.139288378 | 0.178874295 |
| ENSMUSG00000029280 | Smr3a | 544 | 0 | 0 | 0 | 0 | 0 | 0 | 0 | 0 | 0 | 0 | 0 | 0 | 0 | 0 | 0 | 0 | 0 | 0 |
| ENSMUSG00000029281 | Smr2 | 1382 | 0 | 0 | 0 | 0 | 0 | 0 | 0 | 0 | 0 | 0 | 0 | 0 | 0 | 0 | 0 | 0 | 0 | 0 |
| ENSMUSG00000029282 | Amtn | 1013 | 0 | 0 | 0 | 0 | 0 | 0 | 0 | 0 | 0 | 0 | 0 | 0 | 0 | 0 | 0 | 0 | 0 | 0 |
| ENSMUSG00000029283 | Cdc7 | 3994 | 0.415048038 | 0.241881305 | 1.162708792 | 0 | 0.397684898 | 0.198508697 | 0.130305325 | 0.554621768 | 0.464044369 | 0.306939981 | 0.203348596 | 0.041924634 | 0.271733145 | 0.755850195 | 0.649899354 | 0.738201495 | 0.374242759 | 0.358096338 |
| ENSMUSG00000029286 | Enam | 5473 | 0 | 0 | 0 | 0 | 0 | 0 | 0 | 0 | 0 | 0 | 0 | 0 | 0 | 0 | 0 | 0 | 0 | 0 |
| ENSMUSG00000029287 | Tgfbr3 | 7003 | 8.618784801 | 10.48430942 | 11.44580034 | 9.186376496 | 10.75631309 | 12.84281186 | 8.850436869 | 7.602485928 | 6.051824178 | 9.109653221 | 7.422412512 | 7.356540992 | 8.882985533 | 6.751403822 | 6.03050047 | 7.660308578 | 10.97304374 | 9.072614346 |
| ENSMUSG00000029288 | Ambn | 1839 | 0 | 0 | 0 | 0 | 0 | 0 | 0 | 0 | 0 | 0 | 0 | 0 | 0 | 0 | 0 | 0 | 0 | 0 |
| ENSMUSG00000029290 | Zfp326 | 7362 | 5.779364467 | 5.452599502 | 6.005624388 | 4.876677932 | 6.152151613 | 4.832771354 | 6.195248912 | 6.598850293 | 6.41126692 | 7.365295945 | 7.248659144 | 7.64224231 | 8.007557884 | 6.75653594 | 6.928487665 | 8.394599341 | 8.001564567 | 7.718610058 |
| ENSMUSG00000029291 | Rufy3 | 6721 | 3.895728081 | 4.688885275 | 4.707080621 | 4.175807664 | 4.039042546 | 4.777588359 | 3.611278722 | 5.131340481 | 5.067884025 | 4.153134404 | 3.817163027 | 4.318426506 | 4.844378129 | 3.496608871 | 3.500385123 | 5.799708129 | 6.118749677 | 4.264208392 |
| ENSMUSG00000029298 | Gbp9 | 3622 | 36.67274298 | 43.50358632 | 39.30516285 | 52.66816472 | 35.78665666 | 56.73528055 | 27.94086699 | 28.87100988 | 31.40727679 | 32.6878564 | 26.86846781 | 40.46712588 | 24.52975787 | 22.63219609 | 19.64524766 | 30.43585276 | 19.31128985 | 35.69061314 |
| ENSMUSG00000029299 | Abcg3 | 3958 | 0.064434324 | 0.058916185 | 0 | 0 | 0.170249351 | 0 | 0.023907367 | 0 | 0 | 0 | 0 | 0 | 0 | 0 | 0 | 0 | 0 | 0 |
| ENSMUSG00000029304 | Spp1 | 1715 | 0 | 0 | 0 | 0 | 0 | 0 | 0 | 2.783702799 | 0 | 0 | 0 | 0 | 0 | 0 | 0 | 0 | 0 | 0 |
| ENSMUSG00000029306 | Ibsp | 2061 | 0 | 0 | 0 | 0 | 0 | 0 | 0 | 0 | 0 | 0 | 0 | 0 | 0 | 0 | 0 | 0 | 0 | 0 |
| ENSMUSG00000029307 | Dmp1 | 2758 | 0 | 0 | 0 | 0 | 0 | 0 | 0 | 0 | 0 | 0 | 0 | 0 | 0 | 0 | 0 | 0 | 0 | 0 |
| ENSMUSG00000029309 | Sparcl1 | 3103 | 0 | 0.182506994 | 0.140303504 | 0 | 0 | 0 | 0 | 0 | 0.384920708 | 0.075975996 | 0 | 0 | 0 | 0 | 0 | 0 | 0.061756843 | 0.336826628 |
| ENSMUSG00000029310 | Nudt9 | 2380 | 27.27117806 | 24.6347453 | 27.41844221 | 27.14519364 | 24.75356074 | 28.71143681 | 36.3393181 | 29.07757696 | 32.36083381 | 34.27341035 | 29.58835664 | 29.43220863 | 35.31999455 | 37.46749907 | 31.57631846 | 26.46556688 | 28.55148084 | 26.78804819 |

| ENSMUSG00000029311 | Hsd17b11 | 3258 | 3.561667605 | 2.52556319 | 3.177389655 | 2.335388092 | 3.324027878 | 3.239635437 | 2.933444182 | 3.540241107 | 4.007405002 | 3.256264037 | 2.668828251 | 4.437156725 | 3.316049688 | 3.820446722 | 3.376554765 | 5.817633721 | 3.752636192 | 3.798971459 |
| --- | --- | --- | --- | --- | --- | --- | --- | --- | --- | --- | --- | --- | --- | --- | --- | --- | --- | --- | --- | --- |
| ENSMUSG00000029312 | Klhl8 | 4622 | 4.80965103 | 4.533494259 | 5.201567414 | 4.632992392 | 4.759041349 | 6.153887727 | 5.456005597 | 4.817431978 | 3.974293819 | 4.427391868 | 4.909802094 | 4.190401324 | 5.165869312 | 3.456678075 | 4.064887383 | 3.951670214 | 2.869082373 | 3.582375838 |
| ENSMUSG00000029313 | Aff1 | 9428 | 12.10054014 | 12.07362744 | 11.72396233 | 13.03331953 | 11.31825146 | 11.45263115 | 12.38520276 | 11.43989652 | 10.93006602 | 11.13252709 | 8.923600115 | 12.05945107 | 10.4283529 | 9.005069129 | 10.71555102 | 10.70983578 | 11.27267939 | 10.35650874 |
| ENSMUSG00000029314 | Agpat9 | 3390 | 6.056047204 | 6.416908532 | 4.951508859 | 7.687586242 | 4.997769065 | 7.981059744 | 3.265830935 | 4.79939924 | 3.523330257 | 3.43546422 | 1.550219892 | 4.676002411 | 2.67760259 | 4.096395908 | 3.074924378 | 6.506014709 | 3.911769536 | 2.498938771 |
| ENSMUSG00000029319 | Coq2 | 2832 | 33.55987768 | 28.43123776 | 35.08447435 | 29.8592155 | 36.06495128 | 25.80871541 | 25.67782045 | 29.08934039 | 32.0969775 | 26.58886762 | 31.61379668 | 27.06034772 | 27.59242831 | 23.84523413 | 27.3804125 | 23.64769504 | 25.65913098 | 25.32905197 |
| ENSMUSG00000029320 | 1700016H13R | 2271 | 0 | 0.220032326 | 0.042601084 | 0.12037264 | 0 | 0 | 0 | 0.235444136 | 0 | 0.020762089 | 0.084147879 | 0 | 0 | 0.265862235 | 0 | 0 | 0 | 0.290668992 |
| ENSMUSG00000029321 | Slc10a6 | 2118 | 0 | 0 | 0 | 0 | 0 | 0.023396003 | 0.201045378 | 0.144258366 | 0.038891894 | 0.0222619 | 0 | 0 | 0.419251422 | 0.372780683 | 0.019453048 | 0 | 0.036191026 | 0 |
| ENSMUSG00000029322 | Plac8 | 1849 | 47.30970918 | 41.97893106 | 46.88229659 | 37.50346646 | 40.42665087 | 53.51909624 | 61.51415908 | 65.0035102 | 73.81942306 | 60.13053449 | 61.41756425 | 74.53156892 | 47.73109789 | 30.59432041 | 36.29926152 | 39.90589533 | 35.13416617 | 38.46769875 |
| ENSMUSG00000029326 | Enoph1 | 1820 | 21.64961438 | 17.93771227 | 22.80466431 | 24.30756816 | 20.99821285 | 22.13536945 | 17.13134913 | 20.67006394 | 22.10946478 | 24.92251458 | 26.19747723 | 20.30218057 | 19.57013422 | 19.5728654 | 21.09881873 | 23.56345071 | 20.51088636 | 21.67117007 |
| ENSMUSG00000029328 | Hnrpdl | 5694 | 24.75362903 | 24.30893493 | 26.20020506 | 22.4844634 | 23.14457446 | 22.91400553 | 28.7498892 | 30.81417222 | 27.42151058 | 26.20035566 | 32.91555358 | 32.60324794 | 23.35771847 | 25.65273179 | 24.74698299 | 27.02668619 | 24.93161288 | 25.93948921 |
| ENSMUSG00000029330 | Cds1 | 3926 | 51.95678547 | 58.81091947 | 57.18327926 | 55.85463739 | 52.60674591 | 56.49466009 | 49.62649308 | 49.35049857 | 56.15675468 | 57.77942776 | 57.20582777 | 51.83492251 | 49.88479663 | 42.3982703 | 55.82044722 | 49.34085146 | 51.75904895 | 47.65307488 |
| ENSMUSG00000029331 | 4930522N08R | 914 | 0 | 0 | 0 | 0 | 0 | 0 | 0 | 0 | 0 | 0 | 0 | 0 | 0 | 0 | 0 | 0 | 0 | 0 |
| ENSMUSG00000029333 | Rasgef1b | 3271 | 10.20072246 | 15.44960579 | 9.775268592 | 10.43265735 | 17.77540929 | 12.01324296 | 10.84821437 | 24.44967613 | 18.79898136 | 14.45801142 | 23.44200806 | 17.18323822 | 13.422625 | 13.31842066 | 15.75761126 | 21.89038087 | 24.61741051 | 18.66710745 |
| ENSMUSG00000029334 | Prkg2 | 6182 | 0 | 0 | 0 | 0 | 0 | 0 | 0 | 0.018534003 | 0 | 0 | 0 | 0 | 0 | 0 | 0 | 0 | 0 | 0 |
| ENSMUSG00000029335 | Bmp3 | 3109 | 0.123044897 | 0.750049082 | 0.435657398 | 0.52756436 | 0.805036999 | 0.892554867 | 1.537015301 | 2.13749695 | 2.490532314 | 2.199051776 | 1.736433036 | 3.46491569 | 0.84097511 | 2.210911847 | 0.781888645 | 0.923703942 | 1.134132941 | 2.087833578 |
| ENSMUSG00000029337 | Fgf5 | 2498 | 0.051047049 | 0 | 0 | 0 | 0 | 0 | 0 | 0 | 0 | 0 | 0 | 0 | 0 | 0 | 0 | 0 | 0 | 0 |
| ENSMUSG00000029338 | Antxr2 | 3734 | 2.788904158 | 3.666737955 | 3.420088916 | 2.391527769 | 2.61670608 | 2.428535149 | 3.598500486 | 3.743550959 | 1.599369252 | 3.169476839 | 3.659249632 | 2.182401376 | 5.099657222 | 8.196747078 | 7.55840005 | 7.926791516 | 8.416594392 | 7.896325978 |
| ENSMUSG00000029343 | Crybb1 | 959 | 0.487546336 | 0.208422696 | 0.60529965 | 0.617615825 | 0.250948507 | 1.240110121 | 0 | 0.438077608 | 0.042947357 | 0.147499593 | 0 | 0.058201942 | 5.555627806 | 8.136190492 | 5.327417029 | 1.117973939 | 1.838383376 | 3.900541457 |
| ENSMUSG00000029344 | Tpst2 | 2370 | 53.60673888 | 58.00941538 | 43.3320696 | 52.38558895 | 45.79640838 | 60.29961335 | 31.44197017 | 35.59789743 | 32.53213397 | 40.46604659 | 37.23221448 | 33.25388843 | 37.63380018 | 39.97718915 | 42.33155637 | 42.10351177 | 35.10818168 | 41.96475315 |
| ENSMUSG00000029345 | Tfip11 | 4072 | 7.849678873 | 9.948054855 | 7.852382997 | 8.16786895 | 9.822600336 | 10.23424581 | 9.271983739 | 8.431967034 | 7.333060886 | 8.614961499 | 7.414973909 | 5.935211665 | 7.911036832 | 7.151385755 | 8.600521244 | 8.472455125 | 9.628635003 | 7.700194212 |
| ENSMUSG00000029346 | Srrd | 1361 | 14.49111071 | 11.92019066 | 10.80496196 | 13.0556997 | 13.50947453 | 12.19703582 | 11.85424211 | 11.95441839 | 16.58354933 | 12.50654216 | 12.88274046 | 12.67232898 | 11.99769627 | 9.145492594 | 12.59356888 | 13.44813397 | 13.06642304 | 12.97422723 |
| ENSMUSG00000029348 | Asphd2 | 1581 | 0 | 0.105353872 | 0.061193587 | 0.518721567 | 0 | 0 | 0 | 0 | 0 | 0 | 0.271963711 | 0.176520122 | 0 | 0.235011195 | 0 | 0.775015457 | 0.290901684 | 0 |
| ENSMUSG00000029352 | Crybb3 | 2531 | 1.629001215 | 1.474138346 | 1.204082344 | 0.918061344 | 0.950847959 | 1.018072756 | 0.934663748 | 0.663953262 | 0.781095518 | 0.4284734 | 0.207635142 | 0.749795546 | 0.87709849 | 0.899154596 | 0.293017782 | 0.711046443 | 0.196855733 | 0.934567993 |
| ENSMUSG00000029359 | Tesc | 1803 | 41.49146412 | 34.08890174 | 37.40027804 | 32.59775219 | 36.01218249 | 39.98847884 | 41.11978252 | 30.96910278 | 37.50874037 | 35.90566587 | 29.57118894 | 37.42714147 | 33.05211096 | 26.32604399 | 32.70073571 | 39.37370279 | 27.52776178 | 28.98427698 |
| ENSMUSG00000029360 | Gm9754 | 3001 | 0.070818354 | 0.144307772 | 0.080595685 | 0.18218345 | 0 | 0.049536222 | 0.157656378 | 0.025453117 | 0.17841543 | 0.172828303 | 0.031839359 | 0.297584339 | 0 | 0.046428611 | 0 | 0 | 0 | 0 |
| ENSMUSG00000029361 | Nos1 | 10917 | 0.011680455 | 0.119007317 | 0 | 0.020867017 | 0.004408897 | 0 | 0.056340096 | 0.048978074 | 0.120726253 | 0.021595083 | 0.030633389 | 0 | 0.117489223 | 0.233985967 | 0.203799947 | 0.014029718 | 0.021064192 | 0.105815814 |
| ENSMUSG00000029363 | Rfc5 | 5497 | 3.324945551 | 3.763381355 | 4.303193805 | 4.392823995 | 4.614430375 | 3.813135584 | 6.050720991 | 5.426280931 | 6.848185448 | 6.201556932 | 5.744814909 | 4.873843559 | 5.025627374 | 5.973434561 | 6.625827798 | 4.012259389 | 7.265053915 | 5.073606896 |
| ENSMUSG00000029364 | Wsb2 | 6015 | 16.21061909 | 19.86035556 | 17.9179095 | 19.38332709 | 16.32404394 | 19.8622844 | 19.86109963 | 20.52801945 | 16.89229167 | 18.23317303 | 19.44357412 | 18.62377969 | 12.62208158 | 15.02580117 | 18.19307273 | 16.18836486 | 16.00592815 | 17.53158065 |
| ENSMUSG00000029366 | Dck | 3071 | 1.48096836 | 2.104429006 | 2.299751036 | 1.053348794 | 2.507687329 | 1.791062659 | 1.817940773 | 1.840597706 | 2.856635577 | 1.827070559 | 2.271294021 | 0.545252321 | 4.88339396 | 4.022835313 | 4.33347527 | 4.937505871 | 4.742426839 | 3.47501358 |
| ENSMUSG00000029368 | Alb | 2028 | 0 | 0 | 0 | 0 | 0 | 0 | 0 | 0 | 0 | 0 | 0 | 0 | 0.170277821 | 0 | 0 | 0 | 0 | 0 |
| ENSMUSG00000029369 | Afm | 2971 | 0.04292007 | 0.011212687 | 0 | 0.015335256 | 0.097203481 | 0 | 0.031849666 | 0.077130399 | 0.166354153 | 0 | 0 | 0.037573654 | 0 | 0.015632476 | 0.069339542 | 0 | 0.051600534 | 0 |
| ENSMUSG00000029370 | Rassf6 | 1947 | 6.811307098 | 9.752619263 | 6.55912276 | 7.58180707 | 7.243273567 | 8.933235483 | 3.790846386 | 5.531719301 | 4.992304941 | 5.957407808 | 10.25677075 | 6.8228699 | 11.32581768 | 3.435001841 | 5.523166968 | 6.568599318 | 6.417243344 | 10.31244168 |
| ENSMUSG00000029371 | Cxcl5 | 1824 | 0 | 0 | 0 | 0 | 0 | 0 | 0.59659628 | 2.428902785 | 0 | 0 | 0 | 0 | 0.216368058 | 1.324063895 | 0.655068597 | 0.314889863 | 0.25214669 | 0 |
| ENSMUSG00000029372 | Ppbp | 1077 | 0 | 0 | 0 | 0 | 0 | 0 | 0.175720256 | 0 | 0 | 0 | 0 | 0 | 0 | 0 | 0 | 0 | 0 | 0 |
| ENSMUSG00000029373 | Pf4 | 583 | 0.29163071 | 0.05714047 | 0 | 0 | 1.15582664 | 0.33998445 | 2.191153227 | 0.589591118 | 4.168103653 | 0 | 0.327787022 | 0 | 0 | 0.557647705 | 0 | 0 | 0.065739789 | 0 |
| ENSMUSG00000029375 | Cxcl15 | 2123 | 0 | 0 | 0 | 0 | 0 | 0.023340901 | 0 | 0 | 0 | 0 | 0 | 0 | 0 | 0 | 0 | 0 | 0 | 0 |
| ENSMUSG00000029376 | Mthfd2l | 2227 | 6.126700272 | 8.676011965 | 6.407809365 | 6.096628295 | 6.332579091 | 6.564012755 | 5.523707464 | 5.522206365 | 6.066087638 | 4.954317271 | 5.255889011 | 5.363516746 | 5.338568506 | 5.109475263 | 6.530825878 | 4.831459668 | 4.612235101 | 5.631825924 |
| ENSMUSG00000029377 | Ereg | 4131 | 1.224428939 | 1.137041415 | 0.292747097 | 1.180109355 | 0.524312894 | 0.719720168 | 0 | 0.009245316 | 0 | 0.205449687 | 0.046259945 | 0.108091334 | 0 | 0.067456917 | 0.289238712 | 0 | 0 | 0 |
| ENSMUSG00000029378 | Areg | 1193 | 5.094920513 | 5.054177581 | 2.14903362 | 3.551699181 | 4.316945375 | 4.859740007 | 0 | 0.224096242 | 0.034523483 | 0.790456048 | 0 | 0.467859703 | 0 | 0.155722003 | 0.207216543 | 0.674017398 | 0.192755894 | 0.69164845 |
| ENSMUSG00000029379 | Cxcl3 | 1005 | 0 | 0 | 0 | 0 | 0.383139691 | 0 | 0 | 0 | 0 | 0 | 0 | 0 | 0 | 0 | 0 | 0 | 0 | 0 |
| ENSMUSG00000029380 | Cxcl1 | 949 | 1.388472557 | 1.474332515 | 1.019463232 | 2.928581354 | 5.629761356 | 3.289591376 | 6.73046539 | 14.97109978 | 4.253191301 | 6.856477397 | 3.473627118 | 20.11481202 | 6.653830781 | 15.56292918 | 10.41978231 | 21.74777386 | 19.99106108 | 23.82375433 |

| ENSMUSG00000029381 | Shroom3 | 7679 | 4.461410585 | 7.422628208 | 4.982870492 | 5.470410584 | 5.935790039 | 6.15618021 | 2.544619924 | 2.844908735 | 2.901667538 | 3.340276413 | 3.60847453 | 3.852363741 | 4.195043884 | 4.451471327 | 3.616336589 | 4.881690929 | 4.886241004 | 4.65632496 |
| --- | --- | --- | --- | --- | --- | --- | --- | --- | --- | --- | --- | --- | --- | --- | --- | --- | --- | --- | --- | --- |
| ENSMUSG00000029384 | 2010109A12R | 546 | 0.70063477 | 0.671138895 | 0.885962094 | 0.417225681 | 0.617075944 | 0.090755922 | 1.906371678 | 1.818685829 | 0.150866358 | 0.690852795 | 0.43749962 | 2.146756253 | 0.993866465 | 1.190874036 | 0.3018429 | 0.701293176 | 1.403893659 | 0.503746399 |
| ENSMUSG00000029385 | Ccng2 | 3699 | 53.21477972 | 61.91569282 | 53.7352896 | 57.70572931 | 59.43947751 | 58.26029693 | 45.64989216 | 46.66927788 | 49.74894632 | 53.42216744 | 57.16462996 | 58.84863046 | 53.21285481 | 60.04207242 | 61.20642055 | 54.83248105 | 63.88752276 | 59.20282613 |
| ENSMUSG00000029386 | Tctn2 | 7547 | 3.137058838 | 3.553316529 | 3.025348659 | 2.909821567 | 2.308699664 | 3.80821326 | 2.933925234 | 3.274212847 | 2.215678468 | 2.886395244 | 3.0575467 | 3.121002996 | 3.078750899 | 2.966218381 | 3.08452088 | 3.0746135 | 2.249708431 | 2.419905053 |
| ENSMUSG00000029387 | Gtf2h3 | 3862 | 8.573674812 | 10.73913861 | 9.694746891 | 10.0866631 | 9.74602908 | 9.251041147 | 12.14056572 | 11.43200859 | 10.60056879 | 11.37867826 | 12.93956669 | 10.76713325 | 10.32112614 | 11.94173454 | 12.24738119 | 9.894879903 | 10.5094636 | 11.43767807 |
| ENSMUSG00000029388 | Eif2b1 | 2559 | 23.93511475 | 24.20034011 | 21.30401278 | 24.94373705 | 22.72112692 | 22.61726957 | 30.72828151 | 25.71532214 | 26.08962175 | 28.37517898 | 27.66802988 | 26.39193112 | 28.99382711 | 29.51078003 | 25.21361334 | 28.53465741 | 24.06813565 | 24.76377137 |
| ENSMUSG00000029389 | Ddx55 | 5278 | 3.873624416 | 3.471408075 | 3.647719796 | 2.960863624 | 2.899952961 | 2.994945436 | 3.702185752 | 4.023299685 | 4.128015695 | 2.956969075 | 3.557324495 | 2.464010872 | 3.495668257 | 4.901355945 | 3.481601729 | 3.64188801 | 3.739682248 | 3.793731498 |
| ENSMUSG00000029390 | Tmed2 | 2443 | 40.83489486 | 45.03990569 | 46.33404054 | 42.95009624 | 42.75328463 | 44.5021275 | 41.85128906 | 46.74387359 | 43.54677455 | 46.49449208 | 43.84423124 | 43.52387934 | 46.1615894 | 42.20461482 | 44.54085514 | 45.37507508 | 45.49580885 | 43.88569341 |
| ENSMUSG00000029392 | Rilpl1 | 2238 | 24.63324991 | 21.49411048 | 18.32920185 | 22.39372152 | 18.71080143 | 25.35204645 | 22.04965332 | 18.5842387 | 17.70394467 | 19.8041381 | 21.00561177 | 21.02439837 | 18.71438685 | 15.83415491 | 17.72881963 | 22.00255635 | 15.92647727 | 19.83572347 |
| ENSMUSG00000029394 | Cdk2ap1 | 2488 | 4.937297376 | 4.913919686 | 4.21907399 | 4.321706778 | 4.372112037 | 4.023172099 | 4.754087513 | 3.469245556 | 3.84054327 | 4.320884382 | 4.090058744 | 4.195148273 | 3.945760264 | 4.162794006 | 4.057227168 | 4.401573037 | 2.988465273 | 3.758660829 |
| ENSMUSG00000029397 | Rchy1 | 3746 | 24.40700311 | 22.60581343 | 21.78487606 | 25.21303922 | 23.28217984 | 24.19432721 | 30.00932331 | 24.90764524 | 25.48594328 | 26.04239323 | 27.10138461 | 26.03042246 | 32.29093998 | 26.97873309 | 31.52259989 | 33.03663725 | 35.22622537 | 30.48555941 |
| ENSMUSG00000029401 | Rilpl2 | 1387 | 26.50827488 | 26.20358154 | 22.63476655 | 21.74579045 | 21.23773416 | 20.4355902 | 37.42033798 | 30.50986436 | 26.48765109 | 34.84460767 | 34.99593209 | 28.81327643 | 30.69462481 | 27.99369651 | 30.06198599 | 37.60043784 | 30.34050036 | 28.15895153 |
| ENSMUSG00000029402 | Snrnp35 | 1388 | 10.96315059 | 10.77628927 | 7.074803071 | 7.254316137 | 9.120097928 | 9.389314792 | 9.782953064 | 10.20848796 | 10.17793581 | 9.341818009 | 9.12129971 | 8.766436919 | 7.996888607 | 10.13873089 | 6.263348914 | 10.34508485 | 9.333060773 | 3.923560206 |
| ENSMUSG00000029403 | Cdkl2 | 5282 | 4.981201053 | 3.967022049 | 4.972894166 | 4.744145094 | 3.316929234 | 4.775149828 | 5.150471485 | 3.832255448 | 6.331588565 | 4.293731213 | 3.807886694 | 2.039459084 | 3.950662819 | 4.000768603 | 3.299556634 | 2.892455955 | 3.156368639 | 4.769816524 |
| ENSMUSG00000029404 | Arl6ip4 | 1578 | 34.12804691 | 37.59839329 | 31.81985077 | 31.73104419 | 38.64584746 | 36.99183787 | 36.6688253 | 34.29571243 | 35.52271733 | 35.64688782 | 40.23632435 | 36.39690545 | 45.58044068 | 50.65289886 | 42.95092487 | 39.50387126 | 44.05823991 | 49.57094408 |
| ENSMUSG00000029405 | G3bp2 | 7556 | 37.00357966 | 34.63553428 | 37.93186439 | 39.03078878 | 37.47486857 | 38.2990953 | 29.22916692 | 29.38227041 | 29.40725947 | 30.92626857 | 28.45885232 | 31.61607145 | 24.11100721 | 22.99461764 | 25.83549124 | 27.94768394 | 30.47944919 | 24.14838632 |
| ENSMUSG00000029406 | Pitpnm2 | 15707 | 0.018942906 | 0.040297001 | 0.052355639 | 0.020304788 | 0.033707975 | 0.018928911 | 0.231939662 | 0.277197038 | 0.12586444 | 0.099062406 | 0.015208174 | 0.206106095 | 0.16646028 | 0.378483682 | 0.204604403 | 0.056069521 | 0.102483254 | 0.318700497 |
| ENSMUSG00000029407 | Uso1 | 3873 | 22.59699392 | 30.27662991 | 32.22404036 | 27.97422242 | 29.36631439 | 31.20555569 | 34.59579311 | 30.69789535 | 27.94685345 | 32.15208038 | 25.95365676 | 26.83417602 | 28.65913087 | 30.15927699 | 29.80809698 | 27.36596987 | 32.1216524 | 23.35010882 |
| ENSMUSG00000029408 | Abcb9 | 5774 | 1.170475059 | 1.4077496 | 0.904804517 | 0.907433339 | 0.900285375 | 1.167158255 | 1.376607215 | 1.633793452 | 1.490817768 | 1.453554759 | 1.290767843 | 0.657337211 | 1.905268021 | 1.480033266 | 1.484226468 | 1.067680601 | 2.037785443 | 1.648177254 |
| ENSMUSG00000029409 | U90926 | 514 | 0 | 0 | 0 | 0 | 0 | 0 | 0 | 0 | 0 | 0 | 0 | 0 | 0 | 0 | 0.080158669 | 0.14899069 | 0.07456478 | 0.214043217 |
| ENSMUSG00000029410 | Ppef2 | 2258 | 0 | 0 | 0 | 0 | 0.085264701 | 0 | 0 | 0 | 0 | 0 | 0 | 0 | 0 | 0 | 0 | 0 | 0 | 0 |
| ENSMUSG00000029413 | Naaa | 4989 | 0.46858783 | 0.560890582 | 0.271489046 | 0.538805696 | 0.221895018 | 0.526417094 | 0.25605178 | 0.796153508 | 0.47881698 | 0.642663424 | 0.402194479 | 0.358007858 | 0.909708637 | 0.754053133 | 0.644161427 | 0.552600468 | 0.299604245 | 0.253599811 |
| ENSMUSG00000029414 | Kntc1 | 6897 | 0.0246514 | 0.06762078 | 0.021041118 | 0.039635532 | 0.034893377 | 0.071846794 | 0.020579678 | 0.027687692 | 0.149291416 | 0.020509223 | 0.013853837 | 0.01618549 | 0.042915978 | 0.04040373 | 0.017921512 | 0.155449762 | 0.172265507 | 0.055830614 |
| ENSMUSG00000029415 | Sdad1 | 4998 | 5.808530458 | 6.238669266 | 6.097503826 | 5.405702139 | 4.930681256 | 5.254691636 | 6.465498141 | 6.556438828 | 6.889141092 | 5.433934589 | 5.228671926 | 5.058922599 | 5.606348342 | 5.872881799 | 5.712820775 | 4.864853081 | 6.556419338 | 7.396182417 |
| ENSMUSG00000029416 | Slc15a4 | 4038 | 11.72629175 | 13.05126267 | 13.4171258 | 13.5735355 | 12.87332282 | 13.9528128 | 13.69700883 | 12.74025905 | 10.19973149 | 14.71270087 | 12.63587312 | 14.40315018 | 10.12782551 | 10.99567795 | 12.51964068 | 14.3660897 | 14.84455878 | 11.41595627 |
| ENSMUSG00000029417 | Cxcl9 | 5200 | 0.024522217 | 0.044844281 | 0 | 0 | 0 | 0 | 0 | 0 | 0 | 0 | 0 | 0 | 0 | 0 | 0 | 0 | 0 | 0 |
| ENSMUSG00000029419 | Gm996 | 4683 | 0 | 0 | 0 | 0 | 0 | 0 | 0 | 0 | 0 | 0 | 0 | 0 | 0 | 0 | 0 | 0 | 0 | 0 |
| ENSMUSG00000029420 | Rimbp2 | 4242 | 0 | 0 | 0 | 0 | 0 | 0 | 0 | 0 | 0 | 0 | 0 | 0 | 0 | 0 | 0 | 0 | 0 | 0 |
| ENSMUSG00000029422 | Rsrc2 | 2538 | 40.81367769 | 42.38271688 | 36.86146876 | 35.16709451 | 41.05816184 | 40.29820414 | 37.56306069 | 45.61117876 | 47.75883211 | 36.74708079 | 50.89971936 | 42.00469485 | 43.05366303 | 45.62061066 | 42.45156369 | 51.61235931 | 51.69066757 | 50.8476613 |
| ENSMUSG00000029423 | Piwil1 | 3922 | 0 | 0 | 0.086337255 | 0 | 0 | 0 | 0 | 0 | 0 | 0 | 0 | 0 | 0 | 0 | 0 | 0 | 0 | 0 |
| ENSMUSG00000029426 | Scarb2 | 2098 | 28.08015919 | 29.37504972 | 27.11500081 | 28.90455196 | 29.04433525 | 30.75198242 | 20.36384608 | 26.08661232 | 21.67298065 | 25.12606589 | 22.61226586 | 22.69340333 | 28.94546719 | 21.09686143 | 23.44835926 | 25.2593902 | 27.76738384 | 23.12584944 |
| ENSMUSG00000029427 | Zcchc8 | 4272 | 17.94928314 | 19.40132978 | 20.30284174 | 21.61803297 | 19.25502283 | 20.15979657 | 19.60286557 | 16.95056062 | 18.40474218 | 17.93536816 | 18.59778931 | 18.0826023 | 15.37003322 | 16.78597166 | 18.73937807 | 14.69957775 | 16.88438454 | 17.07445588 |
| ENSMUSG00000029428 | Stx2 | 5773 | 1.281119111 | 1.696516698 | 2.170231138 | 1.673123402 | 1.475723279 | 1.236028691 | 1.827598718 | 1.137899396 | 0.349582413 | 1.167945707 | 0.935141228 | 1.063523798 | 1.486879197 | 1.5205149 | 1.327470881 | 1.611747375 | 0.942722009 | 0.781352287 |
| ENSMUSG00000029430 | Ran | 2716 | 48.21739593 | 41.88642627 | 42.54947129 | 42.50798466 | 45.0129183 | 44.59016232 | 47.31267893 | 40.40013701 | 42.58164074 | 42.98421991 | 43.65885381 | 39.90943179 | 40.52264633 | 42.87014988 | 38.18273792 | 42.15350373 | 39.99143057 | 39.04917443 |
| ENSMUSG00000029431 | B3gnt4 | 1422 | 0 | 0 | 0 | 0 | 0 | 0 | 0 | 0 | 0 | 0 | 0 | 0 | 0 | 0 | 0 | 0 | 0 | 0 |
| ENSMUSG00000029432 | Gbas | 2785 | 49.52577961 | 44.0663204 | 44.22226509 | 46.24814088 | 45.03834581 | 43.68113499 | 45.78372342 | 48.35418703 | 52.05620664 | 46.08409848 | 51.49745973 | 44.61244701 | 46.09035862 | 39.42327562 | 42.65137725 | 48.39602803 | 48.81270202 | 42.7629142 |
| ENSMUSG00000029433 | Diablo | 5015 | 21.51956968 | 19.64896134 | 20.13071941 | 21.15885788 | 23.8692112 | 19.9791879 | 22.07610542 | 20.81352657 | 21.35292941 | 22.51763396 | 25.28309866 | 20.20048407 | 24.26756529 | 24.44913075 | 22.76560546 | 26.39494113 | 24.25676299 | 24.23033235 |
| ENSMUSG00000029434 | Vps33a | 4094 | 12.15768469 | 14.19093491 | 14.6278531 | 16.93793588 | 13.21452912 | 13.71354351 | 15.43960407 | 12.68726607 | 14.64767146 | 15.8589444 | 17.0608181 | 13.45629188 | 12.037759 | 13.88557962 | 15.90094243 | 14.15087665 | 17.24402513 | 16.52691775 |
| ENSMUSG00000029436 | Mmp17 | 3479 | 0 | 0.172357602 | 0.027808871 | 0.013096017 | 0 | 0 | 0 | 0.065867897 | 0.071031645 | 0.271058944 | 0.205985736 | 0.336915468 | 0.056719652 | 0.013349838 | 0.047371723 | 0 | 0.088131755 | 0.031623516 |
| ENSMUSG00000029437 | Il31 | 1627 | 0 | 0 | 0 | 0 | 0 | 0 | 0 | 0 | 0 | 0 | 0 | 0 | 0 | 0 | 0 | 0.04706897 | 0 | 0.101430436 |
| ENSMUSG00000029438 | Bcl7a | 8572 | 8.216411185 | 7.034103889 | 6.788772401 | 6.388751201 | 7.361286973 | 6.682566497 | 6.203855706 | 6.077279151 | 5.376541197 | 5.621560748 | 6.247751803 | 4.942147446 | 4.920530713 | 6.014108376 | 5.267954416 | 4.967236984 | 5.105999891 | 5.313525474 |

| ENSMUSG00000029439 | Sfswap | 3949 | 6.619570336 | 8.224885253 | 8.060213465 | 7.337762522 | 6.313582289 | 7.428518176 | 8.410610435 | 8.065956851 | 7.738767965 | 8.668374415 | 9.448529393 | 9.074108731 | 9.056885294 | 6.597906565 | 8.002429316 | 9.512050104 | 7.531326006 | 7.689295242 |
| --- | --- | --- | --- | --- | --- | --- | --- | --- | --- | --- | --- | --- | --- | --- | --- | --- | --- | --- | --- | --- |
| ENSMUSG00000029440 | Psmd9 | 4555 | 11.1791879 | 10.59723024 | 9.88710357 | 12.47302355 | 9.985657051 | 9.758244132 | 13.01488182 | 11.67991582 | 11.10363147 | 10.64125641 | 12.06173485 | 11.94737015 | 12.35734745 | 12.96967709 | 11.52377216 | 13.81992504 | 11.36747028 | 13.03069729 |
| ENSMUSG00000029442 | Wdr66 | 7560 | 0.039356644 | 0.07050348 | 0.044790306 | 0.048212745 | 0.006366657 | 0.085209727 | 0 | 0.03031143 | 0 | 0 | 0.06319439 | 0 | 0.006525386 | 0.049147182 | 0.005449941 | 0 | 0.005069616 | 0.160079411 |
| ENSMUSG00000029445 | Hpd | 2328 | 0 | 0.014309662 | 0 | 0 | 0.020675225 | 0.021285538 | 0 | 0 | 0 | 0 | 0 | 0 | 0 | 0 | 0.017698263 | 0.082239277 | 0 | 0 |
| ENSMUSG00000029446 | Psph | 1808 | 14.01166201 | 10.26287726 | 12.1736484 | 9.601082905 | 9.796763224 | 11.64818129 | 27.18908927 | 22.32819101 | 18.74806553 | 16.32540942 | 12.65719309 | 18.30679641 | 32.33314268 | 45.98170156 | 28.85020451 | 17.85341927 | 16.87374576 | 25.40520139 |
| ENSMUSG00000029447 | Cct6a | 1584 | 131.4333031 | 128.793033 | 129.6984744 | 128.1692005 | 141.509071 | 130.0446424 | 110.2170361 | 120.3880462 | 117.9432042 | 114.959606 | 125.0171735 | 108.0018976 | 106.5743819 | 109.1905186 | 110.3130041 | 111.8018051 | 118.8744297 | 99.87764579 |
| ENSMUSG00000029449 | Rhof | 4595 | 0.27750931 | 0.23199404 | 0.23160341 | 0.267714515 | 0.230446642 | 0.02156811 | 0.339786378 | 0.149611152 | 0.179266663 | 0.17444221 | 0.86296443 | 0.024294086 | 0.934031948 | 0.252688179 | 0.349697645 | 0.433321346 | 0.400361752 | 0.17957271 |
| ENSMUSG00000029451 | Gm13103 | 2037 | 0 | 0 | 0 | 0 | 0 | 0 | 0 | 0 | 0 | 0 | 0 | 0 | 0 | 0 | 0 | 0 | 0 | 0 |
| ENSMUSG00000029452 | Tmem116 | 3714 | 0.78967613 | 0.57405095 | 1.054996219 | 0.576566797 | 0.388787752 | 0.933950283 | 0.535038372 | 0.853518955 | 0.60992417 | 1.041022527 | 0.810399134 | 0.721365591 | 0.624286514 | 0.375154179 | 0.809831335 | 0.659827376 | 0.742997678 | 0.518394921 |
| ENSMUSG00000029454 | Mapkapk5 | 5866 | 5.122938877 | 4.46367795 | 5.599322726 | 4.06989213 | 4.463649246 | 4.156144719 | 4.419936593 | 4.700803706 | 4.935922363 | 4.718285512 | 5.969833714 | 5.766159483 | 3.910559416 | 5.645181437 | 5.373199839 | 5.280787826 | 6.0958805 | 4.998270352 |
| ENSMUSG00000029455 | Aldh2 | 4691 | 242.89837 | 254.0330104 | 236.8354022 | 265.8002772 | 255.3526294 | 241.5207206 | 253.4370062 | 228.3972425 | 205.0458088 | 268.8122805 | 238.5285232 | 219.7169101 | 299.8404659 | 281.1000915 | 284.6780707 | 317.6218536 | 292.8599686 | 301.5826491 |
| ENSMUSG00000029456 | Acad10 | 6397 | 3.58141158 | 4.186894943 | 3.05501114 | 4.344573557 | 3.769594147 | 3.392855605 | 3.964295124 | 4.077756893 | 2.304951171 | 4.584608008 | 5.362266717 | 3.804225244 | 3.477988853 | 3.463159244 | 4.907860808 | 3.411856529 | 4.09804394 | 3.448280725 |
| ENSMUSG00000029458 | Brap | 5142 | 17.32610831 | 18.72311635 | 20.62131049 | 20.25526008 | 19.94732192 | 17.02833143 | 18.2644239 | 18.38315752 | 16.49222792 | 18.99049134 | 16.92842753 | 16.78160528 | 15.80118004 | 19.68138203 | 19.55912056 | 16.68791349 | 18.46996572 | 20.59364653 |
| ENSMUSG00000029461 | Fam168a | 6858 | 3.811706512 | 4.201757582 | 4.380280308 | 3.667205668 | 3.726749995 | 3.48993443 | 3.959970502 | 4.065391332 | 3.555324779 | 3.843284211 | 4.918215323 | 3.79266532 | 3.344902527 | 2.600543826 | 2.99789682 | 3.467259725 | 3.749919105 | 2.334157197 |
| ENSMUSG00000029462 | Vps29 | 3147 | 61.52242669 | 56.96113244 | 56.48958502 | 53.50925321 | 56.02390734 | 56.49672961 | 68.57106088 | 57.41603298 | 62.12659368 | 56.39505785 | 60.80035231 | 56.59605317 | 69.67917772 | 66.500495 | 56.91234937 | 68.61160951 | 58.89608256 | 59.72866783 |
| ENSMUSG00000029463 | 1500011H22R | 1381 | 14.83526057 | 13.36375337 | 15.58741565 | 13.1965371 | 12.16367948 | 14.28094711 | 15.24557721 | 10.45382195 | 13.71889736 | 13.8959712 | 15.63669894 | 14.9946494 | 10.07357036 | 12.98147555 | 12.7990351 | 13.53064186 | 12.1001198 | 13.22449199 |
| ENSMUSG00000029464 | Gpn3 | 2614 | 15.80529116 | 13.12635082 | 14.1937635 | 16.27927139 | 14.73050457 | 14.74828872 | 18.37122001 | 19.63679756 | 18.22983884 | 16.19790801 | 15.62646882 | 18.91839214 | 17.92858509 | 21.69404388 | 20.25401657 | 17.84160666 | 19.91090711 | 16.24599479 |
| ENSMUSG00000029465 | Arpc3 | 2647 | 85.25122007 | 67.56967475 | 68.27484299 | 63.66841821 | 71.93422366 | 66.32615605 | 77.85947465 | 68.36252459 | 75.12221311 | 72.64093989 | 71.13000052 | 62.83742898 | 75.88952291 | 77.25474755 | 66.54199147 | 73.09499025 | 68.38500199 | 68.62110706 |
| ENSMUSG00000029466 | Anapc7 | 3272 | 24.24042129 | 27.97794417 | 21.48127739 | 23.12863528 | 24.68379215 | 23.6405309 | 20.25827114 | 19.16623617 | 28.32202337 | 22.07667402 | 24.77814931 | 21.16969354 | 30.04844472 | 22.31360189 | 25.19691728 | 26.88066174 | 24.29362463 | 24.05807815 |
| ENSMUSG00000029467 | Atp2a2 | 5653 | 37.81329033 | 51.35713305 | 48.20238749 | 46.85058389 | 46.04589478 | 53.39212811 | 35.13508334 | 43.01627916 | 33.34701621 | 42.42979435 | 40.58293837 | 37.08537567 | 33.79842838 | 39.35382604 | 44.66356527 | 35.95374918 | 47.09259832 | 44.61644336 |
| ENSMUSG00000029468 | P2rx7 | 5767 | 4.075838797 | 6.007527308 | 4.269486206 | 5.02459237 | 4.072893836 | 4.897703856 | 3.248798484 | 5.072894094 | 4.74212701 | 3.540186321 | 4.224939973 | 3.668135273 | 5.106841445 | 3.889803053 | 4.065143975 | 4.016961588 | 5.376447754 | 4.549912243 |
| ENSMUSG00000029469 | Ift81 | 4427 | 5.847222092 | 5.267455603 | 4.81877725 | 5.639813033 | 5.47966784 | 5.630229273 | 5.386399408 | 4.69317075 | 4.530795838 | 6.113508848 | 5.708821558 | 6.682245577 | 4.780526883 | 3.535499763 | 5.481752071 | 5.155003843 | 5.809113445 | 4.771515017 |
| ENSMUSG00000029470 | P2rx4 | 3493 | 32.62421326 | 35.75437745 | 30.68873268 | 34.73491585 | 31.63781756 | 34.35921006 | 38.84705501 | 37.2958158 | 37.17752196 | 40.41488851 | 46.72180308 | 39.24513808 | 47.51004 | 46.41749473 | 47.50033369 | 50.06390037 | 44.92065832 | 41.1977736 |
| ENSMUSG00000029471 | Camkk2 | 3402 | 4.273007114 | 3.99519719 | 4.038237102 | 3.013296583 | 4.598140856 | 3.918190868 | 3.894047648 | 3.480201277 | 2.506057837 | 3.035274548 | 3.552929011 | 2.44460133 | 3.509207519 | 2.279883185 | 4.190399276 | 2.532447579 | 2.151770343 | 4.074748646 |
| ENSMUSG00000029472 | Anapc5 | 2733 | 97.03248929 | 100.158087 | 94.32218927 | 98.02376169 | 100.1735755 | 93.03149506 | 87.9430695 | 100.1974114 | 103.290296 | 92.93846998 | 89.08203015 | 90.92255025 | 79.22348515 | 98.39416977 | 86.95593647 | 88.08380854 | 93.73324863 | 99.95434467 |
| ENSMUSG00000029474 | Rnf34 | 1948 | 36.65744134 | 33.22738884 | 31.51232547 | 35.10632831 | 36.46956845 | 34.11202039 | 33.88151289 | 30.5656855 | 38.37450006 | 36.01655362 | 40.12311708 | 33.49512811 | 30.94640807 | 34.14164946 | 40.69393919 | 42.35947585 | 36.37850255 | 35.21373516 |
| ENSMUSG00000029475 | Kdm2b | 12990 | 1.49864285 | 1.292509522 | 1.053865211 | 1.129380776 | 1.578460314 | 1.262660109 | 1.329417075 | 1.340703271 | 1.2206935 | 1.063522406 | 1.228393852 | 1.121469433 | 0.675987781 | 1.061885601 | 1.506600389 | 1.123073242 | 1.020854406 | 1.520267076 |
| ENSMUSG00000029477 | Morn3 | 1546 | 0.082480937 | 0.172382376 | 0.312894763 | 0.206291921 | 0.031133198 | 0.096156663 | 0.642668989 | 0.345856167 | 0.479532525 | 0.152492572 | 0.278120716 | 0.108309824 | 0 | 0.030041454 | 0.026650424 | 0.123837669 | 0.223115571 | 0.106744709 |
| ENSMUSG00000029478 | Ncor2 | 11609 | 9.684399224 | 10.28168662 | 10.55475514 | 10.60435367 | 8.665321773 | 11.23035938 | 7.869823672 | 9.073533106 | 8.273490805 | 8.963873037 | 6.580425412 | 7.264834712 | 5.796255936 | 6.057054725 | 6.285464337 | 6.431792957 | 5.876544792 | 5.842555654 |
| ENSMUSG00000029480 | Dhx37 | 5491 | 2.577713279 | 3.367083644 | 3.180266702 | 3.385340756 | 3.751677897 | 3.104378137 | 2.696934712 | 3.331662858 | 2.062701117 | 2.799331499 | 1.9837353 | 3.130800232 | 2.17416208 | 1.776226252 | 3.421582496 | 1.722414866 | 3.161867146 | 3.516335178 |
| ENSMUSG00000029482 | Aacs | 3651 | 33.95990098 | 26.91674553 | 29.8111321 | 32.17101405 | 27.32880793 | 31.8407869 | 27.00619635 | 26.53906461 | 23.26118748 | 25.02822868 | 19.91604678 | 29.3678137 | 26.59140323 | 24.7930776 | 28.03195421 | 23.90147748 | 24.96300576 | 26.21633681 |
| ENSMUSG00000029483 | Tmem132b | 1805 | 1.012588681 | 3.119046606 | 1.795582567 | 2.423191278 | 1.599953141 | 0.851044178 | 0.183484073 | 0.169273806 | 0 | 0.548567739 | 0 | 0.123691219 | 0.13665351 | 0 | 0.821748483 | 0.615195354 | 0.828102814 | 1.066658579 |
| ENSMUSG00000029484 | Anxa3 | 1584 | 8.560070172 | 15.562842 | 11.08560071 | 7.305874534 | 8.751258849 | 11.19941832 | 5.645262827 | 4.774050301 | 4.212257294 | 1.637177196 | 1.779496559 | 2.748498536 | 2.055496553 | 3.195963084 | 2.080886661 | 3.795218032 | 1.475949565 | 2.569869885 |
| ENSMUSG00000029486 | Mrpl1 | 4285 | 7.300772359 | 5.861825494 | 6.276705456 | 6.900610916 | 6.806988505 | 6.464405617 | 6.73529385 | 7.968263186 | 6.862817329 | 8.989994063 | 8.161323129 | 7.724314566 | 8.841753201 | 8.540943025 | 7.586470851 | 9.096811747 | 8.023031114 | 6.714063668 |
| ENSMUSG00000029490 | Mfsd7a | 2498 | 1.327223271 | 1.38692594 | 0.774596163 | 0.437736215 | 0.462436416 | 1.011685113 | 0.814429621 | 1.070243462 | 0.90682881 | 1.283525949 | 0.860637763 | 2.413167157 | 0.987428288 | 1.338660645 | 1.138073401 | 1.072995404 | 0.398912618 | 0.990956686 |
| ENSMUSG00000029491 | Pde6b | 5275 | 0.435124077 | 0.322077271 | 0.458516875 | 0.224566285 | 0.145992565 | 0.272422611 | 0.224230706 | 0.028961063 | 0.00780787 | 0.286032702 | 0 | 0.169298692 | 0.224448534 | 0 | 0.015621443 | 0 | 0.152578623 | 0.333704534 |
| ENSMUSG00000029499 | Pxmp2 | 1527 | 0.890743702 | 0.589029564 | 1.393867279 | 1.84989178 | 1.229302569 | 1.265590445 | 4.368754243 | 3.176447351 | 2.832078687 | 2.624629847 | 3.097394165 | 3.033857233 | 3.973690783 | 3.406508047 | 1.672885701 | 1.980980999 | 2.40951179 | 3.206162736 |
| ENSMUSG00000029500 | Pgam5 | 3649 | 4.81080781 | 4.172099934 | 4.984501895 | 4.819556896 | 4.484750355 | 4.196161873 | 5.30306541 | 4.804141602 | 4.717994954 | 4.432088577 | 5.106120528 | 4.267626709 | 3.582613888 | 3.754728906 | 3.884169698 | 3.494319612 | 3.875692945 | 3.723554223 |
| ENSMUSG00000029501 | Ankle2 | 5205 | 9.742300673 | 10.5090821 | 10.30667534 | 11.89576547 | 10.30143381 | 9.786783884 | 8.544460748 | 9.979186796 | 9.250151187 | 10.5262473 | 11.25304498 | 9.425930339 | 7.601190712 | 11.41248564 | 8.398626475 | 10.53451485 | 10.86832166 | 10.42055317 |
| ENSMUSG00000029502 | Golga3 | 12161 | 3.523165647 | 3.980234792 | 3.404961926 | 3.353107037 | 3.494818567 | 3.569459306 | 3.408100217 | 4.110998664 | 3.989615621 | 3.04360678 | 2.69890605 | 3.970113324 | 2.527241548 | 3.525030233 | 2.45630524 | 3.384787678 | 3.580188577 | 3.039726973 |
| ENSMUSG00000029503 | P2rx2 | 2455 | 1.038823039 | 1.153399596 | 0.827571599 | 0.890806569 | 0.862649548 | 1.55419979 | 2.775163245 | 6.300579623 | 5.133634959 | 2.419954627 | 2.743897819 | 6.957064257 | 0.663117422 | 0.756726475 | 1.409747737 | 0 | 0.031223052 | 0.739429948 |

| ENSMUSG00000029504 | Ddx51 | 5478 | 3.561496129 | 3.66089126 | 3.214305412 | 4.075376364 | 3.839658752 | 3.211248708 | 3.636114974 | 4.231980332 | 3.075079399 | 3.804419649 | 3.828624821 | 4.748101272 | 3.746271922 | 3.620230981 | 3.97875558 | 4.284801449 | 3.260323906 | 4.719656838 |
| --- | --- | --- | --- | --- | --- | --- | --- | --- | --- | --- | --- | --- | --- | --- | --- | --- | --- | --- | --- | --- |
| ENSMUSG00000029505 | Ep400 | 15551 | 5.245156761 | 6.150171648 | 5.157437678 | 5.033365968 | 5.43190316 | 5.088786285 | 4.323279323 | 5.056790981 | 4.356749947 | 4.748119176 | 4.617436988 | 4.059386174 | 4.203253192 | 4.306628128 | 4.392783722 | 4.390209826 | 4.177420954 | 4.181130741 |
| ENSMUSG00000029507 | Pus1 | 2440 | 9.354622757 | 7.154080562 | 6.423370424 | 7.786457167 | 8.068015074 | 7.981239466 | 10.95559983 | 7.106291267 | 9.90839539 | 9.44946471 | 9.946589716 | 8.715478461 | 10.31117943 | 9.345920864 | 10.14841602 | 7.877821687 | 9.267424251 | 8.860073337 |
| ENSMUSG00000029510 | Gpc2 | 7394 | 3.437665034 | 3.2934441 | 4.376777361 | 3.493793906 | 3.358949501 | 4.000944272 | 3.442550888 | 2.995887665 | 3.570537815 | 2.206402938 | 2.752519923 | 3.366754871 | 2.161690722 | 2.298963482 | 3.159491776 | 1.097864318 | 2.721301849 | 2.447659739 |
| ENSMUSG00000029512 | Ulk1 | 5185 | 17.67428342 | 22.57052987 | 20.07711424 | 21.10658216 | 20.0975149 | 19.95489059 | 19.3995671 | 20.86034399 | 20.61311637 | 19.77874245 | 18.22543257 | 20.61465648 | 16.3361431 | 15.62169497 | 15.95616668 | 15.07254189 | 16.83843864 | 17.04910984 |
| ENSMUSG00000029513 | Prkab1 | 3618 | 33.0478331 | 35.72527075 | 29.36104828 | 33.29557801 | 36.15880833 | 33.08994039 | 26.41559188 | 33.27320409 | 29.27908196 | 38.6406399 | 40.06335656 | 34.04786272 | 27.92475583 | 31.47620295 | 33.05918097 | 30.58591912 | 33.66527683 | 31.42725915 |
| ENSMUSG00000029516 | Cit | 14608 | 0.020368033 | 0.050170021 | 0.062917379 | 0.099805135 | 0.052718427 | 0.078019775 | 0.071254035 | 0.156869123 | 0.107139074 | 0.041960511 | 0.075220704 | 0.08788063 | 0.11819668 | 0.019076159 | 0.076152931 | 0.070772618 | 0.118064305 | 0.067782306 |
| ENSMUSG00000029517 | Ankrd7 | 1187 | 0 | 0 | 0 | 0 | 0 | 0 | 0 | 0 | 0 | 0 | 0 | 0 | 0.041560166 | 0 | 0 | 0 | 0 | 0 |
| ENSMUSG00000029518 | Rab35 | 2850 | 7.576361201 | 5.692413854 | 5.431413935 | 5.355421001 | 5.792719234 | 4.92049951 | 4.698066013 | 5.36033718 | 6.329717159 | 5.261025837 | 5.112758714 | 5.111539626 | 7.027634531 | 5.817733052 | 5.002013452 | 6.193673688 | 5.056381627 | 5.365800586 |
| ENSMUSG00000029521 | Chek2 | 2232 | 3.770620454 | 1.985042532 | 2.839127454 | 3.531389189 | 2.178012675 | 2.220104551 | 2.586087289 | 2.036243677 | 4.170319248 | 2.640608381 | 3.167873591 | 2.850799971 | 1.922883872 | 1.539812933 | 3.32270612 | 1.543976105 | 2.009039757 | 2.218109142 |
| ENSMUSG00000029522 | Pla2g1b | 762 | 0.055781071 | 0.874354179 | 0.888752526 | 0.418539777 | 0.189495762 | 0.520238673 | 0.372540779 | 0 | 0.540505456 | 0.061877563 | 0.501574367 | 0.292995604 | 0 | 0.121900492 | 0.540702833 | 0.100500282 | 0.653860708 | 0.866285145 |
| ENSMUSG00000029524 | Sirt4 | 2787 | 5.886974505 | 5.414690018 | 5.536833937 | 5.705347855 | 5.267397459 | 6.276323987 | 7.537434318 | 6.468178664 | 8.231391919 | 6.46270852 | 6.873971421 | 9.212488263 | 7.186493869 | 5.83259081 | 7.362172527 | 5.660470129 | 5.390709862 | 9.257004325 |
| ENSMUSG00000029526 | 1700123K08R | 1041 | 0 | 0 | 0 | 0 | 0 | 0 | 0 | 0 | 0 | 0.090587326 | 0 | 0 | 0.04738897 | 0 | 0 | 0 | 0 | 0 |
| ENSMUSG00000029528 | Pxn | 8338 | 15.1913438 | 20.02049807 | 17.59617625 | 20.46913793 | 18.56467576 | 20.56872282 | 19.32681511 | 19.05039597 | 15.13498253 | 19.11927653 | 17.01746542 | 17.35788116 | 15.2291143 | 18.44840698 | 17.88793863 | 19.43002638 | 20.0732716 | 18.76959806 |
| ENSMUSG00000029530 | Ccr9 | 3170 | 0 | 0.010508799 | 0.061039155 | 0.071862846 | 0 | 0 | 0 | 0 | 0 | 0 | 0 | 0 | 0.046686357 | 0 | 0 | 0 | 0 | 0 |
| ENSMUSG00000029534 | St7 | 9084 | 1.932478831 | 1.859273158 | 1.735994154 | 1.635061697 | 2.077027089 | 1.756492758 | 1.510422379 | 2.110588508 | 1.618624629 | 1.531204036 | 2.582288046 | 2.082949099 | 1.76495741 | 2.203588911 | 1.75528425 | 2.187673408 | 1.653886876 | 1.132397948 |
| ENSMUSG00000029535 | Triap1 | 1086 | 33.07262776 | 29.14111373 | 24.81036502 | 26.64020548 | 33.72780286 | 26.69277085 | 38.94800641 | 34.74594252 | 37.31816898 | 38.38049878 | 37.43691498 | 30.22063499 | 44.97108481 | 47.29941128 | 37.9767564 | 37.72647322 | 36.56173443 | 45.73961636 |
| ENSMUSG00000029536 | Gatc | 1791 | 35.0293913 | 35.35891229 | 32.73518637 | 38.74342296 | 35.44724027 | 32.81381464 | 38.1724294 | 39.25863363 | 27.89460839 | 35.59338403 | 36.3847255 | 28.8895138 | 30.54667575 | 24.86872128 | 29.76818164 | 26.59604445 | 31.45709461 | 36.15059666 |
| ENSMUSG00000029538 | Srsf9 | 1353 | 109.1060431 | 111.7570043 | 112.2280205 | 104.9958139 | 102.9518013 | 112.7663464 | 118.6139002 | 107.3790826 | 121.6722125 | 112.875926 | 105.7899302 | 101.2767566 | 123.7126351 | 125.1553161 | 123.0260797 | 122.4280618 | 110.7015287 | 113.6773558 |
| ENSMUSG00000029541 | Cyp2w1 | 1503 | 0.056560447 | 0 | 0 | 0 | 0 | 0 | 0 | 0 | 0 | 0 | 0 | 0 | 0 | 0 | 0 | 0 | 0 | 0 |
| ENSMUSG00000029544 | Cabp1 | 2550 | 1.383501808 | 1.201876968 | 1.327900833 | 1.858175926 | 1.812025362 | 1.088216894 | 3.154178595 | 2.216657081 | 2.874980316 | 2.866023139 | 3.503496955 | 2.779838882 | 0.870563246 | 0.418907455 | 1.017920793 | 0.825875846 | 0.721436177 | 0.992321141 |
| ENSMUSG00000029545 | Acads | 2253 | 23.95986398 | 29.48331606 | 27.80458136 | 26.77444416 | 23.90573572 | 25.16126374 | 26.52282001 | 26.20748074 | 22.57671858 | 28.23182365 | 27.56655394 | 25.71533856 | 25.90308839 | 21.41828975 | 25.12691425 | 18.91586597 | 26.84371794 | 23.07306074 |
| ENSMUSG00000029546 | Uncx | 3633 | 0 | 0 | 0 | 0 | 0 | 0 | 0 | 0 | 0 | 0 | 0 | 0 | 0 | 0 | 0 | 0 | 0 | 0 |
| ENSMUSG00000029547 | Ints1 | 7066 | 3.573177832 | 5.747016424 | 4.497793581 | 4.5328919 | 4.202858322 | 5.336771901 | 4.968299995 | 5.999655628 | 3.643019014 | 4.564262812 | 3.711923607 | 4.897496576 | 3.497776756 | 5.39634811 | 3.481082489 | 4.313518751 | 3.861919529 | 4.18056755 |
| ENSMUSG00000029550 | Sppl3 | 4102 | 26.97244593 | 26.40180866 | 26.50992107 | 28.7338912 | 25.4388129 | 28.0259244 | 21.66094857 | 24.43121987 | 23.83636968 | 26.6214112 | 27.26503603 | 25.79875579 | 19.80733002 | 17.4250245 | 19.11422742 | 17.41839917 | 16.73388536 | 21.38945032 |
| ENSMUSG00000029551 | Psmg3 | 836 | 26.6928438 | 20.80063491 | 26.84846661 | 21.74504389 | 25.04472105 | 26.31748051 | 29.88169195 | 25.58342745 | 35.32384187 | 23.4625509 | 29.37359887 | 25.50428601 | 33.16332238 | 32.49975015 | 26.9584343 | 21.93923558 | 25.94818666 | 26.71494896 |
| ENSMUSG00000029552 | Tes | 3320 | 129.3847316 | 132.3685845 | 118.7919858 | 124.5106492 | 131.8263801 | 119.0758158 | 99.17136526 | 113.1392403 | 109.1938891 | 106.7139712 | 105.7812409 | 117.2128914 | 116.0488777 | 100.9179658 | 105.9078548 | 126.0245383 | 113.9052926 | 97.04468014 |
| ENSMUSG00000029553 | Tfec | 1772 | 0.095948479 | 0.056398805 | 0 | 0 | 0 | 0 | 0 | 0 | 0 | 0 | 0 | 0 | 0 | 0 | 0 | 0.280913034 | 0 | 0 |
| ENSMUSG00000029554 | Mad1l1 | 4207 | 6.637960697 | 6.976149229 | 7.12897286 | 8.685514038 | 6.7959027 | 7.137855134 | 6.612753794 | 5.855502627 | 6.020847919 | 7.240160282 | 6.654653118 | 5.174259189 | 5.863075502 | 6.822544808 | 7.031784438 | 5.952473791 | 5.584506775 | 7.217738748 |
| ENSMUSG00000029556 | Hnf1a | 3924 | 0.010832104 | 0.016979049 | 0.012327607 | 0.04644347 | 0 | 0.025256235 | 0 | 0 | 0 | 0.012015979 | 0 | 0.014224175 | 0 | 0 | 0 | 0 | 0 | 0 |
| ENSMUSG00000029557 | Ftsj2 | 1440 | 6.405293888 | 5.343943448 | 5.374836706 | 4.935779803 | 3.576469328 | 4.748803635 | 6.505493353 | 5.596247852 | 6.692808811 | 5.107992853 | 5.208797556 | 5.775370643 | 7.091463108 | 6.676337562 | 6.094396809 | 6.833809793 | 4.418170337 | 2.215644576 |
| ENSMUSG00000029559 | 2210016L21R | 3448 | 14.81763967 | 12.74353465 | 13.25782662 | 13.87444796 | 15.32739333 | 14.74509996 | 15.51932711 | 11.51975015 | 14.09515331 | 14.75510696 | 12.88593718 | 14.16435753 | 13.52049357 | 11.88041911 | 12.36763641 | 14.11466416 | 13.31638738 | 10.80080199 |
| ENSMUSG00000029560 | Snx8 | 2837 | 5.064063975 | 4.43858795 | 3.734156908 | 5.540557031 | 4.954008358 | 5.851309746 | 6.620773188 | 4.765636395 | 4.355288942 | 5.202033881 | 4.614148263 | 4.229949755 | 6.920727203 | 8.742031253 | 5.126594723 | 6.073589472 | 6.552081071 | 7.096698294 |
| ENSMUSG00000029561 | Oasl2 | 4365 | 11.50025496 | 12.56960751 | 10.58344135 | 10.28124368 | 11.95303671 | 13.97466553 | 6.633530241 | 7.498485421 | 7.982541199 | 5.94109663 | 4.65162826 | 7.442088345 | 7.266992625 | 6.437267553 | 9.844953676 | 13.54426067 | 11.10716279 | 13.01819181 |
| ENSMUSG00000029563 | Foxp2 | 11588 | 0 | 0 | 0 | 0 | 0 | 0 | 0 | 0 | 0.017771193 | 0.020344625 | 0 | 0 | 0 | 0.040079468 | 0 | 0 | 0 | 0 |
| ENSMUSG00000029564 | 4930519G04R | 3729 | 0 | 0 | 0 | 0 | 0 | 0 | 0 | 0 | 0 | 0 | 0 | 0 | 0 | 0 | 0 | 0 | 0 | 0 |
| ENSMUSG00000029569 | Tmem168 | 5613 | 6.906239184 | 8.576007865 | 8.497470324 | 8.10082349 | 9.123885049 | 10.62916288 | 6.060540914 | 8.961231778 | 7.374389514 | 8.00545523 | 8.485949332 | 8.571726554 | 5.36120961 | 7.537958954 | 8.602925976 | 6.037268404 | 10.03052381 | 7.683438388 |
| ENSMUSG00000029570 | Lfng | 2274 | 0 | 0.117195758 | 0 | 0.080142558 | 0 | 0.065372999 | 0.166447419 | 0.201543021 | 0.036223849 | 0.290285772 | 0.06302765 | 0.073635439 | 0.30371453 | 0 | 0.289896611 | 0 | 0 | 0.072571381 |
| ENSMUSG00000029571 | Tmem106b | 6877 | 8.15862038 | 11.59193774 | 11.56406629 | 11.3886048 | 11.26134436 | 12.93400564 | 10.29912467 | 12.16247801 | 10.50474751 | 10.98377586 | 12.35882669 | 12.0445606 | 10.61672787 | 10.15056905 | 11.97643016 | 10.72938788 | 11.5140511 | 13.14235311 |
| ENSMUSG00000029575 | Mmab | 3879 | 2.334004252 | 3.58978855 | 2.855771707 | 4.392841088 | 4.268466549 | 4.228397735 | 4.732487606 | 4.962353909 | 4.47010135 | 4.521799848 | 6.601541053 | 4.057751186 | 5.392300316 | 4.96888277 | 4.40800353 | 4.442065824 | 4.762380796 | 4.580546912 |
| ENSMUSG00000029576 | Radil | 5191 | 0 | 0 | 0.046593653 | 0 | 0.027816562 | 0 | 0 | 0 | 0 | 0 | 0.119644473 | 0 | 0 | 0.286305297 | 0 | 0.051634416 | 0 | 0.095373138 |

| ENSMUSG00000029577 | Ube3b | 5384 | 15.02365341 | 16.43370116 | 13.89930376 | 16.21377432 | 17.34322658 | 17.53305302 | 11.68756742 | 17.15253139 | 15.5137916 | 15.26442715 | 16.85966217 | 17.08473476 | 17.82143 | 15.89830109 | 19.65185652 | 15.85248215 | 16.74995851 | 22.2018553 |
| --- | --- | --- | --- | --- | --- | --- | --- | --- | --- | --- | --- | --- | --- | --- | --- | --- | --- | --- | --- | --- |
| ENSMUSG00000029578 | Wipi2 | 8272 | 8.832978432 | 11.12309161 | 10.19282681 | 11.05428143 | 9.269119246 | 10.9864257 | 9.363014435 | 11.18254335 | 9.16638969 | 11.9415768 | 12.23829026 | 10.83655151 | 9.112568859 | 12.09387866 | 13.01993074 | 11.10020268 | 12.11133222 | 12.36241893 |
| ENSMUSG00000029580 | Actb | 2810 | 332.6143472 | 337.8828463 | 333.2437013 | 288.9802467 | 340.7774453 | 302.6596322 | 216.4092037 | 271.4922555 | 246.8845806 | 236.8609705 | 220.7679131 | 234.5846887 | 283.7901932 | 278.284377 | 263.6459703 | 325.2657647 | 283.7782681 | 290.784794 |
| ENSMUSG00000029581 | Fscn1 | 3077 | 1.229431481 | 2.046193373 | 1.509216417 | 1.954519939 | 1.689388286 | 1.578215109 | 1.153217718 | 1.439817575 | 1.204675469 | 2.12997977 | 2.065020955 | 1.922801506 | 1.683409592 | 1.313173742 | 2.584302986 | 1.468408084 | 1.90572747 | 2.484974272 |
| ENSMUSG00000029586 | 4933411G11R | 2311 | 0 | 0 | 0 | 0.039429722 | 0 | 0 | 0 | 0 | 0 | 0 | 0 | 0 | 0 | 0 | 0 | 0 | 0 | 0 |
| ENSMUSG00000029587 | Zfp12 | 5973 | 4.184336766 | 2.911322749 | 3.693006838 | 3.813916318 | 2.876795036 | 3.252079619 | 3.833808237 | 3.414489015 | 3.613215178 | 3.678591615 | 3.183397535 | 3.616383965 | 4.055243828 | 2.946979595 | 3.393799682 | 3.564302313 | 4.337615387 | 3.462820045 |
| ENSMUSG00000029591 | Ung | 2464 | 0.086252386 | 0.175757965 | 0.117792688 | 0.036981367 | 0.03906812 | 0 | 0.115209445 | 0.310003266 | 0.133722454 | 0.267901723 | 0.155113502 | 0.135914763 | 0.120126422 | 0.52777372 | 0.267542571 | 0.186480231 | 0.451080605 | 0.156275871 |
| ENSMUSG00000029592 | Usp30 | 3359 | 5.985396922 | 6.376954743 | 5.530049317 | 4.842302122 | 5.373465727 | 4.661704022 | 5.69047999 | 4.684510209 | 4.561293201 | 5.291996168 | 4.522904673 | 4.968408191 | 5.551492924 | 5.364782943 | 5.090397647 | 5.152531868 | 4.119021489 | 5.764574447 |
| ENSMUSG00000029594 | Rbm19 | 3061 | 2.096792414 | 2.133069999 | 1.769956027 | 1.994505044 | 1.619597562 | 1.748348653 | 1.514747643 | 2.670099351 | 1.803003304 | 1.740617272 | 1.467117314 | 1.40405293 | 2.320743577 | 2.169717248 | 1.642139764 | 2.451799755 | 2.416522477 | 2.174486087 |
| ENSMUSG00000029595 | Lhx5 | 2845 | 0 | 0 | 0 | 0 | 0 | 0 | 0 | 0 | 0 | 0 | 0 | 0 | 0 | 0 | 0 | 0 | 0 | 0 |
| ENSMUSG00000029596 | Sdsl | 1699 | 10.20724651 | 10.13700195 | 8.598473318 | 10.56565713 | 9.263766356 | 10.17887229 | 9.440257891 | 10.85752228 | 10.42389746 | 11.98887805 | 6.411236335 | 7.950200321 | 10.97555311 | 9.020923389 | 8.390664119 | 8.451428944 | 8.526980712 | 10.45788197 |
| ENSMUSG00000029597 | Sds | 1605 | 0.158897854 | 0.041511395 | 0 | 0.056773887 | 0.029988737 | 0.030873977 | 0.029478305 | 0 | 0 | 0.146886926 | 0.208364305 | 0.034776114 | 0.153681985 | 0.14468563 | 0.077012254 | 0 | 0.071637938 | 0 |
| ENSMUSG00000029598 | Plbd2 | 6859 | 22.51987311 | 23.66240278 | 21.53852773 | 24.38469074 | 24.1606959 | 25.86365158 | 25.17732586 | 23.25844363 | 21.87527 | 26.51410737 | 26.55170458 | 23.84310998 | 29.71854238 | 23.00874894 | 25.79377183 | 28.96777399 | 25.72035933 | 26.67448447 |
| ENSMUSG00000029599 | Ddx54 | 3132 | 25.98895661 | 33.84470926 | 28.41867684 | 40.1786732 | 32.77949974 | 35.8039068 | 29.53265878 | 29.92469844 | 26.02430226 | 30.45525948 | 26.60265887 | 27.35537742 | 21.54727421 | 22.85132141 | 27.17829325 | 23.63210221 | 25.73441966 | 21.44512111 |
| ENSMUSG00000029600 | 1110008J03R | 2827 | 3.187512316 | 3.794394036 | 3.867144627 | 3.57783935 | 3.439210676 | 3.610846522 | 4.217472618 | 3.647664892 | 4.589229736 | 5.087005481 | 4.005187534 | 5.548001835 | 4.345117581 | 4.090759731 | 5.421640888 | 4.401997667 | 3.009705664 | 3.405232995 |
| ENSMUSG00000029601 | Iqcd | 1742 | 0.219601943 | 0.420713934 | 0.499841301 | 0.392316685 | 0.690756654 | 0.369796519 | 0.787639316 | 0.306942384 | 0.236432352 | 0.406004907 | 0.082276048 | 0.544699348 | 0.566382518 | 0.586549898 | 0.378429905 | 0.263769971 | 0.088005274 | 0.726297046 |
| ENSMUSG00000029602 | Rasal1 | 3604 | 5.165723391 | 3.771271044 | 4.02665347 | 6.346183201 | 5.38212132 | 3.959818887 | 1.627850218 | 2.840056561 | 3.771240344 | 4.709837173 | 2.982620882 | 3.143889985 | 3.257768122 | 4.239762695 | 3.040958343 | 3.314836157 | 2.584153759 | 4.548477748 |
| ENSMUSG00000029603 | Dtx1 | 5528 | 0.192226737 | 0.108471797 | 0.105007664 | 0.288465368 | 0.243794114 | 0.152387205 | 0.102704802 | 0.179631415 | 0.305471626 | 0.025588298 | 0.2506284 | 0.050484499 | 0.374808344 | 0.369670739 | 0.104345474 | 0.263213293 | 0.048531852 | 0.119411954 |
| ENSMUSG00000029605 | Oas1b | 2097 | 2.614767624 | 1.588597721 | 2.329864838 | 1.260152871 | 1.95098403 | 2.906526576 | 2.572076966 | 1.875926298 | 1.571254774 | 1.798786963 | 1.754254555 | 1.623631577 | 2.164299661 | 1.195985083 | 2.357742826 | 1.625114 | 2.339421079 | 2.780622467 |
| ENSMUSG00000029607 | Ankrd61 | 3427 | 0 | 0.243017904 | 0.028230832 | 0.053178925 | 0.070224575 | 0.014459508 | 0.110446872 | 0.24518029 | 0.108164179 | 0.041275783 | 0.111526019 | 0.081435166 | 0.071975368 | 0.027104807 | 0.036067893 | 0.167598223 | 0.12301992 | 0 |
| ENSMUSG00000029608 | Rph3a | 4105 | 0 | 0 | 0 | 0 | 0 | 0 | 0 | 0 | 0 | 0 | 0 | 0 | 0 | 0 | 0 | 0 | 0 | 0 |
| ENSMUSG00000029610 | Aimp2 | 1650 | 14.65784555 | 13.60781255 | 14.89318389 | 12.59141589 | 13.12688827 | 13.84473344 | 11.81383256 | 15.06863877 | 15.87552971 | 14.71673465 | 15.28798671 | 11.60289228 | 14.26140881 | 14.32729726 | 14.08344092 | 13.01880652 | 12.03213442 | 11.56858184 |
| ENSMUSG00000029613 | Eif2ak1 | 5837 | 18.05213832 | 20.57443612 | 19.36764441 | 19.73245162 | 18.14945417 | 17.18258228 | 18.5538328 | 14.91185285 | 15.84802371 | 19.6212195 | 19.25894763 | 14.56351792 | 16.42988645 | 16.76506632 | 17.42789815 | 15.84891339 | 16.55312566 | 15.27664245 |
| ENSMUSG00000029614 | Rpl6 | 2367 | 48.05401396 | 42.74239955 | 40.48498675 | 37.72693152 | 39.61173946 | 40.09019214 | 47.0928059 | 40.17705196 | 43.34415326 | 41.17469524 | 42.97122374 | 36.99821487 | 54.20883685 | 57.45174815 | 41.81078887 | 48.59526178 | 44.73830095 | 49.87306423 |
| ENSMUSG00000029616 | Erp29 | 9054 | 8.544225797 | 8.613373688 | 7.747031038 | 7.658925279 | 7.873136619 | 7.842839322 | 8.84695885 | 9.482717098 | 9.953180527 | 8.4260921 | 9.170850214 | 8.908066317 | 10.97899418 | 10.44915021 | 9.287870066 | 9.350622152 | 8.356097867 | 9.350454523 |
| ENSMUSG00000029617 | Ccz1 | 1747 | 60.80162272 | 60.21872922 | 56.20965462 | 58.86163541 | 49.3441759 | 49.72292041 | 52.48538739 | 52.07458645 | 61.10786998 | 57.51468154 | 63.30797303 | 53.06801119 | 50.91324948 | 54.3930182 | 58.11141025 | 53.91808484 | 61.97583787 | 52.45859863 |
| ENSMUSG00000029618 | Ocm | 753 | 0.056447777 | 0 | 0 | 0 | 0 | 0 | 0 | 0 | 0 | 0 | 0 | 0 | 0 | 0 | 0 | 0 | 0 | 0 |
| ENSMUSG00000029620 | 1700018F24R | 1114 | 0 | 0 | 0 | 0 | 0 | 0 | 0 | 0 | 0 | 0 | 0 | 0 | 0 | 0 | 0 | 0 | 0 | 0 |
| ENSMUSG00000029621 | Arpc1a | 2462 | 82.5242654 | 76.96333969 | 70.55619314 | 74.31890904 | 75.5018234 | 73.78567071 | 70.41172039 | 74.27507015 | 76.2335306 | 75.41814355 | 61.68829935 | 69.1007878 | 91.33017016 | 93.07681851 | 76.69647874 | 78.47863727 | 65.83343201 | 80.74854253 |
| ENSMUSG00000029622 | Arpc1b | 4835 | 106.3816205 | 118.3003917 | 102.429825 | 115.62234 | 110.7681313 | 114.3966106 | 96.69987449 | 96.33806406 | 97.72320762 | 101.2740545 | 97.73363274 | 95.55041142 | 105.0917783 | 103.954067 | 98.34687829 | 97.40940456 | 93.29104204 | 90.35828864 |
| ENSMUSG00000029623 | Pdap1 | 3486 | 12.92469495 | 12.04080514 | 11.71177843 | 10.5864733 | 10.0240323 | 10.51893943 | 12.4185603 | 10.80255559 | 12.83091111 | 11.06404913 | 10.48417764 | 10.64756616 | 12.97686981 | 12.0573434 | 10.42449004 | 12.45598072 | 9.740992271 | 13.14474639 |
| ENSMUSG00000029624 | Ptcd1 | 3170 | 7.213812209 | 8.154828364 | 7.72145311 | 8.048638747 | 8.639452545 | 7.174985714 | 8.46255172 | 9.277018881 | 9.87436971 | 10.56056761 | 9.811198098 | 7.359920177 | 8.885969957 | 10.21183877 | 10.28089297 | 9.699481938 | 9.176548691 | 5.865324313 |
| ENSMUSG00000029625 | Cpsf4 | 2683 | 3.548699005 | 4.097374242 | 3.461691326 | 3.634015463 | 3.534099502 | 4.247904854 | 5.043394027 | 5.565870049 | 4.774135819 | 3.883826098 | 4.932412784 | 4.972025106 | 5.552828557 | 5.72977746 | 3.839131186 | 4.23865464 | 6.213916941 | 6.150850547 |
| ENSMUSG00000029627 | Zkscan14 | 2668 | 6.627493713 | 6.00581039 | 7.034831251 | 6.198897711 | 6.260036549 | 6.277670146 | 5.887484784 | 6.212707138 | 5.156033083 | 9.066083497 | 9.687500951 | 8.117120346 | 8.024757158 | 7.816115157 | 8.972302838 | 9.070338785 | 7.268780447 | 6.742120652 |
| ENSMUSG00000029629 | Phf14 | 4460 | 8.329489652 | 6.572947738 | 6.648648903 | 5.54700607 | 7.133453261 | 7.021822337 | 6.958994928 | 6.953414967 | 8.800616485 | 5.9625553 | 7.155531898 | 7.921819376 | 7.919653087 | 8.684836072 | 6.355755707 | 8.447972575 | 7.098098933 | 7.597670346 |
| ENSMUSG00000029630 | Cyp3a25 | 2576 | 0 | 0 | 0 | 0 | 0 | 0 | 0 | 0 | 0 | 0 | 0 | 0 | 0 | 0 | 0 | 0 | 0 | 0 |
| ENSMUSG00000029632 | Ndufa4 | 488 | 1007.14621 | 791.317766 | 872.4086899 | 829.9018916 | 937.6848326 | 836.2023791 | 1156.058163 | 913.0958173 | 902.4742071 | 984.0776899 | 975.0790706 | 856.4501669 | 1131.904668 | 1060.79057 | 942.5700204 | 1033.297149 | 970.1736591 | 1008.876035 |
| ENSMUSG00000029633 | Gm5578 | 1235 | 10.15305824 | 13.48700171 | 14.88416319 | 12.83825379 | 14.03035832 | 15.08650026 | 10.11380343 | 12.89573425 | 11.47219548 | 10.65185928 | 14.2357771 | 11.6150812 | 10.06610782 | 10.22898119 | 11.44302322 | 8.960312177 | 15.42361908 | 11.53632279 |
| ENSMUSG00000029634 | Rnf6 | 5847 | 25.87239977 | 29.43294194 | 27.21890112 | 30.21039317 | 28.07901345 | 27.9925909 | 21.43514392 | 25.07621564 | 25.24584787 | 26.68405628 | 28.83492876 | 24.71468282 | 22.88150499 | 21.99481409 | 26.33319897 | 25.86105843 | 28.2645788 | 25.12901044 |
| ENSMUSG00000029635 | Cdk8 | 4416 | 10.03915276 | 9.603105603 | 9.42056977 | 8.233177849 | 8.817646342 | 8.25878893 | 8.571137487 | 8.890803821 | 9.345309962 | 6.790726284 | 7.118641911 | 6.812645411 | 8.802887413 | 7.004475136 | 7.799932229 | 11.28948615 | 10.84002374 | 9.616628259 |
| ENSMUSG00000029636 | Wasf3 | 3986 | 0.074645317 | 0.317584039 | 0.048543432 | 0.171454005 | 0.229429641 | 0.012431694 | 0.023739427 | 0.229959272 | 0.134326318 | 0.094632621 | 0.083899827 | 0.196040962 | 0.27227852 | 0.594241961 | 0.754569388 | 0.509132512 | 0.624989789 | 0.731430671 |

| ENSMUSG00000029638 | Glcci1 |  | 7763 | 0.443503705 | 0.334716701 | 0.373877602 | 0.51647197 | 0.297608184 | 0.223411783 | 0.231596264 | 0.280428563 | 0.153859198 | 0.224729617 | 0.54156851 | 0.35230806 | 0.222416219 | 0.095723998 | 0.318445621 | 0.231825138 | 0.296222828 | 0.403905588 |
| --- | --- | --- | --- | --- | --- | --- | --- | --- | --- | --- | --- | --- | --- | --- | --- | --- | --- | --- | --- | --- | --- |
| ENSMUSG00000029640 | Usp12 |  | 7570 | 3.262286298 | 3.507315283 | 3.335664841 | 2.852831574 | 3.573334359 | 3.626448402 | 2.981261275 | 4.026094732 | 3.068585851 | 3.326086597 | 3.79296566 | 3.362211643 | 2.684907519 | 1.858990552 | 3.107805603 | 2.938816764 | 2.004915928 | 2.572420579 |
| ENSMUSG00000029641 | Rasl11a |  | 1122 | 6.326527983 | 7.571112323 | 3.664661391 | 3.776450199 | 3.860849492 | 3.312348501 | 6.409560782 | 5.990965083 | 7.231500539 | 2.899642179 | 3.789635744 | 7.760466456 | 5.627883611 | 5.753768402 | 4.590191164 | 6.961928618 | 5.465425583 | 7.795408172 |
| ENSMUSG00000029642 | Polr1d |  | 2328 | 41.64703888 | 34.78678945 | 38.46194359 | 34.67962653 | 34.58965133 | 35.5681348 | 40.36210496 | 33.22148405 | 33.08367031 | 33.64145967 | 33.30702647 | 34.33334571 | 39.58420166 | 33.93530612 | 33.06035498 | 32.66544085 | 31.11542145 | 38.208645 |
| ENSMUSG00000029644 | Pdx1 |  | 4316 | 0 | 0 | 0 | 0 | 0 | 0 | 0 | 0 | 0 | 0 | 0 | 0 | 0 | 0 | 0 | 0 | 0 | 0 |
| ENSMUSG00000029646 | Cdx2 |  | 2258 | 0 | 0.088519648 | 0 | 0 | 0 | 0 | 0 | 0 | 0 | 0 | 0 | 0 | 0 | 0 | 0 | 0 | 0 | 0 |
| ENSMUSG00000029647 | Pan3 |  | 7840 | 4.592077053 | 5.451587153 | 4.578209123 | 3.852930152 | 4.070339973 | 3.90607007 | 4.453667991 | 5.29042717 | 4.838364926 | 3.470147421 | 4.363121208 | 5.766669221 | 4.152942016 | 3.471458573 | 3.820603461 | 5.518926832 | 4.898335395 | 5.136054352 |
| ENSMUSG00000029648 | Flt1 |  | 11550 | 0.036801018 | 0.005768467 | 0 | 0.019723396 | 0 | 0.00858056 | 0.008192672 | 0.019840209 | 0.010697796 | 0.053070056 | 0.00413636 | 0.004832525 | 0.038440455 | 0.044232464 | 0.07491192 | 0.066304082 | 0.063047588 | 0 |
| ENSMUSG00000029649 | Pomp |  | 728 | 214.0439221 | 193.4710395 | 202.2651462 | 187.876724 | 198.8747615 | 194.1949848 | 231.8191288 | 221.8097217 | 236.7658907 | 221.6342123 | 212.5591903 | 206.2419401 | 307.9856649 | 277.5161815 | 268.6590464 | 275.9238 | 232.274206 | 282.4506057 |
| ENSMUSG00000029650 | Slc46a3 |  | 3225 | 1.647487443 | 2.85096397 | 3.299899745 | 3.206932423 | 3.104322519 | 2.519890948 | 7.349969657 | 7.76874914 | 5.402138346 | 5.804288122 | 9.258712883 | 5.27869057 | 13.33873856 | 10.08088719 | 11.2936978 | 15.06690877 | 13.16760836 | 9.023199211 |
| ENSMUSG00000029651 | Mtus2 |  | 10159 | 0.748934591 | 0.829625183 | 0.609490293 | 0.632356261 | 0.748581941 | 0.824334282 | 0.908157534 | 0.560160248 | 0.774350282 | 0.733321303 | 0.399731418 | 0.53293821 | 0.674981445 | 0.54860621 | 0.324453634 | 0.75382631 | 0.501761737 | 0.942177829 |
| ENSMUSG00000029655 | N4bp2l2 |  | 12254 | 4.8596174 | 5.121714763 | 5.842404515 | 4.60666998 | 4.941240409 | 4.799991412 | 5.362927292 | 5.850101135 | 5.233181414 | 5.163721542 | 4.740847846 | 6.36318595 | 6.2520375 | 4.771756653 | 6.11601356 | 5.108955696 | 6.28346095 | 5.912109805 |
| ENSMUSG00000029656 | C8b |  | 2623 | 0 | 0 | 0 | 0 | 0 | 0 | 0 | 0 | 0 | 0 | 0 | 0 | 0 | 0 | 0 | 0 | 0 | 0 |
| ENSMUSG00000029657 | Hsph1 |  | 7024 | 4.193634252 | 4.941918533 | 4.641765299 | 4.404320772 | 3.809987124 | 4.747862998 | 2.950306573 | 3.344010176 | 2.990478793 | 4.497575624 | 3.822540812 | 3.091157851 | 2.963848105 | 3.524302189 | 3.625079946 | 3.565213161 | 3.672209256 | 3.297100503 |
| ENSMUSG00000029658 | Wdr95 |  | 3342 | 0 | 0 | 0 | 0 | 0 | 0 | 0 | 0 | 0 | 0 | 0 | 0 | 0 | 0.027794188 | 0 | 0 | 0 | 0 |
| ENSMUSG00000029659 | 6330406I15Ri | k | 5488 | 0.023235337 | 0.018210401 | 0 | 0 | 0 | 0 | 0.008621115 | 0 | 0 | 0 | 0 | 0 | 0 | 0 | 0 | 0 | 0 | 0 |
| ENSMUSG00000029660 | 4930588N13R |  | 1352 | 0 | 0 | 0 | 0 | 0 | 0 | 0 | 0 | 0 | 0 | 0.141346031 | 0 | 0 | 0 | 0 | 0 | 0 | 0 |
| ENSMUSG00000029661 | Col1a2 |  | 11111 | 0 | 0 | 0 | 0 | 0 | 0 | 0 | 0.020624104 | 0 | 0 | 0 | 0 | 0 | 0 | 0 | 0 | 0 | 0 |
| ENSMUSG00000029663 | Gngt1 |  | 3508 | 0 | 0 | 0 | 0 | 0 | 0 | 0 | 0 | 0 | 0 | 0 | 0 | 0 | 0 | 0 | 0 | 0 | 0 |
| ENSMUSG00000029664 | Tfpi2 |  | 1525 | 0 | 0 | 0 | 0 | 0 | 0 | 0 | 0 | 0 | 0.340103433 | 0 | 0 | 0 | 0 | 0 | 0 | 0 | 0 |
| ENSMUSG00000029669 | Tspan12 |  | 2615 | 71.37293611 | 67.19905048 | 69.14732561 | 67.35716918 | 69.85110911 | 72.93631799 | 56.54000828 | 59.92482871 | 49.40806881 | 59.64609039 | 45.76530439 | 53.08357662 | 68.21576018 | 81.18390954 | 79.59856993 | 67.76632163 | 76.03702804 | 68.82975036 |
| ENSMUSG00000029670 | Ing3 |  | 4859 | 6.254620469 | 7.00674581 | 5.585007312 | 6.479251211 | 6.686365194 | 5.884323374 | 5.959119058 | 7.4435491 | 6.586112934 | 7.044949694 | 7.49218324 | 6.605064002 | 6.680469555 | 7.111422313 | 7.283831346 | 8.148279081 | 8.495044611 | 6.328481304 |
| ENSMUSG00000029671 | Wnt16 |  | 4726 | 0 | 0 | 0 | 0 | 0 | 0 | 0 | 0 | 0 | 0 | 0 | 0 | 0 | 0.05896414 | 0 | 0 | 0.008109669 | 0 |
| ENSMUSG00000029672 | Fam3c |  | 4355 | 45.91144616 | 43.76954781 | 42.1199603 | 55.52065724 | 47.00460881 | 53.56929269 | 29.45227843 | 30.9661017 | 27.34103721 | 34.74319328 | 23.79423305 | 31.28496725 | 36.79220834 | 29.5941085 | 26.68876904 | 48.70072891 | 37.61345418 | 34.64752692 |
| ENSMUSG00000029673 | Auts2 |  | 19123 | 3.638601326 | 3.59207174 | 3.569264829 | 3.454662673 | 3.387835029 | 4.029414878 | 3.50088588 | 3.48910354 | 4.413071735 | 3.456847041 | 3.077903511 | 3.420799903 | 3.508414344 | 2.875584347 | 2.779375992 | 3.666271095 | 2.866004527 | 3.080832155 |
| ENSMUSG00000029674 | Limk1 |  | 8482 | 0.581301629 | 1.146824465 | 0.895383667 | 0.768124185 | 0.856864003 | 0.771157844 | 1.054243848 | 1.094170453 | 0.650671199 | 0.756012219 | 0.687166345 | 0.960750617 | 0.878226775 | 0.476377694 | 0.893785226 | 0.17154481 | 0.899190413 | 0.233474162 |
| ENSMUSG00000029675 | Eln |  | 2546 | 0 | 0 | 0 | 0 | 0 | 0 | 0 | 0.015000944 | 0 | 0 | 0 | 0 | 0 | 0 | 0 | 0 | 0.150535337 | 0 |
| ENSMUSG00000029676 | Pot1a |  | 8851 | 3.332797668 | 3.074978486 | 3.426754438 | 3.60329127 | 3.73048239 | 3.168777224 | 3.025531157 | 3.736823012 | 4.811530595 | 3.164333718 | 3.994290959 | 2.793620893 | 2.903844639 | 3.568182061 | 3.975384559 | 3.967064398 | 4.351816562 | 2.88376743 |
| ENSMUSG00000029678 | Hyal5 |  | 2026 | 0 | 0 | 0 | 0 | 0 | 0 | 0 | 0 | 0 | 0 | 0 | 0 | 0 | 0 | 0 | 0 | 0 | 0 |
| ENSMUSG00000029679 | Hyal6 |  | 3672 | 0 | 0 | 0 | 0 | 0 | 0 | 0 | 0 | 0 | 0 | 0 | 0 | 0 | 0 | 0 | 0.15641588 | 0 | 0 |
| ENSMUSG00000029680 | Hyal4 |  | 3264 | 0 | 0 | 0 | 0 | 0 | 0 | 0 | 0 | 0 | 0 | 0 | 0 | 0 | 0 | 0 | 0 | 0 | 0 |
| ENSMUSG00000029681 | Bcl7b |  | 2606 | 33.97478191 | 37.62081646 | 34.50744165 | 33.14801998 | 37.16094797 | 33.29502552 | 33.51466051 | 33.2095103 | 30.55009016 | 30.08887932 | 33.32880833 | 32.29854919 | 36.81910173 | 34.68157869 | 34.10274599 | 35.45481031 | 34.20835249 | 30.56530565 |
| ENSMUSG00000029682 | Spam1 |  | 2010 | 0 | 0 | 0 | 0 | 0 | 0 | 0 | 0 | 0 | 0 | 0 | 0 | 0 | 0 | 0 | 0 | 0 | 0 |
| ENSMUSG00000029683 | Lmod2 |  | 1964 | 0 | 0 | 0 | 0 | 0 | 0 | 0 | 0 | 0 | 0 | 0 | 0 | 0 | 0 | 0 | 0 | 0 | 0 |
| ENSMUSG00000029684 | Wasl |  | 4910 | 23.38217524 | 22.21311928 | 23.21141294 | 22.53926206 | 22.12500035 | 23.21207479 | 19.7248582 | 22.27760499 | 22.31285783 | 22.51902223 | 21.14358142 | 22.78097512 | 21.96325278 | 19.15463889 | 19.88751272 | 22.97436444 | 25.18919758 | 23.4040782 |
| ENSMUSG00000029685 | Asb15 |  | 4487 | 0.530485816 | 0.363791357 | 0.722314806 | 0.477238485 | 0.654345296 | 0.717835454 | 2.203777556 | 3.923935259 | 2.707827535 | 2.479956757 | 2.065598829 | 1.853473083 | 3.133406825 | 3.02243671 | 2.984289205 | 1.706735342 | 2.72478019 | 1.949286376 |
| ENSMUSG00000029686 | Cul1 |  | 4089 | 53.59664651 | 56.98794205 | 58.14524375 | 59.35526612 | 55.42998986 | 58.64164289 | 46.10932392 | 48.10243883 | 59.03501318 | 57.29807887 | 55.36941263 | 49.71402376 | 47.09997678 | 50.55579187 | 52.43651183 | 49.5652201 | 55.22585995 | 51.59205779 |
| ENSMUSG00000029687 | Ezh2 |  | 5116 | 5.583166202 | 5.704084311 | 4.604751619 | 3.954085943 | 4.261876744 | 4.910718515 | 5.132630337 | 4.785248229 | 5.96544335 | 5.548225833 | 5.743080427 | 4.036707419 | 4.792408696 | 4.638961817 | 5.057579574 | 4.864912981 | 5.109174059 | 4.666527036 |
| ENSMUSG00000029695 | Aass |  | 3792 | 2.589318476 | 1.967850291 | 2.194158022 | 4.193250125 | 3.059017301 | 3.097045849 | 3.980154161 | 2.679108397 | 2.161423164 | 4.103304872 | 3.099324838 | 2.369810569 | 4.150021522 | 4.87466951 | 4.248367182 | 6.088933614 | 4.669501363 | 4.33747967 |
| ENSMUSG00000029697 | Fezf1 |  | 2304 | 0 | 0 | 0 | 0 | 0 | 0 | 0 | 0 | 0 | 0 | 0 | 0 | 0 | 0 | 0 | 0 | 0 | 0 |
| ENSMUSG00000029699 | Srcrb4d |  | 3931 | 2.011183602 | 2.118601769 | 2.448825373 | 1.483544562 | 1.603989316 | 1.21014053 | 2.383085838 | 1.554511418 | 2.556476684 | 2.79473769 | 1.336872407 | 2.456400312 | 2.597737694 | 1.004260348 | 2.127681467 | 2.074764532 | 1.618459752 | 3.330492852 |

| ENSMUSG00000029700 | Slc13a1 | 8264 | 0 | 0 | 0 | 0 | 0 | 0 | 0 | 0 | 0 | 0 | 0 | 0 | 0 | 0 | 0 | 0 | 0 | 0 |
| --- | --- | --- | --- | --- | --- | --- | --- | --- | --- | --- | --- | --- | --- | --- | --- | --- | --- | --- | --- | --- |
| ENSMUSG00000029701 | Rbm28 | 6030 | 8.705454792 | 8.463574452 | 5.663633902 | 6.482815264 | 7.104048435 | 6.672772747 | 7.35190384 | 6.618749671 | 6.270185982 | 8.265056939 | 6.163999618 | 5.4982593 | 6.512140324 | 6.361992734 | 6.914755317 | 7.372370679 | 9.158904451 | 7.371037849 |
| ENSMUSG00000029703 | Lrwd1 | 6113 | 3.407089196 | 2.256099821 | 3.133636189 | 2.712942931 | 2.511710069 | 2.788506519 | 3.482857111 | 3.067637752 | 2.89040001 | 2.283102922 | 2.657203645 | 2.994853154 | 2.776080409 | 2.438827425 | 3.120615144 | 2.386507675 | 2.463967722 | 1.745749502 |
| ENSMUSG00000029705 | Cux1 | 13871 | 9.6556708 | 9.596880203 | 9.353169087 | 11.46662863 | 10.70485073 | 11.19232314 | 6.545528863 | 6.806403197 | 7.72302844 | 8.885584191 | 8.01817485 | 7.239014995 | 7.127183492 | 6.519114566 | 8.792199945 | 8.5326413 | 7.554184683 | 7.503224708 |
| ENSMUSG00000029706 | Pax4 | 2012 | 0 | 0 | 0 | 0 | 0 | 0 | 0 | 0 | 0 | 0 | 0 | 0 | 0 | 0 | 0 | 0 | 0 | 0 |
| ENSMUSG00000029707 | Fscn3 | 2075 | 0 | 0.048163221 | 0 | 0 | 0 | 0 | 0 | 0 | 0 | 0 | 0 | 0 | 0 | 0 | 0 | 0 | 0 | 0.18557289 |
| ENSMUSG00000029708 | Gcc1 | 6411 | 4.634396825 | 5.466411592 | 4.202785277 | 4.249805727 | 4.376994462 | 5.518741503 | 3.24716561 | 4.81351757 | 3.295692183 | 4.802590739 | 4.076259911 | 3.848155181 | 4.070594952 | 2.991952596 | 3.843164939 | 4.855757888 | 5.458104675 | 4.96806626 |
| ENSMUSG00000029710 | Ephb4 | 5109 | 4.683972226 | 6.761689431 | 6.410037199 | 8.971307614 | 5.991760316 | 7.55560373 | 5.945339572 | 4.687147447 | 7.013558174 | 6.008046158 | 3.441218383 | 6.117982198 | 2.481562486 | 3.290812221 | 4.266123129 | 2.765557669 | 3.795871253 | 3.197828283 |
| ENSMUSG00000029711 | Epo | 2711 | 0 | 0 | 0 | 0 | 0 | 0 | 0 | 0 | 0 | 0 | 0 | 0 | 0 | 0 | 0 | 0 | 0 | 0 |
| ENSMUSG00000029712 | Actl6b | 1945 | 0 | 0 | 0 | 0 | 0 | 0 | 0 | 0 | 0 | 0 | 0 | 0 | 0 | 0 | 0 | 0 | 0 | 0 |
| ENSMUSG00000029713 | Gnb2 | 3087 | 64.13641397 | 65.09341689 | 66.62917106 | 63.02094568 | 67.6528723 | 61.04601419 | 55.1904622 | 61.60024994 | 67.20326527 | 61.76787949 | 66.88803711 | 66.42913649 | 65.20059037 | 61.6696839 | 65.27917391 | 67.60084559 | 58.63786857 | 65.38011421 |
| ENSMUSG00000029714 | Gigyf1 | 5413 | 5.763679883 | 7.003523669 | 6.729220092 | 5.142767059 | 5.317363819 | 6.316531714 | 5.68136732 | 6.286611961 | 7.030545089 | 6.262951347 | 4.898411594 | 6.393074229 | 5.932953657 | 6.006070787 | 7.649651519 | 6.720132465 | 5.621851051 | 6.331179288 |
| ENSMUSG00000029715 | Pop7 | 1866 | 17.17518903 | 14.40702338 | 15.34680063 | 15.01609982 | 15.14117856 | 12.9856842 | 17.90072418 | 14.14306005 | 16.17884033 | 19.30500391 | 19.25335947 | 16.60112151 | 19.3785077 | 16.30272092 | 16.53803074 | 16.35456275 | 15.13744953 | 19.07336123 |
| ENSMUSG00000029716 | Tfr2 | 2907 | 0 | 0 | 0 | 0 | 0 | 0 | 0 | 0 | 0 | 0 | 0 | 0 | 0 | 0 | 0 | 0 | 0 | 0 |
| ENSMUSG00000029718 | Pcolce | 3737 | 0.068244864 | 0.142629464 | 0.258889646 | 0.195070032 | 0.206077276 | 0.106080243 | 0.468442366 | 0.367922528 | 0.099191502 | 0.201876171 | 0.166196002 | 0.134423592 | 0.422430119 | 0.099425394 | 0.264607263 | 0.092217144 | 0.461515483 | 0.250242123 |
| ENSMUSG00000029720 | Lrch4 | 4442 | 0.526291013 | 0.847446431 | 0.794972471 | 0.728238214 | 0.704316758 | 0.780885041 | 0.873399296 | 1.126340548 | 1.066287554 | 1.050859888 | 0.570029896 | 0.879580455 | 0.77740527 | 0.941010325 | 0.797688835 | 0.827532263 | 0.733393795 | 0.594425287 |
| ENSMUSG00000029722 | Agfg2 | 5126 | 19.8678115 | 16.77992448 | 17.96785053 | 17.75867471 | 16.19734068 | 19.35321354 | 16.30930622 | 15.87003846 | 18.23101917 | 18.39668484 | 12.84313164 | 16.1697735 | 14.97472947 | 13.91691733 | 17.00789939 | 14.88747182 | 14.02655735 | 13.14595313 |
| ENSMUSG00000029723 | Tsc22d4 | 6597 | 11.51383198 | 10.91745904 | 11.47560634 | 11.54738004 | 9.579690128 | 11.65018793 | 9.531385523 | 11.85660757 | 11.51873906 | 11.55717556 | 11.89847761 | 12.67422503 | 12.21894086 | 12.52448559 | 11.11698795 | 14.03466556 | 11.10806119 | 13.84191559 |
| ENSMUSG00000029725 | Ppp1r35 | 1081 | 41.24692845 | 36.61028523 | 37.00731693 | 36.6258534 | 33.57212807 | 36.94681153 | 36.45833631 | 29.00644068 | 31.24231538 | 35.54840255 | 31.42275252 | 34.90415163 | 33.95277193 | 32.9104264 | 27.02303712 | 34.11087412 | 24.35723032 | 30.37968244 |
| ENSMUSG00000029726 | Mepce | 3344 | 12.57105834 | 10.70913914 | 9.142365785 | 9.646297664 | 8.794439402 | 9.380047953 | 9.507811076 | 12.76887138 | 12.31654179 | 11.0544711 | 12.27233533 | 11.65051809 | 14.39831078 | 11.027693 | 10.42359937 | 13.98110994 | 10.72769554 | 11.64666494 |
| ENSMUSG00000029727 | Cyp3a13 | 2943 | 1.285409673 | 1.743182368 | 2.071037997 | 1.238492541 | 1.422860129 | 2.222548703 | 0 | 0 | 0 | 0.016021306 | 0 | 0 | 0 | 0 | 0.013999849 | 0 | 0 | 0 |
| ENSMUSG00000029729 | Zkscan1 | 8646 | 11.76438656 | 13.68964991 | 13.59561401 | 12.21495498 | 12.93761168 | 13.47426285 | 12.36718186 | 13.88814633 | 12.01868833 | 12.74475983 | 12.27251709 | 11.94297661 | 9.380253528 | 11.13025088 | 12.0707311 | 9.530579127 | 12.30111889 | 9.664449816 |
| ENSMUSG00000029730 | Mcm7 | 4644 | 4.649575672 | 6.334040826 | 6.208144723 | 5.984547167 | 4.498116897 | 5.196421458 | 5.623729278 | 5.921302698 | 4.71817967 | 4.761774295 | 5.205454132 | 5.588777585 | 5.406965093 | 4.84042599 | 4.844110576 | 5.994244526 | 4.514316194 | 3.482488669 |
| ENSMUSG00000029735 | Tpk1 | 5945 | 7.342778096 | 8.057853976 | 8.85288495 | 8.660047115 | 8.66293664 | 8.843641771 | 10.11512446 | 10.61292662 | 10.0039241 | 9.96945904 | 11.18633174 | 9.82055224 | 8.754444529 | 7.093562885 | 9.820454953 | 11.03954602 | 9.625090206 | 6.856475708 |
| ENSMUSG00000029736 | Nobox | 2259 | 0 | 0 | 0.10706846 | 0 | 0.127840435 | 0 | 0 | 0 | 0 | 0 | 0.16918976 | 0 | 0 | 0 | 0 | 0.220353208 | 0.780438095 | 0 |
| ENSMUSG00000029752 | Asns | 2776 | 11.8665387 | 10.64428572 | 10.83873771 | 10.66811197 | 11.72088631 | 9.228661117 | 8.675127368 | 9.03905201 | 8.634925135 | 8.288740342 | 8.260799737 | 8.344200279 | 12.20858327 | 13.51830786 | 11.87364723 | 8.63469749 | 8.642745627 | 7.17337775 |
| ENSMUSG00000029754 | Dlx6 | 1804 | 0 | 0.110796766 | 0 | 0 | 0 | 0 | 0 | 0 | 0 | 0 | 0 | 0 | 0 | 0 | 0 | 0 | 0 | 0 |
| ENSMUSG00000029755 | Dlx5 | 1420 | 0.478931561 | 0.797632678 | 0.068131733 | 0.866301547 | 0.135582884 | 0.732822116 | 0.133275152 | 0 | 0 | 0 | 0 | 0 | 0 | 0 | 0 | 0.026965216 | 0.18893245 | 0 |
| ENSMUSG00000029757 | Dync1i1 | 3123 | 0.027220734 | 0 | 0.061957772 | 0 | 0.077060397 | 0 | 0 | 0.036688187 | 0 | 0.226468315 | 0 | 0 | 0.031592646 | 0 | 0 | 0 | 0 | 0 |
| ENSMUSG00000029759 | Pon3 | 3095 | 10.69839487 | 10.01001345 | 10.47181433 | 11.29089532 | 10.21734212 | 11.70373126 | 3.256090666 | 2.35694632 | 2.288879067 | 3.793384527 | 5.603331156 | 2.434608869 | 2.3271276 | 2.430999081 | 2.103342756 | 2.894992611 | 2.315676097 | 2.55938978 |
| ENSMUSG00000029761 | Cald1 | 7452 | 1.289072703 | 2.047410836 | 1.213882203 | 1.192217344 | 1.705156716 | 1.436311118 | 0.266657611 | 0.471511141 | 0.386883535 | 0.240435685 | 0.1025764 | 0.763982499 | 0.304518008 | 0.604545958 | 0.414669443 | 0.673117226 | 0.498879603 | 0.125490447 |
| ENSMUSG00000029762 | Akr1b8 | 1489 | 9.848412176 | 11.43243045 | 12.31267159 | 13.18791814 | 10.44097471 | 8.81898885 | 8.801619921 | 7.771858918 | 10.04076912 | 6.33320393 | 6.737905494 | 6.409992144 | 6.062955582 | 4.990633971 | 6.087536801 | 10.56913341 | 8.236678985 | 8.349266702 |
| ENSMUSG00000029763 | Exoc4 | 12725 | 4.345715836 | 4.340493408 | 4.637776584 | 4.178368467 | 4.951252501 | 5.163609016 | 3.785014314 | 3.8507546 | 3.427624377 | 3.716475864 | 4.494037352 | 4.789839178 | 4.38075179 | 4.638933994 | 4.532980609 | 3.917828749 | 5.123224443 | 5.356092985 |
| ENSMUSG00000029765 | Plxna4 | 17206 | 1.704554891 | 1.90127061 | 1.425396948 | 2.46261602 | 1.96656645 | 1.537903041 | 3.445471736 | 4.532656382 | 3.698312615 | 5.609525141 | 4.709190374 | 4.139299399 | 1.103846806 | 0.736907815 | 1.257167084 | 0.836758595 | 1.24962528 | 1.074221775 |
| ENSMUSG00000029766 | 1700012A03R | 1107 | 0 | 0 | 0 | 0 | 0 | 0 | 0 | 0 | 0 | 0 | 0 | 0 | 0 | 0 | 0 | 0 | 0 | 0 |
| ENSMUSG00000029767 | Calu | 4143 | 66.14310158 | 69.55262732 | 67.59217167 | 72.38300599 | 74.39943016 | 75.00487383 | 46.52470527 | 55.49559799 | 47.59860909 | 53.50119624 | 59.82536437 | 52.97301117 | 47.11716067 | 48.33862052 | 55.72105181 | 46.96907237 | 57.2165451 | 56.15098053 |
| ENSMUSG00000029769 | Ccdc136 | 4132 | 0.555488748 | 0.741719813 | 1.030220395 | 0.231554194 | 0.722211827 | 0.803493018 | 1.019077547 | 1.062954084 | 0.29903085 | 0.467855478 | 0.94809937 | 1.134684331 | 1.516252056 | 0.449603944 | 1.116789511 | 0.59307814 | 0.946099294 | 0.971845303 |
| ENSMUSG00000029771 | Irf5 | 2890 | 1.088367829 | 1.590719516 | 1.238630189 | 2.317464885 | 1.365680879 | 1.474579615 | 2.226479008 | 3.092395212 | 1.923937588 | 3.21407908 | 1.289427945 | 0.984982281 | 4.762493052 | 6.830012851 | 6.885934773 | 7.260641127 | 5.211845911 | 4.815676125 |
| ENSMUSG00000029772 | Ahcyl2 | 11642 | 5.494785254 | 7.170770736 | 6.598279179 | 6.895598705 | 6.056800221 | 6.537794088 | 5.604207545 | 5.918149282 | 7.988245369 | 5.447319694 | 5.330670938 | 5.283358544 | 5.067941338 | 5.146269776 | 6.108390781 | 6.584589935 | 7.01869653 | 6.260699743 |
| ENSMUSG00000029775 | Klhdc10 | 8687 | 6.884399985 | 7.305291066 | 7.907265236 | 7.67829733 | 7.058831674 | 7.757766509 | 7.793765805 | 6.656302203 | 5.684658962 | 7.566257666 | 8.397877472 | 5.956155084 | 5.196121136 | 6.03072759 | 5.677249039 | 4.81773154 | 6.313448932 | 7.16821789 |
| ENSMUSG00000029776 | Hibadh | 3040 | 47.7903591 | 47.20787773 | 45.19106125 | 47.53935284 | 45.80449183 | 45.96668049 | 68.43218725 | 52.17534447 | 59.51722492 | 62.25754042 | 49.03219423 | 52.93307738 | 63.61220912 | 57.9939986 | 53.42649122 | 53.49349001 | 58.64932013 | 56.5290952 |

| ENSMUSG00000029777 | Gars |  | 2373 | 57.21092804 | 65.41849434 | 60.60451148 | 58.42488745 | 62.18814916 | 65.44385463 | 42.36807531 | 44.98430877 | 40.73525179 | 41.52758947 | 50.23115103 | 42.59678253 | 55.85961303 | 54.21412645 | 60.45672887 | 64.41470912 | 62.87580019 | 52.3664442 |
| --- | --- | --- | --- | --- | --- | --- | --- | --- | --- | --- | --- | --- | --- | --- | --- | --- | --- | --- | --- | --- | --- |
| ENSMUSG00000029778 | Adcyap1r1 |  | 6923 | 0 | 0 | 0 | 0 | 0 | 0 | 0 | 0 | 0 | 0 | 0 | 0 | 0 | 0 | 0 | 0 | 0 | 0 |
| ENSMUSG00000029780 | Nt5c3 |  | 4792 | 28.63245163 | 25.54776424 | 26.41768133 | 29.76870405 | 24.64852685 | 27.35120624 | 24.39683423 | 25.26500743 | 28.10515579 | 27.00932397 | 23.41890181 | 25.75301126 | 23.64678923 | 23.86171602 | 25.32967105 | 20.12813857 | 21.41857744 | 23.37198274 |
| ENSMUSG00000029781 | Fkbp9 |  | 3304 | 16.9428925 | 20.23576837 | 19.3991912 | 17.94035069 | 19.94328193 | 24.34143057 | 17.15514206 | 17.01550131 | 16.75383692 | 17.95265881 | 18.19034436 | 14.73101022 | 14.18441931 | 12.52472211 | 15.62516632 | 9.595830184 | 13.47916374 | 17.03217802 |
| ENSMUSG00000029782 | Tmem209 |  | 10157 | 4.268512312 | 5.201757431 | 5.367428248 | 5.378329444 | 5.914014053 | 5.107975983 | 4.383305196 | 4.610011357 | 5.340419539 | 4.72110517 | 6.349925563 | 4.764416606 | 3.530993784 | 4.078775815 | 5.455950839 | 3.558760794 | 5.260102184 | 6.157857078 |
| ENSMUSG00000029784 | 1700025E21R |  | 1802 | 0 | 0 | 0 | 0 | 0.053420559 | 0 | 0.105022595 | 0.445083491 | 0 | 0.156994572 | 0.106048742 | 0 | 0 | 0.644340835 | 0 | 0 | 0 | 0 |
| ENSMUSG00000029787 | Avl9 |  | 7723 | 10.56712909 | 14.16542077 | 12.25153766 | 14.08186104 | 13.60507437 | 16.23954016 | 10.9414016 | 12.89729192 | 11.80719227 | 12.61951361 | 13.02784702 | 13.64495286 | 8.840524862 | 9.333319128 | 13.97747979 | 12.80154711 | 15.36922718 | 11.45340459 |
| ENSMUSG00000029788 | Cpa5 |  | 2828 | 0 | 0 | 0 | 0 | 0 | 0 | 0 | 0 | 0 | 0 | 0 | 0 | 0 | 0 | 0 | 0 | 0 | 0 |
| ENSMUSG00000029790 | Tsga14 |  | 7020 | 0.865846178 | 1.048739262 | 1.564215298 | 1.349956869 | 1.083311102 | 1.136465827 | 1.5703496 | 1.137067251 | 1.149936907 | 1.101526401 | 1.680970761 | 1.685601206 | 0.843280637 | 1.402584975 | 1.831180262 | 1.243626565 | 1.523224621 | 1.817964781 |
| ENSMUSG00000029797 | Sspo |  | 15614 | 0 | 0 | 0.049569392 | 0 | 0 | 0 | 0 | 0 | 0 | 0.003019771 | 0 | 0.046471347 | 0 | 0.02082161 | 0.094995261 | 0 | 0 | 0.017615315 |
| ENSMUSG00000029798 | Herc6 |  | 5381 | 4.605188185 | 2.705395795 | 3.838598302 | 2.912655501 | 3.792591644 | 4.724131635 | 2.171758353 | 2.044120404 | 2.158445911 | 2.120511092 | 2.78783441 | 2.665791727 | 2.585318839 | 2.882795984 | 2.33534186 | 4.788994384 | 3.42593362 | 2.760167035 |
| ENSMUSG00000029802 | Abcg2 |  | 4967 | 0.376530651 | 0.248153229 | 0.068172884 | 0.165109482 | 0.387613639 | 0.359150072 | 0.123830245 | 0 | 0.033168122 | 0.12340631 | 0.009618474 | 0 | 0 | 0.074804248 | 0.107835761 | 0 | 0 | 0.143973905 |
| ENSMUSG00000029804 | Herc3 |  | 7189 | 8.123815811 | 8.95263759 | 9.117559275 | 9.183190046 | 9.098801539 | 10.14628235 | 5.883646538 | 5.928880384 | 4.938486102 | 7.122779766 | 7.230373463 | 7.189498338 | 4.776048745 | 6.873905826 | 6.88889556 | 7.222431998 | 7.740963013 | 7.162126011 |
| ENSMUSG00000029810 | Tmem176b |  | 2363 | 823.3016956 | 722.9306879 | 764.6620734 | 735.6286604 | 768.3386765 | 780.2615789 | 676.8736453 | 656.8511357 | 684.2051106 | 673.0398734 | 678.028357 | 698.2036269 | 685.0101014 | 557.5649052 | 637.8307728 | 646.954084 | 635.765631 | 630.6143428 |
| ENSMUSG00000029811 | Abp1 |  | 3127 | 16.47466369 | 17.73775787 | 25.29284366 | 24.27396862 | 20.68733783 | 26.4957373 | 10.74256541 | 14.85192239 | 15.34451259 | 13.66118873 | 17.60049638 | 19.15260824 | 10.03361029 | 6.832197058 | 10.55402823 | 12.80843471 | 10.82255202 | 12.03269236 |
| ENSMUSG00000029813 | 1600015I10Ri | k | 4240 | 0 | 0 | 0 | 0 | 0.045407475 | 0.058434827 | 0.145062459 | 0 | 0.155420814 | 0.088963591 | 0.10140911 | 0.039492214 | 0.023269772 | 0 | 0.077738785 | 0 | 0.018078442 | 0 |
| ENSMUSG00000029814 | Igf2bp3 |  | 4329 | 0.049093527 | 0.015390573 | 0 | 0.063147671 | 0.055592427 | 0.022893386 | 0.054646199 | 0.097046992 | 0.209310082 | 0.043567293 | 0.022072053 | 0.064467155 | 0 | 0.010728595 | 0.028552707 | 0.061915974 | 0.035413534 | 0 |
| ENSMUSG00000029815 | 2410003K15R |  | 3330 | 11.61552858 | 11.49444909 | 9.4422807 | 8.742794994 | 10.33463226 | 10.53553615 | 11.40903339 | 12.94871541 | 12.2322715 | 11.96466794 | 10.41580176 | 11.16313252 | 13.05147721 | 12.55245605 | 12.84300751 | 11.57917167 | 10.5195902 | 10.68795557 |
| ENSMUSG00000029816 | Gpnmb |  | 4714 | 1.983695105 | 2.162440736 | 2.021549741 | 4.455587917 | 3.400070128 | 2.596420279 | 2.107692528 | 1.450241839 | 1.799835012 | 3.740849177 | 2.006669872 | 1.977347402 | 20.51136145 | 17.44855298 | 12.37619922 | 5.16606413 | 7.105893825 | 11.13251757 |
| ENSMUSG00000029817 | Tra2a |  | 3862 | 10.29061098 | 8.824207868 | 9.907680609 | 8.635599289 | 9.534158883 | 8.070603164 | 8.992104177 | 8.257549458 | 10.22730933 | 7.972400111 | 7.3728315 | 7.457504375 | 10.75543095 | 9.813147413 | 9.174867443 | 11.51097752 | 10.37052829 | 11.11007334 |
| ENSMUSG00000029819 | Npy |  | 562 | 5.672398936 | 2.430300112 | 1.807551846 | 1.053903161 | 0.513863954 | 2.027958848 | 294.2309837 | 281.2782092 | 248.8779493 | 99.41918747 | 44.03457027 | 191.2828579 | 291.4269846 | 585.3442544 | 124.4844162 | 55.18753024 | 49.30589441 | 204.8648761 |
| ENSMUSG00000029821 | Dfna5 |  | 7363 | 0.837056977 | 1.040603785 | 0.893494517 | 0.693037752 | 0.621014905 | 0.800865856 | 0.758236604 | 1.286393562 | 0.889400517 | 0.665988475 | 0.596943662 | 0.621605912 | 0.663297543 | 1.387708709 | 0.895321057 | 1.773339281 | 1.707323833 | 0.978703379 |
| ENSMUSG00000029822 | Osbpl3 |  | 12834 | 1.745381624 | 2.162197357 | 1.616974016 | 0.802306064 | 1.057597201 | 2.146744575 | 0.921627687 | 2.312256247 | 1.700861256 | 0.45923624 | 1.086978952 | 2.06579708 | 1.026306834 | 1.360680759 | 1.415761738 | 0.566870454 | 0.895892868 | 0.818664437 |
| ENSMUSG00000029823 | Luc7l2 |  | 12318 | 3.354037271 | 2.369061157 | 2.297330351 | 2.352396834 | 2.391357143 | 2.594699883 | 2.984409119 | 3.333076196 | 3.019274536 | 2.912947327 | 3.30445402 | 3.992011588 | 3.888723143 | 3.08043671 | 3.070549465 | 4.137424781 | 3.33542704 | 3.143887898 |
| ENSMUSG00000029826 | Zc3hav1 |  | 6505 | 8.30500826 | 7.620228532 | 6.759652435 | 7.823472179 | 6.910871131 | 7.526226099 | 5.840442918 | 7.432987155 | 4.659996249 | 6.2191089 | 6.110494305 | 6.409577241 | 6.575061073 | 6.782764492 | 6.910207145 | 7.263737054 | 6.911106267 | 7.898309866 |
| ENSMUSG00000029828 | 4921507P07R |  | 2368 | 0.538494629 | 0.534581917 | 0.204280111 | 0.250123977 | 0.467497569 | 0.272037811 | 0.079920066 | 0.225799676 | 0.12175068 | 0.418143906 | 0.564906604 | 0.494986873 | 0.270825559 | 0.706075653 | 0.156593751 | 0.565950701 | 1.132956412 | 1.27766084 |
| ENSMUSG00000029829 | Tmem213 |  | 527 | 97.91514933 | 64.0340832 | 71.3211254 | 79.537307 | 87.9526423 | 73.05972294 | 47.67178845 | 33.40929319 | 45.48491874 | 58.15551635 | 58.6535069 | 44.05942246 | 20.59396926 | 14.18880089 | 10.94538487 | 11.47991645 | 12.65422327 | 15.44847779 |
| ENSMUSG00000029830 | Svopl |  | 4276 | 211.5716012 | 260.6211998 | 231.5390659 | 229.9681992 | 237.1026287 | 261.1832342 | 111.7203272 | 139.952468 | 124.416797 | 122.3095418 | 126.4986155 | 138.220779 | 71.96737605 | 71.18675134 | 95.70978828 | 63.87439256 | 92.84894986 | 91.12008955 |
| ENSMUSG00000029831 | Npvf |  | 809 | 0 | 0 | 0 | 0 | 0 | 0 | 0 | 0 | 0 | 0 | 0 | 0 | 0 | 0 | 0 | 0 | 0 | 0 |
| ENSMUSG00000029832 | Nfe2l3 |  | 3583 | 4.828246342 | 4.862585453 | 4.455279101 | 5.467833956 | 5.064397215 | 5.324533192 | 2.931458198 | 4.349009261 | 4.896861768 | 3.224092185 | 1.600054429 | 3.738699141 | 1.376832746 | 2.773942144 | 1.920362778 | 3.793794482 | 2.128644455 | 1.642750326 |
| ENSMUSG00000029833 | Trim24 |  | 7654 | 2.360164595 | 2.863846929 | 2.926175144 | 2.672708245 | 2.244982591 | 2.686749992 | 1.712256606 | 1.936066388 | 2.007129655 | 2.137613539 | 2.215849263 | 2.267921487 | 3.100163602 | 3.228149267 | 2.002479591 | 3.276763502 | 2.258317207 | 2.989781604 |
| ENSMUSG00000029836 | Cbx3 |  | 2279 | 3.786112652 | 3.91744434 | 3.863090182 | 4.738028744 | 5.195459948 | 4.413867888 | 4.816385042 | 4.893453928 | 4.265036297 | 6.806749176 | 4.318403443 | 4.432924497 | 4.069504365 | 4.829859022 | 4.393145275 | 4.771624617 | 4.456546152 | 3.717157716 |
| ENSMUSG00000029837 | 1700111E14R |  | 896 | 0.56926575 | 0.557693542 | 0.161964945 | 0.355945659 | 0.429749318 | 0.276521951 | 0.105608658 | 0.511505389 | 0.045967093 | 0.210494211 | 0.159960798 | 0.186882799 | 0.110115887 | 0.362844433 | 0 | 0.256410317 | 0.128324655 | 0 |
| ENSMUSG00000029838 | Ptn |  | 3844 | 0.011057538 | 0 | 0 | 0 | 0 | 0 | 0.036924567 | 0.109291474 | 0.096430448 | 0.049064207 | 0 | 0.130681832 | 0.089834397 | 0.120822288 | 0.139339289 | 0.109572498 | 0.039881683 | 0.114483052 |
| ENSMUSG00000029840 | Mtpn |  | 3956 | 73.78236698 | 79.97284034 | 80.72852563 | 80.1464377 | 81.3838315 | 87.9573547 | 78.43188787 | 79.09766251 | 73.02381738 | 80.58288795 | 73.77583957 | 76.3867536 | 78.33748846 | 65.86231806 | 72.91508918 | 76.63928954 | 87.60988445 | 72.68265946 |
| ENSMUSG00000029843 | Slc13a4 |  | 4512 | 0.301455149 | 0.531588738 | 0.171537342 | 0.686646945 | 0.810732757 | 0.691893222 | 0.314578982 | 0.694099512 | 0.565949463 | 0.344852218 | 0.974134792 | 0.296891822 | 0.721610492 | 1.585192699 | 1.187101566 | 0.899557709 | 1.112753744 | 0.658353671 |
| ENSMUSG00000029844 | Hoxa1 |  | 3966 | 0.11789131 | 0.117594685 | 0.134167633 | 0.206782349 | 0 | 0.012494386 | 0 | 0.231118925 | 0.041539602 | 0 | 0.096369054 | 0.014073541 | 0.111948375 | 0.105395055 | 0.186996472 | 0.028964151 | 0.29957519 | 0 |
| ENSMUSG00000029847 | 2010107G12R |  | 3384 | 0.037681894 | 0.157507774 | 0.228716456 | 0.040390997 | 0.056893527 | 0.058572971 | 0.754989557 | 0.688456426 | 0.705915459 | 0.167201075 | 0.12706106 | 0.082469951 | 0.422761702 | 0.576433709 | 0.450489825 | 0.067891148 | 0.305794922 | 0.34136857 |
| ENSMUSG00000029848 | Stra8 |  | 1791 | 0 | 0 | 0 | 0 | 0 | 0 | 0 | 0 | 0 | 0.131632337 | 0 | 0 | 0 | 0 | 0 | 0 | 0 | 0 |
| ENSMUSG00000029851 | Fam115c |  | 4768 | 1.667044446 | 1.425300004 | 1.318909175 | 1.586227634 | 1.796871311 | 1.548522924 | 1.399137527 | 2.362994696 | 1.779450975 | 1.31523564 | 2.234441221 | 1.66229532 | 2.876315647 | 2.259863313 | 2.289935468 | 2.160271265 | 2.178361254 | 1.926703193 |
| ENSMUSG00000029859 | Epha1 |  | 3255 | 4.844675977 | 5.874531884 | 5.602709967 | 5.920836176 | 4.081232237 | 5.541380959 | 4.128049406 | 5.420857116 | 4.605803913 | 4.113916966 | 3.713383869 | 4.95567634 | 4.971081065 | 3.481522987 | 5.240382224 | 1.741016865 | 2.201848701 | 4.258769553 |

| ENSMUSG00000029860 | Zyx |  | 3977 | 17.0576467 | 23.94809268 | 16.33534103 | 20.65540934 | 20.99796016 | 20.20984005 | 7.530531999 | 10.82294129 | 5.675184973 | 6.92381461 | 6.126534781 | 6.820822735 | 4.664018329 | 4.939866474 | 5.532217965 | 6.989937383 | 5.974931878 | 5.422069358 |
| --- | --- | --- | --- | --- | --- | --- | --- | --- | --- | --- | --- | --- | --- | --- | --- | --- | --- | --- | --- | --- | --- |
| ENSMUSG00000029861 | Fam131b |  | 7364 | 0 | 0 | 0 | 0 | 0 | 0 | 0 | 0 | 0 | 0 | 0 | 0.015159061 | 0 | 0 | 0 | 0 | 0 | 0 |
| ENSMUSG00000029862 | Clcn1 |  | 5582 | 0.068532172 | 0.059679137 | 0 | 0.016324272 | 0.060358915 | 0 | 0 | 0.027368257 | 0 | 0.016893839 | 0 | 0 | 0.070701422 | 0 | 0 | 0.006859657 | 0.102990766 | 0 |
| ENSMUSG00000029863 | Casp2 |  | 4528 | 6.918355727 | 8.843219711 | 7.456873716 | 6.751647692 | 6.633021283 | 7.376003187 | 5.851391387 | 6.486298023 | 6.821971471 | 5.91466419 | 5.148891285 | 7.950773491 | 6.482440543 | 5.282399515 | 6.533285585 | 5.276797486 | 6.652930514 | 5.29681328 |
| ENSMUSG00000029864 | Gstk1 |  | 922 | 25.86269387 | 26.84542343 | 23.55717476 | 24.16415475 | 26.78056057 | 27.03364966 | 34.63780724 | 32.26885192 | 27.15987157 | 30.47919647 | 41.19424294 | 29.23965838 | 32.69175863 | 27.25189943 | 24.39918603 | 28.28189115 | 26.3545252 | 21.23995878 |
| ENSMUSG00000029865 | Sval1 |  | 611 | 0 | 0 | 0 | 0 | 0 | 0 | 0 | 0 | 0 | 0 | 0 | 0 | 0 | 0 | 0 | 0 | 0 | 0 |
| ENSMUSG00000029866 | Kel |  | 3007 | 0.169624913 | 0 | 0 | 0 | 0 | 0 | 0 | 0 | 0 | 0 | 0 | 0 | 0.13124554 | 0.293461144 | 0.082211285 | 0 | 0 | 0 |
| ENSMUSG00000029867 | 1700034O15R |  | 739 | 0 | 0 | 0 | 0 | 0.065131155 | 0 | 0 | 0 | 0 | 0 | 0 | 0.151057274 | 0.333774812 | 0.628472089 | 0.05575312 | 0 | 0.103724755 | 0 |
| ENSMUSG00000029868 | Trpv6 |  | 2917 | 3.307739101 | 2.580978435 | 0.729665868 | 1.077720966 | 0.825024403 | 1.851644827 | 3.649418155 | 2.697166574 | 1.17191663 | 1.34162097 | 0.491343419 | 1.836922732 | 10.82359515 | 16.54282029 | 6.582079206 | 1.233910557 | 4.16504495 | 6.355183053 |
| ENSMUSG00000029869 | Ephb6 |  | 3705 | 2.61570314 | 5.331861342 | 1.527585169 | 2.607001727 | 2.546250213 | 3.945494306 | 0.127699538 | 0.886517303 | 1.100530381 | 0.699942963 | 0.696315184 | 0.873767457 | 2.410007295 | 2.068360167 | 3.358399428 | 4.495658359 | 3.175755236 | 3.117925077 |
| ENSMUSG00000029875 | AI836003 |  | 2328 | 0.109549423 | 0.071548312 | 0 | 0.058712686 | 0 | 0 | 0 | 0.131245369 | 0 | 0 | 0 | 0 | 0 | 0.099751047 | 0 | 0 | 0.230484603 | 0 |
| ENSMUSG00000029878 | Dbpht2 |  | 2994 | 0 | 0.011126551 | 0.080784119 | 0.01521745 | 0 | 0.066202717 | 0 | 0.03826894 | 0 | 0 | 0 | 0 | 0 | 0.015512387 | 0 | 0 | 0.064005172 | 0 |
| ENSMUSG00000029882 | 2210010C04R |  | 2137 | 0 | 0 | 0 | 0 | 0 | 0 | 0 | 0 | 0 | 0 | 0 | 0 | 0 | 0 | 0 | 0 | 0 | 0 |
| ENSMUSG00000029883 | 1700074P13R |  | 2518 | 0 | 0 | 0 | 0 | 0 | 0 | 0 | 0 | 0 | 0 | 0 | 0 | 0 | 0 | 0 | 0 | 0 | 0 |
| ENSMUSG00000029885 | Moxd2 |  | 1847 | 0 | 0 | 0 | 0 | 0 | 0 | 0 | 0 | 0 | 0 | 0 | 0 | 0 | 0 | 0 | 0 | 0 | 0 |
| ENSMUSG00000029909 | Prss37 |  | 1117 | 0 | 0.02982354 | 0 | 0 | 0 | 0 | 0 | 0 | 0 | 0 | 0 | 0 | 0 | 0 | 0 | 0 | 0 | 0 |
| ENSMUSG00000029910 | Mad2l1 |  | 2126 | 7.917238809 | 5.813303742 | 6.507445758 | 5.850500989 | 7.697485441 | 6.596154093 | 8.634675176 | 8.030105303 | 10.88749852 | 7.629276538 | 8.898816349 | 5.434544758 | 6.636372691 | 8.891224632 | 9.282946976 | 7.384359847 | 9.644670199 | 6.183996475 |
| ENSMUSG00000029911 | Ssbp1 |  | 3116 | 29.24618017 | 27.02663562 | 26.71721622 | 27.89809775 | 28.76175926 | 26.31892621 | 36.33480124 | 33.51027862 | 30.28187022 | 31.92810779 | 34.05269024 | 31.25748755 | 31.99608627 | 29.75044879 | 34.74893738 | 31.88835886 | 31.25388974 | 34.88382378 |
| ENSMUSG00000029913 | Prdm5 |  | 3867 | 0.054958852 | 0.08614661 | 0.112583857 | 0.023564026 | 0.062234191 | 0 | 0.08564488 | 0.04938247 | 0.426030678 | 0.134124059 | 0.074127166 | 1.053670382 | 0.012757155 | 0.636549426 | 0.564696784 | 0.415879367 | 0.386533638 | 0.583235939 |
| ENSMUSG00000029915 | Clec5a |  | 5196 | 0 | 0 | 0 | 0 | 0 | 0 | 0 | 0 | 0 | 0 | 0 | 0 | 0 | 0 | 0 | 0 | 0 | 0 |
| ENSMUSG00000029916 | Agk |  | 2209 | 6.715394492 | 6.560031229 | 8.562268162 | 7.610695047 | 8.824549156 | 6.998846934 | 8.802856967 | 9.180699718 | 9.117340972 | 8.0043068 | 9.559317179 | 8.237168856 | 6.275359328 | 8.094601016 | 8.132131448 | 8.493615947 | 5.881672544 | 9.06442501 |
| ENSMUSG00000029917 | C130060K24R |  | 2629 | 0 | 0 | 0 | 0.01733018 | 0.018308073 | 0 | 0.017996455 | 0 | 0 | 0 | 0 | 0 | 0 | 0 | 0.156719497 | 0.014564704 | 0 | 0 |
| ENSMUSG00000029918 | Mrps33 |  | 1585 | 13.03313284 | 9.710130681 | 11.23120452 | 10.14703385 | 12.29869343 | 9.973073824 | 12.47741312 | 11.20471112 | 13.14850282 | 11.72074264 | 10.67024309 | 11.69135645 | 13.32117388 | 13.21532076 | 11.69760262 | 10.58125302 | 11.09890869 | 13.63948198 |
| ENSMUSG00000029919 | Hpgds |  | 3580 | 0.023745908 | 0.158189721 | 0 | 0.458155753 | 0 | 0.041524637 | 0 | 0.042673075 | 0 | 0.079023525 | 0 | 0 | 0 | 0 | 0 | 0 | 0.032117009 | 0 |
| ENSMUSG00000029920 | Smarcad1 |  | 6578 | 7.967297361 | 8.887827509 | 8.221588163 | 7.46652566 | 8.956137818 | 7.766626379 | 7.228525741 | 7.902076569 | 7.926745052 | 6.013900887 | 7.146634105 | 8.697332647 | 6.937066507 | 6.862823494 | 6.225957214 | 7.386862389 | 7.632631338 | 7.058024639 |
| ENSMUSG00000029922 | Mkrn1 |  | 4494 | 37.18020627 | 39.36169744 | 40.23592712 | 41.46521391 | 41.80215801 | 42.87072278 | 42.04869596 | 48.16118034 | 38.90448585 | 42.06212046 | 43.42694824 | 41.39599542 | 44.00793421 | 52.02481885 | 48.48104752 | 41.04269156 | 43.60533068 | 42.69509663 |
| ENSMUSG00000029923 | Rab19 |  | 1571 | 16.69363056 | 16.11572222 | 12.62453688 | 13.57260901 | 12.95977321 | 12.42761109 | 9.095117146 | 12.71459354 | 14.05217852 | 14.82651013 | 12.7420163 | 13.39433787 | 11.27317524 | 12.94876529 | 13.82127305 | 13.79534296 | 10.58791398 | 11.90521724 |
| ENSMUSG00000029924 | Slc37a3 |  | 5348 | 12.50198988 | 12.20268507 | 13.05216984 | 14.71277554 | 12.11398079 | 12.08241671 | 7.91788475 | 9.998011101 | 6.900358662 | 10.76496049 | 9.558564991 | 9.351315198 | 11.76106854 | 8.580171717 | 9.776509803 | 8.699156319 | 7.338468218 | 10.68707216 |
| ENSMUSG00000029925 | Tbxas1 |  | 9044 | 0 | 0.125236444 | 0.074881626 | 0 | 0.085151568 | 0.032874436 | 0.020925555 | 0.07179023 | 0.091080309 | 0.005213479 | 0.073955044 | 0.074058818 | 0.010909314 | 0.015406044 | 0.013667035 | 0.033870506 | 0.016951038 | 0 |
| ENSMUSG00000029992 | Gfpt1 |  | 7553 | 4.980415833 | 4.520815116 | 6.154767996 | 5.422928486 | 5.429418637 | 5.399430655 | 4.785765484 | 4.449796653 | 5.093102836 | 5.574682644 | 5.983729739 | 5.638468232 | 6.342021935 | 4.193680339 | 6.125956212 | 6.646231471 | 7.038074117 | 6.6348863 |
| ENSMUSG00000029993 | Nfu1 |  | 2067 | 36.00704559 | 25.80258522 | 36.90617193 | 28.34615531 | 29.43335826 | 29.43916634 | 34.63187384 | 29.06465843 | 30.22735579 | 32.75685045 | 34.36930975 | 27.08423298 | 31.93328753 | 30.198768 | 37.31461666 | 37.16059915 | 31.66972187 | 33.61224083 |
| ENSMUSG00000029994 | Anxa4 |  | 5606 | 195.7012305 | 167.8539641 | 200.0261875 | 198.6204714 | 204.8568852 | 196.1248756 | 207.5816359 | 159.5960076 | 206.6966526 | 217.6367459 | 208.5531188 | 185.8364863 | 204.3675431 | 134.8668032 | 177.9913094 | 205.735174 | 191.1187353 | 182.7782074 |
| ENSMUSG00000029998 | Pcyox1 |  | 5173 | 43.66373582 | 38.03324053 | 48.29872111 | 53.06500911 | 47.02469403 | 50.03168905 | 40.7000621 | 36.36879456 | 43.09735353 | 43.96053389 | 39.65700207 | 43.96845642 | 43.32397065 | 34.21581617 | 45.43879303 | 41.85837712 | 48.78767932 | 45.98093514 |
| ENSMUSG00000029999 | Tgfa |  | 3756 | 24.71546977 | 30.1465728 | 29.29972885 | 40.42996826 | 33.07467918 | 35.96399141 | 21.09923781 | 24.84133096 | 19.95725737 | 25.86007687 | 21.9413747 | 21.66646327 | 22.34121174 | 20.11835202 | 28.86083428 | 28.94223493 | 30.51001804 | 31.34171682 |
| ENSMUSG00000030000 | Add2 |  | 3103 | 0 | 0 | 0 | 0 | 0 | 0.015969299 | 0 | 0 | 0 | 0 | 0 | 0 | 0 | 0 | 0 | 0 | 0 | 0 |
| ENSMUSG00000030001 | Figla |  | 756 | 0 | 0 | 0 | 0 | 0 | 0 | 0 | 0 | 0 | 0 | 0 | 0 | 0 | 0 | 0 | 0 | 0 | 0 |
| ENSMUSG00000030002 | Dusp11 |  | 7628 | 6.658847057 | 7.764856571 | 7.800136646 | 8.373962265 | 8.606704757 | 9.432429097 | 10.0852669 | 8.316410644 | 9.405730265 | 9.030830815 | 10.20885977 | 11.35630681 | 9.325724267 | 7.866512966 | 7.464676225 | 7.850879651 | 10.05889439 | 8.747515267 |
| ENSMUSG00000030004 | Nat8 |  | 1121 | 0 | 0 | 0.12945637 | 0 | 0 | 0 | 0 | 0 | 0 | 0 | 0 | 0 | 0 | 0 | 0 | 0 | 0 | 0 |
| ENSMUSG00000030007 | Cct7 |  | 2440 | 134.2222874 | 144.979354 | 130.3508041 | 132.4818072 | 135.4598032 | 143.3982999 | 120.6473313 | 129.196758 | 124.4710521 | 117.5868973 | 130.0888623 | 119.5690444 | 120.3375294 | 124.9612433 | 140.3892359 | 124.0521523 | 121.2304752 | 113.4450103 |
| ENSMUSG00000030008 | 1700040I03Ri | k | 1948 | 4.233061678 | 4.035853714 | 5.761118605 | 5.098720567 | 4.941675941 | 3.968288726 | 6.509136526 | 5.548485565 | 8.309189268 | 6.874127169 | 7.848042461 | 6.647450575 | 9.623269284 | 5.388277079 | 7.423894311 | 7.724953136 | 5.764684288 | 7.991569406 |
| ENSMUSG00000030016 | Zfml |  | 6565 | 10.76064015 | 9.204811898 | 8.716814381 | 9.244068706 | 8.944546363 | 9.555789903 | 10.57959066 | 9.220861814 | 9.241087238 | 9.573783312 | 7.946725767 | 9.037631276 | 8.776798077 | 8.022482117 | 9.125218928 | 9.192078791 | 11.08631193 | 8.538351828 |

| ENSMUSG00000030017 | Reg3g | 1351 | 0 | 0 | 0 | 0 | 0 | 0 | 0 | 0 | 0 | 0 | 0 | 0 | 0 | 0 | 0 | 0 | 0 | 0 |
| --- | --- | --- | --- | --- | --- | --- | --- | --- | --- | --- | --- | --- | --- | --- | --- | --- | --- | --- | --- | --- |
| ENSMUSG00000030019 | Fbxl14 | 4218 | 5.51216958 | 7.076423239 | 6.399343276 | 5.681628573 | 5.249095516 | 6.790298722 | 5.406522343 | 8.665274799 | 5.634096632 | 6.416430458 | 5.414042236 | 6.510504029 | 5.333180246 | 5.846801897 | 7.120894795 | 5.437665679 | 5.987915993 | 5.907805914 |
| ENSMUSG00000030020 | Prickle2 | 14537 | 0.116957215 | 0.109996486 | 0.043258987 | 0.159841319 | 0.185415679 | 0.112488148 | 0.0423103 | 0.1103447 | 0.164326747 | 0.194609768 | 0.207045634 | 0.115186756 | 0.057690211 | 0.038338656 | 0.039679561 | 0.144870565 | 0.145005595 | 0.227044535 |
| ENSMUSG00000030022 | Adamts9 | 14360 | 0.147998524 | 0.213425228 | 0.333494395 | 0.279204171 | 0.288255253 | 0.293313534 | 0.415139105 | 0.428201726 | 0.42161684 | 0.341481416 | 0.339348591 | 0.268195036 | 0.288571104 | 0.320192524 | 0.355779452 | 0.538628321 | 0.320275462 | 0.134075121 |
| ENSMUSG00000030029 | Lrig1 | 5806 | 10.47621545 | 9.989278132 | 11.88092564 | 10.56237783 | 10.52005014 | 11.62432365 | 14.62732668 | 11.6563791 | 11.20818031 | 14.91833308 | 13.60179235 | 12.3052098 | 8.054884185 | 7.975328131 | 10.0910459 | 8.962613755 | 9.584874802 | 8.868157749 |
| ENSMUSG00000030030 | 1700003E16R | 2046 | 0.851765502 | 1.058327529 | 0.236429767 | 0.423098652 | 0.470497788 | 0.605483059 | 2.404945556 | 0.72800767 | 0.603907856 | 0.852676452 | 1.214221819 | 1.554981802 | 0.313448155 | 0.749098184 | 0.98674303 | 0.131004033 | 0.580701468 | 1.075446857 |
| ENSMUSG00000030031 | Kbtbd8 | 5015 | 0.245792643 | 0.338775195 | 0.212206913 | 0.508757424 | 0.153561471 | 0.612616048 | 0.320763925 | 0.738716457 | 0 | 0.272656111 | 0.914535596 | 0.267113839 | 0 | 0.490834822 | 0.402567545 | 0.122163453 | 0.580817261 | 0 |
| ENSMUSG00000030032 | Wdr54 | 5798 | 1.224278095 | 1.200826991 | 0.967804333 | 0.974399706 | 1.153904345 | 0.752093921 | 1.297467394 | 1.119818629 | 1.58409676 | 1.561389277 | 1.318384504 | 1.222592126 | 1.54853552 | 0.833077801 | 1.627312228 | 1.908586675 | 1.467478081 | 1.669817658 |
| ENSMUSG00000030034 | Ino80b | 2034 | 12.91455201 | 11.53012661 | 10.70210849 | 9.631882529 | 12.4470953 | 9.379450554 | 10.28131961 | 11.2661954 | 13.14161688 | 10.2924839 | 9.912012034 | 10.78444218 | 11.37496028 | 13.1066402 | 13.16667224 | 9.054956814 | 7.48059974 | 10.14179696 |
| ENSMUSG00000030035 | Wbp1 | 2751 | 51.86836639 | 53.03906822 | 48.07460269 | 48.32683656 | 52.8733818 | 49.21049369 | 54.48439362 | 50.86767082 | 51.08265786 | 52.39538345 | 48.2612176 | 46.44203986 | 59.57129378 | 50.98551215 | 49.70845656 | 53.82398358 | 50.48873136 | 49.7301521 |
| ENSMUSG00000030036 | Mogs | 2888 | 17.49953404 | 19.78241468 | 20.36780225 | 19.68842913 | 15.09955777 | 20.69272739 | 16.85759924 | 18.32917927 | 13.93324997 | 19.36313506 | 23.17614849 | 20.66029893 | 21.89872505 | 18.07588443 | 24.49552336 | 17.88574425 | 17.11943317 | 18.51414534 |
| ENSMUSG00000030037 | Mrpl53 | 816 | 0 | 0.122473876 | 0.237125149 | 0.223338453 | 0.176955602 | 0.242905557 | 0 | 0.046804415 | 0.201894685 | 0.115565449 | 0 | 0.273606189 | 0.181367343 | 0 | 0.201968411 | 0.046924764 | 0.046968501 | 0.202239363 |
| ENSMUSG00000030041 | D6Mm5e | 2076 | 0.470914763 | 0.096280041 | 0 | 0.087786213 | 0 | 0.167085325 | 0 | 0.036794222 | 0 | 0 | 0 | 0 | 0.023762966 | 0 | 0 | 0 | 0 | 0 |
| ENSMUSG00000030042 | Pole4 | 2501 | 54.35088082 | 51.44118252 | 55.00772504 | 52.33781702 | 53.11639717 | 48.38375666 | 56.35524611 | 47.93520638 | 48.03721171 | 51.03436774 | 52.83707923 | 44.67931087 | 49.11494363 | 52.34941317 | 48.53250046 | 50.46215194 | 44.85448662 | 42.47204521 |
| ENSMUSG00000030043 | Tacr1 | 6468 | 0.059144494 | 0 | 0 | 0 | 0 | 0 | 0 | 0 | 0 | 0 | 0 | 0 | 0 | 0 | 0 | 0 | 0 | 0 |
| ENSMUSG00000030045 | Mrpl19 | 5303 | 6.452322591 | 6.093439071 | 6.814072633 | 6.624093001 | 6.99787161 | 5.634602743 | 6.512965419 | 6.640278148 | 4.908519321 | 6.383972268 | 6.405430036 | 5.346852083 | 6.381613285 | 7.330511247 | 6.479746863 | 5.776444639 | 5.630055683 | 5.466679095 |
| ENSMUSG00000030046 | Bmp10 | 1315 | 0 | 0 | 0 | 0 | 0 | 0 | 0 | 0 | 0 | 0 | 0 | 0 | 0 | 0 | 0 | 0 | 0 | 0 |
| ENSMUSG00000030047 | Arhgap25 | 4986 | 0.059674335 | 0.066812864 | 0.038807485 | 0.173618099 | 0.0675739 | 0.288212851 | 0.749639317 | 0.413636127 | 0.231292106 | 0.179675765 | 0.699472918 | 0 | 0.197881738 | 0.624098246 | 0.289220709 | 0.575971832 | 0 | 0.132392555 |
| ENSMUSG00000030048 | Gkn3 | 838 | 0 | 0 | 0 | 0 | 0 | 0 | 0 | 0 | 0 | 0 | 0 | 0 | 0 | 0 | 0 | 0 | 0 | 0 |
| ENSMUSG00000030049 | Gkn2 | 771 | 0 | 0 | 0 | 0 | 0 | 0 | 0 | 0 | 0 | 0 | 0 | 0 | 0 | 0 | 0 | 0 | 0 | 0 |
| ENSMUSG00000030050 | Gkn1 | 1222 | 0 | 0 | 0 | 0 | 0 | 0 | 0 | 0 | 0 | 0 | 0 | 0 | 0 | 0 | 0 | 0 | 0 | 0 |
| ENSMUSG00000030051 | Aplf | 4760 | 2.652108672 | 1.833608883 | 2.611764139 | 1.818613114 | 1.880785253 | 2.227790963 | 2.305996116 | 2.615698148 | 1.661304837 | 1.753292957 | 2.750071139 | 2.134128275 | 2.228227356 | 1.990460888 | 2.614048294 | 1.986928577 | 1.666710811 | 2.218854725 |
| ENSMUSG00000030054 | Gp9 | 809 | 0 | 0.1235336 | 0 | 0.112635462 | 0 | 0 | 1.052692485 | 0.755350357 | 0.814566443 | 0.233130795 | 0.295271684 | 0.482953817 | 0.548809957 | 2.296370205 | 0.050928994 | 0.567969455 | 0.189499614 | 0.339982118 |
| ENSMUSG00000030055 | Rab43 | 6798 | 5.758644766 | 7.32119211 | 5.664214499 | 7.492975517 | 6.435998619 | 6.188624714 | 4.732659852 | 5.286709424 | 5.022598053 | 4.89679266 | 5.376264083 | 4.351618295 | 6.262642632 | 6.087331842 | 7.188150233 | 6.325441617 | 6.252407071 | 5.688644017 |
| ENSMUSG00000030056 | Isy1 | 1609 | 16.03520314 | 14.69990982 | 15.87397391 | 15.20589236 | 15.58529039 | 12.780848 | 16.99610219 | 15.0253516 | 14.12986743 | 16.79139401 | 15.23216513 | 17.03262295 | 15.4219729 | 16.07790968 | 16.41404433 | 14.82600896 | 14.55398844 | 14.76938105 |
| ENSMUSG00000030057 | Cnbp | 3507 | 258.5822442 | 256.1302765 | 270.2958942 | 265.2466046 | 268.5615861 | 254.7295925 | 263.1944834 | 264.4826589 | 285.721911 | 276.0205127 | 269.7166818 | 261.7145012 | 231.762951 | 230.0617867 | 263.586572 | 245.6953346 | 273.9996909 | 254.8112172 |
| ENSMUSG00000030058 | Copg | 7944 | 26.41056758 | 32.59161806 | 28.38839357 | 32.74269916 | 33.40878992 | 32.42385563 | 24.67477704 | 27.56737606 | 19.80519766 | 25.36783808 | 28.63851363 | 23.71322523 | 23.2190381 | 27.84657455 | 30.93747242 | 25.12218602 | 29.28024873 | 27.71921629 |
| ENSMUSG00000030059 | Tmf1 | 3448 | 24.91385173 | 21.44855718 | 23.14858616 | 19.87349498 | 25.07103681 | 23.59790851 | 22.07833538 | 23.71517794 | 15.38521818 | 23.35655486 | 21.06088657 | 19.00452085 | 22.41969674 | 21.17462453 | 18.59327754 | 23.13205778 | 20.99720848 | 21.56969614 |
| ENSMUSG00000030060 | 8430410A17R | 2353 | 18.01005546 | 18.37659868 | 20.94884294 | 16.61341948 | 18.0622561 | 18.65861536 | 14.43710305 | 15.53341653 | 15.01828752 | 13.16532598 | 14.49694873 | 14.87310686 | 16.96112243 | 14.96158871 | 15.82924204 | 17.90041145 | 14.39882978 | 16.78560928 |
| ENSMUSG00000030061 | Uba3 | 3620 | 39.45231808 | 34.73927505 | 37.97723018 | 37.36760791 | 36.47123387 | 37.19192738 | 43.23490108 | 32.54794514 | 38.64933537 | 36.56133261 | 35.03936872 | 37.05111525 | 34.96842534 | 33.84516645 | 32.16452954 | 34.81060468 | 37.72281654 | 31.24274128 |
| ENSMUSG00000030062 | Rpn1 | 3632 | 124.3908359 | 140.2406808 | 137.7020733 | 138.1257322 | 131.5676592 | 136.5155981 | 112.1982664 | 123.7257177 | 121.7451633 | 123.8225241 | 123.9491833 | 117.4403341 | 115.1123345 | 99.71668322 | 115.334862 | 105.2675426 | 102.949161 | 111.7599665 |
| ENSMUSG00000030064 | Frmd4b | 12839 | 2.622018803 | 3.980214949 | 3.899571923 | 3.921251966 | 5.173460134 | 4.778120116 | 2.738010783 | 5.259301149 | 4.199170429 | 3.48516375 | 4.606698232 | 3.451798122 | 3.281365944 | 2.651570687 | 3.048639154 | 4.011283352 | 4.459808986 | 4.228833113 |
| ENSMUSG00000030067 | Foxp1 | 15253 | 21.62182264 | 21.81838414 | 21.09939667 | 21.35421923 | 21.49890487 | 21.59424507 | 19.34630423 | 19.54064829 | 19.2309949 | 21.48721814 | 22.01914103 | 21.19846151 | 17.89183547 | 16.65263974 | 16.95819628 | 19.62603872 | 20.36547803 | 20.30067932 |
| ENSMUSG00000030068 | Eif4e3 | 3988 | 2.717858547 | 2.581164572 | 2.935404801 | 2.650492048 | 2.86039767 | 2.73360115 | 3.784539764 | 2.671935875 | 3.242870098 | 2.979432603 | 3.066797736 | 3.932848843 | 2.436907649 | 1.677018201 | 3.140740469 | 2.851632497 | 1.970133116 | 2.455271062 |
| ENSMUSG00000030069 | Prok2 | 1509 | 0 | 0 | 0 | 0 | 0.159482848 | 0 | 0 | 0.48088512 | 0.109175655 | 0 | 0.031660012 | 0.110965532 | 0 | 0 | 0 | 0 | 0 | 0 |
| ENSMUSG00000030074 | Gxylt2 | 3039 | 0.055946267 | 0.032885384 | 0.015917582 | 0.269858111 | 0 | 0 | 0 | 0 | 0 | 0 | 0 | 0 | 0 | 0 | 0.094903222 | 0 | 0.063057415 | 0 |
| ENSMUSG00000030075 | Cntn3 | 5192 | 0 | 0.057745772 | 0.093169357 | 0 | 0 | 0 | 0.082013504 | 0.073560097 | 0.055528816 | 0.072651315 | 0.17483132 | 0 | 0.009501525 | 0.044726586 | 0.039677924 | 0.029499698 | 0.184544958 | 0 |
| ENSMUSG00000030077 | Chl1 | 7920 | 0.241506682 | 0.294432146 | 0.067185459 | 0.149569085 | 0.030386315 | 0.050053266 | 0 | 0 | 0 | 0 | 0.012064383 | 0 | 0 | 0 | 0 | 0 | 0 | 0 |
| ENSMUSG00000030079 | Ruvbl1 | 1887 | 12.43394656 | 13.38165014 | 13.15083258 | 10.47879875 | 12.29442883 | 11.00296522 | 13.23852383 | 11.7795327 | 10.91322622 | 12.44358782 | 14.98822227 | 10.41182471 | 16.47011547 | 13.88153963 | 12.05260141 | 12.84470296 | 14.23780293 | 12.97247085 |
| ENSMUSG00000030082 | Sec61a1 | 6890 | 32.04853258 | 37.05032052 | 34.95672099 | 38.06893066 | 34.59351031 | 34.0468274 | 32.82360019 | 35.48179504 | 31.83137829 | 33.82669466 | 41.12529445 | 36.60826984 | 44.8426412 | 43.29613546 | 46.84060773 | 45.40410196 | 46.09724564 | 48.36649761 |
| ENSMUSG00000030083 | Abtb1 | 2407 | 11.51365799 | 14.7672863 | 10.22938386 | 13.24999212 | 11.81801699 | 12.84624252 | 11.22373896 | 11.48786844 | 12.79913327 | 12.69366669 | 14.78701457 | 13.44955725 | 14.16217484 | 17.21151888 | 15.30294598 | 14.79445986 | 15.54070036 | 15.72341729 |

| ENSMUSG00000030084 | Plxna1 | 9140 | 2.869331905 | 3.531749945 | 3.953504067 | 3.399631536 | 3.328159273 | 4.02277115 | 3.27668772 | 3.029484873 | 3.582415758 | 3.987690768 | 2.75986631 | 3.651834379 | 4.112792228 | 2.286634499 | 2.776822585 | 3.372422205 | 3.622967235 | 2.648140805 |
| --- | --- | --- | --- | --- | --- | --- | --- | --- | --- | --- | --- | --- | --- | --- | --- | --- | --- | --- | --- | --- |
| ENSMUSG00000030086 | Chchd6 | 2519 | 4.758419865 | 3.46485839 | 5.300156561 | 4.539826172 | 4.089016143 | 4.799867802 | 7.926141527 | 5.367253057 | 6.68729057 | 5.877459636 | 7.225986177 | 7.666621379 | 6.834791238 | 7.080003794 | 7.867387211 | 7.509154449 | 7.333574874 | 6.354763818 |
| ENSMUSG00000030087 | Klf15 | 2523 | 27.24172399 | 20.00358095 | 24.48394699 | 19.57517719 | 21.34745247 | 25.51288185 | 53.01345356 | 36.01257444 | 47.83055536 | 43.39434521 | 44.10141827 | 44.73217192 | 46.37943233 | 41.28976933 | 44.58194514 | 41.70534647 | 41.66826492 | 51.30258744 |
| ENSMUSG00000030088 | Aldh1l1 | 4414 | 0.934073873 | 1.660361742 | 2.367168681 | 2.34308044 | 1.908266117 | 2.346289379 | 4.233916669 | 4.040745791 | 4.142911871 | 8.267930295 | 5.509164899 | 5.563863058 | 4.246970676 | 2.051789089 | 3.369678676 | 2.533044259 | 1.250336827 | 2.043836317 |
| ENSMUSG00000030089 | Slc41a3 | 2800 | 14.60356405 | 16.98957605 | 14.16653389 | 13.14761565 | 13.66602833 | 18.06905035 | 14.26139322 | 15.69980542 | 12.91491459 | 18.3550952 | 14.24717512 | 15.60845136 | 12.20964953 | 8.542394645 | 11.30099819 | 9.148720126 | 13.77009096 | 10.84465247 |
| ENSMUSG00000030091 | Nup210 | 8506 | 5.876567953 | 8.330181755 | 6.864195996 | 8.377318756 | 9.229152069 | 9.990940288 | 6.268679656 | 10.0846621 | 8.807697175 | 7.294888959 | 6.599526947 | 9.114502155 | 4.18155565 | 3.865790486 | 5.192577935 | 3.727324586 | 5.798958866 | 4.365288859 |
| ENSMUSG00000030092 | Cntn6 | 4367 | 0 | 0 | 0 | 0 | 0 | 0 | 0 | 0 | 0 | 0 | 0 | 0 | 0 | 0 | 0 | 0 | 0 | 0 |
| ENSMUSG00000030093 | Wnt7a | 3448 | 0 | 0 | 0 | 0 | 0 | 0 | 0 | 0 | 0 | 0 | 0 | 0 | 0 | 0 | 0 | 0 | 0 | 0 |
| ENSMUSG00000030094 | Xpc | 5475 | 17.80947467 | 19.90255288 | 18.863468 | 18.74868181 | 20.2901333 | 21.03389093 | 16.85973271 | 16.35123128 | 19.6566878 | 21.6936295 | 19.79933896 | 17.43283708 | 19.16511197 | 20.35905201 | 22.53856802 | 26.35932407 | 24.68983547 | 25.58049054 |
| ENSMUSG00000030095 | Tmem43 | 3899 | 23.54736605 | 30.63009125 | 22.35678423 | 23.54591032 | 27.14596054 | 30.29846547 | 15.58078475 | 18.15094169 | 17.60911048 | 18.65953709 | 15.32866711 | 19.0537694 | 14.36053504 | 16.18812895 | 19.19005527 | 20.11263502 | 19.03044647 | 16.57750716 |
| ENSMUSG00000030096 | Slc6a6 | 6154 | 29.69974926 | 35.21834413 | 31.22191462 | 35.28500769 | 33.38106113 | 39.37485655 | 22.87983953 | 24.57619654 | 24.30767391 | 27.25301454 | 21.17810964 | 25.0054894 | 30.34145383 | 26.1502702 | 28.30012622 | 39.59101965 | 32.57797515 | 23.77709195 |
| ENSMUSG00000030098 | Grip2 | 5980 | 0 | 0.02785359 | 0 | 0.038094519 | 0.040244083 | 0.033145641 | 0 | 0 | 0 | 0.007884733 | 0.02396737 | 0.074669783 | 0.008249484 | 0.01553314 | 0.013779785 | 0.166480902 | 0.173045153 | 0.147181556 |
| ENSMUSG00000030101 | Sumf1 | 3634 | 24.9136007 | 28.51855088 | 29.48469173 | 26.7047398 | 29.65530465 | 32.01701113 | 24.0078646 | 26.68423492 | 25.95407845 | 27.90896057 | 28.83062299 | 30.74930008 | 26.03704385 | 28.8965552 | 28.37850699 | 27.63793704 | 32.25146283 | 26.95960899 |
| ENSMUSG00000030102 | Itpr1 | 10369 | 5.92752286 | 6.059226396 | 6.409994282 | 4.789424084 | 5.686334892 | 6.652271689 | 5.032875384 | 6.405303094 | 5.632411935 | 4.933794294 | 5.565835649 | 6.362630261 | 6.137349145 | 5.836304935 | 5.404003859 | 4.638152464 | 6.834344968 | 5.830360525 |
| ENSMUSG00000030103 | Bhlhe40 | 3460 | 258.949886 | 238.4760338 | 246.8168511 | 291.4326686 | 287.8316857 | 292.9483137 | 186.6115981 | 205.9630163 | 179.4827412 | 193.7539592 | 221.6978998 | 200.9202536 | 117.6551969 | 138.3389493 | 146.1821677 | 216.9173976 | 239.2624315 | 214.4242261 |
| ENSMUSG00000030104 | Edem1 | 5846 | 11.49515623 | 10.38819811 | 10.33502214 | 12.18130556 | 8.579107845 | 11.96012779 | 9.161469816 | 8.898058857 | 11.85010597 | 9.88019355 | 8.580859802 | 11.10393698 | 12.35407576 | 10.32002558 | 10.44486928 | 12.52337348 | 12.03023517 | 11.65861841 |
| ENSMUSG00000030105 | Arl8b | 2921 | 71.4773792 | 74.22126518 | 75.1851516 | 79.08062129 | 88.86527365 | 78.81615892 | 70.86373513 | 70.81480706 | 76.45097174 | 69.68489762 | 73.25711709 | 64.77750639 | 64.37975647 | 69.57868073 | 73.24877773 | 77.45949988 | 78.12214028 | 67.89039019 |
| ENSMUSG00000030107 | Usp18 | 1759 | 16.72176339 | 11.74189563 | 10.97273372 | 16.81018634 | 14.25624345 | 18.39564243 | 13.12597346 | 9.336403089 | 10.20882369 | 13.99242018 | 8.528332554 | 11.99449713 | 29.81229566 | 18.24494849 | 25.7421887 | 38.02938666 | 22.68202108 | 34.15005375 |
| ENSMUSG00000030108 | Slc6a13 | 2928 | 0.014516795 | 0.113773546 | 0 | 0 | 0.065753994 | 0.084618739 | 0.016158702 | 0.11739468 | 0.112531464 | 0.064413529 | 0 | 0 | 0 | 0 | 0 | 0 | 0 | 0 |
| ENSMUSG00000030109 | Slc6a12 | 3035 | 0 | 0.109762419 | 0.191262723 | 0.150118762 | 0.095153721 | 0 | 0.093534126 | 0.100671901 | 0.122134643 | 0 | 0.062965349 | 0.257469284 | 0 | 0.061211318 | 0 | 0 | 0 | 0 |
| ENSMUSG00000030110 | Ret | 9588 | 0.02216582 | 0.059065415 | 0.005045216 | 0 | 0 | 0.041345627 | 0.098691445 | 0 | 0 | 0 | 0 | 0.029107041 | 0.133774494 | 0.06781573 | 0.0042972 | 0.139775893 | 0.031978554 | 0.126220312 |
| ENSMUSG00000030111 | A2m | 4634 | 0 | 0 | 0 | 0 | 0 | 0 | 0 | 0 | 0 | 0 | 0 | 0 | 0 | 0 | 0 | 0 | 0 | 0 |
| ENSMUSG00000030114 | Klrg1 | 1409 | 0 | 0 | 0 | 0 | 0 | 0 | 0 | 0 | 0 | 0 | 0 | 0 | 0 | 0 | 0 | 0 | 0 | 0 |
| ENSMUSG00000030116 | Mfap5 | 3578 | 0 | 0 | 0 | 0 | 0 | 0 | 0 | 0 | 0 | 0 | 0.01335242 | 0 | 0.027575135 | 0 | 0 | 0 | 0 | 0 |
| ENSMUSG00000030117 | Gdf3 | 2123 | 0 | 0 | 0 | 0 | 0 | 0 | 0 | 0 | 0 | 0 | 0 | 0 | 0 | 0 | 0 | 0 | 0 | 0 |
| ENSMUSG00000030120 | Mlf2 | 1524 | 306.2659698 | 306.6360106 | 296.494191 | 302.0960322 | 311.7836945 | 305.3801009 | 311.5993165 | 307.2182816 | 279.2791692 | 305.5204689 | 307.9039647 | 286.8793209 | 291.3625902 | 267.9677562 | 284.4367254 | 300.8475938 | 277.2620888 | 292.5156174 |
| ENSMUSG00000030122 | Ptms | 1313 | 39.91537094 | 33.13529311 | 32.12621361 | 32.06276083 | 30.38946285 | 30.26754938 | 28.8272225 | 28.09890383 | 33.21897958 | 26.60980283 | 25.79774986 | 28.86430685 | 30.39567485 | 29.0761918 | 27.3631049 | 30.15421786 | 23.90650203 | 27.52550123 |
| ENSMUSG00000030123 | Plxnd1 | 7221 | 4.191065688 | 5.199228886 | 4.039501288 | 4.915116125 | 5.185796512 | 4.247769296 | 3.086036806 | 4.225970021 | 3.981192079 | 4.701358031 | 3.625630418 | 4.48318575 | 2.582393675 | 2.28329193 | 2.476315643 | 2.317268444 | 3.322567769 | 3.138589111 |
| ENSMUSG00000030124 | Lag3 | 2100 | 0.04048112 | 0.047589849 | 0.046070029 | 0 | 0 | 0.04719308 | 0 | 0 | 0.019612627 | 0 | 0.068249941 | 0 | 0.070474168 | 0.154813625 | 0.019619789 | 0.018233623 | 0 | 0.314337753 |
| ENSMUSG00000030125 | Lrrc23 | 2020 | 0.315632495 | 0.164915318 | 0.071841877 | 0.338324587 | 0.09531074 | 0.049062112 | 0 | 0.01890713 | 0.224283007 | 0.256761255 | 0.141905817 | 0.110526065 | 0.244217412 | 0.022992122 | 0 | 0.208513209 | 0.170760729 | 0.027232231 |
| ENSMUSG00000030126 | Tmcc1 | 8240 | 2.512138438 | 2.082055889 | 1.80226624 | 1.653246633 | 1.974343471 | 1.62369394 | 2.153186238 | 1.937430122 | 1.804409246 | 1.373321454 | 1.953903035 | 2.336942184 | 1.700274819 | 1.521832961 | 1.930072885 | 2.188698554 | 1.897709847 | 1.982731153 |
| ENSMUSG00000030127 | Cops7a | 2702 | 72.81882303 | 63.92574261 | 67.86974596 | 73.7205351 | 71.93068385 | 69.76261974 | 70.67134425 | 76.44134427 | 69.26407388 | 71.58111945 | 77.42655188 | 68.04470487 | 68.46583634 | 66.86435971 | 72.24758394 | 74.54056067 | 72.72351007 | 74.69593359 |
| ENSMUSG00000030131 | Mug2 | 4509 | 0 | 0.066492803 | 0 | 0.050522338 | 0 | 0 | 0 | 0 | 0 | 0.02091404 | 0 | 0 | 0.010940767 | 0.020600615 | 0.054825756 | 0 | 0.01699991 | 0.024399692 |
| ENSMUSG00000030134 | Rasgef1a | 3325 | 0.166185651 | 0.050094578 | 0.130935872 | 0 | 0.101330366 | 0.149030778 | 0 | 0.172296552 | 0 | 0.127625964 | 0 | 0 | 0.014836667 | 0 | 0 | 0.16122361 | 0 | 0.397058214 |
| ENSMUSG00000030137 | Tuba8 | 2220 | 0 | 0.075029041 | 0 | 0.102614965 | 0 | 0 | 0 | 0.137630279 | 0 | 0.276107722 | 0 | 0 | 0 | 0.02092076 | 0.129914816 | 0.120736149 | 0.069056391 | 0 |
| ENSMUSG00000030138 | Bms1 | 4263 | 5.254569028 | 6.954838343 | 7.273617865 | 9.26610965 | 8.400223131 | 6.881355449 | 7.147399772 | 7.346415663 | 5.700221511 | 6.868540165 | 4.236203215 | 7.476130269 | 6.538243786 | 6.482343888 | 7.171370976 | 5.32637349 | 7.533998781 | 4.335693141 |
| ENSMUSG00000030141 | Vmn2r26 | 2562 | 0 | 0 | 0 | 0 | 0.018786855 | 0 | 0.073868351 | 0 | 0.080379617 | 0.092019327 | 0 | 0 | 0.038510474 | 0.181280591 | 0.28947229 | 0 | 0.119676181 | 0 |
| ENSMUSG00000030142 | Clec4e | 5766 | 0 | 0 | 0 | 0 | 0 | 0 | 0 | 0 | 0 | 0 | 0 | 0 | 0 | 0 | 0 | 0 | 0 | 0 |
| ENSMUSG00000030143 | Gm8882 | 1237 | 0 | 0 | 0 | 0 | 0 | 0 | 0 | 0 | 0 | 0 | 0 | 0 | 0 | 0 | 0 | 0 | 0 | 0 |
| ENSMUSG00000030144 | Clec4d | 1330 | 0 | 0 | 0 | 0 | 0 | 0 | 0 | 0 | 0 | 0 | 0 | 0 | 0 | 0 | 0 | 0 | 0 | 0 |
| ENSMUSG00000030145 | Zfp248 | 6319 | 1.325133673 | 1.117634685 | 1.079390375 | 1.196887697 | 1.256807629 | 1.184121344 | 1.669683083 | 1.535190728 | 1.303577014 | 0.813329111 | 1.2021235 | 0.706639185 | 1.092968574 | 1.727229077 | 0.723749439 | 1.157383449 | 1.231245177 | 0.452678201 |

| ENSMUSG00000030147 | Clec4b1 | 902 | 0 | 0 | 0 | 0 | 0 | 0 | 0 | 0.04234191 | 0 | 0 | 0 | 0 | 0 | 0 | 0.274067999 | 0 | 0 | 0 |
| --- | --- | --- | --- | --- | --- | --- | --- | --- | --- | --- | --- | --- | --- | --- | --- | --- | --- | --- | --- | --- |
| ENSMUSG00000030148 | Clec4a2 | 2958 | 0.014369566 | 0 | 0.049060376 | 0 | 0 | 0 | 0.031989641 | 0 | 0.013923771 | 0.031880124 | 0 | 0 | 0 | 0 | 0.013928856 | 0.012944762 | 0 | 0 |
| ENSMUSG00000030149 | Klrk1 | 4089 | 0 | 0 | 0 | 0 | 0 | 0 | 0 | 0.289548661 | 0 | 0 | 0 | 0 | 0 | 0 | 0 | 0 | 0 | 0 |
| ENSMUSG00000030154 | Klrb1f | 1814 | 0 | 0.01836433 | 0 | 0.050232684 | 0.026533585 | 0 | 0.13040981 | 0.189488215 | 0 | 0 | 0 | 0 | 0.217560826 | 0 | 0 | 0 | 0 | 0 |
| ENSMUSG00000030155 | Clec2e | 2263 | 0.093913336 | 0 | 0.021375842 | 0.060399087 | 0 | 0.087587686 | 0 | 0 | 0 | 0.208354853 | 0 | 0 | 0 | 0 | 0.018206609 | 0 | 0.016936057 | 0 |
| ENSMUSG00000030156 | Cd69 | 1616 | 0 | 0.020614415 | 0 | 0.028193716 | 0 | 0 | 0 | 0.378142598 | 0.892034685 | 0.437661231 | 9.046495851 | 1.174339436 | 0.122108706 | 0.83346444 | 0.458928222 | 0.236946828 | 0 | 0 |
| ENSMUSG00000030157 | Clec2d | 1208 | 1.970438624 | 2.206151935 | 1.882082721 | 1.73494043 | 1.673460922 | 2.13306469 | 2.428299746 | 2.244752128 | 2.557109836 | 2.966434973 | 3.163904535 | 4.481886814 | 4.083767986 | 3.614026668 | 2.182863888 | 3.518425018 | 2.09398642 | 2.45901636 |
| ENSMUSG00000030158 | Clec12b | 1505 | 0 | 0 | 0 | 0 | 0.255850757 | 0 | 0 | 0 | 0 | 0 | 0 | 0 | 0.229450778 | 0 | 0 | 0 | 0 | 0 |
| ENSMUSG00000030159 | Clec1b | 5168 | 0 | 0 | 0 | 0 | 0.009313453 | 0.009588377 | 0.036619721 | 0.081291878 | 0 | 0 | 0 | 0 | 0 | 0 | 0 | 0 | 0 | 0 |
| ENSMUSG00000030160 | D630042F21R | 2870 | 0 | 0 | 0 | 0 | 0 | 0 | 0 | 0 | 0 | 0 | 0 | 0.058343898 | 0 | 0 | 0 | 0 | 0 | 0 |
| ENSMUSG00000030161 | Gabarapl1 | 1761 | 93.24105338 | 94.86891795 | 85.5120954 | 105.4035745 | 94.89723962 | 92.23955745 | 110.9067227 | 99.85111906 | 105.0829161 | 117.1404468 | 112.560081 | 103.6440753 | 146.174869 | 126.9629964 | 140.9416086 | 192.518477 | 158.3326576 | 140.1310919 |
| ENSMUSG00000030162 | Olr1 | 3562 | 0 | 0 | 0 | 0 | 0 | 0 | 0 | 0 | 0 | 0 | 0 | 0 | 0 | 0 | 0 | 0 | 0 | 0 |
| ENSMUSG00000030165 | Klrd1 | 854 | 0 | 0 | 0 | 0 | 0 | 0 | 0 | 0 | 0 | 0 | 0 | 0 | 0 | 0 | 0 | 0 | 0 | 0 |
| ENSMUSG00000030166 | Rad52 | 3398 | 2.827006999 | 2.901888372 | 2.719053649 | 2.373250397 | 2.917944754 | 2.304100031 | 2.840431578 | 2.866116131 | 4.424096013 | 3.857532521 | 3.500872472 | 4.714271678 | 5.095792514 | 4.565133958 | 4.146831111 | 3.290423002 | 3.338606205 | 3.836715213 |
| ENSMUSG00000030167 | Klrc1 | 1977 | 0 | 0 | 0 | 0 | 0 | 0 | 0 | 0 | 0 | 0 | 0 | 0 | 0 | 0 | 0 | 0 | 0 | 0 |
| ENSMUSG00000030168 | Adipor2 | 6322 | 7.315031876 | 5.896413893 | 6.320236646 | 5.750982819 | 6.593205615 | 7.516778156 | 5.328476336 | 5.884118941 | 6.820987346 | 5.981471688 | 4.352795964 | 5.756376464 | 6.835615238 | 6.641166561 | 5.435320723 | 6.704793166 | 7.638584957 | 7.874603224 |
| ENSMUSG00000030170 | Wnt5b | 3941 | 0.420629755 | 0.312757444 | 0.576898231 | 0.716768523 | 0.390819984 | 0.226325604 | 0.396173155 | 0.426405914 | 0.229917114 | 0.51445832 | 0.400044057 | 0.467372968 | 0.075105685 | 0.377115148 | 0.365910798 | 0.223467133 | 0.515425967 | 0.614159019 |
| ENSMUSG00000030172 | Erc1 | 9826 | 4.568030316 | 3.475037307 | 3.190112728 | 3.171560587 | 3.326030549 | 3.247706129 | 2.80235896 | 3.229990474 | 2.52752585 | 3.263024447 | 3.033948106 | 3.550253319 | 3.524425598 | 2.8454448 | 2.524255714 | 4.364490158 | 3.397334073 | 3.661302239 |
| ENSMUSG00000030173 | Klra5 | 1367 | 0 | 0 | 0 | 0 | 0 | 0 | 0 | 0 | 0 | 0 | 0 | 0 | 0 | 0 | 0 | 0 | 0 | 0 |
[truncated: 5,505,052 more chars]
