# Supplementary material for: Proton-Secreting Cells as Drivers of Inflammation and Sperm Dysfunction in LPS-Induced Epididymitis
Source: Function (Oxf). 2025 Jun 2;6(3):zqaf023. doi: 10.1093/function/zqaf023 (PMC12203219; doi:10.1093/function/zqaf023)
Supplement: zqaf023_Supplemental_Files [file zqaf023_supplemental_files.zip › Suppl table 2 23-05.docx]

| Seg 1,2,3 | Seg 4,5,6 | Seg 8,9,10 | Seg 7 | |  |
| --- | --- | --- | --- | --- | --- |
| Abl1 | Abl1 | Abl1 | Abl1 | |  |
| Actr1a | Actr1a | Actr1a | Actr1a | |  |
| Adora2b | Adam8 | Adora2b | Adam8 | |  |
| Ap1g1 | Adora2b | Ap1g1 | Adora2b | |  |
| B2m | Ap1g1 | B2m | Ap1g1 | |  |
| Bax | B2m | Bax | B2m | |  |
| Bcar1 | Bax | Bcar1 | Bax | |  |
| Bcl10 | Bcar1 | Bcl10 | Bcar1 | |  |
| Bcl2 | Bcl10 | Bcl2 | Bcl10 | |  |
| Blk | Bcl2 | Blk | Bcl2 | |  |
| Braf | Braf | Braf | Blk | |  |
| Btk | C1qb | C1qa | Braf | |  |
| C1qa | C1qbp | C1qb | C1qb | |  |
| C1qb | C2 | C1qbp | C1qbp | |  |
| C1qbp | C9 | C2 | C2 | |  |
| C2 | Cav1 | C3 | C3ar1 | |  |
| C3ar1 | Cd14 | C3ar1 | C9 | |  |
| Cav1 | Cd19 | Cav1 | Cav1 | |  |
| Cd14 | Cd1d1 | Cd14 | Cd14 | |  |
| Cd24a | Cd24a | Cd19 | Cd19 | |  |
| Cd3e | Cd3e | Cd1d1 | Cd24a | |  |
| Cd44 | Cd4 | Cd24a | Cd3e | |  |
| Cfi | Cd44 | Cd3e | Cd4 | |  |
| Colec12 | Cfi | Cd4 | Cd44 | |  |
| Crhr1 | Cnr1 | Cd44 | Cfi | |  |
| Cst11 | Colec12 | Cd79a | Cnr1 | |  |
| Cyld | Crhr1 | Cfi | Coch | |  |
| Ddx58 | Crtam | Cnr1 | Colec12 | |  |
| Dhx15 | Cst11 | Colec12 | Crhr1 | |  |
| Exosc3 | Cyld | Crhr1 | Crp | |  |
| Exosc6 | Ddx58 | Crtam | Crtam | |  |
| Fadd | Dhx15 | Cyba | Cst11 | |  |
| Fcgr1 | Exosc3 | Cyld | Cyld | |  |
| Fcna | F2rl1 | Ddx58 | Ddx58 | |  |
| Fcnb | Fadd | Dhx15 | Dhx15 | |  |
| Fgr | Fcer1a | Exosc3 | Exosc3 | |  |
| Flot1 | Fcgr1 | F2rl1 | F2rl1 | |  |
| Fpr-rs4 | Fcna | Fadd | Fadd | |  |
| Gata2 | Fcnb | Fcer1a | Fcer1a | |  |
| Gpr33 | Fgr | Fcgr1 | Fcgr1 | |  |
| Gpx4 | Flot1 | Fcna | Fcna | |  |
| H2-M3 | Gab2 | Fcnb | Fcnb | |  |
| Hmgb1 | Gata2 | Fgr | Fgr | |  |
| Hmgb2 | Gc | Flot1 | Flot1 | |  |
| Hspa8 | Gpr33 | Fpr-rs4 | Foxp3 | |  |
| Il4ra | Gpx4 | Gata2 | Fpr-rs4 | |  |
| Irak1 | H2-M3 | Gata3 | Gata2 | |  |
| Irak2 | Havcr2 | Gpr33 | Gc | |  |
| Irak3 | Hmgb1 | Gpx4 | Gpr33 | |  |
| Irak4 | Hmgb2 | H2-M3 | Gpx4 | |  |
| Irf3 | Hspa8 | Havcr2 | H2-M3 | |  |
| Irf7 | Il4ra | Hmgb1 | Havcr2 | |  |
| Kars | Irak1 | Hmgb2 | Hmgb1 | |  |
| Klrb1c | Irak2 | Hspa8 | Hmgb2 | |  |
| Klre1 | Irak3 | Ifih1 | Hspa8 | |  |
| Klrk1 | Irak4 | Il18 | Ifih1 | |  |
| Lamp1 | Irf3 | Il4 | Il12b | |  |
| Lbp | Irf7 | Irak1 | Il18 | |  |
| Lck | Kars | Irak2 | Il4 | |  |
| Lcp1 | Klk5 | Irak4 | Irak1 | |  |
| Lgals9 | Klrb1c | Irf3 | Irak2 | |  |
| Lrrfip2 | Klre1 | Irf7 | Irak4 | |  |
| Ltf | Lamp1 | Kars | Irf3 | |  |
| Ly96 | Lbp | Klk5 | Irf7 | |  |
| Malt1 | Lck | Klrb1c | Kars | |  |
| Mapk1 | Lcp1 | Klre1 | Klk5 | |  |
| Mapk3 | Lgals9 | Klrk1 | Klrb1c | |  |
| Mapkapk3 | Lrrfip2 | Lamp1 | Klre1 | |  |
| Masp1 | Ly96 | Lbp | Klrk1 | |  |
| Mef2c | Malt1 | Lck | Lag3 | |  |
| Mif | Mapk1 | Lcp1 | Lamp1 | |  |
| Mlh1 | Mapk3 | Lcp2 | Lbp | |  |
| Mmp2 | Mapkapk3 | Lgals9 | Lck | |  |
| Msh2 | Masp1 | Lrrfip2 | Lcp1 | |  |
| Myd88 | Mef2c | Ltf | Lcp2 | |  |
| Myo1g | Mif | Ly96 | Lgals9 | |  |
| Nfatc2 | Mlh1 | Malt1 | Lrrfip2 | |  |
| Nfkbia | Mmp12 | Mapk1 | Ltf | |  |
| Nras | Mmp2 | Mapk3 | Ly96 | |  |
| P2rx7 | Msh2 | Mapkapk3 | Malt1 | |  |
| Park7 | Myb | Masp1 | Mapk1 | |  |
| Pde4b | Myd88 | Mef2c | Mapk3 | |  |
| Peli1 | Nfatc2 | Mif | Mapkapk3 | |  |
| Phb | Nfkbia | Mlh1 | Masp1 | |  |
| Pik3ap1 | Nr4a3 | Mmp12 | Mef2c | |  |
| Plcg1 | Nras | Mmp2 | Mif | |  |
| Plcg2 | P2rx7 | Msh2 | Mmp12 | |  |
| Pms2 | Park7 | Myb | Mmp2 | |  |
| Pnp | Pde4b | Myd88 | Msh2 | |  |
| Polr3b | Peli1 | Nfatc2 | Myb | |  |
| Polr3c | Phb | Nfkbia | Myd88 | |  |
| Polr3d | Pik3ap1 | Nr4a3 | Myo1g | |  |
| Polr3f | Plcg1 | Nras | Nfatc2 | |  |
| Prkch | Plcg2 | P2rx7 | Nfkbia | |  |
| Prkd2 | Pld2 | Park7 | Nr1h3 | |  |
| Psen1 | Pms2 | Pde4b | Nr4a3 | |  |
| Psen2 | Pnp | Peli1 | Nras | |  |
| Pum2 | Polr3b | Phb | P2rx7 | |  |
| Pycard | Polr3c | Pik3ap1 | Park7 | |  |
| Rap1a | Polr3d | Plcg1 | Pde4b | |  |
| Rapgef1 | Polr3f | Plcg2 | Peli1 | |  |
| Rasgrp1 | Prkch | Pld2 | Phb | |  |
| Rela | Prkd2 | Plscr1 | Pik3ap1 | |  |
| Rnf31 | Psen1 | Pms2 | Plcg1 | |  |
| Rps19 | Pum2 | Pnp | Plcg2 | |  |
| Rps3 | Pycard | Polr3b | Pms2 | |  |
| Rsad2 | Rap1a | Polr3c | Pnp | |  |
| Serping1 | Rapgef1 | Polr3d | Polr3b | |  |
| Shb | Rasgrp1 | Polr3f | Polr3c | |  |
| Sin3a | Rela | Prkch | Polr3d | |  |
| Slamf6 | Rnf31 | Prkd2 | Polr3f | |  |
| Slc11a1 | Rps19 | Psen1 | Prkch | |  |
| Slc39a10 | Rps3 | Psen2 | Prkd2 | |  |
| Snx4 | Rsad2 | Pum2 | Psen1 | |  |
| Spon2 | Serping1 | Pycard | Psen2 | |  |
| Stat5b | Shb | Rap1a | Ptafr | |  |
| Stat6 | Sin3a | Rapgef1 | Pum1 | |  |
| Stk11 | Slamf6 | Rasgrp1 | Pum2 | |  |
| Stoml2 | Slc11a1 | Rela | Pycard | |  |
| Stx4a | Slc39a10 | Rnf31 | Rap1a | |  |
| Stx7 | Snx4 | Rps19 | Rapgef1 | |  |
| Tap2 | Spon2 | Rps3 | Rasgrp1 | |  |
| Tec | Stat5a | Rsad2 | Rela | |  |
| Tfrc | Stat5b | S100a14 | Rnf31 | |  |
| Tgfb1 | Stat6 | Serping1 | Rps19 | |  |
| Thy1 | Stk11 | Shb | Rps3 | |  |
| Tlr12 | Stoml2 | Sin3a | Rsad2 | |  |
| Tlr13 | Stx4a | Slamf6 | Serping1 | |  |
| Tlr2 | Stx7 | Slc11a1 | Shb | |  |
| Tlr5 | Syk | Slc39a10 | Sin3a | |  |
| Tnfrsf21 | Tap2 | Snx4 | Sirt1 | |  |
| Tnfsf13b | Tec | Socs5 | Slamf6 | |  |
| Tnfsf4 | Tfrc | Spon2 | Slc11a1 | |  |
| Tnip1 | Tgfb1 | Stat5a | Slc39a10 | |  |
| Traf2 | Thy1 | Stat5b | Snx4 | |  |
| Traf3 | Tlr12 | Stat6 | Spon2 | |  |
| Traf6 | Tlr2 | Stk11 | Stat5a | |  |
| Ube2n | Tlr5 | Stoml2 | Stat5b | |  |
| Unc93b1 | Tlr9 | Stx4a | Stat6 | |  |
| Ung | Tnfrsf21 | Stx7 | Stk11 | |  |
| Usp12 | Tnfsf13b | Syk | Stoml2 | |  |
| Vamp8 | Tnfsf4 | Tap2 | Stx4a | |  |
| Vav1 | Tnip1 | Tec | Stx7 | |  |
| Wdfy1 | Tnip2 | Tfrc | Syk | |  |
| Wnt5a | Traf3 | Thy1 | Tap2 | |  |
| Xbp1 | Traf6 | Tlr12 | Tec | |  |
| Zap70 | Ube2n | Tlr13 | Tfrc | |  |
| Zbtb1 | Ung | Tlr2 | Tgfb1 | |  |
| Abl1 | Usp12 | Tlr3 | Thy1 | |  |
| Actr1a | Vamp8 | Tlr5 | Tlr12 | |  |
| Adora2b | Vav1 | Tlr8 | Tlr2 | |  |
| Ap1g1 | Wdfy1 | Tlr9 | Tlr5 | |  |
| B2m | Wnt5a | Tnfrsf21 | Tlr8 | |  |
| Bax | Xbp1 | Tnfsf13b | Tlr9 | |  |
| Bcar1 | Zap70 | Tnfsf4 | Tnfrsf21 | |  |
| Bcl10 | Zbtb1 | Tnip1 | Tnfsf13b | |  |
| Bcl2 | Abl1 | Tnip2 | Tnfsf4 | |  |
| Braf | Actr1a | Traf2 | Tnip1 | |  |
| C1qa | Adam8 | Traf3 | Tnip2 | |  |
| C1qb | Adora2b | Traf6 | Traf3 | |  |
| C1qbp | Ap1g1 | Ube2n | Traf6 | |  |
| C2 | B2m | Unc93b1 | Txk | |  |
| C3ar1 | Bax | Ung | Ube2n | |  |
| Cav1 | Bcar1 | Usp12 | Ung | |  |
| Cd14 | Bcl10 | Vamp8 | Usp12 | |  |
| Cd19 | Bcl2 | Vav1 | Vamp8 | |  |
| Cd24a | Braf | Wdfy1 | Vav1 | |  |
| Cd3e | C1qb | Wnt5a | Wdfy1 | |  |
| Cd4 | C1qbp | Xbp1 | Wnt5a | |  |
| Cd44 | C2 | Zap70 | Xbp1 | |  |
| Cfi | Cav1 | Zbtb1 | Zap70 | |  |
| Colec12 | Cd14 | Abl1 | Zbtb1 | |  |
| Crhr1 | Cd19 | Actr1a | Acp5 | |  |
| Crtam | Cd1d1 | Adora2b | Adora1 | |  |
| Cst11 | Cd24a | Ap1g1 | Adora2a | |  |
| Cyld | Cd3e | B2m | Adrb2 | |  |
| Ddx58 | Cd4 | Bax | Apod | |  |
| Dhx15 | Cd44 | Bcar1 | Apoe | |  |
| Exosc3 | Cfi | Bcl10 | Ash1l | |  |
| Exosc6 | Colec12 | Bcl2 | Bcr | |  |
| F2rl1 | Crhr1 | Braf | Calcrl | |  |
| Fadd | Crtam | C1qa | Cd44 | |  |
| Fcer1a | Cst11 | C1qb | Chrna7 | |  |
| Fcgr1 | Cyld | C1qbp | Cnr2 | |  |
| Fcna | Ddx58 | C2 | Cuedc2 | |  |
| Fcnb | Dhx15 | C3 | Cx3cr1 | |  |
| Fgr | Exosc3 | C3ar1 | Dusp10 | |  |
| Flot1 | F2rl1 | Cav1 | Ets1 | |  |
| Gab2 | Fadd | Cd14 | Fcgr2b | |  |
| Gata2 | Fcer1a | Cd19 | Fem1a | |  |
| Gpr33 | Fcgr1 | Cd1d1 | Fn1 | |  |
| Gpx4 | Fcna | Cd24a | Foxp3 | |  |
| H2-M3 | Fcnb | Cd3e | Gpx1 | |  |
| Havcr2 | Fgr | Cd4 | Hamp | |  |
| Hmgb1 | Flot1 | Cd44 | Ier3 | |  |
| Hmgb2 | Gab2 | Cd79a | Il12b | |  |
| Hspa8 | Gata2 | Cfi | Il22ra2 | |  |
| Il4ra | Gc | Cnr1 | Il4 | |  |
| Irak1 | Gpr33 | Colec12 | Ins2 | |  |
| Irak2 | Gpx4 | Crhr1 | Lgals9 | |  |
| Irak3 | H2-M3 | Crtam | Mefv | |  |
| Irak4 | Havcr2 | Cst11 | Metrnl | |  |
| Irf3 | Hmgb1 | Cyba | Ndfip1 | |  |
| Irf7 | Hmgb2 | Cyld | Nr1h3 | |  |
| Kars | Hspa8 | Ddx58 | Nt5e | |  |
| Klk5 | Il4ra | Dhx15 | Pbk | |  |
| Klrb1c | Irak1 | Exosc3 | Pparg | |  |
| Klre1 | Irak2 | F2rl1 | Proc | |  |
| Klrk1 | Irak3 | Fadd | Psma1 | |  |
| Lamp1 | Irak4 | Fcer1a | Psmb4 | |  |
| Lbp | Irf3 | Fcgr1 | Ptgds | |  |
| Lck | Irf7 | Fcna | Ptgis | |  |
| Lcp1 | Itk | Fcnb | Ptpn2 | |  |
| Lcp2 | Kars | Fgr | Rabgef1 | |  |
| Lgals9 | Klk5 | Flot1 | Rps19 | |  |
| Lrrfip2 | Klrb1c | Gata2 | Serpinb1a | |  |
| Ly96 | Klre1 | Gata3 | Serpinf1 | |  |
| Malt1 | Lamp1 | Gpr33 | Slamf8 | |  |
| Mapk1 | Lbp | Gpx4 | Slit2 | |  |
| Mapk3 | Lck | H2-M3 | Smad3 | |  |
| Mapkapk3 | Lcp1 | Havcr2 | Smpdl3b | |  |
| Masp1 | Lgals9 | Hmgb1 | Spn | |  |
| Mef2c | Lrrfip2 | Hmgb2 | Tff2 | |  |
| Mif | Ly96 | Hspa8 | Tnfaip3 | |  |
| Mlh1 | Malt1 | Ifih1 | Tnfrsf1a | |  |
| Mmp12 | Mapk1 | Il18 | Tnfrsf1b | |  |
| Mmp2 | Mapk3 | Irak1 | Uaca | |  |
| Msh2 | Mapkapk3 | Irak2 | Zfp36 | |  |
| Myb | Masp1 | Irak4 | Acp5 | |  |
| Myd88 | Mef2c | Irf3 | Adora1 | |  |
| Nfatc2 | Mif | Irf7 | Adora2a | |  |
| Nfkbia | Mlh1 | Kars | Adrb2 | |  |
| Nr4a3 | Mmp12 | Klk5 | Apod | |  |
| Nras | Mmp2 | Klrb1c | Apoe | |  |
| P2rx7 | Msh2 | Klre1 | Ash1l | |  |
| Park7 | Myb | Lamp1 | Bax | |  |
| Pde4b | Myd88 | Lbp | Bcr | |  |
| Peli1 | Nfatc2 | Lck | Calcrl | |  |
| Phb | Nfkbia | Lcp1 | Ccl2 | |  |
| Pik3ap1 | Nr4a3 | Lgals9 | Cd22 | |  |
| Plcg1 | Nras | Lrrfip2 | Cd4 | |  |
| Plcg2 | P2rx7 | Ltf | Cd44 | |  |
| Pms2 | Park7 | Ly96 | Cd5 | |  |
| Pnp | Pde4b | Malt1 | Cd80 | |  |
| Polr3b | Peli1 | Mapk1 | Chrna7 | |  |
| Polr3c | Phb | Mapk3 | Cnr2 | |  |
| Polr3d | Pik3ap1 | Mapkapk3 | Cuedc2 | |  |
| Polr3f | Plcg1 | Masp1 | Cx3cr1 | |  |
| Prkch | Plcg2 | Mef2c | Cxcl16 | |  |
| Prkd2 | Pms2 | Mif | Cybb | |  |
| Psen1 | Pnp | Mmp12 | Dusp10 | |  |
| Pum2 | Polr3b | Mmp2 | Ets1 | |  |
| Pycard | Polr3c | Msh2 | Fcgr2b | |  |
| Rap1a | Polr3d | Myb | Fem1a | |  |
| Rapgef1 | Polr3f | Myd88 | Foxp3 | |  |
| Rasgrp1 | Prkch | Nfatc2 | Gpx1 | |  |
| Rela | Prkd2 | Nfkbia | Hamp | |  |
| Rnf31 | Psen1 | Nr4a3 | Havcr2 | |  |
| Rps19 | Pum2 | Nras | Hmgb1 | |  |
| Rps3 | Pycard | P2rx7 | Ier3 | |  |
| Rsad2 | Rap1a | Park7 | Il12b | |  |
| Serping1 | Rapgef1 | Pde4b | Il22ra2 | |  |
| Shb | Rasgrp1 | Peli1 | Il4 | |  |
| Sin3a | Rela | Phb | Ins2 | |  |
| Slamf6 | Rnf31 | Pik3ap1 | Itgal | |  |
| Slc11a1 | Rps19 | Plcg1 | Lgals9 | |  |
| Slc39a10 | Rps3 | Plcg2 | Ltbr | |  |
| Snx4 | Rsad2 | Pld2 | Mapk1 | |  |
| Spon2 | Serping1 | Plscr1 | Mapk3 | |  |
| Stat5a | Shb | Pms2 | Mefv | |  |
| Stat5b | Sin3a | Pnp | Metrnl | |  |
| Stat6 | Slamf6 | Polr3b | Msn | |  |
| Stk11 | Slc11a1 | Polr3d | Myd88 | |  |
| Stoml2 | Slc39a10 | Polr3f | Ndfip1 | |  |
| Stx4a | Snx4 | Prkch | Nr1h3 | |  |
| Stx7 | Spon2 | Prkd2 | Nt5e | |  |
| Syk | Stat5a | Psen1 | Pbk | |  |
| Tap2 | Stat5b | Psen2 | Pparg | |  |
| Tec | Stat6 | Pum2 | Prf1 | |  |
| Tfrc | Stk11 | Pycard | Prnp | |  |
| Tgfb1 | Stoml2 | Rap1a | Proc | |  |
| Thy1 | Stx4a | Rapgef1 | Psma1 | |  |
| Tlr12 | Stx7 | Rasgrp1 | Psmb4 | |  |
| Tlr13 | Syk | Rela | Pten | |  |
| Tlr2 | Tap2 | Rnf31 | Ptgis | |  |
| Tlr5 | Tec | Rps19 | Ptpn2 | |  |
| Tlr9 | Tfrc | Rps3 | Rabgef1 | |  |
| Tnfrsf21 | Thy1 | Rsad2 | Rap1a | |  |
| Tnfsf13b | Tlr12 | S100a14 | Rps19 | |  |
| Tnip1 | Tlr13 | Serping1 | Serpinf1 | |  |
| Tnip2 | Tlr2 | Shb | Slamf8 | |  |
| Traf3 | Tlr4 | Sin3a | Slit2 | |  |
| Traf6 | Tlr5 | Slamf6 | Smad3 | |  |
| Ube2n | Tlr9 | Slc11a1 | Smpdl3b | |  |
| Unc93b1 | Tnfrsf21 | Slc39a10 | Spn | |  |
| Ung | Tnfsf13b | Snx4 | Stat1 | |  |
| Usp12 | Tnfsf4 | Socs5 | Stat3 | |  |
| Vamp8 | Tnip1 | Spon2 | Stat5a | |  |
| Vav1 | Tnip2 | Stat5a | Stat5b | |  |
| Wdfy1 | Traf3 | Stat5b | Stk11 | |  |
| Wnt5a | Traf6 | Stat6 | Tff2 | |  |
| Xbp1 | Ube2n | Stk11 | Tgfb1 | |  |
| Zap70 | Ung | Stoml2 | Tnfaip3 | |  |
| Zbtb1 | Usp12 | Stx4a | Tnfrsf1a | |  |
| Abl1 | Vamp8 | Stx7 | Tnfrsf1b | |  |
| Actr1a | Vav1 | Syk | Trp53 | |  |
| Adora2b | Wdfy1 | Tap2 | Uaca | |  |
| Ap1g1 | Wnt5a | Tec | Usp18 | |  |
| B2m | Xbp1 | Tfrc | Zap70 | |  |
| Bax | Zap70 | Thy1 | Zfp36 | |  |
| Bcar1 | Zbtb1 | Tlr12 |  | |  |
| Bcl10 | Abl1 | Tlr13 |  | |  |
| Bcl2 | Actr1a | Tlr2 |  | |  |
| Braf | Adora2b | Tlr3 |  | |  |
| C1qb | Ap1g1 | Tlr5 |  | |  |
| C1qbp | B2m | Tlr8 |  | |  |
| C2 | Bax | Tlr9 |  | |  |
| C3ar1 | Bcar1 | Tnfrsf21 |  | |  |
| Cav1 | Bcl10 | Tnfsf13b |  | |  |
| Cd14 | Bcl2 | Tnfsf4 |  | |  |
| Cd19 | Braf | Tnip1 |  | |  |
| Cd1d1 | C1qb | Tnip2 |  | |  |
| Cd24a | C1qbp | Traf3 |  | |  |
| Cd3e | C2 | Traf6 |  | |  |
| Cd4 | Cav1 | Ube2n |  | |  |
| Cd44 | Cd14 | Ung |  | |  |
| Cd79a | Cd19 | Usp12 |  | |  |
| Cfi | Cd1d1 | Vamp8 |  | |  |
| Cnr1 | Cd24a | Vav1 |  | |  |
| Colec12 | Cd3e | Wdfy1 |  | |  |
| Crhr1 | Cd4 | Wnt5a |  | |  |
| Crtam | Cd44 | Xbp1 |  | |  |
| Cst11 | Cfi | Zap70 |  | |  |
| Cyld | Colec12 | Zbtb1 |  | |  |
| Ddx58 | Crhr1 | Abl1 |  | |  |
| Dhx15 | Crtam | Actr1a |  | |  |
| Exosc3 | Cst11 | Adora2b |  | |  |
| F2rl1 | Cyld | Ap1g1 |  | |  |
| Fadd | Ddx58 | B2m |  | |  |
| Fcer1a | Dhx15 | Bax |  | |  |
| Fcgr1 | Exosc3 | Bcar1 |  | |  |
| Fcna | F2rl1 | Bcl10 |  | |  |
| Fcnb | Fadd | Bcl2 |  | |  |
| Fgr | Fcer1a | Braf |  | |  |
| Flot1 | Fcgr1 | C1qa |  | |  |
| Gab2 | Fcna | C1qb |  | |  |
| Gata2 | Fcnb | C1qbp |  | |  |
| Gc | Fgr | C2 |  | |  |
| Gpr33 | Flot1 | C3 |  | |  |
| Gpx4 | Gata2 | C3ar1 |  | |  |
| H2-M3 | Gc | Cav1 |  | |  |
| Havcr2 | Gpr33 | Cd14 |  | |  |
| Hmgb1 | Gpx4 | Cd19 |  | |  |
| Hmgb2 | H2-M3 | Cd1d1 |  | |  |
| Hspa8 | Havcr2 | Cd24a |  | |  |
| Il4ra | Hmgb1 | Cd3e |  | |  |
| Irak1 | Hmgb2 | Cd4 |  | |  |
| Irak2 | Hspa8 | Cd44 |  | |  |
| Irak3 | Il4ra | Cfi |  | |  |
| Irak4 | Irak1 | Cnr1 |  | |  |
| Irf3 | Irak2 | Colec12 |  | |  |
| Irf7 | Irak3 | Crhr1 |  | |  |
| Kars | Irak4 | Crtam |  | |  |
| Klk5 | Irf3 | Cyba |  | |  |
| Klrb1c | Irf7 | Cyld |  | |  |
| Klre1 | Kars | Ddx58 |  | |  |
| Klrk1 | Klk5 | Dhx15 |  | |  |
| Lamp1 | Klrb1c | Exosc3 |  | |  |
| Lbp | Klre1 | F2rl1 |  | |  |
| Lck | Lamp1 | Fadd |  | |  |
| Lcp1 | Lbp | Fcer1a |  | |  |
| Lcp2 | Lck | Fcgr1 |  | |  |
| Lgals9 | Lcp1 | Fcna |  | |  |
| Lrrfip2 | Lgals9 | Fcnb |  | |  |
| Ltf | Lrrfip2 | Fgr |  | |  |
| Ly96 | Ltf | Flot1 |  | |  |
| Malt1 | Ly96 | Gata2 |  | |  |
| Mapk1 | Malt1 | Gata3 |  | |  |
| Mapk3 | Mapk1 | Gpr33 |  | |  |
| Mapkapk3 | Mapk3 | Gpx4 |  | |  |
| Masp1 | Mapkapk3 | H2-M3 |  | |  |
| Mef2c | Masp1 | Havcr2 |  | |  |
| Mif | Mef2c | Hmgb1 |  | |  |
| Mlh1 | Mif | Hmgb2 |  | |  |
| Mmp12 | Mlh1 | Hspa8 |  | |  |
| Mmp2 | Mmp12 | Ifih1 |  | |  |
| Msh2 | Mmp2 | Irak1 |  | |  |
| Myb | Msh2 | Irak2 |  | |  |
| Myd88 | Myb | Irak4 |  | |  |
| Nfatc2 | Myd88 | Irf3 |  | |  |
| Nfkbia | Nfatc2 | Irf7 |  | |  |
| Nr4a3 | Nfkbia | Kars |  | |  |
| Nras | Nr4a3 | Klk5 |  | |  |
| P2rx7 | Nras | Klrb1c |  | |  |
| Park7 | P2rx7 | Klre1 |  | |  |
| Pde4b | Park7 | Lamp1 |  | |  |
| Peli1 | Pde4b | Lbp |  | |  |
| Phb | Peli1 | Lcp1 |  | |  |
| Pik3ap1 | Phb | Lgals9 |  | |  |
| Plcg1 | Pik3ap1 | Lrrfip2 |  | |  |
| Plcg2 | Plcg1 | Ltf |  | |  |
| Pld2 | Plcg2 | Ly96 |  | |  |
| Pms2 | Pms2 | Malt1 |  | |  |
| Pnp | Pnp | Mapk1 |  | |  |
| Polr3b | Polr3b | Mapk3 |  | |  |
| Polr3c | Polr3c | Mapkapk3 |  | |  |
| Polr3d | Polr3d | Masp1 |  | |  |
| Polr3f | Polr3f | Mef2c |  |  | |
| Prkch | Prkch | Mif |  | |  |
| Prkd2 | Prkd2 | Mlh1 |  | |  |
| Psen1 | Psen1 | Mmp12 |  | |  |
| Pum2 | Psen2 | Mmp2 |  | |  |
| Pycard | Pum2 | Msh2 |  | |  |
| Rap1a | Pycard | Myb |  | |  |
| Rapgef1 | Rap1a | Myd88 |  | |  |
| Rasgrp1 | Rapgef1 | Nfatc2 |  | |  |
| Rela | Rasgrp1 | Nfkbia |  | |  |
| Rnf31 | Rela | Nr4a3 |  | |  |
| Rps19 | Rnf31 | Nras |  | |  |
| Rps3 | Rps19 | P2rx7 |  | |  |
| Rsad2 | Rps3 | Park7 |  | |  |
| Serping1 | Rsad2 | Pde4b |  | |  |
| Shb | Serping1 | Peli1 |  | |  |
| Sin3a | Shb | Phb |  | |  |
| Slamf6 | Sin3a | Pik3ap1 |  | |  |
| Slc11a1 | Slamf6 | Plcg1 |  | |  |
| Slc39a10 | Slc11a1 | Plcg2 |  | |  |
| Snx4 | Slc39a10 | Pld2 |  | |  |
| Spon2 | Snx4 | Pms2 |  | |  |
| Stat5a | Socs5 | Pnp |  | |  |
| Stat5b | Spon2 | Polr3b |  | |  |
| Stat6 | Stat5a | Polr3d |  | |  |
| Stk11 | Stat5b | Polr3f |  | |  |
| Stoml2 | Stat6 | Prkch |  | |  |
| Stx4a | Stk11 | Prkd2 |  | |  |
| Stx7 | Stoml2 | Psen1 |  | |  |
| Syk | Stx4a | Psen2 |  | |  |
| Tap2 | Stx7 | Pum2 |  | |  |
| Tec | Syk | Pycard |  | |  |
| Tfrc | Tap2 | Rap1a |  | |  |
| Tgfb1 | Tec | Rapgef1 |  | |  |
| Thy1 | Tfrc | Rasgrp1 |  | |  |
| Tlr12 | Tgfb1 | Rela |  | |  |
| Tlr2 | Thy1 | Rnf31 |  | |  |
| Tlr4 | Tlr12 | Rps19 |  | |  |
| Tlr5 | Tlr13 | Rps3 |  | |  |
| Tlr9 | Tlr2 | Rsad2 |  | |  |
| Tnfrsf21 | Tlr5 | S100a14 |  | |  |
| Tnfsf13b | Tlr9 | Serping1 |  | |  |
| Tnfsf4 | Tnfrsf21 | Shb |  | |  |
| Tnip1 | Tnfsf13b | Sin3a |  | |  |
| Tnip2 | Tnip1 | Slamf6 |  | |  |
| Traf3 | Tnip2 | Slc11a1 |  | |  |
| Traf6 | Traf3 | Slc39a10 |  | |  |
| Ube2n | Traf6 | Snx4 |  | |  |
| Unc93b1 | Ube2n | Spon2 |  | |  |
| Ung | Unc93b1 | Stat5a |  | |  |
| Usp12 | Ung | Stat5b |  | |  |
| Vamp8 | Usp12 | Stat6 |  | |  |
| Vav1 | Vamp8 | Stk11 |  | |  |
| Wdfy1 | Vav1 | Stoml2 |  | |  |
| Wnt5a | Wdfy1 | Stx4a |  | |  |
| Xbp1 | Wnt5a | Stx7 |  | |  |
| Zap70 | Xbp1 | Syk |  | |  |
| Zbtb1 | Zap70 | Tap2 |  | |  |
| Adora1 | Zbtb1 | Tec |  | |  |
| Adora2a | Acp5 | Tfrc |  | |  |
| Apod | Adora2a | Thy1 |  | |  |
| Apoe | Adrb2 | Tlr12 |  | |  |
| Ash1l | Apod | Tlr13 |  | |  |
| Bcr | Apoe | Tlr2 |  | |  |
| Cd44 | Ash1l | Tlr3 |  | |  |
| Cuedc2 | Bax | Tlr5 |  | |  |
| Fcgr2b | Bcr | Tlr9 |  | |  |
| Fem1a | Calcrl | Tnfrsf21 |  | |  |
| Fn1 | Cd22 | Tnfsf13b |  | |  |
| Gpx1 | Cd4 | Tnfsf4 |  | |  |
| Ier3 | Cd44 | Tnip1 |  | |  |
| Il22ra2 | Cd80 | Tnip2 |  | |  |
| Lgals9 | Chrna7 | Traf3 |  | |  |
| Mefv | Cnr2 | Traf6 |  | |  |
| Metrnl | Cuedc2 | Ube2n |  | |  |
| Ndfip1 | Cxcl16 | Ung |  | |  |
| Nt5e | Cybb | Usp12 |  | |  |
| Pbk | Dusp10 | Vamp8 |  | |  |
| Psma1 | Ets1 | Vav1 |  | |  |
| Psmb4 | Fcgr2b | Wdfy1 |  | |  |
| Ptgds | Fem1a | Wnt5a |  | |  |
| Ptgis | Gpx1 | Xbp1 |  | |  |
| Ptpn2 | Hamp | Zap70 |  | |  |
| Rabgef1 | Havcr2 | Zbtb1 |  | |  |
| Rps19 | Hmgb1 | Acp5 |  | |  |
| Serpinf1 | Ier3 | Adora1 |  | |  |
| Slit2 | Itgal | Adora2a |  | |  |
| Smad3 | Lgals9 | Adrb2 |  | |  |
| Spn | Ltbr | Apod |  | |  |
| Tnfaip3 | Mapk1 | Apoe |  | |  |
| Tnfrsf1a | Mapk3 | Ash1l |  | |  |
| Tnfrsf1b | Mefv | Bcr |  | |  |
| Uaca | Metrnl | Calcrl |  | |  |
| Wfdc1 | Msn | Cd44 |  | |  |
| Zfp36 | Myd88 | Chrna7 |  | |  |
| Acp5 | Ndfip1 | Cnr2 |  | |  |
| Adora2a | Nt5e | Cuedc2 |  | |  |
| Adrb2 | Pbk | Dusp10 |  | |  |
| Apod | Pparg | Ets1 |  | |  |
| Apoe | Prf1 | Fcgr2b |  | |  |
| Ash1l | Prnp | Fem1a |  | |  |
| Bcr | Psma1 | Fn1 |  | |  |
| Calcrl | Psmb4 | Gata3 |  | |  |
| Cd44 | Pten | Gpx1 |  | |  |
| Chrna7 | Ptgis | Hamp |  | |  |
| Cnr2 | Ptpn2 | Ier3 |  | |  |
| Cuedc2 | Rabgef1 | Il22ra2 |  | |  |
| Dusp10 | Rap1a | Il4 |  | |  |
| Ets1 | Rps19 | Lgals9 |  | |  |
| Fcgr2b | Serpinf1 | Mefv |  | |  |
| Fem1a | Slit2 | Metrnl |  | |  |
| Fn1 | Smad3 | Ndfip1 |  | |  |
| Gpx1 | Smpdl3b | Nt5e |  | |  |
| Hamp | Spn | Pbk |  | |  |
| Ier3 | Stat1 | Pparg |  | |  |
| Il22ra2 | Stat3 | Psma1 |  | |  |
| Lgals9 | Stat5a | Psmb4 |  | |  |
| Mefv | Stat5b | Ptgds |  | |  |
| Metrnl | Stk11 | Ptgis |  | |  |
| Ndfip1 | Tff2 | Ptpn2 |  | |  |
| Nt5e | Tgfb1 | Rabgef1 |  | |  |
| Pbk | Tnfaip3 | Rps19 |  | |  |
| Pparg | Tnfrsf1a | Serpina3k |  | |  |
| Psma1 | Trp53 | Serpinb1a |  | |  |
| Psmb4 | Wfdc1 | Serpinf1 |  | |  |
| Ptgds | Zap70 | Slit2 |  | |  |
| Ptgis | Zfp36 | Smad3 |  | |  |
| Ptpn2 | Acp5 | Socs5 |  | |  |
| Rabgef1 | Adora2a | Spn |  | |  |
| Rps19 | Adrb2 | Tff2 |  | |  |
| Serpina3k | Apod | Tnfaip3 |  | |  |
| Serpinb1a | Apoe | Tnfrsf1a |  | |  |
| Serpinf1 | Ash1l | Tnfrsf1b |  | |  |
| Slit2 | Bcr | Uaca |  | |  |
| Smad3 | Calcrl | Wfdc1 |  | |  |
| Smpdl3b | Cd44 | Zfp36 |  | |  |
| Spn | Chrna7 | Acp5 |  | |  |
| Tff2 | Cnr2 | Adora1 |  | |  |
| Tnfaip3 | Cuedc2 | Adora2a |  | |  |
| Tnfrsf1a | Dusp10 | Adrb2 |  | |  |
| Tnfrsf1b | Ets1 | Apod |  | |  |
| Uaca | Fcgr2b | Apoe |  | |  |
| Wfdc1 | Fem1a | Ash1l |  | |  |
| Zfp36 | Fn1 | Bcr |  | |  |
| Acp5 | Gpx1 | Calcrl |  | |  |
| Adora2a | Hamp | Cd44 |  | |  |
| Adrb2 | Lgals9 | Chrna7 |  | |  |
| Apod | Mefv | Cnr2 |  | |  |
| Apoe | Metrnl | Cuedc2 |  | |  |
| Ash1l | Ndfip1 | Dusp10 |  | |  |
| Bcr | Nt5e | Ets1 |  | |  |
| Calcrl | Pbk | Fcgr2b |  | |  |
| Cd44 | Pparg | Fem1a |  | |  |
| Chrna7 | Psma1 | Fn1 |  | |  |
| Cnr2 | Psmb4 | Gata3 |  | |  |
| Cuedc2 | Ptgds | Gpx1 |  | |  |
| Dusp10 | Ptgis | Ier3 |  | |  |
| Ets1 | Ptpn2 | Il22ra2 |  | |  |
| Fcgr2b | Rabgef1 | Lgals9 |  | |  |
| Fem1a | Rps19 | Mefv |  | |  |
| Fn1 | Serpina3k | Metrnl |  | |  |
| Gpx1 | Serpinb1a | Ndfip1 |  | |  |
| Hamp | Serpinf1 | Nt5e |  | |  |
| Ier3 | Slit2 | Pbk |  | |  |
| Il22ra2 | Smad3 | Pparg |  | |  |
| Lgals9 | Smpdl3b | Psma1 |  | |  |
| Mefv | Spn | Psmb4 |  | |  |
| Metrnl | Tff2 | Ptgds |  | |  |
| Ndfip1 | Tnfaip3 | Ptgis |  | |  |
| Nt5e | Tnfrsf1a | Ptpn2 |  | |  |
| Pbk | Uaca | Rabgef1 |  | |  |
| Pparg | Wfdc1 | Rps19 |  | |  |
| Psma1 | Acp5 | Serpinb1a |  | |  |
| Psmb4 | Adora2a | Serpinf1 |  | |  |
| Ptgds | Adrb2 | Slit2 |  | |  |
| Ptgis | Apod | Smad3 |  | |  |
| Ptpn2 | Apoe | Socs5 |  | |  |
| Rabgef1 | Ash1l | Spn |  | |  |
| Rps19 | Bcr | Tff2 |  | |  |
| Serpina3k | Calcrl | Tnfaip3 |  | |  |
| Serpinb1a | Cd44 | Tnfrsf1a |  | |  |
| Serpinf1 | Chrna7 | Tnfrsf1b |  | |  |
| Slit2 | Cnr2 | Wfdc1 |  | |  |
| Smad3 | Cuedc2 | Zfp36 |  | |  |
| Smpdl3b | Dusp10 | Acp5 |  | |  |
| Spn | Ets1 | Adora1 |  | |  |
| Tff2 | Fcgr2b | Adora2a |  | |  |
| Tnfaip3 | Fem1a | Adrb2 |  | |  |
| Tnfrsf1a | Fn1 | Apod |  | |  |
| Tnfrsf1b | Gpx1 | Apoe |  | |  |
| Uaca | Hamp | Ash1l |  | |  |
| Wfdc1 | Ier3 | Bcr |  | |  |
| Zfp36 | Il22ra2 | Calcrl |  | |  |
| Adora1 | Lgals9 | Cd44 |  | |  |
| Adora2a | Mefv | Chrna7 |  | |  |
| Apod | Metrnl | Cnr2 |  | |  |
| Apoe | Ndfip1 | Cuedc2 |  | |  |
| Ash1l | Nt5e | D1Ertd622e |  | |  |
| Bax | Pbk | Dusp10 |  | |  |
| Bcr | Pparg | Ets1 |  | |  |
| Ccl2 | Psma1 | Fcgr2b |  | |  |
| Cd22 | Psmb4 | Fem1a |  | |  |
| Cd44 | Ptgds | Fn1 |  | |  |
| Cd80 | Ptgis | Gata3 |  | |  |
| Cuedc2 | Ptpn2 | Gpx1 |  | |  |
| Cxcl16 | Rabgef1 | Ier3 |  | |  |
| Cybb | Rps19 | Il22ra2 |  | |  |
| Fcgr2b | Serpina3k | Lgals9 |  | |  |
| Fem1a | Serpinb1a | Mefv |  | |  |
| Gpx1 | Serpinf1 | Metrnl |  | |  |
| Hmgb1 | Slit2 | Ndfip1 |  | |  |
| Ier3 | Smad3 | Nt5e |  | |  |
| Il22ra2 | Smpdl3b | Pbk |  | |  |
| Itgal | Socs5 | Pparg |  | |  |
| Lgals9 | Tff2 | Psma1 |  | |  |
| Ltbr | Tnfaip3 | Psmb4 |  | |  |
| Mapk1 | Tnfrsf1a | Ptgds |  | |  |
| Mapk3 | Tnfrsf1b | Ptgis |  | |  |
| Mefv | Uaca | Ptpn2 |  | |  |
| Metrnl | Wfdc1 | Rabgef1 |  | |  |
| Msn | Zfp36 | Rps19 |  | |  |
| Myd88 | Acp5 | Serpinb1a |  | |  |
| Ndfip1 | Adora2a | Serpinf1 |  | |  |
| Nt5e | Adrb2 | Slit2 |  | |  |
| Pbk | Apod | Smad3 |  | |  |
| Prf1 | Apoe | Spn |  | |  |
| Prnp | Ash1l | Tff2 |  | |  |
| Psma1 | Bax | Tnfaip3 |  | |  |
| Psmb4 | Bcr | Tnfrsf1a |  | |  |
| Pten | Calcrl | Uaca |  | |  |
| Ptgis | Cd22 | Wfdc1 |  | |  |
| Ptpn2 | Cd4 | Zfp36 |  | |  |
| Rabgef1 | Cd44 | Acp5 |  | |  |
| Rap1a | Cd80 | Adora1 |  | |  |
| Rps19 | Chrna7 | Adora2a |  | |  |
| Serpinf1 | Cnr2 | Adrb2 |  | |  |
| Slit2 | Cuedc2 | Apod |  | |  |
| Smad3 | Cxcl16 | Apoe |  | |  |
| Spn | Cybb | Ash1l |  | |  |
| Stat1 | Dusp10 | Bax |  | |  |
| Stat3 | Ets1 | Bcr |  | |  |
| Stat5b | Fcgr2b | Calcrl |  | |  |
| Stk11 | Fem1a | Cd22 |  | |  |
| Tgfb1 | Gpx1 | Cd4 |  | |  |
| Tnfaip3 | Hamp | Cd44 |  | |  |
| Tnfrsf1a | Havcr2 | Cd80 |  | |  |
| Tnfrsf1b | Hmgb1 | Chrna7 |  | |  |
| Trp53 | Ier3 | Cnr2 |  | |  |
| Uaca | Itgal | Cuedc2 |  | |  |
| Usp18 | Lgals9 | Cxcl16 |  | |  |
| Vip | Ltbr | Cybb |  | |  |
| Wfdc1 | Mapk1 | Dusp10 |  | |  |
| Zap70 | Mapk3 | Ets1 |  | |  |
| Zfp36 | Mefv | Fcgr2b |  | |  |
| Acp5 | Metrnl | Fem1a |  | |  |
| Adora2a | Msn | Gata3 |  | |  |
| Adrb2 | Myd88 | Gpx1 |  | |  |
| Apod | Ndfip1 | Hamp |  | |  |
| Apoe | Nt5e | Havcr2 |  | |  |
| Ash1l | Pbk | Hmgb1 |  | |  |
| Bax | Pparg | Ier3 |  | |  |
| Bcr | Prf1 | Il22ra2 |  | |  |
| Calcrl | Prnp | Il4 |  | |  |
| Cd22 | Psma1 | Itgal |  | |  |
| Cd4 | Psmb4 | Lgals9 |  | |  |
| Cd44 | Pten | Ltbr |  | |  |
| Cd80 | Ptgis | Mapk1 |  | |  |
| Chrna7 | Ptpn2 | Mapk3 |  | |  |
| Cnr2 | Rabgef1 | Mefv |  | |  |
| Cuedc2 | Rap1a | Metrnl |  | |  |
| Cxcl16 | Rps19 | Msn |  | |  |
| Cybb | Serpinf1 | Myd88 |  | |  |
| Dusp10 | Slit2 | Ndfip1 |  | |  |
| Ets1 | Smad3 | Nt5e |  | |  |
| Fcgr2b | Smpdl3b | Pbk |  | |  |
| Fem1a | Spn | Pparg |  | |  |
| Gpx1 | Stat1 | Prf1 |  | |  |
| Hamp | Stat3 | Prnp |  | |  |
| Havcr2 | Stat5a | Psma1 |  | |  |
| Hmgb1 | Stat5b | Psmb4 |  | |  |
| Ier3 | Stk11 | Pten |  | |  |
| Il22ra2 | Tff2 | Ptgis |  | |  |
| Itgal | Tgfb1 | Ptpn2 |  | |  |
| Lgals9 | Tnfaip3 | Rabgef1 |  | |  |
| Ltbr | Tnfrsf1a | Rap1a |  | |  |
| Mapk1 | Trp53 | Rps19 |  | |  |
| Mapk3 | Wfdc1 | Serpinf1 |  | |  |
| Mefv | Zap70 | Slit2 |  | |  |
| Metrnl | Zfp36 | Smad3 |  | |  |
| Msn | Acp5 | Socs5 |  | |  |
| Myd88 | Adora2a | Spn |  | |  |
| Ndfip1 | Adrb2 | Stat1 |  | |  |
| Nt5e | Apod | Stat3 |  | |  |
| Pbk | Apoe | Stat5a |  | |  |
| Pparg | Ash1l | Stat5b |  | |  |
| Prnp | Bax | Stk11 |  | |  |
| Psma1 | Bcr | Tff2 |  | |  |
| Psmb4 | Calcrl | Tnfaip3 |  | |  |
| Pten | Cd22 | Tnfrsf1a |  | |  |
| Ptgis | Cd4 | Tnfrsf1b |  | |  |
| Ptpn2 | Cd44 | Trp53 |  | |  |
| Rabgef1 | Cd80 | Uaca |  | |  |
| Rap1a | Chrna7 | Usp18 |  | |  |
| Rps19 | Cnr2 | Wfdc1 |  | |  |
| Serpinf1 | Cuedc2 | Zap70 |  | |  |
| Slit2 | Cxcl16 | Zfp36 |  | |  |
| Smad3 | Cybb | Acp5 |  | |  |
| Smpdl3b | Dusp10 | Adora1 |  | |  |
| Spn | Ets1 | Adora2a |  | |  |
| Stat1 | Fcgr2b | Adrb2 |  | |  |
| Stat3 | Fem1a | Apod |  | |  |
| Stat5a | Gpx1 | Apoe |  | |  |
| Stat5b | Hamp | Ash1l |  | |  |
| Stk11 | Havcr2 | Bax |  | |  |
| Tff2 | Hmgb1 | Bcr |  | |  |
| Tgfb1 | Itgal | Calcrl |  | |  |
| Tnfaip3 | Lgals9 | Cd22 |  | |  |
| Tnfrsf1a | Ltbr | Cd4 |  | |  |
| Tnfrsf1b | Mapk1 | Cd44 |  | |  |
| Trp53 | Mapk3 | Cd80 |  | |  |
| Uaca | Mefv | Chrna7 |  | |  |
| Usp18 | Metrnl | Cnr2 |  | |  |
| Wfdc1 | Msn | Cuedc2 |  | |  |
| Zap70 | Myd88 | Cxcl16 |  | |  |
| Zfp36 | Ndfip1 | Cybb |  | |  |
| Acp5 | Nt5e | Dusp10 |  | |  |
| Adora2a | Pbk | Ets1 |  | |  |
| Adrb2 | Pparg | Fcgr2b |  | |  |
| Apod | Prf1 | Fem1a |  | |  |
| Apoe | Prnp | Gata3 |  | |  |
| Ash1l | Psma1 | Gpx1 |  | |  |
| Bax | Psmb4 | Havcr2 |  | |  |
| Bcr | Pten | Hmgb1 |  | |  |
| Calcrl | Ptgis | Ier3 |  | |  |
| Cd22 | Ptpn2 | Il22ra2 |  | |  |
| Cd4 | Rabgef1 | Itgal |  | |  |
| Cd44 | Rap1a | Lgals9 |  | |  |
| Cd80 | Rps19 | Ltbr |  | |  |
| Chrna7 | Serpinf1 | Mapk1 |  | |  |
| Cnr2 | Slit2 | Mapk3 |  | |  |
| Cuedc2 | Smad3 | Mefv |  | |  |
| Cxcl16 | Smpdl3b | Metrnl |  | |  |
| Cybb | Spn | Msn |  | |  |
| Dusp10 | Stat1 | Myd88 |  | |  |
| Ets1 | Stat3 | Ndfip1 |  | |  |
| Fcgr2b | Stat5a | Nt5e |  | |  |
| Fem1a | Stat5b | Pbk |  | |  |
| Gpx1 | Stk11 | Pparg |  | |  |
| Hamp | Tff2 | Prnp |  | |  |
| Havcr2 | Tlr4 | Psma1 |  | |  |
| Hmgb1 | Tnfaip3 | Psmb4 |  | |  |
| Ier3 | Tnfrsf1a | Pten |  | |  |
| Il22ra2 | Trp53 | Ptgis |  | |  |
| Itgal | Uaca | Ptpn2 |  | |  |
| Lgals9 | Usp18 | Rabgef1 |  | |  |
| Ltbr | Wfdc1 | Rap1a |  | |  |
| Mapk1 | Zap70 | Rps19 |  | |  |
| Mapk3 | Zfp36 | Serpinf1 |  | |  |
| Mefv | Acp5 | Slit2 |  | |  |
| Metrnl | Adora2a | Smad3 |  | |  |
| Msn | Adrb2 | Socs5 |  | |  |
| Myd88 | Apod | Spn |  | |  |
| Ndfip1 | Apoe | Stat3 |  | |  |
| Nt5e | Ash1l | Stat5a |  | |  |
| Pbk | Bax | Stat5b |  | |  |
| Pparg | Bcr | Stk11 |  | |  |
| Prf1 | Calcrl | Tff2 |  | |  |
| Prnp | Cd22 | Tnfaip3 |  | |  |
| Psma1 | Cd4 | Tnfrsf1a |  | |  |
| Psmb4 | Cd44 | Tnfrsf1b |  | |  |
| Pten | Cd80 | Usp18 |  | |  |
| Ptgis | Chrna7 | Wfdc1 |  | |  |
| Ptpn2 | Cnr2 | Zap70 |  | |  |
| Rabgef1 | Cuedc2 | Zfp36 |  | |  |
| Rap1a | Cxcl16 | Acp5 |  | |  |
| Rps19 | Cybb | Adora1 |  | |  |
| Serpinf1 | Dusp10 | Adora2a |  | |  |
| Slit2 | Ets1 | Adrb2 |  | |  |
| Smad3 | Fcgr2b | Apod |  | |  |
| Smpdl3b | Fem1a | Apoe |  | |  |
| Spn | Gpx1 | Ash1l |  | |  |
| Stat1 | Hamp | Bax |  | |  |
| Stat3 | Havcr2 | Bcr |  | |  |
| Stat5a | Hmgb1 | Calcrl |  | |  |
| Stat5b | Ier3 | Cd22 |  | |  |
| Stk11 | Il22ra2 | Cd4 |  | |  |
| Tff2 | Itgal | Cd44 |  | |  |
| Tgfb1 | Lgals9 | Cd80 |  | |  |
| Tlr4 | Ltbr | Chrna7 |  | |  |
| Tnfaip3 | Mapk1 | Cnr2 |  | |  |
| Tnfrsf1a | Mapk3 | Cuedc2 |  | |  |
| Tnfrsf1b | Mefv | Cxcl16 |  | |  |
| Trp53 | Metrnl | Cybb |  | |  |
| Uaca | Msn | D1Ertd622e |  | |  |
| Usp18 | Myd88 | Dusp10 |  | |  |
| Wfdc1 | Ndfip1 | Ets1 |  | |  |
| Zap70 | Nt5e | Fcgr2b |  | |  |
| Zfp36 | Pbk | Fem1a |  | |  |
|  | Pparg | Gata3 |  | |  |
|  | Prf1 | Gpx1 |  | |  |
|  | Prnp | Havcr2 |  | |  |
|  | Psma1 | Hmgb1 |  | |  |
|  | Psmb4 | Ier3 |  | |  |
|  | Pten | Il22ra2 |  | |  |
|  | Ptgis | Itgal |  | |  |
|  | Ptpn2 | Lgals9 |  | |  |
|  | Rabgef1 | Ltbr |  | |  |
|  | Rap1a | Mapk1 |  | |  |
|  | Rps19 | Mapk3 |  |  | |
|  | Serpinf1 | Mefv |  |  | |
|  | Slit2 | Metrnl |  |  | |
|  | Smad3 | Msn |  |  | |
|  | Smpdl3b | Myd88 |  |  | |
|  | Socs5 | Ndfip1 |  |  | |
|  | Stat1 | Nt5e |  |  | |
|  | Stat3 | Pbk |  |  | |
|  | Stat5a | Pparg |  |  | |
|  | Stat5b | Prnp |  |  | |
|  | Stk11 | Psma1 |  |  | |
|  | Tff2 | Psmb4 |  |  | |
|  | Tgfb1 | Pten |  |  | |
|  | Tnfaip3 | Ptgis |  |  | |
|  | Tnfrsf1a | Ptpn2 |  |  | |
|  | Tnfrsf1b | Rabgef1 |  |  | |
|  | Trp53 | Rap1a |  |  | |
|  | Uaca | Rps19 |  |  | |
|  | Usp18 | Serpinf1 |  |  | |
|  | Wfdc1 | Slit2 |  |  | |
|  | Zap70 | Smad3 |  |  | |
|  | Zfp36 | Spn |  |  | |
|  |  | Stat1 |  |  | |
|  |  | Stat3 |  |  | |
|  |  | Stat5a |  |  | |
|  |  | Stat5b |  |  | |
|  |  | Stk11 |  |  | |
|  |  | Tff2 |  |  | |
|  |  | Tnfaip3 |  |  | |
|  |  | Tnfrsf1a |  |  | |
|  |  | Trp53 |  |  | |
|  |  | Uaca |  |  | |
|  |  | Wfdc1 |  |  | |
|  |  | Zap70 |  |  | |
|  |  | Zfp36 |  |  | |
