# Supplementary material for: Proton-Secreting Cells as Drivers of Inflammation and Sperm Dysfunction in LPS-Induced Epididymitis
Source: Function (Oxf). 2025 Jun 2;6(3):zqaf023. doi: 10.1093/function/zqaf023 (PMC12203219; doi:10.1093/function/zqaf023)
Supplement: zqaf023_Supplemental_Files [file zqaf023_supplemental_files.zip › Suppl Table 3 23-05.docx]

| Seg 1,2,3 | Seg 4,5,6 | Seg 8,9,10 | Seg 7 |
| --- | --- | --- | --- |
| 1600014C10Rik | 1600014C10Rik | 1600014C10Rik | 1600014C10Rik |
| 1700009P17Rik | 1700009P17Rik | 1700009P17Rik | 1700009P17Rik |
| 1700034H14Rik | 1700012A03Rik | 1700012A03Rik | 1700012A03Rik |
| 2300009A05Rik | 1700034H14Rik | 1700034H14Rik | 1700034H14Rik |
| 4930415O20Rik | 2300009A05Rik | 2300009A05Rik | 2300009A05Rik |
| 4930471M23Rik | 4921507P07Rik | 4732415M23Rik | 4921507P07Rik |
| 5730469M10Rik | 4921530D09Rik | 4921507P07Rik | 4921530D09Rik |
| AI314180 | 4930415O20Rik | 4921530D09Rik | 4930415O20Rik |
| Abca3 | 4930471M23Rik | 4930415O20Rik | 4930471M23Rik |
| Abcc3 | 5730469M10Rik | 4930471M23Rik | 5730469M10Rik |
| Acat2 | 9430016H08Rik | 5730469M10Rik | 8430406I07Rik |
| Acbd5 | AI314180 | 8430406I07Rik | 9430016H08Rik |
| Aco1 | Abca3 | 9430016H08Rik | AI314180 |
| Acp1 | Abcc3 | AI314180 | Abca3 |
| Actn1 | Acat2 | Abca3 | Abcc3 |
| Actr3 | Acbd5 | Abcc3 | Acat2 |
| Acyp1 | Aco1 | Acat2 | Acbd5 |
| Adck5 | Acp1 | Acbd5 | Aco1 |
| Adh5 | Actn1 | Aco1 | Acp1 |
| Akap4 | Actr3 | Acp1 | Actn1 |
| Anp32b | Acyp1 | Actn1 | Actr3 |
| Ap2a1 | Adck5 | Actr3 | Acyp1 |
| Ap2s1 | Adh5 | Acyp1 | Adck5 |
| Aprt | Akap4 | Adck5 | Adh5 |
| Arl3 | Anp32b | Adh5 | Akap4 |
| Armc1 | Ap2a1 | Akap4 | Anp32b |
| Arpc4 | Ap2s1 | Anp32b | Anxa13 |
| Arpc5l | Aprt | Anxa13 | Ap2a1 |
| Atp11a | Arl3 | Ap2a1 | Ap2s1 |
| Atp13a4 | Armc1 | Ap2s1 | Aprt |
| Atp6v0c | Arpc4 | Aprt | Arl3 |
| BC051142 | Arpc5l | Arl3 | Armc1 |
| Bloc1s1 | Atp11a | Armc1 | Arpc4 |
| Bsg | Atp13a4 | Arpc4 | Arpc5l |
| Calm3 | Atp6v0c | Arpc5l | Atp11a |
| Cap1 | BC051142 | Atp11a | Atp13a4 |
| Capza1 | Bloc1s1 | Atp13a4 | Atp6v0c |
| Car2 | Bsg | Atp6v0c | BC051142 |
| Cbr3 | C030006K11Rik | BC051142 | Bloc1s1 |
| Cct7 | Calm3 | Bloc1s1 | Bsg |
| Cct8 | Cap1 | Bsg | C030006K11Rik |
| Chchd1 | Capza1 | C030006K11Rik | Cabyr |
| Clca2 | Car2 | Cabyr | Calm3 |
| Clic4 | Cbr3 | Calm3 | Cap1 |
| Coasy | Cct7 | Cap1 | Capza1 |
| Cops4 | Cct8 | Capza1 | Car2 |
| Cops8 | Chchd1 | Car2 | Car4 |
| Crot | Ckmt1 | Car4 | Cbr3 |
| Csnk2a1 | Clca2 | Cbr3 | Cct7 |
| Ctsf | Clic3 | Cct7 | Cct8 |
| Cutc | Clic4 | Cct8 | Chchd1 |
| Cyfip1 | Coasy | Chchd1 | Ckmt1 |
| Ddb1 | Cops4 | Ckmt1 | Clca2 |
| Ddt | Cops8 | Clca2 | Clic3 |
| Dnaja4 | Cox6b2 | Clic3 | Clic4 |
| Dnajb6 | Crisp2 | Clic4 | Coasy |
| Dnm2 | Crot | Coasy | Cops8 |
| Dock7 | Csnk2a1 | Cops4 | Cox6b2 |
| Dpysl2 | Ctsf | Cops8 | Crisp2 |
| Dtymk | Cyfip1 | Cox6b2 | Crot |
| Dusp3 | Cyp17a1 | Crisp2 | Csnk2a1 |
| Ebp | Ddb1 | Crot | Ctsf |
| Eef1d | Ddt | Csnk2a1 | Cyfip1 |
| Eef1g | Defb19 | Ctsf | Cyp17a1 |
| Ehd2 | Defb2 | Cyfip1 | Ddb1 |
| Eif2s1 | Dnaja4 | Cyp17a1 | Ddt |
| Eif5a | Dnajb6 | Ddb1 | Defb11 |
| Endog | Dnm2 | Ddt | Defb2 |
| Eprs | Dock7 | Defb11 | Dnaja4 |
| Erbb2 | Dpysl2 | Defb2 | Dnajb6 |
| Fdx1 | Dtymk | Dnajb6 | Dnm2 |
| Fhl1 | Dusp3 | Dnm2 | Dock7 |
| Fmo2 | Ebp | Dock7 | Dpysl2 |
| Fundc1 | Eef1d | Dpysl2 | Dtymk |
| Fxc1 | Eef1g | Dtymk | Dusp3 |
| Gaa | Ehd2 | Dusp3 | Ebp |
| Galntl4 | Eif2s1 | Ebp | Eef1d |
| Gba | Eif5a | Eef1d | Eef1g |
| Gclm | Eprs | Eef1g | Ehd2 |
| Ghitm | Erbb2 | Ehd2 | Eif2s1 |
| Gm2a | Fdx1 | Eif2s1 | Eif5a |
| Gpi1 | Fhl1 | Eif5a | Eno1 |
| Gpnmb | Fhl4 | Eprs | Eprs |
| Gpx1 | Fmo2 | Erbb2 | Erbb2 |
| Gpx3 | Fundc1 | Fdx1 | Fdx1 |
| Gpx4 | Fxc1 | Fhl1 | Fhl1 |
| Gpx5 | Gaa | Fhl4 | Fhl4 |
| Gsr | Galntl4 | Fmo2 | Fmo2 |
| Gstm1 | Gba | Fundc1 | Fundc1 |
| Gstm2 | Gclm | Fxc1 | Fxc1 |
| Gtl3 | Ghitm | Fxn | Gaa |
| Hdhd2 | Gm2a | Gaa | Galntl4 |
| Hint1 | Gpi1 | Galntl4 | Gba |
| Hrsp12 | Gpnmb | Gba | Gclm |
| Hsd3b7 | Gpx1 | Gclm | Ghitm |
| Htatip2 | Gpx3 | Ghitm | Gm2a |
| Idi1 | Gpx4 | Gm2a | Gpi1 |
| Ipo7 | Gpx5 | Gpi1 | Gpnmb |
| Isg20 | Gsr | Gpnmb | Gpx1 |
| Itpr1 | Gstm1 | Gpx1 | Gpx3 |
| Itpr3 | Gstm2 | Gpx3 | Gpx4 |
| Lactb | Gtl3 | Gpx4 | Gpx5 |
| Lamb2 | Hdhd2 | Gpx5 | Gsr |
| Lamc1 | Hint1 | Gsr | Gstm1 |
| Lamp2 | Hrsp12 | Gstm1 | Gstm2 |
| Lancl1 | Hsd3b7 | Gstm2 | Gtl3 |
| Lancl2 | Htatip2 | Gtl3 | Hdhd2 |
| Lars | Idi1 | Hdhd2 | Hint1 |
| Lass2 | Ipo7 | Hint1 | Hrsp12 |
| Lcn12 | Isg20 | Hrsp12 | Hsd3b7 |
| Lcn8 | Itpr1 | Hsd3b7 | Htatip2 |
| Lgals1 | Itpr3 | Htatip2 | Idi1 |
| Lipe | Klhl10 | Idi1 | Immp2l |
| Lipg | Lactb | Ipo7 | Ipo7 |
| Ly6a | Lamb2 | Isg20 | Isg20 |
| Lypla1 | Lamc1 | Itpr1 | Itpr1 |
| Man2a2 | Lamp2 | Itpr3 | Itpr3 |
| Manba | Lancl1 | Klhl10 | Klhl10 |
| Mdk | Lancl2 | Lactb | Lactb |
| Mipep | Lars | Lamc1 | Lamc1 |
| Mrpl11 | Lass2 | Lamp2 | Lamp2 |
| Mrpl17 | Lcn12 | Lancl1 | Lancl1 |
| Mrpl18 | Lcn8 | Lancl2 | Lancl2 |
| Mrpl43 | Lgals1 | Lars | Lars |
| Mrpl48 | Lipe | Lass2 | Lass2 |
| Mrpl52 | Lipg | Lcn12 | Lcn12 |
| Mrpl55 | Ly6a | Lcn8 | Lcn8 |
| Mrps10 | Lypla1 | Lgals1 | Lgals1 |
| Mrps14 | Man2a2 | Lipe | Lipe |
| Mrps16 | Manba | Lipg | Lipg |
| Mrps18a | Mdk | Ly6a | Ly6a |
| Mrps18b | Mipep | Lypla1 | Lypla1 |
| Mrps21 | Mlf1 | Man2a2 | Man2a2 |
| Mrps24 | Mrpl11 | Manba | Manba |
| Mrps31 | Mrpl17 | Mdk | Mdk |
| Mrps36 | Mrpl18 | Mipep | Mipep |
| Mrps6 | Mrpl43 | Mlf1 | Mrpl11 |
| Mrrf | Mrpl48 | Mrpl11 | Mrpl17 |
| Mtpn | Mrpl52 | Mrpl17 | Mrpl18 |
| Ncam1 | Mrpl55 | Mrpl18 | Mrpl43 |
| Ndufc2 | Mrps10 | Mrpl43 | Mrpl48 |
| Ndufs6 | Mrps14 | Mrpl48 | Mrpl52 |
| Ndufv2 | Mrps16 | Mrpl52 | Mrpl55 |
| Nif3l1 | Mrps18a | Mrpl55 | Mrps10 |
| Nit1 | Mrps18b | Mrps10 | Mrps14 |
| Nqo1 | Mrps21 | Mrps14 | Mrps16 |
| Oplah | Mrps24 | Mrps16 | Mrps18a |
| Pcbp1 | Mrps31 | Mrps18a | Mrps18b |
| Pex11c | Mrps36 | Mrps18b | Mrps21 |
| Pfkm | Mrps6 | Mrps21 | Mrps24 |
| Pgls | Mrrf | Mrps24 | Mrps31 |
| Phb | Mtpn | Mrps31 | Mrps36 |
| Phpt1 | Ncam1 | Mrps36 | Mrps6 |
| Pmm2 | Ndufc2 | Mrps6 | Mrrf |
| Ppap2a | Ndufs6 | Mrrf | Mtpn |
| Ppil1 | Ndufv2 | Mtpn | Ncam1 |
| Ppp2r1b | Nif3l1 | Ncam1 | Ndufc2 |
| Ppp2r4 | Nit1 | Ndufc2 | Ndufs6 |
| Ppp6c | Nqo1 | Ndufs6 | Ndufv2 |
| Prkacb | Oplah | Ndufv2 | Nif3l1 |
| Psen1 | Pcbp1 | Nif3l1 | Nit1 |
| Psma1 | Pex11c | Nit1 | Nme5 |
| Psma2 | Pfkm | Nqo1 | Nqo1 |
| Psma6 | Pgk2 | Oplah | Oplah |
| Psma7 | Pgls | Pcbp1 | Pcbp1 |
| Psmb1 | Phb | Pex11c | Pex11c |
| Psmb5 | Phpt1 | Pfkm | Pfkm |
| Psmb6 | Pmm2 | Pgk2 | Pgk2 |
| Psmd7 | Ppap2a | Pgls | Pgls |
| Psme1 | Ppil1 | Phb | Phb |
| Psme4 | Ppp2r1b | Phpt1 | Phpt1 |
| Ptcd2 | Ppp2r4 | Pmm2 | Pmm2 |
| Ptges | Ppp6c | Ppap2a | Ppap2a |
| Ptp4a2 | Prkacb | Ppil1 | Ppil1 |
| Rab24 | Psen1 | Ppp2r1b | Ppp2r1b |
| Rab28 | Psma1 | Ppp2r4 | Ppp2r4 |
| Rab5b | Psma2 | Ppp6c | Ppp6c |
| Rac2 | Psma6 | Prkacb | Prkacb |
| Rdh13 | Psma7 | Psen1 | Psen1 |
| Recql | Psmb1 | Psma1 | Psma1 |
| Rpia | Psmb5 | Psma2 | Psma2 |
| Rpl37a | Psmb6 | Psma6 | Psma6 |
| Rpl5 | Psmd7 | Psma7 | Psma7 |
| Rplp2 | Psme1 | Psmb1 | Psmb1 |
| Rps10 | Psme4 | Psmb5 | Psmb5 |
| Rps13 | Ptcd2 | Psmb6 | Psmb6 |
| Rps19 | Ptges | Psmd7 | Psmd7 |
| Rps27 | Ptp4a2 | Psme1 | Psme1 |
| Rrbp1 | Rab24 | Psme4 | Psme4 |
| S100a16 | Rab28 | Ptcd2 | Ptcd2 |
| Sbds | Rab2b | Ptges | Ptges |
| Scarb2 | Rab5b | Ptp4a2 | Ptp4a2 |
| Sco1 | Rac2 | Rab24 | Rab24 |
| Sdf2l1 | Recql | Rab28 | Rab28 |
| Sdhc | Ribc1 | Rab2b | Rab2b |
| Sdhd | Rpia | Rab5b | Rab5b |
| Sec23ip | Rpl37a | Rac2 | Rac2 |
| Slc12a9 | Rpl5 | Rdh13 | Rdh13 |
| Slc2a5 | Rplp2 | Recql | Rdh9 |
| Slc35f2 | Rps10 | Ropn1l | Recql |
| Slco4c1 | Rps13 | Rpia | Rpia |
| Snx1 | Rps19 | Rpl37a | Rpl37a |
| Spata6 | Rps27 | Rpl5 | Rpl5 |
| Spnb3 | Rrbp1 | Rplp2 | Rplp2 |
| Stx17 | S100a16 | Rps10 | Rps10 |
| Syne2 | Sbds | Rps13 | Rps13 |
| Tcp1 | Scarb2 | Rps19 | Rps19 |
| Tcte1 | Sco1 | Rps27 | Rps27 |
| Tpm3 | Sdf2l1 | Rrbp1 | Rrbp1 |
| Tpmt | Sdhc | S100a16 | S100a16 |
| Tpp2 | Sdhd | Sbds | Sbds |
| Tram1 | Sec23ip | Scarb2 | Scarb2 |
| Trappc3 | Slc12a9 | Sco1 | Sco1 |
| Trappc4 | Slc16a1 | Sdf2l1 | Sdf2l1 |
| Tsta3 | Slc2a5 | Sdhc | Sdhc |
| Txn1 | Slc35f2 | Sdhd | Sdhd |
| Ube2d3 | Slco4c1 | Sec23ip | Sec23ip |
| Ube2j1 | Snx1 | Slc12a9 | Slc12a9 |
| Ube2n | Spata6 | Slc16a1 | Slc16a1 |
| Usp7 | Spg7 | Slc2a5 | Slc2a5 |
| Usp9x | Spnb3 | Slc35f2 | Slc35f2 |
| Vamp3 | Stx17 | Slco4c1 | Slco4c1 |
| Vars2 | Syne2 | Snx1 | Snx1 |
| Vcl | Tcp1 | Spata6 | Spata6 |
| Vim | Tcte1 | Spg7 | Spnb3 |
| Vkorc1 | Tpm3 | Spnb3 | Stx17 |
| Wrb | Tpmt | Stx17 | Syne2 |
| Xpo7 | Tpp2 | Syne2 | Tcp1 |
| Ywhab | Tram1 | Tcp1 | Tcte1 |
| Zfp185 | Trappc3 | Tcte1 | Tpm3 |
| 1600014C10Rik | Trappc4 | Tpm3 | Tpmt |
| 1700009P17Rik | Tsta3 | Tpmt | Tpp2 |
| 1700012A03Rik | Txn1 | Tpp2 | Tram1 |
| 1700034H14Rik | Ube2d3 | Tram1 | Trappc3 |
| 2300009A05Rik | Ube2j1 | Trappc3 | Trappc4 |
| 4921507P07Rik | Ube2n | Trappc4 | Tsta3 |
| 4921530D09Rik | Usp7 | Tsta3 | Txn1 |
| 4930415O20Rik | Usp9x | Txn1 | Ube2d3 |
| 4930471M23Rik | Vamp3 | Ube2d3 | Ube2j1 |
| 5730469M10Rik | Vars2 | Ube2j1 | Ube2n |
| 9430016H08Rik | Vcl | Ube2n | Usp7 |
| AI314180 | Vim | Usp7 | Usp9x |
| Abca3 | Vkorc1 | Usp9x | Vamp3 |
| Abcc3 | Wbscr16 | Vamp3 | Vars2 |
| Acat2 | Wrb | Vars2 | Vcl |
| Acbd5 | Xpo7 | Vcl | Vim |
| Aco1 | Ywhab | Vim | Vkorc1 |
| Acp1 | Zfp185 | Vkorc1 | Wbscr16 |
| Actn1 | 1600014C10Rik | Wrb | Wrb |
| Actr3 | 1700009P17Rik | Xpo7 | Xpo7 |
| Acyp1 | 1700012A03Rik | Ywhab | Ywhab |
| Adck5 | 1700034H14Rik | Zfp185 | Zfp185 |
| Adh5 | 2300009A05Rik | 1600014C10Rik |  |
| Akap4 | 4921507P07Rik | 1700009P17Rik |  |
| Anp32b | 4921530D09Rik | 1700012A03Rik |  |
| Ap2a1 | 4930415O20Rik | 1700034H14Rik |  |
| Ap2s1 | 4930471M23Rik | 2300009A05Rik |  |
| Aprt | 5730469M10Rik | 4732415M23Rik |  |
| Arl3 | 8430406I07Rik | 4921507P07Rik |  |
| Armc1 | 9430016H08Rik | 4921530D09Rik |  |
| Arpc4 | AI314180 | 4930415O20Rik |  |
| Arpc5l | Abca3 | 4930471M23Rik |  |
| Atp11a | Abcc3 | 5730469M10Rik |  |
| Atp13a4 | Acat2 | 8430406I07Rik |  |
| Atp6v0c | Acbd5 | 9430016H08Rik |  |
| BC051142 | Aco1 | AI314180 |  |
| Bloc1s1 | Acp1 | Abca3 |  |
| Bsg | Actn1 | Abcc3 |  |
| C030006K11Rik | Actr3 | Acat2 |  |
| Calm3 | Acyp1 | Acbd5 |  |
| Cap1 | Adck5 | Aco1 |  |
| Capza1 | Adh5 | Acp1 |  |
| Car2 | Akap4 | Actn1 |  |
| Cbr3 | Anp32b | Actr3 |  |
| Cct7 | Ap2a1 | Acyp1 |  |
| Cct8 | Ap2s1 | Adck5 |  |
| Chchd1 | Aprt | Adh5 |  |
| Ckmt1 | Arl3 | Akap4 |  |
| Clca2 | Armc1 | Anp32b |  |
| Clic3 | Arpc4 | Anxa13 |  |
| Clic4 | Arpc5l | Ap2a1 |  |
| Coasy | Atp11a | Ap2s1 |  |
| Cops4 | Atp13a4 | Aprt |  |
| Cops8 | Atp6v0c | Arl3 |  |
| Cox6b2 | BC051142 | Armc1 |  |
| Crisp2 | Bloc1s1 | Arpc4 |  |
| Crot | Bsg | Arpc5l |  |
| Csnk2a1 | C030006K11Rik | Atp11a |  |
| Ctsf | Calm3 | Atp13a4 |  |
| Cutc | Cap1 | Atp6v0c |  |
| Cyfip1 | Capza1 | BC051142 |  |
| Ddb1 | Car2 | Bloc1s1 |  |
| Ddt | Cbr3 | Bsg |  |
| Defb2 | Cct7 | C030006K11Rik |  |
| Dnaja4 | Cct8 | Cabyr |  |
| Dnajb6 | Chchd1 | Calm3 |  |
| Dnm2 | Ckmt1 | Cap1 |  |
| Dock7 | Clca2 | Capza1 |  |
| Dpysl2 | Clic3 | Car2 |  |
| Dtymk | Clic4 | Cbr3 |  |
| Dusp3 | Coasy | Cct7 |  |
| Ebp | Cops4 | Cct8 |  |
| Eef1d | Cops8 | Chchd1 |  |
| Eef1g | Cox6b2 | Ckmt1 |  |
| Ehd2 | Crisp2 | Clca2 |  |
| Eif2s1 | Crot | Clic3 |  |
| Eif5a | Csnk2a1 | Clic4 |  |
| Endog | Ctsf | Coasy |  |
| Eprs | Cyfip1 | Cops4 |  |
| Erbb2 | Cyp17a1 | Cops8 |  |
| Fdx1 | Ddb1 | Cox6b2 |  |
| Fhl1 | Ddt | Crisp2 |  |
| Fhl4 | Defb11 | Crot |  |
| Fmo2 | Defb19 | Csnk2a1 |  |
| Fundc1 | Defb2 | Ctsf |  |
| Fxc1 | Dnaja4 | Cyfip1 |  |
| Gaa | Dnajb6 | Ddb1 |  |
| Galntl4 | Dnm2 | Ddt |  |
| Gba | Dock7 | Defb11 |  |
| Gclm | Dpysl2 | Defb2 |  |
| Ghitm | Dtymk | Dnajb6 |  |
| Gm2a | Dusp3 | Dnm2 |  |
| Gpi1 | Ebp | Dock7 |  |
| Gpnmb | Eef1d | Dpysl2 |  |
| Gpx1 | Eef1g | Dtymk |  |
| Gpx3 | Ehd2 | Dusp3 |  |
| Gpx4 | Eif2s1 | Ebp |  |
| Gpx5 | Eif5a | Eef1d |  |
| Gsr | Eprs | Eef1g |  |
| Gstm1 | Erbb2 | Ehd2 |  |
| Gstm2 | Fdx1 | Eif2s1 |  |
| Gtl3 | Fhl1 | Eif5a |  |
| Hdhd2 | Fhl4 | Eprs |  |
| Hint1 | Fmo2 | Erbb2 |  |
| Hrsp12 | Fundc1 | Fdx1 |  |
| Hsd3b7 | Fxc1 | Fhl1 |  |
| Htatip2 | Gaa | Fhl4 |  |
| Idi1 | Galntl4 | Fmo2 |  |
| Immp2l | Gba | Fundc1 |  |
| Ipo7 | Gclm | Fxc1 |  |
| Isg20 | Ghitm | Fxn |  |
| Itpr1 | Gm2a | Gaa |  |
| Itpr3 | Gpi1 | Galntl4 |  |
| Klhl10 | Gpnmb | Gba |  |
| Lactb | Gpx1 | Gclm |  |
| Lamb2 | Gpx3 | Ghitm |  |
| Lamc1 | Gpx4 | Gm2a |  |
| Lamp2 | Gpx5 | Gpi1 |  |
| Lancl1 | Gsr | Gpnmb |  |
| Lancl2 | Gstm1 | Gpx1 |  |
| Lars | Gstm2 | Gpx3 |  |
| Lass2 | Gtl3 | Gpx4 |  |
| Lcn12 | Hdhd2 | Gpx5 |  |
| Lcn8 | Hint1 | Gsr |  |
| Lgals1 | Hrsp12 | Gstm1 |  |
| Lipe | Hsd3b7 | Gstm2 |  |
| Lipg | Htatip2 | Gtl3 |  |
| Ly6a | Idi1 | Hdhd2 |  |
| Lypla1 | Ipo7 | Hint1 |  |
| Man2a2 | Isg20 | Hrsp12 |  |
| Manba | Itpr1 | Hsd3b7 |  |
| Mdk | Itpr3 | Htatip2 |  |
| Mipep | Klhl10 | Idi1 |  |
| Mlf1 | Lamc1 | Ipo7 |  |
| Mrpl11 | Lamp2 | Isg20 |  |
| Mrpl17 | Lancl1 | Itpr1 |  |
| Mrpl18 | Lancl2 | Itpr3 |  |
| Mrpl43 | Lars | Klhl10 |  |
| Mrpl48 | Lass2 | Lactb |  |
| Mrpl52 | Lcn12 | Lamc1 |  |
| Mrpl55 | Lcn8 | Lamp2 |  |
| Mrps10 | Lgals1 | Lancl1 |  |
| Mrps14 | Lipe | Lancl2 |  |
| Mrps16 | Lipg | Lars |  |
| Mrps18a | Ly6a | Lass2 |  |
| Mrps18b | Lypla1 | Lcn12 |  |
| Mrps21 | Man2a2 | Lcn8 |  |
| Mrps24 | Manba | Lgals1 |  |
| Mrps31 | Mdk | Lipe |  |
| Mrps36 | Mipep | Lipg |  |
| Mrps6 | Mlf1 | Lpp |  |
| Mrrf | Mrpl11 | Ly6a |  |
| Mtpn | Mrpl17 | Lypla1 |  |
| Ncam1 | Mrpl18 | Man2a2 |  |
| Ndufc2 | Mrpl43 | Manba |  |
| Ndufs6 | Mrpl48 | Mdk |  |
| Ndufv2 | Mrpl52 | Mipep |  |
| Nif3l1 | Mrpl55 | Mlf1 |  |
| Nit1 | Mrps10 | Mrpl11 |  |
| Nqo1 | Mrps14 | Mrpl17 |  |
| Oplah | Mrps16 | Mrpl18 |  |
| Pcbp1 | Mrps18a | Mrpl43 |  |
| Pex11c | Mrps18b | Mrpl48 |  |
| Pfkm | Mrps21 | Mrpl52 |  |
| Pgk2 | Mrps24 | Mrpl55 |  |
| Pgls | Mrps31 | Mrps10 |  |
| Phb | Mrps36 | Mrps14 |  |
| Phpt1 | Mrps6 | Mrps16 |  |
| Pmm2 | Mrrf | Mrps18a |  |
| Ppap2a | Mtpn | Mrps18b |  |
| Ppil1 | Ncam1 | Mrps21 |  |
| Ppp2r1b | Ndufc2 | Mrps24 |  |
| Ppp2r4 | Ndufs6 | Mrps31 |  |
| Ppp6c | Ndufv2 | Mrps36 |  |
| Prkacb | Nif3l1 | Mrps6 |  |
| Psen1 | Nit1 | Mrrf |  |
| Psma1 | Nqo1 | Mtpn |  |
| Psma2 | Oplah | Ncam1 |  |
| Psma6 | Pcbp1 | Ndufc2 |  |
| Psma7 | Pex11c | Ndufs6 |  |
| Psmb1 | Pfkm | Ndufv2 |  |
| Psmb5 | Pgk2 | Nif3l1 |  |
| Psmb6 | Pgls | Nit1 |  |
| Psmd7 | Phb | Nqo1 |  |
| Psme1 | Phpt1 | Oplah |  |
| Psme4 | Pmm2 | Pcbp1 |  |
| Ptcd2 | Ppap2a | Pex11c |  |
| Ptges | Ppil1 | Pfkm |  |
| Ptp4a2 | Ppp2r1b | Pgk2 |  |
| Rab24 | Ppp2r4 | Pgls |  |
| Rab28 | Ppp6c | Phb |  |
| Rab2b | Prkacb | Phpt1 |  |
| Rab5b | Psen1 | Pmm2 |  |
| Rac2 | Psma1 | Ppap2a |  |
| Rdh13 | Psma2 | Ppil1 |  |
| Recql | Psma6 | Ppp2r1b |  |
| Rpia | Psma7 | Ppp2r4 |  |
| Rpl37a | Psmb1 | Ppp6c |  |
| Rpl5 | Psmb5 | Prkacb |  |
| Rplp2 | Psmb6 | Psen1 |  |
| Rps10 | Psmd7 | Psma1 |  |
| Rps13 | Psme1 | Psma2 |  |
| Rps19 | Psme4 | Psma6 |  |
| Rps27 | Ptcd2 | Psma7 |  |
| Rrbp1 | Ptges | Psmb1 |  |
| S100a16 | Ptp4a2 | Psmb5 |  |
| Sbds | Rab24 | Psmb6 |  |
| Scarb2 | Rab28 | Psmd7 |  |
| Sco1 | Rab2b | Psme1 |  |
| Sdf2l1 | Rab5b | Psme4 |  |
| Sdhc | Rac2 | Ptcd2 |  |
| Sdhd | Rdh13 | Ptges |  |
| Sec23ip | Recql | Ptp4a2 |  |
| Slc12a9 | Rpia | Rab24 |  |
| Slc16a1 | Rpl37a | Rab28 |  |
| Slc2a5 | Rpl5 | Rab2b |  |
| Slc35f2 | Rplp2 | Rab5b |  |
| Slco4c1 | Rps10 | Rac2 |  |
| Snx1 | Rps13 | Rdh13 |  |
| Spata6 | Rps19 | Recql |  |
| Spg7 | Rps27 | Ropn1l |  |
| Spnb3 | Rrbp1 | Rpia |  |
| Stx17 | S100a16 | Rpl37a |  |
| Syne2 | Sbds | Rpl5 |  |
| Tcp1 | Scarb2 | Rplp2 |  |
| Tcte1 | Sco1 | Rps10 |  |
| Tpm3 | Sdf2l1 | Rps13 |  |
| Tpmt | Sdhc | Rps19 |  |
| Tpp2 | Sdhd | Rps27 |  |
| Tram1 | Sec23ip | Rrbp1 |  |
| Trappc3 | Slc12a9 | S100a16 |  |
| Trappc4 | Slc16a1 | Sbds |  |
| Tsta3 | Slc2a5 | Scarb2 |  |
| Txn1 | Slc35f2 | Sco1 |  |
| Ube2d3 | Slco4c1 | Sdf2l1 |  |
| Ube2j1 | Snx1 | Sdhc |  |
| Ube2n | Spata6 | Sdhd |  |
| Usp7 | Spnb3 | Sec23ip |  |
| Usp9x | Stx17 | Slc12a9 |  |
| Vamp3 | Syne2 | Slc16a1 |  |
| Vars2 | Tcp1 | Slc2a5 |  |
| Vcl | Tcte1 | Slc35f2 |  |
| Vim | Tpm3 | Slco4c1 |  |
| Vkorc1 | Tpmt | Snx1 |  |
| Wbscr16 | Tpp2 | Spata6 |  |
| Wrb | Tram1 | Spg7 |  |
| Xpo7 | Trappc3 | Spnb3 |  |
| Ywhab | Trappc4 | Stx17 |  |
| Zfp185 | Tsta3 | Syne2 |  |
| 1600014C10Rik | Txn1 | Tcp1 |  |
| 1700009P17Rik | Ube2d3 | Tcte1 |  |
| 1700012A03Rik | Ube2j1 | Tpm3 |  |
| 1700034H14Rik | Ube2n | Tpmt |  |
| 2300009A05Rik | Usp7 | Tpp2 |  |
| 4921507P07Rik | Usp9x | Tram1 |  |
| 4921530D09Rik | Vamp3 | Trappc3 |  |
| 4930415O20Rik | Vars2 | Trappc4 |  |
| 4930471M23Rik | Vcl | Tsta3 |  |
| 5730469M10Rik | Vim | Txn1 |  |
| 9430016H08Rik | Vkorc1 | Ube2d3 |  |
| AI314180 | Wbscr16 | Ube2j1 |  |
| Abca3 | Wrb | Ube2n |  |
| Abcc3 | Xpo7 | Usp7 |  |
| Acat2 | Ywhab | Usp9x |  |
| Acbd5 | Zfp185 | Vamp3 |  |
| Aco1 | 1600014C10Rik | Vars2 |  |
| Acp1 | 1700009P17Rik | Vcl |  |
| Actn1 | 1700012A03Rik | Vim |  |
| Actr3 | 1700034H14Rik | Vkorc1 |  |
| Acyp1 | 2300009A05Rik | Wrb |  |
| Adck5 | 4921507P07Rik | Xpo7 |  |
| Adh5 | 4921530D09Rik | Ywhab |  |
| Akap4 | 4930415O20Rik | Zfp185 |  |
| Anp32b | 4930471M23Rik | 1600014C10Rik |  |
| Ap2a1 | 5730469M10Rik | 1700009P17Rik |  |
| Ap2s1 | 8430406I07Rik | 1700012A03Rik |  |
| Aprt | 9430016H08Rik | 1700034H14Rik |  |
| Arl3 | AI314180 | 2300009A05Rik |  |
| Armc1 | Abca3 | 4921507P07Rik |  |
| Arpc4 | Abcc3 | 4921530D09Rik |  |
| Arpc5l | Acat2 | 4930415O20Rik |  |
| Atp11a | Acbd5 | 4930471M23Rik |  |
| Atp13a4 | Aco1 | 5730469M10Rik |  |
| Atp6v0c | Acp1 | 8430406I07Rik |  |
| BC051142 | Actn1 | 9430016H08Rik |  |
| Bloc1s1 | Actr3 | AI314180 |  |
| Bsg | Acyp1 | Abca3 |  |
| C030006K11Rik | Adck5 | Abcc3 |  |
| Calm3 | Adh5 | Acat2 |  |
| Cap1 | Akap4 | Acbd5 |  |
| Capza1 | Anp32b | Aco1 |  |
| Car2 | Ap2a1 | Acp1 |  |
| Cbr3 | Ap2s1 | Actn1 |  |
| Cct7 | Aprt | Actr3 |  |
| Cct8 | Arl3 | Acyp1 |  |
| Chchd1 | Armc1 | Adck5 |  |
| Ckmt1 | Arpc4 | Adh5 |  |
| Clca2 | Arpc5l | Akap4 |  |
| Clic3 | Atp11a | Anp32b |  |
| Clic4 | Atp13a4 | Anxa13 |  |
| Coasy | Atp6v0c | Ap2a1 |  |
| Cops4 | BC051142 | Ap2s1 |  |
| Cops8 | Bloc1s1 | Aprt |  |
| Cox6b2 | Bsg | Arl3 |  |
| Crisp2 | C030006K11Rik | Armc1 |  |
| Crot | Calm3 | Arpc4 |  |
| Csnk2a1 | Cap1 | Arpc5l |  |
| Ctsf | Capza1 | Atp11a |  |
| Cutc | Car2 | Atp13a4 |  |
| Cyfip1 | Car4 | Atp6v0c |  |
| Ddb1 | Cbr3 | BC051142 |  |
| Ddt | Cct7 | Bloc1s1 |  |
| Defb2 | Cct8 | Bsg |  |
| Dnaja4 | Chchd1 | C030006K11Rik |  |
| Dnajb6 | Ckmt1 | Cabyr |  |
| Dnm2 | Clca2 | Calm3 |  |
| Dock7 | Clic3 | Cap1 |  |
| Dpysl2 | Clic4 | Capza1 |  |
| Dtymk | Coasy | Car2 |  |
| Dusp3 | Cops4 | Cbr3 |  |
| Ebp | Cops8 | Cct7 |  |
| Eef1d | Cox6b2 | Cct8 |  |
| Eef1g | Crisp2 | Chchd1 |  |
| Ehd2 | Crot | Ckmt1 |  |
| Eif2s1 | Csnk2a1 | Clca2 |  |
| Eif5a | Ctsf | Clic3 |  |
| Endog | Cyfip1 | Clic4 |  |
| Eprs | Cyp17a1 | Coasy |  |
| Erbb2 | Ddb1 | Cops4 |  |
| Fdx1 | Ddt | Cops8 |  |
| Fhl1 | Defb11 | Cox6b2 |  |
| Fhl4 | Defb19 | Crisp2 |  |
| Fmo2 | Defb2 | Crot |  |
| Fundc1 | Dnaja4 | Csnk2a1 |  |
| Fxc1 | Dnajb6 | Ctsf |  |
| Gaa | Dnm2 | Cutc |  |
| Galntl4 | Dock7 | Cyfip1 |  |
| Gba | Dpysl2 | Ddb1 |  |
| Gclm | Dtymk | Ddt |  |
| Ghitm | Dusp3 | Defb11 |  |
| Gm2a | Ebp | Defb2 |  |
| Gpi1 | Eef1d | Dnajb6 |  |
| Gpnmb | Eef1g | Dnm2 |  |
| Gpx1 | Ehd2 | Dock7 |  |
| Gpx3 | Eif2s1 | Dpysl2 |  |
| Gpx4 | Eif5a | Dtymk |  |
| Gpx5 | Eno1 | Dusp3 |  |
| Gsr | Eprs | Ebp |  |
| Gstm1 | Erbb2 | Eef1d |  |
| Gstm2 | Fdx1 | Eef1g |  |
| Gtl3 | Fhl1 | Ehd2 |  |
| Hdhd2 | Fhl4 | Eif2s1 |  |
| Hint1 | Fmo2 | Eif5a |  |
| Hrsp12 | Fundc1 | Eprs |  |
| Hsd3b7 | Fxc1 | Erbb2 |  |
| Htatip2 | Gaa | Fdx1 |  |
| Idi1 | Galntl4 | Fhl1 |  |
| Immp2l | Gba | Fhl4 |  |
| Ipo7 | Gclm | Fmo2 |  |
| Isg20 | Ghitm | Fundc1 |  |
| Itpr1 | Gm2a | Fxc1 |  |
| Itpr3 | Gpi1 | Fxn |  |
| Klhl10 | Gpnmb | Gaa |  |
| Lactb | Gpx1 | Galntl4 |  |
| Lamb2 | Gpx3 | Gba |  |
| Lamc1 | Gpx4 | Gclm |  |
| Lamp2 | Gpx5 | Ghitm |  |
| Lancl1 | Gsr | Gm2a |  |
| Lancl2 | Gstm1 | Gpi1 |  |
| Lars | Gstm2 | Gpnmb |  |
| Lass2 | Gtl3 | Gpx1 |  |
| Lcn12 | Hdhd2 | Gpx3 |  |
| Lcn8 | Hint1 | Gpx4 |  |
| Lgals1 | Hrsp12 | Gpx5 |  |
| Lipe | Hsd3b7 | Gsr |  |
| Lipg | Htatip2 | Gstm1 |  |
| Ly6a | Idi1 | Gstm2 |  |
| Lypla1 | Ipo7 | Gtl3 |  |
| Man2a2 | Isg20 | Hdhd2 |  |
| Manba | Itpr1 | Hint1 |  |
| Mdk | Itpr3 | Hrsp12 |  |
| Mipep | Klhl10 | Hsd3b7 |  |
| Mlf1 | Lamc1 | Htatip2 |  |
| Mrpl11 | Lamp2 | Idi1 |  |
| Mrpl17 | Lancl1 | Ipo7 |  |
| Mrpl18 | Lancl2 | Isg20 |  |
| Mrpl43 | Lars | Itpr1 |  |
| Mrpl48 | Lass2 | Itpr3 |  |
| Mrpl52 | Lcn12 | Klhl10 |  |
| Mrpl55 | Lcn8 | Lactb |  |
| Mrps10 | Lgals1 | Lamb2 |  |
| Mrps14 | Lipe | Lamc1 |  |
| Mrps16 | Lipg | Lamp2 |  |
| Mrps18a | Ly6a | Lancl1 |  |
| Mrps18b | Lypla1 | Lancl2 |  |
| Mrps21 | Man2a2 | Lars |  |
| Mrps24 | Manba | Lass2 |  |
| Mrps31 | Mdk | Lcn12 |  |
| Mrps36 | Mipep | Lcn8 |  |
| Mrps6 | Mlf1 | Lgals1 |  |
| Mrrf | Mrpl11 | Lipe |  |
| Mtpn | Mrpl17 | Lipg |  |
| Ncam1 | Mrpl18 | Lpp |  |
| Ndufc2 | Mrpl43 | Ly6a |  |
| Ndufs6 | Mrpl48 | Lypla1 |  |
| Ndufv2 | Mrpl52 | Man2a2 |  |
| Nif3l1 | Mrpl55 | Manba |  |
| Nit1 | Mrps10 | Mdk |  |
| Nme5 | Mrps14 | Mipep |  |
| Nqo1 | Mrps16 | Mrpl11 |  |
| Oplah | Mrps18a | Mrpl17 |  |
| Pcbp1 | Mrps18b | Mrpl18 |  |
| Pex11c | Mrps21 | Mrpl43 |  |
| Pfkm | Mrps24 | Mrpl48 |  |
| Pgk2 | Mrps31 | Mrpl52 |  |
| Pgls | Mrps36 | Mrpl55 |  |
| Phb | Mrps6 | Mrps10 |  |
| Phpt1 | Mrrf | Mrps14 |  |
| Pmm2 | Mtpn | Mrps16 |  |
| Ppap2a | Ncam1 | Mrps18a |  |
| Ppil1 | Ndufc2 | Mrps18b |  |
| Ppp2r1b | Ndufs6 | Mrps21 |  |
| Ppp2r4 | Ndufv2 | Mrps24 |  |
| Ppp6c | Nif3l1 | Mrps31 |  |
| Prkacb | Nit1 | Mrps36 |  |
| Psen1 | Nqo1 | Mrps6 |  |
| Psma1 | Pcbp1 | Mrrf |  |
| Psma2 | Pex11c | Mtpn |  |
| Psma6 | Pfkm | Ncam1 |  |
| Psma7 | Pgk2 | Ndufc2 |  |
| Psmb1 | Pgls | Ndufs6 |  |
| Psmb5 | Phb | Ndufv2 |  |
| Psmb6 | Phpt1 | Nif3l1 |  |
| Psmd7 | Pmm2 | Nit1 |  |
| Psme1 | Ppap2a | Nqo1 |  |
| Psme4 | Ppil1 | Oplah |  |
| Ptcd2 | Ppp2r1b | Pcbp1 |  |
| Ptges | Ppp2r4 | Pex11c |  |
| Ptp4a2 | Ppp6c | Pfkm |  |
| Rab24 | Prkacb | Pgk2 |  |
| Rab28 | Psen1 | Pgls |  |
| Rab2b | Psma1 | Phb |  |
| Rab5b | Psma2 | Phpt1 |  |
| Rac2 | Psma6 | Pmm2 |  |
| Rdh13 | Psma7 | Ppap2a |  |
| Recql | Psmb1 | Ppil1 |  |
| Rpia | Psmb5 | Ppp2r1b |  |
| Rpl37a | Psmb6 | Ppp2r4 |  |
| Rpl5 | Psmd7 | Ppp6c |  |
| Rplp2 | Psme1 | Prkacb |  |
| Rps10 | Psme4 | Psen1 |  |
| Rps13 | Ptcd2 | Psma1 |  |
| Rps19 | Ptges | Psma2 |  |
| Rps27 | Ptp4a2 | Psma6 |  |
| Rrbp1 | Rab24 | Psma7 |  |
| S100a16 | Rab28 | Psmb1 |  |
| Sbds | Rab2b | Psmb5 |  |
| Scarb2 | Rab5b | Psmb6 |  |
| Sco1 | Rac2 | Psmd7 |  |
| Sdf2l1 | Rdh13 | Psme1 |  |
| Sdhc | Recql | Psme4 |  |
| Sdhd | Rpia | Ptcd2 |  |
| Sec23ip | Rpl37a | Ptges |  |
| Slc12a9 | Rpl5 | Ptp4a2 |  |
| Slc16a1 | Rplp2 | Rab24 |  |
| Slc2a5 | Rps10 | Rab28 |  |
| Slc35f2 | Rps13 | Rab2b |  |
| Slco4c1 | Rps19 | Rab5b |  |
| Snx1 | Rps27 | Rac2 |  |
| Spata6 | Rrbp1 | Rdh13 |  |
| Spg7 | S100a16 | Recql |  |
| Spnb3 | Sbds | Ribc1 |  |
| Stx17 | Scarb2 | Ropn1l |  |
| Syne2 | Sco1 | Rpia |  |
| Tcp1 | Sdf2l1 | Rpl37a |  |
| Tcte1 | Sdhc | Rpl5 |  |
| Tpm3 | Sdhd | Rplp2 |  |
| Tpmt | Sec23ip | Rps10 |  |
| Tpp2 | Slc12a9 | Rps13 |  |
| Tram1 | Slc16a1 | Rps19 |  |
| Trappc3 | Slc2a5 | Rps27 |  |
| Trappc4 | Slc35f2 | Rrbp1 |  |
| Tsta3 | Slco4c1 | S100a16 |  |
| Txn1 | Snx1 | Sbds |  |
| Ube2d3 | Spata6 | Scarb2 |  |
| Ube2j1 | Spg7 | Sco1 |  |
| Ube2n | Spnb3 | Sdf2l1 |  |
| Usp7 | Stx17 | Sdhc |  |
| Usp9x | Syne2 | Sdhd |  |
| Vamp3 | Tcp1 | Sec23ip |  |
| Vars2 | Tcte1 | Slc12a9 |  |
| Vcl | Tpm3 | Slc16a1 |  |
| Vim | Tpmt | Slc2a5 |  |
| Vkorc1 | Tpp2 | Slc35f2 |  |
| Wbscr16 | Tram1 | Slco4c1 |  |
| Wrb | Trappc3 | Snx1 |  |
| Xpo7 | Trappc4 | Spata6 |  |
| Ywhab | Tsta3 | Spg7 |  |
| Zfp185 | Txn1 | Spnb3 |  |
|  | Ube2d3 | Stx17 |  |
|  | Ube2j1 | Syne2 |  |
|  | Ube2n | Tcp1 |  |
|  | Usp7 | Tcte1 |  |
|  | Usp9x | Tpm3 |  |
|  | Vamp3 | Tpmt |  |
|  | Vars2 | Tpp2 |  |
|  | Vcl | Tram1 |  |
|  | Vim | Trappc3 |  |
|  | Vkorc1 | Trappc4 |  |
|  | Wbscr16 | Tsta3 |  |
|  | Wrb | Txn1 |  |
|  | Xpo7 | Ube2d3 |  |
|  | Ywhab | Ube2j1 |  |
|  | Zfp185 | Ube2n |  |
|  |  | Usp7 |  |
|  |  | Usp9x |  |
|  |  | Vamp3 |  |
|  |  | Vars2 |  |
|  |  | Vcl |  |
|  |  | Vim |  |
|  |  | Vkorc1 |  |
|  |  | Wbscr16 |  |
|  |  | Wrb |  |
|  |  | Xpo7 |  |
|  |  | Ywhab |  |
|  |  | Zfp185 |  |
