# Supplementary material for: Proton-Secreting Cells as Drivers of Inflammation and Sperm Dysfunction in LPS-Induced Epididymitis
Source: Function (Oxf). 2025 Jun 2;6(3):zqaf023. doi: 10.1093/function/zqaf023 (PMC12203219; doi:10.1093/function/zqaf023)
Supplement: zqaf023_Supplemental_Files [file zqaf023_supplemental_files.zip › Suppl. Table 4.docx]

**Supplementary Table 4: Computer-assisted sperm analysis (CASA) 4 and 24 h post-LPS injections.** Sperm was isolated from distal epididymides 4 and 24 h post-intravasal-epididymal injections of saline or LPS. Distal sperm were capacitated in HTF medium (dilution ratio 1:5) with 0.3 % BSA and incubated for 60 min at 37 °C. Data are shown as mean ±SEM. Sperm analyses were performed using Hamilton Thorne’s CASA version 14.
