# Supplementary material for: Proton-Secreting Cells as Drivers of Inflammation and Sperm Dysfunction in LPS-Induced Epididymitis
Source: Function (Oxf). 2025 Jun 2;6(3):zqaf023. doi: 10.1093/function/zqaf023 (PMC12203219; doi:10.1093/function/zqaf023)
Supplement: zqaf023_Supplemental_Files [file zqaf023_supplemental_files.zip › Supplementary Figures final.docx]

**Supplementary Figures**

**Supplementary Figure 1: Cell sorting strategy for epididymal EGFP^+^ CCs.** Fluorescence-activated cell sorting (FACS) was performed 1 h after intravasal-epididymal injection of saline or LPS to isolate CCs, using the gating strategy illustrated. Key parameters included forward scatter area (FSC-A), side scatter area (SSC-A), side scatter width (SSC-W), and forward scatter width (FSC-W). DAPI was used as a viability dye to exclude dead cells.

**Supplementary Figure 2: Gating strategies for quantitative beads and myeloid cells.** Flow cytometry gating strategy to analyze **A)** quantitative beads and **B)** myeloid cells (CD11b^+^) in the epididymis. Key parameters include forward scatter area (FSC-A) and side scatter area (SSC-A). The previous gating plots are shown in **Fig. 6**. DAPI was used as a viability dye to exclude dead cells.

**Supplementary Figure 3: Enrichment of immune response- and sperm maturation-related genes on segment 7 of the epididymis.** Venn diagrams comparing segment 7 with other segments grouped into proximal (1, 2, and 3), middle (4, 5, and 6), and distal (8, 9, and 10) regions highlighting the distinct patterns of immune response (**A**) and sperm maturation-related gene expression (**B**). Red highlights immune response-related genes in Segment 7.

**Supplementary Figure 4: LPS injection increased apical bleb formation in the proximal epididymis.** Confocal microscopy images of EGFP^+^ CCs in the IS after saline and LPS injections. Low magnification images of **Fig. 3A**. Nuclei are labeled with DAPI (blue). Bars: 200 μm.

**Supplementary Figure 5: LPS or saline injections caused epididymal epithelial damage in segments 7/8**. Epididymal segments were analyzed using AQP9 (red) immunolabeling 24 h after saline and LPS injections. **A)** No epithelial damage was observed in the proximal segments (S1, S2, and S3). **B)** In segments 7 and 8 (S7 and S8), saline injections caused epithelial damage in a few regions (indicated by arrows in **C**). In contrast, LPS injections resulted in more extensive epithelial damage across several regions (arrows). Bars: 20 μm. Inset Bars: 10 μm. Nuclei are labeled with DAPI (blue)—no morphological changes in S9 and S10.

**Supplementary Table 1: Transcriptomic profiles of CCs 1 h after saline or LPS intravasal-epididymal injection.** The complete transcriptome dataset of EGFP^+^ CCs from saline and LPS intravasal-epididymal injected epididymis from the IS/caput, corpus, and cauda regions of the mouse epididymis. RNA-seq was performed in 3 independent samples per group.

**Supplementary Table 2: List of immune response-related genes in different segments of the epididymis.** We analyzed the distinct immune response-related gene expression per epididymal segment using previous transcriptomic data from the whole epididymis ^42^.

**Supplementary Table 3: List of sperm maturation-related genes in different segments of the epididymis.** Using previous transcriptomic data from the whole epididymis ^42^, we analyzed the distinct sperm maturation-related gene expression per epididymal segment.

**Supplementary Table 4: Computer-assisted sperm analysis (CASA) 4 and 24 h post-LPS injections.** Sperm was isolated from distal epididymides 4 and 24 h post-intravasal-epididymal injections of saline or LPS. Distal sperm were capacitated in HTF medium (dilution ratio 1:5) with 0.3 % BSA and incubated for 60 min at 37 °C. Data are shown as mean ±SEM. Sperm analyses were performed using Hamilton Thorne’s CASA version 14.
